# MVI-targeted carbon-ion radiotherapy combined with immunotherapy for advanced hepatocellular carcinoma: Phase Ib DEPARTURE trial

**DOI:** 10.1016/j.jhepr.2026.101765

**Published:** 2026-02-05

**Authors:** Sadahisa Ogasawara, Keisuke Koroki, Hirokazu Makishima, Masaru Wakatsuki, Asahi Takahashi, Makoto Fujiya, Sae Yumita, Miyuki Nakagawa, Hiroaki Kanzaki, Kazufumi Kobayashi, Masanori Inoue, Masato Nakamura, Naoya Kanogawa, Takayuki Kondo, Shingo Nakamoto, Tomoya Kurokawa, Yoshihito Ozawa, Yosuke Inaba, Soumith Paritala, Jingxuan Chen, Jeon Lee, Yujin Hoshida, Hideki Hanaoka, Shigeru Yamada, Hitoshi Ishikawa

**Affiliations:** 1 Department of Gastroenterology, Graduate School of Medicine, Chiba University, Chiba, Japan; 2 National Institutes for Quantum Science and Technology, QST Hospital, Chiba, Japan; 3 Clinical Research Center, Chiba University Hospital, Chiba, Japan; 4 Division of Digestive and Liver Diseases, Department of Internal Medicine, University of Texas Southwestern Medical Center, Dallas, TX, USA; 5 Lyda Hill Department of Bioinformatics, The University of Texas Southwestern Medical Center, Dallas, TX, USA

**Keywords:** Carbon-ion radiotherapy, Durvalumab, Tremelimumab, Hepatocellular carcinoma

## Abstract

**Background & Aims:**

Advanced hepatocellular carcinoma (HCC) with macrovascular invasion (MVI) carries an extremely poor prognosis, necessitating novel therapeutic strategies. This phase Ib trial evaluated the safety and preliminary efficacy of combining carbon-ion radiotherapy (C-ion RT) with immune checkpoint inhibitors (ICIs) in patients with advanced HCC with MVI.

**Methods:**

Fifteen patients with MVI-positive advanced HCC were enrolled (Cohort A: durvalumab monotherapy, n = 3; Cohort B: durvalumab plus tremelimumab, n = 12). C-ion RT (60 Gy, four fractions) was delivered to the MVI-containing primary tumor, while systemic therapy with durvalumab (+tremelimumab) was administered concurrently. The primary endpoints included dose-limiting toxicities and adverse events. Secondary endpoints included progression-free survival and overall survival.

**Results:**

No dose-limiting toxicities were observed, and the combination exhibited a manageable safety profile. The most common adverse events were pyrexia, rash, and elevated lipase levels. Grade 3–4 adverse events occurred in 53.3%, including cytokine release syndrome and meningitis. Median progression-free survival and overall survival were 4.7 and 10.4 months, respectively. Although C-ion RT achieved effective local control of irradiated lesions, non-irradiated lesions showed limited systemic immune responses.

**Conclusions:**

The combination of MVI-targeted C-ion RT and immune checkpoint inhibitors demonstrated safe and effective local tumor control in advanced HCC. This novel approach of selective irradiation to MVI-containing tumors, combined with systemic immunotherapy, warrants further investigation to optimize the synergistic effects and enhance systemic efficacy in this poor-prognosis group.

**Impact and implications:**

Advanced hepatocellular carcinoma with macrovascular invasion (MVI) has a poor prognosis, highlighting the need for new therapeutic strategies. Our phase Ib study suggests that carbon-ion radiotherapy targeting MVI combined with immune checkpoint inhibitors is feasible and achieves sustained local tumor control. RNA-sequencing revealed that immune activation pathways were enriched in responders, while resistance was associated with mesenchymal and angiogenesis signatures. These results reinforce the potential of MVI-targeted irradiation combined with immune checkpoint inhibitors as a promising treatment strategy for these high-risk patients, warranting further investigation to improve systemic tumor control.

**Clinical Trials Registration:**

jRCT2031210046.

## Highlights

• The trial evaluated the C-ion RT plus ICIs for HCC with macrovascular invasion.
• No DLTs were observed, and the combination showed a manageable safety profile.
• Many patients experienced transient high fevers after C-ion RT.
• The approach achieved safety and effective local control.
• The results highlight the need for further optimization of this combination strategy.

## Graphical abstract

**Figure:**
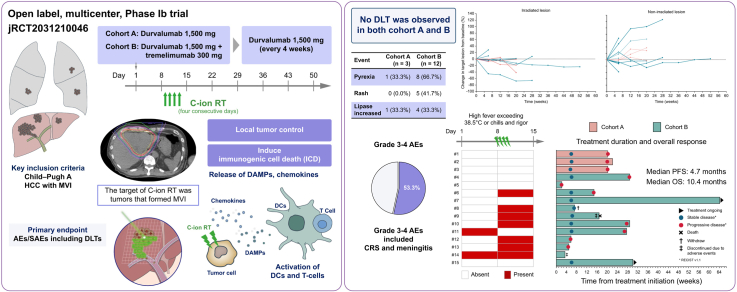

## Introduction

Hepatocellular carcinoma (HCC) is a leading cause of cancer-related mortality worldwide, with a 5-year survival rate of only 18% owing to late diagnosis and limited treatment options.^1^^,^^2^ Even when detected early, its high recurrence rate often leads to advanced disease. A key feature of HCC progression is macrovascular invasion (MVI), characterized by tumor infiltration into hepatic vessels such as the portal and hepatic veins, distinguishing it from other malignancies.^3^^,^^4^ MVI disrupts vascular flow, often leading to liver failure, making it a critical prognostic factor requiring targeted therapies.^5^ Local MVI control through surgical resection, transarterial chemoembolization (TACE), and radiotherapy has been associated with improved outcomes.[6], [7], [8] Recent findings from our group further highlight that effective MVI control with systemic therapy significantly enhances overall survival (OS), underscoring its pivotal role in advanced HCC management.^9^

The treatment paradigm for advanced HCC has evolved from single-agent tyrosine kinase inhibitors (TKIs) to combination immunotherapy strategies.[10], [11], [12] Atezolizumab plus bevacizumab is widely adopted as first-line treatment, targeting both programmed cell death ligand 1 (PD-L1) and vascular endothelial growth factor (VEGF). Bevacizumab inhibits VEGF, reducing angiogenesis and enhancing T-cell infiltration, whereas atezolizumab reactivates exhausted T cells by blocking PD-L1, simultaneously addressing immune evasion and angiogenesis.^13^ In contrast, durvalumab (anti-PD-L1) plus tremelimumab (anti-CTLA-4) focuses on dual checkpoint blockade, with PD-L1 inhibition reactivating T cells in the tumor and CTLA-4 blockade promoting T-cell activation in lymphoid organs, enhancing systemic immunity.^14^ The HIMALAYA trial (NCT03298451) established this combination as a standard first-line therapy, showing significant survival benefits over sorafenib.^12^ Early evidence for combining local therapy with immunotherapy came from a phase I study of tremelimumab plus tumor ablation, demonstrating safety and increased intratumoral CD8+ T-cell infiltration in responders.^15^ These findings provided proof of concept for integrating local therapy with immune checkpoint inhibition. However, therapeutic efficacy in patients with MVI remains suboptimal, underscoring the need for novel strategies.

Carbon-ion radiotherapy (C-ion RT) has emerged as a promising local treatment modality, offering superior dose-distribution characteristics that enable precise targeting of hepatic tumors while preserving surrounding healthy tissue.^16^^,^^17^ The physical properties of carbon ions allow for enhanced biological effectiveness and reduced oxygen dependence compared with conventional radiotherapy, potentially offering advantages in treating radioresistant tumors.^18^ Recent data have demonstrated exceptional local control rates with C-ion RT in HCC, leading to its regulatory approval in Japan for cases unsuitable for radical hepatic resection.^19^ Unlike photon-based approaches such as stereotactic body radiotherapy (SBRT), which are generally recommended for small lesions (<3–5 cm) owing to liver tolerance and carry increased risks of radiation-induced liver injury in large or perihilar tumors, C-ion RT enables safe, high-dose delivery even for large or vascular-invasive lesions by virtue of its Bragg-peak distribution and superior biological effectiveness.[20], [21], [22] This makes C-ion RT particularly suitable for controlling MVI-positive tumors, where SBRT is often not feasible, and provides a rationale for its use in this trial. Importantly, radiation therapy has been shown to enhance immunogenic cell death (ICD), increase neoantigen presentation, and favorably modulate the tumor microenvironment, suggesting potential synergistic effects with immunotherapy.^23^^,^^24^ This precise targeting capability makes C-ion RT particularly suitable for selective irradiation of MVI-positive lesions while sparing other hepatic regions.

Given this scientific rationale, we designed this phase Ib trial to evaluate a novel therapeutic approach that combines selective C-ion RT targeting only the MVI-containing tumors with durvalumab plus tremelimumab immunotherapy. Our strategic focus on MVI-positive lesions, rather than treating all tumor sites, was chosen to address the most critical prognostic factor while relying on systemic immunotherapy to control potential disseminated disease. This study represents the first clinical investigation of this selective targeting approach, aiming to leverage the precise local tumor control capabilities of C-ion RT specifically for MVI-positive lesions, while utilizing dual checkpoint inhibition for systemic disease control.

## Patients and methods

Detailed information on the study rationale, design, and treatment plan has been previously published in the protocol paper.^25^ We conducted a phase Ib trial to evaluate the safety and tolerability of a novel therapeutic approach combining selective C-ion RT directed at MVI-containing tumors with durvalumab plus tremelimumab in patients with advanced HCC (jRCT2031210046). Given the extensive study methodology, including eligibility criteria, treatment regimens, and key aspects such as sample size determination, statistical analysis, dose-limiting toxicity (DLT) definitions, prospective observation after trial completion, exploratory biomarker analysis with tumor biopsy samples, data management, monitoring, and ethics, these details are comprehensively described in the Supplementary Methods.

### Patients

Key eligibility criteria are summarized below, with full details in Table S1. Patients aged ≥20 years with histologically confirmed advanced HCC or typical hypervascular findings on imaging were eligible. Key inclusion criteria included macrovascular invasion, ECOG performance status 0–1, body weight >30 kg, and Child–Pugh class A. The patients except for expansion cohort required prior failure of systemic therapy. Both viral and non-viral HCC patients were eligible. Adequate normal organ and marrow function as defined below: hemoglobin ≥9.0 g/dl, absolute neutrophil count ≥1,500/mm^3^, platelet count ≥75,000/mm^3^, serum bilirubin ≤ upper limit of normal (ULN) × 3.0, aspartate aminotransferase (AST) ≤ ULN × 5.0, alanine aminotransferase (ALT) ≤ ULN × 5.0, and measured creatinine clearance >40 ml/min or calculated creatinine clearance >40 ml/min by the Cockcroft–Gault formula or by 24-h urine collection for determination of creatinine clearance. Patients with disease up to Vp4 and Vv3 could also be included (patients with bile duct invasion were excluded). Patients with extrahepatic spread were also eligible for inclusion in the trial.

Exclusion criteria included unresolved grade ≥2 toxicities, extensive prior radiotherapy, major recent surgery, organ transplantation, primary immunodeficiency, autoimmune disorders, active brain metastases, hepatitis B/C or B/D coinfection, recent immunosuppressive use, or hypersensitivity to study drugs. Eligibility was assessed by investigators. Patients who are not eligible for irradiation in terms of surrounding risk organs cannot be incorporated during C-ion RT, as described below.

### Study design and treatment

A detailed trial methodology is available in the Supplementary material. This phase Ib, multicenter, open-label trial (jRCT2031210046) evaluates the safety and tolerability of combining selective C-ion RT targeting MVI-containing tumors with immunotherapy in advanced HCC. Patients were enrolled at two centers in Japan (Chiba University Hospital and QST Hospital) into two cohorts: durvalumab monotherapy combined with C-ion RT (Cohort A) or durvalumab plus a single tremelimumab dose combined with C-ion RT (Cohort B). Durvalumab was administered every 4 weeks until disease progression in either cohort (Fig. S1).

In this trial, a minimum 7-day hospitalization period was pre-set following completion of C-ion RT to manage adverse events (AEs). During this period, patients received rigorous systemic management, including repeated vital sign monitoring, and blood tests. After the 7-day period, patients were discharged only if their general condition was stable and their blood test results showed no severe AEs.

The primary objective is to assess safety, with DLTs evaluated over 42 days using CTCAE v5.0 (Table S2). Primary endpoints include DLT, AEs, and severe AEs. Secondary endpoints include OS, objective response rate (ORR), progression-free survival (PFS) and time to progression (TTP). Tumor response is assessed via imaging every 6 weeks, with confirmation required for complete and partial responses. The primary criterion used in this study was Response Evaluation Criteria in Solid Tumors (RECIST) version 1.1.

### Definition and assessment period of DLT

Three patients were initially enrolled in Cohort A (durvalumab monotherapy). If no DLTs occurred during the 42-day evaluation period, enrollment proceeded to Cohort B (durvalumab plus tremelimumab). If a DLT occurred, three additional patients were enrolled in the same cohort. A regimen was considered intolerable if more than one DLT occurred among six patients; if Cohort A was intolerable, Cohort B was not initiated. DLTs were assessed during a 42-day period starting from the initiation of investigational treatment (Day 1 of Cycle 1). This period was defined to capture acute radiotherapy-related toxicities and was determined following discussions with the Japanese regulatory authorities. Adverse events were graded using CTCAE v5.0, and DLTs were defined as treatment-related events meeting predefined criteria (Table S2).

### C-ion RT

C-ion RT (60 Gy in four fractions) was initiated on Day 8 of Cycle 1. The gross tumor volume (GTV) included intrahepatic nodules forming MVI and the MVI itself. The clinical target volume and field-specific planning target volume were defined based on respiratory motion analysis using 4D-CT.^26^ Dose calculation was performed using the microdosimetric kinetic model, and all doses are expressed as relative biological equivalent (RBE).^27^ Details of target delineation, motion management, and dose constraints are provided in the Supplementary Methods.^16^^,^[28], [29], [30], [31]

## Results

### Patient characteristics

Informed consent was obtained from 18 patients, of whom 15 were enrolled. All 15 enrolled patients received the investigational treatment. In Cohort A, tolerability was assessed in three individuals, while in Cohort B, 12 patients were enrolled (four for tolerability evaluation, eight for the expansion cohort; Fig. S2).

The median age of the patients was 69 years (range, 25–81 years), and 13 patients (86.7%) were male. Non-viral etiologies were the most common underlying cause of liver disease (HBV: n = 3 [20.0%], HCV: n = 1 [13.3%], non-viral: n = 10 [66.7%]). All patients were classified as Barcelona Clinic Liver Cancer (BCLC) stage C and presented with MVI. Among the 15 patients included in this study, major portal vein invasion (Vp3–4) was absent in eight individuals and present in seven. Among the 15 patients enrolled, four (26.7%) were systemic therapy-naive (Table 1). Representativeness of the study participants is described in Table S3. The median follow-up duration for the overall study population during the trial period was 5.7 months. With the inclusion of the post-trial prospective observational period (Supplementary Methods), the median follow-up duration extended to 10.4 months.

**Table 1:** Patient characteristics.

	Whole population (N = 15)	Cohort A (n = 3)	Cohort B (n = 12)
Age, median (range)	69.0 (25.0–81.0)	61.0 (58.0–74.0)	70.0 (25.0–81.0)
Sex, male, n (%)	13 (86.7)	3 (100.0)	10 (83.3)
HBV positive, n (%)	3 (20.0)	0 (0.0)	3 (25.0)
HCV positive, n (%)	2 (13.3)	1 (33.3)	1 (8.3)
Non-viral, n (%)	10 (66.7)	2 (66.7)	8 (66.7)
Child–Pugh score, n (%)			
5	10 (66.7)	3 (100.0)	7 (58.3)
6	5 (33.3)	0 (0.0)	5 (41.7)
ECOG-PS, n (%)			
0	13 (86.7)	3 (100.0)	10 (83.3)
1	2 (13.3)	0 (0.0)	2 (16.7)
Median maximum tumor size, mm (range)	91.1 (32–221.8)	92.4 (54–103)	89.6 (32–221.8)
Number of intrahepatic tumors ≥8, n (%)	10 (66.7)	3 (100.0)	7 (58.3)
Portal vein invasion, n (%)			
Vp2	7 (46.7)	2 (66.7)	5 (41.7)
Vp3	3 (20.0)	1 (33.3)	2 (16.7)
Vp4	4 (26.7)	0 (0.0)	4 (33.3)
Hepatic vein invasion, n (%)			
Vv2	1 (6.7)	0 (0.0)	1 (8.3)
Vv3	1 (6.7)	0 (0.0)	1 (8.3)
Extrahepatic spread, n (%)	7 (46.7)	1 (33.3)	6 (50.0)
AFP (ng/ml), median (range)	213.7 (3.5–270,208.0)	10,444.3 (56.7–29,082.1)	165.3 (3.5–270,208.0)
Systemic therapy-naive, n (%)	4 (26.7)	—	4 (33.3)
Presence of prior systemic therapy	11	3	8
Treatment regimen of prior systemic therapy			
Atezolizumab + bevacizumab	8	2	6
Lenvatinib	1	0	1
Sorafenib	1	0	1
Ramucirumab	1	1	0

AFP, alpha-fetoprotein; ECOG-PS, Eastern Cooperative Oncology Group performance status; EHM, extrahepatic metastasis; MVI, macrovascular invasion.

### Primary outcomes: safety and adverse events

Of the six patients included in the DLT evaluation (three in Cohort A and three in Cohort B). DLT observation rate were 0% (0/3) in Cohort A, 0% (0/3) in Cohort B, respectively. AEs of any grade were reported in all 15 patients (100%). Serious AEs (SAEs) occurred in five patients (33.3%) for a total of 11 events. Two of these SAEs led to treatment discontinuation. No SAEs were reported in Cohort A. In Cohort B, SAEs were reported in four patients, including mucosal inflammation, adrenal insufficiency, interstitial lung disease, enterocolitis, cytokine release syndrome, and meningitis. Seven patients (46.7%) experienced grade 3 AEs, two patients (13.3%) experienced grade 4 AEs, and one patient (6.7%) experienced grade 5 AE (heart failure). Treatment-emergent AEs (TEAEs) occurring in ≥10% of patients are summarized in Table 2. The most common TEAEs were pyrexia, rash, elevated lipase levels, and radiation-induced skin lesions. In Table 2, we also report the frequency of treatment-emergent AEs (TEAEs) stratified by the presence or absence of major portal vein invasion (Vp3–4). Detailed data on Grade 2–4 treatment-related AEs (TRAEs) are provided in Table S4.

**Table 2:** TEAEs occurring in ≥10% of whole study population, stratified by treatment cohort and major portal vein invasion.

Event, n (%)	Whole population (N = 15)	Cohort A: Durvalumab + C-ion RT (n = 3)	Cohort B: Durvalumab + tremelimumab + C-ion RT (n = 12)	Major portal vein invasion (Vp3, 4)	
				Absent (n = 8)	Present (n = 7)
Any event	15 (100.0)	3 (100.0)	12 (100.0)	8 (100.0)	7 (100.0)
Pyrexia	9 (60.0)	1 (33.3)	8 (66.7)	5 (62.5)	4 (57.1)
Rash	5 (33.3)	0 (0.0)	5 (41.7)	2 (25.0)	3 (42.9)
Lipase increased	5 (33.3)	1 (33.3)	4 (33.3)	3 (37.5)	2 (28.6)
Radiation skin injury	5 (33.3)	2 (66.7)	3 (25.0)	3 (37.5)	2 (28.6)
Decreased appetite	4 (26.7)	1 (33.3)	3 (25.0)	2 (25.0)	2 (28.6)
Constipation	3 (20.0)	0 (0.0)	3 (25.0)	0 (0.0)	3 (42.9)
Diarrhea	3 (20.0)	0 (0.0)	3 (25.0)	1 (12.5)	2 (28.6)
Malaise	3 (20.0)	0 (0.0)	3 (25.0)	1 (12.5)	2 (28.6)
ALT increased	3 (20.0)	1 (33.3)	2 (16.7)	2 (25.0)	1 (14.3)
Amylase increased, n (%)	3 (20.0)	1 (33.3)	2 (16.7)	2 (25.0)	1 (14.3)
AST increased	3 (20.0)	1 (33.3)	2 (16.7)	2 (25.0)	1 (14.3)
COVID-19	2 (13.3)	0 (0.0)	2 (16.7)	2 (25.0)	0 (0.0)
Interstitial lung disease	2 (13.3)	0 (0.0)	2 (16.7)	1 (12.5)	1 (14.3)
Pleural effusion	2 (13.3)	0 (0.0)	2 (16.7)	1 (12.5)	1 (14.3)
Abdominal pain	2 (13.3)	1 (33.3)	1 (8.3)	1 (12.5)	1 (14.3)
Platelet count decreased	2 (13.3)	1 (33.3)	1 (8.3)	2 (25.0)	0 (0.0)

ALT, alanine aminotransferase; AST, aspartate aminotransferase; TEAEs, treatment-emergent adverse events.

### Secondary outcomes: efficacy analysis

The median PFS time for all patients, based on RECIST version 1.1, was 4.7 months (95% CI, 1.4–6.4). In Cohort A, the median PFS time was 4.7 months (95% CI, 4.6–4.7), whereas in Cohort B, it was 3.8 months (95% CI, 1.1–6.6). The 6-month PFS rates were 0% in Cohort A and 47.6% in Cohort B. Consistent with the PFS findings, the median TTP for all patients was 4.7 months (95% CI, 1.3–6.4). The median TTP was 4.7 months (95% CI, 4.6–4.7) in Cohort A and 6.2 months (95% CI, 1.1–6.6) in Cohort B. Based on RECIST version 1.1, the disease control rate was 73.3% in whole population. At the end of the study period, only one death had occurred and OS data were immature. Therefore, OS data are presented based on subsequent follow-up. The median OS was 28.2 months (95% CI, 5.5–50.9) in Cohort A and 10.2 months (95% CI, 4.3–16.1) in Cohort B. The 6-month OS rates were 100% for Cohort A and 63.6% for Cohort B. For the entire cohort of 15 patients, the median OS was 10.4 months (95% CI, 5.6–15.2), with 6-month and 12-month OS rates of 66.7% and 46.7%, respectively. An additional *post-hoc* efficacy analysis was conducted according to the presence of major portal vein invasion (Vp3–4). Median PFS was comparable between patients without and with Vp3–4 (4.6 months, 95% CI, 1.1–6.5 *vs.* 4.7 months, 95% CI, 0.5–6.6; *p* = 0.960). Median OS was 14.5 months (95% CI, 4.9–NE) in the Vp3–4–absent group and 9.5 months (95% CI, 3.2–28.2) in the Vp3–4–present group (*p* = 0.222) (Figs. S3 and S4 and Table S5).

**Fig. 1:**
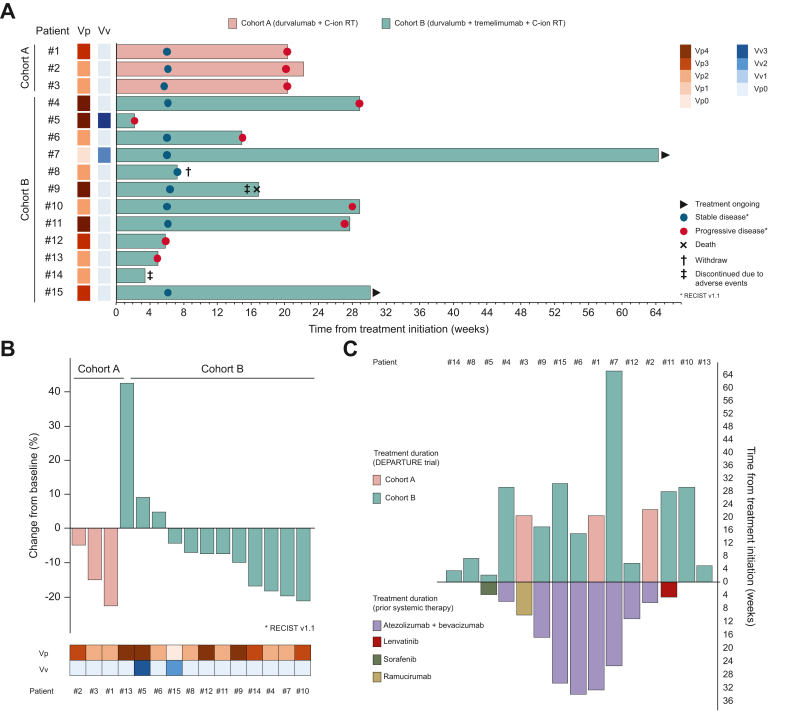
Efficacy of investigational treatment. (A) Swimmer’s plot showing treatment duration and best response. (B) Waterfall plot showing the change in the sum of target lesion diameters. (C) Iceberg plot of treatment duration before and during the investigational therapy. Prior systemic therapy duration is shown below the baseline, and treatment duration with the investigational regimen is shown above.

Fig. 1 provides an overview of treatment outcomes, including a swimmer plot for treatment duration and response timing (Fig. 1A), and a waterfall plot indicating depth of tumor response (Fig. 1B). The median duration of disease control of the overall study population was 3.4 months (95% CI, 1.4–5.3). These data illustrate that, excluding three patients in Cohort B, the majority of patients exhibited tumor shrinkage, supporting the potential efficacy of the treatment. The waterfall plot shows tumor shrinkage in all patients except for three in Cohort B, indicating tumor size reduction in the majority of cases. Among the 15 patients included in this study, 11 had received prior systemic therapy. The prior systemic treatments included atezolizumab plus bevacizumab in eight patients, lenvatinib in one patient, sorafenib in one patient, and ramucirumab in one patient. An iceberg plot illustrating the duration of the immediately preceding systemic therapy and the investigational treatment is shown in Fig. 1C. In this plot, the duration of prior systemic therapy is displayed below the baseline, and the treatment duration of the investigational therapy is displayed above the baseline. The plot includes patients with relatively short durations of prior systemic therapy who subsequently had longer durations of treatment with the investigational therapy.

### Tumor response analysis inside and outside the irradiation field

**Fig. 2:**
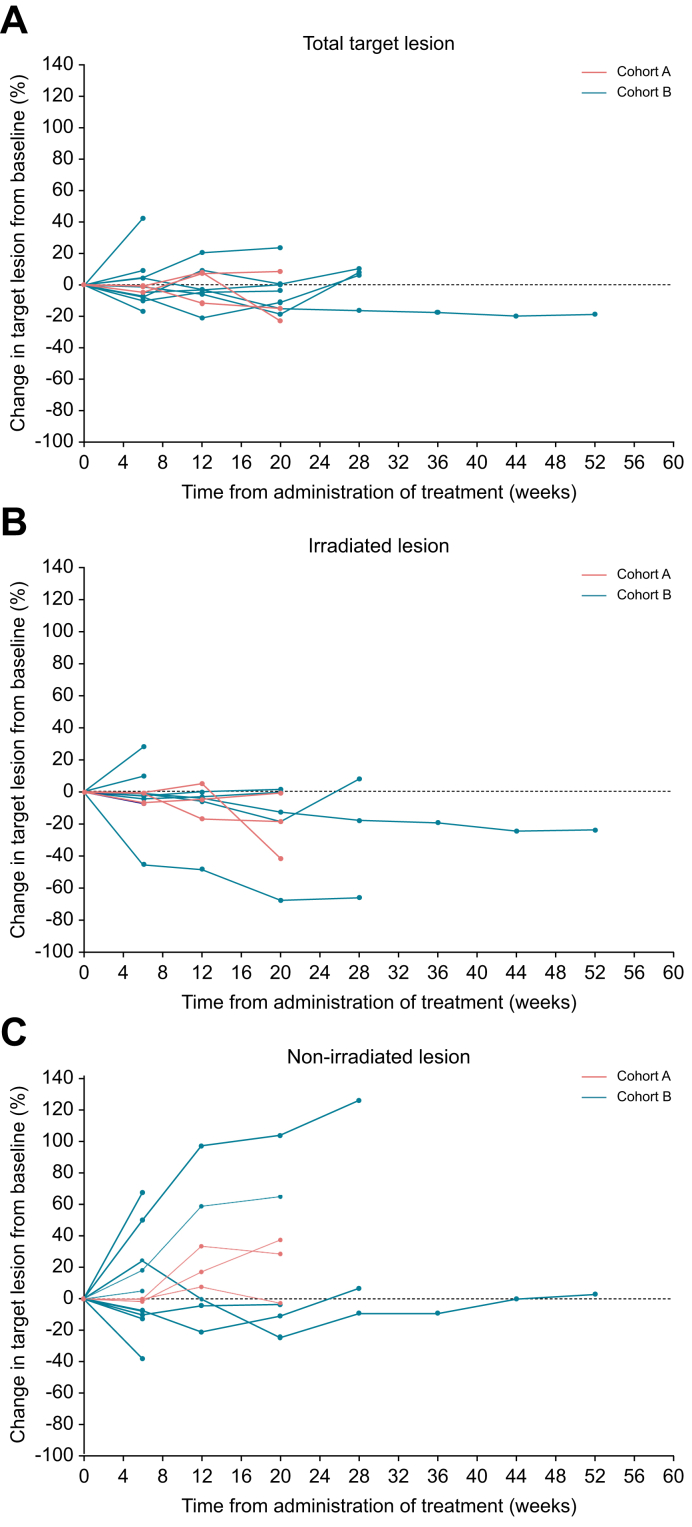
Spider plot showing changes in tumor measurements over time. (A) Sum of all target lesions. (B) MVI-positive lesion treated with C-ion RT. (C) Sum of lesions outside C-ion RT field. C-ion RT, carbon-ion radiotherapy; MVI, macrovascular invasion.

This exploratory analysis investigated tumor behavior inside and outside the C-ion RT field, highlighting significant differences in response. Fig. 2 presents a spider plot summarizing the overall changes in target lesion sizes during the study. Tumor imaging dynamics were compared between lesions located inside the irradiation field and those outside. Of the 11 patients who experienced progressive disease during follow-up, it was attributed to new lesions in eight individuals and target lesion growth in four.

No cases of rapid tumor enlargement were observed in lesions treated with C-ion RT. Tumor control was achieved in the majority of patients, with significant shrinkage shown in one individual. Based on our previously reported criteria for evaluation of MVI progression,^9^ the progression of MVI during the treatment period was evaluated. Regarding MVI progression itself, only one individual experienced progression during the treatment period. In contrast, non-irradiated lesions displayed more heterogeneous behavior. Significant tumor enlargement (≥+50%) was observed in several cases, while some lesions remained stable without noticeable growth over an extended period over 20 weeks. Importantly, no instances of substantial tumor shrinkage were observed among non-irradiated lesions.

### Prolonged pyrexia and clinical course of cytokine release syndrome

**Fig. 3:**
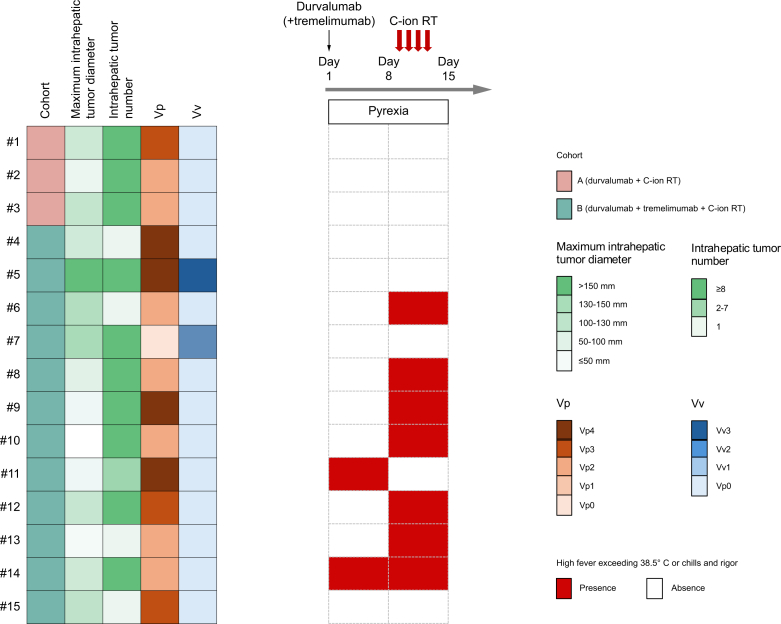
The characteristics of onset of pyrexia. No cases of pyrexia were observed in cohort A. In Cohort B, two patients developed a high fever of 38.5 °C or higher or chills within 7 days of starting durvalumab plus tremelimumab; six patients had a new onset of high fever after starting C-ion RT. C-ion RT, carbon ion radiotherapy.

The most common AE of the present study was pyrexia, which occurred primarily during the administration of the investigational treatment and during C-ion RT. The characteristics of onset of pyrexia is summarized in Fig. 3. Of note, no cases of pyrexia were observed in Cohort A, whereas six cases of pyrexia with chills and high fever occurred immediately after C-ion RT in Cohort B. As a *post-hoc* exploratory analysis, we compared PFS between patients who experienced pyrexia (n = 6) and those who did not (n = 9). Median PFS was 2.8 months (95% CI, 1.1–6.4) in the pyrexia group and 4.7 months (95% CI, 0.5–6.6) in the non-pyrexia group, with no significant difference observed (*p* = 0.204) (Fig. S6).

**Fig. 4:**
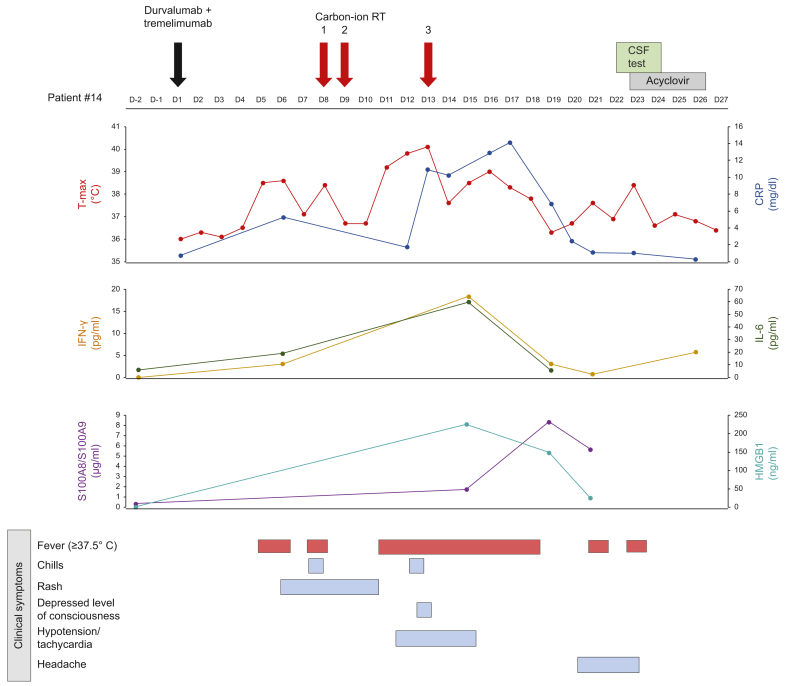
Case presentation of cytokine release syndrome (CRS). The patient experienced chills and fever following his first fraction of C-ion RT. On Day 13, after the third fraction, he developed tachycardia and hypotension. On Day 22, the patient had headache and rigidity. Inflammatory findings were found in the cerebrospinal fluid indicating meningeal involvement. Tests for infection were negative, so the patient was considered to have CNS symptoms associated with CRS. Exploratory analysis using the ELISA method revealed increases in IL-6, IFN-γ, and DAMPs (S100A8/S100A9 and HMGB1) during febrile episodes after C-ion RT, correlating with inflammatory dynamics. C-ion RT, carbon-ion radiotherapy; CNS, central nervous system; CRS, cytokine release syndrome; DAMPs: damage-associated molecular patterns.

One individual demonstrated a notable clinical course (Fig. 4). A man in his 50s with portal vein invasion (Vp2) was enrolled in the expansion cohort and developed cytokine release syndrome (CRS). After receiving durvalumab and tremelimumab on Day 1, he experienced chills and fever following his first fraction of C-ion RT. On Day 13, after the third fraction, he developed tachycardia and hypotension. Despite initial concerns about sepsis, negative cultures led to a CRS diagnosis, and he responded well to appropriate supportive therapy. Additionally, on Day 22, the patient had headache and rigidity, so a lumbar puncture was performed, which revealed inflammatory findings in the cerebrospinal fluid indicating meningeal involvement. Tests for bacterial and viral infection were negative, so the patient was considered to have central nervous system symptoms associated with CRS. Exploratory analysis using the ELISA method revealed increases in IL-6, IFN-γ, and damage-associated molecular patterns (DAMPs) (S100A8/S100A9 and HMGB1) during febrile episodes after radiotherapy, correlating with inflammatory dynamics. These findings support a potential relationship between C-ion RT and inflammatory cytokine activation when under the influence of immune checkpoint inhibitors (ICIs).

### Long-term biliary effects of C-ion RT

**Fig. 5:**
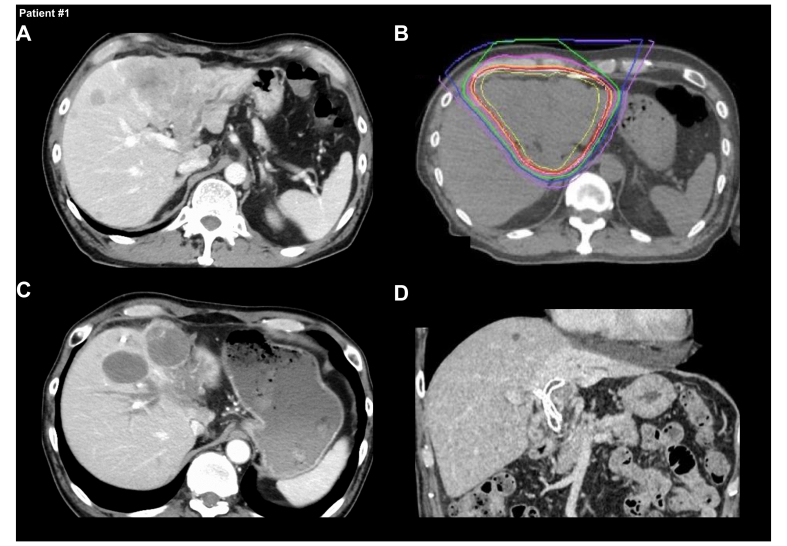
CT imaging of the case which developed intrahepatic bile duct dilatation during long-term observation. (A) CT scan imaging of baseline. (B) Dose distribution of C-ion RT superimposed on contrast-enhanced computed tomography (B). (C) Bile duct dilatation development. (D) After stenting (metallic stent) in the bile duct. C-ion RT, carbon-ion radiotherapy; CT, computed tomography.

In the post-trial observational study, intrahepatic bile duct strictures attributed to C-ion RT were identified as notable AEs. Of the four cases observed, all had tumors that extended beyond the hepatic hilum and were included in the radiation field. Over the long term, these patients developed intrahepatic bile duct dilatation. Three of these four cases required biliary drainage tube placement to manage the condition. Stent placement, performed under endoscopic retrograde cholangiopancreatography (ERCP), was effective in preserving liver function in these patients. Fig. 5 illustrates the imaging of the one case. Table S6 shows the size of nodules with MVI that received carbon-ion irradiation, and the proportion of tumors that were adjacent to the hepatic hilum or contained major portal vein invasion (Vp3, 4) in the four cases with bile duct dilatation and the 11 cases without. To further characterize the relationship between biliary events and the irradiation field, we assessed whether the hepatic hilum was included in the C-ion RT treatment field. C-ion RT involved the hepatic hilum in 12 of 15 patients (80%), among whom biliary strictures with distal bile duct dilatation developed in four patients (33% of those with hilar involvement). In contrast, no biliary strictures were observed in patients whose irradiation fields did not include the hepatic hilum. Radiotherapy treatment plans for all patients are shown in Fig. S5. The determination of whether tumors were adjacent to the hepatic hilum was based on a previously reported evaluation method.^32^

### RNA-sequencing analysis: insights into tumor microenvironment and treatment response

**Fig. 6:**
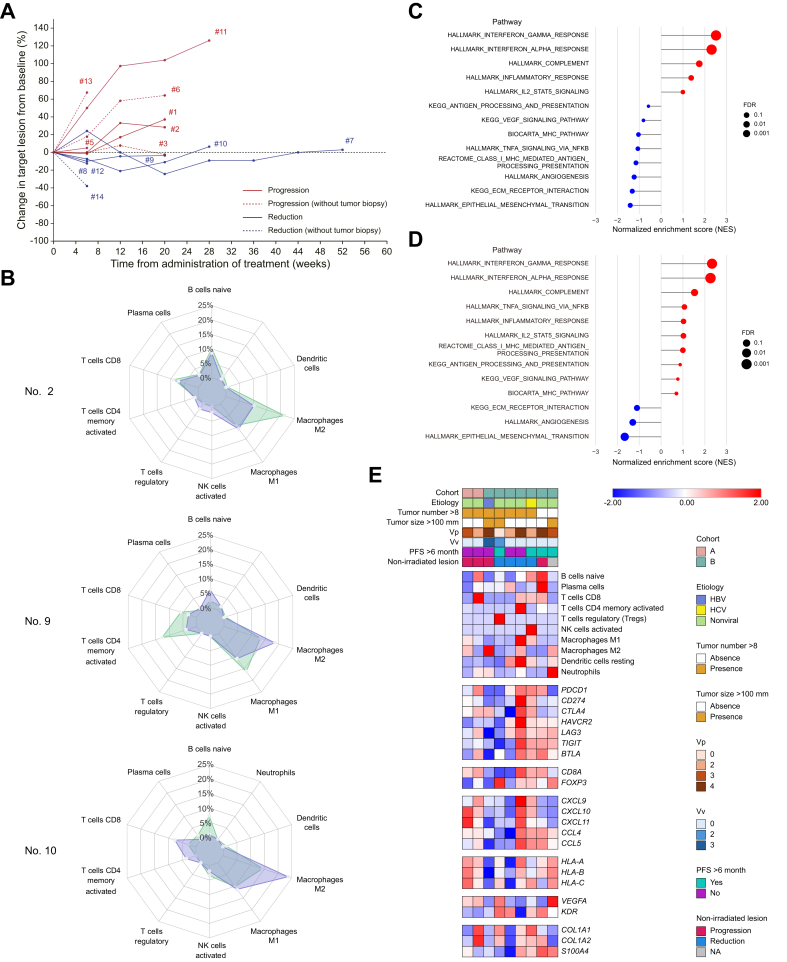
RNA-seq analysis of non-irradiated lesions and tumor microenvironment in patients receiving combined C-ion RT and immunotherapy. (A) Spider plot showing the dynamics of non-irradiated lesions. Patients were stratified into reduction (relative change ≤-5%) and progression groups (relative change >-5%) based on lesion size changes. (B) Radar charts comparing immune cell proportions between pre- and post-treatment biopsy specimens, as calculated by CIBERSORTx. Patient #2 in the progression group showed an increase in Treg proportions after treatment. (C) Lollipop plot displaying Gene Set Enrichment Analysis (GSEA) results comparing reduction and progression groups, highlighting differentially enriched pathways. (D) Lollipop plot showing GSEA results after excluding durvalumab monotherapy cases, comparing pathway enrichment between reduction and progression groups. (E) Heatmap of gene expression profiles from pretreatment biopsy specimens, illustrating the relationship between patient characteristics, treatment outcomes, and gene expression patterns. C-ion RT, carbon-ion radiotherapy; Treg, regulatory T cell.

RNA-sequencing was performed on biopsy specimens collected immediately before treatment initiation from nine patients enrolled in the trial, with follow-up analysis conducted on non-irradiated lesions from three patients at least 42 days after treatment initiation. To assess the impact of combining C-ion RT with immunotherapy while minimizing the influence of direct radiation effects, the analysis focused on non-irradiated lesions. Patients were stratified into reduction (relative change ≤-5%) and progression groups (relative change >-5%) based on lesion dynamics (Fig. 6A). Four patients were classified into the reduction group, four into the progression group, and one was unclassifiable due to the absence of measurable non-irradiated lesions. The analysis examined the relationship between tumor microenvironmental factors, PFS, and treatment outcomes. Immune cell proportions were evaluated using CIBERSORTx, with regulatory T cells (Tregs) assessed via FoxP3 expression. In post-immunotherapy biopsy specimens, one case in the progression group demonstrated an increase in Treg proportions and elevated FoxP3 expression. No consistent changes were observed in other immune cell populations (Fig. 6B). Gene Set Enrichment Analysis (GSEA) was conducted to identify pathways associated with treatment response. The reduction group showed enrichment of pathways related to inflammation, complement activation, which are often associated with immune activity. In contrast, the progression group exhibited enrichment of pathways linked to mesenchymal characteristics and angiogenesis, processes that may correlate with reduced therapy efficacy (Fig. 6C). After excluding cases treated with durvalumab monotherapy, the reduction group demonstrated enrichment of pathways involved in antigen presentation, suggesting enhanced immune recognition in responding patients (Fig. 6D). A heatmap (Fig. 6E) visualizes the gene expression profiles linked to these pathways, aligning immune cell dynamics with molecular alterations. This analysis highlights differences in tumor microenvironmental pathways between reduction and progression groups. Enrichment of inflammatory and antigen presentation pathways in non-irradiated lesions was associated with reduction, whereas mesenchymal and angiogenesis-related pathways were linked to progression.

## Discussion

The present study demonstrated that our novel therapeutic approach combining selective C-ion RT targeting MVI-containing tumors with durvalumab plus tremelimumab was well tolerated, with no DLTs observed and a manageable safety profile. A total of 15 patients were enrolled (Cohort A: durvalumab monotherapy, n = 3, Cohort B: durvalumab plus tremelimumab, n = 12) with a median PFS of 4.7 months and OS of 10.4 months. Considering that all patients in our cohort had MVI, which typically indicates a poor prognosis, these survival outcomes were encouraging. Although the median OS appeared longer in Cohort A (durvalumab monotherapy, 28.2 months) compared with Cohort B (durvalumab plus tremelimumab, 10.2 months), this observation should be interpreted with caution. The analysis is based on very small numbers, with imbalances in prior systemic therapy exposure and MVI status that may have influenced outcomes. Given the exploratory, non-comparative design of this study, the apparent OS difference should be considered hypothesis-generating rather than evidence of regimen-specific effects. In the HIMALAYA trial,^12^ which evaluated durvalumab plus tremelimumab and durvalumab monotherapy in a broader HCC group, the reported PFS durations were comparable to our findings. C-ion RT effectively controlled irradiated lesions, showing no rapid tumor enlargement and in some cases significant tumor shrinkage, while non-irradiated lesions showed variable responses, including progression in some patients. RNA-sequencing analysis revealed that enrichment of inflammatory and antigen presentation pathways correlated with favorable treatment outcomes, whereas mesenchymal and angiogenesis pathways were associated with resistance in unirradiated lesions.

From a safety perspective, a key finding of this study was the frequent occurrence of fever after C-ion RT administration following durvalumab, with or without tremelimumab. The significance of this observation is underscored by the fact that fever has rarely been reported as an AE in prospective studies of particle therapy, including C-ion RT. Additionally, a systematic review of toxicities associated with combined ICI and radiation therapy^33^ evaluated the toxicity profile of this combination and reported no notable adverse events in combined ICI and RT therapy compared to ICI monotherapy. Previous studies have suggested that high-linear energy transfer (LET) radiation, such as carbon ions, induces stronger immune responses compared to low-LET radiation such as X-rays or α-rays.^34^ Specifically, C-ion RT has been shown to cause greater upregulation of HMGB1, which promotes dendritic cell activation, and stronger induction of MHC-I compared with X-rays.^34^^,^^35^ These findings support our observation that the high frequency of fever in our study represents a distinctive response, and the fever pattern suggests a potential immune response induced by C-ion RT when administered after ICI therapy. Emerging evidence indicates that combining C-ion RT with ICIs is feasible. A phase Ib trial of durvalumab plus C-ion RT with cisplatin in cervical cancer showed good tolerability, with only one serious hypothyroidism case,^36^ and a retrospective analysis in melanoma reported grade ≥3 AEs in 21%, similar to rates with either therapy alone.^37^ These data support our finding that C-ion RT with ICIs can be delivered safely with a manageable profile.

Among long-term adverse events, bile duct strictures were observed in patients with tumors extending beyond the hepatic hilum within the radiation field. Three out of 15 cases required biliary drainage, with stent placement under ERCP preserving liver function. The causes of this stricture could include tumor infiltration, scarring of tumor tissue, normal tissue remodeling, or any combination of these factors, with ICIs augmenting the effects. According to past reports, intrahepatic bile duct stricture are rare.^22^^,^^38^ However, recent reports indicate that bile duct strictures may emerge as a late complication of C-ion RT. The frequency and severity of these strictures increase in perihilar-type tumors near the portal vein trunk, necessitating long-term rigorous follow-up.^32^ Our results did not confirm that the presence of Vp3 or Vp4 itself led to worsening bile duct dilatation. However, the occurrence of bile duct dilatation may have been influenced by the fact that many cases involved very large irradiated tumor diameters and tumor progression to the hepatic hilum. Further research is needed on dose planning for cases involving such tumor conditions. Whether caused by tumor infiltration or normal tissue remodeling remains unclear, but these findings highlight the importance of risk assessment, monitoring, and careful treatment planning for tumors near the hepatic hilum. In this context, and in contrast to previous reports describing vascular toxicities after short-course C-ion RT for pancreatic cancer,^39^^,^^40^ no irradiation field-related vascular AEs were observed in the present study, including portal vein stenosis or other clinically significant vascular complications. Given that vascular injury represents a critical determinant of liver function, particularly in treatments involving major vascular structures, the absence of such events provides important safety insights for this treatment strategy in patients with HCC.

Of particular interest was a case presenting with CRS complicated by meningitis following treatment. The CRS was accompanied by elevated levels of DAMPs, including S100A8/S100A9 and HMGB1, suggesting ICD as a potential trigger. In hematologic malignancies, CRS is a well-known complication following CAR-T cell therapy,^41^ which can be accompanied by immune effector cell-associated neurotoxicity syndrome (ICANS). The diagnosis of ICANS typically requires brain MRI and cerebrospinal fluid examination,^42^ with the underlying pathophysiology involving direct damage to the central nervous system by activated effector T cells and cytokines.^43^ The presentation of meningitis in our case, a manifestation rarely seen with ICI therapy alone, coupled with the pattern of immune activation, suggests that this may represent a case of ICANS. The combination of C-ion RT with ICIs appears to have induced distinct patterns of immune activation similar to those observed in CAR-T cell therapy. CRS we experienced was most likely related to the combination of C-ion RT and ICIs. Importantly, with appropriate monitoring and management, the patient's symptoms resolved, enabling hospital discharge. This case highlights the critical importance of rigorous systemic monitoring and proper management of immune-related adverse events when administering this combination therapy. As demonstrated in our trial, strict management of adverse events related to immune enhancement following C-ion RT for at least 7 days under continued hospitalization likely contributed to improved AE management.

The therapeutic efficacy analysis revealed a distinct pattern of responses. In this study, a dissociation was observed between local tumor control within the irradiation field and systemic disease control. Local tumor control was evaluated as an exploratory measure of local effectiveness rather than as a surrogate for systemic efficacy, although sustained control of vascular-invasive lesions may have clinical relevance in HCC with major vascular invasion. Although C-ion RT achieved strong tumor control within irradiated fields, limited antitumor effects were observed at non-irradiated sites, suggesting a potential lack of systemic responses such as abscopal effects, which have been reported in some radiotherapy-immunotherapy combinations.^44^ The clinical experience with radiotherapy-immunotherapy combinations has shown varying degrees of success across different cancer types in phase III trials. The PACIFIC trial (NCT02125461) demonstrated significant improvements in both PFS and OS when durvalumab was administered following concurrent chemoradiotherapy (CRT) in stage III non–small-cell lung cancer.^45^ Similarly, in esophageal and gastroesophageal junction cancers, nivolumab showed significant survival benefits when administered to patients who did not achieve pathological complete response after CRT,^46^ While large-scale trials predominantly support post-radiation ICI administration, as exemplified by the successful PACIFIC trial and esophageal cancer studies, emerging data specifically in HCC suggests potential benefits from alternative approaches. Recent smaller-scale studies have shown promising results with concurrent administration or radiation delivery after ICI initiation, suggesting that optimal sequencing strategies may be disease-specific.[47], [48], [49] In our study, while we did not observe clear synergistic effects, the combination of ICI and C-ion RT demonstrated promising additive effects with acceptable safety. Importantly, C-ion RT achieved effective control of MVI-containing tumors, consistent with several previous reports demonstrating the efficacy of particle beam therapy in treating MVI-positive HCC lesions.^32^^,^[50], [51], [52] The achievement of long-term survival in some patients with highly advanced HCC through this selective targeting approach warrants attention and further investigation. Given the extremely steep dose gradients of C-ion RT, accurate target definition was essential. In this study, irradiation was confined to MVI-containing tumors based on high-quality imaging and careful contouring, which likely contributed to the favorable local control observed in irradiated fields, in contrast to the limitations of photon-based SBRT in large or perihilar lesions. Future studies should focus on enhancing systemic efficacy through optimized treatment scheduling and potentially incorporating additional immunomodulatory agents, with particular attention to HCC-specific sequencing strategies that may differ from established paradigms in other cancer types.

This early-phase study primarily focused on safety and evaluated two treatment regimens: durvalumab plus C-ion RT and durvalumab plus tremelimumab plus C-ion RT. No DLTs were observed with either regimen, and TRAEs were generally manageable. An independent monitoring committee confirmed that no major safety concerns were identified. Based primarily on these safety findings, with preliminary efficacy signals regarded as supportive, the combination of durvalumab, tremelimumab, and C-ion RT is considered a tolerable regimen and a recommended phase II dose candidate for further clinical development.

In this phase Ib study, no clear abscopal effect was observed in non-irradiated lesions, consistent with the rarity of clinically meaningful abscopal responses.^53^ Several factors may account for this. Irradiation was confined to MVI-containing tumors, potentially limiting systemic antigen release and T-cell priming,^54^ and all patients had advanced disease with extensive MVI, where the immunosuppressive tumor microenvironment and large tumor burden likely constrained systemic immunity.[55], [56], [57] Although the high-LET properties of C-ion RT favor local control, the optimal fractionation and timing of ICI administration to elicit systemic responses remain undefined.^58^ Future strategies to enhance systemic activity in HCC may involve optimizing sequencing approaches, for example administering PD-1/PD-L1 blockade early after RT or initiating ICI followed by selective C-ion RT. Another promising avenue is broadening antigen release with multisite hypofractionated RT or using low-dose RT to inflame non-irradiated lesions and enhance ICI sensitivity.^59^^,^^60^ In addition, anti-VEGF–mediated vascular normalization may improve immune cell trafficking and drug delivery, thereby supporting RT–ICI synergy.^61^

This study examined tumor microenvironmental pathways using pretreatment and post-treatment samples, focusing on unirradiated lesions, to evaluate the systemic effects of combining ICIs with C-ion RT. Although the limited sample size precludes definitive conclusions, the results showed clear differences between the reduction and progression groups. Reduction was characterized by enrichment of inflammatory and antigen presentation pathways, findings consistent with previous reports linking these processes to ICI efficacy.^62^ Conversely, progression was associated with mesenchymal and angiogenic pathways, which have also been widely implicated as drivers of immune resistance and tumor progression.^63^

This study has several limitations. First, it was a small, phase Ib trial primarily designed to assess tolerability and safety. Accordingly, the analyses of PFS and OS are exploratory and should be interpreted with caution. The apparent OS difference between Cohorts A and B is also explained by the very small sample size, baseline imbalances, and the non-comparative design, and therefore should be considered hypothesis-generating rather than indicative of regimen-specific differences. In addition, enrollment was restricted to patients with advanced HCC and macrovascular invasion, a population with inherently poor prognosis, which further complicates cross-trial comparisons and interpretation of efficacy outcomes. Second, the RNA-sequencing results were derived from a limited subset of pretreatment biopsies and should be regarded as hypothesis-generating. They do not allow firm conclusions about combination-specific mechanisms or an abscopal effect, and no evidence was obtained that the treatment altered unfavorable pathways such as mesenchymal or angiogenic programs. To clarify potential synergistic effects, future studies will require larger cohorts with paired pre- and post-treatment samples, comparisons across radiation dose levels, and rational combinations such as the addition of VEGF inhibitors.

In conclusion, this study demonstrated the feasibility and safety of a novel therapeutic approach combining selective C-ion RT targeting MVI-containing tumors with ICIs in advanced HCC patients. Although C-ion RT achieved effective local control of MVI-positive lesions, a critical adverse prognostic factor, systemic immune responses in non-irradiated lesions remained limited, emphasizing the need for further optimization of combination strategies. Despite the small cohort, this proof of concept study provides a foundation for further investigation of this selective targeting approach, potentially offering a new treatment paradigm for advanced HCC patients with MVI.

## Abbreviations

AEs, adverse events; AFP, α-fetoprotein; BCLC, Barcelona Clinic Liver Cancer; C-ion RT, carbon-ion radiotherapy; CRS, cytokine release syndrome; CRT, chemoradiotherapy; DAMPs, damage-associated molecular patterns; DLT, dose-limiting toxicity; ECOG-PS, Eastern Cooperative Oncology Group performance status; EHM, extrahepatic metastasis; ERCP, endoscopic retrograde cholangiopancreatography; GSEA, Gene Set Enrichment Analysis; GTV, gross tumor volume; HCC, hepatocellular carcinoma; ICANS, immune effector cell-associated neurotoxicity syndrome; ICD, immunogenic cell death; ICIs, immune checkpoint inhibitors; LET, linear energy transfer; MVI, macrovascular invasion; ORR, objective response rate; OS, overall survival; PD-L1, programmed cell death ligand 1; PFS, progression-free survival; RBE, relative biological equivalent; RECIST, Response Evaluation Criteria in Solid Tumors; SAEs, serious adverse events; SBRT, stereotactic body radiotherapy; TACE, transarterial chemoembolization; TEAEs, treatment-emergent adverse events; TKIs, tyrosine kinase inhibitors; TRAEs, treatment-related adverse events; Tregs, regulatory T cells; TTP, time to progression; VEGF, vascular endothelial growth factor.

## Authors contributions

Drafted the manuscript: SO, KK. Designed the protocol: SO, KK, HM, MW. Primarily involved in and contributed to the procedures for conduct of the clinical trial: SO, KK, HM, MW, AT, HH. Performed statistical analyses for the clinical part of the study: YO, YI. Performed analyses for the exploratory part of the study: KK, MF, HK, SP, JC, JL, YH. Aided in the assessment and revisions of the protocol and manuscript: SY, MN, HK, KKobayashi, MI, MNakamura, NK, TK, SN, TK, SYamada, HI. Recruited and/or treated patients: SO, KK, HM, MW, SY, MN, HK, KKobayashi, MI, MNakamura, NKanogawa, TKondo. Created the automated RNA-seq analysis pipeline onto Astrocyte platform, hosted by the BioHPC supercomputing facility: SP, JC.

## Financial support

This phase Ib trial was supported by 10.13039/100004325AstraZeneca K.K. (Osaka, Japan; grant number ESR-19-20168), which provided funding and study drugs (durvalumab and tremelimumab). The trial operations were partially supported by management expense grants from the National Institutes for Quantum Science and Technology, Chiba, Japan. Analysis of tumor microenvironment using clinical specimens was supported by the Project 10.13039/501100020959Mirai Cancer Research Grants (operated by 10.13039/100019393Relay for Life) and JSPS KAKENHI (grant numbers JP22K08049 and JP23K27577) (to SO). This analysis was additionally supported by the 10.13039/100007914UT Southwestern Simmons Comprehensive Cancer Center (SCCC) grant P30CA142543 (to 10.13039/100006726JC and JL), U.S. National Institute of Health (R01CA233794, R01CA255621, R01CA282178, U01CA288375, U01CA283935, P30CA142543), European Commission (ERC-AdG-2020-101021417), and Cancer Prevention and Research Institute of Texas (RR180016, RP200554) (YH).

## Data availability

This article contains all of the data collected or analyzed during the course of this study. Raw data are not made publicly available because it would jeopardize patient privacy or consent, but deidentified raw data are available upon reasonable request. The RNA-sequencing dataset is publicly available at NCBI GEO (accession numbers: GSE287319). Further inquiries should be directed to the corresponding author.

## Declaration of generative AI and AI-assisted technologies in the writing process

During the preparation of this work the authors used ChatGPT in order to assist with English proofreading. After using this tool/service, the authors reviewed and edited the content as needed and take full responsibility for the content of the publication.

## Conflicts of interest

SO received honoraria from Bayer, Leverkusen, Germany; Eisai, Tokyo, Japan; Eli Lilly, Indianapolis, IN, USA; Chugai Pharma, Tokyo, Japan; AstraZeneca, Cambridge, UK; and Merck & Co., Inc., Kenilworth, NJ, USA; consulting or advisory fees from Bayer, Eisai, Merck & Co., Inc., Chugai Pharma, Eli Lilly, and AstraZeneca; and research grants from Bayer, AstraZeneca, and Eisai. KK received honoraria from Chugai Pharma, Tokyo, Japan, Eisai, Tokyo, Japan and AbbVie Inc. YH serves as an advisory for Helio Genomics, Espervita Therapeutics, Roche Diagnostics, and Elevar Therapeutics, and a shareholder for Alentis Therapeutics and Espervita Therapeutics. The other authors have no conflicts of interest to declare.

Please refer to the accompanying ICMJE disclosure forms for further details.

## Footnotes

Supplementary data to this article can be found online at https://doi.org/10.1016/j.jhepr.2026.101765.
