# Supplementary material for: MVI-targeted carbon-ion radiotherapy combined with immunotherapy for advanced hepatocellular carcinoma: Phase Ib DEPARTURE trial
Source: JHEP Rep. 2026 Feb 5;8(5):101765. doi: 10.1016/j.jhepr.2026.101765 (PMC13054414; doi:10.1016/j.jhepr.2026.101765)
Supplement: Multimedia component 1 [file mmc1.pdf]

MVI-targeted carbon-ion radiotherapy combined with Immunotherapy for advanced hepatocellular carcinoma: Phase Ib DEPARTURE trial

Sadahisa Ogasawara, Keisuke Koroki, Hirokazu Makishima, Masaru Wakatsuki, Asahi Takahashi, Makoto Fujiya, Sae Yumita, Miyuki Nakagawa, Hiroaki Kanzaki, Kazufumi Kobayashi, Masanori Inoue, Masato Nakamura, Naoya Kanogawa, Takayuki Kondo, Shingo Nakamoto, Tomoya Kurokawa, Yoshihito Ozawa, Yosuke Inaba, Soumith Paritala, Jingxuan Chen, Jeon Lee, Yujin Hoshida, Hideki Hanaoka, Shigeru Yamada, Hitoshi Ishikawa

Table of contents

Supplementary methods……………………………………………………………2

Supplementary tables…………………………………………………….……...7

Supplementary figures…………………………………………………………….16

Supplementary methods

This phase Ib, multicenter, open-label trial evaluates the safety and tolerability of a novel approach combining selective C-ion RT directed at MVI-containing tumors with immunotherapy in advanced HCC patients. Treatment arms consisted of durvalumab monotherapy (Cohort A) and durvalumab plus tremelimumab (Cohort B). The trial is conducted at two participating centers in Japan, Chiba University Hospital and QST Hospital.

Patients

Patients aged ≥20 years with histologically confirmed advanced HCC or a diagnosis based on typical hypervascular findings on CT or angiography were eligible. Additional requirements included macrovascular invasion, ECOG performance status 0–1, body weight >30 kg, Child-Pugh class A liver function, and adequate organ and bone marrow function. For the initial cohort, patients must have been refractory or intolerant to at least one prior systemic therapy, including atezolizumab plus bevacizumab, sorafenib, or lenvatinib. A life expectancy of at least 12 weeks and ineligibility for locoregional therapy were also required.

Patients were excluded if they had unresolved grade ≥2 toxicities (except alopecia and vitiligo), prior radiotherapy to >30% of bone marrow or involving the liver, major surgery within 28 days, history of organ transplantation, active primary immunodeficiency, or autoimmune disorders. Other exclusions included prior or current brain metastases, coinfection with hepatitis B and C or B and D viruses, immunosuppressive medication use within 14 days, and hypersensitivity to study drugs or excipients. Eligibility was determined by the principal or associate investigators based on Suppl. Table 1.

Treatment (including the procedure of C-ion RT)

C-ion RT (60 Gy in four fractions) began on day eight of cycle one. The gross tumour volume (GTV) for the radiotherapy were the intrahepatic nodules that formed MVI and the MVI itself.

Even when there were multiple intrahepatic nodules, only the tumors that formed MVI were irradiated. Clinical target volume was formed with a margin of 5mm from the GTV for the intrahepatic nodule and 10 mm along the portal / hepatic vein for the MVI. A fiducial marker was inserted adjacent to the target lesion for patient positioning. 4D-CT was used to determine respiratory motion. The data sets were then reconstructed and subdivided into 10 phases (T00: peak inhalation; T50: around exhalation). Combined with a interfractional margin of 3mm, T50 and phases below 3mm deviation from it were used to form a field-specific planning target volume (FTV) [22, 31]. Irradiation was done within these phases. C-ion RT was performed at a dose of 60 Gy / 4 fractions / week. The microdosimetric kinetic model [26] was used for calculations and all doses described in this report are relative biological equivalent weighted dose. Dose constraints for risk organs are as follows: gastrointestinal tract, D2cm^3 ≤ 30Gy; spinal cord: D_(max) ≤ 25 Gy; Residual liver volume (volume of the liver for which the dose is less than 30 Gy described as liver volume - V_{30}): 500 cm^3. The following liver volume information is also collected to accompany the residual liver volume: Liver V5 Gy and liver V20 Gy. Where dose constraints to the organ at risks and dose requirements for the FTV were in conflict, the dose to the risk organs was prioritized. These restrictions were determined based on previously published reports [16, 28–31]. Furthermore, cases where a metallic stent is present in the irradiation field are also excluded from C-ion RT treatment.

Tumor responses were evaluated via imaging (computed tomography or magnetic resonance imaging) at baseline and every six weeks. All complete and partial responses require confirmation by a subsequent scan at least four weeks after the initial response documentation.

Sample Size Determination, Statistical Analysis, and Definition of Dose-Limiting Toxicity

The study employs a modified 3+3 design to evaluate safety and tolerability, with a planned sample size of 15 patients, including the expansion cohort. We decided on the Rule-Based Dose-Escalation Design, 3+3 design, for this study because it fits the feasibility within the study period

and the need for sufficient safety data.

Grading of AEs follows the Common Terminology Criteria for Adverse Events (CTCAE) version 5.0. DLTs are defined as adverse events (AEs) at least possibly related to the treatment regimen, meeting the criteria outlined in Suppl. Table 2. Any treatment-related toxicity occurring during the DLT assessment period must be followed to resolution to determine whether it qualifies as a DLT, as specified in Suppl. Table 2.

Secondary efficacy endpoints include overall survival (OS), 6-month survival rate, objective response rate (ORR), progression-free survival (PFS) and time to progression (TTP). Survival analyses were conducted using the Kaplan-Meier method, with median survival times and their confidence intervals estimated using the Brookmeyer and Crowley method. All statistical analyses were performed using SAS for Windows, release 9.4 (SAS Institute, Cary, NC, USA).

Prospective Observation After Trial Completion

To assess long-term outcomes, we conducted a prospective observational study after trial completion with a data cutoff date of March 31, 2024. Overall survival was evaluated through regular follow-up visits, phone interviews, and medical records. Extended safety assessments included monitoring for AEs graded per CTCAE v5.0. Data on post-trial therapies, including systemic treatments, locoregional interventions, and supportive care, were analyzed to understand their impact on long-term survival.

Exploratory Biomarker Analysis with Tumor Biopsy Samples

For patients who provided additional informed consent, paired tumor biopsy samples were collected from non-irradiated liver lesions at baseline and 42 days after starting immunotherapy. RNA sequencing (RNA-seq) was conducted to investigate changes in the tumor

microenvironment, aiming to identify molecular signatures predictive of response or resistance to the combination therapy.

Total RNA was isolated from frozen tissue samples using the AllPrep DNA/RNA Mini Kit (Qiagen, CA, USA) according to the manufacturer's instructions. RNA-seq libraries were prepared using the SMART-Seq Stranded Kit (Takara Bio, Shiga, Japan) and sequenced on the NovaSeq 6000 system (Illumina, San Diego, CA, USA) using 150-bp paired-end reads. Raw reads in FASTQ format were trimmed using cutadapt (v. 2.5) with parameters "-q 20 --max-n 3 --cores=0 --minimum-length 75 -a "A{75}" -a "G{75}" -a "C{75}" -a "T{75}" -a Ill_Univ_Adapt". The trimmed reads were aligned to the human reference genome assembly GRCh38 (UCSC version hg38) using STAR (v. 2.7.3a) with parameters "--chimSegmentMin 15 --chimJunctionOverhangMin 15". Reads aligned to each gene annotated in GENCODE (v45) were quantified using featureCounts (v. 1.6.3). Normalized expression levels were calculated using the relative log expression (RLE) method implemented in the R package DESeq2 (v. 1.42.0). To ensure robust downstream analysis, filtering criteria were applied in R (v. 4.3.1) to remove lowly expressed and invariant genes. Specifically, genes with a coefficient of variation (CV) less than 0.01 across samples and those expressed in less than 10% of the samples were excluded from subsequent analyses. Gene Set Enrichment Analysis (GSEA) was performed using GenePattern (https://cloud.genepattern.org) against the Molecular Signatures Database (MSigDB v2024.1.Hs). The relative proportions of infiltrating immune cells were estimated using CIBERSORTx (https://cibersortx.stanford.edu) with the LM22 signature matrix (Supplementary CTAT table).

Data Management, Monitoring, and Ethics

Data management and monitoring are conducted in compliance with J-GCP guidelines. Case report forms are used to accurately record trial data, and monitors ensure adherence to the study protocol and regulatory standards. Independent audits are performed at the investigational sites to confirm quality control measures. A data monitoring committee, composed of clinical trial experts

and biostatisticians not involved in the study, evaluates trial data and assesses the safety and progress of the treatment cohorts.

All participants provided written informed consent before enrollment in the DEPARTURE trial. Ethics approval was obtained from the ethics committees of Chiba University Hospital and the National Institutes for Quantum and Radiological Science and Technology (approval numbers: 2020040 and C20-001). The study complies with the International Committee of Medical Journal Editors (ICMJE) guidelines for authorship. Results will be published in a peer-reviewed journal and registered on the Japan registry of clinical trials (jRCT). Amendments to the protocol are subject to approval by the institutional review boards. A follow-up study for long-term survival was approved by the Chiba University Hospital Ethics Committee (clinical trial No. HK202311-03).

Supplementary tables

Table S1. Eligibility criteria

Inclusion criteria

1. Capable of giving signed informed consent which includes compliance with the requirements and restrictions listed in the informed consent form (ICF) and in this protocol. Written informed consent and any locally required authorization obtained from the patient/legal representative prior to performing any protocol-related procedures, including screening evaluations. For patients aged <20 years and enrolling, a written informed consent should be obtained from the patient and his or her legally acceptable representative.
2. Age ≥20 years at time of study entry
3. Eastern Cooperative Oncology Group (ECOG) performance status of 0 or 1
4. Body weight >30 kg
5. Adequate normal organ and marrow function as defined below:
6. Haemoglobin ≥9.0 g/dL
7. Absolute neutrophil count (ANC) > 1500 per mm3
8. Platelet count ≥75 x 109/L (>75,000 per mm3)
9. Serum bilirubin ≤3.0 x institutional upper limit of normal (ULN)
10. AST (SGOT)/ALT (SGPT) ≤2.5 x institutional upper limit of normal unless liver metastases are present, in which case it must be ≤5x ULN
11. Measured creatinine clearance (CL) >40 mL/min or Calculated creatinine clearance CL>40 mL/min by the Cockcroft-Gault formula (Cockcroft and Gault 1976) or by 24-hour urine collection for determination of creatinine clearance
12. Evidence of post-menopausal status or negative urinary or serum pregnancy test for female pre-menopausal patients. Women will be considered post-menopausal if they have been amenorrheic for 12 months without an alternative medical cause. The following age-specific requirements apply:

    Women <50 years of age would be considered post-menopausal if they have been amenorrheic for 12 months or more following cessation of exogenous hormonal treatments and if they have luteinizing hormone and follicle-stimulating hormone levels in the post-menopausal range for the institution or underwent surgical sterilization (bilateral oophorectomy or hysterectomy).

    Women ≥50 years of age would be considered post-menopausal if they have been amenorrheic for 12 months or more following cessation of all exogenous hormonal treatments, had radiation-induced menopause with last menses >1 year ago, had chemotherapy-induced menopause with last menses >1 year ago, or underwent surgical sterilization (bilateral oophorectomy, bilateral salpingectomy or hysterectomy).

13. Patient is willing and able to comply with the protocol for the duration of the study including undergoing treatment and scheduled visits and examinations including follow up.
14. Advanced HCC confirmed histologically or by the typical findings of a hypervascular tumor on computed tomography or angiography
15. (Cohort A and Cohort B) Patients who have received at least one prior systemic chemotherapy regimen including atezolizumab bevacizumab combination, sorafenib, or lenvatinib and who are judged to be refractory or intolerant to standard therapy (not included in selection criteria in the expansion cohort).
16. Must not be eligible for locoregional therapy for unresectable HCC. For patients who progressed after locoregional therapy for HCC, locoregional therapy must have been completed ≥28 days prior to the baseline scan for the current study. Acceptable locoregional therapy for HCC are Ethanol Infusion Therapy, Radio Wave ablation Therapy, Transcatheter Arterial chemoembolization (TACE), Transcatheter arterial infusion (TAI). Hepatic Arterial Infusion Chemotherapy (HAIC) is not allowed.
17. Patients who have been diagnosed with HCC showing MVI. MVI is defined as a tumor thrombus in the major hepatic and/or portal vein branches (Vp2, Vp3, Vp4, Vv2, and Vv3) identified by imaging studies.
18. Child-Pugh A
19. Must have a life expectancy of at least 12 weeks

Exclusion criteria

1. Persons involved in the planning and conduct of this clinical trial (employees or staff of the sponsor and the site).
2. Patients who have participated in another clinical trial using the investigational drug within 28 days prior to obtaining consent, or who have received another investigational drug within 28 days prior to the first dose of the investigational drug in this study. The exception to this rule is if the patient is in an observational (non-interventional) clinical trial or during the follow-up period of an interventional trial.
3. Any unresolved NCI CTCAE grade ≥2 toxicity from previous anticancer therapy, with the exception of alopecia, vitiligo, and the laboratory values defined in the inclusion criteria.
4. Radiotherapy treatment to more than 30% of the bone marrow or with a wide field of radiation within four weeks of the first dose of the study drug
5. Major surgical procedure, as defined by the investigator, within 28 days prior to the first dose of IP
6. History of allogenic organ transplantation
7. Active or prior documented autoimmune or inflammatory disorders (including inflammatory bowel disease [e.g., colitis or Crohn's disease], diverticulitis [with the exception of diverticulosis], systemic lupus erythematosus, Sarcoidosis syndrome, or Wegener syndrome [granulomatosis with polyangiitis, Graves' disease, rheumatoid arthritis, hypophysitis, uveitis, etc.]). The following are exceptions to this criterion:
    - Patients with vitiligo or alopecia

- Patients with hypothyroidism (e.g., following Hashimoto syndrome) stable on hormone replacement
- Any chronic skin condition that does not require systemic therapy
- Patients without active disease in the last 5 years may be included but only after consultation with the study physician
- Patients with celiac disease controlled by diet alone

8. Uncontrolled intercurrent illness, including but not limited to, ongoing or active infection, symptomatic congestive heart failure, uncontrolled hypertension, unstable angina pectoris, cardiac arrhythmia, interstitial lung disease, serious chronic gastrointestinal conditions associated with diarrhea, or psychiatric illness/social situations that would limit compliance with study requirement, substantially increase risk of incurring AEs or compromise the ability of the patient to give written informed consent.
9. History of another primary malignancy except for
   - Malignancy treated with curative intent and with no known active disease ≥5 years before the first dose of IP and of low potential risk for recurrence
   - Adequately treated non-melanoma skin cancer or lentigo maligna without evidence of disease
   - Adequately treated carcinoma in situ without evidence of disease
10. History of leptomeningeal carcinomatosis
11. Prior or current brain metastases or spinal cord compression
12. Mean QT interval corrected for heart rate using Fridericia's formula (QTcF) ≥470 ms calculated from 3 ECGs (within 15 minutes at 5 minutes apart) Regardless of whether this criteria stays or not, all patients should have a baseline ECG
13. History of active primary immunodeficiency
14. Patients coinfected with hepatitis B and C viruses or with hepatitis B and D viruses
15. Current or prior use of immunosuppressive medication within 14 days before the first dose of durvalumab or tremelimumab
16. Patients who have received attenuated live attenuated vaccine within 30 days prior to the first dose of the investigational drug. Note: After incorporation, no live vaccination is allowed during the administration of the investigational drug and until 30 days after the last dose of the investigational drug.
17. Pregnant or lactating female patients, or male or female patients of reproductive potential who are unwilling to use an effective contraceptive method from screening until 90 days after the last dose of durvalumab monotherapy or 180 days after the last dose of durvalumab plus tremelimumab combination therapy.
18. Known allergy or hypersensitivity to any of the study drugs or any of the study drug excipients
19. Patients randomized or treated in a previous clinical trial using durvalumab and/or tremelimumab (regardless of the dose group to which they were assigned).
20. Patients who are determined by the investigator to be ineligible for participation, e.g., unlikely to comply with study procedures, restrictions, or requirements.
21. Patients who have been treated with anti-PD-1 antibody drugs, anti-PD-L1 antibody drugs, or other drugs acting on other stimulatory or co-inhibitory T-cell receptors and their

combinations (including atezolizumab and bevacizumab combination therapy) and have not tolerated the same therapy.

22. Prior radiotherapy involving the liver
23. Renal failure requiring hemodialysis or peritoneal dialysis
24. Presence of any severe cardiac disease
    - NYHA Class III or IV chronic heart failure
    - Current coronary artery disease or history of ischemic heart disease such as myocardial infarction within 6 months before the study
    - Serious arrhythmia (grade 3 or higher according to the CTCAE ver. 4.0: arrhythmia that cannot be controlled by oral medications or requires mechanical control).
25. Poorly controlled hypertension
26. Serious and active infection, excluding hepatitis virus infection
27. Persistent proteinuria of NCI-CTCAE version 5.0 grade ≥3; urine dipstick result of 3+ is allowed if protein excretion is <3.5 g/ 24 hours
28. Arterial or venous thrombotic or embolic events, such as cerebrovascular accident, deep vein thrombosis, or pulmonary embolism within six months before the start of the study medication
29. Refractory pleural effusion or ascites
30. History of hepatic encephalopathy within the past 12 months
31. Patients who are unable to take oral intake.
32. Patients who test positive for HIV antibodies.
33. Patients with pulmonary fibrosis or interstitial pneumonia.
34. Patients with serious complications such as severe psychiatric disorders, history of gastrointestinal bleeding, or active hemoptysis.
35. Other patients deemed ineligible by the attending physician.

Abbreviations: HCC, hepatocellular carcinoma; NCI CTCAE, National Cancer Institute Common Terminology Criteria for Adverse Events

Table S2. Criteria for DLT

A DLT will be defined as the occurrence of an adverse event (AE) that is at least possibly related with the investigational product (IP) or investigational regimen (IR), with the two following exceptions: any grade of vitiligo or alopecia. AEs that are at least possibly related with durvalumab- and/ or tremelimumab-containing regimens will be defined as DLTs if the following criteria are met:<br>If a patient initiated on C-ion RT is unable to complete the C-ion RT within the allowable time period because of AEs that cannot be ruled out as causally related with durvalumab, tremelimumab, or C-ion RT, the AEs will be considered as DLT.<br><br>**Hematologic toxicity:**<br>• Grade ≥3 neutropenia complicated by fever of >38.3 °C<br>• Grade 4 neutropenia lasting more than seven days<br>• Grade ≥3 thrombocytopenia with significant bleeding<br>• Grade 4 thrombocytopenia, regardless of duration<br>• Grade 4 anemia, regardless of duration<br><br>**Nonhematologic toxicity:**<br>• Any grade 4 nonimmune-mediated AE<br>• Any grade 4 immune-mediated AE, excluding endocrinopathies<br>• Any grade 3 nonimmune-mediated AE that does not resolve to grade ≤1 or baseline within 30 days of optimal medical management<br>• Any grade 3 immune-mediated AE, excluding diarrhea/ colitis, pneumonitis, hepatitis, rash, neurotoxicity, myocarditis, myositis/ polymyositis, endocrinopathies and nephritis, which does not resolve to grade ≤1 or baseline within 30 days after onset of the event despite optimal medical management, including systemic corticosteroids<br>• Grade 3 diarrhea or colitis that does not resolve to grade ≤1 within 14 days (both immune- and nonimmune-mediated; the same applies if not specified in the remaining bullet points below]<br>• Grade 3 noninfectious pneumonitis<br>• Grade 2 noninfectious pneumonitis that does not resolve to grade ≤1 within three days of initiation of maximal supportive care<br>• Aspartate aminotransferase (AST) or alanine aminotransferase (ALT) ≥5 × ULN or 5× the baseline, if the baseline is abnormal, with concurrent increase in total bilirubin (TBL) ≥3 × ULN or 3× the baseline, if the baseline is abnormal without evidence of cholestasis or alternative explanations, such as viral hepatitis, disease progression in the liver (i.e., Hy's Law)<br>• ALT or AST >8 × ULN or 8× the baseline, if the baseline is abnormal, or TBL >5 × ULN or 5× the baseline, if the baseline is abnormal<br>• Grade 3 immune-mediated rash that does not resolve to grade ≤1 or baseline within 30 days<br>• Grade 2 rash covering >30% BSA that does not resolve to grade ≤1 or baseline within 30 days<br>• Any grade of immune-mediated rash with bullous formation

- Grade 3 immune-mediated neurotoxicity, excluding Guillain–Barre and myasthenia gravis, that does not resolve to grade ≤1 within 30 days
- Grade 2 or 3 immune-mediated peripheral neuromotor syndrome, such as Guillain–Barre and myasthenia gravis, that does not resolve to grade ≤1 within 30 days or that exhibits signs of respiratory insufficiency or autonomic instability
- Grade 3 immune-mediated myocarditis
- Any symptomatic immune-mediated myocarditis that does not become asymptomatic within three days of initiating optimal medical management, including systemic corticosteroids
- Grade 2 or 3 immune-mediated myositis/ polymyositis that does not resolve to grade ≤1 within 30 days of initiating optimal medical management, including systemic corticosteroids, or that exhibits signs of respiratory insufficiency, regardless of optimal medical management
- Immune-mediated increase in creatinine >3 × ULN or >3 × the baseline for patients with baseline creatinine that is above the ULN
- Transfusion of red cell concentrate or platelet or use of G-CSF during the DLT period

Table S3. Representativeness of study participants

Cancer type	Hepatocellular Carcinoma (HCC)
Considerations related to:	
Sex	HCC is more common in men, accounting for 70–85% of all patients.
Race/Ethnicity	The age at diagnosis of HCC ranges from 50–70 years, although it varies by race, region, and background liver disease. Currently, the age of onset of HCC in Japanese is reported to be in the early 70s.
Geography	Worldwide, 830,000 people are diagnosed as liver cancer annually, with an age adjusted incidence rate of 10.1 per 100,000 population. Most of them are HCC. In Japan, about 37,000 were diagnosed with HCC and about 25,000 died due to HCC. The age adjusted incidence rate is 12 per 100,000 population in Japan.
Overall representativeness of this study	The subjects in the present study were considered comparable in age (median 69 years) compared to recent articles on clinical trials in Japanese patient populations.

Table S4. Grade ≥2 Treatment related AEs of whole study population, cohort A and B.

	Cohort<br>A: Durvalumab + C-ion RT<br>B: Durvalumab + Tremelimumab + C-ion RT								
	**Whole population (n = 15)**			**Cohort A (n = 3)**			**Cohort B (n = 12)**		
**Event, n (%)**	Grade			Grade			Grade		
	G2	G3	G4	G2	G3	G4	G2	G3	G4
Any event	10 (66.7)	4 (26.7)	1 (6.7)	1 (33.3)	0 (0)	0 (0)	9 (75.0)	4 (33.3)	1 (8.3)
Lipase increased	2 (13.3)	2 (13.3)	0 (0)	0 (0)	0 (0)	0 (0)	2 (16.7)	2 (16.7)	0 (0)
AST increased	2 (13.3)	0 (0)	0 (0)	1 (33.3)	0 (0)	0 (0)	1 (8.3)	0 (0)	0 (0)
ALT increased	2 (13.3)	0 (0)	0 (0)	1 (33.3)	0 (0)	0 (0)	1 (8.3)	0 (0)	0 (0)
Platelet count decreased	1 (6.7)	1 (6.7)	0 (0)	1 (33.3)	0 (0)	0 (0)	0 (0)	1 (8.3)	0 (0)
Abdominal pain	1 (6.7)	0 (0)	0 (0)	1 (33.3)	0 (0)	0 (0)	0 (0)	0 (0)	0 (0)
Amylase increased	2 (13.3)	0 (0)	0 (0)	0 (0)	0 (0)	0 (0)	2 (16.7)	0 (0)	0 (0)
Cytokine release syndrome	0 (0)	1 (6.7)	0 (0)	0 (0)	0 (0)	0 (0)	0 (0)	1 (8.3)	0 (0)
Diarrhea	1 (6.7)	1 (6.7)	0 (0)	0 (0)	0 (0)	0 (0)	1 (8.3)	1 (8.3)	0 (0)
Nausea	1 (6.7)	0 (0)	0 (0)	0 (0)	0 (0)	0 (0)	1 (8.3)	0 (0)	0 (0)
Stasis dermatitis	1 (6.7)	0 (0)	0 (0)	0 (0)	0 (0)	0 (0)	1 (8.3)	0 (0)	0 (0)
Dyshidrotic eczema	1 (6.7)	0 (0)	0 (0)	0 (0)	0 (0)	0 (0)	1 (8.3)	0 (0)	0 (0)
Interstitial lung disease	1 (6.7)	0 (0)	0 (0)	0 (0)	0 (0)	0 (0)	1 (8.3)	0 (0)	0 (0)
Decreased appetite	1 (6.7)	0 (0)	0 (0)	0 (0)	0 (0)	0 (0)	1 (8.3)	0 (0)	0 (0)
Meningitis	0 (0)	1 (6.7)	0 (0)	0 (0)	0 (0)	0 (0)	0 (0)	1 (8.3)	0 (0)
Enterocolitis	0 (0)	1 (6.7)	0 (0)	0 (0)	0 (0)	0 (0)	0 (0)	1 (8.3)	0 (0)
Hypoalbuminemia	1 (6.7)	0 (0)	0 (0)	0 (0)	0 (0)	0 (0)	1 (8.3)	0 (0)	0 (0)
Hypoxia	1 (6.7)	0 (0)	0 (0)	0 (0)	0 (0)	0 (0)	1 (8.3)	0 (0)	0 (0)
Mucosal inflammation	0 (0)	0 (0)	1 (6.7)	0 (0)	0 (0)	0 (0)	0 (0)	0 (0)	1 (8.3)
Pyrexia	1 (6.7)	0 (0)	0 (0)	0 (0)	0 (0)	0 (0)	1 (8.3)	0 (0)	0 (0)
Rash	2 (13.3)	0 (0)	0 (0)	0 (0)	0 (0)	0 (0)	2 (16.7)	0 (0)	0 (0)
Adrenal insufficiency	0 (0)	1 (6.7)	0 (0)	0 (0)	0 (0)	0 (0)	0 (0)	1 (8.3)	0 (0)
Dysgeusia	1 (6.7)	0 (0)	0 (0)	0 (0)	0 (0)	0 (0)	1 (8.3)	0 (0)	0 (0)

Table S5. Progression-free survival (RECIST v1.1), Time to progression (RECIST v1.1) and Overall survival based on subsequent follow-up data (data cutoff date of OS analysis was March 31, 2024).

		**Cohort**<br>**A: Dur + C-ion RT**<br>**B: Dur + Treme + C-ion RT**		**Major portal vein invasion (Vp3, 4)**	
	**Whole population (n = 15)**	**Cohort A (n = 3)**	**Cohort B (n = 12)**	**Absent (n = 8)**	**Present (n = 7)**
PFS, months (95% CI)	4.7 (1.4–6.4)	4.7 (4.6–4.7)	3.8 (1.1–6.6)	4.6 (1.1–6.4)	4.7 (0.5–6.6)
TTP, months (95% CI)	4.7 (1.3–6.4)	4.7 (4.6–4.7)	6.2 (1.1–6.6)	4.6 (0.1–0.8)	4.7 (0.1–0.8)
OS, months (95% CI), based on subsequent follow-up data	10.4 (5.6–15.2)	28.2 (5.5–50.9)	10.2 (4.3–16.1)	14.5 (4.9–NE)	9.5 (3.2–28.2)

Abbreviations: PFS, progression-free survival; OS, Overall survival; Dur, durvalumab; Treme, tremelimumab

Supplementary figures

Fig. S1. Dosing schedule of the study.

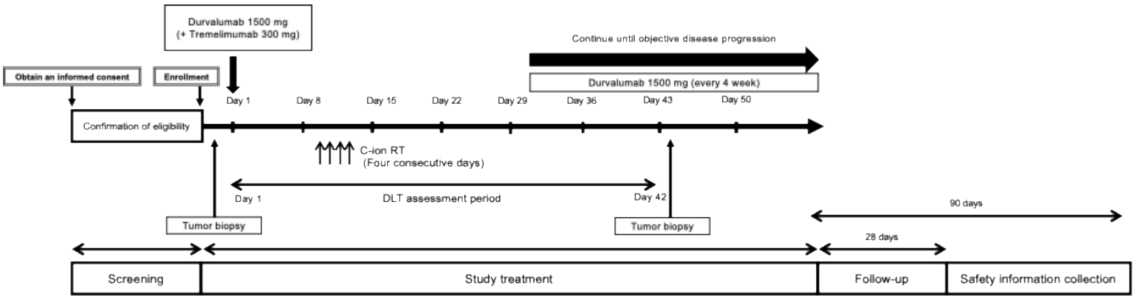

Patients were enrolled at two centers in Japan (Chiba University Hospital and QST Hospital) into two cohorts: durvalumab monotherapy (Cohort A) or durvalumab plus a single tremelimumab dose (Cohort B). C-ion RT (60 Gy in four fractions) begins on day 8 of Cycle 1, targeting MVI-containing intrahepatic lesions. DLTs are assessed during the 42-day evaluation period starting from the administration of durvalumab on Day 1 of Cycle 1. Study treatments continue until disease progression, according to RECIST ver. 1.1.

Abbreviations: C-ion RT, Carbon-ion radiotherapy; DLT, Dose-limiting toxicity

Fig. S2. Patient flow of the DEPARTURE trial

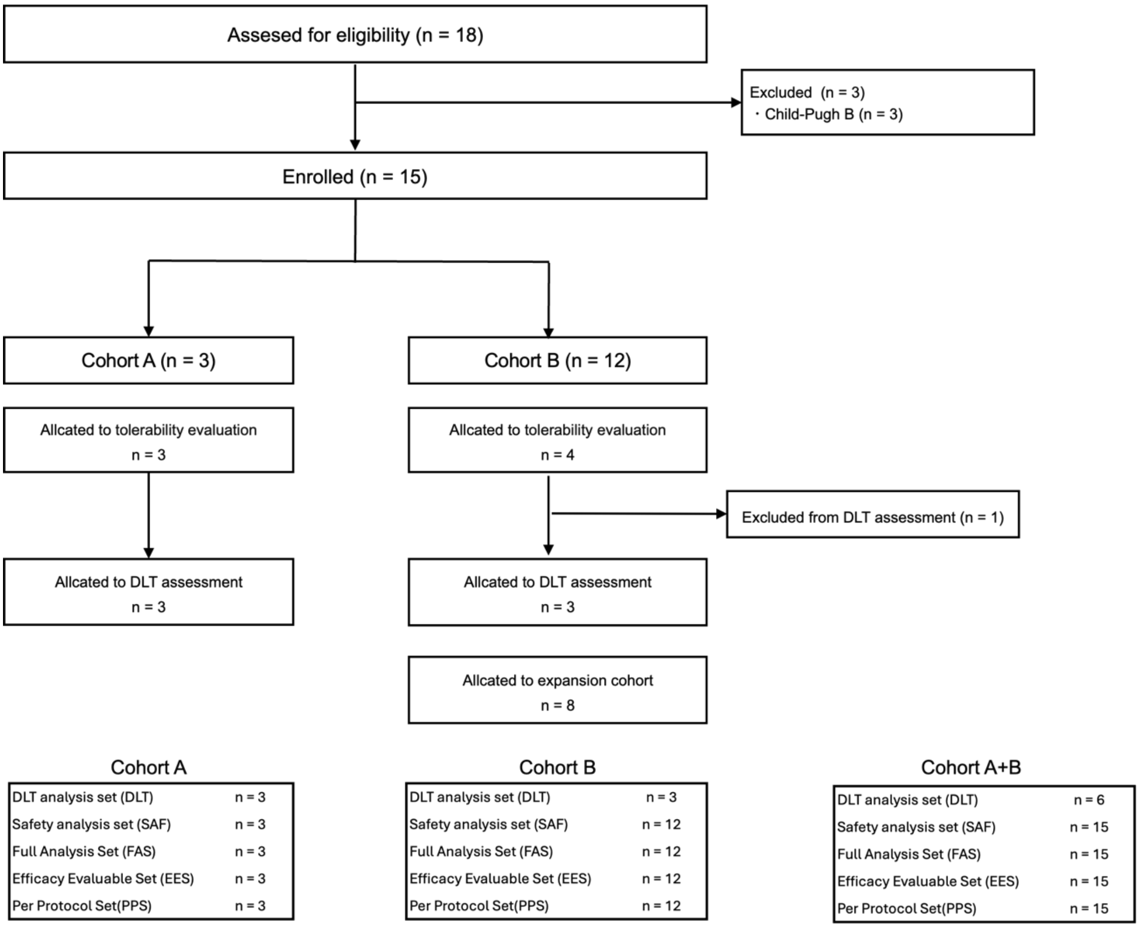

Informed consent was obtained from 18 patients, of whom 15 were enrolled. All 15 enrolled patients received the investigational treatment. In Cohort A, tolerability was assessed in 3 subjects, while in Cohort B, 12 patients were enrolled (4 for tolerability evaluation, 8 for the expansion cohort.

Abbreviation: DLT, Dose-limiting toxicity

Fig. S3. PFS (RECIST ver. 1.1) data of (A) whole population (n = 15), (B) stratified by cohort A and B, (C) stratified by major portal vein invasion (Vp3, 4).

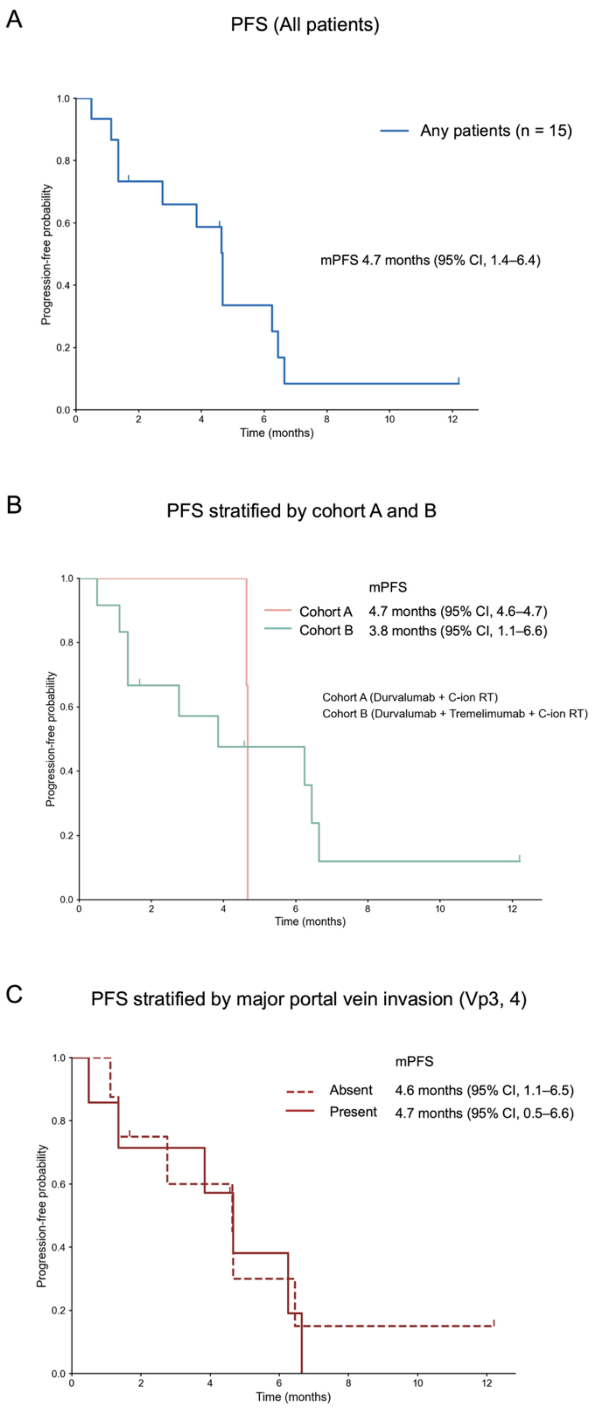

Abbreviations: PFS, Progression-free survival.

Fig. S4. Overall survival based on subsequent follow-up data (data cutoff date of OS analysis was March 31, 2024). (A) All patients (n = 15), (B) stratified by treatment cohort, (C) stratified by major portal vein invasion.

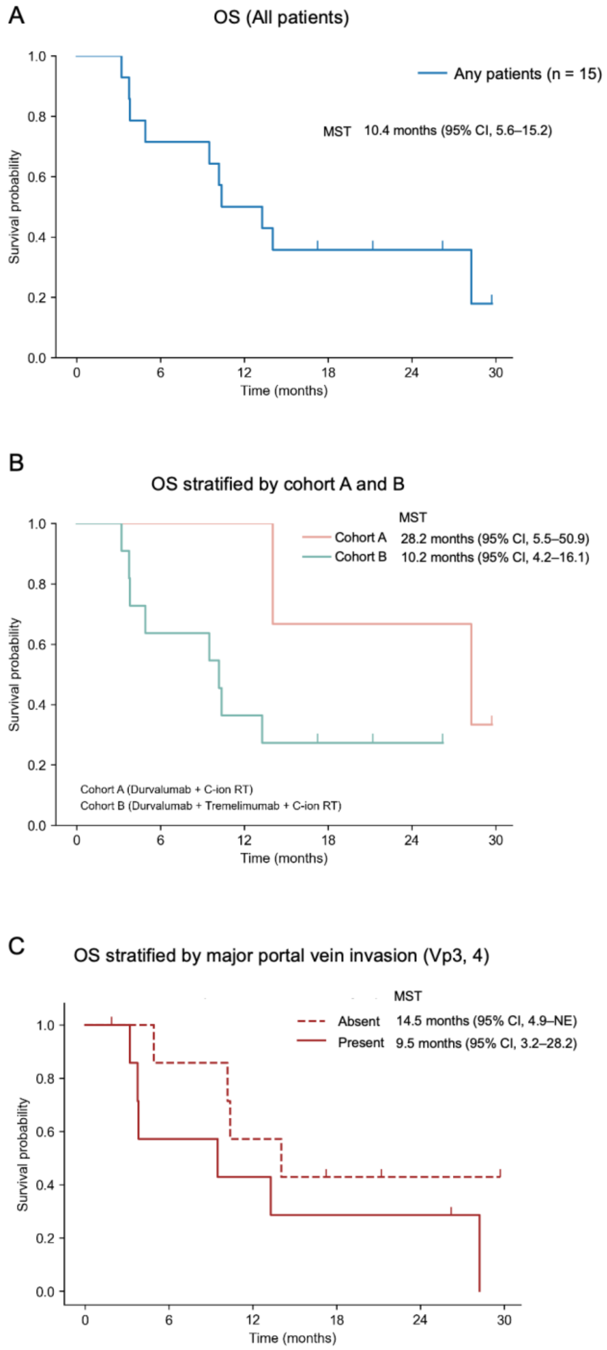

Abbreviations: OS, Overall survival; MST, median survival time

Supplementary Table 6. Tumor characteristics of lesions treated with C-ion RT. (A) Patient who experienced bile duct dilatation. (B) Patient without complications of bile duct.

A

No.	Cohort	Diameter of irradiated lesion		Perihilar type / Distal type		Major portal vein invasion (Vp3, 4)	
1	A	92.4 mm		Perihilar type		Present	
2	A	54.0 mm		Perihilar type		Absent	
7	B	135.0 mm		Perihilar type		Absent	
15	B	109.2 mm		Perihilar type		Present	
		Median diameter	100.8 mm	Rate of perihilar type	100.0 %	Rate of Vp3, 4	50.0 %

B

No.	Cohort	Diameter of irradiated lesion		Perihilar type / Distal type		Major portal vein invasion (Vp3, 4)	
3	A	102.8 mm		Distal type		Absent	
4	B	88.0 mm		Perihilar type		Present	
5	B	227.3 mm		Perihilar type		Present	
6	B	123.0 mm		Perihilar type		Absent	
8	B	77.5 mm		Perihilar type		Absent	
9	B	Diffuse		Perihilar type		Present	
10	B	Diffuse		Distal type		Absent	
11	B	86.7 mm		Perihilar type		Present	
12	B	99.4 mm		Perihilar type		Present	
13	B	44.4 mm		Distal type		Absent	
14	B	42.0 mm		Perihilar type		Absent	
		Median diameter	88.0 mm	Rate of perihilar type	72.7 %	Rate of Vp3, 4	45.5 %

Abbreviations. C-ion RT, Carbon-ion radiotherapy.

Fig. S5. Carbon-ion irradiation planning image. (A) Patient who experienced bile duct during follow-up period. (B) Patient without complications of bileduct during follow up period.

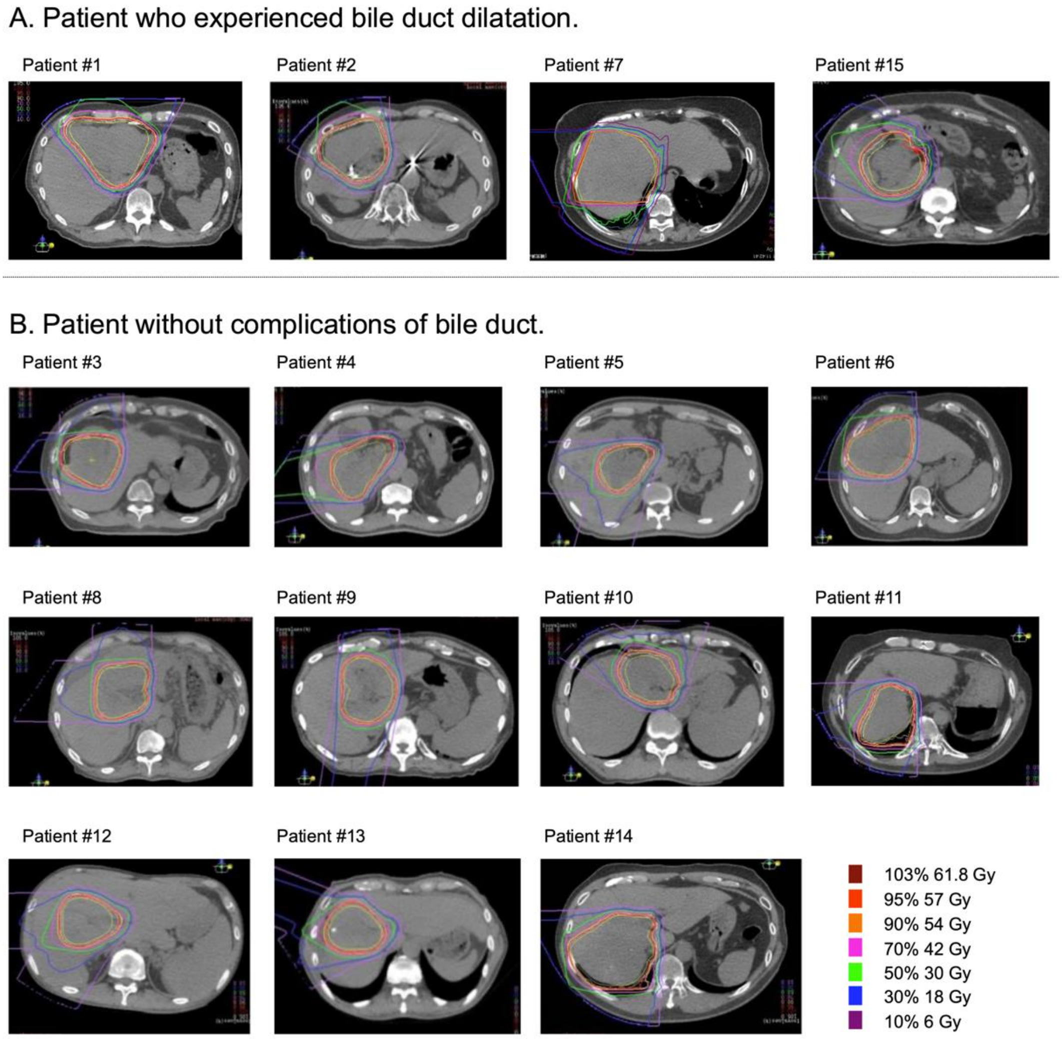

Fig. S6. PFS stratified by present or absent of High fever exceeding 38.5 ℃ or chills and rigor.

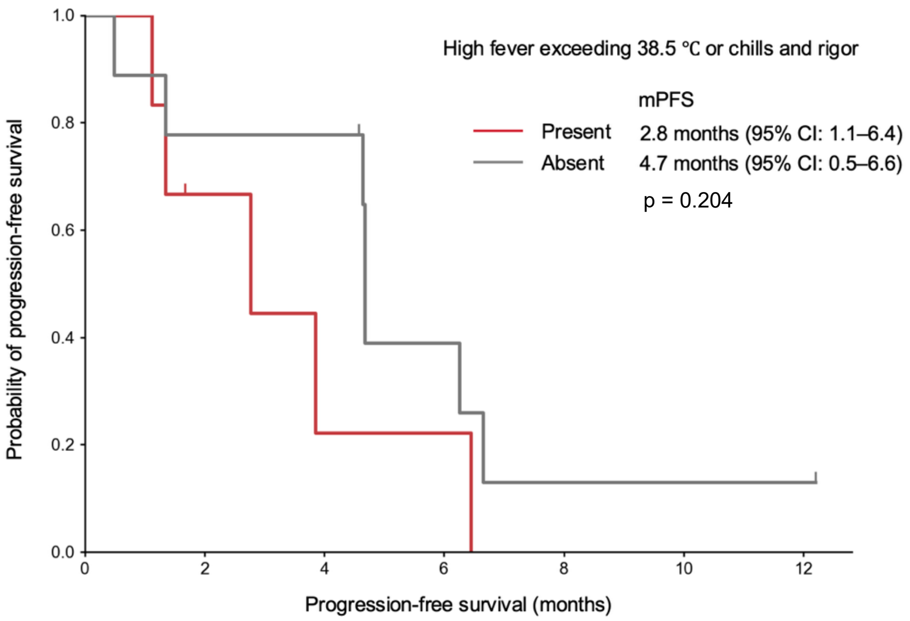

Abbreviations: PFS, Progression-free survival.
