# Supplementary material for: MVI-targeted carbon-ion radiotherapy combined with immunotherapy for advanced hepatocellular carcinoma: Phase Ib DEPARTURE trial
Source: JHEP Rep. 2026 Feb 5;8(5):101765. doi: 10.1016/j.jhepr.2026.101765 (PMC13054414; doi:10.1016/j.jhepr.2026.101765)
Supplement: Multimedia component 2 [file mmc2.docx]

**JHEP Reports**

**CTAT methods**

Tables for a “Complete, Transparent, Accurate and Timely account” (CTAT) are now mandatory for all revised submissions. The aim is to enhance the reproducibility of methods.

- Only include the parts relevant to your study
- Refer to the CTAT in the main text as ‘Supplementary CTAT Table’
- Do not add subheadings
- Add as many rows as needed to include all information
- Only include one item per row

**If the CTAT form is not relevant to your study, please outline the reasons why:**

|  |
| --- |

- 1. **Antibodies**

| **Name** | **Citation** | **Supplier** | **Cat no.** | **Clone no.** |
| --- | --- | --- | --- | --- |
| **N/A** |  |  |  |  |

- 1. **Cell lines**

| **Name** | **Citation** | **Supplier** | **Cat no.** | **Passage no.** | **Authentication test method** |
| --- | --- | --- | --- | --- | --- |
| **N/A** |  |  |  |  |  |

- 1. **Organisms**

| **Name** | **Citation** | **Supplier** | **Strain** | **Sex** | **Age** | **Overall n number** |
| --- | --- | --- | --- | --- | --- | --- |
| **N/A** |  |  |  |  |  |  |

- 1. **Sequence based reagents**

| **Name** | **Sequence** | **Supplier** |
| --- | --- | --- |
| **N/A** |  |  |

- 1. **Biological samples**

| **Description** | **Source** | **Identifier** |
| --- | --- | --- |
| **N/A** |  |  |

- 1. **Deposited data**

| **Name of repository** | **Identifier** | **Link** |
| --- | --- | --- |
| **NCBI GEO** | **GSE287319** | **https://www.ncbi.nlm.nih.gov/geo/query/acc.cgi?acc=GSE287319** |

- 1. **Software**

| **Software name** | **Manufacturer** | **Version** |
| --- | --- | --- |
| **SAS software** | **SAS Institute, Cary, NC, USA** | **ver.9.4** |
| **R** | **R Foundation for Statistical Computing** | **4.4.1** |
| **GenePattern** | **Broad Institute** | **3.9** |
| **FastQC** | **Babraham Bioinformatics** | **0.12.0** |
| **Trim Galore** | **Babraham Bioinformatics** | **0.6.5** |

- 1. **Other (*e.g*. drugs, proteins, vectors etc.)**

| **Durvalumab** | **Astrazeneca** |  |
| --- | --- | --- |
| **Tremelimumab** | **Astrazeneca** |  |
| **TNF-α, Human, ELISA Kit, Quantikine (96well)** | **R＆D** | **DTA00D** |
| **Human IFN-gamma Quantikine ELISA Kit** | **R＆D** | **DIF50C** |
| **HSP70 High Sensitivity ELISA kit** | **ENZO Life Sciences** | **ADI-EKS-715** |
| **HMGB1 human/mouse/rat ELISA kit** | **ARI** | **ARG81351** |
| **S100A/S100A9 Heterodimer Quanntikine ELISA kit** | **R＆D** | **DS8900** |

- 1. **Please provide the details of the corresponding methods author for the manuscript:**

| The corresponding methods author for this manuscript is:  Sadahisa Ogasawara, M.D., Ph.D.  Department of Gastroenterology, Graduate School of Medicine, Chiba University, 1-8-1  Inohana, Chuo-ku, Chiba 260-8670, Japan  Tel: +81-43-226-2083 (ext 72013)  Fax: +81-43-226-2088  Email: ogasawaras@chiba-u.jp |
| --- |

**2.0 Please confirm for randomised controlled trials all versions of the clinical protocol are included in the submission. These will be published online as supplementary information.**

|  |
| --- |
