# Supplementary material for: MVI-targeted carbon-ion radiotherapy combined with immunotherapy for advanced hepatocellular carcinoma: Phase Ib DEPARTURE trial
Source: JHEP Rep. 2026 Feb 5;8(5):101765. doi: 10.1016/j.jhepr.2026.101765 (PMC13054414; doi:10.1016/j.jhepr.2026.101765)
Supplement: Multimedia component 4 [file mmc4.zip › ClinicalTrial/consort-checklist_20260121_2215.pdf]

The CONSORT reporting checklist

For checking that reports of randomised trials can be understood and used by everyone

Note

If you have not used a reporting guideline before, read about how and why to use them and check whether CONSORT is the most applicable reporting guideline for your work.

Reporting guidelines are most useful when used early in research. When writing a manuscript or application, consider using the Full Guidance where you'll see explanations and examples for each item.

After writing, demonstrate adherence by completing this checklist:

1. Specify where each item is described (see Note 1).
2. Cite this checklist (See Note 2).
3. Include your completed checklist as a supplement when submitting to a journal so that future readers can use it to find information.

	Item Description	Location (or reason for not reporting)
**Title and Abstract**		
1a. Title	Identification as a randomised trial.	Not applicable because this study was not a randomised controlled trial.
1b. Structured Abstract	Structured summary of the trial design, methods, results, and conclusions.	Page 5
**Open Science**		
2. Trial Registration	Name of trial registry, identifying number (with URL) and date of registration.	jRCT, jRCT2031210046, (https://jrct.mhlw.go.jp/latest-detail/jRCT2031210046), 22nd, April, 2021
Protocol and statistical analysis plan	Where the trial protocol and statistical analysis plan can be accessed.	Uploaded as Supplementary data
4. Data sharing	Where and how the individual de-identified participant data (including data dictionary), statistical code and any other materials can be accessed.	Page 3, Line 26; Page 4, Lines 1–4
5. Funding and Conflicts of Interest		
5a. Funding	Sources of funding and other support (eg, supply of	Page 2, Lines 23–26; Page 3,

	drugs), and role of funders in the design, conduct, analysis, and reporting of the trial.	Lines 1–8
5b. Conflicts of interest	Financial and other conflicts of interest of the manuscript authors.	Page 2, Lines 12–21
**Introduction**		
6. Background and rationale	Scientific background and rationale.	Page 7–8
7. Objectives	Specific objectives related to benefits and harms.	Page 8–9
**Methods**		
8. Patient and public involvement	Details of patient or public involvement in the design, conduct and reporting of the trial.	Patients and the public were not involved in the design, conduct, reporting, or dissemination of this research.
9. Trial Design	Description of trial design including type of trial (eg, parallel group, crossover), allocation ratio, and framework (eg, superiority, equivalence, non-inferiority, exploratory).	Page 10–12
10. Changes to trial protocol	Important changes to the trial after it commenced including any outcomes or analyses that were not pre-specified, with reason.	Several protocol amendments were made after trial commencement; all versions of the trial protocol are available in the Supplementary Materials.
11. Trial Setting	Settings (eg, community, hospital) and locations (eg, countries, sites) where the trial was conducted.	Page 11, Lines 6–24
12. Eligibility Criteria		
12a. Participants	Eligibility criteria for participants.	Page 10 and Supplementary method.
12b. Other	If applicable, eligibility criteria for sites and for individuals delivering the interventions (eg, surgeons, physiotherapists).	Not applicable; no eligibility criteria were defined for study sites or intervention providers.
13. Intervention and comparator	Intervention and comparator with sufficient details to allow replication. If relevant, where additional materials describing the intervention and comparator (eg, intervention manual) can be accessed.	The intervention is described in detail in the “Patients and Methods (Page 9)”; no comparator was included because this was a single-arm trial.
14. Outcomes	Prespecified primary and secondary outcomes, including the specific measurement variable (eg, systolic blood pressure), analysis metric (eg, change from baseline, final value, time to event), method of aggregation (eg, median, proportion), and time point for each outcome.	Prespecified primary and secondary outcomes are described in the Methods section (Page 11).

15. Harms	How harms were defined and assessed (eg, systematically, non-systematically).	Dose-limiting toxicity (DLT) was predefined and assessed during a specified assessment period, as described in the Methods section (Page 11, Line 26; Page 12, Lines 1–10).
16. Sample Size		
16a. How sample size was determined	How sample size was determined, including all assumptions supporting the sample size calculation.	Supplementary Methods
16b. Interim analyses and stopping criteria	Explanation of any interim analyses and stopping guidelines.	No interim analysis will be conducted in this study.
17. Randomisation		
17a. Sequence Generation	Who generated the random allocation sequence and the method used.	Not applicable because this study was not a randomised controlled trial.
17b. Type of Randomisation	Type of randomisation and details of any restriction (eg, stratification, blocking, and block size).	Not applicable because this study was not a randomised controlled trial.
18. Allocation concealment mechanism	Mechanism used to implement the random allocation sequence (eg, central computer/telephone; sequentially numbered, opaque, sealed containers), describing any steps to conceal the sequence until interventions were assigned.	Not applicable because this study was not a randomised controlled trial.
19. Implementation	Whether the personnel who enrolled and those who assigned participants to the interventions had access to the random allocation sequence.	Not applicable because this study was not a randomised controlled trial.
20. Blinding		
20a. Who was blinded	Who was blinded after assignment to interventions (eg, participants, care providers, outcome assessors, data analysts).	No blinding was performed because of the open-label study design.
20b. How blinding was achieved	If blinded, how blinding was achieved and description of the similarity of interventions.	No blinding was performed because of the open-label study design.
21. Statistical methods		
21a. Comparing groups	Statistical methods used to compare groups for primary and secondary outcomes, including harms.	Not applicable because this study was not a randomised controlled trial.
21b. Definition of who is included in each analysis	Definition of who is included in each analysis (e.g., all randomised participants), and in which group.	Definitions of analysis populations are provided in the Statistical Analysis Plan, available in the Supplementary

		Materials.
21c. Missing Data	How missing data were handled in the analysis.	Described in the Statistical Analysis Plan (Supplementary Materials).
21d. Additional Analyses	Methods for any additional analyses (eg, subgroup and sensitivity analyses), distinguishing pre-specified from post hoc.	Page 14
22. Participant flow, including flow diagram		
22a. Participant Numbers	For each group, the numbers of participants who were randomly assigned, received intended intervention, and were analysed for the primary outcome.	Participant numbers are reported in the Results section, and a participant flow diagram is provided (supplementary data).
22b. Losses and exclusions	For each group, losses and exclusions after randomisation, together with reasons.	In this non-randomised study, losses and exclusions are reported in the participant flow diagram, together with reasons (supplementary data).
23. Recruitment		
23a. Dates	Dates defining the periods of recruitment and follow-up for outcomes of benefits and harms.	Described in the supplementary Appendix (Page 4).
23b. Reasons for stopping recruitment	If relevant, why the trial ended or was stopped.	Not applicable; the trial was completed as planned after reaching the target sample size.
24. Intervention and comparator delivery		
24a. As Administered	Intervention and comparator as they were actually administered (eg, where appropriate, who delivered the intervention/comparator, whether participants adhered, whether they were delivered as intended (fidelity)).	The intervention was delivered as intended with appropriate monitoring, as reported in the supplementary appendix (Page 5).
24b. Concomitant Care	Concomitant care received during the trial for each group.	Not applicable; this was a non-randomised study.
25. Baseline Data	A table showing baseline demographic and clinical characteristics for each group.	Table 1
26. Numbers analysed, outcomes, and estimation	For each primary and secondary outcome, by group:<br>• the number of participants included in the analysis.<br>• the number of participants with available data	Page 13–15

	at the outcome time point.<br>• result for each group, and the estimated effect size and its precision (such as 95% confidence interval).<br>• for binary outcomes, presentation of both absolute and relative effect size.	
27. Harms	All harms or unintended events in each group.	Page 13–14
28. Ancillary Analyses	Any other analyses performed, including subgroup and sensitivity analyses, distinguishing pre-specified from post hoc.	Page 14
**Discussion**		
29. Interpretation	Interpretation consistent with results, balancing benefits and harms, and considering other relevant evidence.	Page 19
30. Limitations	Trial limitations, addressing sources of potential bias, imprecision, generalisability, and, if relevant, multiplicity of analyses.	Page 24, Lines 10–15

1 How to specify where content is

Tell the reader where they can find information. E.g.,

- Results; paragraph 2
- Methods, Participants; paragraphs 1 & 2.
- Table 3
- Supplement B, para. 4

If you have chosen not to describe an item, explain why. You can do this in the checklist, or as a note below it.

You can describe items in the article body, or in tables, figures, or supplementary materials, and should prioritize items you feel are most important to your intended audience. The order of items in your manuscript does not need to match the order of items in this checklist. You can decide how best to structure your work.

2 How to cite

Describe how you used CONSORT at the end of your Methods section, referencing the resources you used e.g.,

> ‘We used the CONSORT reporting guideline(1) to draft this manuscript, and the CONSORT reporting checklist(2) when editing, included in supplement A’

If you use a reporting checklist, remember to include it as a supplement when publishing so that readers can easily find information and see how you have interpreted the guidance.

1. Hopewell S, Chan AW, Collins GS, Hróbjartsson A, Moher D, Schulz KF, et al. CONSORT 2025 statement: Updated guideline for reporting randomised trials. The BMJ [Internet]. 2025 Apr;389:e081123. Available from: https://www.ncbi.nlm.nih.gov/pmc/articles/PMC11995449/

2. Hopewell S, Chan AW, Collins GS, Hróbjartsson A, Moher D, Schulz KF, et al. The CONSORT reporting checklist. In: Harwood J, Albury C, Beyer J de, Schlüssel M, Collins G, editors. The EQUATOR network reporting guideline platform [Internet]. The UK EQUATOR Centre; 2025. Available from: https:/resources.equator-network.org/reporting-guidelines/consort/consort-checklist.docx
