# Supplementary material for: MVI-targeted carbon-ion radiotherapy combined with immunotherapy for advanced hepatocellular carcinoma: Phase Ib DEPARTURE trial
Source: JHEP Rep. 2026 Feb 5;8(5):101765. doi: 10.1016/j.jhepr.2026.101765 (PMC13054414; doi:10.1016/j.jhepr.2026.101765)
Supplement: Multimedia component 4 [file mmc4.zip › ClinicalTrial/IRB_approval_ChibaUniv_20210317.pdf]

(医) 書式5

整理番号	2020040
区分	■治験<br>■医薬品　□医療機器　□再生医療等製品

CHIBA UNIVERSITY

西暦　2021　年　3　月　15　日

治験審査結果通知書

実施医療機関の長
千葉大学医学部附属病院　病院長　殿

治験審査委員会
千葉大学医学部附属病院
千葉市中央区亥鼻1-8-1
委員長　花澤　豊行　㊞

審査依頼のあった件についての審査結果を下記のとおり通知いたします。

記

被験薬の化学名又は識別記号	MEDI4736/Tremelimumab	治験実施計画書番号	
治験課題名	脈管浸潤を伴う進行肝細胞癌患者を対象としたデュルバルマブ・トレメリムマブと重粒子線治療との併用療法の安全性と有効性を評価する第Ⅰb相臨床試験		
審査事項（審査資料）	■治験の実施の適否　（治験実施申請書（西暦2021年2月25日付 (医) 書式3））<br>□治験の継続の適否<br>□重篤な有害事象等に関する報告書<br>（□医薬品治験（西暦　年　月　日付 (医) 書式12））<br>（□医療機器治験（西暦　年　月　日付 (医) 書式14））<br>（□再生医療等製品治験（西暦　年　月　日付 (医) 書式19））<br>□安全性情報等<br>（□安全性情報等に関する報告書（西暦　年　月　日付 (医) 書式16））<br>（□安全性情報等に関する報告書（西暦　年　月　日付 (医) 書式16））<br>□治験に関する変更<br>（□治験に関する変更申請書（西暦　年　月　日付 (医) 書式10））<br>（□治験に関する変更申請書（西暦　年　月　日付 (医) 書式10））<br>□緊急の危険を回避するための治験実施計画書からの逸脱<br>（緊急の危険を回避するための治験実施計画書からの逸脱に関する報告書<br>（西暦　年　月　日付 (医) 書式 8））<br>□継続審査<br>（治験実施状況報告書（西暦　年　月　日付 (医) 書式11））<br>□その他（　　　　　　　　　）		
審査区分	■委員会審査（審　査　日：西暦　2021　年　3　月　15　日）<br>□迅速審査　（審査終了日：西暦　　　年　　月　　日）		
審査結果	□承認　■修正の上で承認　□却下　□既承認事項の取り消し　□保留		
「承認」以外の場合の理由等	治験届提出後のPMDAによる調査期間（2週間程度）及びその対応を考慮した治験実施期間となるように修正すること。また、治験実施計画書の治験実施期間、症例登録期間について年月日までの記載に修正すること。		
備考			

西暦　2021　年　3　月　17　日

自ら治験を実施する者　加藤　直也　　殿

千大院研第　111　号

申請のあった治験に関する審査事項について上記のとおり決定しましたので通知いたします。

実施医療機関の長　横手　幸太郎　㊞

注）安全性情報等について、治験審査委員会が実施医療機関の長及び自ら治験を実施する者に同時提出する場合は、本書式は治験審査委員会が作成し、書式下部の通知日は使用せず、実施医療機関の長欄には"該当せず"と記載する。同時に提出しない場合及び安全性情報等以外の審査事項については、本書式は治験審査委員会が作成し、実施医療機関の長に提出する。治験審査委員会の決定と実施医療機関の長の指示が同じである場合には、実施医療機関の長は、書式下部に通知日及び実施医療機関の長欄を記載し、自ら治験を実施する者に提出する。異なる場合には (医) 参考書式1を使用する。

(医)書式5

整理番号	2020040

西暦 2021 年 3 月 15 日

治験審査委員会委員出欠リスト

氏名	職業、資格及び所属	委員区分	出欠	備考
淺沼　克彦	腎臓内科 教授　医師	④	○	
瀧口　裕一	腫瘍内科 教授　医師	④	○	
伊豫　雅臣	精神神経科 教授　医師	④	○	
大鳥　精司	整形外科 教授　医師	④	○	
花澤　豊行	耳鼻咽喉・頭頸部外科 教授　医師	④	○	委員長
池田　純一郎	病理部 教授　医師	④	○	
花岡　英紀	臨床試験部 教授　医師	④	―	
石井　伊都子	薬剤部 教授　薬剤師	④	○	副委員長
菊田　直美	看護部 副部長　看護師	④	○	
青木　浩子	大学院社会科学研究院 教授	①②③	○	
岡林　伸幸	大学院社会科学研究院 教授	①②③	○	
岡田　忍	看護学部 教授　保健師	②③	○	
大塚　隆司	研究推進課 課長	①	○	
鈴木　庸夫	一般の立場から意見を述べることのできる者（鈴木庸夫法律事務所　弁護士）	①②③	○	
丸　祐一	一般の立場から意見を述べることのできる者（鳥取大学　教授）	①②③	○	

注）委員区分については以下の区分により番号で記載する。
①非専門委員
②実施医療機関と利害関係を有しない委員（①に定める委員を除く）
③治験審査委員会の設置者と利害関係を有しない委員（①に定める委員を除く）
④①～③以外の委員
また、出欠については以下の区分により記号で記載する。
○（出席し、かつ当該治験に関与しない委員）
－（出席したが、当該治験に関与するため審議及び採決に不参加の委員）
×（欠席した委員）

本治験審査委員会は、本治験審査委員会の標準業務手順書及び「医薬品の臨床試験の実施の基準に関する省令」（平成9年厚生省令第28号）、「医療機器の臨床試験の実施の基準に関する省令」（平成17年厚生労働省令第36号）、「再生医療等製品の臨床試験の実施の基準に関する省令」（平成26年厚生労働省令第89号）に従って組織され、活動していることを確認し、保証いたします。
