# Supplementary material for: MVI-targeted carbon-ion radiotherapy combined with immunotherapy for advanced hepatocellular carcinoma: Phase Ib DEPARTURE trial
Source: JHEP Rep. 2026 Feb 5;8(5):101765. doi: 10.1016/j.jhepr.2026.101765 (PMC13054414; doi:10.1016/j.jhepr.2026.101765)
Supplement: Multimedia component 4 [file mmc4.zip › ClinicalTrial/IRB_approval_QSThospital_20210330.pdf]

（医）書式5

整理番号	C20-001
区分	■治験
	■医薬品　□医療機器　□再生医療等製品

西暦2021年3月26日

治験審査結果通知書

実施医療機関の長
国立研究開発法人　量子科学技術研究開発機構
量子医学・医療部門　部門長　殿

治験審査委員会
量子科学技術研究開発機構　臨床研究審査委員会
千葉県千葉市稲毛区穴川4-9-1
早川　和重

審査依頼のあった件についての審査結果を下記のとおり通知いたします。

記

被験薬の化学名又は識別記号	デュルバルマブ、トレメリムマブ	治験実施計画書番号	CCRC2002
治験課題名	脈管浸潤を伴う進行肝細胞癌患者を対象としたデュルバルマブ・トレメリムマブと重粒子線治療との併用療法の安全性と有効性を評価する第Ｉb相臨床試験		
審査事項（審査資料）	■治験の実施の適否　（治験実施申請書（西暦2021年2月25日付(医)書式3)）<br>□治験の継続の適否<br>□重篤な有害事象等に関する報告書<br>（□医薬品治験（西暦　年　月　日付(医)書式12)）<br>（□医療機器治験（西暦　年　月　日付(医)書式14)）<br>（□再生医療等製品治験（西暦　年　月　日付(医)書式19)）<br>□安全性情報等<br>（□安全性情報等に関する報告書（西暦　年　月　日付(医)書式16)）<br>（□安全性情報等に関する報告書（西暦　年　月　日付(医)書式16)）<br>□治験に関する変更<br>（□治験に関する変更申請書（西暦　年　月　日付(医)書式10)）<br>（□治験に関する変更申請書（西暦　年　月　日付(医)書式10)）<br>□緊急の危険を回避するための治験実施計画書からの逸脱<br>（緊急の危険を回避するための治験実施計画書からの逸脱に関する報告書<br>（西暦　年　月　日付(医)書式 8)）<br>□継続審査<br>（治験実施状況報告書（西暦　年　月　日付(医)書式11)）<br>□その他（　　　　）		
審査区分	■委員会審査（審　査　日：西暦　2021年　3月　24日）<br>□迅速審査　（審査終了日：西暦　年　月　日）		
審査結果	■承認　□修正の上で承認　□却下　□既承認事項の取り消し　□保留		
「承認」以外の場合の理由等			
備考			

西暦2021年3月30日

自ら治験を実施する者　若月　優　　殿

申請のあった治験に関する審査事項について上記のとおり決定しましたので通知いたします。

実施医療機関の長
国立研究開発法人　量子科学技術研究開発機構
量子医学・医療部門　部門長

注）安全性情報等について、治験審査委員会が実施医療機関の長及び自ら治験を実施する者に同時提出する場合は、本書式は治験審査委員会が作成し、書式下部の通知日は使用せず、実施医療機関の長欄には“該当せず”と記載する。同時に提出しない場合及び安全性情報等以外の審査事項については、本書式は治験審査委員会が作成し、実施医療機関の長に提出する。治験審査委員会の決定と実施医療機関の長の指示が同じである場合には、実施医療機関の長は、書式下部に通知日及び実施医療機関の長欄を記載し、自ら治験を実施する者に提出する。異なる場合には(医)参考書式１を使用する。

整理番号	C20-001

西暦2021年3月24日

治験審査委員会委員出欠リスト

氏名	職業、資格及び所属	委員区分	出欠	備考
赤松　佳美	学校法人早稲田学園<br>個人指導教室	①	○	一般の立場を代表する者
上原　知也	千葉大学大学院　薬学研究院	②③	○	薬学の専門家
岡林　伸幸	千葉大学大学院<br>社会科学研究院	①	○	法律学の専門家
小畠　隆行	量子科学技術研究開発機構<br>放射線医学総合研究所	④	○	医学の専門家
栗原　千絵子	量子科学技術研究開発機構<br>信頼性保証・監査室	①	○	人文・社会科学の有識者、副委員長
佐藤　紀子	株式会社マイクロン	②③	○	臨床試験に関する専門知識を有する者（看護学の専門家）
立崎　英夫	量子科学技術研究開発機構<br>高度被ばく医療センター	④	○	医学の専門家、副委員長
内藤　明日香	無	①	○	一般の立場を代表する者
中澤　栄輔	東京大学大学院　医学系研究科　医療倫理学分野	①	○	人文・社会科学の有識者
中根　潤	国立病院機構<br>下総精神医療センター	②③	○	医学の専門家
早川　和重	国立病院機構<br>災害医療センター	②③	○	医学の専門家、委員長
東　達也	量子科学技術研究開発機構<br>放射線医学総合研究所	④	○	医学の専門家
三橋　真次	量子科学技術研究開発機構<br>QST病院	④	×	薬学の専門家
森島　隆晴	敬愛大学経済学部	①	○	人文・社会科学の有識者

注）委員区分については以下の区分により番号で記載する。
①非専門委員
②実施医療機関と利害関係を有しない委員（①に定める委員を除く）
③治験審査委員会の設置者と利害関係を有しない委員（①に定める委員を除く）
④①～③以外の委員
また、出欠については以下の区分により記号で記載する。
○（出席し、かつ当該治験に関与しない委員）
－（出席したが、当該治験に関与するため審議及び採決に不参加の委員）
×（欠席した委員）

本治験審査委員会は、本治験審査委員会の標準業務手順書及び「医薬品の臨床試験の実施の基準に関する省令」（平成9年厚生省令第28号）、「医療機器の臨床試験の実施の基準に関する省令」（平成17年厚生労働省令第36号）、「再生医療等製品の臨床試験の実施の基準に関する省令」（平成26年厚生労働省令第89号）に従って組織され、活動していることを確認し、保証いたします。
