# Supplementary material for: MVI-targeted carbon-ion radiotherapy combined with immunotherapy for advanced hepatocellular carcinoma: Phase Ib DEPARTURE trial
Source: JHEP Rep. 2026 Feb 5;8(5):101765. doi: 10.1016/j.jhepr.2026.101765 (PMC13054414; doi:10.1016/j.jhepr.2026.101765)
Supplement: Multimedia component 4 [file mmc4.zip › ClinicalTrial/patient information sheet.pdf]

About a phase Ib study of durvalumab (MEDI4736) tremelimumab combined with particle therapy in advanced hepatocellular carcinoma patients with macrovascular invasion (DEPARTURE trial)

－ Information and Consent Form－

This booklet describes a phase Ib study of durvalumab (MEDI4736) tremelimumab combined with particle therapy in advanced hepatocellular carcinoma patients with macrovascular invasion (DEPARTURE trial)

Please read this information sheet carefully and understand the contents of the clinical trial before making your decision on whether you would like to participate in the clinical trial.

If you have questions or concerns about the contents or terms, please feel free to ask the study doctor or clinical research coordinator.

Chiba University Hospital
Principal investigator：Naoya Kato

Date prepared：02 24, 2021
Version number; 1.0

Table of Contents

1. What is a clinical trial？ 1
2. About your disease and treatment 3
3. Objective of the clinical trial 7
4. Method of the clinical trial 8
5. Specimen (blood and tissue) storage 17
6. Duration of participation in the clinical trial and number of participants 18
7. Foreseeable benefits 19
8. Foreseeable disadvantages and side effects 19
9. Free voluntary participation in the clinical trial and the ability to withdraw consent at any time 31
10. Alternative treatment methods if you do not participate in this clinical trial 31
11. Discontinuation after participation in a clinical trial 32
12. Regarding any new important information obtained 32
13. Compensation for health damage related to clinical trials 33
14. Costs during the study 33
15. Access to medical records and preservation of participant confidentiality 34
16. Conflicts of Interest 35
17. Your responsibilities during the study period 35
18. Institutional Review Board that has reviewed this study 36
19. Contact information 37

Please read this information sheet carefully and understand the contents of the clinical trial before making your decision on whether you would like to participate in the clinical trial. You are free to decide whether or not to participate. Also, you do not have to decide on the spot after receiving the explanation. You may decide after discussing the contents of this explanatory document with your family. You will not be disadvantaged in any manner if you decline to participate. You may withdraw from the clinical trial at any time even after you have agreed to participate in the clinical trial or after the clinical trial has started. If you have questions or concerns about the contents or terms, please feel free to ask the study doctor or clinical research coordinator.

1. What is a clinical trial？

In order to investigate the effectiveness and safety of a drug, it is necessary to conduct trials in which healthy people and patients participate, and these trials are called clinical studies. Of these, clinical studies in which results and data are collected in order to have the drug approved as a drug by the government (Ministry of Health, Labor and Welfare) are called “clinical trials. Clinical trials have a research aspect, but they are also important trials to enable many patients to receive new treatments and require the cooperation of patients and the consideration of specialized physicians. The drug used in a clinical trial is called an “investigational new drug,” and it is stipulated that the clinical trial should be conducted in compliance with the rules set by the government (GCP). This clinical trial is also conducted in compliance with these rules.

There are several phases in a clinical trial.

Overview of Treatment Development

Basic Research	Scientists screen a vast number of candidate compounds to identify those with the potential to become drugs.
Preclinical Testing	The efficacy and safety of the selected compounds are studied using animal models.
Clinical Trials	The safety and effectiveness of the drug are evaluated in humans.<br>Phase I Trial<br>Conducted with a small group of patients to assess the drug's safety and how it is distributed and processed in the body.<br>Phase Ib Trial<br>Evaluates the combination of a new treatment with existing therapies (this applies in the current study).<br>↓<br>Phase II Trial<br>Conducted with a limited number of patients to further assess the drug's effectiveness and safety.<br>↓<br>Phase III Trial<br>Involves a larger group of patients to compare the new drug's efficacy and safety against standard treatments.
Regulatory Review and Approval	Based on preclinical and clinical trial data, the Ministry of Health, Labour and Welfare (MHLW) rigorously evaluates whether the drug is appropriate for use. This process also ensures that the clinical trials were conducted ethically and scientifically. Once approved, the drug can be marketed and made available to patients.

In clinical trials for anticancer drugs, information gathered in three trials (Phase I-III) or two trials (Phase I and II), as described above, is submitted to the Ministry of Health, Labor and Welfare for review.

The clinical trial introduced here is a Phase Ib trial, which is a "investigator-initiated clinical trial" conducted by investigators who are actually involved in the actual medical treatment of the patient and is planned after considering the medical necessity and importance of the drug. This trial is conducted with the permission of the hospital director based on the deliberations of the Chiba University Hospital Clinical Trial Review Committee. Please see p.32 for more information about the committee.

2. About your disease and treatment

As hepatocellular carcinoma progresses, it can develop vascular invasion (tumor invasion of the portal vein and hepatic vein, important blood vessels that pass through the liver) and metastasis to organs other than the liver. This clinical trial is open to patients diagnosed with hepatocellular carcinoma with vascular invasion.

Main medical conditions that occur in patients with hepatocellular carcinoma with vascular invasion (varies from patient to patient)

(1) Fever, loss of appetite, fatigue, etc. due to tumor
(2) Abdominal pain due to the tumor, pain due to the metastatic site, and other symptoms
(3) intrahepatic metastasis
(4) Decrease in liver function due to the tumor
(5) Occurrence of esophageal and gastric varices

Hepatocellular carcinoma with vascular invasion is one of the most rapidly progressing forms of hepatocellular carcinoma and may also rapidly decline in strength, so treatment should be initiated as early as possible.

Currently, anticancer drug therapy (systemic chemotherapy) is the standard of care for patients with hepatocellular carcinoma with vascular invasion. The efficacy of drugs such as atezolizumab bevacizumab combination therapy, sorafenib, lenvatinib, regorafenib, ramucirumab, and

cabozantinib is known, but they are not yet fully effective. Other treatment modalities have similarly failed to show adequate results, and there is a need to develop treatments.

This clinical trial presented here was planned as one of the studies to develop safer and more effective treatment.

Studies have reported the importance of aggressive treatment of the area of vascular invasion itself, such as surgery and radiation. Immune checkpoint inhibitors (ICIs) are being actively developed as anticancer agents, and it is expected that more patients will benefit from treatment by combining ICIs with other therapies rather than ICIs alone.

<u>About the investigational drugs "Durvalumab" and "Tremelimumab</u>

Previous studies have shown that human immune action slows or controls the rate of cancer growth. However, there are cases where the natural immune response does not work and the human immune response does not kill the cancer. Research has shown that some cancer cells and immune cells produce a signal that blocks the cancer-killing action. A new drug in development blocks this signal and amplifies the immune response. These are two new drugs, durvalumab and tremelimumab. These are antibodies (proteins produced by the body's defense system). Durvalumab alone or in combination with tremelimumab may enhance the immune system's ability to detect and fight cancer. The two drugs target different signals. Durvalumab targets a cancer cell signal called PD-L1 (Programmed Cell Death Ligand 1), while tremelimumab targets an immune cell signal called CTLA-4 (Cytotoxic T-Lymphocyte-associated Antigen 4) CTLA-4 (Cytotoxic T-Lymphocyte-associated Antigen 4). By blocking these signals, it is hoped that immune cells can again control or slow the growth rate of cancer.

Durvalumab is approved in Japan under the brand name Imfinzi® for the treatment of unresectable locally advanced non-small cell lung cancer, while tremelimumab is not yet approved in Japan. Clinical trials are underway in lung cancer, bladder cancer, head and neck cancer, and other types of cancer. Outside of Japan, it is approved by the U.S. Food and Drug Administration (FDA) for the treatment of patients with locally advanced or metastatic urothelial carcinoma whose cancer has progressed during or after platinum-based chemotherapy. It is also approved by the U.S. FDA and the European Medicines Agency (EMA) in Europe for the treatment of patients with locally advanced non-small cell lung cancer who have received chemoradiation therapy. However, durvalumab is not approved in combination with tremelimumab (either in Japan or overseas) for the treatment of cancer patients.

Although neither durvalumab nor tremelimumab has been approved for the treatment of hepatocellular carcinoma, studies to date have confirmed that there are no problems with its safety and studies are underway to confirm its efficacy. If you have any questions about the investigational drug, please ask your physician at any time.

Durvalumab

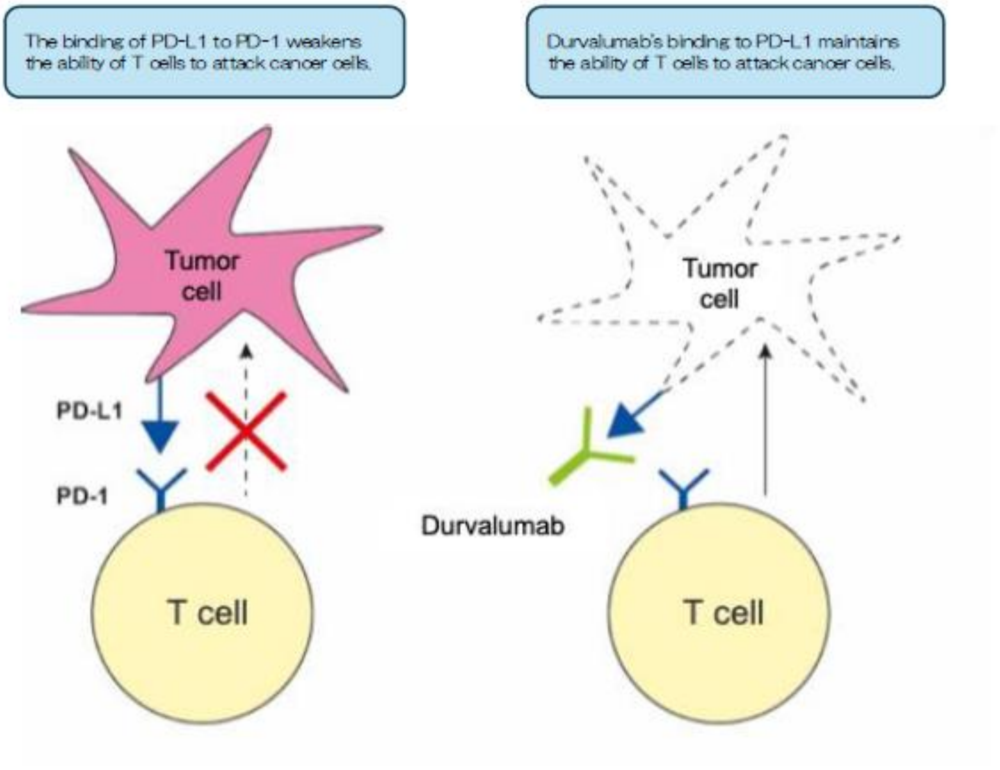

Tremelimumab

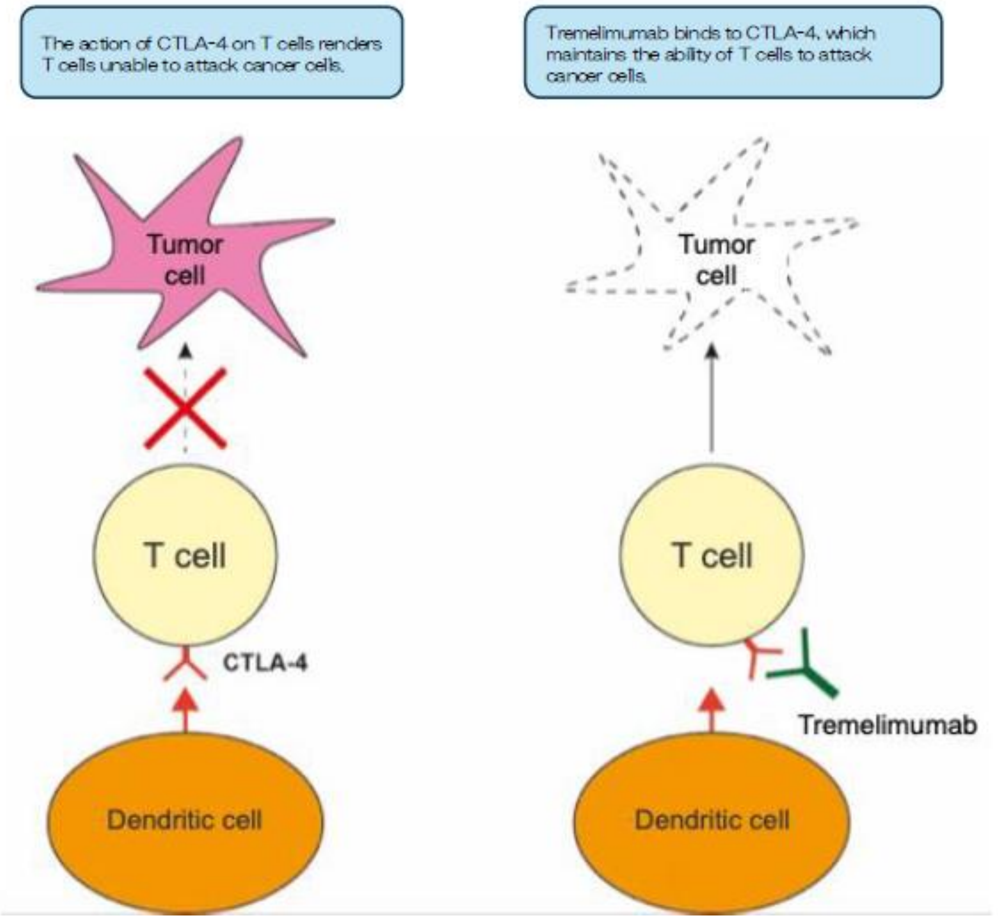

<u>About Carbon Ion Radio Therapy</u>

Carbon ion radio therapy is one of the radiation therapy methods for hepatocellular carcinoma. It is a new-generation treatment method classified as particle beam therapy along with proton beam therapy.

In contrast to conventional radiotherapy using X-rays, it can deliver high doses of radiation to the affected area while greatly minimizing irradiation to the normal liver. Studies of heavy-ion radiation therapy alone for hepatocellular carcinoma have reported that it is an effective treatment with minimal side effects.

Currently, research is being conducted in Japan under the advanced medical care system, in which patients bear the cost of their own treatment.

The participants in this study are scheduled to receive heavy particle therapy at the Hospital of the National Institute of Quantum Science and Technology.

3. Objective of the clinical trial

In this clinical trial, patients with advanced hepatocellular carcinoma will receive an infusion of durvalumab (or both durvalumab and tremelimumab) followed by treatment with heavy ion radiation. The main purpose of the study is to see what side effects may occur during this process and to determine whether this treatment can be safely administered.

The first 3-6 patients in the trial will receive durvalumab only, followed by heavy particle radiation. We will first review the physical condition of those who receive this treatment to make sure that the treatment can be safely administered.

Patients who participate after the review will receive both durvalumab and tremelimumab infusions and heavy particle therapy, which will also be evaluated again with respect to safety.

Based on the results of both treatments, we also aim to determine which treatment regimen is considered appropriate.

4. Method of the clinical trial

If you agree to participate in this clinical trial, you will first undergo a screening test. There are three periods in this clinical trial: the screening period, the treatment period, and the follow-up period after discontinuation of the clinical trial treatment.

During the screening period, you will undergo several tests and medical examinations to confirm that you are eligible to participate in this clinical trial treatment. Your investigator and the clinical trial staff will explain the clinical trial to you. The screening period is 28 days.

The treatment period is the time during which you will receive the study drug and heavy particle irradiation.

During the follow-up period, we will check your physical condition and blood test values after treatment is discontinued.

1) Eligibility

The conditions under which a patient may or may not participate in this clinical trial are as follows

≪ Patients who can participate in this study ≫

1) Patients diagnosed with hepatocellular carcinoma with vascular invasion

2) Patients with hepatocellular carcinoma not amenable to local therapies such as radiofrequency ablation (RFA) and trans arterial chemoembolization (TACE)

3) Patients must be 20 years of age or older

4) Weigh 30 kg or more

5) Good general condition and no major obstacles in daily life

6) Your test values (neutrophils, platelets, hemoglobin, liver function, renal function, etc.) meet certain criteria

7) Your consent to participate in this clinical trial has been obtained

≪ Patients who cannot participate in this study ≫

1) Brain metastasis

2) Currently suffering from cancer of an organ other than the liver, or suffered from cancer within 3 years

3) Received treatment with immunosuppressive agents within 14 days (28 days for antibody drugs) prior to the start of treatment with the investigational drug

4) Currently or previously had an autoimmune or inflammatory disease (unless the patient is determined to have had no active inflammatory disease within the past 5 years)

5) Have any of the following conditions or diseases

- Has serious heart disease (heart failure, myocardial infarction, angina pectoris, arrhythmia requiring treatment, etc.)
- Electrocardiogram (ECG) abnormalities
- Infectious disease requiring treatment with oral or injectable medications
- Severe respiratory illness (interstitial pneumonia or pulmonary fibrosis)
- Severe mental disorders (e.g., dementia)

- Positive test result for human immunodeficiency virus (HIV)
- Current or former hepatic encephalopathy
- Had cerebrovascular disease, thrombosis, or thromboembolism within 180 days prior to initiation of investigational therapy

6) Previous radiation therapy with liver effects.

7) Pregnant or lactating women.

8) Unable to consent to proper method of contraception during participation in the study and for 180 days from the date of the last dose of study drug (both sexes)

There are many other criteria, and we will make a judgment based on our detailed examination and consultation. Therefore, please understand that you may not be able to participate in the clinical trial even after you have given your consent, and even after the clinical trial has started, we may decide to terminate your treatment or participation in the clinical trial at our discretion. Patients who participate in this trial will be assigned to one of two treatment groups: durvalumab alone or a combination of durvalumab and tremelimumab as the drug to be combined with carbon ion radio therapy. In either group, you will be admitted to the hospital to start receiving durvalumab and tremelimumab, and you will also receive carbon ion radio therapy at QST Hospital. After completion of carbon ion radio therapy, patients will be discharged after confirming that they are in good physical condition, but the length of hospital stay will not change regardless of which group they are in. The length of hospitalization will be the same for both groups. Although the length of hospitalization may be longer depending on your physical condition, the expected length of hospitalization is approximately 3 weeks. After discharge from the hospital, patients will be followed up with outpatient visits.

The flow of this clinical trial is shown below.

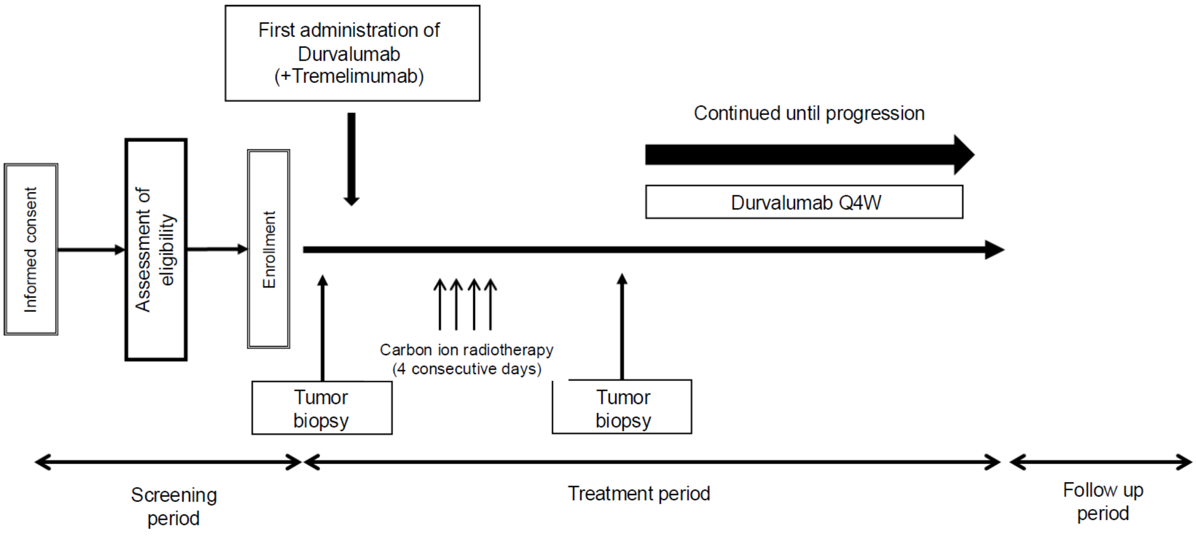

2) Detailed methods

The major objective of this study is to confirm that the combination of the investigational drug and carbon-ion radiation is safe for the human body. This confirmation of safety is called a Dose Limiting Toxicity (DLT) evaluation.

The first step in this evaluation is to assess whether the treatment is safe in two phases, with three to six patients receiving durvalumab followed by carbon ion radio therapy. This evaluation period will last 42 days from the start of treatment; if all three of the three patients, or at least five of the six patients, are found to be safe and there is no need to discontinue the trial, the next step, durvalumab and tremelimumab with carbon ion radio therapy will be given to an additional 3 to 6 patients. The same DLT evaluation will be performed on this group of patients. We plan to proceed with the clinical trial while confirming safety, and we expect a total of 15 patients to participate in the trial at our institution.

Depending on the timing of your participation in the clinical trial as described above, you will either receive durvalumab and carbon ion radio

therapy or durvalumab plus tremelimumab and carbon ion radio therapy. Your investigator or clinical trial coordinator will explain this to you each time.

As a general rule, to ensure your safety, you will be hospitalized from the time you receive your first dose of study drug until the time you receive carbon ion radio therapy. Specific administration methods and other details will be explained next.

① Dosing schedule

In this trial, 28 days are counted as one cycle.

Durvalumab (or both durvalumab and tremelimumab) will be administered intravenously on Day 1 of Cycle 1. The infusion treatment takes about 1.5 hours when only durvalumab is given, and about 3 hours when both durvalumab and tremelimumab are given.

Carbon ion radio therapy is then administered over the next 8 days (4 days total).

From Cycle 2 onward, durvalumab will be administered intravenously once every 4 weeks (on Day 1 of each cycle) after confirming that it can be administered safely according to the "Guidelines for the Management of Toxicity in Investigational New Drugs".

Your physical condition will be monitored especially carefully during the first 42 days after the first dose. Durvalumab will be continued and treatment will be continued while monitoring side effects and changes in your condition.

②Carbon ion radio therapy (and targeted marker insertion)

In this treatment, carbon-ion radiation must be focused on the lesion as precisely as possible. Otherwise, normal tissue, not cancer cells, will be

damaged. During the actual irradiation, you will lie on a treatment bed in a special treatment room, and you will be placed in a fixture to hold your body in place, and you will be asked to cooperate by not moving your body for a while. If you experience pain because the fixture does not fit properly, do not be patient and let us know. Carbon-ion radiation is given for a few minutes, but you will be in the treatment room for a total of 20 to 30 minutes, including the time needed for preparation and treatment before and after the irradiation. During the irradiation, you will be alone in the treatment room, but you do not need to worry because we will be watching you from outside the room via a TV camera, and if necessary, you can press the emergency buzzer to stop the irradiation and we will come to your room. You will not feel any pain or heat from the carbon-ion radiation during the irradiation.

Carbon ion radio therapy will be given once a day, four times a week, for a total dose of 60 Gy (RBE). This therapy will be completed in one week.

In this trial, a "target marker" is implanted in advance to accurately identify the lesion site during treatment. When the target marker is inserted from the surface of the body, local anesthesia is administered to the skin and subcutaneous tissue of the abdomen, a thin needle is inserted, and a tube is inserted into the liver. When inserted through a blood vessel in the liver, the target marker is implanted through a thin tube from an artery at the base of the groin to a site near the disease. The implanted target marker is not removed after treatment and remains in the organ, but there have been no reports of the marker directly harming the patient's health. When inserted from the surface of the body, a needle is inserted into the liver, which may cause bleeding, organ damage, and other side effects similar to liver biopsy (details are described in the explanation section of liver biopsy on p. 23). When inserting a catheter through a blood vessel in the liver, a method called abdominal angiography is applied. A thin tube called a catheter is inserted from a blood vessel in the leg or arm, and the catheter is advanced to a

blood vessel near the tumor in the liver. A targeted marker will then be implanted in a blood vessel near the tumor. Side effects associated with abdominal angiography (such as allergic reactions to contrast media, renal dysfunction, vascular injury, bleeding, infection, and organ damage) may occur.

３）Details and schedule of tests during the clinical trial

After you agree to participate in a clinical trial and sign the Clinical Trial Consent Form, screening tests will be performed to determine if you are suitable to participate in the trial. If the results of these tests can be substituted for the tests that were performed prior to your consent, we may use the results of those tests as data for the clinical trial. However, please understand that depending on the results of the tests, you may not be able to participate in the clinical trial. During the clinical trial period, including the screening tests, you will be taken for periodic medical examinations and consultations in accordance with the schedule shown in Table 1. In addition, after the administration of the investigational drug has been completed, we will also conduct medical examinations and consultations.

(A) Schedule of medical examinations and tests.

Investigations include measurement tests by blood and urine collection, electrocardiogram, CT and MRI, and follow-up by biopsy to collect tissue (liver tumor and non-tumor tissue). These tests are commonly performed to treat your disease and ensure your safety and are necessary for this clinical trial. If the results of the tests are deemed necessary by your physician, your visits and tests may be more frequent than those planned in Table 1.

(B) Tumor marker

There are many types of cancer, some of which produce substances characteristic of each cancer. Among such substances, those that can be measured in body fluids (mainly blood) are called tumor markers.

are called tumor markers.

Together with blood tests and imaging tests, tumor markers are used as one of the tests to check the progress of cancer and the progress of treatment.

In this case, blood will be used to measure AFP, AFP-L3, and PIVKA2, which are tumor markers for hepatocellular carcinoma. Blood samples will be drawn prior to the start of the study and prior to the administration on the first day of each of Cycle 2 and thereafter.

(C) Liver biopsy and liver tumor biopsy

Liver biopsy and liver tumor biopsy (tissue collection from non-tumor and tumor areas) will be performed on patients who have agreed to undergo liver biopsy and liver tumor biopsy. The timing of the liver biopsy/liver tumor biopsy will be before treatment and after the safety of the treatment has been confirmed for 42 days after the start of treatment. Liver tissue (non-tumor and tumor areas) obtained by biopsy will be used for exploratory studies.

Table 1: Schedule of medical examinations and tests

Cycle	Screening period	First tumor biopsy	DLT evaluation period							Second tumor biopsy	Durvalumab q4W dosing period					ST	Follow up period	Safety information collection
			Cycle 1				Cycle 2				Cycle 3	Cycle4	Cycle 5	Cycle 6	Cycle 7 and beyond			
Cycle Day	D -28 ～D -1	D -28〜D -1	1	8 to 14	15	22	1	8	14		1	1	1	1	1	—	—	—
Allowable period（Day）			—	±3	±3	±3	±3	±3	±3		—	±3	±3	±3	±3	+14	+14	+14
Informed Consent ／ Subject backgroud information／ Review of eligibility	●																	
Durvalumab administration (cohort A and B)			●				●				●	●	●	●	●			
Tremelimumab administration (cohort B)			●															
Tumor biopsy		●								●								
CIRT（QST hospital）				● ● ● ●														
Fiducial marker insertion		●																
Fixation, simulation CT (for CIRT)	●																	
Weight	●		●	●	●	●	●	●	●		●	●	●	●	●	●	●	
Height	●																	
Clinical Chemistry／Hematology	●		●	●	●	●	●	●	●		●	●	●	●	●	●	●	
Urinalysis	●		●								●	●	●	●	●	●	●	
ECG	●		●													●		
Chest X ray	●															●		
CT/MRI	●		Every 6 weeks (±1 week) for the first 12 weeks from Cycle1 day1, and every 8 weeks (±1 week) thereafter until PD.															
Tumor marker（AFP、PIVKA-II）	●						●				●	●	●	●	●			
Assessment of AE/SAE	←――――――――――――――――――――――――――――→																	

Table 2: Laboratory Tests for Blood and Urinalysis

Hematological Tests	Red blood cell count, Hemoglobin, Hematocrit, Platelet count, White blood cell count, White blood cell fraction (Neutrophils, Lymphocytes, Monocytes, Eosinophils, Basophils)
Biochemical examination of blood	Total protein, Albumin, Total bilirubin, AST, ALT, ALP, γ-GTP, LDH, BUN, Amylase, Lipase, Creatinine, Uric acid, Na, K, Cl, Ca, P, Mg, Blood sugar
Blood coagulation test	PT-INR、APTT
Urinalysis	Specific gravity, pH, Sugar, Protein, Ketones, Occult blood, Bilirubin, Color and appearance, Urinary sediment

5. Specimen (blood and tissue) storage

In principle, the specimens you provide for testing (blood and liver tissue (tumor and non-tumor parts)) will be stored until the end of the clinical trial.

If you give your consent, we plan to keep the specimens remaining after testing for a certain period of time (up to 20 years from the start of the clinical trial) at the Department of Gastroenterology of our hospital even after the trial has ended. This is because there is a possibility that additional research on hepatocellular carcinoma may be conducted in the future based on newly obtained findings. If used for research, it will be used only after approval by the Review Committee. When storing the specimens, we will anonymize them so that it will not be known that the specimens were collected from you.

You may participate in a clinical trial even if you do not consent to the storage of specimens after the trial is completed. You will not be disadvantaged by not consenting. Please make your decision based on your

own judgment, as there is a confirmation box on the clinical trial participation consent form.

You can withdraw your consent to the storage of specimens after the completion of the clinical trial at any time. If you withdraw, you will not be disadvantaged in any way. If you wish to withdraw your consent, please sign the Consent Withdrawal Form and submit it to your investigator or study coordinator.

However, if the person in charge at the hospital has already collected the analysis results before you submit the withdrawal of consent form, the results will not be used as they are.

If the results of the analysis have been collected by the person in charge at the hospital before you submit the withdrawal of consent, the results may be used as they are and shared with other parties.

If it is determined that there is no further need to store specimens before 20 years have passed from the start of the clinical trial, the specimens may be discarded without waiting 20 years from the start of the clinical trial.

In addition, since the results obtained are still in the research phase, they will not be disclosed to you in principle. If you wish to request disclosure, please contact us.

6. Duration of participation in the clinical trial and number of participants

Approximately 15 patients are expected to participate. The expected duration of participation in the clinical trial will be the total of the screening period (28 days) and the treatment and post-treatment follow-up periods (28

days after completion of treatment). The treatment period will be the sum of 42 days after the first dose and the period of continued durvalumab administration.

The duration of durvalumab treatment will depend on your condition and will continue as long as your investigator determines that it is effective for your disease. However, it may be discontinued if serious side effects occur and it becomes difficult to continue treatment, even if it is effective.

7. Foreseeable benefits

The combination of durvalumab tremelimumab with carbon ion radio therapy may demonstrate efficacy not seen with existing therapies.

Results of an AstraZeneca-led study of durvalumab-tremelimumab in advanced hepatocellular carcinoma have shown that each agent is safe as a single agent and that the combination of both agents has promising results. carbon ion radio therapy for hepatocellular carcinoma has also been shown to be safe and effective and is currently approved as an advanced medical care.

However, the safety and efficacy of durvalumab-tremelimumab in combination with carbon ion radio therapy is not known. Information obtained from this clinical trial may be used in future research on the treatment of this disease.

8. Foreseeable disadvantages and side effects

The side effects that may develop as a result of treatment vary greatly from person to person, and what symptoms may occur and their severity vary from person to person, and cannot be completely predicted before treatment begins. When side effects occur, treatment may be temporarily stopped, or treatment may be given to alleviate symptoms.

Serious side effects may also occur when treatment is resumed after a suspension or postponement of treatment.

If you feel that your condition is unusual, please contact your investigator for appropriate treatment.

Risks associated with durvalumab and tremelimumab.

Most of the side effects seen with durvalumab and tremelimumab were mild or moderate. However, some are serious, life-threatening and sometimes fatal. Some side effects do not require treatment, but symptoms usually recover with treatment. It may be necessary to delay the administration of durvalumab and tremelimumab to improve the symptoms of side effects. The most important side effects that may occur are listed below. These can occur as a result of the action of durvalumab and tremelimumab on the immune system and have been seen in patients who received either or both durvalumab and tremelimumab in previous studies. These side effects have also been seen in trials using other medicines similar to durvalumab and tremelimumab. The management of these side effects may require the administration of steroids or other medications that can work on the immune system and reduce inflammation.

The types of side effects were very similar when these two investigational drugs were given together and when durvalumab was given alone. However, the probability and severity of many, but not all, of these side effects were higher when they were given in combination than when durvalumab was given alone.

Very common side effects (>10%)

・ Diarrhea

・Rash / Dry and itchy skin

・ Liver dysfunction: Blood levels of substances called enzymes, which are found in liver cells, may increase. Changes in the enzyme do not often make you feel sick. However, if this enzyme level is very high, your investigator may need to discontinue the study drug. It may also cause inflammation of the liver, known as hepatitis, but this is rare. It may be accompanied by signs and symptoms such as yellowing of the skin and white eye area, dark urine, severe nausea and vomiting, pain in the upper right abdomen, itchy skin, inability to feel hunger, and bleeding or bruising more easily than normal.

In addition to the above, important anticipated side effects reported to be very common (i.e., >10%) in clinical trials in which patients with different types of cancer were treated with durvalumab alone or in combination with tremelimumab were fatigue, abdominal pain, swelling due to fluid retention, upper respiratory tract infection, nausea, vomiting, decreased appetite, shortness of breath, cough, fever, and muscle and joint pain.

Most common side effects (≥1% to <10%)

・ Hypothyroidism: This is caused by a decrease in the amount of thyroid hormones produced by the thyroid gland and a very slow metabolism. Symptoms include, but are not limited to, fatigue, feeling cold more easily, constipation, dry skin, unexplained weight gain, facial swelling, muscle weakness, decreased heart rate, thinning hair, and memory problems. These symptoms can be improved by thyroid hormone replacement. This eventis a very common side effect in patients receiving the combination of durvalumab and tremelimumab, but is classified as a common side effect in patients receiving durvalumab alone.

・ Pneumonia: Symptoms include, but are not limited to, new or worsening cough, shortness of breath (sometimes accompanied by fever). Pneumonia can be fatal. Limited data (not yet fully established) suggest that the incidence and severity of the disease may be higher in Japanese than in non-Japanese. If you have any of these symptoms, contact your investigator immediately.

・ Hyperthyroidism: This condition occurs when the thyroid gland produces too much thyroid hormone. Symptoms include anxiety, nervousness, weight loss, frequent bowel movements, diarrhea, shortness of breath, hot flashes, and heart palpitations. Depending on the severity of the symptoms, treatment may include observation only, symptomatic treatment, or treatment to stop the secretion of thyroid hormones.

・ Renal dysfunction: Even without symptoms or feeling sick, blood tests may show increased creatinine levels (creatinine is a protein marker that assesses kidney function). Less frequently, nephritis may occur, in which the kidneys become inflamed and lose normal function.

・Nervous system disorders: Symptoms include abnormal weakness of the leg, arm, or facial muscles, or numbness or tingling in the limbs. Rarely, severe inflammation of the nervous system may occur, which can damage nerve cells and interfere with communication between nerves and muscles. If you experience trouble swallowing, sudden weakness, or difficulty breathing, contact your doctor immediately.

・ Injection reactions: Reactions may occur during or after injection of the investigational drug. Injection reactions may cause fever or chills, changes in blood pressure, or significant dyspnea. Contact your investigator immediately if you experience any of these symptoms, even if it is several days after the injection.

・Intestinal inflammation (colitis): May cause abdominal pain and diarrhea (with or without bleeding). May be accompanied by fever. Additional intravenous fluids may be needed. If left untreated, it can cause serious and life-threatening tears in the intestinal wall. If you experience any of these symptoms, contact your doctor immediately.

・ Elevated levels of pancreatic enzymes (amylase and lipase). These enzymes are indicators of pancreatic function. In rare cases, elevated levels of these enzymes may be associated with pancreatitis (see "Rare Side Effects" below).

In addition to the above, the most common adverse reactions reported as serious (i.e., ≥1% to <10%) in clinical trials in which durvalumab was administered alone or in combination with tremelimumab to patients with various types of cancer are pneumonia, hoarse voice, urinary pain, night sweats, oral candidiasis, and muscle and joint pain.

Common side effect (≥1% to <10%) (occurs in 1 out of 10-100 patients) / Rare side effect (<1%) (occurs in 1 out of 100-1000 patients)

・ Adrenal Injury: May cause stomach pain, vomiting, muscle weakness, fatigue, mood swings, hypotension, weight loss, kidney problems, and mood and personality changes. It has been reported to occur more frequently in patients receiving the combination of durvalumab and tremelimumab, but less frequently in patients receiving durvalumab as a single agent. These complications may require permanent hormone replacement therapy.

・ Dental and oral soft tissue infections and influenza were more common in patients who received durvalumab as a single agent and rare in those who received it in combination with tremelimumab.

Rare (<1%) (occurs in 1 out of 100 to 1000 patients)

・ Pancreatitis: Pancreatitis usually presents with persistent pain in the upper abdomen (which may be made worse by eating or drinking), nausea, vomiting and weakness. Pancreatitis usually improves with simple treatment but can be severe and life-threatening. Contact your investigator immediately if you have any of these symptoms.

・ Allergic reaction: An allergic reaction may cause swelling of the face, lips, or throat, or breathing difficulties accompanied by hives or a hives-like rash. If any of these symptoms occur, contact your investigator immediately.

Rare (<1%) (occurs in 1 out of 100-1000 patients) / Very Rare (<0.1%) (occurs in 1 out of 1000-10000 patients)

・Myositis/Polymyositis: Symptoms include muscle weakness, myalgia, fatigue while standing or walking, and muscle pain lasting several weeks. It was rare in patients treated with durvalumab as a single agent and very rare in patients treated with tremelimumab in combination.

・Pituitary disorder (hypopituitarism): Hypopituitarism is a decrease in the hormone secreted by the pituitary gland in the brain and is caused by inflammation of the pituitary gland (hypopituitarism). Symptoms include headache, thirst, difficulty seeing or double vision, leakage of breast milk in women, or irregular menstruation. These complications may require permanent hormone replacement therapy. They were rare in patients who received durvalumab in combination with tremelimumab and very rare in patients who received durvalumab as a single agent.

・ Inflammation of the heart muscle (myocarditis): symptoms include chest pain, tachycardia, irregular heartbeat, shortness of breath, and swelling of the legs. If you have any of these symptoms, contact your physician immediately. This side effect is classified as an uncommon side effect in patients receiving the combination of durvalumab and tremelimumab, but very rare in patients receiving durvalumab alone.

Very Rare Adverse Reactions (<0.1%) (occurs in 1 out of 1000-10000 patients)

・Type 1 diabetes (may cause elevated blood glucose levels, known as hyperglycemia): Symptoms include weight loss, increased urination, increased thirst and hunger. type 1 diabetes requires insulin replacement by injection. If you have any of these symptoms, contact your doctor immediately.

In addition to the above, important predicted disadvantages reported as rare (i.e., less than 0.1% of patients) in clinical trials of single-agent durvalumab in patients with different types of cancer are inflammation of the membranes surrounding the heart, increased number of small clusters of inflammatory cells in various parts of the body, inflammation of the middle layer of the eye or Other eye abnormalities (e.g., inflammation of the cornea and optic nerve), inflammation of the brain or membranes lining the brain and spinal cord, hardening or straining of skin and connective tissue and loss of skin color, hematologic abnormalities (e.g., abnormal red blood cell destruction, thrombocytopenia), vascular inflammation, and rheumatic abnormalities (muscle pain and stiffness caused by inflammatory disease and autoimmune autoimmune arthritis).

In addition to these expected disadvantages when durvalumab is administered alone or in combination with durvalumab and tremelimumab, other immune-mediated side effects not previously observed may occur, and inflammatory side effects may occur in any organ or tissue.

<u>Side effects of carbon ion radio therapy</u>

１）Gastrointestinal tract

In the case that the cancer is located close to the liver surface and in close proximity to the gastrointestinal tract, such as the stomach, duodenum, or large intestine, carbon ion radio therapy may cause damage to these digestive tracts. Anorexia, nausea, diarrhea, and abdominal pain are the most common symptoms, but it is important to note that anemia due to bleeding may be detected without obvious symptoms. The symptoms range from mild erosions and ulcers that can be treated with medication and diet to severe cases that require hospitalization, such as bleeding, perforation (hole), stenosis (narrowing, making it difficult for objects to pass through), obstruction (blockage of the lumen, making it impossible to pass objects through), and adhesions. Based on the results of clinical trials on cancers in other parts of the body, we can predict to some extent the safe dose when irradiated to the gastrointestinal tract, and we will make sure that the dose irradiated to the gastrointestinal tract is less than the dose considered safe to avoid serious complications. To date, a very small number of patients have experienced mild side effects (less than 1%) that improve with medication, but there have been no serious side effects that would require invasive treatment such as surgery.

２）Skin

Various degrees of radiation dermatitis may occur as a result of carbon ion radio therapy. In most cases, the symptoms are similar to a mild sunburn (redness, itching, mild pain, etc.), but in some cases, the surface of the skin may peel, producing exudates, hyperpigmentation, and small scars (scars). If the lesion is located relatively close to the skin, the skin is also exposed to high doses of radiation, which may cause erythema, erosions, ulcers, atrophy, etc., which may require treatment with ointments. Surgical procedures such as skin grafts may be necessary, although rarely (0-2.8% in past reports), and are becoming less common due to improvements in treatment techniques.

３）Liver

Liver function in the area exposed to carbon-ion radiation will be reduced or eliminated, and overall liver function may also be reduced. In this study, treatment is designed with the expectation that sufficient liver function will remain, but it is known that radiation-induced liver injury (RILD) will occur in 1-2% of patients.

４）Bile duct

Carbon-ion radiation therapy may cause cholangitis, but there have been no cases of cholangitis directly attributable to this treatment. Although rare (less than 1% of cases), bile duct stricture may require medical treatment.

５）Lung

If a portion of the lung is irradiated with carbon-ion radiation, imaging changes, pleural effusion, coughing, fever, and dull pain may occur. In severe cases, sputum, blood sputum, shortness of breath, interstitial pneumonia-like shadows, pulmonary fibrosis-like shadows, and respiratory failure are possible, but since only a small portion of the lung is usually irradiated in the treatment of hepatocellular carcinoma, no serious side effects that could be caused by carbon ion radio therapy have been observed.

６）Blood

Decreases in peripheral blood white blood cell count, red blood cell count, and platelet count, and decreases in hemoglobin concentration may occur.

７）Possibility of secondary carcinogenesis

In the case of conventional radiotherapy, there is a rare possibility of new cancer development (secondary cancer) caused by radiation after a long period of time, and the same is possible with carbon ion radio therapy.

Other risks anticipated from participation in this clinical trial include

Blood sampling: Risks associated with blood sampling include temporary discomfort from needle puncture, internal bleeding, hemorrhage, and in rare instances, infection and anemia may be caused.

ECG: Risks associated with ECG testing include temporary discomfort such as itching, mild irritation, or redness of the skin where the small adhesive pad is applied. If it is necessary to shave the area where this small adhesive pad is applied, the shaving may cause irritation.

CT or MRI: You may experience some discomfort or anxiety when lying down inside the CT scan machine. The injection of contrast may cause a metallic taste in the mouth, a feeling of warmth, and in rare cases, nausea or vomiting. A reaction to the contrast agent may also occur. MRI is safe for most people. People with metal implants near vital organs cannot undergo MRI. This is because the metal can be drawn from the body to a large magnet, which can cause damage.

Biopsy (collection of liver tumor and non-tumor tissue): A biopsy is the collection of a piece of your tissue using a scalpel or needle. This time, the needle will be inserted directly into your liver, which may cause mild discomfort and internal bleeding at the site of the needle puncture. Bleeding, liver dysfunction, jaundice, infection, pneumothorax, and peritoneal seeding may also occur. The frequency of percutaneous puncture of the liver in routine examinations is about 2%. Although rare, surgery or other procedures and blood transfusions may be required for the above treatments. In addition, anesthetics and sedatives used prior to administration may cause a sudden drop in blood pressure or an irregular heartbeat, which may strain the heart. Side effects such as allergic reactions and fever may also occur.

If you become pregnant during a clinical trial, or if your partner becomes pregnant, please notify your investigator or the study coordinator immediately. If you become pregnant, your participation in the clinical trial will be terminated.

If you or your partner becomes pregnant, we will ask you for this information. If you or your partner gave birth, had a miscarriage or an abortion, please tell your investigator. If you or your partner gave birth, you may be

asked to provide information including the baby's date of birth, height and weight at birth, sex of the baby, any complications during pregnancy or delivery, and any birth defects of the baby.

9. Free voluntary participation in the clinical trial and the ability to withdraw consent at any time

Participation in this clinical trial is not mandatory, so please make your own decision.

You may withdraw from the study at any time after participating in the study, regardless of the reason, if you wish to discontinue the study or if it is difficult to continue, so please consult your physician. You will not be disadvantaged by the discontinuation of the clinical trial. After the discontinuation of a clinical trial, you will be informed of the best possible treatment by your physician.

Your doctor will then explain the best treatment options to you.

10. Alternative treatment methods if you do not participate in this clinical trial

If you do not participate in this clinical trial, please ask your physician to explain in detail the other treatment options that may be available to you. You will not be disadvantaged by not participating in this clinical trial. Your physician will discuss with you the best treatment for your condition. Options include participating in a clinical trial for another drug, treatment with an already approved drug, or radiation therapy. Supportive care (pain and suffering control) is also available.

11. Discontinuation after participation in a clinical trial

Even after you have given your consent to participate in a clinical trial, we may discontinue the clinical trial treatment for the following reasons

① When you request discontinuation.
② In case of strong side effects during the clinical trial
③ When cancer has become large
④ When it is deemed difficult to continue the clinical trial due to circumstances at the hospital where the clinical trial is being conducted.
⑤ Other cases in which the investigator determines that discontinuation of the clinical trial is necessary.

28 days after the end of treatment, an examination will be conducted to confirm safety. Even if the clinical trial is terminated due to side effects, we ask for your cooperation as we may conduct tests and medical examinations until the side effects are no longer present.

Even if the clinical trial is terminated during the course of the study, we would like to use the records up to that point, as they will be valuable materials for future cancer research. If you have any concerns about the use of your records, please contact us.

12. Regarding any new important information obtained

We will promptly inform you if we obtain new information on important efficacy, safety, or other information that we believe may affect your decision to continue participating in this clinical trial. In that case, you will be asked to make a new decision as to whether or not you wish to continue participating in the clinical trial.

13. Compensation for health damage related to clinical trials

If you experience any problems related to the investigational drug, or if you experience any health problems as a result of participating in a clinical trial, you should notify your investigator immediately. The physician will treat you for any health problems that arise as a result of your participation in a clinical trial, and you may be eligible for compensation. However, compensation may not be provided in the following cases

① When there is no causal relationship between the health hazard and the clinical trial

② In case of progression of hepatocellular carcinoma due to inadequate efficacy of the investigational drug

③ If the health damage was caused by your intentional or gross negligence

For more information, please consult your investigator or consultation service.

14. Costs during the study

If you participate in this clinical trial, the investigational drug will be provided by the investigational drug provider (AstraZeneca Corporation). In addition, the hospital will cover the costs associated with heavy particle irradiation. We will also cover the cost of transportation to and from the QST Hospital for carbon ion radio therapy while you are hospitalized at Chiba University Hospital. The costs of medical examination fees, tests, drugs normally used, drugs used for side effects, tests and diagnostic imaging, etc. will be borne by the patient according to the type of health insurance as in the past. You will also be responsible for the cost of hospitalization for this clinical trial treatment.

15. Access to medical records and preservation of participant confidentiality

Any personally identifiable information that may be used to identify an individual who has cooperated in a clinical trial will be strictly protected and will not be released to outside parties.Data obtained from patients will be anonymized by code numbers, etc., and will be handled in such a way that they will not be identified as belonging to that patient in reports, etc.

The results of this clinical trial will be submitted to the Ministry of Health, Labour and Welfare (MHLW) in order to have the government (MHLW) approve the combination therapy of durvalumab tremelimumab and heavy ion therapy as a treatment for advanced hepatocellular carcinoma with vascular invasion. In addition, in order to study the efficacy and safety of this investigational drug in more detail, data necessary to evaluate the investigational drug, such as tests, images, and electrocardiograms used as data in the clinical trial, may be submitted to an outside organization.

Furthermore, information obtained from this clinical trial may be compiled from records collected from hospitals in various regions and published in academic societies and medical journals. In all cases, however, your name will not be used and your personal information (name, address, telephone number, etc.) will be kept confidential and will not be leaked to outside parties.

In addition, in order to check whether the clinical trial is being conducted properly, personnel from the investigational drug development organization, the Clinical Trial Review Committee of this hospital, and regulatory authorities such as the Ministry of Health, Labor and Welfare may access your medical records, including your medical records from other departments and the period before your participation in the clinical trial. Even in such cases, these officials are obligated to maintain confidentiality and your privacy will be protected.

Even if you have received treatment at another medical institution, we may contact your physician at the other institution by phone or letter to request

medical information. By signing the consent form at the end of this letter, you are also giving your consent to access your records and to the collection of information from other medical institutions.

You may withdraw your consent to the use of your medical information at any time, but please note that you will not be able to participate in this clinical trial after that. If you wish to withdraw your consent, please inform your doctor or hospital staff in charge. The clinical trial data collected before you revoke your consent will be used in the same way as described above, but after you revoke your consent, your medical information will not be used except to confirm that this clinical trial has been conducted properly.

16. Conflicts of Interest

This study is being funded and conducted by AstraZeneca Inc. At the same time, we are receiving the investigational drug and information on the safety of the drug, but there is no profit from the conduct of this study itself or from the analysis and reporting of the results. This has been reviewed by our Conflict-of-interest Management Committee, which has confirmed that no conflicts of interest* (possible conflicts of interest) exist with regard to the implementation of this clinical trial.

*Conflicts of interest are defined as actions that may compromise patient safety or distort the interpretation of data for the benefit of oneself or a pharmaceutical company, for example, when receiving funding or other benefits from a pharmaceutical company.

17. Your responsibilities during the study period

If you agree to participate in this clinical trial, please observe the following

1) Please follow the trial schedule during your participation in the trial.

2) If you are currently receiving treatment or taking medication at another department or hospital, please tell your investigator or coordinator about

your situation in detail. We will inform your treating physician that you are participating in this clinical trial.Also, if you are taking any over-the-counter medications, please consult your investigator or the clinical trial coordinator in advance.

3) The tests that will be performed during the clinical trial are very important for us to know about any changes in your condition or side effects, so please follow the instructions of your investigator and the clinical trial coordinator.

4) If you experience any changes in your physical condition while using the investigational drug, please inform your investigator or the study coordinator.

5) Please follow any other precautions or instructions given by the investigator in charge of the clinical trial.

18. Institutional Review Board that has reviewed this study

The clinical trial is reviewed by the Institutional Review Board below, not only from a scientific and medical perspective, but also from an ethical perspective, including patient safety and human rights. The Clinical Trial Review Committee includes members who do not specialize in medicine or have no vested interest in our hospital.

1)Name: Chiba University Hospital Institutional Review Board
2)Type: Institutional Review Board
3)Founder: Director of Chiba University Hospital
4)Address: 1-8-1 Inohana, Chuouku, Chiba

After the clinical trial has started, we will review, upon request from the director of this hospital, whether this clinical trial can be continued if the above information is changed or if safety information, such as the occurrence of serious side effects, is obtained.

The results of the review will be reported to the director of this hospital, who will decide whether to initiate or continue the clinical trial at this hospital based on the contents of the report.

19. Contact information

If you have any questions, doubts, questions, or would like to ask again or get more detailed information about this clinical trial or treatment, please do not hesitate to ask us at any time. Even after the clinical trial has started, we will be happy to answer any questions you may have. Also, if you have any concerns about the use of this investigational drug, please do not hesitate to contact us at any time.

If you agree to participate in this clinical trial after fully understanding the details of this trial, please indicate the date of your consent and sign the consent document at the end of this explanatory document.

Please keep this Explanatory Document and the Consent Document for patients in a safe place.

Site：Chiba University Hospital

Principal Investigator：Naoya Kato

Your Investigator：______________________________

Contact us below

Weekdays (8:30-17:00)

Outpatient Gastroenterology Tel：+8143-222-7171

Clinical Trial Coordinator Office Tel：+8143-226-2630

Nighttime and Holiday Phone Service Tel：+8143-222-7171

*Please inform us that you are participating in a clinical trial in gastroenterology.

For investigator

Consent Form

I hereby give my consent to participate in the “Phase Ib Clinical Trial to Evaluate the Safety and Efficacy of Durvalumab Tremelimumab in Combination with Heavy Ion Beam Therapy in Patients with Advanced Hepatocellular Carcinoma with Vascular Invasion” on my own free will, after having received and fully understood the following information. I agree to participate in this clinical trial of my own free will after having received and fully understood the following information.

- What is a clinical trial?
- About your disease and treatment
- Objective of the clinical trial
- Method of the clinical trial
- 治験のスケジュール
- Specimen (blood and tissue) storage • Duration of participation in the clinical trial and number of participants
- Foreseeable benefits
- Foreseeable disadvantages and side effects
- Free voluntary participation in the clinical trial and the ability to withdraw consent at any time
- Alternative treatment methods if you do not participate in this clinical trial
- Discontinuation after participation in a clinical trial
- Regarding any new important information obtained
- Compensation for health damage related to clinical trials
- Costs during the study
- Access to medical records and preservation of participant confidentiality
- Conflicts of Interest
- Your responsibilities during the study period
- Institutional Review Board that has reviewed this study
- Contact information

●Performing liver biopsy and liver tumor biopsy (before/after 42 days of treatment)

☐ Agree　☐ Disagree

●Storage of specimens after completion of the clinical trial

☐ Agree　☐ Disagree

Participant

Date of consent：＿＿＿＿＿＿＿ (Month, Day, Year)

Signature:：＿＿＿＿＿＿＿＿＿＿＿＿

The investigator who obtained consent

Date of signature ＿＿＿＿＿＿ (Month, Day, Year)

Signeture：＿＿＿＿＿＿＿＿＿＿

The person who provided supplementary explanation

Date of signature ＿＿＿＿＿＿ (Month, Day, Year)

Signeture：＿＿＿＿＿＿＿＿＿＿

For site

Consent Form

I hereby give my consent to participate in the "Phase Ib Clinical Trial to Evaluate the Safety and Efficacy of Durvalumab Tremelimumab in Combination with Heavy Ion Beam Therapy in Patients with Advanced Hepatocellular Carcinoma with Vascular Invasion" on my own free will, after having received and fully understood the following information. I agree to participate in this clinical trial of my own free will after having received and fully understood the following information.

・What is a clinical trial?
・About your disease and treatment
・Objective of the clinical trial
・Method of the clinical trial
・治験のスケジュール
・Specimen (blood and tissue) storage・Duration of participation in the clinical trial and number of participants
・Foreseeable benefits
・Foreseeable disadvantages and side effects
・Free voluntary participation in the clinical trial and the ability to withdraw consent at any time
・Alternative treatment methods if you do not participate in this clinical trial
・Discontinuation after participation in a clinical trial
・Regarding any new important information obtained
・Compensation for health damage related to clinical trials
・Costs during the study
・Access to medical records and preservation of participant confidentiality
・Conflicts of Interest
・Your responsibilities during the study period
・Institutional Review Board that has reviewed this study
・Contact information

●Performing liver biopsy and liver tumor biopsy (before/after 42 days of treatment)

☐ Agree ☐ Disagree

●Storage of specimens after completion of the clinical trial

☐ Agree ☐ Disagree

Participant

Date of consent：________ (Month, Day, Year)

Signature:：________________

The investigator who obtained consent

Date of signature ________ (Month, Day, Year)

Signeture：________________

The person who provided supplementary explanation

Date of signature ________ (Month, Day, Year)

Signeture：________________

For particopant

Consent Form

I hereby give my consent to participate in the “Phase Ib Clinical Trial to Evaluate the Safety and Efficacy of Durvalumab Tremelimumab in Combination with Heavy Ion Beam Therapy in Patients with Advanced Hepatocellular Carcinoma with Vascular Invasion” on my own free will, after having received and fully understood the following information. I agree to participate in this clinical trial of my own free will after having received and fully understood the following information.

- What is a clinical trial?
- About your disease and treatment
- Objective of the clinical trial
- Method of the clinical trial
- 治験のスケジュール
- Specimen (blood and tissue) storage • Duration of participation in the clinical trial and number of participants
- Foreseeable benefits
- Foreseeable disadvantages and side effects
- Free voluntary participation in the clinical trial and the ability to withdraw consent at any time
- Alternative treatment methods if you do not participate in this clinical trial
- Discontinuation after participation in a clinical trial
- Regarding any new important information obtained
- Compensation for health damage related to clinical trials
- Costs during the study
- Access to medical records and preservation of participant confidentiality
- Conflicts of Interest
- Your responsibilities during the study period
- Institutional Review Board that has reviewed this study
- Contact information

●Performing liver biopsy and liver tumor biopsy (before/after 42 days of treatment)

☐ Agree ☐ Disagree

●Storage of specimens after completion of the clinical trial

☐ Agree ☐ Disagree

Participant

Date of consent：________ (Month, Day, Year)

Signature:：________________

The investigator who obtained consent

Date of signature ________ (Month, Day, Year)

Signeture：________________

The person who provided supplementary explanation

Date of signature ________ (Month, Day, Year)

Signeture：________________

For investigator

Consent Withdrawal Form

I am participating in the “Phase Ib Clinical Trial to Evaluate the Safety and Efficacy of Durvalumab Tremelimumab in Combination with Heavy Grain Therapy in Patients with Advanced Hepatocellular Carcinoma with Vascular Invasion”.I have read and agreed to the following terms and conditions,However, after reexamination of the information, I hereby withdraw my consent as follows

I withdraw my consent for the storage of the specimen provided in the above study.

Participant

Date of consent to withdrawal：_____________ (Month, Day, Year)

Signature:：____________________________

The investigator who obtained consent

Date of signature ___________ (Month, Day, Year)

Signeture：_____________________

The person who provided supplementary explanation

Date of signature ___________ (Month, Day, Year)

Signeture：_____________________

For site

Consent Withdrawal Form

I am participating in the “Phase Ib Clinical Trial to Evaluate the Safety and Efficacy of Durvalumab Tremelimumab in Combination with Heavy Grain Therapy in Patients with Advanced Hepatocellular Carcinoma with Vascular Invasion”.I have read and agreed to the following terms and conditions,However, after reexamination of the information, I hereby withdraw my consent as follows

I withdraw my consent for the storage of the specimen provided in the above study.

Participant

Date of consent to withdrawal：_______________ (Month, Day, Year)

Signature:：_______________________________

The investigator who obtained consent

Date of signature _____________ (Month, Day, Year)

Signeture：________________________

The person who provided supplementary explanation

Date of signature _____________ (Month, Day, Year)

Signeture：________________________

For participant

Consent Withdrawal Form

I am participating in the “Phase Ib Clinical Trial to Evaluate the Safety and Efficacy of Durvalumab Tremelimumab in Combination with Heavy Grain Therapy in Patients with Advanced Hepatocellular Carcinoma with Vascular Invasion”.I have read and agreed to the following terms and conditions,However, after reexamination of the information, I hereby withdraw my consent as follows

I withdraw my consent for the storage of the specimen provided in the above study.

Participant

Date of consent to withdrawal：______ (Month, Day, Year)

Signature:：______

The investigator who obtained consent

Date of signature ______ (Month, Day, Year)

Signeture：______

The person who provided supplementary explanation

Date of signature ______ (Month, Day, Year)

Signeture：______

About a phase Ib study of durvalumab (MEDI4736) tremelimumab combined with particle therapy in advanced hepatocellular carcinoma patients with macrovascular invasion (DEPARTURE trial)

－ Information and Consent Form－

This booklet describes a phase Ib study of durvalumab (MEDI4736) tremelimumab combined with particle therapy in advanced hepatocellular carcinoma patients with macrovascular invasion (DEPARTURE trial)<br>Please read this information sheet carefully and understand the contents of the clinical trial before making your decision on whether you would like to participate in the clinical trial.<br>If you have questions or concerns about the contents or terms, please feel free to ask the study doctor or clinical research coordinator.

Chiba University Hospital
Principal investigator：Naoya Kato

Date prepared：05 06, 2021
Version number; 1.1

Table of Contents

1. What is a clinical trial？ 1
2. About your disease and treatment 3
3. Objective of the clinical trial 7
4. Method of the clinical trial 8
5. Specimen (blood and tissue) storage 17
6. Duration of participation in the clinical trial and number of participants 18
7. Foreseeable benefits 19
8. Foreseeable disadvantages and side effects 19
9. Free voluntary participation in the clinical trial and the ability to withdraw consent at any time 31
10. Alternative treatment methods if you do not participate in this clinical trial 31
11. Discontinuation after participation in a clinical trial 32
12. Regarding any new important information obtained 32
13. Compensation for health damage related to clinical trials 33
14. Costs during the study 33
15. Access to medical records and preservation of participant confidentiality 34
16. Conflicts of Interest 35
17. Your responsibilities during the study period 35
18. Institutional Review Board that has reviewed this study 36
19. Contact information 37

Please read this information sheet carefully and understand the contents of the clinical trial before making your decision on whether you would like to participate in the clinical trial. You are free to decide whether or not to participate. Also, you do not have to decide on the spot after receiving the explanation. You may decide after discussing the contents of this explanatory document with your family. You will not be disadvantaged in any manner if you decline to participate. You may withdraw from the clinical trial at any time even after you have agreed to participate in the clinical trial or after the clinical trial has started. If you have questions or concerns about the contents or terms, please feel free to ask the study doctor or clinical research coordinator.

1. What is a clinical trial？

In order to investigate the effectiveness and safety of a drug, it is necessary to conduct trials in which healthy people and patients participate, and these trials are called clinical studies. Of these, clinical studies in which results and data are collected in order to have the drug approved as a drug by the government (Ministry of Health, Labor and Welfare) are called “clinical trials. Clinical trials have a research aspect, but they are also important trials to enable many patients to receive new treatments and require the cooperation of patients and the consideration of specialized physicians. The drug used in a clinical trial is called an “investigational new drug,” and it is stipulated that the clinical trial should be conducted in compliance with the rules set by the government (GCP). This clinical trial is also conducted in compliance with these rules.

There are several phases in a clinical trial.

Overview of Treatment Development

Basic Research	Scientists screen a vast number of candidate compounds to identify those with the potential to become drugs.
Preclinical Testing	The efficacy and safety of the selected compounds are studied using animal models.
Clinical Trials	The safety and effectiveness of the drug are evaluated in humans.<br><br>Phase I Trial<br>Conducted with a small group of patients to assess the drug's safety and how it is distributed and processed in the body.<br><br>Phase Ib Trial<br>Evaluates the combination of a new treatment with existing therapies (this applies in the current study).<br><br>↓<br><br>Phase II Trial<br>Conducted with a limited number of patients to further assess the drug's effectiveness and safety.<br><br>↓<br><br>Phase III Trial<br>Involves a larger group of patients to compare the new drug's efficacy and safety against standard treatments.
Regulatory Review and Approval	Based on preclinical and clinical trial data, the Ministry of Health, Labour and Welfare (MHLW) rigorously evaluates whether the drug is appropriate for use. This process also ensures that the clinical trials were conducted ethically and scientifically. Once approved, the drug can be marketed and made available to patients.

In clinical trials for anticancer drugs, information gathered in three trials (Phase I-III) or two trials (Phase I and II), as described above, is submitted to the Ministry of Health, Labor and Welfare for review.

The clinical trial introduced here is a Phase Ib trial, which is a "investigator-initiated clinical trial" conducted by investigators who are actually involved in the actual medical treatment of the patient and is planned after considering the medical necessity and importance of the drug. This trial is conducted with the permission of the hospital director based on the deliberations of the Chiba University Hospital Clinical Trial Review Committee. Please see p.32 for more information about the committee.

2. About your disease and treatment

As hepatocellular carcinoma progresses, it can develop vascular invasion (tumor invasion of the portal vein and hepatic vein, important blood vessels that pass through the liver) and metastasis to organs other than the liver. This clinical trial is open to patients diagnosed with hepatocellular carcinoma with vascular invasion.

Main medical conditions that occur in patients with hepatocellular carcinoma with vascular invasion (varies from patient to patient)

(1) Fever, loss of appetite, fatigue, etc. due to tumor
(2) Abdominal pain due to the tumor, pain due to the metastatic site, and other symptoms
(3) intrahepatic metastasis
(4) Decrease in liver function due to the tumor
(5) Occurrence of esophageal and gastric varices

Hepatocellular carcinoma with vascular invasion is one of the most rapidly progressing forms of hepatocellular carcinoma and may also rapidly decline in strength, so treatment should be initiated as early as possible.

Currently, anticancer drug therapy (systemic chemotherapy) is the standard of care for patients with hepatocellular carcinoma with vascular invasion. The efficacy of drugs such as atezolizumab bevacizumab combination therapy, sorafenib, lenvatinib, regorafenib, ramucirumab, and

cabozantinib is known, but they are not yet fully effective. Other treatment modalities have similarly failed to show adequate results, and there is a need to develop treatments.

This clinical trial presented here was planned as one of the studies to develop safer and more effective treatment.

Studies have reported the importance of aggressive treatment of the area of vascular invasion itself, such as surgery and radiation. Immune checkpoint inhibitors (ICIs) are being actively developed as anticancer agents, and it is expected that more patients will benefit from treatment by combining ICIs with other therapies rather than ICIs alone.

<u>About the investigational drugs “Durvalumab” and “Tremelimumab</u>

Previous studies have shown that human immune action slows or controls the rate of cancer growth. However, there are cases where the natural immune response does not work and the human immune response does not kill the cancer. Research has shown that some cancer cells and immune cells produce a signal that blocks the cancer-killing action. A new drug in development blocks this signal and amplifies the immune response. These are two new drugs, durvalumab and tremelimumab. These are antibodies (proteins produced by the body’s defense system). Durvalumab alone or in combination with tremelimumab may enhance the immune system’s ability to detect and fight cancer. The two drugs target different signals. Durvalumab targets a cancer cell signal called PD-L1 (Programmed Cell Death Ligand 1), while tremelimumab targets an immune cell signal called CTLA-4 (Cytotoxic T-Lymphocyte-associated Antigen 4) CTLA-4 (Cytotoxic T-Lymphocyte-associated Antigen 4). By blocking these signals, it is hoped that immune cells can again control or slow the growth rate of cancer.

Durvalumab is approved in Japan under the brand name Imfinzi® for the treatment of unresectable locally advanced non-small cell lung cancer, while tremelimumab is not yet approved in Japan. Clinical trials are underway in lung cancer, bladder cancer, head and neck cancer, and other types of cancer. Outside of Japan, it is approved by the U.S. Food and Drug Administration (FDA) for the treatment of patients with locally advanced or metastatic urothelial carcinoma whose cancer has progressed during or after platinum-based chemotherapy. It is also approved by the U.S. FDA and the European Medicines Agency (EMA) in Europe for the treatment of patients with locally advanced non-small cell lung cancer who have received chemoradiation therapy. However, durvalumab is not approved in combination with tremelimumab (either in Japan or overseas) for the treatment of cancer patients.

Although neither durvalumab nor tremelimumab has been approved for the treatment of hepatocellular carcinoma, studies to date have confirmed that there are no problems with its safety and studies are underway to confirm its efficacy. If you have any questions about the investigational drug, please ask your physician at any time.

Durvalumab

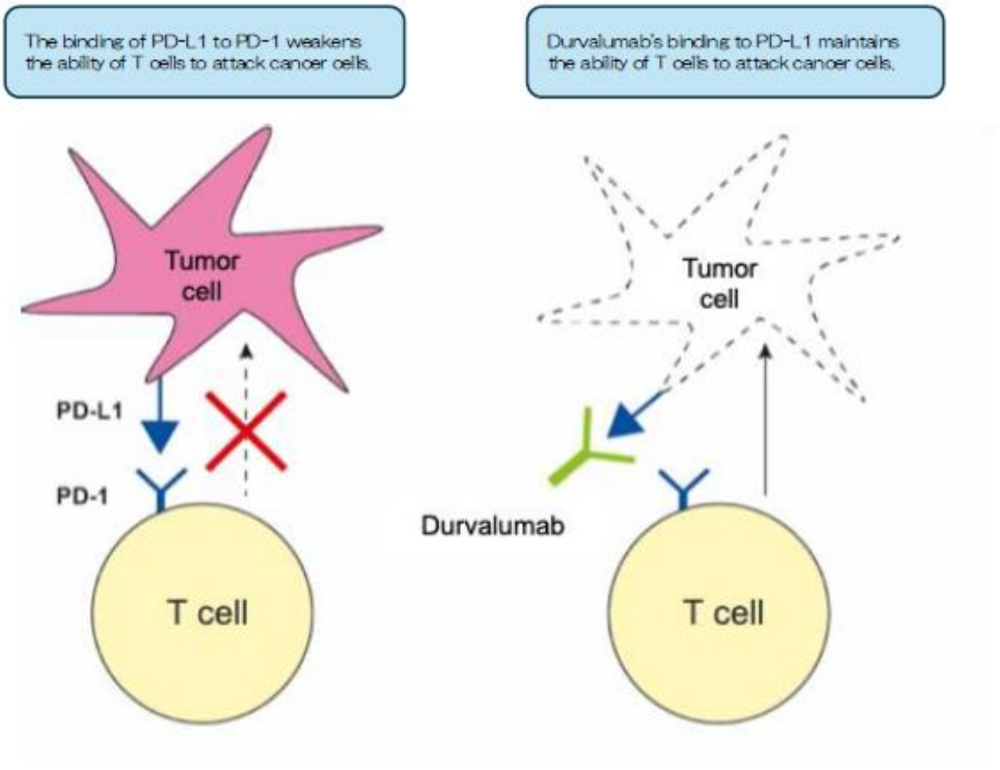

Tremelimumab

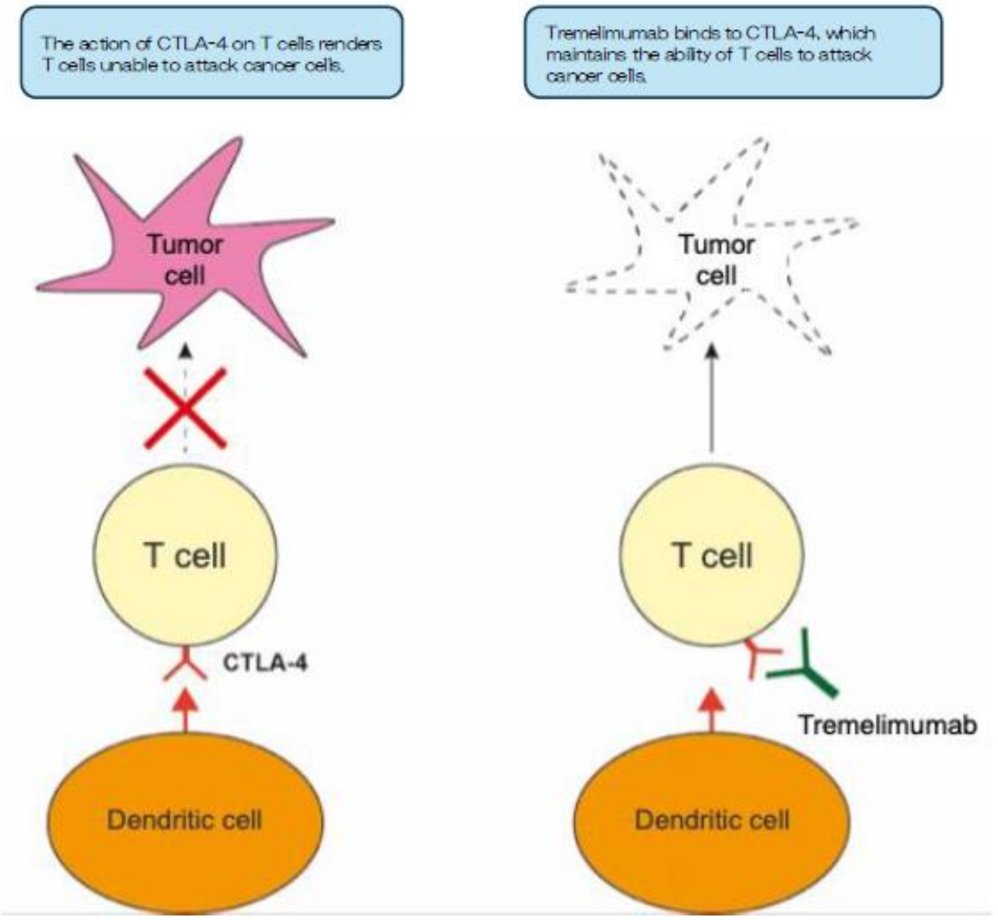

About Carbon Ion Radio Therapy

Carbon ion radio therapy is one of the radiation therapy methods for hepatocellular carcinoma. It is a new-generation treatment method classified as particle beam therapy along with proton beam therapy.

In contrast to conventional radiotherapy using X-rays, it can deliver high doses of radiation to the affected area while greatly minimizing irradiation to the normal liver. Studies of heavy-ion radiation therapy alone for hepatocellular carcinoma have reported that it is an effective treatment with minimal side effects.

Currently, research is being conducted in Japan under the advanced medical care system, in which patients bear the cost of their own treatment.

The participants in this study are scheduled to receive heavy particle therapy at the Hospital of the National Institute of Quantum Science and Technology.

3. Objective of the clinical trial

In this clinical trial, patients with advanced hepatocellular carcinoma will receive an infusion of durvalumab (or both durvalumab and tremelimumab) followed by treatment with heavy ion radiation. The main purpose of the study is to see what side effects may occur during this process and to determine whether this treatment can be safely administered.

The first 3-6 patients in the trial will receive durvalumab only, followed by heavy particle radiation. We will first review the physical condition of those who receive this treatment to make sure that the treatment can be safely administered.

Patients who participate after the review will receive both durvalumab and tremelimumab infusions and heavy particle therapy, which will also be evaluated again with respect to safety.

Based on the results of both treatments, we also aim to determine which treatment regimen is considered appropriate.

4. Method of the clinical trial

If you agree to participate in this clinical trial, you will first undergo a screening test. There are three periods in this clinical trial: the screening period, the treatment period, and the follow-up period after discontinuation of the clinical trial treatment.

During the screening period, you will undergo several tests and medical examinations to confirm that you are eligible to participate in this clinical trial treatment. Your investigator and the clinical trial staff will explain the clinical trial to you. The screening period is 28 days.

The treatment period is the time during which you will receive the study drug and heavy particle irradiation.

During the follow-up period, we will check your physical condition and blood test values after treatment is discontinued.

1）Eligibility

The conditions under which a patient may or may not participate in this clinical trial are as follows

≪ Patients who can participate in this study ≫

1) Patients diagnosed with hepatocellular carcinoma with vascular invasion

2) Patients with hepatocellular carcinoma not amenable to local therapies such as radiofrequency ablation (RFA) and trans arterial chemoembolization (TACE)

3) Patients must be 20 years of age or older

4) Weigh 30 kg or more

5) Good general condition and no major obstacles in daily life

6) Your test values (neutrophils, platelets, hemoglobin, liver function, renal function, etc.) meet certain criteria

7) Your consent to participate in this clinical trial has been obtained

《 Patients who cannot participate in this study 》

1) Brain metastasis

2) Currently suffering from cancer of an organ other than the liver, or suffered from cancer within 3 years

3) Received treatment with immunosuppressive agents within 14 days (28 days for antibody drugs) prior to the start of treatment with the investigational drug

4) Currently or previously had an autoimmune or inflammatory disease (unless the patient is determined to have had no active inflammatory disease within the past 5 years)

5) Have any of the following conditions or diseases

- Has serious heart disease (heart failure, myocardial infarction, angina pectoris, arrhythmia requiring treatment, etc.)
- Electrocardiogram (ECG) abnormalities
- Infectious disease requiring treatment with oral or injectable medications
- Severe respiratory illness (interstitial pneumonia or pulmonary fibrosis)
- Severe mental disorders (e.g., dementia)

- Positive test result for human immunodeficiency virus (HIV)
- Current or former hepatic encephalopathy
- Had cerebrovascular disease, thrombosis, or thromboembolism within 180 days prior to initiation of investigational therapy

6) Previous radiation therapy with liver effects.

7) Pregnant or lactating women.

8) Unable to consent to proper method of contraception during participation in the study and for 180 days from the date of the last dose of study drug (both sexes)

There are many other criteria, and we will make a judgment based on our detailed examination and consultation. Therefore, please understand that you may not be able to participate in the clinical trial even after you have given your consent, and even after the clinical trial has started, we may decide to terminate your treatment or participation in the clinical trial at our discretion. Patients who participate in this trial will be assigned to one of two treatment groups: durvalumab alone or a combination of durvalumab and tremelimumab as the drug to be combined with carbon ion radio therapy. In either group, you will be admitted to the hospital to start receiving durvalumab and tremelimumab, and you will also receive carbon ion radio therapy at QST Hospital. After completion of carbon ion radio therapy, patients will be discharged after confirming that they are in good physical condition, but the length of hospital stay will not change regardless of which group they are in. The length of hospitalization will be the same for both groups. Although the length of hospitalization may be longer depending on your physical condition, the expected length of hospitalization is approximately 3 weeks. After discharge from the hospital, patients will be followed up with outpatient visits.

The flow of this clinical trial is shown below.

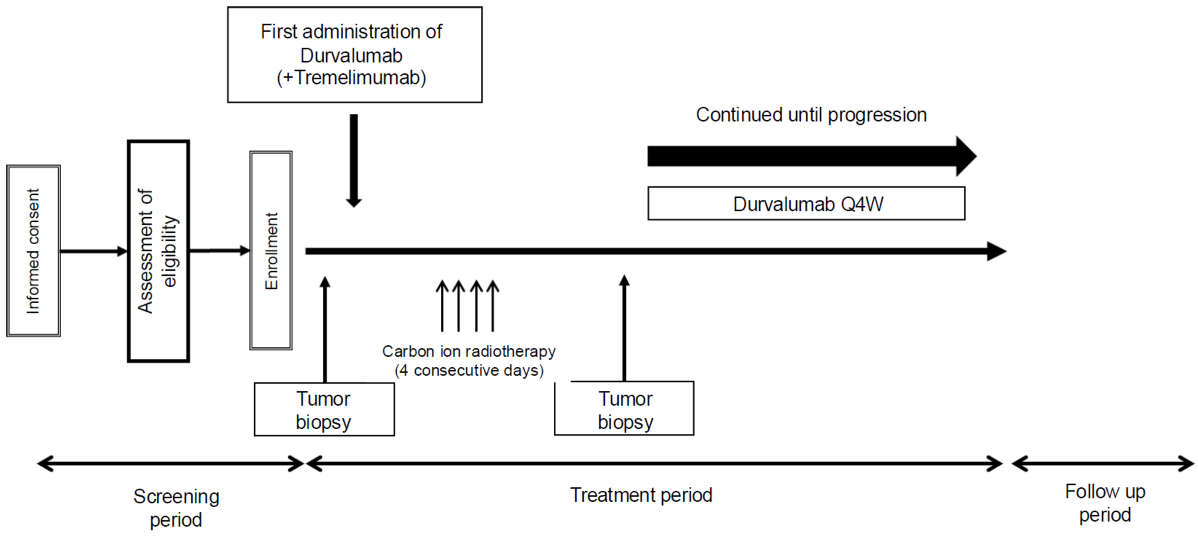

2) Detailed methods

The major objective of this study is to confirm that the combination of the investigational drug and carbon-ion radiation is safe for the human body. This confirmation of safety is called a Dose Limiting Toxicity (DLT) evaluation.

The first step in this evaluation is to assess whether the treatment is safe in two phases, with three to six patients receiving durvalumab followed by carbon ion radio therapy. This evaluation period will last 42 days from the start of treatment; if all three of the three patients, or at least five of the six patients, are found to be safe and there is no need to discontinue the trial, the next step, durvalumab and tremelimumab with carbon ion radio therapy will be given to an additional 3 to 6 patients. The same DLT evaluation will be performed on this group of patients. We plan to proceed with the clinical trial while confirming safety, and we expect a total of 15 patients to participate in the trial at our institution.

Depending on the timing of your participation in the clinical trial as described above, you will either receive durvalumab and carbon ion radio therapy or durvalumab plus tremelimumab and carbon ion radio therapy. Your investigator or clinical trial coordinator will explain this to you each time.

As a general rule, to ensure your safety, you will be hospitalized from the time you receive your first dose of study drug until the time you receive carbon ion radio therapy. Specific administration methods and other details will be explained next.

① Dosing schedule

In this trial, 28 days are counted as one cycle.

Durvalumab (or both durvalumab and tremelimumab) will be administered intravenously on Day 1 of Cycle 1. The infusion treatment takes about 1.5 hours when only durvalumab is given, and about 3 hours when both durvalumab and tremelimumab are given.

Carbon ion radio therapy is then administered over the next 8 days (4 days total).

From Cycle 2 onward, durvalumab will be administered intravenously once every 4 weeks (on Day 1 of each cycle) after confirming that it can be administered safely according to the "Guidelines for the Management of Toxicity in Investigational New Drugs".

Your physical condition will be monitored especially carefully during the first 42 days after the first dose. Durvalumab will be continued and treatment will be continued while monitoring side effects and changes in your condition.

②Carbon ion radio therapy (and targeted marker insertion)

In this treatment, carbon-ion radiation must be focused on the lesion as precisely as possible. Otherwise, normal tissue, not cancer cells, will be damaged. During the actual irradiation, you will lie on a treatment bed in a special treatment room, and you will be placed in a fixture to hold your body in place, and you will be asked to cooperate by not moving your body for a while. If you experience pain because the fixture does not fit properly, do not be patient and let us know. Carbon-ion radiation is given for a few minutes, but you will be in the treatment room for a total of 20 to 30 minutes, including the time needed for preparation and treatment before and after the irradiation. During the irradiation, you will be alone in the treatment room, but you do not need to worry because we will be watching you from outside the room via a TV camera, and if necessary, you can press the emergency buzzer to stop the irradiation and we will come to your room. You will not feel any pain or heat from the carbon-ion radiation during the irradiation.

Carbon ion radio therapy will be given once a day, four times a week, for a total dose of 60 Gy (RBE). This therapy will be completed in one week.

In this trial, a "target marker" is implanted in advance to accurately identify the lesion site during treatment. When the target marker is inserted from the surface of the body, local anesthesia is administered to the skin and subcutaneous tissue of the abdomen, a thin needle is inserted, and a tube is inserted into the liver. When inserted through a blood vessel in the liver, the target marker is implanted through a thin tube from an artery at the base of the groin to a site near the disease. The implanted target marker is not removed after treatment and remains in the organ, but there have been no reports of the marker directly harming the patient's health. When inserted from the surface of the body, a needle is inserted into the liver, which may cause bleeding, organ damage, and other side effects similar to liver biopsy

(details are described in the explanation section of liver biopsy on p. 23). When inserting a catheter through a blood vessel in the liver, a method called abdominal angiography is applied. A thin tube called a catheter is inserted from a blood vessel in the leg or arm, and the catheter is advanced to a blood vessel near the tumor in the liver. A targeted marker will then be implanted in a blood vessel near the tumor. Side effects associated with abdominal angiography (such as allergic reactions to contrast media, renal dysfunction, vascular injury, bleeding, infection, and organ damage) may occur.

3）Details and schedule of tests during the clinical trial

After you agree to participate in a clinical trial and sign the Clinical Trial Consent Form, screening tests will be performed to determine if you are suitable to participate in the trial. If the results of these tests can be substituted for the tests that were performed prior to your consent, we may use the results of those tests as data for the clinical trial. However, please understand that depending on the results of the tests, you may not be able to participate in the clinical trial. During the clinical trial period, including the screening tests, you will be taken for periodic medical examinations and consultations in accordance with the schedule shown in Table 1. In addition, after the administration of the investigational drug has been completed, we will also conduct medical examinations and consultations.

(A) Schedule of medical examinations and tests.

Investigations include measurement tests by blood and urine collection, electrocardiogram, CT and MRI, and follow-up by biopsy to collect tissue (liver tumor and non-tumor tissue). These tests are commonly performed

to treat your disease and ensure your safety and are necessary for this clinical trial. If the results of the tests are deemed necessary by your physician, your visits and tests may be more frequent than those planned in Table 1.

(B) Tumor marker

There are many types of cancer, some of which produce substances characteristic of each cancer. Among such substances, those that can be measured in body fluids (mainly blood) are called tumor markers.

are called tumor markers.

Together with blood tests and imaging tests, tumor markers are used as one of the tests to check the progress of cancer and the progress of treatment.

In this case, blood will be used to measure AFP, AFP-L3, and PIVKA2, which are tumor markers for hepatocellular carcinoma. Blood samples will be drawn prior to the start of the study and prior to the administration on the first day of each of Cycle 2 and thereafter.

(C) Liver biopsy and liver tumor biopsy

Liver biopsy and liver tumor biopsy (tissue collection from non-tumor and tumor areas) will be performed on patients who have agreed to undergo liver biopsy and liver tumor biopsy. The timing of the liver biopsy/liver tumor biopsy will be before treatment and after the safety of the treatment has been confirmed for 42 days after the start of treatment. Liver tissue (non-tumor and tumor areas) obtained by biopsy will be used for exploratory studies.

Table 1: Schedule of medical examinations and tests

Cycle	Screening period	First tumor biopsy	DLT evaluation period							Second tumor biopsy	Durvalumab q4W dosing period					ST	Follow up period	Safety information collection
			Cycle 1				Cycle 2				Cycle 3	Cycle4	Cycle 5	Cycle 6	Cycle 7 and beyond			
Cycle Day	D -28 ～D -1	D -28～D -1	1	8 to 14	15	22	1	8	14		1	1	1	1	1	—	—	—
Allowable period（Day）			—	±3	±3	±3	±3	±3	±3		—	±3	±3	±3	±3	+14	+14	+14
Informed Consent ／ Subject backgroud information／ Review of eligibility	●																	
Durvalumab administration (cohort A and B)			●				●				●	●	●	●	●			
Tremelimumab administration (cohort B)			●															
Tumor biopsy		●								●								
CIRT（QST hospital）				● ● ● ●														
Fiducial marker insertion		●																
Fixation, simulation CT (for CIRT)	●																	
Weight	●		●	●	●	●	●	●	●		●	●	●	●	●	●	●	
Height	●																	
Clinical Chemistry／Hematology	●		●	●	●	●	●	●	●		●	●	●	●	●	●	●	
Urinalysis	●		●								●	●	●	●	●	●	●	
ECG	●		●													●		
Chest X ray	●															●		
CT/MRI	●		Every 6 weeks (±1 week) for the first 12 weeks from Cycle1 day1, and every 8 weeks (±1 week) thereafter until PD.															
Tumor marker（AFP、PIVKA-II）	●						●				●	●	●	●	●			
Assessment of AE/SAE	←→																	

Table 2: Laboratory Tests for Blood and Urinalysis

Hematological Tests	Red blood cell count, Hemoglobin, Hematocrit, Platelet count, White blood cell count, White blood cell fraction（Neutrophils, Lymphocytes, Monocytes, Eosinophils, Basophils)
Biochemical examination of blood	Total protein, Albumin, Total bilirubin, AST, ALT, ALP, γ-GTP, LDH, BUN, Amylase, Lipase, Creatinine, Uric acid, Na, K, Cl, Ca, P, Mg, Blood sugar
Blood coagulation test	PT-INR、APTT
Urinalysis	Specific gravity, pH, Sugar, Protein, Ketones, Occult blood, Bilirubin, Color and appearance, Urinary sediment

5. Specimen (blood and tissue) storage

In principle, the specimens you provide for testing (blood and liver tissue (tumor and non-tumor parts)) will be stored until the end of the clinical trial.

If you give your consent, we plan to keep the specimens remaining after testing for a certain period of time (up to 20 years from the start of the clinical trial) at the Department of Gastroenterology of our hospital even after the trial has ended. This is because there is a possibility that additional research on hepatocellular carcinoma may be conducted in the future based on newly obtained findings. If used for research, it will be used only after approval by the Review Committee. When storing the specimens, we will anonymize them so that it will not be known that the specimens were collected from you.

You may participate in a clinical trial even if you do not consent to the storage of specimens after the trial is completed. You will not be disadvantaged by not consenting. Please make your decision based on your

own judgment, as there is a confirmation box on the clinical trial participation consent form.

You can withdraw your consent to the storage of specimens after the completion of the clinical trial at any time. If you withdraw, you will not be disadvantaged in any way. If you wish to withdraw your consent, please sign the Consent Withdrawal Form and submit it to your investigator or study coordinator.

However, if the person in charge at the hospital has already collected the analysis results before you submit the withdrawal of consent form, the results will not be used as they are.

If the results of the analysis have been collected by the person in charge at the hospital before you submit the withdrawal of consent, the results may be used as they are and shared with other parties.

If it is determined that there is no further need to store specimens before 20 years have passed from the start of the clinical trial, the specimens may be discarded without waiting 20 years from the start of the clinical trial.

In addition, since the results obtained are still in the research phase, they will not be disclosed to you in principle. If you wish to request disclosure, please contact us.

6. Duration of participation in the clinical trial and number of participants

Approximately 15 patients are expected to participate. The expected duration of participation in the clinical trial will be the total of the screening period (28 days) and the treatment and post-treatment follow-up periods (28

days after completion of treatment). The treatment period will be the sum of 42 days after the first dose and the period of continued durvalumab administration.

The duration of durvalumab treatment will depend on your condition and will continue as long as your investigator determines that it is effective for your disease. However, it may be discontinued if serious side effects occur and it becomes difficult to continue treatment, even if it is effective.

7. Foreseeable benefits

The combination of durvalumab tremelimumab with carbon ion radio therapy may demonstrate efficacy not seen with existing therapies.

Results of an AstraZeneca-led study of durvalumab-tremelimumab in advanced hepatocellular carcinoma have shown that each agent is safe as a single agent and that the combination of both agents has promising results. carbon ion radio therapy for hepatocellular carcinoma has also been shown to be safe and effective and is currently approved as an advanced medical care.

However, the safety and efficacy of durvalumab-tremelimumab in combination with carbon ion radio therapy is not known. Information obtained from this clinical trial may be used in future research on the treatment of this disease.

8. Foreseeable disadvantages and side effects

The side effects that may develop as a result of treatment vary greatly from person to person, and what symptoms may occur and their severity vary from person to person, and cannot be completely predicted before treatment begins. When side effects occur, treatment may be temporarily stopped, or treatment may be given to alleviate symptoms.

Serious side effects may also occur when treatment is resumed after a suspension or postponement of treatment.

If you feel that your condition is unusual, please contact your investigator for appropriate treatment.

Risks associated with durvalumab and tremelimumab.

Most of the side effects seen with durvalumab and tremelimumab were mild or moderate. However, some are serious, life-threatening and sometimes fatal. Some side effects do not require treatment, but symptoms usually recover with treatment. It may be necessary to delay the administration of durvalumab and tremelimumab to improve the symptoms of side effects. The most important side effects that may occur are listed below. These can occur as a result of the action of durvalumab and tremelimumab on the immune system and have been seen in patients who received either or both durvalumab and tremelimumab in previous studies. These side effects have also been seen in trials using other medicines similar to durvalumab and tremelimumab. The management of these side effects may require the administration of steroids or other medications that can work on the immune system and reduce inflammation.

The types of side effects were very similar when these two investigational drugs were given together and when durvalumab was given alone. However, the probability and severity of many, but not all, of these side effects were higher when they were given in combination than when durvalumab was given alone.

Very common side effects (>10%)

・ Diarrhea

・Rash / Dry and itchy skin

・ Liver dysfunction: Blood levels of substances called enzymes, which are found in liver cells, may increase. Changes in the enzyme do not often make you feel sick. However, if this enzyme level is very high, your investigator may need to discontinue the study drug. It may also cause inflammation of the liver, known as hepatitis, but this is rare. It may be accompanied by signs and symptoms such as yellowing of the skin and white eye area, dark urine, severe nausea and vomiting, pain in the upper right abdomen, itchy skin, inability to feel hunger, and bleeding or bruising more easily than normal.

In addition to the above, important anticipated side effects reported to be very common (i.e., >10%) in clinical trials in which patients with different types of cancer were treated with durvalumab alone or in combination with tremelimumab were fatigue, abdominal pain, swelling due to fluid retention, upper respiratory tract infection, nausea, vomiting, decreased appetite, shortness of breath, cough, fever, and muscle and joint pain.

Most common side effects (≥1% to <10%)

・ Hypothyroidism: This is caused by a decrease in the amount of thyroid hormones produced by the thyroid gland and a very slow metabolism. Symptoms include, but are not limited to, fatigue, feeling cold more easily, constipation, dry skin, unexplained weight gain, facial swelling, muscle weakness, decreased heart rate, thinning hair, and memory problems. These symptoms can be improved by thyroid hormone replacement. This eventis a very common side effect in patients receiving the combination of durvalumab and tremelimumab, but is classified as a common side effect in patients receiving durvalumab alone.

・ Pneumonia: Symptoms include, but are not limited to, new or worsening cough, shortness of breath (sometimes accompanied by fever). Pneumonia can be fatal. Limited data (not yet fully established) suggest that the incidence and severity of the disease may be higher in Japanese than in non-Japanese. If you have any of these symptoms, contact your investigator immediately.

・ Hyperthyroidism: This condition occurs when the thyroid gland produces too much thyroid hormone. Symptoms include anxiety, nervousness, weight loss, frequent bowel movements, diarrhea, shortness of breath, hot flashes, and heart palpitations. Depending on the severity of the symptoms, treatment may include observation only, symptomatic treatment, or treatment to stop the secretion of thyroid hormones.

・ Renal dysfunction: Even without symptoms or feeling sick, blood tests may show increased creatinine levels (creatinine is a protein marker that assesses kidney function). Less frequently, nephritis may occur, in which the kidneys become inflamed and lose normal function.

・Nervous system disorders: Symptoms include abnormal weakness of the leg, arm, or facial muscles, or numbness or tingling in the limbs. Rarely, severe inflammation of the nervous system may occur, which can damage nerve cells and interfere with communication between nerves and muscles. If you experience trouble swallowing, sudden weakness, or difficulty breathing, contact your doctor immediately.

・ Injection reactions: Reactions may occur during or after injection of the investigational drug. Injection reactions may cause fever or chills, changes in blood pressure, or significant dyspnea. Contact your investigator immediately if you experience any of these symptoms, even if it is several days after the injection.

・Intestinal inflammation (colitis): May cause abdominal pain and diarrhea (with or without bleeding). May be accompanied by fever. Additional intravenous fluids may be needed. If left untreated, it can cause serious and life-threatening tears in the intestinal wall. If you experience any of these symptoms, contact your doctor immediately.

・ Elevated levels of pancreatic enzymes (amylase and lipase). These enzymes are indicators of pancreatic function. In rare cases, elevated levels of these enzymes may be associated with pancreatitis (see "Rare Side Effects" below).

In addition to the above, the most common adverse reactions reported as serious (i.e., ≥1% to <10%) in clinical trials in which durvalumab was administered alone or in combination with tremelimumab to patients with various types of cancer are pneumonia, hoarse voice, urinary pain, night sweats, oral candidiasis, and muscle and joint pain.

Common side effect (≥1% to <10%) (occurs in 1 out of 10-100 patients) / Rare side effect (<1%) (occurs in 1 out of 100-1000 patients)

・ Adrenal Injury: May cause stomach pain, vomiting, muscle weakness, fatigue, mood swings, hypotension, weight loss, kidney problems, and mood and personality changes. It has been reported to occur more frequently in patients receiving the combination of durvalumab and tremelimumab, but less frequently in patients receiving durvalumab as a single agent. These complications may require permanent hormone replacement therapy.

・ Dental and oral soft tissue infections and influenza were more common in patients who received durvalumab as a single agent and rare in those who received it in combination with tremelimumab.

Rare (<1%) (occurs in 1 out of 100 to 1000 patients)

・ Pancreatitis: Pancreatitis usually presents with persistent pain in the upper abdomen (which may be made worse by eating or drinking), nausea, vomiting and weakness. Pancreatitis usually improves with simple treatment but can be severe and life-threatening. Contact your investigator immediately if you have any of these symptoms.

・ Allergic reaction: An allergic reaction may cause swelling of the face, lips, or throat, or breathing difficulties accompanied by hives or a hives-like rash. If any of these symptoms occur, contact your investigator immediately.

Rare (<1%) (occurs in 1 out of 100-1000 patients) / Very Rare (<0.1%) (occurs in 1 out of 1000-10000 patients)

・Myositis/Polymyositis: Symptoms include muscle weakness, myalgia, fatigue while standing or walking, and muscle pain lasting several weeks. It was rare in patients treated with durvalumab as a single agent and very rare in patients treated with tremelimumab in combination.

・Pituitary disorder (hypopituitarism): Hypopituitarism is a decrease in the hormone secreted by the pituitary gland in the brain and is caused by inflammation of the pituitary gland (hypopituitarism). Symptoms include headache, thirst, difficulty seeing or double vision, leakage of breast milk in women, or irregular menstruation. These complications may require permanent hormone replacement therapy. They were rare in patients who received durvalumab in combination with tremelimumab and very rare in patients who received durvalumab as a single agent.

・ Inflammation of the heart muscle (myocarditis): symptoms include chest pain, tachycardia, irregular heartbeat, shortness of breath, and swelling of the legs. If you have any of these symptoms, contact your physician immediately. This side effect is classified as an uncommon side effect in patients receiving the combination of durvalumab and tremelimumab, but very rare in patients receiving durvalumab alone.

Very Rare Adverse Reactions (<0.1%) (occurs in 1 out of 1000-10000 patients)

・Type 1 diabetes (may cause elevated blood glucose levels, known as hyperglycemia): Symptoms include weight loss, increased urination, increased thirst and hunger. type 1 diabetes requires insulin replacement by injection. If you have any of these symptoms, contact your doctor immediately.

In addition to the above, important predicted disadvantages reported as rare (i.e., less than 0.1% of patients) in clinical trials of single-agent durvalumab in patients with different types of cancer are inflammation of the membranes surrounding the heart, increased number of small clusters of inflammatory cells in various parts of the body, inflammation of the middle layer of the eye or Other eye abnormalities (e.g., inflammation of the cornea and optic nerve), inflammation of the brain or membranes lining the brain and spinal cord, hardening or straining of skin and connective tissue and loss of skin color, hematologic abnormalities (e.g., abnormal red blood cell destruction, thrombocytopenia), vascular inflammation, and rheumatic abnormalities (muscle pain and stiffness caused by inflammatory disease and autoimmune autoimmune arthritis).

In addition to these expected disadvantages when durvalumab is administered alone or in combination with durvalumab and tremelimumab, other immune-mediated side effects not previously observed may occur, and inflammatory side effects may occur in any organ or tissue.

<u>Side effects of carbon ion radio therapy</u>

１）Gastrointestinal tract

In the case that the cancer is located close to the liver surface and in close proximity to the gastrointestinal tract, such as the stomach, duodenum, or large intestine, carbon ion radio therapy may cause damage to these digestive tracts. Anorexia, nausea, diarrhea, and abdominal pain are the most common symptoms, but it is important to note that anemia due to bleeding may be detected without obvious symptoms. The symptoms range from mild erosions and ulcers that can be treated with medication and diet to severe cases that require hospitalization, such as bleeding, perforation (hole), stenosis (narrowing, making it difficult for objects to pass through), obstruction (blockage of the lumen, making it impossible to pass objects through), and adhesions. Based on the results of clinical trials on cancers in other parts of the body, we can predict to some extent the safe dose when irradiated to the gastrointestinal tract, and we will make sure that the dose irradiated to the gastrointestinal tract is less than the dose considered safe to avoid serious complications. To date, a very small number of patients have experienced mild side effects (less than 1%) that improve with medication, but there have been no serious side effects that would require invasive treatment such as surgery.

２）Skin

Various degrees of radiation dermatitis may occur as a result of carbon ion radio therapy. In most cases, the symptoms are similar to a mild sunburn (redness, itching, mild pain, etc.), but in some cases, the surface of the skin may peel, producing exudates, hyperpigmentation, and small scars (scars). If the lesion is located relatively close to the skin, the skin is also exposed to high doses of radiation, which may cause erythema, erosions, ulcers, atrophy, etc., which may require treatment with ointments. Surgical procedures such as skin grafts may be necessary, although rarely (0-2.8% in past reports), and are becoming less common due to improvements in treatment techniques.

３）Liver

Liver function in the area exposed to carbon-ion radiation will be reduced or eliminated, and overall liver function may also be reduced. In this study, treatment is designed with the expectation that sufficient liver function will remain, but it is known that radiation-induced liver injury (RILD) will occur in 1-2% of patients.

４）Bile duct

Carbon-ion radiation therapy may cause cholangitis, but there have been no cases of cholangitis directly attributable to this treatment. Although rare (less than 1% of cases), bile duct stricture may require medical treatment.

5）Lung

If a portion of the lung is irradiated with carbon-ion radiation, imaging changes, pleural effusion, coughing, fever, and dull pain may occur. In severe cases, sputum, blood sputum, shortness of breath, interstitial pneumonia-like shadows, pulmonary fibrosis-like shadows, and respiratory failure are possible, but since only a small portion of the lung is usually irradiated in the treatment of hepatocellular carcinoma, no serious side effects that could be caused by carbon ion radio therapy have been observed.

6）Blood

Decreases in peripheral blood white blood cell count, red blood cell count, and platelet count, and decreases in hemoglobin concentration may occur.

7）Possibility of secondary carcinogenesis

In the case of conventional radiotherapy, there is a rare possibility of new cancer development (secondary cancer) caused by radiation after a long period of time, and the same is possible with carbon ion radio therapy.

Other risks anticipated from participation in this clinical trial include

Blood sampling: Risks associated with blood sampling include temporary discomfort from needle puncture, internal bleeding, hemorrhage, and in rare instances, infection and anemia may be caused.

ECG: Risks associated with ECG testing include temporary discomfort such as itching, mild irritation, or redness of the skin where the small adhesive pad is applied. If it is necessary to shave the area where this small adhesive pad is applied, the shaving may cause irritation.

CT or MRI: You may experience some discomfort or anxiety when lying down inside the CT scan machine. The injection of contrast may cause a metallic taste in the mouth, a feeling of warmth, and in rare cases, nausea or vomiting. A reaction to the contrast agent may also occur. MRI is safe for most people. People with metal implants near vital organs cannot undergo MRI. This is because the metal can be drawn from the body to a large magnet, which can cause damage.

Biopsy (collection of liver tumor and non-tumor tissue): A biopsy is the collection of a piece of your tissue using a scalpel or needle. This time, the needle will be inserted directly into your liver, which may cause mild discomfort and internal bleeding at the site of the needle puncture. Bleeding, liver dysfunction, jaundice, infection, pneumothorax, and peritoneal seeding may also occur. The frequency of percutaneous puncture of the liver in routine examinations is about 2%. Although rare, surgery or other procedures and blood transfusions may be required for the above treatments. In addition, anesthetics and sedatives used prior to administration may cause a sudden drop in blood pressure or an irregular heartbeat, which may strain the heart. Side effects such as allergic reactions and fever may also occur.

If you become pregnant during a clinical trial, or if your partner becomes pregnant, please notify your investigator or the study coordinator immediately. If you become pregnant, your participation in the clinical trial will be terminated.

If you or your partner becomes pregnant, we will ask you for this information. If you or your partner gave birth, had a miscarriage or an abortion, please tell your investigator. If you or your partner gave birth, you may be

asked to provide information including the baby's date of birth, height and weight at birth, sex of the baby, any complications during pregnancy or delivery, and any birth defects of the baby.

9. Free voluntary participation in the clinical trial and the ability to withdraw consent at any time

Participation in this clinical trial is not mandatory, so please make your own decision.

You may withdraw from the study at any time after participating in the study, regardless of the reason, if you wish to discontinue the study or if it is difficult to continue, so please consult your physician. You will not be disadvantaged by the discontinuation of the clinical trial. After the discontinuation of a clinical trial, you will be informed of the best possible treatment by your physician.

Your doctor will then explain the best treatment options to you.

10. Alternative treatment methods if you do not participate in this clinical trial

If you do not participate in this clinical trial, please ask your physician to explain in detail the other treatment options that may be available to you. You will not be disadvantaged by not participating in this clinical trial. Your physician will discuss with you the best treatment for your condition. Options include participating in a clinical trial for another drug, treatment with an already approved drug, or radiation therapy. Supportive care (pain and suffering control) is also available.

11. Discontinuation after participation in a clinical trial

Even after you have given your consent to participate in a clinical trial, we may discontinue the clinical trial treatment for the following reasons

① When you request discontinuation.
② In case of strong side effects during the clinical trial
③ When cancer has become large
④ When it is deemed difficult to continue the clinical trial due to circumstances at the hospital where the clinical trial is being conducted.
⑤ Other cases in which the investigator determines that discontinuation of the clinical trial is necessary.

28 days after the end of treatment, an examination will be conducted to confirm safety. Even if the clinical trial is terminated due to side effects, we ask for your cooperation as we may conduct tests and medical examinations until the side effects are no longer present.

Even if the clinical trial is terminated during the course of the study, we would like to use the records up to that point, as they will be valuable materials for future cancer research. If you have any concerns about the use of your records, please contact us.

12. Regarding any new important information obtained

We will promptly inform you if we obtain new information on important efficacy, safety, or other information that we believe may affect your decision to continue participating in this clinical trial. In that case, you will be asked to make a new decision as to whether or not you wish to continue participating in the clinical trial.

13. Compensation for health damage related to clinical trials

If you experience any problems related to the investigational drug, or if you experience any health problems as a result of participating in a clinical trial, you should notify your investigator immediately. The physician will treat you for any health problems that arise as a result of your participation in a clinical trial, and you may be eligible for compensation. However, compensation may not be provided in the following cases

① When there is no causal relationship between the health hazard and the clinical trial
② In case of progression of hepatocellular carcinoma due to inadequate efficacy of the investigational drug
③ If the health damage was caused by your intentional or gross negligence

For more information, please consult your investigator or consultation service.

14. Costs during the study

If you participate in this clinical trial, the investigational drug will be provided by the investigational drug provider (AstraZeneca Corporation). In addition, the hospital will cover the costs associated with heavy particle irradiation. We will also cover the cost of transportation to and from the QST Hospital for carbon ion radio therapy while you are hospitalized at Chiba University Hospital. The costs of medical examination fees, tests, drugs normally used, drugs used for side effects, tests and diagnostic imaging, etc. will be borne by the patient according to the type of health insurance as in the past. You will also be responsible for the cost of hospitalization for this clinical trial treatment.

15. Access to medical records and preservation of participant confidentiality

Any personally identifiable information that may be used to identify an individual who has cooperated in a clinical trial will be strictly protected and will not be released to outside parties.Data obtained from patients will be anonymized by code numbers, etc., and will be handled in such a way that they will not be identified as belonging to that patient in reports, etc.

The results of this clinical trial will be submitted to the Ministry of Health, Labour and Welfare (MHLW) in order to have the government (MHLW) approve the combination therapy of durvalumab tremelimumab and heavy ion therapy as a treatment for advanced hepatocellular carcinoma with vascular invasion. In addition, in order to study the efficacy and safety of this investigational drug in more detail, data necessary to evaluate the investigational drug, such as tests, images, and electrocardiograms used as data in the clinical trial, may be submitted to an outside organization.

Furthermore, information obtained from this clinical trial may be compiled from records collected from hospitals in various regions and published in academic societies and medical journals. In all cases, however, your name will not be used and your personal information (name, address, telephone number, etc.) will be kept confidential and will not be leaked to outside parties.

In addition, in order to check whether the clinical trial is being conducted properly, personnel from the investigational drug development organization, the Clinical Trial Review Committee of this hospital, and regulatory authorities such as the Ministry of Health, Labor and Welfare may access your medical records, including your medical records from other departments and the period before your participation in the clinical trial. Even in such cases, these officials are obligated to maintain confidentiality and your privacy will be protected.

Even if you have received treatment at another medical institution, we may contact your physician at the other institution by phone or letter to request

medical information. By signing the consent form at the end of this letter, you are also giving your consent to access your records and to the collection of information from other medical institutions.

You may withdraw your consent to the use of your medical information at any time, but please note that you will not be able to participate in this clinical trial after that. If you wish to withdraw your consent, please inform your doctor or hospital staff in charge. The clinical trial data collected before you revoke your consent will be used in the same way as described above, but after you revoke your consent, your medical information will not be used except to confirm that this clinical trial has been conducted properly.

16. Conflicts of Interest

This study is being funded and conducted by AstraZeneca Inc. At the same time, we are receiving the investigational drug and information on the safety of the drug, but there is no profit from the conduct of this study itself or from the analysis and reporting of the results. This has been reviewed by our Conflict-of-interest Management Committee, which has confirmed that no conflicts of interest* (possible conflicts of interest) exist with regard to the implementation of this clinical trial.

*Conflicts of interest are defined as actions that may compromise patient safety or distort the interpretation of data for the benefit of oneself or a pharmaceutical company, for example, when receiving funding or other benefits from a pharmaceutical company.

17. Your responsibilities during the study period

If you agree to participate in this clinical trial, please observe the following

1) Please follow the trial schedule during your participation in the trial.

2) If you are currently receiving treatment or taking medication at another department or hospital, please tell your investigator or coordinator about

your situation in detail. We will inform your treating physician that you are participating in this clinical trial.Also, if you are taking any over-the-counter medications, please consult your investigator or the clinical trial coordinator in advance.

3) The tests that will be performed during the clinical trial are very important for us to know about any changes in your condition or side effects, so please follow the instructions of your investigator and the clinical trial coordinator.

4) If you experience any changes in your physical condition while using the investigational drug, please inform your investigator or the study coordinator.

5) Please follow any other precautions or instructions given by the investigator in charge of the clinical trial.

18. Institutional Review Board that has reviewed this study

The clinical trial is reviewed by the Institutional Review Board below, not only from a scientific and medical perspective, but also from an ethical perspective, including patient safety and human rights. The Clinical Trial Review Committee includes members who do not specialize in medicine or have no vested interest in our hospital.

1)Name: Chiba University Hospital Institutional Review Board

2)Type: Institutional Review Board

3)Founder: Director of Chiba University Hospital

4)Address: 1-8-1 Inohana, Chuouku, Chiba

After the clinical trial has started, we will review, upon request from the director of this hospital, whether this clinical trial can be continued if the above information is changed or if safety information, such as the occurrence of serious side effects, is obtained.

The results of the review will be reported to the director of this hospital, who will decide whether to initiate or continue the clinical trial at this hospital based on the contents of the report.

19. Contact information

If you have any questions, doubts, questions, or would like to ask again or get more detailed information about this clinical trial or treatment, please do not hesitate to ask us at any time. Even after the clinical trial has started, we will be happy to answer any questions you may have. Also, if you have any concerns about the use of this investigational drug, please do not hesitate to contact us at any time.

If you agree to participate in this clinical trial after fully understanding the details of this trial, please indicate the date of your consent and sign the consent document at the end of this explanatory document.

Please keep this Explanatory Document and the Consent Document for patients in a safe place.

The information of the Institutional Review Board (procedure manual, committee roster, and summary of meeting records) is available to the public on our website (http://www.ho.chiba-u.ac.jp) and can be freely accessed. If you would like to confirm the procedure manual, etc. directly, please ask the clinical trial coordinator or others.

Site：Chiba University Hospital

Principal Investigator：Naoya Kato

Your Investigator：______________________________

Contact us below

Weekdays (8:30-17:00)

Outpatient Gastroenterology Tel：+8143-222-7171

Clinical Trial Coordinator Office Tel：+8143-226-2630

Nighttime and Holiday Phone Service Tel：+8143-222-7171

*Please inform us that you are participating in a clinical trial in gastroenterology.

For investigator

Consent Form

I hereby give my consent to participate in the “Phase Ib Clinical Trial to Evaluate the Safety and Efficacy of Durvalumab Tremelimumab in Combination with Heavy Ion Beam Therapy in Patients with Advanced Hepatocellular Carcinoma with Vascular Invasion” on my own free will, after having received and fully understood the following information. I agree to participate in this clinical trial of my own free will after having received and fully understood the following information.

- What is a clinical trial?
- About your disease and treatment
- Objective of the clinical trial
- Method of the clinical trial
- 治験のスケジュール
- Specimen (blood and tissue) storage • Duration of participation in the clinical trial and number of participants
- Foreseeable benefits
- Foreseeable disadvantages and side effects
- Free voluntary participation in the clinical trial and the ability to withdraw consent at any time
- Alternative treatment methods if you do not participate in this clinical trial
- Discontinuation after participation in a clinical trial
- Regarding any new important information obtained
- Compensation for health damage related to clinical trials
- Costs during the study
- Access to medical records and preservation of participant confidentiality
- Conflicts of Interest
- Your responsibilities during the study period
- Institutional Review Board that has reviewed this study
- Contact information

●Performing liver biopsy and liver tumor biopsy (before/after 42 days of treatment)

☐ Agree ☐ Disagree

●Storage of specimens after completion of the clinical trial

☐ Agree ☐ Disagree

Participant

Date of consent：　　　　　　(Month, Day, Year)

Signature:：

The investigator who obtained consent

Date of signature　　　　　(Month, Day, Year)

Signeture：

The person who provided supplementary explanation

Date of signature　　　　　(Month, Day, Year)

Signeture：

For site

Consent Form

I hereby give my consent to participate in the “Phase Ib Clinical Trial to Evaluate the Safety and Efficacy of Durvalumab Tremelimumab in Combination with Heavy Ion Beam Therapy in Patients with Advanced Hepatocellular Carcinoma with Vascular Invasion” on my own free will, after having received and fully understood the following information. I agree to participate in this clinical trial of my own free will after having received and fully understood the following information.

- What is a clinical trial?
- About your disease and treatment
- Objective of the clinical trial
- Method of the clinical trial
- 治験のスケジュール
- Specimen (blood and tissue) storage • Duration of participation in the clinical trial and number of participants
- Foreseeable benefits
- Foreseeable disadvantages and side effects
- Free voluntary participation in the clinical trial and the ability to withdraw consent at any time
- Alternative treatment methods if you do not participate in this clinical trial
- Discontinuation after participation in a clinical trial
- Regarding any new important information obtained
- Compensation for health damage related to clinical trials
- Costs during the study
- Access to medical records and preservation of participant confidentiality
- Conflicts of Interest
- Your responsibilities during the study period
- Institutional Review Board that has reviewed this study
- Contact information

●Performing liver biopsy and liver tumor biopsy (before/after 42 days of treatment)

☐ Agree ☐ Disagree

●Storage of specimens after completion of the clinical trial

☐ Agree ☐ Disagree

Participant

Date of consent：＿＿＿＿＿＿(Month, Day, Year)

Signature:：＿＿＿＿＿＿＿＿＿＿＿＿

The investigator who obtained consent

Date of signature ＿＿＿＿＿＿(Month, Day, Year)

Signeture：＿＿＿＿＿＿＿＿＿＿

The person who provided supplementary explanation

Date of signature ＿＿＿＿＿＿(Month, Day, Year)

Signeture：＿＿＿＿＿＿＿＿＿＿

For particopant

Consent Form

I hereby give my consent to participate in the “Phase Ib Clinical Trial to Evaluate the Safety and Efficacy of Durvalumab Tremelimumab in Combination with Heavy Ion Beam Therapy in Patients with Advanced Hepatocellular Carcinoma with Vascular Invasion” on my own free will, after having received and fully understood the following information. I agree to participate in this clinical trial of my own free will after having received and fully understood the following information.

・What is a clinical trial?
・About your disease and treatment
・Objective of the clinical trial
・Method of the clinical trial
・治験のスケジュール
・Specimen (blood and tissue) storage・
Duration of participation in the clinical trial and number of participants
・Foreseeable benefits
・Foreseeable disadvantages and side effects
・Free voluntary participation in the clinical trial and the ability to withdraw consent at any time
・Alternative treatment methods if you do not participate in this clinical trial
・Discontinuation after participation in a clinical trial
・Regarding any new important information obtained
・Compensation for health damage related to clinical trials
・Costs during the study
・Access to medical records and preservation of participant confidentiality
・Conflicts of Interest
・Your responsibilities during the study period
・Institutional Review Board that has reviewed this study
・Contact information

●Performing liver biopsy and liver tumor biopsy (before/after 42 days of treatment)

□ Agree □ Disagree

●Storage of specimens after completion of the clinical trial

□ Agree □ Disagree

Participant

Date of consent：__________ (Month, Day, Year)

Signature:：____________________

The investigator who obtained consent

Date of signature __________ (Month, Day, Year)

Signeture：____________________

The person who provided supplementary explanation

Date of signature __________ (Month, Day, Year)

Signeture：____________________

For investigator

Consent Withdrawal Form

I am participating in the “Phase Ib Clinical Trial to Evaluate the Safety and Efficacy of Durvalumab Tremelimumab in Combination with Heavy Grain Therapy in Patients with Advanced Hepatocellular Carcinoma with Vascular Invasion”.I have read and agreed to the following terms and conditions,However, after reexamination of the information, I hereby withdraw my consent as follows

I withdraw my consent for the storage of the specimen provided in the above study.

Participant

Date of consent to withdrawal：__________ (Month, Day, Year)

Signature:：____________________

The investigator who obtained consent

Date of signature ________ (Month, Day, Year)

Signeture：________________

The person who provided supplementary explanation

Date of signature ________ (Month, Day, Year)

Signeture：________________

For site

Consent Withdrawal Form

I am participating in the “Phase Ib Clinical Trial to Evaluate the Safety and Efficacy of Durvalumab Tremelimumab in Combination with Heavy Grain Therapy in Patients with Advanced Hepatocellular Carcinoma with Vascular Invasion”.I have read and agreed to the following terms and conditions,However, after reexamination of the information, I hereby withdraw my consent as follows

I withdraw my consent for the storage of the specimen provided in the above study.

Participant

Date of consent to withdrawal：＿＿＿＿＿＿ (Month, Day, Year)

Signature:：＿＿＿＿＿＿＿＿＿＿＿＿＿＿

The investigator who obtained consent

Date of signature ＿＿＿＿＿ (Month, Day, Year)

Signeture：＿＿＿＿＿＿＿＿＿＿

The person who provided supplementary explanation

Date of signature ＿＿＿＿＿ (Month, Day, Year)

Signeture：＿＿＿＿＿＿＿＿＿＿

For participant

Consent Withdrawal Form

I am participating in the “Phase Ib Clinical Trial to Evaluate the Safety and Efficacy of Durvalumab Tremelimumab in Combination with Heavy Grain Therapy in Patients with Advanced Hepatocellular Carcinoma with Vascular Invasion”.I have read and agreed to the following terms and conditions,However, after reexamination of the information, I hereby withdraw my consent as follows

I withdraw my consent for the storage of the specimen provided in the above study.

Participant

Date of consent to withdrawal：＿＿＿＿＿＿＿(Month, Day, Year)

Signature:：＿＿＿＿＿＿＿＿＿＿＿＿＿＿＿

The investigator who obtained consent

Date of signature＿＿＿＿＿＿(Month, Day, Year)

Signeture：＿＿＿＿＿＿＿＿＿＿＿

The person who provided supplementary explanation

Date of signature＿＿＿＿＿＿(Month, Day, Year)

Signeture：＿＿＿＿＿＿＿＿＿＿＿

About a phase Ib study of durvalumab (MEDI4736) tremelimumab combined with particle therapy in advanced hepatocellular carcinoma patients with macrovascular invasion （DEPARTURE trial)

― Information and Consent Form―

This booklet describes a phase Ib study of durvalumab (MEDI4736) tremelimumab combined with particle therapy in advanced hepatocellular carcinoma patients with macrovascular invasion （DEPARTURE trial)<br>Please read this information sheet carefully and understand the contents of the clinical trial before making your decision on whether you would like to participate in the clinical trial.<br>If you have questions or concerns about the contents or terms, please feel free to ask the study doctor or clinical research coordinator.

Chiba University Hospital
Principal investigator：Naoya Kato

Date prepared：06 21, 2022
Version number; 2.0

Table of Contents

1. What is a clinical trial？ 1
2. About your disease and treatment 3
3. Objective of the clinical trial 7
4. Method of the clinical trial 8
5. Specimen (blood and tissue) storage 17
6. Duration of participation in the clinical trial and number of participants 18
7. Foreseeable benefits 19
8. Foreseeable disadvantages and side effects 19
9. Free voluntary participation in the clinical trial and the ability to withdraw consent at any time 31
10. Alternative treatment methods if you do not participate in this clinical trial 31
11. Discontinuation after participation in a clinical trial 32
12. Regarding any new important information obtained 32
13. Compensation for health damage related to clinical trials 33
14. Costs during the study 33
15. Access to medical records and preservation of participant confidentiality 34
16. Conflicts of Interest 35
17. Your responsibilities during the study period 35
18. Institutional Review Board that has reviewed this study 36
19. Contact information 37

Please read this information sheet carefully and understand the contents of the clinical trial before making your decision on whether you would like to participate in the clinical trial. You are free to decide whether or not to participate. Also, you do not have to decide on the spot after receiving the explanation. You may decide after discussing the contents of this explanatory document with your family. You will not be disadvantaged in any manner if you decline to participate. You may withdraw from the clinical trial at any time even after you have agreed to participate in the clinical trial or after the clinical trial has started. If you have questions or concerns about the contents or terms, please feel free to ask the study doctor or clinical research coordinator.

1. What is a clinical trial？

In order to investigate the effectiveness and safety of a drug, it is necessary to conduct trials in which healthy people and patients participate, and these trials are called clinical studies. Of these, clinical studies in which results and data are collected in order to have the drug approved as a drug by the government (Ministry of Health, Labor and Welfare) are called "clinical trials. Clinical trials have a research aspect, but they are also important trials to enable many patients to receive new treatments and require the cooperation of patients and the consideration of specialized physicians. The drug used in a clinical trial is called an "investigational new drug," and it is stipulated that the clinical trial should be conducted in compliance with the rules set by the government (GCP). This clinical trial is also conducted in compliance with these rules.

There are several phases in a clinical trial.

Overview of Treatment Development

Basic Research	Scientists screen a vast number of candidate compounds to identify those with the potential to become drugs.
Preclinical Testing	The efficacy and safety of the selected compounds are studied using animal models.
Clinical Trials	The safety and effectiveness of the drug are evaluated in humans.<br>Phase I Trial<br>Conducted with a small group of patients to assess the drug's safety and how it is distributed and processed in the body.<br>Phase Ib Trial<br>Evaluates the combination of a new treatment with existing therapies (this applies in the current study).<br>Phase II Trial<br>Conducted with a limited number of patients to further assess the drug's effectiveness and safety.<br>Phase III Trial<br>Involves a larger group of patients to compare the new drug's efficacy and safety against standard treatments.
Regulatory Review and Approval	Based on preclinical and clinical trial data, the Ministry of Health, Labour and Welfare (MHLW) rigorously evaluates whether the drug is appropriate for use. This process also ensures that the clinical trials were conducted ethically and scientifically. Once approved, the drug can be marketed and made available to patients.

In clinical trials for anticancer drugs, information gathered in three trials (Phase I-III) or two trials (Phase I and II), as described above, is submitted to the Ministry of Health, Labor and Welfare for review.

The clinical trial introduced here is a Phase Ib trial, which is a "investigator-initiated clinical trial" conducted by investigators who are actually involved in the actual medical treatment of the patient and is planned after considering the medical necessity and importance of the drug. This trial is conducted with the permission of the hospital director based on the deliberations of the Chiba University Hospital Clinical Trial Review Committee. Please see p.32 for more information about the committee.

2. About your disease and treatment

As hepatocellular carcinoma progresses, it can develop vascular invasion (tumor invasion of the portal vein and hepatic vein, important blood vessels that pass through the liver) and metastasis to organs other than the liver. This clinical trial is open to patients diagnosed with hepatocellular carcinoma with vascular invasion.

Main medical conditions that occur in patients with hepatocellular carcinoma with vascular invasion (varies from patient to patient)

(1) Fever, loss of appetite, fatigue, etc. due to tumor
(2) Abdominal pain due to the tumor, pain due to the metastatic site, and other symptoms
(3) intrahepatic metastasis
(4) Decrease in liver function due to the tumor
(5) Occurrence of esophageal and gastric varices

Hepatocellular carcinoma with vascular invasion is one of the most rapidly progressing forms of hepatocellular carcinoma and may also rapidly decline in strength, so treatment should be initiated as early as possible.

Currently, anticancer drug therapy (systemic chemotherapy) is the standard of care for patients with hepatocellular carcinoma with vascular invasion. The efficacy of drugs such as atezolizumab bevacizumab combination therapy, sorafenib, lenvatinib, regorafenib, ramucirumab, and

cabozantinib is known, but they are not yet fully effective. Other treatment modalities have similarly failed to show adequate results, and there is a need to develop treatments.

This clinical trial presented here was planned as one of the studies to develop safer and more effective treatment.

Studies have reported the importance of aggressive treatment of the area of vascular invasion itself, such as surgery and radiation. Immune checkpoint inhibitors (ICIs) are being actively developed as anticancer agents, and it is expected that more patients will benefit from treatment by combining ICIs with other therapies rather than ICIs alone.

<u>About the investigational drugs “Durvalumab” and “Tremelimumab</u>

Previous studies have shown that human immune action slows or controls the rate of cancer growth. However, there are cases where the natural immune response does not work and the human immune response does not kill the cancer. Research has shown that some cancer cells and immune cells produce a signal that blocks the cancer-killing action. A new drug in development blocks this signal and amplifies the immune response. These are two new drugs, durvalumab and tremelimumab. These are antibodies (proteins produced by the body's defense system). Durvalumab alone or in combination with tremelimumab may enhance the immune system's ability to detect and fight cancer. The two drugs target different signals. Durvalumab targets a cancer cell signal called PD-L1 (Programmed Cell Death Ligand 1), while tremelimumab targets an immune cell signal called CTLA-4 (Cytotoxic T-Lymphocyte-associated Antigen 4) CTLA-4 (Cytotoxic T-Lymphocyte-associated Antigen 4). By blocking these signals, it is hoped that immune cells can again control or slow the growth rate of cancer.

Durvalumab is approved in Japan under the brand name Imfinzi® for the treatment of unresectable locally advanced non-small cell lung cancer, while tremelimumab is not yet approved in Japan. Clinical trials are underway in lung cancer, bladder cancer, head and neck cancer, and other types of cancer. Outside of Japan, it is approved by the U.S. Food and Drug Administration (FDA) for the treatment of patients with locally advanced or metastatic urothelial carcinoma whose cancer has progressed during or after platinum-based chemotherapy. It is also approved by the U.S. FDA and the European Medicines Agency (EMA) in Europe for the treatment of patients with locally advanced non-small cell lung cancer who have received chemoradiation therapy. However, durvalumab is not approved in combination with tremelimumab (either in Japan or overseas) for the treatment of cancer patients.

Although neither durvalumab nor tremelimumab has been approved for the treatment of hepatocellular carcinoma, studies to date have confirmed that there are no problems with its safety and studies are underway to confirm its efficacy. If you have any questions about the investigational drug, please ask your physician at any time.

Durvalumab

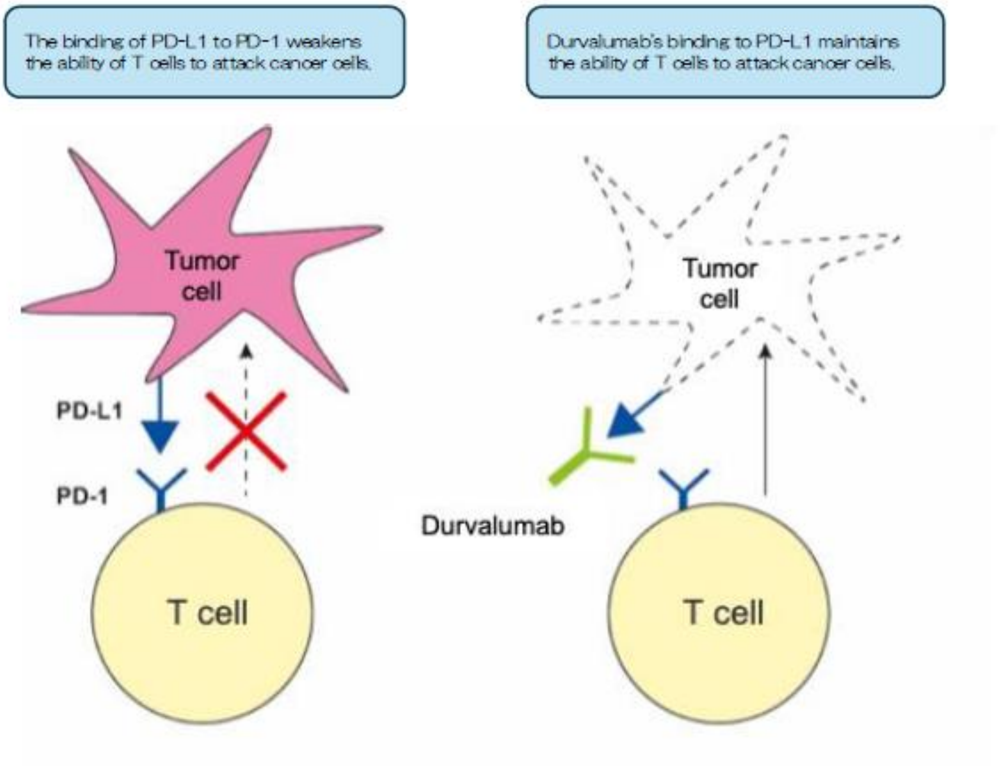

Tremelimumab

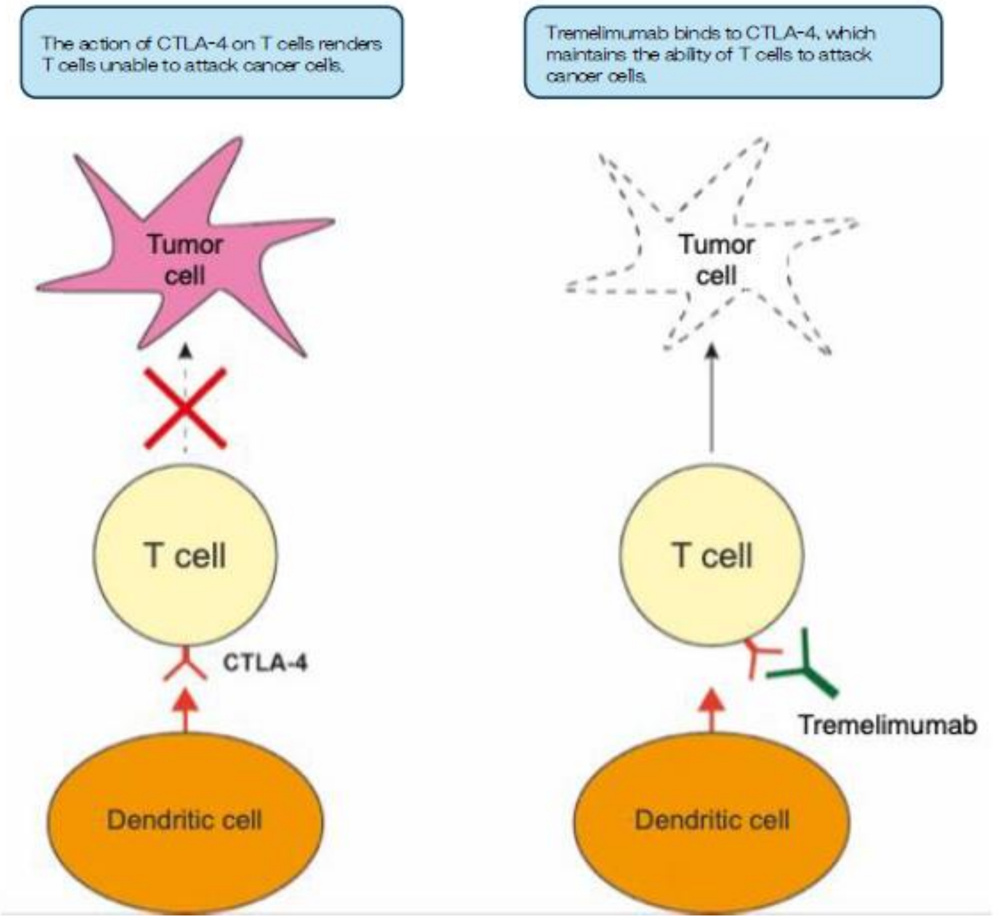

<u>About Carbon Ion Radio Therapy</u>

Carbon ion radio therapy is one of the radiation therapy methods for hepatocellular carcinoma. It is a new-generation treatment method classified as particle beam therapy along with proton beam therapy.

In contrast to conventional radiotherapy using X-rays, it can deliver high doses of radiation to the affected area while greatly minimizing irradiation to the normal liver. Studies of heavy-ion radiation therapy alone for hepatocellular carcinoma have reported that it is an effective treatment with minimal side effects.

Currently, hepatocellular carcinoma of 4 cm or larger is treated by medical insurance system in Japan. For patients with hepatocellular carcinoma smaller than 4 cm, research is being conducted under the advanced medical treatment system, in which the beneficiary bears the cost of the main treatment.

The participants in this study are scheduled to receive heavy particle therapy at the Hospital of the National Institute of Quantum Science and Technology.

3. Objective of the clinical trial

In this clinical trial, patients with advanced hepatocellular carcinoma will receive an infusion of durvalumab (or both durvalumab and tremelimumab) followed by treatment with heavy ion radiation. The main purpose of the study is to see what side effects may occur during this process and to determine whether this treatment can be safely administered.

The first 3-6 patients in the trial will receive durvalumab only, followed by heavy particle radiation. We will first review the physical condition of those

who receive this treatment to make sure that the treatment can be safely administered.

Patients who participate after the review will receive both durvalumab and tremelimumab infusions and heavy particle therapy, which will also be evaluated again with respect to safety.

Based on the results of both treatments, we also aim to determine which treatment regimen is considered appropriate.

4. Method of the clinical trial

If you agree to participate in this clinical trial, you will first undergo a screening test. There are three periods in this clinical trial: the screening period, the treatment period, and the follow-up period after discontinuation of the clinical trial treatment.

During the screening period, you will undergo several tests and medical examinations to confirm that you are eligible to participate in this clinical trial treatment. Your investigator and the clinical trial staff will explain the clinical trial to you. The screening period is 28 days.

The treatment period is the time during which you will receive the study drug and heavy particle irradiation.

During the follow-up period, we will check your physical condition and blood test values after treatment is discontinued.

1）Eligibility

The conditions under which a patient may or may not participate in this clinical trial are as follows

≪ Patients who can participate in this study ≫

1）Patients diagnosed with hepatocellular carcinoma with vascular invasion

2) Patients with hepatocellular carcinoma not amenable to local therapies such as radiofrequency ablation (RFA) and trans arterial chemoembolization (TACE)

3) Patients must be 20 years of age or older

4) Weigh 30 kg or more

5) Good general condition and no major obstacles in daily life

6) Your test values (neutrophils, platelets, hemoglobin, liver function, renal function, etc.) meet certain criteria

7) Your consent to participate in this clinical trial has been obtained

≪ Patients who cannot participate in this study ≫

1) Brain metastasis

2) Currently suffering from cancer of an organ other than the liver, or suffered from cancer within 3 years

3) Received treatment with immunosuppressive agents within 14 days (28 days for antibody drugs) prior to the start of treatment with the investigational drug

4) Currently or previously had an autoimmune or inflammatory disease (unless the patient is determined to have had no active inflammatory disease within the past 5 years)

5) Have any of the following conditions or diseases

- Has serious heart disease (heart failure, myocardial infarction, angina pectoris, arrhythmia requiring treatment, etc.)
- Electrocardiogram (ECG) abnormalities
- Infectious disease requiring treatment with oral or injectable medications

- Severe respiratory illness (interstitial pneumonia or pulmonary fibrosis)

- Severe mental disorders (e.g., dementia)

- Positive test result for human immunodeficiency virus (HIV)

- Current or former hepatic encephalopathy

- Had cerebrovascular disease, thrombosis, or thromboembolism within 180 days prior to initiation of investigational therapy

6) Previous radiation therapy with liver effects.

7) Pregnant or lactating women.

8) Unable to consent to proper method of contraception during participation in the study and for 180 days from the date of the last dose of study drug (both sexes)

There are many other criteria, and we will make a judgment based on our detailed examination and consultation. Therefore, please understand that you may not be able to participate in the clinical trial even after you have given your consent, and even after the clinical trial has started, we may decide to terminate your treatment or participation in the clinical trial at our discretion. Patients who participate in this trial will be assigned to one of two treatment groups: durvalumab alone or a combination of durvalumab and tremelimumab as the drug to be combined with carbon ion radio therapy. In either group, you will be admitted to the hospital to start receiving durvalumab and tremelimumab, and you will also receive carbon ion radio therapy at QST Hospital. After completion of carbon ion radio therapy, patients will be discharged after confirming that they are in good physical condition, but the length of hospital stay will not change regardless of which group they are in. The length of hospitalization will be the same for both groups. Although the length of hospitalization may be longer depending on

your physical condition, the expected length of hospitalization is approximately 3 weeks. After discharge from the hospital, patients will be followed up with outpatient visits.

The flow of this clinical trial is shown below.

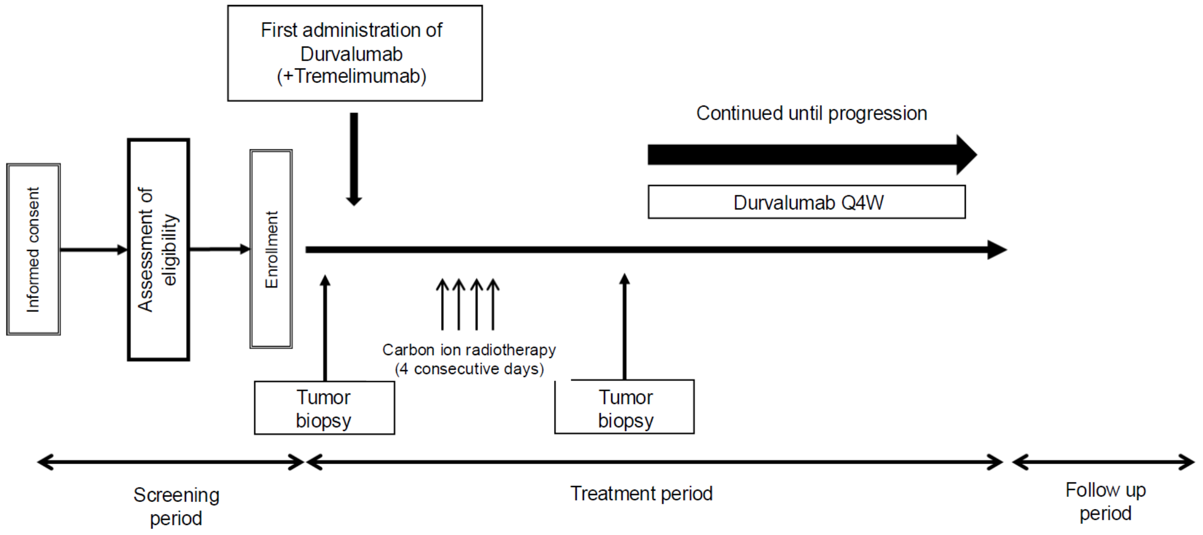

2) Detailed methods

The major objective of this study is to confirm that the combination of the investigational drug and carbon-ion radiation is safe for the human body. This confirmation of safety is called a Dose Limiting Toxicity (DLT) evaluation.

The first step in this evaluation is to assess whether the treatment is safe in two phases, with three to six patients receiving durvalumab followed by carbon ion radio therapy. This evaluation period will last 42 days from the start of treatment; if all three of the three patients, or at least five of the six patients, are found to be safe and there is no need to discontinue the trial, the next step, durvalumab and tremelimumab with carbon ion radio therapy will be given to an additional 3 to 6 patients. The same DLT evaluation will be performed on this group of patients. We plan to proceed with the clinical trial

while confirming safety, and we expect a total of 15 patients to participate in the trial at our institution.

Depending on the timing of your participation in the clinical trial as described above, you will either receive durvalumab and carbon ion radio therapy or durvalumab plus tremelimumab and carbon ion radio therapy. Your investigator or clinical trial coordinator will explain this to you each time.

As a general rule, to ensure your safety, you will be hospitalized from the time you receive your first dose of study drug until the time you receive carbon ion radio therapy. Specific administration methods and other details will be explained next.

① Dosing schedule

In this trial, 28 days are counted as one cycle.

Durvalumab (or both durvalumab and tremelimumab) will be administered intravenously on Day 1 of Cycle 1. The infusion treatment takes about 1.5 hours when only durvalumab is given, and about 3 hours when both durvalumab and tremelimumab are given.

Carbon ion radio therapy is then administered over the next 8 days (4 days total).

From Cycle 2 onward, durvalumab will be administered intravenously once every 4 weeks (on Day 1 of each cycle) after confirming that it can be administered safely according to the “Guidelines for the Management of Toxicity in Investigational New Drugs”.

Your physical condition will be monitored especially carefully during the first 42 days after the first dose. Durvalumab will be continued and treatment will be continued while monitoring side effects and changes in your condition.

②Carbon ion radio therapy (and targeted marker insertion)

In this treatment, carbon-ion radiation must be focused on the lesion as precisely as possible. Otherwise, normal tissue, not cancer cells, will be damaged. During the actual irradiation, you will lie on a treatment bed in a special treatment room, and you will be placed in a fixture to hold your body in place, and you will be asked to cooperate by not moving your body for a while. If you experience pain because the fixture does not fit properly, do not be patient and let us know. Carbon-ion radiation is given for a few minutes, but you will be in the treatment room for a total of 20 to 30 minutes, including the time needed for preparation and treatment before and after the irradiation. During the irradiation, you will be alone in the treatment room, but you do not need to worry because we will be watching you from outside the room via a TV camera, and if necessary, you can press the emergency buzzer to stop the irradiation and we will come to your room. You will not feel any pain or heat from the carbon-ion radiation during the irradiation.

Carbon ion radio therapy will be given once a day, for a total dose of 60 Gy (RBE). This therapy will be completed in one week.

In this trial, a "target marker" is implanted in advance to accurately identify the lesion site during treatment. When the target marker is inserted from the surface of the body, local anesthesia is administered to the skin and subcutaneous tissue of the abdomen, a thin needle is inserted, and a tube is inserted into the liver. When inserted through a blood vessel in the liver, the target marker is implanted through a thin tube from an artery at the base of the groin to a site near the disease. The implanted target marker is not removed after treatment and remains in the organ, but there have been no reports of the marker directly harming the patient's health. When inserted from the surface of the body, a needle is inserted into the liver, which may

cause bleeding, organ damage, and other side effects similar to liver biopsy (details are described in the explanation section of liver biopsy on p. 23). When inserting a catheter through a blood vessel in the liver, a method called abdominal angiography is applied. A thin tube called a catheter is inserted from a blood vessel in the leg or arm, and the catheter is advanced to a blood vessel near the tumor in the liver. A targeted marker will then be implanted in a blood vessel near the tumor. Side effects associated with abdominal angiography (such as allergic reactions to contrast media, renal dysfunction, vascular injury, bleeding, infection, and organ damage) may occur.

3）Details and schedule of tests during the clinical trial

After you agree to participate in a clinical trial and sign the Clinical Trial Consent Form, screening tests will be performed to determine if you are suitable to participate in the trial. If the results of these tests can be substituted for the tests that were performed prior to your consent, we may use the results of those tests as data for the clinical trial. However, please understand that depending on the results of the tests, you may not be able to participate in the clinical trial. During the clinical trial period, including the screening tests, you will be taken for periodic medical examinations and consultations in accordance with the schedule shown in Table 1. In addition, after the administration of the investigational drug has been completed, we will also conduct medical examinations and consultations.

(A) Schedule of medical examinations and tests.

Investigations include measurement tests by blood and urine collection, electrocardiogram, CT and MRI, and follow-up by biopsy to collect tissue

(liver tumor and non-tumor tissue). These tests are commonly performed to treat your disease and ensure your safety and are necessary for this clinical trial. If the results of the tests are deemed necessary by your physician, your visits and tests may be more frequent than those planned in Table 1.

(B) Tumor marker

There are many types of cancer, some of which produce substances characteristic of each cancer. Among such substances, those that can be measured in body fluids (mainly blood) are called tumor markers.

are called tumor markers.

Together with blood tests and imaging tests, tumor markers are used as one of the tests to check the progress of cancer and the progress of treatment.

In this case, blood will be used to measure AFP, AFP-L3, and PIVKA2, which are tumor markers for hepatocellular carcinoma. Blood samples will be drawn prior to the start of the study and prior to the administration on the first day of each of Cycle 2 and thereafter.

(C) Liver biopsy and liver tumor biopsy

Liver biopsy and liver tumor biopsy (tissue collection from non-tumor and tumor areas) will be performed on patients who have agreed to undergo liver biopsy and liver tumor biopsy. The timing of the liver biopsy/liver tumor biopsy will be before treatment and after the safety of the treatment has been confirmed for 42 days after the start of treatment. Liver tissue (non-tumor and tumor areas) obtained by biopsy will be used for exploratory studies.

Table 1: Schedule of medical examinations and tests

Cycle	Screening period	First tumor biopsy	DLT evaluation period							Second tumor biopsy	Durvalumab q4W dosing period					ST	Follow up period	Safety information collection
			Cycle 1				Cycle 2				Cycle 3	Cycle4	Cycle 5	Cycle 6	Cycle 7 and beyond			
Cycle Day	D -28<br>～D -1	D -28〜D -1	1	8 to 14	15	22	1	8	14		1	1	1	1	1	—	—	—
Allowable period（Day）			—	±3	±3	±3	±3	±3	±3		—	±3	±3	±3	±3	+14	+14	+14
Informed Consent ／ Subject backgroud information／<br>Review of eligibility	●																	
Durvalumab administration<br>(cohort A and B)			●				●				●	●	●	●	●			
Tremelimumab administration<br>(cohort B)			●															
Tumor biopsy		●								●								
CIRT（QST hospital）				4 times between Day8 and Day14														
Fiducial marker insertion		●																
Fixation, simulation CT (for CIRT)	●																	
Weight	●		●	●	●	●	●	●	●		●	●	●	●	●	●	●	
Height	●																	
Clinical Chemistry／Hematology	●		●	●	●	●	●	●	●		●	●	●	●	●	●	●	
Urinalysis	●		●								●	●	●	●	●	●	●	
ECG	●		●								(●)	(●)	(●)	(●)	(●)	(●)	(●)	
Chest X ray	●															●	●	
CT/MRI	●		←Every 6 weeks (±1 week) for the first 12 weeks from Cycle1 day1, and every 8 weeks (±1 week) thereafter until PD.→														●	
Tumor marker（AFP、PIVKA-II）	●						●				●	●	●	●	●			
Assessment of AE/SAE	←→																	

Table 2: Laboratory Tests for Blood and Urinalysis

Hematological Tests	Red blood cell count, Hemoglobin, Hematocrit, Platelet count, White blood cell count, White blood cell fraction (Neutrophils, Lymphocytes, Monocytes, Eosinophils, Basophils)
Biochemical examination of blood	Total protein, Albumin, Total bilirubin, AST, ALT, ALP, γ-GTP, LDH, BUN, Amylase, Lipase, Creatinine, Uric acid, Na, K, Cl, Ca, P, Mg, Blood sugar
Blood coagulation test	PT-INR、APTT
Urinalysis	Specific gravity, pH, Sugar, Protein, Ketones, Occult blood, Bilirubin, Color and appearance, Urinary sediment

5. Specimen (blood and tissue) storage

In principle, the specimens you provide for testing (blood and liver tissue (tumor and non-tumor parts)) will be stored until the end of the clinical trial.

If you give your consent, we plan to keep the specimens remaining after testing for a certain period of time (up to 20 years from the start of the clinical trial) at the Department of Gastroenterology of our hospital even after the trial has ended. This is because there is a possibility that additional research on hepatocellular carcinoma may be conducted in the future based on newly obtained findings. If used for research, it will be used only after approval by the Review Committee. When storing the specimens, we will anonymize them so that it will not be known that the specimens were collected from you.

You may participate in a clinical trial even if you do not consent to the storage of specimens after the trial is completed. You will not be disadvantaged by not consenting. Please make your decision based on your

own judgment, as there is a confirmation box on the clinical trial participation consent form.

You can withdraw your consent to the storage of specimens after the completion of the clinical trial at any time. If you withdraw, you will not be disadvantaged in any way. If you wish to withdraw your consent, please sign the Consent Withdrawal Form and submit it to your investigator or study coordinator.

However, if the person in charge at the hospital has already collected the analysis results before you submit the withdrawal of consent form, the results will not be used as they are.

If the results of the analysis have been collected by the person in charge at the hospital before you submit the withdrawal of consent, the results may be used as they are and shared with other parties.

If it is determined that there is no further need to store specimens before 20 years have passed from the start of the clinical trial, the specimens may be discarded without waiting 20 years from the start of the clinical trial.

In addition, since the results obtained are still in the research phase, they will not be disclosed to you in principle. If you wish to request disclosure, please contact us.

6. Duration of participation in the clinical trial and number of participants

Approximately 15 patients are expected to participate. The expected duration of participation in the clinical trial will be the total of the screening period (28 days) and the treatment and post-treatment follow-up periods (28

days after completion of treatment). The treatment period will be the sum of 42 days after the first dose and the period of continued durvalumab administration.

The duration of durvalumab treatment will depend on your condition and will continue as long as your investigator determines that it is effective for your disease. However, it may be discontinued if serious side effects occur and it becomes difficult to continue treatment, even if it is effective.

7. Foreseeable benefits

The combination of durvalumab tremelimumab with carbon ion radio therapy may demonstrate efficacy not seen with existing therapies.

Results of an AstraZeneca-led study of durvalumab-tremelimumab in advanced hepatocellular carcinoma have shown that each agent is safe as a single agent and that the combination of both agents has promising results. carbon ion radio therapy for hepatocellular carcinoma has also been shown to be safe and effective and hepatocellular carcinoma of 4 cm or larger is treated by medical insurance system in Japan.

However, the safety and efficacy of durvalumab-tremelimumab in combination with carbon ion radio therapy is not known. Information obtained from this clinical trial may be used in future research on the treatment of this disease.

8. Foreseeable disadvantages and side effects

The side effects that may develop as a result of treatment vary greatly from person to person, and what symptoms may occur and their severity vary from person to person, and cannot be completely predicted before treatment begins. When side effects occur, treatment may be temporarily stopped, or treatment may be given to alleviate symptoms.

Serious side effects may also occur when treatment is resumed after a suspension or postponement of treatment.

If you feel that your condition is unusual, please contact your investigator for appropriate treatment.

Risks associated with durvalumab and tremelimumab.

Most of the side effects seen with durvalumab and tremelimumab were mild or moderate. However, some are serious, life-threatening and sometimes fatal. Some side effects do not require treatment, but symptoms usually recover with treatment. It may be necessary to delay the administration of durvalumab and tremelimumab to improve the symptoms of side effects. The most important side effects that may occur are listed below. These can occur as a result of the action of durvalumab and tremelimumab on the immune system and have been seen in patients who received either or both durvalumab and tremelimumab in previous studies. These side effects have also been seen in trials using other medicines similar to durvalumab and tremelimumab. The management of these side effects may require the administration of steroids or other medications that can work on the immune system and reduce inflammation.

The types of side effects were very similar when these two investigational drugs were given together and when durvalumab was given alone. However, the probability and severity of many, but not all, of these side effects were higher when they were given in combination than when durvalumab was given alone.

Very common side effects (>10%)

・ Diarrhea

・Rash / Dry and itchy skin

・ Liver dysfunction: Blood levels of substances called enzymes, which are found in liver cells, may increase. Changes in the enzyme do not often make you feel sick. However, if this enzyme level is very high, your investigator may need to discontinue the study drug. It may also cause inflammation of the liver, known as hepatitis, but this is rare. It may be accompanied by signs and symptoms such as yellowing of the skin and white eye area, dark urine, severe nausea and vomiting, pain in the upper right abdomen, itchy skin, inability to feel hunger, and bleeding or bruising more easily than normal.

In addition to the above, important anticipated side effects reported to be very common (i.e., >10%) in clinical trials in which patients with different types of cancer were treated with durvalumab alone or in combination with tremelimumab were fatigue, abdominal pain, swelling due to fluid retention, upper respiratory tract infection, nausea, vomiting, decreased appetite, shortness of breath, cough, fever, and muscle and joint pain.

Most common side effects (≥1% to <10%)

・ Hypothyroidism: This is caused by a decrease in the amount of thyroid hormones produced by the thyroid gland and a very slow metabolism. Symptoms include, but are not limited to, fatigue, feeling cold more easily, constipation, dry skin, unexplained weight gain, facial swelling, muscle weakness, decreased heart rate, thinning hair, and memory problems. These symptoms can be improved by thyroid hormone replacement. This eventis a very common side effect in patients receiving the combination of durvalumab and tremelimumab, but is classified as a common side effect in patients receiving durvalumab alone.

・ Pneumonia: Symptoms include, but are not limited to, new or worsening cough, shortness of breath (sometimes accompanied by fever). Pneumonia can be fatal. Limited data (not yet fully established) suggest that the incidence and severity of the disease may be higher in Japanese than in non-Japanese. If you have any of these symptoms, contact your investigator immediately.

・ Hyperthyroidism: This condition occurs when the thyroid gland produces too much thyroid hormone. Symptoms include anxiety, nervousness, weight loss, frequent bowel movements, diarrhea, shortness of breath, hot flashes, and heart palpitations. Depending on the severity of the symptoms, treatment may include observation only, symptomatic treatment, or treatment to stop the secretion of thyroid hormones.

・ Renal dysfunction: Even without symptoms or feeling sick, blood tests may show increased creatinine levels (creatinine is a protein marker that assesses kidney function). Less frequently, nephritis may occur, in which the kidneys become inflamed and lose normal function.

・Nervous system disorders: Symptoms include abnormal weakness of the leg, arm, or facial muscles, or numbness or tingling in the limbs. Rarely, severe inflammation of the nervous system may occur, which can damage nerve cells and interfere with communication between nerves and muscles. If you experience trouble swallowing, sudden weakness, or difficulty breathing, contact your doctor immediately.

・ Injection reactions: Reactions may occur during or after injection of the investigational drug. Injection reactions may cause fever or chills, changes in blood pressure, or significant dyspnea. Contact your investigator immediately if you experience any of these symptoms, even if it is several days after the injection.

・Intestinal inflammation (colitis): May cause abdominal pain and diarrhea (with or without bleeding). May be accompanied by fever. Additional intravenous fluids may be needed. If left untreated, it can cause serious and life-threatening tears in the intestinal wall. If you experience any of these symptoms, contact your doctor immediately.

・ Elevated levels of pancreatic enzymes (amylase and lipase). These enzymes are indicators of pancreatic function. In rare cases, elevated levels of these enzymes may be associated with pancreatitis (see "Rare Side Effects" below).

In addition to the above, the most common adverse reactions reported as serious (i.e., ≥1% to <10%) in clinical trials in which durvalumab was administered alone or in combination with tremelimumab to patients with various types of cancer are pneumonia, hoarse voice, urinary pain, night sweats, oral candidiasis, and muscle and joint pain.

Common side effect (≥1% to <10%) (occurs in 1 out of 10-100 patients) / Rare side effect (<1%) (occurs in 1 out of 100-1000 patients)

・ Adrenal Injury: May cause stomach pain, vomiting, muscle weakness, fatigue, mood swings, hypotension, weight loss, kidney problems, and mood and personality changes. It has been reported to occur more frequently in patients receiving the combination of durvalumab and tremelimumab, but less frequently in patients receiving durvalumab as a single agent. These complications may require permanent hormone replacement therapy.

・ Dental and oral soft tissue infections and influenza were more common in patients who received durvalumab as a single agent and rare in those who received it in combination with tremelimumab.

Rare (<1%) (occurs in 1 out of 100 to 1000 patients)

・ Pancreatitis: Pancreatitis usually presents with persistent pain in the upper abdomen (which may be made worse by eating or drinking), nausea, vomiting and weakness. Pancreatitis usually improves with simple treatment but can be severe and life-threatening. Contact your investigator immediately if you have any of these symptoms.

・ Allergic reaction: An allergic reaction may cause swelling of the face, lips, or throat, or breathing difficulties accompanied by hives or a hives-like rash. If any of these symptoms occur, contact your investigator immediately.

Rare (<1%) (occurs in 1 out of 100-1000 patients) / Very Rare (<0.1%) (occurs in 1 out of 1000-10000 patients)

・Myositis/Polymyositis: Symptoms include muscle weakness, myalgia, fatigue while standing or walking, and muscle pain lasting several weeks. It was rare in patients treated with durvalumab as a single agent and very rare in patients treated with tremelimumab in combination.

・Pituitary disorder (hypopituitarism): Hypopituitarism is a decrease in the hormone secreted by the pituitary gland in the brain and is caused by inflammation of the pituitary gland (hypopituitarism). Symptoms include headache, thirst, difficulty seeing or double vision, leakage of breast milk in women, or irregular menstruation. These complications may require permanent hormone replacement therapy. They were rare in patients who received durvalumab in combination with tremelimumab and very rare in patients who received durvalumab as a single agent.

・ Inflammation of the heart muscle (myocarditis): symptoms include chest pain, tachycardia, irregular heartbeat, shortness of breath, and swelling of the legs. If you have any of these symptoms, contact your physician immediately. This side effect is classified as an uncommon side effect in patients receiving the combination of durvalumab and tremelimumab, but very rare in patients receiving durvalumab alone.

Very Rare Adverse Reactions (<0.1%) (occurs in 1 out of 1000-10000 patients)

・Type 1 diabetes (may cause elevated blood glucose levels, known as hyperglycemia): Symptoms include weight loss, increased urination, increased thirst and hunger. type 1 diabetes requires insulin replacement by injection. If you have any of these symptoms, contact your doctor immediately.

In addition to the above, important predicted disadvantages reported as rare (i.e., less than 0.1% of patients) in clinical trials of single-agent durvalumab in patients with different types of cancer are inflammation of the membranes surrounding the heart, increased number of small clusters of inflammatory cells in various parts of the body, inflammation of the middle layer of the eye or Other eye abnormalities (e.g., inflammation of the cornea and optic nerve), inflammation of the brain or membranes lining the brain and spinal cord, hardening or straining of skin and connective tissue and loss of skin color, hematologic abnormalities (e.g., abnormal red blood cell destruction, thrombocytopenia), vascular inflammation, and rheumatic abnormalities (muscle pain and stiffness caused by inflammatory disease and autoimmune autoimmune arthritis).

In addition to these expected disadvantages when durvalumab is administered alone or in combination with durvalumab and tremelimumab, other immune-mediated side effects not previously observed may occur, and inflammatory side effects may occur in any organ or tissue.

<u>Side effects of carbon ion radio therapy</u>

１）Gastrointestinal tract

In the case that the cancer is located close to the liver surface and in close proximity to the gastrointestinal tract, such as the stomach, duodenum, or large intestine, carbon ion radio therapy may cause damage to these digestive tracts. Anorexia, nausea, diarrhea, and abdominal pain are the most common symptoms, but it is important to note that anemia due to bleeding may be detected without obvious symptoms. The symptoms range from mild erosions and ulcers that can be treated with medication and diet to severe cases that require hospitalization, such as bleeding, perforation (hole), stenosis (narrowing, making it difficult for objects to pass through), obstruction (blockage of the lumen, making it impossible to pass objects through), and adhesions. Based on the results of clinical trials on cancers in other parts of the body, we can predict to some extent the safe dose when irradiated to the gastrointestinal tract, and we will make sure that the dose irradiated to the gastrointestinal tract is less than the dose considered safe to avoid serious complications. To date, a very small number of patients have experienced mild side effects (less than 1%) that improve with medication, but there have been no serious side effects that would require invasive treatment such as surgery.

２）Skin

Various degrees of radiation dermatitis may occur as a result of carbon ion radio therapy. In most cases, the symptoms are similar to a mild sunburn (redness, itching, mild pain, etc.), but in some cases, the surface of the skin may peel, producing exudates, hyperpigmentation, and small scars (scars). If the lesion is located relatively close to the skin, the skin is also exposed to high doses of radiation, which may cause erythema, erosions, ulcers, atrophy, etc., which may require treatment with ointments. Surgical procedures such as skin grafts may be necessary, although rarely (0-2.8% in past reports), and are becoming less common due to improvements in treatment techniques.

３）Liver

Liver function in the area exposed to carbon-ion radiation will be reduced or eliminated, and overall liver function may also be reduced. In this study, treatment is designed with the expectation that sufficient liver function will remain, but it is known that radiation-induced liver injury (RILD) will occur in 1-2% of patients.

４）Bile duct

Carbon-ion radiation therapy may cause cholangitis, but there have been no cases of cholangitis directly attributable to this treatment. Although rare (less than 1% of cases), bile duct stricture may require medical treatment.

5）Lung

If a portion of the lung is irradiated with carbon-ion radiation, imaging changes, pleural effusion, coughing, fever, and dull pain may occur. In severe cases, sputum, blood sputum, shortness of breath, interstitial pneumonia-like shadows, pulmonary fibrosis-like shadows, and respiratory failure are possible, but since only a small portion of the lung is usually irradiated in the treatment of hepatocellular carcinoma, no serious side effects that could be caused by carbon ion radio therapy have been observed.

6）Blood

Decreases in peripheral blood white blood cell count, red blood cell count, and platelet count, and decreases in hemoglobin concentration may occur.

7）Possibility of secondary carcinogenesis

In the case of conventional radiotherapy, there is a rare possibility of new cancer development (secondary cancer) caused by radiation after a long period of time, and the same is possible with carbon ion radio therapy.

Other risks anticipated from participation in this clinical trial include

Blood sampling: Risks associated with blood sampling include temporary discomfort from needle puncture, internal bleeding, hemorrhage, and in rare instances, infection and anemia may be caused.

ECG: Risks associated with ECG testing include temporary discomfort such as itching, mild irritation, or redness of the skin where the small adhesive pad is applied. If it is necessary to shave the area where this small adhesive pad is applied, the shaving may cause irritation.

CT or MRI: You may experience some discomfort or anxiety when lying down inside the CT scan machine. The injection of contrast may cause a metallic taste in the mouth, a feeling of warmth, and in rare cases, nausea or vomiting. A reaction to the contrast agent may also occur. MRI is safe for most people. People with metal implants near vital organs cannot undergo MRI. This is because the metal can be drawn from the body to a large magnet, which can cause damage.

Biopsy (collection of liver tumor and non-tumor tissue): A biopsy is the collection of a piece of your tissue using a scalpel or needle. This time, the needle will be inserted directly into your liver, which may cause mild discomfort and internal bleeding at the site of the needle puncture. Bleeding, liver dysfunction, jaundice, infection, pneumothorax, and peritoneal seeding may also occur. The frequency of percutaneous puncture of the liver in routine examinations is about 2%. Although rare, surgery or other procedures and blood transfusions may be required for the above treatments. In addition, anesthetics and sedatives used prior to administration may cause a sudden drop in blood pressure or an irregular heartbeat, which may strain the heart. Side effects such as allergic reactions and fever may also occur.

If you become pregnant during a clinical trial, or if your partner becomes pregnant, please notify your investigator or the study coordinator immediately. If you become pregnant, your participation in the clinical trial will be terminated.

If you or your partner becomes pregnant, we will ask you for this information. If you or your partner gave birth, had a miscarriage or an abortion, please tell your investigator. If you or your partner gave birth, you may be

asked to provide information including the baby's date of birth, height and weight at birth, sex of the baby, any complications during pregnancy or delivery, and any birth defects of the baby.

9. Free voluntary participation in the clinical trial and the ability to withdraw consent at any time

Participation in this clinical trial is not mandatory, so please make your own decision.

You may withdraw from the study at any time after participating in the study, regardless of the reason, if you wish to discontinue the study or if it is difficult to continue, so please consult your physician. You will not be disadvantaged by the discontinuation of the clinical trial. After the discontinuation of a clinical trial, you will be informed of the best possible treatment by your physician.

Your doctor will then explain the best treatment options to you.

10. Alternative treatment methods if you do not participate in this clinical trial

If you do not participate in this clinical trial, please ask your physician to explain in detail the other treatment options that may be available to you. You will not be disadvantaged by not participating in this clinical trial. Your physician will discuss with you the best treatment for your condition. Options include participating in a clinical trial for another drug, treatment with an already approved drug, or radiation therapy. Supportive care (pain and suffering control) is also available.

11. Discontinuation after participation in a clinical trial

Even after you have given your consent to participate in a clinical trial, we may discontinue the clinical trial treatment for the following reasons

① When you request discontinuation.
② In case of strong side effects during the clinical trial
③ When cancer has become large
④ When it is deemed difficult to continue the clinical trial due to circumstances at the hospital where the clinical trial is being conducted.
⑤ Other cases in which the investigator determines that discontinuation of the clinical trial is necessary.

28 days after the end of treatment, an examination will be conducted to confirm safety. Even if the clinical trial is terminated due to side effects, we ask for your cooperation as we may conduct tests and medical examinations until the side effects are no longer present.

Even if the clinical trial is terminated during the course of the study, we would like to use the records up to that point, as they will be valuable materials for future cancer research. If you have any concerns about the use of your records, please contact us.

12. Regarding any new important information obtained

We will promptly inform you if we obtain new information on important efficacy, safety, or other information that we believe may affect your decision to continue participating in this clinical trial. In that case, you will be asked to make a new decision as to whether or not you wish to continue participating in the clinical trial.

13. Compensation for health damage related to clinical trials

If you experience any problems related to the investigational drug, or if you experience any health problems as a result of participating in a clinical trial, you should notify your investigator immediately. The physician will treat you for any health problems that arise as a result of your participation in a clinical trial, and you may be eligible for compensation. However, compensation may not be provided in the following cases

① When there is no causal relationship between the health hazard and the clinical trial

② In case of progression of hepatocellular carcinoma due to inadequate efficacy of the investigational drug

③ If the health damage was caused by your intentional or gross negligence

For more information, please consult your investigator or consultation service.

14. Costs during the study

If you participate in this clinical trial, the investigational drug will be provided by the investigational drug provider (AstraZeneca Corporation). In addition, the hospital will cover the costs associated with heavy particle irradiation. We will also cover the cost of transportation to and from the QST Hospital for carbon ion radio therapy while you are hospitalized at Chiba University Hospital. The costs of medical examination fees, tests, drugs normally used, drugs used for side effects, tests and diagnostic imaging, etc. will be borne by the patient according to the type of health insurance as in the past. You will also be responsible for the cost of hospitalization for this clinical trial treatment.

15. Access to medical records and preservation of participant confidentiality

Any personally identifiable information that may be used to identify an individual who has cooperated in a clinical trial will be strictly protected and will not be released to outside parties.Data obtained from patients will be anonymized by code numbers, etc., and will be handled in such a way that they will not be identified as belonging to that patient in reports, etc.

The results of this clinical trial will be submitted to the Ministry of Health, Labour and Welfare (MHLW) in order to have the government (MHLW) approve the combination therapy of durvalumab tremelimumab and heavy ion therapy as a treatment for advanced hepatocellular carcinoma with vascular invasion. In addition, in order to study the efficacy and safety of this investigational drug in more detail, data necessary to evaluate the investigational drug, such as tests, images, and electrocardiograms used as data in the clinical trial, may be submitted to an outside organization.

Furthermore, information obtained from this clinical trial may be compiled from records collected from hospitals in various regions and published in academic societies and medical journals. In all cases, however, your name will not be used and your personal information (name, address, telephone number, etc.) will be kept confidential and will not be leaked to outside parties.

In addition, in order to check whether the clinical trial is being conducted properly, personnel from the investigational drug development organization, the Clinical Trial Review Committee of this hospital, and regulatory authorities such as the Ministry of Health, Labor and Welfare may access your medical records, including your medical records from other departments and the period before your participation in the clinical trial. Even in such cases, these officials are obligated to maintain confidentiality and your privacy will be protected.

Even if you have received treatment at another medical institution, we may contact your physician at the other institution by phone or letter to request

medical information. By signing the consent form at the end of this letter, you are also giving your consent to access your records and to the collection of information from other medical institutions.

You may withdraw your consent to the use of your medical information at any time, but please note that you will not be able to participate in this clinical trial after that. If you wish to withdraw your consent, please inform your doctor or hospital staff in charge. The clinical trial data collected before you revoke your consent will be used in the same way as described above, but after you revoke your consent, your medical information will not be used except to confirm that this clinical trial has been conducted properly.

16. Conflicts of Interest

This study is being funded and conducted by AstraZeneca Inc. At the same time, we are receiving the investigational drug and information on the safety of the drug, but there is no profit from the conduct of this study itself or from the analysis and reporting of the results. This has been reviewed by our Conflict-of-interest Management Committee, which has confirmed that no conflicts of interest* (possible conflicts of interest) exist with regard to the implementation of this clinical trial.
*Conflicts of interest are defined as actions that may compromise patient safety or distort the interpretation of data for the benefit of oneself or a pharmaceutical company, for example, when receiving funding or other benefits from a pharmaceutical company.

17. Your responsibilities during the study period

If you agree to participate in this clinical trial, please observe the following

1) Please follow the trial schedule during your participation in the trial.
2) If you are currently receiving treatment or taking medication at another department or hospital, please tell your investigator or coordinator about

your situation in detail. We will inform your treating physician that you are participating in this clinical trial.Also, if you are taking any over-the-counter medications, please consult your investigator or the clinical trial coordinator in advance.

3) The tests that will be performed during the clinical trial are very important for us to know about any changes in your condition or side effects, so please follow the instructions of your investigator and the clinical trial coordinator.

4) If you experience any changes in your physical condition while using the investigational drug, please inform your investigator or the study coordinator.

5) Please follow any other precautions or instructions given by the investigator in charge of the clinical trial.

18. Institutional Review Board that has reviewed this study

The clinical trial is reviewed by the Institutional Review Board below, not only from a scientific and medical perspective, but also from an ethical perspective, including patient safety and human rights. The Clinical Trial Review Committee includes members who do not specialize in medicine or have no vested interest in our hospital.

1)Name: Chiba University Hospital Institutional Review Board

2)Type: Institutional Review Board

3)Founder: Director of Chiba University Hospital

4)Address: 1-8-1 Inohana, Chuouku, Chiba

After the clinical trial has started, we will review, upon request from the director of this hospital, whether this clinical trial can be continued if the above information is changed or if safety information, such as the occurrence of serious side effects, is obtained.

The results of the review will be reported to the director of this hospital, who will decide whether to initiate or continue the clinical trial at this hospital based on the contents of the report.

19. Contact information

If you have any questions, doubts, questions, or would like to ask again or get more detailed information about this clinical trial or treatment, please do not hesitate to ask us at any time. Even after the clinical trial has started, we will be happy to answer any questions you may have. Also, if you have any concerns about the use of this investigational drug, please do not hesitate to contact us at any time.

If you agree to participate in this clinical trial after fully understanding the details of this trial, please indicate the date of your consent and sign the consent document at the end of this explanatory document.

Please keep this Explanatory Document and the Consent Document for patients in a safe place.

The information of the Institutional Review Board (procedure manual, committee roster, and summary of meeting records) is available to the public on our website (http://www.ho.chiba-u.ac.jp) and can be freely accessed. If you would like to confirm the procedure manual, etc. directly, please ask the clinical trial coordinator or others.

Site：Chiba University Hospital

Principal Investigator：Naoya Kato

Your Investigator：________________________________

Contact us below

Weekdays (8:30-17:00)

Outpatient Gastroenterology Tel：+8143-222-7171

Clinical Trial Coordinator Office Tel：+8143-226-2630

Nighttime and Holiday Phone Service Tel：+8143-222-7171

*Please inform us that you are participating in a clinical trial in gastroenterology.

For investigator

Consent Form

I hereby give my consent to participate in the “Phase Ib Clinical Trial to Evaluate the Safety and Efficacy of Durvalumab Tremelimumab in Combination with Heavy Ion Beam Therapy in Patients with Advanced Hepatocellular Carcinoma with Vascular Invasion” on my own free will, after having received and fully understood the following information. I agree to participate in this clinical trial of my own free will after having received and fully understood the following information.

・What is a clinical trial?
・About your disease and treatment
・Objective of the clinical trial
・Method of the clinical trial
・治験のスケジュール
・Specimen (blood and tissue) storage・Duration of participation in the clinical trial and number of participants
・Foreseeable benefits
・Foreseeable disadvantages and side effects
・Free voluntary participation in the clinical trial and the ability to withdraw consent at any time
・Alternative treatment methods if you do not participate in this clinical trial
・Discontinuation after participation in a clinical trial
・Regarding any new important information obtained
・Compensation for health damage related to clinical trials
・Costs during the study
・Access to medical records and preservation of participant confidentiality
・Conflicts of Interest
・Your responsibilities during the study period
・Institutional Review Board that has reviewed this study
・Contact information

●Performing liver biopsy and liver tumor biopsy (before/after 42 days of treatment)

□ Agree □ Disagree

●Storage of specimens after completion of the clinical trial

□ Agree □ Disagree

Participant

Date of consent：＿＿＿＿＿＿ (Month, Day, Year)

Signature:：＿＿＿＿＿＿＿＿＿＿＿＿

The investigator who obtained consent

Date of signature ＿＿＿＿＿＿ (Month, Day, Year)

Signeture：＿＿＿＿＿＿＿＿＿＿＿＿

The person who provided supplementary explanation

Date of signature ＿＿＿＿＿＿ (Month, Day, Year)

Signeture：＿＿＿＿＿＿＿＿＿＿＿＿

For site

Consent Form

I hereby give my consent to participate in the “Phase Ib Clinical Trial to Evaluate the Safety and Efficacy of Durvalumab Tremelimumab in Combination with Heavy Ion Beam Therapy in Patients with Advanced Hepatocellular Carcinoma with Vascular Invasion” on my own free will, after having received and fully understood the following information. I agree to participate in this clinical trial of my own free will after having received and fully understood the following information.

- What is a clinical trial?
- About your disease and treatment
- Objective of the clinical trial
- Method of the clinical trial
- 治験のスケジュール
- Specimen (blood and tissue) storage • Duration of participation in the clinical trial and number of participants
- Foreseeable benefits
- Foreseeable disadvantages and side effects
- Free voluntary participation in the clinical trial and the ability to withdraw consent at any time
- Alternative treatment methods if you do not participate in this clinical trial
- Discontinuation after participation in a clinical trial
- Regarding any new important information obtained
- Compensation for health damage related to clinical trials
- Costs during the study
- Access to medical records and preservation of participant confidentiality
- Conflicts of Interest
- Your responsibilities during the study period
- Institutional Review Board that has reviewed this study
- Contact information

●Performing liver biopsy and liver tumor biopsy (before/after 42 days of treatment)

☐ Agree ☐ Disagree

●Storage of specimens after completion of the clinical trial

☐ Agree ☐ Disagree

Participant

Date of consent：　　　　　　(Month, Day, Year)

Signature:：

The investigator who obtained consent

Date of signature　　　　　　(Month, Day, Year)

Signeture：

The person who provided supplementary explanation

Date of signature　　　　　　(Month, Day, Year)

Signeture：

For particopant

Consent Form

I hereby give my consent to participate in the “Phase Ib Clinical Trial to Evaluate the Safety and Efficacy of Durvalumab Tremelimumab in Combination with Heavy Ion Beam Therapy in Patients with Advanced Hepatocellular Carcinoma with Vascular Invasion” on my own free will, after having received and fully understood the following information. I agree to participate in this clinical trial of my own free will after having received and fully understood the following information.

・What is a clinical trial?
・About your disease and treatment
・Objective of the clinical trial
・Method of the clinical trial
・治験のスケジュール
・Specimen (blood and tissue) storage・Duration of participation in the clinical trial and number of participants
・Foreseeable benefits
・Foreseeable disadvantages and side effects
・Free voluntary participation in the clinical trial and the ability to withdraw consent at any time
・Alternative treatment methods if you do not participate in this clinical trial
・Discontinuation after participation in a clinical trial
・Regarding any new important information obtained
・Compensation for health damage related to clinical trials
・Costs during the study
・Access to medical records and preservation of participant confidentiality
・Conflicts of Interest
・Your responsibilities during the study period
・Institutional Review Board that has reviewed this study
・Contact information

●Performing liver biopsy and liver tumor biopsy (before/after 42 days of treatment)

☐ Agree ☐ Disagree

●Storage of specimens after completion of the clinical trial

☐ Agree ☐ Disagree

Participant

Date of consent：＿＿＿＿＿＿＿ (Month, Day, Year)

Signature:：＿＿＿＿＿＿＿＿＿＿＿＿＿＿

The investigator who obtained consent

Date of signature ＿＿＿＿＿＿ (Month, Day, Year)

Signeture：＿＿＿＿＿＿＿＿＿＿＿

The person who provided supplementary explanation

Date of signature ＿＿＿＿＿＿ (Month, Day, Year)

Signeture：＿＿＿＿＿＿＿＿＿＿＿

For investigator

Consent Withdrawal Form

I am participating in the "Phase Ib Clinical Trial to Evaluate the Safety and Efficacy of Durvalumab Tremelimumab in Combination with Heavy Grain Therapy in Patients with Advanced Hepatocellular Carcinoma with Vascular Invasion".I have read and agreed to the following terms and conditions,However, after reexamination of the information, I hereby withdraw my consent as follows

I withdraw my consent for the storage of the specimen provided in the above study.

Participant

Date of consent to withdrawal：＿＿＿＿＿＿ (Month, Day, Year)

Signature:：＿＿＿＿＿＿＿＿＿＿＿＿

The investigator who obtained consent

Date of signature ＿＿＿＿＿ (Month, Day, Year)

Signeture：＿＿＿＿＿＿＿＿＿

The person who provided supplementary explanation

Date of signature ＿＿＿＿＿ (Month, Day, Year)

Signeture：＿＿＿＿＿＿＿＿＿

For site

Consent Withdrawal Form

I am participating in the “Phase Ib Clinical Trial to Evaluate the Safety and Efficacy of Durvalumab Tremelimumab in Combination with Heavy Grain Therapy in Patients with Advanced Hepatocellular Carcinoma with Vascular Invasion”.I have read and agreed to the following terms and conditions,However, after reexamination of the information, I hereby withdraw my consent as follows

I withdraw my consent for the storage of the specimen provided in the above study.

Participant

Date of consent to withdrawal：__________ (Month, Day, Year)

Signature:：____________________

The investigator who obtained consent

Date of signature __________ (Month, Day, Year)

Signeture：________________

The person who provided supplementary explanation

Date of signature __________ (Month, Day, Year)

Signeture：________________

For participant

Consent Withdrawal Form

I am participating in the “Phase Ib Clinical Trial to Evaluate the Safety and Efficacy of Durvalumab Tremelimumab in Combination with Heavy Grain Therapy in Patients with Advanced Hepatocellular Carcinoma with Vascular Invasion”.I have read and agreed to the following terms and conditions,However, after reexamination of the information, I hereby withdraw my consent as follows

I withdraw my consent for the storage of the specimen provided in the above study.

Participant

Date of consent to withdrawal：_______________ (Month, Day, Year)

Signature:：________________________________

The investigator who obtained consent

Date of signature ____________ (Month, Day, Year)

Signeture：_______________________

The person who provided supplementary explanation

Date of signature ____________ (Month, Day, Year)

Signeture：_______________________

About a phase Ib study of durvalumab (MEDI4736) tremelimumab combined with particle therapy in advanced hepatocellular carcinoma patients with macrovascular invasion （DEPARTURE trial)

－ Information and Consent Form－

This booklet describes a phase Ib study of durvalumab (MEDI4736) tremelimumab combined with particle therapy in advanced hepatocellular carcinoma patients with macrovascular invasion （DEPARTURE trial)<br>Please read this information sheet carefully and understand the contents of the clinical trial before making your decision on whether you would like to participate in the clinical trial.<br>If you have questions or concerns about the contents or terms, please feel free to ask the study doctor or clinical research coordinator.

Chiba University Hospital
Principal investigator：Naoya Kato

Date prepared：02 07, 2023
Version number; 3.0

Table of Contents

1. What is a clinical trial？ 1
2. About your disease and treatment 3
3. Objective of the clinical trial 7
4. Method of the clinical trial 8
5. Specimen (blood and tissue) storage 17
6. Duration of participation in the clinical trial and number of participants 18
7. Foreseeable benefits 19
8. Foreseeable disadvantages and side effects 19
9. Free voluntary participation in the clinical trial and the ability to withdraw consent at any time 31
10. Alternative treatment methods if you do not participate in this clinical trial 31
11. Discontinuation after participation in a clinical trial 32
12. Regarding any new important information obtained 32
13. Compensation for health damage related to clinical trials 33
14. Costs during the study 33
15. Access to medical records and preservation of participant confidentiality 34
16. Conflicts of Interest 35
17. Your responsibilities during the study period 35
18. Institutional Review Board that has reviewed this study 36
19. Contact information 37

Please read this information sheet carefully and understand the contents of the clinical trial before making your decision on whether you would like to participate in the clinical trial. You are free to decide whether or not to participate. Also, you do not have to decide on the spot after receiving the explanation. You may decide after discussing the contents of this explanatory document with your family. You will not be disadvantaged in any manner if you decline to participate. You may withdraw from the clinical trial at any time even after you have agreed to participate in the clinical trial or after the clinical trial has started. If you have questions or concerns about the contents or terms, please feel free to ask the study doctor or clinical research coordinator.

1. What is a clinical trial？

In order to investigate the effectiveness and safety of a drug, it is necessary to conduct trials in which healthy people and patients participate, and these trials are called clinical studies. Of these, clinical studies in which results and data are collected in order to have the drug approved as a drug by the government (Ministry of Health, Labor and Welfare) are called “clinical trials. Clinical trials have a research aspect, but they are also important trials to enable many patients to receive new treatments and require the cooperation of patients and the consideration of specialized physicians. The drug used in a clinical trial is called an “investigational new drug,” and it is stipulated that the clinical trial should be conducted in compliance with the rules set by the government (GCP). This clinical trial is also conducted in compliance with these rules.

There are several phases in a clinical trial.

Overview of Treatment Development

Basic Research	Scientists screen a vast number of candidate compounds to identify those with the potential to become drugs.
Preclinical Testing	The efficacy and safety of the selected compounds are studied using animal models.
Clinical Trials	The safety and effectiveness of the drug are evaluated in humans.<br>Phase I Trial<br>Conducted with a small group of patients to assess the drug's safety and how it is distributed and processed in the body.<br>Phase Ib Trial<br>Evaluates the combination of a new treatment with existing therapies (this applies in the current study).<br>Phase II Trial<br>Conducted with a limited number of patients to further assess the drug's effectiveness and safety.<br>Phase III Trial<br>Involves a larger group of patients to compare the new drug's efficacy and safety against standard treatments.
Regulatory Review and Approval	Based on preclinical and clinical trial data, the Ministry of Health, Labour and Welfare (MHLW) rigorously evaluates whether the drug is appropriate for use. This process also ensures that the clinical trials were conducted ethically and scientifically. Once approved, the drug can be marketed and made available to patients.

In clinical trials for anticancer drugs, information gathered in three trials (Phase I-III) or two trials (Phase I and II), as described above, is submitted to the Ministry of Health, Labor and Welfare for review.

The clinical trial introduced here is a Phase Ib trial, which is a "investigator-initiated clinical trial" conducted by investigators who are actually involved in the actual medical treatment of the patient and is planned after considering the medical necessity and importance of the drug. This trial is conducted with the permission of the hospital director based on the deliberations of the Chiba University Hospital Clinical Trial Review Committee. Please see p.32 for more information about the committee.

2. About your disease and treatment

As hepatocellular carcinoma progresses, it can develop vascular invasion (tumor invasion of the portal vein and hepatic vein, important blood vessels that pass through the liver) and metastasis to organs other than the liver. This clinical trial is open to patients diagnosed with hepatocellular carcinoma with vascular invasion.

Main medical conditions that occur in patients with hepatocellular carcinoma with vascular invasion (varies from patient to patient)

(1) Fever, loss of appetite, fatigue, etc. due to tumor
(2) Abdominal pain due to the tumor, pain due to the metastatic site, and other symptoms
(3) intrahepatic metastasis
(4) Decrease in liver function due to the tumor
(5) Occurrence of esophageal and gastric varices

Hepatocellular carcinoma with vascular invasion is one of the most rapidly progressing forms of hepatocellular carcinoma and may also rapidly decline in strength, so treatment should be initiated as early as possible.

Currently, anticancer drug therapy (systemic chemotherapy) is the standard of care for patients with hepatocellular carcinoma with vascular invasion. The efficacy of drugs such as atezolizumab bevacizumab combination therapy, sorafenib, lenvatinib, regorafenib, ramucirumab, and

cabozantinib is known, but they are not yet fully effective. Other treatment modalities have similarly failed to show adequate results, and there is a need to develop treatments.

This clinical trial presented here was planned as one of the studies to develop safer and more effective treatment.

Studies have reported the importance of aggressive treatment of the area of vascular invasion itself, such as surgery and radiation. Immune checkpoint inhibitors (ICIs) are being actively developed as anticancer agents, and it is expected that more patients will benefit from treatment by combining ICIs with other therapies rather than ICIs alone.

<u>About the investigational drugs “Durvalumab” and “Tremelimumab</u>

Previous studies have shown that human immune action slows or controls the rate of cancer growth. However, there are cases where the natural immune response does not work and the human immune response does not kill the cancer. Research has shown that some cancer cells and immune cells produce a signal that blocks the cancer-killing action. A new drug in development blocks this signal and amplifies the immune response. These are two new drugs, durvalumab and tremelimumab. These are antibodies (proteins produced by the body's defense system). Durvalumab alone or in combination with tremelimumab may enhance the immune system's ability to detect and fight cancer. The two drugs target different signals. Durvalumab targets a cancer cell signal called PD-L1 (Programmed Cell Death Ligand 1), while tremelimumab targets an immune cell signal called CTLA-4 (Cytotoxic T-Lymphocyte-associated Antigen 4) CTLA-4 (Cytotoxic T-Lymphocyte-associated Antigen 4). By blocking these signals, it is hoped that immune cells can again control or slow the growth rate of cancer.

Durvalumab is approved in Japan under the brand name Imfinzi® for the treatment of unresectable locally advanced non-small cell lung cancer. Durvalumab monotherapy and the combination of durvalumab and tremelimumab (brand name Ijudo®) for the treatment of hepatocellular carcinoma were approved in Japan in December 2022. In addition, clinical trials are underway in lung cancer, bladder cancer, head and neck cancer, and other types of cancer. Outside of Japan, durvalumab monotherapy and the combination of durvalumab and tremelimumab for the treatment of hepatocellular carcinoma were approved the U.S. Food and Drug Administration (FDA). Also, Durvalumab is approved for the treatment of patients with locally advanced or metastatic urothelial carcinoma whose cancer has progressed during or after platinum-based chemotherapy by U.S. FDA. It is also approved by the U.S. FDA and the European Medicines Agency (EMA) in Europe for the treatment of patients with locally advanced non-small cell lung cancer who have received chemoradiation therapy. If you have any questions about the investigational drug, please ask your physician at any time.

Durvalumab

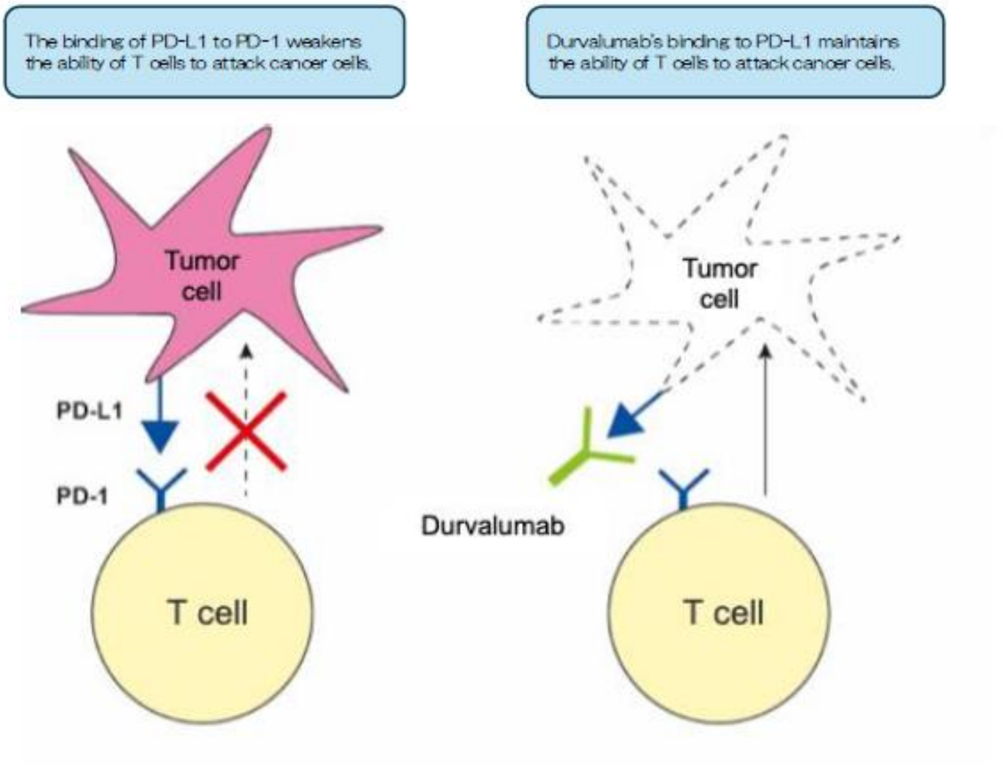

Tremelimumab

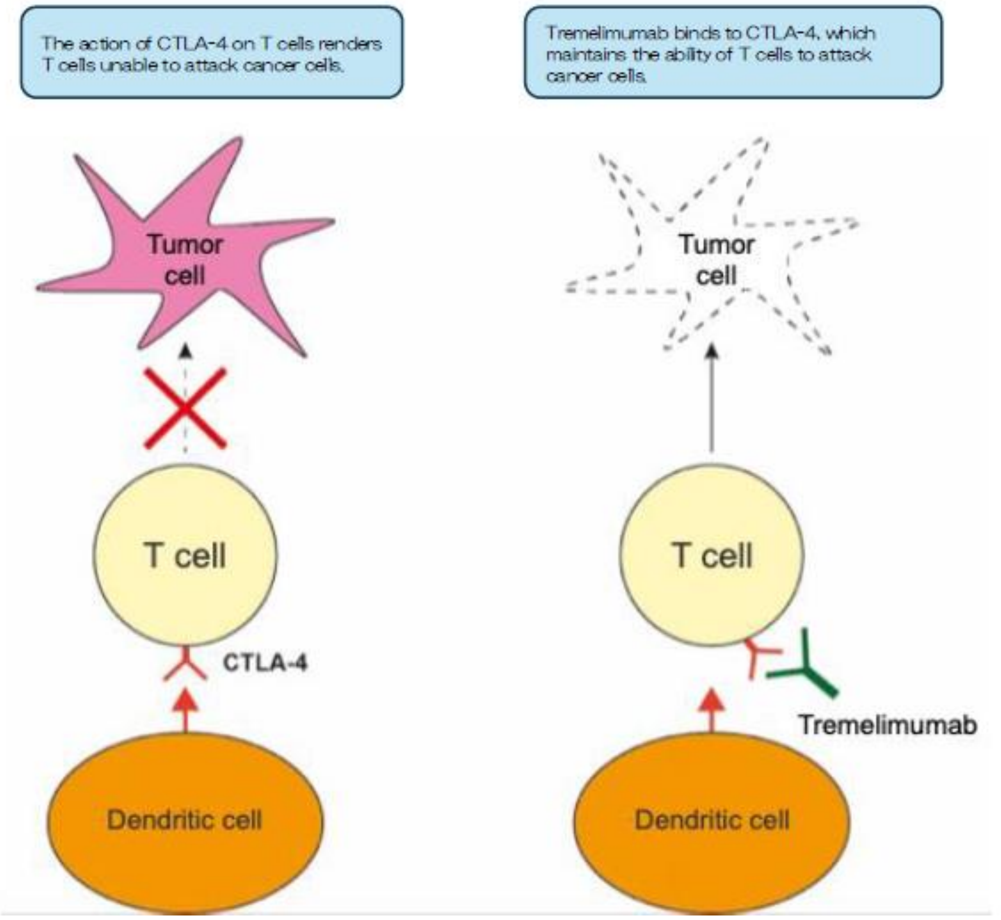

About Carbon Ion Radio Therapy

Carbon ion radio therapy is one of the radiation therapy methods for hepatocellular carcinoma. It is a new-generation treatment method classified as particle beam therapy along with proton beam therapy.

In contrast to conventional radiotherapy using X-rays, it can deliver high doses of radiation to the affected area while greatly minimizing irradiation to the normal liver. Studies of heavy-ion radiation therapy alone for hepatocellular carcinoma have reported that it is an effective treatment with minimal side effects.

Currently, hepatocellular carcinoma of 4 cm or larger is treated by medical insurance system in Japan. For patients with hepatocellular carcinoma smaller than 4 cm, research is being conducted under the advanced medical treatment system, in which the beneficiary bears the cost of the main treatment.

The participants in this study are scheduled to receive heavy particle therapy at the Hospital of the National Institute of Quantum Science and Technology.

3. Objective of the clinical trial

In this clinical trial, patients with advanced hepatocellular carcinoma will receive an infusion of durvalumab (or both durvalumab and tremelimumab) followed by treatment with heavy ion radiation. The main purpose of the study is to see what side effects may occur during this process and to determine whether this treatment can be safely administered.

The first 3-6 patients in the trial will receive durvalumab only, followed by heavy particle radiation. We will first review the physical condition of those

who receive this treatment to make sure that the treatment can be safely administered.

Patients who participate after the review will receive both durvalumab and tremelimumab infusions and heavy particle therapy, which will also be evaluated again with respect to safety.

Based on the results of both treatments, we also aim to determine which treatment regimen is considered appropriate.

4. Method of the clinical trial

If you agree to participate in this clinical trial, you will first undergo a screening test. There are three periods in this clinical trial: the screening period, the treatment period, and the follow-up period after discontinuation of the clinical trial treatment.

During the screening period, you will undergo several tests and medical examinations to confirm that you are eligible to participate in this clinical trial treatment. Your investigator and the clinical trial staff will explain the clinical trial to you. The screening period is 28 days.

The treatment period is the time during which you will receive the study drug and heavy particle irradiation.

During the follow-up period, we will check your physical condition and blood test values after treatment is discontinued.

1)Eligibility

The conditions under which a patient may or may not participate in this clinical trial are as follows

≪ Patients who can participate in this study ≫

1) Patients diagnosed with hepatocellular carcinoma with vascular invasion

2) Patients with hepatocellular carcinoma not amenable to local therapies such as radiofrequency ablation (RFA) and trans arterial chemoembolization (TACE)

3) Patients must be 20 years of age or older

4) Weigh 30 kg or more

5) Good general condition and no major obstacles in daily life

6) Your test values (neutrophils, platelets, hemoglobin, liver function, renal function, etc.) meet certain criteria

7) Your consent to participate in this clinical trial has been obtained

≪ Patients who cannot participate in this study ≫

1) Brain metastasis

2) Currently suffering from cancer of an organ other than the liver, or suffered from cancer within 3 years

3) Received treatment with immunosuppressive agents within 14 days (28 days for antibody drugs) prior to the start of treatment with the investigational drug

4) Currently or previously had an autoimmune or inflammatory disease (unless the patient is determined to have had no active inflammatory disease within the past 5 years)

5) Have any of the following conditions or diseases

- Has serious heart disease (heart failure, myocardial infarction, angina pectoris, arrhythmia requiring treatment, etc.)
- Electrocardiogram (ECG) abnormalities
- Infectious disease requiring treatment with oral or injectable medications

- Severe respiratory illness (interstitial pneumonia or pulmonary fibrosis)

- Severe mental disorders (e.g., dementia)

- Positive test result for human immunodeficiency virus (HIV)

- Current or former hepatic encephalopathy

- Had cerebrovascular disease, thrombosis, or thromboembolism within 180 days prior to initiation of investigational therapy

6) Previous radiation therapy with liver effects.

7) Pregnant or lactating women.

8) Unable to consent to proper method of contraception during participation in the study and for 180 days from the date of the last dose of study drug (both sexes)

There are many other criteria, and we will make a judgment based on our detailed examination and consultation. Therefore, please understand that you may not be able to participate in the clinical trial even after you have given your consent, and even after the clinical trial has started, we may decide to terminate your treatment or participation in the clinical trial at our discretion. Patients who participate in this trial will be assigned to one of two treatment groups: durvalumab alone or a combination of durvalumab and tremelimumab as the drug to be combined with carbon ion radio therapy. In either group, you will be admitted to the hospital to start receiving durvalumab and tremelimumab, and you will also receive carbon ion radio therapy at QST Hospital. After completion of carbon ion radio therapy, patients will be discharged after confirming that they are in good physical condition, but the length of hospital stay will not change regardless of which group they are in. The length of hospitalization will be the same for both groups. Although the length of hospitalization may be longer depending on

your physical condition, the expected length of hospitalization is approximately 3 weeks. After discharge from the hospital, patients will be followed up with outpatient visits.

The flow of this clinical trial is shown below.

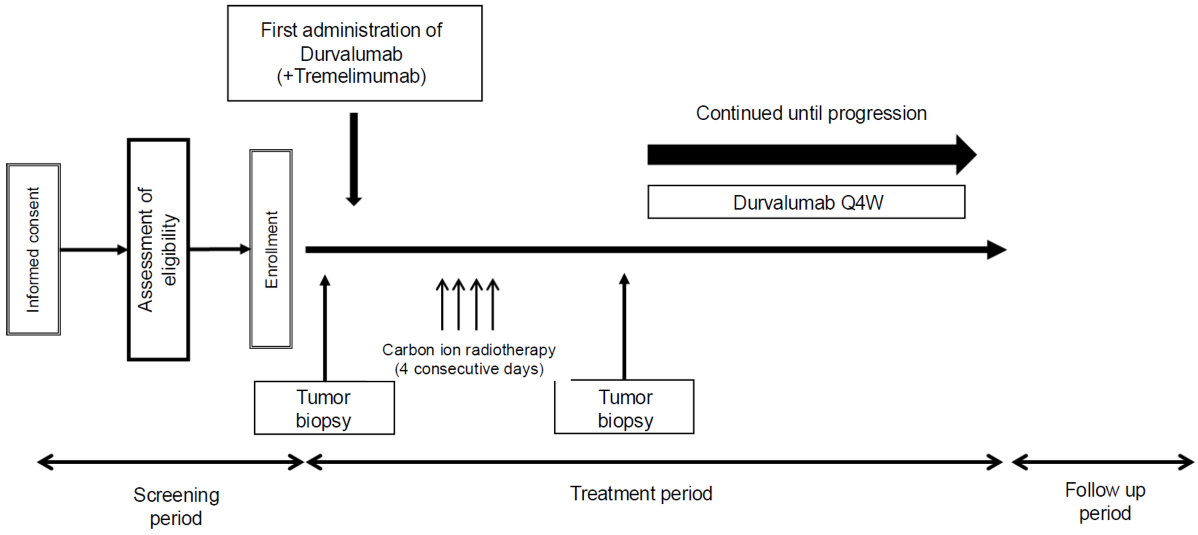

2) Detailed methods

The major objective of this study is to confirm that the combination of the investigational drug and carbon-ion radiation is safe for the human body. This confirmation of safety is called a Dose Limiting Toxicity (DLT) evaluation.

The first step in this evaluation is to assess whether the treatment is safe in two phases, with three to six patients receiving durvalumab followed by carbon ion radio therapy. This evaluation period will last 42 days from the start of treatment; if all three of the three patients, or at least five of the six patients, are found to be safe and there is no need to discontinue the trial, the next step, durvalumab and tremelimumab with carbon ion radio therapy will be given to an additional 3 to 6 patients. The same DLT evaluation will be performed on this group of patients. We plan to proceed with the clinical trial

while confirming safety, and we expect a total of 15 patients to participate in the trial at our institution.

Depending on the timing of your participation in the clinical trial as described above, you will either receive durvalumab and carbon ion radio therapy or durvalumab plus tremelimumab and carbon ion radio therapy. Your investigator or clinical trial coordinator will explain this to you each time.

As a general rule, to ensure your safety, you will be hospitalized from the time you receive your first dose of study drug until the time you receive carbon ion radio therapy. Specific administration methods and other details will be explained next.

① Dosing schedule

In this trial, 28 days are counted as one cycle.

Durvalumab (or both durvalumab and tremelimumab) will be administered intravenously on Day 1 of Cycle 1. The infusion treatment takes about 1.5 hours when only durvalumab is given, and about 3 hours when both durvalumab and tremelimumab are given.

Carbon ion radio therapy is then administered over the next 8 days (4 days total).

From Cycle 2 onward, durvalumab will be administered intravenously once every 4 weeks (on Day 1 of each cycle) after confirming that it can be administered safely according to the "Guidelines for the Management of Toxicity in Investigational New Drugs".

Your physical condition will be monitored especially carefully during the first 42 days after the first dose. Durvalumab will be continued and treatment will be continued while monitoring side effects and changes in your condition.

②Carbon ion radio therapy (and targeted marker insertion)

In this treatment, carbon-ion radiation must be focused on the lesion as precisely as possible. Otherwise, normal tissue, not cancer cells, will be damaged. During the actual irradiation, you will lie on a treatment bed in a special treatment room, and you will be placed in a fixture to hold your body in place, and you will be asked to cooperate by not moving your body for a while. If you experience pain because the fixture does not fit properly, do not be patient and let us know. Carbon-ion radiation is given for a few minutes, but you will be in the treatment room for a total of 20 to 30 minutes, including the time needed for preparation and treatment before and after the irradiation. During the irradiation, you will be alone in the treatment room, but you do not need to worry because we will be watching you from outside the room via a TV camera, and if necessary, you can press the emergency buzzer to stop the irradiation and we will come to your room. You will not feel any pain or heat from the carbon-ion radiation during the irradiation.

Carbon ion radio therapy will be given once a day, for a total dose of 60 Gy (RBE). This therapy will be completed in one week.

In this trial, a “target marker” is implanted in advance to accurately identify the lesion site during treatment. When the target marker is inserted from the surface of the body, local anesthesia is administered to the skin and subcutaneous tissue of the abdomen, a thin needle is inserted, and a tube is inserted into the liver. When inserted through a blood vessel in the liver, the target marker is implanted through a thin tube from an artery at the base of the groin to a site near the disease. The implanted target marker is not removed after treatment and remains in the organ, but there have been no reports of the marker directly harming the patient’s health. When inserted from the surface of the body, a needle is inserted into the liver, which may

cause bleeding, organ damage, and other side effects similar to liver biopsy (details are described in the explanation section of liver biopsy on p. 23). When inserting a catheter through a blood vessel in the liver, a method called abdominal angiography is applied. A thin tube called a catheter is inserted from a blood vessel in the leg or arm, and the catheter is advanced to a blood vessel near the tumor in the liver. A targeted marker will then be implanted in a blood vessel near the tumor. Side effects associated with abdominal angiography (such as allergic reactions to contrast media, renal dysfunction, vascular injury, bleeding, infection, and organ damage) may occur.

3）Details and schedule of tests during the clinical trial

After you agree to participate in a clinical trial and sign the Clinical Trial Consent Form, screening tests will be performed to determine if you are suitable to participate in the trial. If the results of these tests can be substituted for the tests that were performed prior to your consent, we may use the results of those tests as data for the clinical trial. However, please understand that depending on the results of the tests, you may not be able to participate in the clinical trial. During the clinical trial period, including the screening tests, you will be taken for periodic medical examinations and consultations in accordance with the schedule shown in Table 1. In addition, after the administration of the investigational drug has been completed, we will also conduct medical examinations and consultations.

(A) Schedule of medical examinations and tests.

Investigations include measurement tests by blood and urine collection, electrocardiogram, CT and MRI, and follow-up by biopsy to collect tissue

(liver tumor and non-tumor tissue). These tests are commonly performed to treat your disease and ensure your safety and are necessary for this clinical trial. If the results of the tests are deemed necessary by your physician, your visits and tests may be more frequent than those planned in Table 1.

(B) Tumor marker

There are many types of cancer, some of which produce substances characteristic of each cancer. Among such substances, those that can be measured in body fluids (mainly blood) are called tumor markers.

are called tumor markers.

Together with blood tests and imaging tests, tumor markers are used as one of the tests to check the progress of cancer and the progress of treatment.

In this case, blood will be used to measure AFP, AFP-L3, and PIVKA2, which are tumor markers for hepatocellular carcinoma. Blood samples will be drawn prior to the start of the study and prior to the administration on the first day of each of Cycle 2 and thereafter.

(C) Liver biopsy and liver tumor biopsy

Liver biopsy and liver tumor biopsy (tissue collection from non-tumor and tumor areas) will be performed on patients who have agreed to undergo liver biopsy and liver tumor biopsy. The timing of the liver biopsy/liver tumor biopsy will be before treatment and after the safety of the treatment has been confirmed for 42 days after the start of treatment. Liver tissue (non-tumor and tumor areas) obtained by biopsy will be used for exploratory studies.

Table 1: Schedule of medical examinations and tests

Cycle	Screening period	First tumor biopsy	DLT evaluation period							Second tumor biopsy	Durvalumab q4W dosing period					ST	Follow up period	Safety information collection
			Cycle 1				Cycle 2				Cycle 3	Cycle4	Cycle 5	Cycle 6	Cycle 7 and beyond			
Cycle Day	D -28 ～D -1	D -28～D -1	1	8 to 14	15	22	1	8	14		1	1	1	1	1	—	—	—
Allowable period（Day）			—	±3	±3	±3	±3	±3	±3		—	±3	±3	±3	±3	+14	+14	+14
Informed Consent ／ Subject backgroud information／<br>Review of eligibility	●																	
Durvalumab administration<br>(cohort A and B)			●				●				●	●	●	●	●			
Tremelimumab administration<br>(cohort B)			●															
Tumor biopsy		●								●								
CIRT（QST hospital）				4 times between Day8 and Day14														
Fiducial marker insertion		●																
Fixation, simulation CT (for CIRT)	●																	
Weight	●		●	●	●	●	●	●	●		●	●	●	●	●	●	●	
Height	●																	
Clinical Chemistry／Hematology	●		●	●	●	●	●	●	●		●	●	●	●	●	●	●	
Urinalysis	●		●								●	●	●	●	●	●	●	
ECG	●		●								(●)	(●)	(●)	(●)	(●)	(●)	(●)	
Chest X ray	●															●	●	
CT/MRI	●		←Every 6 weeks (±1 week) for the first 12 weeks from Cycle1 day1, and every 8 weeks (±1 week) thereafter until PD.→														●	
Tumor marker（AFP、PIVKA-II）	●						●				●	●	●	●	●			
Assessment of AE/SAE	←→																	

Table 2: Laboratory Tests for Blood and Urinalysis

Hematological Tests	Red blood cell count, Hemoglobin, Hematocrit, Platelet count, White blood cell count, White blood cell fraction（Neutrophils, Lymphocytes, Monocytes, Eosinophils, Basophils)
Biochemical examination of blood	Total protein, Albumin, Total bilirubin, AST, ALT, ALP, γ-GTP, LDH, BUN, Amylase, Lipase, Creatinine, Uric acid, Na, K, Cl, Ca, P, Mg, Blood sugar
Blood coagulation test	PT-INR、APTT
Urinalysis	Specific gravity, pH, Sugar, Protein, Ketones, Occult blood, Bilirubin, Color and appearance, Urinary sediment

5. Specimen (blood and tissue) storage

In principle, the specimens you provide for testing (blood and liver tissue (tumor and non-tumor parts)) will be stored until the end of the clinical trial.

If you give your consent, we plan to keep the specimens remaining after testing for a certain period of time (up to 20 years from the start of the clinical trial) at the Department of Gastroenterology of our hospital even after the trial has ended. This is because there is a possibility that additional research on hepatocellular carcinoma may be conducted in the future based on newly obtained findings. If used for research, it will be used only after approval by the Review Committee. When storing the specimens, we will anonymize them so that it will not be known that the specimens were collected from you.

You may participate in a clinical trial even if you do not consent to the storage of specimens after the trial is completed. You will not be disadvantaged by not consenting. Please make your decision based on your

own judgment, as there is a confirmation box on the clinical trial participation consent form.

You can withdraw your consent to the storage of specimens after the completion of the clinical trial at any time. If you withdraw, you will not be disadvantaged in any way. If you wish to withdraw your consent, please sign the Consent Withdrawal Form and submit it to your investigator or study coordinator.

However, if the person in charge at the hospital has already collected the analysis results before you submit the withdrawal of consent form, the results will not be used as they are.

If the results of the analysis have been collected by the person in charge at the hospital before you submit the withdrawal of consent, the results may be used as they are and shared with other parties.

If it is determined that there is no further need to store specimens before 20 years have passed from the start of the clinical trial, the specimens may be discarded without waiting 20 years from the start of the clinical trial.

In addition, since the results obtained are still in the research phase, they will not be disclosed to you in principle. If you wish to request disclosure, please contact us.

6. Duration of participation in the clinical trial and number of participants

Approximately 15 patients are expected to participate. The expected duration of participation in the clinical trial will be the total of the screening period (28 days) and the treatment and post-treatment follow-up periods (28

days after completion of treatment). The treatment period will be the sum of 42 days after the first dose and the period of continued durvalumab administration.

The duration of durvalumab treatment will depend on your condition and will continue as long as your investigator determines that it is effective for your disease. However, it may be discontinued if serious side effects occur and it becomes difficult to continue treatment, even if it is effective.

7. Foreseeable benefits

The combination of durvalumab tremelimumab with carbon ion radio therapy may demonstrate efficacy not seen with existing therapies.

Results of an AstraZeneca-led study of durvalumab-tremelimumab in advanced hepatocellular carcinoma have shown that safety of each agent and efficacy of the combination therapy. Carbon ion radio therapy for hepatocellular carcinoma has also been shown to be safe and effective and hepatocellular carcinoma of 4 cm or larger is treated by medical insurance system in Japan.

However, the safety and efficacy of durvalumab-tremelimumab in combination with carbon ion radio therapy is not known. Information obtained from this clinical trial may be used in future research on the treatment of this disease.

8. Foreseeable disadvantages and side effects

The side effects that may develop as a result of treatment vary greatly from person to person, and what symptoms may occur and their severity vary from person to person, and cannot be completely predicted before treatment begins. When side effects occur, treatment may be temporarily stopped, or treatment may be given to alleviate symptoms.

Serious side effects may also occur when treatment is resumed after a suspension or postponement of treatment.

If you feel that your condition is unusual, please contact your investigator for appropriate treatment.

<u>Risks associated with durvalumab and tremelimumab.</u>

Most of the side effects seen with durvalumab and tremelimumab were mild or moderate. However, some are serious, life-threatening and sometimes fatal. Some side effects do not require treatment, but symptoms usually recover with treatment. It may be necessary to delay the administration of durvalumab and tremelimumab to improve the symptoms of side effects. The most important side effects that may occur are listed below. These can occur as a result of the action of durvalumab and tremelimumab on the immune system and have been seen in patients who received either or both durvalumab and tremelimumab in previous studies. These side effects have also been seen in trials using other medicines similar to durvalumab and tremelimumab. The management of these side effects may require the administration of steroids or other medications that can work on the immune system and reduce inflammation.

The types of side effects were very similar when these two investigational drugs were given together and when durvalumab was given alone. However, the probability and severity of many, but not all, of these side effects were higher when they were given in combination than when durvalumab was given alone.

Very common side effects (>10%)

・ Diarrhea

・Rash / Dry and itchy skin

・ Liver dysfunction: Blood levels of substances called enzymes, which are found in liver cells, may increase. Changes in the enzyme do not often make you feel sick. However, if this enzyme level is very high, your investigator may need to discontinue the study drug. It may also cause inflammation of the liver, known as hepatitis, but this is rare. It may be accompanied by signs and symptoms such as yellowing of the skin and white eye area, dark urine, severe nausea and vomiting, pain in the upper right abdomen, itchy skin, inability to feel hunger, and bleeding or bruising more easily than normal.

In addition to the above, important anticipated side effects reported to be very common (i.e., >10%) in clinical trials in which patients with different types of cancer were treated with durvalumab alone or in combination with tremelimumab were fatigue, abdominal pain, swelling due to fluid retention, upper respiratory tract infection, nausea, vomiting, decreased appetite, shortness of breath, cough, fever, and muscle and joint pain.

Most common side effects (≥1% to <10%)

・ Hypothyroidism: This is caused by a decrease in the amount of thyroid hormones produced by the thyroid gland and a very slow metabolism. Symptoms include, but are not limited to, fatigue, feeling cold more easily, constipation, dry skin, unexplained weight gain, facial swelling, muscle weakness, decreased heart rate, thinning hair, and memory problems. These symptoms can be improved by thyroid hormone replacement. This eventis a very common side effect in patients receiving the combination of durvalumab and tremelimumab, but is classified as a common side effect in patients receiving durvalumab alone.

・ Pneumonia: Symptoms include, but are not limited to, new or worsening cough, shortness of breath (sometimes accompanied by fever). Pneumonia can be fatal. Limited data (not yet fully established) suggest that the incidence and severity of the disease may be higher in Japanese than in non-Japanese. If you have any of these symptoms, contact your investigator immediately.

・ Hyperthyroidism: This condition occurs when the thyroid gland produces too much thyroid hormone. Symptoms include anxiety, nervousness, weight loss, frequent bowel movements, diarrhea, shortness of breath, hot flashes, and heart palpitations. Depending on the severity of the symptoms, treatment may include observation only, symptomatic treatment, or treatment to stop the secretion of thyroid hormones.

・ Renal dysfunction: Even without symptoms or feeling sick, blood tests may show increased creatinine levels (creatinine is a protein marker that assesses kidney function). Less frequently, nephritis may occur, in which the kidneys become inflamed and lose normal function.

・Nervous system disorders: Symptoms include abnormal weakness of the leg, arm, or facial muscles, or numbness or tingling in the limbs. Rarely, severe inflammation of the nervous system may occur, which can damage nerve cells and interfere with communication between nerves and muscles. If you experience trouble swallowing, sudden weakness, or difficulty breathing, contact your doctor immediately.

・ Injection reactions: Reactions may occur during or after injection of the investigational drug. Injection reactions may cause fever or chills, changes in blood pressure, or significant dyspnea. Contact your investigator immediately if you experience any of these symptoms, even if it is several days after the injection.

・Intestinal inflammation (colitis): May cause abdominal pain and diarrhea (with or without bleeding). May be accompanied by fever. Additional intravenous fluids may be needed. If left untreated, it can cause serious and life-threatening tears in the intestinal wall. If you experience any of these symptoms, contact your doctor immediately.

・ Elevated levels of pancreatic enzymes (amylase and lipase). These enzymes are indicators of pancreatic function. In rare cases, elevated levels of these enzymes may be associated with pancreatitis (see "Rare Side Effects" below).

In addition to the above, the most common adverse reactions reported as serious (i.e., ≥1% to <10%) in clinical trials in which durvalumab was administered alone or in combination with tremelimumab to patients with various types of cancer are pneumonia, hoarse voice, urinary pain, night sweats, oral candidiasis, and muscle and joint pain.

Common side effect (≥1% to <10%) (occurs in 1 out of 10-100 patients) / Rare side effect (<1%) (occurs in 1 out of 100-1000 patients)

・ Adrenal Injury: May cause stomach pain, vomiting, muscle weakness, fatigue, mood swings, hypotension, weight loss, kidney problems, and mood and personality changes. It has been reported to occur more frequently in patients receiving the combination of durvalumab and tremelimumab, but less frequently in patients receiving durvalumab as a single agent. These complications may require permanent hormone replacement therapy.

・ Dental and oral soft tissue infections and influenza were more common in patients who received durvalumab as a single agent and rare in those who received it in combination with tremelimumab.

Rare (<1%) (occurs in 1 out of 100 to 1000 patients)

・ Pancreatitis: Pancreatitis usually presents with persistent pain in the upper abdomen (which may be made worse by eating or drinking), nausea, vomiting and weakness. Pancreatitis usually improves with simple treatment but can be severe and life-threatening. Contact your investigator immediately if you have any of these symptoms.

・ Allergic reaction: An allergic reaction may cause swelling of the face, lips, or throat, or breathing difficulties accompanied by hives or a hives-like rash. If any of these symptoms occur, contact your investigator immediately.

Rare (<1%) (occurs in 1 out of 100-1000 patients) / Very Rare (<0.1%) (occurs in 1 out of 1000-10000 patients)

・Myositis/Polymyositis: Symptoms include muscle weakness, myalgia, fatigue while standing or walking, and muscle pain lasting several weeks. It was rare in patients treated with durvalumab as a single agent and very rare in patients treated with tremelimumab in combination.

・Pituitary disorder (hypopituitarism): Hypopituitarism is a decrease in the hormone secreted by the pituitary gland in the brain and is caused by inflammation of the pituitary gland (hypopituitarism). Symptoms include headache, thirst, difficulty seeing or double vision, leakage of breast milk in women, or irregular menstruation. These complications may require permanent hormone replacement therapy. They were rare in patients who received durvalumab in combination with tremelimumab and very rare in patients who received durvalumab as a single agent.

・ Inflammation of the heart muscle (myocarditis): symptoms include chest pain, tachycardia, irregular heartbeat, shortness of breath, and swelling of the legs. If you have any of these symptoms, contact your physician immediately. This side effect is classified as an uncommon side effect in patients receiving the combination of durvalumab and tremelimumab, but very rare in patients receiving durvalumab alone.

Very Rare Adverse Reactions (<0.1%) (occurs in 1 out of 1000-10000 patients)

・Type 1 diabetes (may cause elevated blood glucose levels, known as hyperglycemia): Symptoms include weight loss, increased urination, increased thirst and hunger. type 1 diabetes requires insulin replacement by injection. If you have any of these symptoms, contact your doctor immediately.

In addition to the above, important predicted disadvantages reported as rare (i.e., less than 0.1% of patients) in clinical trials of single-agent durvalumab in patients with different types of cancer are inflammation of the membranes surrounding the heart, increased number of small clusters of inflammatory cells in various parts of the body, inflammation of the middle layer of the eye or Other eye abnormalities (e.g., inflammation of the cornea and optic nerve), inflammation of the brain or membranes lining the brain and spinal cord, hardening or straining of skin and connective tissue and loss of skin color, hematologic abnormalities (e.g., abnormal red blood cell destruction, thrombocytopenia), vascular inflammation, and rheumatic abnormalities (muscle pain and stiffness caused by inflammatory disease and autoimmune autoimmune arthritis).

In addition to these expected disadvantages when durvalumab is administered alone or in combination with durvalumab and tremelimumab, other immune-mediated side effects not previously observed may occur, and inflammatory side effects may occur in any organ or tissue.

<u>Side effects of carbon ion radio therapy</u>

１）Gastrointestinal tract

In the case that the cancer is located close to the liver surface and in close proximity to the gastrointestinal tract, such as the stomach, duodenum, or large intestine, carbon ion radio therapy may cause damage to these digestive tracts. Anorexia, nausea, diarrhea, and abdominal pain are the most common symptoms, but it is important to note that anemia due to bleeding may be detected without obvious symptoms. The symptoms range from mild erosions and ulcers that can be treated with medication and diet to severe cases that require hospitalization, such as bleeding, perforation (hole), stenosis (narrowing, making it difficult for objects to pass through), obstruction (blockage of the lumen, making it impossible to pass objects through), and adhesions. Based on the results of clinical trials on cancers in other parts of the body, we can predict to some extent the safe dose when irradiated to the gastrointestinal tract, and we will make sure that the dose irradiated to the gastrointestinal tract is less than the dose considered safe to avoid serious complications. To date, a very small number of patients have experienced mild side effects (less than 1%) that improve with medication, but there have been no serious side effects that would require invasive treatment such as surgery.

２）Skin

Various degrees of radiation dermatitis may occur as a result of carbon ion radio therapy. In most cases, the symptoms are similar to a mild sunburn (redness, itching, mild pain, etc.), but in some cases, the surface of the skin may peel, producing exudates, hyperpigmentation, and small scars (scars). If the lesion is located relatively close to the skin, the skin is also exposed to high doses of radiation, which may cause erythema, erosions, ulcers, atrophy, etc., which may require treatment with ointments. Surgical procedures such as skin grafts may be necessary, although rarely (0-2.8% in past reports), and are becoming less common due to improvements in treatment techniques.

３）Liver

Liver function in the area exposed to carbon-ion radiation will be reduced or eliminated, and overall liver function may also be reduced. In this study, treatment is designed with the expectation that sufficient liver function will remain, but it is known that radiation-induced liver injury (RILD) will occur in 1-2% of patients.

４）Bile duct

Carbon-ion radiation therapy may cause cholangitis, but there have been no cases of cholangitis directly attributable to this treatment. Although rare (less than 1% of cases), bile duct stricture may require medical treatment.

５）Lung

If a portion of the lung is irradiated with carbon-ion radiation, imaging changes, pleural effusion, coughing, fever, and dull pain may occur. In severe cases, sputum, blood sputum, shortness of breath, interstitial pneumonia-like shadows, pulmonary fibrosis-like shadows, and respiratory failure are possible, but since only a small portion of the lung is usually irradiated in the treatment of hepatocellular carcinoma, no serious side effects that could be caused by carbon ion radio therapy have been observed.

６）Blood

Decreases in peripheral blood white blood cell count, red blood cell count, and platelet count, and decreases in hemoglobin concentration may occur.

７）Possibility of secondary carcinogenesis

In the case of conventional radiotherapy, there is a rare possibility of new cancer development (secondary cancer) caused by radiation after a long period of time, and the same is possible with carbon ion radio therapy.

Other risks anticipated from participation in this clinical trial include

Blood sampling: Risks associated with blood sampling include temporary discomfort from needle puncture, internal bleeding, hemorrhage, and in rare instances, infection and anemia may be caused.

ECG: Risks associated with ECG testing include temporary discomfort such as itching, mild irritation, or redness of the skin where the small adhesive pad is applied. If it is necessary to shave the area where this small adhesive pad is applied, the shaving may cause irritation.

CT or MRI: You may experience some discomfort or anxiety when lying down inside the CT scan machine. The injection of contrast may cause a metallic taste in the mouth, a feeling of warmth, and in rare cases, nausea or vomiting. A reaction to the contrast agent may also occur. MRI is safe for most people. People with metal implants near vital organs cannot undergo MRI. This is because the metal can be drawn from the body to a large magnet, which can cause damage.

Biopsy (collection of liver tumor and non-tumor tissue): A biopsy is the collection of a piece of your tissue using a scalpel or needle. This time, the needle will be inserted directly into your liver, which may cause mild discomfort and internal bleeding at the site of the needle puncture. Bleeding, liver dysfunction, jaundice, infection, pneumothorax, and peritoneal seeding may also occur. The frequency of percutaneous puncture of the liver in routine examinations is about 2%. Although rare, surgery or other procedures and blood transfusions may be required for the above treatments. In addition, anesthetics and sedatives used prior to administration may cause a sudden drop in blood pressure or an irregular heartbeat, which may strain the heart. Side effects such as allergic reactions and fever may also occur.

If you become pregnant during a clinical trial, or if your partner becomes pregnant, please notify your investigator or the study coordinator immediately. If you become pregnant, your participation in the clinical trial will be terminated.

If you or your partner becomes pregnant, we will ask you for this information. If you or your partner gave birth, had a miscarriage or an abortion, please tell your investigator. If you or your partner gave birth, you may be

asked to provide information including the baby's date of birth, height and weight at birth, sex of the baby, any complications during pregnancy or delivery, and any birth defects of the baby.

9. Free voluntary participation in the clinical trial and the ability to withdraw consent at any time

Participation in this clinical trial is not mandatory, so please make your own decision.
You may withdraw from the study at any time after participating in the study, regardless of the reason, if you wish to discontinue the study or if it is difficult to continue, so please consult your physician. You will not be disadvantaged by the discontinuation of the clinical trial. After the discontinuation of a clinical trial, you will be informed of the best possible treatment by your physician.

Your doctor will then explain the best treatment options to you.

10. Alternative treatment methods if you do not participate in this clinical trial

If you do not participate in this clinical trial, please ask your physician to explain in detail the other treatment options that may be available to you. You will not be disadvantaged by not participating in this clinical trial. Your physician will discuss with you the best treatment for your condition. Options include participating in a clinical trial for another drug, treatment with an already approved drug, or radiation therapy. Supportive care (pain and suffering control) is also available.

11. Discontinuation after participation in a clinical trial

Even after you have given your consent to participate in a clinical trial, we may discontinue the clinical trial treatment for the following reasons

① When you request discontinuation.
② In case of strong side effects during the clinical trial
③ When cancer has become large
④ When it is deemed difficult to continue the clinical trial due to circumstances at the hospital where the clinical trial is being conducted.
⑤ Other cases in which the investigator determines that discontinuation of the clinical trial is necessary.

28 days after the end of treatment, an examination will be conducted to confirm safety. Even if the clinical trial is terminated due to side effects, we ask for your cooperation as we may conduct tests and medical examinations until the side effects are no longer present.

Even if the clinical trial is terminated during the course of the study, we would like to use the records up to that point, as they will be valuable materials for future cancer research. If you have any concerns about the use of your records, please contact us.

12. Regarding any new important information obtained

We will promptly inform you if we obtain new information on important efficacy, safety, or other information that we believe may affect your decision to continue participating in this clinical trial. In that case, you will be asked to make a new decision as to whether or not you wish to continue participating in the clinical trial.

13. Compensation for health damage related to clinical trials

If you experience any problems related to the investigational drug, or if you experience any health problems as a result of participating in a clinical trial, you should notify your investigator immediately. The physician will treat you for any health problems that arise as a result of your participation in a clinical trial, and you may be eligible for compensation. However, compensation may not be provided in the following cases

① When there is no causal relationship between the health hazard and the clinical trial

② In case of progression of hepatocellular carcinoma due to inadequate efficacy of the investigational drug

③ If the health damage was caused by your intentional or gross negligence

For more information, please consult your investigator or consultation service.

14. Costs during the study

If you participate in this clinical trial, the investigational drug will be provided by the investigational drug provider (AstraZeneca Corporation). In addition, the hospital will cover the costs associated with heavy particle irradiation. We will also cover the cost of transportation to and from the QST Hospital for carbon ion radio therapy while you are hospitalized at Chiba University Hospital. The costs of medical examination fees, tests, drugs normally used, drugs used for side effects, tests and diagnostic imaging, etc. will be borne by the patient according to the type of health insurance as in the past. You will also be responsible for the cost of hospitalization for this clinical trial treatment.

15. Access to medical records and preservation of participant confidentiality

Any personally identifiable information that may be used to identify an individual who has cooperated in a clinical trial will be strictly protected and will not be released to outside parties.Data obtained from patients will be anonymized by code numbers, etc., and will be handled in such a way that they will not be identified as belonging to that patient in reports, etc.

The results of this clinical trial will be submitted to the Ministry of Health, Labour and Welfare (MHLW) in order to have the government (MHLW) approve the combination therapy of durvalumab tremelimumab and heavy ion therapy as a treatment for advanced hepatocellular carcinoma with vascular invasion. In addition, in order to study the efficacy and safety of this investigational drug in more detail, data necessary to evaluate the investigational drug, such as tests, images, and electrocardiograms used as data in the clinical trial, may be submitted to an outside organization.

Furthermore, information obtained from this clinical trial may be compiled from records collected from hospitals in various regions and published in academic societies and medical journals. In all cases, however, your name will not be used and your personal information (name, address, telephone number, etc.) will be kept confidential and will not be leaked to outside parties.

In addition, in order to check whether the clinical trial is being conducted properly, personnel from the investigational drug development organization, the Clinical Trial Review Committee of this hospital, and regulatory authorities such as the Ministry of Health, Labor and Welfare may access your medical records, including your medical records from other departments and the period before your participation in the clinical trial. Even in such cases, these officials are obligated to maintain confidentiality and your privacy will be protected.

Even if you have received treatment at another medical institution, we may contact your physician at the other institution by phone or letter to request

medical information. By signing the consent form at the end of this letter, you are also giving your consent to access your records and to the collection of information from other medical institutions.

You may withdraw your consent to the use of your medical information at any time, but please note that you will not be able to participate in this clinical trial after that. If you wish to withdraw your consent, please inform your doctor or hospital staff in charge. The clinical trial data collected before you revoke your consent will be used in the same way as described above, but after you revoke your consent, your medical information will not be used except to confirm that this clinical trial has been conducted properly.

16. Conflicts of Interest

This study is being funded and conducted by AstraZeneca Inc. At the same time, we are receiving the investigational drug and information on the safety of the drug, but there is no profit from the conduct of this study itself or from the analysis and reporting of the results. This has been reviewed by our Conflict-of-interest Management Committee, which has confirmed that no conflicts of interest* (possible conflicts of interest) exist with regard to the implementation of this clinical trial.

*Conflicts of interest are defined as actions that may compromise patient safety or distort the interpretation of data for the benefit of oneself or a pharmaceutical company, for example, when receiving funding or other benefits from a pharmaceutical company.

17. Your responsibilities during the study period

If you agree to participate in this clinical trial, please observe the following

1) Please follow the trial schedule during your participation in the trial.

2) If you are currently receiving treatment or taking medication at another department or hospital, please tell your investigator or coordinator about

your situation in detail. We will inform your treating physician that you are participating in this clinical trial.Also, if you are taking any over-the-counter medications, please consult your investigator or the clinical trial coordinator in advance.

3) The tests that will be performed during the clinical trial are very important for us to know about any changes in your condition or side effects, so please follow the instructions of your investigator and the clinical trial coordinator.

4) If you experience any changes in your physical condition while using the investigational drug, please inform your investigator or the study coordinator.

5) Please follow any other precautions or instructions given by the investigator in charge of the clinical trial.

18. Institutional Review Board that has reviewed this study

The clinical trial is reviewed by the Institutional Review Board below, not only from a scientific and medical perspective, but also from an ethical perspective, including patient safety and human rights. The Clinical Trial Review Committee includes members who do not specialize in medicine or have no vested interest in our hospital.

1)Name: Chiba University Hospital Institutional Review Board

2)Type: Institutional Review Board

3)Founder: Director of Chiba University Hospital

4)Address: 1-8-1 Inohana, Chuouku, Chiba

After the clinical trial has started, we will review, upon request from the director of this hospital, whether this clinical trial can be continued if the above information is changed or if safety information, such as the occurrence of serious side effects, is obtained.

The results of the review will be reported to the director of this hospital, who will decide whether to initiate or continue the clinical trial at this hospital based on the contents of the report.

19. Contact information

If you have any questions, doubts, questions, or would like to ask again or get more detailed information about this clinical trial or treatment, please do not hesitate to ask us at any time. Even after the clinical trial has started, we will be happy to answer any questions you may have. Also, if you have any concerns about the use of this investigational drug, please do not hesitate to contact us at any time.

If you agree to participate in this clinical trial after fully understanding the details of this trial, please indicate the date of your consent and sign the consent document at the end of this explanatory document.

Please keep this Explanatory Document and the Consent Document for patients in a safe place.

The information of the Institutional Review Board (procedure manual, committee roster, and summary of meeting records) is available to the public on our website (http://www.ho.chiba-u.ac.jp) and can be freely accessed. If you would like to confirm the procedure manual, etc. directly, please ask the clinical trial coordinator or others.

Site：Chiba University Hospital

Principal Investigator：Naoya Kato

Your Investigator：______________________________

Contact us below

Weekdays (8:30-17:00)

Outpatient Gastroenterology Tel：+8143-222-7171

Clinical Trial Coordinator Office Tel：+8143-226-2630

Nighttime and Holiday Phone Service Tel：+8143-222-7171

*Please inform us that you are participating in a clinical trial in gastroenterology.

For investigator

Consent Form

I hereby give my consent to participate in the “Phase Ib Clinical Trial to Evaluate the Safety and Efficacy of Durvalumab Tremelimumab in Combination with Heavy Ion Beam Therapy in Patients with Advanced Hepatocellular Carcinoma with Vascular Invasion” on my own free will, after having received and fully understood the following information. I agree to participate in this clinical trial of my own free will after having received and fully understood the following information.

・What is a clinical trial?
・About your disease and treatment
・Objective of the clinical trial
・Method of the clinical trial
・治験のスケジュール
・Specimen (blood and tissue) storage・Duration of participation in the clinical trial and number of participants
・Foreseeable benefits
・Foreseeable disadvantages and side effects
・Free voluntary participation in the clinical trial and the ability to withdraw consent at any time
・Alternative treatment methods if you do not participate in this clinical trial
・Discontinuation after participation in a clinical trial
・Regarding any new important information obtained
・Compensation for health damage related to clinical trials
・Costs during the study
・Access to medical records and preservation of participant confidentiality
・Conflicts of Interest
・Your responsibilities during the study period
・Institutional Review Board that has reviewed this study
・Contact information

●Performing liver biopsy and liver tumor biopsy (before/after 42 days of treatment)

☐ Agree ☐ Disagree

●Storage of specimens after completion of the clinical trial

☐ Agree ☐ Disagree

Participant

Date of consent：________ (Month, Day, Year)

Signature:：____________

The investigator who obtained consent

Date of signature ________ (Month, Day, Year)

Signeture：____________

The person who provided supplementary explanation

Date of signature ________ (Month, Day, Year)

Signeture：____________

For site

Consent Form

I hereby give my consent to participate in the "Phase Ib Clinical Trial to Evaluate the Safety and Efficacy of Durvalumab Tremelimumab in Combination with Heavy Ion Beam Therapy in Patients with Advanced Hepatocellular Carcinoma with Vascular Invasion" on my own free will, after having received and fully understood the following information. I agree to participate in this clinical trial of my own free will after having received and fully understood the following information.

- What is a clinical trial?
- About your disease and treatment
- Objective of the clinical trial
- Method of the clinical trial
- 治験のスケジュール
- Specimen (blood and tissue) storage • Duration of participation in the clinical trial and number of participants
- Foreseeable benefits
- Foreseeable disadvantages and side effects
- Free voluntary participation in the clinical trial and the ability to withdraw consent at any time
- Alternative treatment methods if you do not participate in this clinical trial
- Discontinuation after participation in a clinical trial
- Regarding any new important information obtained
- Compensation for health damage related to clinical trials
- Costs during the study
- Access to medical records and preservation of participant confidentiality
- Conflicts of Interest
- Your responsibilities during the study period
- Institutional Review Board that has reviewed this study
- Contact information

●Performing liver biopsy and liver tumor biopsy (before/after 42 days of treatment)

☐ Agree ☐ Disagree

●Storage of specimens after completion of the clinical trial

☐ Agree ☐ Disagree

Participant

Date of consent：＿＿＿＿＿＿ (Month, Day, Year)

Signature:：＿＿＿＿＿＿＿＿＿＿

The investigator who obtained consent

Date of signature ＿＿＿＿＿＿ (Month, Day, Year)

Signeture：＿＿＿＿＿＿＿＿＿＿

The person who provided supplementary explanation

Date of signature ＿＿＿＿＿＿ (Month, Day, Year)

Signeture：＿＿＿＿＿＿＿＿＿＿

For particopant

Consent Form

I hereby give my consent to participate in the “Phase Ib Clinical Trial to Evaluate the Safety and Efficacy of Durvalumab Tremelimumab in Combination with Heavy Ion Beam Therapy in Patients with Advanced Hepatocellular Carcinoma with Vascular Invasion” on my own free will, after having received and fully understood the following information. I agree to participate in this clinical trial of my own free will after having received and fully understood the following information.

- What is a clinical trial?
- About your disease and treatment
- Objective of the clinical trial
- Method of the clinical trial
- 治験のスケジュール
- Specimen (blood and tissue) storage • Duration of participation in the clinical trial and number of participants
- Foreseeable benefits
- Foreseeable disadvantages and side effects
- Free voluntary participation in the clinical trial and the ability to withdraw consent at any time
- Alternative treatment methods if you do not participate in this clinical trial
- Discontinuation after participation in a clinical trial
- Regarding any new important information obtained
- Compensation for health damage related to clinical trials
- Costs during the study
- Access to medical records and preservation of participant confidentiality
- Conflicts of Interest
- Your responsibilities during the study period
- Institutional Review Board that has reviewed this study
- Contact information

●Performing liver biopsy and liver tumor biopsy (before/after 42 days of treatment)

☐ Agree ☐ Disagree

●Storage of specimens after completion of the clinical trial

☐ Agree ☐ Disagree

Participant

Date of consent：__________ (Month, Day, Year)

Signature:：____________________

The investigator who obtained consent

Date of signature __________ (Month, Day, Year)

Signeture：____________________

The person who provided supplementary explanation

Date of signature __________ (Month, Day, Year)

Signeture：____________________

For investigator

Consent Withdrawal Form

I am participating in the “Phase Ib Clinical Trial to Evaluate the Safety and Efficacy of Durvalumab Tremelimumab in Combination with Heavy Grain Therapy in Patients with Advanced Hepatocellular Carcinoma with Vascular Invasion”.I have read and agreed to the following terms and conditions,However, after reexamination of the information, I hereby withdraw my consent as follows

I withdraw my consent for the storage of the specimen provided in the above study.

Participant

Date of consent to withdrawal：＿＿＿＿＿＿(Month, Day, Year)

Signature:：＿＿＿＿＿＿＿＿＿＿＿＿

The investigator who obtained consent

Date of signature ＿＿＿＿＿(Month, Day, Year)

Signeture：＿＿＿＿＿＿＿＿

The person who provided supplementary explanation

Date of signature ＿＿＿＿＿(Month, Day, Year)

Signeture：＿＿＿＿＿＿＿＿

For site

Consent Withdrawal Form

I am participating in the “Phase Ib Clinical Trial to Evaluate the Safety and Efficacy of Durvalumab Tremelimumab in Combination with Heavy Grain Therapy in Patients with Advanced Hepatocellular Carcinoma with Vascular Invasion”.I have read and agreed to the following terms and conditions,However, after reexamination of the information, I hereby withdraw my consent as follows

I withdraw my consent for the storage of the specimen provided in the above study.

Participant

Date of consent to withdrawal：______________ (Month, Day, Year)

Signature:：______________________________

The investigator who obtained consent

Date of signature ____________ (Month, Day, Year)

Signeture：_______________________

The person who provided supplementary explanation

Date of signature ____________ (Month, Day, Year)

Signeture：_______________________

For participant

Consent Withdrawal Form

I am participating in the "Phase Ib Clinical Trial to Evaluate the Safety and Efficacy of Durvalumab Tremelimumab in Combination with Heavy Grain Therapy in Patients with Advanced Hepatocellular Carcinoma with Vascular Invasion" .I have read and agreed to the following terms and conditions,However, after reexamination of the information, I hereby withdraw my consent as follows

I withdraw my consent for the storage of the specimen provided in the above study.

Participant

Date of consent to withdrawal：____________ (Month, Day, Year)

Signature:：________________________

The investigator who obtained consent

Date of signature __________ (Month, Day, Year)

Signeture：____________________

The person who provided supplementary explanation

Date of signature __________ (Month, Day, Year)

Signeture：____________________
