# Supplementary material for: MVI-targeted carbon-ion radiotherapy combined with immunotherapy for advanced hepatocellular carcinoma: Phase Ib DEPARTURE trial
Source: JHEP Rep. 2026 Feb 5;8(5):101765. doi: 10.1016/j.jhepr.2026.101765 (PMC13054414; doi:10.1016/j.jhepr.2026.101765)
Supplement: Multimedia component 4 [file mmc4.zip › ClinicalTrial/Protocol.pdf]

Clinical Study Protocol

A phase Ib study of durvalumab (MEDI4736) ± tremelimumab combined with Carbon ion radiotherapy in advanced hepatocellular carcinoma patients with macrovascular invasion

Study Number: CCRC2002
Investigational drugs : Durvalumab, Tremelimumab（Concominant therapy : Carbon ion radiotherapy）
Version Number　1.0
Date　24 Feb 2021

Revision history

Date	Version Number
24 Feb 2021	1.0

This protocol includes information and data that contain privileged or confidential information and, is provided only to the investigators, clinical team staff, associates, IRBs, or the Data Monitoring Committee. Therefore, this information must not be made public without written permission from the Chief Investigator, and AstraZeneca except when explaining to subjects. These restrictions on disclosure will apply equally to all or part of the data obtained in the clinical trial for publishing or presentation.

＜Abbreviation and terms＞

Definitions of abbreviations, acronyms, and terms in this study protocol are as follows

Abbreviation /terms	Definition
AE	adverse event
AESI	adverse event of special interest
AFP	α-fetoprotein
AFP-L3	α-fetoprotein - L3
ALP	alkaline phosphatase
ALT	alanine aminotransferase
AMED	Japan Agency for Medical Research and Development
APTT	activated partial thromboplastin time
AST	aspartate aminotransferase
AUC	area under the blood concentration time curve
BICR	blinded independent central review
BP	blood pressure
BSA	body surface area
CD	cluster of differentiation
CI	confidence interval
CIRT	carbon-ion radiotherapy
Cmax	maximum observed concentration
Cmin	minimum concentration during a dosing interval
COI	conflicts of interest
CRP	C-reactive protein
CT	computed tomography
Ctrough	drug concentration at the end of the dosing interval
CTCAE	common terminology criteria for adverse events
CTLA-4	cytotoxic T lymphocyte antigen 4
CTV	clinical target volume
DAMP	damage-associated molecular pattern
DC	dendritic cell
DLT	dose limiting toxicity
DMC	data monitoring committee
DNA	deoxyribonucleic acid
ECG	electrocardiogram
ECOG	Eastern Cooperative Oncology Group
eCRF	electronic case report form
EDC	electronic data capture
EGFR	epidermal growth factor receptor
ER	electronic record
ES	electronic signature
FAS	full analysis set
FTIH	first-time-in-human
FU	follow-up
G-CSF	granulocyte-colony stimulating factor
GCP	Good Clinical Practice
GI	gastrointestinal
HAIC	hepatic arterial infusion chemotherapy

Abbreviation /terms	Definition
HBc	hepatitis B core
HBs	hepatitis B surface
HBV	hepatitis B virus
HCC	hepatocellular carcinoma
HCV	hepatitis C virus
HDV	hepatitis D virus
HIMAC	Heavy Ion Medical Accelerator in Chiba
HIV	human immunodeficiency virus
HMGB1	high mobility group box protein1
HR	hazard ratio
HR	heart rate
HRQoL	health-related quality of life
ICH	International Council for Harmonisation of Technical Requirements for Pharmaceuticals for Human Use
ICI	immune checkpoint inhibitor
IFN-γ	interferon-γ
Ig	immunoglobulin
IL	interleukin
ILD	interstitial lung disease
imAE	immune mediated adverse event
IR	investigational regimen
IRB	institutional review board
KL-6	Krebs von den Lungen-6
LDH	lactate dehydrogenase
LET	linear energy transfer
LFT	liver function test
mAb	monoclonal antibody
MedDRA	Medical Dictionary for Regulatory Activities
MOA	mechanism of action
mRECIST	modified RECIST
MRI	magnetic resonance imaging
MST	mean survival time
MTD	maximum tolerated dose
MVI	macroscopic vascular invasion
NIRS	National Institute of Radiological Sciences
NSCLC	non-small cell lung cancer
NTL	non-target lesion
NYHA	New York Heart Association
OAR	off-axis ratio
ORR	objective response rate
OS	overall survival
PD	progressive disease
PD-1	programmed cell death 1
PD-L1	programmed cell death ligand 1
PD-L2	programmed cell death ligand 2
PIVKA-II	protein induced by vitamin K absence-II

Abbreviation /terms	Definition
PK	Pharmacokinetics
PMDA	Pharmaceuticals and Medical Devices Agency
PPS	per protocol set
PR	partial response
PRO	patient reported outcome
PS	performance status
PT	preferred term
PT-INR	prothrombin time-international normalized ratio
PTV	planning target volume
QxD	quaque x day
QxW	quaque x week
QST	National Institutes for Quantum and Radiological Science and Technology
QTcF	corrected QT interval using Fridericia's formula
Q2W	quaque 2 weeks
Q3W	quaque 3 weeks
Q4W	quaque 4 weeks
RBE	relative biological effectiveness
RECIST	Response Evaluation Criteria In Solid Tumors
RESORCE	Regorafenib after Sorafenib in Patients with Hepatocellular Carcinoma
RFA	radiofrequency ablation
RILD	radiation induced liver damage
RNA	ribonucleic acid
SAE	serious adverse event
SD	stable disease
SHARP	Sorafenib Hepatocellular Carcinoma Assessment Randomized Protocol
SOP	standard operating procedure
SP-D	surfactant protein-D
SpO2	saturation of peripheral oxygen
TACE	transcatheter arterial chemoembolization
TAI	transcatheter arterial infusion
TBL	total bilirubin
TKI	tyrosine kinase inhibitor
TL	target lesion
TLR4	Toll-like receptor 4
TMGs	Toxicity Management Guidelines
TSH	thyroid stimulating hormone
TTP	time to progression
ULN	upper limit of normal
VEGF	vascular endothelial growth factor

TABLE OF CONTENTS

0. CLINICAL TRIAL OVERVIEW 9
1. INTRODUCTION 16
1.1. Introduction 16
1.2. Standard Treatment for Advanced Hepatocellular Carcinoma 16
1.3. HCC with vascular invasion and its treatment 17
1.4. Immunotherapy 17
1.5. Carbon ion radiotherapy 18
1.6. Induction of Immunogenicity by Radiotherapy 18
1.7. Combination of immune checkpoint inhibitors and carbon ion radiotherapy 19
1.8. Investigational drugs 19
1.8.1. Durvalumab 19
1.8.2. Tremelimumab 20
1.8.3. Durvalumab + tremelimumab combination therapy 20
1.8.4. Rationale for Durvalumab and Tremelimumab as Treatment Options for HCC 20
1.9. hypothesis 21
2. STUDTY OBJECTIVE 23
2.1. Objective 23
2.2. Study results regarding the appropriateness of conducting this clinical trial, efficacy, and safety for the subject disease 23
2.2.1. Durvalumab + tremelimumab combination therapy dose rationale 23
2.2.2. Dose rationale for combination regimen of durvalumab 1500 mg Q4W plus tremelimumab 300 mg × 1 dose 23
2.2.2.1.Rationale for utilizing a fixed-dose regimen for durvalumab and tremelimumab 24
2.2.3 Rationale for carbon-ion radiotherapy 24
2.2.4 Rationale for combined treatment of carbon-ion radiotherapy and immunotherapy 25
2.3. Benefit-risk and ethical assessment 25
2.3.1. Potential benefits 25
2.3.2. Overall risks 25
2.3.3. Overall benefit-risk 28
3. ELIGIBILITY 29
3.1. Diagnostic Criteria and Stage, Type, and Condition Classification 29
3.2. Inclusion criteria 29
3.3. Exclusion criteria 30
4. INFORMED CONSENT 33
4.1. Preparation and revision of informed consent form 33
4.2. Method of Obtaining Informed Consent 33
4.3. Information to be provided to subjects 34
5. STUDY DESIGN 35
5.1. Overview of study design 35
5.2. Target number of subjects and study duration 38
5.3. Monitoring for safety assessment 38
5.4. Institutional and case registration methods 39
5.4.1. Facility registration 39
5.4.2. Subject resistration 39
5.4.3. Handling of subjects who are found to be ineligible after enrollment 40
5.5. Dosing schedule and dosage/administration method 40
5.5.1. Criterion for reduction 40
5.6. Discontinuation of Investigational Drug 41
5.7. Discontinuation of individual cases from participation in clinical trials 41
5.7.1. In case of untraceable cases 42
5.7.2. Withdrawal of consent 42
5.7.3. Clinical investigator's decision 42

5.7.4. Subject weight loss 42
5.7.5. Other cases 42
5.8. Subject replacement 42
5.9. Concomitant Restricted Drugs and Concomitant Restricted Therapy 42
5.10. Follow-up treatment 44
5.11. After discontinuation of this clinical trial 44
6. CLINICAL TRIAL TREATMENT 45
6.1. Durvalumab and tremelimumab 45
6.1.1. Durvalumab 45
6.2. Control Drugs 46
6.3. Monitoring during administration 46
6.4. Management of investigational drugs 46
6.5. Disposal of unused investigational drugs 47
6.6. Packaging and labeling of investigational drugs 47
6.7. Carbon ion radiotherapy 47
6.8. subject inclusion 48
6.9. Definition of Dose-Limiting Toxicity (DLT) 49
6.10. Toxicity Management 51
6.11. Restrictions during the clinical trial 51
6.11.1. Restrictions during the clinical trial 51
6.12. Clinical Trial Procedures 52
7. OBSERVATION, EXAMINATION, AND ASSESSMENT, METHODS, AND TIMING OF IMPLEMENTATION 53
7.1. Implementation Schedule and Procedures 53
7.2. Observation, tests and assessment 55
7.2.1. Screening period 55
7.2.2. DLT assessment period 56
7.2.3. Durvalumab q4W dosing period 58
7.2.4. At the time of discontinuation of investigational drug administration 59
7.2.5. Follow up period 59
7.3. Biological sampling procedures 59
7.3.1. Guideline for blood sampling volume 59
7.3.2. Blood samples for archiving 60
7.3.3. Hepatic tumor biopsy sample 60
7.4. Asessment of efficiency 60
7.5. Assessment of safety 61
7.5.1. Clinical laboratory tests 61
7.5.2. Physical examinations 63
7.5.3. Electrocardiogram (ECG) 63
7.5.4. Vital signs 63
7.5.5. ECOG performance status 64
7.5.6. Other safety assesements 64
8. HANDLING OF ADVERSE EVENT 64
8.1. Definition 64
8.1.1. Adverse event 64
8.1.2. Severe adverse event 65
8.1.3. Adverse Events of Special Interest (AESI) 65
8.1.4. Confirmation of interstitial lung disease (ILD) 66
8.2. Assessment of severity 67
8.3. Record of adverse events and serious adverse events 67
8.4. Duration of recording and follow-up of adverse events and serious adverse events 68
8.5. Causal relationship with investigational therapy 68
8.6. Outcome definition 69
8.7. Treatment of investigational drug in the event of an adverse event 69

8.8. Treatment of heavy particle therapy equipment in the event of an adverse event 69
8.9. Relationship to Protocol Procedures 69
8.10. Adverse events based on signs and symptoms 70
8.11. Adverse events based on tests and examinations 70
8.12. Hy's Law 70
8.13. Disease progression 70
8.14. New cancer 70
8.15. Deaths 70
8.16. Reportable Adverse Events 71
8.17. Reporting of serious adverse events 71
8.17.1. Response to subjects 72
8.18. OTHER EVENTS REQUIRING REPORTING 72
8.18.1. Overdose 72
8.18.2. Hepatic function abnormality 73
8.18.3. Pregnancy 73
8.18.4. Exposure to pregnant woman 73
8.18.5. Exposure to partner 73
8.19. Medication error 74
8.20. Predicted Side Effects 74
9. ENDPOINT 77
9.1. Primary endpoint 77
9.2. Secondary endpoint 77
9.3. Exploratory endpoints 77
10. STATISTICAL METHODS AND SAMPLE SIZE DETERMINATION 77
10.1. Description of Analysis set 77
10.1.1. DLT analysis set 77
10.1.2. Safety analysis set: SAF 77
10.1.3. Full Analysis set: FAS 77
10.1.4 Efficacy Evaluable set: EES 77
10.1.5 Per protocol set ： PPS 78
10.2. Target number of cases and rationale for setting 78
10.3. Case Handling 78
10.5.1. Analysis of subject background 78
10.5.2. Analysis of primary endpoints 78
10.5.2.1. DLT Evaluation 78
10.5.2.2. Safety Evaluation 79
11. COMPLIANCE AND DEVIATION FROM THE PROTOCOL 80
12. CHANGES TO THE CLINICAL TRIAL PROTOCOL, CASE REPORT FORM, OR ANALYSIS PLAN 80
13. DISCONTINUATION, SUSPENSION, OR TERMINATION OF THE CLINICAL TRIAL 81
14. DATA MANAGEMENT 82
15. RETENTION OF SOURCE DOCUMENTS AND OTHER RECORDS 82
16. RETENTION OF SAMPLES AND USE OF SAMPLES PROVIDED BY OTHER INSTITUTIONS 83
17. SOURCE DOCUMENT VERIFICATION 83
18. QUALITY ASSURANCE 83
19. QUALITY CONTROL FOR THIS STUDY 84
20. ETHICAL CONDUCT AND GOOD CLINICAL PRACTICE(GCP) 84
21. INSTITUTIONAL REVIEW BOARD(IRB) 84
22. HEALTH DAMAGE COVERAGE AND INSURANCE 85
23. COST BURDEN FOR THIS TRIAL 85
24. TRIAL FUNDS AND CONFLICT OF INTEREST 85

25. PROVISION OF THE INVESTIGATIONAL PRODUCTS AND INTELLECTUAL PROPERTY RIGHTS....85
26.PUBLICATION....85
27. REGISTRATION FOR CLINICAL TRIAL....85
28. STUDY IMPLEMANTATION GRPUP....86
29. LIST OF INVESTIGATIONAL PRODUCT(S) FOR THIS STUDY....86
30. LIST OF REFERENCES....86

0. Clinical Trial Overview

<table>
<tr><td>Title</td><td>A phase Ib study of durvalumab (MEDI4736) ± tremelimumab combined with particle therapy in advanced hepatocellular carcinoma patients with macrovascular invasion</td></tr>
<tr><td>Objective</td><td>

Objectives:

Primary Objective:

To assess the safety and tolerability of combination therapy of durvalumab ± tremelimumab with particle therapy in advanced hepatocellular carcinoma patients with macrovascular invasion

Secondary Objectives:

To assess the efficacy of combination therapy of durvalumab ± tremelimumab with particle therapy in advanced hepatocellular carcinoma patients with macrovascular invasion

</td></tr>
<tr><td>Design</td><td>

Ib Phase, interventional, open-label, single arm

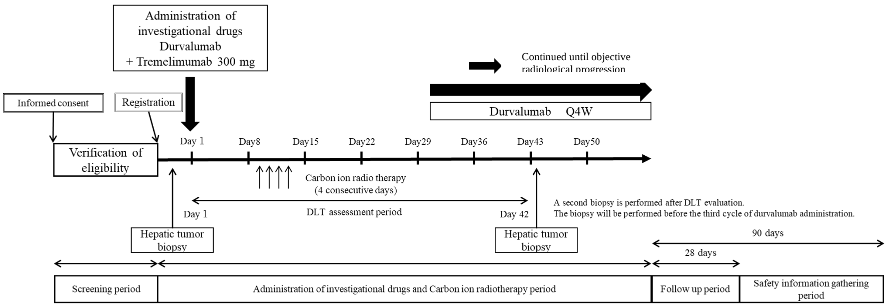

</td></tr>
<tr><td>Phase</td><td>Ib Phase</td></tr>
<tr><td>Investigational Products and Combination Therapy</td><td>

Investigational Products and Combination Therapy:

Investigational Products: Durvalumab and Tremelimumab

Durvalumab (MEDI4736) solution for infusion after dilution will be supplied in glass vials containing 500 mg durvalumab at a concentration of 50 mg/mL.

Tremelimumab solution for infusion after dilution will be supplied in glass vials containing 400 mg or 25 mg tremelimumab at a concentration of 20 mg/mL.

Combination Therapy

Carbon-Ion Radiation Therapy (CIRT) as particle therapy by Toshiba, Carbon ion radio therapy CI-1000S (PMDA approval no. 22800BZX00096000).

</td></tr>
<tr><td>Inclusion Criteria</td><td>

1) Capable of giving signed informed consent which includes compliance with the requirements and restrictions listed in the informed consent form (ICF) and in this protocol. Written informed consent and any locally required authorization obtained from the patient/legal representative prior to performing any protocol-related procedures, including screening

</td></tr>
</table>

	evaluations. For patients aged <20 years and enrolling, a written informed consent should be obtained from the patient and his or her legally acceptable representative.<br>2) Age ≥20 years at time of study entry<br>3) Eastern Cooperative Oncology Group (ECOG) performance status of 0 or 1<br>4) Body weight >30 kg<br>5) Adequate normal organ and marrow function as defined below:<br>- Haemoglobin ≧9.0 g/dL<br>- Absolute neutrophil count (ANC) ≧1,500/mm^3<br>- Platelet count ≧75×10^9/L（≧75,000/mm^3）<br>- Serum bilirubin ≦ULN×3.0<br>- AST ≦ULN×5.0<br>- ALT ≦ULN×5.0<br>- Measured creatinine clearance (CL) >40 mL/min or Calculated creatinine clearance CL>40 mL/min by the Cockcroft-Gault formula (Cockcroft and Gault 1976) or by 24-hour urine collection for determination of creatinine clearance<br>6) Evidence of post-menopausal status or negative urinary or serum pregnancy test for female pre-menopausal patients. Women will be considered post-menopausal if they have been amenorrheic for 12 months without an alternative medical cause. The following age-specific requirements apply:<br>- Women <50 years of age would be considered post-menopausal if they have been amenorrheic for 12 months or more following cessation of exogenous hormonal treatments and if they have luteinizing hormone and follicle-stimulating hormone levels in the post-menopausal range for the institution or underwent surgical sterilization (bilateral oophorectomy or hysterectomy).<br><br>- Women ≥50 years of age would be considered post-menopausal if they have been amenorrheic for 12 months or more following cessation of all exogenous hormonal treatments, had radiation-induced menopause with last menses >1 year ago, had chemotherapy-induced menopause with last menses >1 year ago, or underwent surgical sterilization (bilateral oophorectomy, bilateral salpingectomy or hysterectomy).<br><br>7) Patient is willing and able to comply with the protocol for the duration of the study including undergoing treatment and scheduled visits and examinations including follow up.<br>8) Advanced HCC confirmed histologically or by the typical findings of a hypervascular tumor on computed tomography or angiography<br>9) (Cohort A and Cohort B only) Patients who have received at least one prior systemic chemotherapy regimen including atezolizumab bevacizumab combination, sorafenib, or lenvatinib and who are determined to be refractory or intolerant to standard therapy.<br>10) Must not be eligible for locoregional therapy for unresectable HCC. For patients who progressed after locoregional therapy for HCC, locoregional therapy must have been completed ≥28 days prior to the baseline scan for the current study. Acceptable locoregional therapy for HCC are Ethanol Infusion Therapy, Radio Wave ablation Therapy, Transcatheter Arterial chemoembolization (TACE), Transcatheter arterial infusion (TAI). Hepatic Arterial Infusion Chemotherapy (HAIC) is not allowed.<br><br>11) Patients who have been diagnosed with HCC showing MVI. MVI is defined

	as a tumor thrombus in the major hepatic and/or portal vein branches (Vp2, Vp3, Vp4, Vv2, and Vv3) identified by imaging studies.<br>12) Child-Pugh A<br>13) At least one measurable lesion other than the MVI and feeding nodule based on mRECIST.
Exclusion Criteria	1. Involvement in the planning and/or conduct of the study (applies to both sponser and/or staff at the study site)<br>2. Patients who have participated in another clinical trial using the investigational drug within 28 days prior to obtaining consent or who have received another investigational drug within 28 days prior to the first dose of the investigational drug in this study. The exception is if the patient is in the follow-up period of an interventional trial or is participating in an observational (non-interventional) clinical trial.<br>3. Any unresolved toxicity NCI CTCAE Grade ≥2 from previous anticancer therapy with the exception of alopecia, vitiligo, and the laboratory values defined in the inclusion criteria<br>– Patients with Grade ≥2 neuropathy will be evaluated on a case-by-case basis after consultation with the Study Physician.<br>– Patients with irreversible toxicity not reasonably expected to be exacerbated by treatment with durvalumab or tremelimumab may be included only after consultation with the Study Physician.<br>4. Radiotherapy treatment to more than 30% of the bone marrow or with a wide field of radiation within 4 weeks of the first dose of study drug<br>5. Major surgical procedure (as defined by the Investigator) within 28 days prior to the first dose of IP. Note: Local surgery of isolated lesions for palliative intent is acceptable.<br>6. History of allogenic organ transplantation.<br>7. Active or prior documented autoimmune or inflammatory disorders (including inflammatory bowel disease [e.g., colitis or Crohn's disease], diverticulitis [with the exception of diverticulosis], systemic lupus erythematosus, Sarcoidosis syndrome, or Wegener syndrome [granulomatosis with polyangiitis, Graves' disease, rheumatoid arthritis, hypophysitis, uveitis, etc.]). The following are exceptions to this criterion:<br>– Patients with vitiligo or alopecia<br>– Patients with hypothyroidism (e.g., following Hashimoto syndrome) stable on hormone replacement<br>– Any chronic skin condition that does not require systemic therapy<br>– Patients without active disease in the last 5 years may be included but only after consultation with the study physician<br>– Patients with celiac disease controlled by diet alone<br>8. Uncontrolled intercurrent illness, including but not limited to, ongoing or active infection, symptomatic congestive heart failure, uncontrolled hypertension, unstable angina pectoris, cardiac arrhythmia, interstitial lung disease, serious chronic gastrointestinal conditions associated with diarrhea, or psychiatric illness/social situations that would limit compliance with study requirement, substantially increase risk of incurring AEs or compromise the ability of the patient to give written informed consent<br>9. History of another primary malignancy except for<br>– Malignancy treated with curative intent and with no known active disease ≥5 years before the first dose of IP and of low potential risk for recurrence<br>– Adequately treated non-melanoma skin cancer or lentigo maligna without

	evidence of disease<br>– Adequately treated carcinoma in situ without evidence of disease<br>However, the following cases are eligible for enrollment<br>Early stage cancer (epithelial cancer of the cervix, basal cell carcinoma, superficial bladder cancer (Tis and T1), early stage gastric cancer, and early stage colorectal cancer) that has been treated for curative purposes, has not been confirmed active for at least 3 years prior to inclusion in the study, and has a low risk of recurrence.<br>10. History of leptomeningeal carcinomatosis<br>11. History of, or current, brain metastases or spinal cord compression. Patients with suspected brain metastases at screening should have an MRI (preferred) or CT, each preferably with IV contrast of the brain prior to study entry.<br>12. Mean QT interval corrected for heart rate using Fridericia's formula (QTcF) ≥470 ms calculated from 3 ECGs (within 15 minutes at 5 minutes apart) Regardless of whether this criteria stays or not, all patients should have a baseline ECG<br>13. History of active primary immunodeficiency<br>14. Patients co-infected with HBV and HCV, or co-infected with HBV and hepatitis D virus (HDV). HBV positive (presence of HBsAg and/or anti-HBcAb with detectable HBV DNA); HCV positive (presence of anti-HCV antibodies); HDV positive (presence of anti-HDV antibodies), and active infection including tuberculosis (clinical evaluation that includes clinical history, physical examination and radiographic findings, and TB testing in line with local practice).<br>15. Current or prior use of immunosuppressive medication within 14 days before the first dose of durvalumab or tremelimumab. The following are exceptions to this criterion:<br>– Intranasal, inhaled, topical steroids, or local steroid injections (e.g., intra articular injection)<br>– Systemic corticosteroids at physiologic doses not to exceed 10 mg/day of prednisone or its equivalent<br>– Steroids as premedication for hypersensitivity reactions (e.g., CT scan premedication)<br>16. Receipt of live attenuated vaccine within 30 days prior to the first dose of IP. Note: Patients, if enrolled, should not receive live vaccine whilst receiving IP and up to 30 days after the last dose of IP.<br>17. Female patients who are pregnant or breastfeeding or male or female patients of reproductive potential who are not willing to employ effective birth control from screening to 90 days after the last dose of durvalumab monotherapy or180 days after the last dose of durvalumab + tremelimumab combination therapy.<br>18. Known allergy or hypersensitivity to any of the study drugs or any of the study drug excipients.<br>19. Prior randomisation or treatment in a previous durvalumab and/or tremelimumab clinical study regardless of treatment arm assignment.<br>20. Judgment by the investigator that the patient is unsuitable to participate in the study and the patient is unlikely to comply with study procedures, restrictions and requirements.<br>21. Patients who have been treated with anti-PD-1, anti-PD-L1 inhibitors, or other drugs that act on other stimulatory or co-suppressive T-cell receptors and their combinations (including atezolizumab plus bevacizumab) and

	have failed to tolerate the same treatment.<br>22. Prior radiotherapy involving the liver.<br>23. Renal failure requiring hemodialysis or peritoneal dialysis<br>24. Any of the following cardiac diseases:<br>– NYHA Class III or IV chronic heart failure<br>– Current coronary artery disease or history of ischemic heart disease such as myocardial infarction within 6 months before the study<br>– Serious arrhythmia (grade 3 or higher according to the CTCAE ver. 5.0: arrhythmia that cannot be controlled by oral medications or requires mechanical control).<br>25. Poorly controlled hypertension<br>26. Serious and active infection, excluding hepatitis viral infection<br>27. Persistent proteinuria of NCI-CTCAE version 5.0 grade 3 or higher.<br>28. Arterial or venous thrombotic or embolic events such as cerebrovascular accident, deep vein thrombosis, or pulmonary embolism within 6 months before the start of study medication.<br>29. Refractory pleural effusion or ascites<br>30. History of hepatic encephalopathy within past 12 months<br>31. Oral intake impossible<br>32. HIV-positive<br>33. Pulmonary fibrosis or interstitial pneumonitis<br>34. Other serious complications as follows: serious mental disease or history of gastrointestinal bleeding or active hemoptysis<br>35. Unsatisfactory general condition for participation in the study as judged by the primary physician
Endoints	**Primary Endpoints:**<br>AEs/SAEs including DLTs<br><br>**Secondary Endpoints:**<br>Overall Survival (OS), Survival Rate at 6 months, Objective Response Rate (ORR), Time to Progression (TTP), Progression Free Survival (PFS) at 6 months (in accordance with mRECIST)_
Dosage and treatment	Each cycle of this study will last for 28 days, and the DLT evaluation period will be 42 days from the start of study drug administration.<br>Durvalumab 1,500 mg alone or in combination with 300 mg tremelimumab IV on Cycle 1, Day 1.<br>Carbon ion radio theray (60 Gy (RBE) / 4 times / 4 days) is administered between days 8-15 of the first cycle.<br>After the DLT evaluation period, durvalumab is continued every 4 weeks until tumor progression is confirmed.
Criteria for discontinuation of individual cases	1) In case it is judged difficult to continue the clinical trial for some reason on the subject's side, such as non-attendance or transfer to a different hospital.<br>2) In case the subject requests to discontinue participation in the clinical trial<br>3) In case the investigator or subinvestigator determines that the subject is unable to continue the clinical trial.<br>4) In case the subject's body weight becomes less than 30 kg.<br>5) In case the investigator/participating investigator determines that a decision to discontinue.

<table>
<tr><td>Number of Patients Planned:</td><td>

Number of Patients Planned:

15 subjects.

The number of patients enrolled for DLT assessment in either Cohort A or Cohort B may vary from 3 to 6 depending on the frequency of DLT. If both of Cohort A and B regimens were confirmed tolerable after DLT assessment, additional patients will be enrolled in Cohort B up to a total of 15 subjects. If only Cohort A regimen was determined to be tolerable, additional patients may be enrolled in Cohort A up to a total of 15 subjects.

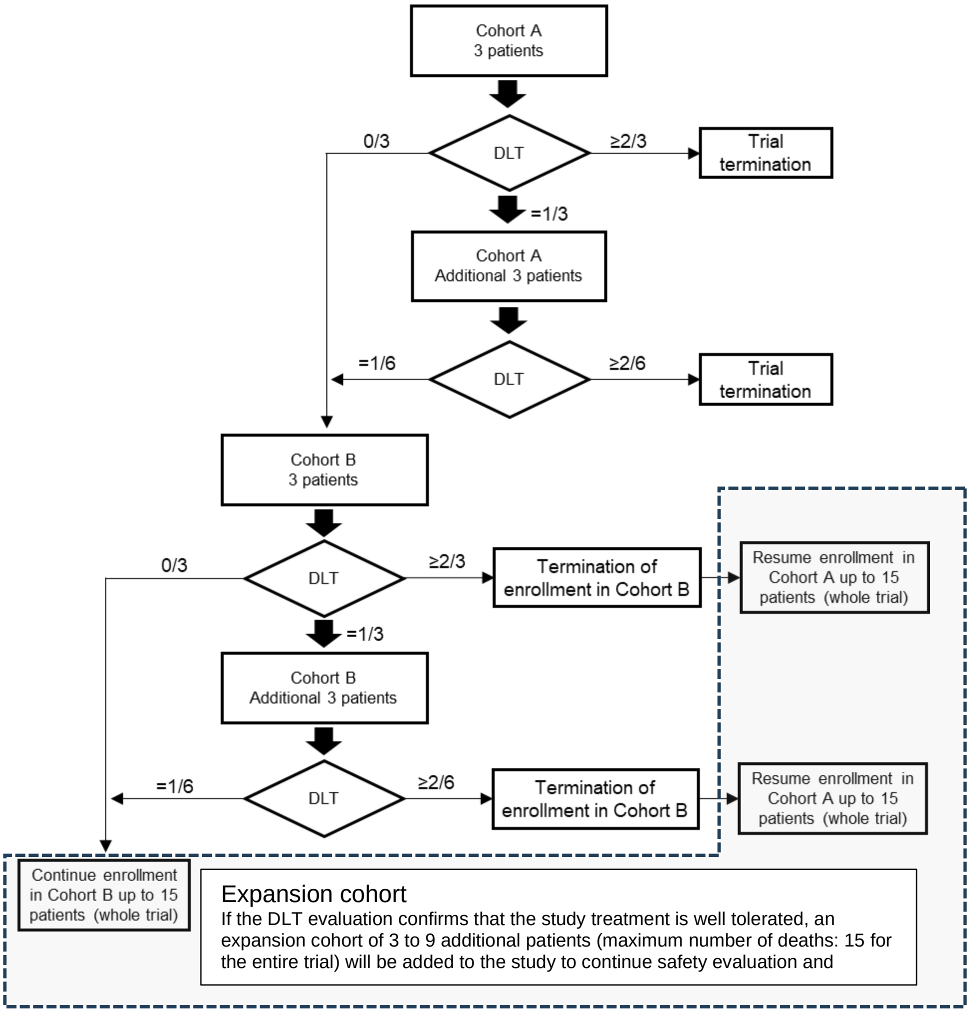

</td></tr>
<tr><td>Clinical trial period</td><td>Estimated study start date Apr 2021
Estimated study completion date Dec 2022</td></tr>
<tr><td>Subject registration period</td><td>Estimated date of first patient enrolled Apr 2021
Estimated date of last patient enrolled Jun 2022</td></tr>
<tr><td>Clnical trial sites</td><td>2Sites
Enrollment of patients in the trial and administration of IPs will be conducted at Chiba University Hospital, and CIRT will be administered at QST Hospital.</td></tr>
</table>

ETHICAL AND REGULATORY REQUIREMENTS	The study will be performed in accordance with ethical principles that have their origin in the Declaration of Helsinki and are consistent with Good Clinical Practice, and applicable regulatory requirements Patient data protection.
Institutional Review Board (IRB)	Prior to the start of the study, the Institutional Review Board (IRB) will evaluate the ethical, scientific and medical relevance of this study. The study will be conducted after obtaining approval from the IRB. If the evaluation results of the IRB are “approved with modification”, the study will be conducted after the protocol, case report, or consent forms are modified based on the review results. At the frequency of more than once a year, the IRB will continuously review whether the study is being performed appropriately.

1. INTRODUCTION

1.1. Introduction

Primary liver cancer is broadly classified into hepatocellular carcinoma (HCC) and intrahepatic cholangiocarcinoma, of which HCC accounts for more than 90% in Japan. According to a recent survey report, the number of patients with hepatocellular carcinoma in Japan is approximately 47,000 [1], and the number of deaths is approximately 27,000 per year [2].

The development of HCC is strongly associated with chronic hepatitis and cirrhosis caused mainly by hepatitis B virus (HBV) or hepatitis C virus (HCV) infection, and carriers of these viruses are considered to be at high risk of developing HCC ([3] El Serag HB, et al. NEJM. 2011). Risk factors other than viral hepatitis include cirrhosis, male age, older age, alcohol consumption, smoking, obesity, fatty liver, and diabetes mellitus [4]. Many patients are diagnosed after the disease has reached an advanced stage. The choice of treatment depends on the stage of the disease and the patient's condition. Since most patients with HCC also have chronic hepatitis or cirrhosis, individual assessment of liver function is essential to patient selection. Although several agents have been shown to be effective in the treatment of unresectable advanced HCC, there are still limited options and further therapeutic development is needed.

1.2. Standard Treatment for Advanced Hepatocellular Carcinoma

Sorafenib demonstrated a survival advantage over placebo in unresectable advanced HCC in the Sorafenib Hepatocellular Carcinoma Assessment Randomized Protocol (SHARP) trial in 2008, followed by the Asia-Pacific trial in 2009 ([5] Llovet JM, et al. Asia-Pacific trial also demonstrated a survival benefit ([5] Llovet JM, et al. NEJM. 2008, [6] Cheng AL, et al Lancet Oncol. 2009). In the SHARP trial, median survival (OS) was 10.7 months in the sorafenib group versus 7.9 months in the placebo group, with a hazard ratio (HR) of 0.69 (95% confidence interval (CI): 0.55, 0.87), P < 0.001. months vs. 4.2 months, HR 0.68 (95% CI: 0.50, 0.93), P = 0.014. The median progression-free survival in the SHARP trial was 5.5 months in the sorafenib group versus 2.8 months in the placebo group, HR 0.58 (95% CI: 0.5, 0.7), and in the Asia-Pacific trial the median progression-free survival was 2.8 months versus 1.4 months, HR 0.6 (95% CI: 0.4, 0.8).

Regorafenib after Sorafenib in Child-Pugh classification A patients with image progression after sorafenib treatment and who tolerated sorafenib (400 mg or more for at least 20 days in the 28 days before the end of treatment) compared regorafenib to placebo Patients with Hepatocellular Carcinoma (RESORCE) trial, regorafenib showed for the first time a survival advantage as second-line therapy after sorafenib treatment ([7] Bruix J, et al. Lancet. 2017). In that study, the median OS was 10.6 months (95% CI: 9.1, 12.1) in the regorafenib group and 7.8 months (95% CI: 6.3, 8.8) in the placebo group, HR 0-63 (95% CI: 0.50, 0.79) P < 0-0001. 2017, lenvatinib was non-inferiority to sorafenib for the primary endpoint of survival, making it the first-line treatment of choice for advanced hepatocellular carcinoma ([8] Kudo M, et al. Lancet. 2018). In that study, the median OS was 13.6 months (95%CI: 12.1, 14.9) in the lenvatinib arm and 12.3 months (95%CI: 10.4, 13.9) in the sorafenib arm with a HR of 0.92 (95%CI : 0.79, 1.06). In addition, ramucirumab became available in June 2019 for unresectable HCC with serum AFP levels of 400 ng/mL or higher, exacerbated after cancer chemotherapy ([9]Zhu AX, et al. Lancet Oncol. 2019).

Combination therapy with atezolizumab, an anti-PD-L1 humanized monoclonal antibody, and bevacizumab, an anti-VEGF monoclonal antibody, was shown to significantly prolong OS versus sorafenib in patients with Child-Pugh classification A who had not received systemic therapy (IMbrave 150 study) ([7 Bomze D et al. NEJM. 2020). In that study, 12-month survival rates were reported as 67.2% (95% CI: 61.3, 73.1) for atezolizumab plus bevacizumab and 54.6% (95% CI: 45.2, 64.0) for sorafenib, with an HR for OS of 0.58 (95% CI: 0.42, 0.79), P < 0 .001. Based on these results, the combination of atezolizumab and bevacizumab became available in Japan in September 2020. Based on these results, the combination of atezolizumab and

bevacizumab is now the first-line treatment for advanced hepatocellular carcinoma. Other first-line treatment options include sorafenib or lenvatinib, and second-line treatment options include regorafenib, ramucirumab, and cabozantinib. However, the survival gains obtained have been limited and do not fully meet the medical needs of patients.

1.3. HCC with vascular invasion and its treatment

HCC with vascular invasion (MVI) extending into the portal and/or hepatic veins has a poorer prognosis compared to cases without MVI ([11]Costentin CE, et al. Liver Cancer 2017). Although systemic chemotherapy is the standard of care, several studies have suggested the efficacy of alternative treatment approaches (e.g., resection, hepatic arterial chemoembolization, intravenous chemotherapy, and radiation therapy) after determining the benefit for each patient, and are recommended in Japanese guidelines. MVI Although it is empirically known that controlling MVI dramatically improves the prognosis of advanced HCC with MVI, sufficient evidence does not exist.

1.4. Immunotherapy

In recent years, research on cancer immunity has advanced dramatically, and the mechanisms by which the immune system controls or eliminates tumors are becoming clearer ([12] Dunn, et al. Annu Rev Immunol. 2004). PD-L1 is part of a complex system of receptors and ligands involved in the regulation of T cell activation. The PD-1 receptor (CD279) is expressed on the surface of activated T cells ([13] Keir ME, et al. Rev Immunol. 2008). It has two known ligands: PD-L1 (B7 H1; CD274) and PD-L2 (B7 DC; CD273) ([14] Okazaki and Honjo 2007). PD-1 and PD-L1 / PD-L2 act as co-inhibitors that can arrest or limit T cell responses When PD-L1 binds to PD-1, inhibitory signals are transmitted to T cells, cytokine production is reduced, and T cell proliferation is inhibited. Tumor cells have been shown to utilize this immune checkpoint pathway as a mechanism to evade detection and inhibit immune responses.

PD-L1 has been found to be expressed in a wide range of carcinomas, and anti-PD-L1 antibodies can be used therapeutically to augment anti-tumor immune responses in cancer patients.Results from preclinical and clinical studies of monoclonal antibodies (mAbs) targeting the PD-L1 / PD-1 pathway provide evidence of clinical activity and manageable Anti-PD-L1 antibodies can be used to augment anti-tumor immune responses in cancer patients for therapeutic purposes, showing evidence of clinical activity and manageable safety profiles ([17] Brahmer JR, et al. N Engl J Med. 2012, [18] Hirano F, et al. Cancer Res. 2005, [19] Iwai Y, et al. Proc Natl Acad Sci USA. 2002,[20] Okudaira K, et al. Int J Oncol. 2009,[21] Topalian SL, et al. N Engl J Med. 2012,[22] Zhang C,et al. Cytotherapy. 2008), The hypothesis that its efficacy is higher in patients with tumors expressing PD-L1 ([23] Powles T, et al. Nature. 2014; [24] Rizvi N, et al. J Clin Oncol. 2015; [25] Segal NH, et al. J Clin Oncol. 2015) is now supported The hypothesis of a “bladder cancer” (e.g., [24] Rizvi N et al. The high frequency and number of mutations in bladder cancer ([26] Alexandrov et al. Nature. 2013), for example, may contribute to the responses seen with immunotherapy.

In contrast, cytotoxic T lymphocyte-associated antigen 4 (CTLA-4) is structurally expressed on regulatory T cells and is enhanced on the surface of activated T cells; CTLA-4 sends negative regulatory signals to T cells when it binds to CD80 (B7.1) or CD86 (B7.2) ligands on antigen-presenting cells ([27] (Fife BT, Bluestone JA. Immnol Rev. 2008). In animal models, blockade of CTLA-4 binding to CD80 / 86 by anti-CTLA-4 antibodies has also been shown to markedly enhance T cell activation and antitumor activity, as exemplified by the killing of established solid tumors in mice and induction of protective antitumor immunity. Therefore, treatment with anti-CTLA-4 antibodies is expected to enhance activation of the human immune system and anti-tumor activity in patients with solid tumors.

Preclinical data have been added along with abundant clinical data, indicating that inhibition of negative regulatory signals to T cells, such as cytotoxic T lymphocyte antigen 4 (CTLA-4) and PD-L1, is a promising approach with promising clinical results. Ipilimumab has received U.S. Food and Drug Administration (FDA) approval for the treatment of metastatic melanoma,

and nivolumab and pembrolizumab (two anti-PD-1 agents) and atezolizumab (an anti-PD- L1 agent) are currently approved by agencies such as the U.S. Food and Drug Administration and the European Medicines Agency for the treatment of metastatic melanoma, squamous cell and non-squamous cell non-small cell lung cancer, urothelial carcinoma, and other malignancies. In addition, data from agents in the anti-PD-1 / PD-L1 class have shown clinical activity against a wide range of carcinomas.

In HCC as in other cancer types, two clinical trials (nivolumab and pembrolizumab) using ICI showed that 17-20% of patients achieved an objective response and most of the responders had a durable response ([28]El-Khoueiry AB, et al . Lancet 2017, [29]Zhu AX, et al. Lancet Oncol. 2018). The combination of ICI with tyrosine kinase inhibitors or anti-VEGF monoclonal antibodies has shown promise with the potential to achieve extremely high sustained response rates in a variety of cancer types, including HCC ([30]Kudo M. World J Gastroenterol 2019). Recently, the IMbrave 150 trial showed that atezolizumab plus bevacizumab therapy extended both OS and PFS compared with sorafenib, positioning atezolizumab plus bevacizumab as the first-line treatment for advanced hepatocellular carcinoma. Immunotherapy is also playing an increasingly important role in advanced HCC, and a phase I study of durvalumab, an anti-PD-L1 antibody, and tremelimumab, an anti-cytotoxic T lymphocyte-associated antigen 4 (CTLA-4) monoclonal antibody, in patients with HCC demonstrated a response rate of 25% ([31 was demonstrated ([31] Kelly RK et al. ASCO2017). An expanded portion of a Phase II trial is underway. A Phase III trial evaluating the efficacy and safety of the combination of durvalumab and tremelimumab, as well as monotherapy with sorafenib and durvalumab (HIMALAYA trial) are also ongoing and awaiting results.

1.5. Carbon ion radiotherapy

Particle therapy, especially carbon ion radio therapy, has advantages in dose concentration and biological effects over photon therapy, such as x-rays and gamma rays, which are usually used in radiation therapy.

X-rays and gamma rays are most intense near the body's surface and penetrate the body with decreasing intensity as they travel deeper. For this reason, the most powerful way to treat a specific area with X-rays or gamma rays is to focus the radiation from many directions, concentrating the high-dose area on the lesion. However, for tumors that are close to vital organs and spread malformedly, it is difficult to avoid surrounding normal organs. In contrast, heavy particle beams, which become intense at a certain depth depending on their energy, but are weak before and after that point, can be easily focused by aligning the peak with the tumor ([32] Pedroni E, et al. Med Phys. 1995).

The principle of action of radiotherapy is the double-strand break of DNA by ionizing radiation. Carbon ion radio therapy is known to cause dense ionization. Therefore, DNA double-strand breaks can be caused more efficiently and more densely than with X-rays. This is the reason for the favorable biological effects of heavy particle beams. This, combined with the good dose distribution resulting from the physical characteristics of heavy-ion beams, enables them to efficiently exert their anti-tumor effects. In Japan, the National Institute of Radiological Sciences (now renamed the National Institute of Quantum Science and Technology) started heavy particle therapy in 1994 and has treated more than 10,000 patients with good results ([33] Mohamad O., et al. Cancer (Basel), 2018).

In HCC, high local control rates have also been shown with particle therapy ([34] Kasuya G, et al. Cancer. 2017, [35] Fukuda, K, et al. Cancer Sci. 2017, [36] Sorin Y, et al. Liver Cancer 2018, [37] (Igaki H, et al. Int J Clin Oncol. 2018). Furthermore, good outcomes have been shown for hepatocellular carcinoma with vascular invasion. ([38] Komatsu S, et al. J Gastroenterol. 2011, [39] Lee SU, et al. Strahlenther Onkol. 2014, [40] Sugahara S, et al. Strahlenther Onkol. 2009).

1.6. Induction of Immunogenicity by Radiotherapy

Radiation therapy is known to stimulate immunogenicity through multiple mechanisms. The major immunological effects of irradiation include increased antigen presentation through

elevated expression of major histocompatibility gene complex class I, induction of apoptosis through elevated membrane expression of Fas ligands, calreticulin expression and high mobility group box-1 (HMGB1) and other (HMGB1), and induction of phagocytosis and immunity through the release of damage-associated molecular patterns (DAMPs).

Clinically, the PACIFIC trial showed that the combination of durvalumab and chemoradiotherapy can provide high therapeutic efficacy with intolerable toxicity ([41]Antonia Sj, et al. NEJM. 2017). Despite the fact that this study did not include only PD-L1-high expressing patients, it showed significant improvements in both PFS and OS compared to existing therapy. On the other hand, in the combination of durvalumab and tremelimumab (MYSTIC trial), there was no improvement from existing therapy, even when only PD-L1 high-expressing patients were targeted ([42 Rizvi NA, et al. Annals of Oncol. 2018). The difference between these two trials was the presence or absence of radiotherapy, and a subset analysis of the PACIFIC trial showed better results in patients with a shorter time between completion of radiotherapy and durvalumab administration, suggesting that radiotherapy plays an important role in tumor immunity Radiotherapy plays an important role in tumor immunity.

1.7. Combination of immune checkpoint inhibitors and carbon ion radiotherapy

Heavy ion therapy is known to enhance both local immunostimulation and immunosuppression more strongly than conventional photon beam therapy in both animal models and human clinical specimens ([43] Helm A, et al. Int J Part Ther. 2018, [44] Takahashi Y, et al. Oncotarget. 2019, [45] Iijima M, et al. J Gynecol Oncol. 2020).

Irradiated tumor cells present HMGB1, a key factor among damage-related molecules, alongside numerous others ([46] Golden EB, et al. Front Oncol, 2012). HMGB1 functions as an immune-activating cytokine and is a key factor for toll-like receptor 4 (TLR4) and activates dendritic cells.

It has been suggested that HMGB1 is strongly induced by heavy particle therapy ([47] Onishi M, et al. J Radiat Res. 2018). A study in mice showed stronger immune activation when heavy ion therapy was combined with dendritic cell infusion. The combination of heavy particle therapy and immunotherapy has also been shown to enhance anti-tumor immunity and reduce metastases compared to x-ray therapy, immunotherapy alone, or a combination of both ([48] Matsunaga A, et al. Cancer. 2010, [49] Ando K, et al. J Radiat Res. 2017).

On the other hand, immunosuppressive molecules such as PD-L1 have also been found to be induced more strongly than with X-ray therapy ([45] Iijima et al. J Gynecol Oncol. 2020). Enhanced PD-L1 expression by irradiation has been reported via the AMT/AT/Chk1 pathway induced by DNA double-strand breaks ([50] Sato N, et al. Nat Commun. 2017). Heavy ion therapy is known to efficiently generate complex DNA double-strand breaks ([51] Oike T, et al. Sci Rep. 2016), supporting this phenomenon.

Clinical trials investigating the combination of immune checkpoint inhibitors with radiation in a number of carcinomas, including HCC, are ongoing. However, most of them are limited to combinations with conventional radiation therapy using X-rays, and only a few with particle therapy. Currently, there are no clinical trials testing the combination of heavy ion beams and immunotherapy, and this clinical trial is a pilot case. From basic research and clinical studies using X-rays, the combination of heavy ion therapy and immune checkpoint inhibitors is expected to have a greater synergistic effect.

1.8. Investigational drugs

1.8.1. Durvalumab

Durvalumab is a human immunoglobulin (Ig) subclass Grade 1κ (IgGrade 1κ) mAb that inhibits the interaction of PD-L1 with PD-1 on T cells and CD80 on immune cells but not with PD-L2. Durvalumab is being developed by AstraZeneca/MedImmune for the treatment of cancer.

The mechanism of action of durvalumab is said to be inhibition of the interaction of PD-L1 with PD-1 and CD80, which disrupts the suppression of the immune response and immune

response to tumor elimination in in vitro studies, durvalumab was found to antagonize PD-L1-mediated suppression on primary human T cells, restore T cell proliferation, and release interferon gamma (IFN-γ) ([52] Stewart R, et al. Cancer Immunol Res. 2015) In vivo studies showed that durvalumab inhibited tumor growth in a xenograft model through a T cell-dependent mechanism ([52] Stewart R, et al. Cancer Immunol Res. 2015). Based on these data, durvalumab is expected to stimulate anti-tumor immune responses in patients by binding to PD-L1 and shifting the balance toward anti-tumor immune responses. Durvalumab is designed to reduce antibody-dependent and complement-dependent cellular injury.

To date, more than 8,000 patients have received durvalumab as a single agent or in combination with other anticancer agents in studies sponsored by AstraZeneca throughout the clinical development program. Please refer to the most recent Durvalumab investigational brochure for nonclinical and clinical information, including safety, efficacy, and pharmacokinetics.

1.8.2. Tremelimumab

Tremelimumab is a human immunoglobulin (Ig) Grade 2 mAb that targets CTLA-4 (CTLA-4; cluster of differentiation [CD]152), a cell surface receptor that is primarily expressed on activated T cells and blocks their activation. Tremelimumab completely inhibits the interaction of human CTLA-4 with CD80 and CD86 and increases the release of cytokines (interleukin [IL]-2 and interferon [IFN]-γ) from human T cells, peripheral blood mononuclear cells and whole blood ([53] Tarhini and Kirkwood. Expert Opin Biol Ther. 2008). Tremelimumab is being developed by AstraZeneca for the treatment of cancer.

To date, it has been administered to more than 1,500 patients as a single agent or in combination with other anticancer agents. For a summary of nonclinical and clinical information, including safety, efficacy, and pharmacokinetics, please refer to the Tremelimumab investigational brochure.

1.8.3. Durvalumab + tremelimumab combination therapy

Since there is no overlap in the mechanisms of action of CTLA-4 and PD-1, targeting both PD-1 and CTLA-4 pathways may provide additive or synergistic effects ([16] Pardoll DM, et al. Nat Rev Cancer. 2012). Therefore, in addition to investigating both drugs as monotherapy for various cancer indications, AstraZeneca is also investigating the combination of durvalumab plus tremelimumab for the treatment of cancer.

Study D4190C00006 is a late phase I dose escalation study to establish the safety, pharmacokinetics/pharmacodynamics and preliminary antitumor activity of the combination of durvalumab plus tremelimumab in patients with advanced non-small cell lung cancer (NSCLC). The dosing schedule used in this study is durvalumab administered Q2W or Q4W until Month 12, followed by 7 doses of tremelimumab at Q4W until Week 24, then 2 additional doses every 12 weeks for up to 12 months. The study is ongoing and enrollment is ongoing. Other trials investigating combination therapy for NSCLC and other tumor indications have also been initiated.

To date, 3,000 patients have received the combination in various doses and dosing regimens throughout the clinical development program. For a complete summary of non-clinical and clinical trial information, including safety, pharmacokinetics, and efficacy, please refer to the most recent Durvalumab and Tremelimumab investigational brochure.

1.8.4. Rationale for Durvalumab and Tremelimumab as Treatment Options for HCC

The liver has multiple regulatory mechanisms to maintain an immunosuppressive environment. The normal liver is inherently prone to induce immune tolerance to prevent aberrant immunity to exposed pathogens ([54]Pardee AD and Butterfield LH.Oncoimmunology. 2012). Clinical and nonclinical data indicate that HCC increases the expression of immunosuppressive cell

populations such as regulatory T cells (Treg) and myeloid-derived suppressor cells, as well as suppressive signaling molecules including CTLA-4 and PD-1 ([54] ,[55] Gao O, et al. Clin Cancer Res. 2009, [56] Hato T, et al. Hepatology. 2014); HBV and HCV infection also increase Treg and PD-L1/PD-1 expression, suggesting that this pathway is involved in HBV and HCV-mediated hepatocellular carcinogenesis ([54],[57] Miroux C, et al. Expert Opin Biol Ther. 2010,[58] Golden-Mason L, et al. J Virol. 2007,[59]Peng G, et al. Mol Immunol. 2008).

It has also been shown that overexpression of PD-L1 results in higher malignancy, disease progression, and mortality in HCC ([54], [60] Klein Jp, et al. Stat Med. 2007). Therefore, suppression of PD-L51 and CTLA-4 function could reverse the immunosuppressive nature of HCC and promote host immunity against HCC and improve clinical outcome.

At this time, early promising clinical data suggest that anti-CTLA-4 and anti-PD-L1/PD-1 antibody agents are active against HCC and may help improve response rates and survival.

In 20 patients with HCV-associated HCC (43% Child-Pugh classification B) treated with 15 mg/kg of intravenous tremelimumab every 90 days ([61] Sangro B, et al. J Hepatol. 2013), tremelimumab was generally well tolerated and no patients received systemic steroids No patients received systemic steroids and there were no deaths related to the study drug. The majority of patients had transient increases in transaminases after the first dose, 45% of which were Grade 3 or higher, but not accompanied by a concurrent decline in liver function. 17 responses could be evaluated, with 3 (17.6%) patients achieving a definite partial response (PR).

In another phase I/II study, 32 patients with unresectable advanced HCC (Child-Pugh classification A/B7) received two dose levels of tremelimumab (3.5 and 10 mg/kg IV Q4W) with partial ablation (RFA/TACE) during a 6-week treatment period ([62] Duffy AG,. et al. J Hepatol. 2017). Safety assessment results showed no clear trend in the occurrence of adverse events throughout the entire dose-specific cohort of tremelimumab, and no dose-limiting toxicities (DLTs) were observed in the study. The major Grade 3 or 4 adverse events were increased AST (21%), increased ALT (9%), and hyperbilirubinemia (9%); no Grade 3 or 4 diarrhea, colitis, or pneumonitis occurred; of the 19 patients with evaluable response outside the area directly treated with TACE/RFA, 5 (26.3%) had confirmed PR. The safety and efficacy of durvalumab (anti-PD-L1 antibody) monotherapy were investigated in a phase I/II study (CDON-MEDI4736-1108) in 40 patients with HCC, with an objective response rate (ORR) of 10.3% and a median OS of 13.2 months. Detailed safety and efficacy data from the study are described in Section 1.2.2.1. Similar results were obtained with another anti-PD-1 antibody, with an ORR of 18.6% and a median OS of 13.2 months in patients with advanced HCC ([63] Melero I, et al. J Clin Oncol. 2017).

Although the data from these trials are exploratory, they suggest that monotherapy with both durvalumab and tremelimumab has an antitumor effect against HCC. The combination of durvalumab plus tremelimumab in patients with unresectable HCC is being investigated in an ongoing phase I/II study (D4190C00022). In that study, patients with unresectable HCC who had not been previously treated with immunotherapy received the combination of durvalumab plus tremelimumab four times in Q4W followed by durvalumab alone in Q4W. Interim data from 40 patients followed for more than 16 weeks in the study showed an ORR of 18%. The combination of durvalumab plus tremelimumab was generally well tolerated in the population of patients with unresectable HCC. phase II results presented at ASCO 2020 reported promising results for durvalumab 1,500 mg Q4W plus tremelimumab 300 mg once. ORR was 24% and median OS was 18.73 months (95% CI: 10.78-27.27). It was reported to have the most favorable risk-benefit profile compared to the other dose arms ([64]Kelly RK, et al. J Clin Oncol. 2020).

The combined clinical evidence suggests that both durvalumab and tremelimumab have clinical activity as monotherapy in patients with HCC, and that combination therapy with both agents may provide an even greater antitumor effect in this patient population. A phase III, randomized, global study is underway to evaluate the safety and efficacy of these two agents in patients with HCC.

1.9. hypothesis

The purpose of this study is to confirm the synergistic effect of durvalumab-tremelimumab in

combination with heavy ion therapy in patients with advanced HCC. The study was designed based on the following two hypotheses

- Activation of tumor immunity by heavy ion therapy will be enhanced by immune checkpoint inhibitors.
- The control of primary lesions with MVI in patients with advanced HCC will have clinically significant results.

2. STUDTY OBJECTIVE

2.1. Objective

A phase Ib, open-label, uncontrolled study to evaluate the safety and efficacy of durvalumab-tremelimumab in combination with heavy ion therapy in patients with advanced hepatocellular carcinoma with MVI to assess safety and tolerability as measured by frequency of adverse events including DLT. Efficacy will be evaluated based on overall survival, 6-month survival, objective response rate, 6-month progression-free survival, and progression-free interval.

2.2. Study results regarding the appropriateness of conducting this clinical trial, efficacy, and safety for the subject disease

2.2.1. Durvalumab + tremelimumab combination therapy dose rationale

The durvalumab + tremelimumab doses and regimen selected for this study are based on the goal of selecting an optimal combination dose of durvalumab and tremelimumab that would yield sustained target suppression (sPD-L1), demonstrate promising efficacy, and have an acceptable safety profile.

2.2.2. Dose rationale for combination regimen of durvalumab 1500 mg Q4W plus tremelimumab 300 mg × 1 dose

A summary of the existing PK and pharmacodynamic data has been utilized to guide theregimen selection for the combination of durvalumab 1500 mg plus single dose of tremelimumab 300 mg.

Pharmacokinetics/Pharmacodynamics data

The supporting data for this regimen are based on PK and pharmacodyamic data from regimens that used tremelimumab doses of greater than 1 mg/kg from Study D4190C00006. An approximate dose-proportional increases in PK exposure (maximum serum concentration and area under the serum drug concentration-time curve from time 0 to Day 28 post-dose) was observed with increasing doses of tremelimumab (1, 3, and 10 mg/kg). An exploratory pharmacodynamic analysis bioanalytically evaluated the effects of tremelimumab on proliferating T-cells from NSCLC patients who received tremelimumab (1, 3, or 10 mg/kg) and durvalumab (15 or 20 mg/kg) combination treatment. Monotonic increases in pharmacodynamic activity with the combination (increased activation/ proliferation markers on CD4 and CD8 T-cells in periphery) were observed with increasing doses of tremelimumab (1, 3, 10 mg/kg). The peak increase (%) from baseline of CD4+Ki67+ T-cells was observed 8 days post administration, and the peak level was significantly increased ($p \leq 0.05$) as increasing dose of tremelimumab in the range of 1 to 10 mg/kg. Study data also suggested that higher peak exposure (maximum serum concentration [Cmax]) of tremelimumab is related to a higher maximum pharmacodynamic effect in the NSCLC patient population. Overall, the PK/pharmacodynamic data suggest that tremelimumab of dose greater than 1 mg/kg with a higher peak exposure may be associated with a higher pharmacodynamic effect.

Additionally, based on simulation data, the Cmax (78 μg/mL) post single dose administration of tremelimumab 4 mg/kg is approximately 4-fold higher than the predicted Cmax (19 μg/mL) post the first dose of tremelimumab 1 mg/kg, and is 3-fold higher than the predicted Cmax (25 μg/mL) post the fourth dose of tremelimumab 1 mg/kg in a Q4W×4 doses setting.

Clinical data

The safety and preliminary efficacy of combination of durvalumab 1500 mg plus single dose of tremelimumab 300 mg in unresectable HCC population is being evaluated in the ongoing Phase I/II study (Study D4190C00022).

In summary, a single dose of tremelimumab 4 mg/kg, while maintaining a similar overall

exposure, has a 3- to 4-fold higher Cmax compared to the 4 doses of tremelimumab 1 mg/kg. Therefore, this single administration of the higher dose of tremelimumab may have the potential for better anti-tumor activity while potentially avoiding any cumulative toxicity associated with repeated dosing of the 1 mg/kg tremelimumab. Therefore, the regimen of durvalumab 1500 mg plus tremelimumab 300 mg×1 dose is being evaluated in the current study.

Rationale for fixed dosing

A fixed-dose regimen of 1500 mg (equivalent to 20 mg/kg) of durvalumab plus 300 mg (equivalent to 4 mg/kg) of tremelimumab will be used in this study.

2.2.2.1. Rationale for utilizing a fixed-dose regimen for durvalumab and tremelimumab

A population PK model was developed for durvalumab using monotherapy data from a Phase I study (Study CD-ON-MEDI4736-1108; N=292; doses=0.1 to 10 mg/kg Q2W or 15 mg/kg Q3W; solid tumors). Similarly, a population PK model was developed for tremelimumab using data from Phase I through Phase III (N=654; doses=0.01 to 15 mg/kg Q4W or Q90D; metastatic melanoma) ([66]Wang et al 2014).

Population PK analysis indicated only minor impact of body weight on the PK of durvalumab and also tremelimumab (coefficient of ≤0.5). The weight-based versus fixed-dose (based on median weight of approximately 75 kg) regimens of both durvalumab and tremelimumab were compared using predicted PK concentrations (5th, 50th, and 95th percentiles) using a population PK model. A total of 1000 patients were simulated using weight distribution of 40 kg to 120 kg. Simulation results demonstrate that weight-based versus fixed dosing regimens of both durvalumab and tremelimumab yield similar median steady state PK concentrations with slightly less overall between-subject variability.

Similar findings have been reported by others ([22], [67] Narwal R, et al. Clin Pharmacokinet. 2013、[68] Ng CM, et al. Pharm Res. 2006, [69] Wang DD, et al. J Clin Pharmacol 2009, [70] Wolchok JD, et.al. N Engl J Med. 2013). Wang and colleagues investigated 12 monoclonal antibodies and found that fixed and body size-based dosing perform similarly, with fixed dosing being better for 7 of 12 antibodies ([21], [70] Wolchok JD, et al. N Engl J Med. 2013). In addition, they investigated 18 therapeutic proteins and peptides and showed that fixed dosing performed better for 12 of 18 in terms of reducing the between-subject variability in PK/pharmacodynamics parameters ([70] Wolchok JD, et al. N Engl J Med. 2013).

A fixed-dose approach is preferred by the prescribing community due to ease of use and reduced dosing errors. Given expectation of similar PK exposure and variability, we considered it feasible to switch to fixed-dose regimens. Based on the average body weight of 75 kg, a fixed dose of 1500 mg durvalumab (equivalent to 20 mg/kg) and a fixed dose of 300 mg tremelimumab (equivalent to 4 mg/kg) are selected for the current study. Therefore, the selected regimen of the durvalumab (+tremelimumab) cohort combined with particle therapy are:

【Cohort A】
Durvalumab monotherapy of 1500 mg Q4W

【Cohort B】
Durvalumab 1500 mg plus tremelimumab 300 mg for 1 dose, followed by durvalumab monotherapy 1500 mg Q4W

2.2.3 Rationale for carbon-ion radiotherapy

Historically, the role of radiation therapy in the treatment of liver tumors has been limited because of radiation-induced hepatic insufficiency caused by whole-liver irradiation ([71] Reed GB, et al. Am J Pathol, 1966). Particle therapy, including proton and carbon, overcame this problem with its physical dose distribution, enabling to treat large tumors while sparing normal liver tissue ([32], [72] Kanai et al. Int J Radiat Oncol Biol Phys, 1999). Results of CIRT have

been reported in multiple single institutional prospective studies and multi-institutional retrospective studies with high efficacy and mild toxicities ([34], [73] Shibuya K, et al. Liver Int. 2018, [74] Yasuda S, et al. Adv Radiat Oncol. 2019). Cases with MVI are also well treated with CIRT [35] Komatsu S, et al. J Gastroenterol. 2011). A single-arm clinical trial of CIRT in patients without large vessel or bile duct invasion is currently underway as an advanced medical treatment B, with the aim of developing a curative treatment for first-episode HCC that is not suitable for surgery (liver transplantation is not suitable) or radiofrequency ablation (jRCT1032200036).

Dose escalation studies have been conducted for 4 fraction CIRT ([34], [75]Shiba S, et al. Radiat Oncol. 2019). 60Gy (RBE) in 4 fractions appear to be well tolerated and multi-institutional retrospective study confirms its efficacy ([73] Shibuya K, et al. Liver Int. 2018). Constraints for each risk organ were determined based on previously published reports ([34], [73], [76] Shiba S, et al. Radiat Oncol 2020, [77] Makishima et al. Cancer Sci. 2018).

2.2.4 Rationale for combined treatment of carbon-ion radiotherapy and immunotherapy

In the PACIFIC trial, the combination of durvalumab and chemoradiotherapy (X-rays) has been shown to provide high therapeutic efficacy with intolerable toxicity, as discussed in 1.7 above ([41]Antonia SJ et al. NEJM. 2017). There are only ongoing trials of the combination of immune checkpoint inhibitors, including durvalumab, with particle therapy, and no reports exist as of November 2020. Trials of combinations with heavy particle therapy are similarly unregistered. As noted in 1.7 above, the results of basic research and clinical trials of combination therapy with X-rays suggest a higher synergistic effect.

2.3. Benefit-risk and ethical assessment

2.3.1. Potential benefits

2.3.1.1. Durvalumab monotherapy

Information on the potential benefit of durvalumab 1500 mg monotherapy or equivalent in patients with HCC are based on Study CD-ON-MEDI4736-1108 and are presented in Section 1.8.1. For other tumor types, see the most current durvalumab IB.

2.3.1.2. Durvalumab plus tremelimumab combination therapy

The potential benefits of adding tremelimumab to durvalumab is presented in Section 1.8.3. Information on the data supporting the selected combination regimen of durvalumab plus tremelimumab in patients with HCC are presented in Section 1.8.4. For other tumor types, see the most current durvalumab and tremelimumab IBs.

2.3.1.3. Durvalumab (+tremelimumab) combined with particle therapy

As mentioned in section 1.7, CIRT, as with traditional photon irradiation, is known to modify cancer immune reactions, but at a stronger level (Helm A, et al. Int J Part Ther. 2018, Ebner et al. Front Oncol. 2017). By combining CIRT with innunotherapy [durvalumab (+ tremelimumab)], there may be a larger improvement in efficacy compared to monotherapy of immunotherapy drugs.

2.3.2. Overall risks

Monoclonal antibodies directed against immune checkpoint proteins, such as programmed cell death ligand 1 (PD-L1) as well as those directed against programmed cell death-1 (PD-1) or cytotoxic T-lymphocyte antigen-4 (CTLA-4), aim to boost endogenous immune responses directed against tumor cells. By stimulating the immune system however, there is the potential for adverse effects on other tissues.

Most adverse drug reactions seen with the immune checkpoint inhibitor class of agents are thought to be due to the effects of inflammatory cells on specific tissues. These risks are generally

events with a potential inflammatory or immune mediated mechanism and which may require more frequent monitoring and/or unique interventions such as immunosuppressants and/or endocrine therapy. These immune mediated effects, can occur in nearly any organ system, and are most commonly seen as gastrointestinal AEs such as colitis and diarrhea, pneumonitis/interstitial lung disease (ILD), hepatic AEs such as hepatitis and liver enzyme elevations, skin events such as rash and dermatitis and endocrinopathies including hypo- and hyper-thyroidism.

2.3.2.1. Durvalumab

Risks with durvalumab include, but are not limited to, diarrhea/colitis, pneumonitis/ILD, endocrinopathies (hypo- and hyper-thyroidism, type I diabetes mellitus, hypophysitis and adrenal insufficiency) hepatitis/increases in transaminases, nephritis/increases in creatinine, pancreatitis/increases in amylase and lipase, rash/pruritus/dermatitis, myocarditis, myositis/polymyositis, other rare or less frequent inflammatory events including neurotoxicities, infusion-related reactions, hypersensitivity reactions and infections/serious infections.

For information on all identified and potential risks with durvalumab please always refer to the current version of the durvalumab IB.

In monotherapy clinical studies AEs (all grades) reported very commonly (≥ 15% of patients) are fatigue, nausea, decreased appetite, dyspnea, cough, constipation, diarrhea, vomiting, back pain, pyrexia, asthenia, anemia, arthralgia, peripheral edema, headache, rash, and pruritus. Approximately 9.4% of patients experienced an AE that resulted in permanent discontinuation of durvalumab and approximately 6.5% of patients experienced an SAE that was related to durvalumab by the study investigator.

Most treatment-related AEs were manageable with dose delays, symptomatic treatment, and in the case of events suspected to have an immune basis, the use of established treatment guidelines for immune-mediated toxicity.

A detailed summary of durvalumab monotherapy AE data can be found in the current version of the durvalumab IB.

2.3.2.2. Tremelimumab

Risks with tremelimumab monotherapy include, but are not limited to, GI effects (colitis, diarrhoea, enterocolitis and intestinal perforation), endocrine disorders (hypo and hyperthyroidism, hypophysitis and adrenal insufficiency), skin effects (rash, and pruritus), elevations in lipase and amylase and clinical manifestations of pancreatitis, other gastrointestinal events e.g. ulcerative colitis, dehydration, nausea and vomiting; hepatic events including hepatitis, and liver enzyme elevations; pneumonitis and ILD; nervous system events including encephalitis, peripheral motor and sensory neuropathies, Guillain-Barre and proximal muscle weakness; cytopenias including thrombocytopenia, anemia and neutropenia; infusion-related reactions, anaphylaxis, and allergic reactions; renal events including renal failure, acute kidney injury, nephritis, nephrotic syndrome, autoimmune nephritis and electrolyte abnormalities such as hypokalemia; autoimmune diseases including autoimmune arthritis, Sjogren's syndrome and giant cell temporal arteritis; hyperglycemia and diabetes mellitus; and pyrexia.

For information on all identified and potential risks with tremelimumab please always refer to the current version of the tremelimumab IB.

Using pooled data from monotherapy clinical studies AEs (all grades) reported very commonly (≥ 10% of patients) were diarrhea, nausea, fatigue, pruritus, decreased appetite, rash, vomiting, dyspnoea, constipation, cough, pyrexia, abdominal pain, decreased weight, headache, asthenia, and anaemia. Approximately 16% of patients experienced an AE that resulted in permanent discontinuation of tremelimumab and approximately 45% of patients experienced an SAE.

A detailed summary of tremelimumab monotherapy AE data can be found in the current version of the tremelimumab IB.

2.3.2.3. Durvalumab + tremelimumab

The safety of durvalumab + tremelimumab combination therapy was initially evaluated in the ongoing dose escalation and dose expansion Study 006, in patients with NSCLC, and is being studied in a number of other ongoing clinical trials, in a number of different indications, and has to date shown a manageable safety and tolerability profile.

The types of risks with the combination of durvalumab + tremelimumab (based on an equivalent durvalumab dose of 20m/kg and a tremelimumab dose of 1mg/kg) are similar to those for durvalumab and tremelimumab monotherapy. Emerging data from study 006, other studies evaluating the combination, and from combinations of other agents in the same class indicate an increased frequency and/or severity of some of these immune-mediated toxicities.

For information on all identified and potential risks with the durvalumab+tremelimumab combination please always refer to the current version of the durvalumab IB

In durvalumab+tremelimumab combination studies at the dose of durvalumab 20mg/kg and tremelimumab 1mg/kg AEs (all grades) reported very commonly (≥ 10% of patients) are fatigue, diarrhoea,nausea, dyspnea, decreased appetite, pruritus, vomiting, anaemia, constipation, cough, abdominal pain, pyrexia, back pain, arthralgia, hypothyroidism, asthenia, oedema peripheral, decreased weight, decreased hyponatraemia and rash.

Approximately 15% of patients experienced an AE that resulted in permanent discontinuation of study drug and approximately 16% of patients experienced an SAE that was considered to be related to durvalumab and tremelimumab by the study investigator.

A detailed summary of durvalumab + tremelimumab combination AE data can be found in the current version of the durvalumab IB.

2.3.2.4. Carbon-ion radiotherapy

Safety of CIRT for HCCs as a monotherapy are confirmed through multiple single institutional prospective studies and multi-institutional retrospective studies ([34], [73], [74]). Grade 3 or severe acute toxicities were seen in skin (1%) and ALT elevation (0.6%), with both being G3. G3 or severe late toxicities were seen in skin (G3 2%, G4 0.6%), hepatic coma (G3 1%), myositis and rib fracture (both G3 0.6%). RILD was seen in 2%. No treatment related deaths were observed.

Since CIRT is a form of radiotherapy, potential risks will be confined within the radiation field. The following may be observed.

- Bone marrow suppression
- Radiation pneumonitis, pleuritis, pleural effusion
- Nausea, vomiting, anorexia, diarrhoea, GI bleeding, ulceration, perforation, stricture
- Loss of hepatic function, hepatic failure, RILD, bile duct stricture, occlusion, cholangitis, aneurism
- Radiation dermatitis, ulceration
- Pericarditis, pericardial effusion, congestive heart failure, myocarditis, arrythmia
- Myelopathy, Peripheral neuropathy
- Rib fracture, subcutaneous induration, subsequent primary cancer, renal failure

2.3.2.5. durvalumab (+tremelimumab) combined with carbon-ion radiotherapy

Currently, there are no clinical trial results open yet for durvalumab + tremelimumab + radiotherapy for liver tumors, thus risks are unknown. Combination of immuno-oncology drugs and radiotherapy appear to have minimal excessive toxicity compared to immune-oncology drugs alone across multiple trials, including thoracic irradiation ([41] Antonia SJ, et al. NEJM 2018).

Potential risks would be toxicities closely related to immune reaction and microangiopathy, such as RILD, radiation pneumonitis and GI tract ulceration. In CIRT as a monotherapy, these toxicities are less common compared to photon radiotherapy, owing to the better dose distribution of CIRT as explained above ([34], [73], [74]). While the impact of difference in immune-modulation against toxicity between photon and carbon-ion is unknown, there is no definite evidence that excessive toxicity will be intolerable.

2.3.3. Overall benefit-risk

Durvalumab and tremelimumab have shown encouraging anti-tumor activity as single agents in advanced HCC population. The summary of this efficacy data is presented in Section 1.8.4. The combination regimen of these two agents shows a higher response rate in HCC population compared to either of the monotherapies. Thus, durvalumab plus tremelimumab combination therapy may potentially offer benefit to this patient population. Both durvalumab monotherapy and durvalumab plus tremelimumab combination therapy was tolerable in advanced HCC. The current study design aims to minimize potential risks by providing for early and intensive safety monitoring for any unexpected safety signals and for managing those risks deemed to be most likely based on prior experience with durvalumab, tremelimumab, and carbon-ion therapy. Two combination dose regimens of durvaluamb plus tremelimumab combination therapy were selected for this study with the aim to select the regimen with the most benefit for patients with advanced HCC.

HCC patients with MVI have very limited systemic therapeutic options and a poor life expectancy and health-related quality of life (HRQoL) based on the currently available treatments. While the main prognosticator in these patients is the intravascular growth of MVI, thus focal treatment by particle therapy has shown prolonged MST up to 2 years if lesions are confined in number ([40] Sugahara S, et al. Strahlether Onkol. 2009)). But those with extensive disease (ie multiple hepatic lesions and extrahepatic lesions) are currently not treated with particle therapy in fear of the out-of-treatment-field lesions. HCC patients with MVI, therefore, represents a significant unmet medical need and underlines the need for novel therapies for this patient population. CIRT combined with durvalumab or durvalumab plus tremelimumab proposed in this study may demonstrate a meaningful clinical benefit and a manageable safety profile. The overall benefit-risk profile of durvalumab (+tremelimumab) combined with CIRT is expected to be favorable, therefore supporting the current study design.

3. ELIGIBILITY

Each patient must meet all of the inclusion criteria (Section 3.2) and none of the exclusion criteria (Section 3.3) for this study. Under no circumstances will there be exceptions to this rule.

3.1. Diagnostic Criteria and Stage, Type, and Condition Classification

【CohortA & CohortB】

Eligible patients will be advanced HCC with MVI and Child-Pugh classification A over 20 years of age who are refractory or intolerant to standard systemic chemotherapy.

【Expansion cohort】

Eligible patients are advanced HCC with MVI, aged 20 years or older, with Child-Pugh classification A, with or without prior drug therapy.

3.2. Inclusion criteria

For inclusion in the study, patients should fulfill the following criteria:

1）Capable of giving signed informed consent which includes compliance with the requirements and restrictions listed in the informed consent form (ICF) and in this protocol. Written informed consent and any locally required authorization obtained from the patient/legal representative prior to performing any protocol-related procedures, including screening evaluations. For patients aged <20 years and enrolling, a written informed consent should be obtained from the patient and his or her legally acceptable representative.

2）Age >20 years at time of study entry

3）Eastern Cooperative Oncology Group (ECOG) performance status of 0 or 1

4）Body weight >30 kg

5）Adequate normal organ and marrow function as defined below:

- Haemoglobin ≧9.0 g/dL
- Absolute neutrophil count (ANC) ≧1,500/mm3
- Platelet count ≧75×109/L（≧75,000/mm3）
- Serum bilirubin ≦ULN×3.0
- AST ≦ULN×5.0
- ALT ≦ULN×5.0
- Measured creatinine clearance (CL) >40 mL/min or Calculated creatinine clearance CL>40 mL/min by the Cockcroft-Gault formula (Cockcroft and Gault 1976) or by 24-hour urine collection for determination of creatinine clearance

6）Evidence of post-menopausal status or negative urinary or serum pregnancy test for female pre-menopausal patients. Women will be considered post-menopausal if they have been amenorrheic for 12 months without an alternative medical cause. The following age-specific requirements apply:

- Women <50 years of age would be considered post-menopausal if they have been amenorrheic for 12 months or more following cessation of exogenous hormonal treatments and if they have luteinizing hormone and follicle-stimulating hormone levels in the post-menopausal range for the institution or underwent surgical sterilization (bilateral oophorectomy or hysterectomy).
- Women ≥50 years of age would be considered post-menopausal if they have been amenorrheic for 12 months or more following cessation of all exogenous hormonal treatments, had radiation-induced menopause with last menses >1 year ago, had chemotherapy-induced menopause with last menses >1 year ago, or underwent surgical sterilization (bilateral oophorectomy, bilateral salpingectomy or hysterectomy).

7）Patient is willing and able to comply with the protocol for the duration of the study including undergoing treatment and scheduled visits and examinations including follow up.

8）Advanced HCC confirmed histologically or by the typical findings of a hypervascular tumor

on computed tomography or angiography

9) (Cohort A and Cohort B only) Patients who have received at least one prior systemic chemotherapy regimen including atezolizumab bevacizumab combination, sorafenib, or lenvatinib and who are determined to be refractory or intolerant to standard therapy.

10) Must not be eligible for locoregional therapy for unresectable HCC. For patients who progressed after locoregional therapy for HCC, locoregional therapy must have been completed ≥28 days prior to the baseline scan for the current study. Acceptable locoregional therapy for HCC are Ethanol Infusion Therapy, Radio Wave ablation Therapy, Transcatheter Arterial chemoembolization (TACE), Transcatheter arterial infusion (TAI). Hepatic Arterial Infusion Chemotherapy (HAIC) is not allowed.

11) Patients who have been diagnosed with HCC showing MVI. MVI is defined as a tumor thrombus in the major hepatic and/or portal vein branches (Vp2, Vp3, Vp4, Vv2, and Vv3) identified by imaging studies.

12) Child-Pugh A

13) At least one measurable lesion other than the MVI and feeding nodule based on mRECIST.

3.3. Exclusion criteria

Patients should not enter the study if any of the following exclusion criteria are fulfilled:

1) Involvement in the planning and/or conduct of the study (applies to both sponser and/or staff at the study site)

2) Patients who have participated in another clinical trial using the investigational drug within 28 days prior to obtaining consent or who have received another investigational drug within 28 days prior to the first dose of the investigational drug in this study. The exception is if the patient is in the follow-up period of an interventional trial or is participating in an observational (non-interventional) clinical trial.

3) Any unresolved toxicity NCI CTCAE Grade ≥2 from previous anticancer therapy with the exception of alopecia, vitiligo, and the laboratory values defined in the inclusion criteria
   - Patients with Grade ≥2 neuropathy will be evaluated on a case-by-case basis after consultation with the Study Physician.
   - Patients with irreversible toxicity not reasonably expected to be exacerbated by treatment with durvalumab or tremelimumab may be included only after consultation with the Study Physician.

4) Radiotherapy treatment to more than 30% of the bone marrow or with a wide field of radiation within 4 weeks of the first dose of study drug

5) Major surgical procedure (as defined by the Investigator) within 28 days prior to the first dose of IP. Note: Local surgery of isolated lesions for palliative intent is acceptable.

6) History of allogenic organ transplantation.

7) Active or prior documented autoimmune or inflammatory disorders (including inflammatory bowel disease [e.g., colitis or Crohn's disease], diverticulitis [with the exception of diverticulosis], systemic lupus erythematosus, Sarcoidosis syndrome, or Wegener syndrome [granulomatosis with polyangiitis, Graves' disease, rheumatoid arthritis, hypophysitis, uveitis, etc.]). The following are exceptions to this criterion:
   - Patients with vitiligo or alopecia
   - Patients with hypothyroidism (e.g., following Hashimoto syndrome) stable on hormone replacement
   - Any chronic skin condition that does not require systemic therapy
   - Patients without active disease in the last 5 years may be included but only after consultation with the study physician
   - Patients with celiac disease controlled by diet alone

8) Uncontrolled intercurrent illness, including but not limited to, ongoing or active infection, symptomatic congestive heart failure, uncontrolled hypertension, unstable angina pectoris,

cardiac arrhythmia, interstitial lung disease, serious chronic gastrointestinal conditions associated with diarrhea, or psychiatric illness/social situations that would limit compliance with study requirement, substantially increase risk of incurring AEs or compromise the ability of the patient to give written informed consent

9) History of another primary malignancy except for
   - Malignancy treated with curative intent and with no known active disease ≥5 years before the first dose of IP and of low potential risk for recurrence
   - Adequately treated non-melanoma skin cancer or lentigo maligna without evidence of disease
   - Adequately treated carcinoma in situ without evidence of disease
   - However, the following cases are eligible for enrollment
   - Early stage cancer (epithelial cancer of the cervix, basal cell carcinoma, superficial bladder cancer (Tis and T1), early stage gastric cancer, and early stage colorectal cancer) that has been treated for curative purposes, has not been confirmed active for at least 3 years prior to inclusion in the study, and has a low risk of recurrence.

10) History of leptomeningeal carcinomatosis

11) History of, or current, brain metastases or spinal cord compression. Patients with suspected brain metastases at screening should have an MRI (preferred) or CT, each preferably with IV contrast of the brain prior to study entry.

12) Mean QT interval corrected for heart rate using Fridericia's formula (QTcF) ≥470 ms calculated from 3 ECGs (within 15 minutes at 5 minutes apart) Regardless of whether this criteria stays or not, all patients should have a baseline ECG

13) History of active primary immunodeficiency

14) Patients co-infected with HBV and HCV, or co-infected with HBV and hepatitis D virus (HDV). HBV positive (presence of HBsAg and/or anti-HBcAb with detectable HBV DNA); HCV positive (presence of anti-HCV antibodies); HDV positive (presence of anti-HDV antibodies), and active infection including tuberculosis (clinical evaluation that includes clinical history, physical examination and radiographic findings, and TB testing in line with local practice).

15) Current or prior use of immunosuppressive medication within 14 days before the first dose of durvalumab or tremelimumab. The following are exceptions to this criterion:
   - Intranasal, inhaled, topical steroids, or local steroid injections (e.g., intra articular injection)
   - Systemic corticosteroids at physiologic doses not to exceed 10 mg/day of prednisone or its equivalent
   - Steroids as premedication for hypersensitivity reactions (e.g., CT scan premedication)

16) Receipt of live attenuated vaccine within 30 days prior to the first dose of IP. Note: Patients, if enrolled, should not receive live vaccine whilst receiving IP and up to 30 days after the last dose of IP.

17) Female patients who are pregnant or breastfeeding or male or female patients of reproductive potential who are not willing to employ effective birth control from screening to 90 days after the last dose of durvalumab monotherapy or180 days after the last dose of durvalumab + tremelimumab combination therapy.

18) Known allergy or hypersensitivity to any of the study drugs or any of the study drug excipients.

19) Prior randomisation or treatment in a previous durvalumab and/or tremelimumab clinical study regardless of treatment arm assignment.

20) Judgment by the investigator that the patient is unsuitable to participate in the study and the patient is unlikely to comply with study procedures, restrictions and requirements.

21) Patients who have been treated with anti-PD-1, anti-PD-L1 inhibitors, or other drugs that act on other stimulatory or co-suppressive T-cell receptors and their combinations (including atezolizumab plus bevacizumab) and have failed to tolerate the same treatment.

22) Prior radiotherapy involving the liver.

23） Renal failure requiring hemodialysis or peritoneal dialysis
24） Any of the following cardiac diseases:
- NYHA Class III or IV chronic heart failure
- Current coronary artery disease or history of ischemic heart disease such as myocardial infarction within 6 months before the study
- Serious arrhythmia (grade 3 or higher according to the CTCAE ver. 5.0: arrhythmia that cannot be controlled by oral medications or requires mechanical control).

25） Poorly controlled hypertension
26） Serious and active infection, excluding hepatitis viral infection
27） Persistent proteinuria of NCI-CTCAE version 5.0 grade 3 or higher.
28） Arterial or venous thrombotic or embolic events such as cerebrovascular accident, deep vein thrombosis, or pulmonary embolism within 6 months before the start of study medication.
29） Refractory pleural effusion or ascites
30） History of hepatic encephalopathy within past 12 months
31） Oral intake impossible
32） HIV-positive
33） Pulmonary fibrosis or interstitial pneumonitis
34） Other serious complications as follows: serious mental disease or history of gastrointestinal bleeding or active hemoptysis
35） Unsatisfactory general condition for participation in the study as judged by the primary physician

4. Informed consent

4.1. Preparation and revision of informed consent form

The investigator prepares the consent form and other information documents used to obtain consent for participation in the clinical trial from the subject in plain language as much as possible. If it is considered necessary to revise the consent document and other explanatory documents, the investigator revises these documents.

The investigator submits the prepared or revised consent documents and other explanatory documents to the IRB for approval.

Amendments to the study protocol and informed consent forms will follow the below procedures:

1. When amendments are considered to be necessary, the Principal Investigator will provide to the Investigator(s) the study protocol amendment draft, informed consent form amendment drafts, and the latest version of the investigator's brochure and other necessary material/information.
2. The Principal Investigator will provide the Investigator(s) with necessary time to adequately consider the aforementioned study protocol amendment draft and material/information and discuss the details with the Principal Investigator.
3. After discussion with the Principal Investigator, the Investigator(s) will promptly submit the amended version of the study protocol or informed consent form to the head of the trial site, and receive approval of the IRB via the head of the trial site.
4. Within acceptable limits of the Principal Investigator, the same procedures will apply to amendments to be made to the study protocol and informed consent form according to instructions given by the head of the trial site based the opinions of the IRB.

4.2. Method of Obtaining Informed Consent

1) Informed consent

The investigator or subinvestigator should hand the consent document and other explanatory documents to the subject and provide sufficient explanation of the contents as indicated in "4.3". If necessary, the clinical trial coordinator also provides supplementary explanations to the subject. After confirming that the patient has a good understanding of the contents of the clinical trial, the subject's signed and dated informed consent should be obtained before the pre-study (screening) test is conducted.

2) When explaining to subjects

The investigator or sub-investigator shall give the subject the opportunity to ask questions and sufficient time to decide whether or not to participate in the trial before obtaining informed consent and shall answer the subject's questions to the subject's satisfaction.

3) Signing and delivery of consent form

The investigator or subinvestigator who provided the explanation should sign the consent form with the date of the explanation. The subject signs the consent form with the date of consent. If a collaborator provides supplementary explanation, the collaborator should also sign and enter the date of the explanation. After obtaining informed consent, a copy of the information document and the consent form shall be given to the subject.

4) Amendments to informed consent forms

When the investigator or subinvestigator revises the informed consent form or other explanatory documents due to the acquisition of new information that may be relevant to the subject's consent, the investigator or subinvestigator shall explain to the subject again using the revised informed consent form and other explanatory documents, and obtain consent in writing for the subject's continued participation in the clinical trial. If new important information is obtained that may affect the subject's consent, the information shall be immediately provided to the subject, recorded in writing, and the subject's continued participation in the clinical trial shall be confirmed.

4.3. Information to be provided to subjects

The informed consent form to be prepared by the investigator shall include the following information.

1. What is a clinical trial?
2. The purpose of the clinical trial
3. Name, title and contact information of the investigator
4. Method of the clinical trial
5. Anticipated clinical benefits and risks or inconveniences
6. Availability of other treatment options for the subject and the expected important benefits and risks associated with such treatment options
7. The expected duration of the subject's participation in the clinical trial
8. That participation in the clinical trial is of the subject's own free will and that the subject may refuse or withdraw from participation in the clinical trial at any time. Furthermore, the subject shall not be treated unfavorably due to refusal or withdrawal, and shall not lose any benefits that he/she would have received if he/she had not participated in the clinical trial.
9. Monitors, auditors, clinical trial review committees, and regulatory authorities must be able to view source documents related to medical care. In such cases, the confidentiality of the subject shall be maintained. In addition, the subject's signature on the consent document shall be considered as authorization for access.
10. Subjects' confidentiality shall be maintained even if the results of the clinical trial are made public.
11. Compensation and treatment to which subjects are entitled in the event of adverse health effects related to the clinical trial.
12. Information that may influence the subject's decision to continue participation in the clinical trial will be promptly communicated to the subject.
13. Conditions or reasons for discontinuation of participation in the clinical trial
14. Expenses to be borne by the subject in relation to the clinical trial
15. Details of any financial or other payments to be made to the subject in connection with the clinical trial (e.g., arrangements for calculating the amount to be paid)
16. The medical institution's contact person to whom subjects should refer or contact if they require further information regarding the clinical trial and their rights or if they experience any health problems related to the clinical trial.
17. Items to be observed by the subject
18. Types of investigational review committees that will investigate and deliberate on the appropriateness of the clinical trial, matters to be investigated and deliberated by each investigational review committee, and other matters related to the investigational review committee for the clinical trial in question
19. Intellectual property
20. Conflicts of interest

5. STUDY DESIGN

5.1. Overview of study design

This is a Phase Ib study to assess the safety of durvalumab combined with particle therapy (Cohort A) and durvalumab plus tremelimumab combined with particle therapy (Cohort B) in advanced hepatocellular carcinoma patients with macrovascular invasion. This study consists of four periods: the screening period, DLT assessment period, durvalumab q4W dosing period, and follow up period. After the signed informed consent is obtained and the screening is conducted, the patient will be registered for enrollment in the trial. Patients will be administered with the first IP followed by administration of CIRT.

DLT assessment period is for 42days starting from the first dose of durvalumab on Day1 of Cycle1. The first administration of continuous durvalumab q4W on Day 1 of Cycle 2 starts only after the safety of Cycle 1 was confirmed (durvalumab q4W: 28 day cycle).

DLT analysis will be made when more than one DLT was observed in each cohort.

Patients will to continue to receive durvalumab every 4 week after completion of the DLT assessment period until clinical progression/withdrawal from the trial if there may be potential clinical benefit at the investigator’s discretion.

Follow up visit will be made 28 days after study termination due to PD or withdrawal from the study. Safety information will be collected until 90 days after the last dose of study treatment or until initiation of alternative anticancer therapy. In this study, three patients are initially enrolled into cohort A. If there is no DLT observed in any of these subjects, the trial proceeds to enroll additional subjects into the cohort B, whose regimen does not contain higher dose of durvalumab but contains an additional drug of tremelimumab instead. If one subject develops a DLT at cohort A or cohort B, an additional three subjects are enrolled into that same cohort. Development of DLTs in more than 1 of 6 subjects in either cohort suggests that the regimen is not tolerable. If cohort A turns out to be intolerable, then cohort B regimen will not be pursued The evaluation of DLTs shall be performed by the investigator of Chiba University Hospital in consultation with the investigator(s). The DLTs determined shall be discussed with the Independent Data Monitoring Committee and their opinion shall be sought in accordance with the standards separately stipulated (10.7. Independent Data Monitoring Committee).

Duration of DLT assessment is defined for 42 days starting from the first administration of IP on Day1 of Cycle 1. Dose of durvalumab is fixed on 1500 mg. CIRT will be performed between Day 8 to Day 14 of Cycle 1 after the first duvalumab administration on Day1 (CIRT within 14 days after 1stcycle of durvalumab).

In both cohorts, if the investigators determined that there may be potential clinical benefit, patients will to continue to receive durvalumab every 4 week until clinical progression (Durvalumab q4W dosing period).

- **Cohort A**: Durvalumab 1500mg will be administered every 4 weeks in principle. Particle therapy, in form of CIRT, will be performed after Day8 of Cycle1 following the 1st dose of Durvalumab on Day1. 2nd cycle of durvalumab will be administrated only after the safety during DLT assessment was confirmed.

- **Cohort B**: Durvalumab 1500mg will be administered every 4 weeks in principle, and Tremelimumab 300mg will be administered only on Day1 of Cycle1. Particle therapy, in form of CIRT, will be performed after Day8 of Cycle1 following 1st cycle of Durvalumab + Tremelimumab. 2nd cycle of durvalumab will be administrated only after the safety during DLT assessment was confirmed.

CIRT will be given to both arms. Dose prescription and fractionations will be 60Gy (RBE) / 4Fr /

1week. Target lesion of the particle therapy will be focused on intrahepatic nodule with MVI. A 1cm margin will be taken as a clinical target volume margin for the feeding nodule, and 2cm margin alongside the vessel for the MVI lesion. Internal motion will be compensated according to 4D-CT movement assessment. Inter-fractional margin will be set to 3mm and combined with internal motion compensation forming a field specific planning treatment volume. Dose constraints for OARs will be prioritized over target volume coverage.

If both of Cohort A and B regimens were confirmed tolerable after DLT assessment, additional patients will be enrolled in Cohort B up to a total of 15 subjects. If only Cohort A regimen was determined to be tolerable, additional patients may be enrolled in Cohort A up to a total of 15 subjects.

Schedule for administration of investigational drugs and carbon ion radio therapy（Figure1）

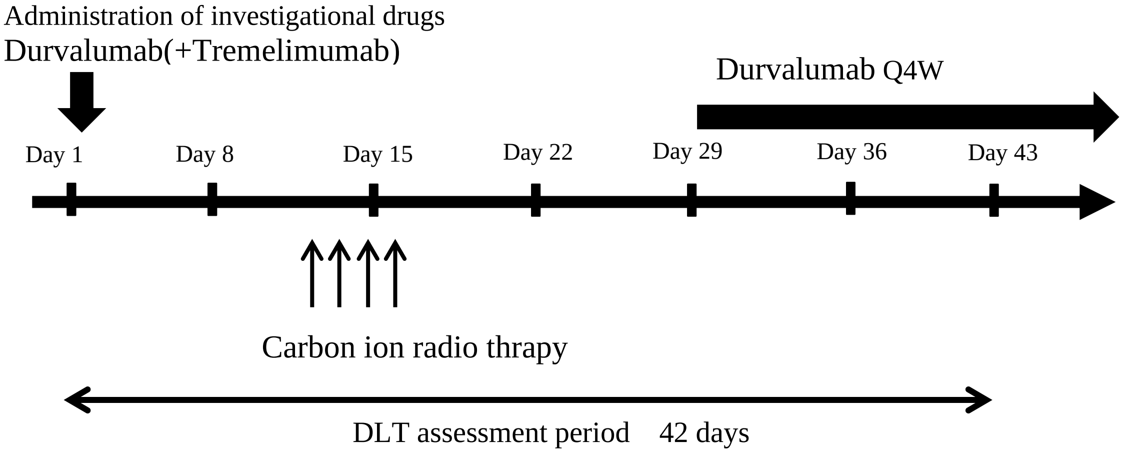

Tumor assessments, based on RECIST v.1.1, mRECIST and irRECIST, will be performed every 6 weeks (Q6W) (±1 week) for the first 12 weeks from the date of randomization and then Q8W (±1 week) thereafter until RECIST 1.1-defined radiological progression followed by a subsequent scan if clinically feasible, evaluated by Confirmation of Radiological Progression criteria (Appendix B). Patients who permanently discontinue study drug(s) for reasons other than PD should continue to have radiographic scans performed per their original schedule until confirmed PD

Subjects with rapid tumor progression or tumor-associated syndromes requiring urgent medical intervention (e.g., central nervous system metastases, respiratory failure due to tumor compression, spinal cord compression) will be deemed ineligible for continued durvalumab.

The overview of this clinical trial is as follows.

Cohort A

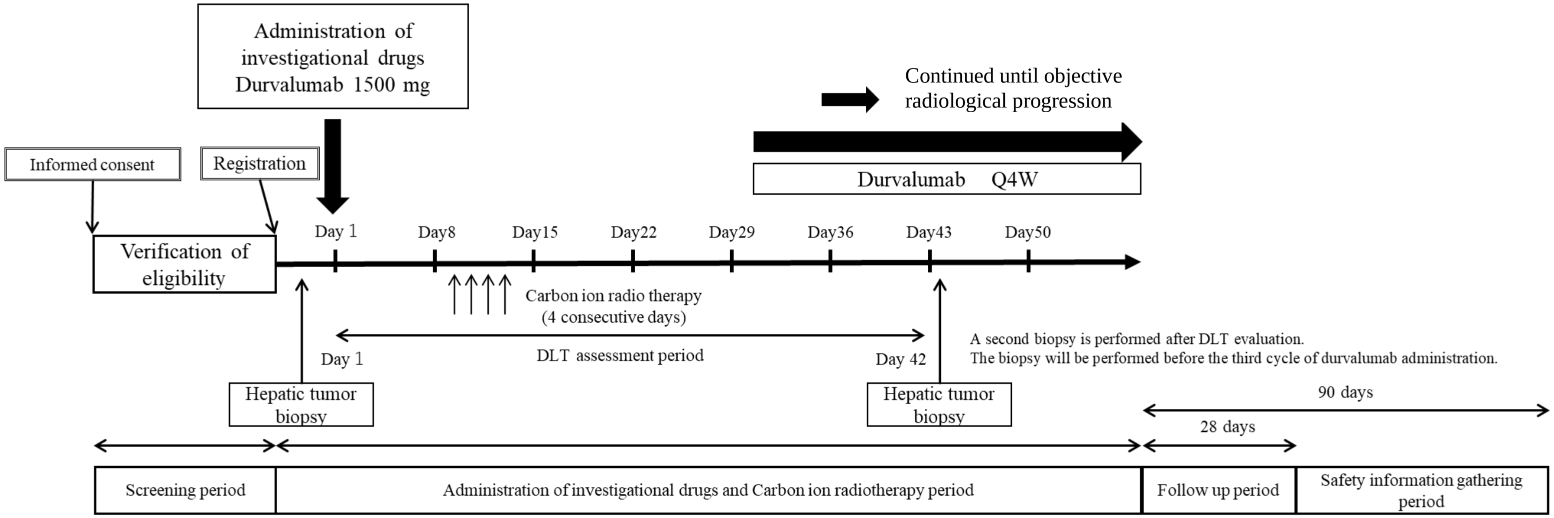

Cohort B

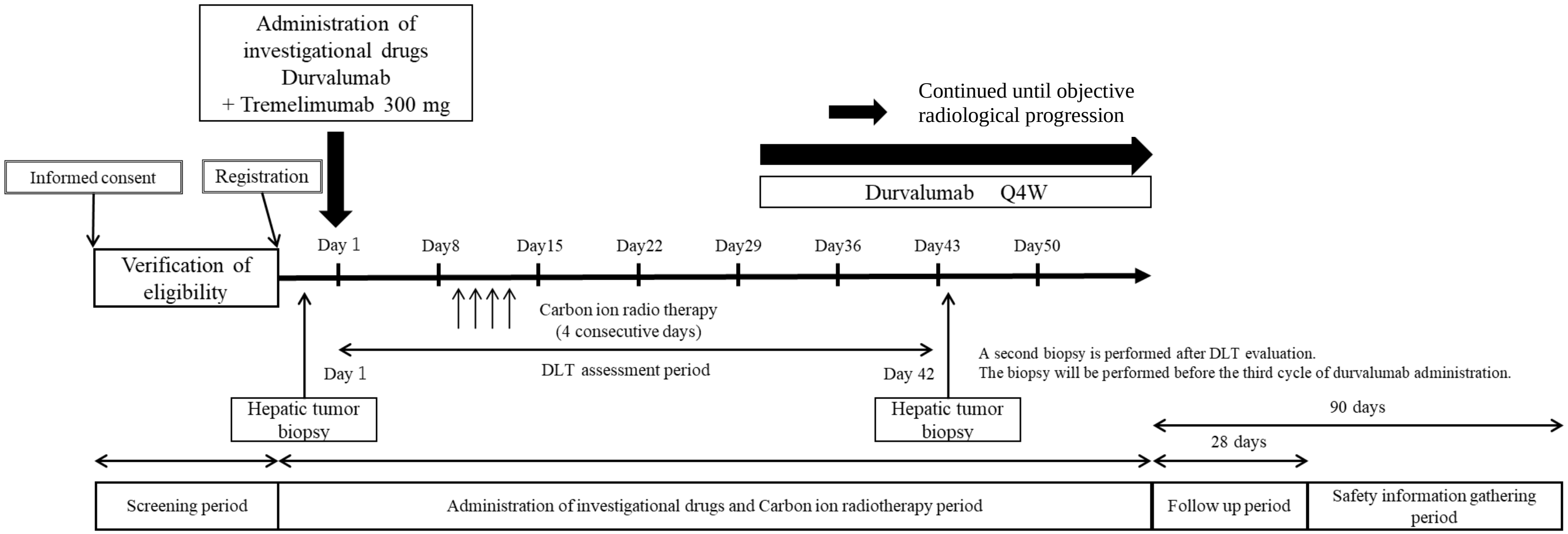

5.2. Target number of subjects and study duration

Target number of subjects：15

Study Period：
Estimated study start date Apr 2021
Estimated study completion date Dec 2022
Subject registration period:
Estimated date of first patient enrolled Apr 2021
Estimated date of last patient enrolled Jun 2022

Study schema

This study consists of four periods: the screening period, DLT assessment period, durvalumab q4W dosing period, and follow up period.

Signed informed consent will be obtained and patients will be screened prior to enrollment. The investigational treatment will include carbon ion radiotherapy on or after day 8 of cycle 1 after the first dose of study drug; the DLT evaluation period is 42 days from the first dose of durvalumab on day 1 of cycle 1. After confirmation of the safety of Cycle 1, durvalumab will be administered sequentially in Q4W beginning on Day 1 of Cycle 2. If multiple DLTs are observed in each cohort, an independent data monitoring committee will be convened to provide input on the determination of intolerability.

If no DLT is observed after the DLT evaluation period, durvalumab will be continued every 4 weeks until objective confirmation of disease progression (7.4) or until the criteria for 5.6 are met. Post-study follow-up for individual cases will occur 28 days after study completion. Adverse events and serious adverse event outcomes in subjects will be collected 90 days after the last dose of study treatment or until initiation of alternative anticancer therapy.

Figure2.

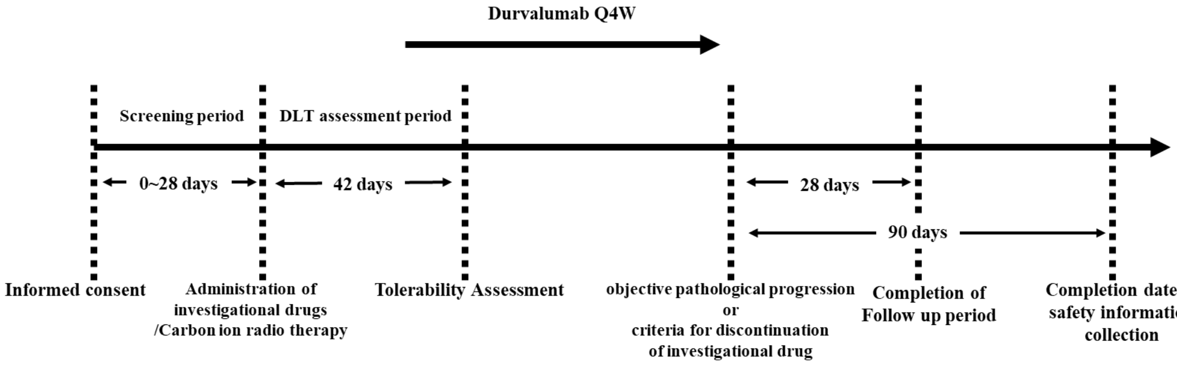

5.3. Monitoring for safety assessment

In situations where the below information is obtained and study patients are placed at under risk, or the continuation of the clinical trial is determined to be difficult, the Principal Investigator may decide on the termination or suspension of the entire clinical trial upon discussion with the Investigators. In addition, the study may be stopped based on the opinions by the DMC.

1. Occurrence of an unpredictable serious adverse reaction
2. Any information that indicates that the number, frequency, and condition of predictable serious adverse reactions cannot be predicted from the investigator’s brochure
3. SAEs that have been determined to have no causal relations with the IP, but are later determined that there is a reasonable possibility, due to the number, frequency, and condition of occurrences

4. Research reports indicating the tendency of the number, frequency, and condition of occurrences of adverse reactions having changed drastically
5. Research reports indicating the possibility that cancer, other serious diseases, disabilities, or death may occur
6. Information indicating that efficacy of the study drug cannot be expected in this clinical trial
7. Information indicating that the IP does not have any efficacy or effect on the target disease of the clinical trial
8. Information on any of the following related to marketed drugs that include the same ingredients as the IP:
    - Termination of manufacturing, import, or retail
    - Collection or disposal
    - Any other measures taken against health and hygiene related risks

Regardless of the reason for discontinuation, all data available to the subject at the time of discontinuation must be documented in the eCRF. All reasons for discontinuation should be documented. In terminating a trial, the investigator ensures that the protection of the subject's interests is given due consideration.

5.4. Institutional and case registration methods

Site registration and case registration will be conducted under the central registration system at the Data Management Office, Department of Clinical Trials, Chiba University Hospital. Once the site registration is completed, case registration will be available from the site. The following procedures will be used for site registration by fax and case registration via the Web.

5.4.1. Facility registration

1) The investigator at each participating site shall send a copy of the approval letter from the investigational review committee and a request form for site registration to the site registration center by fax after approval is obtained from the investigational review committee at the site.
2) The site registration center registers the sites and sends a notification of completion of site registration to the investigator.

Registration Center (Department of Data Management, Department of Clinical Trials, Chiba University Hospital)<br>fax number: 043-222-1207<br>Tel：043-222-1206<br>※Open hours are Monday through Friday, 9:00 a.m. to 5:00 p.m. (except Saturdays, Sundays, national holidays, and year-end and New Year holidays)<br>If a fax is received outside of the receptionist's office hours, it will be accepted on the next working day. (except at the beginning of the New Year)

5.4.2. Subject resistration

1) The investigator or subinvestigator obtains written consent and registers the subject in the case registration system. The case registration will be done via the website. After registration, a screening test is conducted to confirm that subjects meet the selection criteria and do not violate the exclusion criteria.
2) The investigator, subinvestigator or collaborator accesses the designated URL and enters the information necessary for case registration on the website. The investigator or sub investigator confirms the eligibility determination on the screen, and if the subject is

determined to be eligible, protocol treatment is initiated. Once a subject is enrolled, enrollment will not be cancelled.

＊ The investigator or sub-investigator shall not administer the investigational drug until the subject is enrolled and "eligible" by screening test.

Registration Web site（DATATRAK Enterprise Cloud）<br>URL：https://secure.datatrak.net<br>TEL：043-222-1206<br>※Open 365 days a year, 24 hours a day, including Saturdays, Sundays, and holidays (except for system downtime due to maintenance checks, etc.)

5.4.3. Handling of subjects who are found to be ineligible after enrollment

Subjects who do not meet "eligibility" for any reason, such as ineligibility, on the post-enrollment screening test are not eligible for enrollment and administration of the investigational product. The investigator or sub-investigator will explain to the subject that he/she is not eligible for enrollment in the study. The investigator or sub-investigator will also record the reason for the subject's ineligibility in the source documents.

If a subject is enrolled as a subject, but it is later determined that the subject does not meet all eligibility criteria, the subject will not be assigned and administered the investigational product and will be terminated from the clinical trial. If a subject who does not meet all eligibility criteria is inadvertently enrolled or started on the investigational product, the investigator will discuss whether or not to continue the investigational product. The investigator will ensure that all decisions resulting from this discussion are properly documented. If consensus cannot be reached, administration of the investigational product to the subject will be discontinued.

5.5. Dosing schedule and dosage/administration method

- Durvalumab 1,500 mg single agent arm (Cohort A)

  Cycle 1 Durvalumab 1,500 mg intravenous infusion will start on Day 1; after the first dose of durvalumab, heavy ion therapy will be administered on or after Day 8 of Cycle 1. Cycle 2 durvalumab will be administered after the safety of Cycle 1 is confirmed. Thereafter, dosing will be continued every 4 weeks until objective disease progression is confirmed (7.4.), "5.6. Discontinuation of Investigational Drug" is met, and the study is terminated.

- Durvalumab 1,500 mg + tremelimumab 300 mg once combination therapy (Cohort B)

  Cycle 1 One dose of combination therapy with durvalumab 1,500 mg and tremelimumab (both intravenous infusions) will be administered starting on Day 1. Durvalumab infusion will be started approximately 1 hour (maximum 2 hours) after completion of tremelimumab administration; after the first dose of durvalumab plus tremelimumab, heavy ion therapy will be administered on or after Day 8 of Cycle 1. Begin durvalumab 1,500 mg monotherapy Q4W after Cycle 2. Cycle 2 durvalumab will be administered after the safety of Cycle 1 is confirmed. Thereafter, durvalumab will be administered every 4 weeks until the objective disease progression is confirmed (7.4.), "5.6. Discontinuation of Investigational New Drug" is met, and the study is terminated.

5.5.1. Criterion for reduction

No dose reductions will be made for either durvalumab or tremelimumab in this study.

5.6. Discontinuation of Investigational Drug

If any of the following criteria are met, the investigator or sub-investigator will discontinue the investigational product and perform an Investigational Product Discontinuation Study. A follow-up visit will be scheduled 28 days after the last dose of investigational product.

1) When a subject requests to withdraw from the clinical trial treatment.
2) When it is difficult to continue administration of the investigational drug due to exacerbation of complications
3) When it is difficult to continue administration of the investigational drug due to adverse events.
4) Pregnancy is detected.
5) Other cases in which the investigator or subinvestigator judges it necessary to discontinue the administration of the investigational drug.

Subjects may discontinue investigational treatment at any time, at their own discretion, without prejudice to subsequent treatment. Subjects who decide to discontinue an investigational drug should always be questioned about the reason for discontinuation and the presence or absence of AEs. Subjects who completely discontinue subsequent doses of study medication, regardless of the reason, must continue to attend the clinic for evaluation according to the study protocol. If the subject does not agree to continue the visit, the follow-up procedure may be modified to ensure collection of endpoints and safety information. This follow-up may include telephone contact with the subject, contact with relatives or the treating physician, or information from medical records. Any change in the method of follow-up should be documented in the medical record. Subjects who agree to the change in follow-up are not considered to have withdrawn their consent or to have withdrawn from the trial.

Subjects who discontinue continued treatment with the investigational drug for any reason will be identified as treatment discontinuation. Subjects who discontinue treatment will be transferred to the follow-up period (refer to 7.1.).

Subjects who discontinue treatment for reasons other than objective tumor progression as assessed by imaging will continue to receive imaging every 6 weeks (±1 week) until 12 weeks after study drug initiation, then radiologic PD with imaging every 8 weeks (±1 week), plus additional imaging as defined in the study plan, or death (whichever occurs first), RECIST assessment will continue.

If a subject is determined to have PD as defined by RECIST 1.1, additional imaging studies should be performed within 4 weeks of the determination. (refer to 7.4.).

All subjects will be followed for survival until the end of the trial. Subjects who are unable to return for evaluation will be contacted by telephone as indicated in the trial schedule as an alternative.

5.7. Discontinuation of individual cases from participation in clinical trials

If any of the following criteria are met, the investigator or subinvestigator will discontinue administration of the investigational drug and the subject's participation in the clinical trial.

1) When it is judged difficult to continue the clinical trial for some reason on the subject's side, such as non-attendance or transfer to another hospital.
2) 2When the subject requests to discontinue participation in the clinical trial.
3) When the investigator or subinvestigator determines that the subject is unable to continue the clinical trial.
4) If the subject weighs less than 30 kg
5) When the investigator/divisional investigator determines that a decision to discontinue the study is necessary due to a serious violation of the study protocol, etc.

5.7.1. In case of untraceable cases

A subject is considered lost only if the subject cannot be contacted until the end of the trial and there is insufficient information to determine the subject's status at that time. Subjects who refuse to continue participation in the trial (including telephone contact) will be recorded as "withdrawing consent" rather than "untraceable". Investigators will document the means used to re-establish contact with subjects lost to follow-up throughout the duration of the trial. If the subject who was lost to follow-up is re-contacted, the subject will not be marked as "lost to follow-up" and the evaluation will resume according to the study protocol.

At the time of the OS analysis, the survival status of all subjects in the overall analysis population (FAS) and the safety analysis population will be reconfirmed. Subjects who withdrew consent and subjects classified as "possibly untraceable" will also be subject to this reconfirmation.

5.7.2. Withdrawal of consent

The subject is free to withdraw consent for this clinical trial at any time without prejudice to subsequent treatment.

Subjects who withdraw their consent will not receive any further doses of the investigational drug or follow-up as specified in the protocol. However, consent for survival follow-up will be confirmed separately. Additional tests may be performed after discontinuation to ensure subject safety.

If a subject withdraws consent, the investigator must confirm the reason and the presence or absence of adverse events.

The subject withdrawing consent shall be specifically asked about the following items regarding the details of the withdrawal of consent.

- Withdraw consent for all further participation in the clinical trial, including subsequent follow-up (e.g., telephone calls to investigate survival status).
- Withdraw consent for use of clinical trial data.
- Withdraw consent for the use of any sample.

5.7.3. Clinical investigator's decision

If the risks of participation in a clinical trial outweigh the benefits of the subject's participation in the trial, such as if a life-threatening infusion reaction or systemic infection occurs, the investigator will determine that the subject's participation in the trial cannot continue.

5.7.4. Subject weight loss

If the subject weighs less than 30 kg after enrollment, he/she will be removed from the study.

5.7.5. Other cases

If the investigator determines that the subject is unable to continue participation in the study for other reasons, such as a serious deviation from the protocol, the subject will be removed from the study.

5.8. Subject replacement

If a subject is found to be ineligible for the clinical trial prior to enrollment, the subject will not be enrolled in this trial. In such cases, the investigator will explain to the subject that enrollment in this trial is not possible.

For subjects who do not complete the study treatment for reasons other than DLT criteria during the DLT evaluation period, the investigator will seek input from the Data Monitoring Committee on whether to recruit a new subject to replace that subject.

5.9. Concomitant Restricted Drugs and Concomitant Restricted Therapy

The coordinating investigator must be informed of all medications taken from the time of screening until the end of the clinical phase (last visit) as soon as possible. All concomitant medications, including herbal medications taken during the trial are recorded in the CRF.

Restricted, prohibited, and permitted concomitant medications are listed in Tables 1 and 2.

Table1. 5.9. Concomitant Restricted Drugs and Concomitant Restricted Therapy

**Prohibited drug**	**Rules of use**
Anticancer drugs as investigational drugs other than the investigational drugs in this study	Concurrent use is prohibited during administration of investigational drugs.
mAb against CTLA-4, PD-1 or PD-L1 other than the investigational drug mAb for PD-L1 in this study	Concurrent use is prohibited during administration of investigational drugs.
Any concomitant chemotherapy, radiation therapy, immunotherapy, biologic therapy, or hormonal therapy for the treatment of cancer other than the investigational drug in this study	Any concomitant chemotherapy, radiation therapy, immunotherapy, biologic therapy, or hormonal therapy for the treatment of cancer other than the investigational drug in this study
Immunosuppressive agents such as systemic corticosteroids, methotrexate, azathioprine, or tumor necrosis factor alpha inhibitors at doses of prednisone or its equivalent greater than 10 mg/day<br>Immunosuppressive agents such as systemic corticosteroids, methotrexate, azathioprine, or tumor necrosis factor alpha inhibitors in doses greater than 10 mg/day. Immunosuppressive agents such as, but not limited to, the following	Concomitant administration or premedication is prohibited. The following exceptions are permitted<br>・ Use of immunosuppressive agents for the management of adverse events related to the investigational drug<br>・ Use in subjects allergic to contrast media<br>・ Use of inhaled, topical, and intranasal corticosteroids<br>・ Non-immunotherapy that is clinically necessary and has occurred in the subject<br>・ Temporary use of steroids is acceptable if deemed essential for the management of related events (e.g., chronic obstructive pulmonary disease, radiation therapy, nausea)
Epidermal Growth Factor Receptor Tyrosine Kinase Inhibitors（Epidermal Growth Factor Receptor Tyrosine Kinase Inhibitors (EGFR TKIs)	Prohibit concomitant use.<br>Use with caution for 90 days after the last dose of durvalumab. An increased incidence of pulmonary inflammation (in combination with a third-generation EGFR TKI) and an increased incidence of transaminases (in combination with a first-generation EGFR TKI) have been reported when durvalumab is used concomitantly.
Attenuated live vaccine	Concomitant use of the investigational drug is prohibited until 30 days after the last dose of the investigational drug.
Drugs with laxative action and herbs or natural remedies for constipation	Concomitant use is prohibited.
Blood transfusion (red blood cell concentrate, platelets)	Concomitant use is prohibited during the DLT evaluation period.
Granulocyte colony-forming stimulating factor (G-CSF) preparation	Concomitant use is prohibited during the DLT evaluation period.

Table2. Concomitant tolerated drugs

Tolerated Drugs	Rules of use
Concomitant medications or treatments (e.g., acetaminophen or diphenhydramine) as deemed necessary for appropriate prophylactic or symptomatic treatment. However, drugs included in the “Prohibited Drugs” section above are excluded.	Administer according to the prescription of the investigator, etc.
Best supportive care (including antimicrobials, nutritional support, correction of metabolic disturbances, optimal symptom control and pain management [e.g., palliative radiation therapy for non-target lesions])	For all subjects, use when necessary.
Inactivated viruses such as influenza vaccines	Can be inoculated (e.g. with vaccine)

5.10. Follow-up treatment

Post-treatment after completion or discontinuation of the clinical trial is not specified.

5.11. After discontinuation of this clinical trial

Subjects who discontinue the clinical trial will be subjected to necessary examination and observation, and appropriate measures will be taken as necessary, until it can be medically determined that the subject can be discharged or transferred to a hospital.

6. Clinical trial treatment

6.1. Durvalumab and tremelimumab

Refer to the investigator's brochure for details and handling of the investigational drug. The following are the investigational drugs to be used in this clinical trial.

6.1.1. Durvalumab

Durvalumab (MEDI4736) will be supplied by AstraZeneca as a 500-mg vial solution for infusion after dilution. The solution contains 50 mg/mL durvalumab, 26 mM histidine/histidine hydrochloride, 275 mM trehalose dihydrate, and 0.02% weight/volume (w/v) polysorbate 80; it has a pH of 6.0 and density of 1.054 g/mL. The nominal fill volume is 10.0 mL.

Durvalumab is a sterile, clear to opalescent, colorless to slightly yellow solution, free from visible particles.

Investigational product vials are stored at 2°C to 8°C (36°F to 46°F) and must not be frozen. Investigational products should be kept in original packaging until use to prevent prolonged light exposure.

6.1.1.1. Preparation of Durvalumab Dose by Infusion Bag

Each dose of durvalumab must be prepared by aseptic manipulation by the investigator or other investigational drug administrator designated by the site. The time between the puncture of the vial and the start of administration of Durvalumab must not exceed the following

- 2°C to 8°C for 24 hours
- 4 hours at room temperature
- Dosing solutions must be brought to room temperature before administration.

Dose 1,500 mg of durvalumab is administered with an infusion bag containing 0.9% saline or 5% dextrose to achieve a final concentration of 1 to 20 mg/mL of durvalumab and an intravenous administration set with a 0.2 μm or 0.22 μm filter. Add 1,500 mg of durvalumab (i.e., 30.0 mL of durvalumab) to the infusion bag. The infusion bag should be selected to achieve a final concentration within 1 to 20 mg/mL. Gently invert and mix the infusion bag until the administered fluid in the bag is uniform. The standard infusion time is 1 hour (±5 minutes). If the infusion is interrupted, the total infusion time should not exceed 8 hours at room temperature. No other drugs should be administered simultaneously in the same IV line.

After the contents of the infusion bag have been completely administered, flush the IV line with an IV diluent equal to the priming volume of the IV set used, or complete the infusion according to the provider's policy to ensure that the full volume is administered. If the line is not flushed, document this in the record. If the preparation time or infusion time limit is exceeded, a new vial must be used to prepare a new dose solution. Since durvalumab does not contain preservatives, unused preparation solutions must be discarded.

The preparation should be made in accordance with the “Procedures for the Administration of Investigational Drugs” for this clinical trial.

6.1.2. Tremelimumab

Tremelimumab will be supplied by AstraZeneca either as a 400-mg or a 25-mg vial solution for infusion after dilution. The solution contains 20 mg/mL tremelimumab, 20 mM histidine/histidine hydrochloride, 222 mM trehalose dihydrate, 0.27 mM disodium edetate dihydrate, and 0.02% weight/volume (w/v) polysorbate 80; it has a pH of 5.5 and density of 1.034 g/mL. The nominal fill volume is 20.0 mL for the 400-mg vial and 1.25 mL for the 25-mg vial.

Tremelimumab is a sterile, clear to opalescent, colorless to slightly yellow solution, free from or practically free from visible particles.

Investigational product vials are stored at 2°C to 8°C (36°F to 46°F) and must not be frozen.

Investigational products should be kept in original container packaging until use to prevent prolonged light exposure.

6.1.2.1. Preparation of Tremelimumab Dose by Infusion Bag

Each dose of tremelimumab must be prepared by aseptic manipulation by the investigator or the investigation drug manager designated by the site. The time from vial puncture to administration must not exceed

- 2°2°C to 8°C for 24 hours
- 4 hours at room temperature
- Doing solutions must be brought to room temperature before administration.

Tremelimumab is administered in an infusion bag containing 0.9% saline or 5% dextrose to achieve a final concentration of 0.10-10 mg/mL of tremelimumab, using an intravenous administration set with a 0.2 μm or 0.22 μm filter.

Add 300 mg of tremelimumab (i.e., 15.0 mL) to the infusion bag. Gently invert and mix the infusion bag until the dosing solution in the bag is homogeneous. The standard infusion time is 1 hour (±5 minutes); any infusion time less than 55 minutes will be considered a deviation from the study protocol. If the infusion is interrupted, the total infusion time should not exceed 8 hours at room temperature. No other drugs should be administered simultaneously in the same IV line. After the contents of the infusion bag have been completely administered, flush the IV line with an IV diluent equal to the priming volume of the IV set used, or complete the infusion according to the site's policy to ensure that the full volume is administered. If the line is not flushed, document this. If the preparation time or infusion time limit is exceeded, a new vial must be used to prepare a new dose solution. Since tremelimumab does not contain preservatives, unused preparation solutions must be discarded. The preparation should be made in accordance with the “Protocol for the Administration of Investigational Medicinal Products” for this clinical trial.

6.2. Control Drugs

There is no control drug in this study.

6.3. Monitoring during administration

During and after the infusion, the subject's condition should be monitored by assessment of vital signs at the times specified in the study protocol.

If an infusion-related reaction of grade 2 or less is observed, the infusion rate of the study drug may be reduced by 50% or discontinued until the event is resolved, and the infusion may be restarted at 50% of the initial infusion rate until the infusion is completed. Subjects who experience an infusion-related reaction of grade 2 or less may receive subsequent infusions at 50% of the initial rate. Acetaminophen and/or antihistamines (e.g., diphenhydramine) or equivalent drugs according to institutional standards may be administered at the investigator's discretion. If infusion-related reactions are grade 3 or greater, the investigational drug should be discontinued. The standard infusion duration is 1 hour, but if interrupted, the infusion should not exceed 8 hours at room temperature. Refer to the Toxicology and Management Guidelines in the protocol appendix for management of subjects who experience an infusion-related reaction.

As with other antibodies, allergic reactions to dose administration may occur. Appropriate drugs and medical devices to treat acute anaphylactic reactions must be readily available, and investigators must be trained to recognize and treat anaphylaxis. The site must have immediate access to an emergency resuscitation team and medical equipment, and the ability to admit subjects to the intensive care unit if necessary.

6.4. Management of investigational drugs

1) The investigator coordinator will deliver the investigational drug to the investigator of Chiba University Hospital in accordance with the agreement with the investigational drug

provider.

2) The investigator coordinator will properly manage the investigational drug in accordance with the protocol provided by the investigator through the site director.
3) The investigator shall prepare a document explaining the storage conditions, expiration date, and other handling methods of the investigational product and deliver it to the site manager, investigators collaborators, and investigational product manager.

6.5. Disposal of unused investigational drugs

1） After the investigational monitor checks the inventory and obtains the investigator's approval, the investigational drug manager discards used, unused, expired, or damaged investigational drugs and empty containers.
2） The investigational drug manager shall dispose of the investigational drug in accordance with the guidelines for disposal of pharmaceuticals.

6.6. Packaging and labeling of investigational drugs

The label should indicate that the product is for investigational use, the name, title and address of the coordinating investigator (representative), chemical name, volume, serial number, storage method and expiration date of the investigational drug. The label should appear on the bottle and on the package insert.

Labels for investigational new drugs shall be prepared in accordance with “Good Manufacturing Practice (GMP)” and GCP ordinances. The investigational drug label should be written in Japanese. Durvalumab and tremelimumab will be provided in a single sheet format or in a multilingual booklet format.

Investigational drug: Durvalumab (genetical recombination) (MEDI4736) + Tremelimumab (genetical recombination)

The drug name on the durvalumab label shall be “MEDI4736” or “Durvalumab (MEDI4736)” depending on the agreed upon drug name used in the approved clinical trial master label document. During this transition period, either name shall be correct.

6.7. Carbon ion radiotherapy

Information on Carbon ion radotherapy
- Name of medical device: Cardon ion radiotherapy Device
- Indications: Treatment of solid tumors
- Model Number: CI-1000S
- PMDA approval number: 22800BZX00096000
- Manufacturing facility: Toshiba Energy Systems&Solutions Corporation

Carbon Ion Therapy will be performed at the Quantum Science and Technology Agency QST Hospital, and irradiation will be performed for four consecutive days between Day 8 and Day 14 of Cycle 1 as per the schedule in Section 7.1. (If it is a holiday, it is acceptable to irradiate over the holiday.)

Dose prescription and fractionations will be 60Gy (RBE) / 4Fr / 1week for CIRT. RBE calculation is done by modified microdosimetric kinetic model (Inaniwa et al. Phys Med Biol. 2010). Pencil beam scanning technique will be used. The pencil beam covers the PTV voxel by voxel in successive layers. An optimization function drives the dose distribution in each treatment spot to reach the desired target coverage and organs at risk sparing.

All cases will undergo fiducial marker insertion prior to treatment preparation. Fiducial markers may be implanted under ultrasonography/fluoroscopy surveillance percutaneously or transarterialy.

For cases treated in an orthogonal fixed beam room, immobilization will be achieved with a relatively thick shell (3-mm thickness) made of a low-temperature thermoplastic and hydraulic urethane resin or vacuum-formed cushion to allow a range of beam angles by rotating the treatment table. However, a thinner shell may be used if a rotating gantry is available. In either case, the shell device is fixed by tapping to the table bottom, with tightening or loosening adjustment as required.

A 4D-CT simulation is required to allow for assessment of tumor motion. Fasting for 3 to 8 hours prior to simulation is required to control stomach/duodenum volume. Simulation CTs should be done with a CT slice thickness no greater than 3 mm. The 4D dataset is separated into 10 separate breathing phase bins. The simulation scan used for planning should NOT be performed with intravenous contrast; simulation CT after this planning CT may include contrast (for anatomic information).

Target lesion of the particle therapy will be focused on intrahepatic nodule with MVI. A 5mm margin will be taken as a clinical target volume margin for the feeding nodule, and 1cm margin alongside the vessel for the MVI lesion. Internal motion will be compensated according to 4D-CT movement assessment. Inter-fractional margin will be set to 3mm and combined with internal motion compensation forming a field specific planning treatment volume.

Dose constraints for risk organs are set as follows

- GI tract: D2cm3 ≤ 30Gy (RBE)
- Spinal cord: Dmax ≤ 25Gy (RBE)
- Remnant liver volume (liver volume receiving 30Gy (RBE) or less): 500cm3

In addition, the following liver volume information is collected in conjunction with the remaining liver volume

- Liver V5 Gy (RBE)
- Liver V20 Gy（RBE）

Confirmation of patient positioning is confirmed by orthogonal X-ray images. A maximum displacement of 3 mm in all directions is allowed between the reference and treatment images and is achieved by movement of the couch. Respiratory gating during treatment is mandatory. External respiratory surrogate systems or fluoroscopic tracking may be used for respiratory motion detection.

6.8. subject inclusion

In this “modified 3 + 3 design”, the first three subjects will be enrolled in cohort A. If no DLT is observed in any of these subjects, the trial will enroll additional subjects in Cohort B, who will also receive tremelimumab as follows.

If one subject develops DLT in any cohort, three additional subjects will be enrolled in that same cohort. The development of two or more DLTs in Cohort A will mean that the entire trial will be terminated, and two or more DLTs in Cohort B will indicate that the MTD has been exceeded and the regime in Cohort B will be discontinued. In that case, up to a total of 15 additional subjects will be enrolled in Cohort A.

Schematic of subject incorporation

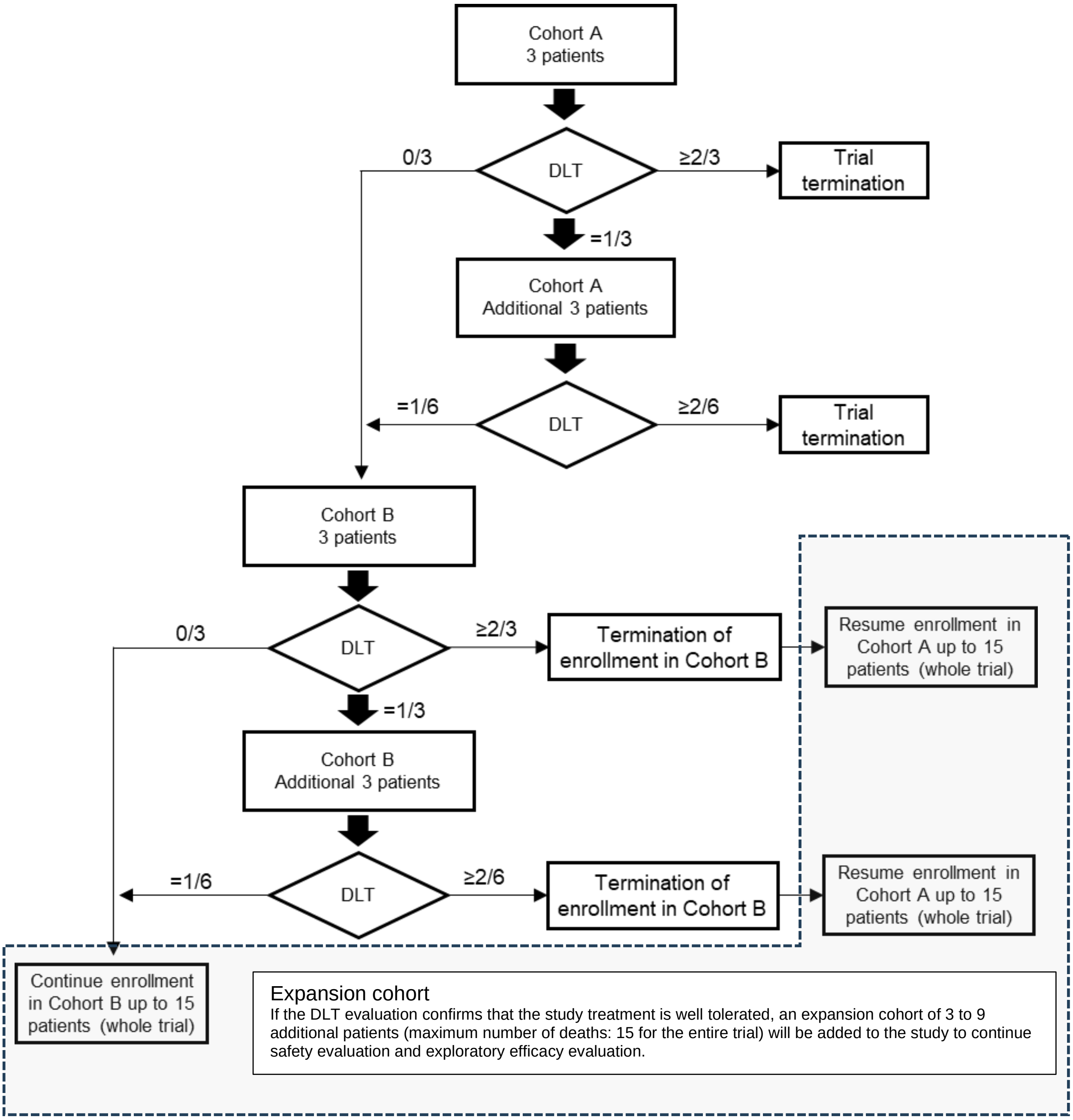

6.9. Definition of Dose-Limiting Toxicity (DLT)

During the 42-day period from the start of study drug administration (Cycle 1/DLT evaluation period), the following toxicities associated with the study treatment are considered DLTs: Grade determination follows CTCAE ver. 5.0.

Dose-limiting toxicity (DLT) will be assessed during the DLT evaluation period of this study, which will be 42 days from the time of the first dose on Cycle 1, Day 1. The severity of DLT will follow the guidelines described in the CTCAE ver. 5.0.

DLT is defined as the occurrence of an adverse event (AE) that is at least potentially related to the investigational drug or regimen (IR), with two exceptions: a Grade of vitiligo or alopecia is not a DLT. An AE that is at least potentially related to a regimen containing durvalumab and/or tremelimumab will be evaluated as a DLT if it meets one of the following criteria.

Patients who have not received heavy ion therapy are excluded from DLT assessment. However, if a patient initiates heavy ion therapy but does not complete heavy ion therapy within the allowed time due to an adverse event that cannot be excluded as being causally

related to durvalumab, tremelimumab, or heavy ion therapy, such adverse event will be considered a DLT.

- **Hematologic toxicity:**
  - Grade ≥3 neutropenia complicated by fever >38.3°C
  - Grade 4 neutropenia (lasting more than 7 days)
  - Grade ≥3 thrombocytopenia with significant bleeding
  - Grade 4 thrombocytopenia (regardless of duration)
  - Grade 4 anemia (regardless of duration)
  -
- **Non-hematologic toxicity:**
  - Any Grade 4 non-immune-mediated AE
  - Any Grade 4 immune-mediated AE, excluding endocrinopathies
  - Any Grade 3 non-immune mediated AE that does not resolve to ≤Grade 1 or baseline within 30 days with optimal medical management
  - Any Grade 3 immune-mediated AE – excluding diarrhea/colitis, pneumonitis, hepatitis, rash, neurotoxicity, myocarditis, myositis/polymyositis, endocrinopathies and nephritis – that does not resolve to ≤Grade 1 or baseline within 30 days after onset of the event despite optimal medical management including systemic corticosteroids
  - Grade 3 diarrhea or colitis that does not resolve to ≤Grade 1 within 14 days
    [both immune- and non-immune-mediated indicated here; the same is the case if not specified in remaining bullet points below]
  - Grade 3 noninfectious pneumonitis
  - Grade 2 noninfectious pneumonitis that does not resolve to ≤Grade 1 within 3 days of the initiation of maximal supportive care
  - Aspartate aminotransferase (AST) or alanine aminotransferase (ALT) ≥3×ULN with concurrent increase in total bilirubin (TBL) ≥2×ULN without evidence of cholestasis or alternative explanations (e.g., viral hepatitis, disease progression in the liver; i.e., “Hy’s Law”)
  - ALT or AST >8×ULN or TBL>5×ULN
  - Grade 3 immune-mediated rash that does not resolve to ≤Grade 1 or baseline within 30 days
  - Grade 2 rash covering > 30% BSA that does not resolve to ≤Grade 1 or baseline within 30 days
  - Any grade of immune-mediated rash with bullous formation
  - Grade 3 immune-mediated neurotoxicity (excluding Guillain-Barre and myasthenia gravis) that does not resolve to ≤Grade 1 within 30 days
  - Grade 2 or 3 immune-mediated peripheral neuromotor syndrome (such as Guillain-Barre and myasthenia gravis) that does not resolve to ≤Grade 1 within 30 days or that exhibits signs of respiratory insufficiency or autonomic instability
  - Grade 3 immune-mediated myocarditis
  - Any symptomatic immune-mediated myocarditis that does not become asymptomatic within 3 days of initiating optimal medical management including systemic corticosteroids
  - Grade 2 or 3 immune-mediated myositis/polymyositis that does not resolve to Grade ≤1 within 30 days of initiating optimal medical management including systemic corticosteroids or that exhibits signs of respiratory insufficiency regardless of optimal medical management
  - Immune-mediated increase in creatinine >3×ULN, or >3×baseline for patients with a baseline creatinine elevated above ULN
  - Transfusion (Red cell concentrate, Platelet), or the use of G-CSF during DLT period

The DLT evaluation period will be from the time of the first dose of study drug/IR until 42 days

post-dose. The first dose of durvalumab in Cycle 2 will be administered after the safety of Cycle 1 is confirmed.

The first treatment-related toxicity that occurs during the DLT period must be followed up to determine if the event qualifies as a DLT as defined in the DLT criteria above.

An immune-related adverse event is defined as an immune (inflammatory) adverse event without a definite other etiology. If an immune-related adverse event is suspected but there are no significant laboratory findings, a repeat laboratory test should be performed before a determination of DLT is made. Subjects who do not complete the DLT evaluation period for reasons other than DLT will not be considered DLT evaluable cases, and DLT will be evaluated after supplementation. The investigator will ask the Independent Data Monitoring Committee for an opinion on the supplementation decision.

Subjects will be administered the first dose of the study drug after hospitalization at Chiba University Hospital. After administration of the investigational drug, the subject will continue to be closely monitored in the hospital and will be transferred to the QST Hospital only for heavy ion therapy, taking into consideration the subject's condition and the means of transportation (taxi, private ambulance, etc.). After completion of heavy particle therapy, the patient should remain in the hospital for at least 7 days. During the transition to outpatient treatment, the investigator will perform the tests specified in 7.2.2 and confirm the safety of the treatment before allowing the patient to continue treatment as an outpatient. In addition, the investigator should be able to contact the site and the medical facility near the home in the event of an adverse event and establish a system to promptly contact the subject in an emergency.

6.10. Toxicity Management

Guidelines for the management of immune-mediated reactions, infusion reactions, and non-immune-mediated reactions to durvalumab are provided in the Durvalumab/Tremelimumab Toxicity Management Guidelines (TMGs).

Appropriate efforts should be made to thoroughly evaluate the subject and rule out neoplastic, infectious, metabolic, toxic, or other etiologies of imAE. Serologic, immunologic, and histologic (biopsy) data should be used to support the diagnosis of imAE, if appropriate. In the absence of a clear alternative etiology, the possibility of an immune-related etiology should be considered. In addition, there are situations in which durvalumab and tremelimumab should be discontinued (see Toxicity Management Guidelines). Dose reductions are not permitted. In case of doubt, consult the investigator. All toxicities will be evaluated according to CTCAE ver. 5.0.

6.11. Restrictions during the clinical trial

6.11.1. Restrictions during the clinical trial

The following restrictions apply during and before and after a given period of time while undergoing investigational treatment.

<u>About Women of Childbearing Potential</u>

Female subjects of childbearing potential who are not abstinent and who plan to have sex with an unprotected male partner must use at least one effective method of contraception from the time of screening until the drug treatment and drug discontinuation period (180 days after the last dose of durvalumab + tremelimumab combination therapy). Unprotected male partners of female subjects of childbearing potential must use a male condom and spermicide during this period. Discussion of stopping contraception after this time should be discussed with the family physician. Temporary abstinence, the rhythm method, and external ejaculation are not acceptable methods of contraception. Females should also not breast-feed during this period.

<u>About men with female partners of childbearing potential</u>

For non-contraceptive male subjects who are unprotected and plan to have sex with a female

partner of childbearing potential, the use of a male condom plus spermicide is mandatory from screening through the entire drug treatment and drug washout period (180 days after the last dose of durvalumab + tremelimumab combination therapy). However, periodic abstinence, rhythm and withdrawal methods are not acceptable methods of contraception. Male subjects will refrain from donating sperm during this period.

Female partners of male subjects (of childbearing potential) should also use highly effective contraceptive methods during this period.

Note: Pregnant women are defined as women who have not undergone sterilization (i.e., bilateral oophorectomy, bilateral oophorectomy, or total hysterectomy) or as premenopausal.

A woman is considered postmenopausal if she has been amenorrheic for 12 months without another medical cause. The following age-specific requirements apply

- Women under age 50 are considered postmenopausal if they have been amenorrheic for at least 12 months after discontinuation of exogenous hormone therapy and have luteinizing hormone and follicle stimulating hormone levels in the postmenopausal range of the institution.
- Women over age 50 are considered postmenopausal if they have been amenorrheic for at least 12 months after discontinuation of all exogenous hormone therapy, if they had radiation-induced menopause more than 1 year before their last menstrual period, and if they had chemotherapy-induced menopause more than 1 year before their last menstrual period.

A highly effective contraceptive method is defined as having a low failure rate (i.e., less than 1% per year) when used consistently and correctly.

6.12. Clinical Trial Procedures

The RECIST evaluation date must be performed as scheduled, regardless of any dosing delays. All other scheduled assessments must be performed at the start of the dosing cycle, and all laboratory and other tests required for dosing must be performed at least 3 days prior to dosing.

Subjects may be allowed to delay dosing under certain circumstances, as described below.

- Dosing may be delayed for either immune- or non-immune-related AEs in accordance with toxicity management guidelines.
- If dosing must be delayed for reasons other than treatment-related toxicities, dosing should be resumed as soon as possible.
- The dosing interval may be shortened as clinically appropriate to gradually match the treatment cycle to the tumor response plan (RECIST). Based on the half-life of durvalumab and tremelimumab, the interval between two consecutive doses should not be less than 22 days (for durvalumab and tremelimumab, see the current investigator's brochure).

7. OBSERVATION, EXAMINATION, AND ASSESSMENT, METHODS, AND TIMING OF IMPLEMENTATION

7.1. Implementation Schedule and Procedures

Cycle	Screening period	First tumor biopsy ※only in consented patients	DLT evaluation period							Second tumor biopsy ※only in consented patients	Durvalumab q4W dosing period					ST	Follow up period（28 days after the last administration date）	Safety information collection（90 days after the last administration date）
			Cycle 1				Cycle 2 [*13]				Cycle 3	Cycle43	Cycle 5	Cycle 6	Cycle 6< until PD			
Cycle Day	D -28 ～D -1	D -28〜D -1	1	8 to 14	15	22	1	8	14	After DLT evaluation	1[*1]	1	1	1	1	—	—	—
Allowable period（Day）			—	±3	±3	±3	±3	±3	±3		—	±3	±3	±3	±3	+14	+14	+14
Informed Consent ／ Subject backgroud information／ Review of eligibility	●[*2]																	
Durvalumab administration (cohort A and B)			●				●				●	●	●	●	●			
Tremelimumab administration (cohort B)[*3]			●															
Tumor biopsy		●								●								
CIRT				●●●●														
Fiducial marker insertion[*4]		●																
Fixation, simulation CT (for CIRT)[*4]	●																	
Weight*5	●		●	●	●	●	●	●	●		●	●	●	●	●	●	●	
Height	●																	
Physical exam	●		●	●	●	●	●	●	●		●	●	●	●	●	●	●	
Physical exam（Specific site based on case）	●		●	●	●	●	●	●	●		●	●	●	●	●	●	●	
Vital signs	●		●	●	●	●	●	●	●		●	●	●	●	●	●	●	
ECOG PS	●		●	●	●	●	●	●	●		●	●	●	●	●	●	●	
Clinical Chemistry／Hematology[*6]	●		●	●	●	●	●	●	●		●	●	●	●	●	●	●	
Coagulation	●		●	●	●	●	●	●	●		●	●	●	●	●	●	●	
Urinalysis	●		●								●	●	●	●	●	●	●	
ECG[*7]	●		●													●		
Hepatitis serology [*8]	●											(●)	(●)	(●)	(●)	(●)		
HIVtests	●																	
TSH、fT3、fT4 [*9]	●		●	●	●	●	●	●	●		●	●	●	●	●	●	●	
Pregnancy test [*10]	●																	
Chest X ray	●															●		
Assesment of Child-Pugh score	●		●				●				●	●	●	●	●	●	●	
CT/MRI[*11]	●		Every 6 weeks (±1 week) for the first 12 weeks from Cycle1 day1, and every 8 weeks (±1 week) thereafter until PD.															
Tumor marker（AFP、PIVKA-II）	●						●				●	●	●	●	●			
Cocmitant medication	←――――――――――――――――――――――――――――→																	
Assessment of AE/SAE[*12]	←――――――――――――――――――――――――――――――――→																	

*1. The baseline for the durvalumab Q4W dosing period will be the first day of the third cycle.
*2. Whenever possible, minimize the time between enrollment and initiation of therapy.
*3. In combination therapy, tremelimumab should be administered first, and durvalumab infusion should be started approximately 1 hour (maximum 2 hours) after tremelimumab administration is completed.
*4. To be administered to subjects who are eligible by other screening tests.
*5. Weight will be measured along with vital signs at the visit.
*6. Serum or plasma biochemical tests (including LFT monitoring) and blood tests may be performed more frequently if clinically indicated.
If screening biochemical and hematological evaluations were performed no more than 3 days prior to Day 1 (the first infusion day), they need not be performed again on Day 1.
Results of LFTs, electrolytes, complete blood count, and creatinine must be obtained prior to the start of infusion (within 3 days) and confirmed by the attending physician or investigator prior to administration.
*7. Three ECG results are required if clinically significant abnormalities are detected.
*8. If HBs-Ag, HBs-Ab, or HBc-Ab is positive, HBV-DNA should be measured (before and every 4 weeks after administration).
If HCV antibodies are positive, measure HCV-RNA (pre-test).
*9. Free T3 or free T4 should be measured only if TSH is abnormal or endocrine system-related AEs are clinically suspected. If TSH is measured up to 14 days prior to the first dose, it need not be measured again on Day 1.
*10. For women of childbearing potential only. For women of childbearing potential, a pregnancy test should be performed every 4 weeks starting 7 days prior to the first dose. Pregnancy tests can be performed on Day 1, but results must be confirmed by the treating physician or investigator before administration is initiated.
*11. RECIST evaluation is performed with CT (preferred) or MRI imaging of the chest, abdomen (including liver and adrenal glands), and pelvis IV contrast preferred. Imaging of the pelvis is recommended only if there is a possibility of primary or metastatic disease in the pelvic region. Additional anatomic imaging should be performed based on the individual subject's signs and symptoms at baseline and at follow-up. Baseline assessments should be performed no later than 28 days prior to the start of study drug administration for each cohort and should be performed as close as possible to the start of study drug or prior to the start of study drug if possible.
Confirmatory testing should be performed within 4 weeks of the prior PD evaluation, preferably at the next scheduled imaging visit (provided there is no clinically significant deterioration). If an unscheduled evaluation is performed and the subject has not progressed, every effort should be made to perform a subsequent evaluation at the next scheduled visit.
*12. AEs and SAEs should be collected from the time of the first dose of study drug until the follow-up date 28 days after the last dose of study drug. However, AE and SAE outcomes should be collected until the end of the safety information collection period (90 days after the last dose of study drug) or until the start of alternative anticancer therapy. In addition, events occurring after the 28-day follow-up period after the last dose of the study drug and considered to be attributable to delayed toxicity to the study drug will be collected as AEs or SAEs until the end of the safety information collection period or until the initiation of alternative anticancer therapy.
*13. *n the Expansion cohort, patients will be moved to Cycle 2 after the end of Cycle 1.

Note: All assessments on the treatment day shall be performed prior to infusion unless otherwise indicated.
ECG Electrocardiogram; LFT Liver function tests; T3 Triiodothyronine; T4 Thyroxine; TSH Thyroid stimulating hormone.

7.2. Observation, tests and assessment

7.2.1. Screening period

The investigator or sub-investigator will perform the following screening tests and enroll subjects who meet the selection criteria and do not violate the exclusion criteria. The tests will be performed after consent is obtained and between 28 days before the start of the investigational drug and the day before the start of the investigational drug. Test items will be as described below. However, test results obtained prior to consent as part of routine medical care using procedures similar to those used in this study may be used as screening tests if they fall within the 28-day screening period.

- Informed Consent
- Subject background information*
- Review of eligibility criteria
- Complete physical exam
- ECOG Performance Status
- Vital signs**, weight and height
- Chest X ray
- 12-lead ECG (in triplicate [2-5 minutes apart])
- Assessment of Child-Pugh score
- Imaging by CT/MRI, if applicable to study
- Clinical laboratory tests for:
  - Clinical Chemistry (see Table 5)
  - Hematology (see Table 4)
  - TSH, fT3, fT4
  - Coagulation (PT, PTT, INR)
  - Creatinine Clearance
  - Pregnancy test (for women of childbearing potential only)
  - Hepatitis serology
  - HIV test
  - Urinalysis
  - Tumor marker (AFP, PIVKA-II)
- Fiducial marker insertion
- Fixation, simulation CT (for CIRT)
- Concomitant medication

* **Subject background information and medical history**

Subject identification code number, race, sex, age at obtaining IC, medical history, concurrent diseases, alcohol consumption, smoking history, and information for HCC with initial diagnosis, pathological diagnosis and past treatment.

** **Vital signs**

Vital signs will be measured at every visit and will include assessments of systolic and diastolic BP, temperature, and HR. Systolic and diastolic BPs will be documented in mmHg. Temperature will be obtained in degrees Celsius. HR will be documented in beats per minute. Generally, each patient will have blood pressure tested in the same arm. Measurement device and measurement time will not be indicated.

7.2.2. DLT assessment period

Day1

- Durvalumab administration (cohort A and B)
- Tremelimumab administration (cohort B）
- Complete physical exam
- ECOG Performance Status
- Vitals signs and weight
- 12-lead ECG (in triplicate [2-5 minutes apart])
- Assessment of Child-Pugh score
- Clinical laboratory tests for:
  - Clinical chemistry
  - Hematology
  - TSH、fT3、fT4
  - Coagulation（PT、APTT、PT-INR）
  - Creatinine Clearence
  - Pregnancy test (women of childbearing potential only)
  - Urinalysis
- Assessment of AE/SAE
- Confirmation of concomitant therapy

Day 8-14

- CIRT (60 Gy (RBE) / 4 Fr) (performed at QST hospital)
- Complete physical exam
- ECOG Performance Status
- Vitals signs and weight
- Assessment of Child-Pugh score
- Clinical laboratory tests for:
  - Clinical chemistry
  - Hematology
  - TSH、fT3、fT4
  - Coagulation（PT、APTT、PT-INR）
  - Creatinine Clearence
- Assessment of AE/SAE
- Confirmation of concomitant therapy

Day 15

- Complete physical exam
- ECOG Performance Status
- Vitals signs and weight
- Assessment of Child-Pugh score
- Clinical laboratory tests for:
  - Clinical chemistry
  - Hematology
  - TSH、fT3、fT4
  - Coagulation（PT、APTT、PT-INR）
  - Creatinine Clearence

- Assessment of AE/SAE
- Confirmation of concomitant therapy

Day 22

- Complete physical exam
- ECOG Performance Status
- Vitals signs and weight
- Assessment of Child-Pugh score
- Clinical laboratory tests for:
    - Clinical chemistry
    - Hematology
    - TSH、fT3、fT4
    - Coagulation（PT、APTT、PT-INR）
    - Creatinine Clearence
- Assessment of AE/SAE
- Confirmation of concomitant therapy

Day 29（Cycle 2 Day1）

- Durvalumab administration (cohort A and B)
- Tremelimumab administration (cohort B）
- Complete physical exam
- ECOG Performance Status
- Vitals signs and weight
- 12-lead ECG (in triplicate [2-5 minutes apart])
- Assessment of Child-Pugh score
- Clinical laboratory tests for:
    - Clinical chemistry
    - Hematology
    - TSH、fT3、fT4
    - Coagulation（PT、APTT、PT-INR）
    - Creatinine Clearence
    - Pregnancy test (women of childbearing potential only)
    - Tumor maker (AFP, PIVKA-II)
- Assessment of AE/SAE
- Confirmation of concomitant therapy

Day 36（Cycle2 Day8）

- Complete physical exam
- ECOG Performance Status
- Vitals signs and weight
- Assessment of Child-Pugh score
- Clinical laboratory tests for:
    - Clinical chemistry
    - Hematology
    - TSH、fT3、fT4
    - Coagulation（PT、APTT、PT-INR）

- Creatinine Clearence

- Assessment of AE/SAE
- Confirmation of concomitant therapy

Day 43（Cycle 2 Day14）

- Complete physical exam
- ECOG Performance Status
- Vitals signs and weight
- Assessment of Child-Pugh score
- Clinical laboratory tests for:
  - Clinical chemistry
  - Hematology
  - TSH、fT3、fT4
  - Coagulation（PT、APTT、PT-INR）
  - Creatinine Clearence
- Assessment of AE/SAE
- Confirmation of concomitant therapy

At discharge during the DLT evaluation period

- Complete physical exam
- ECOG Performance Status
- Vitals signs and weight
- Assessment of Child-Pugh score
- Clinical laboratory tests for:
  - Clinical chemistry
  - Hematology
  - TSH、fT3、fT4
  - Coagulation（PT、APTT、PT-INR）
  - Creatinine Clearence
- Assessment of AE/SAE
- Confirmation of concomitant therapy

7.2.3. Durvalumab q4W dosing period

At Each cycle Day1

- Durvalumab administration (cohort A and B)
- Complete physical exam
- ECOG Performance Status
- Vitals signs and weight
- 12-lead ECG (in triplicate [2-5 minutes apart])
- Assessment of Child-Pugh score
- Clinical laboratory tests for:
  - Clinical chemistry
  - Hematology
  - TSH、fT3、fT4
  - Coagulation（PT、APTT、PT-INR）
  - Creatinine Clearence
  - Pregnancy test (women of childbearing potential only)

- Tumor maker (AFP, PIVKA-II)

- Assessment of AE/SAE
- Confirmation of concomitant therapy

7.2.4. At the time of discontinuation of investigational drug administration

- Complete physical exam
- ECOG Performance Status
- Vitals signs and weight
- 12-lead ECG (in triplicate [2-5 minutes apart])
- Assessment of Child-Pugh score
- Clinical laboratory tests for:
  - Clinical chemistry
  - Hematology
  - TSH、fT3、fT4
  - Coagulation（PT、APTT、PT-INR）
  - Creatinine Clearence
  - Tumor maker (AFP, PIVKA-II)
- Assessment of AE/SAE
- Confirmation of concomitant therapy

7.2.5. Follow up period

- Complete physical exam
- ECOG Performance Status
- Vitals signs and weight
- 12-lead ECG (in triplicate [2-5 minutes apart])
- Assessment of Child-Pugh score
- Clinical laboratory tests for:
  - Clinical chemistry
  - Hematology
  - TSH、fT3、fT4
  - Coagulation（PT、APTT、PT-INR）
  - Creatinine Clearence
  - Urinalysis
  - Tumor maker (AFP, PIVKA-II)
- confirmation of survival
- Assessment of AE/SAE
- Confirmation of concomitant therapy

7.3. Biological sampling procedures

7.3.1. Guideline for blood sampling volume

The total volume of blood to be drawn from each subject in this study is as follows

Amount of blood to be collected from each subject

Assesement		Sample volume (mL) / visit
**Safety**	**Clinical Chemistry Tests**	10

Amount of blood to be collected from each subject

Assesement	Sample volume (mL) / visit
**Hematology Tests**	10
**Total**	20

7.3.2. Blood samples for archiving

When consent is obtained from the subject for storage of blood specimens, residual blood specimens designated for biochemistry and blood tests will be stored at the Department of Gastroenterology, Chiba University Hospital. Blood specimens will be handled in such a way as to prevent leakage, confusion, theft, or loss of personal information by anonymization with an identification code. Blood specimens will be stored for a period not exceeding 20 years after the completion of the clinical trial, after which all specimens will be properly disposed of. If a subject withdraws consent for specimen storage, the specimens will be destroyed and this will be documented. In addition, if specimens are to be used in future research, a new research protocol must document the use of this specimen and be submitted to the IRB for approval.

7.3.3. Hepatic tumor biopsy sample

In this study, a percutaneous liver biopsy/liver tumor biopsy will be performed before the first dose and between 43 and 56 days after the first dose, if the subject is deemed safe for such a biopsy/liver tumor biopsy by the investigator or sub-investigator and if the subject consents. The tissue samples (tumor and non-tumor) obtained will be used for exploratory studies (refer to 9.3.). At that time, the tissue samples will be handled to prevent disclosure, mix-up, theft, or loss of personal information by anonymization with an identification code. The investigator or sub-investigator may discontinue the percutaneous liver biopsy or liver tumor biopsy for the safety of the subject. Failure to perform a percutaneous liver biopsy/liver tumor biopsy at the discretion of the investigator or sub-investigator, or failure to perform a percutaneous liver biopsy/liver tumor biopsy without the consent of the subject, will not preclude enrollment or dosing in this study. Tissue specimens will be retained for a maximum of 20 years after completion of the study, after which all specimens will be properly disposed of. If a subject withdraws consent for specimen storage, the specimen will be discarded and this will be documented. In addition, if specimens are to be used in future research, the use of that specimen must be documented in a new research protocol and submitted to the IRB for approval.

7.4. Asesement of efficiency

The following are guidelines for confirming image evaluations to assess efficacy.

- Imaging evaluations are performed for subject management and treatment decisions.
- Image evaluation is performed using RECIST ver. 1.1.
- In the absence of clinically evident evidence of disease progression, the patient should be re-evaluated after disease progression (PD) is determined by RECSIST ver. 1.1 in order to distinguish between immune checkpoint inhibitor-induced pseudo progression and true disease progression (this is to reduce the risk of study termination due to incorrect evaluation by the investigator/participating physician). (This is to reduce the risk of termination of the study due to incorrect assessment by the investigator/associated investigator.)

The definition of objective disease progression (definite PD) is as follows

1) The presence of clinically evident evidence of disease progression and disease progression (PD) according to RECIST ver. 1.1 is confirmed as objective disease progression.
2) In the absence of clinically evident findings of disease progression (PD) by RECIST ver. 1.1, a second imaging study to evaluate PD according to the specific criteria below should be

performed after disease progression (PD). The partially modified RECIST ver. 1.1 used for objective confirmation of progression is used only to confirm objective disease progression (confirmed PD). Imaging evaluations to determine objective disease progression should be performed within 4 weeks of the first imaging evaluation that determined PD using RECIST ver. 1.1.

The following is RECIST ver. 1.1 modified to establish objective disease progression.

- On two consecutive image evaluations, the sum of the diameters of the target lesions (TL) increases by more than 20% compared to the sum of the smallest diameters and the sum of the diameters increases by more than 5 mm compared to the sum of the smallest diameters.
- Non-target lesions (NTL) and/or pre-existing new lesions showed significant progression (worsening) at the time of the confirmatory examination compared to the most recent imaging evaluation (note: new lesions at the time of imaging evaluation that are determined for the first time to be PD by RECIST ver. 1.1 are evaluated as NTL at the second imaging evaluation).
- The appearance of an obvious new lesion that was not present at the first imaging evaluation that determined PD according to RECIST ver. 1.1 but was present at the second imaging evaluation.

Two consecutive assessments meeting the definition of PD (first PD by RECIST ver. 1.1 and a second PD using the progression confirmation criteria (above)) are required to establish objective disease progression (determination of definite PD). If PD by RECIST ver. 1.1 does not confirm objective disease progression, evaluation will continue until the next PD by RECIST ver. 1.1. In the absence of significant clinical progression, treatment with investigational agents may continue between the first assessment of progression and imaging studies to confirm progression. If PD is confirmed on confirmatory imaging, the date of progression will be the date PD was confirmed on the previous visit. If objective disease progression is not confirmed, the subject will continue to receive study drug and on-therapy evaluation until the next PD, if there is no clinically significant worsening, at which time another confirmatory scan will be required, even if objective disease progression is confirmed. If the initial PD is not immediately confirmed at the next scan, the investigator should not change the PD assessment from the initial scan.

If subjects discontinue treatment (and/or receive subsequent anticancer therapy) prior to radiographic progression, subjects should be followed until objective disease progression is confirmed. Once progression is confirmed, subjects should continue to be followed for survival every 2 months (8 weeks) according to the evaluation follow-up schedule.

7.5. Assessment of safety

7.5.1. Clinical laboratory tests

Blood and urine samples for determination of clinical chemistry, hematology, and urinalysis will be taken at the times indicated in the assessment schedules and as clinically indicated（refer to 7.1.）

Clinical laboratory safety testing, including serum pregnancy testing, is performed in a licensed clinical laboratory according to local standard procedures. Specimen tubes and specimen sizes may vary depending on the laboratory method used and routine practices at the site. Pregnancy testing may be performed at the site using an approved test (urine or serum pregnancy test). Abnormal clinically significant laboratory results should be repeated as soon as possible (preferably within 24 to 48 hours).

Additional safety samples may be collected if clinically indicated at the discretion of the Investigator. The date, time of collection, and results (values, units, and reference ranges) will be recorded on the appropriate eCRF.

The laboratory variables to be measured are presented in Table 4 (Hematology/ Coagulation), Table 5 (Clinical chemistry), and Table 6 (urinalysis).

Other safety tests to be performed at screening include assessment for hepatitis B surface antigen, hepatitis C antibodies, and HIV antibodies.

The following laboratory variables will be measured:

Table4. Hematology/Coagulation Laboratory Tests

Basophils	Monocytes
Eosinophils	Neutrophils
Hematocrit	Platelet count
Hemoglobin	Red blood cell count
Lymphocytes	Total white cell count [a]
PT-INR	APTT

Table5. Clinical Chemistry (Serum or Plasma) Laboratory Tests

Albumin	Lactate dehydrogenase
Alkaline phosphatase	Lipase
Alanine aminotransferase	Magnesium
Amylase	Potassium
Aspartate aminotransferase	Sodium
Calcium	Total bilirubin[a]
Chloride	Total protein
Creatinine	Urea or blood urea nitrogen, depending on local practice
Gamma glutamyltransferase[b]	Uric acid
Glucose	

a. Tests for ALT, AST, alkaline phosphatase, and total bilirubin must be conducted and assessed concurrently. If total bilirubin is ≥2 × upper limit of normal (and no evidence of Gilbert's syndrome) then fractionate into direct and indirect bilirubin.

b. It is preferable that both amylase and lipase parameters are assessed. For sites where only 1 of these parameters is routinely measured then either lipase or amylase is acceptable.

c. Bicarbonate (where available), chloride, creatinine clearance, gamma glutamyltransferase, and magnesium testing are to be performed at baseline, on Day 1 (unless all screening laboratory clinical chemistry assessments are performed within 3 days prior to Day 1), and if clinically indicated.

d. Creatinine Clearance will be calculated by data management using Cockcroft-Gault (using actual body weight).

e. If TSH is measured within 14 days prior to Day 1 (first infusion day), it does not need to be repeated at day Free T3 or free T4 will only be measured if TSH is abnormal or if there is a clinical suspicion of an AE related to the endocrine system

Table 1. Urinalysis Tests[a]

Bilirubin	pH
Blood	Protein
Glucose	Specific gravity
Ketones	Colour and appearance

a. Microscopy should be used as appropriate to investigate white blood cells and use the high-power field for red blood cells

If a patient shows an AST or ALT ≥3xULN together with total bilirubin ≥2xULN, refer to 8.18.3 for further instructions on cases of increases in liver biochemistry and evaluation of Hy's Law. These cases should be reported as SAEs if, after evaluation, they meet the criteria for a Hy's law case or if any of the individual liver test parameters fulfill any of the SAE criteria.

All patients should have further chemistry profiles performed at 30 days (±3 days), 2 months (±1 week) and 3 months (±1 week) after permanent discontinuation of IP

Any clinically significant abnormal laboratory values should be repeated as clinically indicated and recorded on the eCRF. Situations in which laboratory safety results should be reported as AEs are described in Section8.1.

All patients with Grade 3 or 4 laboratory values at the time of completion or discontinuation from IP must have further tests performed until the laboratory values have returned to Grade 1 or 2, unless these values are likely to improve because of the underlying disease.

7.5.2. Physical examinations

Physical examinations will be performed according to the assessment schedules. Full physical examinations will include assessments of the head, eyes, ears, nose, and throat and the respiratory, cardiovascular, GI, urogenital, musculoskeletal, neurological, dermatological, hematologic/lymphatic, and endocrine systems. Height will be measured at screening only. Targeted physical examinations are to be utilized by the Investigator on the basis of clinical observations and symptomatology. Situations in which physical examination results should be reported as AEs are described in Section 8.1.

7.5.3. Electrocardiogram (ECG)

Resting 12-lead ECGs will be recorded at screening and as clinically indicated throughout the study. ECGs should be obtained after the patient has been in a supine position for 5 minutes and recorded while the patient remains in that position.

In case of clinically significant ECG abnormalities, including a QTcF value >470 ms, 2 additional 12-lead ECGs should be obtained over a brief period (e.g., 30 minutes) to confirm the finding.

Situations in which ECG results should be reported as AEs are described in Section 8.1.

7.5.4. Vital signs

Vital signs (blood pressure [BP], pulse, temperature, and respiration rate) will be evaluated according to the schedule of this clinical trial. Body weight is also recorded at each visit along with vital signs.

First infusion

On the first infusion day, patients will be monitored, and vital signs collected/recorded in eCRF prior to, during and after infusion of IP as presented in the bulleted list below.

BP and pulse will be collected from a subject before, during, and after each infusion at the

following times (based on a 60-minute infusion):

- Prior to the beginning of the infusion (measured once from approximately 30 minutes before up to 0 minutes [i.e., the beginning of the infusion])
- Approximately 30 minutes during the infusion (**halfway** through infusion)
- At the end of the infusion (approximately 60 minutes ±5 minutes)

If the infusion takes longer than 60 minutes, then BP and pulse measurements should follow the principles as described above or be taken more frequently if clinically indicated. A 1-hour observation period is recommended after the first infusion of durvalumab.

Subsequent infusions

BP, pulse and other vital signs should be measured, collected/recorded in eCRF prior to the start of the infusion. Patients should be carefully monitored and BP and other vital signs should be measured during and post infusion as per institution standard and as clinically indicated.

7.5.5. ECOG performance status

ECOG performance status will be assessed at the times specified in the assessment schedules based on the following:

0. Fully active; able to carry out all usual activities without restrictions
1. Restricted in strenuous activity, but ambulatory and able to carry out light work or work of a sedentary nature (e.g., light housework or office work)
2. Ambulatory and capable of self-care, but unable to carry out any work activities; up and about more than 50% of waking hours.
3. Capable of only limited self-care; confined to bed or chair more than 50% of waking hours
4. Completely disabled; unable to carry out any self-care and totally confined to bed or chair
5. Dead

Any significant change from baseline or screening must be reported as an AE.

7.5.6. Other safety assesements

If new pulmonary symptoms (e.g., dyspnea) or radiological abnormalities suggestive of pneumonia/interstitial lung damage (ILD) are observed, toxicity management as detailed in the Toxicity Management Guidelines (see Appendix of Protocol 2) will be applied. Complete diagnostic results (including high-resolution computed tomography (HRCT), blood and sputum cultures, hematological parameters, etc.) should be recorded in the source documents. An accurate diagnosis including consultation with a specialist is strongly recommended to rule out alternative causes such as lymphangitic carcinomatosis, infection, allergy, cardiogenic edema, or pulmonary hemorrhage. In the presence of a confirmatory HRCT scan that excludes other causes of respiratory symptoms, the diagnosis of interstitial lung disease (ILD) should be considered and toxicity management guidelines followed. The investigator is responsible for ensuring that all staff involved in the study are familiar with the contents of this section.

8. HANDLING OF ADVERSE EVENT

8.1. Definition

8.1.1. Adverse event

Adverse events are all unwanted or unintended signs (including abnormal changes in laboratory values), symptoms, or illnesses that occur after the first administration of an investigational drug, regardless of causal relationship to the study treatment.

8.1.2. Severe adverse event

A serious adverse event is defined as any of the following

(1) results in death
(2) is life-threaening
(3) requires inpatient hospitalisation or prolongation of existing hospitalisation
(4) results in persistent or significant disability/incapacity
(5) is a congenital anomaly/birth defect
(6) Other serious cases according to the above

The term “life-threatening” for the purposes of this definition is defined as an event that the investigator/participating physician determines poses an imminent risk of death to the subject as a result of its manifestation. It does not mean hypothetically that death might have resulted had the manifestation of the event been more severe.

“Hospitalization” in (3) below is not considered a serious adverse event if any of the following apply: (However, a new occurrence during the hospitalization is treated as an adverse event. (However, any new occurrence during that hospitalization will be treated as an adverse event.)

- Hospitalization as defined in this study protocol
- Hospitalization or prolongation of hospital stay that was planned prior to the start of the clinical trial.
- Hospitalization or prolonged hospitalization for social reasons (reasons of convenience or other non-medical necessity)
- Hospitalization or extended hospital stay for examination, education
- Hospitalization for follow-up or prolongation of hospitalization for those who are cured or have a mild illness.
- Hospitalization or prolonged hospitalization for new treatment of the underlying disease after completion of the investigational drug

(6) Other serious, in accordance with the above, are “significant medical events” that may not be immediately life-threatening or result in death or hospitalization, but which may endanger the subject or require treatment or therapy to avoid the consequences as listed in these definitions.

Adverse events (AEs) for malignancy reported during a clinical trial will generally be evaluated as serious AEs. If no other severity criteria are met, the AE will be judged to be a “Significant Medical Event” as described above. However, in certain circumstances, medical judgment based on individual events should be applied to clarify that malignancy events should be evaluated and reported as non-serious AEs. For example, if medical history includes malignancy and the malignancy progresses during the clinical trial, but the progression does not change the treatment or prognosis of the malignancy, the malignancy progression should be reported as an AE but may not meet the attributes to be evaluated as serious.

The causal relationship of the SAE (relationship to all investigational treatments/procedures) should be evaluated by the investigator and reported to AstraZeneca.

8.1.3. Adverse Events of Special Interest (AESI)

AESIs of durvalumab and tremelimumab for heavy particle irradiation include events due to potential inflammatory or immune-mediated mechanisms, which require more frequent monitoring and treatment with steroids, immunosuppressive agents, and/or hormone replacement therapy.Careful monitoring of these AESIs will be implemented in clinical trials of durvalumab monotherapy and durvalumab plus tremelimumab combination therapy. Immune-mediated

adverse events (imAEs) are AESIs, defined as events that occur with the administration of (exposure to) an investigation agent, are consistent with an immune-mediated mechanism of action, and have no apparent other cause. imAEs should be diagnosed using serologic, immunologic, and histologic (biopsy) data, as appropriate, for support. The diagnosis of imAE should be supported by serologic, immunologic, and histologic (biopsy) data as appropriate, and efforts should be made to rule out tumor, infection, metabolism, toxins, and other causes of imAE.

If there is any doubt as to whether an adverse event is an imAE, the investigator should immediately contact the sponsor's medical experts. AESIs for durvalumab and/or tremelimumab and heavy particle irradiation include the following.

- Dysentery/colitis, intestinal perforation
- Pneumonitis/ILD
- Hepatitis/transaminases increase
- Endocrine disorders (i.e., hypophysitis, hypopituitarism, adrenal insufficiency, hyperthyroidism, hypothyroidism and type I diabetes mellitus)
- Rash/dermatitis
- Nephritis/increased blood creatinine
- Pancreatitis/increased serum lipase and amylase
- myocarditis
- pericarditis
- Myositis/polymyositis
- Neuropathy/neuromuscular toxicity (Guillain-Barré syndrome, myasthenia gravis, etc.)
- Other inflammatory reactions of rare / infrequent immune-mediated (but not limited to pericarditis, sarcoidosis, uveitis, and ocular, skin, blood system and rheumatology-related events)

In addition, reactions associated with infusion and hypersensitivity/anaphylactic reactions due to various pharmacologic causes are also considered AESIs.

Details of these risks and presenting symptoms are described in the most recent versions of the durvalumab and tremelimumab investigational new drug summaries. Specific guidelines for the evaluation and treatment of these AESIs are provided in the Dose Modification and Toxicity Management Guidelines. These guidelines were developed by the investigational drug provider to assist investigators and others in making clinical decisions when treating this type of toxicity. This guideline applies to adverse events that the reporting investigator determines are causally related to the investigational drug/regimen.

If new or worsening pulmonary symptoms (e.g., dyspnea) or radiological abnormalities suggestive of pneumonia/interstitial lung disease are observed, toxicity management as detailed in the “Toxicity Management Guidelines” (see Appendix F) will be applied. Results of complete diagnostic tests (including high-resolution computed tomography (HRCT), blood and sputum cultures, hematological parameters, etc.) should be recorded in the source documents.

An accurate diagnosis, including consultation with a specialist, is strongly recommended to rule out alternative causes such as lymphangitic carcinomatosis, infection, allergy, cardiogenic edema, or pulmonary hemorrhage. On confirmatory HRCT scans where other causes of respiratory symptoms have been ruled out, the diagnosis of interstitial lung disease (ILD) should be considered and toxicity management guidelines followed.

8.1.4. Confirmation of interstitial lung disease (ILD)

To ensure thorough investigation and diagnosis of possible cases of pneumonia, the following evaluations, and additional evaluations as needed, will be performed. Collect the results of the evaluation.

- physical examination

- Evaluate signs and symptoms (cough, shortness of breath, fever, etc.), including auscultation of the lung field.
- Peripheral oxygen saturation (SpO2)

Other

If pneumonia (ILD) is suspected during clinical trial treatment, the following markers should be measured.

if possible

(i) ILD markers (KL-6, SP-D) and β-D-glucan

(ii) Tumor markers. Specific tumor markers associated with disease progression.

Other biochemistry: CRP, LDH

8.2. Assessment of severity

AEs and SAEs; severity will be determined in accordance with CTCAE ver. 5.0 The severity of all other events not listed in the CTCAE will be determined by the severity category from Grade 1 to 5, as determined by the investigator based on medical judgment, will be as follows.

- Grade 1 (mild)
  Events that are usually transient and require only minimal Treatment or therapeutic intervention. The event generally does not interfere with normal activities of daily living.

- Grade 2(Moderate)
  An event that is usually alleviated by additional specific therapeutic intervention. The event interferes with normal daily activities and causes discomfort, but does not pose a risk of serious or permanent harm to the subject.

- Grade 3(Severe)
  An event requiring intensive therapeutic intervention. An event that interferes with normal daily activities or significantly affects the subject's clinical condition.

- Grade 4(life-threatening）
  Events and/or immediate sequelae related to the following Imminent risk of death or a physical or mental impairment that affects or limits the ability to perform activities of daily living (eating, walking, toileting, etc.).

- Grade 5(deadly)
  Death as a result of an event. it is important to distinguish between serious criteria and severity of AEs.

It is important to distinguish between severity and severity of an AE. Severity is a measure of intensity, and severity is defined by the criteria in section 10.3.1 A Grade 3 AE need not necessarily be considered an SAE. For example, a Grade 3 headache lasting several hours would not meet the regulatory definition of an SAE and would be considered a non-serious event, whereas a Grade 2 attack leading to hospitalization would be considered an SAE.

8.3. Record of adverse events and serious adverse events

AEs and SAEs will be collected from the time of the first dose of study drug until the follow-up date 28 days after the last dose of study drug. However, AE and SAE outcomes will be collected until the end of the safety information collection period (90 days after the last dose of study drug) or until the start of alternative anticancer therapy. Of events occurring after the 28-day follow-up period after the last dose of the investigational drug, if the event is considered to be due to delayed toxicity to the investigational drug, it will be collected as an AE or SAE until the end of the safety information collection period or until the start of alternative anticancer

therapy. All AEs and SAEs will be actively followed up for each subject for the duration of the trial as long as the event is ongoing. Every effort will be made to resolve all events, even if the event continues after the subject discontinues the investigational drug or after the study is terminated.

AEs unresolved at the subject's last clinical trial visit will be followed up by the investigator as long as medically necessary but will not be further documented in the eCRF. AstraZeneca reserves the right to request additional information from subjects with ongoing AEs/SAEs at the end of the trial if deemed necessary.

For each AE, the following information should be collected

- Adverse event name
- Date of adverse event and date of disappearance
- Maximum CTCAE Grade
- Severity
- Assessment of causal relationship with study treatment
- Treatments related to investigational drugs
- Treatment of adverse events: Administration of AE medication
- outcome

In addition to the above, the following are also confirmed for SAE.

- Date of serious adverse event
- Date when the investigator becomes aware of the onset of the serious adverse event
- Definitions applicable to the determination of serious adverse events
- Date of admission
- Date of discharge
- Presumed reason for death
- Date of death
- Autopsy
- Evaluation of causal relationship with clinical trial procedures
- Evaluation of causal relationship with other drugs
- Details of serious adverse events

The grading scale described in CTCAE ver. 5.0 is used for all events that have been assigned to a CTCAE Grade; for events that have not been assigned a CTCAE Grade, the CTCAE criteria for converting mild, moderate, and severe events to a CTCAE Grade A copy of CTCAE ver. 5.0 can be downloaded from the Cancer Treatment Evaluation Program website at http://www.jcog.jp/doctor/tool/ctcaev5.html.

8.4. Duration of recording and follow-up of adverse events and serious adverse events

If the subject discontinues treatment for reasons other than objective confirmation of disease progression and therefore continues tumor evaluation, the drug- or treatment-related SAEs will be tracked until the subject confirms PD and no further tumor evaluation is performed.

The investigator is responsible for tracking all SAEs until the subject returns to baseline status or until the condition stabilizes with the expectation that the chronic condition will be maintained, even if it continues beyond study participation, until all SAEs are resolved.

8.5. Causal relationship with investigational therapy

The following examples will be used to determine the causal relationship between the clinical trial treatment and the patient.

Causative: if a reasonable possibility can be explained that the investigational treatment caused the adverse event in question (examples are given below).

1) If the event is time-related to the onset of the event, and the event attenuates with the passage of time after the study treatment but recurs or worsens with subsequent re-administration of the study treatment.
2) The presence of confounding risk factors is negative, such as the subject's general condition, complications, concomitant medications, or concomitant therapies.

No causal relationship: other than above

8.6. Outcome definition

Outcomes after adverse events are determined from the following

1) Recovery: when the patient recovers to the state before the adverse event occurred.
2) Recovered but with sequelae: When the adverse event has recovered but the effects of the adverse event remain as sequelae
3) Death: when the adverse event that occurred was the direct cause of death
4) Lightening of symptoms: adverse events continue, but symptoms are improving
5) Unrecovered: adverse events continue (symptoms are not improving)
6) Unknown: When the subject is no longer traceable

8.7. Treatment of investigational drug in the event of an adverse event

1) No change: When an adverse event occurs but there is no change in the conditions under which the investigational drug is administered.
2) Discontinuation: Discontinuation of study drug administration due to the occurrence of an adverse event
3) Withdrawal: Temporary suspension of study drug administration due to the occurrence of an adverse event
4) Not applicable: Adverse events occur before the start of study drug administration or after the end of the study period

8.8. Treatment of heavy particle therapy equipment in the event of an adverse event

1) Suspension: Temporary suspension of heavy-ion radiation therapy due to the occurrence of adverse events
2) Discontinuation: Discontinuation of heavy-ion radiation therapy due to the occurrence of an adverse event
3) Not applicable: Adverse events occur before or after the start or completion of carbon ion radio therapy

8.9. Relationship to Protocol Procedures

The investigator must also provide an assessment of the relationship between SAEs and protocol procedures on the SAE Report Form. This includes both non-therapeutic emergencies (SAEs that occur prior to the administration of the investigational drug) and therapeutic emergency SAEs. Protocol-related SAEs may occur because of a required procedure or intervention (e.g., blood draw) during the clinical trial. The investigator should use the following guidelines to assess the relationship between SAEs and protocols

- Protocol-related: The event occurred because of a procedure or intervention described in the protocol for which no alternative etiology exists in the subject's medical record.
- Not protocol-related: The event is related to an etiology other than the procedure or intervention described in the protocol. The alternative etiology must be documented in the study subject's medical record.

In the case of AEs, AEs on treatment (or AEs appearing on treatment) are defined as AEs that began after or before administration and worsened after exposure to treatment between the start dose date and 90 days after discontinuation of study treatment.

8.10. Adverse events based on signs and symptoms

All AEs reported spontaneously by the subject or in response to questions from the investigator, subinvestigator, or collaborator (example of a visit: “Have you had any health problems since your last visit / have you been asked any questions since your last visit?”) “)

When collecting AEs, recording the diagnosis is preferred over recording a list of signs and symptoms, if possible. However, if the diagnosis is known and there are other signs and symptoms that are not generally part of the diagnosis, record the diagnosis and each sign or symptom separately.

8.11. Adverse events based on tests and examinations

Protocol-mandated laboratory values and vital signs measurements will be summarized in the CSR. Therefore, protocol-mandated worsening of laboratory values and vital signs relative to baseline should only be reported as an AE if it meets one of the SAE criteria or is a reason for discontinuation of treatment with the investigational drug.

If the worsening of laboratory values or vital signs is associated with clinical signs or symptoms, the signs or symptoms should be reported as an AE and the associated laboratory results or vital signs should be reported as additional information, as appropriate. Whenever possible, the reporter uses clinical terms rather than laboratory terms (e.g., “anemia” rather than “low hemoglobin level”). In the absence of clinical signs or symptoms, the worsening of such laboratory values should be reported as an AE.

Laboratory deterioration attributable to apparent disease progression shall not be considered an adverse event or serious adverse event.

Report as an adverse event any new or worsening clinically significant abnormal findings at the time of presentation compared to the baseline evaluation.

8.12. Hy's Law

Biochemical elevations suggestive of abnormal liver function may require further evaluation, and the occurrence of AST or ALT ≥ 3 x ULN and total bilirubin ≥ 2 x ULN may require reporting as an SAE. For cases of elevated liver biochemistry and Hy's Law evaluation, see Ref.

8.13. Disease progression

Disease progression is considered a worsening of the subject's condition due to the disease for which the investigational drug is being studied and is an increase in the severity of the disease under study and/or an increase in the symptoms of the disease. The development of new metastases or progression of existing metastases to the primary cancer being studied is considered disease progression and is not considered an AE. Events that are clearly attributable to disease progression should not be reported as AEs during a clinical trial.

8.14. New cancer

The development of new cancer shall be considered a serious adverse event. New cancers are those that occur after a patient is enrolled in the study, rather than those that were the primary reason for enrollment in the study. New metastatic lesions are considered progression of the cancer under study and are not reported as a second cancer.

8.15. Deaths

All deaths occurring during the investigational treatment period or within the protocol-defined follow-up period after the last dose of the investigational drug shall be reported as follows.

- Events that are clearly attributable to disease progression will not be treated as investigational AEs, but if the outcome of the event is found to be severe, all SAEs, not limited to death, will be reported, as described in Section 10.14.
- The investigator shall immediately report to the site director and the investigational drug provider, regardless of the causal relationship between the SAE and the investigational drug/IR, regardless of whether the death is due to progression of the disease under study. In addition, a note will be made in the eCRF.
- The causes are identified and described in the eCRF.
- Deaths of unknown cause should always be reported as SAEs.
- If an autopsy is performed, the autopsy results should be reported to AstraZeneca.

Deaths that occur after the last dose of the investigational drug and after the protocol-defined safety information collection period should also be noted in the eCRF as required. If the death is attributable to an event that occurred after the safety information collection period and the event is considered to be due to delayed toxicity to the investigational drug, it must also be reported as an SAE.

AstraZeneca retains the right to request additional information for subjects with ongoing AE(s)/SAE(s) at the end of the trial if deemed necessary.

8.16. Reportable Adverse Events

The Investigator or Co-Investigator will ensure that all adverse events that occurred from the time consent is obtained until 28 days after the end of study drug administration are consistently described in the case report form and that adverse events disappear or that the study period is observed until 4 weeks after the end of the study period (after discontinuation). Adverse events that are judged to have a causal relationship with the study drug will continue to be observed as much as possible until the end of the study period. However, this does not apply if the investigator determines that the safety of the subject is sufficiently ensured and further follow-up is not necessary.

8.17. Reporting of serious adverse events

All serious adverse events during the clinical trial (regardless of whether there is a causal relationship with the investigational drug or not) and serious adverse events suspected to be related to the investigational drug after the completion (discontinuation) of the clinical trial shall be reported according to the following procedures. The details of the reporting procedures are separately stipulated in the “Standard Operating Procedures for Handling Safety Information". An outline of the reporting procedure is provided below.

1） Reports from the investigator to the head of the site and to investigators at other sites

If any serious safety information is recognized during the clinical trial period, the investigator shall immediately report the details to the head of his/her institution, the investigational drug supplier, and the investigators at other sites using the “Report on Serious Adverse Events and Malfunctions” (Uniform Form 12, or the same detailed description form, if necessary). In reporting, the investigator shall specify whether or not the serious safety information is an event that cannot be predicted from the investigator’s brochures, etc.

2） Discussion between the investigator and the Clinical Trial Coordinating Committee

The investigator shall consult with the Trial Coordinating Committee and report his/her opinion as the investigator (including the necessity of reporting to the Minister of Health, Labour and Welfare) to the Trial Coordinating Committee. The investigator shall report his/her opinion (including the necessity of reporting to the Minister of Health, Labour and Welfare) to the Trial Coordinating Committee. If the Independent Data Monitoring Committee is consulted regarding the investigator's judgment, the opinion of the Independent Data Monitoring Committee shall be

followed.

3） Report to the Minister of Health, Labour and Welfare and the heads of other medical institutions

1 Report to the Minister of Health, Labor and Welfare

If the Clinical Trial Coordinating Committee determines that the case is subject to reporting as stipulated in Article 273 of the Enforcement Regulations of the Act on Quality, Efficacy and Safety Assurance of Pharmaceuticals, Medical Devices and Other Products (Pharmaceuticals and Medical Devices Law), it shall report to the Minister of Health, Labor and Welfare (the Pharmaceuticals and Medical Devices Agency, an independent administrative agency (PMDA)). In addition, when the Independent Data Monitoring Committee is consulted, it shall be notified of the contents of the report made to this authority.

2 Reporting to the heads of other implementing medical institutions

When a report is made to the Minister of Health, Labour and Welfare, the investigator of the other site shall report the contents of the “Failure/Infectious Case Report” obtained from the Trial Coordinating Committee to the head of his/her site as soon as possible.

4） Actions to be taken when additional information is obtained

When additional information regarding the adverse event is obtained, the investigator of the site where the adverse event occurred shall make an additional report to the head of the site as soon as possible, as well as to the Clinical Trial Coordinating Committee and the investigational drug supplier. The handling of such additional information shall be in accordance with the procedures described in 1) to 3) above, and reports shall be made to the NIH as necessary.

5） Annual report to the Minister of Health, Labor and Welfare

If the investigational drug has not been approved in Japan, or if the sponsor is not conducting a clinical trial, the person conducting the clinical trial shall submit an annual report to PMDA in accordance with Article 273 of the Ordinance for Enforcement of the Pharmaceutical Affairs Law.

The investigator will report all SAEs to AstraZeneca. The investigator will send a copy of the SAE report submitted to PMDA to the investigational drug provider in accordance with a separate Standard Operating Procedure (SOP). If any changes are made to the follow-up report, the following information will also be promptly sent to the investigational drug provider.

8.17.1. Response to subjects

When a serious adverse event is observed, the investigator or subinvestigator should immediately take appropriate measures, discontinue administration of the investigational drug to ensure the safety of the subject, and inform the subject if treatment for the adverse event becomes necessary. If an adverse event that was considered to be causally related to the investigational drug at the time of discontinuation has not yet recovered, observation will be continued as much as possible until the adverse event recovers or becomes mild, in principle. However, this does not apply when the investigator determines that the subject's safety has been sufficiently ensured and further follow-up is not necessary.

8.18. OTHER EVENTS REQUIRING REPORTING

8.18.1. Overdose

Use of durvalumab or tremelimumab in excess of the prescribed dose of the drug constitutes an overdose. Currently, there is no established treatment for durvalumab or tremelimumab overdose and no established symptoms of a possible overdose. Overdoses with associated AEs are recorded as an AE diagnosis or symptom in the relevant AE module of the eCRF. Overdoses without symptoms are only recorded and reported to the site investigator.

Overdoses of durvalumab or tremelimumab, with or without associated AEs/SAEs, must be recorded and reported to the site investigator. The investigator must report these to the investigational drug provider as described in the SOP.

8.18.2. Hepatic function abnormality

Abnormal liver function that meets Hy's Law criteria should be reported as an SAE regardless of the presence or absence of clinical symptoms. Hy's Law criteria are met if AST or ALT is at least 3 times the upper limit of normal (ULN) or TBL is at least 2 times the upper limit of normal at any time after initiation of treatment with the investigational drug, regardless of elevated ALP.

If it cannot be determined that there is no causal relationship to the investigational drug (e.g., due to cholelithiasis or bile duct obstruction), the investigator should report these events to the study provider in accordance with SOPs.

If the cause of the hepatic dysfunction is established and there is no causal relationship to the investigational drug, the investigator will decide whether to continue the subject's treatment based on the investigator's clinical judgment.

- If the cause of the liver function abnormality is not established, the subject's administration should be discontinued immediately. Follow-up investigations and inquiries will be initiated by the investigational site without delay.

The investigator will follow up on each reported event of liver function abnormality.

8.18.3. Pregnancy

All pregnancies and pregnancy outcomes should be reported to AstraZeneca and the site investigator, except in the following cases

- Pregnancy discovered before the subject received the investigational drug.
- Pregnancy of the male subject's female partner. (If the male subject is not restricted from having children.)

8.18.4. Exposure to pregnant woman

If a subject becomes pregnant during a clinical trial, the investigational drug should be discontinued immediately. Pregnancy itself shall not be considered an AE unless there is a suspicion that the investigational drug interfered with the effectiveness of the contraceptive. Birth defects or birth defects and spontaneous abortions should be reported and treated as SAEs. Uncomplicated elective abortions shall not be treated as AEs. The outcome of all pregnancies (spontaneous abortion, elective abortion, ectopic pregnancy, normal delivery, and congenital anomalies) shall be followed up and documented even if the clinical trial is discontinued. If pregnancy occurs during the clinical trial, the investigator shall notify AstraZeneca in accordance with the SOP.

8.18.5. Exposure to partner

Male subjects should refrain from sexual intercourse or sperm donation with their partners during the study and for 180 days after the last dose of durvalumab plus tremelimumab combination therapy or 90 days after the last dose of durvalumab monotherapy, whichever is longer. Pregnancy in the subject's partner is not considered an AE. However, the outcome of all pregnancies (spontaneous abortion, elective abortion, ectopic pregnancy, normal delivery, or congenital anomaly) occurring between the date of first dose and 180 days after the last dose of durvalumab + tremelimumab combination therapy or 90 days after the last dose of durvalumab

monotherapy, whichever is longer, will be followed up and documented if possible. Follow-up and documentation, if possible, is desirable. Upon receipt of a report of pregnancy, the investigator must obtain the consent of the subject's partner prior to obtaining any information regarding the pregnancy. Therefore, the study team should adopt the generic ICF template according to the procedure and submit it to the Institutional Review Board (IRB) prior to use.

8.19. Medication error

For this study, a medication error is an unintentional error in the course of treatment with an investigational drug that may cause harm to the subject. A medication error is not a lack of efficacy of the investigational drug, but an artificial or process-related failure while the investigational drug is under the control of the site staff or subject. Medication errors include the circumstances under which the error occurred.

- A medication error occurred.
- A medication error occurred, but was identified before the subject received the medication.
- No medication errors occurred, but circumstances were observed that could have resulted in errors.

Examples of events that should be reported in a clinical trial as medication errors

- Confusion of drug names
- Dispensing errors (e.g., some medications were incorrectly dispensed even though they were not actually administered to the subject.
- Drugs that were not administered as directed, such as by incorrect route or site of administration
- Drugs not taken as directed, such as tablets dissolved in water when they should be taken as solid tablets
- Drugs not stored as directed (e.g., in a refrigerator when they should be at room temperature.)
- Subjects who received medication by mistake
  Drugs administered to the wrong subject

Examples of events that need not to be reported as medication errors in clinical research

- Including those that lead to any of the above events or that result in a dose error.
- If the subject fails to take the medication (e.g., forgets to take the medication）
- Overdose Accidents
- Subject did not return unused or empty packaged medications
- Errors associated with background or rescue drugs, even AstraZeneca products, or standard of care drugs in open label studies

Medication errors are not considered AEs, but AEs may occur because of medication errors. If a medication error occurs during a clinical trial, the investigator or other site personnel should contact AstraZeneca within one day.

8.20. Predicted Side Effects

The following is a tabulation of adverse events that occurred in clinical trials of durvalumab. For other adverse events, please refer to the Investigators brochure.

（1）Information on adverse events in 2769 patients treated with durvalumab (all grades, incidence >5%)

Side effect	Occurrence frequency
Fatigue	769 （27.8 %）
anorexia	584 （21.1 %）
cough	529 （19.1 %）
Nausea	515 （18.6 %）

Breathe bitterly	500	(18.1 %)
constipation	473	(17.1 %)
diarrhea	467	(16.9 %)
fever	393	(14.2 %)
anaemia	348	(12.6 %)
Back pain	342	(12.4 %)
vomiting	339	(12.2 %)
Itching	314	(11.3 %)
lethargy	306	(11.1 %)
Joint pain	299	(10.8 %)
Hypothyroidism	269	(9.7 %)
rash	266	(9.6 %)
headache	265	(9.6 %)
Peripheral edema	258	(9.3 %)
sleeplessness	234	(8.5 %)
bellyache	219	(7.9 %)
Weight loss	205	(7.4 %)
Musculoskeletal pain	194	(7.0 %)
dizziness	189	(6.8 %)
pneumonia	186	(6.7 %)
Urinary tract infections	182	(6.6 %)
Muscle pain	173	(6.2 %)
Upper respiratory tract infections	163	(5.9 %)
AST elevation	162	(5.9 %)
Pain in the extremities	158	(5.7 %)
Hyponatremia	152	(5.5 %)
ALT elevation	149	(5.4 %)
Wet cough	142	(5.1 %)
Nasopharyngitis	140	(5.1 %)

（2）Information on adverse events in 1822 patients treated with durvalumab and tremelimumab in combination (all grades, incidence >5%)

Side effect	Occurrence frequency	
fatigue	542	(29.7 %)
diarrhea	485	(26.6 %)
anorexia	461	(25.3 %)
nousea	447	(24.5 %)
pruritic	395	(21.7 %)
constipation	365	(20.0 %)
dyspnea	361	(19.8 %)
anaemia	339	(18.6 %)
fever	305	(16.7 %)
vomiting	286	(15.7 %)
coughing	271	(14.9 %)
backache	247	(13.6 %)
rash	246	(13.5 %)
lethargy	219	(12.0 %)
abdominal pain	217	(11.9 %)
peripheral edema	203	(11.1 %)
weight loss	203	(11.1 %)
arthralgia	195	(10.7 %)
hypothyroidism	186	(10.2 %)

insomnia	175	(9.6 %)
AST elevation	172	(9.4 %)
hyponatremia	172	(9.4 %)
ALT elevation	154	(8.5 %)
headache	172	(8.3 %)
Lipase rise elevation	141	(7.7 %)
dizziness	134	(7.4 %)
amylase elevation	133	(7.3 %)
hypokalemia	131	(7.2 %)
pneumonia	125	(6.9 %)
maculopapular eruption	125	(6.9 %)
Urinary tract infections	117	(6.4 %)
ALP elevation	115	(6.3 %)
dehydration	114	(5.4 %)
Hyperthyroidism	110	(5.1 %)
hyperglycemia	109	(6.0 %)
γ-GTP elevation	103	(5.7 %)
Hypoalbuminemia	100	(5.5 %)
Musculoskeletal pain	99	(5.4 %)
Muscle pain	99	(5.4 %)
Dry skin	98	(5.4 %)
Wet cough	98	(5.4 %)
Xerostomia	95	(5.2 %)
musculoskeletal chest pain	93	(5.1 %)
upper respiratory tract infection	92	(5.0 %)

（3）Refer to Section 2.3.2.3 for more information on the risks of Carbon ion radio therapy.

9. ENDPOINT

9.1. Primary endpoint

Percentage of dose-limiting toxicities (DLT) and adverse events/serious adverse events

Rationale for Setting the Primary Endpoint<br><br>To evaluate the tolerability and safety of durvalumab tremelimumab in combination with carbon ion radio therapy in subjects with advanced hepatocellular carcinoma with vascular invasion.

9.2. Secondary endpoint

Overall survival (OS), 6-month survival, objective response rate (ORR) calculated by RECIST ver 1.1 and mRECIST, 6-month progression-free survival (PFS), Time to progression (TTP)

Rationale for Setting Secondary Endpoints<br><br>To evaluate the efficacy of durvalumab tremelimumab in combination with carbon ion radio therapy in subjects with advanced hepatocellular carcinoma with vascular invasion.

9.3. Exploratory endpoints

In addition to consent for this study, consent for “Exploratory Study of Tumor Cells and Tumor Microenvironment in Hepatocellular Carcinoma Using Tumor and Non-tumor Biopsy Specimens, Blood Specimens, and Stool Specimens” and “Exploratory Study of Tumor Cells and Tumor Microenvironment by Secondary Use of Biological Specimens in Hepatobiliary Pancreatic Cancer” and a liver biopsy or liver tumor biopsy specimen must be In patients with sufficient liver biopsy/tumor biopsy samples, biomarkers associated with clinical outcome of combination therapy with durvalumab/tremelimumab and carbon ion radio therapy will be explored using blood and liver biopsy/tumor biopsy samples. The analysis will be performed in the laboratory of the Department of Gastroenterology, Graduate School of Medicine, Chiba University, and may be contracted to an outside vendor or outside research organization.

10. STATISTICAL METHODS AND SAMPLE SIZE DETERMINATION

10.1. Description of Analysis set

10.1.1. DLT analysis set

All subjects enrolled in the study, who have completed at least one investigational drug regimen (IR), and for whom a DLT evaluation has been performed, are considered the DLT analysis set.

10.1.2. Safety analysis set: SAF

All subjects who have had at least one dose of the investigational drug or investigational drug regimen (IR) are considered in the Safety analysis set.

10.1.3. Full Analysis set: FAS

All subjects with eligible disease who are properly enrolled and have received at least one dose of the investigational drug regimen are considered Full Analysis set (FAS).

10.1.4 Efficacy Evaluable set: EES

The efficacy evaluation analysis(EES) population is defined as a subset of the FAS. That is, it consists of all subjects who are eligible for the trial, receive the study drug/IR, and have had at least one post-dose efficacy endpoint assessed. In addition to subjects with at least one baseline and at least one post-dose efficacy evaluation, the population will include cases of early death or early progression before the evaluation.

10.1.5 Per protocol set：PPS

Subjects from the FAS who do not have any of the following serious violations of the study protocol, including the study protocol, such as the method of study or concomitant therapy, will be considered to be in compliance with the study protocol (PPS).

- Violation of inclusive criteria
- Violation of exclusion criteria
- Violation of concomitant use of prohibited drugs
- Violation of concomitant use of prohibited therapies

10.2. Target number of cases and rationale for setting

Target cases: 3 to 15

A modified “3+3 design” will be used in this trial. Cohort A will be used to evaluate DLT for carbon ion radio therapy + durumab, and instead of dose escalation, the group with toremelimumab will be used as Cohort B to evaluate DLT.

10.3. Case Handling

The coordinating investigators and the statistical analyst will discuss and decide how to handle the registered cases. The coordinating investigator and the statistical analyst will also discuss and decide how to handle cases in case new problems arise.

10.4. Data Handling

In principle, the handling of data during data compilation and analysis shall be as follows. In case of doubt, the statistician and the coordinating Investigators shall discuss and decide the handling of the data. Details are described in the statistical analysis plan.

1) Missing values: Supplementation of missing values is not performed.
2) Reference values: Data with unreliable measured values due to hemolysis, etc. are not used in the aggregate analysis.
3) Time lag: Data not conducted during the specified observation period will not be used in the aggregate analysis for that period. For other data requiring consideration, the coordinating investigators will consult with medical experts to determine how to handle the data for analysis.

10.5. Statistical analysis items and analysis plan

All patients will be analyzed after completion of treatment with the investigational drug and after the data have been fixed, using the DLT evaluation population for DLT evaluation, the ESS analysis population for efficacy evaluation, and the safety analysis population for safety evaluation. For the efficacy evaluation using RECIST without survival analysis, FAS and PPS analysis and sensitivity analysis will be performed as necessary.

10.5.1. Analysis of subject background

The distribution of subject background data and summary statistics are calculated for each cohort. For nominal variables, frequencies and proportions of categories are shown. For continuous variables, summary statistics (number of cases, mean, standard deviation, median, range, and interquartile range) are calculated.

10.5.2. Analysis of primary endpoints

10.5.2.1. DLT Evaluation

1) DLT incidence rate

The number of DLT cases and incidence rate will be calculated for each cohort.

10.5.2.2. Safety Evaluation

1) Adverse event rate
2) Serious adverse event rate
3) Adverse events coded by MedDRA
4) List of SOC, PT, severity, relevance, etc.

10.5.3. Analysis of secondary endpoints

10.5.3.1 Efficacy Analysis

Overall survival (OS), 6-month survival rate, objective response rate (ORR), 6-month progression-free survival (PFS), and time to progression (TTP) will be determined as secondary parameters to evaluate efficacy in each cohort. For survival analysis, the Kaplan-Meier method will be used to determine the incidence rate at 6 months and the median and mean survival times using the ESS analysis group.

Overall Response Rate（ORR）

ORR (based on evaluation by investigators using RECIST 1.1) is defined as the percentage of patients with a CR or PR at one or more visits. Patients who had a response after discontinuation of treatment without PD and initiation of post-treatment are not included in the ORR response cases.

Progression-Free Survival（PFS）

PFS (based on evaluation using RECIST 1.1 by investigators and others) is defined as the time from allocation to objective disease progression or death (or cause of death in the absence of disease progression), regardless of whether the patient discontinued treatment or received other anticancer therapy before disease progression. Patients who have not progressed or died at the time of analysis will be censored at the date of the last RECIST 1.1-based evaluation. However, if a patient has progressed or died after two or more consecutive missed visits, the patient will be censored as of the date of the last RECIST 1.1-based evaluation. In the absence of evaluable visit data or baseline data, censor at Day 1 unless the patient has died between baseline and the second visit, in which case treat as an event with the date of death as the date of the event.

The PFS is always calculated based on the date the imaging study/evaluation was performed, not the date of the visit.

It is possible that an evaluation/imaging study based on RECIST 1.1 scheduled for a specific visit may be performed over several different days. In such cases, the following principles apply.

- For evaluation by the investigator, the earliest RECIST 1.1 evaluation/imaging test date when an element indicating progression is identified is the progression date.PFS
- When an evaluation is terminated, it shall be terminated on the last imaging inspection date of the inspection related to the specific overall effectiveness evaluation.

Time to Progression (TTP)

TTP (based on evaluation using RECIST 1.1 by the investigator and others) is defined as the period from the date of random assignment to the date of objective tumor progression. Death is not included in the definition of TTP. Death without progression is not included in the definition of TTP.

Patients who die without progression will be terminated at the time of death.

Image Evaluation

Imaging evaluation For all imaging evaluations, in addition to evaluation by the investigator or subinvestigators, a central judgment will be made by multiple radiologists to be separately determined.

10.5.4. Interim Analysis

No interim analysis will be performed in this clinical trial.

10.6. Final Analysis

After the follow-up period, analysis will be conducted after the data are obtained and the cases are fixed. The person in charge of statistical analysis compiles the “Analysis Report” and submits it to the coordinating investigator and the principal investigator. The coordinating investigator summarizes the contents of the analysis report and prepares a “summary report” summarizing the overall conclusions of the trial, problems, interpretation and discussion of the results, and future policies mainly from a clinical perspective, and obtains approval from the principal investigator.

10.7. Data Monitoring Committee

An Independent Data Monitoring Committee will be established for this clinical trial. The Independent Data Monitoring Committee will be established as an independent body from the investigators and will consist of three or more expert members who are independent of the study. The Independent Data Monitoring Committee will be established for the purpose of ensuring the safety of subjects. It will provide appropriate advice and recommendations in accordance with a separate protocol If the study is terminated before the end of the DLT evaluation period for reasons other than DLT criteria, the investigator will ask the Independent Data Monitoring Committee for its opinion on the addition of a case If a second DLT occurs, the investigator will ask the Independent Data Monitoring Committee to determine whether the study regimen is If a second DLT occurs, the investigator will ask the Independent Data Monitoring Committee for an opinion on whether the study regimen is intolerable. If necessary, the investigator will confirm whether tolerability was not an issue in the other cases.

If an SAE is reported during the trial, the investigator will check with the Independent Data Monitoring Committee whether to continue the trial and whether any changes should be made to the protocol. The Independent Data Monitoring Committee will provide the investigator with the results of the discussion in writing.

If the investigator determines that precautionary emergency measures are warranted, depending on the importance and scope of the report, actions may include suspension of enrollment and emergency communication to all participating sites.

11. COMPLIANCE AND DEVIATION FROM THE PROTOCOL

1) The investigator or subinvestigator shall conduct the clinical trial in compliance with this protocol.
2) The investigator or subinvestigator shall record the details and reasons for all deviations from the study protocol.
3) In the event of deviation from the protocol for the purpose of avoiding immediate danger to subjects or for other unavoidable medical reasons, the investigator shall immediately submit a document describing the details of the deviation and the reasons for it to the head of the implementing medical institution, and shall also promptly report the contents of said document to the Trial Review Committee via the head of the implementing medical institution. The contents of said documents shall be promptly reported to the Clinical Trial Review Committee via the head of the investigational institution.

12. CHANGES TO THE CLINICAL TRIAL PROTOCOL, CASE REPORT FORM, OR ANALYSIS PLAN

12.1. Revision of Clinical Trial Protocol and Case Report Form

The following procedures shall be followed when revising the clinical trial protocol and case report forms.

1) When the investigator becomes aware of matters related to the quality, efficacy and safety of the investigational drug or other information important for the proper conduct of the clinical trial, the investigator shall revise the relevant protocol as necessary. When a revision is made, a history of the revision shall be prepared and stored.
2) The coordinating investigator shall revise the case report form as necessary in conjunction with the revision of the protocol or for other reasons. Whenever necessary, the coordinating investigator shall revise the case report form in conjunction with the revision of the protocol or for other reasons.
3) The investigator shall promptly submit the revised protocol and revised case report form to the head of the site and obtain approval from the investigational review committee via the head of the site.
4) The same procedure shall be followed when the investigator amends the protocol and case report form within the scope of the investigator's acceptable instructions from the head of the site based on the opinion of the investigational review committee.

12.2. Changes in statistical analysis plan

If the statistical analysis manager changes the contents of the statistical analysis plan, all changes shall be documented in the statistical analysis report for this clinical trial. In addition, the circumstances of any changes to the statistical analysis plan shall be recorded.

13. DISCONTINUATION, SUSPENSION, OR TERMINATION OF THE CLINICAL TRIAL

13.1. Criteria for discontinuation or suspension of the clinical trial as a whole

When the following information is obtained and it is considered difficult to continue the entire clinical trial, the coordinating investigator will consult with the principal investigator and make a decision to discontinue or suspend the entire clinical trial.

- When it becomes difficult to ensure the safety of this clinical trial due to new safety information or serious adverse event information concerning the investigational drug, etc.
- When the site has committed a serious violation of the drug GCP ordinance or a serious deviation from the clinical trial protocol and no improvement has been made.
- Other new information obtained during the conduct of the clinical trial that may necessitate discontinuation or suspension of the clinical trial.

13.2. Procedures for discontinuation or suspension of a clinical trial as a whole

If the coordinating investigator, after consultation with other investigators, decides to discontinue or suspend the entire clinical trial, he/she shall promptly notify the head of the investigational institution and the regulatory authorities in writing to that effect and the reasons in detail. In addition, the investigator shall promptly inform the subjects undergoing the clinical trial and take appropriate measures such as changing to appropriate treatment.

13.3. Procedures for discontinuation or suspension of this clinical trial at an individual clinical site

When the investigator discontinues or suspends a clinical trial, the investigator shall promptly notify the head of the site in writing to that effect and explain the details of the discontinuation or suspension in writing in detail. When notified of the discontinuation or suspension of a clinical trial, the investigator shall promptly notify in writing all investigators and regulatory authorities involved in the said clinical trial to that effect and explain the discontinuation or suspension in detail.

13.4. Termination of this clinical trial

After the completion of the clinical trial, the investigator shall notify the head of the investigational institution in writing that the clinical trial has been completed and report a summary of the results of the clinical trial in writing.

14. DATA MANAGEMENT

14.1. Data management procedure

Detailed procedures for data management shall be described in the data management plan.

14.2. Data Collection

The investigator or sub-investigator shall prepare a case report using Electronic Data Capture (EDC) that meets the requirements of 21 CFR Part 11, the Pharmaceutical GCP Ordinance, and the ER/ES guidelines. The investigator or subinvestigator shall prepare a case report using EDC. The investigator or subinvestigator shall make any changes, corrections or additions to the contents of the case report form on the EDC that generated the case report form, and record all of the changes, corrections or additions as electronic information. When a subinvestigator prepares a case report form or when a collaborator transcribes a case report form from source documents (source data), the investigator shall check the contents of the case report form before submitting it to the EDC and confirm that there are no problems. The investigator provides the final electronic case report form to the institution on an electronic medium (CD-R, etc.). The investigator shall ensure the readability and archivability of the electronic case report form.

When using the EDC system, the site should receive training on EDC and refer to the manual for details on how to input data.

14.3. Identification of documents that are directly described in the case report and should be interpreted as source documents (source data)

In this clinical trial, the following documents and others shall be considered source documents (source data).

1） Medical records, nursing records, clinical laboratory data, imaging films, and other records that form the basis for preparing case reports. Data stored in electronic medical records are also considered source documents.
2） Records of investigational drug administration
3） Documents or records related to the clinical trial that are required under the GCP ordinance for pharmaceutical products related to the clinical trial

Of the data described in the case report form, the following items shall be regarded as source documents (source data) when they are described in the case report form. However, if the data are recorded in the medical record, the medical record shall be regarded as the source documents (original data).

1） Purpose of concomitant medications/adjunctive therapy
2） Determination of the extent of adverse events, outcomes (including results at follow-up), severity, and causal relationship to the study treatment and the basis for the determination
3） Reasons for discontinuation of clinical trials by subjects
4） Comments by the investigator or subinvestigator

15. RETENTION OF SOURCE DOCUMENTS AND OTHER RECORDS

15.1. Retention of records by the clinical site

Documents or records pertaining to a clinical trial to be retained at the investigational site as stipulated in the Pharmaceutical GCP ordinance shall be retained by the hospital director until the later of the following dates.

1) The date on which five years have elapsed since the date of marketing approval for the relevant indication of the test product (if development has been discontinued, the date on which three years have elapsed since the date on which the decision to discontinue

development was made). However, for drugs that are subject to post-approval reexamination in accordance with the provisions of the "Law Concerning Quality, Efficacy and Safety Assurance of Pharmaceuticals and Medical Devices" and for which the period until the reexamination is completed exceeds five years, the date on which the reexamination is completed.
2) The date on which 3 years have elapsed since the discontinuation or termination of the clinical trial.

The investigator shall notify the clinicalsite when the records to be retained by the investigational site or the investigational review committee are no longer required to be retained.

15.2. Retention of records by principal investigators

Documents or records pertaining to clinical trials to be retained by principal investigators as stipulated in the Pharmaceutical GCP Ordinance shall be retained at a storage location deemed appropriate until the later of the following dates.

1) Three years have elapsed since the date of marketing approval for the relevant indication of the test product (if development has been discontinued, three years have elapsed since the date on which the decision to discontinue development was made).
2) The date on which 3 years have elapsed since the discontinuation or termination of the clinical trial.

16. RETENTION OF SAMPLES AND USE OF SAMPLES PROVIDED BY OTHER INSTITUTIONS

16.1. Retention of sample

Samples will be stored in the Laboratory of Gastroenterology, Graduate School of Medicine, Chiba University for a period not exceeding 20 years after completion of the clinical trial. The method of preservation shall be cryopreservation using liquid nitrogen.

16.2. Disposal of samples

If a subject withdraws consent, if a specimen is mistaken or contaminated or is strongly suspected of being mistaken or contaminated, or if the need for disposal is otherwise recognized, the anonymizing numbers, etc., will be deleted and the specimen will be disposed of.

16.3. Reuse of samples

Secondary use of samples and sample-related information (genomic or epigenomic analysis at Chiba University) may be conducted. In such cases, the ethical review and method of obtaining consent shall be in accordance with the corresponding ethical guidelines, etc.

17. SOURCE DOCUMENT VERIFICATION

The head of the investigational site and the investigator ensure that the personnel in charge of monitoring, audits and Institutinal Review Board or regulatory authorities have access to all records, including source documents. In anddtion , The head of the investigational site and the investigator confirm that the clinical trial is conducted appropriately and that the data are sufficiently reliable. The method and timing of source document verification shall be specified separately in the monitoring procedures.

18. Quality Assurance

In order to ensure that clinical trials are conducted and that data preparation, recording and reporting are appropriately conducted in compliance with the protocol and the GCP ordinances for pharmaceutical products, independent auditors from the departments related to the clinical trials, including the department in charge of monitoring, will conduct audits at the investigational

site and other sites where the clinical trials are conducted to confirm that quality control is appropriately conducted.The audit shall be conducted by an auditor independent from the departments related to the clinical trial, including the department in charge of monitoring, to confirm that quality control is appropriately implemented. Audits shall be conducted in accordance with the “Standard Operating Procedures for Audits” and “Audit Plan” separately stipulated.

19. QUALITY CONTROL FOR THIS STUDY

19.1 Training of study site personnel

The Principal Investigator will ensure that appropriate training relevant to the study is given to all of these staff, and that any new information relevant to the performance of this study is forwarded to the staff involved.

19.2. Quality control

In conducting clinical trial monitoring, the investigator shall consider priorities and develop a systematic risk-based approach. In the event of any deviation from this protocol, the investigator or subinvestigator shall follow the provisions of this protocol. The investigator or subinvestigator shall prepare the case report form in accordance with this protocol. The investigator will ensure that all data and other records in the case report form are accurate and complete. If any of the data in the case report form is inconsistent in any way with the original data, the investigator shall prepare and maintain a record explaining the reason for the inconsistency. The investigator shall designate a person who is not engaged in the relevant clinical trial at the site subject to the monitoring as a monitor and have him/her conduct the monitoring in accordance with the monitoring protocol that has been reviewed by the investigational review committee. The monitors shall confirm the following items in accordance with the monitoring protocol separately prepared.

- The human rights, safety and welfare of subjects are protected.
- The clinical trial is conducted in compliance with the Pharmaceutical GCP ordinance, the latest clinical trial protocol, and the procedure manual for the relevant clinical trial.
- To confirm that the data, etc. reported by the investigator or subinvestigator are accurate and complete and check them against the source documents and other clinical trial-related records.

The person in charge of data management shall formulate the data management plan in accordance with the separately established standard operating procedures and shall ensure the quality of the data through quality control at each stage of data handling.

20. ETHICAL CONDUCT AND GOOD CLINICAL PRACTICE(GCP)

This clinical trial will be conducted in accordance with the “Declaration of Helsinki”,the “Law Concerning Quality, Efficacy and Safety Assurance of Pharmaceuticals and Medical Devices” and the “Pharmaceutical GCP Ministerial Ordinance”. In addition, this clinical trial shall be conducted in compliance with the protocol and procedures for this clinical trial.

In selecting subjects, the investigator or subinvestigator shall carefully consider the appropriateness of requesting subjects to participate in this clinical trial based on the selection criteria and exclusion criteria from the perspective of protecting human rights, taking into consideration the subjects' health condition, symptoms, age, gender, ability to consent, degree of dependence on the investigator, and participation status in clinical trials including other clinical trials.

21. INSTITUTIONAL REVIEW BOARD(IRB)

Prior to the implementation of this clinical trial, the Institutional Review Board of the site will review the ethical, scientific and medical appropriateness of this clinical trial. This clinical trial shall be conducted after obtaining the approval of the Clinical Trial Review Committee. If the result of the deliberation by the Institutional Review Board is “Approval with modification,” the protocol or case report form, consent document, etc. shall be modified and approved based on the result of the deliberation, and then this clinical trial shall be conducted. The Institutional Review Board shall also continuously review whether or not this clinical trial is being conducted appropriately at least once a year.

22. HEALTH DAMAGE COVERAGE AND INSURANCE

If a subject suffers health problems as a result of participation in this clinical trial, the investigator will provide treatment and appropriate medical care for the subject's recovery.

As a response to liability for compensation and indemnification arising from health damage caused by this clinical trial, the investigators, subinvestigators, medical institution, Clinical Trial Coordinating Committee and other parties involved in this clinical trial will be covered by the Physician-initiated Clinical Trial Insurance.

23. COST BURDEN FOR THIS TRIAL

The investigational drug to be used in this study will be provided by AstraZeneca. Payment of the burden reduction fee and other expenses to subjects will be in accordance with the rules separately established by each investigational site.

24. TRIAL FUNDS AND CONFLICT OF INTEREST

This clinical trial will be conducted with funds provided to Chiba University Hospital by AstraZeneca. AstraZeneca personnel will not be involved in the conduct or analysis of this clinical trial when the investigators conduct the clinical trial.

In addition, prior to the deliberation of the Clinical Trial Review Committee at each site, the Conflict of Interest Management Committee will deliberate whether conflicts of interest are being properly managed, and it will be confirmed that the investigators and collaborators are not in a state of conflict of interest.

25. PROVISION OF THE INVESTIGATIONAL PRODUCTS AND INTELLECTUAL PROPERTY RIGHTS

In this study, the investigational products are provided by AstraZeneca Pharmaceuticals, Inc. (England) The ownership of the rights to any inventions, discoveries, or improvements of any nature (the “Inventions”) derived from this study will be decided in accordance with the contracts with AstraZeneca.

26.PUBLICATION

The results of the clinical trial will be submitted as a report by the Principal Investigator to the head of the clinical trial site upon completion of this clinical. Results that do not meet expected outcomes despite the proper conduct of the clinical trial must also be disclosed in publication. In case of disclosure of the results to the public, the subject’s personal information must be kept confidential.

27. REGISTRATION FOR CLINICAL TRIAL

This clinical trial will be registered in Japan Registry of Clinical Trial(jRCT) (https://jrct.niph.go.jp/) prior to obtaining consent from the first subject.

28. STUDY IMPLEMANTATION GRPUP

See ANNEX TO PROTOCOL 1

29. LIST OF INVESTIGATIONAL PRODUCT(S) FOR THIS STUDY

Investigational product	Dosage form and strength	Manufacturer
Durvalumab	*50 mg/mL solution for infusion after dilution*	MedImmune/AstraZeneca
Tremelimumab	*20 mg/mL solution for infusion after dilution*	MedImmune/AstraZeneca

30. LIST OF REFERENCES

1) Ministry of Health, Labor and Welfare 2014 Patient Survey
2) Ministry of Health, Labor and Welfare 2017 Vital Statistics
3) El Serag HB, et al., Hepatocellular Carcinoma N Engl J Med 2011
4) The Japan Society of Hepatology Clinical Practice Guidelines for Hepatocellular Carcinoma
5) Llovet JM, Ricci S, Mazzaferro V, et al. Sorafenib in advanced hepatocellular carcinoma. N Engl J Med 2008; 359: 378-90.
6) Cheng AL, Kang YK, Chen Z, et al. Efficacy and safety of sorafenib in patients in the Asia-Pacific region with advanced hepatocellular carcinoma: a phase III randmised, double- blind, placebo-controlled trial. Lancet Oncol 2009; 10:25-34.
7) Bruix J, Qin S, Merle P, et al. Regorafenib for patients with hepatocellular carcinoma who progressed on sorafenib treatment (RESORCE): a randmised, double-blind, placebo-controlled, phase 3 trial. Lancet 2017; 389: 56-66.
8) Kudo M, Finn RS, Qin S, et al. Lenvatinib versus sorafenib in first-line treatment of patients with unresectable hepatocellular carcinoma: a randomised phase 3 non-inferiority trial. Lancet 2018 Mar 24, 391 (10126): 1163-1173
9) Zhu AX, Kan YK, Yen CJ, et al. Ramucirumab after sorafenib in patients with advanced hepatocellular carcinoma and increased α-fetoprotein concentrations (REACH-2): a randomised, double-blind, placebo-controlled, phase 3 trial. Lancet Oncol.2019 Feb;20(2):282-296.
10) Bonze D, Meirson T, Azoulay D. Atezolizumab and Bevacizumab in Hepatocellular Carcinoma. NEJM. 2020 Aug 13;383(7):693-694.
11) Costentin CE, Ferroone CR, Arellano RS, et al. Hepatocellular Carcinoma with Macrovascular Invasion: Defining the Optimal Treatment Strategy Liver Cancer 2017; Nov;6(4):360-374.
12) Dunn GP, Old LJ, Schreiber RD. The three Es of cancer immunoediting. Annu Rev Immunol 2004;22:329-60.
13) Keir ME, Butte MJ, Freeman GJ, Sharpe AH. PD-1 and its ligands in tolerance and immunity. Annu Rev Immunol. 2008;26:677-704.
14) Okazaki T, Honjo T. PD-1 and PD-1 ligands: from discovery to clinical application. Int Immunol 2007;19(7):813-824.
15) Qin A, Coffey DG, Warren EH, Ramnath N. Mechanisms of immune evasion and current status of checkpoint inhibitors in non-small cell lung cancer. Cancer Med 2016;9:2567–2578.
16) Pardoll DM. The blockade of immune checkpoints in cancer immunotherapy. Nat Rev Cancer 2012;12:252-64.
17) Brahmer JR, Tykodi SS, Chow LQM, Hwu WJ, Topalian SL, Hwu P, et al. Safety and activity of

anti-PD-L1 antibody in patients with advanced cancer. N Engl J Med. 2012 Jun;366 (26):2455-65.

18) Hirano F, Kaneko K, Tamura H, Dong H, Wang S, Ichikawa M, et al. Blockade of B7-H1 and PD-1 by monoclonal antibodies potentiates cancer therapeutic immunity. Cancer Res. 2005;65(3):1089-96.

19) Iwai Y, Ishida M, Tanaka Y, Okazaki T, Honjo T, Minato N. Involvement of PD-L1 on tumor cells in the escape from host immune system and tumor immunotherapy by PD-L1 blockade. Proc Natl Acad Sci USA. 2002 Sep 17;99:12293-7.

20) Okudaira K, Hokari R, Tsuzuki Y, Okada Y, Komoto S, Watanabe C, et al. Blockade of B7-H1 or B7-DC induces an anti-tumor effect in a mouse pancreatic cancer model. Int J Oncol. 2009 Sep;35(4):741-9.

21) Topalian SL, Hodi FS, Brahmer JR, Gettinger SN, Smith DC, McDermott DF, et al. Safety, activity, and immune correlates of anti-PD-1 antibody in cancer. N Engl J Med. 2012;366:2443-54.

22) Zhang C, Wu S, Xue X, Li M, Qin X, Li W, et al. Anti-tumor immunotherapy by blockade of the PD-1/PD-L1 pathway with recombinant human PD-1-IgV. Cytotherapy. 2008;10(7):711-9.

23) Powles T, Eder JP, Fine GD, Braiteh FS, Loriot Y, Cruz C, et al. MPDL3280A (anti-PD-L1) treatment leads to clinical activity in metastatic bladder cancer. Nature. 2014 Nov 27;515(7528):558-62.

24) Rizvi N, Brahmer J, Ou S-H, Segal NH, Khleif SN, Hwu WJ. Safety and clinical activity of MEDI4736, an anti-programmed cell death-ligand-1 (PD-L1) antibody, in patients with nonsmall cell lung cancer (NSCLC). J Clin Oncol 2015;33:Abstract 8032.

25) Segal NH, Ou S-HI, Balmanoukian AS, Fury MG, Massarelli E, Brahmer JR, et al. Safety and efficacy of MEDI4736, an anti-PD-L1 antibody, in patients from a squamous cell carcinoma of the head and neck (SCCHN) expansion cohort. J Clin Oncol 2015;33:Abstract 3011.

26) Alexandrov LB, Nik-Zainal S, Wedge DC, Aparicio SAJR, Behjati S, Blankin AV, et al. Signatures of mutational processes in human cancer. Nature. 2013 Aug 22;500:415-21.

27) Fife BT, Bluestone JA. Control of peripheral T-cell tolerance and autoimmunity via the CTLA-4 and PD-1 pathways. Immunol Rev. 2008;224:166-82.

28) El-Khoueiry AB, Sangro B, Yau T, et al. Nivolumab in patients with advanced hepatocellular carcinoma (CheckMate 040): an open-label, non-comparative, phase 1/2 dose escalation and expansion trial Lancet 2017; Jun 24;389(10088):2492-2502.

29) Zhu AX, Finn RS, Edeline J, et al. Pembrolizumab in patients with advanced hepatocellular carcinoma previously treated with sorafenib (KEYNOTE-224): a non-randomised, open-label phase 2 trial. Lancet Oncol. 2018; Jul;19(7):940-952.

30) Kudo M. Targeted and immune therapies for hepatocellular carcinoma: Predictions for 2019 and beyond. World J Gastroenterol 2019 Feb 21;25(7):789-807.

31) Kelly RK, Abou-Alfa GK, Bendell JC, et al. Phase I/II study of durvalumab and tremelimumab in patients with unresectable hepatocellular carcinoma (HCC): Phase I safety and efficacy analyses. J Clin Oncol.2017; Abstract 4073.

32) Pedroni E, Bacher R, Blattmann H, Böhringer T, Coray A, Lomax A, Lin S, Munkel G, Scheib S, Schneider U, et al. The 200-MeV proton therapy project at the Paul Scherrer Institute: conceptual design and practical realization. Med Phys. 1995 Jan;22(1):37-53.

33) Mohamad O., Makishima H., Kamada T. Evolution of Carbon Ion Radiotherapy at the National Institute of Radiological Sciences in Japan. Cancers (Basel) 2018;10(3): pii: E66

34) Kasuya G, Kato H, Yasuda S, Tsuji H, Yamada S, Haruyama Y, Kobashi G, Ebner DK, Okada NN, Makishima H, Miyazaki M, Kamada T, Tsujii H; Liver Cancer Working Group. Progressive

hypofractionated carbon-ion radiotherapy for hepatocellular carcinoma: Combined analyses of 2 prospective trials. Cancer. 2017;123(20):3955-3965

35) Fukuda K, Okumura T, Abei M, Fukumitsu N, Ishige K, Mizumoto M et al. Long-term outcomes of proton beam therapy in patients with previously untreated hepatocellular carcinoma. Cancer Sci. 2017;108(3):497-503

36) Sorin Y, Ikeda K, Kawamura Y, Fujiyama S, Kobayashi M, Hosaka T. Effectiveness of Particle Radiotherapy in Various Stages of Hepatocellular Carcinoma: A Pilot Study. Liver Cancer. 2018 Oct;7(4):323-334

37) Igaki H, Mizumoto M, Okumura T, Hasegawa K, Kokudo N, Sakurai H. A systematic review of publications on charged particle therapy for hepatocellular carcinoma. Int J Clin Oncol. 2018;23(3):423-433

38) Komatsu S, Fukumoto T, Demizu Y, Miyawaki D, Terashima K, Niwa Y, Mima M, Fujii O, Sasaki R, Yamada I, Hori Y, Hishikawa Y, Abe M, Ku Y, Murakami M. The effectiveness of particle radiotherapy for hepatocellular carcinoma associated with inferior vena cava tumor thrombus. J Gastroenterol. 2011 Jul;46(7):913-20.

39) Lee SU, Park JW, Kim TH, Kim YJ, Woo SM, Koh YH, Lee WJ, Park SJ, Kim DY, Kim CM. Effectiveness and safety of proton beam therapy for advanced hepatocellular carcinoma with portal vein tumor thrombosis. Strahlenther Onkol. 2014 Sep;190(9):806-14.

40) Sugahara S, Nakayama H, Fukuda K, Mizumoto M, Tokita M, Abei M, Shoda J, Matsuzaki Y, Thono E, Tsuboi K, Tokuuye K. Proton-beam therapy for hepatocellular carcinoma associated with portal vein tumor thrombosis. Strahlenther Onkol 2009;185:782–788.

41) Antonia SJ, Daniel VD, Vicente D, et al. Durvalumab after Chemoradiotherapy in Stage III Non-Small-Cell Lung Cancer. N Engl J Med. 377;1919-29. 2017

42) Rizvi NA, Cho BC, Reinmuth N, et al. Durvalumab with or without tremelimumab vs platinum-based chemotherapy as first-line treatment for metastatic non-small cell lung cancer: MYSTIC Annals of Oncology.2018 Dec 1;29;supp10

43) Helm A, Ebner DK, Tinganelli W, Simoniello P, Bisio A, Marchesano V, Durante M, Yamada S, Shimokawa T. Combining Heavy-Ion Therapy with Immunotherapy: An Update on Recent Developments. Int J Part Ther. 2018 Summer;5(1):84-93.

44) Takahashi Y, Yasui T, Minami K, Tamari K, Hayashi K, Otani K, Seo Y, Isohashi F, Koizumi M, Ogawa K. Carbon ion irradiation enhances the antitumor efficacy of dual immune checkpoint blockade therapy both for local and distant sites in murine osteosarcoma. Oncotarget. 2019 Jan 18;10(6):633-646.

45) Iijima M, Okonogi N,Izumi Nakajima N, Morokoshi Y, Kanda H, Yamada T, Kobayashi Y, Banno K, Wakatsuki M, Yamada S, Kamada T, Aoki D, Hasegawa S. Significance of PD-L1 expression in carbon-ion radiotherapy for uterine cervical adeno/adenosquamous carcinoma. J Gynecol Oncol. 2020;31:e19.

46) Golden EB, Pellicciotta I, Demaria S, et al. The convergence of radiation and immunogenic cell death signaling pathways. Front Oncol, 2012 Aug 7;2:88.

47) Onishi M, Okonogi N, Oike T, Yoshimoto Y, Sato H, Suzuki Y, Kamada T, Nakano T. High linear energy transfer carbon-ion irradiation increases the release of the immune mediator high mobility group box 1 from human cancer cells. J Radiat Res. 2018 Sep 1;59(5):541-546.

48) Matsunaga A, Ueda Y, Yamada S, Harada Y, Shimada H, Hasegawa M, Tsujii H, Ochiai T, Yonemitsu Y. Carbon-ion beam treatment induces systemic antitumor immunity against murine squamous cell carcinoma. Cancer. 2010 Aug 1;116(15):3740-8.

49) Ando K, Fujita H, Hosoi A, Ma L, Wakatsuki M, Seino KI, Kakimi K, Imai T, Shimokawa T,

Nakano T. Intravenous dendritic cell administration enhances suppression of lung metastasis induced by carbon-ion irradiation. J Radiat Res. 2017 Jul 1;58(4):446-455.

50) Sato H, Niimi A, Yasuhara T, Permata TBM, Hagiwara Y, Isono M, Nuryadi E, Sekine R, Oike T, Kakoti S, Yoshimoto Y, Held KD, Suzuki Y, Kono K, Miyagawa K, Nakano T, Shibata A. DNA double-strand break repair pathway regulates PD-L1 expression in cancer cells. Nat Commun. 2017 Nov 24;8(1):1751. doi: 10.1038/s41467-017-01883-9.

51) Oike T, Niimi A, Okonogi N, Murata K, Matsumura A, Noda SE, Kobayashi D, Iwanaga M, Tsuchida K, Kanai T, Ohno T, Shibata A, Nakano T. Visualization of complex DNA double-strand breaks in a tumor treated with carbon ion radiotherapy. Sci Rep. 2016 Mar 1;6:22275. doi: 10.1038/srep22275.

52) Stewart R, Morrow M, Hammond SA, Mulgrew K, Marcus D, Poon E, et al. Identification and characterization of MEDI4736, an antagonistic anti-PD-L1 monoclonal antibody. Cancer Immunol Res 2015;3(9):1052-62.

53) Tarhini AA, Kirkwood JM. Tremelimumab (CP-675,206): a fully human anticytotoxic T lymphocyte-associated antigen 4 monoclonal antibody for treatment of patients with advanced cancers. Expert Opin Biol Ther 2008;8:1583-93.

54) Pardee AD, Butterfield LH. Immunotherapy of hepatocellular carcinoma: Unique challenges and clinical opportunities. Oncoimmunology 2012;1(1):48-55.

55) Gao Q, Wang XY, Qiu SJ, Yamato I, Sho M, Nakajima Y, et al. Overexpression of PD-L1 significantly associates with tumor aggressiveness and postoperative recurrence in human hepatocellular carcinoma. Clin Cancer Res 2009;15(3):971-9.

56) Hato T, Goyal L, Greten TF, Duda DG, Zhu AX. Immune checkpoint blockade in hepatocellular carcinoma: current progress and future directions. Hepatology 2014;60(5):1776-82.

57) Miroux C, Vausselin T, Delhem N. Regulatory T cells in HBV and HCV liver diseases: implication of regulatory T lymphocytes in the control of immune response. Expert Opin Biol Ther 2010;10(11):1563-72.

58) Golden-Mason L, Palmer B, Klarquist J, Mengshol JA, Castelblanco N, Rosen HR. Upregulation of PD-1 expression on circulating and intrahepatic hepatitis C virus-specific CD8+ T cells associated with reversible immune dysfunction. J Virol 2007;81(17):9249-58.

59) Peng G, Li S, Wu W, Tan X, Chen Y, Chen Z. PD-1 upregulation is associated with HBV-specific T cell dysfunction in chronic hepatitis B patients. Mol Immunol 2008;45(4):963-70.

60) Klein JP, Logan B, Harhoff M, Andersen PK. Analyzing survival curves at a fixed point in time. Stat Med 2007;26(24):4505-19.

61) Sangro B, Gomez MC, Mata M, et al. A clinical trial of CTLA-4 blockade with tremelimumab in patients with hepatocellular carcinoma and chronic hepatitis Cq. J Hepatol. 2013 Jul;59(1):81-8.

62) Duffy AG, Ulahannan SV, Makorova-Rusher O, Rahmer O, Wedemeyer H, Pratt D, et al. Tremelimumab in combination with ablation in patients with advanced hepatocellular carcinoma. J Hepatol 2017;66(3):545-51.

63) Melero I, Sangro B, Cheung Yae T, Hsu C, Kudo M, Crocenzi TS, et al. Nivolumab dose escalation and expansion in patients with advanced hepatocellular carcinoma (HCC): The CheckMate 040 study. J Clin Oncol 2017;35 (suppl 4S:abstract 226).

64) Kelly RK, Sangro B, Harris W, et al. Efficacy, tolerability, and biologic activity of a novel regimen of tremelimumab in combination with durvalumab for patients with advanced hepatocellular carcinoma, J Clin Oncol. 2020; Abstract 4508.

65) Takahashi Y, Fujikawa K, Sagawa T, et al. A phase 1 study to assess the safety and tolerability of tremelimumab alone and in combination with MEDI4736 in Japanese patients withadvanced solid

malignancies. Eur J Cancer. 2015(suppl 3; abst512)

66) Wang E, Kang D, Bae KS, Marshall MA, Pavlov D, Parivar K. Population pharmacokinetic and pharmacodynamics analysis of tremelimumab in patients with metastatic melanoma. J Clin Pharmacol 2014;54(10):1108-16.

67) Narwal R, Roskos LK, Robbie GJ. Population pharmacokinetics of sifalimumab, an investigational anti-interferonalpha monoclonal antibody, in systemic lupus erythematosus. Clin Pharmacokinet 2013;52:1021–27.

68) Ng CM, Lum BL, Gimenez V, Kelsey S, Allison D. Rationale for fixed dosing of pertuzumab in cancer patients based on population pharmacokinetic analysis. Pharm Res 2006;23(6):1275–84.

69) Wang DD, Zhang S, Zhao H, Men AY, Parivar K. Fixed dosing versus body size based dosing of monoclonal antibodies in adult clinical trials. J Clin Pharmacol 2009;49(9):1012–24.

70) Wolchok JD, Kluger H, Callahan MK, Postow MA, Rizvi NA, Lesokhin AM, et al. Nivolumab plus ipilimumab in advanced melanoma. N Engl J Med 2013;369:122-33.

71) Reed GB Jr, Cox AJ Jr. The human liver after radiation injury. A form of veno-occlusive disease. Am J Pathol. 1966 Apr;48(4):597-611.

72) Kanai T, Endo M, Minohara S, Miyahara N, Koyama-ito H, Tomura H, Matsufuji N, Futami Y, Fukumura A, Hiraoka T, Furusawa Y, Ando K, Suzuki M, Soga F, Kawachi K. Biophysical characteristics of HIMAC clinical irradiation system for heavy-ion radiation therapy. Int J Radiat Oncol Biol Phys. 1999 Apr 1;44(1):201-10.

73) Shibuya K, Ohno T, Terashima K, Toyama S, Yasuda S, Tsuji H, et al. Short-course carbon-ion radiotherapy for hepatocellular carcinoma: a multi-institutional retrospective study. Liver Int. 2018;38(12):2239-47.

74) Yasuda S, Kato H, Imada H, et al. Long-Term Results of High-Dose 2-Fraction Carbon Ion Radiation Therapy for Hepatocellular Carcinoma. Adv Radiat Oncol. 2019 Sep 27;5(2):196-203.

75) Shiba S, Shibuya K, Katoh H, et al. A comparison of carbon ion radiotherapy and transarterial chemoembolization treatment outcomes for single hepatocellular carcinoma: a propensity score matching study Radiat Oncol. 2019 Aug 2;14(1):137.

76) Shiba S, Shibuya K, Okamoto M, et al. Clinical impact of Hypofractionated carbon ion radiotherapy on locally advanced hepatocellular carcinoma. Radiat Oncol 2020 Aug 14;15(1):195.

77) Makishima S, Yasuda S, Isozaki Y, et al. Single fraction carbon ion radiotherapy for colorectal cancer liver metastasis: A dose escalation study. Cancer Sci. 2019 Jan;110(1):303-309.

78) Ebner DK, Tinganelli W, Helm A, Bisio A, Yamada S, Kamada T, Shimokawa T, Durante M. The immunoregulatory potential of particle radiation in cancer therapy. Front Immunol. 2017;8:1–8.

Clinical Study Protocol

A phase Ib study of durvalumab (MEDI4736) ± tremelimumab combined with Carbon ion radiotherapy in advanced hepatocellular carcinoma patients with macrovascular invasion

Study Number: CCRC2002
Investigational drugs : Durvalumab, Tremelimumab（Concominant therapy : Carbon ion radiotherapy）
Version Number 1.1
Date 26 Mar 2021

Revision history

Date	Version Number
24 Feb 2021	1.0
26 Mar 2021	1.1

This protocol includes information and data that contain privileged or confidential information and, is provided only to the investigators, clinical team staff, associates, IRBs, or the Data Monitoring Committee. Therefore, this information must not be made public without written permission from the Chief Investigator, and AstraZeneca except when explaining to subjects. These restrictions on disclosure will apply equally to all or part of the data obtained in the clinical trial for publishing or presentation.

＜Abbreviation and terms＞

Definitions of abbreviations, acronyms, and terms in this study protocol are as follows

Abbreviation /terms	Definition
AE	adverse event
AESI	adverse event of special interest
AFP	α-fetoprotein
AFP-L3	α-fetoprotein - L3
ALP	alkaline phosphatase
ALT	alanine aminotransferase
AMED	Japan Agency for Medical Research and Development
APTT	activated partial thromboplastin time
AST	aspartate aminotransferase
AUC	area under the blood concentration time curve
BICR	blinded independent central review
BP	blood pressure
BSA	body surface area
CD	cluster of differentiation
CI	confidence interval
CIRT	carbon-ion radiotherapy
Cmax	maximum observed concentration
Cmin	minimum concentration during a dosing interval
COI	conflicts of interest
CRP	C-reactive protein
CT	computed tomography
Ctrough	drug concentration at the end of the dosing interval
CTCAE	common terminology criteria for adverse events
CTLA-4	cytotoxic T lymphocyte antigen 4
CTV	clinical target volume
DAMP	damage-associated molecular pattern
DC	dendritic cell
DLT	dose limiting toxicity
DMC	data monitoring committee
DNA	deoxyribonucleic acid
ECG	electrocardiogram
ECOG	Eastern Cooperative Oncology Group
eCRF	electronic case report form
EDC	electronic data capture
EGFR	epidermal growth factor receptor
ER	electronic record
ES	electronic signature
FAS	full analysis set
FTIH	first-time-in-human
FU	follow-up
G-CSF	granulocyte-colony stimulating factor
GCP	Good Clinical Practice
GI	gastrointestinal
HAIC	hepatic arterial infusion chemotherapy

Abbreviation /terms	Definition
HBc	hepatitis B core
HBs	hepatitis B surface
HBV	hepatitis B virus
HCC	hepatocellular carcinoma
HCV	hepatitis C virus
HDV	hepatitis D virus
HIMAC	Heavy Ion Medical Accelerator in Chiba
HIV	human immunodeficiency virus
HMGB1	high mobility group box protein1
HR	hazard ratio
HR	heart rate
HRQoL	health-related quality of life
ICH	International Council for Harmonisation of Technical Requirements for Pharmaceuticals for Human Use
ICI	immune checkpoint inhibitor
IFN-γ	interferon-γ
Ig	immunoglobulin
IL	interleukin
ILD	interstitial lung disease
imAE	immune mediated adverse event
IR	investigational regimen
IRB	institutional review board
KL-6	Krebs von den Lungen-6
LDH	lactate dehydrogenase
LET	linear energy transfer
LFT	liver function test
mAb	monoclonal antibody
MedDRA	Medical Dictionary for Regulatory Activities
MOA	mechanism of action
mRECIST	modified RECIST
MRI	magnetic resonance imaging
MST	mean survival time
MTD	maximum tolerated dose
MVI	macroscopic vascular invasion
NIRS	National Institute of Radiological Sciences
NSCLC	non-small cell lung cancer
NTL	non-target lesion
NYHA	New York Heart Association
OAR	off-axis ratio
ORR	objective response rate
OS	overall survival
PD	progressive disease
PD-1	programmed cell death 1
PD-L1	programmed cell death ligand 1
PD-L2	programmed cell death ligand 2
PIVKA-II	protein induced by vitamin K absence-II

Abbreviation /terms	Definition
PK	Pharmacokinetics
PMDA	Pharmaceuticals and Medical Devices Agency
PPS	per protocol set
PR	partial response
PRO	patient reported outcome
PS	performance status
PT	preferred term
PT-INR	prothrombin time-international normalized ratio
PTV	planning target volume
QxD	quaque x day
QxW	quaque x week
QST	National Institutes for Quantum and Radiological Science and Technology
QTcF	corrected QT interval using Fridericia's formula
Q2W	quaque 2 weeks
Q3W	quaque 3 weeks
Q4W	quaque 4 weeks
RBE	relative biological effectiveness
RECIST	Response Evaluation Criteria In Solid Tumors
RESORCE	Regorafenib after Sorafenib in Patients with Hepatocellular Carcinoma
RFA	radiofrequency ablation
RILD	radiation induced liver damage
RNA	ribonucleic acid
SAE	serious adverse event
SD	stable disease
SHARP	Sorafenib Hepatocellular Carcinoma Assessment Randomized Protocol
SOP	standard operating procedure
SP-D	surfactant protein-D
SpO2	saturation of peripheral oxygen
TACE	transcatheter arterial chemoembolization
TAI	transcatheter arterial infusion
TBL	total bilirubin
TKI	tyrosine kinase inhibitor
TL	target lesion
TLR4	Toll-like receptor 4
TMGs	Toxicity Management Guidelines
TSH	thyroid stimulating hormone
TTP	time to progression
ULN	upper limit of normal
VEGF	vascular endothelial growth factor

TABLE OF CONTENTS

0. CLINICAL TRIAL OVERVIEW....9
1. INTRODUCTION....16
1.1. Introduction....16
1.2. Standard Treatment for Advanced Hepatocellular Carcinoma....16
1.3. HCC with vascular invasion and its treatment....17
1.4. Immunotherapy....17
1.5. Carbon ion radiotherapy....18
1.6. Induction of Immunogenicity by Radiotherapy....18
1.7. Combination of immune checkpoint inhibitors and carbon ion radiotherapy....19
1.8. Investigational drugs....19
1.8.1. Durvalumab....19
1.8.2. Tremelimumab....20
1.8.3. Durvalumab + tremelimumab combination therapy....20
1.8.4. Rationale for Durvalumab and Tremelimumab as Treatment Options for HCC....20
1.9. hypothesis....21
2. STUDTY OBJECTIVE....23
2.1. Objective....23
2.2. Study results regarding the appropriateness of conducting this clinical trial, efficacy, and safety for the subject disease....23
2.2.1. Durvalumab + tremelimumab combination therapy dose rationale....23
2.2.2. Dose rationale for combination regimen of durvalumab 1500 mg Q4W plus tremelimumab 300 mg × 1 dose....23
2.2.2.1.Rationale for utilizing a fixed-dose regimen for durvalumab and tremelimumab....24
2.2.3 Rationale for carbon-ion radiotherapy....24
2.2.4 Rationale for combined treatment of carbon-ion radiotherapy and immunotherapy....25
2.3. Benefit-risk and ethical assessment....25
2.3.1. Potential benefits....25
2.3.2. Overall risks....25
2.3.3. Overall benefit-risk....28
3. ELIGIBILITY....29
3.1. Diagnostic Criteria and Stage, Type, and Condition Classification....29
3.2. Inclusion criteria....29
3.3. Exclusion criteria....30
4. INFORMED CONSENT....33
4.1. Preparation and revision of informed consent form....33
4.2. Method of Obtaining Informed Consent....33
4.3. Information to be provided to subjects....34
5. STUDY DESIGN....35
5.1. Overview of study design....35
5.2. Target number of subjects and study duration....38
5.3. Monitoring for safety assessment....38
5.4. Institutional and case registration methods....39
5.4.1. Facility registration....39
5.4.2. Subject resistration....39
5.4.3. Handling of subjects who are found to be ineligible after enrollment....40
5.5. Dosing schedule and dosage/administration method....40
5.5.1. Criterion for reduction....40
5.6. Discontinuation of Investigational Drug....41
5.7. Discontinuation of individual cases from participation in clinical trials....41
5.7.1. In case of untraceable cases....42
5.7.2. Withdrawal of consent....42
5.7.3. Clinical investigator's decision....42

5.7.4. Subject weight loss ... 42
5.7.5. Other cases ... 42
5.8. Subject replacement ... 42
5.9. Concomitant Restricted Drugs and Concomitant Restricted Therapy ... 42
5.10. Follow-up treatment ... 44
5.11. After discontinuation of this clinical trial ... 44
6. CLINICAL TRIAL TREATMENT ... 45
6.1. Durvalumab and tremelimumab ... 45
6.1.1. Durvalumab ... 45
6.2. Control Drugs ... 46
6.3. Monitoring during administration ... 46
6.4. Management of investigational drugs ... 46
6.5. Disposal of unused investigational drugs ... 47
6.6. Packaging and labeling of investigational drugs ... 47
6.7. Carbon ion radiotherapy ... 47
6.8. subject inclusion ... 48
6.9. Definition of Dose-Limiting Toxicity (DLT) ... 49
6.10. Toxicity Management ... 51
6.11. Restrictions during the clinical trial ... 51
6.11.1. Restrictions during the clinical trial ... 51
6.12. Clinical Trial Procedures ... 52
7. OBSERVATION, EXAMINATION, AND ASSESSMENT, METHODS, AND TIMING OF IMPLEMENTATION ... 53
7.1. Implementation Schedule and Procedures ... 53
7.2. Observation, tests and assessment ... 55
7.2.1. Screening period ... 55
7.2.2. DLT assessment period ... 56
7.2.3. Durvalumab q4W dosing period ... 58
7.2.4. At the time of discontinuation of investigational drug administration ... 59
7.2.5. Follow up period ... 59
7.3. Biological sampling procedures ... 59
7.3.1. Guideline for blood sampling volume ... 59
7.3.2. Blood samples for archiving ... 60
7.3.3. Hepatic tumor biopsy sample ... 60
7.4. Asessment of efficiency ... 60
7.5. Assessment of safety ... 61
7.5.1. Clinical laboratory tests ... 61
7.5.2. Physical examinations ... 63
7.5.3. Electrocardiogram (ECG) ... 63
7.5.4. Vital signs ... 63
7.5.5. ECOG performance status ... 64
7.5.6. Other safety assesements ... 64
8. HANDLING OF ADVERSE EVENT ... 64
8.1. Definition ... 64
8.1.1. Adverse event ... 64
8.1.2. Severe adverse event ... 65
8.1.3. Adverse Events of Special Interest (AESI) ... 65
8.1.4. Confirmation of interstitial lung disease (ILD) ... 66
8.2. Assessment of severity ... 67
8.3. Record of adverse events and serious adverse events ... 67
8.4. Duration of recording and follow-up of adverse events and serious adverse events ... 68
8.5. Causal relationship with investigational therapy ... 68
8.6. Outcome definition ... 69
8.7. Treatment of investigational drug in the event of an adverse event ... 69

8.8. Treatment of heavy particle therapy equipment in the event of an adverse event 69
8.9. Relationship to Protocol Procedures 69
8.10. Adverse events based on signs and symptoms 70
8.11. Adverse events based on tests and examinations 70
8.12. Hy's Law 70
8.13. Disease progression 70
8.14. New cancer 70
8.15. Deaths 70
8.16. Reportable Adverse Events 71
8.17. Reporting of serious adverse events 71
8.17.1. Response to subjects 72
8.18. OTHER EVENTS REQUIRING REPORTING 72
8.18.1. Overdose 72
8.18.2. Hepatic function abnormality 73
8.18.3. Pregnancy 73
8.18.4. Exposure to pregnant woman 73
8.18.5. Exposure to partner 73
8.19. Medication error 74
8.20. Predicted Side Effects 74
9. ENDPOINT 77
9.1. Primary endpoint 77
9.2. Secondary endpoint 77
9.3. Exploratory endpoints 77
10. STATISTICAL METHODS AND SAMPLE SIZE DETERMINATION 77
10.1. Description of Analysis set 77
10.1.1. DLT analysis set 77
10.1.2. Safety analysis set: SAF 77
10.1.3. Full Analysis set: FAS 77
10.1.4 Efficacy Evaluable set: EES 77
10.1.5 Per protocol set ： PPS 78
10.2. Target number of cases and rationale for setting 78
10.3. Case Handling 78
10.5.1. Analysis of subject background 78
10.5.2. Analysis of primary endpoints 78
10.5.2.1. DLT Evaluation 78
10.5.2.2. Safety Evaluation 79
11. COMPLIANCE AND DEVIATION FROM THE PROTOCOL 80
12. CHANGES TO THE CLINICAL TRIAL PROTOCOL, CASE REPORT FORM, OR ANALYSIS PLAN 80
13. DISCONTINUATION, SUSPENSION, OR TERMINATION OF THE CLINICAL TRIAL 81
14. DATA MANAGEMENT 82
15. RETENTION OF SOURCE DOCUMENTS AND OTHER RECORDS 82
16. RETENTION OF SAMPLES AND USE OF SAMPLES PROVIDED BY OTHER INSTITUTIONS 83
17. SOURCE DOCUMENT VERIFICATION 83
18. QUALITY ASSURANCE 83
19. QUALITY CONTROL FOR THIS STUDY 84
20. ETHICAL CONDUCT AND GOOD CLINICAL PRACTICE(GCP) 84
21. INSTITUTIONAL REVIEW BOARD(IRB) 84
22. HEALTH DAMAGE COVERAGE AND INSURANCE 85
23. COST BURDEN FOR THIS TRIAL 85
24. TRIAL FUNDS AND CONFLICT OF INTEREST 85

25. PROVISION OF THE INVESTIGATIONAL PRODUCTS AND INTELLECTUAL PROPERTY RIGHTS....85
26.PUBLICATION....85
27. REGISTRATION FOR CLINICAL TRIAL....85
28. STUDY IMPLEMANTATION GRPUP....86
29. LIST OF INVESTIGATIONAL PRODUCT(S) FOR THIS STUDY....86
30. LIST OF REFERENCES....86

0. Clinical Trial Overview

Title	A phase Ib study of durvalumab (MEDI4736) ± tremelimumab combined with particle therapy in advanced hepatocellular carcinoma patients with macrovascular invasion
Objective	**Objectives:**<br><br>**Primary Objective:**<br>To assess the safety and tolerability of combination therapy of durvalumab ± tremelimumab with particle therapy in advanced hepatocellular carcinoma patients with macrovascular invasion<br><br>**Secondary Objectives**:<br>To assess the efficacy of combination therapy of durvalumab ± tremelimumab with particle therapy in advanced hepatocellular carcinoma patients with macrovascular invasion
Design	Ib Phase, interventional, open-label, single arm<br>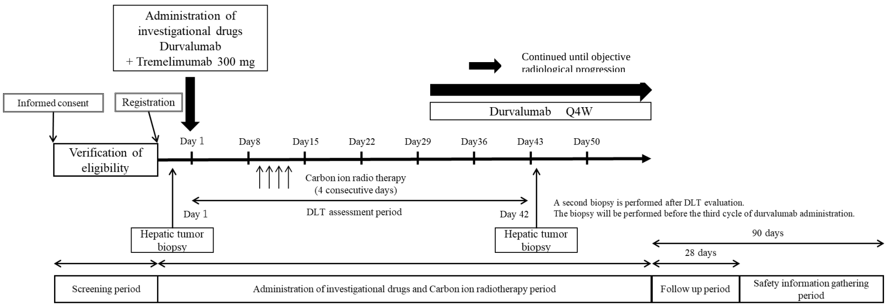
Phase	Ib Phase
Investigational Products and Combination Therapy	**Investigational Products and Combination Therapy:**<br><br>Investigational Products: Durvalumab and Tremelimumab<br>Durvalumab (MEDI4736) solution for infusion after dilution will be supplied in glass vials containing 500 mg durvalumab at a concentration of 50 mg/mL.<br><br>Tremelimumab solution for infusion after dilution will be supplied in glass vials containing 400 mg or 25 mg tremelimumab at a concentration of 20 mg/mL.<br><br>Combination Therapy<br><br>Carbon-Ion Radiation Therapy (CIRT) as particle therapy by Toshiba, Carbon ion radio therapy CI-1000S (PMDA approval no. 22800BZX00096000).
Inclusion Criteria	1) Capable of giving signed informed consent which includes compliance with the requirements and restrictions listed in the informed consent form (ICF) and in this protocol. Written informed consent and any locally required authorization obtained from the patient/legal representative prior to performing any protocol-related procedures, including screening

	evaluations. For patients aged <20 years and enrolling, a written informed consent should be obtained from the patient and his or her legally acceptable representative.<br>2) Age ≥20 years at time of study entry<br>3) Eastern Cooperative Oncology Group (ECOG) performance status of 0 or 1<br>4) Body weight >30 kg<br>5) Adequate normal organ and marrow function as defined below:<br>- Haemoglobin ≧9.0 g/dL<br>- Absolute neutrophil count (ANC) ≧1,500/mm^3<br>- Platelet count ≧75×10^9/L （≧75,000/mm^3）<br>- Serum bilirubin ≦ULN×3.0<br>- AST ≦ULN×5.0<br>- ALT ≦ULN×5.0<br>- Measured creatinine clearance (CL) >40 mL/min or Calculated creatinine clearance CL>40 mL/min by the Cockcroft-Gault formula (Cockcroft and Gault 1976) or by 24-hour urine collection for determination of creatinine clearance<br>6) Evidence of post-menopausal status or negative urinary or serum pregnancy test for female pre-menopausal patients. Women will be considered post-menopausal if they have been amenorrheic for 12 months without an alternative medical cause. The following age-specific requirements apply:<br>- Women <50 years of age would be considered post-menopausal if they have been amenorrheic for 12 months or more following cessation of exogenous hormonal treatments and if they have luteinizing hormone and follicle-stimulating hormone levels in the post-menopausal range for the institution or underwent surgical sterilization (bilateral oophorectomy or hysterectomy).<br>- Women ≥50 years of age would be considered post-menopausal if they have been amenorrheic for 12 months or more following cessation of all exogenous hormonal treatments, had radiation-induced menopause with last menses >1 year ago, had chemotherapy-induced menopause with last menses >1 year ago, or underwent surgical sterilization (bilateral oophorectomy, bilateral salpingectomy or hysterectomy).<br>7) Patient is willing and able to comply with the protocol for the duration of the study including undergoing treatment and scheduled visits and examinations including follow up.<br>8) Advanced HCC confirmed histologically or by the typical findings of a hypervascular tumor on computed tomography or angiography<br>9) (Cohort A and Cohort B only) Patients who have received at least one prior systemic chemotherapy regimen including atezolizumab bevacizumab combination, sorafenib, or lenvatinib and who are determined to be refractory or intolerant to standard therapy.<br>10) Must not be eligible for locoregional therapy for unresectable HCC. For patients who progressed after locoregional therapy for HCC, locoregional therapy must have been completed ≥28 days prior to the baseline scan for the current study. Acceptable locoregional therapy for HCC are Ethanol Infusion Therapy, Radio Wave ablation Therapy, Transcatheter Arterial chemoembolization (TACE), Transcatheter arterial infusion (TAI). Hepatic Arterial Infusion Chemotherapy (HAIC) is not allowed.<br>11) Patients who have been diagnosed with HCC showing MVI. MVI is defined

	as a tumor thrombus in the major hepatic and/or portal vein branches (Vp2, Vp3, Vp4, Vv2, and Vv3) identified by imaging studies.<br>12) Child-Pugh A<br>13) At least one measurable lesion other than the MVI and feeding nodule based on mRECIST.
Exclusion Criteria	1. Involvement in the planning and/or conduct of the study (applies to both sponser and/or staff at the study site)<br>2. Patients who have participated in another clinical trial using the investigational drug within 28 days prior to obtaining consent or who have received another investigational drug within 28 days prior to the first dose of the investigational drug in this study. The exception is if the patient is in the follow-up period of an interventional trial or is participating in an observational (non-interventional) clinical trial.<br>3. Any unresolved toxicity NCI CTCAE Grade ≥2 from previous anticancer therapy with the exception of alopecia, vitiligo, and the laboratory values defined in the inclusion criteria<br>– Patients with Grade ≥2 neuropathy will be evaluated on a case-by-case basis after consultation with the Study Physician.<br>– Patients with irreversible toxicity not reasonably expected to be exacerbated by treatment with durvalumab or tremelimumab may be included only after consultation with the Study Physician.<br>4. Radiotherapy treatment to more than 30% of the bone marrow or with a wide field of radiation within 4 weeks of the first dose of study drug<br>5. Major surgical procedure (as defined by the Investigator) within 28 days prior to the first dose of IP. Note: Local surgery of isolated lesions for palliative intent is acceptable.<br>6. History of allogenic organ transplantation.<br>7. Active or prior documented autoimmune or inflammatory disorders (including inflammatory bowel disease [e.g., colitis or Crohn's disease], diverticulitis [with the exception of diverticulosis], systemic lupus erythematosus, Sarcoidosis syndrome, or Wegener syndrome [granulomatosis with polyangiitis, Graves' disease, rheumatoid arthritis, hypophysitis, uveitis, etc.]). The following are exceptions to this criterion:<br>– Patients with vitiligo or alopecia<br>– Patients with hypothyroidism (e.g., following Hashimoto syndrome) stable on hormone replacement<br>– Any chronic skin condition that does not require systemic therapy<br>– Patients without active disease in the last 5 years may be included but only after consultation with the study physician<br>– Patients with celiac disease controlled by diet alone<br>8. Uncontrolled intercurrent illness, including but not limited to, ongoing or active infection, symptomatic congestive heart failure, uncontrolled hypertension, unstable angina pectoris, cardiac arrhythmia, interstitial lung disease, serious chronic gastrointestinal conditions associated with diarrhea, or psychiatric illness/social situations that would limit compliance with study requirement, substantially increase risk of incurring AEs or compromise the ability of the patient to give written informed consent<br>9. History of another primary malignancy except for<br>– Malignancy treated with curative intent and with no known active disease ≥5 years before the first dose of IP and of low potential risk for recurrence<br>– Adequately treated non-melanoma skin cancer or lentigo maligna without

	evidence of disease<br>– Adequately treated carcinoma in situ without evidence of disease<br>However, the following cases are eligible for enrollment<br>Early stage cancer (epithelial cancer of the cervix, basal cell carcinoma, superficial bladder cancer (Tis and T1), early stage gastric cancer, and early stage colorectal cancer) that has been treated for curative purposes, has not been confirmed active for at least 3 years prior to inclusion in the study, and has a low risk of recurrence.<br>10. History of leptomeningeal carcinomatosis<br>11. History of, or current, brain metastases or spinal cord compression. Patients with suspected brain metastases at screening should have an MRI (preferred) or CT, each preferably with IV contrast of the brain prior to study entry.<br>12. Mean QT interval corrected for heart rate using Fridericia's formula (QTcF) ≥470 ms calculated from 3 ECGs (within 15 minutes at 5 minutes apart) Regardless of whether this criteria stays or not, all patients should have a baseline ECG<br>13. History of active primary immunodeficiency<br>14. Patients co-infected with HBV and HCV, or co-infected with HBV and hepatitis D virus (HDV). HBV positive (presence of HBsAg and/or anti-HBcAb with detectable HBV DNA); HCV positive (presence of anti-HCV antibodies); HDV positive (presence of anti-HDV antibodies), and active infection including tuberculosis (clinical evaluation that includes clinical history, physical examination and radiographic findings, and TB testing in line with local practice).<br>15. Current or prior use of immunosuppressive medication within 14 days before the first dose of durvalumab or tremelimumab. The following are exceptions to this criterion:<br>– Intranasal, inhaled, topical steroids, or local steroid injections (e.g., intra articular injection)<br>– Systemic corticosteroids at physiologic doses not to exceed 10 mg/day of prednisone or its equivalent<br>– Steroids as premedication for hypersensitivity reactions (e.g., CT scan premedication)<br>16. Receipt of live attenuated vaccine within 30 days prior to the first dose of IP. Note: Patients, if enrolled, should not receive live vaccine whilst receiving IP and up to 30 days after the last dose of IP.<br>17. Female patients who are pregnant or breastfeeding or male or female patients of reproductive potential who are not willing to employ effective birth control from screening to 90 days after the last dose of durvalumab monotherapy or180 days after the last dose of durvalumab + tremelimumab combination therapy.<br>18. Known allergy or hypersensitivity to any of the study drugs or any of the study drug excipients.<br>19. Prior randomisation or treatment in a previous durvalumab and/or tremelimumab clinical study regardless of treatment arm assignment.<br>20. Judgment by the investigator that the patient is unsuitable to participate in the study and the patient is unlikely to comply with study procedures, restrictions and requirements.<br>21. Patients who have been treated with anti-PD-1, anti-PD-L1 inhibitors, or other drugs that act on other stimulatory or co-suppressive T-cell receptors and their combinations (including atezolizumab plus bevacizumab) and

	have failed to tolerate the same treatment.<br>22. Prior radiotherapy involving the liver.<br>23. Renal failure requiring hemodialysis or peritoneal dialysis<br>24. Any of the following cardiac diseases:<br>– NYHA Class III or IV chronic heart failure<br>– Current coronary artery disease or history of ischemic heart disease such as myocardial infarction within 6 months before the study<br>– Serious arrhythmia (grade 3 or higher according to the CTCAE ver. 5.0: arrhythmia that cannot be controlled by oral medications or requires mechanical control).<br>25. Poorly controlled hypertension<br>26. Serious and active infection, excluding hepatitis viral infection<br>27. Persistent proteinuria of NCI-CTCAE version 5.0 grade 3 or higher.<br>28. Arterial or venous thrombotic or embolic events such as cerebrovascular accident, deep vein thrombosis, or pulmonary embolism within 6 months before the start of study medication.<br>29. Refractory pleural effusion or ascites<br>30. History of hepatic encephalopathy within past 12 months<br>31. Oral intake impossible<br>32. HIV-positive<br>33. Pulmonary fibrosis or interstitial pneumonitis<br>34. Other serious complications as follows: serious mental disease or history of gastrointestinal bleeding or active hemoptysis<br>35. Unsatisfactory general condition for participation in the study as judged by the primary physician
Endoints	**Primary Endpoints:**<br>AEs/SAEs including DLTs<br><br>**Secondary Endpoints:**<br>Overall Survival (OS), Survival Rate at 6 months, Objective Response Rate (ORR), Time to Progression (TTP), Progression Free Survival (PFS) at 6 months (in accordance with mRECIST)_
Dosage and treatment	Each cycle of this study will last for 28 days, and the DLT evaluation period will be 42 days from the start of study drug administration.<br>Durvalumab 1,500 mg alone or in combination with 300 mg tremelimumab IV on Cycle 1, Day 1.<br>Carbon ion radio theray (60 Gy (RBE) / 4 times / 4 days) is administered between days 8-15 of the first cycle.<br>After the DLT evaluation period, durvalumab is continued every 4 weeks until tumor progression is confirmed.
Criteria for discontinuation of individual cases	1) In case it is judged difficult to continue the clinical trial for some reason on the subject's side, such as non-attendance or transfer to a different hospital.<br>2) In case the subject requests to discontinue participation in the clinical trial<br>3) In case the investigator or subinvestigator determines that the subject is unable to continue the clinical trial.<br>4) In case the subject's body weight becomes less than 30 kg.<br>5) In case the investigator/participating investigator determines that a decision to discontinue.

Number of Patients Planned:	**Number of Patients Planned:**<br>15 subjects.<br><br>The number of patients enrolled for DLT assessment in either Cohort A or Cohort B may vary from 3 to 6 depending on the frequency of DLT. If both of Cohort A and B regimens were confirmed tolerable after DLT assessment, additional patients will be enrolled in Cohort B up to a total of 15 subjects. If only Cohort A regimen was determined to be tolerable, additional patients may be enrolled in Cohort A up to a total of 15 subjects.<br><br>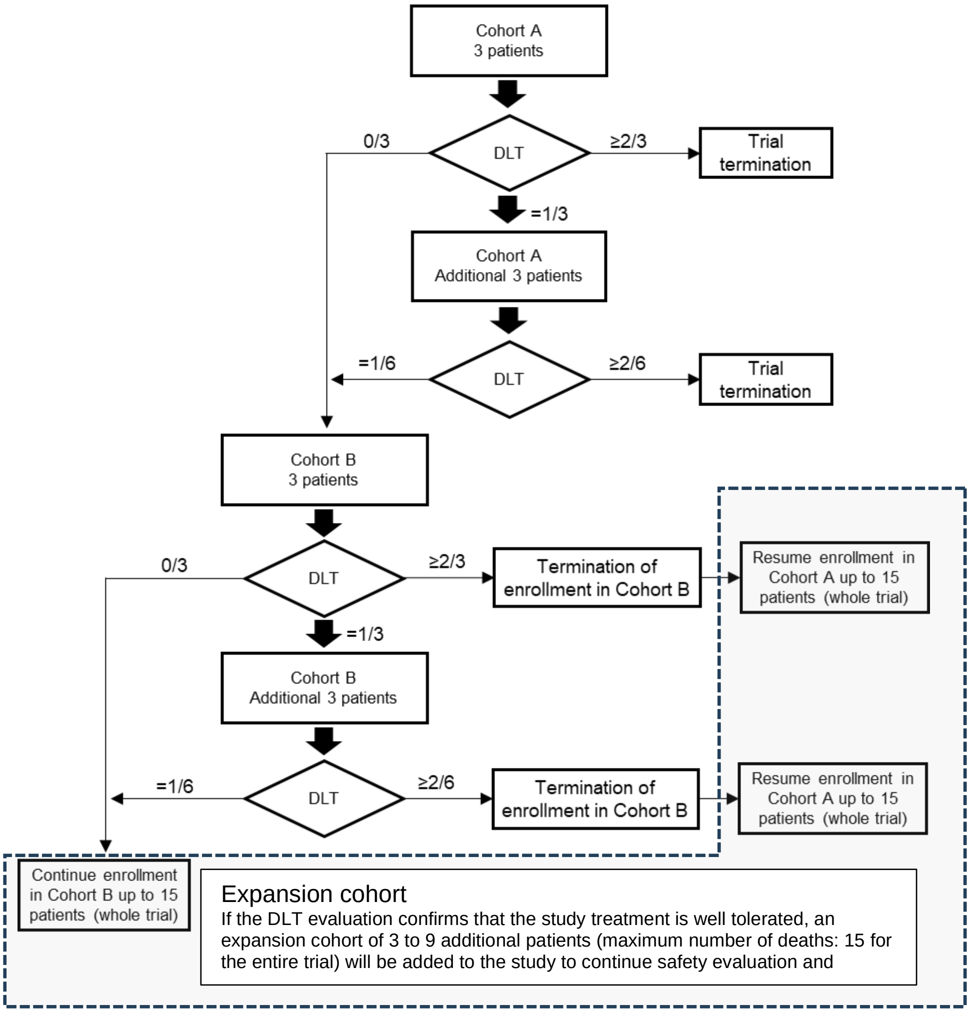
Clinical trial period	Estimated study start date 30 Apr 2021<br>Estimated study completion date 31 Dec 2022
Subject registration period	Estimated date of first patient enrolled 30 Apr 2021<br>Estimated date of last patient enrolled 30 Jun 2022
Clnical trial sites	2Sites<br>Enrollment of patients in the trial and administration of IPs will be conducted at Chiba University Hospital, and CIRT will be administered at QST Hospital.

ETHICAL AND REGULATORY REQUIREMENTS	The study will be performed in accordance with ethical principles that have their origin in the Declaration of Helsinki and are consistent with Good Clinical Practice, and applicable regulatory requirements Patient data protection.
Institutional Review Board (IRB)	Prior to the start of the study, the Institutional Review Board (IRB) will evaluate the ethical, scientific and medical relevance of this study. The study will be conducted after obtaining approval from the IRB. If the evaluation results of the IRB are “approved with modification”, the study will be conducted after the protocol, case report, or consent forms are modified based on the review results. At the frequency of more than once a year, the IRB will continuously review whether the study is being performed appropriately.

1. INTRODUCTION

1.1. Introduction

Primary liver cancer is broadly classified into hepatocellular carcinoma (HCC) and intrahepatic cholangiocarcinoma, of which HCC accounts for more than 90% in Japan. According to a recent survey report, the number of patients with hepatocellular carcinoma in Japan is approximately 47,000 [1], and the number of deaths is approximately 27,000 per year [2].

The development of HCC is strongly associated with chronic hepatitis and cirrhosis caused mainly by hepatitis B virus (HBV) or hepatitis C virus (HCV) infection, and carriers of these viruses are considered to be at high risk of developing HCC ([3] El Serag HB, et al. NEJM. 2011). Risk factors other than viral hepatitis include cirrhosis, male age, older age, alcohol consumption, smoking, obesity, fatty liver, and diabetes mellitus [4]. Many patients are diagnosed after the disease has reached an advanced stage. The choice of treatment depends on the stage of the disease and the patient's condition. Since most patients with HCC also have chronic hepatitis or cirrhosis, individual assessment of liver function is essential to patient selection. Although several agents have been shown to be effective in the treatment of unresectable advanced HCC, there are still limited options and further therapeutic development is needed.

1.2. Standard Treatment for Advanced Hepatocellular Carcinoma

Sorafenib demonstrated a survival advantage over placebo in unresectable advanced HCC in the Sorafenib Hepatocellular Carcinoma Assessment Randomized Protocol (SHARP) trial in 2008, followed by the Asia-Pacific trial in 2009 ([5] Llovet JM, et al. Asia-Pacific trial also demonstrated a survival benefit ([5] Llovet JM, et al. NEJM. 2008, [6] Cheng AL, et al Lancet Oncol. 2009). In the SHARP trial, median survival (OS) was 10.7 months in the sorafenib group versus 7.9 months in the placebo group, with a hazard ratio (HR) of 0.69 (95% confidence interval (CI): 0.55, 0.87), P < 0.001. months vs. 4.2 months, HR 0.68 (95% CI: 0.50, 0.93), P = 0.014. The median progression-free survival in the SHARP trial was 5.5 months in the sorafenib group versus 2.8 months in the placebo group, HR 0.58 (95% CI: 0.5, 0.7), and in the Asia-Pacific trial the median progression-free survival was 2.8 months versus 1.4 months, HR 0.6 (95% CI: 0.4, 0.8).

Regorafenib after Sorafenib in Child-Pugh classification A patients with image progression after sorafenib treatment and who tolerated sorafenib (400 mg or more for at least 20 days in the 28 days before the end of treatment) compared regorafenib to placebo Patients with Hepatocellular Carcinoma (RESORCE) trial, regorafenib showed for the first time a survival advantage as second-line therapy after sorafenib treatment ([7] Bruix J, et al. Lancet. 2017). In that study, the median OS was 10.6 months (95% CI: 9.1, 12.1) in the regorafenib group and 7.8 months (95% CI: 6.3, 8.8) in the placebo group, HR 0-63 (95% CI: 0.50, 0.79) P < 0-0001. 2017, lenvatinib was non-inferiority to sorafenib for the primary endpoint of survival, making it the first-line treatment of choice for advanced hepatocellular carcinoma ([8] Kudo M, et al. Lancet. 2018). In that study, the median OS was 13.6 months (95%CI: 12.1, 14.9) in the lenvatinib arm and 12.3 months (95%CI: 10.4, 13.9) in the sorafenib arm with a HR of 0.92 (95%CI : 0.79, 1.06). In addition, ramucirumab became available in June 2019 for unresectable HCC with serum AFP levels of 400 ng/mL or higher, exacerbated after cancer chemotherapy ([9]Zhu AX, et al. Lancet Oncol. 2019).

Combination therapy with atezolizumab, an anti-PD-L1 humanized monoclonal antibody, and bevacizumab, an anti-VEGF monoclonal antibody, was shown to significantly prolong OS versus sorafenib in patients with Child-Pugh classification A who had not received systemic therapy (IMbrave 150 study) ([7 Bomze D et al. NEJM. 2020). In that study, 12-month survival rates were reported as 67.2% (95% CI: 61.3, 73.1) for atezolizumab plus bevacizumab and 54.6% (95% CI: 45.2, 64.0) for sorafenib, with an HR for OS of 0.58 (95% CI: 0.42, 0.79), P < 0 .001. Based on these results, the combination of atezolizumab and bevacizumab became available in Japan in September 2020. Based on these results, the combination of atezolizumab and

bevacizumab is now the first-line treatment for advanced hepatocellular carcinoma. Other first-line treatment options include sorafenib or lenvatinib, and second-line treatment options include regorafenib, ramucirumab, and cabozantinib. However, the survival gains obtained have been limited and do not fully meet the medical needs of patients.

1.3. HCC with vascular invasion and its treatment

HCC with vascular invasion (MVI) extending into the portal and/or hepatic veins has a poorer prognosis compared to cases without MVI ([11]Costentin CE, et al. Liver Cancer 2017). Although systemic chemotherapy is the standard of care, several studies have suggested the efficacy of alternative treatment approaches (e.g., resection, hepatic arterial chemoembolization, intravenous chemotherapy, and radiation therapy) after determining the benefit for each patient, and are recommended in Japanese guidelines. MVI Although it is empirically known that controlling MVI dramatically improves the prognosis of advanced HCC with MVI, sufficient evidence does not exist.

1.4. Immunotherapy

In recent years, research on cancer immunity has advanced dramatically, and the mechanisms by which the immune system controls or eliminates tumors are becoming clearer ([12] Dunn, et al. Annu Rev Immunol. 2004). PD-L1 is part of a complex system of receptors and ligands involved in the regulation of T cell activation. The PD-1 receptor (CD279) is expressed on the surface of activated T cells ([13] Keir ME, et al. Rev Immunol. 2008). It has two known ligands: PD-L1 (B7 H1; CD274) and PD-L2 (B7 DC; CD273) ([14] Okazaki and Honjo 2007). PD-1 and PD-L1 / PD-L2 act as co-inhibitors that can arrest or limit T cell responses When PD-L1 binds to PD-1, inhibitory signals are transmitted to T cells, cytokine production is reduced, and T cell proliferation is inhibited. Tumor cells have been shown to utilize this immune checkpoint pathway as a mechanism to evade detection and inhibit immune responses.

PD-L1 has been found to be expressed in a wide range of carcinomas, and anti-PD-L1 antibodies can be used therapeutically to augment anti-tumor immune responses in cancer patients.Results from preclinical and clinical studies of monoclonal antibodies (mAbs) targeting the PD-L1 / PD-1 pathway provide evidence of clinical activity and manageable Anti-PD-L1 antibodies can be used to augment anti-tumor immune responses in cancer patients for therapeutic purposes, showing evidence of clinical activity and manageable safety profiles ([17] Brahmer JR, et al. N Engl J Med. 2012, [18] Hirano F, et al. Cancer Res. 2005, [19] Iwai Y, et al. Proc Natl Acad Sci USA. 2002,[20] Okudaira K, et al. Int J Oncol. 2009,[21] Topalian SL, et al. N Engl J Med. 2012,[22] Zhang C,et al. Cytotherapy. 2008), The hypothesis that its efficacy is higher in patients with tumors expressing PD-L1 ([23] Powles T, et al. Nature. 2014; [24] Rizvi N, et al. J Clin Oncol. 2015; [25] Segal NH, et al. J Clin Oncol. 2015) is now supported The hypothesis of a “bladder cancer” (e.g., [24] Rizvi N et al. The high frequency and number of mutations in bladder cancer ([26] Alexandrov et al. Nature. 2013), for example, may contribute to the responses seen with immunotherapy.

In contrast, cytotoxic T lymphocyte-associated antigen 4 (CTLA-4) is structurally expressed on regulatory T cells and is enhanced on the surface of activated T cells; CTLA-4 sends negative regulatory signals to T cells when it binds to CD80 (B7.1) or CD86 (B7.2) ligands on antigen-presenting cells ([27] (Fife BT, Bluestone JA. Immnol Rev. 2008). In animal models, blockade of CTLA-4 binding to CD80 / 86 by anti-CTLA-4 antibodies has also been shown to markedly enhance T cell activation and antitumor activity, as exemplified by the killing of established solid tumors in mice and induction of protective antitumor immunity. Therefore, treatment with anti-CTLA-4 antibodies is expected to enhance activation of the human immune system and anti-tumor activity in patients with solid tumors.

Preclinical data have been added along with abundant clinical data, indicating that inhibition of negative regulatory signals to T cells, such as cytotoxic T lymphocyte antigen 4 (CTLA-4) and PD-L1, is a promising approach with promising clinical results. Ipilimumab has received U.S. Food and Drug Administration (FDA) approval for the treatment of metastatic melanoma,

and nivolumab and pembrolizumab (two anti-PD-1 agents) and atezolizumab (an anti-PD- L1 agent) are currently approved by agencies such as the U.S. Food and Drug Administration and the European Medicines Agency for the treatment of metastatic melanoma, squamous cell and non-squamous cell non-small cell lung cancer, urothelial carcinoma, and other malignancies. In addition, data from agents in the anti-PD-1 / PD-L1 class have shown clinical activity against a wide range of carcinomas.

In HCC as in other cancer types, two clinical trials (nivolumab and pembrolizumab) using ICI showed that 17-20% of patients achieved an objective response and most of the responders had a durable response ([28]El-Khoueiry AB, et al . Lancet 2017, [29]Zhu AX, et al. Lancet Oncol. 2018). The combination of ICI with tyrosine kinase inhibitors or anti-VEGF monoclonal antibodies has shown promise with the potential to achieve extremely high sustained response rates in a variety of cancer types, including HCC ([30]Kudo M. World J Gastroenterol 2019). Recently, the IMbrave 150 trial showed that atezolizumab plus bevacizumab therapy extended both OS and PFS compared with sorafenib, positioning atezolizumab plus bevacizumab as the first-line treatment for advanced hepatocellular carcinoma. Immunotherapy is also playing an increasingly important role in advanced HCC, and a phase I study of durvalumab, an anti-PD-L1 antibody, and tremelimumab, an anti-cytotoxic T lymphocyte-associated antigen 4 (CTLA-4) monoclonal antibody, in patients with HCC demonstrated a response rate of 25% ([31 was demonstrated ([31] Kelly RK et al. ASCO2017). An expanded portion of a Phase II trial is underway. A Phase III trial evaluating the efficacy and safety of the combination of durvalumab and tremelimumab, as well as monotherapy with sorafenib and durvalumab (HIMALAYA trial) are also ongoing and awaiting results.

1.5. Carbon ion radiotherapy

Particle therapy, especially carbon ion radio therapy, has advantages in dose concentration and biological effects over photon therapy, such as x-rays and gamma rays, which are usually used in radiation therapy.

X-rays and gamma rays are most intense near the body's surface and penetrate the body with decreasing intensity as they travel deeper. For this reason, the most powerful way to treat a specific area with X-rays or gamma rays is to focus the radiation from many directions, concentrating the high-dose area on the lesion. However, for tumors that are close to vital organs and spread malformedly, it is difficult to avoid surrounding normal organs. In contrast, heavy particle beams, which become intense at a certain depth depending on their energy, but are weak before and after that point, can be easily focused by aligning the peak with the tumor ([32] Pedroni E, et al. Med Phys. 1995).

The principle of action of radiotherapy is the double-strand break of DNA by ionizing radiation. Carbon ion radio therapy is known to cause dense ionization. Therefore, DNA double-strand breaks can be caused more efficiently and more densely than with X-rays. This is the reason for the favorable biological effects of heavy particle beams. This, combined with the good dose distribution resulting from the physical characteristics of heavy-ion beams, enables them to efficiently exert their anti-tumor effects. In Japan, the National Institute of Radiological Sciences (now renamed the National Institute of Quantum Science and Technology) started heavy particle therapy in 1994 and has treated more than 10,000 patients with good results ([33] Mohamad O., et al. Cancer (Basel), 2018).

In HCC, high local control rates have also been shown with particle therapy ([34] Kasuya G, et al. Cancer. 2017, [35] Fukuda, K, et al. Cancer Sci. 2017, [36] Sorin Y, et al. Liver Cancer 2018, [37] (Igaki H, et al. Int J Clin Oncol. 2018). Furthermore, good outcomes have been shown for hepatocellular carcinoma with vascular invasion. ([38] Komatsu S, et al. J Gastroenterol. 2011, [39] Lee SU, et al. Strahlenther Onkol. 2014, [40] Sugahara S, et al. Strahlenther Onkol. 2009).

1.6. Induction of Immunogenicity by Radiotherapy

Radiation therapy is known to stimulate immunogenicity through multiple mechanisms. The major immunological effects of irradiation include increased antigen presentation through

elevated expression of major histocompatibility gene complex class I, induction of apoptosis through elevated membrane expression of Fas ligands, calreticulin expression and high mobility group box-1 (HMGB1) and other (HMGB1), and induction of phagocytosis and immunity through the release of damage-associated molecular patterns (DAMPs).

Clinically, the PACIFIC trial showed that the combination of durvalumab and chemoradiotherapy can provide high therapeutic efficacy with intolerable toxicity ([41]Antonia Sj, et al. NEJM. 2017). Despite the fact that this study did not include only PD-L1-high expressing patients, it showed significant improvements in both PFS and OS compared to existing therapy. On the other hand, in the combination of durvalumab and tremelimumab (MYSTIC trial), there was no improvement from existing therapy, even when only PD-L1 high-expressing patients were targeted ([42 Rizvi NA, et al. Annals of Oncol. 2018). The difference between these two trials was the presence or absence of radiotherapy, and a subset analysis of the PACIFIC trial showed better results in patients with a shorter time between completion of radiotherapy and durvalumab administration, suggesting that radiotherapy plays an important role in tumor immunity Radiotherapy plays an important role in tumor immunity.

1.7. Combination of immune checkpoint inhibitors and carbon ion radiotherapy

Heavy ion therapy is known to enhance both local immunostimulation and immunosuppression more strongly than conventional photon beam therapy in both animal models and human clinical specimens ([43] Helm A, et al. Int J Part Ther. 2018, [44] Takahashi Y, et al. Oncotarget. 2019, [45] Iijima M, et al. J Gynecol Oncol. 2020).

Irradiated tumor cells present HMGB1, a key factor among damage-related molecules, alongside numerous others ([46] Golden EB, et al. Front Oncol, 2012). HMGB1 functions as an immune-activating cytokine and is a key factor for toll-like receptor 4 (TLR4) and activates dendritic cells.

It has been suggested that HMGB1 is strongly induced by heavy particle therapy ([47] Onishi M, et al. J Radiat Res. 2018). A study in mice showed stronger immune activation when heavy ion therapy was combined with dendritic cell infusion. The combination of heavy particle therapy and immunotherapy has also been shown to enhance anti-tumor immunity and reduce metastases compared to x-ray therapy, immunotherapy alone, or a combination of both ([48] Matsunaga A, et al. Cancer. 2010, [49] Ando K, et al. J Radiat Res. 2017).

On the other hand, immunosuppressive molecules such as PD-L1 have also been found to be induced more strongly than with X-ray therapy ([45] Iijima et al. J Gynecol Oncol. 2020). Enhanced PD-L1 expression by irradiation has been reported via the AMT/AT/Chk1 pathway induced by DNA double-strand breaks ([50] Sato N, et al. Nat Commun. 2017). Heavy ion therapy is known to efficiently generate complex DNA double-strand breaks ([51] Oike T, et al. Sci Rep. 2016), supporting this phenomenon.

Clinical trials investigating the combination of immune checkpoint inhibitors with radiation in a number of carcinomas, including HCC, are ongoing. However, most of them are limited to combinations with conventional radiation therapy using X-rays, and only a few with particle therapy. Currently, there are no clinical trials testing the combination of heavy ion beams and immunotherapy, and this clinical trial is a pilot case. From basic research and clinical studies using X-rays, the combination of heavy ion therapy and immune checkpoint inhibitors is expected to have a greater synergistic effect.

1.8. Investigational drugs

1.8.1. Durvalumab

Durvalumab is a human immunoglobulin (Ig) subclass Grade 1κ (IgGrade 1κ) mAb that inhibits the interaction of PD-L1 with PD-1 on T cells and CD80 on immune cells but not with PD-L2. Durvalumab is being developed by AstraZeneca/MedImmune for the treatment of cancer.

The mechanism of action of durvalumab is said to be inhibition of the interaction of PD-L1 with PD-1 and CD80, which disrupts the suppression of the immune response and immune

response to tumor elimination in in vitro studies, durvalumab was found to antagonize PD-L1-mediated suppression on primary human T cells, restore T cell proliferation, and release interferon gamma (IFN-γ) ([52] Stewart R, et al. Cancer Immunol Res. 2015) In vivo studies showed that durvalumab inhibited tumor growth in a xenograft model through a T cell-dependent mechanism ([52] Stewart R, et al. Cancer Immunol Res. 2015). Based on these data, durvalumab is expected to stimulate anti-tumor immune responses in patients by binding to PD-L1 and shifting the balance toward anti-tumor immune responses. Durvalumab is designed to reduce antibody-dependent and complement-dependent cellular injury.

To date, more than 8,000 patients have received durvalumab as a single agent or in combination with other anticancer agents in studies sponsored by AstraZeneca throughout the clinical development program. Please refer to the most recent Durvalumab investigational brochure for nonclinical and clinical information, including safety, efficacy, and pharmacokinetics.

1.8.2. Tremelimumab

Tremelimumab is a human immunoglobulin (Ig) Grade 2 mAb that targets CTLA-4 (CTLA-4; cluster of differentiation [CD]152), a cell surface receptor that is primarily expressed on activated T cells and blocks their activation. Tremelimumab completely inhibits the interaction of human CTLA-4 with CD80 and CD86 and increases the release of cytokines (interleukin [IL]-2 and interferon [IFN]-γ) from human T cells, peripheral blood mononuclear cells and whole blood ([53] Tarhini and Kirkwood. Expert Opin Biol Ther. 2008). Tremelimumab is being developed by AstraZeneca for the treatment of cancer.
To date, it has been administered to more than 1,500 patients as a single agent or in combination with other anticancer agents. For a summary of nonclinical and clinical information, including safety, efficacy, and pharmacokinetics, please refer to the Tremelimumab investigational brochure.

1.8.3. Durvalumab + tremelimumab combination therapy

Since there is no overlap in the mechanisms of action of CTLA-4 and PD-1, targeting both PD-1 and CTLA-4 pathways may provide additive or synergistic effects ([16] Pardoll DM, et al. Nat Rev Cancer. 2012). Therefore, in addition to investigating both drugs as monotherapy for various cancer indications, AstraZeneca is also investigating the combination of durvalumab plus tremelimumab for the treatment of cancer.

Study D4190C00006 is a late phase I dose escalation study to establish the safety, pharmacokinetics/pharmacodynamics and preliminary antitumor activity of the combination of durvalumab plus tremelimumab in patients with advanced non-small cell lung cancer (NSCLC). The dosing schedule used in this study is durvalumab administered Q2W or Q4W until Month 12, followed by 7 doses of tremelimumab at Q4W until Week 24, then 2 additional doses every 12 weeks for up to 12 months. The study is ongoing and enrollment is ongoing. Other trials investigating combination therapy for NSCLC and other tumor indications have also been initiated.

To date, 3,000 patients have received the combination in various doses and dosing regimens throughout the clinical development program. For a complete summary of non-clinical and clinical trial information, including safety, pharmacokinetics, and efficacy, please refer to the most recent Durvalumab and Tremelimumab investigational brochure.

1.8.4. Rationale for Durvalumab and Tremelimumab as Treatment Options for HCC

The liver has multiple regulatory mechanisms to maintain an immunosuppressive environment. The normal liver is inherently prone to induce immune tolerance to prevent aberrant immunity to exposed pathogens ([54]Pardee AD and Butterfield LH.Oncoimmunology. 2012). Clinical and nonclinical data indicate that HCC increases the expression of immunosuppressive cell

populations such as regulatory T cells (Treg) and myeloid-derived suppressor cells, as well as suppressive signaling molecules including CTLA-4 and PD-1 ([54] ,[55] Gao O, et al. Clin Cancer Res. 2009, [56] Hato T, et al. Hepatology. 2014); HBV and HCV infection also increase Treg and PD-L1/PD-1 expression, suggesting that this pathway is involved in HBV and HCV-mediated hepatocellular carcinogenesis ([54],[57] Miroux C, et al. Expert Opin Biol Ther. 2010,[58] Golden-Mason L, et al. J Virol. 2007,[59]Peng G, et al. Mol Immunol. 2008).

It has also been shown that overexpression of PD-L1 results in higher malignancy, disease progression, and mortality in HCC ([54], [60] Klein Jp, et al. Stat Med. 2007). Therefore, suppression of PD-L51 and CTLA-4 function could reverse the immunosuppressive nature of HCC and promote host immunity against HCC and improve clinical outcome.

At this time, early promising clinical data suggest that anti-CTLA-4 and anti-PD-L1/PD-1 antibody agents are active against HCC and may help improve response rates and survival.

In 20 patients with HCV-associated HCC (43% Child-Pugh classification B) treated with 15 mg/kg of intravenous tremelimumab every 90 days ([61] Sangro B, et al. J Hepatol. 2013), tremelimumab was generally well tolerated and no patients received systemic steroids No patients received systemic steroids and there were no deaths related to the study drug. The majority of patients had transient increases in transaminases after the first dose, 45% of which were Grade 3 or higher, but not accompanied by a concurrent decline in liver function. 17 responses could be evaluated, with 3 (17.6%) patients achieving a definite partial response (PR).

In another phase I/II study, 32 patients with unresectable advanced HCC (Child-Pugh classification A/B7) received two dose levels of tremelimumab (3.5 and 10 mg/kg IV Q4W) with partial ablation (RFA/TACE) during a 6-week treatment period ([62] Duffy AG,. et al. J Hepatol. 2017). Safety assessment results showed no clear trend in the occurrence of adverse events throughout the entire dose-specific cohort of tremelimumab, and no dose-limiting toxicities (DLTs) were observed in the study. The major Grade 3 or 4 adverse events were increased AST (21%), increased ALT (9%), and hyperbilirubinemia (9%); no Grade 3 or 4 diarrhea, colitis, or pneumonitis occurred; of the 19 patients with evaluable response outside the area directly treated with TACE/RFA, 5 (26.3%) had confirmed PR. The safety and efficacy of durvalumab (anti-PD-L1 antibody) monotherapy were investigated in a phase I/II study (CDON-MEDI4736-1108) in 40 patients with HCC, with an objective response rate (ORR) of 10.3% and a median OS of 13.2 months. Detailed safety and efficacy data from the study are described in Section 1.2.2.1. Similar results were obtained with another anti-PD-1 antibody, with an ORR of 18.6% and a median OS of 13.2 months in patients with advanced HCC ([63] Melero I, et al. J Clin Oncol. 2017).

Although the data from these trials are exploratory, they suggest that monotherapy with both durvalumab and tremelimumab has an antitumor effect against HCC. The combination of durvalumab plus tremelimumab in patients with unresectable HCC is being investigated in an ongoing phase I/II study (D4190C00022). In that study, patients with unresectable HCC who had not been previously treated with immunotherapy received the combination of durvalumab plus tremelimumab four times in Q4W followed by durvalumab alone in Q4W. Interim data from 40 patients followed for more than 16 weeks in the study showed an ORR of 18%. The combination of durvalumab plus tremelimumab was generally well tolerated in the population of patients with unresectable HCC. phase II results presented at ASCO 2020 reported promising results for durvalumab 1,500 mg Q4W plus tremelimumab 300 mg once. ORR was 24% and median OS was 18.73 months (95% CI: 10.78-27.27). It was reported to have the most favorable risk-benefit profile compared to the other dose arms ([64]Kelly RK, et al. J Clin Oncol. 2020).

The combined clinical evidence suggests that both durvalumab and tremelimumab have clinical activity as monotherapy in patients with HCC, and that combination therapy with both agents may provide an even greater antitumor effect in this patient population. A phase III, randomized, global study is underway to evaluate the safety and efficacy of these two agents in patients with HCC.

1.9. hypothesis

The purpose of this study is to confirm the synergistic effect of durvalumab-tremelimumab in

combination with heavy ion therapy in patients with advanced HCC. The study was designed based on the following two hypotheses

- Activation of tumor immunity by heavy ion therapy will be enhanced by immune checkpoint inhibitors.
- The control of primary lesions with MVI in patients with advanced HCC will have clinically significant results.

2. STUDTY OBJECTIVE

2.1. Objective

A phase Ib, open-label, uncontrolled study to evaluate the safety and efficacy of durvalumab-tremelimumab in combination with heavy ion therapy in patients with advanced hepatocellular carcinoma with MVI to assess safety and tolerability as measured by frequency of adverse events including DLT. Efficacy will be evaluated based on overall survival, 6-month survival, objective response rate, 6-month progression-free survival, and progression-free interval.

2.2. Study results regarding the appropriateness of conducting this clinical trial, efficacy, and safety for the subject disease

2.2.1. Durvalumab + tremelimumab combination therapy dose rationale

The durvalumab + tremelimumab doses and regimen selected for this study are based on the goal of selecting an optimal combination dose of durvalumab and tremelimumab that would yield sustained target suppression (sPD-L1), demonstrate promising efficacy, and have an acceptable safety profile.

2.2.2. Dose rationale for combination regimen of durvalumab 1500 mg Q4W plus tremelimumab 300 mg × 1 dose

A summary of the existing PK and pharmacodynamic data has been utilized to guide theregimen selection for the combination of durvalumab 1500 mg plus single dose of tremelimumab 300 mg.

Pharmacokinetics/Pharmacodynamics data

The supporting data for this regimen are based on PK and pharmacodyamic data from regimens that used tremelimumab doses of greater than 1 mg/kg from Study D4190C00006. An approximate dose-proportional increases in PK exposure (maximum serum concentration and area under the serum drug concentration-time curve from time 0 to Day 28 post-dose) was observed with increasing doses of tremelimumab (1, 3, and 10 mg/kg). An exploratory pharmacodynamic analysis bioanalytically evaluated the effects of tremelimumab on proliferating T-cells from NSCLC patients who received tremelimumab (1, 3, or 10 mg/kg) and durvalumab (15 or 20 mg/kg) combination treatment. Monotonic increases in pharmacodynamic activity with the combination (increased activation/ proliferation markers on CD4 and CD8 T-cells in periphery) were observed with increasing doses of tremelimumab (1, 3, 10 mg/kg). The peak increase (%) from baseline of CD4+Ki67+ T-cells was observed 8 days post administration, and the peak level was significantly increased ($p \leq 0.05$) as increasing dose of tremelimumab in the range of 1 to 10 mg/kg. Study data also suggested that higher peak exposure (maximum serum concentration [Cmax]) of tremelimumab is related to a higher maximum pharmacodynamic effect in the NSCLC patient population. Overall, the PK/pharmacodynamic data suggest that tremelimumab of dose greater than 1 mg/kg with a higher peak exposure may be associated with a higher pharmacodynamic effect.

Additionally, based on simulation data, the Cmax (78 μg/mL) post single dose administration of tremelimumab 4 mg/kg is approximately 4-fold higher than the predicted Cmax (19 μg/mL) post the first dose of tremelimumab 1 mg/kg, and is 3-fold higher than the predicted Cmax (25 μg/mL) post the fourth dose of tremelimumab 1 mg/kg in a Q4W×4 doses setting.

Clinical data

The safety and preliminary efficacy of combination of durvalumab 1500 mg plus single dose of tremelimumab 300 mg in unresectable HCC population is being evaluated in the ongoing Phase I/II study (Study D4190C00022).

In summary, a single dose of tremelimumab 4 mg/kg, while maintaining a similar overall

exposure, has a 3- to 4-fold higher Cmax compared to the 4 doses of tremelimumab 1 mg/kg. Therefore, this single administration of the higher dose of tremelimumab may have the potential for better anti-tumor activity while potentially avoiding any cumulative toxicity associated with repeated dosing of the 1 mg/kg tremelimumab. Therefore, the regimen of durvalumab 1500 mg plus tremelimumab 300 mg×1 dose is being evaluated in the current study.

Rationale for fixed dosing

A fixed-dose regimen of 1500 mg (equivalent to 20 mg/kg) of durvalumab plus 300 mg (equivalent to 4 mg/kg) of tremelimumab will be used in this study.

2.2.2.1. Rationale for utilizing a fixed-dose regimen for durvalumab and tremelimumab

A population PK model was developed for durvalumab using monotherapy data from a Phase I study (Study CD-ON-MEDI4736-1108; N=292; doses=0.1 to 10 mg/kg Q2W or 15 mg/kg Q3W; solid tumors). Similarly, a population PK model was developed for tremelimumab using data from Phase I through Phase III (N=654; doses=0.01 to 15 mg/kg Q4W or Q90D; metastatic melanoma) ([66]Wang et al 2014).

Population PK analysis indicated only minor impact of body weight on the PK of durvalumab and also tremelimumab (coefficient of ≤0.5). The weight-based versus fixed-dose (based on median weight of approximately 75 kg) regimens of both durvalumab and tremelimumab were compared using predicted PK concentrations (5th, 50th, and 95th percentiles) using a population PK model. A total of 1000 patients were simulated using weight distribution of 40 kg to 120 kg. Simulation results demonstrate that weight-based versus fixed dosing regimens of both durvalumab and tremelimumab yield similar median steady state PK concentrations with slightly less overall between-subject variability.

Similar findings have been reported by others ([22], [67] Narwal R, et al. Clin Pharmacokinet. 2013、[68] Ng CM, et al. Pharm Res. 2006, [69] Wang DD, et al. J Clin Pharmacol 2009, [70] Wolchok JD, et.al. N Engl J Med. 2013). Wang and colleagues investigated 12 monoclonal antibodies and found that fixed and body size-based dosing perform similarly, with fixed dosing being better for 7 of 12 antibodies ([21], [70] Wolchok JD, et al. N Engl J Med. 2013). In addition, they investigated 18 therapeutic proteins and peptides and showed that fixed dosing performed better for 12 of 18 in terms of reducing the between-subject variability in PK/pharmacodynamics parameters ([70] Wolchok JD, et al. N Engl J Med. 2013).

A fixed-dose approach is preferred by the prescribing community due to ease of use and reduced dosing errors. Given expectation of similar PK exposure and variability, we considered it feasible to switch to fixed-dose regimens. Based on the average body weight of 75 kg, a fixed dose of 1500 mg durvalumab (equivalent to 20 mg/kg) and a fixed dose of 300 mg tremelimumab (equivalent to 4 mg/kg) are selected for the current study. Therefore, the selected regimen of the durvalumab (+tremelimumab) cohort combined with particle therapy are:

【Cohort A】
Durvalumab monotherapy of 1500 mg Q4W

【Cohort B】
Durvalumab 1500 mg plus tremelimumab 300 mg for 1 dose, followed by durvalumab monotherapy 1500 mg Q4W

2.2.3 Rationale for carbon-ion radiotherapy

Historically, the role of radiation therapy in the treatment of liver tumors has been limited because of radiation-induced hepatic insufficiency caused by whole-liver irradiation ([71] Reed GB, et al. Am J Pathol, 1966). Particle therapy, including proton and carbon, overcame this problem with its physical dose distribution, enabling to treat large tumors while sparing normal liver tissue ([32], [72] Kanai et al. Int J Radiat Oncol Biol Phys, 1999). Results of CIRT have

been reported in multiple single institutional prospective studies and multi-institutional retrospective studies with high efficacy and mild toxicities ([34], [73] Shibuya K, et al. Liver Int. 2018, [74] Yasuda S, et al. Adv Radiat Oncol. 2019). Cases with MVI are also well treated with CIRT [35] Komatsu S, et al. J Gastroenterol. 2011). A single-arm clinical trial of CIRT in patients without large vessel or bile duct invasion is currently underway as an advanced medical treatment B, with the aim of developing a curative treatment for first-episode HCC that is not suitable for surgery (liver transplantation is not suitable) or radiofrequency ablation (jRCT1032200036).

Dose escalation studies have been conducted for 4 fraction CIRT ([34], [75]Shiba S, et al. Radiat Oncol. 2019). 60Gy (RBE) in 4 fractions appear to be well tolerated and multi-institutional retrospective study confirms its efficacy ([73] Shibuya K, et al. Liver Int. 2018). Constraints for each risk organ were determined based on previously published reports ([34], [73], [76] Shiba S, et al. Radiat Oncol 2020, [77] Makishima et al. Cancer Sci. 2018).

2.2.4 Rationale for combined treatment of carbon-ion radiotherapy and immunotherapy

In the PACIFIC trial, the combination of durvalumab and chemoradiotherapy (X-rays) has been shown to provide high therapeutic efficacy with intolerable toxicity, as discussed in 1.7 above ([41]Antonia SJ et al. NEJM. 2017). There are only ongoing trials of the combination of immune checkpoint inhibitors, including durvalumab, with particle therapy, and no reports exist as of November 2020. Trials of combinations with heavy particle therapy are similarly unregistered. As noted in 1.7 above, the results of basic research and clinical trials of combination therapy with X-rays suggest a higher synergistic effect.

2.3. Benefit-risk and ethical assessment

2.3.1. Potential benefits

2.3.1.1. Durvalumab monotherapy

Information on the potential benefit of durvalumab 1500 mg monotherapy or equivalent in patients with HCC are based on Study CD-ON-MEDI4736-1108 and are presented in Section 1.8.1. For other tumor types, see the most current durvalumab IB.

2.3.1.2. Durvalumab plus tremelimumab combination therapy

The potential benefits of adding tremelimumab to durvalumab is presented in Section 1.8.3. Information on the data supporting the selected combination regimen of durvalumab plus tremelimumab in patients with HCC are presented in Section 1.8.4. For other tumor types, see the most current durvalumab and tremelimumab IBs.

2.3.1.3. Durvalumab (+tremelimumab) combined with particle therapy

As mentioned in section 1.7, CIRT, as with traditional photon irradiation, is known to modify cancer immune reactions, but at a stronger level (Helm A, et al. Int J Part Ther. 2018, Ebner et al. Front Oncol. 2017). By combining CIRT with innunotherapy [durvalumab (+ tremelimumab)], there may be a larger improvement in efficacy compared to monotherapy of immunotherapy drugs.

2.3.2. Overall risks

Monoclonal antibodies directed against immune checkpoint proteins, such as programmed cell death ligand 1 (PD-L1) as well as those directed against programmed cell death-1 (PD-1) or cytotoxic T-lymphocyte antigen-4 (CTLA-4), aim to boost endogenous immune responses directed against tumor cells. By stimulating the immune system however, there is the potential for adverse effects on other tissues.

Most adverse drug reactions seen with the immune checkpoint inhibitor class of agents are thought to be due to the effects of inflammatory cells on specific tissues. These risks are generally

events with a potential inflammatory or immune mediated mechanism and which may require more frequent monitoring and/or unique interventions such as immunosuppressants and/or endocrine therapy. These immune mediated effects, can occur in nearly any organ system, and are most commonly seen as gastrointestinal AEs such as colitis and diarrhea, pneumonitis/interstitial lung disease (ILD), hepatic AEs such as hepatitis and liver enzyme elevations, skin events such as rash and dermatitis and endocrinopathies including hypo- and hyper-thyroidism.

2.3.2.1. Durvalumab

Risks with durvalumab include, but are not limited to, diarrhea/colitis, pneumonitis/ILD, endocrinopathies (hypo- and hyper-thyroidism, type I diabetes mellitus, hypophysitis and adrenal insufficiency) hepatitis/increases in transaminases, nephritis/increases in creatinine, pancreatitis/increases in amylase and lipase, rash/pruritus/dermatitis, myocarditis, myositis/polymyositis, other rare or less frequent inflammatory events including neurotoxicities, infusion-related reactions, hypersensitivity reactions and infections/serious infections.

For information on all identified and potential risks with durvalumab please always refer to the current version of the durvalumab IB.

In monotherapy clinical studies AEs (all grades) reported very commonly (≥ 15% of patients) are fatigue, nausea, decreased appetite, dyspnea, cough, constipation, diarrhea, vomiting, back pain, pyrexia, asthenia, anemia, arthralgia, peripheral edema, headache, rash, and pruritus. Approximately 9.4% of patients experienced an AE that resulted in permanent discontinuation of durvalumab and approximately 6.5% of patients experienced an SAE that was related to durvalumab by the study investigator.

Most treatment-related AEs were manageable with dose delays, symptomatic treatment, and in the case of events suspected to have an immune basis, the use of established treatment guidelines for immune-mediated toxicity.

A detailed summary of durvalumab monotherapy AE data can be found in the current version of the durvalumab IB.

2.3.2.2. Tremelimumab

Risks with tremelimumab monotherapy include, but are not limited to, GI effects (colitis, diarrhoea, enterocolitis and intestinal perforation), endocrine disorders (hypo and hyperthyroidism, hypophysitis and adrenal insufficiency), skin effects (rash, and pruritus), elevations in lipase and amylase and clinical manifestations of pancreatitis, other gastrointestinal events e.g. ulcerative colitis, dehydration, nausea and vomiting; hepatic events including hepatitis, and liver enzyme elevations; pneumonitis and ILD; nervous system events including encephalitis, peripheral motor and sensory neuropathies, Guillain-Barre and proximal muscle weakness; cytopenias including thrombocytopenia, anemia and neutropenia; infusion-related reactions, anaphylaxis, and allergic reactions; renal events including renal failure, acute kidney injury, nephritis, nephrotic syndrome, autoimmune nephritis and electrolyte abnormalities such as hypokalemia; autoimmune diseases including autoimmune arthritis, Sjogren's syndrome and giant cell temporal arteritis; hyperglycemia and diabetes mellitus; and pyrexia.

For information on all identified and potential risks with tremelimumab please always refer to the current version of the tremelimumab IB.

Using pooled data from monotherapy clinical studies AEs (all grades) reported very commonly (≥ 10% of patients) were diarrhea, nausea, fatigue, pruritus, decreased appetite, rash, vomiting, dyspnoea, constipation, cough, pyrexia, abdominal pain, decreased weight, headache, asthenia, and anaemia. Approximately 16% of patients experienced an AE that resulted in permanent discontinuation of tremelimumab and approximately 45% of patients experienced an SAE.

A detailed summary of tremelimumab monotherapy AE data can be found in the current version of the tremelimumab IB.

2.3.2.3. Durvalumab + tremelimumab

The safety of durvalumab + tremelimumab combination therapy was initially evaluated in the ongoing dose escalation and dose expansion Study 006, in patients with NSCLC, and is being studied in a number of other ongoing clinical trials, in a number of different indications, and has to date shown a manageable safety and tolerability profile.

The types of risks with the combination of durvalumab + tremelimumab (based on an equivalent durvalumab dose of 20m/kg and a tremelimumab dose of 1mg/kg) are similar to those for durvalumab and tremelimumab monotherapy. Emerging data from study 006, other studies evaluating the combination, and from combinations of other agents in the same class indicate an increased frequency and/or severity of some of these immune-mediated toxicities.

For information on all identified and potential risks with the durvalumab+tremelimumab combination please always refer to the current version of the durvalumab IB

In durvalumab+tremelimumab combination studies at the dose of durvalumab 20mg/kg and tremelimumab 1mg/kg AEs (all grades) reported very commonly (≥ 10% of patients) are fatigue, diarrhoea,nausea, dyspnea, decreased appetite, pruritus, vomiting, anaemia, constipation, cough, abdominal pain, pyrexia, back pain, arthralgia, hypothyroidism, asthenia, oedema peripheral, decreased weight, decreased hyponatraemia and rash.

Approximately 15% of patients experienced an AE that resulted in permanent discontinuation of study drug and approximately 16% of patients experienced an SAE that was considered to be related to durvalumab and tremelimumab by the study investigator.

A detailed summary of durvalumab + tremelimumab combination AE data can be found in the current version of the durvalumab IB.

2.3.2.4. Carbon-ion radiotherapy

Safety of CIRT for HCCs as a monotherapy are confirmed through multiple single institutional prospective studies and multi-institutional retrospective studies ([34], [73], [74]). Grade 3 or severe acute toxicities were seen in skin (1%) and ALT elevation (0.6%), with both being G3. G3 or severe late toxicities were seen in skin (G3 2%, G4 0.6%), hepatic coma (G3 1%), myositis and rib fracture (both G3 0.6%). RILD was seen in 2%. No treatment related deaths were observed.

Since CIRT is a form of radiotherapy, potential risks will be confined within the radiation field. The following may be observed.

- Bone marrow suppression
- Radiation pneumonitis, pleuritis, pleural effusion
- Nausea, vomiting, anorexia, diarrhoea, GI bleeding, ulceration, perforation, stricture
- Loss of hepatic function, hepatic failure, RILD, bile duct stricture, occlusion, cholangitis, aneurism
- Radiation dermatitis, ulceration
- Pericarditis, pericardial effusion, congestive heart failure, myocarditis, arrythmia
- Myelopathy, Peripheral neuropathy
- Rib fracture, subcutaneous induration, subsequent primary cancer, renal failure

2.3.2.5. durvalumab (+tremelimumab) combined with carbon-ion radiotherapy

Currently, there are no clinical trial results open yet for durvalumab + tremelimumab + radiotherapy for liver tumors, thus risks are unknown. Combination of immuno-oncology drugs and radiotherapy appear to have minimal excessive toxicity compared to immune-oncology drugs alone across multiple trials, including thoracic irradiation ([41] Antonia SJ, et al. NEJM 2018).

Potential risks would be toxicities closely related to immune reaction and microangiopathy, such as RILD, radiation pneumonitis and GI tract ulceration. In CIRT as a monotherapy, these toxicities are less common compared to photon radiotherapy, owing to the better dose distribution of CIRT as explained above ([34], [73], [74]). While the impact of difference in immune-modulation against toxicity between photon and carbon-ion is unknown, there is no definite evidence that excessive toxicity will be intolerable.

2.3.3. Overall benefit-risk

Durvalumab and tremelimumab have shown encouraging anti-tumor activity as single agents in advanced HCC population. The summary of this efficacy data is presented in Section 1.8.4. The combination regimen of these two agents shows a higher response rate in HCC population compared to either of the monotherapies. Thus, durvalumab plus tremelimumab combination therapy may potentially offer benefit to this patient population. Both durvalumab monotherapy and durvalumab plus tremelimumab combination therapy was tolerable in advanced HCC. The current study design aims to minimize potential risks by providing for early and intensive safety monitoring for any unexpected safety signals and for managing those risks deemed to be most likely based on prior experience with durvalumab, tremelimumab, and carbon-ion therapy. Two combination dose regimens of durvaluamb plus tremelimumab combination therapy were selected for this study with the aim to select the regimen with the most benefit for patients with advanced HCC.

HCC patients with MVI have very limited systemic therapeutic options and a poor life expectancy and health-related quality of life (HRQoL) based on the currently available treatments. While the main prognosticator in these patients is the intravascular growth of MVI, thus focal treatment by particle therapy has shown prolonged MST up to 2 years if lesions are confined in number ([40] Sugahara S, et al. Strahlether Onkol. 2009)). But those with extensive disease (ie multiple hepatic lesions and extrahepatic lesions) are currently not treated with particle therapy in fear of the out-of-treatment-field lesions. HCC patients with MVI, therefore, represents a significant unmet medical need and underlines the need for novel therapies for this patient population. CIRT combined with durvalumab or durvalumab plus tremelimumab proposed in this study may demonstrate a meaningful clinical benefit and a manageable safety profile. The overall benefit-risk profile of durvalumab (+tremelimumab) combined with CIRT is expected to be favorable, therefore supporting the current study design.

3. ELIGIBILITY

Each patient must meet all of the inclusion criteria (Section 3.2) and none of the exclusion criteria (Section 3.3) for this study. Under no circumstances will there be exceptions to this rule.

3.1. Diagnostic Criteria and Stage, Type, and Condition Classification

【CohortA & CohortB】

Eligible patients will be advanced HCC with MVI and Child-Pugh classification A over 20 years of age who are refractory or intolerant to standard systemic chemotherapy.

【Expansion cohort】

Eligible patients are advanced HCC with MVI, aged 20 years or older, with Child-Pugh classification A, with or without prior drug therapy.

3.2. Inclusion criteria

For inclusion in the study, patients should fulfill the following criteria:

1） Capable of giving signed informed consent which includes compliance with the requirements and restrictions listed in the informed consent form (ICF) and in this protocol. Written informed consent and any locally required authorization obtained from the patient/legal representative prior to performing any protocol-related procedures, including screening evaluations. For patients aged <20 years and enrolling, a written informed consent should be obtained from the patient and his or her legally acceptable representative.

2） Age >20 years at time of study entry

3） Eastern Cooperative Oncology Group (ECOG) performance status of 0 or 1

4） Body weight >30 kg

5） Adequate normal organ and marrow function as defined below:
   - Haemoglobin ≧9.0 g/dL
   - Absolute neutrophil count (ANC) ≧1,500/mm3
   - Platelet count ≧75×109/L （≧75,000/mm3）
   - Serum bilirubin ≦ULN×3.0
   - AST ≦ULN×5.0
   - ALT ≦ULN×5.0
   - Measured creatinine clearance (CL) >40 mL/min or Calculated creatinine clearance CL>40 mL/min by the Cockcroft-Gault formula (Cockcroft and Gault 1976) or by 24-hour urine collection for determination of creatinine clearance

6） Evidence of post-menopausal status or negative urinary or serum pregnancy test for female pre-menopausal patients. Women will be considered post-menopausal if they have been amenorrheic for 12 months without an alternative medical cause. The following age-specific requirements apply:
   - Women <50 years of age would be considered post-menopausal if they have been amenorrheic for 12 months or more following cessation of exogenous hormonal treatments and if they have luteinizing hormone and follicle-stimulating hormone levels in the post-menopausal range for the institution or underwent surgical sterilization (bilateral oophorectomy or hysterectomy).
   - Women ≥50 years of age would be considered post-menopausal if they have been amenorrheic for 12 months or more following cessation of all exogenous hormonal treatments, had radiation-induced menopause with last menses >1 year ago, had chemotherapy-induced menopause with last menses >1 year ago, or underwent surgical sterilization (bilateral oophorectomy, bilateral salpingectomy or hysterectomy).

7） Patient is willing and able to comply with the protocol for the duration of the study including undergoing treatment and scheduled visits and examinations including follow up.

8） Advanced HCC confirmed histologically or by the typical findings of a hypervascular tumor

on computed tomography or angiography

9) (Cohort A and Cohort B only) Patients who have received at least one prior systemic chemotherapy regimen including atezolizumab bevacizumab combination, sorafenib, or lenvatinib and who are determined to be refractory or intolerant to standard therapy.

10) Must not be eligible for locoregional therapy for unresectable HCC. For patients who progressed after locoregional therapy for HCC, locoregional therapy must have been completed ≥28 days prior to the baseline scan for the current study. Acceptable locoregional therapy for HCC are Ethanol Infusion Therapy, Radio Wave ablation Therapy, Transcatheter Arterial chemoembolization (TACE), Transcatheter arterial infusion (TAI). Hepatic Arterial Infusion Chemotherapy (HAIC) is not allowed.

11) Patients who have been diagnosed with HCC showing MVI. MVI is defined as a tumor thrombus in the major hepatic and/or portal vein branches (Vp2, Vp3, Vp4, Vv2, and Vv3) identified by imaging studies.

12) Child-Pugh A

13) At least one measurable lesion other than the MVI and feeding nodule based on mRECIST.

3.3. Exclusion criteria

Patients should not enter the study if any of the following exclusion criteria are fulfilled:

1) Involvement in the planning and/or conduct of the study (applies to both sponser and/or staff at the study site)

2) Patients who have participated in another clinical trial using the investigational drug within 28 days prior to obtaining consent or who have received another investigational drug within 28 days prior to the first dose of the investigational drug in this study. The exception is if the patient is in the follow-up period of an interventional trial or is participating in an observational (non-interventional) clinical trial.

3) Any unresolved toxicity NCI CTCAE Grade ≥2 from previous anticancer therapy with the exception of alopecia, vitiligo, and the laboratory values defined in the inclusion criteria
   - Patients with Grade ≥2 neuropathy will be evaluated on a case-by-case basis after consultation with the Study Physician.
   - Patients with irreversible toxicity not reasonably expected to be exacerbated by treatment with durvalumab or tremelimumab may be included only after consultation with the Study Physician.

4) Radiotherapy treatment to more than 30% of the bone marrow or with a wide field of radiation within 4 weeks of the first dose of study drug

5) Major surgical procedure (as defined by the Investigator) within 28 days prior to the first dose of IP. Note: Local surgery of isolated lesions for palliative intent is acceptable.

6) History of allogenic organ transplantation.

7) Active or prior documented autoimmune or inflammatory disorders (including inflammatory bowel disease [e.g., colitis or Crohn's disease], diverticulitis [with the exception of diverticulosis], systemic lupus erythematosus, Sarcoidosis syndrome, or Wegener syndrome [granulomatosis with polyangiitis, Graves' disease, rheumatoid arthritis, hypophysitis, uveitis, etc.]). The following are exceptions to this criterion:
   - Patients with vitiligo or alopecia
   - Patients with hypothyroidism (e.g., following Hashimoto syndrome) stable on hormone replacement
   - Any chronic skin condition that does not require systemic therapy
   - Patients without active disease in the last 5 years may be included but only after consultation with the study physician
   - Patients with celiac disease controlled by diet alone

8) Uncontrolled intercurrent illness, including but not limited to, ongoing or active infection, symptomatic congestive heart failure, uncontrolled hypertension, unstable angina pectoris,

cardiac arrhythmia, interstitial lung disease, serious chronic gastrointestinal conditions associated with diarrhea, or psychiatric illness/social situations that would limit compliance with study requirement, substantially increase risk of incurring AEs or compromise the ability of the patient to give written informed consent

9) History of another primary malignancy except for
   - Malignancy treated with curative intent and with no known active disease ≥5 years before the first dose of IP and of low potential risk for recurrence
   - Adequately treated non-melanoma skin cancer or lentigo maligna without evidence of disease
   - Adequately treated carcinoma in situ without evidence of disease
   - However, the following cases are eligible for enrollment
   - Early stage cancer (epithelial cancer of the cervix, basal cell carcinoma, superficial bladder cancer (Tis and T1), early stage gastric cancer, and early stage colorectal cancer) that has been treated for curative purposes, has not been confirmed active for at least 3 years prior to inclusion in the study, and has a low risk of recurrence.

10) History of leptomeningeal carcinomatosis

11) History of, or current, brain metastases or spinal cord compression. Patients with suspected brain metastases at screening should have an MRI (preferred) or CT, each preferably with IV contrast of the brain prior to study entry.

12) Mean QT interval corrected for heart rate using Fridericia's formula (QTcF) ≥470 ms calculated from 3 ECGs (within 15 minutes at 5 minutes apart) Regardless of whether this criteria stays or not, all patients should have a baseline ECG

13) History of active primary immunodeficiency

14) Patients co-infected with HBV and HCV, or co-infected with HBV and hepatitis D virus (HDV). HBV positive (presence of HBsAg and/or anti-HBcAb with detectable HBV DNA); HCV positive (presence of anti-HCV antibodies); HDV positive (presence of anti-HDV antibodies), and active infection including tuberculosis (clinical evaluation that includes clinical history, physical examination and radiographic findings, and TB testing in line with local practice).

15) Current or prior use of immunosuppressive medication within 14 days before the first dose of durvalumab or tremelimumab. The following are exceptions to this criterion:
   - Intranasal, inhaled, topical steroids, or local steroid injections (e.g., intra articular injection)
   - Systemic corticosteroids at physiologic doses not to exceed 10 mg/day of prednisone or its equivalent
   - Steroids as premedication for hypersensitivity reactions (e.g., CT scan premedication)

16) Receipt of live attenuated vaccine within 30 days prior to the first dose of IP. Note: Patients, if enrolled, should not receive live vaccine whilst receiving IP and up to 30 days after the last dose of IP.

17) Female patients who are pregnant or breastfeeding or male or female patients of reproductive potential who are not willing to employ effective birth control from screening to 90 days after the last dose of durvalumab monotherapy or180 days after the last dose of durvalumab + tremelimumab combination therapy.

18) Known allergy or hypersensitivity to any of the study drugs or any of the study drug excipients.

19) Prior randomisation or treatment in a previous durvalumab and/or tremelimumab clinical study regardless of treatment arm assignment.

20) Judgment by the investigator that the patient is unsuitable to participate in the study and the patient is unlikely to comply with study procedures, restrictions and requirements.

21) Patients who have been treated with anti-PD-1, anti-PD-L1 inhibitors, or other drugs that act on other stimulatory or co-suppressive T-cell receptors and their combinations (including atezolizumab plus bevacizumab) and have failed to tolerate the same treatment.

22) Prior radiotherapy involving the liver.

23） Renal failure requiring hemodialysis or peritoneal dialysis

24） Any of the following cardiac diseases:

- NYHA Class III or IV chronic heart failure
- Current coronary artery disease or history of ischemic heart disease such as myocardial infarction within 6 months before the study
- Serious arrhythmia (grade 3 or higher according to the CTCAE ver. 5.0: arrhythmia that cannot be controlled by oral medications or requires mechanical control).

25） Poorly controlled hypertension

26） Serious and active infection, excluding hepatitis viral infection

27） Persistent proteinuria of NCI-CTCAE version 5.0 grade 3 or higher.

28） Arterial or venous thrombotic or embolic events such as cerebrovascular accident, deep vein thrombosis, or pulmonary embolism within 6 months before the start of study medication.

29） Refractory pleural effusion or ascites

30） History of hepatic encephalopathy within past 12 months

31） Oral intake impossible

32） HIV-positive

33） Pulmonary fibrosis or interstitial pneumonitis

34） Other serious complications as follows: serious mental disease or history of gastrointestinal bleeding or active hemoptysis

35） Unsatisfactory general condition for participation in the study as judged by the primary physician

4. Informed consent

4.1. Preparation and revision of informed consent form

The investigator prepares the consent form and other information documents used to obtain consent for participation in the clinical trial from the subject in plain language as much as possible. If it is considered necessary to revise the consent document and other explanatory documents, the investigator revises these documents.

The investigator submits the prepared or revised consent documents and other explanatory documents to the IRB for approval.

Amendments to the study protocol and informed consent forms will follow the below procedures:

1. When amendments are considered to be necessary, the Principal Investigator will provide to the Investigator(s) the study protocol amendment draft, informed consent form amendment drafts, and the latest version of the investigator's brochure and other necessary material/information.
2. The Principal Investigator will provide the Investigator(s) with necessary time to adequately consider the aforementioned study protocol amendment draft and material/information and discuss the details with the Principal Investigator.
3. After discussion with the Principal Investigator, the Investigator(s) will promptly submit the amended version of the study protocol or informed consent form to the head of the trial site, and receive approval of the IRB via the head of the trial site.
4. Within acceptable limits of the Principal Investigator, the same procedures will apply to amendments to be made to the study protocol and informed consent form according to instructions given by the head of the trial site based the opinions of the IRB.

4.2. Method of Obtaining Informed Consent

1) Informed consent

The investigator or subinvestigator should hand the consent document and other explanatory documents to the subject and provide sufficient explanation of the contents as indicated in "4.3". If necessary, the clinical trial coordinator also provides supplementary explanations to the subject. After confirming that the patient has a good understanding of the contents of the clinical trial, the subject's signed and dated informed consent should be obtained before the pre-study (screening) test is conducted.

2) When explaining to subjects

The investigator or sub-investigator shall give the subject the opportunity to ask questions and sufficient time to decide whether or not to participate in the trial before obtaining informed consent and shall answer the subject's questions to the subject's satisfaction.

3) Signing and delivery of consent form

The investigator or subinvestigator who provided the explanation should sign the consent form with the date of the explanation. The subject signs the consent form with the date of consent. If a collaborator provides supplementary explanation, the collaborator should also sign and enter the date of the explanation. After obtaining informed consent, a copy of the information document and the consent form shall be given to the subject.

4) Amendments to informed consent forms

When the investigator or subinvestigator revises the informed consent form or other explanatory documents due to the acquisition of new information that may be relevant to the subject's consent, the investigator or subinvestigator shall explain to the subject again using the revised informed consent form and other explanatory documents, and obtain consent in writing for the subject's continued participation in the clinical trial. If new important information is obtained that may affect the subject's consent, the information shall be immediately provided to the subject, recorded in writing, and the subject's continued participation in the clinical trial shall be confirmed.

4.3. Information to be provided to subjects

The informed consent form to be prepared by the investigator shall include the following information.

1. What is a clinical trial?
2. The purpose of the clinical trial
3. Name, title and contact information of the investigator
4. Method of the clinical trial
5. Anticipated clinical benefits and risks or inconveniences
6. Availability of other treatment options for the subject and the expected important benefits and risks associated with such treatment options
7. The expected duration of the subject's participation in the clinical trial
8. That participation in the clinical trial is of the subject's own free will and that the subject may refuse or withdraw from participation in the clinical trial at any time. Furthermore, the subject shall not be treated unfavorably due to refusal or withdrawal, and shall not lose any benefits that he/she would have received if he/she had not participated in the clinical trial.
9. Monitors, auditors, clinical trial review committees, and regulatory authorities must be able to view source documents related to medical care. In such cases, the confidentiality of the subject shall be maintained. In addition, the subject's signature on the consent document shall be considered as authorization for access.
10. Subjects' confidentiality shall be maintained even if the results of the clinical trial are made public.
11. Compensation and treatment to which subjects are entitled in the event of adverse health effects related to the clinical trial.
12. Information that may influence the subject's decision to continue participation in the clinical trial will be promptly communicated to the subject.
13. Conditions or reasons for discontinuation of participation in the clinical trial
14. Expenses to be borne by the subject in relation to the clinical trial
15. Details of any financial or other payments to be made to the subject in connection with the clinical trial (e.g., arrangements for calculating the amount to be paid)
16. The medical institution's contact person to whom subjects should refer or contact if they require further information regarding the clinical trial and their rights or if they experience any health problems related to the clinical trial.
17. Items to be observed by the subject
18. Types of investigational review committees that will investigate and deliberate on the appropriateness of the clinical trial, matters to be investigated and deliberated by each investigational review committee, and other matters related to the investigational review committee for the clinical trial in question
19. Intellectual property
20. Conflicts of interest

5. STUDY DESIGN

5.1. Overview of study design

This is a Phase Ib study to assess the safety of durvalumab combined with particle therapy (Cohort A) and durvalumab plus tremelimumab combined with particle therapy (Cohort B) in advanced hepatocellular carcinoma patients with macrovascular invasion. This study consists of four periods: the screening period, DLT assessment period, durvalumab q4W dosing period, and follow up period. After the signed informed consent is obtained and the screening is conducted, the patient will be registered for enrollment in the trial. Patients will be administered with the first IP followed by administration of CIRT.

DLT assessment period is for 42days starting from the first dose of durvalumab on Day1 of Cycle1. The first administration of continuous durvalumab q4W on Day 1 of Cycle 2 starts only after the safety of Cycle 1 was confirmed (durvalumab q4W: 28 day cycle).

DLT analysis will be made when more than one DLT was observed in each cohort.

Patients will to continue to receive durvalumab every 4 week after completion of the DLT assessment period until clinical progression/withdrawal from the trial if there may be potential clinical benefit at the investigator’s discretion.

Follow up visit will be made 28 days after study termination due to PD or withdrawal from the study. Safety information will be collected until 90 days after the last dose of study treatment or until initiation of alternative anticancer therapy. In this study, three patients are initially enrolled into cohort A. If there is no DLT observed in any of these subjects, the trial proceeds to enroll additional subjects into the cohort B, whose regimen does not contain higher dose of durvalumab but contains an additional drug of tremelimumab instead. If one subject develops a DLT at cohort A or cohort B, an additional three subjects are enrolled into that same cohort. Development of DLTs in more than 1 of 6 subjects in either cohort suggests that the regimen is not tolerable. If cohort A turns out to be intolerable, then cohort B regimen will not be pursued The evaluation of DLTs shall be performed by the investigator of Chiba University Hospital in consultation with the investigator(s). The DLTs determined shall be discussed with the Independent Data Monitoring Committee and their opinion shall be sought in accordance with the standards separately stipulated (10.7. Independent Data Monitoring Committee).

Duration of DLT assessment is defined for 42 days starting from the first administration of IP on Day1 of Cycle 1. Dose of durvalumab is fixed on 1500 mg. CIRT will be performed between Day 8 to Day 14 of Cycle 1 after the first duvalumab administration on Day1 (CIRT within 14 days after 1stcycle of durvalumab).

In both cohorts, if the investigators determined that there may be potential clinical benefit, patients will to continue to receive durvalumab every 4 week until clinical progression (Durvalumab q4W dosing period).

- **Cohort A**: Durvalumab 1500mg will be administered every 4 weeks in principle. Particle therapy, in form of CIRT, will be performed after Day8 of Cycle1 following the 1st dose of Durvalumab on Day1. 2nd cycle of durvalumab will be administrated only after the safety during DLT assessment was confirmed.

- **Cohort B**: Durvalumab 1500mg will be administered every 4 weeks in principle, and Tremelimumab 300mg will be administered only on Day1 of Cycle1. Particle therapy, in form of CIRT, will be performed after Day8 of Cycle1 following 1st cycle of Durvalumab + Tremelimumab. 2nd cycle of durvalumab will be administrated only after the safety during DLT assessment was confirmed.

CIRT will be given to both arms. Dose prescription and fractionations will be 60Gy (RBE) / 4Fr /

1week. Target lesion of the particle therapy will be focused on intrahepatic nodule with MVI. A 1cm margin will be taken as a clinical target volume margin for the feeding nodule, and 2cm margin alongside the vessel for the MVI lesion. Internal motion will be compensated according to 4D-CT movement assessment. Inter-fractional margin will be set to 3mm and combined with internal motion compensation forming a field specific planning treatment volume. Dose constraints for OARs will be prioritized over target volume coverage.

If both of Cohort A and B regimens were confirmed tolerable after DLT assessment, additional patients will be enrolled in Cohort B up to a total of 15 subjects. If only Cohort A regimen was determined to be tolerable, additional patients may be enrolled in Cohort A up to a total of 15 subjects.

Schedule for administration of investigational drugs and carbon ion radio therapy（Figure1）

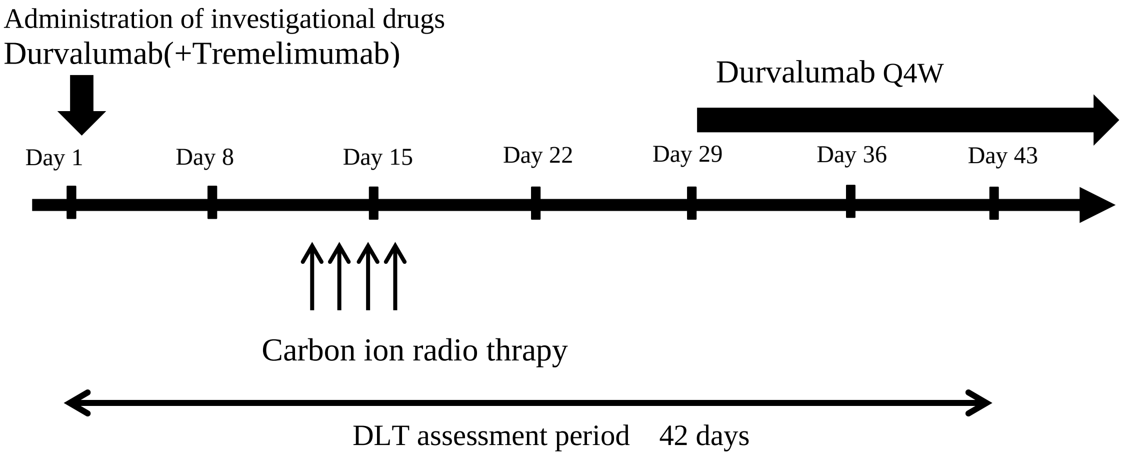

Tumor assessments, based on RECIST v.1.1, mRECIST and irRECIST, will be performed every 6 weeks (Q6W) (±1 week) for the first 12 weeks from the date of randomization and then Q8W (±1 week) thereafter until RECIST 1.1-defined radiological progression followed by a subsequent scan if clinically feasible, evaluated by Confirmation of Radiological Progression criteria (Appendix B). Patients who permanently discontinue study drug(s) for reasons other than PD should continue to have radiographic scans performed per their original schedule until confirmed PD

Subjects with rapid tumor progression or tumor-associated syndromes requiring urgent medical intervention (e.g., central nervous system metastases, respiratory failure due to tumor compression, spinal cord compression) will be deemed ineligible for continued durvalumab.

The overview of this clinical trial is as follows.

Cohort A

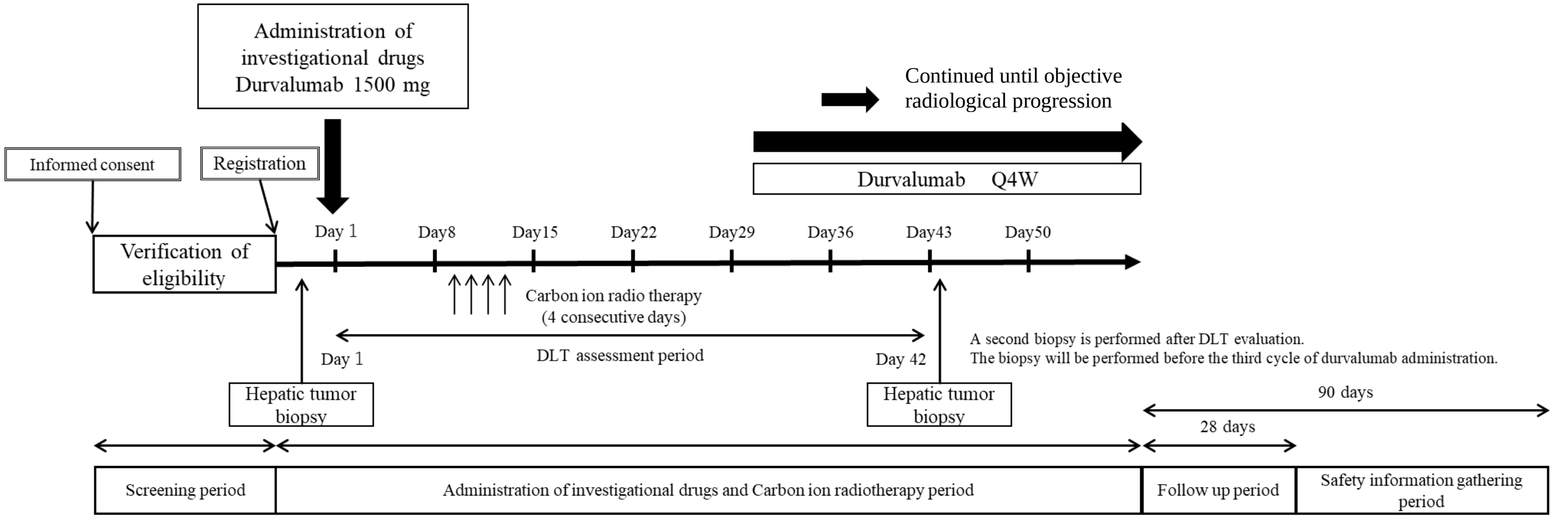

Cohort B

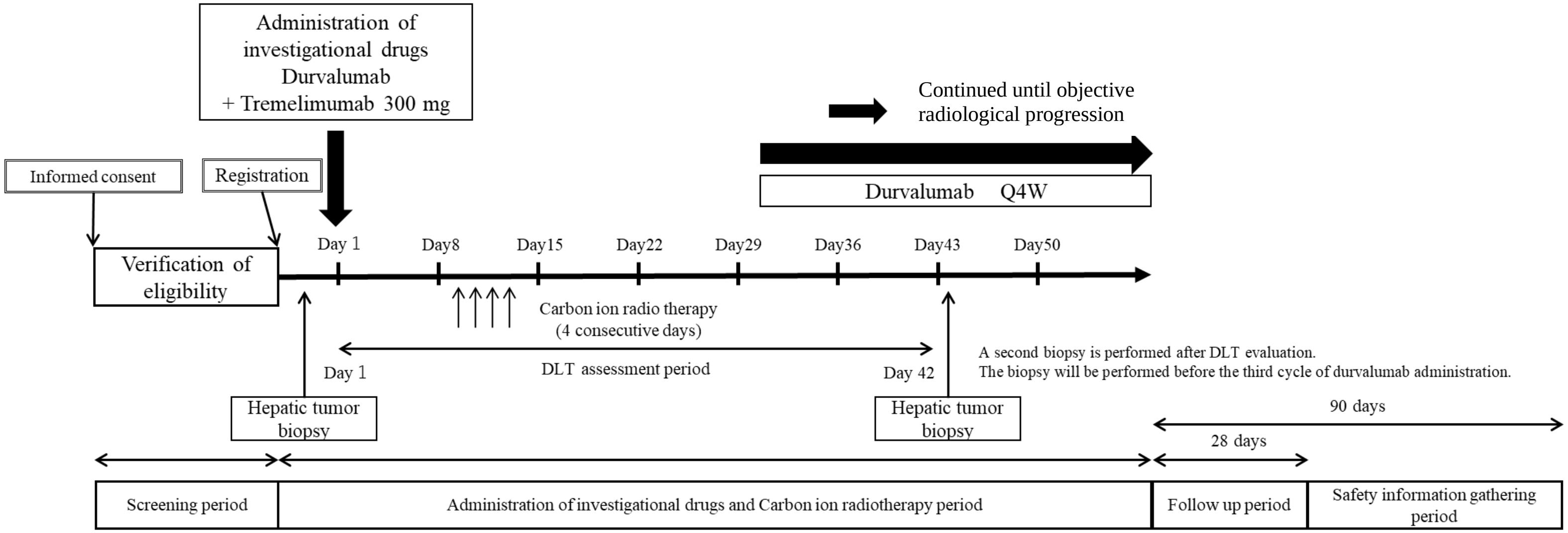

5.2. Target number of subjects and study duration

Target number of subjects：15

Study Period：
Estimated study start date 30 Apr 2021
Estimated study completion date 31 Dec 2022
Subject registration period:
Estimated date of first patient enrolled 30 Apr 2021
Estimated date of last patient enrolled 30 Jun 2022

Study schema

This study consists of four periods: the screening period, DLT assessment period, durvalumab q4W dosing period, and follow up period.

Signed informed consent will be obtained and patients will be screened prior to enrollment. The investigational treatment will include carbon ion radiotherapy on or after day 8 of cycle 1 after the first dose of study drug; the DLT evaluation period is 42 days from the first dose of durvalumab on day 1 of cycle 1. After confirmation of the safety of Cycle 1, durvalumab will be administered sequentially in Q4W beginning on Day 1 of Cycle 2. If multiple DLTs are observed in each cohort, an independent data monitoring committee will be convened to provide input on the determination of intolerability.

If no DLT is observed after the DLT evaluation period, durvalumab will be continued every 4 weeks until objective confirmation of disease progression (7.4) or until the criteria for 5.6 are met. Post-study follow-up for individual cases will occur 28 days after study completion. Adverse events and serious adverse event outcomes in subjects will be collected 90 days after the last dose of study treatment or until initiation of alternative anticancer therapy.

Figure2.

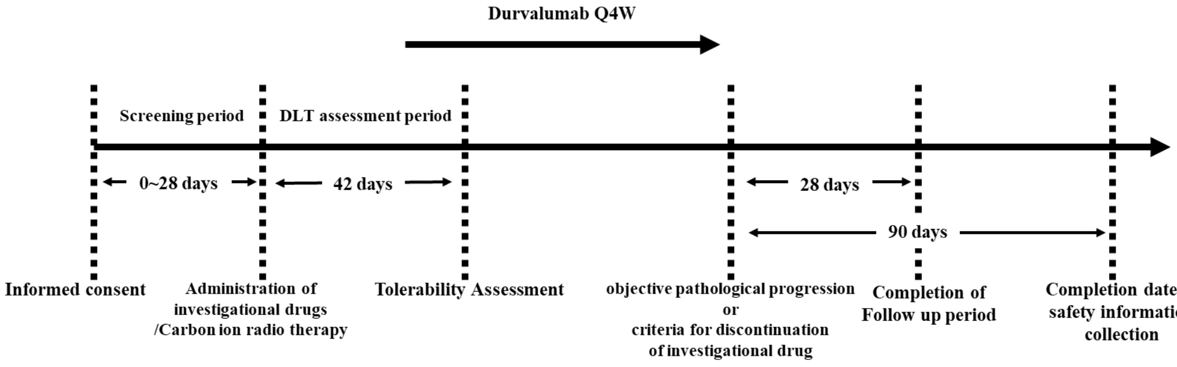

5.3. Monitoring for safety assessment

In situations where the below information is obtained and study patients are placed at under risk, or the continuation of the clinical trial is determined to be difficult, the Principal Investigator may decide on the termination or suspension of the entire clinical trial upon discussion with the Investigators. In addition, the study may be stopped based on the opinions by the DMC.

1. Occurrence of an unpredictable serious adverse reaction
2. Any information that indicates that the number, frequency, and condition of predictable serious adverse reactions cannot be predicted from the investigator’s brochure
3. SAEs that have been determined to have no causal relations with the IP, but are later determined that there is a reasonable possibility, due to the number, frequency, and condition of occurrences

4. Research reports indicating the tendency of the number, frequency, and condition of occurrences of adverse reactions having changed drastically
5. Research reports indicating the possibility that cancer, other serious diseases, disabilities, or death may occur
6. Information indicating that efficacy of the study drug cannot be expected in this clinical trial
7. Information indicating that the IP does not have any efficacy or effect on the target disease of the clinical trial
8. Information on any of the following related to marketed drugs that include the same ingredients as the IP:
    - Termination of manufacturing, import, or retail
    - Collection or disposal
    - Any other measures taken against health and hygiene related risks

Regardless of the reason for discontinuation, all data available to the subject at the time of discontinuation must be documented in the eCRF. All reasons for discontinuation should be documented. In terminating a trial, the investigator ensures that the protection of the subject's interests is given due consideration.

5.4. Institutional and case registration methods

Site registration and case registration will be conducted under the central registration system at the Data Management Office, Department of Clinical Trials, Chiba University Hospital. Once the site registration is completed, case registration will be available from the site. The following procedures will be used for site registration by fax and case registration via the Web.

5.4.1. Facility registration

1) The investigator at each participating site shall send a copy of the approval letter from the investigational review committee and a request form for site registration to the site registration center by fax after approval is obtained from the investigational review committee at the site.
2) The site registration center registers the sites and sends a notification of completion of site registration to the investigator.

Registration Center (Department of Data Management, Department of Clinical Trials, Chiba University Hospital)<br>fax number: 043-222-1207<br>Tel：043-222-1206<br>※Open hours are Monday through Friday, 9:00 a.m. to 5:00 p.m. (except Saturdays, Sundays, national holidays, and year-end and New Year holidays)<br>If a fax is received outside of the receptionist's office hours, it will be accepted on the next working day. (except at the beginning of the New Year)

5.4.2. Subject resistration

1) The investigator or subinvestigator obtains written consent and registers the subject in the case registration system. The case registration will be done via the website. After registration, a screening test is conducted to confirm that subjects meet the selection criteria and do not violate the exclusion criteria.
2) The investigator, subinvestigator or collaborator accesses the designated URL and enters the information necessary for case registration on the website. The investigator or sub investigator confirms the eligibility determination on the screen, and if the subject is

determined to be eligible, protocol treatment is initiated. Once a subject is enrolled, enrollment will not be cancelled.

＊ The investigator or sub-investigator shall not administer the investigational drug until the subject is enrolled and "eligible" by screening test.

Registration Web site（DATATRAK Enterprise Cloud）<br>URL：https://secure.datatrak.net<br>TEL：043-222-1206<br>※Open 365 days a year, 24 hours a day, including Saturdays, Sundays, and holidays (except for system downtime due to maintenance checks, etc.)

5.4.3. Handling of subjects who are found to be ineligible after enrollment

Subjects who do not meet "eligibility" for any reason, such as ineligibility, on the post-enrollment screening test are not eligible for enrollment and administration of the investigational product. The investigator or sub-investigator will explain to the subject that he/she is not eligible for enrollment in the study. The investigator or sub-investigator will also record the reason for the subject's ineligibility in the source documents.

If a subject is enrolled as a subject, but it is later determined that the subject does not meet all eligibility criteria, the subject will not be assigned and administered the investigational product and will be terminated from the clinical trial. If a subject who does not meet all eligibility criteria is inadvertently enrolled or started on the investigational product, the investigator will discuss whether or not to continue the investigational product. The investigator will ensure that all decisions resulting from this discussion are properly documented. If consensus cannot be reached, administration of the investigational product to the subject will be discontinued.

5.5. Dosing schedule and dosage/administration method

- Durvalumab 1,500 mg single agent arm (Cohort A)

  Cycle 1 Durvalumab 1,500 mg intravenous infusion will start on Day 1; after the first dose of durvalumab, heavy ion therapy will be administered on or after Day 8 of Cycle 1. Cycle 2 durvalumab will be administered after the safety of Cycle 1 is confirmed. Thereafter, dosing will be continued every 4 weeks until objective disease progression is confirmed (7.4.), "5.6. Discontinuation of Investigational Drug" is met, and the study is terminated.

- Durvalumab 1,500 mg + tremelimumab 300 mg once combination therapy (Cohort B)

  Cycle 1 One dose of combination therapy with durvalumab 1,500 mg and tremelimumab (both intravenous infusions) will be administered starting on Day 1. Durvalumab infusion will be started approximately 1 hour (maximum 2 hours) after completion of tremelimumab administration; after the first dose of durvalumab plus tremelimumab, heavy ion therapy will be administered on or after Day 8 of Cycle 1. Begin durvalumab 1,500 mg monotherapy Q4W after Cycle 2. Cycle 2 durvalumab will be administered after the safety of Cycle 1 is confirmed. Thereafter, durvalumab will be administered every 4 weeks until the objective disease progression is confirmed (7.4.), "5.6. Discontinuation of Investigational New Drug" is met, and the study is terminated.

5.5.1. Criterion for reduction

No dose reductions will be made for either durvalumab or tremelimumab in this study.

5.6. Discontinuation of Investigational Drug

If any of the following criteria are met, the investigator or sub-investigator will discontinue the investigational product and perform an Investigational Product Discontinuation Study. A follow-up visit will be scheduled 28 days after the last dose of investigational product.

1) When a subject requests to withdraw from the clinical trial treatment.
2) When it is difficult to continue administration of the investigational drug due to exacerbation of complications
3) When it is difficult to continue administration of the investigational drug due to adverse events.
4) Pregnancy is detected.
5) Other cases in which the investigator or subinvestigator judges it necessary to discontinue the administration of the investigational drug.

Subjects may discontinue investigational treatment at any time, at their own discretion, without prejudice to subsequent treatment. Subjects who decide to discontinue an investigational drug should always be questioned about the reason for discontinuation and the presence or absence of AEs. Subjects who completely discontinue subsequent doses of study medication, regardless of the reason, must continue to attend the clinic for evaluation according to the study protocol. If the subject does not agree to continue the visit, the follow-up procedure may be modified to ensure collection of endpoints and safety information. This follow-up may include telephone contact with the subject, contact with relatives or the treating physician, or information from medical records. Any change in the method of follow-up should be documented in the medical record. Subjects who agree to the change in follow-up are not considered to have withdrawn their consent or to have withdrawn from the trial.

Subjects who discontinue continued treatment with the investigational drug for any reason will be identified as treatment discontinuation. Subjects who discontinue treatment will be transferred to the follow-up period (refer to 7.1.).

Subjects who discontinue treatment for reasons other than objective tumor progression as assessed by imaging will continue to receive imaging every 6 weeks (±1 week) until 12 weeks after study drug initiation, then radiologic PD with imaging every 8 weeks (±1 week), plus additional imaging as defined in the study plan, or death (whichever occurs first), RECIST assessment will continue.

If a subject is determined to have PD as defined by RECIST 1.1, additional imaging studies should be performed within 4 weeks of the determination. (refer to 7.4.).

All subjects will be followed for survival until the end of the trial. Subjects who are unable to return for evaluation will be contacted by telephone as indicated in the trial schedule as an alternative.

5.7. Discontinuation of individual cases from participation in clinical trials

If any of the following criteria are met, the investigator or subinvestigator will discontinue administration of the investigational drug and the subject's participation in the clinical trial.

1) When it is judged difficult to continue the clinical trial for some reason on the subject's side, such as non-attendance or transfer to another hospital.
2) 2When the subject requests to discontinue participation in the clinical trial.
3) When the investigator or subinvestigator determines that the subject is unable to continue the clinical trial.
4) If the subject weighs less than 30 kg
5) When the investigator/divisional investigator determines that a decision to discontinue the study is necessary due to a serious violation of the study protocol, etc.

5.7.1. In case of untraceable cases

A subject is considered lost only if the subject cannot be contacted until the end of the trial and there is insufficient information to determine the subject's status at that time. Subjects who refuse to continue participation in the trial (including telephone contact) will be recorded as "withdrawing consent" rather than "untraceable". Investigators will document the means used to re-establish contact with subjects lost to follow-up throughout the duration of the trial. If the subject who was lost to follow-up is re-contacted, the subject will not be marked as "lost to follow-up" and the evaluation will resume according to the study protocol.

At the time of the OS analysis, the survival status of all subjects in the overall analysis population (FAS) and the safety analysis population will be reconfirmed. Subjects who withdrew consent and subjects classified as "possibly untraceable" will also be subject to this reconfirmation.

5.7.2. Withdrawal of consent

The subject is free to withdraw consent for this clinical trial at any time without prejudice to subsequent treatment.

Subjects who withdraw their consent will not receive any further doses of the investigational drug or follow-up as specified in the protocol. However, consent for survival follow-up will be confirmed separately. Additional tests may be performed after discontinuation to ensure subject safety.

If a subject withdraws consent, the investigator must confirm the reason and the presence or absence of adverse events.

The subject withdrawing consent shall be specifically asked about the following items regarding the details of the withdrawal of consent.

- Withdraw consent for all further participation in the clinical trial, including subsequent follow-up (e.g., telephone calls to investigate survival status).
- Withdraw consent for use of clinical trial data.
- Withdraw consent for the use of any sample.

5.7.3. Clinical investigator's decision

If the risks of participation in a clinical trial outweigh the benefits of the subject's participation in the trial, such as if a life-threatening infusion reaction or systemic infection occurs, the investigator will determine that the subject's participation in the trial cannot continue.

5.7.4. Subject weight loss

If the subject weighs less than 30 kg after enrollment, he/she will be removed from the study.

5.7.5. Other cases

If the investigator determines that the subject is unable to continue participation in the study for other reasons, such as a serious deviation from the protocol, the subject will be removed from the study.

5.8. Subject replacement

If a subject is found to be ineligible for the clinical trial prior to enrollment, the subject will not be enrolled in this trial. In such cases, the investigator will explain to the subject that enrollment in this trial is not possible.

For subjects who do not complete the study treatment for reasons other than DLT criteria during the DLT evaluation period, the investigator will seek input from the Data Monitoring Committee on whether to recruit a new subject to replace that subject.

5.9. Concomitant Restricted Drugs and Concomitant Restricted Therapy

The coordinating investigator must be informed of all medications taken from the time of screening until the end of the clinical phase (last visit) as soon as possible. All concomitant medications, including herbal medications taken during the trial are recorded in the CRF.

Restricted, prohibited, and permitted concomitant medications are listed in Tables 1 and 2.

Table1. 5.9. Concomitant Restricted Drugs and Concomitant Restricted Therapy

**Prohibited drug**	**Rules of use**
Anticancer drugs as investigational drugs other than the investigational drugs in this study	Concurrent use is prohibited during administration of investigational drugs.
mAb against CTLA-4, PD-1 or PD-L1 other than the investigational drug mAb for PD-L1 in this study	Concurrent use is prohibited during administration of investigational drugs.
Any concomitant chemotherapy, radiation therapy, immunotherapy, biologic therapy, or hormonal therapy for the treatment of cancer other than the investigational drug in this study	Any concomitant chemotherapy, radiation therapy, immunotherapy, biologic therapy, or hormonal therapy for the treatment of cancer other than the investigational drug in this study
Immunosuppressive agents such as systemic corticosteroids, methotrexate, azathioprine, or tumor necrosis factor alpha inhibitors at doses of prednisone or its equivalent greater than 10 mg/day<br>Immunosuppressive agents such as systemic corticosteroids, methotrexate, azathioprine, or tumor necrosis factor alpha inhibitors in doses greater than 10 mg/day. Immunosuppressive agents such as, but not limited to, the following	Concomitant administration or premedication is prohibited. The following exceptions are permitted<br>・ Use of immunosuppressive agents for the management of adverse events related to the investigational drug<br>・ Use in subjects allergic to contrast media<br>・ Use of inhaled, topical, and intranasal corticosteroids<br>・ Non-immunotherapy that is clinically necessary and has occurred in the subject<br>・ Temporary use of steroids is acceptable if deemed essential for the management of related events (e.g., chronic obstructive pulmonary disease, radiation therapy, nausea)
Epidermal Growth Factor Receptor Tyrosine Kinase Inhibitors（Epidermal Growth Factor Receptor Tyrosine Kinase Inhibitors (EGFR TKIs)	Prohibit concomitant use.<br>Use with caution for 90 days after the last dose of durvalumab. An increased incidence of pulmonary inflammation (in combination with a third-generation EGFR TKI) and an increased incidence of transaminases (in combination with a first-generation EGFR TKI) have been reported when durvalumab is used concomitantly.
Attenuated live vaccine	Concomitant use of the investigational drug is prohibited until 30 days after the last dose of the investigational drug.
Drugs with laxative action and herbs or natural remedies for constipation	Concomitant use is prohibited.
Blood transfusion (red blood cell concentrate, platelets)	Concomitant use is prohibited during the DLT evaluation period.
Granulocyte colony-forming stimulating factor (G-CSF) preparation	Concomitant use is prohibited during the DLT evaluation period.

Table2. Concomitant tolerated drugs

Tolerated Drugs	Rules of use
Concomitant medications or treatments (e.g., acetaminophen or diphenhydramine) as deemed necessary for appropriate prophylactic or symptomatic treatment. However, drugs included in the "Prohibited Drugs" section above are excluded.	Administer according to the prescription of the investigator, etc.
Best supportive care (including antimicrobials, nutritional support, correction of metabolic disturbances, optimal symptom control and pain management [e.g., palliative radiation therapy for non-target lesions])	For all subjects, use when necessary.
Inactivated viruses such as influenza vaccines	Can be inoculated (e.g. with vaccine)

5.10. Follow-up treatment

Post-treatment after completion or discontinuation of the clinical trial is not specified.

5.11. After discontinuation of this clinical trial

Subjects who discontinue the clinical trial will be subjected to necessary examination and observation, and appropriate measures will be taken as necessary, until it can be medically determined that the subject can be discharged or transferred to a hospital.

6. Clinical trial treatment

6.1. Durvalumab and tremelimumab

Refer to the investigator's brochure for details and handling of the investigational drug. The following are the investigational drugs to be used in this clinical trial.

6.1.1. Durvalumab

Durvalumab (MEDI4736) will be supplied by AstraZeneca as a 500-mg vial solution for infusion after dilution. The solution contains 50 mg/mL durvalumab, 26 mM histidine/histidine hydrochloride, 275 mM trehalose dihydrate, and 0.02% weight/volume (w/v) polysorbate 80; it has a pH of 6.0 and density of 1.054 g/mL. The nominal fill volume is 10.0 mL.

Durvalumab is a sterile, clear to opalescent, colorless to slightly yellow solution, free from visible particles.

Investigational product vials are stored at 2°C to 8°C (36°F to 46°F) and must not be frozen. Investigational products should be kept in original packaging until use to prevent prolonged light exposure.

6.1.1.1. Preparation of Durvalumab Dose by Infusion Bag

Each dose of durvalumab must be prepared by aseptic manipulation by the investigator or other investigational drug administrator designated by the site. The time between the puncture of the vial and the start of administration of Durvalumab must not exceed the following

- 2°C to 8°C for 24 hours
- 4 hours at room temperature
- Dosing solutions must be brought to room temperature before administration.

Dose 1,500 mg of durvalumab is administered with an infusion bag containing 0.9% saline or 5% dextrose to achieve a final concentration of 1 to 20 mg/mL of durvalumab and an intravenous administration set with a 0.2 μm or 0.22 μm filter. Add 1,500 mg of durvalumab (i.e., 30.0 mL of durvalumab) to the infusion bag. The infusion bag should be selected to achieve a final concentration within 1 to 20 mg/mL. Gently invert and mix the infusion bag until the administered fluid in the bag is uniform. The standard infusion time is 1 hour (±5 minutes). If the infusion is interrupted, the total infusion time should not exceed 8 hours at room temperature. No other drugs should be administered simultaneously in the same IV line.

After the contents of the infusion bag have been completely administered, flush the IV line with an IV diluent equal to the priming volume of the IV set used, or complete the infusion according to the provider's policy to ensure that the full volume is administered. If the line is not flushed, document this in the record. If the preparation time or infusion time limit is exceeded, a new vial must be used to prepare a new dose solution. Since durvalumab does not contain preservatives, unused preparation solutions must be discarded.

The preparation should be made in accordance with the "Procedures for the Administration of Investigational Drugs" for this clinical trial.

6.1.2. Tremelimumab

Tremelimumab will be supplied by AstraZeneca either as a 400-mg or a 25-mg vial solution for infusion after dilution. The solution contains 20 mg/mL tremelimumab, 20 mM histidine/histidine hydrochloride, 222 mM trehalose dihydrate, 0.27 mM disodium edetate dihydrate, and 0.02% weight/volume (w/v) polysorbate 80; it has a pH of 5.5 and density of 1.034 g/mL. The nominal fill volume is 20.0 mL for the 400-mg vial and 1.25 mL for the 25-mg vial.

Tremelimumab is a sterile, clear to opalescent, colorless to slightly yellow solution, free from or practically free from visible particles.

Investigational product vials are stored at 2°C to 8°C (36°F to 46°F) and must not be frozen.

Investigational products should be kept in original container packaging until use to prevent prolonged light exposure.

6.1.2.1. Preparation of Tremelimumab Dose by Infusion Bag

Each dose of tremelimumab must be prepared by aseptic manipulation by the investigator or the investigation drug manager designated by the site. The time from vial puncture to administration must not exceed

- 2°2°C to 8°C for 24 hours
- 4 hours at room temperature
- Doing solutions must be brought to room temperature before administration.

Tremelimumab is administered in an infusion bag containing 0.9% saline or 5% dextrose to achieve a final concentration of 0.10-10 mg/mL of tremelimumab, using an intravenous administration set with a 0.2 µm or 0.22 µm filter.

Add 300 mg of tremelimumab (i.e., 15.0 mL) to the infusion bag. Gently invert and mix the infusion bag until the dosing solution in the bag is homogeneous. The standard infusion time is 1 hour (±5 minutes); any infusion time less than 55 minutes will be considered a deviation from the study protocol. If the infusion is interrupted, the total infusion time should not exceed 8 hours at room temperature. No other drugs should be administered simultaneously in the same IV line. After the contents of the infusion bag have been completely administered, flush the IV line with an IV diluent equal to the priming volume of the IV set used, or complete the infusion according to the site's policy to ensure that the full volume is administered. If the line is not flushed, document this. If the preparation time or infusion time limit is exceeded, a new vial must be used to prepare a new dose solution. Since tremelimumab does not contain preservatives, unused preparation solutions must be discarded. The preparation should be made in accordance with the “Protocol for the Administration of Investigational Medicinal Products” for this clinical trial.

6.2. Control Drugs

There is no control drug in this study.

6.3. Monitoring during administration

During and after the infusion, the subject's condition should be monitored by assessment of vital signs at the times specified in the study protocol.

If an infusion-related reaction of grade 2 or less is observed, the infusion rate of the study drug may be reduced by 50% or discontinued until the event is resolved, and the infusion may be restarted at 50% of the initial infusion rate until the infusion is completed. Subjects who experience an infusion-related reaction of grade 2 or less may receive subsequent infusions at 50% of the initial rate. Acetaminophen and/or antihistamines (e.g., diphenhydramine) or equivalent drugs according to institutional standards may be administered at the investigator's discretion. If infusion-related reactions are grade 3 or greater, the investigational drug should be discontinued. The standard infusion duration is 1 hour, but if interrupted, the infusion should not exceed 8 hours at room temperature. Refer to the Toxicology and Management Guidelines in the protocol appendix for management of subjects who experience an infusion-related reaction.

As with other antibodies, allergic reactions to dose administration may occur. Appropriate drugs and medical devices to treat acute anaphylactic reactions must be readily available, and investigators must be trained to recognize and treat anaphylaxis. The site must have immediate access to an emergency resuscitation team and medical equipment, and the ability to admit subjects to the intensive care unit if necessary.

6.4. Management of investigational drugs

1) The investigator coordinator will deliver the investigational drug to the investigator of Chiba University Hospital in accordance with the agreement with the investigational drug

provider.

2) The investigator coordinator will properly manage the investigational drug in accordance with the protocol provided by the investigator through the site director.
3) The investigator shall prepare a document explaining the storage conditions, expiration date, and other handling methods of the investigational product and deliver it to the site manager, investigators collaborators, and investigational product manager.

6.5. Disposal of unused investigational drugs

1) After the investigational monitor checks the inventory and obtains the investigator's approval, the investigational drug manager discards used, unused, expired, or damaged investigational drugs and empty containers.
2) The investigational drug manager shall dispose of the investigational drug in accordance with the guidelines for disposal of pharmaceuticals.

6.6. Packaging and labeling of investigational drugs

The label should indicate that the product is for investigational use, the name, title and address of the coordinating investigator (representative), chemical name, volume, serial number, storage method and expiration date of the investigational drug. The label should appear on the bottle and on the package insert.

Labels for investigational new drugs shall be prepared in accordance with "Good Manufacturing Practice (GMP)" and GCP ordinances. The investigational drug label should be written in Japanese. Durvalumab and tremelimumab will be provided in a single sheet format or in a multilingual booklet format.

Investigational drug: Durvalumab (genetical recombination) (MEDI4736) + Tremelimumab (genetical recombination)

The drug name on the durvalumab label shall be "MEDI4736" or "Durvalumab (MEDI4736)" depending on the agreed upon drug name used in the approved clinical trial master label document. During this transition period, either name shall be correct.

6.7. Carbon ion radiotherapy

Information on Carbon ion radotherapy

- Name of medical device: Cardon ion radiotherapy Device
- Indications: Treatment of solid tumors
- Model Number: CI-1000S
- PMDA approval number: 22800BZX00096000
- Manufacturing facility: Toshiba Energy Systems&Solutions Corporation

Carbon Ion Therapy will be performed at the Quantum Science and Technology Agency QST Hospital, and irradiation will be performed for four consecutive days between Day 8 and Day 14 of Cycle 1 as per the schedule in Section 7.1. (If it is a holiday, it is acceptable to irradiate over the holiday.)

Dose prescription and fractionations will be 60Gy (RBE) / 4Fr / 1week for CIRT. RBE calculation is done by modified microdosimetric kinetic model (Inaniwa et al. Phys Med Biol. 2010). Pencil beam scanning technique will be used. The pencil beam covers the PTV voxel by voxel in successive layers. An optimization function drives the dose distribution in each treatment spot to reach the desired target coverage and organs at risk sparing.

All cases will undergo fiducial marker insertion prior to treatment preparation. Fiducial markers may be implanted under ultrasonography/fluoroscopy surveillance percutaneously or transarterialy.

For cases treated in an orthogonal fixed beam room, immobilization will be achieved with a relatively thick shell (3-mm thickness) made of a low-temperature thermoplastic and hydraulic urethane resin or vacuum-formed cushion to allow a range of beam angles by rotating the treatment table. However, a thinner shell may be used if a rotating gantry is available. In either case, the shell device is fixed by tapping to the table bottom, with tightening or loosening adjustment as required.

A 4D-CT simulation is required to allow for assessment of tumor motion. Fasting for 3 to 8 hours prior to simulation is required to control stomach/duodenum volume. Simulation CTs should be done with a CT slice thickness no greater than 3 mm. The 4D dataset is separated into 10 separate breathing phase bins. The simulation scan used for planning should NOT be performed with intravenous contrast; simulation CT after this planning CT may include contrast (for anatomic information).

Target lesion of the particle therapy will be focused on intrahepatic nodule with MVI. A 5mm margin will be taken as a clinical target volume margin for the feeding nodule, and 1cm margin alongside the vessel for the MVI lesion. Internal motion will be compensated according to 4D-CT movement assessment. Inter-fractional margin will be set to 3mm and combined with internal motion compensation forming a field specific planning treatment volume.

Dose constraints for risk organs are set as follows

- GI tract: D2cm3 ≤ 30Gy (RBE)
- Spinal cord: Dmax ≤ 25Gy (RBE)
- Remnant liver volume (liver volume receiving 30Gy (RBE) or less): 500cm3

In addition, the following liver volume information is collected in conjunction with the remaining liver volume

- Liver V5 Gy (RBE)
- Liver V20 Gy（RBE）

Confirmation of patient positioning is confirmed by orthogonal X-ray images. A maximum displacement of 3 mm in all directions is allowed between the reference and treatment images and is achieved by movement of the couch. Respiratory gating during treatment is mandatory. External respiratory surrogate systems or fluoroscopic tracking may be used for respiratory motion detection.

6.8. subject inclusion

In this “modified 3 + 3 design”, the first three subjects will be enrolled in cohort A. If no DLT is observed in any of these subjects, the trial will enroll additional subjects in Cohort B, who will also receive tremelimumab as follows.

If one subject develops DLT in any cohort, three additional subjects will be enrolled in that same cohort. The development of two or more DLTs in Cohort A will mean that the entire trial will be terminated, and two or more DLTs in Cohort B will indicate that the MTD has been exceeded and the regime in Cohort B will be discontinued. In that case, up to a total of 15 additional subjects will be enrolled in Cohort A.

Schematic of subject incorporation

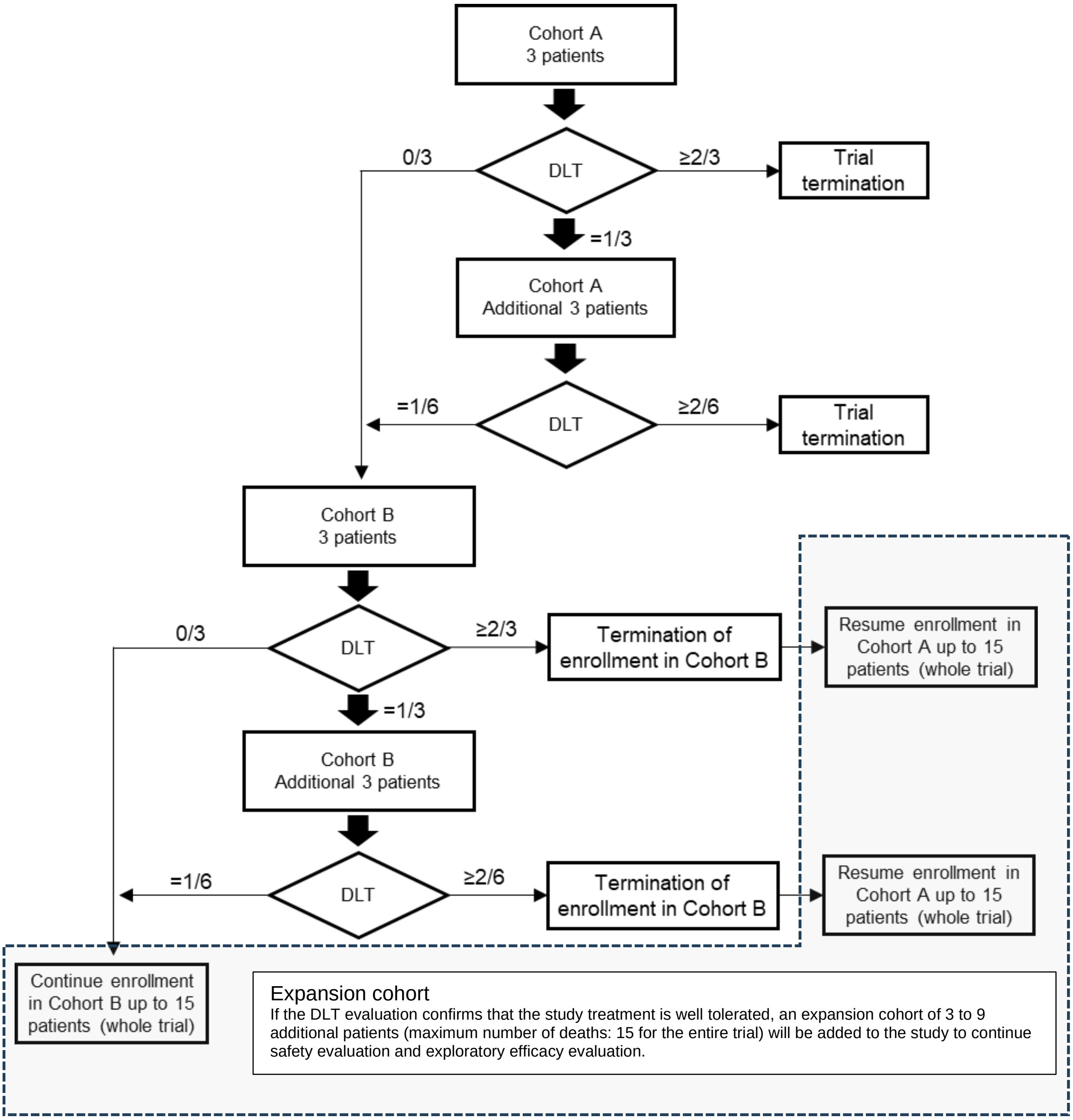

6.9. Definition of Dose-Limiting Toxicity (DLT)

During the 42-day period from the start of study drug administration (Cycle 1/DLT evaluation period), the following toxicities associated with the study treatment are considered DLTs: Grade determination follows CTCAE ver. 5.0.

Dose-limiting toxicity (DLT) will be assessed during the DLT evaluation period of this study, which will be 42 days from the time of the first dose on Cycle 1, Day 1. The severity of DLT will follow the guidelines described in the CTCAE ver. 5.0.

DLT is defined as the occurrence of an adverse event (AE) that is at least potentially related to the investigational drug or regimen (IR), with two exceptions: a Grade of vitiligo or alopecia is not a DLT. An AE that is at least potentially related to a regimen containing durvalumab and/or tremelimumab will be evaluated as a DLT if it meets one of the following criteria.

Patients who have not received heavy ion therapy are excluded from DLT assessment. However, if a patient initiates heavy ion therapy but does not complete heavy ion therapy within the allowed time due to an adverse event that cannot be excluded as being causally

related to durvalumab, tremelimumab, or heavy ion therapy, such adverse event will be considered a DLT.

- **Hematologic toxicity:**
  - Grade ≥3 neutropenia complicated by fever >38.3°C
  - Grade 4 neutropenia (lasting more than 7 days)
  - Grade ≥3 thrombocytopenia with significant bleeding
  - Grade 4 thrombocytopenia (regardless of duration)
  - Grade 4 anemia (regardless of duration)
  -
- **Non-hematologic toxicity:**
  - Any Grade 4 non-immune-mediated AE
  - Any Grade 4 immune-mediated AE, excluding endocrinopathies
  - Any Grade 3 non-immune mediated AE that does not resolve to ≤Grade 1 or baseline within 30 days with optimal medical management
  - Any Grade 3 immune-mediated AE – excluding diarrhea/colitis, pneumonitis, hepatitis, rash, neurotoxicity, myocarditis, myositis/polymyositis, endocrinopathies and nephritis – that does not resolve to ≤Grade 1 or baseline within 30 days after onset of the event despite optimal medical management including systemic corticosteroids
  - Grade 3 diarrhea or colitis that does not resolve to ≤Grade 1 within 14 days
    [both immune- and non-immune-mediated indicated here; the same is the case if not specified in remaining bullet points below]
  - Grade 3 noninfectious pneumonitis
  - Grade 2 noninfectious pneumonitis that does not resolve to ≤Grade 1 within 3 days of the initiation of maximal supportive care
  - Aspartate aminotransferase (AST) or alanine aminotransferase (ALT) ≥3×ULN with concurrent increase in total bilirubin (TBL) ≥2×ULN without evidence of cholestasis or alternative explanations (e.g., viral hepatitis, disease progression in the liver; i.e., “Hy’s Law”)
  - ALT or AST >8×ULN or TBL>5×ULN
  - Grade 3 immune-mediated rash that does not resolve to ≤Grade 1 or baseline within 30 days
  - Grade 2 rash covering > 30% BSA that does not resolve to ≤Grade 1 or baseline within 30 days
  - Any grade of immune-mediated rash with bullous formation
  - Grade 3 immune-mediated neurotoxicity (excluding Guillain-Barre and myasthenia gravis) that does not resolve to ≤Grade 1 within 30 days
  - Grade 2 or 3 immune-mediated peripheral neuromotor syndrome (such as Guillain-Barre and myasthenia gravis) that does not resolve to ≤Grade 1 within 30 days or that exhibits signs of respiratory insufficiency or autonomic instability
  - Grade 3 immune-mediated myocarditis
  - Any symptomatic immune-mediated myocarditis that does not become asymptomatic within 3 days of initiating optimal medical management including systemic corticosteroids
  - Grade 2 or 3 immune-mediated myositis/polymyositis that does not resolve to Grade ≤1 within 30 days of initiating optimal medical management including systemic corticosteroids or that exhibits signs of respiratory insufficiency regardless of optimal medical management
  - Immune-mediated increase in creatinine >3×ULN, or >3×baseline for patients with a baseline creatinine elevated above ULN
  - Transfusion (Red cell concentrate, Platelet), or the use of G-CSF during DLT period

The DLT evaluation period will be from the time of the first dose of study drug/IR until 42 days

post-dose. The first dose of durvalumab in Cycle 2 will be administered after the safety of Cycle 1 is confirmed.

The first treatment-related toxicity that occurs during the DLT period must be followed up to determine if the event qualifies as a DLT as defined in the DLT criteria above.

An immune-related adverse event is defined as an immune (inflammatory) adverse event without a definite other etiology. If an immune-related adverse event is suspected but there are no significant laboratory findings, a repeat laboratory test should be performed before a determination of DLT is made. Subjects who do not complete the DLT evaluation period for reasons other than DLT will not be considered DLT evaluable cases, and DLT will be evaluated after supplementation. The investigator will ask the Independent Data Monitoring Committee for an opinion on the supplementation decision.

Subjects will be administered the first dose of the study drug after hospitalization at Chiba University Hospital. After administration of the investigational drug, the subject will continue to be closely monitored in the hospital and will be transferred to the QST Hospital only for heavy ion therapy, taking into consideration the subject's condition and the means of transportation (taxi, private ambulance, etc.). After completion of heavy particle therapy, the patient should remain in the hospital for at least 7 days. During the transition to outpatient treatment, the investigator will perform the tests specified in 7.2.2 and confirm the safety of the treatment before allowing the patient to continue treatment as an outpatient. In addition, the investigator should be able to contact the site and the medical facility near the home in the event of an adverse event and establish a system to promptly contact the subject in an emergency.

6.10. Toxicity Management

Guidelines for the management of immune-mediated reactions, infusion reactions, and non-immune-mediated reactions to durvalumab are provided in the Durvalumab/Tremelimumab Toxicity Management Guidelines (TMGs).

Appropriate efforts should be made to thoroughly evaluate the subject and rule out neoplastic, infectious, metabolic, toxic, or other etiologies of imAE. Serologic, immunologic, and histologic (biopsy) data should be used to support the diagnosis of imAE, if appropriate. In the absence of a clear alternative etiology, the possibility of an immune-related etiology should be considered. In addition, there are situations in which durvalumab and tremelimumab should be discontinued (see Toxicity Management Guidelines). Dose reductions are not permitted. In case of doubt, consult the investigator. All toxicities will be evaluated according to CTCAE ver. 5.0.

6.11. Restrictions during the clinical trial

6.11.1. Restrictions during the clinical trial

The following restrictions apply during and before and after a given period of time while undergoing investigational treatment.

About Women of Childbearing Potential

Female subjects of childbearing potential who are not abstinent and who plan to have sex with an unprotected male partner must use at least one effective method of contraception from the time of screening until the drug treatment and drug discontinuation period (180 days after the last dose of durvalumab + tremelimumab combination therapy). Unprotected male partners of female subjects of childbearing potential must use a male condom and spermicide during this period. Discussion of stopping contraception after this time should be discussed with the family physician. Temporary abstinence, the rhythm method, and external ejaculation are not acceptable methods of contraception. Females should also not breast-feed during this period.

About men with female partners of childbearing potential

For non-contraceptive male subjects who are unprotected and plan to have sex with a female

partner of childbearing potential, the use of a male condom plus spermicide is mandatory from screening through the entire drug treatment and drug washout period (180 days after the last dose of durvalumab + tremelimumab combination therapy). However, periodic abstinence, rhythm and withdrawal methods are not acceptable methods of contraception. Male subjects will refrain from donating sperm during this period.

Female partners of male subjects (of childbearing potential) should also use highly effective contraceptive methods during this period.

Note: Pregnant women are defined as women who have not undergone sterilization (i.e., bilateral oophorectomy, bilateral oophorectomy, or total hysterectomy) or as premenopausal.

A woman is considered postmenopausal if she has been amenorrheic for 12 months without another medical cause. The following age-specific requirements apply

- Women under age 50 are considered postmenopausal if they have been amenorrheic for at least 12 months after discontinuation of exogenous hormone therapy and have luteinizing hormone and follicle stimulating hormone levels in the postmenopausal range of the institution.
- Women over age 50 are considered postmenopausal if they have been amenorrheic for at least 12 months after discontinuation of all exogenous hormone therapy, if they had radiation-induced menopause more than 1 year before their last menstrual period, and if they had chemotherapy-induced menopause more than 1 year before their last menstrual period.

A highly effective contraceptive method is defined as having a low failure rate (i.e., less than 1% per year) when used consistently and correctly.

6.12. Clinical Trial Procedures

The RECIST evaluation date must be performed as scheduled, regardless of any dosing delays. All other scheduled assessments must be performed at the start of the dosing cycle, and all laboratory and other tests required for dosing must be performed at least 3 days prior to dosing.

Subjects may be allowed to delay dosing under certain circumstances, as described below.

- Dosing may be delayed for either immune- or non-immune-related AEs in accordance with toxicity management guidelines.
- If dosing must be delayed for reasons other than treatment-related toxicities, dosing should be resumed as soon as possible.
- The dosing interval may be shortened as clinically appropriate to gradually match the treatment cycle to the tumor response plan (RECIST). Based on the half-life of durvalumab and tremelimumab, the interval between two consecutive doses should not be less than 22 days (for durvalumab and tremelimumab, see the current investigator's brochure).

7. OBSERVATION, EXAMINATION, AND ASSESSMENT, METHODS, AND TIMING OF IMPLEMENTATION

7.1. Implementation Schedule and Procedures

Cycle	Screening period	First tumor biopsy ※only in consented patients	DLT evaluation period							Second tumor biopsy ※only in consented patients	Durvalumab q4W dosing period					ST	Follow up period（28 days after the last administration date）	Safety information collection（90 days after the last administration date）
			Cycle 1				Cycle 2 [*13]				Cycle 3	Cycle43	Cycle 5	Cycle 6	Cycle 6< until PD			
Cycle Day	D -28 ～D -1	D -28～D -1	1	8 to 14	15	22	1	8	14	After DLT evaluation	1[*1]	1	1	1	1	—	—	—
Allowable period（Day）			—	±3	±3	±3	±3	±3	±3		—	±3	±3	±3	±3	+14	+14	+14
Informed Consent ／ Subject backgroud information／ Review of eligibility	●[*2]																	
Durvalumab administration (cohort A and B)			●				●				●	●	●	●	●			
Tremelimumab administration (cohort B)[*3]			●															
Tumor biopsy		●								●								
CIRT				●●●●														
Fiducial marker insertion[*4]		●																
Fixation, simulation CT (for CIRT)[*4]	●																	
Weight*5	●		●	●	●	●	●	●	●		●	●	●	●	●	●	●	
Height	●																	
Physical exam	●		●	●	●	●	●	●	●		●	●	●	●	●	●	●	
Physical exam（Specific site based on case）	●		●	●	●	●	●	●	●		●	●	●	●	●	●	●	
Vital signs	●		●	●	●	●	●	●	●		●	●	●	●	●	●	●	
ECOG PS	●		●	●	●	●	●	●	●		●	●	●	●	●	●	●	
Clinical Chemistry／Hematology[*6]	●		●	●	●	●	●	●	●		●	●	●	●	●	●	●	
Coagulation	●		●	●	●	●	●	●	●		●	●	●	●	●	●	●	
Urinalysis	●		●								●	●	●	●	●	●	●	
ECG[*7]	●		●													●		
Hepatitis serology [*8]	●											(●)	(●)	(●)	(●)	(●)		
HIVtests	●																	
TSH、fT3、fT4 [*9]	●		●	●	●	●	●	●	●		●	●	●	●	●	●	●	
Pregnancy test [*10]	●																	
Chest X ray	●															●		
Assesment of Child-Pugh score	●		●				●				●	●	●	●	●	●	●	
CT/MRI[*11]	●		Every 6 weeks (±1 week) for the first 12 weeks from Cycle1 day1, and every 8 weeks (±1 week) thereafter until PD.															
Tumor marker（AFP、PIVKA-II）	●						●				●	●	●	●	●			
Cocmitant medication	←――――――――――――――――――――――――――→																	
Assessment of AE/SAE[*12]	←――――――――――――――――――――――――――――――→																	

*1. The baseline for the durvalumab Q4W dosing period will be the first day of the third cycle.
*2. Whenever possible, minimize the time between enrollment and initiation of therapy.
*3. In combination therapy, tremelimumab should be administered first, and durvalumab infusion should be started approximately 1 hour (maximum 2 hours) after tremelimumab administration is completed.
*4. To be administered to subjects who are eligible by other screening tests.
*5. Weight will be measured along with vital signs at the visit.
*6. Serum or plasma biochemical tests (including LFT monitoring) and blood tests may be performed more frequently if clinically indicated.
If screening biochemical and hematological evaluations were performed no more than 3 days prior to Day 1 (the first infusion day), they need not be performed again on Day 1.
Results of LFTs, electrolytes, complete blood count, and creatinine must be obtained prior to the start of infusion (within 3 days) and confirmed by the attending physician or investigator prior to administration.
*7. Three ECG results are required if clinically significant abnormalities are detected.
*8. If HBs-Ag, HBs-Ab, or HBc-Ab is positive, HBV-DNA should be measured (before and every 4 weeks after administration).
If HCV antibodies are positive, measure HCV-RNA (pre-test).
*9. Free T3 or free T4 should be measured only if TSH is abnormal or endocrine system-related AEs are clinically suspected. If TSH is measured up to 14 days prior to the first dose, it need not be measured again on Day 1.
*10. For women of childbearing potential only. For women of childbearing potential, a pregnancy test should be performed every 4 weeks starting 7 days prior to the first dose. Pregnancy tests can be performed on Day 1, but results must be confirmed by the treating physician or investigator before administration is initiated.
*11. RECIST evaluation is performed with CT (preferred) or MRI imaging of the chest, abdomen (including liver and adrenal glands), and pelvis IV contrast preferred. Imaging of the pelvis is recommended only if there is a possibility of primary or metastatic disease in the pelvic region. Additional anatomic imaging should be performed based on the individual subject's signs and symptoms at baseline and at follow-up. Baseline assessments should be performed no later than 28 days prior to the start of study drug administration for each cohort and should be performed as close as possible to the start of study drug or prior to the start of study drug if possible.
Confirmatory testing should be performed within 4 weeks of the prior PD evaluation, preferably at the next scheduled imaging visit (provided there is no clinically significant deterioration). If an unscheduled evaluation is performed and the subject has not progressed, every effort should be made to perform a subsequent evaluation at the next scheduled visit.
*12. AEs and SAEs should be collected from the time of the first dose of study drug until the follow-up date 28 days after the last dose of study drug. However, AE and SAE outcomes should be collected until the end of the safety information collection period (90 days after the last dose of study drug) or until the start of alternative anticancer therapy. In addition, events occurring after the 28-day follow-up period after the last dose of the study drug and considered to be attributable to delayed toxicity to the study drug will be collected as AEs or SAEs until the end of the safety information collection period or until the initiation of alternative anticancer therapy.
*13. *n the Expansion cohort, patients will be moved to Cycle 2 after the end of Cycle 1.

Note: All assessments on the treatment day shall be performed prior to infusion unless otherwise indicated.
ECG Electrocardiogram; LFT Liver function tests; T3 Triiodothyronine; T4 Thyroxine; TSH Thyroid stimulating hormone.

7.2. Observation, tests and assessment

7.2.1. Screening period

The investigator or sub-investigator will perform the following screening tests and enroll subjects who meet the selection criteria and do not violate the exclusion criteria. The tests will be performed after consent is obtained and between 28 days before the start of the investigational drug and the day before the start of the investigational drug. Test items will be as described below. However, test results obtained prior to consent as part of routine medical care using procedures similar to those used in this study may be used as screening tests if they fall within the 28-day screening period.

- Informed Consent
- Subject background information*
- Review of eligibility criteria
- Complete physical exam
- ECOG Performance Status
- Vital signs**, weight and height
- Chest X ray
- 12-lead ECG (in triplicate [2-5 minutes apart])
- Assessment of Child-Pugh score
- Imaging by CT/MRI, if applicable to study
- Clinical laboratory tests for:
    - Clinical Chemistry (see Table 5)
    - Hematology (see Table 4)
    - TSH, fT3, fT4
    - Coagulation (PT, PTT, INR)
    - Creatinine Clearance
    - Pregnancy test (for women of childbearing potential only)
    - Hepatitis serology
    - HIV test
    - Urinalysis
    - Tumor marker (AFP, PIVKA-II)
- Fiducial marker insertion
- Fixation, simulation CT (for CIRT)
- Concomitant medication

* **Subject background information and medical history**

Subject identification code number, race, sex, age at obtaining IC, medical history, concurrent diseases, alcohol consumption, smoking history, and information for HCC with initial diagnosis, pathological diagnosis and past treatment.

** **Vital signs**

Vital signs will be measured at every visit and will include assessments of systolic and diastolic BP, temperature, and HR. Systolic and diastolic BPs will be documented in mmHg. Temperature will be obtained in degrees Celsius. HR will be documented in beats per minute. Generally, each patient will have blood pressure tested in the same arm. Measurement device and measurement time will not be indicated.

7.2.2. DLT assessment period

Day1

- Durvalumab administration (cohort A and B)
- Tremelimumab administration (cohort B）
- Complete physical exam
- ECOG Performance Status
- Vitals signs and weight
- 12-lead ECG (in triplicate [2-5 minutes apart])
- Assessment of Child-Pugh score
- Clinical laboratory tests for:
    - Clinical chemistry
    - Hematology
    - TSH、fT3、fT4
    - Coagulation（PT、APTT、PT-INR）
    - Creatinine Clearence
    - Pregnancy test (women of childbearing potential only)
    - Urinalysis
- Assessment of AE/SAE
- Confirmation of concomitant therapy

Day 8-14

- CIRT (60 Gy (RBE) / 4 Fr) (performed at QST hospital)
- Complete physical exam
- ECOG Performance Status
- Vitals signs and weight
- Assessment of Child-Pugh score
- Clinical laboratory tests for:
    - Clinical chemistry
    - Hematology
    - TSH、fT3、fT4
    - Coagulation（PT、APTT、PT-INR）
    - Creatinine Clearence
- Assessment of AE/SAE
- Confirmation of concomitant therapy

Day 15

- Complete physical exam
- ECOG Performance Status
- Vitals signs and weight
- Assessment of Child-Pugh score
- Clinical laboratory tests for:
    - Clinical chemistry
    - Hematology
    - TSH、fT3、fT4
    - Coagulation（PT、APTT、PT-INR）
    - Creatinine Clearence

- Assessment of AE/SAE
- Confirmation of concomitant therapy

Day 22

- Complete physical exam
- ECOG Performance Status
- Vitals signs and weight
- Assessment of Child-Pugh score
- Clinical laboratory tests for:
    - Clinical chemistry
    - Hematology
    - TSH、fT3、fT4
    - Coagulation（PT、APTT、PT-INR）
    - Creatinine Clearence
- Assessment of AE/SAE
- Confirmation of concomitant therapy

Day 29（Cycle 2 Day1）

- Durvalumab administration (cohort A and B)
- Tremelimumab administration (cohort B）
- Complete physical exam
- ECOG Performance Status
- Vitals signs and weight
- 12-lead ECG (in triplicate [2-5 minutes apart])
- Assessment of Child-Pugh score
- Clinical laboratory tests for:
    - Clinical chemistry
    - Hematology
    - TSH、fT3、fT4
    - Coagulation（PT、APTT、PT-INR）
    - Creatinine Clearence
    - Pregnancy test (women of childbearing potential only)
    - Tumor maker (AFP, PIVKA-II)
- Assessment of AE/SAE
- Confirmation of concomitant therapy

Day 36（Cycle2 Day8）

- Complete physical exam
- ECOG Performance Status
- Vitals signs and weight
- Assessment of Child-Pugh score
- Clinical laboratory tests for:
    - Clinical chemistry
    - Hematology
    - TSH、fT3、fT4
    - Coagulation（PT、APTT、PT-INR）

- Creatinine Clearence

- Assessment of AE/SAE
- Confirmation of concomitant therapy

Day 43（Cycle 2 Day14）

- Complete physical exam
- ECOG Performance Status
- Vitals signs and weight
- Assessment of Child-Pugh score
- Clinical laboratory tests for:
  - Clinical chemistry
  - Hematology
  - TSH、fT3、fT4
  - Coagulation（PT、APTT、PT-INR）
  - Creatinine Clearence
- Assessment of AE/SAE
- Confirmation of concomitant therapy

At discharge during the DLT evaluation period

- Complete physical exam
- ECOG Performance Status
- Vitals signs and weight
- Assessment of Child-Pugh score
- Clinical laboratory tests for:
  - Clinical chemistry
  - Hematology
  - TSH、fT3、fT4
  - Coagulation（PT、APTT、PT-INR）
  - Creatinine Clearence
- Assessment of AE/SAE
- Confirmation of concomitant therapy

7.2.3. Durvalumab q4W dosing period

At Each cycle Day1

- Durvalumab administration (cohort A and B)
- Complete physical exam
- ECOG Performance Status
- Vitals signs and weight
- 12-lead ECG (in triplicate [2-5 minutes apart])
- Assessment of Child-Pugh score
- Clinical laboratory tests for:
  - Clinical chemistry
  - Hematology
  - TSH、fT3、fT4
  - Coagulation（PT、APTT、PT-INR）
  - Creatinine Clearence
  - Pregnancy test (women of childbearing potential only)

- Tumor maker (AFP, PIVKA-II)
- Assessment of AE/SAE
- Confirmation of concomitant therapy

7.2.4. At the time of discontinuation of investigational drug administration

- Complete physical exam
- ECOG Performance Status
- Vitals signs and weight
- 12-lead ECG (in triplicate [2-5 minutes apart])
- Assessment of Child-Pugh score
- Clinical laboratory tests for:
  - Clinical chemistry
  - Hematology
  - TSH、fT3、fT4
  - Coagulation（PT、APTT、PT-INR）
  - Creatinine Clearence
  - Tumor maker (AFP, PIVKA-II)
- Assessment of AE/SAE
- Confirmation of concomitant therapy

7.2.5. Follow up period

- Complete physical exam
- ECOG Performance Status
- Vitals signs and weight
- 12-lead ECG (in triplicate [2-5 minutes apart])
- Assessment of Child-Pugh score
- Clinical laboratory tests for:
  - Clinical chemistry
  - Hematology
  - TSH、fT3、fT4
  - Coagulation（PT、APTT、PT-INR）
  - Creatinine Clearence
  - Urinalysis
  - Tumor maker (AFP, PIVKA-II)
- confirmation of survival
- Assessment of AE/SAE
- Confirmation of concomitant therapy

7.3. Biological sampling procedures

7.3.1. Guideline for blood sampling volume

The total volume of blood to be drawn from each subject in this study is as follows

Amount of blood to be collected from each subject

Assesment		Sample volume (mL) / visit
**Safety**	**Clinical Chemistry Tests**	10

Amount of blood to be collected from each subject

Assesment	Sample volume (mL) / visit
**Hematology Tests**	10
**Total**	20

7.3.2. Blood samples for archiving

When consent is obtained from the subject for storage of blood specimens, residual blood specimens designated for biochemistry and blood tests will be stored at the Department of Gastroenterology, Chiba University Hospital. Blood specimens will be handled in such a way as to prevent leakage, confusion, theft, or loss of personal information by anonymization with an identification code. Blood specimens will be stored for a period not exceeding 20 years after the completion of the clinical trial, after which all specimens will be properly disposed of. If a subject withdraws consent for specimen storage, the specimens will be destroyed and this will be documented. In addition, if specimens are to be used in future research, a new research protocol must document the use of this specimen and be submitted to the IRB for approval.

7.3.3. Hepatic tumor biopsy sample

In this study, a percutaneous liver biopsy/liver tumor biopsy will be performed before the first dose and between 43 and 56 days after the first dose, if the subject is deemed safe for such a biopsy/liver tumor biopsy by the investigator or sub-investigator and if the subject consents. The tissue samples (tumor and non-tumor) obtained will be used for exploratory studies (refer to 9.3.). At that time, the tissue samples will be handled to prevent disclosure, mix-up, theft, or loss of personal information by anonymization with an identification code. The investigator or sub-investigator may discontinue the percutaneous liver biopsy or liver tumor biopsy for the safety of the subject. Failure to perform a percutaneous liver biopsy/liver tumor biopsy at the discretion of the investigator or sub-investigator, or failure to perform a percutaneous liver biopsy/liver tumor biopsy without the consent of the subject, will not preclude enrollment or dosing in this study. Tissue specimens will be retained for a maximum of 20 years after completion of the study, after which all specimens will be properly disposed of. If a subject withdraws consent for specimen storage, the specimen will be discarded and this will be documented. In addition, if specimens are to be used in future research, the use of that specimen must be documented in a new research protocol and submitted to the IRB for approval.

7.4. Asessment of efficiency

The following are guidelines for confirming image evaluations to assess efficacy.

- Imaging evaluations are performed for subject management and treatment decisions.
- Image evaluation is performed using RECIST ver. 1.1.
- In the absence of clinically evident evidence of disease progression, the patient should be re-evaluated after disease progression (PD) is determined by RECSIST ver. 1.1 in order to distinguish between immune checkpoint inhibitor-induced pseudo progression and true disease progression (this is to reduce the risk of study termination due to incorrect evaluation by the investigator/participating physician). (This is to reduce the risk of termination of the study due to incorrect assessment by the investigator/associated investigator.)

The definition of objective disease progression (definite PD) is as follows

1) The presence of clinically evident evidence of disease progression and disease progression (PD) according to RECIST ver. 1.1 is confirmed as objective disease progression.
2) In the absence of clinically evident findings of disease progression (PD) by RECIST ver. 1.1, a second imaging study to evaluate PD according to the specific criteria below should be

performed after disease progression (PD). The partially modified RECIST ver. 1.1 used for objective confirmation of progression is used only to confirm objective disease progression (confirmed PD). Imaging evaluations to determine objective disease progression should be performed within 4 weeks of the first imaging evaluation that determined PD using RECIST ver. 1.1.

The following is RECIST ver. 1.1 modified to establish objective disease progression.

- On two consecutive image evaluations, the sum of the diameters of the target lesions (TL) increases by more than 20% compared to the sum of the smallest diameters and the sum of the diameters increases by more than 5 mm compared to the sum of the smallest diameters.
- Non-target lesions (NTL) and/or pre-existing new lesions showed significant progression (worsening) at the time of the confirmatory examination compared to the most recent imaging evaluation (note: new lesions at the time of imaging evaluation that are determined for the first time to be PD by RECIST ver. 1.1 are evaluated as NTL at the second imaging evaluation).
- The appearance of an obvious new lesion that was not present at the first imaging evaluation that determined PD according to RECIST ver. 1.1 but was present at the second imaging evaluation.

Two consecutive assessments meeting the definition of PD (first PD by RECIST ver. 1.1 and a second PD using the progression confirmation criteria (above)) are required to establish objective disease progression (determination of definite PD). If PD by RECIST ver. 1.1 does not confirm objective disease progression, evaluation will continue until the next PD by RECIST ver. 1.1. In the absence of significant clinical progression, treatment with investigational agents may continue between the first assessment of progression and imaging studies to confirm progression. If PD is confirmed on confirmatory imaging, the date of progression will be the date PD was confirmed on the previous visit. If objective disease progression is not confirmed, the subject will continue to receive study drug and on-therapy evaluation until the next PD, if there is no clinically significant worsening, at which time another confirmatory scan will be required, even if objective disease progression is confirmed. If the initial PD is not immediately confirmed at the next scan, the investigator should not change the PD assessment from the initial scan.

If subjects discontinue treatment (and/or receive subsequent anticancer therapy) prior to radiographic progression, subjects should be followed until objective disease progression is confirmed. Once progression is confirmed, subjects should continue to be followed for survival every 2 months (8 weeks) according to the evaluation follow-up schedule.

7.5. Assessment of safety

7.5.1. Clinical laboratory tests

Blood and urine samples for determination of clinical chemistry, hematology, and urinalysis will be taken at the times indicated in the assessment schedules and as clinically indicated（refer to 7.1.）

Clinical laboratory safety testing, including serum pregnancy testing, is performed in a licensed clinical laboratory according to local standard procedures. Specimen tubes and specimen sizes may vary depending on the laboratory method used and routine practices at the site. Pregnancy testing may be performed at the site using an approved test (urine or serum pregnancy test). Abnormal clinically significant laboratory results should be repeated as soon as possible (preferably within 24 to 48 hours).

Additional safety samples may be collected if clinically indicated at the discretion of the Investigator. The date, time of collection, and results (values, units, and reference ranges) will be recorded on the appropriate eCRF.

The laboratory variables to be measured are presented in Table 4 (Hematology/ Coagulation), Table 5 (Clinical chemistry), and Table 6 (urinalysis).

Other safety tests to be performed at screening include assessment for hepatitis B surface antigen, hepatitis C antibodies, and HIV antibodies.

The following laboratory variables will be measured:

Table4. Hematology/Coagulation Laboratory Tests

Basophils	Monocytes
Eosinophils	Neutrophils
Hematocrit	Platelet count
Hemoglobin	Red blood cell count
Lymphocytes	Total white cell count [a]
PT-INR	APTT

Table5. Clinical Chemistry (Serum or Plasma) Laboratory Tests

Albumin	Lactate dehydrogenase
Alkaline phosphatase	Lipase
Alanine aminotransferase	Magnesium
Amylase	Potassium
Aspartate aminotransferase	Sodium
Calcium	Total bilirubin[a]
Chloride	Total protein
Creatinine	Urea or blood urea nitrogen, depending on local practice
Gamma glutamyltransferase[b]	Uric acid
Glucose	

a. Tests for ALT, AST, alkaline phosphatase, and total bilirubin must be conducted and assessed concurrently. If total bilirubin is ≥2 × upper limit of normal (and no evidence of Gilbert's syndrome) then fractionate into direct and indirect bilirubin.

b. It is preferable that both amylase and lipase parameters are assessed. For sites where only 1 of these parameters is routinely measured then either lipase or amylase is acceptable.

c. Bicarbonate (where available), chloride, creatinine clearance, gamma glutamyltransferase, and magnesium testing are to be performed at baseline, on Day 1 (unless all screening laboratory clinical chemistry assessments are performed within 3 days prior to Day 1), and if clinically indicated.

d. Creatinine Clearance will be calculated by data management using Cockcroft-Gault (using actual body weight).

e. If TSH is measured within 14 days prior to Day 1 (first infusion day), it does not need to be repeated at day Free T3 or free T4 will only be measured if TSH is abnormal or if there is a clinical suspicion of an AE related to the endocrine system

Table 1. Urinalysis Tests[a]

Bilirubin	pH
Blood	Protein
Glucose	Specific gravity
Ketones	Colour and appearance

a. Microscopy should be used as appropriate to investigate white blood cells and use the high-power field for red blood cells

If a patient shows an AST or ALT ≥3xULN together with total bilirubin ≥2xULN, refer to 8.18.3 for further instructions on cases of increases in liver biochemistry and evaluation of Hy's Law. These cases should be reported as SAEs if, after evaluation, they meet the criteria for a Hy's law case or if any of the individual liver test parameters fulfill any of the SAE criteria.

All patients should have further chemistry profiles performed at 30 days (±3 days), 2 months (±1 week) and 3 months (±1 week) after permanent discontinuation of IP

Any clinically significant abnormal laboratory values should be repeated as clinically indicated and recorded on the eCRF. Situations in which laboratory safety results should be reported as AEs are described in Section8.1.

All patients with Grade 3 or 4 laboratory values at the time of completion or discontinuation from IP must have further tests performed until the laboratory values have returned to Grade 1 or 2, unless these values are likely to improve because of the underlying disease.

7.5.2. Physical examinations

Physical examinations will be performed according to the assessment schedules. Full physical examinations will include assessments of the head, eyes, ears, nose, and throat and the respiratory, cardiovascular, GI, urogenital, musculoskeletal, neurological, dermatological, hematologic/lymphatic, and endocrine systems. Height will be measured at screening only. Targeted physical examinations are to be utilized by the Investigator on the basis of clinical observations and symptomatology. Situations in which physical examination results should be reported as AEs are described in Section 8.1.

7.5.3. Electrocardiogram (ECG)

Resting 12-lead ECGs will be recorded at screening and as clinically indicated throughout the study. ECGs should be obtained after the patient has been in a supine position for 5 minutes and recorded while the patient remains in that position.

In case of clinically significant ECG abnormalities, including a QTcF value >470 ms, 2 additional 12-lead ECGs should be obtained over a brief period (e.g., 30 minutes) to confirm the finding.

Situations in which ECG results should be reported as AEs are described in Section 8.1.

7.5.4. Vital signs

Vital signs (blood pressure [BP], pulse, temperature, and respiration rate) will be evaluated according to the schedule of this clinical trial. Body weight is also recorded at each visit along with vital signs.

First infusion

On the first infusion day, patients will be monitored, and vital signs collected/recorded in eCRF prior to, during and after infusion of IP as presented in the bulleted list below.

BP and pulse will be collected from a subject before, during, and after each infusion at the

following times (based on a 60-minute infusion):

- Prior to the beginning of the infusion (measured once from approximately 30 minutes before up to 0 minutes [i.e., the beginning of the infusion])
- Approximately 30 minutes during the infusion (**halfway** through infusion)
- At the end of the infusion (approximately 60 minutes ±5 minutes)

If the infusion takes longer than 60 minutes, then BP and pulse measurements should follow the principles as described above or be taken more frequently if clinically indicated. A 1-hour observation period is recommended after the first infusion of durvalumab.

Subsequent infusions

BP, pulse and other vital signs should be measured, collected/recorded in eCRF prior to the start of the infusion. Patients should be carefully monitored and BP and other vital signs should be measured during and post infusion as per institution standard and as clinically indicated.

7.5.5. ECOG performance status

ECOG performance status will be assessed at the times specified in the assessment schedules based on the following:

0. Fully active; able to carry out all usual activities without restrictions
1. Restricted in strenuous activity, but ambulatory and able to carry out light work or work of a sedentary nature (e.g., light housework or office work)
2. Ambulatory and capable of self-care, but unable to carry out any work activities; up and about more than 50% of waking hours.
3. Capable of only limited self-care; confined to bed or chair more than 50% of waking hours
4. Completely disabled; unable to carry out any self-care and totally confined to bed or chair
5. Dead

Any significant change from baseline or screening must be reported as an AE.

7.5.6. Other safety assesements

If new pulmonary symptoms (e.g., dyspnea) or radiological abnormalities suggestive of pneumonia/interstitial lung damage (ILD) are observed, toxicity management as detailed in the Toxicity Management Guidelines (see Appendix of Protocol 2) will be applied. Complete diagnostic results (including high-resolution computed tomography (HRCT), blood and sputum cultures, hematological parameters, etc.) should be recorded in the source documents. An accurate diagnosis including consultation with a specialist is strongly recommended to rule out alternative causes such as lymphangitic carcinomatosis, infection, allergy, cardiogenic edema, or pulmonary hemorrhage. In the presence of a confirmatory HRCT scan that excludes other causes of respiratory symptoms, the diagnosis of interstitial lung disease (ILD) should be considered and toxicity management guidelines followed. The investigator is responsible for ensuring that all staff involved in the study are familiar with the contents of this section.

8. HANDLING OF ADVERSE EVENT

8.1. Definition

8.1.1. Adverse event

Adverse events are all unwanted or unintended signs (including abnormal changes in laboratory values), symptoms, or illnesses that occur after the first administration of an investigational drug, regardless of causal relationship to the study treatment.

8.1.2. Severe adverse event

A serious adverse event is defined as any of the following

(1) results in death
(2) is life-threaening
(3) requires inpatient hospitalisation or prolongation of existing hospitalisation
(4) results in persistent or significant disability/incapacity
(5) is a congenital anomaly/birth defect
(6) Other serious cases according to the above

The term "life-threatening" for the purposes of this definition is defined as an event that the investigator/participating physician determines poses an imminent risk of death to the subject as a result of its manifestation. It does not mean hypothetically that death might have resulted had the manifestation of the event been more severe.

"Hospitalization" in (3) below is not considered a serious adverse event if any of the following apply: (However, a new occurrence during the hospitalization is treated as an adverse event. (However, any new occurrence during that hospitalization will be treated as an adverse event.)

- Hospitalization as defined in this study protocol
- Hospitalization or prolongation of hospital stay that was planned prior to the start of the clinical trial.
- Hospitalization or prolonged hospitalization for social reasons (reasons of convenience or other non-medical necessity)
- Hospitalization or extended hospital stay for examination, education
- Hospitalization for follow-up or prolongation of hospitalization for those who are cured or have a mild illness.
- Hospitalization or prolonged hospitalization for new treatment of the underlying disease after completion of the investigational drug

(6) Other serious, in accordance with the above, are "significant medical events" that may not be immediately life-threatening or result in death or hospitalization, but which may endanger the subject or require treatment or therapy to avoid the consequences as listed in these definitions.

Adverse events (AEs) for malignancy reported during a clinical trial will generally be evaluated as serious AEs. If no other severity criteria are met, the AE will be judged to be a "Significant Medical Event" as described above. However, in certain circumstances, medical judgment based on individual events should be applied to clarify that malignancy events should be evaluated and reported as non-serious AEs. For example, if medical history includes malignancy and the malignancy progresses during the clinical trial, but the progression does not change the treatment or prognosis of the malignancy, the malignancy progression should be reported as an AE but may not meet the attributes to be evaluated as serious.

The causal relationship of the SAE (relationship to all investigational treatments/procedures) should be evaluated by the investigator and reported to AstraZeneca.

8.1.3. Adverse Events of Special Interest (AESI)

AESIs of durvalumab and tremelimumab for heavy particle irradiation include events due to potential inflammatory or immune-mediated mechanisms, which require more frequent monitoring and treatment with steroids, immunosuppressive agents, and/or hormone replacement therapy.Careful monitoring of these AESIs will be implemented in clinical trials of durvalumab monotherapy and durvalumab plus tremelimumab combination therapy. Immune-mediated

adverse events (imAEs) are AESIs, defined as events that occur with the administration of (exposure to) an investigation agent, are consistent with an immune-mediated mechanism of action, and have no apparent other cause. imAEs should be diagnosed using serologic, immunologic, and histologic (biopsy) data, as appropriate, for support. The diagnosis of imAE should be supported by serologic, immunologic, and histologic (biopsy) data as appropriate, and efforts should be made to rule out tumor, infection, metabolism, toxins, and other causes of imAE.

If there is any doubt as to whether an adverse event is an imAE, the investigator should immediately contact the sponsor's medical experts. AESIs for durvalumab and/or tremelimumab and heavy particle irradiation include the following.

- Dysentery/colitis, intestinal perforation
- Pneumonitis/ILD
- Hepatitis/transaminases increase
- Endocrine disorders (i.e., hypophysitis, hypopituitarism, adrenal insufficiency, hyperthyroidism, hypothyroidism and type I diabetes mellitus)
- Rash/dermatitis
- Nephritis/increased blood creatinine
- Pancreatitis/increased serum lipase and amylase
- myocarditis
- pericarditis
- Myositis/polymyositis
- Neuropathy/neuromuscular toxicity (Guillain-Barré syndrome, myasthenia gravis, etc.)
- Other inflammatory reactions of rare / infrequent immune-mediated (but not limited to pericarditis, sarcoidosis, uveitis, and ocular, skin, blood system and rheumatology-related events)

In addition, reactions associated with infusion and hypersensitivity/anaphylactic reactions due to various pharmacologic causes are also considered AESIs.

Details of these risks and presenting symptoms are described in the most recent versions of the durvalumab and tremelimumab investigational new drug summaries. Specific guidelines for the evaluation and treatment of these AESIs are provided in the Dose Modification and Toxicity Management Guidelines. These guidelines were developed by the investigational drug provider to assist investigators and others in making clinical decisions when treating this type of toxicity. This guideline applies to adverse events that the reporting investigator determines are causally related to the investigational drug/regimen.

If new or worsening pulmonary symptoms (e.g., dyspnea) or radiological abnormalities suggestive of pneumonia/interstitial lung disease are observed, toxicity management as detailed in the “Toxicity Management Guidelines” (see Appendix F) will be applied. Results of complete diagnostic tests (including high-resolution computed tomography (HRCT), blood and sputum cultures, hematological parameters, etc.) should be recorded in the source documents.

An accurate diagnosis, including consultation with a specialist, is strongly recommended to rule out alternative causes such as lymphangitic carcinomatosis, infection, allergy, cardiogenic edema, or pulmonary hemorrhage. On confirmatory HRCT scans where other causes of respiratory symptoms have been ruled out, the diagnosis of interstitial lung disease (ILD) should be considered and toxicity management guidelines followed.

8.1.4. Confirmation of interstitial lung disease (ILD)

To ensure thorough investigation and diagnosis of possible cases of pneumonia, the following evaluations, and additional evaluations as needed, will be performed. Collect the results of the evaluation.

- physical examination

- Evaluate signs and symptoms (cough, shortness of breath, fever, etc.), including auscultation of the lung field.
- Peripheral oxygen saturation (SpO2)

Other

If pneumonia (ILD) is suspected during clinical trial treatment, the following markers should be measured.

if possible

(i) ILD markers (KL-6, SP-D) and β-D-glucan

(ii) Tumor markers. Specific tumor markers associated with disease progression.

Other biochemistry: CRP, LDH

8.2. Assessment of severity

AEs and SAEs; severity will be determined in accordance with CTCAE ver. 5.0 The severity of all other events not listed in the CTCAE will be determined by the severity category from Grade 1 to 5, as determined by the investigator based on medical judgment, will be as follows.

- Grade 1 (mild)
  Events that are usually transient and require only minimal Treatment or therapeutic intervention. The event generally does not interfere with normal activities of daily living.

- Grade 2(Moderate)
  An event that is usually alleviated by additional specific therapeutic intervention. The event interferes with normal daily activities and causes discomfort, but does not pose a risk of serious or permanent harm to the subject.

- Grade 3(Severe)
  An event requiring intensive therapeutic intervention. An event that interferes with normal daily activities or significantly affects the subject's clinical condition.

- Grade 4(life-threatening)
  Events and/or immediate sequelae related to the following Imminent risk of death or a physical or mental impairment that affects or limits the ability to perform activities of daily living (eating, walking, toileting, etc.).

- Grade 5(deadly)
  Death as a result of an event. it is important to distinguish between serious criteria and severity of AEs.

It is important to distinguish between severity and severity of an AE. Severity is a measure of intensity, and severity is defined by the criteria in section 10.3.1 A Grade 3 AE need not necessarily be considered an SAE. For example, a Grade 3 headache lasting several hours would not meet the regulatory definition of an SAE and would be considered a non-serious event, whereas a Grade 2 attack leading to hospitalization would be considered an SAE.

8.3. Record of adverse events and serious adverse events

AEs and SAEs will be collected from the time of the first dose of study drug until the follow-up date 28 days after the last dose of study drug. However, AE and SAE outcomes will be collected until the end of the safety information collection period (90 days after the last dose of study drug) or until the start of alternative anticancer therapy. Of events occurring after the 28-day follow-up period after the last dose of the investigational drug, if the event is considered to be due to delayed toxicity to the investigational drug, it will be collected as an AE or SAE until the end of the safety information collection period or until the start of alternative anticancer

therapy. All AEs and SAEs will be actively followed up for each subject for the duration of the trial as long as the event is ongoing. Every effort will be made to resolve all events, even if the event continues after the subject discontinues the investigational drug or after the study is terminated.

AEs unresolved at the subject's last clinical trial visit will be followed up by the investigator as long as medically necessary but will not be further documented in the eCRF. AstraZeneca reserves the right to request additional information from subjects with ongoing AEs/SAEs at the end of the trial if deemed necessary.

For each AE, the following information should be collected

- Adverse event name
- Date of adverse event and date of disappearance
- Maximum CTCAE Grade
- Severity
- Assessment of causal relationship with study treatment
- Treatments related to investigational drugs
- Treatment of adverse events: Administration of AE medication
- outcome

In addition to the above, the following are also confirmed for SAE.

- Date of serious adverse event
- Date when the investigator becomes aware of the onset of the serious adverse event
- Definitions applicable to the determination of serious adverse events
- Date of admission
- Date of discharge
- Presumed reason for death
- Date of death
- Autopsy
- Evaluation of causal relationship with clinical trial procedures
- Evaluation of causal relationship with other drugs
- Details of serious adverse events

The grading scale described in CTCAE ver. 5.0 is used for all events that have been assigned to a CTCAE Grade; for events that have not been assigned a CTCAE Grade, the CTCAE criteria for converting mild, moderate, and severe events to a CTCAE Grade A copy of CTCAE ver. 5.0 can be downloaded from the Cancer Treatment Evaluation Program website at http://www.jcog.jp/doctor/tool/ctcaev5.html.

8.4. Duration of recording and follow-up of adverse events and serious adverse events

If the subject discontinues treatment for reasons other than objective confirmation of disease progression and therefore continues tumor evaluation, the drug- or treatment-related SAEs will be tracked until the subject confirms PD and no further tumor evaluation is performed.

The investigator is responsible for tracking all SAEs until the subject returns to baseline status or until the condition stabilizes with the expectation that the chronic condition will be maintained, even if it continues beyond study participation, until all SAEs are resolved.

8.5. Causal relationship with investigational therapy

The following examples will be used to determine the causal relationship between the clinical trial treatment and the patient.

Causative: if a reasonable possibility can be explained that the investigational treatment caused the adverse event in question (examples are given below).

1) If the event is time-related to the onset of the event, and the event attenuates with the passage of time after the study treatment but recurs or worsens with subsequent re-administration of the study treatment.
2) The presence of confounding risk factors is negative, such as the subject's general condition, complications, concomitant medications, or concomitant therapies.

No causal relationship: other than above

8.6. Outcome definition

Outcomes after adverse events are determined from the following

1) Recovery: when the patient recovers to the state before the adverse event occurred.
2) Recovered but with sequelae: When the adverse event has recovered but the effects of the adverse event remain as sequelae
3) Death: when the adverse event that occurred was the direct cause of death
4) Lightening of symptoms: adverse events continue, but symptoms are improving
5) Unrecovered: adverse events continue (symptoms are not improving)
6) Unknown: When the subject is no longer traceable

8.7. Treatment of investigational drug in the event of an adverse event

1) No change: When an adverse event occurs but there is no change in the conditions under which the investigational drug is administered.
2) Discontinuation: Discontinuation of study drug administration due to the occurrence of an adverse event
3) Withdrawal: Temporary suspension of study drug administration due to the occurrence of an adverse event
4) Not applicable: Adverse events occur before the start of study drug administration or after the end of the study period

8.8. Treatment of heavy particle therapy equipment in the event of an adverse event

1) Suspension: Temporary suspension of heavy-ion radiation therapy due to the occurrence of adverse events
2) Discontinuation: Discontinuation of heavy-ion radiation therapy due to the occurrence of an adverse event
3) Not applicable: Adverse events occur before or after the start or completion of carbon ion radio therapy

8.9. Relationship to Protocol Procedures

The investigator must also provide an assessment of the relationship between SAEs and protocol procedures on the SAE Report Form. This includes both non-therapeutic emergencies (SAEs that occur prior to the administration of the investigational drug) and therapeutic emergency SAEs. Protocol-related SAEs may occur because of a required procedure or intervention (e.g., blood draw) during the clinical trial. The investigator should use the following guidelines to assess the relationship between SAEs and protocols

- Protocol-related: The event occurred because of a procedure or intervention described in the protocol for which no alternative etiology exists in the subject's medical record.
- Not protocol-related: The event is related to an etiology other than the procedure or intervention described in the protocol. The alternative etiology must be documented in the study subject's medical record.

In the case of AEs, AEs on treatment (or AEs appearing on treatment) are defined as AEs that began after or before administration and worsened after exposure to treatment between the start dose date and 90 days after discontinuation of study treatment.

8.10. Adverse events based on signs and symptoms

All AEs reported spontaneously by the subject or in response to questions from the investigator, subinvestigator, or collaborator (example of a visit: “Have you had any health problems since your last visit / have you been asked any questions since your last visit?”) “)

When collecting AEs, recording the diagnosis is preferred over recording a list of signs and symptoms, if possible. However, if the diagnosis is known and there are other signs and symptoms that are not generally part of the diagnosis, record the diagnosis and each sign or symptom separately.

8.11. Adverse events based on tests and examinations

Protocol-mandated laboratory values and vital signs measurements will be summarized in the CSR. Therefore, protocol-mandated worsening of laboratory values and vital signs relative to baseline should only be reported as an AE if it meets one of the SAE criteria or is a reason for discontinuation of treatment with the investigational drug.

If the worsening of laboratory values or vital signs is associated with clinical signs or symptoms, the signs or symptoms should be reported as an AE and the associated laboratory results or vital signs should be reported as additional information, as appropriate. Whenever possible, the reporter uses clinical terms rather than laboratory terms (e.g., “anemia” rather than “low hemoglobin level”). In the absence of clinical signs or symptoms, the worsening of such laboratory values should be reported as an AE.

Laboratory deterioration attributable to apparent disease progression shall not be considered an adverse event or serious adverse event.

Report as an adverse event any new or worsening clinically significant abnormal findings at the time of presentation compared to the baseline evaluation.

8.12. Hy's Law

Biochemical elevations suggestive of abnormal liver function may require further evaluation, and the occurrence of AST or ALT ≥ 3 x ULN and total bilirubin ≥ 2 x ULN may require reporting as an SAE. For cases of elevated liver biochemistry and Hy's Law evaluation, see Ref.

8.13. Disease progression

Disease progression is considered a worsening of the subject's condition due to the disease for which the investigational drug is being studied and is an increase in the severity of the disease under study and/or an increase in the symptoms of the disease. The development of new metastases or progression of existing metastases to the primary cancer being studied is considered disease progression and is not considered an AE. Events that are clearly attributable to disease progression should not be reported as AEs during a clinical trial.

8.14. New cancer

The development of new cancer shall be considered a serious adverse event. New cancers are those that occur after a patient is enrolled in the study, rather than those that were the primary reason for enrollment in the study. New metastatic lesions are considered progression of the cancer under study and are not reported as a second cancer.

8.15. Deaths

All deaths occurring during the investigational treatment period or within the protocol-defined follow-up period after the last dose of the investigational drug shall be reported as follows.

- Events that are clearly attributable to disease progression will not be treated as investigational AEs, but if the outcome of the event is found to be severe, all SAEs, not limited to death, will be reported, as described in Section 10.14.
- The investigator shall immediately report to the site director and the investigational drug provider, regardless of the causal relationship between the SAE and the investigational drug/IR, regardless of whether the death is due to progression of the disease under study. In addition, a note will be made in the eCRF.
- The causes are identified and described in the eCRF.
- Deaths of unknown cause should always be reported as SAEs.
- If an autopsy is performed, the autopsy results should be reported to AstraZeneca.

Deaths that occur after the last dose of the investigational drug and after the protocol-defined safety information collection period should also be noted in the eCRF as required. If the death is attributable to an event that occurred after the safety information collection period and the event is considered to be due to delayed toxicity to the investigational drug, it must also be reported as an SAE.

AstraZeneca retains the right to request additional information for subjects with ongoing AE(s)/SAE(s) at the end of the trial if deemed necessary.

8.16. Reportable Adverse Events

The Investigator or Co-Investigator will ensure that all adverse events that occurred from the time consent is obtained until 28 days after the end of study drug administration are consistently described in the case report form and that adverse events disappear or that the study period is observed until 4 weeks after the end of the study period (after discontinuation). Adverse events that are judged to have a causal relationship with the study drug will continue to be observed as much as possible until the end of the study period. However, this does not apply if the investigator determines that the safety of the subject is sufficiently ensured and further follow-up is not necessary.

8.17. Reporting of serious adverse events

All serious adverse events during the clinical trial (regardless of whether there is a causal relationship with the investigational drug or not) and serious adverse events suspected to be related to the investigational drug after the completion (discontinuation) of the clinical trial shall be reported according to the following procedures. The details of the reporting procedures are separately stipulated in the “Standard Operating Procedures for Handling Safety Information". An outline of the reporting procedure is provided below.

1） Reports from the investigator to the head of the site and to investigators at other sites

If any serious safety information is recognized during the clinical trial period, the investigator shall immediately report the details to the head of his/her institution, the investigational drug supplier, and the investigators at other sites using the “Report on Serious Adverse Events and Malfunctions” (Uniform Form 12, or the same detailed description form, if necessary). In reporting, the investigator shall specify whether or not the serious safety information is an event that cannot be predicted from the investigator’s brochures, etc.

2） Discussion between the investigator and the Clinical Trial Coordinating Committee

The investigator shall consult with the Trial Coordinating Committee and report his/her opinion as the investigator (including the necessity of reporting to the Minister of Health, Labour and Welfare) to the Trial Coordinating Committee. The investigator shall report his/her opinion (including the necessity of reporting to the Minister of Health, Labour and Welfare) to the Trial Coordinating Committee. If the Independent Data Monitoring Committee is consulted regarding the investigator's judgment, the opinion of the Independent Data Monitoring Committee shall be

followed.

3） Report to the Minister of Health, Labour and Welfare and the heads of other medical institutions

1 Report to the Minister of Health, Labor and Welfare

If the Clinical Trial Coordinating Committee determines that the case is subject to reporting as stipulated in Article 273 of the Enforcement Regulations of the Act on Quality, Efficacy and Safety Assurance of Pharmaceuticals, Medical Devices and Other Products (Pharmaceuticals and Medical Devices Law), it shall report to the Minister of Health, Labor and Welfare (the Pharmaceuticals and Medical Devices Agency, an independent administrative agency (PMDA)). In addition, when the Independent Data Monitoring Committee is consulted, it shall be notified of the contents of the report made to this authority.

2 Reporting to the heads of other implementing medical institutions

When a report is made to the Minister of Health, Labour and Welfare, the investigator of the other site shall report the contents of the “Failure/Infectious Case Report” obtained from the Trial Coordinating Committee to the head of his/her site as soon as possible.

4） Actions to be taken when additional information is obtained

When additional information regarding the adverse event is obtained, the investigator of the site where the adverse event occurred shall make an additional report to the head of the site as soon as possible, as well as to the Clinical Trial Coordinating Committee and the investigational drug supplier. The handling of such additional information shall be in accordance with the procedures described in 1) to 3) above, and reports shall be made to the NIH as necessary.

5） Annual report to the Minister of Health, Labor and Welfare

If the investigational drug has not been approved in Japan, or if the sponsor is not conducting a clinical trial, the person conducting the clinical trial shall submit an annual report to PMDA in accordance with Article 273 of the Ordinance for Enforcement of the Pharmaceutical Affairs Law.

The investigator will report all SAEs to AstraZeneca. The investigator will send a copy of the SAE report submitted to PMDA to the investigational drug provider in accordance with a separate Standard Operating Procedure (SOP). If any changes are made to the follow-up report, the following information will also be promptly sent to the investigational drug provider.

8.17.1. Response to subjects

When a serious adverse event is observed, the investigator or subinvestigator should immediately take appropriate measures, discontinue administration of the investigational drug to ensure the safety of the subject, and inform the subject if treatment for the adverse event becomes necessary. If an adverse event that was considered to be causally related to the investigational drug at the time of discontinuation has not yet recovered, observation will be continued as much as possible until the adverse event recovers or becomes mild, in principle. However, this does not apply when the investigator determines that the subject's safety has been sufficiently ensured and further follow-up is not necessary.

8.18. OTHER EVENTS REQUIRING REPORTING

8.18.1. Overdose

Use of durvalumab or tremelimumab in excess of the prescribed dose of the drug constitutes an overdose. Currently, there is no established treatment for durvalumab or tremelimumab overdose and no established symptoms of a possible overdose. Overdoses with associated AEs are recorded as an AE diagnosis or symptom in the relevant AE module of the eCRF. Overdoses without symptoms are only recorded and reported to the site investigator.

Overdoses of durvalumab or tremelimumab, with or without associated AEs/SAEs, must be recorded and reported to the site investigator. The investigator must report these to the investigational drug provider as described in the SOP.

8.18.2. Hepatic function abnormality

Abnormal liver function that meets Hy's Law criteria should be reported as an SAE regardless of the presence or absence of clinical symptoms. Hy's Law criteria are met if AST or ALT is at least 3 times the upper limit of normal (ULN) or TBL is at least 2 times the upper limit of normal at any time after initiation of treatment with the investigational drug, regardless of elevated ALP.

If it cannot be determined that there is no causal relationship to the investigational drug (e.g., due to cholelithiasis or bile duct obstruction), the investigator should report these events to the study provider in accordance with SOPs.

If the cause of the hepatic dysfunction is established and there is no causal relationship to the investigational drug, the investigator will decide whether to continue the subject's treatment based on the investigator's clinical judgment.

- If the cause of the liver function abnormality is not established, the subject's administration should be discontinued immediately. Follow-up investigations and inquiries will be initiated by the investigational site without delay.

The investigator will follow up on each reported event of liver function abnormality.

8.18.3. Pregnancy

All pregnancies and pregnancy outcomes should be reported to AstraZeneca and the site investigator, except in the following cases

- Pregnancy discovered before the subject received the investigational drug.
- Pregnancy of the male subject's female partner. (If the male subject is not restricted from having children.)

8.18.4. Exposure to pregnant woman

If a subject becomes pregnant during a clinical trial, the investigational drug should be discontinued immediately. Pregnancy itself shall not be considered an AE unless there is a suspicion that the investigational drug interfered with the effectiveness of the contraceptive. Birth defects or birth defects and spontaneous abortions should be reported and treated as SAEs. Uncomplicated elective abortions shall not be treated as AEs. The outcome of all pregnancies (spontaneous abortion, elective abortion, ectopic pregnancy, normal delivery, and congenital anomalies) shall be followed up and documented even if the clinical trial is discontinued. If pregnancy occurs during the clinical trial, the investigator shall notify AstraZeneca in accordance with the SOP.

8.18.5. Exposure to partner

Male subjects should refrain from sexual intercourse or sperm donation with their partners during the study and for 180 days after the last dose of durvalumab plus tremelimumab combination therapy or 90 days after the last dose of durvalumab monotherapy, whichever is longer. Pregnancy in the subject's partner is not considered an AE. However, the outcome of all pregnancies (spontaneous abortion, elective abortion, ectopic pregnancy, normal delivery, or congenital anomaly) occurring between the date of first dose and 180 days after the last dose of durvalumab + tremelimumab combination therapy or 90 days after the last dose of durvalumab

monotherapy, whichever is longer, will be followed up and documented if possible. Follow-up and documentation, if possible, is desirable. Upon receipt of a report of pregnancy, the investigator must obtain the consent of the subject's partner prior to obtaining any information regarding the pregnancy. Therefore, the study team should adopt the generic ICF template according to the procedure and submit it to the Institutional Review Board (IRB) prior to use.

8.19. Medication error

For this study, a medication error is an unintentional error in the course of treatment with an investigational drug that may cause harm to the subject. A medication error is not a lack of efficacy of the investigational drug, but an artificial or process-related failure while the investigational drug is under the control of the site staff or subject. Medication errors include the circumstances under which the error occurred.

- A medication error occurred.
- A medication error occurred, but was identified before the subject received the medication.
- No medication errors occurred, but circumstances were observed that could have resulted in errors.

Examples of events that should be reported in a clinical trial as medication errors

- Confusion of drug names
- Dispensing errors (e.g., some medications were incorrectly dispensed even though they were not actually administered to the subject.
- Drugs that were not administered as directed, such as by incorrect route or site of administration
- Drugs not taken as directed, such as tablets dissolved in water when they should be taken as solid tablets
- Drugs not stored as directed (e.g., in a refrigerator when they should be at room temperature.)
- Subjects who received medication by mistake
  Drugs administered to the wrong subject

Examples of events that need not to be reported as medication errors in clinical research

- Including those that lead to any of the above events or that result in a dose error.
- If the subject fails to take the medication (e.g., forgets to take the medication）
- Overdose Accidents
- Subject did not return unused or empty packaged medications
- Errors associated with background or rescue drugs, even AstraZeneca products, or standard of care drugs in open label studies

Medication errors are not considered AEs, but AEs may occur because of medication errors. If a medication error occurs during a clinical trial, the investigator or other site personnel should contact AstraZeneca within one day.

8.20. Predicted Side Effects

The following is a tabulation of adverse events that occurred in clinical trials of durvalumab. For other adverse events, please refer to the Investigators brochure.

（1）Information on adverse events in 2769 patients treated with durvalumab (all grades, incidence >5%)

Side effect	Occurrence frequency	
Fatigue	769	（27.8 %）
anorexia	584	（21.1 %）
cough	529	（19.1 %）
Nausea	515	（18.6 %）

Breathe bitterly	500 (18.1 %)
constipation	473 (17.1 %)
diarrhea	467 (16.9 %)
fever	393 (14.2 %)
anaemia	348 (12.6 %)
Back pain	342 (12.4 %)
vomiting	339 (12.2 %)
Itching	314 (11.3 %)
lethargy	306 (11.1 %)
Joint pain	299 (10.8 %)
Hypothyroidism	269 (9.7 %)
rash	266 (9.6 %)
headache	265 (9.6 %)
Peripheral edema	258 (9.3 %)
sleeplessness	234 (8.5 %)
bellyache	219 (7.9 %)
Weight loss	205 (7.4 %)
Musculoskeletal pain	194 (7.0 %)
dizziness	189 (6.8 %)
pneumonia	186 (6.7 %)
Urinary tract infections	182 (6.6 %)
Muscle pain	173 (6.2 %)
Upper respiratory tract infections	163 (5.9 %)
AST elevation	162 (5.9 %)
Pain in the extremities	158 (5.7 %)
Hyponatremia	152 (5.5 %)
ALT elevation	149 (5.4 %)
Wet cough	142 (5.1 %)
Nasopharyngitis	140 (5.1 %)

（2）Information on adverse events in 1822 patients treated with durvalumab and tremelimumab in combination (all grades, incidence >5%)

Side effect	Occurrence frequency
fatigue	542 (29.7 %)
diarrhea	485 (26.6 %)
anorexia	461 (25.3 %)
nousea	447 (24.5 %)
pruritic	395 (21.7 %)
constipation	365 (20.0 %)
dyspnea	361 (19.8 %)
anaemia	339 (18.6 %)
fever	305 (16.7 %)
vomiting	286 (15.7 %)
coughing	271 (14.9 %)
backache	247 (13.6 %)
rash	246 (13.5 %)
lethargy	219 (12.0 %)
abdominal pain	217 (11.9 %)
peripheral edema	203 (11.1 %)
weight loss	203 (11.1 %)
arthralgia	195 (10.7 %)
hypothyroidism	186 (10.2 %)

insomnia	175	(9.6 %)
AST elevation	172	(9.4 %)
hyponatremia	172	(9.4 %)
ALT elevation	154	(8.5 %)
headache	172	(8.3 %)
Lipase rise elevation	141	(7.7 %)
dizziness	134	(7.4 %)
amylase elevation	133	(7.3 %)
hypokalemia	131	(7.2 %)
pneumonia	125	(6.9 %)
maculopapular eruption	125	(6.9 %)
Urinary tract infections	117	(6.4 %)
ALP elevation	115	(6.3 %)
dehydration	114	(5.4 %)
Hyperthyroidism	110	(5.1 %)
hyperglycemia	109	(6.0 %)
γ-GTP elevation	103	(5.7 %)
Hypoalbuminemia	100	(5.5 %)
Musculoskeletal pain	99	(5.4 %)
Muscle pain	99	(5.4 %)
Dry skin	98	(5.4 %)
Wet cough	98	(5.4 %)
Xerostomia	95	(5.2 %)
musculoskeletal chest pain	93	(5.1 %)
upper respiratory tract infection	92	(5.0 %)

（3）Refer to Section 2.3.2.3 for more information on the risks of Carbon ion radio therapy.

9. ENDPOINT

9.1. Primary endpoint

Percentage of dose-limiting toxicities (DLT) and adverse events/serious adverse events

Rationale for Setting the Primary Endpoint<br><br>To evaluate the tolerability and safety of durvalumab tremelimumab in combination with carbon ion radio therapy in subjects with advanced hepatocellular carcinoma with vascular invasion.

9.2. Secondary endpoint

Overall survival (OS), 6-month survival, objective response rate (ORR) calculated by RECIST ver 1.1 and mRECIST, 6-month progression-free survival (PFS), Time to progression (TTP)

Rationale for Setting Secondary Endpoints<br><br>To evaluate the efficacy of durvalumab tremelimumab in combination with carbon ion radio therapy in subjects with advanced hepatocellular carcinoma with vascular invasion.

9.3. Exploratory endpoints

In addition to consent for this study, consent for “Exploratory Study of Tumor Cells and Tumor Microenvironment in Hepatocellular Carcinoma Using Tumor and Non-tumor Biopsy Specimens, Blood Specimens, and Stool Specimens” and “Exploratory Study of Tumor Cells and Tumor Microenvironment by Secondary Use of Biological Specimens in Hepatobiliary Pancreatic Cancer” and a liver biopsy or liver tumor biopsy specimen must be In patients with sufficient liver biopsy/tumor biopsy samples, biomarkers associated with clinical outcome of combination therapy with durvalumab/tremelimumab and carbon ion radio therapy will be explored using blood and liver biopsy/tumor biopsy samples. The analysis will be performed in the laboratory of the Department of Gastroenterology, Graduate School of Medicine, Chiba University, and may be contracted to an outside vendor or outside research organization.

10. STATISTICAL METHODS AND SAMPLE SIZE DETERMINATION

10.1. Description of Analysis set

10.1.1. DLT analysis set

All subjects enrolled in the study, who have completed at least one investigational drug regimen (IR), and for whom a DLT evaluation has been performed, are considered the DLT analysis set.

10.1.2. Safety analysis set: SAF

All subjects who have had at least one dose of the investigational drug or investigational drug regimen (IR) are considered in the Safety analysis set.

10.1.3. Full Analysis set: FAS

All subjects with eligible disease who are properly enrolled and have received at least one dose of the investigational drug regimen are considered Full Analysis set (FAS).

10.1.4 Efficacy Evaluable set: EES

The efficacy evaluation analysis(EES) population is defined as a subset of the FAS. That is, it consists of all subjects who are eligible for the trial, receive the study drug/IR, and have had at least one post-dose efficacy endpoint assessed. In addition to subjects with at least one baseline and at least one post-dose efficacy evaluation, the population will include cases of early death or early progression before the evaluation.

10.1.5 Per protocol set：PPS

Subjects from the FAS who do not have any of the following serious violations of the study protocol, including the study protocol, such as the method of study or concomitant therapy, will be considered to be in compliance with the study protocol (PPS).

- Violation of inclusive criteria
- Violation of exclusion criteria
- Violation of concomitant use of prohibited drugs
- Violation of concomitant use of prohibited therapies

10.2. Target number of cases and rationale for setting

Target cases: 3 to 15

A modified "3+3 design" will be used in this trial. Cohort A will be used to evaluate DLT for carbon ion radio therapy + durumab, and instead of dose escalation, the group with toremelimumab will be used as Cohort B to evaluate DLT.

10.3. Case Handling

The coordinating investigators and the statistical analyst will discuss and decide how to handle the registered cases. The coordinating investigator and the statistical analyst will also discuss and decide how to handle cases in case new problems arise.

10.4. Data Handling

In principle, the handling of data during data compilation and analysis shall be as follows. In case of doubt, the statistician and the coordinating Investigators shall discuss and decide the handling of the data. Details are described in the statistical analysis plan.

1) Missing values: Supplementation of missing values is not performed.
2) Reference values: Data with unreliable measured values due to hemolysis, etc. are not used in the aggregate analysis.
3) Time lag: Data not conducted during the specified observation period will not be used in the aggregate analysis for that period. For other data requiring consideration, the coordinating investigators will consult with medical experts to determine how to handle the data for analysis.

10.5. Statistical analysis items and analysis plan

All patients will be analyzed after completion of treatment with the investigational drug and after the data have been fixed, using the DLT evaluation population for DLT evaluation, the ESS analysis population for efficacy evaluation, and the safety analysis population for safety evaluation. For the efficacy evaluation using RECIST without survival analysis, FAS and PPS analysis and sensitivity analysis will be performed as necessary.

10.5.1. Analysis of subject background

The distribution of subject background data and summary statistics are calculated for each cohort. For nominal variables, frequencies and proportions of categories are shown. For continuous variables, summary statistics (number of cases, mean, standard deviation, median, range, and interquartile range) are calculated.

10.5.2. Analysis of primary endpoints

10.5.2.1. DLT Evaluation

1) DLT incidence rate

The number of DLT cases and incidence rate will be calculated for each cohort.

10.5.2.2. Safety Evaluation

1) Adverse event rate
2) Serious adverse event rate
3) Adverse events coded by MedDRA
4) List of SOC, PT, severity, relevance, etc.

10.5.3. Analysis of secondary endpoints

10.5.3.1 Efficacy Analysis

Overall survival (OS), 6-month survival rate, objective response rate (ORR), 6-month progression-free survival (PFS), and time to progression (TTP) will be determined as secondary parameters to evaluate efficacy in each cohort. For survival analysis, the Kaplan-Meier method will be used to determine the incidence rate at 6 months and the median and mean survival times using the ESS analysis group.

Overall Response Rate（ORR）

ORR (based on evaluation by investigators using RECIST 1.1) is defined as the percentage of patients with a CR or PR at one or more visits. Patients who had a response after discontinuation of treatment without PD and initiation of post-treatment are not included in the ORR response cases.

Progression-Free Survival（PFS）

PFS (based on evaluation using RECIST 1.1 by investigators and others) is defined as the time from allocation to objective disease progression or death (or cause of death in the absence of disease progression), regardless of whether the patient discontinued treatment or received other anticancer therapy before disease progression. Patients who have not progressed or died at the time of analysis will be censored at the date of the last RECIST 1.1-based evaluation. However, if a patient has progressed or died after two or more consecutive missed visits, the patient will be censored as of the date of the last RECIST 1.1-based evaluation. In the absence of evaluable visit data or baseline data, censor at Day 1 unless the patient has died between baseline and the second visit, in which case treat as an event with the date of death as the date of the event.

The PFS is always calculated based on the date the imaging study/evaluation was performed, not the date of the visit.

It is possible that an evaluation/imaging study based on RECIST 1.1 scheduled for a specific visit may be performed over several different days. In such cases, the following principles apply.

- For evaluation by the investigator, the earliest RECIST 1.1 evaluation/imaging test date when an element indicating progression is identified is the progression date.PFS
- When an evaluation is terminated, it shall be terminated on the last imaging inspection date of the inspection related to the specific overall effectiveness evaluation.

Time to Progression (TTP)

TTP (based on evaluation using RECIST 1.1 by the investigator and others) is defined as the period from the date of random assignment to the date of objective tumor progression. Death is not included in the definition of TTP. Death without progression is not included in the definition of TTP.

Patients who die without progression will be terminated at the time of death.

Image Evaluation

Imaging evaluation For all imaging evaluations, in addition to evaluation by the investigator or subinvestigators, a central judgment will be made by multiple radiologists to be separately determined.

10.5.4. Interim Analysis

No interim analysis will be performed in this clinical trial.

10.6. Final Analysis

After the follow-up period, analysis will be conducted after the data are obtained and the cases are fixed. The person in charge of statistical analysis compiles the "Analysis Report" and submits it to the coordinating investigator and the principal investigator. The coordinating investigator summarizes the contents of the analysis report and prepares a "summary report" summarizing the overall conclusions of the trial, problems, interpretation and discussion of the results, and future policies mainly from a clinical perspective, and obtains approval from the principal investigator.

10.7. Data Monitoring Committee

An Independent Data Monitoring Committee will be established for this clinical trial. The Independent Data Monitoring Committee will be established as an independent body from the investigators and will consist of three or more expert members who are independent of the study. The Independent Data Monitoring Committee will be established for the purpose of ensuring the safety of subjects. It will provide appropriate advice and recommendations in accordance with a separate protocol If the study is terminated before the end of the DLT evaluation period for reasons other than DLT criteria, the investigator will ask the Independent Data Monitoring Committee for its opinion on the addition of a case If a second DLT occurs, the investigator will ask the Independent Data Monitoring Committee to determine whether the study regimen is If a second DLT occurs, the investigator will ask the Independent Data Monitoring Committee for an opinion on whether the study regimen is intolerable. If necessary, the investigator will confirm whether tolerability was not an issue in the other cases.

If an SAE is reported during the trial, the investigator will check with the Independent Data Monitoring Committee whether to continue the trial and whether any changes should be made to the protocol. The Independent Data Monitoring Committee will provide the investigator with the results of the discussion in writing.

If the investigator determines that precautionary emergency measures are warranted, depending on the importance and scope of the report, actions may include suspension of enrollment and emergency communication to all participating sites.

11. COMPLIANCE AND DEVIATION FROM THE PROTOCOL

1) The investigator or subinvestigator shall conduct the clinical trial in compliance with this protocol.
2) The investigator or subinvestigator shall record the details and reasons for all deviations from the study protocol.
3) In the event of deviation from the protocol for the purpose of avoiding immediate danger to subjects or for other unavoidable medical reasons, the investigator shall immediately submit a document describing the details of the deviation and the reasons for it to the head of the implementing medical institution, and shall also promptly report the contents of said document to the Trial Review Committee via the head of the implementing medical institution. The contents of said documents shall be promptly reported to the Clinical Trial Review Committee via the head of the investigational institution.

12. CHANGES TO THE CLINICAL TRIAL PROTOCOL, CASE REPORT FORM, OR ANALYSIS PLAN

12.1. Revision of Clinical Trial Protocol and Case Report Form

The following procedures shall be followed when revising the clinical trial protocol and case report forms.

1) When the investigator becomes aware of matters related to the quality, efficacy and safety of the investigational drug or other information important for the proper conduct of the clinical trial, the investigator shall revise the relevant protocol as necessary. When a revision is made, a history of the revision shall be prepared and stored.
2) The coordinating investigator shall revise the case report form as necessary in conjunction with the revision of the protocol or for other reasons. Whenever necessary, the coordinating investigator shall revise the case report form in conjunction with the revision of the protocol or for other reasons.
3) The investigator shall promptly submit the revised protocol and revised case report form to the head of the site and obtain approval from the investigational review committee via the head of the site.
4) The same procedure shall be followed when the investigator amends the protocol and case report form within the scope of the investigator's acceptable instructions from the head of the site based on the opinion of the investigational review committee.

12.2. Changes in statistical analysis plan

If the statistical analysis manager changes the contents of the statistical analysis plan, all changes shall be documented in the statistical analysis report for this clinical trial. In addition, the circumstances of any changes to the statistical analysis plan shall be recorded.

13. DISCONTINUATION, SUSPENSION, OR TERMINATION OF THE CLINICAL TRIAL

13.1. Criteria for discontinuation or suspension of the clinical trial as a whole

When the following information is obtained and it is considered difficult to continue the entire clinical trial, the coordinating investigator will consult with the principal investigator and make a decision to discontinue or suspend the entire clinical trial.

- When it becomes difficult to ensure the safety of this clinical trial due to new safety information or serious adverse event information concerning the investigational drug, etc.
- When the site has committed a serious violation of the drug GCP ordinance or a serious deviation from the clinical trial protocol and no improvement has been made.
- Other new information obtained during the conduct of the clinical trial that may necessitate discontinuation or suspension of the clinical trial.

13.2. Procedures for discontinuation or suspension of a clinical trial as a whole

If the coordinating investigator, after consultation with other investigators, decides to discontinue or suspend the entire clinical trial, he/she shall promptly notify the head of the investigational institution and the regulatory authorities in writing to that effect and the reasons in detail. In addition, the investigator shall promptly inform the subjects undergoing the clinical trial and take appropriate measures such as changing to appropriate treatment.

13.3. Procedures for discontinuation or suspension of this clinical trial at an individual clinical site

When the investigator discontinues or suspends a clinical trial, the investigator shall promptly notify the head of the site in writing to that effect and explain the details of the discontinuation or suspension in writing in detail. When notified of the discontinuation or suspension of a clinical trial, the investigator shall promptly notify in writing all investigators and regulatory authorities involved in the said clinical trial to that effect and explain the discontinuation or suspension in detail.

13.4. Termination of this clinical trial

After the completion of the clinical trial, the investigator shall notify the head of the investigational institution in writing that the clinical trial has been completed and report a summary of the results of the clinical trial in writing.

14. DATA MANAGEMENT

14.1. Data management procedure

Detailed procedures for data management shall be described in the data management plan.

14.2. Data Collection

The investigator or sub-investigator shall prepare a case report using Electronic Data Capture (EDC) that meets the requirements of 21 CFR Part 11, the Pharmaceutical GCP Ordinance, and the ER/ES guidelines. The investigator or subinvestigator shall prepare a case report using EDC. The investigator or subinvestigator shall make any changes, corrections or additions to the contents of the case report form on the EDC that generated the case report form, and record all of the changes, corrections or additions as electronic information. When a subinvestigator prepares a case report form or when a collaborator transcribes a case report form from source documents (source data), the investigator shall check the contents of the case report form before submitting it to the EDC and confirm that there are no problems. The investigator provides the final electronic case report form to the institution on an electronic medium (CD-R, etc.). The investigator shall ensure the readability and archivability of the electronic case report form.

When using the EDC system, the site should receive training on EDC and refer to the manual for details on how to input data.

14.3. Identification of documents that are directly described in the case report and should be interpreted as source documents (source data)

In this clinical trial, the following documents and others shall be considered source documents (source data).

1） Medical records, nursing records, clinical laboratory data, imaging films, and other records that form the basis for preparing case reports. Data stored in electronic medical records are also considered source documents.
2） Records of investigational drug administration
3） Documents or records related to the clinical trial that are required under the GCP ordinance for pharmaceutical products related to the clinical trial

Of the data described in the case report form, the following items shall be regarded as source documents (source data) when they are described in the case report form. However, if the data are recorded in the medical record, the medical record shall be regarded as the source documents (original data).

1） Purpose of concomitant medications/adjunctive therapy
2） Determination of the extent of adverse events, outcomes (including results at follow-up), severity, and causal relationship to the study treatment and the basis for the determination
3） Reasons for discontinuation of clinical trials by subjects
4） Comments by the investigator or subinvestigator

15. RETENTION OF SOURCE DOCUMENTS AND OTHER RECORDS

15.1. Retention of records by the clinical site

Documents or records pertaining to a clinical trial to be retained at the investigational site as stipulated in the Pharmaceutical GCP ordinance shall be retained by the hospital director until the later of the following dates.

1) The date on which five years have elapsed since the date of marketing approval for the relevant indication of the test product (if development has been discontinued, the date on which three years have elapsed since the date on which the decision to discontinue

development was made). However, for drugs that are subject to post-approval reexamination in accordance with the provisions of the “Law Concerning Quality, Efficacy and Safety Assurance of Pharmaceuticals and Medical Devices” and for which the period until the reexamination is completed exceeds five years, the date on which the reexamination is completed.

2) The date on which 3 years have elapsed since the discontinuation or termination of the clinical trial.

The investigator shall notify the clinicalsite when the records to be retained by the investigational site or the investigational review committee are no longer required to be retained.

15.2. Retention of records by principal investigators

Documents or records pertaining to clinical trials to be retained by principal investigators as stipulated in the Pharmaceutical GCP Ordinance shall be retained at a storage location deemed appropriate until the later of the following dates.

1) Three years have elapsed since the date of marketing approval for the relevant indication of the test product (if development has been discontinued, three years have elapsed since the date on which the decision to discontinue development was made).
2) The date on which 3 years have elapsed since the discontinuation or termination of the clinical trial.

16. RETENTION OF SAMPLES AND USE OF SAMPLES PROVIDED BY OTHER INSTITUTIONS

16.1. Retention of sample

Samples will be stored in the Laboratory of Gastroenterology, Graduate School of Medicine, Chiba University for a period not exceeding 20 years after completion of the clinical trial. The method of preservation shall be cryopreservation using liquid nitrogen.

16.2. Disposal of samples

If a subject withdraws consent, if a specimen is mistaken or contaminated or is strongly suspected of being mistaken or contaminated, or if the need for disposal is otherwise recognized, the anonymizing numbers, etc., will be deleted and the specimen will be disposed of.

16.3. Reuse of samples

Secondary use of samples and sample-related information (genomic or epigenomic analysis at Chiba University) may be conducted. In such cases, the ethical review and method of obtaining consent shall be in accordance with the corresponding ethical guidelines, etc.

17. SOURCE DOCUMENT VERIFICATION

The head of the investigational site and the investigator ensure that the personnel in charge of monitoring, audits and Institutinal Review Board or regulatory authorities have access to all records, including source documents. In anddtion , The head of the investigational site and the investigator confirm that the clinical trial is conducted appropriately and that the data are sufficiently reliable. The method and timing of source document verification shall be specified separately in the monitoring procedures.

18. Quality Assurance

In order to ensure that clinical trials are conducted and that data preparation, recording and reporting are appropriately conducted in compliance with the protocol and the GCP ordinances for pharmaceutical products, independent auditors from the departments related to the clinical trials, including the department in charge of monitoring, will conduct audits at the investigational

site and other sites where the clinical trials are conducted to confirm that quality control is appropriately conducted.The audit shall be conducted by an auditor independent from the departments related to the clinical trial, including the department in charge of monitoring, to confirm that quality control is appropriately implemented. Audits shall be conducted in accordance with the “Standard Operating Procedures for Audits” and “Audit Plan” separately stipulated.

19. QUALITY CONTROL FOR THIS STUDY

19.1 Training of study site personnel

The Principal Investigator will ensure that appropriate training relevant to the study is given to all of these staff, and that any new information relevant to the performance of this study is forwarded to the staff involved.

19.2. Quality control

In conducting clinical trial monitoring, the investigator shall consider priorities and develop a systematic risk-based approach. In the event of any deviation from this protocol, the investigator or subinvestigator shall follow the provisions of this protocol. The investigator or subinvestigator shall prepare the case report form in accordance with this protocol. The investigator will ensure that all data and other records in the case report form are accurate and complete. If any of the data in the case report form is inconsistent in any way with the original data, the investigator shall prepare and maintain a record explaining the reason for the inconsistency. The investigator shall designate a person who is not engaged in the relevant clinical trial at the site subject to the monitoring as a monitor and have him/her conduct the monitoring in accordance with the monitoring protocol that has been reviewed by the investigational review committee. The monitors shall confirm the following items in accordance with the monitoring protocol separately prepared.

- The human rights, safety and welfare of subjects are protected.
- The clinical trial is conducted in compliance with the Pharmaceutical GCP ordinance, the latest clinical trial protocol, and the procedure manual for the relevant clinical trial.
- To confirm that the data, etc. reported by the investigator or subinvestigator are accurate and complete and check them against the source documents and other clinical trial-related records.

The person in charge of data management shall formulate the data management plan in accordance with the separately established standard operating procedures and shall ensure the quality of the data through quality control at each stage of data handling.

20. ETHICAL CONDUCT AND GOOD CLINICAL PRACTICE(GCP)

This clinical trial will be conducted in accordance with the “Declaration of Helsinki”,the “Law Concerning Quality, Efficacy and Safety Assurance of Pharmaceuticals and Medical Devices” and the “Pharmaceutical GCP Ministerial Ordinance”. In addition, this clinical trial shall be conducted in compliance with the protocol and procedures for this clinical trial.

In selecting subjects, the investigator or subinvestigator shall carefully consider the appropriateness of requesting subjects to participate in this clinical trial based on the selection criteria and exclusion criteria from the perspective of protecting human rights, taking into consideration the subjects' health condition, symptoms, age, gender, ability to consent, degree of dependence on the investigator, and participation status in clinical trials including other clinical trials.

21. INSTITUTIONAL REVIEW BOARD(IRB)

Prior to the implementation of this clinical trial, the Institutional Review Board of the site will review the ethical, scientific and medical appropriateness of this clinical trial. This clinical trial shall be conducted after obtaining the approval of the Clinical Trial Review Committee. If the result of the deliberation by the Institutional Review Board is “Approval with modification,” the protocol or case report form, consent document, etc. shall be modified and approved based on the result of the deliberation, and then this clinical trial shall be conducted. The Institutional Review Board shall also continuously review whether or not this clinical trial is being conducted appropriately at least once a year.

22. HEALTH DAMAGE COVERAGE AND INSURANCE

If a subject suffers health problems as a result of participation in this clinical trial, the investigator will provide treatment and appropriate medical care for the subject's recovery.

As a response to liability for compensation and indemnification arising from health damage caused by this clinical trial, the investigators, subinvestigators, medical institution, Clinical Trial Coordinating Committee and other parties involved in this clinical trial will be covered by the Physician-initiated Clinical Trial Insurance.

23. COST BURDEN FOR THIS TRIAL

The investigational drug to be used in this study will be provided by AstraZeneca. Payment of the burden reduction fee and other expenses to subjects will be in accordance with the rules separately established by each investigational site.

24. TRIAL FUNDS AND CONFLICT OF INTEREST

This clinical trial will be conducted with funds provided to Chiba University Hospital by AstraZeneca. AstraZeneca personnel will not be involved in the conduct or analysis of this clinical trial when the investigators conduct the clinical trial.

In addition, prior to the deliberation of the Clinical Trial Review Committee at each site, the Conflict of Interest Management Committee will deliberate whether conflicts of interest are being properly managed, and it will be confirmed that the investigators and collaborators are not in a state of conflict of interest.

25. PROVISION OF THE INVESTIGATIONAL PRODUCTS AND INTELLECTUAL PROPERTY RIGHTS

In this study, the investigational products are provided by AstraZeneca Pharmaceuticals, Inc. (England) The ownership of the rights to any inventions, discoveries, or improvements of any nature (the “Inventions”) derived from this study will be decided in accordance with the contracts with AstraZeneca.

26.PUBLICATION

The results of the clinical trial will be submitted as a report by the Principal Investigator to the head of the clinical trial site upon completion of this clinical. Results that do not meet expected outcomes despite the proper conduct of the clinical trial must also be disclosed in publication. In case of disclosure of the results to the public, the subject’s personal information must be kept confidential.

27. REGISTRATION FOR CLINICAL TRIAL

This clinical trial will be registered in Japan Registry of Clinical Trial(jRCT) (https://jrct.niph.go.jp/) prior to obtaining consent from the first subject.

28. STUDY IMPLEMANTATION GRPUP

See ANNEX TO PROTOCOL 1

29. LIST OF INVESTIGATIONAL PRODUCT(S) FOR THIS STUDY

Investigational product	Dosage form and strength	Manufacturer
Durvalumab	*50 mg/mL solution for infusion after dilution*	MedImmune/AstraZeneca
Tremelimumab	*20 mg/mL solution for infusion after dilution*	MedImmune/AstraZeneca

30. LIST OF REFERENCES

1) Ministry of Health, Labor and Welfare 2014 Patient Survey
2) Ministry of Health, Labor and Welfare 2017 Vital Statistics
3) El Serag HB, et al., Hepatocellular Carcinoma N Engl J Med 2011
4) The Japan Society of Hepatology Clinical Practice Guidelines for Hepatocellular Carcinoma
5) Llovet JM, Ricci S, Mazzaferro V, et al. Sorafenib in advanced hepatocellular carcinoma. N Engl J Med 2008; 359: 378-90.
6) Cheng AL, Kang YK, Chen Z, et al. Efficacy and safety of sorafenib in patients in the Asia-Pacific region with advanced hepatocellular carcinoma: a phase III randmised, double- blind, placebo-controlled trial. Lancet Oncol 2009; 10:25-34.
7) Bruix J, Qin S, Merle P, et al. Regorafenib for patients with hepatocellular carcinoma who progressed on sorafenib treatment (RESORCE): a randmised, double-blind, placebo-controlled, phase 3 trial. Lancet 2017; 389: 56-66.
8) Kudo M, Finn RS, Qin S, et al. Lenvatinib versus sorafenib in first-line treatment of patients with unresectable hepatocellular carcinoma: a randomised phase 3 non-inferiority trial. Lancet 2018 Mar 24, 391 (10126): 1163-1173
9) Zhu AX, Kan YK, Yen CJ, et al. Ramucirumab after sorafenib in patients with advanced hepatocellular carcinoma and increased α-fetoprotein concentrations (REACH-2): a randomised, double-blind, placebo-controlled, phase 3 trial. Lancet Oncol.2019 Feb;20(2):282-296.
10) Bonze D, Meirson T, Azoulay D. Atezolizumab and Bevacizumab in Hepatocellular Carcinoma. NEJM. 2020 Aug 13;383(7):693-694.
11) Costentin CE, Ferroone CR, Arellano RS, et al. Hepatocellular Carcinoma with Macrovascular Invasion: Defining the Optimal Treatment Strategy Liver Cancer 2017; Nov;6(4):360-374.
12) Dunn GP, Old LJ, Schreiber RD. The three Es of cancer immunoediting. Annu Rev Immunol 2004;22:329-60.
13) Keir ME, Butte MJ, Freeman GJ, Sharpe AH. PD-1 and its ligands in tolerance and immunity. Annu Rev Immunol. 2008;26:677-704.
14) Okazaki T, Honjo T. PD-1 and PD-1 ligands: from discovery to clinical application. Int Immunol 2007;19(7):813-824.
15) Qin A, Coffey DG, Warren EH, Ramnath N. Mechanisms of immune evasion and current status of checkpoint inhibitors in non-small cell lung cancer. Cancer Med 2016;9:2567–2578.
16) Pardoll DM. The blockade of immune checkpoints in cancer immunotherapy. Nat Rev Cancer 2012;12:252-64.
17) Brahmer JR, Tykodi SS, Chow LQM, Hwu WJ, Topalian SL, Hwu P, et al. Safety and activity of

anti-PD-L1 antibody in patients with advanced cancer. N Engl J Med. 2012 Jun;366 (26):2455-65.

18) Hirano F, Kaneko K, Tamura H, Dong H, Wang S, Ichikawa M, et al. Blockade of B7-H1 and PD-1 by monoclonal antibodies potentiates cancer therapeutic immunity. Cancer Res. 2005;65(3):1089-96.

19) Iwai Y, Ishida M, Tanaka Y, Okazaki T, Honjo T, Minato N. Involvement of PD-L1 on tumor cells in the escape from host immune system and tumor immunotherapy by PD-L1 blockade. Proc Natl Acad Sci USA. 2002 Sep 17;99:12293-7.

20) Okudaira K, Hokari R, Tsuzuki Y, Okada Y, Komoto S, Watanabe C, et al. Blockade of B7-H1 or B7-DC induces an anti-tumor effect in a mouse pancreatic cancer model. Int J Oncol. 2009 Sep;35(4):741-9.

21) Topalian SL, Hodi FS, Brahmer JR, Gettinger SN, Smith DC, McDermott DF, et al. Safety, activity, and immune correlates of anti-PD-1 antibody in cancer. N Engl J Med. 2012;366:2443-54.

22) Zhang C, Wu S, Xue X, Li M, Qin X, Li W, et al. Anti-tumor immunotherapy by blockade of the PD-1/PD-L1 pathway with recombinant human PD-1-IgV. Cytotherapy. 2008;10(7):711-9.

23) Powles T, Eder JP, Fine GD, Braiteh FS, Loriot Y, Cruz C, et al. MPDL3280A (anti-PD-L1) treatment leads to clinical activity in metastatic bladder cancer. Nature. 2014 Nov 27;515(7528):558-62.

24) Rizvi N, Brahmer J, Ou S-H, Segal NH, Khleif SN, Hwu WJ. Safety and clinical activity of MEDI4736, an anti-programmed cell death-ligand-1 (PD-L1) antibody, in patients with nonsmall cell lung cancer (NSCLC). J Clin Oncol 2015;33:Abstract 8032.

25) Segal NH, Ou S-HI, Balmanoukian AS, Fury MG, Massarelli E, Brahmer JR, et al. Safety and efficacy of MEDI4736, an anti-PD-L1 antibody, in patients from a squamous cell carcinoma of the head and neck (SCCHN) expansion cohort. J Clin Oncol 2015;33:Abstract 3011.

26) Alexandrov LB, Nik-Zainal S, Wedge DC, Aparicio SAJR, Behjati S, Blankin AV, et al. Signatures of mutational processes in human cancer. Nature. 2013 Aug 22;500:415-21.

27) Fife BT, Bluestone JA. Control of peripheral T-cell tolerance and autoimmunity via the CTLA-4 and PD-1 pathways. Immunol Rev. 2008;224:166-82.

28) El-Khoueiry AB, Sangro B, Yau T, et al. Nivolumab in patients with advanced hepatocellular carcinoma (CheckMate 040): an open-label, non-comparative, phase 1/2 dose escalation and expansion trial Lancet 2017; Jun 24;389(10088):2492-2502.

29) Zhu AX, Finn RS, Edeline J, et al. Pembrolizumab in patients with advanced hepatocellular carcinoma previously treated with sorafenib (KEYNOTE-224): a non-randomised, open-label phase 2 trial. Lancet Oncol. 2018; Jul;19(7):940-952.

30) Kudo M. Targeted and immune therapies for hepatocellular carcinoma: Predictions for 2019 and beyond. World J Gastroenterol 2019 Feb 21;25(7):789-807.

31) Kelly RK, Abou-Alfa GK, Bendell JC, et al. Phase I/II study of durvalumab and tremelimumab in patients with unresectable hepatocellular carcinoma (HCC): Phase I safety and efficacy analyses. J Clin Oncol.2017; Abstract 4073.

32) Pedroni E, Bacher R, Blattmann H, Böhringer T, Coray A, Lomax A, Lin S, Munkel G, Scheib S, Schneider U, et al. The 200-MeV proton therapy project at the Paul Scherrer Institute: conceptual design and practical realization. Med Phys. 1995 Jan;22(1):37-53.

33) Mohamad O., Makishima H., Kamada T. Evolution of Carbon Ion Radiotherapy at the National Institute of Radiological Sciences in Japan. Cancers (Basel) 2018;10(3): pii: E66

34) Kasuya G, Kato H, Yasuda S, Tsuji H, Yamada S, Haruyama Y, Kobashi G, Ebner DK, Okada NN, Makishima H, Miyazaki M, Kamada T, Tsujii H; Liver Cancer Working Group. Progressive

hypofractionated carbon-ion radiotherapy for hepatocellular carcinoma: Combined analyses of 2 prospective trials. Cancer. 2017;123(20):3955-3965

35) Fukuda K, Okumura T, Abei M, Fukumitsu N, Ishige K, Mizumoto M et al. Long-term outcomes of proton beam therapy in patients with previously untreated hepatocellular carcinoma. Cancer Sci. 2017;108(3):497-503

36) Sorin Y, Ikeda K, Kawamura Y, Fujiyama S, Kobayashi M, Hosaka T. Effectiveness of Particle Radiotherapy in Various Stages of Hepatocellular Carcinoma: A Pilot Study. Liver Cancer. 2018 Oct;7(4):323-334

37) Igaki H, Mizumoto M, Okumura T, Hasegawa K, Kokudo N, Sakurai H. A systematic review of publications on charged particle therapy for hepatocellular carcinoma. Int J Clin Oncol. 2018;23(3):423-433

38) Komatsu S, Fukumoto T, Demizu Y, Miyawaki D, Terashima K, Niwa Y, Mima M, Fujii O, Sasaki R, Yamada I, Hori Y, Hishikawa Y, Abe M, Ku Y, Murakami M. The effectiveness of particle radiotherapy for hepatocellular carcinoma associated with inferior vena cava tumor thrombus. J Gastroenterol. 2011 Jul;46(7):913-20.

39) Lee SU, Park JW, Kim TH, Kim YJ, Woo SM, Koh YH, Lee WJ, Park SJ, Kim DY, Kim CM. Effectiveness and safety of proton beam therapy for advanced hepatocellular carcinoma with portal vein tumor thrombosis. Strahlenther Onkol. 2014 Sep;190(9):806-14.

40) Sugahara S, Nakayama H, Fukuda K, Mizumoto M, Tokita M, Abei M, Shoda J, Matsuzaki Y, Thono E, Tsuboi K, Tokuuye K. Proton-beam therapy for hepatocellular carcinoma associated with portal vein tumor thrombosis. Strahlenther Onkol 2009;185:782–788.

41) Antonia SJ, Daniel VD, Vicente D, et al. Durvalumab after Chemoradiotherapy in Stage III Non-Small-Cell Lung Cancer. N Engl J Med. 377;1919-29. 2017

42) Rizvi NA, Cho BC, Reinmuth N, et al. Durvalumab with or without tremelimumab vs platinum-based chemotherapy as first-line treatment for metastatic non-small cell lung cancer: MYSTIC Annals of Oncology.2018 Dec 1;29;supp10

43) Helm A, Ebner DK, Tinganelli W, Simoniello P, Bisio A, Marchesano V, Durante M, Yamada S, Shimokawa T. Combining Heavy-Ion Therapy with Immunotherapy: An Update on Recent Developments. Int J Part Ther. 2018 Summer;5(1):84-93.

44) Takahashi Y, Yasui T, Minami K, Tamari K, Hayashi K, Otani K, Seo Y, Isohashi F, Koizumi M, Ogawa K. Carbon ion irradiation enhances the antitumor efficacy of dual immune checkpoint blockade therapy both for local and distant sites in murine osteosarcoma. Oncotarget. 2019 Jan 18;10(6):633-646.

45) Iijima M, Okonogi N,Izumi Nakajima N, Morokoshi Y, Kanda H, Yamada T, Kobayashi Y, Banno K, Wakatsuki M, Yamada S, Kamada T, Aoki D, Hasegawa S. Significance of PD-L1 expression in carbon-ion radiotherapy for uterine cervical adeno/adenosquamous carcinoma. J Gynecol Oncol. 2020;31:e19.

46) Golden EB, Pellicciotta I, Demaria S, et al. The convergence of radiation and immunogenic cell death signaling pathways. Front Oncol, 2012 Aug 7;2:88.

47) Onishi M, Okonogi N, Oike T, Yoshimoto Y, Sato H, Suzuki Y, Kamada T, Nakano T. High linear energy transfer carbon-ion irradiation increases the release of the immune mediator high mobility group box 1 from human cancer cells. J Radiat Res. 2018 Sep 1;59(5):541-546.

48) Matsunaga A, Ueda Y, Yamada S, Harada Y, Shimada H, Hasegawa M, Tsujii H, Ochiai T, Yonemitsu Y. Carbon-ion beam treatment induces systemic antitumor immunity against murine squamous cell carcinoma. Cancer. 2010 Aug 1;116(15):3740-8.

49) Ando K, Fujita H, Hosoi A, Ma L, Wakatsuki M, Seino KI, Kakimi K, Imai T, Shimokawa T,

Nakano T. Intravenous dendritic cell administration enhances suppression of lung metastasis induced by carbon-ion irradiation. J Radiat Res. 2017 Jul 1;58(4):446-455.

50) Sato H, Niimi A, Yasuhara T, Permata TBM, Hagiwara Y, Isono M, Nuryadi E, Sekine R, Oike T, Kakoti S, Yoshimoto Y, Held KD, Suzuki Y, Kono K, Miyagawa K, Nakano T, Shibata A. DNA double-strand break repair pathway regulates PD-L1 expression in cancer cells. Nat Commun. 2017 Nov 24;8(1):1751. doi: 10.1038/s41467-017-01883-9.

51) Oike T, Niimi A, Okonogi N, Murata K, Matsumura A, Noda SE, Kobayashi D, Iwanaga M, Tsuchida K, Kanai T, Ohno T, Shibata A, Nakano T. Visualization of complex DNA double-strand breaks in a tumor treated with carbon ion radiotherapy. Sci Rep. 2016 Mar 1;6:22275. doi: 10.1038/srep22275.

52) Stewart R, Morrow M, Hammond SA, Mulgrew K, Marcus D, Poon E, et al. Identification and characterization of MEDI4736, an antagonistic anti-PD-L1 monoclonal antibody. Cancer Immunol Res 2015;3(9):1052-62.

53) Tarhini AA, Kirkwood JM. Tremelimumab (CP-675,206): a fully human anticytotoxic T lymphocyte-associated antigen 4 monoclonal antibody for treatment of patients with advanced cancers. Expert Opin Biol Ther 2008;8:1583-93.

54) Pardee AD, Butterfield LH. Immunotherapy of hepatocellular carcinoma: Unique challenges and clinical opportunities. Oncoimmunology 2012;1(1):48-55.

55) Gao Q, Wang XY, Qiu SJ, Yamato I, Sho M, Nakajima Y, et al. Overexpression of PD-L1 significantly associates with tumor aggressiveness and postoperative recurrence in human hepatocellular carcinoma. Clin Cancer Res 2009;15(3):971-9.

56) Hato T, Goyal L, Greten TF, Duda DG, Zhu AX. Immune checkpoint blockade in hepatocellular carcinoma: current progress and future directions. Hepatology 2014;60(5):1776-82.

57) Miroux C, Vausselin T, Delhem N. Regulatory T cells in HBV and HCV liver diseases: implication of regulatory T lymphocytes in the control of immune response. Expert Opin Biol Ther 2010;10(11):1563-72.

58) Golden-Mason L, Palmer B, Klarquist J, Mengshol JA, Castelblanco N, Rosen HR. Upregulation of PD-1 expression on circulating and intrahepatic hepatitis C virus-specific CD8+ T cells associated with reversible immune dysfunction. J Virol 2007;81(17):9249-58.

59) Peng G, Li S, Wu W, Tan X, Chen Y, Chen Z. PD-1 upregulation is associated with HBV-specific T cell dysfunction in chronic hepatitis B patients. Mol Immunol 2008;45(4):963-70.

60) Klein JP, Logan B, Harhoff M, Andersen PK. Analyzing survival curves at a fixed point in time. Stat Med 2007;26(24):4505-19.

61) Sangro B, Gomez MC, Mata M, et al. A clinical trial of CTLA-4 blockade with tremelimumab in patients with hepatocellular carcinoma and chronic hepatitis Cq. J Hepatol. 2013 Jul;59(1):81-8.

62) Duffy AG, Ulahannan SV, Makorova-Rusher O, Rahmer O, Wedemeyer H, Pratt D, et al. Tremelimumab in combination with ablation in patients with advanced hepatocellular carcinoma. J Hepatol 2017;66(3):545-51.

63) Melero I, Sangro B, Cheung Yae T, Hsu C, Kudo M, Crocenzi TS, et al. Nivolumab dose escalation and expansion in patients with advanced hepatocellular carcinoma (HCC): The CheckMate 040 study. J Clin Oncol 2017;35 (suppl 4S:abstract 226).

64) Kelly RK, Sangro B, Harris W, et al. Efficacy, tolerability, and biologic activity of a novel regimen of tremelimumab in combination with durvalumab for patients with advanced hepatocellular carcinoma, J Clin Oncol. 2020; Abstract 4508.

65) Takahashi Y, Fujikawa K, Sagawa T, et al. A phase 1 study to assess the safety and tolerability of tremelimumab alone and in combination with MEDI4736 in Japanese patients withadvanced solid

malignancies. Eur J Cancer. 2015(suppl 3; abst512)

66) Wang E, Kang D, Bae KS, Marshall MA, Pavlov D, Parivar K. Population pharmacokinetic and pharmacodynamics analysis of tremelimumab in patients with metastatic melanoma. J Clin Pharmacol 2014;54(10):1108-16.

67) Narwal R, Roskos LK, Robbie GJ. Population pharmacokinetics of sifalimumab, an investigational anti-interferonalpha monoclonal antibody, in systemic lupus erythematosus. Clin Pharmacokinet 2013;52:1021–27.

68) Ng CM, Lum BL, Gimenez V, Kelsey S, Allison D. Rationale for fixed dosing of pertuzumab in cancer patients based on population pharmacokinetic analysis. Pharm Res 2006;23(6):1275–84.

69) Wang DD, Zhang S, Zhao H, Men AY, Parivar K. Fixed dosing versus body size based dosing of monoclonal antibodies in adult clinical trials. J Clin Pharmacol 2009;49(9):1012–24.

70) Wolchok JD, Kluger H, Callahan MK, Postow MA, Rizvi NA, Lesokhin AM, et al. Nivolumab plus ipilimumab in advanced melanoma. N Engl J Med 2013;369:122-33.

71) Reed GB Jr, Cox AJ Jr. The human liver after radiation injury. A form of veno-occlusive disease. Am J Pathol. 1966 Apr;48(4):597-611.

72) Kanai T, Endo M, Minohara S, Miyahara N, Koyama-ito H, Tomura H, Matsufuji N, Futami Y, Fukumura A, Hiraoka T, Furusawa Y, Ando K, Suzuki M, Soga F, Kawachi K. Biophysical characteristics of HIMAC clinical irradiation system for heavy-ion radiation therapy. Int J Radiat Oncol Biol Phys. 1999 Apr 1;44(1):201-10.

73) Shibuya K, Ohno T, Terashima K, Toyama S, Yasuda S, Tsuji H, et al. Short-course carbon-ion radiotherapy for hepatocellular carcinoma: a multi-institutional retrospective study. Liver Int. 2018;38(12):2239-47.

74) Yasuda S, Kato H, Imada H, et al. Long-Term Results of High-Dose 2-Fraction Carbon Ion Radiation Therapy for Hepatocellular Carcinoma. Adv Radiat Oncol. 2019 Sep 27;5(2):196-203.

75) Shiba S, Shibuya K, Katoh H, et al. A comparison of carbon ion radiotherapy and transarterial chemoembolization treatment outcomes for single hepatocellular carcinoma: a propensity score matching study Radiat Oncol. 2019 Aug 2;14(1):137.

76) Shiba S, Shibuya K, Okamoto M, et al. Clinical impact of Hypofractionated carbon ion radiotherapy on locally advanced hepatocellular carcinoma. Radiat Oncol 2020 Aug 14;15(1):195.

77) Makishima S, Yasuda S, Isozaki Y, et al. Single fraction carbon ion radiotherapy for colorectal cancer liver metastasis: A dose escalation study. Cancer Sci. 2019 Jan;110(1):303-309.

78) Ebner DK, Tinganelli W, Helm A, Bisio A, Yamada S, Kamada T, Shimokawa T, Durante M. The immunoregulatory potential of particle radiation in cancer therapy. Front Immunol. 2017;8:1–8.

Clinical Study Protocol

A phase Ib study of durvalumab (MEDI4736) ± tremelimumab combined with Carbon ion radiotherapy in advanced hepatocellular carcinoma patients with macrovascular invasion

Study Number: CCRC2002
Investigational drugs : Durvalumab, Tremelimumab（Concominant therapy : Carbon ion radiotherapy）
Version Number　1.2
Date　20 Apl 2021

Revision history

Date	Version Number
24 Feb 2021	1.0
26 Mar 2021	1.1
20 Apl 2021	1.2

This protocol includes information and data that contain privileged or confidential information and, is provided only to the investigators, clinical team staff, associates, IRBs, or the Data Monitoring Committee. Therefore, this information must not be made public without written permission from the Chief Investigator, and AstraZeneca except when explaining to subjects. These restrictions on disclosure will apply equally to all or part of the data obtained in the clinical trial for publishing or presentation.

＜Abbreviation and terms＞

Definitions of abbreviations, acronyms, and terms in this study protocol are as follows

Abbreviation /terms	Definition
AE	adverse event
AESI	adverse event of special interest
AFP	α-fetoprotein
AFP-L3	α-fetoprotein - L3
ALP	alkaline phosphatase
ALT	alanine aminotransferase
AMED	Japan Agency for Medical Research and Development
APTT	activated partial thromboplastin time
AST	aspartate aminotransferase
AUC	area under the blood concentration time curve
BICR	blinded independent central review
BP	blood pressure
BSA	body surface area
CD	cluster of differentiation
CI	confidence interval
CIRT	carbon-ion radiotherapy
Cmax	maximum observed concentration
Cmin	minimum concentration during a dosing interval
COI	conflicts of interest
CRP	C-reactive protein
CT	computed tomography
Ctrough	drug concentration at the end of the dosing interval
CTCAE	common terminology criteria for adverse events
CTLA-4	cytotoxic T lymphocyte antigen 4
CTV	clinical target volume
DAMP	damage-associated molecular pattern
DC	dendritic cell
DLT	dose limiting toxicity
DMC	data monitoring committee
DNA	deoxyribonucleic acid
ECG	electrocardiogram
ECOG	Eastern Cooperative Oncology Group
eCRF	electronic case report form
EDC	electronic data capture
EGFR	epidermal growth factor receptor
ER	electronic record
ES	electronic signature
FAS	full analysis set
FTIH	first-time-in-human
FU	follow-up
G-CSF	granulocyte-colony stimulating factor
GCP	Good Clinical Practice
GI	gastrointestinal
HAIC	hepatic arterial infusion chemotherapy

Abbreviation /terms	Definition
HBc	hepatitis B core
HBs	hepatitis B surface
HBV	hepatitis B virus
HCC	hepatocellular carcinoma
HCV	hepatitis C virus
HDV	hepatitis D virus
HIMAC	Heavy Ion Medical Accelerator in Chiba
HIV	human immunodeficiency virus
HMGB1	high mobility group box protein1
HR	hazard ratio
HR	heart rate
HRQoL	health-related quality of life
ICH	International Council for Harmonisation of Technical Requirements for Pharmaceuticals for Human Use
ICI	immune checkpoint inhibitor
IFN-γ	interferon-γ
Ig	immunoglobulin
IL	interleukin
ILD	interstitial lung disease
imAE	immune mediated adverse event
IR	investigational regimen
IRB	institutional review board
KL-6	Krebs von den Lungen-6
LDH	lactate dehydrogenase
LET	linear energy transfer
LFT	liver function test
mAb	monoclonal antibody
MedDRA	Medical Dictionary for Regulatory Activities
MOA	mechanism of action
mRECIST	modified RECIST
MRI	magnetic resonance imaging
MST	mean survival time
MTD	maximum tolerated dose
MVI	macroscopic vascular invasion
NIRS	National Institute of Radiological Sciences
NSCLC	non-small cell lung cancer
NTL	non-target lesion
NYHA	New York Heart Association
OAR	off-axis ratio
ORR	objective response rate
OS	overall survival
PD	progressive disease
PD-1	programmed cell death 1
PD-L1	programmed cell death ligand 1
PD-L2	programmed cell death ligand 2
PIVKA-II	protein induced by vitamin K absence-II

Abbreviation /terms	Definition
PK	Pharmacokinetics
PMDA	Pharmaceuticals and Medical Devices Agency
PPS	per protocol set
PR	partial response
PRO	patient reported outcome
PS	performance status
PT	preferred term
PT-INR	prothrombin time-international normalized ratio
PTV	planning target volume
QxD	quaque x day
QxW	quaque x week
QST	National Institutes for Quantum and Radiological Science and Technology
QTcF	corrected QT interval using Fridericia's formula
Q2W	quaque 2 weeks
Q3W	quaque 3 weeks
Q4W	quaque 4 weeks
RBE	relative biological effectiveness
RECIST	Response Evaluation Criteria In Solid Tumors
RESORCE	Regorafenib after Sorafenib in Patients with Hepatocellular Carcinoma
RFA	radiofrequency ablation
RILD	radiation induced liver damage
RNA	ribonucleic acid
SAE	serious adverse event
SD	stable disease
SHARP	Sorafenib Hepatocellular Carcinoma Assessment Randomized Protocol
SOP	standard operating procedure
SP-D	surfactant protein-D
SpO2	saturation of peripheral oxygen
TACE	transcatheter arterial chemoembolization
TAI	transcatheter arterial infusion
TBL	total bilirubin
TKI	tyrosine kinase inhibitor
TL	target lesion
TLR4	Toll-like receptor 4
TMGs	Toxicity Management Guidelines
TSH	thyroid stimulating hormone
TTP	time to progression
ULN	upper limit of normal
VEGF	vascular endothelial growth factor

TABLE OF CONTENTS

0. CLINICAL TRIAL OVERVIEW....9
1. INTRODUCTION....16
1.1. Introduction....16
1.2. Standard Treatment for Advanced Hepatocellular Carcinoma....16
1.3. HCC with vascular invasion and its treatment....17
1.4. Immunotherapy....17
1.5. Carbon ion radiotherapy....18
1.6. Induction of Immunogenicity by Radiotherapy....18
1.7. Combination of immune checkpoint inhibitors and carbon ion radiotherapy....19
1.8. Investigational drugs....19
1.8.1. Durvalumab....19
1.8.2. Tremelimumab....20
1.8.3. Durvalumab + tremelimumab combination therapy....20
1.8.4. Rationale for Durvalumab and Tremelimumab as Treatment Options for HCC....20
1.9. hypothesis....21
2. STUDTY OBJECTIVE....23
2.1. Objective....23
2.2. Study results regarding the appropriateness of conducting this clinical trial, efficacy, and safety for the subject disease....23
2.2.1. Durvalumab + tremelimumab combination therapy dose rationale....23
2.2.2. Dose rationale for combination regimen of durvalumab 1500 mg Q4W plus tremelimumab 300 mg × 1 dose....23
2.2.2.1.Rationale for utilizing a fixed-dose regimen for durvalumab and tremelimumab....24
2.2.3 Rationale for carbon-ion radiotherapy....24
2.2.4 Rationale for combined treatment of carbon-ion radiotherapy and immunotherapy....25
2.3. Benefit-risk and ethical assessment....25
2.3.1. Potential benefits....25
2.3.2. Overall risks....25
2.3.3. Overall benefit-risk....28
3. ELIGIBILITY....29
3.1. Diagnostic Criteria and Stage, Type, and Condition Classification....29
3.2. Inclusion criteria....29
3.3. Exclusion criteria....30
4. INFORMED CONSENT....33
4.1. Preparation and revision of informed consent form....33
4.2. Method of Obtaining Informed Consent....33
4.3. Information to be provided to subjects....34
5. STUDY DESIGN....35
5.1. Overview of study design....35
5.2. Target number of subjects and study duration....38
5.3. Monitoring for safety assessment....38
5.4. Institutional and case registration methods....39
5.4.1. Facility registration....39
5.4.2. Subject resistration....39
5.4.3. Handling of subjects who are found to be ineligible after enrollment....40
5.5. Dosing schedule and dosage/administration method....40
5.5.1. Criterion for reduction....40
5.6. Discontinuation of Investigational Drug....41
5.7. Discontinuation of individual cases from participation in clinical trials....41
5.7.1. In case of untraceable cases....42
5.7.2. Withdrawal of consent....42
5.7.3. Clinical investigator's decision....42

5.7.4. Subject weight loss 42
5.7.5. Other cases 42
5.8. Subject replacement 42
5.9. Concomitant Restricted Drugs and Concomitant Restricted Therapy 42
5.10. Follow-up treatment 44
5.11. After discontinuation of this clinical trial 44
6. CLINICAL TRIAL TREATMENT 45
6.1. Durvalumab and tremelimumab 45
6.1.1. Durvalumab 45
6.2. Control Drugs 46
6.3. Monitoring during administration 46
6.4. Management of investigational drugs 46
6.5. Disposal of unused investigational drugs 47
6.6. Packaging and labeling of investigational drugs 47
6.7. Carbon ion radiotherapy 47
6.8. subject inclusion 48
6.9. Definition of Dose-Limiting Toxicity (DLT) 49
6.10. Toxicity Management 51
6.11. Restrictions during the clinical trial 51
6.11.1. Restrictions during the clinical trial 51
6.12. Clinical Trial Procedures 52
7. OBSERVATION, EXAMINATION, AND ASSESSMENT, METHODS, AND TIMING OF IMPLEMENTATION 53
7.1. Implementation Schedule and Procedures 53
7.2. Observation, tests and assessment 55
7.2.1. Screening period 55
7.2.2. DLT assessment period 56
7.2.3. Durvalumab q4W dosing period 58
7.2.4. At the time of discontinuation of investigational drug administration 59
7.2.5. Follow up period 59
7.3. Biological sampling procedures 59
7.3.1. Guideline for blood sampling volume 59
7.3.2. Blood samples for archiving 60
7.3.3. Hepatic tumor biopsy sample 60
7.4. Asessment of efficiency 60
7.5. Assessment of safety 61
7.5.1. Clinical laboratory tests 61
7.5.2. Physical examinations 63
7.5.3. Electrocardiogram (ECG) 63
7.5.4. Vital signs 63
7.5.5. ECOG performance status 64
7.5.6. Other safety assesements 64
8. HANDLING OF ADVERSE EVENT 64
8.1. Definition 64
8.1.1. Adverse event 64
8.1.2. Severe adverse event 65
8.1.3. Adverse Events of Special Interest (AESI) 65
8.1.4. Confirmation of interstitial lung disease (ILD) 66
8.2. Assessment of severity 67
8.3. Record of adverse events and serious adverse events 67
8.4. Duration of recording and follow-up of adverse events and serious adverse events 68
8.5. Causal relationship with investigational therapy 68
8.6. Outcome definition 69
8.7. Treatment of investigational drug in the event of an adverse event 69

8.8. Treatment of heavy particle therapy equipment in the event of an adverse event 69
8.9. Relationship to Protocol Procedures 69
8.10. Adverse events based on signs and symptoms 70
8.11. Adverse events based on tests and examinations 70
8.12. Hy's Law 70
8.13. Disease progression 70
8.14. New cancer 70
8.15. Deaths 70
8.16. Reportable Adverse Events 71
8.17. Reporting of serious adverse events 71
8.17.1. Response to subjects 72
8.18. OTHER EVENTS REQUIRING REPORTING 72
8.18.1. Overdose 72
8.18.2. Hepatic function abnormality 73
8.18.3. Pregnancy 73
8.18.4. Exposure to pregnant woman 73
8.18.5. Exposure to partner 73
8.19. Medication error 74
8.20. Predicted Side Effects 74
9. ENDPOINT 77
9.1. Primary endpoint 77
9.2. Secondary endpoint 77
9.3. Exploratory endpoints 77
10. STATISTICAL METHODS AND SAMPLE SIZE DETERMINATION 77
10.1. Description of Analysis set 77
10.1.1. DLT analysis set 77
10.1.2. Safety analysis set: SAF 77
10.1.3. Full Analysis set: FAS 77
10.1.4 Efficacy Evaluable set: EES 77
10.1.5 Per protocol set ： PPS 78
10.2. Target number of cases and rationale for setting 78
10.3. Case Handling 78
10.5.1. Analysis of subject background 78
10.5.2. Analysis of primary endpoints 78
10.5.2.1. DLT Evaluation 78
10.5.2.2. Safety Evaluation 79
11. COMPLIANCE AND DEVIATION FROM THE PROTOCOL 80
12. CHANGES TO THE CLINICAL TRIAL PROTOCOL, CASE REPORT FORM, OR ANALYSIS PLAN 80
13. DISCONTINUATION, SUSPENSION, OR TERMINATION OF THE CLINICAL TRIAL 81
14. DATA MANAGEMENT 82
15. RETENTION OF SOURCE DOCUMENTS AND OTHER RECORDS 82
16. RETENTION OF SAMPLES AND USE OF SAMPLES PROVIDED BY OTHER INSTITUTIONS 83
17. SOURCE DOCUMENT VERIFICATION 83
18. QUALITY ASSURANCE 83
19. QUALITY CONTROL FOR THIS STUDY 84
20. ETHICAL CONDUCT AND GOOD CLINICAL PRACTICE(GCP) 84
21. INSTITUTIONAL REVIEW BOARD(IRB) 84
22. HEALTH DAMAGE COVERAGE AND INSURANCE 85
23. COST BURDEN FOR THIS TRIAL 85
24. TRIAL FUNDS AND CONFLICT OF INTEREST 85

25. PROVISION OF THE INVESTIGATIONAL PRODUCTS AND INTELLECTUAL PROPERTY RIGHTS....85
26.PUBLICATION....85
27. REGISTRATION FOR CLINICAL TRIAL....85
28. STUDY IMPLEMANTATION GRPUP....86
29. LIST OF INVESTIGATIONAL PRODUCT(S) FOR THIS STUDY....86
30. LIST OF REFERENCES....86

0. Clinical Trial Overview

<table>
<tr><td>Title</td><td>A phase Ib study of durvalumab (MEDI4736) ± tremelimumab combined with particle therapy in advanced hepatocellular carcinoma patients with macrovascular invasion</td></tr>
<tr><td>Objective</td><td>

Objectives:

Primary Objective:

To assess the safety and tolerability of combination therapy of durvalumab ± tremelimumab with particle therapy in advanced hepatocellular carcinoma patients with macrovascular invasion

Secondary Objectives:

To assess the efficacy of combination therapy of durvalumab ± tremelimumab with particle therapy in advanced hepatocellular carcinoma patients with macrovascular invasion

</td></tr>
<tr><td>Design</td><td>

Ib Phase, interventional, open-label, single arm

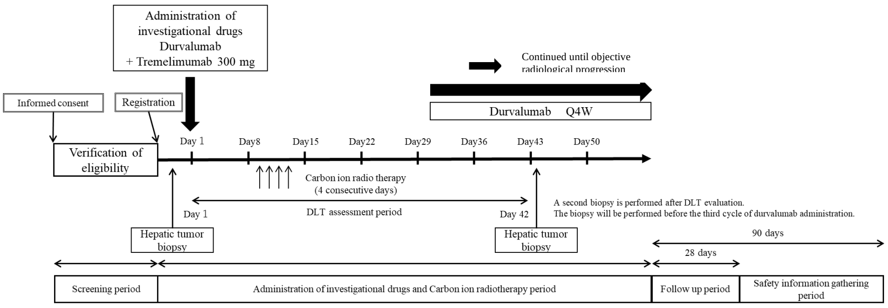

</td></tr>
<tr><td>Phase</td><td>Ib Phase</td></tr>
<tr><td>Investigational Products and Combination Therapy</td><td>

Investigational Products and Combination Therapy:

Investigational Products: Durvalumab and Tremelimumab

Durvalumab (MEDI4736) solution for infusion after dilution will be supplied in glass vials containing 500 mg durvalumab at a concentration of 50 mg/mL.

Tremelimumab solution for infusion after dilution will be supplied in glass vials containing 400 mg or 25 mg tremelimumab at a concentration of 20 mg/mL.

Combination Therapy

Carbon-Ion Radiation Therapy (CIRT) as particle therapy by Toshiba, Carbon ion radio therapy CI-1000S (PMDA approval no. 22800BZX00096000).

</td></tr>
<tr><td>Inclusion Criteria</td><td>

1) Capable of giving signed informed consent which includes compliance with the requirements and restrictions listed in the informed consent form (ICF) and in this protocol. Written informed consent and any locally required authorization obtained from the patient/legal representative prior to performing any protocol-related procedures, including screening

</td></tr>
</table>

	evaluations. For patients aged <20 years and enrolling, a written informed consent should be obtained from the patient and his or her legally acceptable representative.<br>2) Age $\geq$20 years at time of study entry<br>3) Eastern Cooperative Oncology Group (ECOG) performance status of 0 or 1<br>4) Body weight >30 kg<br>5) Adequate normal organ and marrow function as defined below:<br>- Haemoglobin ≧9.0 g/dL<br>- Absolute neutrophil count (ANC) ≧1,500/mm^3<br>- Platelet count ≧75×10^9/L（≧75,000/mm^3）<br>- Serum bilirubin ≦ULN×3.0<br>- AST ≦ULN×5.0<br>- ALT ≦ULN×5.0<br>- Measured creatinine clearance (CL) >40 mL/min or Calculated creatinine clearance CL>40 mL/min by the Cockcroft-Gault formula (Cockcroft and Gault 1976) or by 24-hour urine collection for determination of creatinine clearance<br>6) Evidence of post-menopausal status or negative urinary or serum pregnancy test for female pre-menopausal patients. Women will be considered post-menopausal if they have been amenorrheic for 12 months without an alternative medical cause. The following age-specific requirements apply:<br>- Women <50 years of age would be considered post-menopausal if they have been amenorrheic for 12 months or more following cessation of exogenous hormonal treatments and if they have luteinizing hormone and follicle-stimulating hormone levels in the post-menopausal range for the institution or underwent surgical sterilization (bilateral oophorectomy or hysterectomy).<br><br>- Women ≥50 years of age would be considered post-menopausal if they have been amenorrheic for 12 months or more following cessation of all exogenous hormonal treatments, had radiation-induced menopause with last menses >1 year ago, had chemotherapy-induced menopause with last menses >1 year ago, or underwent surgical sterilization (bilateral oophorectomy, bilateral salpingectomy or hysterectomy).<br><br>7) Patient is willing and able to comply with the protocol for the duration of the study including undergoing treatment and scheduled visits and examinations including follow up.<br>8) Advanced HCC confirmed histologically or by the typical findings of a hypervascular tumor on computed tomography or angiography<br>9) (Cohort A and Cohort B only) Patients who have received at least one prior systemic chemotherapy regimen including atezolizumab bevacizumab combination, sorafenib, or lenvatinib and who are determined to be refractory or intolerant to standard therapy.<br>10) Must not be eligible for locoregional therapy for unresectable HCC. For patients who progressed after locoregional therapy for HCC, locoregional therapy must have been completed ≥28 days prior to the baseline scan for the current study. Acceptable locoregional therapy for HCC are Ethanol Infusion Therapy, Radio Wave ablation Therapy, Transcatheter Arterial chemoembolization (TACE), Transcatheter arterial infusion (TAI). Hepatic Arterial Infusion Chemotherapy (HAIC) is not allowed.<br><br>11) Patients who have been diagnosed with HCC showing MVI. MVI is defined

	as a tumor thrombus in the major hepatic and/or portal vein branches (Vp2, Vp3, Vp4, Vv2, and Vv3) identified by imaging studies.<br>12) Child-Pugh A<br>13) At least one measurable lesion other than the MVI and feeding nodule based on mRECIST.
Exclusion Criteria	1. Involvement in the planning and/or conduct of the study (applies to both sponser and/or staff at the study site)<br>2. Patients who have participated in another clinical trial using the investigational drug within 28 days prior to obtaining consent or who have received another investigational drug within 28 days prior to the first dose of the investigational drug in this study. The exception is if the patient is in the follow-up period of an interventional trial or is participating in an observational (non-interventional) clinical trial.<br>3. Any unresolved toxicity NCI CTCAE Grade ≥2 from previous anticancer therapy with the exception of alopecia, vitiligo, and the laboratory values defined in the inclusion criteria<br>– Patients with Grade ≥2 neuropathy will be evaluated on a case-by-case basis after consultation with the Study Physician.<br>– Patients with irreversible toxicity not reasonably expected to be exacerbated by treatment with durvalumab or tremelimumab may be included only after consultation with the Study Physician.<br>4. Radiotherapy treatment to more than 30% of the bone marrow or with a wide field of radiation within 4 weeks of the first dose of study drug<br>5. Major surgical procedure (as defined by the Investigator) within 28 days prior to the first dose of IP. Note: Local surgery of isolated lesions for palliative intent is acceptable.<br>6. History of allogenic organ transplantation.<br>7. Active or prior documented autoimmune or inflammatory disorders (including inflammatory bowel disease [e.g., colitis or Crohn's disease], diverticulitis [with the exception of diverticulosis], systemic lupus erythematosus, Sarcoidosis syndrome, or Wegener syndrome [granulomatosis with polyangiitis, Graves' disease, rheumatoid arthritis, hypophysitis, uveitis, etc.]). The following are exceptions to this criterion:<br>– Patients with vitiligo or alopecia<br>– Patients with hypothyroidism (e.g., following Hashimoto syndrome) stable on hormone replacement<br>– Any chronic skin condition that does not require systemic therapy<br>– Patients without active disease in the last 5 years may be included but only after consultation with the study physician<br>– Patients with celiac disease controlled by diet alone<br>8. Uncontrolled intercurrent illness, including but not limited to, ongoing or active infection, symptomatic congestive heart failure, uncontrolled hypertension, unstable angina pectoris, cardiac arrhythmia, interstitial lung disease, serious chronic gastrointestinal conditions associated with diarrhea, or psychiatric illness/social situations that would limit compliance with study requirement, substantially increase risk of incurring AEs or compromise the ability of the patient to give written informed consent<br>9. History of another primary malignancy except for<br>– Malignancy treated with curative intent and with no known active disease ≥5 years before the first dose of IP and of low potential risk for recurrence<br>– Adequately treated non-melanoma skin cancer or lentigo maligna without

	evidence of disease<br>– Adequately treated carcinoma in situ without evidence of disease<br>However, the following cases are eligible for enrollment<br>Early stage cancer (epithelial cancer of the cervix, basal cell carcinoma, superficial bladder cancer (Tis and T1), early stage gastric cancer, and early stage colorectal cancer) that has been treated for curative purposes, has not been confirmed active for at least 3 years prior to inclusion in the study, and has a low risk of recurrence.<br>10. History of leptomeningeal carcinomatosis<br>11. History of, or current, brain metastases or spinal cord compression. Patients with suspected brain metastases at screening should have an MRI (preferred) or CT, each preferably with IV contrast of the brain prior to study entry.<br>12. Mean QT interval corrected for heart rate using Fridericia's formula (QTcF) ≥470 ms calculated from 3 ECGs (within 15 minutes at 5 minutes apart) Regardless of whether this criteria stays or not, all patients should have a baseline ECG<br>13. History of active primary immunodeficiency<br>14. Patients co-infected with HBV and HCV, or co-infected with HBV and hepatitis D virus (HDV). HBV positive (presence of HBsAg and/or anti-HBcAb with detectable HBV DNA); HCV positive (presence of anti-HCV antibodies); HDV positive (presence of anti-HDV antibodies), and active infection including tuberculosis (clinical evaluation that includes clinical history, physical examination and radiographic findings, and TB testing in line with local practice).<br>15. Current or prior use of immunosuppressive medication within 14 days before the first dose of durvalumab or tremelimumab. The following are exceptions to this criterion:<br>– Intranasal, inhaled, topical steroids, or local steroid injections (e.g., intra articular injection)<br>– Systemic corticosteroids at physiologic doses not to exceed 10 mg/day of prednisone or its equivalent<br>– Steroids as premedication for hypersensitivity reactions (e.g., CT scan premedication)<br>16. Receipt of live attenuated vaccine within 30 days prior to the first dose of IP. Note: Patients, if enrolled, should not receive live vaccine whilst receiving IP and up to 30 days after the last dose of IP.<br>17. Female patients who are pregnant or breastfeeding or male or female patients of reproductive potential who are not willing to employ effective birth control from screening to 90 days after the last dose of durvalumab monotherapy or180 days after the last dose of durvalumab + tremelimumab combination therapy.<br>18. Known allergy or hypersensitivity to any of the study drugs or any of the study drug excipients.<br>19. Prior randomisation or treatment in a previous durvalumab and/or tremelimumab clinical study regardless of treatment arm assignment.<br>20. Judgment by the investigator that the patient is unsuitable to participate in the study and the patient is unlikely to comply with study procedures, restrictions and requirements.<br>21. Patients who have been treated with anti-PD-1, anti-PD-L1 inhibitors, or other drugs that act on other stimulatory or co-suppressive T-cell receptors and their combinations (including atezolizumab plus bevacizumab) and

	have failed to tolerate the same treatment.<br>22. Prior radiotherapy involving the liver.<br>23. Renal failure requiring hemodialysis or peritoneal dialysis<br>24. Any of the following cardiac diseases:<br>– NYHA Class III or IV chronic heart failure<br>– Current coronary artery disease or history of ischemic heart disease such as myocardial infarction within 6 months before the study<br>– Serious arrhythmia (grade 3 or higher according to the CTCAE ver. 5.0: arrhythmia that cannot be controlled by oral medications or requires mechanical control).<br>25. Poorly controlled hypertension<br>26. Serious and active infection, excluding hepatitis viral infection<br>27. Persistent proteinuria of NCI-CTCAE version 5.0 grade 3 or higher.<br>28. Arterial or venous thrombotic or embolic events such as cerebrovascular accident, deep vein thrombosis, or pulmonary embolism within 6 months before the start of study medication.<br>29. Refractory pleural effusion or ascites<br>30. History of hepatic encephalopathy within past 12 months<br>31. Oral intake impossible<br>32. HIV-positive<br>33. Pulmonary fibrosis or interstitial pneumonitis<br>34. Other serious complications as follows: serious mental disease or history of gastrointestinal bleeding or active hemoptysis<br>35. Unsatisfactory general condition for participation in the study as judged by the primary physician
Endoints	**Primary Endpoints:**<br>AEs/SAEs including DLTs<br><br>**Secondary Endpoints:**<br>Overall Survival (OS), Survival Rate at 6 months, Objective Response Rate (ORR), Time to Progression (TTP), Progression Free Survival (PFS) at 6 months (in accordance with mRECIST)_
Dosage and treatment	Each cycle of this study will last for 28 days, and the DLT evaluation period will be 42 days from the start of study drug administration.<br>Durvalumab 1,500 mg alone or in combination with 300 mg tremelimumab IV on Cycle 1, Day 1.<br>Carbon ion radio theray (60 Gy (RBE) / 4 times / 4 days) is administered between days 8-15 of the first cycle.<br>After the DLT evaluation period, durvalumab is continued every 4 weeks until tumor progression is confirmed.
Criteria for discontinuation of individual cases	1) In case it is judged difficult to continue the clinical trial for some reason on the subject's side, such as non-attendance or transfer to a different hospital.<br>2) In case the subject requests to discontinue participation in the clinical trial<br>3) In case the investigator or subinvestigator determines that the subject is unable to continue the clinical trial.<br>4) In case the subject's body weight becomes less than 30 kg.<br>5) In case the investigator/participating investigator determines that a decision to discontinue.

<table>
<tr>
<td>Number of Patients Planned:</td>
<td>

Number of Patients Planned:

15 subjects.

The number of patients enrolled for DLT assessment in either Cohort A or Cohort B may vary from 3 to 6 depending on the frequency of DLT. If both of Cohort A and B regimens were confirmed tolerable after DLT assessment, additional patients will be enrolled in Cohort B up to a total of 15 subjects. If only Cohort A regimen was determined to be tolerable, additional patients may be enrolled in Cohort A up to a total of 15 subjects.

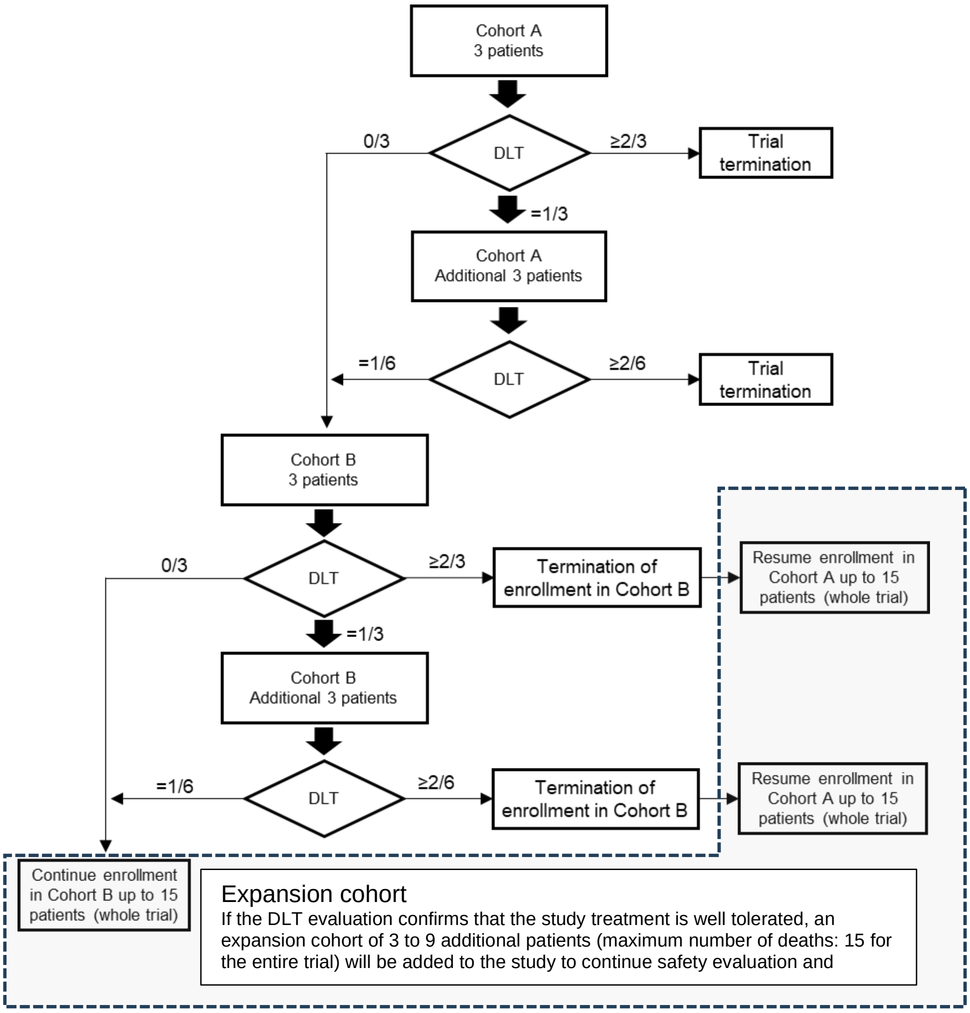

</td>
</tr>
<tr>
<td>Clinical trial period</td>
<td>Estimated study start date 30 Apr 2021
Estimated study completion date 31 Dec 2022</td>
</tr>
<tr>
<td>Subject registration period</td>
<td>Estimated date of first patient enrolled 30 Apr 2021
Estimated date of last patient enrolled 30 Jun 2022</td>
</tr>
<tr>
<td>Clnical trial sites</td>
<td>2Sites
Enrollment of patients in the trial and administration of IPs will be conducted at Chiba University Hospital, and CIRT will be administered at QST Hospital.</td>
</tr>
</table>

ETHICAL AND REGULATORY REQUIREMENTS	The study will be performed in accordance with ethical principles that have their origin in the Declaration of Helsinki and are consistent with Good Clinical Practice, and applicable regulatory requirements Patient data protection.
Institutional Review Board (IRB)	Prior to the start of the study, the Institutional Review Board (IRB) will evaluate the ethical, scientific and medical relevance of this study. The study will be conducted after obtaining approval from the IRB. If the evaluation results of the IRB are “approved with modification”, the study will be conducted after the protocol, case report, or consent forms are modified based on the review results. At the frequency of more than once a year, the IRB will continuously review whether the study is being performed appropriately.

1. INTRODUCTION

1.1. Introduction

Primary liver cancer is broadly classified into hepatocellular carcinoma (HCC) and intrahepatic cholangiocarcinoma, of which HCC accounts for more than 90% in Japan. According to a recent survey report, the number of patients with hepatocellular carcinoma in Japan is approximately 47,000 [1], and the number of deaths is approximately 27,000 per year [2].

The development of HCC is strongly associated with chronic hepatitis and cirrhosis caused mainly by hepatitis B virus (HBV) or hepatitis C virus (HCV) infection, and carriers of these viruses are considered to be at high risk of developing HCC ([3] El Serag HB, et al. NEJM. 2011). Risk factors other than viral hepatitis include cirrhosis, male age, older age, alcohol consumption, smoking, obesity, fatty liver, and diabetes mellitus [4]. Many patients are diagnosed after the disease has reached an advanced stage. The choice of treatment depends on the stage of the disease and the patient's condition. Since most patients with HCC also have chronic hepatitis or cirrhosis, individual assessment of liver function is essential to patient selection. Although several agents have been shown to be effective in the treatment of unresectable advanced HCC, there are still limited options and further therapeutic development is needed.

1.2. Standard Treatment for Advanced Hepatocellular Carcinoma

Sorafenib demonstrated a survival advantage over placebo in unresectable advanced HCC in the Sorafenib Hepatocellular Carcinoma Assessment Randomized Protocol (SHARP) trial in 2008, followed by the Asia-Pacific trial in 2009 ([5] Llovet JM, et al. Asia-Pacific trial also demonstrated a survival benefit ([5] Llovet JM, et al. NEJM. 2008, [6] Cheng AL, et al Lancet Oncol. 2009). In the SHARP trial, median survival (OS) was 10.7 months in the sorafenib group versus 7.9 months in the placebo group, with a hazard ratio (HR) of 0.69 (95% confidence interval (CI): 0.55, 0.87), P < 0.001. months vs. 4.2 months, HR 0.68 (95% CI: 0.50, 0.93), P = 0.014. The median progression-free survival in the SHARP trial was 5.5 months in the sorafenib group versus 2.8 months in the placebo group, HR 0.58 (95% CI: 0.5, 0.7), and in the Asia-Pacific trial the median progression-free survival was 2.8 months versus 1.4 months, HR 0.6 (95% CI: 0.4, 0.8).

Regorafenib after Sorafenib in Child-Pugh classification A patients with image progression after sorafenib treatment and who tolerated sorafenib (400 mg or more for at least 20 days in the 28 days before the end of treatment) compared regorafenib to placebo Patients with Hepatocellular Carcinoma (RESORCE) trial, regorafenib showed for the first time a survival advantage as second-line therapy after sorafenib treatment ([7] Bruix J, et al. Lancet. 2017). In that study, the median OS was 10.6 months (95% CI: 9.1, 12.1) in the regorafenib group and 7.8 months (95% CI: 6.3, 8.8) in the placebo group, HR 0-63 (95% CI: 0.50, 0.79) P < 0-0001. 2017, lenvatinib was non-inferiority to sorafenib for the primary endpoint of survival, making it the first-line treatment of choice for advanced hepatocellular carcinoma ([8] Kudo M, et al. Lancet. 2018). In that study, the median OS was 13.6 months (95%CI: 12.1, 14.9) in the lenvatinib arm and 12.3 months (95%CI: 10.4, 13.9) in the sorafenib arm with a HR of 0.92 (95%CI : 0.79, 1.06). In addition, ramucirumab became available in June 2019 for unresectable HCC with serum AFP levels of 400 ng/mL or higher, exacerbated after cancer chemotherapy ([9]Zhu AX, et al. Lancet Oncol. 2019).

Combination therapy with atezolizumab, an anti-PD-L1 humanized monoclonal antibody, and bevacizumab, an anti-VEGF monoclonal antibody, was shown to significantly prolong OS versus sorafenib in patients with Child-Pugh classification A who had not received systemic therapy (IMbrave 150 study) ([7 Bomze D et al. NEJM. 2020). In that study, 12-month survival rates were reported as 67.2% (95% CI: 61.3, 73.1) for atezolizumab plus bevacizumab and 54.6% (95% CI: 45.2, 64.0) for sorafenib, with an HR for OS of 0.58 (95% CI: 0.42, 0.79), P < 0 .001. Based on these results, the combination of atezolizumab and bevacizumab became available in Japan in September 2020. Based on these results, the combination of atezolizumab and

bevacizumab is now the first-line treatment for advanced hepatocellular carcinoma. Other first-line treatment options include sorafenib or lenvatinib, and second-line treatment options include regorafenib, ramucirumab, and cabozantinib. However, the survival gains obtained have been limited and do not fully meet the medical needs of patients.

1.3. HCC with vascular invasion and its treatment

HCC with vascular invasion (MVI) extending into the portal and/or hepatic veins has a poorer prognosis compared to cases without MVI ([11]Costentin CE, et al. Liver Cancer 2017). Although systemic chemotherapy is the standard of care, several studies have suggested the efficacy of alternative treatment approaches (e.g., resection, hepatic arterial chemoembolization, intravenous chemotherapy, and radiation therapy) after determining the benefit for each patient, and are recommended in Japanese guidelines. MVI Although it is empirically known that controlling MVI dramatically improves the prognosis of advanced HCC with MVI, sufficient evidence does not exist.

1.4. Immunotherapy

In recent years, research on cancer immunity has advanced dramatically, and the mechanisms by which the immune system controls or eliminates tumors are becoming clearer ([12] Dunn, et al. Annu Rev Immunol. 2004). PD-L1 is part of a complex system of receptors and ligands involved in the regulation of T cell activation. The PD-1 receptor (CD279) is expressed on the surface of activated T cells ([13] Keir ME, et al. Rev Immunol. 2008). It has two known ligands: PD-L1 (B7 H1; CD274) and PD-L2 (B7 DC; CD273) ([14] Okazaki and Honjo 2007). PD-1 and PD-L1 / PD-L2 act as co-inhibitors that can arrest or limit T cell responses When PD-L1 binds to PD-1, inhibitory signals are transmitted to T cells, cytokine production is reduced, and T cell proliferation is inhibited. Tumor cells have been shown to utilize this immune checkpoint pathway as a mechanism to evade detection and inhibit immune responses.

PD-L1 has been found to be expressed in a wide range of carcinomas, and anti-PD-L1 antibodies can be used therapeutically to augment anti-tumor immune responses in cancer patients.Results from preclinical and clinical studies of monoclonal antibodies (mAbs) targeting the PD-L1 / PD-1 pathway provide evidence of clinical activity and manageable Anti-PD-L1 antibodies can be used to augment anti-tumor immune responses in cancer patients for therapeutic purposes, showing evidence of clinical activity and manageable safety profiles ([17] Brahmer JR, et al. N Engl J Med. 2012, [18] Hirano F, et al. Cancer Res. 2005, [19] Iwai Y, et al. Proc Natl Acad Sci USA. 2002,[20] Okudaira K, et al. Int J Oncol. 2009,[21] Topalian SL, et al. N Engl J Med. 2012,[22] Zhang C,et al. Cytotherapy. 2008), The hypothesis that its efficacy is higher in patients with tumors expressing PD-L1 ([23] Powles T, et al. Nature. 2014; [24] Rizvi N, et al. J Clin Oncol. 2015; [25] Segal NH, et al. J Clin Oncol. 2015) is now supported The hypothesis of a “bladder cancer” (e.g., [24] Rizvi N et al. The high frequency and number of mutations in bladder cancer ([26] Alexandrov et al. Nature. 2013), for example, may contribute to the responses seen with immunotherapy.

In contrast, cytotoxic T lymphocyte-associated antigen 4 (CTLA-4) is structurally expressed on regulatory T cells and is enhanced on the surface of activated T cells; CTLA-4 sends negative regulatory signals to T cells when it binds to CD80 (B7.1) or CD86 (B7.2) ligands on antigen-presenting cells ([27] (Fife BT, Bluestone JA. Immnol Rev. 2008). In animal models, blockade of CTLA-4 binding to CD80 / 86 by anti-CTLA-4 antibodies has also been shown to markedly enhance T cell activation and antitumor activity, as exemplified by the killing of established solid tumors in mice and induction of protective antitumor immunity. Therefore, treatment with anti-CTLA-4 antibodies is expected to enhance activation of the human immune system and anti-tumor activity in patients with solid tumors.

Preclinical data have been added along with abundant clinical data, indicating that inhibition of negative regulatory signals to T cells, such as cytotoxic T lymphocyte antigen 4 (CTLA-4) and PD-L1, is a promising approach with promising clinical results. Ipilimumab has received U.S. Food and Drug Administration (FDA) approval for the treatment of metastatic melanoma,

and nivolumab and pembrolizumab (two anti-PD-1 agents) and atezolizumab (an anti-PD- L1 agent) are currently approved by agencies such as the U.S. Food and Drug Administration and the European Medicines Agency for the treatment of metastatic melanoma, squamous cell and non-squamous cell non-small cell lung cancer, urothelial carcinoma, and other malignancies. In addition, data from agents in the anti-PD-1 / PD-L1 class have shown clinical activity against a wide range of carcinomas.

In HCC as in other cancer types, two clinical trials (nivolumab and pembrolizumab) using ICI showed that 17-20% of patients achieved an objective response and most of the responders had a durable response ([28]El-Khoueiry AB, et al . Lancet 2017, [29]Zhu AX, et al. Lancet Oncol. 2018). The combination of ICI with tyrosine kinase inhibitors or anti-VEGF monoclonal antibodies has shown promise with the potential to achieve extremely high sustained response rates in a variety of cancer types, including HCC ([30]Kudo M. World J Gastroenterol 2019). Recently, the IMbrave 150 trial showed that atezolizumab plus bevacizumab therapy extended both OS and PFS compared with sorafenib, positioning atezolizumab plus bevacizumab as the first-line treatment for advanced hepatocellular carcinoma. Immunotherapy is also playing an increasingly important role in advanced HCC, and a phase I study of durvalumab, an anti-PD-L1 antibody, and tremelimumab, an anti-cytotoxic T lymphocyte-associated antigen 4 (CTLA-4) monoclonal antibody, in patients with HCC demonstrated a response rate of 25% ([31 was demonstrated ([31] Kelly RK et al. ASCO2017). An expanded portion of a Phase II trial is underway. A Phase III trial evaluating the efficacy and safety of the combination of durvalumab and tremelimumab, as well as monotherapy with sorafenib and durvalumab (HIMALAYA trial) are also ongoing and awaiting results.

1.5. Carbon ion radiotherapy

Particle therapy, especially carbon ion radio therapy, has advantages in dose concentration and biological effects over photon therapy, such as x-rays and gamma rays, which are usually used in radiation therapy.

X-rays and gamma rays are most intense near the body's surface and penetrate the body with decreasing intensity as they travel deeper. For this reason, the most powerful way to treat a specific area with X-rays or gamma rays is to focus the radiation from many directions, concentrating the high-dose area on the lesion. However, for tumors that are close to vital organs and spread malformedly, it is difficult to avoid surrounding normal organs. In contrast, heavy particle beams, which become intense at a certain depth depending on their energy, but are weak before and after that point, can be easily focused by aligning the peak with the tumor ([32] Pedroni E, et al. Med Phys. 1995).

The principle of action of radiotherapy is the double-strand break of DNA by ionizing radiation. Carbon ion radio therapy is known to cause dense ionization. Therefore, DNA double-strand breaks can be caused more efficiently and more densely than with X-rays. This is the reason for the favorable biological effects of heavy particle beams. This, combined with the good dose distribution resulting from the physical characteristics of heavy-ion beams, enables them to efficiently exert their anti-tumor effects. In Japan, the National Institute of Radiological Sciences (now renamed the National Institute of Quantum Science and Technology) started heavy particle therapy in 1994 and has treated more than 10,000 patients with good results ([33] Mohamad O., et al. Cancer (Basel), 2018).

In HCC, high local control rates have also been shown with particle therapy ([34] Kasuya G, et al. Cancer. 2017, [35] Fukuda, K, et al. Cancer Sci. 2017, [36] Sorin Y, et al. Liver Cancer 2018, [37] (Igaki H, et al. Int J Clin Oncol. 2018). Furthermore, good outcomes have been shown for hepatocellular carcinoma with vascular invasion. ([38] Komatsu S, et al. J Gastroenterol. 2011, [39] Lee SU, et al. Strahlenther Onkol. 2014, [40] Sugahara S, et al. Strahlenther Onkol. 2009).

1.6. Induction of Immunogenicity by Radiotherapy

Radiation therapy is known to stimulate immunogenicity through multiple mechanisms. The major immunological effects of irradiation include increased antigen presentation through

elevated expression of major histocompatibility gene complex class I, induction of apoptosis through elevated membrane expression of Fas ligands, calreticulin expression and high mobility group box-1 (HMGB1) and other (HMGB1), and induction of phagocytosis and immunity through the release of damage-associated molecular patterns (DAMPs).

Clinically, the PACIFIC trial showed that the combination of durvalumab and chemoradiotherapy can provide high therapeutic efficacy with intolerable toxicity ([41]Antonia Sj, et al. NEJM. 2017). Despite the fact that this study did not include only PD-L1-high expressing patients, it showed significant improvements in both PFS and OS compared to existing therapy. On the other hand, in the combination of durvalumab and tremelimumab (MYSTIC trial), there was no improvement from existing therapy, even when only PD-L1 high-expressing patients were targeted ([42 Rizvi NA, et al. Annals of Oncol. 2018). The difference between these two trials was the presence or absence of radiotherapy, and a subset analysis of the PACIFIC trial showed better results in patients with a shorter time between completion of radiotherapy and durvalumab administration, suggesting that radiotherapy plays an important role in tumor immunity Radiotherapy plays an important role in tumor immunity.

1.7. Combination of immune checkpoint inhibitors and carbon ion radiotherapy

Heavy ion therapy is known to enhance both local immunostimulation and immunosuppression more strongly than conventional photon beam therapy in both animal models and human clinical specimens ([43] Helm A, et al. Int J Part Ther. 2018, [44] Takahashi Y, et al. Oncotarget. 2019, [45] Iijima M, et al. J Gynecol Oncol. 2020).

Irradiated tumor cells present HMGB1, a key factor among damage-related molecules, alongside numerous others ([46] Golden EB, et al. Front Oncol, 2012). HMGB1 functions as an immune-activating cytokine and is a key factor for toll-like receptor 4 (TLR4) and activates dendritic cells.

It has been suggested that HMGB1 is strongly induced by heavy particle therapy ([47] Onishi M, et al. J Radiat Res. 2018). A study in mice showed stronger immune activation when heavy ion therapy was combined with dendritic cell infusion. The combination of heavy particle therapy and immunotherapy has also been shown to enhance anti-tumor immunity and reduce metastases compared to x-ray therapy, immunotherapy alone, or a combination of both ([48] Matsunaga A, et al. Cancer. 2010, [49] Ando K, et al. J Radiat Res. 2017).

On the other hand, immunosuppressive molecules such as PD-L1 have also been found to be induced more strongly than with X-ray therapy ([45] Iijima et al. J Gynecol Oncol. 2020). Enhanced PD-L1 expression by irradiation has been reported via the AMT/AT/Chk1 pathway induced by DNA double-strand breaks ([50] Sato N, et al. Nat Commun. 2017). Heavy ion therapy is known to efficiently generate complex DNA double-strand breaks ([51] Oike T, et al. Sci Rep. 2016), supporting this phenomenon.

Clinical trials investigating the combination of immune checkpoint inhibitors with radiation in a number of carcinomas, including HCC, are ongoing. However, most of them are limited to combinations with conventional radiation therapy using X-rays, and only a few with particle therapy. Currently, there are no clinical trials testing the combination of heavy ion beams and immunotherapy, and this clinical trial is a pilot case. From basic research and clinical studies using X-rays, the combination of heavy ion therapy and immune checkpoint inhibitors is expected to have a greater synergistic effect.

1.8. Investigational drugs

1.8.1. Durvalumab

Durvalumab is a human immunoglobulin (Ig) subclass Grade 1κ (IgGrade 1κ) mAb that inhibits the interaction of PD-L1 with PD-1 on T cells and CD80 on immune cells but not with PD-L2. Durvalumab is being developed by AstraZeneca/MedImmune for the treatment of cancer.

The mechanism of action of durvalumab is said to be inhibition of the interaction of PD-L1 with PD-1 and CD80, which disrupts the suppression of the immune response and immune

response to tumor elimination in in vitro studies, durvalumab was found to antagonize PD-L1-mediated suppression on primary human T cells, restore T cell proliferation, and release interferon gamma (IFN-γ) ([52] Stewart R, et al. Cancer Immunol Res. 2015) In vivo studies showed that durvalumab inhibited tumor growth in a xenograft model through a T cell-dependent mechanism ([52] Stewart R, et al. Cancer Immunol Res. 2015). Based on these data, durvalumab is expected to stimulate anti-tumor immune responses in patients by binding to PD-L1 and shifting the balance toward anti-tumor immune responses. Durvalumab is designed to reduce antibody-dependent and complement-dependent cellular injury.

To date, more than 8,000 patients have received durvalumab as a single agent or in combination with other anticancer agents in studies sponsored by AstraZeneca throughout the clinical development program. Please refer to the most recent Durvalumab investigational brochure for nonclinical and clinical information, including safety, efficacy, and pharmacokinetics.

1.8.2. Tremelimumab

Tremelimumab is a human immunoglobulin (Ig) Grade 2 mAb that targets CTLA-4 (CTLA-4; cluster of differentiation [CD]152), a cell surface receptor that is primarily expressed on activated T cells and blocks their activation. Tremelimumab completely inhibits the interaction of human CTLA-4 with CD80 and CD86 and increases the release of cytokines (interleukin [IL]-2 and interferon [IFN]-γ) from human T cells, peripheral blood mononuclear cells and whole blood ([53] Tarhini and Kirkwood. Expert Opin Biol Ther. 2008). Tremelimumab is being developed by AstraZeneca for the treatment of cancer.
To date, it has been administered to more than 1,500 patients as a single agent or in combination with other anticancer agents. For a summary of nonclinical and clinical information, including safety, efficacy, and pharmacokinetics, please refer to the Tremelimumab investigational brochure.

1.8.3. Durvalumab + tremelimumab combination therapy

Since there is no overlap in the mechanisms of action of CTLA-4 and PD-1, targeting both PD-1 and CTLA-4 pathways may provide additive or synergistic effects ([16] Pardoll DM, et al. Nat Rev Cancer. 2012). Therefore, in addition to investigating both drugs as monotherapy for various cancer indications, AstraZeneca is also investigating the combination of durvalumab plus tremelimumab for the treatment of cancer.

Study D4190C00006 is a late phase I dose escalation study to establish the safety, pharmacokinetics/pharmacodynamics and preliminary antitumor activity of the combination of durvalumab plus tremelimumab in patients with advanced non-small cell lung cancer (NSCLC). The dosing schedule used in this study is durvalumab administered Q2W or Q4W until Month 12, followed by 7 doses of tremelimumab at Q4W until Week 24, then 2 additional doses every 12 weeks for up to 12 months. The study is ongoing and enrollment is ongoing. Other trials investigating combination therapy for NSCLC and other tumor indications have also been initiated.

To date, 3,000 patients have received the combination in various doses and dosing regimens throughout the clinical development program. For a complete summary of non-clinical and clinical trial information, including safety, pharmacokinetics, and efficacy, please refer to the most recent Durvalumab and Tremelimumab investigational brochure.

1.8.4. Rationale for Durvalumab and Tremelimumab as Treatment Options for HCC

The liver has multiple regulatory mechanisms to maintain an immunosuppressive environment. The normal liver is inherently prone to induce immune tolerance to prevent aberrant immunity to exposed pathogens ([54]Pardee AD and Butterfield LH.Oncoimmunology. 2012). Clinical and nonclinical data indicate that HCC increases the expression of immunosuppressive cell

populations such as regulatory T cells (Treg) and myeloid-derived suppressor cells, as well as suppressive signaling molecules including CTLA-4 and PD-1 ([54] ,[55] Gao O, et al. Clin Cancer Res. 2009, [56] Hato T, et al. Hepatology. 2014); HBV and HCV infection also increase Treg and PD-L1/PD-1 expression, suggesting that this pathway is involved in HBV and HCV-mediated hepatocellular carcinogenesis ([54],[57] Miroux C, et al. Expert Opin Biol Ther. 2010,[58] Golden-Mason L, et al. J Virol. 2007,[59]Peng G, et al. Mol Immunol. 2008).

It has also been shown that overexpression of PD-L1 results in higher malignancy, disease progression, and mortality in HCC ([54], [60] Klein Jp, et al. Stat Med. 2007). Therefore, suppression of PD-L51 and CTLA-4 function could reverse the immunosuppressive nature of HCC and promote host immunity against HCC and improve clinical outcome.

At this time, early promising clinical data suggest that anti-CTLA-4 and anti-PD-L1/PD-1 antibody agents are active against HCC and may help improve response rates and survival.

In 20 patients with HCV-associated HCC (43% Child-Pugh classification B) treated with 15 mg/kg of intravenous tremelimumab every 90 days ([61] Sangro B, et al. J Hepatol. 2013), tremelimumab was generally well tolerated and no patients received systemic steroids No patients received systemic steroids and there were no deaths related to the study drug. The majority of patients had transient increases in transaminases after the first dose, 45% of which were Grade 3 or higher, but not accompanied by a concurrent decline in liver function. 17 responses could be evaluated, with 3 (17.6%) patients achieving a definite partial response (PR).

In another phase I/II study, 32 patients with unresectable advanced HCC (Child-Pugh classification A/B7) received two dose levels of tremelimumab (3.5 and 10 mg/kg IV Q4W) with partial ablation (RFA/TACE) during a 6-week treatment period ([62] Duffy AG,. et al. J Hepatol. 2017). Safety assessment results showed no clear trend in the occurrence of adverse events throughout the entire dose-specific cohort of tremelimumab, and no dose-limiting toxicities (DLTs) were observed in the study. The major Grade 3 or 4 adverse events were increased AST (21%), increased ALT (9%), and hyperbilirubinemia (9%); no Grade 3 or 4 diarrhea, colitis, or pneumonitis occurred; of the 19 patients with evaluable response outside the area directly treated with TACE/RFA, 5 (26.3%) had confirmed PR. The safety and efficacy of durvalumab (anti-PD-L1 antibody) monotherapy were investigated in a phase I/II study (CDON-MEDI4736-1108) in 40 patients with HCC, with an objective response rate (ORR) of 10.3% and a median OS of 13.2 months. Detailed safety and efficacy data from the study are described in Section 1.2.2.1. Similar results were obtained with another anti-PD-1 antibody, with an ORR of 18.6% and a median OS of 13.2 months in patients with advanced HCC ([63] Melero I, et al. J Clin Oncol. 2017).

Although the data from these trials are exploratory, they suggest that monotherapy with both durvalumab and tremelimumab has an antitumor effect against HCC. The combination of durvalumab plus tremelimumab in patients with unresectable HCC is being investigated in an ongoing phase I/II study (D4190C00022). In that study, patients with unresectable HCC who had not been previously treated with immunotherapy received the combination of durvalumab plus tremelimumab four times in Q4W followed by durvalumab alone in Q4W. Interim data from 40 patients followed for more than 16 weeks in the study showed an ORR of 18%. The combination of durvalumab plus tremelimumab was generally well tolerated in the population of patients with unresectable HCC. phase II results presented at ASCO 2020 reported promising results for durvalumab 1,500 mg Q4W plus tremelimumab 300 mg once. ORR was 24% and median OS was 18.73 months (95% CI: 10.78-27.27). It was reported to have the most favorable risk-benefit profile compared to the other dose arms ([64]Kelly RK, et al. J Clin Oncol. 2020).

The combined clinical evidence suggests that both durvalumab and tremelimumab have clinical activity as monotherapy in patients with HCC, and that combination therapy with both agents may provide an even greater antitumor effect in this patient population. A phase III, randomized, global study is underway to evaluate the safety and efficacy of these two agents in patients with HCC.

1.9. hypothesis

The purpose of this study is to confirm the synergistic effect of durvalumab-tremelimumab in

combination with heavy ion therapy in patients with advanced HCC. The study was designed based on the following two hypotheses

- Activation of tumor immunity by heavy ion therapy will be enhanced by immune checkpoint inhibitors.
- The control of primary lesions with MVI in patients with advanced HCC will have clinically significant results.

2. STUDTY OBJECTIVE

2.1. Objective

A phase Ib, open-label, uncontrolled study to evaluate the safety and efficacy of durvalumab-tremelimumab in combination with heavy ion therapy in patients with advanced hepatocellular carcinoma with MVI to assess safety and tolerability as measured by frequency of adverse events including DLT. Efficacy will be evaluated based on overall survival, 6-month survival, objective response rate, 6-month progression-free survival, and progression-free interval.

2.2. Study results regarding the appropriateness of conducting this clinical trial, efficacy, and safety for the subject disease

2.2.1. Durvalumab + tremelimumab combination therapy dose rationale

The durvalumab + tremelimumab doses and regimen selected for this study are based on the goal of selecting an optimal combination dose of durvalumab and tremelimumab that would yield sustained target suppression (sPD-L1), demonstrate promising efficacy, and have an acceptable safety profile.

2.2.2. Dose rationale for combination regimen of durvalumab 1500 mg Q4W plus tremelimumab 300 mg × 1 dose

A summary of the existing PK and pharmacodynamic data has been utilized to guide theregimen selection for the combination of durvalumab 1500 mg plus single dose of tremelimumab 300 mg.

Pharmacokinetics/Pharmacodynamics data

The supporting data for this regimen are based on PK and pharmacodyamic data from regimens that used tremelimumab doses of greater than 1 mg/kg from Study D4190C00006. An approximate dose-proportional increases in PK exposure (maximum serum concentration and area under the serum drug concentration-time curve from time 0 to Day 28 post-dose) was observed with increasing doses of tremelimumab (1, 3, and 10 mg/kg). An exploratory pharmacodynamic analysis bioanalytically evaluated the effects of tremelimumab on proliferating T-cells from NSCLC patients who received tremelimumab (1, 3, or 10 mg/kg) and durvalumab (15 or 20 mg/kg) combination treatment. Monotonic increases in pharmacodynamic activity with the combination (increased activation/ proliferation markers on CD4 and CD8 T-cells in periphery) were observed with increasing doses of tremelimumab (1, 3, 10 mg/kg). The peak increase (%) from baseline of CD4+Ki67+ T-cells was observed 8 days post administration, and the peak level was significantly increased ($p \leq 0.05$) as increasing dose of tremelimumab in the range of 1 to 10 mg/kg. Study data also suggested that higher peak exposure (maximum serum concentration [Cmax]) of tremelimumab is related to a higher maximum pharmacodynamic effect in the NSCLC patient population. Overall, the PK/pharmacodynamic data suggest that tremelimumab of dose greater than 1 mg/kg with a higher peak exposure may be associated with a higher pharmacodynamic effect.

Additionally, based on simulation data, the Cmax (78 µg/mL) post single dose administration of tremelimumab 4 mg/kg is approximately 4-fold higher than the predicted Cmax (19 µg/mL) post the first dose of tremelimumab 1 mg/kg, and is 3-fold higher than the predicted Cmax (25 µg/mL) post the fourth dose of tremelimumab 1 mg/kg in a Q4W×4 doses setting.

Clinical data

The safety and preliminary efficacy of combination of durvalumab 1500 mg plus single dose of tremelimumab 300 mg in unresectable HCC population is being evaluated in the ongoing Phase I/II study (Study D4190C00022).

In summary, a single dose of tremelimumab 4 mg/kg, while maintaining a similar overall

exposure, has a 3- to 4-fold higher Cmax compared to the 4 doses of tremelimumab 1 mg/kg. Therefore, this single administration of the higher dose of tremelimumab may have the potential for better anti-tumor activity while potentially avoiding any cumulative toxicity associated with repeated dosing of the 1 mg/kg tremelimumab. Therefore, the regimen of durvalumab 1500 mg plus tremelimumab 300 mg×1 dose is being evaluated in the current study.

Rationale for fixed dosing

A fixed-dose regimen of 1500 mg (equivalent to 20 mg/kg) of durvalumab plus 300 mg (equivalent to 4 mg/kg) of tremelimumab will be used in this study.

2.2.2.1. Rationale for utilizing a fixed-dose regimen for durvalumab and tremelimumab

A population PK model was developed for durvalumab using monotherapy data from a Phase I study (Study CD-ON-MEDI4736-1108; N=292; doses=0.1 to 10 mg/kg Q2W or 15 mg/kg Q3W; solid tumors). Similarly, a population PK model was developed for tremelimumab using data from Phase I through Phase III (N=654; doses=0.01 to 15 mg/kg Q4W or Q90D; metastatic melanoma) ([66]Wang et al 2014).

Population PK analysis indicated only minor impact of body weight on the PK of durvalumab and also tremelimumab (coefficient of ≤0.5). The weight-based versus fixed-dose (based on median weight of approximately 75 kg) regimens of both durvalumab and tremelimumab were compared using predicted PK concentrations (5th, 50th, and 95th percentiles) using a population PK model. A total of 1000 patients were simulated using weight distribution of 40 kg to 120 kg. Simulation results demonstrate that weight-based versus fixed dosing regimens of both durvalumab and tremelimumab yield similar median steady state PK concentrations with slightly less overall between-subject variability.

Similar findings have been reported by others ([22], [67] Narwal R, et al. Clin Pharmacokinet. 2013、[68] Ng CM, et al. Pharm Res. 2006, [69] Wang DD, et al. J Clin Pharmacol 2009, [70] Wolchok JD, et.al. N Engl J Med. 2013). Wang and colleagues investigated 12 monoclonal antibodies and found that fixed and body size-based dosing perform similarly, with fixed dosing being better for 7 of 12 antibodies ([21], [70] Wolchok JD, et al. N Engl J Med. 2013). In addition, they investigated 18 therapeutic proteins and peptides and showed that fixed dosing performed better for 12 of 18 in terms of reducing the between-subject variability in PK/pharmacodynamics parameters ([70] Wolchok JD, et al. N Engl J Med. 2013).

A fixed-dose approach is preferred by the prescribing community due to ease of use and reduced dosing errors. Given expectation of similar PK exposure and variability, we considered it feasible to switch to fixed-dose regimens. Based on the average body weight of 75 kg, a fixed dose of 1500 mg durvalumab (equivalent to 20 mg/kg) and a fixed dose of 300 mg tremelimumab (equivalent to 4 mg/kg) are selected for the current study. Therefore, the selected regimen of the durvalumab (+tremelimumab) cohort combined with particle therapy are:

【Cohort A】
Durvalumab monotherapy of 1500 mg Q4W

【Cohort B】
Durvalumab 1500 mg plus tremelimumab 300 mg for 1 dose, followed by durvalumab monotherapy 1500 mg Q4W

2.2.3 Rationale for carbon-ion radiotherapy

Historically, the role of radiation therapy in the treatment of liver tumors has been limited because of radiation-induced hepatic insufficiency caused by whole-liver irradiation ([71] Reed GB, et al. Am J Pathol, 1966). Particle therapy, including proton and carbon, overcame this problem with its physical dose distribution, enabling to treat large tumors while sparing normal liver tissue ([32], [72] Kanai et al. Int J Radiat Oncol Biol Phys, 1999). Results of CIRT have

been reported in multiple single institutional prospective studies and multi-institutional retrospective studies with high efficacy and mild toxicities ([34], [73] Shibuya K, et al. Liver Int. 2018, [74] Yasuda S, et al. Adv Radiat Oncol. 2019). Cases with MVI are also well treated with CIRT [35] Komatsu S, et al. J Gastroenterol. 2011). A single-arm clinical trial of CIRT in patients without large vessel or bile duct invasion is currently underway as an advanced medical treatment B, with the aim of developing a curative treatment for first-episode HCC that is not suitable for surgery (liver transplantation is not suitable) or radiofrequency ablation (jRCT1032200036).

Dose escalation studies have been conducted for 4 fraction CIRT ([34], [75]Shiba S, et al. Radiat Oncol. 2019). 60Gy (RBE) in 4 fractions appear to be well tolerated and multi-institutional retrospective study confirms its efficacy ([73] Shibuya K, et al. Liver Int. 2018). Constraints for each risk organ were determined based on previously published reports ([34], [73], [76] Shiba S, et al. Radiat Oncol 2020, [77] Makishima et al. Cancer Sci. 2018).

2.2.4 Rationale for combined treatment of carbon-ion radiotherapy and immunotherapy

In the PACIFIC trial, the combination of durvalumab and chemoradiotherapy (X-rays) has been shown to provide high therapeutic efficacy with intolerable toxicity, as discussed in 1.7 above ([41]Antonia SJ et al. NEJM. 2017). There are only ongoing trials of the combination of immune checkpoint inhibitors, including durvalumab, with particle therapy, and no reports exist as of November 2020. Trials of combinations with heavy particle therapy are similarly unregistered. As noted in 1.7 above, the results of basic research and clinical trials of combination therapy with X-rays suggest a higher synergistic effect.

2.3. Benefit-risk and ethical assessment

2.3.1. Potential benefits

2.3.1.1. Durvalumab monotherapy

Information on the potential benefit of durvalumab 1500 mg monotherapy or equivalent in patients with HCC are based on Study CD-ON-MEDI4736-1108 and are presented in Section 1.8.1. For other tumor types, see the most current durvalumab IB.

2.3.1.2. Durvalumab plus tremelimumab combination therapy

The potential benefits of adding tremelimumab to durvalumab is presented in Section 1.8.3. Information on the data supporting the selected combination regimen of durvalumab plus tremelimumab in patients with HCC are presented in Section 1.8.4. For other tumor types, see the most current durvalumab and tremelimumab IBs.

2.3.1.3. Durvalumab (+tremelimumab) combined with particle therapy

As mentioned in section 1.7, CIRT, as with traditional photon irradiation, is known to modify cancer immune reactions, but at a stronger level (Helm A, et al. Int J Part Ther. 2018, Ebner et al. Front Oncol. 2017). By combining CIRT with innunotherapy [durvalumab (+ tremelimumab)], there may be a larger improvement in efficacy compared to monotherapy of immunotherapy drugs.

2.3.2. Overall risks

Monoclonal antibodies directed against immune checkpoint proteins, such as programmed cell death ligand 1 (PD-L1) as well as those directed against programmed cell death-1 (PD-1) or cytotoxic T-lymphocyte antigen-4 (CTLA-4), aim to boost endogenous immune responses directed against tumor cells. By stimulating the immune system however, there is the potential for adverse effects on other tissues.

Most adverse drug reactions seen with the immune checkpoint inhibitor class of agents are thought to be due to the effects of inflammatory cells on specific tissues. These risks are generally

events with a potential inflammatory or immune mediated mechanism and which may require more frequent monitoring and/or unique interventions such as immunosuppressants and/or endocrine therapy. These immune mediated effects, can occur in nearly any organ system, and are most commonly seen as gastrointestinal AEs such as colitis and diarrhea, pneumonitis/interstitial lung disease (ILD), hepatic AEs such as hepatitis and liver enzyme elevations, skin events such as rash and dermatitis and endocrinopathies including hypo- and hyper-thyroidism.

2.3.2.1. Durvalumab

Risks with durvalumab include, but are not limited to, diarrhea/colitis, pneumonitis/ILD, endocrinopathies (hypo- and hyper-thyroidism, type I diabetes mellitus, hypophysitis and adrenal insufficiency) hepatitis/increases in transaminases, nephritis/increases in creatinine, pancreatitis/increases in amylase and lipase, rash/pruritus/dermatitis, myocarditis, myositis/polymyositis, other rare or less frequent inflammatory events including neurotoxicities, infusion-related reactions, hypersensitivity reactions and infections/serious infections.

For information on all identified and potential risks with durvalumab please always refer to the current version of the durvalumab IB.

In monotherapy clinical studies AEs (all grades) reported very commonly (≥ 15% of patients) are fatigue, nausea, decreased appetite, dyspnea, cough, constipation, diarrhea, vomiting, back pain, pyrexia, asthenia, anemia, arthralgia, peripheral edema, headache, rash, and pruritus. Approximately 9.4% of patients experienced an AE that resulted in permanent discontinuation of durvalumab and approximately 6.5% of patients experienced an SAE that was related to durvalumab by the study investigator.

Most treatment-related AEs were manageable with dose delays, symptomatic treatment, and in the case of events suspected to have an immune basis, the use of established treatment guidelines for immune-mediated toxicity.

A detailed summary of durvalumab monotherapy AE data can be found in the current version of the durvalumab IB.

2.3.2.2. Tremelimumab

Risks with tremelimumab monotherapy include, but are not limited to, GI effects (colitis, diarrhoea, enterocolitis and intestinal perforation), endocrine disorders (hypo and hyperthyroidism, hypophysitis and adrenal insufficiency), skin effects (rash, and pruritus), elevations in lipase and amylase and clinical manifestations of pancreatitis, other gastrointestinal events e.g. ulcerative colitis, dehydration, nausea and vomiting; hepatic events including hepatitis, and liver enzyme elevations; pneumonitis and ILD; nervous system events including encephalitis, peripheral motor and sensory neuropathies, Guillain-Barre and proximal muscle weakness; cytopenias including thrombocytopenia, anemia and neutropenia; infusion-related reactions, anaphylaxis, and allergic reactions; renal events including renal failure, acute kidney injury, nephritis, nephrotic syndrome, autoimmune nephritis and electrolyte abnormalities such as hypokalemia; autoimmune diseases including autoimmune arthritis, Sjogren's syndrome and giant cell temporal arteritis; hyperglycemia and diabetes mellitus; and pyrexia.

For information on all identified and potential risks with tremelimumab please always refer to the current version of the tremelimumab IB.

Using pooled data from monotherapy clinical studies AEs (all grades) reported very commonly (≥ 10% of patients) were diarrhea, nausea, fatigue, pruritus, decreased appetite, rash, vomiting, dyspnoea, constipation, cough, pyrexia, abdominal pain, decreased weight, headache, asthenia, and anaemia. Approximately 16% of patients experienced an AE that resulted in permanent discontinuation of tremelimumab and approximately 45% of patients experienced an SAE.

A detailed summary of tremelimumab monotherapy AE data can be found in the current version of the tremelimumab IB.

2.3.2.3. Durvalumab + tremelimumab

The safety of durvalumab + tremelimumab combination therapy was initially evaluated in the ongoing dose escalation and dose expansion Study 006, in patients with NSCLC, and is being studied in a number of other ongoing clinical trials, in a number of different indications, and has to date shown a manageable safety and tolerability profile.

The types of risks with the combination of durvalumab + tremelimumab (based on an equivalent durvalumab dose of 20m/kg and a tremelimumab dose of 1mg/kg) are similar to those for durvalumab and tremelimumab monotherapy. Emerging data from study 006, other studies evaluating the combination, and from combinations of other agents in the same class indicate an increased frequency and/or severity of some of these immune-mediated toxicities.

For information on all identified and potential risks with the durvalumab+tremelimumab combination please always refer to the current version of the durvalumab IB

In durvalumab+tremelimumab combination studies at the dose of durvalumab 20mg/kg and tremelimumab 1mg/kg AEs (all grades) reported very commonly (≥ 10% of patients) are fatigue, diarrhoea,nausea, dyspnea, decreased appetite, pruritus, vomiting, anaemia, constipation, cough, abdominal pain, pyrexia, back pain, arthralgia, hypothyroidism, asthenia, oedema peripheral, decreased weight, decreased hyponatraemia and rash.

Approximately 15% of patients experienced an AE that resulted in permanent discontinuation of study drug and approximately 16% of patients experienced an SAE that was considered to be related to durvalumab and tremelimumab by the study investigator.

A detailed summary of durvalumab + tremelimumab combination AE data can be found in the current version of the durvalumab IB.

2.3.2.4. Carbon-ion radiotherapy

Safety of CIRT for HCCs as a monotherapy are confirmed through multiple single institutional prospective studies and multi-institutional retrospective studies ([34], [73], [74]). Grade 3 or severe acute toxicities were seen in skin (1%) and ALT elevation (0.6%), with both being G3. G3 or severe late toxicities were seen in skin (G3 2%, G4 0.6%), hepatic coma (G3 1%), myositis and rib fracture (both G3 0.6%). RILD was seen in 2%. No treatment related deaths were observed.

Since CIRT is a form of radiotherapy, potential risks will be confined within the radiation field. The following may be observed.

- Bone marrow suppression
- Radiation pneumonitis, pleuritis, pleural effusion
- Nausea, vomiting, anorexia, diarrhoea, GI bleeding, ulceration, perforation, stricture
- Loss of hepatic function, hepatic failure, RILD, bile duct stricture, occlusion, cholangitis, aneurism
- Radiation dermatitis, ulceration
- Pericarditis, pericardial effusion, congestive heart failure, myocarditis, arrythmia
- Myelopathy, Peripheral neuropathy
- Rib fracture, subcutaneous induration, subsequent primary cancer, renal failure

2.3.2.5. durvalumab (+tremelimumab) combined with carbon-ion radiotherapy

Currently, there are no clinical trial results open yet for durvalumab + tremelimumab + radiotherapy for liver tumors, thus risks are unknown. Combination of immuno-oncology drugs and radiotherapy appear to have minimal excessive toxicity compared to immune-oncology drugs alone across multiple trials, including thoracic irradiation ([41] Antonia SJ, et al. NEJM 2018).

Potential risks would be toxicities closely related to immune reaction and microangiopathy, such as RILD, radiation pneumonitis and GI tract ulceration. In CIRT as a monotherapy, these toxicities are less common compared to photon radiotherapy, owing to the better dose distribution of CIRT as explained above ([34], [73], [74]). While the impact of difference in immune-modulation against toxicity between photon and carbon-ion is unknown, there is no definite evidence that excessive toxicity will be intolerable.

2.3.3. Overall benefit-risk

Durvalumab and tremelimumab have shown encouraging anti-tumor activity as single agents in advanced HCC population. The summary of this efficacy data is presented in Section 1.8.4. The combination regimen of these two agents shows a higher response rate in HCC population compared to either of the monotherapies. Thus, durvalumab plus tremelimumab combination therapy may potentially offer benefit to this patient population. Both durvalumab monotherapy and durvalumab plus tremelimumab combination therapy was tolerable in advanced HCC. The current study design aims to minimize potential risks by providing for early and intensive safety monitoring for any unexpected safety signals and for managing those risks deemed to be most likely based on prior experience with durvalumab, tremelimumab, and carbon-ion therapy. Two combination dose regimens of durvaluamb plus tremelimumab combination therapy were selected for this study with the aim to select the regimen with the most benefit for patients with advanced HCC.

HCC patients with MVI have very limited systemic therapeutic options and a poor life expectancy and health-related quality of life (HRQoL) based on the currently available treatments. While the main prognosticator in these patients is the intravascular growth of MVI, thus focal treatment by particle therapy has shown prolonged MST up to 2 years if lesions are confined in number ([40] Sugahara S, et al. Strahlether Onkol. 2009)). But those with extensive disease (ie multiple hepatic lesions and extrahepatic lesions) are currently not treated with particle therapy in fear of the out-of-treatment-field lesions. HCC patients with MVI, therefore, represents a significant unmet medical need and underlines the need for novel therapies for this patient population. CIRT combined with durvalumab or durvalumab plus tremelimumab proposed in this study may demonstrate a meaningful clinical benefit and a manageable safety profile. The overall benefit-risk profile of durvalumab (+tremelimumab) combined with CIRT is expected to be favorable, therefore supporting the current study design.

3. ELIGIBILITY

Each patient must meet all of the inclusion criteria (Section 3.2) and none of the exclusion criteria (Section 3.3) for this study. Under no circumstances will there be exceptions to this rule.

3.1. Diagnostic Criteria and Stage, Type, and Condition Classification

【CohortA & CohortB】

Eligible patients will be advanced HCC with MVI and Child-Pugh classification A over 20 years of age who are refractory or intolerant to standard systemic chemotherapy.

【Expansion cohort】

Eligible patients are advanced HCC with MVI, aged 20 years or older, with Child-Pugh classification A, with or without prior drug therapy.

3.2. Inclusion criteria

For inclusion in the study, patients should fulfill the following criteria:

1） Capable of giving signed informed consent which includes compliance with the requirements and restrictions listed in the informed consent form (ICF) and in this protocol. Written informed consent and any locally required authorization obtained from the patient/legal representative prior to performing any protocol-related procedures, including screening evaluations. For patients aged <20 years and enrolling, a written informed consent should be obtained from the patient and his or her legally acceptable representative.

2） Age >20 years at time of study entry

3） Eastern Cooperative Oncology Group (ECOG) performance status of 0 or 1

4） Body weight >30 kg

5） Adequate normal organ and marrow function as defined below:

- Haemoglobin ≧9.0 g/dL
- Absolute neutrophil count (ANC) ≧1,500/mm3
- Platelet count ≧75×109/L （≧75,000/mm3）
- Serum bilirubin ≦ULN×3.0
- AST ≦ULN×5.0
- ALT ≦ULN×5.0
- Measured creatinine clearance (CL) >40 mL/min or Calculated creatinine clearance CL>40 mL/min by the Cockcroft-Gault formula (Cockcroft and Gault 1976) or by 24-hour urine collection for determination of creatinine clearance

6） Evidence of post-menopausal status or negative urinary or serum pregnancy test for female pre-menopausal patients. Women will be considered post-menopausal if they have been amenorrheic for 12 months without an alternative medical cause. The following age-specific requirements apply:

- Women <50 years of age would be considered post-menopausal if they have been amenorrheic for 12 months or more following cessation of exogenous hormonal treatments and if they have luteinizing hormone and follicle-stimulating hormone levels in the post-menopausal range for the institution or underwent surgical sterilization (bilateral oophorectomy or hysterectomy).
- Women ≥50 years of age would be considered post-menopausal if they have been amenorrheic for 12 months or more following cessation of all exogenous hormonal treatments, had radiation-induced menopause with last menses >1 year ago, had chemotherapy-induced menopause with last menses >1 year ago, or underwent surgical sterilization (bilateral oophorectomy, bilateral salpingectomy or hysterectomy).

7） Patient is willing and able to comply with the protocol for the duration of the study including undergoing treatment and scheduled visits and examinations including follow up.

8） Advanced HCC confirmed histologically or by the typical findings of a hypervascular tumor

on computed tomography or angiography

9) (Cohort A and Cohort B only) Patients who have received at least one prior systemic chemotherapy regimen including atezolizumab bevacizumab combination, sorafenib, or lenvatinib and who are determined to be refractory or intolerant to standard therapy.
10) Must not be eligible for locoregional therapy for unresectable HCC. For patients who progressed after locoregional therapy for HCC, locoregional therapy must have been completed ≥28 days prior to the baseline scan for the current study. Acceptable locoregional therapy for HCC are Ethanol Infusion Therapy, Radio Wave ablation Therapy, Transcatheter Arterial chemoembolization (TACE), Transcatheter arterial infusion (TAI). Hepatic Arterial Infusion Chemotherapy (HAIC) is not allowed.
11) Patients who have been diagnosed with HCC showing MVI. MVI is defined as a tumor thrombus in the major hepatic and/or portal vein branches (Vp2, Vp3, Vp4, Vv2, and Vv3) identified by imaging studies.
12) Child-Pugh A
13) At least one measurable lesion other than the MVI and feeding nodule based on mRECIST.

3.3. Exclusion criteria

Patients should not enter the study if any of the following exclusion criteria are fulfilled:

1) Involvement in the planning and/or conduct of the study (applies to both sponser and/or staff at the study site)
2) Patients who have participated in another clinical trial using the investigational drug within 28 days prior to obtaining consent or who have received another investigational drug within 28 days prior to the first dose of the investigational drug in this study. The exception is if the patient is in the follow-up period of an interventional trial or is participating in an observational (non-interventional) clinical trial.
3) Any unresolved toxicity NCI CTCAE Grade ≥2 from previous anticancer therapy with the exception of alopecia, vitiligo, and the laboratory values defined in the inclusion criteria
   - Patients with Grade ≥2 neuropathy will be evaluated on a case-by-case basis after consultation with the Study Physician.
   - Patients with irreversible toxicity not reasonably expected to be exacerbated by treatment with durvalumab or tremelimumab may be included only after consultation with the Study Physician.
4) Radiotherapy treatment to more than 30% of the bone marrow or with a wide field of radiation within 4 weeks of the first dose of study drug
5) Major surgical procedure (as defined by the Investigator) within 28 days prior to the first dose of IP. Note: Local surgery of isolated lesions for palliative intent is acceptable.
6) History of allogenic organ transplantation.
7) Active or prior documented autoimmune or inflammatory disorders (including inflammatory bowel disease [e.g., colitis or Crohn's disease], diverticulitis [with the exception of diverticulosis], systemic lupus erythematosus, Sarcoidosis syndrome, or Wegener syndrome [granulomatosis with polyangiitis, Graves' disease, rheumatoid arthritis, hypophysitis, uveitis, etc.]). The following are exceptions to this criterion:
   - Patients with vitiligo or alopecia
   - Patients with hypothyroidism (e.g., following Hashimoto syndrome) stable on hormone replacement
   - Any chronic skin condition that does not require systemic therapy
   - Patients without active disease in the last 5 years may be included but only after consultation with the study physician
   - Patients with celiac disease controlled by diet alone
8) Uncontrolled intercurrent illness, including but not limited to, ongoing or active infection, symptomatic congestive heart failure, uncontrolled hypertension, unstable angina pectoris,

cardiac arrhythmia, interstitial lung disease, serious chronic gastrointestinal conditions associated with diarrhea, or psychiatric illness/social situations that would limit compliance with study requirement, substantially increase risk of incurring AEs or compromise the ability of the patient to give written informed consent

9) History of another primary malignancy except for
   - Malignancy treated with curative intent and with no known active disease ≥5 years before the first dose of IP and of low potential risk for recurrence
   - Adequately treated non-melanoma skin cancer or lentigo maligna without evidence of disease
   - Adequately treated carcinoma in situ without evidence of disease
   - However, the following cases are eligible for enrollment
   - Early stage cancer (epithelial cancer of the cervix, basal cell carcinoma, superficial bladder cancer (Tis and T1), early stage gastric cancer, and early stage colorectal cancer) that has been treated for curative purposes, has not been confirmed active for at least 3 years prior to inclusion in the study, and has a low risk of recurrence.

10) History of leptomeningeal carcinomatosis

11) History of, or current, brain metastases or spinal cord compression. Patients with suspected brain metastases at screening should have an MRI (preferred) or CT, each preferably with IV contrast of the brain prior to study entry.

12) Mean QT interval corrected for heart rate using Fridericia's formula (QTcF) ≥470 ms calculated from 3 ECGs (within 15 minutes at 5 minutes apart) Regardless of whether this criteria stays or not, all patients should have a baseline ECG

13) History of active primary immunodeficiency

14) Patients co-infected with HBV and HCV, or co-infected with HBV and hepatitis D virus (HDV). HBV positive (presence of HBsAg and/or anti-HBcAb with detectable HBV DNA); HCV positive (presence of anti-HCV antibodies); HDV positive (presence of anti-HDV antibodies), and active infection including tuberculosis (clinical evaluation that includes clinical history, physical examination and radiographic findings, and TB testing in line with local practice).

15) Current or prior use of immunosuppressive medication within 14 days before the first dose of durvalumab or tremelimumab. The following are exceptions to this criterion:
   - Intranasal, inhaled, topical steroids, or local steroid injections (e.g., intra articular injection)
   - Systemic corticosteroids at physiologic doses not to exceed 10 mg/day of prednisone or its equivalent
   - Steroids as premedication for hypersensitivity reactions (e.g., CT scan premedication)

16) Receipt of live attenuated vaccine within 30 days prior to the first dose of IP. Note: Patients, if enrolled, should not receive live vaccine whilst receiving IP and up to 30 days after the last dose of IP.

17) Female patients who are pregnant or breastfeeding or male or female patients of reproductive potential who are not willing to employ effective birth control from screening to 90 days after the last dose of durvalumab monotherapy or180 days after the last dose of durvalumab + tremelimumab combination therapy.

18) Known allergy or hypersensitivity to any of the study drugs or any of the study drug excipients.

19) Prior randomisation or treatment in a previous durvalumab and/or tremelimumab clinical study regardless of treatment arm assignment.

20) Judgment by the investigator that the patient is unsuitable to participate in the study and the patient is unlikely to comply with study procedures, restrictions and requirements.

21) Patients who have been treated with anti-PD-1, anti-PD-L1 inhibitors, or other drugs that act on other stimulatory or co-suppressive T-cell receptors and their combinations (including atezolizumab plus bevacizumab) and have failed to tolerate the same treatment.

22) Prior radiotherapy involving the liver.

23） Renal failure requiring hemodialysis or peritoneal dialysis

24） Any of the following cardiac diseases:

- NYHA Class III or IV chronic heart failure
- Current coronary artery disease or history of ischemic heart disease such as myocardial infarction within 6 months before the study
- Serious arrhythmia (grade 3 or higher according to the CTCAE ver. 5.0: arrhythmia that cannot be controlled by oral medications or requires mechanical control).

25） Poorly controlled hypertension

26） Serious and active infection, excluding hepatitis viral infection

27） Persistent proteinuria of NCI-CTCAE version 5.0 grade 3 or higher.

28） Arterial or venous thrombotic or embolic events such as cerebrovascular accident, deep vein thrombosis, or pulmonary embolism within 6 months before the start of study medication.

29） Refractory pleural effusion or ascites

30） History of hepatic encephalopathy within past 12 months

31） Oral intake impossible

32） HIV-positive

33） Pulmonary fibrosis or interstitial pneumonitis

34） Other serious complications as follows: serious mental disease or history of gastrointestinal bleeding or active hemoptysis

35） Unsatisfactory general condition for participation in the study as judged by the primary physician

4. Informed consent

4.1. Preparation and revision of informed consent form

The investigator prepares the consent form and other information documents used to obtain consent for participation in the clinical trial from the subject in plain language as much as possible. If it is considered necessary to revise the consent document and other explanatory documents, the investigator revises these documents.

The investigator submits the prepared or revised consent documents and other explanatory documents to the IRB for approval.

Amendments to the study protocol and informed consent forms will follow the below procedures:

1. When amendments are considered to be necessary, the Principal Investigator will provide to the Investigator(s) the study protocol amendment draft, informed consent form amendment drafts, and the latest version of the investigator's brochure and other necessary material/information.
2. The Principal Investigator will provide the Investigator(s) with necessary time to adequately consider the aforementioned study protocol amendment draft and material/information and discuss the details with the Principal Investigator.
3. After discussion with the Principal Investigator, the Investigator(s) will promptly submit the amended version of the study protocol or informed consent form to the head of the trial site, and receive approval of the IRB via the head of the trial site.
4. Within acceptable limits of the Principal Investigator, the same procedures will apply to amendments to be made to the study protocol and informed consent form according to instructions given by the head of the trial site based the opinions of the IRB.

4.2. Method of Obtaining Informed Consent

1) Informed consent
The investigator or subinvestigator should hand the consent document and other explanatory documents to the subject and provide sufficient explanation of the contents as indicated in "4.3". If necessary, the clinical trial coordinator also provides supplementary explanations to the subject. After confirming that the patient has a good understanding of the contents of the clinical trial, the subject's signed and dated informed consent should be obtained before the pre-study (screening) test is conducted.

2) When explaining to subjects
The investigator or sub-investigator shall give the subject the opportunity to ask questions and sufficient time to decide whether or not to participate in the trial before obtaining informed consent and shall answer the subject's questions to the subject's satisfaction.

3) Signing and delivery of consent form
The investigator or subinvestigator who provided the explanation should sign the consent form with the date of the explanation. The subject signs the consent form with the date of consent. If a collaborator provides supplementary explanation, the collaborator should also sign and enter the date of the explanation. After obtaining informed consent, a copy of the information document and the consent form shall be given to the subject.

4) Amendments to informed consent forms

When the investigator or subinvestigator revises the informed consent form or other explanatory documents due to the acquisition of new information that may be relevant to the subject's consent, the investigator or subinvestigator shall explain to the subject again using the revised informed consent form and other explanatory documents, and obtain consent in writing for the subject's continued participation in the clinical trial. If new important information is obtained that may affect the subject's consent, the information shall be immediately provided to the subject, recorded in writing, and the subject's continued participation in the clinical trial shall be confirmed.

4.3. Information to be provided to subjects

The informed consent form to be prepared by the investigator shall include the following information.

1. What is a clinical trial?
2. The purpose of the clinical trial
3. Name, title and contact information of the investigator
4. Method of the clinical trial
5. Anticipated clinical benefits and risks or inconveniences
6. Availability of other treatment options for the subject and the expected important benefits and risks associated with such treatment options
7. The expected duration of the subject's participation in the clinical trial
8. That participation in the clinical trial is of the subject's own free will and that the subject may refuse or withdraw from participation in the clinical trial at any time. Furthermore, the subject shall not be treated unfavorably due to refusal or withdrawal, and shall not lose any benefits that he/she would have received if he/she had not participated in the clinical trial.
9. Monitors, auditors, clinical trial review committees, and regulatory authorities must be able to view source documents related to medical care. In such cases, the confidentiality of the subject shall be maintained. In addition, the subject's signature on the consent document shall be considered as authorization for access.
10. Subjects' confidentiality shall be maintained even if the results of the clinical trial are made public.
11. Compensation and treatment to which subjects are entitled in the event of adverse health effects related to the clinical trial.
12. Information that may influence the subject's decision to continue participation in the clinical trial will be promptly communicated to the subject.
13. Conditions or reasons for discontinuation of participation in the clinical trial
14. Expenses to be borne by the subject in relation to the clinical trial
15. Details of any financial or other payments to be made to the subject in connection with the clinical trial (e.g., arrangements for calculating the amount to be paid)
16. The medical institution's contact person to whom subjects should refer or contact if they require further information regarding the clinical trial and their rights or if they experience any health problems related to the clinical trial.
17. Items to be observed by the subject
18. Types of investigational review committees that will investigate and deliberate on the appropriateness of the clinical trial, matters to be investigated and deliberated by each investigational review committee, and other matters related to the investigational review committee for the clinical trial in question
19. Intellectual property
20. Conflicts of interest

5. STUDY DESIGN

5.1. Overview of study design

This is a Phase Ib study to assess the safety of durvalumab combined with particle therapy (Cohort A) and durvalumab plus tremelimumab combined with particle therapy (Cohort B) in advanced hepatocellular carcinoma patients with macrovascular invasion. This study consists of four periods: the screening period, DLT assessment period, durvalumab q4W dosing period, and follow up period. After the signed informed consent is obtained and the screening is conducted, the patient will be registered for enrollment in the trial. Patients will be administered with the first IP followed by administration of CIRT.

DLT assessment period is for 42days starting from the first dose of durvalumab on Day1 of Cycle1. The first administration of continuous durvalumab q4W on Day 1 of Cycle 2 starts only after the safety of Cycle 1 was confirmed (durvalumab q4W: 28 day cycle).

DLT analysis will be made when more than one DLT was observed in each cohort.

Patients will to continue to receive durvalumab every 4 week after completion of the DLT assessment period until clinical progression/withdrawal from the trial if there may be potential clinical benefit at the investigator’s discretion.

Follow up visit will be made 28 days after study termination due to PD or withdrawal from the study. Safety information will be collected until 90 days after the last dose of study treatment or until initiation of alternative anticancer therapy. In this study, three patients are initially enrolled into cohort A. If there is no DLT observed in any of these subjects, the trial proceeds to enroll additional subjects into the cohort B, whose regimen does not contain higher dose of durvalumab but contains an additional drug of tremelimumab instead. If one subject develops a DLT at cohort A or cohort B, an additional three subjects are enrolled into that same cohort. Development of DLTs in more than 1 of 6 subjects in either cohort suggests that the regimen is not tolerable. If cohort A turns out to be intolerable, then cohort B regimen will not be pursued The evaluation of DLTs shall be performed by the investigator of Chiba University Hospital in consultation with the investigator(s). The DLTs determined shall be discussed with the Independent Data Monitoring Committee and their opinion shall be sought in accordance with the standards separately stipulated (10.7. Independent Data Monitoring Committee).

Duration of DLT assessment is defined for 42 days starting from the first administration of IP on Day1 of Cycle 1. Dose of durvalumab is fixed on 1500 mg. CIRT will be performed between Day 8 to Day 14 of Cycle 1 after the first duvalumab administration on Day1 (CIRT within 14 days after 1stcycle of durvalumab).

In both cohorts, if the investigators determined that there may be potential clinical benefit, patients will to continue to receive durvalumab every 4 week until clinical progression (Durvalumab q4W dosing period).

- **Cohort A**: Durvalumab 1500mg will be administered every 4 weeks in principle. Particle therapy, in form of CIRT, will be performed after Day8 of Cycle1 following the 1st dose of Durvalumab on Day1. 2nd cycle of durvalumab will be administrated only after the safety during DLT assessment was confirmed.

- **Cohort B**: Durvalumab 1500mg will be administered every 4 weeks in principle, and Tremelimumab 300mg will be administered only on Day1 of Cycle1. Particle therapy, in form of CIRT, will be performed after Day8 of Cycle1 following 1st cycle of Durvalumab + Tremelimumab. 2nd cycle of durvalumab will be administrated only after the safety during DLT assessment was confirmed.

CIRT will be given to both arms. Dose prescription and fractionations will be 60Gy (RBE) / 4Fr /

1week. Target lesion of the particle therapy will be focused on intrahepatic nodule with MVI. A 1cm margin will be taken as a clinical target volume margin for the feeding nodule, and 2cm margin alongside the vessel for the MVI lesion. Internal motion will be compensated according to 4D-CT movement assessment. Inter-fractional margin will be set to 3mm and combined with internal motion compensation forming a field specific planning treatment volume. Dose constraints for OARs will be prioritized over target volume coverage.

If both of Cohort A and B regimens were confirmed tolerable after DLT assessment, additional patients will be enrolled in Cohort B up to a total of 15 subjects. If only Cohort A regimen was determined to be tolerable, additional patients may be enrolled in Cohort A up to a total of 15 subjects.

Schedule for administration of investigational drugs and carbon ion radio therapy（Figure1）

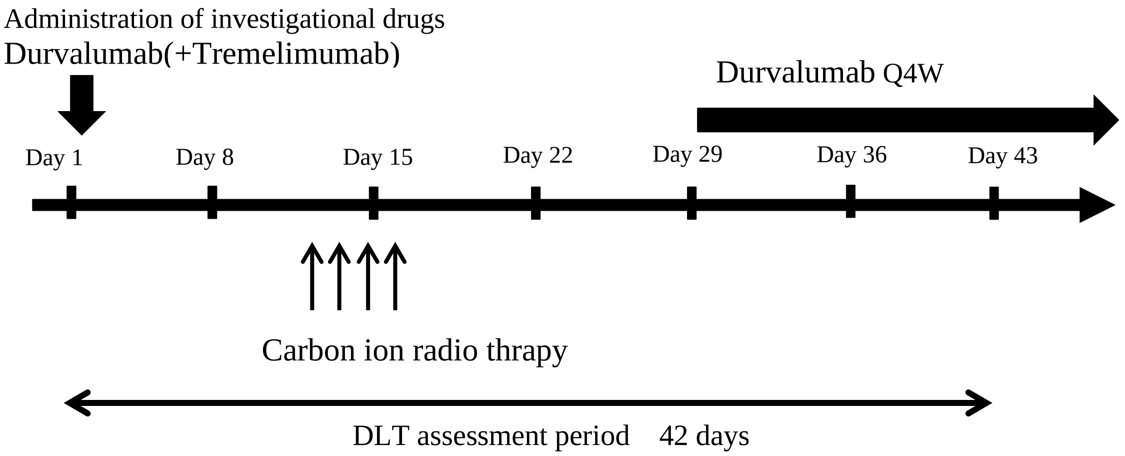

Tumor assessments, based on RECIST v.1.1, mRECIST and irRECIST, will be performed every 6 weeks (Q6W) (±1 week) for the first 12 weeks from the date of randomization and then Q8W (±1 week) thereafter until RECIST 1.1-defined radiological progression followed by a subsequent scan if clinically feasible, evaluated by Confirmation of Radiological Progression criteria (Appendix B). Patients who permanently discontinue study drug(s) for reasons other than PD should continue to have radiographic scans performed per their original schedule until confirmed PD

Subjects with rapid tumor progression or tumor-associated syndromes requiring urgent medical intervention (e.g., central nervous system metastases, respiratory failure due to tumor compression, spinal cord compression) will be deemed ineligible for continued durvalumab.

The overview of this clinical trial is as follows.

Cohort A

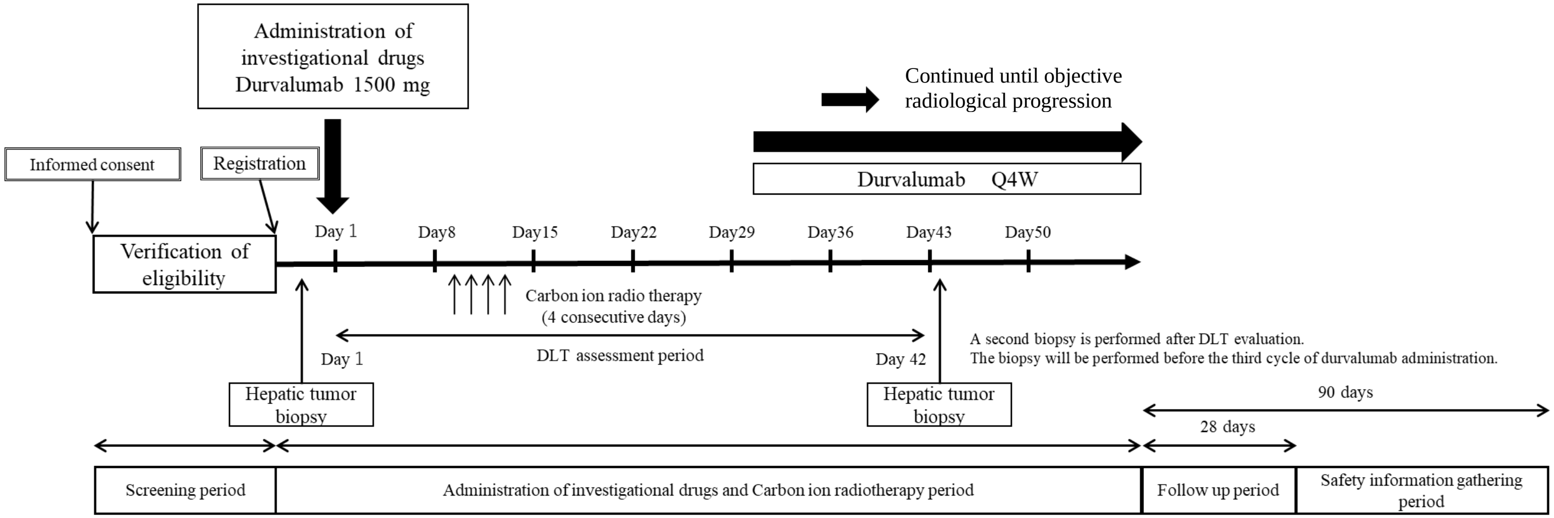

Cohort B

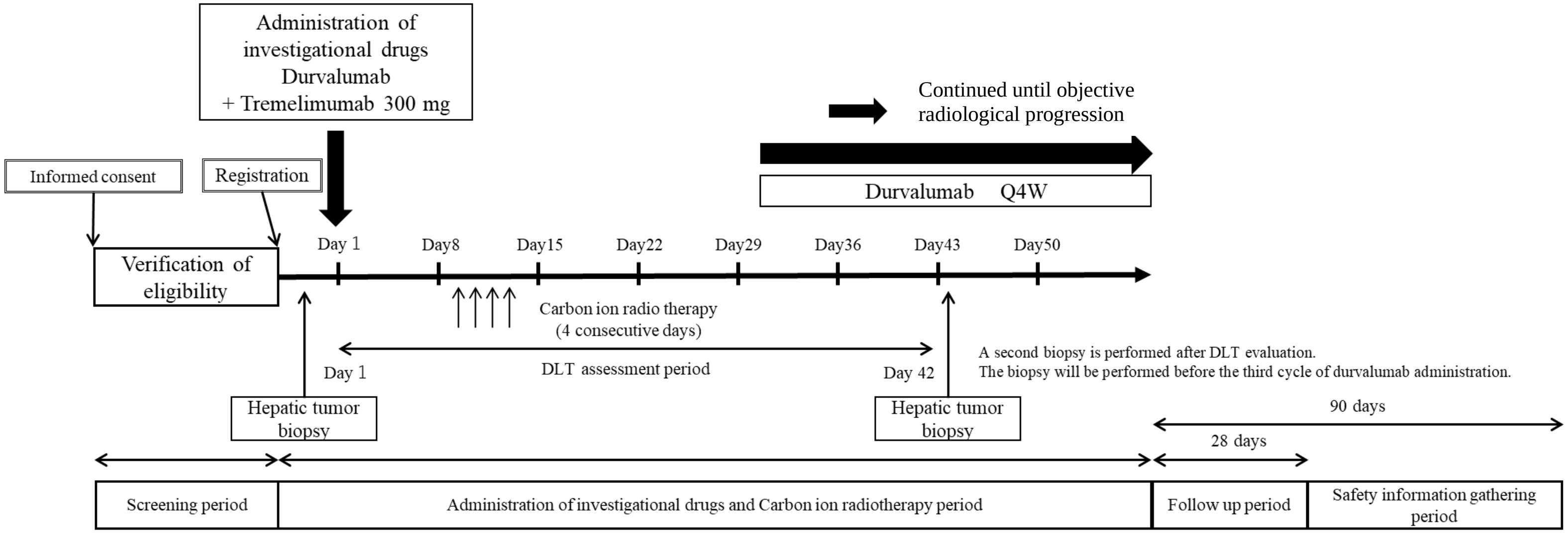

5.2. Target number of subjects and study duration

Target number of subjects：15

Study Period：
Estimated study start date 30 Apr 2021
Estimated study completion date 31 Dec 2022
Subject registration period:
Estimated date of first patient enrolled 30 Apr 2021
Estimated date of last patient enrolled 30 Jun 2022

Study schema

This study consists of four periods: the screening period, DLT assessment period, durvalumab q4W dosing period, and follow up period.

Signed informed consent will be obtained and patients will be screened prior to enrollment. The investigational treatment will include carbon ion radiotherapy on or after day 8 of cycle 1 after the first dose of study drug; the DLT evaluation period is 42 days from the first dose of durvalumab on day 1 of cycle 1. After confirmation of the safety of Cycle 1, durvalumab will be administered sequentially in Q4W beginning on Day 1 of Cycle 2. If multiple DLTs are observed in each cohort, an independent data monitoring committee will be convened to provide input on the determination of intolerability.

If no DLT is observed after the DLT evaluation period, durvalumab will be continued every 4 weeks until objective confirmation of disease progression (7.4) or until the criteria for 5.6 are met. Post-study follow-up for individual cases will occur 28 days after study completion. Adverse events and serious adverse event outcomes in subjects will be collected 90 days after the last dose of study treatment or until initiation of alternative anticancer therapy.

Figure2.

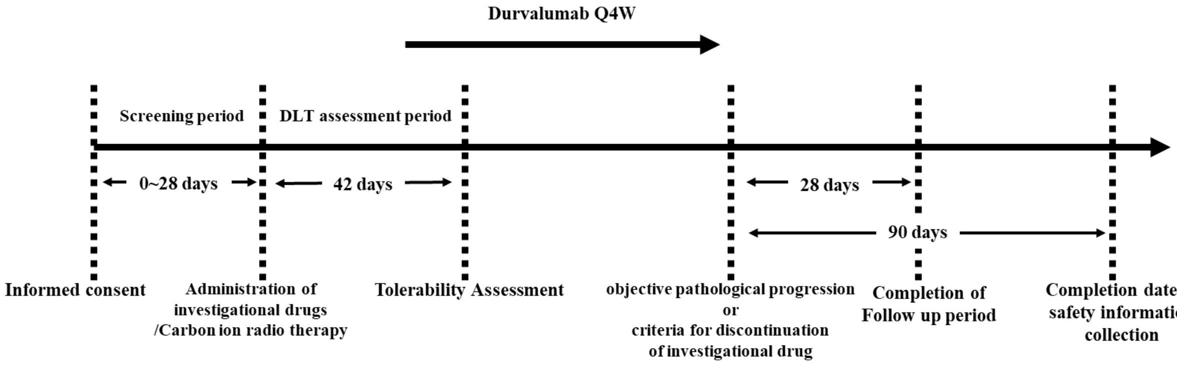

5.3. Monitoring for safety assessment

In situations where the below information is obtained and study patients are placed at under risk, or the continuation of the clinical trial is determined to be difficult, the Principal Investigator may decide on the termination or suspension of the entire clinical trial upon discussion with the Investigators. In addition, the study may be stopped based on the opinions by the DMC.

1. Occurrence of an unpredictable serious adverse reaction
2. Any information that indicates that the number, frequency, and condition of predictable serious adverse reactions cannot be predicted from the investigator’s brochure
3. SAEs that have been determined to have no causal relations with the IP, but are later determined that there is a reasonable possibility, due to the number, frequency, and condition of occurrences

4. Research reports indicating the tendency of the number, frequency, and condition of occurrences of adverse reactions having changed drastically
5. Research reports indicating the possibility that cancer, other serious diseases, disabilities, or death may occur
6. Information indicating that efficacy of the study drug cannot be expected in this clinical trial
7. Information indicating that the IP does not have any efficacy or effect on the target disease of the clinical trial
8. Information on any of the following related to marketed drugs that include the same ingredients as the IP:
    - Termination of manufacturing, import, or retail
    - Collection or disposal
    - Any other measures taken against health and hygiene related risks

Regardless of the reason for discontinuation, all data available to the subject at the time of discontinuation must be documented in the eCRF. All reasons for discontinuation should be documented. In terminating a trial, the investigator ensures that the protection of the subject's interests is given due consideration.

5.4. Institutional and case registration methods

Site registration and case registration will be conducted under the central registration system at the Data Management Office, Department of Clinical Trials, Chiba University Hospital. Once the site registration is completed, case registration will be available from the site. The following procedures will be used for site registration by fax and case registration via the Web.

5.4.1. Facility registration

1) The investigator at each participating site shall send a copy of the approval letter from the investigational review committee and a request form for site registration to the site registration center by fax after approval is obtained from the investigational review committee at the site.
2) The site registration center registers the sites and sends a notification of completion of site registration to the investigator.

Registration Center (Department of Data Management, Department of Clinical Trials, Chiba University Hospital)<br>fax number: 043-222-1207<br>Tel：043-222-1206<br>※Open hours are Monday through Friday, 9:00 a.m. to 5:00 p.m. (except Saturdays, Sundays, national holidays, and year-end and New Year holidays)<br>If a fax is received outside of the receptionist's office hours, it will be accepted on the next working day. (except at the beginning of the New Year)

5.4.2. Subject resistration

1) The investigator or subinvestigator obtains written consent and registers the subject in the case registration system. The case registration will be done via the website. After registration, a screening test is conducted to confirm that subjects meet the selection criteria and do not violate the exclusion criteria.
2) The investigator, subinvestigator or collaborator accesses the designated URL and enters the information necessary for case registration on the website. The investigator or sub investigator confirms the eligibility determination on the screen, and if the subject is

determined to be eligible, protocol treatment is initiated. Once a subject is enrolled, enrollment will not be cancelled.

∗ The investigator or sub-investigator shall not administer the investigational drug until the subject is enrolled and “eligible” by screening test.

Registration Web site（DATATRAK Enterprise Cloud）<br>URL：https://secure.datatrak.net<br>TEL：043-222-1206<br>※Open 365 days a year, 24 hours a day, including Saturdays, Sundays, and holidays (except for system downtime due to maintenance checks, etc.)

5.4.3. Handling of subjects who are found to be ineligible after enrollment

Subjects who do not meet "eligibility" for any reason, such as ineligibility, on the post-enrollment screening test are not eligible for enrollment and administration of the investigational product. The investigator or sub-investigator will explain to the subject that he/she is not eligible for enrollment in the study. The investigator or sub-investigator will also record the reason for the subject's ineligibility in the source documents.

If a subject is enrolled as a subject, but it is later determined that the subject does not meet all eligibility criteria, the subject will not be assigned and administered the investigational product and will be terminated from the clinical trial. If a subject who does not meet all eligibility criteria is inadvertently enrolled or started on the investigational product, the investigator will discuss whether or not to continue the investigational product. The investigator will ensure that all decisions resulting from this discussion are properly documented. If consensus cannot be reached, administration of the investigational product to the subject will be discontinued.

5.5. Dosing schedule and dosage/administration method

- Durvalumab 1,500 mg single agent arm (Cohort A)

  Cycle 1 Durvalumab 1,500 mg intravenous infusion will start on Day 1; after the first dose of durvalumab, heavy ion therapy will be administered on or after Day 8 of Cycle 1. Cycle 2 durvalumab will be administered after the safety of Cycle 1 is confirmed. Thereafter, dosing will be continued every 4 weeks until objective disease progression is confirmed (7.4.), “5.6. Discontinuation of Investigational Drug” is met, and the study is terminated.

- Durvalumab 1,500 mg + tremelimumab 300 mg once combination therapy (Cohort B)

  Cycle 1 One dose of combination therapy with durvalumab 1,500 mg and tremelimumab (both intravenous infusions) will be administered starting on Day 1. Durvalumab infusion will be started approximately 1 hour (maximum 2 hours) after completion of tremelimumab administration; after the first dose of durvalumab plus tremelimumab, heavy ion therapy will be administered on or after Day 8 of Cycle 1. Begin durvalumab 1,500 mg monotherapy Q4W after Cycle 2. Cycle 2 durvalumab will be administered after the safety of Cycle 1 is confirmed. Thereafter, durvalumab will be administered every 4 weeks until the objective disease progression is confirmed (7.4.), “5.6. Discontinuation of Investigational New Drug” is met, and the study is terminated.

5.5.1. Criterion for reduction

No dose reductions will be made for either durvalumab or tremelimumab in this study.

5.6. Discontinuation of Investigational Drug

If any of the following criteria are met, the investigator or sub-investigator will discontinue the investigational product and perform an Investigational Product Discontinuation Study. A follow-up visit will be scheduled 28 days after the last dose of investigational product.

1) When a subject requests to withdraw from the clinical trial treatment.
2) When it is difficult to continue administration of the investigational drug due to exacerbation of complications
3) When it is difficult to continue administration of the investigational drug due to adverse events.
4) Pregnancy is detected.
5) Other cases in which the investigator or subinvestigator judges it necessary to discontinue the administration of the investigational drug.

Subjects may discontinue investigational treatment at any time, at their own discretion, without prejudice to subsequent treatment. Subjects who decide to discontinue an investigational drug should always be questioned about the reason for discontinuation and the presence or absence of AEs. Subjects who completely discontinue subsequent doses of study medication, regardless of the reason, must continue to attend the clinic for evaluation according to the study protocol. If the subject does not agree to continue the visit, the follow-up procedure may be modified to ensure collection of endpoints and safety information. This follow-up may include telephone contact with the subject, contact with relatives or the treating physician, or information from medical records. Any change in the method of follow-up should be documented in the medical record. Subjects who agree to the change in follow-up are not considered to have withdrawn their consent or to have withdrawn from the trial.

Subjects who discontinue continued treatment with the investigational drug for any reason will be identified as treatment discontinuation. Subjects who discontinue treatment will be transferred to the follow-up period (refer to 7.1.).

Subjects who discontinue treatment for reasons other than objective tumor progression as assessed by imaging will continue to receive imaging every 6 weeks (±1 week) until 12 weeks after study drug initiation, then radiologic PD with imaging every 8 weeks (±1 week), plus additional imaging as defined in the study plan, or death (whichever occurs first), RECIST assessment will continue.

If a subject is determined to have PD as defined by RECIST 1.1, additional imaging studies should be performed within 4 weeks of the determination. (refer to 7.4.).

All subjects will be followed for survival until the end of the trial. Subjects who are unable to return for evaluation will be contacted by telephone as indicated in the trial schedule as an alternative.

5.7. Discontinuation of individual cases from participation in clinical trials

If any of the following criteria are met, the investigator or subinvestigator will discontinue administration of the investigational drug and the subject's participation in the clinical trial.

1) When it is judged difficult to continue the clinical trial for some reason on the subject's side, such as non-attendance or transfer to another hospital.
2) 2When the subject requests to discontinue participation in the clinical trial.
3) When the investigator or subinvestigator determines that the subject is unable to continue the clinical trial.
4) If the subject weighs less than 30 kg
5) When the investigator/divisional investigator determines that a decision to discontinue the study is necessary due to a serious violation of the study protocol, etc.

5.7.1. In case of untraceable cases

A subject is considered lost only if the subject cannot be contacted until the end of the trial and there is insufficient information to determine the subject's status at that time. Subjects who refuse to continue participation in the trial (including telephone contact) will be recorded as "withdrawing consent" rather than "untraceable". Investigators will document the means used to re-establish contact with subjects lost to follow-up throughout the duration of the trial. If the subject who was lost to follow-up is re-contacted, the subject will not be marked as "lost to follow-up" and the evaluation will resume according to the study protocol.

At the time of the OS analysis, the survival status of all subjects in the overall analysis population (FAS) and the safety analysis population will be reconfirmed. Subjects who withdrew consent and subjects classified as "possibly untraceable" will also be subject to this reconfirmation.

5.7.2. Withdrawal of consent

The subject is free to withdraw consent for this clinical trial at any time without prejudice to subsequent treatment.

Subjects who withdraw their consent will not receive any further doses of the investigational drug or follow-up as specified in the protocol. However, consent for survival follow-up will be confirmed separately. Additional tests may be performed after discontinuation to ensure subject safety.

If a subject withdraws consent, the investigator must confirm the reason and the presence or absence of adverse events.

The subject withdrawing consent shall be specifically asked about the following items regarding the details of the withdrawal of consent.

- Withdraw consent for all further participation in the clinical trial, including subsequent follow-up (e.g., telephone calls to investigate survival status).
- Withdraw consent for use of clinical trial data.
- Withdraw consent for the use of any sample.

5.7.3. Clinical investigator's decision

If the risks of participation in a clinical trial outweigh the benefits of the subject's participation in the trial, such as if a life-threatening infusion reaction or systemic infection occurs, the investigator will determine that the subject's participation in the trial cannot continue.

5.7.4. Subject weight loss

If the subject weighs less than 30 kg after enrollment, he/she will be removed from the study.

5.7.5. Other cases

If the investigator determines that the subject is unable to continue participation in the study for other reasons, such as a serious deviation from the protocol, the subject will be removed from the study.

5.8. Subject replacement

If a subject is found to be ineligible for the clinical trial prior to enrollment, the subject will not be enrolled in this trial. In such cases, the investigator will explain to the subject that enrollment in this trial is not possible.

For subjects who do not complete the study treatment for reasons other than DLT criteria during the DLT evaluation period, the investigator will seek input from the Data Monitoring Committee on whether to recruit a new subject to replace that subject.

5.9. Concomitant Restricted Drugs and Concomitant Restricted Therapy

The coordinating investigator must be informed of all medications taken from the time of screening until the end of the clinical phase (last visit) as soon as possible. All concomitant medications, including herbal medications taken during the trial are recorded in the CRF.
Restricted, prohibited, and permitted concomitant medications are listed in Tables 1 and 2.

Table1. 5.9. Concomitant Restricted Drugs and Concomitant Restricted Therapy

**Prohibited drug**	**Rules of use**
Anticancer drugs as investigational drugs other than the investigational drugs in this study	Concurrent use is prohibited during administration of investigational drugs.
mAb against CTLA-4, PD-1 or PD-L1 other than the investigational drug mAb for PD-L1 in this study	Concurrent use is prohibited during administration of investigational drugs.
Any concomitant chemotherapy, radiation therapy, immunotherapy, biologic therapy, or hormonal therapy for the treatment of cancer other than the investigational drug in this study	Any concomitant chemotherapy, radiation therapy, immunotherapy, biologic therapy, or hormonal therapy for the treatment of cancer other than the investigational drug in this study
Immunosuppressive agents such as systemic corticosteroids, methotrexate, azathioprine, or tumor necrosis factor alpha inhibitors at doses of prednisone or its equivalent greater than 10 mg/day<br>Immunosuppressive agents such as systemic corticosteroids, methotrexate, azathioprine, or tumor necrosis factor alpha inhibitors in doses greater than 10 mg/day. Immunosuppressive agents such as, but not limited to, the following	Concomitant administration or premedication is prohibited. The following exceptions are permitted<br>・ Use of immunosuppressive agents for the management of adverse events related to the investigational drug<br>・ Use in subjects allergic to contrast media<br>・ Use of inhaled, topical, and intranasal corticosteroids<br>・ Non-immunotherapy that is clinically necessary and has occurred in the subject<br>・ Temporary use of steroids is acceptable if deemed essential for the management of related events (e.g., chronic obstructive pulmonary disease, radiation therapy, nausea)
Epidermal Growth Factor Receptor Tyrosine Kinase Inhibitors（Epidermal Growth Factor Receptor Tyrosine Kinase Inhibitors (EGFR TKIs)	Prohibit concomitant use.<br>Use with caution for 90 days after the last dose of durvalumab. An increased incidence of pulmonary inflammation (in combination with a third-generation EGFR TKI) and an increased incidence of transaminases (in combination with a first-generation EGFR TKI) have been reported when durvalumab is used concomitantly.
Attenuated live vaccine	Concomitant use of the investigational drug is prohibited until 30 days after the last dose of the investigational drug.
Drugs with laxative action and herbs or natural remedies for constipation	Concomitant use is prohibited.
Blood transfusion (red blood cell concentrate, platelets)	Concomitant use is prohibited during the DLT evaluation period.
Granulocyte colony-forming stimulating factor (G-CSF) preparation	Concomitant use is prohibited during the DLT evaluation period.

Table2. Concomitant tolerated drugs

Tolerated Drugs	Rules of use
Concomitant medications or treatments (e.g., acetaminophen or diphenhydramine) as deemed necessary for appropriate prophylactic or symptomatic treatment. However, drugs included in the "Prohibited Drugs" section above are excluded.	Administer according to the prescription of the investigator, etc.
Best supportive care (including antimicrobials, nutritional support, correction of metabolic disturbances, optimal symptom control and pain management [e.g., palliative radiation therapy for non-target lesions])	For all subjects, use when necessary.
Inactivated viruses such as influenza vaccines	Can be inoculated (e.g. with vaccine)

5.10. Follow-up treatment

Post-treatment after completion or discontinuation of the clinical trial is not specified.

5.11. After discontinuation of this clinical trial

Subjects who discontinue the clinical trial will be subjected to necessary examination and observation, and appropriate measures will be taken as necessary, until it can be medically determined that the subject can be discharged or transferred to a hospital.

6. Clinical trial treatment

6.1. Durvalumab and tremelimumab

Refer to the investigator's brochure for details and handling of the investigational drug. The following are the investigational drugs to be used in this clinical trial.

6.1.1. Durvalumab

Durvalumab (MEDI4736) will be supplied by AstraZeneca as a 500-mg vial solution for infusion after dilution. The solution contains 50 mg/mL durvalumab, 26 mM histidine/histidine hydrochloride, 275 mM trehalose dihydrate, and 0.02% weight/volume (w/v) polysorbate 80; it has a pH of 6.0 and density of 1.054 g/mL. The nominal fill volume is 10.0 mL.

Durvalumab is a sterile, clear to opalescent, colorless to slightly yellow solution, free from visible particles.

Investigational product vials are stored at 2°C to 8°C (36°F to 46°F) and must not be frozen. Investigational products should be kept in original packaging until use to prevent prolonged light exposure.

6.1.1.1. Preparation of Durvalumab Dose by Infusion Bag

Each dose of durvalumab must be prepared by aseptic manipulation by the investigator or other investigational drug administrator designated by the site. The time between the puncture of the vial and the start of administration of Durvalumab must not exceed the following

- 2°C to 8°C for 24 hours
- 4 hours at room temperature
- Dosing solutions must be brought to room temperature before administration.

Dose 1,500 mg of durvalumab is administered with an infusion bag containing 0.9% saline or 5% dextrose to achieve a final concentration of 1 to 20 mg/mL of durvalumab and an intravenous administration set with a 0.2 μm or 0.22 μm filter. Add 1,500 mg of durvalumab (i.e., 30.0 mL of durvalumab) to the infusion bag. The infusion bag should be selected to achieve a final concentration within 1 to 20 mg/mL. Gently invert and mix the infusion bag until the administered fluid in the bag is uniform. The standard infusion time is 1 hour (±5 minutes). If the infusion is interrupted, the total infusion time should not exceed 8 hours at room temperature. No other drugs should be administered simultaneously in the same IV line.

After the contents of the infusion bag have been completely administered, flush the IV line with an IV diluent equal to the priming volume of the IV set used, or complete the infusion according to the provider's policy to ensure that the full volume is administered. If the line is not flushed, document this in the record. If the preparation time or infusion time limit is exceeded, a new vial must be used to prepare a new dose solution. Since durvalumab does not contain preservatives, unused preparation solutions must be discarded.

The preparation should be made in accordance with the “Procedures for the Administration of Investigational Drugs” for this clinical trial.

6.1.2. Tremelimumab

Tremelimumab will be supplied by AstraZeneca either as a 400-mg or a 25-mg vial solution for infusion after dilution. The solution contains 20 mg/mL tremelimumab, 20 mM histidine/histidine hydrochloride, 222 mM trehalose dihydrate, 0.27 mM disodium edetate dihydrate, and 0.02% weight/volume (w/v) polysorbate 80; it has a pH of 5.5 and density of 1.034 g/mL. The nominal fill volume is 20.0 mL for the 400-mg vial and 1.25 mL for the 25-mg vial.

Tremelimumab is a sterile, clear to opalescent, colorless to slightly yellow solution, free from or practically free from visible particles.

Investigational product vials are stored at 2°C to 8°C (36°F to 46°F) and must not be frozen.

Investigational products should be kept in original container packaging until use to prevent prolonged light exposure.

6.1.2.1. Preparation of Tremelimumab Dose by Infusion Bag

Each dose of tremelimumab must be prepared by aseptic manipulation by the investigator or the investigation drug manager designated by the site. The time from vial puncture to administration must not exceed

- 2°2°C to 8°C for 24 hours
- 4 hours at room temperature
- Doing solutions must be brought to room temperature before administration.

Tremelimumab is administered in an infusion bag containing 0.9% saline or 5% dextrose to achieve a final concentration of 0.10-10 mg/mL of tremelimumab, using an intravenous administration set with a 0.2 µm or 0.22 µm filter.

Add 300 mg of tremelimumab (i.e., 15.0 mL) to the infusion bag. Gently invert and mix the infusion bag until the dosing solution in the bag is homogeneous. The standard infusion time is 1 hour (±5 minutes); any infusion time less than 55 minutes will be considered a deviation from the study protocol. If the infusion is interrupted, the total infusion time should not exceed 8 hours at room temperature. No other drugs should be administered simultaneously in the same IV line. After the contents of the infusion bag have been completely administered, flush the IV line with an IV diluent equal to the priming volume of the IV set used, or complete the infusion according to the site's policy to ensure that the full volume is administered. If the line is not flushed, document this. If the preparation time or infusion time limit is exceeded, a new vial must be used to prepare a new dose solution. Since tremelimumab does not contain preservatives, unused preparation solutions must be discarded. The preparation should be made in accordance with the “Protocol for the Administration of Investigational Medicinal Products” for this clinical trial.

6.2. Control Drugs

There is no control drug in this study.

6.3. Monitoring during administration

During and after the infusion, the subject's condition should be monitored by assessment of vital signs at the times specified in the study protocol.

If an infusion-related reaction of grade 2 or less is observed, the infusion rate of the study drug may be reduced by 50% or discontinued until the event is resolved, and the infusion may be restarted at 50% of the initial infusion rate until the infusion is completed. Subjects who experience an infusion-related reaction of grade 2 or less may receive subsequent infusions at 50% of the initial rate. Acetaminophen and/or antihistamines (e.g., diphenhydramine) or equivalent drugs according to institutional standards may be administered at the investigator's discretion. If infusion-related reactions are grade 3 or greater, the investigational drug should be discontinued. The standard infusion duration is 1 hour, but if interrupted, the infusion should not exceed 8 hours at room temperature. Refer to the Toxicology and Management Guidelines in the protocol appendix for management of subjects who experience an infusion-related reaction.

As with other antibodies, allergic reactions to dose administration may occur. Appropriate drugs and medical devices to treat acute anaphylactic reactions must be readily available, and investigators must be trained to recognize and treat anaphylaxis. The site must have immediate access to an emergency resuscitation team and medical equipment, and the ability to admit subjects to the intensive care unit if necessary.

6.4. Management of investigational drugs

1) The investigator coordinator will deliver the investigational drug to the investigator of Chiba University Hospital in accordance with the agreement with the investigational drug

provider.

2) The investigator coordinator will properly manage the investigational drug in accordance with the protocol provided by the investigator through the site director.
3) The investigator shall prepare a document explaining the storage conditions, expiration date, and other handling methods of the investigational product and deliver it to the site manager, investigators collaborators, and investigational product manager.

6.5. Disposal of unused investigational drugs

1） After the investigational monitor checks the inventory and obtains the investigator's approval, the investigational drug manager discards used, unused, expired, or damaged investigational drugs and empty containers.
2） The investigational drug manager shall dispose of the investigational drug in accordance with the guidelines for disposal of pharmaceuticals.

6.6. Packaging and labeling of investigational drugs

The label should indicate that the product is for investigational use, the name, title and address of the coordinating investigator (representative), chemical name, volume, serial number, storage method and expiration date of the investigational drug. The label should appear on the bottle and on the package insert.

Labels for investigational new drugs shall be prepared in accordance with “Good Manufacturing Practice (GMP)” and GCP ordinances. The investigational drug label should be written in Japanese. Durvalumab and tremelimumab will be provided in a single sheet format or in a multilingual booklet format.

Investigational drug: Durvalumab (genetical recombination) (MEDI4736) + Tremelimumab (genetical recombination)

The drug name on the durvalumab label shall be “MEDI4736” or “Durvalumab (MEDI4736)” depending on the agreed upon drug name used in the approved clinical trial master label document. During this transition period, either name shall be correct.

6.7. Carbon ion radiotherapy

Information on Carbon ion radotherapy

- Name of medical device: Cardon ion radiotherapy Device
- Indications: Treatment of solid tumors
- Model Number: CI-1000S
- PMDA approval number: 22800BZX00096000
- Manufacturing facility: Toshiba Energy Systems&Solutions Corporation

Carbon Ion Therapy will be performed at the Quantum Science and Technology Agency QST Hospital, and irradiation will be performed for four consecutive days between Day 8 and Day 14 of Cycle 1 as per the schedule in Section 7.1. (If it is a holiday, it is acceptable to irradiate over the holiday.)

Dose prescription and fractionations will be 60Gy (RBE) / 4Fr / 1week for CIRT. RBE calculation is done by modified microdosimetric kinetic model (Inaniwa et al. Phys Med Biol. 2010). Pencil beam scanning technique will be used. The pencil beam covers the PTV voxel by voxel in successive layers. An optimization function drives the dose distribution in each treatment spot to reach the desired target coverage and organs at risk sparing.

All cases will undergo fiducial marker insertion prior to treatment preparation. Fiducial markers may be implanted under ultrasonography/fluoroscopy surveillance percutaneously or transarterialy.

For cases treated in an orthogonal fixed beam room, immobilization will be achieved with a relatively thick shell (3-mm thickness) made of a low-temperature thermoplastic and hydraulic urethane resin or vacuum-formed cushion to allow a range of beam angles by rotating the treatment table. However, a thinner shell may be used if a rotating gantry is available. In either case, the shell device is fixed by tapping to the table bottom, with tightening or loosening adjustment as required.

A 4D-CT simulation is required to allow for assessment of tumor motion. Fasting for 3 to 8 hours prior to simulation is required to control stomach/duodenum volume. Simulation CTs should be done with a CT slice thickness no greater than 3 mm. The 4D dataset is separated into 10 separate breathing phase bins. The simulation scan used for planning should NOT be performed with intravenous contrast; simulation CT after this planning CT may include contrast (for anatomic information).

Target lesion of the particle therapy will be focused on intrahepatic nodule with MVI. A 5mm margin will be taken as a clinical target volume margin for the feeding nodule, and 1cm margin alongside the vessel for the MVI lesion. Internal motion will be compensated according to 4D-CT movement assessment. Inter-fractional margin will be set to 3mm and combined with internal motion compensation forming a field specific planning treatment volume.

Dose constraints for risk organs are set as follows

- GI tract: D2cm3 ≤ 30Gy (RBE)
- Spinal cord: Dmax ≤ 25Gy (RBE)
- Remnant liver volume (liver volume receiving 30Gy (RBE) or less): 500cm3

In addition, the following liver volume information is collected in conjunction with the remaining liver volume

- Liver V5 Gy (RBE)
- Liver V20 Gy（RBE）

Confirmation of patient positioning is confirmed by orthogonal X-ray images. A maximum displacement of 3 mm in all directions is allowed between the reference and treatment images and is achieved by movement of the couch. Respiratory gating during treatment is mandatory. External respiratory surrogate systems or fluoroscopic tracking may be used for respiratory motion detection.

6.8. subject inclusion

In this “modified 3 + 3 design”, the first three subjects will be enrolled in cohort A. If no DLT is observed in any of these subjects, the trial will enroll additional subjects in Cohort B, who will also receive tremelimumab as follows.

If one subject develops DLT in any cohort, three additional subjects will be enrolled in that same cohort. The development of two or more DLTs in Cohort A will mean that the entire trial will be terminated, and two or more DLTs in Cohort B will indicate that the MTD has been exceeded and the regime in Cohort B will be discontinued. In that case, up to a total of 15 additional subjects will be enrolled in Cohort A.

Schematic of subject incorporation

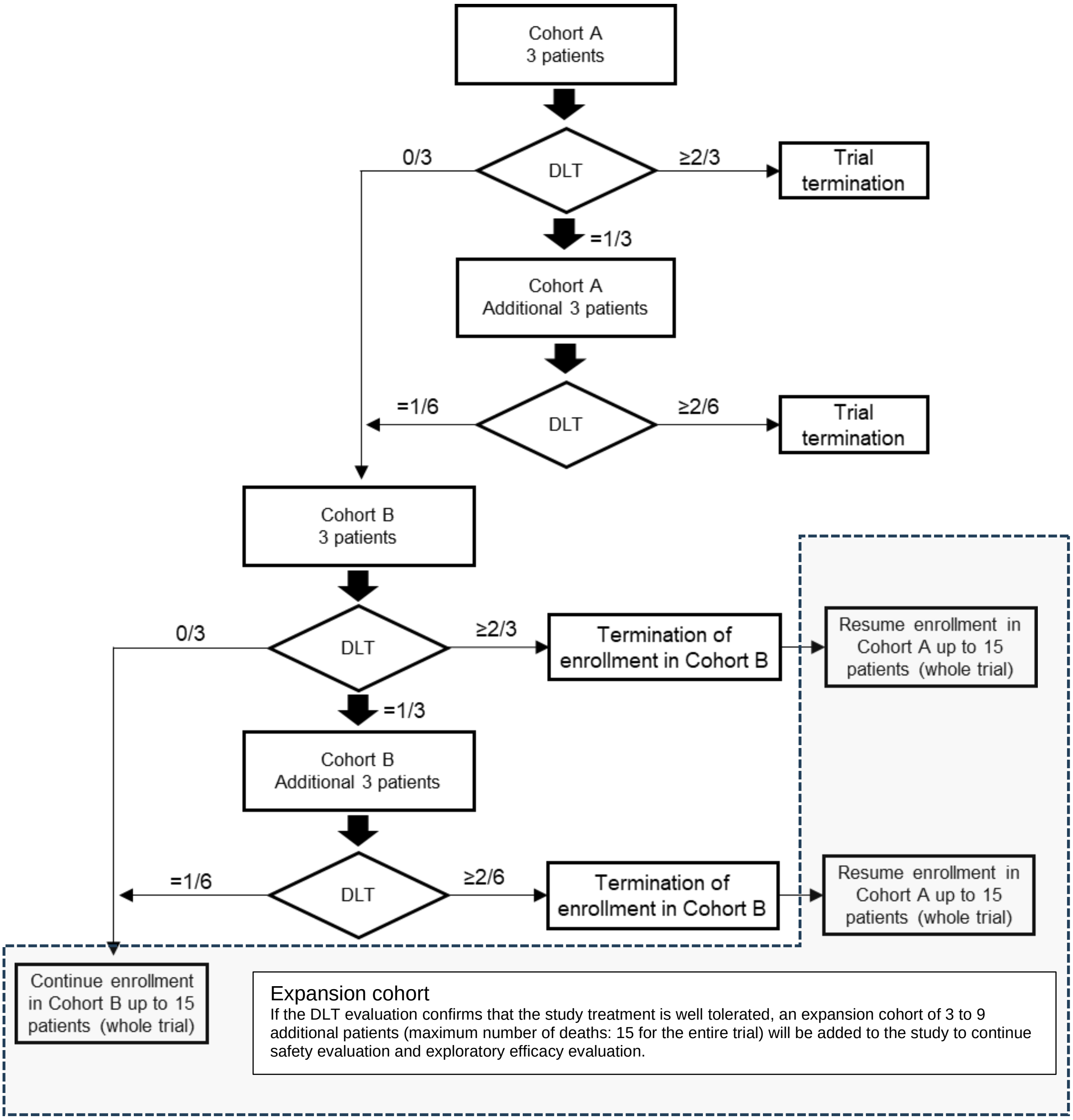

6.9. Definition of Dose-Limiting Toxicity (DLT)

During the 42-day period from the start of study drug administration (Cycle 1/DLT evaluation period), the following toxicities associated with the study treatment are considered DLTs: Grade determination follows CTCAE ver. 5.0.

Dose-limiting toxicity (DLT) will be assessed during the DLT evaluation period of this study, which will be 42 days from the time of the first dose on Cycle 1, Day 1. The severity of DLT will follow the guidelines described in the CTCAE ver. 5.0.

DLT is defined as the occurrence of an adverse event (AE) that is at least potentially related to the investigational drug or regimen (IR), with two exceptions: a Grade of vitiligo or alopecia is not a DLT. An AE that is at least potentially related to a regimen containing durvalumab and/or tremelimumab will be evaluated as a DLT if it meets one of the following criteria.

If a patient initiates heavy ion therapy but does not complete heavy ion therapy within the allowed time due to an adverse event that cannot be excluded as being causally related to durvalumab, tremelimumab, or heavy ion therapy, such adverse event will be considered a

DLT.

- **Hematologic toxicity:**
  - Grade ≥3 neutropenia complicated by fever >38.3°C
  - Grade 4 neutropenia (lasting more than 7 days)
  - Grade ≥3 thrombocytopenia with significant bleeding
  - Grade 4 thrombocytopenia (regardless of duration)
  - Grade 4 anemia (regardless of duration)
  -
- **Non-hematologic toxicity:**
  - Any Grade 4 non-immune-mediated AE
  - Any Grade 4 immune-mediated AE, excluding endocrinopathies
  - Any Grade 3 non-immune mediated AE that does not resolve to ≤Grade 1 or baseline within 30 days with optimal medical management
  - Any Grade 3 immune-mediated AE – excluding diarrhea/colitis, pneumonitis, hepatitis, rash, neurotoxicity, myocarditis, myositis/polymyositis, endocrinopathies and nephritis – that does not resolve to ≤Grade 1 or baseline within 30 days after onset of the event despite optimal medical management including systemic corticosteroids
  - Grade 3 diarrhea or colitis that does not resolve to ≤Grade 1 within 14 days [both immune- and non-immune-mediated indicated here; the same is the case if not specified in remaining bullet points below]
  - Grade 3 noninfectious pneumonitis
  - Grade 2 noninfectious pneumonitis that does not resolve to ≤Grade 1 within 3 days of the initiation of maximal supportive care
  - Aspartate aminotransferase (AST) or alanine aminotransferase (ALT) ≥3×ULN with concurrent increase in total bilirubin (TBL) ≥2×ULN without evidence of cholestasis or alternative explanations (e.g., viral hepatitis, disease progression in the liver; i.e., "Hy's Law")
  - ALT or AST >8×ULN or TBL>5×ULN
  - Grade 3 immune-mediated rash that does not resolve to ≤Grade 1 or baseline within 30 days
  - Grade 2 rash covering > 30% BSA that does not resolve to ≤Grade 1 or baseline within 30 days
  - Any grade of immune-mediated rash with bullous formation
  - Grade 3 immune-mediated neurotoxicity (excluding Guillain-Barre and myasthenia gravis) that does not resolve to ≤Grade 1 within 30 days
  - Grade 2 or 3 immune-mediated peripheral neuromotor syndrome (such as Guillain-Barre and myasthenia gravis) that does not resolve to ≤Grade 1 within 30 days or that exhibits signs of respiratory insufficiency or autonomic instability
  - Grade 3 immune-mediated myocarditis
  - Any symptomatic immune-mediated myocarditis that does not become asymptomatic within 3 days of initiating optimal medical management including systemic corticosteroids
  - Grade 2 or 3 immune-mediated myositis/polymyositis that does not resolve to Grade ≤1 within 30 days of initiating optimal medical management including systemic corticosteroids or that exhibits signs of respiratory insufficiency regardless of optimal medical management
  - Immune-mediated increase in creatinine >3×ULN, or >3×baseline for patients with a baseline creatinine elevated above ULN
  - Transfusion (Red cell concentrate, Platelet), or the use of G-CSF during DLT period

The DLT evaluation period will be from the time of the first dose of study drug/IR until 42 days post-dose. The first dose of durvalumab in Cycle 2 will be administered after the safety of Cycle 1

is confirmed.

The first treatment-related toxicity that occurs during the DLT period must be followed up to determine if the event qualifies as a DLT as defined in the DLT criteria above.

An immune-related adverse event is defined as an immune (inflammatory) adverse event without a definite other etiology. If an immune-related adverse event is suspected but there are no significant laboratory findings, a repeat laboratory test should be performed before a determination of DLT is made. Subjects who do not complete the DLT evaluation period for reasons other than DLT will not be considered DLT evaluable cases, and DLT will be evaluated after supplementation. The investigator will ask the Independent Data Monitoring Committee for an opinion on the supplementation decision.

Subjects will be administered the first dose of the study drug after hospitalization at Chiba University Hospital. After administration of the investigational drug, the subject will continue to be closely monitored in the hospital and will be transferred to the QST Hospital only for heavy ion therapy, taking into consideration the subject's condition and the means of transportation (taxi, private ambulance, etc.). After completion of heavy particle therapy, the patient should remain in the hospital for at least 7 days. During the transition to outpatient treatment, the investigator will perform the tests specified in 7.2.2 and confirm the safety of the treatment before allowing the patient to continue treatment as an outpatient. In addition, the investigator should be able to contact the site and the medical facility near the home in the event of an adverse event and establish a system to promptly contact the subject in an emergency.

6.10. Toxicity Management

Guidelines for the management of immune-mediated reactions, infusion reactions, and non-immune-mediated reactions to durvalumab are provided in the Durvalumab/Tremelimumab Toxicity Management Guidelines (TMGs).

Appropriate efforts should be made to thoroughly evaluate the subject and rule out neoplastic, infectious, metabolic, toxic, or other etiologies of imAE. Serologic, immunologic, and histologic (biopsy) data should be used to support the diagnosis of imAE, if appropriate. In the absence of a clear alternative etiology, the possibility of an immune-related etiology should be considered. In addition, there are situations in which durvalumab and tremelimumab should be discontinued (see Toxicity Management Guidelines). Dose reductions are not permitted. In case of doubt, consult the investigator. All toxicities will be evaluated according to CTCAE ver. 5.0.

6.11. Restrictions during the clinical trial

6.11.1. Restrictions during the clinical trial

The following restrictions apply during and before and after a given period of time while undergoing investigational treatment.

About Women of Childbearing Potential

Female subjects of childbearing potential who are not abstinent and who plan to have sex with an unprotected male partner must use at least one effective method of contraception from the time of screening until the drug treatment and drug discontinuation period (180 days after the last dose of durvalumab + tremelimumab combination therapy). Unprotected male partners of female subjects of childbearing potential must use a male condom and spermicide during this period. Discussion of stopping contraception after this time should be discussed with the family physician. Temporary abstinence, the rhythm method, and external ejaculation are not acceptable methods of contraception. Females should also not breast-feed during this period.

About men with female partners of childbearing potential

For non-contraceptive male subjects who are unprotected and plan to have sex with a female partner of childbearing potential, the use of a male condom plus spermicide is mandatory from

screening through the entire drug treatment and drug washout period (180 days after the last dose of durvalumab + tremelimumab combination therapy). However, periodic abstinence, rhythm and withdrawal methods are not acceptable methods of contraception. Male subjects will refrain from donating sperm during this period.

Female partners of male subjects (of childbearing potential) should also use highly effective contraceptive methods during this period.

Note: Pregnant women are defined as women who have not undergone sterilization (i.e., bilateral oophorectomy, bilateral oophorectomy, or total hysterectomy) or as premenopausal.

A woman is considered postmenopausal if she has been amenorrheic for 12 months without another medical cause. The following age-specific requirements apply

- Women under age 50 are considered postmenopausal if they have been amenorrheic for at least 12 months after discontinuation of exogenous hormone therapy and have luteinizing hormone and follicle stimulating hormone levels in the postmenopausal range of the institution.
- Women over age 50 are considered postmenopausal if they have been amenorrheic for at least 12 months after discontinuation of all exogenous hormone therapy, if they had radiation-induced menopause more than 1 year before their last menstrual period, and if they had chemotherapy-induced menopause more than 1 year before their last menstrual period.

A highly effective contraceptive method is defined as having a low failure rate (i.e., less than 1% per year) when used consistently and correctly.

6.12. Clinical Trial Procedures

The RECIST evaluation date must be performed as scheduled, regardless of any dosing delays. All other scheduled assessments must be performed at the start of the dosing cycle, and all laboratory and other tests required for dosing must be performed at least 3 days prior to dosing.

Subjects may be allowed to delay dosing under certain circumstances, as described below.

- Dosing may be delayed for either immune- or non-immune-related AEs in accordance with toxicity management guidelines.
- If dosing must be delayed for reasons other than treatment-related toxicities, dosing should be resumed as soon as possible.
- The dosing interval may be shortened as clinically appropriate to gradually match the treatment cycle to the tumor response plan (RECIST). Based on the half-life of durvalumab and tremelimumab, the interval between two consecutive doses should not be less than 22 days (for durvalumab and tremelimumab, see the current investigator's brochure).

7. OBSERVATION, EXAMINATION, AND ASSESSMENT, METHODS, AND TIMING OF IMPLEMENTATION

7.1. Implementation Schedule and Procedures

Cycle	Screening period	First tumor biopsy ※only in consented patients	DLT evaluation period							Second tumor biopsy ※only in consented patients	Durvalumab q4W dosing period					ST	Follow up period (28 days after the last administration date)	Safety information collection (90 days after the last administration date)
			Cycle 1				Cycle 2 [*13]				Cycle 3	Cycle43	Cycle 5	Cycle 6	Cycle 6< until PD			
Cycle Day	D -28 ～D -1	D -28〜D -1	1	8 to 14	15	22	1	8	14	After DLT evaluation	1[*1]	1	1	1	1	—	—	—
Allowable period (Day)			—	±3	±3	±3	±3	±3	±3		—	±3	±3	±3	±3	+14	+14	+14
Informed Consent ／ Subject backgroud information／ Review of eligibility	●[*2]																	
Durvalumab administration (cohort A and B)			●				●				●	●	●	●	●			
Tremelimumab administration (cohort B)[*3]			●															
Tumor biopsy		●								●								
CIRT				●●●●														
Fiducial marker insertion[*4]		●																
Fixation, simulation CT (for CIRT)[*4]	●																	
Weight*5	●		●	●	●	●	●	●	●		●	●	●	●	●	●	●	
Height	●																	
Physical exam	●		●	●	●	●	●	●	●		●	●	●	●	●	●	●	
Physical exam (Specific site based on case)	●		●	●	●	●	●	●	●		●	●	●	●	●	●	●	
Vital signs	●		●	●	●	●	●	●	●		●	●	●	●	●	●	●	
ECOG PS	●		●	●	●	●	●	●	●		●	●	●	●	●	●	●	
Clinical Chemistry／Hematology[*6]	●		●	●	●	●	●	●	●		●	●	●	●	●	●	●	
Coagulation	●		●	●	●	●	●	●	●		●	●	●	●	●	●	●	
Urinalysis	●		●								●	●	●	●	●	●	●	
ECG[*7]	●		●													●		
Hepatitis serology [*8]	●											(●)	(●)	(●)	(●)	(●)		
HIVtests	●																	
TSH、fT3、fT4 [*9]	●		●	●	●	●	●	●	●		●	●	●	●	●	●	●	
Pregnancy test [*10]	●																	
Chest X ray	●															●		
Assesment of Child-Pugh score	●		●				●				●	●	●	●	●	●	●	
CT/MRI[*11]	●		Every 6 weeks (±1 week) for the first 12 weeks from Cycle1 day1, and every 8 weeks (±1 week) thereafter until PD.															
Tumor marker (AFP、PIVKA-II)	●						●				●	●	●	●	●			
Cocmitant medication	◄──	────	────	────	────	────	────	────	────	────	────	────	────	────	────	────	──►	
Assessment of AE/SAE[*12]	◄──	────	────	────	────	────	────	────	────	────	────	────	────	────	────	────	────	──►

*1. The baseline for the durvalumab Q4W dosing period will be the first day of the third cycle.
*2. Whenever possible, minimize the time between enrollment and initiation of therapy.
*3. In combination therapy, tremelimumab should be administered first, and durvalumab infusion should be started approximately 1 hour (maximum 2 hours) after tremelimumab administration is completed.
*4. To be administered to subjects who are eligible by other screening tests.
*5. Weight will be measured along with vital signs at the visit.
*6. Serum or plasma biochemical tests (including LFT monitoring) and blood tests may be performed more frequently if clinically indicated.
If screening biochemical and hematological evaluations were performed no more than 3 days prior to Day 1 (the first infusion day), they need not be performed again on Day 1.
Results of LFTs, electrolytes, complete blood count, and creatinine must be obtained prior to the start of infusion (within 3 days) and confirmed by the attending physician or investigator prior to administration.
*7. Three ECG results are required if clinically significant abnormalities are detected.
*8. If HBs-Ag, HBs-Ab, or HBc-Ab is positive, HBV-DNA should be measured (before and every 4 weeks after administration).
If HCV antibodies are positive, measure HCV-RNA (pre-test).
*9. Free T3 or free T4 should be measured only if TSH is abnormal or endocrine system-related AEs are clinically suspected. If TSH is measured up to 14 days prior to the first dose, it need not be measured again on Day 1.
*10. For women of childbearing potential only. For women of childbearing potential, a pregnancy test should be performed every 4 weeks starting 7 days prior to the first dose. Pregnancy tests can be performed on Day 1, but results must be confirmed by the treating physician or investigator before administration is initiated.
*11. RECIST evaluation is performed with CT (preferred) or MRI imaging of the chest, abdomen (including liver and adrenal glands), and pelvis IV contrast preferred. Imaging of the pelvis is recommended only if there is a possibility of primary or metastatic disease in the pelvic region. Additional anatomic imaging should be performed based on the individual subject's signs and symptoms at baseline and at follow-up. Baseline assessments should be performed no later than 28 days prior to the start of study drug administration for each cohort and should be performed as close as possible to the start of study drug or prior to the start of study drug if possible. Confirmatory testing should be performed within 4 weeks of the prior PD evaluation, preferably at the next scheduled imaging visit (provided there is no clinically significant deterioration). If an unscheduled evaluation is performed and the subject has not progressed, every effort should be made to perform a subsequent evaluation at the next scheduled visit.
*12. AEs and SAEs should be collected from the time of the first dose of study drug until the follow-up date 28 days after the last dose of study drug. However, AE and SAE outcomes should be collected until the end of the safety information collection period (90 days after the last dose of study drug) or until the start of alternative anticancer therapy. In addition, events occurring after the 28-day follow-up period after the last dose of the study drug and considered to be attributable to delayed toxicity to the study drug will be collected as AEs or SAEs until the end of the safety information collection period or until the initiation of alternative anticancer therapy.
*13. *n the Expansion cohort, patients will be moved to Cycle 2 after the end of Cycle 1.

Note: All assessments on the treatment day shall be performed prior to infusion unless otherwise indicated.
ECG Electrocardiogram; LFT Liver function tests; T3 Triiodothyronine; T4 Thyroxine; TSH Thyroid stimulating hormone.

7.2. Observation, tests and assessment

7.2.1. Screening period

The investigator or sub-investigator will perform the following screening tests and enroll subjects who meet the selection criteria and do not violate the exclusion criteria. The tests will be performed after consent is obtained and between 28 days before the start of the investigational drug and the day before the start of the investigational drug. Test items will be as described below. However, test results obtained prior to consent as part of routine medical care using procedures similar to those used in this study may be used as screening tests if they fall within the 28-day screening period.

- Informed Consent
- Subject background information*
- Review of eligibility criteria
- Complete physical exam
- ECOG Performance Status
- Vital signs**, weight and height
- Chest X ray
- 12-lead ECG (in triplicate [2-5 minutes apart])
- Assessment of Child-Pugh score
- Imaging by CT/MRI, if applicable to study
- Clinical laboratory tests for:
    - Clinical Chemistry (see Table 5)
    - Hematology (see Table 4)
    - TSH, fT3, fT4
    - Coagulation (PT, PTT, INR)
    - Creatinine Clearance
    - Pregnancy test (for women of childbearing potential only)
    - Hepatitis serology
    - HIV test
    - Urinalysis
    - Tumor marker (AFP, PIVKA-II)
- Fiducial marker insertion
- Fixation, simulation CT (for CIRT)
- Concomitant medication

* **Subject background information and medical history**

Subject identification code number, race, sex, age at obtaining IC, medical history, concurrent diseases, alcohol consumption, smoking history, and information for HCC with initial diagnosis, pathological diagnosis and past treatment.

** **Vital signs**

Vital signs will be measured at every visit and will include assessments of systolic and diastolic BP, temperature, and HR. Systolic and diastolic BPs will be documented in mmHg. Temperature will be obtained in degrees Celsius. HR will be documented in beats per minute. Generally, each patient will have blood pressure tested in the same arm. Measurement device and measurement time will not be indicated.

7.2.2. DLT assessment period

Day1

- Durvalumab administration (cohort A and B)
- Tremelimumab administration (cohort B）
- Complete physical exam
- ECOG Performance Status
- Vitals signs and weight
- 12-lead ECG (in triplicate [2-5 minutes apart])
- Assessment of Child-Pugh score
- Clinical laboratory tests for:
  - Clinical chemistry
  - Hematology
  - TSH、fT3、fT4
  - Coagulation（PT、APTT、PT-INR）
  - Creatinine Clearence
  - Pregnancy test (women of childbearing potential only)
  - Urinalysis
- Assessment of AE/SAE
- Confirmation of concomitant therapy

Day 8-14

- CIRT (60 Gy (RBE) / 4 Fr) (performed at QST hospital)
- Complete physical exam
- ECOG Performance Status
- Vitals signs and weight
- Assessment of Child-Pugh score
- Clinical laboratory tests for:
  - Clinical chemistry
  - Hematology
  - TSH、fT3、fT4
  - Coagulation（PT、APTT、PT-INR）
  - Creatinine Clearence
- Assessment of AE/SAE
- Confirmation of concomitant therapy

Day 15

- Complete physical exam
- ECOG Performance Status
- Vitals signs and weight
- Assessment of Child-Pugh score
- Clinical laboratory tests for:
  - Clinical chemistry
  - Hematology
  - TSH、fT3、fT4
  - Coagulation（PT、APTT、PT-INR）
  - Creatinine Clearence

- Assessment of AE/SAE
- Confirmation of concomitant therapy

Day 22

- Complete physical exam
- ECOG Performance Status
- Vitals signs and weight
- Assessment of Child-Pugh score
- Clinical laboratory tests for:
  - Clinical chemistry
  - Hematology
  - TSH、fT3、fT4
  - Coagulation（PT、APTT、PT-INR）
  - Creatinine Clearence
- Assessment of AE/SAE
- Confirmation of concomitant therapy

Day 29（Cycle 2 Day1）

- Durvalumab administration (cohort A and B)
- Tremelimumab administration (cohort B）
- Complete physical exam
- ECOG Performance Status
- Vitals signs and weight
- 12-lead ECG (in triplicate [2-5 minutes apart])
- Assessment of Child-Pugh score
- Clinical laboratory tests for:
  - Clinical chemistry
  - Hematology
  - TSH、fT3、fT4
  - Coagulation（PT、APTT、PT-INR）
  - Creatinine Clearence
  - Pregnancy test (women of childbearing potential only)
  - Tumor maker (AFP, PIVKA-II)
- Assessment of AE/SAE
- Confirmation of concomitant therapy

Day 36（Cycle2 Day8）

- Complete physical exam
- ECOG Performance Status
- Vitals signs and weight
- Assessment of Child-Pugh score
- Clinical laboratory tests for:
  - Clinical chemistry
  - Hematology
  - TSH、fT3、fT4
  - Coagulation（PT、APTT、PT-INR）

- Creatinine Clearence

- Assessment of AE/SAE
- Confirmation of concomitant therapy

Day 43（Cycle 2 Day14）

- Complete physical exam
- ECOG Performance Status
- Vitals signs and weight
- Assessment of Child-Pugh score
- Clinical laboratory tests for:
    - Clinical chemistry
    - Hematology
    - TSH、fT3、fT4
    - Coagulation（PT、APTT、PT-INR）
    - Creatinine Clearence
- Assessment of AE/SAE
- Confirmation of concomitant therapy

At discharge during the DLT evaluation period

- Complete physical exam
- ECOG Performance Status
- Vitals signs and weight
- Assessment of Child-Pugh score
- Clinical laboratory tests for:
    - Clinical chemistry
    - Hematology
    - TSH、fT3、fT4
    - Coagulation（PT、APTT、PT-INR）
    - Creatinine Clearence
- Assessment of AE/SAE
- Confirmation of concomitant therapy

7.2.3. Durvalumab q4W dosing period

At Each cycle Day1

- Durvalumab administration (cohort A and B)
- Complete physical exam
- ECOG Performance Status
- Vitals signs and weight
- 12-lead ECG (in triplicate [2-5 minutes apart])
- Assessment of Child-Pugh score
- Clinical laboratory tests for:
    - Clinical chemistry
    - Hematology
    - TSH、fT3、fT4
    - Coagulation（PT、APTT、PT-INR）
    - Creatinine Clearence
    - Pregnancy test (women of childbearing potential only)

- Tumor maker (AFP, PIVKA-II)
- Assessment of AE/SAE
- Confirmation of concomitant therapy

7.2.4. At the time of discontinuation of investigational drug administration

- Complete physical exam
- ECOG Performance Status
- Vitals signs and weight
- 12-lead ECG (in triplicate [2-5 minutes apart])
- Assessment of Child-Pugh score
- Clinical laboratory tests for:
  - Clinical chemistry
  - Hematology
  - TSH、fT3、fT4
  - Coagulation（PT、APTT、PT-INR）
  - Creatinine Clearence
  - Tumor maker (AFP, PIVKA-II)
- Assessment of AE/SAE
- Confirmation of concomitant therapy

7.2.5. Follow up period

- Complete physical exam
- ECOG Performance Status
- Vitals signs and weight
- 12-lead ECG (in triplicate [2-5 minutes apart])
- Assessment of Child-Pugh score
- Clinical laboratory tests for:
  - Clinical chemistry
  - Hematology
  - TSH、fT3、fT4
  - Coagulation（PT、APTT、PT-INR）
  - Creatinine Clearence
  - Urinalysis
  - Tumor maker (AFP, PIVKA-II)
- confirmation of survival
- Assessment of AE/SAE
- Confirmation of concomitant therapy

7.3. Biological sampling procedures

7.3.1. Guideline for blood sampling volume

The total volume of blood to be drawn from each subject in this study is as follows

Amount of blood to be collected from each subject

Assesement		Sample volume (mL) / visit
**Safety**	**Clinical Chemistry Tests**	10

Amount of blood to be collected from each subject

Assesment	Sample volume (mL) / visit
**Hematology Tests**	10
**Total**	20

7.3.2. Blood samples for archiving

When consent is obtained from the subject for storage of blood specimens, residual blood specimens designated for biochemistry and blood tests will be stored at the Department of Gastroenterology, Chiba University Hospital. Blood specimens will be handled in such a way as to prevent leakage, confusion, theft, or loss of personal information by anonymization with an identification code. Blood specimens will be stored for a period not exceeding 20 years after the completion of the clinical trial, after which all specimens will be properly disposed of. If a subject withdraws consent for specimen storage, the specimens will be destroyed and this will be documented. In addition, if specimens are to be used in future research, a new research protocol must document the use of this specimen and be submitted to the IRB for approval.

7.3.3. Hepatic tumor biopsy sample

In this study, a percutaneous liver biopsy/liver tumor biopsy will be performed before the first dose and between 43 and 56 days after the first dose, if the subject is deemed safe for such a biopsy/liver tumor biopsy by the investigator or sub-investigator and if the subject consents. The tissue samples (tumor and non-tumor) obtained will be used for exploratory studies (refer to 9.3.). At that time, the tissue samples will be handled to prevent disclosure, mix-up, theft, or loss of personal information by anonymization with an identification code. The investigator or sub-investigator may discontinue the percutaneous liver biopsy or liver tumor biopsy for the safety of the subject. Failure to perform a percutaneous liver biopsy/liver tumor biopsy at the discretion of the investigator or sub-investigator, or failure to perform a percutaneous liver biopsy/liver tumor biopsy without the consent of the subject, will not preclude enrollment or dosing in this study. Tissue specimens will be retained for a maximum of 20 years after completion of the study, after which all specimens will be properly disposed of. If a subject withdraws consent for specimen storage, the specimen will be discarded and this will be documented. In addition, if specimens are to be used in future research, the use of that specimen must be documented in a new research protocol and submitted to the IRB for approval.

7.4. Asesment of efficiency

The following are guidelines for confirming image evaluations to assess efficacy.

- Imaging evaluations are performed for subject management and treatment decisions.
- Image evaluation is performed using RECIST ver. 1.1.
- In the absence of clinically evident evidence of disease progression, the patient should be re-evaluated after disease progression (PD) is determined by RECSIST ver. 1.1 in order to distinguish between immune checkpoint inhibitor-induced pseudo progression and true disease progression (this is to reduce the risk of study termination due to incorrect evaluation by the investigator/participating physician). (This is to reduce the risk of termination of the study due to incorrect assessment by the investigator/associated investigator.)

The definition of objective disease progression (definite PD) is as follows

1) The presence of clinically evident evidence of disease progression and disease progression (PD) according to RECIST ver. 1.1 is confirmed as objective disease progression.
2) In the absence of clinically evident findings of disease progression (PD) by RECIST ver. 1.1, a second imaging study to evaluate PD according to the specific criteria below should be

performed after disease progression (PD). The partially modified RECIST ver. 1.1 used for objective confirmation of progression is used only to confirm objective disease progression (confirmed PD). Imaging evaluations to determine objective disease progression should be performed within 4 weeks of the first imaging evaluation that determined PD using RECIST ver. 1.1.

The following is RECIST ver. 1.1 modified to establish objective disease progression.

- On two consecutive image evaluations, the sum of the diameters of the target lesions (TL) increases by more than 20% compared to the sum of the smallest diameters and the sum of the diameters increases by more than 5 mm compared to the sum of the smallest diameters.
- Non-target lesions (NTL) and/or pre-existing new lesions showed significant progression (worsening) at the time of the confirmatory examination compared to the most recent imaging evaluation (note: new lesions at the time of imaging evaluation that are determined for the first time to be PD by RECIST ver. 1.1 are evaluated as NTL at the second imaging evaluation).
- The appearance of an obvious new lesion that was not present at the first imaging evaluation that determined PD according to RECIST ver. 1.1 but was present at the second imaging evaluation.

Two consecutive assessments meeting the definition of PD (first PD by RECIST ver. 1.1 and a second PD using the progression confirmation criteria (above)) are required to establish objective disease progression (determination of definite PD). If PD by RECIST ver. 1.1 does not confirm objective disease progression, evaluation will continue until the next PD by RECIST ver. 1.1. In the absence of significant clinical progression, treatment with investigational agents may continue between the first assessment of progression and imaging studies to confirm progression. If PD is confirmed on confirmatory imaging, the date of progression will be the date PD was confirmed on the previous visit. If objective disease progression is not confirmed, the subject will continue to receive study drug and on-therapy evaluation until the next PD, if there is no clinically significant worsening, at which time another confirmatory scan will be required, even if objective disease progression is confirmed. If the initial PD is not immediately confirmed at the next scan, the investigator should not change the PD assessment from the initial scan.

If subjects discontinue treatment (and/or receive subsequent anticancer therapy) prior to radiographic progression, subjects should be followed until objective disease progression is confirmed. Once progression is confirmed, subjects should continue to be followed for survival every 2 months (8 weeks) according to the evaluation follow-up schedule.

7.5. Assessment of safety

7.5.1. Clinical laboratory tests

Blood and urine samples for determination of clinical chemistry, hematology, and urinalysis will be taken at the times indicated in the assessment schedules and as clinically indicated（refer to 7.1.）

Clinical laboratory safety testing, including serum pregnancy testing, is performed in a licensed clinical laboratory according to local standard procedures. Specimen tubes and specimen sizes may vary depending on the laboratory method used and routine practices at the site. Pregnancy testing may be performed at the site using an approved test (urine or serum pregnancy test). Abnormal clinically significant laboratory results should be repeated as soon as possible (preferably within 24 to 48 hours).

Additional safety samples may be collected if clinically indicated at the discretion of the Investigator. The date, time of collection, and results (values, units, and reference ranges) will be recorded on the appropriate eCRF.

The laboratory variables to be measured are presented in Table 4 (Hematology/ Coagulation), Table 5 (Clinical chemistry), and Table 6 (urinalysis).

Other safety tests to be performed at screening include assessment for hepatitis B surface antigen, hepatitis C antibodies, and HIV antibodies.

The following laboratory variables will be measured:

Table4. Hematology/Coagulation Laboratory Tests

Basophils	Monocytes
Eosinophils	Neutrophils
Hematocrit	Platelet count
Hemoglobin	Red blood cell count
Lymphocytes	Total white cell count [a]
PT-INR	APTT

Table5. Clinical Chemistry (Serum or Plasma) Laboratory Tests

Albumin	Lactate dehydrogenase
Alkaline phosphatase	Lipase
Alanine aminotransferase	Magnesium
Amylase	Potassium
Aspartate aminotransferase	Sodium
Calcium	Total bilirubin[a]
Chloride	Total protein
Creatinine	Urea or blood urea nitrogen, depending on local practice
Gamma glutamyltransferase[b]	Uric acid
Glucose	

a. Tests for ALT, AST, alkaline phosphatase, and total bilirubin must be conducted and assessed concurrently. If total bilirubin is ≥2 × upper limit of normal (and no evidence of Gilbert's syndrome) then fractionate into direct and indirect bilirubin.

b. It is preferable that both amylase and lipase parameters are assessed. For sites where only 1 of these parameters is routinely measured then either lipase or amylase is acceptable.

c. Bicarbonate (where available), chloride, creatinine clearance, gamma glutamyltransferase, and magnesium testing are to be performed at baseline, on Day 1 (unless all screening laboratory clinical chemistry assessments are performed within 3 days prior to Day 1), and if clinically indicated.

d. Creatinine Clearance will be calculated by data management using Cockcroft-Gault (using actual body weight).

e. If TSH is measured within 14 days prior to Day 1 (first infusion day), it does not need to be repeated at day Free T3 or free T4 will only be measured if TSH is abnormal or if there is a clinical suspicion of an AE related to the endocrine system

Table 1. Urinalysis Tests[a]

Bilirubin	pH
Blood	Protein
Glucose	Specific gravity
Ketones	Colour and appearance

a. Microscopy should be used as appropriate to investigate white blood cells and use the high-power field for red blood cells

If a patient shows an AST or ALT ≥3xULN together with total bilirubin ≥2xULN, refer to 8.18.3 for further instructions on cases of increases in liver biochemistry and evaluation of Hy's Law. These cases should be reported as SAEs if, after evaluation, they meet the criteria for a Hy's law case or if any of the individual liver test parameters fulfill any of the SAE criteria.

All patients should have further chemistry profiles performed at 30 days (±3 days), 2 months (±1 week) and 3 months (±1 week) after permanent discontinuation of IP

Any clinically significant abnormal laboratory values should be repeated as clinically indicated and recorded on the eCRF. Situations in which laboratory safety results should be reported as AEs are described in Section8.1.

All patients with Grade 3 or 4 laboratory values at the time of completion or discontinuation from IP must have further tests performed until the laboratory values have returned to Grade 1 or 2, unless these values are likely to improve because of the underlying disease.

7.5.2. Physical examinations

Physical examinations will be performed according to the assessment schedules. Full physical examinations will include assessments of the head, eyes, ears, nose, and throat and the respiratory, cardiovascular, GI, urogenital, musculoskeletal, neurological, dermatological, hematologic/lymphatic, and endocrine systems. Height will be measured at screening only. Targeted physical examinations are to be utilized by the Investigator on the basis of clinical observations and symptomatology. Situations in which physical examination results should be reported as AEs are described in Section 8.1.

7.5.3. Electrocardiogram (ECG)

Resting 12-lead ECGs will be recorded at screening and as clinically indicated throughout the study. ECGs should be obtained after the patient has been in a supine position for 5 minutes and recorded while the patient remains in that position.

In case of clinically significant ECG abnormalities, including a QTcF value >470 ms, 2 additional 12-lead ECGs should be obtained over a brief period (e.g., 30 minutes) to confirm the finding.

Situations in which ECG results should be reported as AEs are described in Section 8.1.

7.5.4. Vital signs

Vital signs (blood pressure [BP], pulse, temperature, and respiration rate) will be evaluated according to the schedule of this clinical trial. Body weight is also recorded at each visit along with vital signs.

First infusion

On the first infusion day, patients will be monitored, and vital signs collected/recorded in eCRF prior to, during and after infusion of IP as presented in the bulleted list below.

BP and pulse will be collected from a subject before, during, and after each infusion at the

following times (based on a 60-minute infusion):

- Prior to the beginning of the infusion (measured once from approximately 30 minutes before up to 0 minutes [i.e., the beginning of the infusion])
- Approximately 30 minutes during the infusion (**halfway** through infusion)
- At the end of the infusion (approximately 60 minutes ±5 minutes)

If the infusion takes longer than 60 minutes, then BP and pulse measurements should follow the principles as described above or be taken more frequently if clinically indicated. A 1-hour observation period is recommended after the first infusion of durvalumab.

Subsequent infusions

BP, pulse and other vital signs should be measured, collected/recorded in eCRF prior to the start of the infusion. Patients should be carefully monitored and BP and other vital signs should be measured during and post infusion as per institution standard and as clinically indicated.

7.5.5. ECOG performance status

ECOG performance status will be assessed at the times specified in the assessment schedules based on the following:

0. Fully active; able to carry out all usual activities without restrictions
1. Restricted in strenuous activity, but ambulatory and able to carry out light work or work of a sedentary nature (e.g., light housework or office work)
2. Ambulatory and capable of self-care, but unable to carry out any work activities; up and about more than 50% of waking hours.
3. Capable of only limited self-care; confined to bed or chair more than 50% of waking hours
4. Completely disabled; unable to carry out any self-care and totally confined to bed or chair
5. Dead

Any significant change from baseline or screening must be reported as an AE.

7.5.6. Other safety assesements

If new pulmonary symptoms (e.g., dyspnea) or radiological abnormalities suggestive of pneumonia/interstitial lung damage (ILD) are observed, toxicity management as detailed in the Toxicity Management Guidelines (see Appendix of Protocol 2) will be applied. Complete diagnostic results (including high-resolution computed tomography (HRCT), blood and sputum cultures, hematological parameters, etc.) should be recorded in the source documents. An accurate diagnosis including consultation with a specialist is strongly recommended to rule out alternative causes such as lymphangitic carcinomatosis, infection, allergy, cardiogenic edema, or pulmonary hemorrhage. In the presence of a confirmatory HRCT scan that excludes other causes of respiratory symptoms, the diagnosis of interstitial lung disease (ILD) should be considered and toxicity management guidelines followed. The investigator is responsible for ensuring that all staff involved in the study are familiar with the contents of this section.

8. HANDLING OF ADVERSE EVENT

8.1. Definition

8.1.1. Adverse event

Adverse events are all unwanted or unintended signs (including abnormal changes in laboratory values), symptoms, or illnesses that occur after the first administration of an investigational drug, regardless of causal relationship to the study treatment.

8.1.2. Severe adverse event

A serious adverse event is defined as any of the following

（1） results in death
（2） is life-threaening
（3） requires inpatient hospitalisation or prolongation of existing hospitalisation
（4） results in persistent or significant disability/incapacity
（5） is a congenital anomaly/birth defect
（6） Other serious cases according to the above

The term “life-threatening” for the purposes of this definition is defined as an event that the investigator/participating physician determines poses an imminent risk of death to the subject as a result of its manifestation. It does not mean hypothetically that death might have resulted had the manifestation of the event been more severe.

“Hospitalization” in (3) below is not considered a serious adverse event if any of the following apply: (However, a new occurrence during the hospitalization is treated as an adverse event. (However, any new occurrence during that hospitalization will be treated as an adverse event.)

- Hospitalization as defined in this study protocol
- Hospitalization or prolongation of hospital stay that was planned prior to the start of the clinical trial.
- Hospitalization or prolonged hospitalization for social reasons (reasons of convenience or other non-medical necessity)
- Hospitalization or extended hospital stay for examination, education
- Hospitalization for follow-up or prolongation of hospitalization for those who are cured or have a mild illness.
- Hospitalization or prolonged hospitalization for new treatment of the underlying disease after completion of the investigational drug

(6) Other serious, in accordance with the above, are “significant medical events” that may not be immediately life-threatening or result in death or hospitalization, but which may endanger the subject or require treatment or therapy to avoid the consequences as listed in these definitions.

Adverse events (AEs) for malignancy reported during a clinical trial will generally be evaluated as serious AEs. If no other severity criteria are met, the AE will be judged to be a “Significant Medical Event” as described above. However, in certain circumstances, medical judgment based on individual events should be applied to clarify that malignancy events should be evaluated and reported as non-serious AEs. For example, if medical history includes malignancy and the malignancy progresses during the clinical trial, but the progression does not change the treatment or prognosis of the malignancy, the malignancy progression should be reported as an AE but may not meet the attributes to be evaluated as serious.

The causal relationship of the SAE (relationship to all investigational treatments/procedures) should be evaluated by the investigator and reported to AstraZeneca.

8.1.3. Adverse Events of Special Interest (AESI)

AESIs of durvalumab and tremelimumab for heavy particle irradiation include events due to potential inflammatory or immune-mediated mechanisms, which require more frequent monitoring and treatment with steroids, immunosuppressive agents, and/or hormone replacement therapy.Careful monitoring of these AESIs will be implemented in clinical trials of durvalumab monotherapy and durvalumab plus tremelimumab combination therapy. Immune-mediated

adverse events (imAEs) are AESIs, defined as events that occur with the administration of (exposure to) an investigation agent, are consistent with an immune-mediated mechanism of action, and have no apparent other cause. imAEs should be diagnosed using serologic, immunologic, and histologic (biopsy) data, as appropriate, for support. The diagnosis of imAE should be supported by serologic, immunologic, and histologic (biopsy) data as appropriate, and efforts should be made to rule out tumor, infection, metabolism, toxins, and other causes of imAE.

If there is any doubt as to whether an adverse event is an imAE, the investigator should immediately contact the sponsor's medical experts. AESIs for durvalumab and/or tremelimumab and heavy particle irradiation include the following.

- Dysentery/colitis, intestinal perforation
- Pneumonitis/ILD
- Hepatitis/transaminases increase
- Endocrine disorders (i.e., hypophysitis, hypopituitarism, adrenal insufficiency, hyperthyroidism, hypothyroidism and type I diabetes mellitus)
- Rash/dermatitis
- Nephritis/increased blood creatinine
- Pancreatitis/increased serum lipase and amylase
- myocarditis
- pericarditis
- Myositis/polymyositis
- Neuropathy/neuromuscular toxicity (Guillain-Barré syndrome, myasthenia gravis, etc.)
- Other inflammatory reactions of rare / infrequent immune-mediated (but not limited to pericarditis, sarcoidosis, uveitis, and ocular, skin, blood system and rheumatology-related events)

In addition, reactions associated with infusion and hypersensitivity/anaphylactic reactions due to various pharmacologic causes are also considered AESIs.

Details of these risks and presenting symptoms are described in the most recent versions of the durvalumab and tremelimumab investigational new drug summaries. Specific guidelines for the evaluation and treatment of these AESIs are provided in the Dose Modification and Toxicity Management Guidelines. These guidelines were developed by the investigational drug provider to assist investigators and others in making clinical decisions when treating this type of toxicity. This guideline applies to adverse events that the reporting investigator determines are causally related to the investigational drug/regimen.

If new or worsening pulmonary symptoms (e.g., dyspnea) or radiological abnormalities suggestive of pneumonia/interstitial lung disease are observed, toxicity management as detailed in the “Toxicity Management Guidelines” (see Appendix F) will be applied. Results of complete diagnostic tests (including high-resolution computed tomography (HRCT), blood and sputum cultures, hematological parameters, etc.) should be recorded in the source documents.

An accurate diagnosis, including consultation with a specialist, is strongly recommended to rule out alternative causes such as lymphangitic carcinomatosis, infection, allergy, cardiogenic edema, or pulmonary hemorrhage. On confirmatory HRCT scans where other causes of respiratory symptoms have been ruled out, the diagnosis of interstitial lung disease (ILD) should be considered and toxicity management guidelines followed.

8.1.4. Confirmation of interstitial lung disease (ILD)

To ensure thorough investigation and diagnosis of possible cases of pneumonia, the following evaluations, and additional evaluations as needed, will be performed. Collect the results of the evaluation.

- physical examination

- Evaluate signs and symptoms (cough, shortness of breath, fever, etc.), including auscultation of the lung field.
- Peripheral oxygen saturation (SpO2)

Other

If pneumonia (ILD) is suspected during clinical trial treatment, the following markers should be measured.

if possible

(i) ILD markers (KL-6, SP-D) and β-D-glucan

(ii) Tumor markers. Specific tumor markers associated with disease progression.

Other biochemistry: CRP, LDH

8.2. Assessment of severity

AEs and SAEs; severity will be determined in accordance with CTCAE ver. 5.0 The severity of all other events not listed in the CTCAE will be determined by the severity category from Grade 1 to 5, as determined by the investigator based on medical judgment, will be as follows.

- Grade 1 (mild)
  Events that are usually transient and require only minimal Treatment or therapeutic intervention. The event generally does not interfere with normal activities of daily living.

- Grade 2(Moderate)
  An event that is usually alleviated by additional specific therapeutic intervention. The event interferes with normal daily activities and causes discomfort, but does not pose a risk of serious or permanent harm to the subject.

- Grade 3(Severe)
  An event requiring intensive therapeutic intervention. An event that interferes with normal daily activities or significantly affects the subject's clinical condition.

- Grade 4(life-threatening）
  Events and/or immediate sequelae related to the following Imminent risk of death or a physical or mental impairment that affects or limits the ability to perform activities of daily living (eating, walking, toileting, etc.).

- Grade 5(deadly)
  Death as a result of an event. it is important to distinguish between serious criteria and severity of AEs.

It is important to distinguish between severity and severity of an AE. Severity is a measure of intensity, and severity is defined by the criteria in section 10.3.1 A Grade 3 AE need not necessarily be considered an SAE. For example, a Grade 3 headache lasting several hours would not meet the regulatory definition of an SAE and would be considered a non-serious event, whereas a Grade 2 attack leading to hospitalization would be considered an SAE.

8.3. Record of adverse events and serious adverse events

AEs and SAEs will be collected from the time of the first dose of study drug until the follow-up date 28 days after the last dose of study drug. However, AE and SAE outcomes will be collected until the end of the safety information collection period (90 days after the last dose of study drug) or until the start of alternative anticancer therapy. Of events occurring after the 28-day follow-up period after the last dose of the investigational drug, if the event is considered to be due to delayed toxicity to the investigational drug, it will be collected as an AE or SAE until the end of the safety information collection period or until the start of alternative anticancer

therapy. All AEs and SAEs will be actively followed up for each subject for the duration of the trial as long as the event is ongoing. Every effort will be made to resolve all events, even if the event continues after the subject discontinues the investigational drug or after the study is terminated.

AEs unresolved at the subject's last clinical trial visit will be followed up by the investigator as long as medically necessary but will not be further documented in the eCRF. AstraZeneca reserves the right to request additional information from subjects with ongoing AEs/SAEs at the end of the trial if deemed necessary.

For each AE, the following information should be collected

- Adverse event name
- Date of adverse event and date of disappearance
- Maximum CTCAE Grade
- Severity
- Assessment of causal relationship with study treatment
- Treatments related to investigational drugs
- Treatment of adverse events: Administration of AE medication
- outcome

In addition to the above, the following are also confirmed for SAE.

- Date of serious adverse event
- Date when the investigator becomes aware of the onset of the serious adverse event
- Definitions applicable to the determination of serious adverse events
- Date of admission
- Date of discharge
- Presumed reason for death
- Date of death
- Autopsy
- Evaluation of causal relationship with clinical trial procedures
- Evaluation of causal relationship with other drugs
- Details of serious adverse events

The grading scale described in CTCAE ver. 5.0 is used for all events that have been assigned to a CTCAE Grade; for events that have not been assigned a CTCAE Grade, the CTCAE criteria for converting mild, moderate, and severe events to a CTCAE Grade A copy of CTCAE ver. 5.0 can be downloaded from the Cancer Treatment Evaluation Program website at http://www.jcog.jp/doctor/tool/ctcaev5.html.

8.4. Duration of recording and follow-up of adverse events and serious adverse events

If the subject discontinues treatment for reasons other than objective confirmation of disease progression and therefore continues tumor evaluation, the drug- or treatment-related SAEs will be tracked until the subject confirms PD and no further tumor evaluation is performed.

The investigator is responsible for tracking all SAEs until the subject returns to baseline status or until the condition stabilizes with the expectation that the chronic condition will be maintained, even if it continues beyond study participation, until all SAEs are resolved.

8.5. Causal relationship with investigational therapy

The following examples will be used to determine the causal relationship between the clinical trial treatment and the patient.

Causative: if a reasonable possibility can be explained that the investigational treatment caused the adverse event in question (examples are given below).

1) If the event is time-related to the onset of the event, and the event attenuates with the passage of time after the study treatment but recurs or worsens with subsequent re-administration of the study treatment.
2) The presence of confounding risk factors is negative, such as the subject's general condition, complications, concomitant medications, or concomitant therapies.

No causal relationship: other than above

8.6. Outcome definition

Outcomes after adverse events are determined from the following

1) Recovery: when the patient recovers to the state before the adverse event occurred.
2) Recovered but with sequelae: When the adverse event has recovered but the effects of the adverse event remain as sequelae
3) Death: when the adverse event that occurred was the direct cause of death
4) Lightening of symptoms: adverse events continue, but symptoms are improving
5) Unrecovered: adverse events continue (symptoms are not improving)
6) Unknown: When the subject is no longer traceable

8.7. Treatment of investigational drug in the event of an adverse event

1) No change: When an adverse event occurs but there is no change in the conditions under which the investigational drug is administered.
2) Discontinuation: Discontinuation of study drug administration due to the occurrence of an adverse event
3) Withdrawal: Temporary suspension of study drug administration due to the occurrence of an adverse event
4) Not applicable: Adverse events occur before the start of study drug administration or after the end of the study period

8.8. Treatment of heavy particle therapy equipment in the event of an adverse event

1) Suspension: Temporary suspension of heavy-ion radiation therapy due to the occurrence of adverse events
2) Discontinuation: Discontinuation of heavy-ion radiation therapy due to the occurrence of an adverse event
3) Not applicable: Adverse events occur before or after the start or completion of carbon ion radio therapy

8.9. Relationship to Protocol Procedures

The investigator must also provide an assessment of the relationship between SAEs and protocol procedures on the SAE Report Form. This includes both non-therapeutic emergencies (SAEs that occur prior to the administration of the investigational drug) and therapeutic emergency SAEs. Protocol-related SAEs may occur because of a required procedure or intervention (e.g., blood draw) during the clinical trial. The investigator should use the following guidelines to assess the relationship between SAEs and protocols

- Protocol-related: The event occurred because of a procedure or intervention described in the protocol for which no alternative etiology exists in the subject's medical record.
- Not protocol-related: The event is related to an etiology other than the procedure or intervention described in the protocol. The alternative etiology must be documented in the study subject's medical record.

In the case of AEs, AEs on treatment (or AEs appearing on treatment) are defined as AEs that began after or before administration and worsened after exposure to treatment between the start dose date and 90 days after discontinuation of study treatment.

8.10. Adverse events based on signs and symptoms

All AEs reported spontaneously by the subject or in response to questions from the investigator, subinvestigator, or collaborator (example of a visit: "Have you had any health problems since your last visit / have you been asked any questions since your last visit?") ")

When collecting AEs, recording the diagnosis is preferred over recording a list of signs and symptoms, if possible. However, if the diagnosis is known and there are other signs and symptoms that are not generally part of the diagnosis, record the diagnosis and each sign or symptom separately.

8.11. Adverse events based on tests and examinations

Protocol-mandated laboratory values and vital signs measurements will be summarized in the CSR. Therefore, protocol-mandated worsening of laboratory values and vital signs relative to baseline should only be reported as an AE if it meets one of the SAE criteria or is a reason for discontinuation of treatment with the investigational drug.

If the worsening of laboratory values or vital signs is associated with clinical signs or symptoms, the signs or symptoms should be reported as an AE and the associated laboratory results or vital signs should be reported as additional information, as appropriate. Whenever possible, the reporter uses clinical terms rather than laboratory terms (e.g., "anemia" rather than "low hemoglobin level"). In the absence of clinical signs or symptoms, the worsening of such laboratory values should be reported as an AE.

Laboratory deterioration attributable to apparent disease progression shall not be considered an adverse event or serious adverse event.

Report as an adverse event any new or worsening clinically significant abnormal findings at the time of presentation compared to the baseline evaluation.

8.12. Hy's Law

Biochemical elevations suggestive of abnormal liver function may require further evaluation, and the occurrence of AST or ALT ≥ 3 x ULN and total bilirubin ≥ 2 x ULN may require reporting as an SAE. For cases of elevated liver biochemistry and Hy's Law evaluation, see Ref.

8.13. Disease progression

Disease progression is considered a worsening of the subject's condition due to the disease for which the investigational drug is being studied and is an increase in the severity of the disease under study and/or an increase in the symptoms of the disease. The development of new metastases or progression of existing metastases to the primary cancer being studied is considered disease progression and is not considered an AE. Events that are clearly attributable to disease progression should not be reported as AEs during a clinical trial.

8.14. New cancer

The development of new cancer shall be considered a serious adverse event. New cancers are those that occur after a patient is enrolled in the study, rather than those that were the primary reason for enrollment in the study. New metastatic lesions are considered progression of the cancer under study and are not reported as a second cancer.

8.15. Deaths

All deaths occurring during the investigational treatment period or within the protocol-defined follow-up period after the last dose of the investigational drug shall be reported as follows.

- Events that are clearly attributable to disease progression will not be treated as investigational AEs, but if the outcome of the event is found to be severe, all SAEs, not limited to death, will be reported, as described in Section 10.14.
- The investigator shall immediately report to the site director and the investigational drug provider, regardless of the causal relationship between the SAE and the investigational drug/IR, regardless of whether the death is due to progression of the disease under study. In addition, a note will be made in the eCRF.
- The causes are identified and described in the eCRF.
- Deaths of unknown cause should always be reported as SAEs.
- If an autopsy is performed, the autopsy results should be reported to AstraZeneca.

Deaths that occur after the last dose of the investigational drug and after the protocol-defined safety information collection period should also be noted in the eCRF as required. If the death is attributable to an event that occurred after the safety information collection period and the event is considered to be due to delayed toxicity to the investigational drug, it must also be reported as an SAE.

AstraZeneca retains the right to request additional information for subjects with ongoing AE(s)/SAE(s) at the end of the trial if deemed necessary.

8.16. Reportable Adverse Events

The Investigator or Co-Investigator will ensure that all adverse events that occurred from the time consent is obtained until 28 days after the end of study drug administration are consistently described in the case report form and that adverse events disappear or that the study period is observed until 4 weeks after the end of the study period (after discontinuation). Adverse events that are judged to have a causal relationship with the study drug will continue to be observed as much as possible until the end of the study period. However, this does not apply if the investigator determines that the safety of the subject is sufficiently ensured and further follow-up is not necessary.

8.17. Reporting of serious adverse events

All serious adverse events during the clinical trial (regardless of whether there is a causal relationship with the investigational drug or not) and serious adverse events suspected to be related to the investigational drug after the completion (discontinuation) of the clinical trial shall be reported according to the following procedures. The details of the reporting procedures are separately stipulated in the “Standard Operating Procedures for Handling Safety Information". An outline of the reporting procedure is provided below.

1） Reports from the investigator to the head of the site and to investigators at other sites

If any serious safety information is recognized during the clinical trial period, the investigator shall immediately report the details to the head of his/her institution, the investigational drug supplier, and the investigators at other sites using the “Report on Serious Adverse Events and Malfunctions” (Uniform Form 12, or the same detailed description form, if necessary). In reporting, the investigator shall specify whether or not the serious safety information is an event that cannot be predicted from the investigator’s brochures, etc.

2） Discussion between the investigator and the Clinical Trial Coordinating Committee

The investigator shall consult with the Trial Coordinating Committee and report his/her opinion as the investigator (including the necessity of reporting to the Minister of Health, Labour and Welfare) to the Trial Coordinating Committee. The investigator shall report his/her opinion (including the necessity of reporting to the Minister of Health, Labour and Welfare) to the Trial Coordinating Committee. If the Independent Data Monitoring Committee is consulted regarding the investigator's judgment, the opinion of the Independent Data Monitoring Committee shall be

followed.

3） Report to the Minister of Health, Labour and Welfare and the heads of other medical institutions

1 Report to the Minister of Health, Labor and Welfare

If the Clinical Trial Coordinating Committee determines that the case is subject to reporting as stipulated in Article 273 of the Enforcement Regulations of the Act on Quality, Efficacy and Safety Assurance of Pharmaceuticals, Medical Devices and Other Products (Pharmaceuticals and Medical Devices Law), it shall report to the Minister of Health, Labor and Welfare (the Pharmaceuticals and Medical Devices Agency, an independent administrative agency (PMDA)). In addition, when the Independent Data Monitoring Committee is consulted, it shall be notified of the contents of the report made to this authority.

2 Reporting to the heads of other implementing medical institutions

When a report is made to the Minister of Health, Labour and Welfare, the investigator of the other site shall report the contents of the “Failure/Infectious Case Report” obtained from the Trial Coordinating Committee to the head of his/her site as soon as possible.

4） Actions to be taken when additional information is obtained

When additional information regarding the adverse event is obtained, the investigator of the site where the adverse event occurred shall make an additional report to the head of the site as soon as possible, as well as to the Clinical Trial Coordinating Committee and the investigational drug supplier. The handling of such additional information shall be in accordance with the procedures described in 1) to 3) above, and reports shall be made to the NIH as necessary.

5） Annual report to the Minister of Health, Labor and Welfare

If the investigational drug has not been approved in Japan, or if the sponsor is not conducting a clinical trial, the person conducting the clinical trial shall submit an annual report to PMDA in accordance with Article 273 of the Ordinance for Enforcement of the Pharmaceutical Affairs Law.

The investigator will report all SAEs to AstraZeneca. The investigator will send a copy of the SAE report submitted to PMDA to the investigational drug provider in accordance with a separate Standard Operating Procedure (SOP). If any changes are made to the follow-up report, the following information will also be promptly sent to the investigational drug provider.

8.17.1. Response to subjects

When a serious adverse event is observed, the investigator or subinvestigator should immediately take appropriate measures, discontinue administration of the investigational drug to ensure the safety of the subject, and inform the subject if treatment for the adverse event becomes necessary. If an adverse event that was considered to be causally related to the investigational drug at the time of discontinuation has not yet recovered, observation will be continued as much as possible until the adverse event recovers or becomes mild, in principle. However, this does not apply when the investigator determines that the subject's safety has been sufficiently ensured and further follow-up is not necessary.

8.18. OTHER EVENTS REQUIRING REPORTING

8.18.1. Overdose

Use of durvalumab or tremelimumab in excess of the prescribed dose of the drug constitutes an overdose. Currently, there is no established treatment for durvalumab or tremelimumab overdose and no established symptoms of a possible overdose. Overdoses with associated AEs are recorded as an AE diagnosis or symptom in the relevant AE module of the eCRF. Overdoses without symptoms are only recorded and reported to the site investigator.

Overdoses of durvalumab or tremelimumab, with or without associated AEs/SAEs, must be recorded and reported to the site investigator. The investigator must report these to the investigational drug provider as described in the SOP.

8.18.2. Hepatic function abnormality

Abnormal liver function that meets Hy's Law criteria should be reported as an SAE regardless of the presence or absence of clinical symptoms. Hy's Law criteria are met if AST or ALT is at least 3 times the upper limit of normal (ULN) or TBL is at least 2 times the upper limit of normal at any time after initiation of treatment with the investigational drug, regardless of elevated ALP.

If it cannot be determined that there is no causal relationship to the investigational drug (e.g., due to cholelithiasis or bile duct obstruction), the investigator should report these events to the study provider in accordance with SOPs.

If the cause of the hepatic dysfunction is established and there is no causal relationship to the investigational drug, the investigator will decide whether to continue the subject's treatment based on the investigator's clinical judgment.

- If the cause of the liver function abnormality is not established, the subject's administration should be discontinued immediately. Follow-up investigations and inquiries will be initiated by the investigational site without delay.

The investigator will follow up on each reported event of liver function abnormality.

8.18.3. Pregnancy

All pregnancies and pregnancy outcomes should be reported to AstraZeneca and the site investigator, except in the following cases

- Pregnancy discovered before the subject received the investigational drug.
- Pregnancy of the male subject's female partner. (If the male subject is not restricted from having children.)

8.18.4. Exposure to pregnant woman

If a subject becomes pregnant during a clinical trial, the investigational drug should be discontinued immediately. Pregnancy itself shall not be considered an AE unless there is a suspicion that the investigational drug interfered with the effectiveness of the contraceptive. Birth defects or birth defects and spontaneous abortions should be reported and treated as SAEs. Uncomplicated elective abortions shall not be treated as AEs. The outcome of all pregnancies (spontaneous abortion, elective abortion, ectopic pregnancy, normal delivery, and congenital anomalies) shall be followed up and documented even if the clinical trial is discontinued. If pregnancy occurs during the clinical trial, the investigator shall notify AstraZeneca in accordance with the SOP.

8.18.5. Exposure to partner

Male subjects should refrain from sexual intercourse or sperm donation with their partners during the study and for 180 days after the last dose of durvalumab plus tremelimumab combination therapy or 90 days after the last dose of durvalumab monotherapy, whichever is longer. Pregnancy in the subject's partner is not considered an AE. However, the outcome of all pregnancies (spontaneous abortion, elective abortion, ectopic pregnancy, normal delivery, or congenital anomaly) occurring between the date of first dose and 180 days after the last dose of durvalumab + tremelimumab combination therapy or 90 days after the last dose of durvalumab

monotherapy, whichever is longer, will be followed up and documented if possible. Follow-up and documentation, if possible, is desirable. Upon receipt of a report of pregnancy, the investigator must obtain the consent of the subject's partner prior to obtaining any information regarding the pregnancy. Therefore, the study team should adopt the generic ICF template according to the procedure and submit it to the Institutional Review Board (IRB) prior to use.

8.19. Medication error

For this study, a medication error is an unintentional error in the course of treatment with an investigational drug that may cause harm to the subject. A medication error is not a lack of efficacy of the investigational drug, but an artificial or process-related failure while the investigational drug is under the control of the site staff or subject. Medication errors include the circumstances under which the error occurred.

- A medication error occurred.
- A medication error occurred, but was identified before the subject received the medication.
- No medication errors occurred, but circumstances were observed that could have resulted in errors.

Examples of events that should be reported in a clinical trial as medication errors

- Confusion of drug names
- Dispensing errors (e.g., some medications were incorrectly dispensed even though they were not actually administered to the subject.
- Drugs that were not administered as directed, such as by incorrect route or site of administration
- Drugs not taken as directed, such as tablets dissolved in water when they should be taken as solid tablets
- Drugs not stored as directed (e.g., in a refrigerator when they should be at room temperature.)
- Subjects who received medication by mistake
  Drugs administered to the wrong subject

Examples of events that need not to be reported as medication errors in clinical research

- Including those that lead to any of the above events or that result in a dose error.
- If the subject fails to take the medication (e.g., forgets to take the medication）
- Overdose Accidents
- Subject did not return unused or empty packaged medications
- Errors associated with background or rescue drugs, even AstraZeneca products, or standard of care drugs in open label studies

Medication errors are not considered AEs, but AEs may occur because of medication errors. If a medication error occurs during a clinical trial, the investigator or other site personnel should contact AstraZeneca within one day.

8.20. Predicted Side Effects

The following is a tabulation of adverse events that occurred in clinical trials of durvalumab. For other adverse events, please refer to the Investigators brochure.

（1）Information on adverse events in 2769 patients treated with durvalumab (all grades, incidence >5%)

Side effect	Occurrence frequency
Fatigue	769 （27.8 %）
anorexia	584 （21.1 %）
cough	529 （19.1 %）
Nausea	515 （18.6 %）

Breathe bitterly	500	(18.1 %)
constipation	473	(17.1 %)
diarrhea	467	(16.9 %)
fever	393	(14.2 %)
anaemia	348	(12.6 %)
Back pain	342	(12.4 %)
vomiting	339	(12.2 %)
Itching	314	(11.3 %)
lethargy	306	(11.1 %)
Joint pain	299	(10.8 %)
Hypothyroidism	269	(9.7 %)
rash	266	(9.6 %)
headache	265	(9.6 %)
Peripheral edema	258	(9.3 %)
sleeplessness	234	(8.5 %)
bellyache	219	(7.9 %)
Weight loss	205	(7.4 %)
Musculoskeletal pain	194	(7.0 %)
dizziness	189	(6.8 %)
pneumonia	186	(6.7 %)
Urinary tract infections	182	(6.6 %)
Muscle pain	173	(6.2 %)
Upper respiratory tract infections	163	(5.9 %)
AST elevation	162	(5.9 %)
Pain in the extremities	158	(5.7 %)
Hyponatremia	152	(5.5 %)
ALT elevation	149	(5.4 %)
Wet cough	142	(5.1 %)
Nasopharyngitis	140	(5.1 %)

(2) Information on adverse events in 1822 patients treated with durvalumab and tremelimumab in combination (all grades, incidence >5%)

Side effect	Occurrence frequency	
fatigue	542	(29.7 %)
diarrhea	485	(26.6 %)
anorexia	461	(25.3 %)
nousea	447	(24.5 %)
pruritic	395	(21.7 %)
constipation	365	(20.0 %)
dyspnea	361	(19.8 %)
anaemia	339	(18.6 %)
fever	305	(16.7 %)
vomiting	286	(15.7 %)
coughing	271	(14.9 %)
backache	247	(13.6 %)
rash	246	(13.5 %)
lethargy	219	(12.0 %)
abdominal pain	217	(11.9 %)
peripheral edema	203	(11.1 %)
weight loss	203	(11.1 %)
arthralgia	195	(10.7 %)
hypothyroidism	186	(10.2 %)

insomnia	175	(9.6 %)
AST elevation	172	(9.4 %)
hyponatremia	172	(9.4 %)
ALT elevation	154	(8.5 %)
headache	172	(8.3 %)
Lipase rise elevation	141	(7.7 %)
dizziness	134	(7.4 %)
amylase elevation	133	(7.3 %)
hypokalemia	131	(7.2 %)
pneumonia	125	(6.9 %)
maculopapular eruption	125	(6.9 %)
Urinary tract infections	117	(6.4 %)
ALP elevation	115	(6.3 %)
dehydration	114	(5.4 %)
Hyperthyroidism	110	(5.1 %)
hyperglycemia	109	(6.0 %)
γ-GTP elevation	103	(5.7 %)
Hypoalbuminemia	100	(5.5 %)
Musculoskeletal pain	99	(5.4 %)
Muscle pain	99	(5.4 %)
Dry skin	98	(5.4 %)
Wet cough	98	(5.4 %)
Xerostomia	95	(5.2 %)
musculoskeletal chest pain	93	(5.1 %)
upper respiratory tract infection	92	(5.0 %)

（3）Refer to Section 2.3.2.3 for more information on the risks of Carbon ion radio therapy.

9. ENDPOINT

9.1. Primary endpoint

Percentage of dose-limiting toxicities (DLT) and adverse events/serious adverse events

Rationale for Setting the Primary Endpoint<br><br>To evaluate the tolerability and safety of durvalumab tremelimumab in combination with carbon ion radio therapy in subjects with advanced hepatocellular carcinoma with vascular invasion.

9.2. Secondary endpoint

Overall survival (OS), 6-month survival, objective response rate (ORR) calculated by RECIST ver 1.1 and mRECIST, 6-month progression-free survival (PFS), Time to progression (TTP)

Rationale for Setting Secondary Endpoints<br><br>To evaluate the efficacy of durvalumab tremelimumab in combination with carbon ion radio therapy in subjects with advanced hepatocellular carcinoma with vascular invasion.

9.3. Exploratory endpoints

In addition to consent for this study, consent for “Exploratory Study of Tumor Cells and Tumor Microenvironment in Hepatocellular Carcinoma Using Tumor and Non-tumor Biopsy Specimens, Blood Specimens, and Stool Specimens” and “Exploratory Study of Tumor Cells and Tumor Microenvironment by Secondary Use of Biological Specimens in Hepatobiliary Pancreatic Cancer” and a liver biopsy or liver tumor biopsy specimen must be In patients with sufficient liver biopsy/tumor biopsy samples, biomarkers associated with clinical outcome of combination therapy with durvalumab/tremelimumab and carbon ion radio therapy will be explored using blood and liver biopsy/tumor biopsy samples. The analysis will be performed in the laboratory of the Department of Gastroenterology, Graduate School of Medicine, Chiba University, and may be contracted to an outside vendor or outside research organization.

10. STATISTICAL METHODS AND SAMPLE SIZE DETERMINATION

10.1. Description of Analysis set

10.1.1. DLT analysis set

All subjects enrolled in the study, who have completed at least one investigational drug regimen (IR), and for whom a DLT evaluation has been performed, are considered the DLT analysis set.

10.1.2. Safety analysis set: SAF

All subjects who have had at least one dose of the investigational drug or investigational drug regimen (IR) are considered in the Safety analysis set.

10.1.3. Full Analysis set: FAS

All subjects with eligible disease who are properly enrolled and have received at least one dose of the investigational drug regimen are considered Full Analysis set (FAS).

10.1.4 Efficacy Evaluable set: EES

The efficacy evaluation analysis(EES) population is defined as a subset of the FAS. That is, it consists of all subjects who are eligible for the trial, receive the study drug/IR, and have had at least one post-dose efficacy endpoint assessed. In addition to subjects with at least one baseline and at least one post-dose efficacy evaluation, the population will include cases of early death or early progression before the evaluation.

10.1.5 Per protocol set：PPS

Subjects from the FAS who do not have any of the following serious violations of the study protocol, including the study protocol, such as the method of study or concomitant therapy, will be considered to be in compliance with the study protocol (PPS).

- Violation of inclusive criteria
- Violation of exclusion criteria
- Violation of concomitant use of prohibited drugs
- Violation of concomitant use of prohibited therapies

10.2. Target number of cases and rationale for setting

Target cases: 3 to 15

A modified "3+3 design" will be used in this trial. Cohort A will be used to evaluate DLT for carbon ion radio therapy + durumab, and instead of dose escalation, the group with toremelimumab will be used as Cohort B to evaluate DLT.

10.3. Case Handling

The coordinating investigators and the statistical analyst will discuss and decide how to handle the registered cases. The coordinating investigator and the statistical analyst will also discuss and decide how to handle cases in case new problems arise.

10.4. Data Handling

In principle, the handling of data during data compilation and analysis shall be as follows. In case of doubt, the statistician and the coordinating Investigators shall discuss and decide the handling of the data. Details are described in the statistical analysis plan.

1) Missing values: Supplementation of missing values is not performed.
2) Reference values: Data with unreliable measured values due to hemolysis, etc. are not used in the aggregate analysis.
3) Time lag: Data not conducted during the specified observation period will not be used in the aggregate analysis for that period. For other data requiring consideration, the coordinating investigators will consult with medical experts to determine how to handle the data for analysis.

10.5. Statistical analysis items and analysis plan

All patients will be analyzed after completion of treatment with the investigational drug and after the data have been fixed, using the DLT evaluation population for DLT evaluation, the ESS analysis population for efficacy evaluation, and the safety analysis population for safety evaluation. For the efficacy evaluation using RECIST without survival analysis, FAS and PPS analysis and sensitivity analysis will be performed as necessary.

10.5.1. Analysis of subject background

The distribution of subject background data and summary statistics are calculated for each cohort. For nominal variables, frequencies and proportions of categories are shown. For continuous variables, summary statistics (number of cases, mean, standard deviation, median, range, and interquartile range) are calculated.

10.5.2. Analysis of primary endpoints

10.5.2.1. DLT Evaluation

1) DLT incidence rate

The number of DLT cases and incidence rate will be calculated for each cohort.

10.5.2.2. Safety Evaluation

1) Adverse event rate
2) Serious adverse event rate
3) Adverse events coded by MedDRA
4) List of SOC, PT, severity, relevance, etc.

10.5.3. Analysis of secondary endpoints

10.5.3.1 Efficacy Analysis

Overall survival (OS), 6-month survival rate, objective response rate (ORR), 6-month progression-free survival (PFS), and time to progression (TTP) will be determined as secondary parameters to evaluate efficacy in each cohort. For survival analysis, the Kaplan-Meier method will be used to determine the incidence rate at 6 months and the median and mean survival times using the ESS analysis group.

Overall Response Rate（ORR）

ORR (based on evaluation by investigators using RECIST 1.1) is defined as the percentage of patients with a CR or PR at one or more visits. Patients who had a response after discontinuation of treatment without PD and initiation of post-treatment are not included in the ORR response cases.

Progression-Free Survival（PFS）

PFS (based on evaluation using RECIST 1.1 by investigators and others) is defined as the time from allocation to objective disease progression or death (or cause of death in the absence of disease progression), regardless of whether the patient discontinued treatment or received other anticancer therapy before disease progression. Patients who have not progressed or died at the time of analysis will be censored at the date of the last RECIST 1.1-based evaluation. However, if a patient has progressed or died after two or more consecutive missed visits, the patient will be censored as of the date of the last RECIST 1.1-based evaluation. In the absence of evaluable visit data or baseline data, censor at Day 1 unless the patient has died between baseline and the second visit, in which case treat as an event with the date of death as the date of the event.

The PFS is always calculated based on the date the imaging study/evaluation was performed, not the date of the visit.

It is possible that an evaluation/imaging study based on RECIST 1.1 scheduled for a specific visit may be performed over several different days. In such cases, the following principles apply.

- For evaluation by the investigator, the earliest RECIST 1.1 evaluation/imaging test date when an element indicating progression is identified is the progression date.PFS
- When an evaluation is terminated, it shall be terminated on the last imaging inspection date of the inspection related to the specific overall effectiveness evaluation.

Time to Progression (TTP)

TTP (based on evaluation using RECIST 1.1 by the investigator and others) is defined as the period from the date of random assignment to the date of objective tumor progression. Death is not included in the definition of TTP. Death without progression is not included in the definition of TTP.

Patients who die without progression will be terminated at the time of death.

Image Evaluation

Imaging evaluation For all imaging evaluations, in addition to evaluation by the investigator or subinvestigators, a central judgment will be made by multiple radiologists to be separately determined.

10.5.4. Interim Analysis

No interim analysis will be performed in this clinical trial.

10.6. Final Analysis

After the follow-up period, analysis will be conducted after the data are obtained and the cases are fixed. The person in charge of statistical analysis compiles the "Analysis Report" and submits it to the coordinating investigator and the principal investigator. The coordinating investigator summarizes the contents of the analysis report and prepares a "summary report" summarizing the overall conclusions of the trial, problems, interpretation and discussion of the results, and future policies mainly from a clinical perspective, and obtains approval from the principal investigator.

10.7. Data Monitoring Committee

An Independent Data Monitoring Committee will be established for this clinical trial. The Independent Data Monitoring Committee will be established as an independent body from the investigators and will consist of three or more expert members who are independent of the study. The Independent Data Monitoring Committee will be established for the purpose of ensuring the safety of subjects. It will provide appropriate advice and recommendations in accordance with a separate protocol If the study is terminated before the end of the DLT evaluation period for reasons other than DLT criteria, the investigator will ask the Independent Data Monitoring Committee for its opinion on the addition of a case If a second DLT occurs, the investigator will ask the Independent Data Monitoring Committee to determine whether the study regimen is If a second DLT occurs, the investigator will ask the Independent Data Monitoring Committee for an opinion on whether the study regimen is intolerable. If necessary, the investigator will confirm whether tolerability was not an issue in the other cases.

If an SAE is reported during the trial, the investigator will check with the Independent Data Monitoring Committee whether to continue the trial and whether any changes should be made to the protocol. The Independent Data Monitoring Committee will provide the investigator with the results of the discussion in writing.

If the investigator determines that precautionary emergency measures are warranted, depending on the importance and scope of the report, actions may include suspension of enrollment and emergency communication to all participating sites.

11. COMPLIANCE AND DEVIATION FROM THE PROTOCOL

1) The investigator or subinvestigator shall conduct the clinical trial in compliance with this protocol.
2) The investigator or subinvestigator shall record the details and reasons for all deviations from the study protocol.
3) In the event of deviation from the protocol for the purpose of avoiding immediate danger to subjects or for other unavoidable medical reasons, the investigator shall immediately submit a document describing the details of the deviation and the reasons for it to the head of the implementing medical institution, and shall also promptly report the contents of said document to the Trial Review Committee via the head of the implementing medical institution. The contents of said documents shall be promptly reported to the Clinical Trial Review Committee via the head of the investigational institution.

12. CHANGES TO THE CLINICAL TRIAL PROTOCOL, CASE REPORT FORM, OR ANALYSIS PLAN

12.1. Revision of Clinical Trial Protocol and Case Report Form

The following procedures shall be followed when revising the clinical trial protocol and case report forms.

1) When the investigator becomes aware of matters related to the quality, efficacy and safety of the investigational drug or other information important for the proper conduct of the clinical trial, the investigator shall revise the relevant protocol as necessary. When a revision is made, a history of the revision shall be prepared and stored.
2) The coordinating investigator shall revise the case report form as necessary in conjunction with the revision of the protocol or for other reasons. Whenever necessary, the coordinating investigator shall revise the case report form in conjunction with the revision of the protocol or for other reasons.
3) The investigator shall promptly submit the revised protocol and revised case report form to the head of the site and obtain approval from the investigational review committee via the head of the site.
4) The same procedure shall be followed when the investigator amends the protocol and case report form within the scope of the investigator's acceptable instructions from the head of the site based on the opinion of the investigational review committee.

12.2. Changes in statistical analysis plan

If the statistical analysis manager changes the contents of the statistical analysis plan, all changes shall be documented in the statistical analysis report for this clinical trial. In addition, the circumstances of any changes to the statistical analysis plan shall be recorded.

13. DISCONTINUATION, SUSPENSION, OR TERMINATION OF THE CLINICAL TRIAL

13.1. Criteria for discontinuation or suspension of the clinical trial as a whole

When the following information is obtained and it is considered difficult to continue the entire clinical trial, the coordinating investigator will consult with the principal investigator and make a decision to discontinue or suspend the entire clinical trial.

- When it becomes difficult to ensure the safety of this clinical trial due to new safety information or serious adverse event information concerning the investigational drug, etc.
- When the site has committed a serious violation of the drug GCP ordinance or a serious deviation from the clinical trial protocol and no improvement has been made.
- Other new information obtained during the conduct of the clinical trial that may necessitate discontinuation or suspension of the clinical trial.

13.2. Procedures for discontinuation or suspension of a clinical trial as a whole

If the coordinating investigator, after consultation with other investigators, decides to discontinue or suspend the entire clinical trial, he/she shall promptly notify the head of the investigational institution and the regulatory authorities in writing to that effect and the reasons in detail. In addition, the investigator shall promptly inform the subjects undergoing the clinical trial and take appropriate measures such as changing to appropriate treatment.

13.3. Procedures for discontinuation or suspension of this clinical trial at an individual clinical site

When the investigator discontinues or suspends a clinical trial, the investigator shall promptly notify the head of the site in writing to that effect and explain the details of the discontinuation or suspension in writing in detail. When notified of the discontinuation or suspension of a clinical trial, the investigator shall promptly notify in writing all investigators and regulatory authorities involved in the said clinical trial to that effect and explain the discontinuation or suspension in detail.

13.4. Termination of this clinical trial

After the completion of the clinical trial, the investigator shall notify the head of the investigational institution in writing that the clinical trial has been completed and report a summary of the results of the clinical trial in writing.

14. DATA MANAGEMENT

14.1. Data management procedure

Detailed procedures for data management shall be described in the data management plan.

14.2. Data Collection

The investigator or sub-investigator shall prepare a case report using Electronic Data Capture (EDC) that meets the requirements of 21 CFR Part 11, the Pharmaceutical GCP Ordinance, and the ER/ES guidelines. The investigator or subinvestigator shall prepare a case report using EDC. The investigator or subinvestigator shall make any changes, corrections or additions to the contents of the case report form on the EDC that generated the case report form, and record all of the changes, corrections or additions as electronic information. When a subinvestigator prepares a case report form or when a collaborator transcribes a case report form from source documents (source data), the investigator shall check the contents of the case report form before submitting it to the EDC and confirm that there are no problems. The investigator provides the final electronic case report form to the institution on an electronic medium (CD-R, etc.). The investigator shall ensure the readability and archivability of the electronic case report form.

When using the EDC system, the site should receive training on EDC and refer to the manual for details on how to input data.

14.3. Identification of documents that are directly described in the case report and should be interpreted as source documents (source data)

In this clinical trial, the following documents and others shall be considered source documents (source data).

1） Medical records, nursing records, clinical laboratory data, imaging films, and other records that form the basis for preparing case reports. Data stored in electronic medical records are also considered source documents.
2） Records of investigational drug administration
3） Documents or records related to the clinical trial that are required under the GCP ordinance for pharmaceutical products related to the clinical trial

Of the data described in the case report form, the following items shall be regarded as source documents (source data) when they are described in the case report form. However, if the data are recorded in the medical record, the medical record shall be regarded as the source documents (original data).

1） Purpose of concomitant medications/adjunctive therapy
2） Determination of the extent of adverse events, outcomes (including results at follow-up), severity, and causal relationship to the study treatment and the basis for the determination
3） Reasons for discontinuation of clinical trials by subjects
4） Comments by the investigator or subinvestigator

15. RETENTION OF SOURCE DOCUMENTS AND OTHER RECORDS

15.1. Retention of records by the clinical site

Documents or records pertaining to a clinical trial to be retained at the investigational site as stipulated in the Pharmaceutical GCP ordinance shall be retained by the hospital director until the later of the following dates.

1) The date on which five years have elapsed since the date of marketing approval for the relevant indication of the test product (if development has been discontinued, the date on which three years have elapsed since the date on which the decision to discontinue

development was made). However, for drugs that are subject to post-approval reexamination in accordance with the provisions of the "Law Concerning Quality, Efficacy and Safety Assurance of Pharmaceuticals and Medical Devices" and for which the period until the reexamination is completed exceeds five years, the date on which the reexamination is completed.

2) The date on which 3 years have elapsed since the discontinuation or termination of the clinical trial.

The investigator shall notify the clinicalsite when the records to be retained by the investigational site or the investigational review committee are no longer required to be retained.

15.2. Retention of records by principal investigators

Documents or records pertaining to clinical trials to be retained by principal investigators as stipulated in the Pharmaceutical GCP Ordinance shall be retained at a storage location deemed appropriate until the later of the following dates.

1) Three years have elapsed since the date of marketing approval for the relevant indication of the test product (if development has been discontinued, three years have elapsed since the date on which the decision to discontinue development was made).
2) The date on which 3 years have elapsed since the discontinuation or termination of the clinical trial.

16. RETENTION OF SAMPLES AND USE OF SAMPLES PROVIDED BY OTHER INSTITUTIONS

16.1. Retention of sample

Samples will be stored in the Laboratory of Gastroenterology, Graduate School of Medicine, Chiba University for a period not exceeding 20 years after completion of the clinical trial. The method of preservation shall be cryopreservation using liquid nitrogen.

16.2. Disposal of samples

If a subject withdraws consent, if a specimen is mistaken or contaminated or is strongly suspected of being mistaken or contaminated, or if the need for disposal is otherwise recognized, the anonymizing numbers, etc., will be deleted and the specimen will be disposed of.

16.3. Reuse of samples

Secondary use of samples and sample-related information (genomic or epigenomic analysis at Chiba University) may be conducted. In such cases, the ethical review and method of obtaining consent shall be in accordance with the corresponding ethical guidelines, etc.

17. SOURCE DOCUMENT VERIFICATION

The head of the investigational site and the investigator ensure that the personnel in charge of monitoring, audits and Institutinal Review Board or regulatory authorities have access to all records, including source documents. In anddtion , The head of the investigational site and the investigator confirm that the clinical trial is conducted appropriately and that the data are sufficiently reliable. The method and timing of source document verification shall be specified separately in the monitoring procedures.

18. Quality Assurance

In order to ensure that clinical trials are conducted and that data preparation, recording and reporting are appropriately conducted in compliance with the protocol and the GCP ordinances for pharmaceutical products, independent auditors from the departments related to the clinical trials, including the department in charge of monitoring, will conduct audits at the investigational

site and other sites where the clinical trials are conducted to confirm that quality control is appropriately conducted.The audit shall be conducted by an auditor independent from the departments related to the clinical trial, including the department in charge of monitoring, to confirm that quality control is appropriately implemented. Audits shall be conducted in accordance with the “Standard Operating Procedures for Audits” and “Audit Plan” separately stipulated.

19. QUALITY CONTROL FOR THIS STUDY

19.1 Training of study site personnel

The Principal Investigator will ensure that appropriate training relevant to the study is given to all of these staff, and that any new information relevant to the performance of this study is forwarded to the staff involved.

19.2. Quality control

In conducting clinical trial monitoring, the investigator shall consider priorities and develop a systematic risk-based approach. In the event of any deviation from this protocol, the investigator or subinvestigator shall follow the provisions of this protocol. The investigator or subinvestigator shall prepare the case report form in accordance with this protocol. The investigator will ensure that all data and other records in the case report form are accurate and complete. If any of the data in the case report form is inconsistent in any way with the original data, the investigator shall prepare and maintain a record explaining the reason for the inconsistency. The investigator shall designate a person who is not engaged in the relevant clinical trial at the site subject to the monitoring as a monitor and have him/her conduct the monitoring in accordance with the monitoring protocol that has been reviewed by the investigational review committee. The monitors shall confirm the following items in accordance with the monitoring protocol separately prepared.

- The human rights, safety and welfare of subjects are protected.
- The clinical trial is conducted in compliance with the Pharmaceutical GCP ordinance, the latest clinical trial protocol, and the procedure manual for the relevant clinical trial.
- To confirm that the data, etc. reported by the investigator or subinvestigator are accurate and complete and check them against the source documents and other clinical trial-related records.

The person in charge of data management shall formulate the data management plan in accordance with the separately established standard operating procedures and shall ensure the quality of the data through quality control at each stage of data handling.

20. ETHICAL CONDUCT AND GOOD CLINICAL PRACTICE(GCP)

This clinical trial will be conducted in accordance with the “Declaration of Helsinki”,the “Law Concerning Quality, Efficacy and Safety Assurance of Pharmaceuticals and Medical Devices” and the “Pharmaceutical GCP Ministerial Ordinance”. In addition, this clinical trial shall be conducted in compliance with the protocol and procedures for this clinical trial.

In selecting subjects, the investigator or subinvestigator shall carefully consider the appropriateness of requesting subjects to participate in this clinical trial based on the selection criteria and exclusion criteria from the perspective of protecting human rights, taking into consideration the subjects' health condition, symptoms, age, gender, ability to consent, degree of dependence on the investigator, and participation status in clinical trials including other clinical trials.

21. INSTITUTIONAL REVIEW BOARD(IRB)

Prior to the implementation of this clinical trial, the Institutional Review Board of the site will review the ethical, scientific and medical appropriateness of this clinical trial. This clinical trial shall be conducted after obtaining the approval of the Clinical Trial Review Committee. If the result of the deliberation by the Institutional Review Board is “Approval with modification,” the protocol or case report form, consent document, etc. shall be modified and approved based on the result of the deliberation, and then this clinical trial shall be conducted. The Institutional Review Board shall also continuously review whether or not this clinical trial is being conducted appropriately at least once a year.

22. HEALTH DAMAGE COVERAGE AND INSURANCE

If a subject suffers health problems as a result of participation in this clinical trial, the investigator will provide treatment and appropriate medical care for the subject's recovery.

As a response to liability for compensation and indemnification arising from health damage caused by this clinical trial, the investigators, subinvestigators, medical institution, Clinical Trial Coordinating Committee and other parties involved in this clinical trial will be covered by the Physician-initiated Clinical Trial Insurance.

23. COST BURDEN FOR THIS TRIAL

The investigational drug to be used in this study will be provided by AstraZeneca. Payment of the burden reduction fee and other expenses to subjects will be in accordance with the rules separately established by each investigational site.

24. TRIAL FUNDS AND CONFLICT OF INTEREST

This clinical trial will be conducted with funds provided to Chiba University Hospital by AstraZeneca. AstraZeneca personnel will not be involved in the conduct or analysis of this clinical trial when the investigators conduct the clinical trial.

In addition, prior to the deliberation of the Clinical Trial Review Committee at each site, the Conflict of Interest Management Committee will deliberate whether conflicts of interest are being properly managed, and it will be confirmed that the investigators and collaborators are not in a state of conflict of interest.

25. PROVISION OF THE INVESTIGATIONAL PRODUCTS AND INTELLECTUAL PROPERTY RIGHTS

In this study, the investigational products are provided by AstraZeneca Pharmaceuticals, Inc. (England) The ownership of the rights to any inventions, discoveries, or improvements of any nature (the “Inventions”) derived from this study will be decided in accordance with the contracts with AstraZeneca.

26.PUBLICATION

The results of the clinical trial will be submitted as a report by the Principal Investigator to the head of the clinical trial site upon completion of this clinical. Results that do not meet expected outcomes despite the proper conduct of the clinical trial must also be disclosed in publication. In case of disclosure of the results to the public, the subject’s personal information must be kept confidential.

27. REGISTRATION FOR CLINICAL TRIAL

This clinical trial will be registered in Japan Registry of Clinical Trial(jRCT) (https://jrct.niph.go.jp/) prior to obtaining consent from the first subject.

28. STUDY IMPLEMANTATION GRPUP

See ANNEX TO PROTOCOL 1

29. LIST OF INVESTIGATIONAL PRODUCT(S) FOR THIS STUDY

Investigational product	Dosage form and strength	Manufacturer
Durvalumab	*50 mg/mL solution for infusion after dilution*	MedImmune/AstraZeneca
Tremelimumab	*20 mg/mL solution for infusion after dilution*	MedImmune/AstraZeneca

30. LIST OF REFERENCES

1) Ministry of Health, Labor and Welfare 2014 Patient Survey
2) Ministry of Health, Labor and Welfare 2017 Vital Statistics
3) El Serag HB, et al., Hepatocellular Carcinoma N Engl J Med 2011
4) The Japan Society of Hepatology Clinical Practice Guidelines for Hepatocellular Carcinoma
5) Llovet JM, Ricci S, Mazzaferro V, et al. Sorafenib in advanced hepatocellular carcinoma. N Engl J Med 2008; 359: 378-90.
6) Cheng AL, Kang YK, Chen Z, et al. Efficacy and safety of sorafenib in patients in the Asia-Pacific region with advanced hepatocellular carcinoma: a phase III randmised, double- blind, placebo-controlled trial. Lancet Oncol 2009; 10:25-34.
7) Bruix J, Qin S, Merle P, et al. Regorafenib for patients with hepatocellular carcinoma who progressed on sorafenib treatment (RESORCE): a randmised, double-blind, placebo-controlled, phase 3 trial. Lancet 2017; 389: 56-66.
8) Kudo M, Finn RS, Qin S, et al. Lenvatinib versus sorafenib in first-line treatment of patients with unresectable hepatocellular carcinoma: a randomised phase 3 non-inferiority trial. Lancet 2018 Mar 24, 391 (10126): 1163-1173
9) Zhu AX, Kan YK, Yen CJ, et al. Ramucirumab after sorafenib in patients with advanced hepatocellular carcinoma and increased α-fetoprotein concentrations (REACH-2): a randomised, double-blind, placebo-controlled, phase 3 trial. Lancet Oncol.2019 Feb;20(2):282-296.
10) Bonze D, Meirson T, Azoulay D. Atezolizumab and Bevacizumab in Hepatocellular Carcinoma. NEJM. 2020 Aug 13;383(7):693-694.
11) Costentin CE, Ferroone CR, Arellano RS, et al. Hepatocellular Carcinoma with Macrovascular Invasion: Defining the Optimal Treatment Strategy Liver Cancer 2017; Nov;6(4):360-374.
12) Dunn GP, Old LJ, Schreiber RD. The three Es of cancer immunoediting. Annu Rev Immunol 2004;22:329-60.
13) Keir ME, Butte MJ, Freeman GJ, Sharpe AH. PD-1 and its ligands in tolerance and immunity. Annu Rev Immunol. 2008;26:677-704.
14) Okazaki T, Honjo T. PD-1 and PD-1 ligands: from discovery to clinical application. Int Immunol 2007;19(7):813-824.
15) Qin A, Coffey DG, Warren EH, Ramnath N. Mechanisms of immune evasion and current status of checkpoint inhibitors in non-small cell lung cancer. Cancer Med 2016;9:2567–2578.
16) Pardoll DM. The blockade of immune checkpoints in cancer immunotherapy. Nat Rev Cancer 2012;12:252-64.
17) Brahmer JR, Tykodi SS, Chow LQM, Hwu WJ, Topalian SL, Hwu P, et al. Safety and activity of

anti-PD-L1 antibody in patients with advanced cancer. N Engl J Med. 2012 Jun;366 (26):2455-65.

18) Hirano F, Kaneko K, Tamura H, Dong H, Wang S, Ichikawa M, et al. Blockade of B7-H1 and PD-1 by monoclonal antibodies potentiates cancer therapeutic immunity. Cancer Res. 2005;65(3):1089-96.

19) Iwai Y, Ishida M, Tanaka Y, Okazaki T, Honjo T, Minato N. Involvement of PD-L1 on tumor cells in the escape from host immune system and tumor immunotherapy by PD-L1 blockade. Proc Natl Acad Sci USA. 2002 Sep 17;99:12293-7.

20) Okudaira K, Hokari R, Tsuzuki Y, Okada Y, Komoto S, Watanabe C, et al. Blockade of B7-H1 or B7-DC induces an anti-tumor effect in a mouse pancreatic cancer model. Int J Oncol. 2009 Sep;35(4):741-9.

21) Topalian SL, Hodi FS, Brahmer JR, Gettinger SN, Smith DC, McDermott DF, et al. Safety, activity, and immune correlates of anti-PD-1 antibody in cancer. N Engl J Med. 2012;366:2443-54.

22) Zhang C, Wu S, Xue X, Li M, Qin X, Li W, et al. Anti-tumor immunotherapy by blockade of the PD-1/PD-L1 pathway with recombinant human PD-1-IgV. Cytotherapy. 2008;10(7):711-9.

23) Powles T, Eder JP, Fine GD, Braiteh FS, Loriot Y, Cruz C, et al. MPDL3280A (anti-PD-L1) treatment leads to clinical activity in metastatic bladder cancer. Nature. 2014 Nov 27;515(7528):558-62.

24) Rizvi N, Brahmer J, Ou S-H, Segal NH, Khleif SN, Hwu WJ. Safety and clinical activity of MEDI4736, an anti-programmed cell death-ligand-1 (PD-L1) antibody, in patients with nonsmall cell lung cancer (NSCLC). J Clin Oncol 2015;33:Abstract 8032.

25) Segal NH, Ou S-HI, Balmanoukian AS, Fury MG, Massarelli E, Brahmer JR, et al. Safety and efficacy of MEDI4736, an anti-PD-L1 antibody, in patients from a squamous cell carcinoma of the head and neck (SCCHN) expansion cohort. J Clin Oncol 2015;33:Abstract 3011.

26) Alexandrov LB, Nik-Zainal S, Wedge DC, Aparicio SAJR, Behjati S, Blankin AV, et al. Signatures of mutational processes in human cancer. Nature. 2013 Aug 22;500:415-21.

27) Fife BT, Bluestone JA. Control of peripheral T-cell tolerance and autoimmunity via the CTLA-4 and PD-1 pathways. Immunol Rev. 2008;224:166-82.

28) El-Khoueiry AB, Sangro B, Yau T, et al. Nivolumab in patients with advanced hepatocellular carcinoma (CheckMate 040): an open-label, non-comparative, phase 1/2 dose escalation and expansion trial Lancet 2017; Jun 24;389(10088):2492-2502.

29) Zhu AX, Finn RS, Edeline J, et al. Pembrolizumab in patients with advanced hepatocellular carcinoma previously treated with sorafenib (KEYNOTE-224): a non-randomised, open-label phase 2 trial. Lancet Oncol. 2018; Jul;19(7):940-952.

30) Kudo M. Targeted and immune therapies for hepatocellular carcinoma: Predictions for 2019 and beyond. World J Gastroenterol 2019 Feb 21;25(7):789-807.

31) Kelly RK, Abou-Alfa GK, Bendell JC, et al. Phase I/II study of durvalumab and tremelimumab in patients with unresectable hepatocellular carcinoma (HCC): Phase I safety and efficacy analyses. J Clin Oncol.2017; Abstract 4073.

32) Pedroni E, Bacher R, Blattmann H, Böhringer T, Coray A, Lomax A, Lin S, Munkel G, Scheib S, Schneider U, et al. The 200-MeV proton therapy project at the Paul Scherrer Institute: conceptual design and practical realization. Med Phys. 1995 Jan;22(1):37-53.

33) Mohamad O., Makishima H., Kamada T. Evolution of Carbon Ion Radiotherapy at the National Institute of Radiological Sciences in Japan. Cancers (Basel) 2018;10(3): pii: E66

34) Kasuya G, Kato H, Yasuda S, Tsuji H, Yamada S, Haruyama Y, Kobashi G, Ebner DK, Okada NN, Makishima H, Miyazaki M, Kamada T, Tsujii H; Liver Cancer Working Group. Progressive

hypofractionated carbon-ion radiotherapy for hepatocellular carcinoma: Combined analyses of 2 prospective trials. Cancer. 2017;123(20):3955-3965

35) Fukuda K, Okumura T, Abei M, Fukumitsu N, Ishige K, Mizumoto M et al. Long-term outcomes of proton beam therapy in patients with previously untreated hepatocellular carcinoma. Cancer Sci. 2017;108(3):497-503

36) Sorin Y, Ikeda K, Kawamura Y, Fujiyama S, Kobayashi M, Hosaka T. Effectiveness of Particle Radiotherapy in Various Stages of Hepatocellular Carcinoma: A Pilot Study. Liver Cancer. 2018 Oct;7(4):323-334

37) Igaki H, Mizumoto M, Okumura T, Hasegawa K, Kokudo N, Sakurai H. A systematic review of publications on charged particle therapy for hepatocellular carcinoma. Int J Clin Oncol. 2018;23(3):423-433

38) Komatsu S, Fukumoto T, Demizu Y, Miyawaki D, Terashima K, Niwa Y, Mima M, Fujii O, Sasaki R, Yamada I, Hori Y, Hishikawa Y, Abe M, Ku Y, Murakami M. The effectiveness of particle radiotherapy for hepatocellular carcinoma associated with inferior vena cava tumor thrombus. J Gastroenterol. 2011 Jul;46(7):913-20.

39) Lee SU, Park JW, Kim TH, Kim YJ, Woo SM, Koh YH, Lee WJ, Park SJ, Kim DY, Kim CM. Effectiveness and safety of proton beam therapy for advanced hepatocellular carcinoma with portal vein tumor thrombosis. Strahlenther Onkol. 2014 Sep;190(9):806-14.

40) Sugahara S, Nakayama H, Fukuda K, Mizumoto M, Tokita M, Abei M, Shoda J, Matsuzaki Y, Thono E, Tsuboi K, Tokuuye K. Proton-beam therapy for hepatocellular carcinoma associated with portal vein tumor thrombosis. Strahlenther Onkol 2009;185:782–788.

41) Antonia SJ, Daniel VD, Vicente D, et al. Durvalumab after Chemoradiotherapy in Stage III Non-Small-Cell Lung Cancer. N Engl J Med. 377;1919-29. 2017

42) Rizvi NA, Cho BC, Reinmuth N, et al. Durvalumab with or without tremelimumab vs platinum-based chemotherapy as first-line treatment for metastatic non-small cell lung cancer: MYSTIC Annals of Oncology.2018 Dec 1;29;supp10

43) Helm A, Ebner DK, Tinganelli W, Simoniello P, Bisio A, Marchesano V, Durante M, Yamada S, Shimokawa T. Combining Heavy-Ion Therapy with Immunotherapy: An Update on Recent Developments. Int J Part Ther. 2018 Summer;5(1):84-93.

44) Takahashi Y, Yasui T, Minami K, Tamari K, Hayashi K, Otani K, Seo Y, Isohashi F, Koizumi M, Ogawa K. Carbon ion irradiation enhances the antitumor efficacy of dual immune checkpoint blockade therapy both for local and distant sites in murine osteosarcoma. Oncotarget. 2019 Jan 18;10(6):633-646.

45) Iijima M, Okonogi N,Izumi Nakajima N, Morokoshi Y, Kanda H, Yamada T, Kobayashi Y, Banno K, Wakatsuki M, Yamada S, Kamada T, Aoki D, Hasegawa S. Significance of PD-L1 expression in carbon-ion radiotherapy for uterine cervical adeno/adenosquamous carcinoma. J Gynecol Oncol. 2020;31:e19.

46) Golden EB, Pellicciotta I, Demaria S, et al. The convergence of radiation and immunogenic cell death signaling pathways. Front Oncol, 2012 Aug 7;2:88.

47) Onishi M, Okonogi N, Oike T, Yoshimoto Y, Sato H, Suzuki Y, Kamada T, Nakano T. High linear energy transfer carbon-ion irradiation increases the release of the immune mediator high mobility group box 1 from human cancer cells. J Radiat Res. 2018 Sep 1;59(5):541-546.

48) Matsunaga A, Ueda Y, Yamada S, Harada Y, Shimada H, Hasegawa M, Tsujii H, Ochiai T, Yonemitsu Y. Carbon-ion beam treatment induces systemic antitumor immunity against murine squamous cell carcinoma. Cancer. 2010 Aug 1;116(15):3740-8.

49) Ando K, Fujita H, Hosoi A, Ma L, Wakatsuki M, Seino KI, Kakimi K, Imai T, Shimokawa T,

Nakano T. Intravenous dendritic cell administration enhances suppression of lung metastasis induced by carbon-ion irradiation. J Radiat Res. 2017 Jul 1;58(4):446-455.

50) Sato H, Niimi A, Yasuhara T, Permata TBM, Hagiwara Y, Isono M, Nuryadi E, Sekine R, Oike T, Kakoti S, Yoshimoto Y, Held KD, Suzuki Y, Kono K, Miyagawa K, Nakano T, Shibata A. DNA double-strand break repair pathway regulates PD-L1 expression in cancer cells. Nat Commun. 2017 Nov 24;8(1):1751. doi: 10.1038/s41467-017-01883-9.

51) Oike T, Niimi A, Okonogi N, Murata K, Matsumura A, Noda SE, Kobayashi D, Iwanaga M, Tsuchida K, Kanai T, Ohno T, Shibata A, Nakano T. Visualization of complex DNA double-strand breaks in a tumor treated with carbon ion radiotherapy. Sci Rep. 2016 Mar 1;6:22275. doi: 10.1038/srep22275.

52) Stewart R, Morrow M, Hammond SA, Mulgrew K, Marcus D, Poon E, et al. Identification and characterization of MEDI4736, an antagonistic anti-PD-L1 monoclonal antibody. Cancer Immunol Res 2015;3(9):1052-62.

53) Tarhini AA, Kirkwood JM. Tremelimumab (CP-675,206): a fully human anticytotoxic T lymphocyte-associated antigen 4 monoclonal antibody for treatment of patients with advanced cancers. Expert Opin Biol Ther 2008;8:1583-93.

54) Pardee AD, Butterfield LH. Immunotherapy of hepatocellular carcinoma: Unique challenges and clinical opportunities. Oncoimmunology 2012;1(1):48-55.

55) Gao Q, Wang XY, Qiu SJ, Yamato I, Sho M, Nakajima Y, et al. Overexpression of PD-L1 significantly associates with tumor aggressiveness and postoperative recurrence in human hepatocellular carcinoma. Clin Cancer Res 2009;15(3):971-9.

56) Hato T, Goyal L, Greten TF, Duda DG, Zhu AX. Immune checkpoint blockade in hepatocellular carcinoma: current progress and future directions. Hepatology 2014;60(5):1776-82.

57) Miroux C, Vausselin T, Delhem N. Regulatory T cells in HBV and HCV liver diseases: implication of regulatory T lymphocytes in the control of immune response. Expert Opin Biol Ther 2010;10(11):1563-72.

58) Golden-Mason L, Palmer B, Klarquist J, Mengshol JA, Castelblanco N, Rosen HR. Upregulation of PD-1 expression on circulating and intrahepatic hepatitis C virus-specific CD8+ T cells associated with reversible immune dysfunction. J Virol 2007;81(17):9249-58.

59) Peng G, Li S, Wu W, Tan X, Chen Y, Chen Z. PD-1 upregulation is associated with HBV-specific T cell dysfunction in chronic hepatitis B patients. Mol Immunol 2008;45(4):963-70.

60) Klein JP, Logan B, Harhoff M, Andersen PK. Analyzing survival curves at a fixed point in time. Stat Med 2007;26(24):4505-19.

61) Sangro B, Gomez MC, Mata M, et al. A clinical trial of CTLA-4 blockade with tremelimumab in patients with hepatocellular carcinoma and chronic hepatitis Cq. J Hepatol. 2013 Jul;59(1):81-8.

62) Duffy AG, Ulahannan SV, Makorova-Rusher O, Rahmer O, Wedemeyer H, Pratt D, et al. Tremelimumab in combination with ablation in patients with advanced hepatocellular carcinoma. J Hepatol 2017;66(3):545-51.

63) Melero I, Sangro B, Cheung Yae T, Hsu C, Kudo M, Crocenzi TS, et al. Nivolumab dose escalation and expansion in patients with advanced hepatocellular carcinoma (HCC): The CheckMate 040 study. J Clin Oncol 2017;35 (suppl 4S:abstract 226).

64) Kelly RK, Sangro B, Harris W, et al. Efficacy, tolerability, and biologic activity of a novel regimen of tremelimumab in combination with durvalumab for patients with advanced hepatocellular carcinoma, J Clin Oncol. 2020; Abstract 4508.

65) Takahashi Y, Fujikawa K, Sagawa T, et al. A phase 1 study to assess the safety and tolerability of tremelimumab alone and in combination with MEDI4736 in Japanese patients withadvanced solid

malignancies. Eur J Cancer. 2015(suppl 3; abst512)

66) Wang E, Kang D, Bae KS, Marshall MA, Pavlov D, Parivar K. Population pharmacokinetic and pharmacodynamics analysis of tremelimumab in patients with metastatic melanoma. J Clin Pharmacol 2014;54(10):1108-16.

67) Narwal R, Roskos LK, Robbie GJ. Population pharmacokinetics of sifalimumab, an investigational anti-interferonalpha monoclonal antibody, in systemic lupus erythematosus. Clin Pharmacokinet 2013;52:1021–27.

68) Ng CM, Lum BL, Gimenez V, Kelsey S, Allison D. Rationale for fixed dosing of pertuzumab in cancer patients based on population pharmacokinetic analysis. Pharm Res 2006;23(6):1275–84.

69) Wang DD, Zhang S, Zhao H, Men AY, Parivar K. Fixed dosing versus body size based dosing of monoclonal antibodies in adult clinical trials. J Clin Pharmacol 2009;49(9):1012–24.

70) Wolchok JD, Kluger H, Callahan MK, Postow MA, Rizvi NA, Lesokhin AM, et al. Nivolumab plus ipilimumab in advanced melanoma. N Engl J Med 2013;369:122-33.

71) Reed GB Jr, Cox AJ Jr. The human liver after radiation injury. A form of veno-occlusive disease. Am J Pathol. 1966 Apr;48(4):597-611.

72) Kanai T, Endo M, Minohara S, Miyahara N, Koyama-ito H, Tomura H, Matsufuji N, Futami Y, Fukumura A, Hiraoka T, Furusawa Y, Ando K, Suzuki M, Soga F, Kawachi K. Biophysical characteristics of HIMAC clinical irradiation system for heavy-ion radiation therapy. Int J Radiat Oncol Biol Phys. 1999 Apr 1;44(1):201-10.

73) Shibuya K, Ohno T, Terashima K, Toyama S, Yasuda S, Tsuji H, et al. Short-course carbon-ion radiotherapy for hepatocellular carcinoma: a multi-institutional retrospective study. Liver Int. 2018;38(12):2239-47.

74) Yasuda S, Kato H, Imada H, et al. Long-Term Results of High-Dose 2-Fraction Carbon Ion Radiation Therapy for Hepatocellular Carcinoma. Adv Radiat Oncol. 2019 Sep 27;5(2):196-203.

75) Shiba S, Shibuya K, Katoh H, et al. A comparison of carbon ion radiotherapy and transarterial chemoembolization treatment outcomes for single hepatocellular carcinoma: a propensity score matching study Radiat Oncol. 2019 Aug 2;14(1):137.

76) Shiba S, Shibuya K, Okamoto M, et al. Clinical impact of Hypofractionated carbon ion radiotherapy on locally advanced hepatocellular carcinoma. Radiat Oncol 2020 Aug 14;15(1):195.

77) Makishima S, Yasuda S, Isozaki Y, et al. Single fraction carbon ion radiotherapy for colorectal cancer liver metastasis: A dose escalation study. Cancer Sci. 2019 Jan;110(1):303-309.

78) Ebner DK, Tinganelli W, Helm A, Bisio A, Yamada S, Kamada T, Shimokawa T, Durante M. The immunoregulatory potential of particle radiation in cancer therapy. Front Immunol. 2017;8:1–8.

Clinical Study Protocol

A phase Ib study of durvalumab (MEDI4736) ± tremelimumab combined with Carbon ion radiotherapy in advanced hepatocellular carcinoma patients with macrovascular invasion

Study Number: CCRC2002
Investigational drugs : Durvalumab, Tremelimumab（Concominant therapy : Carbon ion radiotherapy）
Version Number 2.0
Date 24 May 2022

Revision history

Date	Version Number
24 Feb 2021	1.0
26 Mar 2021	1.1
20 Apl 2021	1.2
24 May 2022	2.0

This protocol includes information and data that contain privileged or confidential information and, is provided only to the investigators, clinical team staff, associates, IRBs, or the Data Monitoring Committee. Therefore, this information must not be made public without written permission from the Chief Investigator, and AstraZeneca except when explaining to subjects. These restrictions on disclosure will apply equally to all or part of the data obtained in the clinical trial for publishing or presentation.

＜Abbreviation and terms＞

Definitions of abbreviations, acronyms, and terms in this study protocol are as follows

Abbreviation /terms	Definition
AE	adverse event
AESI	adverse event of special interest
AFP	α-fetoprotein
AFP-L3	α-fetoprotein - L3
ALP	alkaline phosphatase
ALT	alanine aminotransferase
AMED	Japan Agency for Medical Research and Development
APTT	activated partial thromboplastin time
AST	aspartate aminotransferase
AUC	area under the blood concentration time curve
BICR	blinded independent central review
BP	blood pressure
BSA	body surface area
CD	cluster of differentiation
CI	confidence interval
CIRT	carbon-ion radiotherapy
Cmax	maximum observed concentration
Cmin	minimum concentration during a dosing interval
COI	conflicts of interest
CRP	C-reactive protein
CT	computed tomography
Ctrough	drug concentration at the end of the dosing interval
CTCAE	common terminology criteria for adverse events
CTLA-4	cytotoxic T lymphocyte antigen 4
CTV	clinical target volume
DAMP	damage-associated molecular pattern
DC	dendritic cell
DLT	dose limiting toxicity
DMC	data monitoring committee
DNA	deoxyribonucleic acid
ECG	electrocardiogram
ECOG	Eastern Cooperative Oncology Group
eCRF	electronic case report form
EDC	electronic data capture
EGFR	epidermal growth factor receptor
ER	electronic record
ES	electronic signature
FAS	full analysis set
FTIH	first-time-in-human
FU	follow-up
G-CSF	granulocyte-colony stimulating factor
GCP	Good Clinical Practice
GI	gastrointestinal
HAIC	hepatic arterial infusion chemotherapy

Abbreviation /terms	Definition
HBc	hepatitis B core
HBs	hepatitis B surface
HBV	hepatitis B virus
HCC	hepatocellular carcinoma
HCV	hepatitis C virus
HIMAC	Heavy Ion Medical Accelerator in Chiba
HIV	human immunodeficiency virus
HMGB1	high mobility group box protein1
HR	hazard ratio
HR	heart rate
HRQoL	health-related quality of life
ICH	International Council for Harmonisation of Technical Requirements for Pharmaceuticals for Human Use
ICI	immune checkpoint inhibitor
IFN-γ	interferon-γ
Ig	immunoglobulin
IL	interleukin
ILD	interstitial lung disease
imAE	immune mediated adverse event
IR	investigational regimen
IRB	institutional review board
KL-6	Krebs von den Lungen-6
LDH	lactate dehydrogenase
LET	linear energy transfer
LFT	liver function test
mAb	monoclonal antibody
MedDRA	Medical Dictionary for Regulatory Activities
MOA	mechanism of action
mRECIST	modified RECIST
MRI	magnetic resonance imaging
MST	mean survival time
MTD	maximum tolerated dose
MVI	macroscopic vascular invasion
NIRS	National Institute of Radiological Sciences
NSCLC	non-small cell lung cancer
NTL	non-target lesion
NYHA	New York Heart Association
OAR	off-axis ratio
ORR	objective response rate
OS	overall survival
PD	progressive disease
PD-1	programmed cell death 1
PD-L1	programmed cell death ligand 1
PD-L2	programmed cell death ligand 2
PIVKA-II	protein induced by vitamin K absence-II
PK	Pharmacokinetics

Abbreviation /terms	Definition
PMDA	Pharmaceuticals and Medical Devices Agency
PPS	per protocol set
PR	partial response
PRO	patient reported outcome
PS	performance status
PT	preferred term
PT-INR	prothrombin time-international normalized ratio
PTV	planning target volume
QxD	quaque x day
QxW	quaque x week
QST	National Institutes for Quantum and Radiological Science and Technology
QTcF	corrected QT interval using Fridericia's formula
Q2W	quaque 2 weeks
Q3W	quaque 3 weeks
Q4W	quaque 4 weeks
RBE	relative biological effectiveness
RECIST	Response Evaluation Criteria In Solid Tumors
RESORCE	Regorafenib after Sorafenib in Patients with Hepatocellular Carcinoma
RFA	radiofrequency ablation
RILD	radiation induced liver damage
RNA	ribonucleic acid
SAE	serious adverse event
SD	stable disease
SHARP	Sorafenib Hepatocellular Carcinoma Assessment Randomized Protocol
SOP	standard operating procedure
SP-D	surfactant protein-D
SpO2	saturation of peripheral oxygen
TACE	transcatheter arterial chemoembolization
TAI	transcatheter arterial infusion
TBL	total bilirubin
TKI	tyrosine kinase inhibitor
TL	target lesion
TLR4	Toll-like receptor 4
TMGs	Toxicity Management Guidelines
TSH	thyroid stimulating hormone
TTP	time to progression
ULN	upper limit of normal
VEGF	vascular endothelial growth factor

TABLE OF CONTENTS

0. CLINICAL TRIAL OVERVIEW........9
1. INTRODUCTION........16
1.1. Introduction........16
1.2. Standard Treatment for Advanced Hepatocellular Carcinoma........16
1.3. HCC with vascular invasion and its treatment........17
1.4. Immunotherapy........17
1.5. Carbon ion radiotherapy........18
1.6. Induction of Immunogenicity by Radiotherapy........18
1.7. Combination of immune checkpoint inhibitors and carbon ion radiotherapy........19
1.8. Investigational drugs........19
1.8.1. Durvalumab........19
1.8.2. Tremelimumab........20
1.8.3. Durvalumab + tremelimumab combination therapy........20
1.8.4. Rationale for Durvalumab and Tremelimumab as Treatment Options for HCC 20
1.9. hypothesis........21
2. STUDTY OBJECTIVE........23
2.1. Objective........23
2.2. Study results regarding the appropriateness of conducting this clinical trial, efficacy, and safety for the subject disease........23
2.2.1. Durvalumab + tremelimumab combination therapy dose rationale........23
2.2.2. Dose rationale for combination regimen of durvalumab 1500 mg Q4W plus tremelimumab 300 mg × 1 dose........23
2.2.2.1. Rationale for utilizing a fixed-dose regimen for durvalumab and tremelimumab........24
2.2.3 Rationale for carbon-ion radiotherapy........24
2.2.4 Rationale for combined treatment of carbon-ion radiotherapy and immunotherapy........25
2.3. Benefit-risk and ethical assessment........25
2.3.1. Potential benefits........25
2.3.2. Overall risks........25
2.3.3. Overall benefit-risk........28
3. ELIGIBILITY........29
3.1. Diagnostic Criteria and Stage, Type, and Condition Classification........29
3.2. Inclusion criteria........29
3.3. Exclusion criteria........30
4. INFORMED CONSENT........33
4.1. Preparation and revision of informed consent form........33
4.2. Method of Obtaining Informed Consent........33
4.3. Information to be provided to subjects........34
5. STUDY DESIGN........35
5.1. Overview of study design........35
5.2. Target number of subjects and study duration........38
5.3. Monitoring for safety assessment........38
5.4. Institutional and case registration methods........39
5.4.1. Facility registration........39
5.4.2. Subject resistration........39
5.4.3. Handling of subjects who are found to be ineligible........40
5.5. Dosing schedule and dosage/administration method........40
5.5.1. Criterion for reduction........40
5.5.2. Criterion for drug withdrawal........41
5.6. Discontinuation of Investigational Drug........41
5.7. Discontinuation of individual cases from participation in clinical trials........41

5.7.1. In case of untraceable cases 42
5.7.2. Withdrawal of consent 42
5.7.3. Clinical investigator's decision 42
5.7.4. Subject weight loss 42
5.7.5. Other cases 42
5.8. Subject replacement 42
5.9. Concomitant Restricted Drugs and Concomitant Restricted Therapy 43
5.10. Follow-up treatment 44
5.11. After discontinuation of this clinical trial 44
6. CLINICAL TRIAL TREATMENT 45
6.1. Durvalumab and tremelimumab 45
6.1.1. Durvalumab 45
6.2. Control Drugs 46
6.3. Monitoring during administration 46
6.4. Management of investigational drugs 46
6.5. Disposal of unused investigational drugs 47
6.6. Packaging and labeling of investigational drugs 47
6.7. Carbon ion radiotherapy 47
6.8. subject inclusion 48
6.9. Definition of Dose-Limiting Toxicity (DLT) 49
6.10. Toxicity Management 51
6.11. Restrictions during the clinical trial 51
6.11.1. Restrictions during the clinical trial 51
6.12. Clinical Trial Procedures 52
7. OBSERVATION, EXAMINATION, AND ASSESSMENT, METHODS, AND TIMING OF IMPLEMENTATION 53
7.1. Implementation Schedule and Procedures 53
7.2. Observation, tests and assessment 56
7.2.1. Screening period 56
7.2.2. DLT assessment period 57
7.2.3. Durvalumab q4W dosing period 59
7.2.4. At the time of discontinuation of investigational drug administration 60
7.2.5. Follow up period 60
7.3. Biological sampling procedures 60
7.3.1. Guideline for blood sampling volume 60
7.3.2. Blood samples for archiving 61
7.3.3. Hepatic tumor biopsy sample 61
7.4. Asessment of efficiency 61
7.5. Assessment of safety 62
7.5.1. Clinical laboratory tests 62
7.5.2. Physical examinations 64
7.5.3. Electrocardiogram (ECG) 64
7.5.4. Vital signs 64
7.5.5. ECOG performance status 65
7.5.6. Other safety assesements 65
8. HANDLING OF ADVERSE EVENT 65
8.1. Definition 65
8.1.1. Adverse event 65
8.1.2. Severe adverse event 66
8.1.3. Adverse Events of Special Interest (AESI) 66
8.1.4. Confirmation of interstitial lung disease (ILD) 67
8.2. Assessment of severity 68
8.3. Record of adverse events and serious adverse events 68
8.4. Duration of recording and follow-up of adverse events and serious adverse events 69

8.5. Causal relationship with investigational therapy 69
8.6. Outcome definition 70
8.7. Treatment of investigational drug in the event of an adverse event 70
8.8. Treatment of heavy particle therapy equipment in the event of an adverse event 70
8.9. Relationship to Protocol Procedures 70
8.10. Adverse events based on signs and symptoms 71
8.11. Adverse events based on tests and examinations 71
8.12. Hy's Law 71
8.13. Disease progression 71
8.14. New cancer 71
8.15. Deaths 71
8.16. Reportable Adverse Events 72
8.17. Reporting of serious adverse events 72
8.17.1. Response to subjects 73
8.18. OTHER EVENTS REQUIRING REPORTING 73
8.18.1. Overdose 74
8.18.2. Hepatic function abnormality 74
8.18.3. Pregnancy 74
8.18.4. Exposure to pregnant woman 74
8.18.5. Exposure to partner 74
8.19. Medication error 75
8.20. Predicted Side Effects 75
9. ENDPOINT 78
9.1. Primary endpoint 78
9.2. Secondary endpoint 78
9.3. Exploratory endpoints 78
10. STATISTICAL METHODS AND SAMPLE SIZE DETERMINATION 78
10.1. Description of Analysis set 78
10.1.1. DLT analysis set 78
10.1.2. Safety analysis set: SAF 78
10.1.3. Full Analysis set: FAS 78
10.1.4 Efficacy Evaluable set: EES 78
10.1.5 Per protocol set ： PPS 79
10.2. Target number of cases and rationale for setting 79
10.3. Case Handling 79
10.5.1. Analysis of subject background 79
10.5.2. Analysis of primary endpoints 79
10.5.2.1. DLT Evaluation 79
10.5.2.2. Safety Evaluation 80
11. COMPLIANCE AND DEVIATION FROM THE PROTOCOL 81
12. CHANGES TO THE CLINICAL TRIAL PROTOCOL, CASE REPORT FORM, OR ANALYSIS PLAN 81
13. DISCONTINUATION, SUSPENSION, OR TERMINATION OF THE CLINICAL TRIAL 82
14. DATA MANAGEMENT 83
15. RETENTION OF SOURCE DOCUMENTS AND OTHER RECORDS 83
16. RETENTION OF SAMPLES AND USE OF SAMPLES PROVIDED BY OTHER INSTITUTIONS 84
17. SOURCE DOCUMENT VERIFICATION 84
18. QUALITY ASSURANCE 84
19. QUALITY CONTROL FOR THIS STUDY 85
20. ETHICAL CONDUCT AND GOOD CLINICAL PRACTICE(GCP) 85
21. INSTITUTIONAL REVIEW BOARD(IRB) 85
22. HEALTH DAMAGE COVERAGE AND INSURANCE 86

23. COST BURDEN FOR THIS TRIAL 86
24. TRIAL FUNDS AND CONFLICT OF INTEREST 86
25. PROVISION OF THE INVESTIGATIONAL PRODUCTS AND INTELLECTUAL PROPERTY RIGHTS 86
26.PUBLICATION 86
27. REGISTRATION FOR CLINICAL TRIAL 86
28. STUDY IMPLEMANTATION GRPUP 87
29. LIST OF INVESTIGATIONAL PRODUCT(S) FOR THIS STUDY 87
30. LIST OF REFERENCES 87

0. Clinical Trial Overview

<table>
<tr><td>Title</td><td>A phase Ib study of durvalumab (MEDI4736) ± tremelimumab combined with particle therapy in advanced hepatocellular carcinoma patients with macrovascular invasion</td></tr>
<tr><td>Objective</td><td>Objectives:

Primary Objective:
To assess the safety and tolerability of combination therapy of durvalumab ± tremelimumab with particle therapy in advanced hepatocellular carcinoma patients with macrovascular invasion

Secondary Objectives:
To assess the efficacy of combination therapy of durvalumab ± tremelimumab with particle therapy in advanced hepatocellular carcinoma patients with macrovascular invasion</td></tr>
<tr><td>Design</td><td>Ib Phase, interventional, open-label, single arm
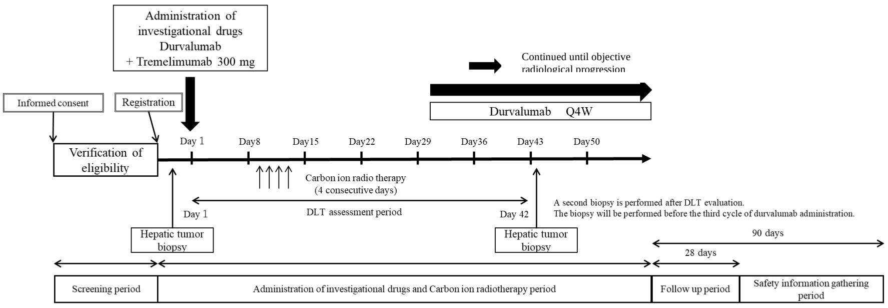
</td></tr>
<tr><td>Phase</td><td>Ib Phase</td></tr>
<tr><td>Investigational Products and Combination Therapy</td><td>Investigational Products and Combination Therapy:

Investigational Products: Durvalumab and Tremelimumab
Durvalumab (MEDI4736) solution for infusion after dilution will be supplied in glass vials containing 500 mg durvalumab at a concentration of 50 mg/mL.

Tremelimumab solution for infusion after dilution will be supplied in glass vials containing 400 mg or 25 mg tremelimumab at a concentration of 20 mg/mL.

Combination Therapy

Carbon-Ion Radiation Therapy (CIRT) as particle therapy by Toshiba, Carbon ion radio therapy CI-1000S (PMDA approval no. 22800BZX00096000).</td></tr>
<tr><td>Inclusion Criteria</td><td>1) Capable of giving signed informed consent which includes compliance with the requirements and restrictions listed in the informed consent form (ICF) and in this protocol. Written informed consent and any locally required authorization obtained from the patient/legal representative prior to performing any protocol-related procedures, including screening</td></tr>
</table>

evaluations. For patients aged <20 years and enrolling, a written informed consent should be obtained from the patient and his or her legally acceptable representative.

2) Age >20 years at time of study entry
3) Eastern Cooperative Oncology Group (ECOG) performance status of 0 or 1
4) Body weight >30 kg
5) Adequate normal organ and marrow function as defined below:
   - Haemoglobin ≧9.0 g/dL
   - Absolute neutrophil count (ANC) ≧1,500/mm^3
   - Platelet count ≧75×10^9/L（≧75,000/mm^3）
   - Serum bilirubin ≦ULN×3.0
   - AST ≦ULN×5.0
   - ALT ≦ULN×5.0
   - Measured creatinine clearance (CL) >40 mL/min or Calculated creatinine clearance CL>40 mL/min by the Cockcroft-Gault formula (Cockcroft and Gault 1976) or by 24-hour urine collection for determination of creatinine clearance
6) Evidence of post-menopausal status or negative urinary or serum pregnancy test for female pre-menopausal patients. Women will be considered post-menopausal if they have been amenorrheic for 12 months without an alternative medical cause. The following age-specific requirements apply:
   - Women <50 years of age would be considered post-menopausal if they have been amenorrheic for 12 months or more following cessation of exogenous hormonal treatments and if they have luteinizing hormone and follicle-stimulating hormone levels in the post-menopausal range for the institution or underwent surgical sterilization (bilateral oophorectomy or hysterectomy).

   - Women ≥50 years of age would be considered post-menopausal if they have been amenorrheic for 12 months or more following cessation of all exogenous hormonal treatments, had radiation-induced menopause with last menses >1 year ago, had chemotherapy-induced menopause with last menses >1 year ago, or underwent surgical sterilization (bilateral oophorectomy, bilateral salpingectomy or hysterectomy).

7) Patient is willing and able to comply with the protocol for the duration of the study including undergoing treatment and scheduled visits and examinations including follow up.
8) Advanced HCC confirmed histologically or by the typical findings of a hypervascular tumor on computed tomography or angiography
9) (Cohort A and Cohort B only) Patients who have received at least one prior systemic chemotherapy regimen including atezolizumab bevacizumab combination, sorafenib, or lenvatinib and who are determined to be refractory or intolerant to standard therapy.
10) Must not be eligible for locoregional therapy for unresectable HCC. For patients who progressed after locoregional therapy for HCC, locoregional therapy must have been completed ≥28 days prior to the baseline scan for the current study. Acceptable locoregional therapy for HCC are Ethanol Infusion Therapy, Radio Wave ablation Therapy, Transcatheter Arterial chemoembolization (TACE), Transcatheter arterial infusion (TAI). Hepatic Arterial Infusion Chemotherapy (HAIC) is not treated as a locoregional therapy.

<table>
<tr><td></td><td>11) Patients who have been diagnosed with HCC showing MVI. MVI is defined as a tumor thrombus in the major hepatic and/or portal vein branches (Vp2, Vp3, Vp4, Vv2, and Vv3) identified by imaging studies.
12) Child-Pugh A
13) At least one measurable lesion other than the MVI and feeding nodule based on mRECIST.</td></tr>
<tr><td>Exclusion Criteria</td><td>1. Involvement in the planning and/or conduct of the study (applies to both sponser and/or staff at the study site)
2. Patients who have participated in another clinical trial using the investigational drug within 28 days prior to obtaining consent or who have received another investigational drug within 28 days prior to the first dose of the investigational drug in this study. The exception is if the patient is in the follow-up period of an interventional trial or is participating in an observational (non-interventional) clinical trial.
3. Any unresolved toxicity NCI CTCAE Grade ≥2 from previous anticancer therapy with the exception of alopecia, vitiligo, and the laboratory values defined in the inclusion criteria
− Patients with Grade ≥2 neuropathy will be evaluated on a case-by-case basis after consultation with the Study Physician.
− Patients with irreversible toxicity not reasonably expected to be exacerbated by treatment with durvalumab or tremelimumab may be included only after consultation with the Study Physician.
4. Radiotherapy treatment to more than 30% of the bone marrow or with a wide field of radiation within 4 weeks of the first dose of study drug
5. Major surgical procedure (as defined by the Investigator) within 28 days prior to the first dose of IP. Note: Local surgery of isolated lesions for palliative intent is acceptable.
6. History of allogenic organ transplantation.
7. Active or prior documented autoimmune or inflammatory disorders (including inflammatory bowel disease [e.g., colitis or Crohn's disease], diverticulitis [with the exception of diverticulosis], systemic lupus erythematosus, Sarcoidosis syndrome, or Wegener syndrome [granulomatosis with polyangiitis, Graves' disease, rheumatoid arthritis, hypophysitis, uveitis, etc.]). The following are exceptions to this criterion:
– Patients with vitiligo or alopecia
– Patients with hypothyroidism (e.g., following Hashimoto syndrome) stable on hormone replacement
– Any chronic skin condition that does not require systemic therapy
– Patients without active disease in the last 5 years may be included but only after consultation with the study physician
– Patients with celiac disease controlled by diet alone
8. Uncontrolled intercurrent illness, including but not limited to, ongoing or active infection, symptomatic congestive heart failure, uncontrolled hypertension, unstable angina pectoris, cardiac arrhythmia, interstitial lung disease, serious chronic gastrointestinal conditions associated with diarrhea, or psychiatric illness/social situations that would limit compliance with study requirement, substantially increase risk of incurring AEs or compromise the ability of the patient to give written informed consent
9. History of another primary malignancy except for
– Malignancy treated with curative intent and with no known active disease ≥5 years before the first dose of IP and of low potential risk for recurrence</td></tr>
</table>

– Adequately treated non-melanoma skin cancer or lentigo maligna without evidence of disease
– Adequately treated carcinoma in situ without evidence of disease
However, the following cases are eligible for enrollment
Early stage cancer (epithelial cancer of the cervix, basal cell carcinoma, superficial bladder cancer (Tis and T1), early stage gastric cancer, and early stage colorectal cancer) that has been treated for curative purposes, has not been confirmed active for at least 3 years prior to inclusion in the study, and has a low risk of recurrence.

10. History of leptomeningeal carcinomatosis
11. History of, or current, brain metastases or spinal cord compression. Patients with suspected brain metastases at screening should have an MRI (preferred) or CT, each preferably with IV contrast of the brain prior to study entry.
12. Mean QT interval corrected for heart rate using Fridericia's formula (QTcF) ≥470 ms calculated from 3 ECGs (within 15 minutes at 5 minutes apart) Regardless of whether this criteria stays or not, all patients should have a baseline ECG
13. History of active primary immunodeficiency
14. Patients co-infected with HBV and HCV. HBV positive (presence of HBsAg and/or anti-HBcAb with detectable HBV DNA); HCV positive (presence of anti-HCV antibodies), and active infection including tuberculosis (clinical evaluation that includes clinical history, physical examination and radiographic findings, and TB testing in line with local practice).
15. Current or prior use of immunosuppressive medication within 14 days before the first dose of durvalumab or tremelimumab. The following are exceptions to this criterion:
– Intranasal, inhaled, topical steroids, or local steroid injections (e.g., intra articular injection)
– Systemic corticosteroids at physiologic doses not to exceed 10 mg/day of prednisone or its equivalent
– Steroids as premedication for hypersensitivity reactions (e.g., CT scan premedication)

16. Receipt of live attenuated vaccine within 30 days prior to the first dose of IP. Note: Patients, if enrolled, should not receive live vaccine whilst receiving IP and up to 30 days after the last dose of IP.
17. Female patients who are pregnant or breastfeeding or male or female patients of reproductive potential who are not willing to employ effective birth control from screening to 90 days after the last dose of durvalumab monotherapy or180 days after the last dose of durvalumab + tremelimumab combination therapy.
18. Known allergy or hypersensitivity to any of the study drugs or any of the study drug excipients.
19. Prior randomisation or treatment in a previous durvalumab and/or tremelimumab clinical study regardless of treatment arm assignment.
20. Judgment by the investigator that the patient is unsuitable to participate in the study and the patient is unlikely to comply with study procedures, restrictions and requirements.
21. Patients who have been treated with anti-PD-1, anti-PD-L1 inhibitors, or other drugs that act on other stimulatory or co-suppressive T-cell receptors and their combinations (including atezolizumab plus bevacizumab) and

	have failed to tolerate the same treatment.<br>22. Prior radiotherapy involving the liver.<br>23. Renal failure requiring hemodialysis or peritoneal dialysis<br>24. Any of the following cardiac diseases:<br>– NYHA Class III or IV chronic heart failure<br>– Current coronary artery disease or history of ischemic heart disease such as myocardial infarction within 6 months before the study<br>– Serious arrhythmia (grade 3 or higher according to the CTCAE ver. 5.0: arrhythmia that cannot be controlled by oral medications or requires mechanical control).<br>25. Poorly controlled hypertension<br>26. Serious and active infection, excluding hepatitis viral infection<br>27. Persistent proteinuria of NCI-CTCAE version 5.0 grade 3 or higher.<br>28. Arterial or venous thrombotic or embolic events such as cerebrovascular accident, deep vein thrombosis, or pulmonary embolism within 6 months before the start of study medication.<br>29. Refractory pleural effusion or ascites<br>30. History of hepatic encephalopathy within past 12 months<br>31. Oral intake impossible<br>32. HIV-positive<br>33. Pulmonary fibrosis or interstitial pneumonitis<br>34. Other serious complications as follows: serious mental disease or history of gastrointestinal bleeding or active hemoptysis<br>35. Unsatisfactory general condition for participation in the study as judged by the primary physician
Endoints	**Primary Endpoints:**<br>AEs/SAEs including DLTs<br><br>**Secondary Endpoints:**<br>Overall Survival (OS), Survival Rate at 6 months, Objective Response Rate (ORR), Time to Progression (TTP), Progression Free Survival (PFS) at 6 months (in accordance with mRECIST)_
Dosage and treatment	Each cycle of this study will last for 28 days, and the DLT evaluation period will be 42 days from the start of study drug administration.<br>Durvalumab 1,500 mg alone or in combination with 300 mg tremelimumab IV on Cycle 1, Day 1.<br>Carbon ion radio theray (60 Gy (RBE) / 4 times / 4 days) is administered between days 8-15 of the first cycle.<br>After the DLT evaluation period, durvalumab is continued every 4 weeks until tumor progression is confirmed.
Criteria for discontinuation of individual cases	1) In case it is judged difficult to continue the clinical trial for some reason on the subject's side, such as non-attendance or transfer to a different hospital.<br>2) In case the subject requests to discontinue participation in the clinical trial<br>3) In case the investigator or subinvestigator determines that the subject is unable to continue the clinical trial.<br>4) In case the subject's body weight becomes less than 30 kg.<br>5) In case the investigator/participating investigator determines that a decision to discontinue.

<table>
<tr>
<td>Number of Patients Planned:</td>
<td>

Number of Patients Planned:

15 subjects.

The number of patients enrolled for DLT assessment in either Cohort A or Cohort B may vary from 3 to 6 depending on the frequency of DLT. If both of Cohort A and B regimens were confirmed tolerable after DLT assessment, additional patients will be enrolled in Cohort B up to a total of 15 subjects. If only Cohort A regimen was determined to be tolerable, additional patients may be enrolled in Cohort A up to a total of 15 subjects.

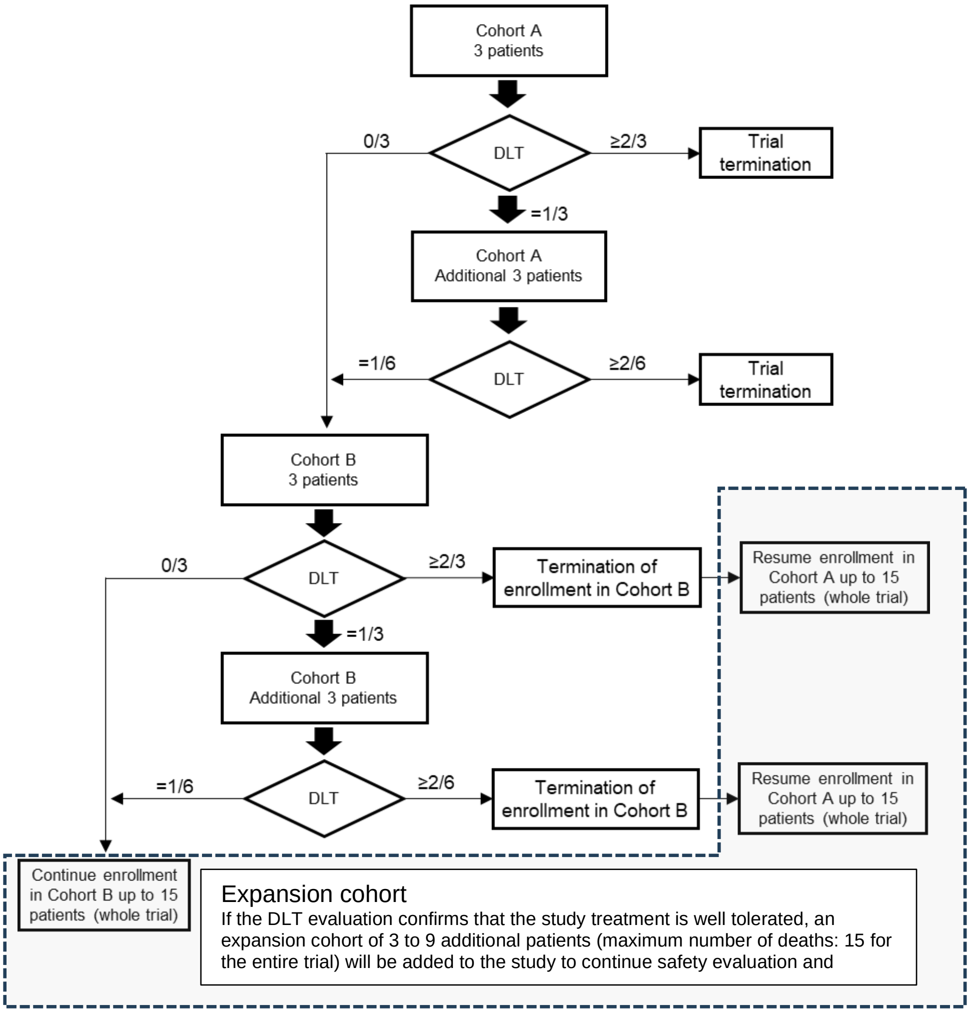

</td>
</tr>
<tr>
<td>Clinical trial period</td>
<td>Estimated study start date 30 Apr 2021
Estimated study completion date 30 Sep 2023</td>
</tr>
<tr>
<td>Subject registration period</td>
<td>Estimated date of first patient enrolled 30 Apr 2021
Estimated date of last patient enrolled 31 Mar 2023</td>
</tr>
<tr>
<td>Clnical trial sites</td>
<td>2Sites
Enrollment of patients in the trial and administration of IPs will be conducted at Chiba University Hospital, and CIRT will be administered at QST Hospital.</td>
</tr>
</table>

ETHICAL AND REGULATORY REQUIREMENTS	The study will be performed in accordance with ethical principles that have their origin in the Declaration of Helsinki and are consistent with Good Clinical Practice, and applicable regulatory requirements Patient data protection.
Institutional Review Board (IRB)	Prior to the start of the study, the Institutional Review Board (IRB) will evaluate the ethical, scientific and medical relevance of this study. The study will be conducted after obtaining approval from the IRB. If the evaluation results of the IRB are “approved with modification”, the study will be conducted after the protocol, case report, or consent forms are modified based on the review results. At the frequency of more than once a year, the IRB will continuously review whether the study is being performed appropriately.

1. INTRODUCTION

1.1. Introduction

Primary liver cancer is broadly classified into hepatocellular carcinoma (HCC) and intrahepatic cholangiocarcinoma, of which HCC accounts for more than 90% in Japan. According to a recent survey report, the number of patients with hepatocellular carcinoma in Japan is approximately 47,000 [1], and the number of deaths is approximately 27,000 per year [2].

The development of HCC is strongly associated with chronic hepatitis and cirrhosis caused mainly by hepatitis B virus (HBV) or hepatitis C virus (HCV) infection, and carriers of these viruses are considered to be at high risk of developing HCC ([3] El Serag HB, et al. NEJM. 2011). Risk factors other than viral hepatitis include cirrhosis, male age, older age, alcohol consumption, smoking, obesity, fatty liver, and diabetes mellitus [4]. Many patients are diagnosed after the disease has reached an advanced stage. The choice of treatment depends on the stage of the disease and the patient's condition. Since most patients with HCC also have chronic hepatitis or cirrhosis, individual assessment of liver function is essential to patient selection. Although several agents have been shown to be effective in the treatment of unresectable advanced HCC, there are still limited options and further therapeutic development is needed.

1.2. Standard Treatment for Advanced Hepatocellular Carcinoma

Sorafenib demonstrated a survival advantage over placebo in unresectable advanced HCC in the Sorafenib Hepatocellular Carcinoma Assessment Randomized Protocol (SHARP) trial in 2008, followed by the Asia-Pacific trial in 2009 ([5] Llovet JM, et al. Asia-Pacific trial also demonstrated a survival benefit ([5] Llovet JM, et al. NEJM. 2008, [6] Cheng AL, et al Lancet Oncol. 2009). In the SHARP trial, median survival (OS) was 10.7 months in the sorafenib group versus 7.9 months in the placebo group, with a hazard ratio (HR) of 0.69 (95% confidence interval (CI): 0.55, 0.87), P < 0.001. months vs. 4.2 months, HR 0.68 (95% CI: 0.50, 0.93), P = 0.014. The median progression-free survival in the SHARP trial was 5.5 months in the sorafenib group versus 2.8 months in the placebo group, HR 0.58 (95% CI: 0.5, 0.7), and in the Asia-Pacific trial the median progression-free survival was 2.8 months versus 1.4 months, HR 0.6 (95% CI: 0.4, 0.8).

Regorafenib after Sorafenib in Child-Pugh classification A patients with image progression after sorafenib treatment and who tolerated sorafenib (400 mg or more for at least 20 days in the 28 days before the end of treatment) compared regorafenib to placebo Patients with Hepatocellular Carcinoma (RESORCE) trial, regorafenib showed for the first time a survival advantage as second-line therapy after sorafenib treatment ([7] Bruix J, et al. Lancet. 2017). In that study, the median OS was 10.6 months (95% CI: 9.1, 12.1) in the regorafenib group and 7.8 months (95% CI: 6.3, 8.8) in the placebo group, HR 0-63 (95% CI: 0.50, 0.79) P < 0-0001. 2017, lenvatinib was non-inferiority to sorafenib for the primary endpoint of survival, making it the first-line treatment of choice for advanced hepatocellular carcinoma ([8] Kudo M, et al. Lancet. 2018). In that study, the median OS was 13.6 months (95%CI: 12.1, 14.9) in the lenvatinib arm and 12.3 months (95%CI: 10.4, 13.9) in the sorafenib arm with a HR of 0.92 (95%CI : 0.79, 1.06). In addition, ramucirumab became available in June 2019 for unresectable HCC with serum AFP levels of 400 ng/mL or higher, exacerbated after cancer chemotherapy ([9]Zhu AX, et al. Lancet Oncol. 2019).

Combination therapy with atezolizumab, an anti-PD-L1 humanized monoclonal antibody, and bevacizumab, an anti-VEGF monoclonal antibody, was shown to significantly prolong OS versus sorafenib in patients with Child-Pugh classification A who had not received systemic therapy (IMbrave 150 study) ([7 Bomze D et al. NEJM. 2020). In that study, 12-month survival rates were reported as 67.2% (95% CI: 61.3, 73.1) for atezolizumab plus bevacizumab and 54.6% (95% CI: 45.2, 64.0) for sorafenib, with an HR for OS of 0.58 (95% CI: 0.42, 0.79), P < 0 .001. Based on these results, the combination of atezolizumab and bevacizumab became available in Japan in September 2020. Based on these results, the combination of atezolizumab and

bevacizumab is now the first-line treatment for advanced hepatocellular carcinoma. Other first-line treatment options include sorafenib or lenvatinib, and second-line treatment options include regorafenib, ramucirumab, and cabozantinib. However, the survival gains obtained have been limited and do not fully meet the medical needs of patients.

1.3. HCC with vascular invasion and its treatment

HCC with vascular invasion (MVI) extending into the portal and/or hepatic veins has a poorer prognosis compared to cases without MVI ([11]Costentin CE, et al. Liver Cancer 2017). Although systemic chemotherapy is the standard of care, several studies have suggested the efficacy of alternative treatment approaches (e.g., resection, hepatic arterial chemoembolization, intravenous chemotherapy, and radiation therapy) after determining the benefit for each patient, and are recommended in Japanese guidelines. MVI Although it is empirically known that controlling MVI dramatically improves the prognosis of advanced HCC with MVI, sufficient evidence does not exist.

1.4. Immunotherapy

In recent years, research on cancer immunity has advanced dramatically, and the mechanisms by which the immune system controls or eliminates tumors are becoming clearer ([12] Dunn, et al. Annu Rev Immunol. 2004). PD-L1 is part of a complex system of receptors and ligands involved in the regulation of T cell activation. The PD-1 receptor (CD279) is expressed on the surface of activated T cells ([13] Keir ME, et al. Rev Immunol. 2008). It has two known ligands: PD-L1 (B7 H1; CD274) and PD-L2 (B7 DC; CD273) ([14] Okazaki and Honjo 2007). PD-1 and PD-L1 / PD-L2 act as co-inhibitors that can arrest or limit T cell responses When PD-L1 binds to PD-1, inhibitory signals are transmitted to T cells, cytokine production is reduced, and T cell proliferation is inhibited. Tumor cells have been shown to utilize this immune checkpoint pathway as a mechanism to evade detection and inhibit immune responses.

PD-L1 has been found to be expressed in a wide range of carcinomas, and anti-PD-L1 antibodies can be used therapeutically to augment anti-tumor immune responses in cancer patients.Results from preclinical and clinical studies of monoclonal antibodies (mAbs) targeting the PD-L1 / PD-1 pathway provide evidence of clinical activity and manageable Anti-PD-L1 antibodies can be used to augment anti-tumor immune responses in cancer patients for therapeutic purposes, showing evidence of clinical activity and manageable safety profiles ([17] Brahmer JR, et al. N Engl J Med. 2012, [18] Hirano F, et al. Cancer Res. 2005, [19] Iwai Y, et al. Proc Natl Acad Sci USA. 2002,[20] Okudaira K, et al. Int J Oncol. 2009,[21] Topalian SL, et al. N Engl J Med. 2012,[22] Zhang C,et al. Cytotherapy. 2008), The hypothesis that its efficacy is higher in patients with tumors expressing PD-L1 ([23] Powles T, et al. Nature. 2014; [24] Rizvi N, et al. J Clin Oncol. 2015; [25] Segal NH, et al. J Clin Oncol. 2015) is now supported The hypothesis of a “bladder cancer” (e.g., [24] Rizvi N et al. The high frequency and number of mutations in bladder cancer ([26] Alexandrov et al. Nature. 2013), for example, may contribute to the responses seen with immunotherapy.

In contrast, cytotoxic T lymphocyte-associated antigen 4 (CTLA-4) is structurally expressed on regulatory T cells and is enhanced on the surface of activated T cells; CTLA-4 sends negative regulatory signals to T cells when it binds to CD80 (B7.1) or CD86 (B7.2) ligands on antigen-presenting cells ([27] (Fife BT, Bluestone JA. Immnol Rev. 2008). In animal models, blockade of CTLA-4 binding to CD80 / 86 by anti-CTLA-4 antibodies has also been shown to markedly enhance T cell activation and antitumor activity, as exemplified by the killing of established solid tumors in mice and induction of protective antitumor immunity. Therefore, treatment with anti-CTLA-4 antibodies is expected to enhance activation of the human immune system and anti-tumor activity in patients with solid tumors.

Preclinical data have been added along with abundant clinical data, indicating that inhibition of negative regulatory signals to T cells, such as cytotoxic T lymphocyte antigen 4 (CTLA-4) and PD-L1, is a promising approach with promising clinical results. Ipilimumab has received U.S. Food and Drug Administration (FDA) approval for the treatment of metastatic melanoma,

and nivolumab and pembrolizumab (two anti-PD-1 agents) and atezolizumab (an anti-PD- L1 agent) are currently approved by agencies such as the U.S. Food and Drug Administration and the European Medicines Agency for the treatment of metastatic melanoma, squamous cell and non-squamous cell non-small cell lung cancer, urothelial carcinoma, and other malignancies. In addition, data from agents in the anti-PD-1 / PD-L1 class have shown clinical activity against a wide range of carcinomas.

In HCC as in other cancer types, two clinical trials (nivolumab and pembrolizumab) using ICI showed that 17-20% of patients achieved an objective response and most of the responders had a durable response ([28]El-Khoueiry AB, et al . Lancet 2017, [29]Zhu AX, et al. Lancet Oncol. 2018). The combination of ICI with tyrosine kinase inhibitors or anti-VEGF monoclonal antibodies has shown promise with the potential to achieve extremely high sustained response rates in a variety of cancer types, including HCC ([30]Kudo M. World J Gastroenterol 2019). Recently, the IMbrave 150 trial showed that atezolizumab plus bevacizumab therapy extended both OS and PFS compared with sorafenib, positioning atezolizumab plus bevacizumab as the first-line treatment for advanced hepatocellular carcinoma. Immunotherapy is also playing an increasingly important role in advanced HCC, and a phase I study of durvalumab, an anti-PD-L1 antibody, and tremelimumab, an anti-cytotoxic T lymphocyte-associated antigen 4 (CTLA-4) monoclonal antibody, in patients with HCC demonstrated a response rate of 25% ([31 was demonstrated ([31] Kelly RK et al. ASCO2017). An expanded portion of a Phase II trial is underway. A Phase III trial evaluating the efficacy and safety of the combination of durvalumab and tremelimumab, as well as monotherapy with sorafenib and durvalumab (HIMALAYA trial) are also ongoing and awaiting results.

1.5. Carbon ion radiotherapy

Particle therapy, especially carbon ion radio therapy, has advantages in dose concentration and biological effects over photon therapy, such as x-rays and gamma rays, which are usually used in radiation therapy.

X-rays and gamma rays are most intense near the body's surface and penetrate the body with decreasing intensity as they travel deeper. For this reason, the most powerful way to treat a specific area with X-rays or gamma rays is to focus the radiation from many directions, concentrating the high-dose area on the lesion. However, for tumors that are close to vital organs and spread malformedly, it is difficult to avoid surrounding normal organs. In contrast, heavy particle beams, which become intense at a certain depth depending on their energy, but are weak before and after that point, can be easily focused by aligning the peak with the tumor ([32] Pedroni E, et al. Med Phys. 1995).

The principle of action of radiotherapy is the double-strand break of DNA by ionizing radiation. Carbon ion radio therapy is known to cause dense ionization. Therefore, DNA double-strand breaks can be caused more efficiently and more densely than with X-rays. This is the reason for the favorable biological effects of heavy particle beams. This, combined with the good dose distribution resulting from the physical characteristics of heavy-ion beams, enables them to efficiently exert their anti-tumor effects. In Japan, the National Institute of Radiological Sciences (now renamed the National Institute of Quantum Science and Technology) started heavy particle therapy in 1994 and has treated more than 10,000 patients with good results ([33] Mohamad O., et al. Cancer (Basel), 2018).

In HCC, high local control rates have also been shown with particle therapy ([34] Kasuya G, et al. Cancer. 2017, [35] Fukuda, K, et al. Cancer Sci. 2017, [36] Sorin Y, et al. Liver Cancer 2018, [37] (Igaki H, et al. Int J Clin Oncol. 2018). Furthermore, good outcomes have been shown for hepatocellular carcinoma with vascular invasion. ([38] Komatsu S, et al. J Gastroenterol. 2011, [39] Lee SU, et al. Strahlenther Onkol. 2014, [40] Sugahara S, et al. Strahlenther Onkol. 2009).

1.6. Induction of Immunogenicity by Radiotherapy

Radiation therapy is known to stimulate immunogenicity through multiple mechanisms. The major immunological effects of irradiation include increased antigen presentation through

elevated expression of major histocompatibility gene complex class I, induction of apoptosis through elevated membrane expression of Fas ligands, calreticulin expression and high mobility group box-1 (HMGB1) and other (HMGB1), and induction of phagocytosis and immunity through the release of damage-associated molecular patterns (DAMPs).

Clinically, the PACIFIC trial showed that the combination of durvalumab and chemoradiotherapy can provide high therapeutic efficacy with intolerable toxicity ([41]Antonia Sj, et al. NEJM. 2017). Despite the fact that this study did not include only PD-L1-high expressing patients, it showed significant improvements in both PFS and OS compared to existing therapy. On the other hand, in the combination of durvalumab and tremelimumab (MYSTIC trial), there was no improvement from existing therapy, even when only PD-L1 high-expressing patients were targeted ([42 Rizvi NA, et al. Annals of Oncol. 2018). The difference between these two trials was the presence or absence of radiotherapy, and a subset analysis of the PACIFIC trial showed better results in patients with a shorter time between completion of radiotherapy and durvalumab administration, suggesting that radiotherapy plays an important role in tumor immunity Radiotherapy plays an important role in tumor immunity.

1.7. Combination of immune checkpoint inhibitors and carbon ion radiotherapy

Heavy ion therapy is known to enhance both local immunostimulation and immunosuppression more strongly than conventional photon beam therapy in both animal models and human clinical specimens ([43] Helm A, et al. Int J Part Ther. 2018, [44] Takahashi Y, et al. Oncotarget. 2019, [45] Iijima M, et al. J Gynecol Oncol. 2020).

Irradiated tumor cells present HMGB1, a key factor among damage-related molecules, alongside numerous others ([46] Golden EB, et al. Front Oncol, 2012). HMGB1 functions as an immune-activating cytokine and is a key factor for toll-like receptor 4 (TLR4) and activates dendritic cells.

It has been suggested that HMGB1 is strongly induced by heavy particle therapy ([47] Onishi M, et al. J Radiat Res. 2018). A study in mice showed stronger immune activation when heavy ion therapy was combined with dendritic cell infusion. The combination of heavy particle therapy and immunotherapy has also been shown to enhance anti-tumor immunity and reduce metastases compared to x-ray therapy, immunotherapy alone, or a combination of both ([48] Matsunaga A, et al. Cancer. 2010, [49] Ando K, et al. J Radiat Res. 2017).

On the other hand, immunosuppressive molecules such as PD-L1 have also been found to be induced more strongly than with X-ray therapy ([45] Iijima et al. J Gynecol Oncol. 2020). Enhanced PD-L1 expression by irradiation has been reported via the AMT/AT/Chk1 pathway induced by DNA double-strand breaks ([50] Sato N, et al. Nat Commun. 2017). Heavy ion therapy is known to efficiently generate complex DNA double-strand breaks ([51] Oike T, et al. Sci Rep. 2016), supporting this phenomenon.

Clinical trials investigating the combination of immune checkpoint inhibitors with radiation in a number of carcinomas, including HCC, are ongoing. However, most of them are limited to combinations with conventional radiation therapy using X-rays, and only a few with particle therapy. Currently, there are no clinical trials testing the combination of heavy ion beams and immunotherapy, and this clinical trial is a pilot case. From basic research and clinical studies using X-rays, the combination of heavy ion therapy and immune checkpoint inhibitors is expected to have a greater synergistic effect.

1.8. Investigational drugs

1.8.1. Durvalumab

Durvalumab is a human immunoglobulin (Ig) subclass Grade 1κ (IgGrade 1κ) mAb that inhibits the interaction of PD-L1 with PD-1 on T cells and CD80 on immune cells but not with PD-L2. Durvalumab is being developed by AstraZeneca/MedImmune for the treatment of cancer.

The mechanism of action of durvalumab is said to be inhibition of the interaction of PD-L1 with PD-1 and CD80, which disrupts the suppression of the immune response and immune

response to tumor elimination in in vitro studies, durvalumab was found to antagonize PD-L1-mediated suppression on primary human T cells, restore T cell proliferation, and release interferon gamma (IFN-γ) ([52] Stewart R, et al. Cancer Immunol Res. 2015) In vivo studies showed that durvalumab inhibited tumor growth in a xenograft model through a T cell-dependent mechanism ([52] Stewart R, et al. Cancer Immunol Res. 2015). Based on these data, durvalumab is expected to stimulate anti-tumor immune responses in patients by binding to PD-L1 and shifting the balance toward anti-tumor immune responses. Durvalumab is designed to reduce antibody-dependent and complement-dependent cellular injury.

To date, more than 8,000 patients have received durvalumab as a single agent or in combination with other anticancer agents in studies sponsored by AstraZeneca throughout the clinical development program. Please refer to the most recent Durvalumab investigational brochure for nonclinical and clinical information, including safety, efficacy, and pharmacokinetics.

1.8.2. Tremelimumab

Tremelimumab is a human immunoglobulin (Ig) Grade 2 mAb that targets CTLA-4 (CTLA-4; cluster of differentiation [CD]152), a cell surface receptor that is primarily expressed on activated T cells and blocks their activation. Tremelimumab completely inhibits the interaction of human CTLA-4 with CD80 and CD86 and increases the release of cytokines (interleukin [IL]-2 and interferon [IFN]-γ) from human T cells, peripheral blood mononuclear cells and whole blood ([53] Tarhini and Kirkwood. Expert Opin Biol Ther. 2008). Tremelimumab is being developed by AstraZeneca for the treatment of cancer.

To date, it has been administered to more than 1,500 patients as a single agent or in combination with other anticancer agents. For a summary of nonclinical and clinical information, including safety, efficacy, and pharmacokinetics, please refer to the Tremelimumab investigational brochure.

1.8.3. Durvalumab + tremelimumab combination therapy

Since there is no overlap in the mechanisms of action of CTLA-4 and PD-1, targeting both PD-1 and CTLA-4 pathways may provide additive or synergistic effects ([16] Pardoll DM, et al. Nat Rev Cancer. 2012). Therefore, in addition to investigating both drugs as monotherapy for various cancer indications, AstraZeneca is also investigating the combination of durvalumab plus tremelimumab for the treatment of cancer.

Study D4190C00006 is a late phase I dose escalation study to establish the safety, pharmacokinetics/pharmacodynamics and preliminary antitumor activity of the combination of durvalumab plus tremelimumab in patients with advanced non-small cell lung cancer (NSCLC). The dosing schedule used in this study is durvalumab administered Q2W or Q4W until Month 12, followed by 7 doses of tremelimumab at Q4W until Week 24, then 2 additional doses every 12 weeks for up to 12 months. The study is ongoing and enrollment is ongoing. Other trials investigating combination therapy for NSCLC and other tumor indications have also been initiated.

To date, 3,000 patients have received the combination in various doses and dosing regimens throughout the clinical development program. For a complete summary of non-clinical and clinical trial information, including safety, pharmacokinetics, and efficacy, please refer to the most recent Durvalumab and Tremelimumab investigational brochure.

1.8.4. Rationale for Durvalumab and Tremelimumab as Treatment Options for HCC

The liver has multiple regulatory mechanisms to maintain an immunosuppressive environment. The normal liver is inherently prone to induce immune tolerance to prevent aberrant immunity to exposed pathogens ([54]Pardee AD and Butterfield LH.Oncoimmunology. 2012). Clinical and nonclinical data indicate that HCC increases the expression of immunosuppressive cell

populations such as regulatory T cells (Treg) and myeloid-derived suppressor cells, as well as suppressive signaling molecules including CTLA-4 and PD-1 ([54] ,[55] Gao O, et al. Clin Cancer Res. 2009, [56] Hato T, et al. Hepatology. 2014); HBV and HCV infection also increase Treg and PD-L1/PD-1 expression, suggesting that this pathway is involved in HBV and HCV-mediated hepatocellular carcinogenesis ([54],[57] Miroux C, et al. Expert Opin Biol Ther. 2010,[58] Golden-Mason L, et al. J Virol. 2007,[59]Peng G, et al. Mol Immunol. 2008).

It has also been shown that overexpression of PD-L1 results in higher malignancy, disease progression, and mortality in HCC ([54], [60] Klein Jp, et al. Stat Med. 2007). Therefore, suppression of PD-L51 and CTLA-4 function could reverse the immunosuppressive nature of HCC and promote host immunity against HCC and improve clinical outcome.

At this time, early promising clinical data suggest that anti-CTLA-4 and anti-PD-L1/PD-1 antibody agents are active against HCC and may help improve response rates and survival.

In 20 patients with HCV-associated HCC (43% Child-Pugh classification B) treated with 15 mg/kg of intravenous tremelimumab every 90 days ([61] Sangro B, et al. J Hepatol. 2013), tremelimumab was generally well tolerated and no patients received systemic steroids No patients received systemic steroids and there were no deaths related to the study drug. The majority of patients had transient increases in transaminases after the first dose, 45% of which were Grade 3 or higher, but not accompanied by a concurrent decline in liver function. 17 responses could be evaluated, with 3 (17.6%) patients achieving a definite partial response (PR).

In another phase I/II study, 32 patients with unresectable advanced HCC (Child-Pugh classification A/B7) received two dose levels of tremelimumab (3.5 and 10 mg/kg IV Q4W) with partial ablation (RFA/TACE) during a 6-week treatment period ([62] Duffy AG,. et al. J Hepatol. 2017). Safety assessment results showed no clear trend in the occurrence of adverse events throughout the entire dose-specific cohort of tremelimumab, and no dose-limiting toxicities (DLTs) were observed in the study. The major Grade 3 or 4 adverse events were increased AST (21%), increased ALT (9%), and hyperbilirubinemia (9%); no Grade 3 or 4 diarrhea, colitis, or pneumonitis occurred; of the 19 patients with evaluable response outside the area directly treated with TACE/RFA, 5 (26.3%) had confirmed PR. The safety and efficacy of durvalumab (anti-PD-L1 antibody) monotherapy were investigated in a phase I/II study (CDON-MEDI4736-1108) in 40 patients with HCC, with an objective response rate (ORR) of 10.3% and a median OS of 13.2 months. Detailed safety and efficacy data from the study are described in Section 1.2.2.1. Similar results were obtained with another anti-PD-1 antibody, with an ORR of 18.6% and a median OS of 13.2 months in patients with advanced HCC ([63] Melero I, et al. J Clin Oncol. 2017).

Although the data from these trials are exploratory, they suggest that monotherapy with both durvalumab and tremelimumab has an antitumor effect against HCC. The combination of durvalumab plus tremelimumab in patients with unresectable HCC is being investigated in an ongoing phase I/II study (D4190C00022). In that study, patients with unresectable HCC who had not been previously treated with immunotherapy received the combination of durvalumab plus tremelimumab four times in Q4W followed by durvalumab alone in Q4W. Interim data from 40 patients followed for more than 16 weeks in the study showed an ORR of 18%. The combination of durvalumab plus tremelimumab was generally well tolerated in the population of patients with unresectable HCC. phase II results presented at ASCO 2020 reported promising results for durvalumab 1,500 mg Q4W plus tremelimumab 300 mg once. ORR was 24% and median OS was 18.73 months (95% CI: 10.78-27.27). It was reported to have the most favorable risk-benefit profile compared to the other dose arms ([64]Kelly RK, et al. J Clin Oncol. 2020).

The combined clinical evidence suggests that both durvalumab and tremelimumab have clinical activity as monotherapy in patients with HCC, and that combination therapy with both agents may provide an even greater antitumor effect in this patient population. A phase III, randomized, global study is underway to evaluate the safety and efficacy of these two agents in patients with HCC.

1.9. hypothesis

The purpose of this study is to confirm the synergistic effect of durvalumab-tremelimumab in

combination with heavy ion therapy in patients with advanced HCC. The study was designed based on the following two hypotheses

- Activation of tumor immunity by heavy ion therapy will be enhanced by immune checkpoint inhibitors.
- The control of primary lesions with MVI in patients with advanced HCC will have clinically significant results.

2. STUDTY OBJECTIVE

2.1. Objective

A phase Ib, open-label, uncontrolled study to evaluate the safety and efficacy of durvalumab-tremelimumab in combination with heavy ion therapy in patients with advanced hepatocellular carcinoma with MVI to assess safety and tolerability as measured by frequency of adverse events including DLT. Efficacy will be evaluated based on overall survival, 6-month survival, objective response rate, 6-month progression-free survival, and progression-free interval.

2.2. Study results regarding the appropriateness of conducting this clinical trial, efficacy, and safety for the subject disease

2.2.1. Durvalumab + tremelimumab combination therapy dose rationale

The durvalumab + tremelimumab doses and regimen selected for this study are based on the goal of selecting an optimal combination dose of durvalumab and tremelimumab that would yield sustained target suppression (sPD-L1), demonstrate promising efficacy, and have an acceptable safety profile.

2.2.2. Dose rationale for combination regimen of durvalumab 1500 mg Q4W plus tremelimumab 300 mg × 1 dose

A summary of the existing PK and pharmacodynamic data has been utilized to guide theregimen selection for the combination of durvalumab 1500 mg plus single dose of tremelimumab 300 mg.

Pharmacokinetics/Pharmacodynamics data

The supporting data for this regimen are based on PK and pharmacodyamic data from regimens that used tremelimumab doses of greater than 1 mg/kg from Study D4190C00006. An approximate dose-proportional increases in PK exposure (maximum serum concentration and area under the serum drug concentration-time curve from time 0 to Day 28 post-dose) was observed with increasing doses of tremelimumab (1, 3, and 10 mg/kg). An exploratory pharmacodynamic analysis bioanalytically evaluated the effects of tremelimumab on proliferating T-cells from NSCLC patients who received tremelimumab (1, 3, or 10 mg/kg) and durvalumab (15 or 20 mg/kg) combination treatment. Monotonic increases in pharmacodynamic activity with the combination (increased activation/ proliferation markers on CD4 and CD8 T-cells in periphery) were observed with increasing doses of tremelimumab (1, 3, 10 mg/kg). The peak increase (%) from baseline of CD4+Ki67+ T-cells was observed 8 days post administration, and the peak level was significantly increased ($p \leq 0.05$) as increasing dose of tremelimumab in the range of 1 to 10 mg/kg. Study data also suggested that higher peak exposure (maximum serum concentration [Cmax]) of tremelimumab is related to a higher maximum pharmacodynamic effect in the NSCLC patient population. Overall, the PK/pharmacodynamic data suggest that tremelimumab of dose greater than 1 mg/kg with a higher peak exposure may be associated with a higher pharmacodynamic effect.

Additionally, based on simulation data, the Cmax (78 μg/mL) post single dose administration of tremelimumab 4 mg/kg is approximately 4-fold higher than the predicted Cmax (19 μg/mL) post the first dose of tremelimumab 1 mg/kg, and is 3-fold higher than the predicted Cmax (25 μg/mL) post the fourth dose of tremelimumab 1 mg/kg in a Q4W×4 doses setting.

Clinical data

The safety and preliminary efficacy of combination of durvalumab 1500 mg plus single dose of tremelimumab 300 mg in unresectable HCC population is being evaluated in the ongoing Phase I/II study (Study D4190C00022).

In summary, a single dose of tremelimumab 4 mg/kg, while maintaining a similar overall

exposure, has a 3- to 4-fold higher Cmax compared to the 4 doses of tremelimumab 1 mg/kg. Therefore, this single administration of the higher dose of tremelimumab may have the potential for better anti-tumor activity while potentially avoiding any cumulative toxicity associated with repeated dosing of the 1 mg/kg tremelimumab. Therefore, the regimen of durvalumab 1500 mg plus tremelimumab 300 mg×1 dose is being evaluated in the current study.

Rationale for fixed dosing

A fixed-dose regimen of 1500 mg (equivalent to 20 mg/kg) of durvalumab plus 300 mg (equivalent to 4 mg/kg) of tremelimumab will be used in this study.

2.2.2.1. Rationale for utilizing a fixed-dose regimen for durvalumab and tremelimumab

A population PK model was developed for durvalumab using monotherapy data from a Phase I study (Study CD-ON-MEDI4736-1108; N=292; doses=0.1 to 10 mg/kg Q2W or 15 mg/kg Q3W; solid tumors). Similarly, a population PK model was developed for tremelimumab using data from Phase I through Phase III (N=654; doses=0.01 to 15 mg/kg Q4W or Q90D; metastatic melanoma) ([66]Wang et al 2014).

Population PK analysis indicated only minor impact of body weight on the PK of durvalumab and also tremelimumab (coefficient of ≤0.5). The weight-based versus fixed-dose (based on median weight of approximately 75 kg) regimens of both durvalumab and tremelimumab were compared using predicted PK concentrations (5th, 50th, and 95th percentiles) using a population PK model. A total of 1000 patients were simulated using weight distribution of 40 kg to 120 kg. Simulation results demonstrate that weight-based versus fixed dosing regimens of both durvalumab and tremelimumab yield similar median steady state PK concentrations with slightly less overall between-subject variability.

Similar findings have been reported by others ([22], [67] Narwal R, et al. Clin Pharmacokinet. 2013、[68] Ng CM, et al. Pharm Res. 2006, [69] Wang DD, et al. J Clin Pharmacol 2009, [70] Wolchok JD, et.al. N Engl J Med. 2013). Wang and colleagues investigated 12 monoclonal antibodies and found that fixed and body size-based dosing perform similarly, with fixed dosing being better for 7 of 12 antibodies ([21], [70] Wolchok JD, et al. N Engl J Med. 2013). In addition, they investigated 18 therapeutic proteins and peptides and showed that fixed dosing performed better for 12 of 18 in terms of reducing the between-subject variability in PK/pharmacodynamics parameters ([70] Wolchok JD, et al. N Engl J Med. 2013).

A fixed-dose approach is preferred by the prescribing community due to ease of use and reduced dosing errors. Given expectation of similar PK exposure and variability, we considered it feasible to switch to fixed-dose regimens. Based on the average body weight of 75 kg, a fixed dose of 1500 mg durvalumab (equivalent to 20 mg/kg) and a fixed dose of 300 mg tremelimumab (equivalent to 4 mg/kg) are selected for the current study. Therefore, the selected regimen of the durvalumab (+tremelimumab) cohort combined with particle therapy are:

【Cohort A】
Durvalumab monotherapy of 1500 mg Q4W

【Cohort B】
Durvalumab 1500 mg plus tremelimumab 300 mg for 1 dose, followed by durvalumab monotherapy 1500 mg Q4W

2.2.3 Rationale for carbon-ion radiotherapy

Historically, the role of radiation therapy in the treatment of liver tumors has been limited because of radiation-induced hepatic insufficiency caused by whole-liver irradiation ([71] Reed GB, et al. Am J Pathol, 1966). Particle therapy, including proton and carbon, overcame this problem with its physical dose distribution, enabling to treat large tumors while sparing normal liver tissue ([32], [72] Kanai et al. Int J Radiat Oncol Biol Phys, 1999). Results of CIRT have

been reported in multiple single institutional prospective studies and multi-institutional retrospective studies with high efficacy and mild toxicities ([34], [73] Shibuya K, et al. Liver Int. 2018, [74] Yasuda S, et al. Adv Radiat Oncol. 2019). Cases with MVI are also well treated with CIRT [35] Komatsu S, et al. J Gastroenterol. 2011). A single-arm clinical trial of CIRT in patients without large vessel or bile duct invasion is currently underway as an advanced medical treatment B, with the aim of developing a curative treatment for first-episode HCC that is not suitable for surgery (liver transplantation is not suitable) or radiofrequency ablation (jRCT1032200036).

Dose escalation studies have been conducted for 4 fraction CIRT ([34], [75]Shiba S, et al. Radiat Oncol. 2019). 60Gy (RBE) in 4 fractions appear to be well tolerated and multi-institutional retrospective study confirms its efficacy ([73] Shibuya K, et al. Liver Int. 2018). Constraints for each risk organ were determined based on previously published reports ([34], [73], [76] Shiba S, et al. Radiat Oncol 2020, [77] Makishima et al. Cancer Sci. 2018).

2.2.4 Rationale for combined treatment of carbon-ion radiotherapy and immunotherapy

In the PACIFIC trial, the combination of durvalumab and chemoradiotherapy (X-rays) has been shown to provide high therapeutic efficacy with intolerable toxicity, as discussed in 1.7 above ([41]Antonia SJ et al. NEJM. 2017). There are only ongoing trials of the combination of immune checkpoint inhibitors, including durvalumab, with particle therapy, and no reports exist as of November 2020. Trials of combinations with heavy particle therapy are similarly unregistered. As noted in 1.7 above, the results of basic research and clinical trials of combination therapy with X-rays suggest a higher synergistic effect.

2.3. Benefit-risk and ethical assessment

2.3.1. Potential benefits

2.3.1.1. Durvalumab monotherapy

Information on the potential benefit of durvalumab 1500 mg monotherapy or equivalent in patients with HCC are based on Study CD-ON-MEDI4736-1108 and are presented in Section 1.8.1. For other tumor types, see the most current durvalumab IB.

2.3.1.2. Durvalumab plus tremelimumab combination therapy

The potential benefits of adding tremelimumab to durvalumab is presented in Section 1.8.3. Information on the data supporting the selected combination regimen of durvalumab plus tremelimumab in patients with HCC are presented in Section 1.8.4. For other tumor types, see the most current durvalumab and tremelimumab IBs.

2.3.1.3. Durvalumab (+tremelimumab) combined with particle therapy

As mentioned in section 1.7, CIRT, as with traditional photon irradiation, is known to modify cancer immune reactions, but at a stronger level (Helm A, et al. Int J Part Ther. 2018, Ebner et al. Front Oncol. 2017). By combining CIRT with innunotherapy [durvalumab (+ tremelimumab)], there may be a larger improvement in efficacy compared to monotherapy of immunotherapy drugs.

2.3.2. Overall risks

Monoclonal antibodies directed against immune checkpoint proteins, such as programmed cell death ligand 1 (PD-L1) as well as those directed against programmed cell death-1 (PD-1) or cytotoxic T-lymphocyte antigen-4 (CTLA-4), aim to boost endogenous immune responses directed against tumor cells. By stimulating the immune system however, there is the potential for adverse effects on other tissues.

Most adverse drug reactions seen with the immune checkpoint inhibitor class of agents are thought to be due to the effects of inflammatory cells on specific tissues. These risks are generally

events with a potential inflammatory or immune mediated mechanism and which may require more frequent monitoring and/or unique interventions such as immunosuppressants and/or endocrine therapy. These immune mediated effects, can occur in nearly any organ system, and are most commonly seen as gastrointestinal AEs such as colitis and diarrhea, pneumonitis/interstitial lung disease (ILD), hepatic AEs such as hepatitis and liver enzyme elevations, skin events such as rash and dermatitis and endocrinopathies including hypo- and hyper-thyroidism.

2.3.2.1. Durvalumab

Risks with durvalumab include, but are not limited to, diarrhea/colitis, pneumonitis/ILD, endocrinopathies (hypo- and hyper-thyroidism, type I diabetes mellitus, hypophysitis and adrenal insufficiency) hepatitis/increases in transaminases, nephritis/increases in creatinine, diabetes insipidus, pancreatitis/increases in amylase and lipase, rash/pruritus/dermatitis, encephalitis, myocarditis, immune thrombocytopenia, myositis/polymyositis, other rare or less frequent inflammatory events including neurotoxicities, infusion-related reactions, injection site reactions, hypersensitivity reactions and infections/serious infections.

For information on all identified and potential risks with durvalumab please always refer to the current version of the durvalumab IB.

In monotherapy clinical studies AEs (all grades) reported very commonly (≥ 15% of patients) are fatigue, nausea, decreased appetite, dyspnea, cough, constipation, diarrhea, vomiting, back pain, pyrexia, asthenia, anemia, arthralgia, peripheral edema, headache, rash, and pruritus. Approximately 9.4% of patients experienced an AE that resulted in permanent discontinuation of durvalumab and approximately 6.5% of patients experienced an SAE that was related to durvalumab by the study investigator.

Most treatment-related AEs were manageable with dose delays, symptomatic treatment, and in the case of events suspected to have an immune basis, the use of established treatment guidelines for immune-mediated toxicity.

A detailed summary of durvalumab monotherapy AE data can be found in the current version of the durvalumab IB.

2.3.2.2. Tremelimumab

Risks with tremelimumab monotherapy include, but are not limited to, GI effects (colitis, diarrhoea, enterocolitis and intestinal perforation), endocrine disorders (hypo and hyperthyroidism, hypophysitis and adrenal insufficiency), skin effects (rash, and pruritus), elevations in lipase and amylase and clinical manifestations of pancreatitis, other gastrointestinal events e.g. ulcerative colitis, dehydration, nausea and vomiting; hepatic events including hepatitis, and liver enzyme elevations; pneumonitis and ILD; nervous system events including encephalitis, peripheral motor and sensory neuropathies, Guillain-Barre and proximal muscle weakness; cytopenias including thrombocytopenia, anemia and neutropenia; infusion-related reactions, anaphylaxis, and allergic reactions; renal events including renal failure, acute kidney injury, nephritis, nephrotic syndrome, autoimmune nephritis and electrolyte abnormalities such as hypokalemia; autoimmune diseases including autoimmune arthritis, Sjogren's syndrome and giant cell temporal arteritis; hyperglycemia and diabetes mellitus; and pyrexia.

For information on all identified and potential risks with tremelimumab please always refer to the current version of the tremelimumab IB.

Using pooled data from monotherapy clinical studies AEs (all grades) reported very commonly (≥ 10% of patients) were diarrhea, nausea, fatigue, pruritus, decreased appetite, rash, vomiting, dyspnoea, constipation, cough, pyrexia, abdominal pain, decreased weight, headache, asthenia, and anaemia. Approximately 16% of patients experienced an AE that resulted in permanent discontinuation of tremelimumab and approximately 45% of patients experienced an SAE.

A detailed summary of tremelimumab monotherapy AE data can be found in the current version of the tremelimumab IB.

2.3.2.3. Durvalumab + tremelimumab

The safety of durvalumab + tremelimumab combination therapy was initially evaluated in the ongoing dose escalation and dose expansion Study 006, in patients with NSCLC, and is being studied in a number of other ongoing clinical trials, in a number of different indications, and has to date shown a manageable safety and tolerability profile.

The types of risks with the combination of durvalumab + tremelimumab (based on an equivalent durvalumab dose of 20m/kg and a tremelimumab dose of 1mg/kg) are similar to those for durvalumab and tremelimumab monotherapy. Emerging data from study 006, other studies evaluating the combination, and from combinations of other agents in the same class indicate an increased frequency and/or severity of some of these immune-mediated toxicities.

For information on all identified and potential risks with the durvalumab+tremelimumab combination please always refer to the current version of the durvalumab IB

In durvalumab+tremelimumab combination studies at the dose of durvalumab 20mg/kg and tremelimumab 1mg/kg AEs (all grades) reported very commonly (≥ 10% of patients) are fatigue, diarrhoea,nausea, dyspnea, decreased appetite, pruritus, vomiting, anaemia, constipation, cough, abdominal pain, pyrexia, back pain, arthralgia, hypothyroidism, asthenia, oedema peripheral, decreased weight, decreased hyponatraemia and rash.

Approximately 15% of patients experienced an AE that resulted in permanent discontinuation of study drug and approximately 16% of patients experienced an SAE that was considered to be related to durvalumab and tremelimumab by the study investigator.

A detailed summary of durvalumab + tremelimumab combination AE data can be found in the current version of the durvalumab IB.

2.3.2.4. Carbon-ion radiotherapy

Safety of CIRT for HCCs as a monotherapy are confirmed through multiple single institutional prospective studies and multi-institutional retrospective studies ([34], [73], [74]). Grade 3 or severe acute toxicities were seen in skin (1%) and ALT elevation (0.6%), with both being G3. G3 or severe late toxicities were seen in skin (G3 2%, G4 0.6%), hepatic coma (G3 1%), myositis and rib fracture (both G3 0.6%). RILD was seen in 2%. No treatment related deaths were observed.

Since CIRT is a form of radiotherapy, potential risks will be confined within the radiation field. The following may be observed.

- Bone marrow suppression
- Radiation pneumonitis, pleuritis, pleural effusion
- Nausea, vomiting, anorexia, diarrhoea, GI bleeding, ulceration, perforation, stricture
- Loss of hepatic function, hepatic failure, RILD, bile duct stricture, occlusion, cholangitis, aneurism
- Radiation dermatitis, ulceration
- Pericarditis, pericardial effusion, congestive heart failure, myocarditis, arrythmia
- Myelopathy, Peripheral neuropathy
- Rib fracture, subcutaneous induration, subsequent primary cancer, renal failure

2.3.2.5. durvalumab (+tremelimumab) combined with carbon-ion radiotherapy

Currently, there are no clinical trial results open yet for durvalumab + tremelimumab + radiotherapy for liver tumors, thus risks are unknown. Combination of immuno-oncology drugs and radiotherapy appear to have minimal excessive toxicity compared to immune-oncology drugs alone across multiple trials, including thoracic irradiation ([41] Antonia SJ, et al. NEJM 2018).

Potential risks would be toxicities closely related to immune reaction and microangiopathy, such as RILD, radiation pneumonitis and GI tract ulceration. In CIRT as a monotherapy, these toxicities are less common compared to photon radiotherapy, owing to the better dose distribution of CIRT as explained above ([34], [73], [74]). While the impact of difference in immune-modulation against toxicity between photon and carbon-ion is unknown, there is no definite

evidence that excessive toxicity will be intolerable.

2.3.3. Overall benefit-risk

Durvalumab and tremelimumab have shown encouraging anti-tumor activity as single agents in advanced HCC population. The summary of this efficacy data is presented in Section 1.8.4. The combination regimen of these two agents shows a higher response rate in HCC population compared to either of the monotherapies. Thus, durvalumab plus tremelimumab combination therapy may potentially offer benefit to this patient population. Both durvalumab monotherapy and durvalumab plus tremelimumab combination therapy was tolerable in advanced HCC. The current study design aims to minimize potential risks by providing for early and intensive safety monitoring for any unexpected safety signals and for managing those risks deemed to be most likely based on prior experience with durvalumab, tremelimumab, and carbon-ion therapy. Two combination dose regimens of durvaluamb plus tremelimumab combination therapy were selected for this study with the aim to select the regimen with the most benefit for patients with advanced HCC.

HCC patients with MVI have very limited systemic therapeutic options and a poor life expectancy and health-related quality of life (HRQoL) based on the currently available treatments. While the main prognosticator in these patients is the intravascular growth of MVI, thus focal treatment by particle therapy has shown prolonged MST up to 2 years if lesions are confined in number ([40] Sugahara S, et al. Strahlether Onkol. 2009)). But those with extensive disease (ie multiple hepatic lesions and extrahepatic lesions) are currently not treated with particle therapy in fear of the out-of-treatment-field lesions. HCC patients with MVI, therefore, represents a significant unmet medical need and underlines the need for novel therapies for this patient population. CIRT combined with durvalumab or durvalumab plus tremelimumab proposed in this study may demonstrate a meaningful clinical benefit and a manageable safety profile. The overall benefit-risk profile of durvalumab (+tremelimumab) combined with CIRT is expected to be favorable, therefore supporting the current study design.

3. ELIGIBILITY

Each patient must meet all of the inclusion criteria (Section 3.2) and none of the exclusion criteria (Section 3.3) for this study. Under no circumstances will there be exceptions to this rule.

3.1. Diagnostic Criteria and Stage, Type, and Condition Classification

【CohortA & CohortB】

Eligible patients will be advanced HCC with MVI and Child-Pugh classification A over 20 years of age who are refractory or intolerant to standard systemic chemotherapy.

【Expansion cohort】

Eligible patients are advanced HCC with MVI, aged 20 years or older, with Child-Pugh classification A, with or without prior drug therapy.

3.2. Inclusion criteria

For inclusion in the study, patients should fulfill the following criteria:

1） Capable of giving signed informed consent which includes compliance with the requirements and restrictions listed in the informed consent form (ICF) and in this protocol. Written informed consent and any locally required authorization obtained from the patient/legal representative prior to performing any protocol-related procedures, including screening evaluations. For patients aged <20 years and enrolling, a written informed consent should be obtained from the patient and his or her legally acceptable representative.

2） Age >20 years at time of study entry

3） Eastern Cooperative Oncology Group (ECOG) performance status of 0 or 1

4） Body weight >30 kg

5） Adequate normal organ and marrow function as defined below:

- Haemoglobin ≧9.0 g/dL
- Absolute neutrophil count (ANC) ≧1,500/mm3
- Platelet count ≧75×109/L （≧75,000/mm3）
- Serum bilirubin ≦ULN×3.0
- AST ≦ULN×5.0
- ALT ≦ULN×5.0
- Measured creatinine clearance (CL) >40 mL/min or Calculated creatinine clearance CL>40 mL/min by the Cockcroft-Gault formula (Cockcroft and Gault 1976) or by 24-hour urine collection for determination of creatinine clearance

6） Evidence of post-menopausal status or negative urinary or serum pregnancy test for female pre-menopausal patients. Women will be considered post-menopausal if they have been amenorrheic for 12 months without an alternative medical cause. The following age-specific requirements apply:

- Women <50 years of age would be considered post-menopausal if they have been amenorrheic for 12 months or more following cessation of exogenous hormonal treatments and if they have luteinizing hormone and follicle-stimulating hormone levels in the post-menopausal range for the institution or underwent surgical sterilization (bilateral oophorectomy or hysterectomy).
- Women ≥50 years of age would be considered post-menopausal if they have been amenorrheic for 12 months or more following cessation of all exogenous hormonal treatments, had radiation-induced menopause with last menses >1 year ago, had chemotherapy-induced menopause with last menses >1 year ago, or underwent surgical sterilization (bilateral oophorectomy, bilateral salpingectomy or hysterectomy).

7） Patient is willing and able to comply with the protocol for the duration of the study including undergoing treatment and scheduled visits and examinations including follow up.

8） Advanced HCC confirmed histologically or by the typical findings of a hypervascular tumor

on computed tomography or angiography

9） (Cohort A and Cohort B only) Patients who have received at least one prior systemic chemotherapy regimen including atezolizumab bevacizumab combination, sorafenib, or lenvatinib and who are determined to be refractory or intolerant to standard therapy.

10） Must not be eligible for locoregional therapy for unresectable HCC. For patients who progressed after locoregional therapy for HCC, locoregional therapy must have been completed ≥28 days prior to the baseline scan for the current study. Acceptable locoregional therapy for HCC are Ethanol Infusion Therapy, Radio Wave ablation Therapy, Transcatheter Arterial chemoembolization (TACE), Transcatheter arterial infusion (TAI). Hepatic Arterial Infusion Chemotherapy (HAIC) is not treated as a locoregional therapy.

11） Patients who have been diagnosed with HCC showing MVI. MVI is defined as a tumor thrombus in the major hepatic and/or portal vein branches (Vp2, Vp3, Vp4, Vv2, and Vv3) identified by imaging studies.

12） Child-Pugh A

13） At least one measurable lesion other than the MVI and feeding nodule based on mRECIST.

3.3. Exclusion criteria

Patients should not enter the study if any of the following exclusion criteria are fulfilled:

1） Involvement in the planning and/or conduct of the study (applies to both sponser and/or staff at the study site)

2） Patients who have participated in another clinical trial using the investigational drug within 28 days prior to obtaining consent or who have received another investigational drug within 28 days prior to the first dose of the investigational drug in this study. The exception is if the patient is in the follow-up period of an interventional trial or is participating in an observational (non-interventional) clinical trial.

3） Any unresolved toxicity NCI CTCAE Grade ≥2 from previous anticancer therapy with the exception of alopecia, vitiligo, and the laboratory values defined in the inclusion criteria
- Patients with Grade ≥2 neuropathy will be evaluated on a case-by-case basis after consultation with the Study Physician.
- Patients with irreversible toxicity not reasonably expected to be exacerbated by treatment with durvalumab or tremelimumab may be included only after consultation with the Study Physician.

4） Radiotherapy treatment to more than 30% of the bone marrow or with a wide field of radiation within 4 weeks of the first dose of study drug

5） Major surgical procedure (as defined by the Investigator) within 28 days prior to the first dose of IP. Note: Local surgery of isolated lesions for palliative intent is acceptable.

6） History of allogenic organ transplantation.

7） Active or prior documented autoimmune or inflammatory disorders (including inflammatory bowel disease [e.g., colitis or Crohn's disease], diverticulitis [with the exception of diverticulosis], systemic lupus erythematosus, Sarcoidosis syndrome, or Wegener syndrome [granulomatosis with polyangiitis, Graves' disease, rheumatoid arthritis, hypophysitis, uveitis, etc.]). The following are exceptions to this criterion:
- Patients with vitiligo or alopecia
- Patients with hypothyroidism (e.g., following Hashimoto syndrome) stable on hormone replacement
- Any chronic skin condition that does not require systemic therapy
- Patients without active disease in the last 5 years may be included but only after consultation with the study physician
- Patients with celiac disease controlled by diet alone

8） Uncontrolled intercurrent illness, including but not limited to, ongoing or active infection, symptomatic congestive heart failure, uncontrolled hypertension, unstable angina pectoris,

cardiac arrhythmia, interstitial lung disease, serious chronic gastrointestinal conditions associated with diarrhea, or psychiatric illness/social situations that would limit compliance with study requirement, substantially increase risk of incurring AEs or compromise the ability of the patient to give written informed consent

9) History of another primary malignancy except for
   - Malignancy treated with curative intent and with no known active disease ≥5 years before the first dose of IP and of low potential risk for recurrence
   - Adequately treated non-melanoma skin cancer or lentigo maligna without evidence of disease
   - Adequately treated carcinoma in situ without evidence of disease
   - However, the following cases are eligible for enrollment
   - Early stage cancer (epithelial cancer of the cervix, basal cell carcinoma, superficial bladder cancer (Tis and T1), early stage gastric cancer, and early stage colorectal cancer) that has been treated for curative purposes, has not been confirmed active for at least 3 years prior to inclusion in the study, and has a low risk of recurrence.

10) History of leptomeningeal carcinomatosis

11) History of, or current, brain metastases or spinal cord compression. Patients with suspected brain metastases at screening should have an MRI (preferred) or CT, each preferably with IV contrast of the brain prior to study entry.

12) Mean QT interval corrected for heart rate using Fridericia's formula (QTcF) ≥470 ms calculated from 3 ECGs (within 15 minutes at 5 minutes apart) Regardless of whether this criteria stays or not, all patients should have a baseline ECG

13) History of active primary immunodeficiency

14) Patients co-infected with HBV and HCV. HBV positive (presence of HBsAg and/or anti-HBcAb with detectable HBV DNA); HCV positive (presence of anti-HCV antibodies), and active infection including tuberculosis (clinical evaluation that includes clinical history, physical examination and radiographic findings, and TB testing in line with local practice).

15) Current or prior use of immunosuppressive medication within 14 days before the first dose of durvalumab or tremelimumab. The following are exceptions to this criterion:
   - Intranasal, inhaled, topical steroids, or local steroid injections (e.g., intra articular injection)
   - Systemic corticosteroids at physiologic doses not to exceed 10 mg/day of prednisone or its equivalent
   - Steroids as premedication for hypersensitivity reactions (e.g., CT scan premedication)

16) Receipt of live attenuated vaccine within 30 days prior to the first dose of IP. Note: Patients, if enrolled, should not receive live vaccine whilst receiving IP and up to 30 days after the last dose of IP.

17) Female patients who are pregnant or breastfeeding or male or female patients of reproductive potential who are not willing to employ effective birth control from screening to 90 days after the last dose of durvalumab monotherapy or180 days after the last dose of durvalumab + tremelimumab combination therapy.

18) Known allergy or hypersensitivity to any of the study drugs or any of the study drug excipients.

19) Prior randomisation or treatment in a previous durvalumab and/or tremelimumab clinical study regardless of treatment arm assignment.

20) Judgment by the investigator that the patient is unsuitable to participate in the study and the patient is unlikely to comply with study procedures, restrictions and requirements.

21) Patients who have been treated with anti-PD-1, anti-PD-L1 inhibitors, or other drugs that act on other stimulatory or co-suppressive T-cell receptors and their combinations (including atezolizumab plus bevacizumab) and have failed to tolerate the same treatment.

22) Prior radiotherapy involving the liver.

23) Renal failure requiring hemodialysis or peritoneal dialysis

24) Any of the following cardiac diseases:

- NYHA Class III or IV chronic heart failure
- Current coronary artery disease or history of ischemic heart disease such as myocardial infarction within 6 months before the study
- Serious arrhythmia (grade 3 or higher according to the CTCAE ver. 5.0: arrhythmia that cannot be controlled by oral medications or requires mechanical control).

25） Poorly controlled hypertension
26） Serious and active infection, excluding hepatitis viral infection
27） Persistent proteinuria of NCI-CTCAE version 5.0 grade 3 or higher.
28） Arterial or venous thrombotic or embolic events such as cerebrovascular accident, deep vein thrombosis, or pulmonary embolism within 6 months before the start of study medication.
29） Refractory pleural effusion or ascites
30） History of hepatic encephalopathy within past 12 months
31） Oral intake impossible
32） HIV-positive
33） Pulmonary fibrosis or interstitial pneumonitis
34） Other serious complications as follows: serious mental disease or history of gastrointestinal bleeding or active hemoptysis
35） Unsatisfactory general condition for participation in the study as judged by the primary physician

4. Informed consent

4.1. Preparation and revision of informed consent form

The investigator prepares the consent form and other information documents used to obtain consent for participation in the clinical trial from the subject in plain language as much as possible. If it is considered necessary to revise the consent document and other explanatory documents, the investigator revises these documents.

The investigator submits the prepared or revised consent documents and other explanatory documents to the IRB for approval.

Amendments to the study protocol and informed consent forms will follow the below procedures:

1. When amendments are considered to be necessary, the Principal Investigator will provide to the Investigator(s) the study protocol amendment draft, informed consent form amendment drafts, and the latest version of the investigator's brochure and other necessary material/information.
2. The Principal Investigator will provide the Investigator(s) with necessary time to adequately consider the aforementioned study protocol amendment draft and material/information and discuss the details with the Principal Investigator.
3. After discussion with the Principal Investigator, the Investigator(s) will promptly submit the amended version of the study protocol or informed consent form to the head of the trial site, and receive approval of the IRB via the head of the trial site.
4. Within acceptable limits of the Principal Investigator, the same procedures will apply to amendments to be made to the study protocol and informed consent form according to instructions given by the head of the trial site based the opinions of the IRB.

4.2. Method of Obtaining Informed Consent

1) Informed consent

The investigator or subinvestigator should hand the consent document and other explanatory documents to the subject and provide sufficient explanation of the contents as indicated in "4.3". If necessary, the clinical trial coordinator also provides supplementary explanations to the subject. After confirming that the patient has a good understanding of the contents of the clinical trial, the subject's signed and dated informed consent should be obtained before the pre-study (screening) test is conducted.

2) When explaining to subjects

The investigator or sub-investigator shall give the subject the opportunity to ask questions and sufficient time to decide whether or not to participate in the trial before obtaining informed consent and shall answer the subject's questions to the subject's satisfaction.

3) Signing and delivery of consent form

The investigator or subinvestigator who provided the explanation should sign the consent form with the date of the explanation. The subject signs the consent form with the date of consent. If a collaborator provides supplementary explanation, the collaborator should also sign and enter the date of the explanation. After obtaining informed consent, a copy of the information document and the consent form shall be given to the subject.

4) Amendments to informed consent forms

When the investigator or subinvestigator revises the informed consent form or other explanatory documents due to the acquisition of new information that may be relevant to the subject's consent, the investigator or subinvestigator shall explain to the subject again using the revised informed consent form and other explanatory documents, and obtain consent in writing for the subject's continued participation in the clinical trial. If new important information is obtained that may affect the subject's consent, the information shall be immediately provided to the subject, recorded in writing, and the subject's continued participation in the clinical trial shall be confirmed.

4.3. Information to be provided to subjects

The informed consent form to be prepared by the investigator shall include the following information.

1. What is a clinical trial?
2. The purpose of the clinical trial
3. Name, title and contact information of the investigator
4. Method of the clinical trial
5. Anticipated clinical benefits and risks or inconveniences
6. Availability of other treatment options for the subject and the expected important benefits and risks associated with such treatment options
7. The expected duration of the subject's participation in the clinical trial
8. That participation in the clinical trial is of the subject's own free will and that the subject may refuse or withdraw from participation in the clinical trial at any time. Furthermore, the subject shall not be treated unfavorably due to refusal or withdrawal, and shall not lose any benefits that he/she would have received if he/she had not participated in the clinical trial.
9. Monitors, auditors, clinical trial review committees, and regulatory authorities must be able to view source documents related to medical care. In such cases, the confidentiality of the subject shall be maintained. In addition, the subject's signature on the consent document shall be considered as authorization for access.
10. Subjects' confidentiality shall be maintained even if the results of the clinical trial are made public.
11. Compensation and treatment to which subjects are entitled in the event of adverse health effects related to the clinical trial.
12. Information that may influence the subject's decision to continue participation in the clinical trial will be promptly communicated to the subject.
13. Conditions or reasons for discontinuation of participation in the clinical trial
14. Expenses to be borne by the subject in relation to the clinical trial
15. Details of any financial or other payments to be made to the subject in connection with the clinical trial (e.g., arrangements for calculating the amount to be paid)
16. The medical institution's contact person to whom subjects should refer or contact if they require further information regarding the clinical trial and their rights or if they experience any health problems related to the clinical trial.
17. Items to be observed by the subject
18. Types of investigational review committees that will investigate and deliberate on the appropriateness of the clinical trial, matters to be investigated and deliberated by each investigational review committee, and other matters related to the investigational review committee for the clinical trial in question
19. Intellectual property
20. Conflicts of interest

5. STUDY DESIGN

5.1. Overview of study design

This is a Phase Ib study to assess the safety of durvalumab combined with particle therapy (Cohort A) and durvalumab plus tremelimumab combined with particle therapy (Cohort B) in advanced hepatocellular carcinoma patients with macrovascular invasion. This study consists of four periods: the screening period, DLT assessment period, durvalumab q4W dosing period, and follow up period. After the signed informed consent is obtained and the screening is conducted, the patient will be registered for enrollment in the trial. Patients will be administered with the first IP followed by administration of CIRT.

DLT assessment period is for 42days starting from the first dose of durvalumab on Day1 of Cycle1. The first administration of continuous durvalumab q4W on Day 1 of Cycle 2 starts only after the safety of Cycle 1 was confirmed (durvalumab q4W: 28 day cycle).

DLT analysis will be made when more than one DLT was observed in each cohort.

Patients will to continue to receive durvalumab every 4 week after completion of the DLT assessment period until clinical progression/withdrawal from the trial if there may be potential clinical benefit at the investigator's discretion.

Follow up visit will be made 28 days after study termination due to PD or withdrawal from the study. Safety information will be collected until 90 days after the last dose of study treatment or until initiation of alternative anticancer therapy. In this study, three patients are initially enrolled into cohort A. If there is no DLT observed in any of these subjects, the trial proceeds to enroll additional subjects into the cohort B, whose regimen does not contain higher dose of durvalumab but contains an additional drug of tremelimumab instead. If one subject develops a DLT at cohort A or cohort B, an additional three subjects are enrolled into that same cohort. Development of DLTs in more than 1 of 6 subjects in either cohort suggests that the regimen is not tolerable. If cohort A turns out to be intolerable, then cohort B regimen will not be pursued The evaluation of DLTs shall be performed by the investigator of Chiba University Hospital in consultation with the investigator(s). The DLTs determined shall be discussed with the Independent Data Monitoring Committee and their opinion shall be sought in accordance with the standards separately stipulated (10.7. Independent Data Monitoring Committee).

Duration of DLT assessment is defined for 42 days starting from the first administration of IP on Day1 of Cycle 1. Dose of durvalumab is fixed on 1500 mg. CIRT will be performed between Day 8 to Day 14 of Cycle 1 after the first duvalumab administration on Day1 (CIRT within 14 days after 1stcycle of durvalumab).

In both cohorts, if the investigators determined that there may be potential clinical benefit, patients will to continue to receive durvalumab every 4 week until clinical progression (Durvalumab q4W dosing period).

- **Cohort A**: Durvalumab 1500mg will be administered every 4 weeks in principle. Particle therapy, in form of CIRT, will be performed after Day8 of Cycle1 following the 1st dose of Durvalumab on Day1. 2nd cycle of durvalumab will be administrated only after the safety during DLT assessment was confirmed.

- **Cohort B**: Durvalumab 1500mg will be administered every 4 weeks in principle, and Tremelimumab 300mg will be administered only on Day1 of Cycle1. Particle therapy, in form of CIRT, will be performed after Day8 of Cycle1 following 1st cycle of Durvalumab + Tremelimumab. 2nd cycle of durvalumab will be administrated only after the safety during DLT assessment was confirmed.

CIRT will be given to both arms. Dose prescription and fractionations will be 60Gy (RBE) / 4Fr /

1week. Target lesion of the particle therapy will be focused on intrahepatic nodule with MVI. A 1cm margin will be taken as a clinical target volume margin for the feeding nodule, and 2cm margin alongside the vessel for the MVI lesion. Internal motion will be compensated according to 4D-CT movement assessment. Inter-fractional margin will be set to 3mm and combined with internal motion compensation forming a field specific planning treatment volume. Dose constraints for OARs will be prioritized over target volume coverage.

If both of Cohort A and B regimens were confirmed tolerable after DLT assessment, additional patients will be enrolled in Cohort B up to a total of 15 subjects. If only Cohort A regimen was determined to be tolerable, additional patients may be enrolled in Cohort A up to a total of 15 subjects.

Schedule for administration of investigational drugs and carbon ion radio therapy（Figure1）

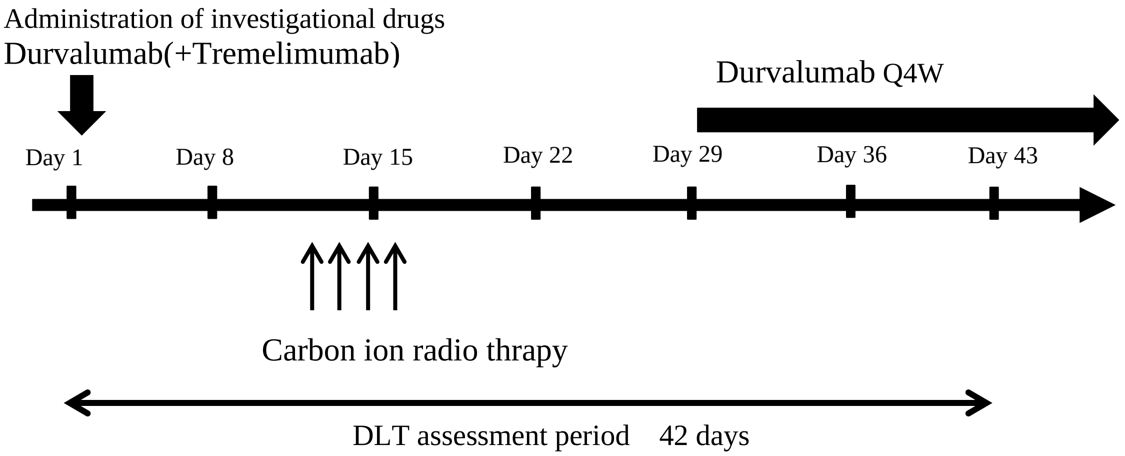

Tumor assessments, based on RECIST v.1.1 and mRECIST, will be performed every 6 weeks (Q6W) (±1 week) for the first 12 weeks from the date of randomization and then Q8W (±1 week) thereafter until RECIST 1.1-defined radiological progression followed by a subsequent scan if clinically feasible, evaluated by Confirmation of Radiological Progression criteria (Appendix B). Patients who permanently discontinue study drug(s) for reasons other than PD should continue to have radiographic scans performed per their original schedule until confirmed PD

Subjects with rapid tumor progression or tumor-associated syndromes requiring urgent medical intervention (e.g., central nervous system metastases, respiratory failure due to tumor compression, spinal cord compression) will be deemed ineligible for continued durvalumab.

The overview of this clinical trial is as follows.

Cohort A

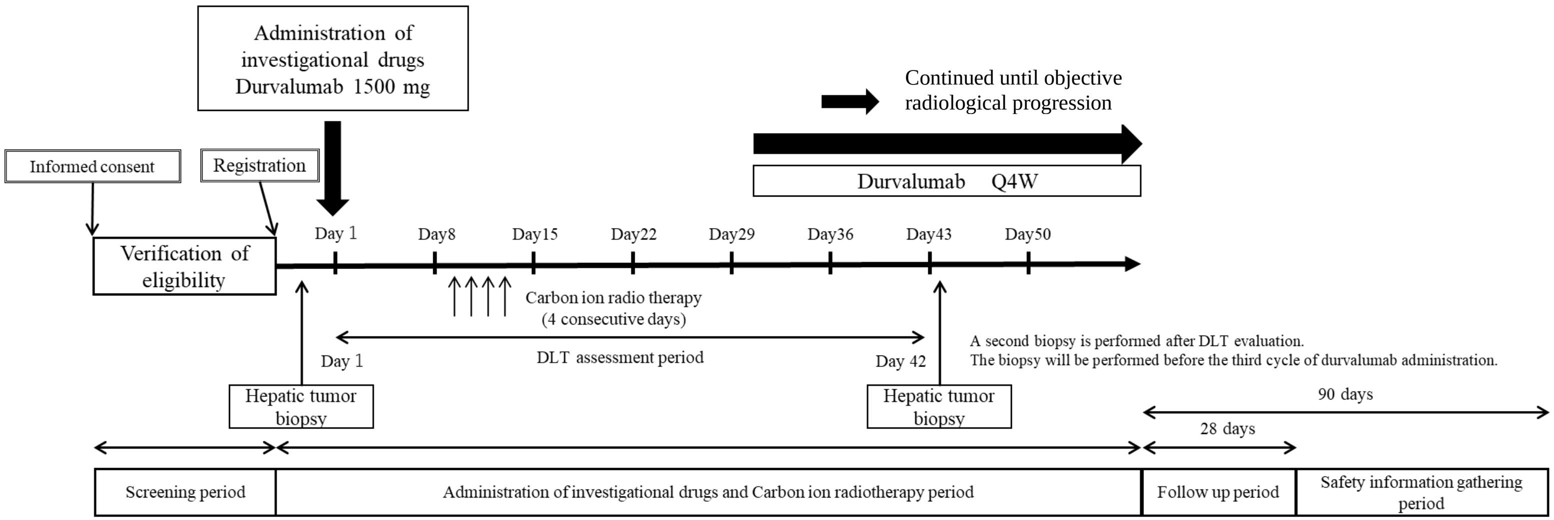

Cohort B

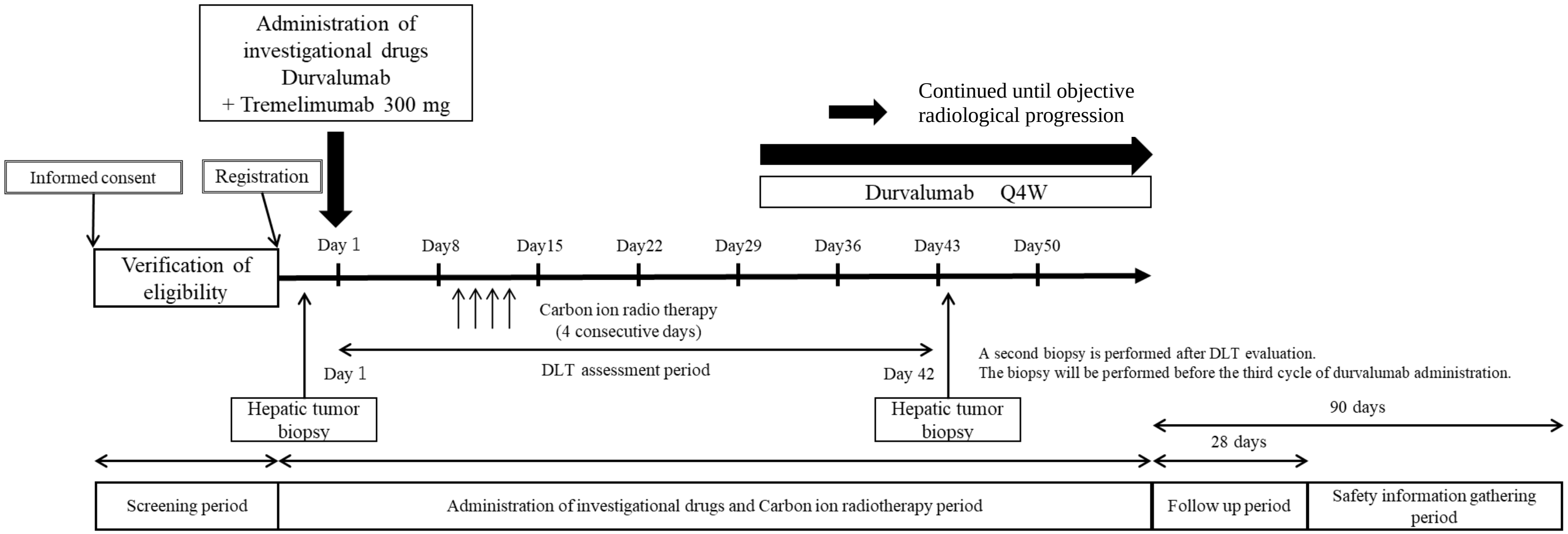

5.2. Target number of subjects and study duration

Target number of subjects：15

Study Period：
Estimated study start date 30 Apr 2021
Estimated study completion date 30 Sep 2023
Subject registration period:
Estimated date of first patient enrolled 30 Apr 2021
Estimated date of last patient enrolled 31 Mar 2023

Study schema

This study consists of four periods: the screening period, DLT assessment period, durvalumab q4W dosing period, and follow up period.

Signed informed consent will be obtained and patients will be screened prior to enrollment. The investigational treatment will include carbon ion radiotherapy on or after day 8 of cycle 1 after the first dose of study drug; the DLT evaluation period is 42 days from the first dose of durvalumab on day 1 of cycle 1. After confirmation of the safety of Cycle 1, durvalumab will be administered sequentially in Q4W beginning on Day 1 of Cycle 2. If multiple DLTs are observed in each cohort, an independent data monitoring committee will be convened to provide input on the determination of intolerability.

If no DLT is observed after the DLT evaluation period, durvalumab will be continued every 4 weeks until objective confirmation of disease progression (7.4) or until the criteria for 5.6 are met. Post-study follow-up for individual cases will occur 28 days after discontinuation of investigational drug (date of decision to discontinue). Adverse events and serious adverse event outcomes in subjects will be collected 90 days after discontinuation of study treatment (date of decision to discontinue) or until initiation of alternative anticancer therapy.

Figure2.

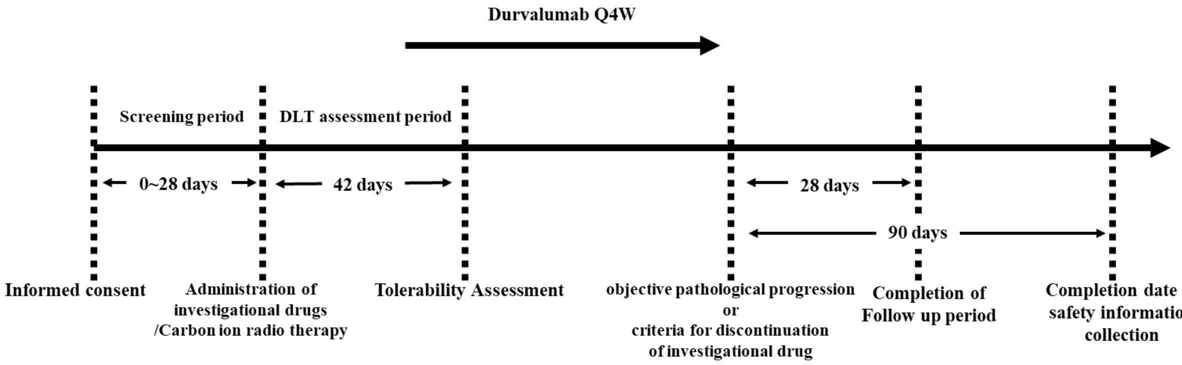

5.3. Monitoring for safety assessment

In situations where the below information is obtained and study patients are placed at under risk, or the continuation of the clinical trial is determined to be difficult, the Principal Investigator may decide on the termination or suspension of the entire clinical trial upon discussion with the Investigators. In addition, the study may be stopped based on the opinions by the DMC.

1. Occurrence of an unpredictable serious adverse reaction
2. Any information that indicates that the number, frequency, and condition of predictable serious adverse reactions cannot be predicted from the investigator’s brochure

3. SAEs that have been determined to have no causal relations with the IP, but are later determined that there is a reasonable possibility, due to the number, frequency, and condition of occurrences
4. Research reports indicating the tendency of the number, frequency, and condition of occurrences of adverse reactions having changed drastically
5. Research reports indicating the possibility that cancer, other serious diseases, disabilities, or death may occur
6. Information indicating that efficacy of the study drug cannot be expected in this clinical trial
7. Information indicating that the IP does not have any efficacy or effect on the target disease of the clinical trial
8. Information on any of the following related to marketed drugs that include the same ingredients as the IP:
    - Termination of manufacturing, import, or retail
    - Collection or disposal
    - Any other measures taken against health and hygiene related risks

Regardless of the reason for discontinuation, all data available to the subject at the time of discontinuation must be documented in the eCRF. All reasons for discontinuation should be documented. In terminating a trial, the investigator ensures that the protection of the subject's interests is given due consideration.

5.4. Institutional and case registration methods

Site registration and case registration will be conducted under the central registration system at the Data Management Office, Department of Clinical Trials, Chiba University Hospital. Once the site registration is completed, case registration will be available from the site. The following procedures will be used for site registration by fax and case registration via the Web.

5.4.1. Facility registration

1) The investigator at each participating site shall send a copy of the approval letter from the investigational review committee and a request form for site registration to the site registration center by fax after approval is obtained from the investigational review committee at the site.
2) The site registration center registers the sites and sends a notification of completion of site registration to the investigator.

Registration Center (Department of Data Management, Department of Clinical Trials, Chiba University Hospital)<br>fax number: 043-222-1207<br>Tel：043-222-1206<br>※Open hours are Monday through Friday, 9:00 a.m. to 5:00 p.m. (except Saturdays, Sundays, national holidays, and year-end and New Year holidays)<br>If a fax is received outside of the receptionist's office hours, it will be accepted on the next working day. (except at the beginning of the New Year)

5.4.2. Subject resistration

1) The investigator or subinvestigator obtains written consent and registers the subject in the case registration system. The case registration will be done via the website. After registration, a screening test is conducted to confirm that subjects meet the selection criteria and do not violate the exclusion criteria.

2) The investigator, subinvestigator or collaborator accesses the designated URL and enters the information necessary for case registration on the website. The investigator or sub investigator confirms the eligibility determination on the screen, and if the subject is determined to be eligible, protocol treatment is initiated. Once a subject is enrolled, enrollment will not be cancelled.

＊ The investigator or sub-investigator shall not administer the investigational drug until the subject is enrolled and “eligible” by screening test.

Registration Web site（DATATRAK Enterprise Cloud）<br>URL：https://secure.datatrak.net<br>TEL：043-222-1206<br>※Open 365 days a year, 24 hours a day, including Saturdays, Sundays, and holidays<br>(except for system downtime due to maintenance checks, etc.)

5.4.3. Handling of subjects who are found to be ineligible

Subjects who do not meet "eligibility" for any reason, such as ineligibility, are not eligible for enrollment and administration of the investigational product. The investigator or sub-investigator will explain to the subject that he/she is not eligible for enrollment in the study. The investigator or sub-investigator will also record the reason for the subject's ineligibility in the source documents.

If a subject who does not meet all eligibility criteria is inadvertently enrolled or started on the investigational product, the investigator will discuss whether or not to continue the investigational product. The investigator will ensure that all decisions resulting from this discussion are properly documented. If consensus cannot be reached, administration of the investigational product to the subject will be discontinued.

5.5. Dosing schedule and dosage/administration method

- Durvalumab 1,500 mg single agent arm (Cohort A)

  Cycle 1 Durvalumab 1,500 mg intravenous infusion will start on Day 1; after the first dose of durvalumab, heavy ion therapy will be administered on or after Day 8 of Cycle 1. Cycle 2 durvalumab will be administered after the safety of Cycle 1 is confirmed. Thereafter, dosing will be continued every 4 weeks until objective disease progression is confirmed (7.4.), “5.6. Discontinuation of Investigational Drug” is met, and the study is terminated.

- Durvalumab 1,500 mg + tremelimumab 300 mg once combination therapy (Cohort B)

  Cycle 1 One dose of combination therapy with durvalumab 1,500 mg and tremelimumab (both intravenous infusions) will be administered starting on Day 1. Durvalumab infusion will be started approximately 1 hour (maximum 2 hours) after completion of tremelimumab administration; after the first dose of durvalumab plus tremelimumab, heavy ion therapy will be administered on or after Day 8 of Cycle 1. Begin durvalumab 1,500 mg monotherapy Q4W after Cycle 2. Cycle 2 durvalumab will be administered after the safety of Cycle 1 is confirmed. Thereafter, durvalumab will be administered every 4 weeks until the objective disease progression is confirmed (7.4.), “5.6. Discontinuation of Investigational New Drug” is met, and the study is terminated.

5.5.1. Criterion for reduction

No dose reductions will be made for either durvalumab or tremelimumab in this study.

5.5.2. Criterion for drug withdrawal

With reference to Appendix 2 "Guidelines for Toxicity Management", investigators and subinvestigators may suspend the investigational drug.

5.6. Discontinuation of Investigational Drug

If any of the following criteria are met, the investigator or sub-investigator will discontinue the investigational product and perform an Investigational Product Discontinuation Study. A follow-up visit will be scheduled 28 days after discontinuation of investigational drug (date of decision to discontinue).

1) When a subject requests to withdraw from the clinical trial treatment.
2) When it is difficult to continue administration of the investigational drug due to exacerbation of complications
3) When it is difficult to continue administration of the investigational drug due to adverse events.
4) Pregnancy is detected.
5) Other cases in which the investigator or subinvestigator judges it necessary to discontinue the administration of the investigational drug.

Subjects may discontinue investigational treatment at any time, at their own discretion, without prejudice to subsequent treatment. Subjects who decide to discontinue an investigational drug should always be questioned about the reason for discontinuation and the presence or absence of AEs. Subjects who completely discontinue subsequent doses of study medication, regardless of the reason, must continue to attend the clinic for evaluation according to the study protocol. If the subject does not agree to continue the visit, the follow-up procedure may be modified to ensure collection of endpoints and safety information. This follow-up may include telephone contact with the subject, contact with relatives or the treating physician, or information from medical records. Any change in the method of follow-up should be documented in the medical record. Subjects who agree to the change in follow-up are not considered to have withdrawn their consent or to have withdrawn from the trial.

Subjects who discontinue continued treatment with the investigational drug for any reason will be identified as treatment discontinuation. Subjects who discontinue treatment will be transferred to the follow-up period (refer to 7.1.).

Subjects who discontinue treatment for reasons other than objective tumor progression as assessed by imaging will continue to receive imaging every 6 weeks (±1 week) until 12 weeks after study drug initiation, then radiologic PD with imaging every 8 weeks (±1 week), plus additional imaging as defined in the study plan, or death (whichever occurs first), RECIST assessment will continue.

If a subject is determined to have PD as defined by RECIST 1.1, additional imaging studies should be performed within 4 weeks of the determination. (refer to 7.4.).

All subjects will be followed for survival until the end of the trial. Subjects who are unable to return for evaluation will be contacted by telephone as indicated in the trial schedule as an alternative.

5.7. Discontinuation of individual cases from participation in clinical trials

If any of the following criteria are met, the investigator or subinvestigator will discontinue administration of the investigational drug and the subject's participation in the clinical trial.

1) When it is judged difficult to continue the clinical trial for some reason on the subject's side, such as non-attendance or transfer to another hospital.
2) 2When the subject requests to discontinue participation in the clinical trial.

3) When the investigator or subinvestigator determines that the subject is unable to continue the clinical trial.
4) If the subject weighs less than 30 kg
5) When the investigator/divisional investigator determines that a decision to discontinue the study is necessary due to a serious violation of the study protocol, etc.

5.7.1. In case of untraceable cases

A subject is considered lost only if the subject cannot be contacted until the end of the trial and there is insufficient information to determine the subject's status at that time. Subjects who refuse to continue participation in the trial (including telephone contact) will be recorded as "withdrawing consent" rather than "untraceable". Investigators will document the means used to re-establish contact with subjects lost to follow-up throughout the duration of the trial. If the subject who was lost to follow-up is re-contacted, the subject will not be marked as "lost to follow-up" and the evaluation will resume according to the study protocol.

At the time of the OS analysis, the survival status of all subjects in the overall analysis population (FAS) and the safety analysis population will be reconfirmed. Subjects who withdrew consent and subjects classified as "possibly untraceable" will also be subject to this reconfirmation.

5.7.2. Withdrawal of consent

The subject is free to withdraw consent for this clinical trial at any time without prejudice to subsequent treatment.
Subjects who withdraw their consent will not receive any further doses of the investigational drug or follow-up as specified in the protocol. However, consent for survival follow-up will be confirmed separately. Additional tests may be performed after discontinuation to ensure subject safety.

If a subject withdraws consent, the investigator must confirm the reason and the presence or absence of adverse events.

The subject withdrawing consent shall be specifically asked about the following items regarding the details of the withdrawal of consent.

- Withdraw consent for all further participation in the clinical trial, including subsequent follow-up (e.g., telephone calls to investigate survival status).
- Withdraw consent for use of clinical trial data.
- Withdraw consent for the use of any sample.

5.7.3. Clinical investigator's decision

If the risks of participation in a clinical trial outweigh the benefits of the subject's participation in the trial, such as if a life-threatening infusion reaction or systemic infection occurs, the investigator will determine that the subject's participation in the trial cannot continue.

5.7.4. Subject weight loss

If the subject weighs less than 30 kg after enrollment, he/she will be removed from the study.

5.7.5. Other cases

If the investigator determines that the subject is unable to continue participation in the study for other reasons, such as a serious deviation from the protocol, the subject will be removed from the study.

5.8. Subject replacement

If a subject is found to be ineligible for the clinical trial prior to enrollment, the subject will not be enrolled in this trial. In such cases, the investigator will explain to the subject that enrollment in this trial is not possible.

For subjects who do not complete the study treatment for reasons other than DLT criteria during the DLT evaluation period, the investigator will seek input from the Data Monitoring Committee on whether to recruit a new subject to replace that subject.

5.9. Concomitant Restricted Drugs and Concomitant Restricted Therapy

The coordinating investigator must be informed of all medications taken from the time of screening until the end of the clinical phase (last visit) as soon as possible. All concomitant medications, including herbal medications taken during the trial are recorded in the CRF.

Restricted, prohibited, and permitted concomitant medications are listed in Tables 1 and 2.

Table1. 5.9. Concomitant Restricted Drugs and Concomitant Restricted Therapy

Prohibited drug	Rules of use
Anticancer drugs as investigational drugs other than the investigational drugs in this study	Concurrent use is prohibited during administration of investigational drugs.
mAb against CTLA-4, PD-1 or PD-L1 other than the investigational drug mAb for PD-L1 in this study	Concurrent use is prohibited during administration of investigational drugs.
Any concomitant chemotherapy, radiation therapy, immunotherapy, biologic therapy, or hormonal therapy for the treatment of cancer other than the investigational drug in this study	Any concomitant chemotherapy, radiation therapy, immunotherapy, biologic therapy, or hormonal therapy for the treatment of cancer other than the investigational drug in this study
Immunosuppressive agents such as systemic corticosteroids, methotrexate, azathioprine, or tumor necrosis factor alpha inhibitors at doses of prednisone or its equivalent greater than 10 mg/day<br>Immunosuppressive agents such as systemic corticosteroids, methotrexate, azathioprine, or tumor necrosis factor alpha inhibitors in doses greater than 10 mg/day. Immunosuppressive agents such as, but not limited to, the following	Concomitant administration or premedication is prohibited. The following exceptions are permitted<br>・ Use of immunosuppressive agents for the management of adverse events related to the investigational drug<br>・ Use in subjects allergic to contrast media<br>・ Use of inhaled, topical, and intranasal corticosteroids<br>・ Non-immunotherapy that is clinically necessary and has occurred in the subject<br>・ Temporary use of steroids is acceptable if deemed essential for the management of related events (e.g., chronic obstructive pulmonary disease, radiation therapy, nausea)
Epidermal Growth Factor Receptor Tyrosine Kinase Inhibitors（Epidermal Growth Factor Receptor Tyrosine Kinase Inhibitors (EGFR TKIs)	Prohibit concomitant use.<br>Use with caution for 90 days after the last dose of durvalumab. An increased incidence of pulmonary inflammation (in combination with a third-generation EGFR TKI) and an increased incidence of transaminases (in combination with a first-generation EGFR TKI) have been reported when durvalumab is used concomitantly.
Attenuated live vaccine	Concomitant use of the investigational drug is prohibited until 30 days after the last dose of the investigational drug.
Drugs with laxative action and herbs or natural remedies for constipation	Use with caution during participation in clinical trials.

Prohibited drug	Rules of use
Blood transfusion (red blood cell concentrate, platelets)	Concomitant use is prohibited during the DLT evaluation period.
Granulocyte colony-forming stimulating factor (G-CSF) preparation	Concomitant use is prohibited during the DLT evaluation period.

Table2. Concomitant tolerated drugs

Tolerated Drugs	Rules of use
Concomitant medications or treatments (e.g., acetaminophen or diphenhydramine) as deemed necessary for appropriate prophylactic or symptomatic treatment. However, drugs included in the "Prohibited Drugs" section above are excluded.	Administer according to the prescription of the investigator, etc.
Best supportive care (including antimicrobials, nutritional support, correction of metabolic disturbances, optimal symptom control and pain management [e.g., palliative radiation therapy for non-target lesions])	For all subjects, use when necessary.
Inactivated viruses such as influenza vaccines	Can be inoculated (e.g. with vaccine)

5.10. Follow-up treatment

Post-treatment after completion or discontinuation of the clinical trial is not specified.

5.11. After discontinuation of this clinical trial

Subjects who discontinue the clinical trial will be subjected to necessary examination and observation, and appropriate measures will be taken as necessary, until it can be medically determined that the subject can be discharged or transferred to a hospital.

6. Clinical trial treatment

6.1. Durvalumab and tremelimumab

Refer to the investigator's brochure for details and handling of the investigational drug. The following are the investigational drugs to be used in this clinical trial.

6.1.1. Durvalumab

Durvalumab (MEDI4736) will be supplied by AstraZeneca as a 500-mg vial solution for infusion after dilution. The solution contains 50 mg/mL durvalumab, 26 mM histidine/histidine hydrochloride, 275 mM trehalose dihydrate, and 0.02% weight/volume (w/v) polysorbate 80; it has a pH of 6.0 and density of 1.054 g/mL. The nominal fill volume is 10.0 mL.

Durvalumab is a sterile, clear to opalescent, colorless to slightly yellow solution, free from visible particles.

Investigational product vials are stored at 2°C to 8°C (36°F to 46°F) and must not be frozen. Investigational products should be kept in original packaging until use to prevent prolonged light exposure.

6.1.1.1. Preparation of Durvalumab Dose by Infusion Bag

Each dose of durvalumab must be prepared by aseptic manipulation by the investigator or other investigational drug administrator designated by the site. The time between the puncture of the vial and the start of administration of Durvalumab must not exceed the following

- 2°C to 8°C for 24 hours
- 4 hours at room temperature
- Dosing solutions must be brought to room temperature before administration.

Dose 1,500 mg of durvalumab is administered with an infusion bag containing 0.9% saline or 5% dextrose to achieve a final concentration of 1 to 20 mg/mL of durvalumab and an intravenous administration set with a 0.2 μm or 0.22 μm filter. Add 1,500 mg of durvalumab (i.e., 30.0 mL of durvalumab) to the infusion bag. The infusion bag should be selected to achieve a final concentration within 1 to 20 mg/mL. Gently invert and mix the infusion bag until the administered fluid in the bag is uniform. The standard infusion time is 1 hour (±5 minutes). If the infusion is interrupted, the total infusion time should not exceed 8 hours at room temperature. No other drugs should be administered simultaneously in the same IV line.

After the contents of the infusion bag have been completely administered, flush the IV line with an IV diluent equal to the priming volume of the IV set used, or complete the infusion according to the provider's policy to ensure that the full volume is administered. If the line is not flushed, document this in the record. If the preparation time or infusion time limit is exceeded, a new vial must be used to prepare a new dose solution. Since durvalumab does not contain preservatives, unused preparation solutions must be discarded.

The preparation should be made in accordance with the “Procedures for the Administration of Investigational Drugs” for this clinical trial.

6.1.2. Tremelimumab

Tremelimumab will be supplied by AstraZeneca either as a 400-mg or a 25-mg vial solution for infusion after dilution. The solution contains 20 mg/mL tremelimumab, 20 mM histidine/histidine hydrochloride, 222 mM trehalose dihydrate, 0.27 mM disodium edetate dihydrate, and 0.02% weight/volume (w/v) polysorbate 80; it has a pH of 5.5 and density of 1.034 g/mL. The nominal fill volume is 20.0 mL for the 400-mg vial and 1.25 mL for the 25-mg vial.

Tremelimumab is a sterile, clear to opalescent, colorless to slightly yellow solution, free from or practically free from visible particles.

Investigational product vials are stored at 2°C to 8°C (36°F to 46°F) and must not be frozen.

Investigational products should be kept in original container packaging until use to prevent prolonged light exposure.

6.1.2.1. Preparation of Tremelimumab Dose by Infusion Bag

Each dose of tremelimumab must be prepared by aseptic manipulation by the investigator or the investigation drug manager designated by the site. The time from vial puncture to administration must not exceed

- 2°2°C to 8°C for 24 hours
- 4 hours at room temperature
- Doing solutions must be brought to room temperature before administration.

Tremelimumab is administered in an infusion bag containing 0.9% saline or 5% dextrose to achieve a final concentration of 0.10-10 mg/mL of tremelimumab, using an intravenous administration set with a 0.2 µm or 0.22 µm filter.

Add 300 mg of tremelimumab (i.e., 15.0 mL) to the infusion bag. Gently invert and mix the infusion bag until the dosing solution in the bag is homogeneous. The standard infusion time is 1 hour (±5 minutes); any infusion time less than 55 minutes will be considered a deviation from the study protocol. If the infusion is interrupted, the total infusion time should not exceed 8 hours at room temperature. No other drugs should be administered simultaneously in the same IV line. After the contents of the infusion bag have been completely administered, flush the IV line with an IV diluent equal to the priming volume of the IV set used, or complete the infusion according to the site's policy to ensure that the full volume is administered. If the line is not flushed, document this. If the preparation time or infusion time limit is exceeded, a new vial must be used to prepare a new dose solution. Since tremelimumab does not contain preservatives, unused preparation solutions must be discarded. The preparation should be made in accordance with the “Protocol for the Administration of Investigational Medicinal Products” for this clinical trial.

6.2. Control Drugs

There is no control drug in this study.

6.3. Monitoring during administration

During and after the infusion, the subject's condition should be monitored by assessment of vital signs at the times specified in the study protocol.

If an infusion-related reaction of grade 2 or less is observed, the infusion rate of the study drug may be reduced by 50% or discontinued until the event is resolved, and the infusion may be restarted at 50% of the initial infusion rate until the infusion is completed. Subjects who experience an infusion-related reaction of grade 2 or less may receive subsequent infusions at 50% of the initial rate. Acetaminophen and/or antihistamines (e.g., diphenhydramine) or equivalent drugs according to institutional standards may be administered at the investigator's discretion. If infusion-related reactions are grade 3 or greater, the investigational drug should be discontinued. The standard infusion duration is 1 hour, but if interrupted, the infusion should not exceed 8 hours at room temperature. Refer to the Toxicology and Management Guidelines in the protocol appendix for management of subjects who experience an infusion-related reaction.

As with other antibodies, allergic reactions to dose administration may occur. Appropriate drugs and medical devices to treat acute anaphylactic reactions must be readily available, and investigators must be trained to recognize and treat anaphylaxis. The site must have immediate access to an emergency resuscitation team and medical equipment, and the ability to admit subjects to the intensive care unit if necessary.

6.4. Management of investigational drugs

1) The investigator coordinator will deliver the investigational drug to the investigator of Chiba University Hospital in accordance with the agreement with the investigational drug

provider.

2) The investigator coordinator will properly manage the investigational drug in accordance with the protocol provided by the investigator through the site director.
3) The investigator shall prepare a document explaining the storage conditions, expiration date, and other handling methods of the investigational product and deliver it to the site manager, investigators collaborators, and investigational product manager.

6.5. Disposal of unused investigational drugs

1） After the investigational monitor checks the inventory and obtains the investigator's approval, the investigational drug manager discards used, unused, expired, or damaged investigational drugs and empty containers.
2） The investigational drug manager shall dispose of the investigational drug in accordance with the guidelines for disposal of pharmaceuticals.

6.6. Packaging and labeling of investigational drugs

The label should indicate that the product is for investigational use, the name, title and address of the coordinating investigator (representative), chemical name, volume, serial number, storage method and expiration date of the investigational drug. The label should appear on the package insert.

Labels for investigational new drugs shall be prepared in accordance with “Good Manufacturing Practice (GMP)” and GCP ordinances. The investigational drug label should be written in Japanese.

Investigational drug: Durvalumab (genetical recombination) (MEDI4736) + Tremelimumab (genetical recombination)

The drug name on the durvalumab label shall be “MEDI4736” or “Durvalumab (MEDI4736)”.

6.7. Carbon ion radiotherapy

Information on Carbon ion radotherapy
- Name of medical device: Cardon ion radiotherapy Device
- Indications: Treatment of solid tumors
- Model Number: CI-1000S
- PMDA approval number: 22800BZX00096000
- Manufacturing facility: Toshiba Energy Systems&Solutions Corporation

Carbon Ion Therapy will be performed at the Quantum Science and Technology Agency QST Hospital, and irradiation will be performed for four consecutive days between Day 8 and Day 14 of Cycle 1 as per the schedule in Section 7.1. (excluding holidays)

Dose prescription and fractionations will be 60Gy (RBE) / 4Fr / 1week for CIRT. RBE calculation is done by modified microdosimetric kinetic model (Inaniwa et al. Phys Med Biol. 2010). Pencil beam scanning technique will be used. The pencil beam covers the PTV voxel by voxel in successive layers. An optimization function drives the dose distribution in each treatment spot to reach the desired target coverage and organs at risk sparing. If a coil or other device implanted in the previous treatment is considered to be substitutable as a marker for Carbon ion radiotherapy, the marker may be omitted after consultation with the investigators.

All cases will undergo fiducial marker insertion prior to treatment preparation. Fiducial markers may be implanted under ultrasonography/fluoroscopy surveillance percutaneously or transarterialy.

For cases treated in an orthogonal fixed beam room, immobilization will be achieved with a relatively thick shell (3-mm thickness) made of a low-temperature thermoplastic and hydraulic urethane resin or vacuum-formed cushion to allow a range of beam angles by rotating the treatment

table. However, a thinner shell may be used if a rotating gantry is available. In either case, the shell device is fixed by tapping to the table bottom, with tightening or loosening adjustment as required.

A 4D-CT simulation is required to allow for assessment of tumor motion. Fasting for 3 to 8 hours prior to simulation is required to control stomach/duodenum volume. Simulation CTs should be done with a CT slice thickness no greater than 3 mm. The 4D dataset is separated into 10 separate breathing phase bins. The simulation scan used for planning should NOT be performed with intravenous contrast; simulation CT after this planning CT may include contrast (for anatomic information).

Target lesion of the particle therapy will be focused on intrahepatic nodule with MVI. A 5mm margin will be taken as a clinical target volume margin for the feeding nodule, and 1cm margin alongside the vessel for the MVI lesion. Internal motion will be compensated according to 4D-CT movement assessment. Inter-fractional margin will be set to 3mm and combined with internal motion compensation forming a field specific planning treatment volume.

Dose constraints for risk organs are set as follows

- GI tract: D2cm3 ≤ 30Gy (RBE)
- Spinal cord: Dmax ≤ 25Gy (RBE)
- Remnant liver volume (liver volume receiving 30Gy (RBE) or less): 500cm3

In addition, the following liver volume information is collected in conjunction with the remaining liver volume

- Liver V5 Gy (RBE)
- Liver V20 Gy（RBE）

Confirmation of patient positioning is confirmed by orthogonal X-ray images. A maximum displacement of 3 mm in all directions is allowed between the reference and treatment images and is achieved by movement of the couch. Respiratory gating during treatment is mandatory. External respiratory surrogate systems or fluoroscopic tracking may be used for respiratory motion detection.

6.8. subject inclusion

In this “modified 3 + 3 design”, the first three subjects will be enrolled in cohort A. If no DLT is observed in any of these subjects, the trial will enroll additional subjects in Cohort B, who will also receive tremelimumab as follows.

If one subject develops DLT in any cohort, three additional subjects will be enrolled in that same cohort. The development of two or more DLTs in Cohort A will mean that the entire trial will be terminated, and two or more DLTs in Cohort B will indicate that the MTD has been exceeded and the regime in Cohort B will be discontinued. In that case, up to a total of 15 additional subjects will be enrolled in Cohort A.

Schematic of subject incorporation

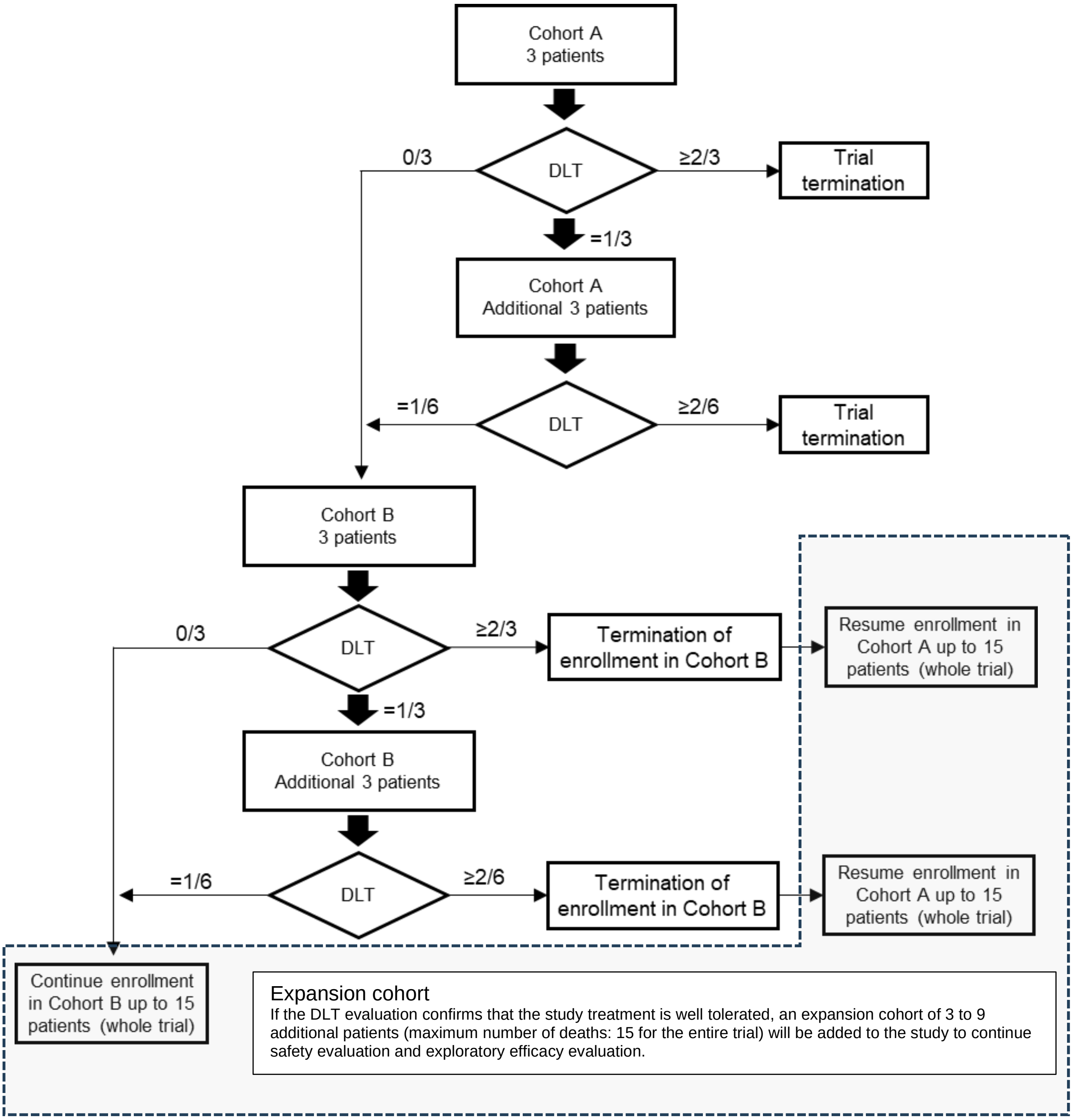

6.9. Definition of Dose-Limiting Toxicity (DLT)

During the 42-day period from the start of study drug administration (Cycle 1/DLT evaluation period), the following toxicities associated with the study treatment are considered DLTs: Grade determination follows CTCAE ver. 5.0.

Dose-limiting toxicity (DLT) will be assessed during the DLT evaluation period of this study, which will be 42 days from the time of the first dose on Cycle 1, Day 1. The severity of DLT will follow the guidelines described in the CTCAE ver. 5.0.

DLT is defined as the occurrence of an adverse event (AE) that is at least potentially related to the investigational drug or regimen (IR), with two exceptions: a Grade of vitiligo or alopecia is not a DLT. An AE that is at least potentially related to a regimen containing durvalumab and/or tremelimumab will be evaluated as a DLT if it meets one of the following criteria.

If a patient initiates heavy ion therapy but does not complete heavy ion therapy within the allowed time due to an adverse event that cannot be excluded as being causally related to durvalumab, tremelimumab, or heavy ion therapy, such adverse event will be considered a

DLT.

- **Hematologic toxicity:**
  - Grade ≥3 neutropenia complicated by fever >38.3°C
  - Grade 4 neutropenia (lasting more than 7 days)
  - Grade ≥3 thrombocytopenia with significant bleeding
  - Grade 4 thrombocytopenia (regardless of duration)
  - Grade 4 anemia (regardless of duration)
  -
- **Non-hematologic toxicity:**
  - Any Grade 4 non-immune-mediated AE
  - Any Grade 4 immune-mediated AE, excluding endocrinopathies
  - Any Grade 3 non-immune mediated AE that does not resolve to ≤Grade 1 or baseline within 30 days with optimal medical management
  - Any Grade 3 immune-mediated AE – excluding diarrhea/colitis, pneumonitis, hepatitis, rash, neurotoxicity, myocarditis, myositis/polymyositis, endocrinopathies and nephritis – that does not resolve to ≤Grade 1 or baseline within 30 days after onset of the event despite optimal medical management including systemic corticosteroids
  - Grade 3 diarrhea or colitis that does not resolve to ≤Grade 1 within 14 days [both immune- and non-immune-mediated indicated here; the same is the case if not specified in remaining bullet points below]
  - Grade 3 noninfectious pneumonitis
  - Grade 2 noninfectious pneumonitis that does not resolve to ≤Grade 1 within 3 days of the initiation of maximal supportive care
  - Aspartate aminotransferase (AST) or alanine aminotransferase (ALT) ≥3×ULN with concurrent increase in total bilirubin (TBL) ≥2×ULN without evidence of cholestasis or alternative explanations (e.g., viral hepatitis, disease progression in the liver; i.e., "Hy's Law")
  - ALT or AST >8×ULN or TBL>5×ULN
  - Grade 3 immune-mediated rash that does not resolve to ≤Grade 1 or baseline within 30 days
  - Grade 2 rash covering > 30% BSA that does not resolve to ≤Grade 1 or baseline within 30 days
  - Any grade of immune-mediated rash with bullous formation
  - Grade 3 immune-mediated neurotoxicity (excluding Guillain-Barre and myasthenia gravis) that does not resolve to ≤Grade 1 within 30 days
  - Grade 2 or 3 immune-mediated peripheral neuromotor syndrome (such as Guillain-Barre and myasthenia gravis) that does not resolve to ≤Grade 1 within 30 days or that exhibits signs of respiratory insufficiency or autonomic instability
  - Grade 3 immune-mediated myocarditis
  - Any symptomatic immune-mediated myocarditis that does not become asymptomatic within 3 days of initiating optimal medical management including systemic corticosteroids
  - Grade 2 or 3 immune-mediated myositis/polymyositis that does not resolve to Grade ≤1 within 30 days of initiating optimal medical management including systemic corticosteroids or that exhibits signs of respiratory insufficiency regardless of optimal medical management
  - Immune-mediated increase in creatinine >3×ULN, or >3×baseline for patients with a baseline creatinine elevated above ULN
  - Transfusion (Red cell concentrate, Platelet), or the use of G-CSF during DLT period

The DLT evaluation period will be from the time of the first dose of study drug/IR until 42 days post-dose. The first dose of durvalumab in Cycle 2 will be administered after the safety of Cycle 1

is confirmed.

The first treatment-related toxicity that occurs during the DLT period must be followed up to determine if the event qualifies as a DLT as defined in the DLT criteria above.

An immune-related adverse event is defined as an immune (inflammatory) adverse event without a definite other etiology. If an immune-related adverse event is suspected but there are no significant laboratory findings, a repeat laboratory test should be performed before a determination of DLT is made. Subjects who do not complete the DLT evaluation period for reasons other than DLT will not be considered DLT evaluable cases, and DLT will be evaluated after supplementation. The investigator will ask the Independent Data Monitoring Committee for an opinion on the supplementation decision.

Subjects will be administered the first dose of the study drug after hospitalization at Chiba University Hospital. After administration of the investigational drug, the subject will continue to be closely monitored in the hospital and will be transferred to the QST Hospital only for heavy ion therapy, taking into consideration the subject's condition and the means of transportation (taxi, private ambulance, etc.). After completion of heavy particle therapy, the patient should remain in the hospital for at least 7 days. During the transition to outpatient treatment, the investigator will perform the tests specified in 7.2.2 and confirm the safety of the treatment before allowing the patient to continue treatment as an outpatient.This inspection may be combined with the immediately preceding regulatory inspection.In addition, the investigator should be able to contact the site and the medical facility near the home in the event of an adverse event and establish a system to promptly contact the subject in an emergency.

6.10. Toxicity Management

Guidelines for the management of immune-mediated reactions, infusion reactions, and non-immune-mediated reactions to durvalumab are provided in the Durvalumab/Tremelimumab Toxicity Management Guidelines (TMGs).

Appropriate efforts should be made to thoroughly evaluate the subject and rule out neoplastic, infectious, metabolic, toxic, or other etiologies of imAE. Serologic, immunologic, and histologic (biopsy) data should be used to support the diagnosis of imAE, if appropriate. In the absence of a clear alternative etiology, the possibility of an immune-related etiology should be considered. In addition, there are situations in which durvalumab and tremelimumab should be discontinued (see Toxicity Management Guidelines). Dose reductions are not permitted. In case of doubt, consult the investigator. All toxicities will be evaluated according to CTCAE ver. 5.0.

6.11. Restrictions during the clinical trial

6.11.1. Restrictions during the clinical trial

The following restrictions apply during and before and after a given period of time while undergoing investigational treatment.

<u>About Women of Childbearing Potential</u>

Female subjects of childbearing potential who are not abstinent and who plan to have sex with an unprotected male partner must use at least one effective method of contraception from the time of screening until the drug treatment and drug discontinuation period (180 days after the last dose of durvalumab + tremelimumab combination therapy). Unprotected male partners of female subjects of childbearing potential must use a male condom and spermicide during this period. Discussion of stopping contraception after this time should be discussed with the family physician. Temporary abstinence, the rhythm method, and external ejaculation are not acceptable methods of contraception. Females should also not breast-feed during this period.

<u>About men with female partners of childbearing potential</u>

For non-contraceptive male subjects who are unprotected and plan to have sex with a female

partner of childbearing potential, the use of a male condom plus spermicide is mandatory from screening through the entire drug treatment and drug washout period (180 days after the last dose of durvalumab + tremelimumab combination therapy). However, periodic abstinence, rhythm and withdrawal methods are not acceptable methods of contraception. Male subjects will refrain from donating sperm during this period.

Female partners of male subjects (of childbearing potential) should also use highly effective contraceptive methods during this period.

Note: Pregnant women are defined as women who have not undergone sterilization (i.e., bilateral oophorectomy, bilateral oophorectomy, or total hysterectomy) or as premenopausal.

A woman is considered postmenopausal if she has been amenorrheic for 12 months without another medical cause. The following age-specific requirements apply

- Women under age 50 are considered postmenopausal if they have been amenorrheic for at least 12 months after discontinuation of exogenous hormone therapy and have luteinizing hormone and follicle stimulating hormone levels in the postmenopausal range of the institution.
- Women over age 50 are considered postmenopausal if they have been amenorrheic for at least 12 months after discontinuation of all exogenous hormone therapy, if they had radiation-induced menopause more than 1 year before their last menstrual period, and if they had chemotherapy-induced menopause more than 1 year before their last menstrual period.

A highly effective contraceptive method is defined as having a low failure rate (i.e., less than 1% per year) when used consistently and correctly.

6.12. Clinical Trial Procedures

The RECIST evaluation date must be performed as scheduled, regardless of any dosing delays. All other scheduled assessments must be performed at the start of the dosing cycle, and all laboratory and other tests required for dosing must be performed at least 3 days prior to dosing.

Subjects may be allowed to delay dosing under certain circumstances, as described below.

- With the judgment of the principal investigator and subinvestigator,dosing may be delayed for either immune- or non-immune-related AEs in accordance with toxicity management guidelines.
- If dosing must be delayed for reasons other than treatment-related toxicities, dosing should be resumed as soon as possible.
- The dosing interval may be shortened as clinically appropriate to gradually match the treatment cycle to the tumor response plan (RECIST). Based on the half-life of durvalumab and tremelimumab, the interval between two consecutive doses should not be less than 22 days (for durvalumab and tremelimumab, see the current investigator's brochure).

7. OBSERVATION, EXAMINATION, AND ASSESSMENT, METHODS, AND TIMING OF IMPLEMENTATION

7.1. Implementation Schedule and Procedures

Cycle	Screening period	First tumor biopsy ※only in consented patients		DLT evaluation period							Second tumor biopsy ※only in consented patients	Durvalumab q4W dosing period					ST	Follow up period（28 days after the last administration date）	Safety information collection（90 days after the last administration date）
				Cycle 1				Cycle 2 [*15]				Cycle 3	Cycle43	Cycle 5	Cycle 6	Cycle 6< until PD			
Cycle Day	D -28 ～D -1	D -28〜D -1		1	8 to 14	15	22	1	8	14	DLT評価終了後	1[*1]	1	1	1	1	—	—	—
Allowable period（Day）				—	±3	±3	±3	±3	±3	±3		—	±3	±3	±3	±3	+14	+14	+14
Informed Consent ／ Subject backgroud information／ Review of eligibility	●[*2]																		
Enrollment			●[*2]																
Durvalumab administration (cohort A and B)				●				●				●	●	●	●	●			
Tremelimumab administration (cohort B)[*3]				●															
Tumor biopsy		●									●								
CIRT					4days between Day8 and Day14[*4]														
Fiducial marker insertion[*5*6]		●																	
Fixation, simulation CT (for CIRT)[*5]	●																		
Weight[*7]	●			●	●	●	●	●	●	●		●	●	●	●	●	●	●	
Height	●																		
Physical exam	●			●	●	●	●	●	●	●		●	●	●	●	●	●	●	
Physical exam（Specific site based on case）	●			●	●	●	●	●	●	●		●	●	●	●	●	●	●	
Vital signs	●			●	●	●	●	●	●	●		●	●	●	●	●	●	●	
ECOG PS	●			●	●	●	●	●	●	●		●	●	●	●	●	●	●	
Clinical Chemistry／Hematology[*8]	●			●	●	●	●	●	●	●		●	●	●	●	●	●	●	
Coagulation	●			●	●	●	●	●	●	●		●	●	●	●	●	●	●	
Urinalysis	●			●								●	●	●	●	●	●	●	
ECG[*9]	●			(●)								(●)	(●)	(●)	(●)	(●)	(●)	(●)	
Hepatitis serology [*10]	●			(●)				(●)				(●)	(●)	(●)	(●)	(●)	(●)		
HIVtests	●																		
TSH、fT3、fT4 [*11]	●			●	●	●	●	●	●	●		●	●	●	●	●	●	●	
Pregnancy test [*12]	●			(●)				(●)				(●)	(●)	(●)	(●)	(●)			
Chest X ray	●																●	●	
Assesment of Child-Pugh score	●			●				●				●	●	●	●	●	●	●	
CT/MRI[*13]	●			←Every 6 weeks (±1 week) for the first 12 weeks from Cycle1 day1, and every 8 weeks (±1 week) thereafter until PD.→														●	
Tumor marker（AFP、PIVKA-II）	●							●				●	●	●	●	●			
Confirmation of survival																		●	
Cocmitant medication	←→																		
Assessment of AE/SAE[*14]	←→																		

*1. The baseline for the durvalumab Q4W dosing period will be the first day of the third cycle.
*2. To be performed after completion of all screening tests and prior to administration of Cycle1Day1.Whenever possible, minimize the time between enrollment and Cycle1Day1.
*3. In combination therapy, tremelimumab should be administered first, and durvalumab infusion should be started approximately 1 hour (maximum 2 hours) after tremelimumab administration is completed.
*4. Excluding holidays.
*5. To be administered to subjects who are eligible by other screening tests.
*6. If a coil or other device implanted in the previous treatment is considered to be substitutable as a marker for Carbon ion radiotherapy, the marker may be omitted after consultation with the investigators.
*7. Weight will be measured along with vital signs at the visit.
*8. Serum or plasma biochemical tests (including LFT monitoring) and blood tests may be performed more frequently if clinically indicated.
If screening biochemical and hematological evaluations were performed no more than 3 days prior to Day 1 (the first infusion day), they need not be performed again on Day 1.
Results of LFTs, electrolytes, complete blood count, and creatinine must be obtained prior to the start of infusion (within 3 days) and confirmed by the attending physician or investigator prior to administration.
*9. At a screening period,if clinically significant abnormalities are detected, two additional ECG measurements should be performed.
*10. If HBs-Ag, HBs-Ab, or HBc-Ab is positive, HBV-DNA should be measured (before and every 4 weeks after administration).
If HCV antibodies are positive, measure HCV-RNA (pre-test).
*11. Free T3 or free T4 should be measured only if TSH is abnormal or endocrine system-related AEs are clinically suspected. If TSH is measured up to 14 days prior to the first dose, it need not be measured again on Day 1.
*12. For women of childbearing potential only. For women of childbearing potential, a pregnancy test should be performed every 4 weeks starting 7 days prior to the first dose. Pregnancy tests can be performed on Day 1, but results must be confirmed by the treating physician or investigator before administration is initiated.
*13. RECIST evaluation is performed with CT (preferred) or MRI imaging of the chest, abdomen (including liver and adrenal glands), and pelvis IV contrast preferred. Imaging of the pelvis is recommended only if there is a possibility of primary or metastatic disease in the pelvic region. Additional anatomic imaging should be performed based on the individual subject's signs and symptoms at baseline and at follow-up. Baseline assessments should be performed no later than 28 days prior to the start of study drug administration for each cohort and should be performed as close as possible to the start of study drug or prior to the start of study drug if possible. Confirmatory testing should be performed within 4 weeks of the prior PD evaluation, preferably at the next scheduled imaging visit (provided there is no clinically significant deterioration). If an unscheduled evaluation is performed and the subject has not progressed, every effort should be made to perform a subsequent evaluation at the next scheduled visit.
*14. AEs and SAEs should be collected from the time of the first dose of study drug until the follow-up date 28 days after discoutinuation of study drug. However, AE and SAE outcomes should be collected until the end of the safety information collection period (90 days after the discontinuation of study drug) or until the start of alternative anticancer therapy. In addition, events occurring after the 28-day follow-up period after the discoutinuation of the study drug and considered to be attributable to delayed toxicity to the study drug will be collected as AEs or SAEs until the end of the safety information collection period or until the initiation of alternative anticancer therapy.
*15. *n the Expansion cohort, patients will be moved to Cycle 2 after the end of Cycle 1.
*16. After completion of heavy particle therapy, the patient should remain in the hospital for at least 7 days. During the transition to outpatient treatment, the investigator will perform the tests specified in 7.2.2 and confirm the safety of the treatment before allowing the patient to continue treatment as an outpatient. This inspection may be combined with the immediately preceding regulatory inspection.

Note: All assessments on the treatment day shall be performed prior to infusion unless otherwise indicated.

ECG Electrocardiogram; LFT Liver function tests; T3 Triiodothyronine; T4 Thyroxine; TSH Thyroid stimulating hormone.

7.2. Observation, tests and assessment

7.2.1. Screening period

The investigator or sub-investigator will perform the following screening tests and enroll subjects who meet the selection criteria and do not violate the exclusion criteria. The tests will be performed after consent is obtained and between 28 days before the start of the investigational drug and the day before the start of the investigational drug. Test items will be as described below. However, test results obtained prior to consent as part of routine medical care using procedures similar to those used in this study may be used as screening tests if they fall within the 28-day screening period.

- Informed Consent
- Subject background information*
- Review of eligibility criteria
- Complete physical exam
- ECOG Performance Status
- Vital signs**, weight and height
- Chest X ray
- 12-lead ECG (in triplicate [2-5 minutes apart])
- Assessment of Child-Pugh score
- Imaging by CT/MRI, if applicable to study
- Clinical laboratory tests for:
    - Clinical Chemistry (see Table 5)
    - Hematology (see Table 4)
    - TSH, fT3, fT4
    - Coagulation (PT, APTT, PT-INR)
    - Creatinine Clearance
    - Pregnancy test (for women of childbearing potential only)
    - Hepatitis serology（HBs antigen, HBs antibody, HBc antibody, and HCV antibody）
    - HIV test
    - Urinalysis
    - Tumor marker (AFP, PIVKA-II)
- Fiducial marker insertion
- Fixation, simulation CT (for CIRT)
- Concomitant medication

* **Subject background information and medical history**

Subject identification code number, race, sex, age at obtaining IC, medical history, concurrent diseases, alcohol consumption, smoking history, and information for HCC with initial diagnosis, pathological diagnosis and past treatment.

** **Vital signs**

Vital signs will be measured at every visit and will include assessments of systolic and diastolic BP, temperature, and HR. Systolic and diastolic BPs will be documented in mmHg. Temperature will be obtained in degrees Celsius. HR will be documented in beats per minute. Generally, each patient will have blood pressure tested in the same arm. Measurement device and measurement time will not be indicated.

7.2.2. DLT assessment period

Day1

- Durvalumab administration (cohort A and B)
- Tremelimumab administration (cohort B）
- Complete physical exam
- ECOG Performance Status
- Vitals signs and weight
- 12-lead ECG (if necessary)
- Assessment of Child-Pugh score
- Clinical laboratory tests for:
  - Clinical chemistry
  - Hematology
  - TSH、fT3、fT4
  - Coagulation（PT、APTT、PT-INR）
  - Creatinine Clearence
  - Pregnancy test (women of childbearing potential only)
  - Urinalysis
- Assessment of AE/SAE
- Confirmation of concomitant therapy

Day 8-14

- CIRT (60 Gy (RBE) / 4 Fr) (performed at QST hospital)
- Complete physical exam
- ECOG Performance Status
- Vitals signs and weight
- Assessment of Child-Pugh score
- Clinical laboratory tests for:
  - Clinical chemistry
  - Hematology
  - TSH、fT3、fT4
  - Coagulation（PT、APTT、PT-INR）
  - Creatinine Clearence
- Assessment of AE/SAE
- Confirmation of concomitant therapy

Day 15

- Complete physical exam
- ECOG Performance Status
- Vitals signs and weight
- Assessment of Child-Pugh score
- Clinical laboratory tests for:
  - Clinical chemistry
  - Hematology
  - TSH、fT3、fT4
  - Coagulation（PT、APTT、PT-INR）
  - Creatinine Clearence

- Assessment of AE/SAE
- Confirmation of concomitant therapy

Day 22

- Complete physical exam
- ECOG Performance Status
- Vitals signs and weight
- Assessment of Child-Pugh score
- Clinical laboratory tests for:
    - Clinical chemistry
    - Hematology
    - TSH、fT3、fT4
    - Coagulation（PT、APTT、PT-INR）
    - Creatinine Clearence
- Assessment of AE/SAE
- Confirmation of concomitant therapy

Day 29（Cycle 2 Day1）

- Durvalumab administration (cohort A and B)
- Tremelimumab administration (cohort B）
- Complete physical exam
- ECOG Performance Status
- Vitals signs and weight
- 12-lead ECG (if necessary)
- Assessment of Child-Pugh score
- Clinical laboratory tests for:
    - Clinical chemistry
    - Hematology
    - TSH、fT3、fT4
    - Coagulation（PT、APTT、PT-INR）
    - Creatinine Clearence
    - Pregnancy test (women of childbearing potential only)
    - Tumor maker (AFP, PIVKA-II)
- Assessment of AE/SAE
- Confirmation of concomitant therapy

Day 36（Cycle2 Day8）

- Complete physical exam
- ECOG Performance Status
- Vitals signs and weight
- Assessment of Child-Pugh score
- Clinical laboratory tests for:
    - Clinical chemistry
    - Hematology
    - TSH、fT3、fT4
    - Coagulation（PT、APTT、PT-INR）

- Creatinine Clearence

- Assessment of AE/SAE
- Confirmation of concomitant therapy

Day 43（Cycle 2 Day14）

- Complete physical exam
- ECOG Performance Status
- Vitals signs and weight
- Assessment of Child-Pugh score
- Clinical laboratory tests for:
  - Clinical chemistry
  - Hematology
  - TSH、fT3、fT4
  - Coagulation（PT、APTT、PT-INR）
  - Creatinine Clearence
- Assessment of AE/SAE
- Confirmation of concomitant therapy

At discharge during the DLT evaluation period

- Complete physical exam
- ECOG Performance Status
- Vitals signs and weight
- Assessment of Child-Pugh score
- Clinical laboratory tests for:
  - Clinical chemistry
  - Hematology
  - TSH、fT3、fT4
  - Coagulation（PT、APTT、PT-INR）
  - Creatinine Clearence
- Assessment of AE/SAE
- Confirmation of concomitant therapy

7.2.3. Durvalumab q4W dosing period

At Each cycle Day1

- Durvalumab administration (cohort A and B)
- Complete physical exam
- ECOG Performance Status
- Vitals signs and weight
- 12-lead ECG (if necessary)
- Assessment of Child-Pugh score
- Clinical laboratory tests for:
  - Clinical chemistry
  - Hematology
  - TSH、fT3、fT4
  - Coagulation（PT、APTT、PT-INR）
  - Creatinine Clearence
  - Pregnancy test (women of childbearing potential only)

- Tumor maker (AFP, PIVKA-II)
- Assessment of AE/SAE
- Confirmation of concomitant therapy

7.2.4. At the time of discontinuation of investigational drug administration

- Complete physical exam
- ECOG Performance Status
- Vitals signs and weight
- 12-lead ECG (if necessary)
- Assessment of Child-Pugh score
- Clinical laboratory tests for:
  - Clinical chemistry
  - Hematology
  - TSH、fT3、fT4
  - Coagulation（PT、APTT、PT-INR）
  - Creatinine Clearence
  - Tumor maker (AFP, PIVKA-II)
- Assessment of AE/SAE
- Confirmation of concomitant therapy

7.2.5. Follow up period

- Complete physical exam
- ECOG Performance Status
- Vitals signs and weight
- 12-lead ECG (if necessary)
- Assessment of Child-Pugh score
- Clinical laboratory tests for:
  - Clinical chemistry
  - Hematology
  - TSH、fT3、fT4
  - Coagulation（PT、APTT、PT-INR）
  - Creatinine Clearence
  - Urinalysis
  - Tumor maker (AFP, PIVKA-II)
- confirmation of survival
- Assessment of AE/SAE
- Confirmation of concomitant therapy

7.3. Biological sampling procedures

7.3.1. Guideline for blood sampling volume

The total volume of blood to be drawn from each subject in this study is as follows

Amount of blood to be collected from each subject

Assesment		Sample volume (mL) / visit
**Safety**	**Clinical Chemistry Tests**	10

Amount of blood to be collected from each subject

**Assesement**	**Sample volume (mL) / visit**
**Hematology Tests**	10
**Total**	20

7.3.2. Blood samples for archiving

When consent is obtained from the subject for storage of blood specimens, residual blood specimens designated for biochemistry and blood tests will be stored at the Department of Gastroenterology, Chiba University Hospital. Blood specimens will be handled in such a way as to prevent leakage, confusion, theft, or loss of personal information by anonymization with an identification code. Blood specimens will be stored for a period not exceeding 20 years after the completion of the clinical trial, after which all specimens will be properly disposed of. If a subject withdraws consent for specimen storage, the specimens will be destroyed and this will be documented. In addition, if specimens are to be used in future research, a new research protocol must document the use of this specimen and be submitted to the IRB for approval.

7.3.3. Hepatic tumor biopsy sample

In this study, a percutaneous liver biopsy/liver tumor biopsy will be performed before the first dose and between 43 and 56 days after the first dose, if the subject is deemed safe for such a biopsy/liver tumor biopsy by the investigator or sub-investigator and if the subject consents. The tissue samples (tumor and non-tumor) obtained will be used for exploratory studies (refer to 9.3.). At that time, the tissue samples will be handled to prevent disclosure, mix-up, theft, or loss of personal information by anonymization with an identification code. The investigator or sub-investigator may discontinue the percutaneous liver biopsy or liver tumor biopsy for the safety of the subject. Failure to perform a percutaneous liver biopsy/liver tumor biopsy at the discretion of the investigator or sub-investigator, or failure to perform a percutaneous liver biopsy/liver tumor biopsy without the consent of the subject, will not preclude enrollment or dosing in this study. Tissue specimens will be retained for a maximum of 20 years after completion of the study, after which all specimens will be properly disposed of. If a subject withdraws consent for specimen storage, the specimen will be discarded and this will be documented. In addition, if specimens are to be used in future research, the use of that specimen must be documented in a new research protocol and submitted to the IRB for approval.

7.4. Asesement of efficiency

(Tumor evaluation by mRECIST in addition to RECIST ver. 1.1.)
The following are guidelines for confirming image evaluations to assess efficacy.

- Imaging evaluations are performed for subject management and treatment decisions.
- Image evaluation is performed using RECIST ver. 1.1.
- In the absence of clinically evident evidence of disease progression, the patient should be re-evaluated after disease progression (PD) is determined by RECSIST ver. 1.1 in order to distinguish between immune checkpoint inhibitor-induced pseudo progression and true disease progression (this is to reduce the risk of study termination due to incorrect evaluation by the investigator/participating physician). (This is to reduce the risk of termination of the study due to incorrect assessment by the investigator/associated investigator.)

The definition of objective disease progression (definite PD) is as follows

1) The presence of clinically evident evidence of disease progression and disease progression (PD) according to RECIST ver. 1.1 is confirmed as objective disease progression.
2) In the absence of clinically evident findings of disease progression (PD) by RECIST ver. 1.1,

a second imaging study to evaluate PD according to the specific criteria below should be performed after disease progression (PD). The partially modified RECIST ver. 1.1 used for objective confirmation of progression is used only to confirm objective disease progression (confirmed PD). Imaging evaluations to determine objective disease progression should be performed within 4 weeks of the first imaging evaluation that determined PD using RECIST ver. 1.1.

The following is RECIST ver. 1.1 modified to establish objective disease progression.

- On two consecutive image evaluations, the sum of the diameters of the target lesions (TL) increases by more than 20% compared to the sum of the smallest diameters and the sum of the diameters increases by more than 5 mm compared to the sum of the smallest diameters.
- Non-target lesions (NTL) and/or pre-existing new lesions showed significant progression (worsening) at the time of the confirmatory examination compared to the most recent imaging evaluation (note: new lesions at the time of imaging evaluation that are determined for the first time to be PD by RECIST ver. 1.1 are evaluated as NTL at the second imaging evaluation).
- The appearance of an obvious new lesion that was not present at the first imaging evaluation that determined PD according to RECIST ver. 1.1 but was present at the second imaging evaluation.

Two consecutive assessments meeting the definition of PD (first PD by RECIST ver. 1.1 and a second PD using the progression confirmation criteria (above)) are required to establish objective disease progression (determination of definite PD). If PD by RECIST ver. 1.1 does not confirm objective disease progression, evaluation will continue until the next PD by RECIST ver. 1.1. In the absence of significant clinical progression, treatment with investigational agents may continue between the first assessment of progression and imaging studies to confirm progression. If PD is confirmed on confirmatory imaging, the date of progression will be the date PD was confirmed on the previous visit. If objective disease progression is not confirmed, the subject will continue to receive study drug and on-therapy evaluation until the next PD, if there is no clinically significant worsening, at which time another confirmatory scan will be required, even if objective disease progression is confirmed. If the initial PD is not immediately confirmed at the next scan, the investigator should not change the PD assessment from the initial scan.

If subjects discontinue treatment (and/or receive subsequent anticancer therapy) prior to radiographic progression, subjects should be followed until objective disease progression is confirmed. Once progression is confirmed, subjects should continue to be followed for survival every 2 months (8 weeks) according to the evaluation follow-up schedule.

7.5. Assessment of safety

7.5.1. Clinical laboratory tests

Blood and urine samples for determination of clinical chemistry, hematology, and urinalysis will be taken at the times indicated in the assessment schedules and as clinically indicated（refer to 7.1.）

Clinical laboratory safety testing, including serum pregnancy testing, is performed in a licensed clinical laboratory according to local standard procedures. Specimen tubes and specimen sizes may vary depending on the laboratory method used and routine practices at the site. Pregnancy testing may be performed at the site using an approved test (urine or serum pregnancy test). Abnormal clinically significant laboratory results should be repeated as soon as possible (preferably within 24 to 48 hours).

Additional safety samples may be collected if clinically indicated at the discretion of the Investigator. The date, time of collection, and results (values, units, and reference ranges) will be recorded on the appropriate eCRF.

The laboratory variables to be measured are presented in Table 4 (Hematology/ Coagulation), Table 5 (Clinical chemistry), and Table 6 (urinalysis).

Other safety tests to be performed at screening include assessment for HBs antigen, HBs antibody, HBc antibody, and HCV antibody, and HIV antibodies.

The following laboratory variables will be measured:

Table4. Hematology/Coagulation Laboratory Tests

Basophils	Monocytes
Eosinophils	Neutrophils
Hematocrit	Platelet count
Hemoglobin	Red blood cell count
Lymphocytes	Total white cell count [a]
PT-INR	APTT

Table5. Clinical Chemistry (Serum or Plasma) Laboratory Tests

Albumin	Lactate dehydrogenase
Alkaline phosphatase	Lipase
Alanine aminotransferase	Magnesium
Amylase	Potassium
Aspartate aminotransferase	Sodium
Calcium	Total bilirubin[a]
Chloride	Total protein
Creatinine	Urea or blood urea nitrogen, depending on local practice
Gamma glutamyltransferase[b]	Uric acid
Glucose	

a. Tests for ALT, AST, alkaline phosphatase, and total bilirubin must be conducted and assessed concurrently. If total bilirubin is ≥2 × upper limit of normal (and no evidence of Gilbert's syndrome) then fractionate into direct and indirect bilirubin.

b. It is preferable that both amylase and lipase parameters are assessed. For sites where only 1 of these parameters is routinely measured then either lipase or amylase is acceptable.

c. Bicarbonate (where available), chloride, creatinine clearance, gamma glutamyltransferase, and magnesium testing are to be performed at baseline, on Day 1 (unless all screening laboratory clinical chemistry assessments are performed within 3 days prior to Day 1), and if clinically indicated.

d. Creatinine Clearance will be calculated by data management using Cockcroft-Gault (using actual body weight).

e. If TSH is measured within 14 days prior to Day 1 (first infusion day), it does not need to be repeated at day Free T3 or free T4 will only be measured if TSH is abnormal or if there is a clinical suspicion of an AE related to the endocrine system

Table 1. Urinalysis Tests[a]

Bilirubin	pH
Blood	Protein
Glucose	Specific gravity
Ketones	Colour and appearance

a. Microscopy should be used as appropriate to investigate white blood cells and use the high-power field for red blood cells

If a patient shows an AST or ALT ≥3xULN together with total bilirubin ≥2xULN, refer to 8.18.3 for further instructions on cases of increases in liver biochemistry and evaluation of Hy's Law. These cases should be reported as SAEs if, after evaluation, they meet the criteria for a Hy's law case or if any of the individual liver test parameters fulfill any of the SAE criteria.

All patients should have further chemistry profiles performed at 30 days (±3 days), 2 months (±1 week) and 3 months (±1 week) after permanent discontinuation of IP

Any clinically significant abnormal laboratory values should be repeated as clinically indicated and recorded on the eCRF. Situations in which laboratory safety results should be reported as AEs are described in Section8.1.

All patients with Grade 3 or 4 laboratory values at the time of completion or discontinuation from IP must have further tests performed until the laboratory values have returned to Grade 1 or 2, unless these values are likely to improve because of the underlying disease.

7.5.2. Physical examinations

Physical examinations will be performed according to the assessment schedules. Full physical examinations will include assessments of the head, eyes, ears, nose, and throat and the respiratory, cardiovascular, GI, urogenital, musculoskeletal, neurological, dermatological, hematologic/lymphatic, and endocrine systems. Height will be measured at screening only. Targeted physical examinations are to be utilized by the Investigator on the basis of clinical observations and symptomatology. Situations in which physical examination results should be reported as AEs are described in Section 8.1.

7.5.3. Electrocardiogram (ECG)

Resting 12-lead ECGs will be recorded at screening and as clinically indicated throughout the study. ECGs should be obtained after the patient has been in a supine position for 5 minutes and recorded while the patient remains in that position.

At a screening period, in case of clinically significant ECG abnormalities, including a QTcF value >470 ms, 2 additional 12-lead ECGs should be obtained over a brief period (e.g., 30 minutes) to confirm the finding.

Situations in which ECG results should be reported as AEs are described in Section 8.1.

7.5.4. Vital signs

Vital signs (blood pressure [BP], pulse, temperature, and respiration rate) will be evaluated according to the schedule of this clinical trial. Body weight is also recorded at each visit along with vital signs.

First infusion

On the first infusion day, patients will be monitored, and vital signs collected/recorded in eCRF prior to, during and after infusion of IP as presented in the bulleted list below.

BP and pulse will be collected from a subject before, during, and after each infusion at the following times (based on a 60-minute infusion):

- Prior to the beginning of the infusion (measured once from approximately 30 minutes before up to 0 minutes [i.e., the beginning of the infusion])
- Approximately 30 minutes during the infusion (**halfway** through infusion)
- At the end of the infusion ±5 minutes

If the infusion takes longer than 60 minutes, then BP and pulse measurements should follow the principles as described above or be taken more frequently if clinically indicated. A 1-hour observation period is recommended after the first infusion of durvalumab.

Subsequent infusions

BP, pulse and other vital signs should be measured, collected/recorded in eCRF prior to the start of the infusion. Patients should be carefully monitored and BP and other vital signs should be measured during and post infusion as per institution standard and as clinically indicated.

7.5.5. ECOG performance status

ECOG performance status will be assessed at the times specified in the assessment schedules based on the following:

0. Fully active; able to carry out all usual activities without restrictions
1. Restricted in strenuous activity, but ambulatory and able to carry out light work or work of a sedentary nature (e.g., light housework or office work)
2. Ambulatory and capable of self-care, but unable to carry out any work activities; up and about more than 50% of waking hours.
3. Capable of only limited self-care; confined to bed or chair more than 50% of waking hours
4. Completely disabled; unable to carry out any self-care and totally confined to bed or chair
5. Dead

Any significant change from baseline or screening must be reported as an AE.

7.5.6. Other safety assesements

If new pulmonary symptoms (e.g., dyspnea) or radiological abnormalities suggestive of pneumonia/interstitial lung damage (ILD) are observed, toxicity management as detailed in the Toxicity Management Guidelines (see Appendix of Protocol 2) will be applied. Complete diagnostic results (including high-resolution computed tomography (HRCT), blood and sputum cultures, hematological parameters, etc.) should be recorded in the source documents. An accurate diagnosis including consultation with a specialist is strongly recommended to rule out alternative causes such as lymphangitic carcinomatosis, infection, allergy, cardiogenic edema, or pulmonary hemorrhage. In the presence of a confirmatory HRCT scan that excludes other causes of respiratory symptoms, the diagnosis of interstitial lung disease (ILD) should be considered and toxicity management guidelines followed. The investigator is responsible for ensuring that all staff involved in the study are familiar with the contents of this section.

8. HANDLING OF ADVERSE EVENT

8.1. Definition

8.1.1. Adverse event

Adverse events are all unwanted or unintended signs (including abnormal changes in laboratory values), symptoms, or illnesses that occur after the first administration of an

investigational drug, regardless of causal relationship to the study treatment.

8.1.2. Severe adverse event

A serious adverse event is defined as any of the following

（1） results in death
（2） is life-threaening
（3） requires inpatient hospitalisation or prolongation of existing hospitalisation
（4） results in persistent or significant disability/incapacity
（5） is a congenital anomaly/birth defect
（6） Other serious cases according to the above

The term “life-threatening” for the purposes of this definition is defined as an event that the investigator/participating physician determines poses an imminent risk of death to the subject as a result of its manifestation. It does not mean hypothetically that death might have resulted had the manifestation of the event been more severe.

“Hospitalization” in (3) below is not considered a serious adverse event if any of the following apply: (However, a new occurrence during the hospitalization is treated as an adverse event. (However, any new occurrence during that hospitalization will be treated as an adverse event.)

- Hospitalization as defined in this study protocol
- Hospitalization or prolongation of hospital stay that was planned prior to the start of the clinical trial.
- Hospitalization or prolonged hospitalization for social reasons (reasons of convenience or other non-medical necessity)
- Hospitalization or extended hospital stay for examination, education
- Hospitalization for follow-up or prolongation of hospitalization for those who are cured or have a mild illness.
- Hospitalization or prolonged hospitalization for new treatment of the underlying disease after completion of the investigational drug

(6) Other serious, in accordance with the above, are “significant medical events” that may not be immediately life-threatening or result in death or hospitalization, but which may endanger the subject or require treatment or therapy to avoid the consequences as listed in these definitions.

Adverse events (AEs) for malignancy reported during a clinical trial will generally be evaluated as serious AEs. If no other severity criteria are met, the AE will be judged to be a “Significant Medical Event” as described above. However, in certain circumstances, medical judgment based on individual events should be applied to clarify that malignancy events should be evaluated and reported as non-serious AEs. For example, if medical history includes malignancy and the malignancy progresses during the clinical trial, but the progression does not change the treatment or prognosis of the malignancy, the malignancy progression should be reported as an AE but may not meet the attributes to be evaluated as serious.

The causal relationship of the SAE (relationship to all investigational treatments/procedures) should be evaluated by the investigator and reported to AstraZeneca.

8.1.3. Adverse Events of Special Interest (AESI)

AESIs of durvalumab and tremelimumab for heavy particle irradiation include events due to potential inflammatory or immune-mediated mechanisms, which require more frequent monitoring and treatment with steroids, immunosuppressive agents, and/or hormone replacement

therapy.Careful monitoring of these AESIs will be implemented in clinical trials of durvalumab monotherapy and durvalumab plus tremelimumab combination therapy. Immune-mediated adverse events (imAEs) are AESIs, defined as events that occur with the administration of (exposure to) an investigation agent, are consistent with an immune-mediated mechanism of action, and have no apparent other cause. imAEs should be diagnosed using serologic, immunologic, and histologic (biopsy) data, as appropriate, for support. The diagnosis of imAE should be supported by serologic, immunologic, and histologic (biopsy) data as appropriate, and efforts should be made to rule out tumor, infection, metabolism, toxins, and other causes of imAE.

If there is any doubt as to whether an adverse event is an imAE, the investigator should immediately contact the sponsor's medical experts. AESIs for durvalumab and/or tremelimumab and heavy particle irradiation include the following.

- Dysentery/colitis, intestinal perforation
- Pneumonitis/ILD
- Hepatitis/transaminases increase
- Endocrine disorders (i.e., hypophysitis, hypopituitarism, adrenal insufficiency, hyperthyroidism, hypothyroidism and type I diabetes mellitus)
- Rash/dermatitis
- Nephritis/increased blood creatinine
- Pancreatitis/increased serum lipase and amylase
- myocarditis
- pericarditis
- Myositis/polymyositis
- Neuropathy/neuromuscular toxicity (Guillain-Barré syndrome, myasthenia gravis, etc.)
- Other inflammatory reactions of rare / infrequent immune-mediated (but not limited to pericarditis, sarcoidosis, uveitis, and ocular, skin, blood system and rheumatology-related events)

In addition, reactions associated with infusion and hypersensitivity/anaphylactic reactions due to various pharmacologic causes are also considered AESIs.

Details of these risks and presenting symptoms are described in the most recent versions of the durvalumab and tremelimumab investigational new drug summaries. Specific guidelines for the evaluation and treatment of these AESIs are provided in the Dose Modification and Toxicity Management Guidelines. These guidelines were developed by the investigational drug provider to assist investigators and others in making clinical decisions when treating this type of toxicity. This guideline applies to adverse events that the reporting investigator determines are causally related to the investigational drug/regimen.

If new or worsening pulmonary symptoms (e.g., dyspnea) or radiological abnormalities suggestive of pneumonia/interstitial lung disease are observed, toxicity management as detailed in the “Toxicity Management Guidelines” (see Appendix F) will be applied. Results of complete diagnostic tests (including high-resolution computed tomography (HRCT), blood and sputum cultures, hematological parameters, etc.) should be recorded in the source documents.

An accurate diagnosis, including consultation with a specialist, is strongly recommended to rule out alternative causes such as lymphangitic carcinomatosis, infection, allergy, cardiogenic edema, or pulmonary hemorrhage. On confirmatory HRCT scans where other causes of respiratory symptoms have been ruled out, the diagnosis of interstitial lung disease (ILD) should be considered and toxicity management guidelines followed.

8.1.4. Confirmation of interstitial lung disease (ILD)

To ensure thorough investigation and diagnosis of possible cases of pneumonia, the following evaluations, and additional evaluations as needed, will be performed. Collect the results of the evaluation.

- physical examination
- Evaluate signs and symptoms (cough, shortness of breath, fever, etc.), including auscultation of the lung field.
- Peripheral oxygen saturation (SpO2)

Other

If pneumonia (ILD) is suspected during clinical trial treatment, the following markers should be measured.

if possible

(i) ILD markers (KL-6, SP-D) and β-D-glucan

(ii) Tumor markers. Specific tumor markers associated with disease progression.

Other biochemistry: CRP, LDH

8.2. Assessment of severity

AEs and SAEs; severity will be determined in accordance with CTCAE ver. 5.0 The severity of all other events not listed in the CTCAE will be determined by the severity category from Grade 1 to 5, as determined by the investigator based on medical judgment, will be as follows.

- Grade 1 (mild)
  Events that are usually transient and require only minimal Treatment or therapeutic intervention. The event generally does not interfere with normal activities of daily living.

- Grade 2(Moderate)
  An event that is usually alleviated by additional specific therapeutic intervention. The event interferes with normal daily activities and causes discomfort, but does not pose a risk of serious or permanent harm to the subject.

- Grade 3(Severe)
  An event requiring intensive therapeutic intervention. An event that interferes with normal daily activities or significantly affects the subject's clinical condition.

- Grade 4(life-threatening）
  Events and/or immediate sequelae related to the following Imminent risk of death or a physical or mental impairment that affects or limits the ability to perform activities of daily living (eating, walking, toileting, etc.).

- Grade 5(deadly)
  Death as a result of an event. it is important to distinguish between serious criteria and severity of AEs.

It is important to distinguish between severity and severity of an AE. Severity is a measure of intensity, and severity is defined by the criteria in section 10.3.1 A Grade 3 AE need not necessarily be considered an SAE. For example, a Grade 3 headache lasting several hours would not meet the regulatory definition of an SAE and would be considered a non-serious event, whereas a Grade 2 attack leading to hospitalization would be considered an SAE.

8.3. Record of adverse events and serious adverse events

AEs and SAEs will be collected from the time of the first dose of study drug until the follow-up date 28 days after the discontinuation of study drug. However, AE and SAE outcomes will be collected until the end of the safety information collection period (90 days after the discontinuation of study drug) or until the start of alternative anticancer therapy. Of events occurring after the 28-day follow-up period after the discontinuation of the investigational drug,

if the event is considered to be due to delayed toxicity to the investigational drug, it will be collected as an AE or SAE until the end of the safety information collection period or until the start of alternative anticancer therapy. All AEs and SAEs will be actively followed up for each subject for the duration of the trial as long as the event is ongoing. Every effort will be made to resolve all events, even if the event continues after the subject discontinues the investigational drug or after the study is terminated.

AEs unresolved at the subject's last clinical trial visit will be followed up by the investigator as long as medically necessary but will not be further documented in the eCRF. AstraZeneca reserves the right to request additional information from subjects with ongoing AEs/SAEs at the end of the trial if deemed necessary.

For each AE, the following information should be collected

- Adverse event name
- Date of adverse event and date of disappearance
- Maximum CTCAE Grade
- Severity
- Assessment of causal relationship with study treatment
- Treatments related to investigational drugs
- Treatment of adverse events: Administration of AE medication
- outcome

In addition to the above, the following are also confirmed for SAE.

- Date of serious adverse event
- Date when the investigator becomes aware of the onset of the serious adverse event
- Definitions applicable to the determination of serious adverse events
- Date of admission
- Date of discharge
- Presumed reason for death
- Date of death
- Autopsy
- Evaluation of causal relationship with clinical trial procedures
- Evaluation of causal relationship with other drugs
- Details of serious adverse events

The grading scale described in CTCAE ver. 5.0 is used for all events that have been assigned to a CTCAE Grade; for events that have not been assigned a CTCAE Grade, the CTCAE criteria for converting mild, moderate, and severe events to a CTCAE Grade A copy of CTCAE ver. 5.0 can be downloaded from the Cancer Treatment Evaluation Program website at http://www.jcog.jp/doctor/tool/ctcaev5.html.

8.4. Duration of recording and follow-up of adverse events and serious adverse events

If the subject discontinues treatment for reasons other than objective confirmation of disease progression and therefore continues tumor evaluation, the drug- or treatment-related SAEs will be tracked until the subject confirms PD and no further tumor evaluation is performed.

The investigator is responsible for tracking all SAEs until the subject returns to baseline status or until the condition stabilizes with the expectation that the chronic condition will be maintained, even if it continues beyond study participation, until all SAEs are resolved.

8.5. Causal relationship with investigational therapy

The following examples will be used to determine the causal relationship between the clinical trial treatment and the patient.

Causative: if a reasonable possibility can be explained that the investigational treatment caused the adverse event in question (examples are given below).

1) If the event is time-related to the onset of the event, and the event attenuates with the passage of time after the study treatment but recurs or worsens with subsequent re-administration of the study treatment.
2) The presence of confounding risk factors is negative, such as the subject's general condition, complications, concomitant medications, or concomitant therapies.

No causal relationship: other than above

8.6. Outcome definition

Outcomes after adverse events are determined from the following

1) Recovery: when the patient recovers to the state before the adverse event occurred.
2) Recovered but with sequelae: When the adverse event has recovered but the effects of the adverse event remain as sequelae
3) Death: when the adverse event that occurred was the direct cause of death
4) Lightening of symptoms: adverse events continue, but symptoms are improving
5) Unrecovered: adverse events continue (symptoms are not improving)
6) Unknown: When the subject is no longer traceable

8.7. Treatment of investigational drug in the event of an adverse event

1) No change: When an adverse event occurs but there is no change in the conditions under which the investigational drug is administered.
2) Discontinuation: Discontinuation of study drug administration due to the occurrence of an adverse event
3) Withdrawal: Temporary suspension of study drug administration due to the occurrence of an adverse event
4) Not applicable: Adverse events occur before the start of study drug administration or after the end of the study period

8.8. Treatment of heavy particle therapy equipment in the event of an adverse event

1) Suspension: Temporary suspension of heavy-ion radiation therapy due to the occurrence of adverse events
2) Discontinuation: Discontinuation of heavy-ion radiation therapy due to the occurrence of an adverse event
3) Not applicable: Adverse events occur before or after the start or completion of carbon ion radio therapy

8.9. Relationship to Protocol Procedures

The investigator must also provide an assessment of the relationship between SAEs and protocol procedures on the SAE Report Form. This includes both non-therapeutic emergencies (SAEs that occur prior to the administration of the investigational drug) and therapeutic emergency SAEs. Protocol-related SAEs may occur because of a required procedure or intervention (e.g., blood draw) during the clinical trial. The investigator should use the following guidelines to assess the relationship between SAEs and protocols

- Protocol-related: The event occurred because of a procedure or intervention described in the protocol for which no alternative etiology exists in the subject's medical record.
- Not protocol-related: The event is related to an etiology other than the procedure or intervention

described in the protocol. The alternative etiology must be documented in the study subject's medical record.

In the case of AEs, AEs on treatment (or AEs appearing on treatment) are defined as AEs that began after or before administration and worsened after exposure to treatment between the start dose date and 90 days after discontinuation of study treatment.

8.10. Adverse events based on signs and symptoms

All AEs reported spontaneously by the subject or in response to questions from the investigator, subinvestigator, or collaborator (example of a visit: "Have you had any health problems since your last visit / have you been asked any questions since your last visit?") ")

When collecting AEs, recording the diagnosis is preferred over recording a list of signs and symptoms, if possible. However, if the diagnosis is known and there are other signs and symptoms that are not generally part of the diagnosis, record the diagnosis and each sign or symptom separately.

8.11. Adverse events based on tests and examinations

Protocol-mandated laboratory values and vital signs measurements will be summarized in the CSR. Therefore, protocol-mandated worsening of laboratory values and vital signs relative to baseline should only be reported as an AE if it meets one of the SAE criteria or is a reason for discontinuation of treatment with the investigational drug.

If the worsening of laboratory values or vital signs is associated with clinical signs or symptoms, the signs or symptoms should be reported as an AE and the associated laboratory results or vital signs should be reported as additional information, as appropriate. Whenever possible, the reporter uses clinical terms rather than laboratory terms (e.g., "anemia" rather than "low hemoglobin level"). In the absence of clinical signs or symptoms, the worsening of such laboratory values should be reported as an AE.

Laboratory deterioration attributable to apparent disease progression shall not be considered an adverse event or serious adverse event.

Report as an adverse event any new or worsening clinically significant abnormal findings at the time of presentation compared to the baseline evaluation.

8.12. Hy's Law

Biochemical elevations suggestive of abnormal liver function may require further evaluation, and the occurrence of AST or ALT ≥ 3 x ULN and total bilirubin ≥ 2 x ULN may require reporting as an SAE. For cases of elevated liver biochemistry and Hy's Law evaluation, see Ref.

8.13. Disease progression

Disease progression is considered a worsening of the subject's condition due to the disease for which the investigational drug is being studied and is an increase in the severity of the disease under study and/or an increase in the symptoms of the disease. The development of new metastases or progression of existing metastases to the primary cancer being studied is considered disease progression and is not considered an AE. Events that are clearly attributable to disease progression should not be reported as AEs during a clinical trial.

8.14. New cancer

The development of new cancer shall be considered a serious adverse event. New cancers are those that occur after a patient is enrolled in the study, rather than those that were the primary reason for enrollment in the study. New metastatic lesions are considered progression of the cancer under study and are not reported as a second cancer.

8.15. Deaths

All deaths occurring during the investigational treatment period or within the protocol-defined follow-up period after the discontinuation of the investigational drug shall be reported as follows.

- Events that are clearly attributable to disease progression will not be treated as investigational AEs, but if the outcome of the event is found to be severe, all SAEs, not limited to death, will be reported, as described in Section 10.14.
- The investigator shall immediately report to the site director and the investigational drug provider, regardless of the causal relationship between the SAE and the investigational drug/IR, regardless of whether the death is due to progression of the disease under study. In addition, a note will be made in the eCRF.
- The causes are identified and described in the eCRF.
- Deaths of unknown cause should always be reported as SAEs.
- If an autopsy is performed, the autopsy results should be reported to AstraZeneca.

Deaths that occur after the discontinuation of the investigational drug and after the protocol-defined safety information collection period should also be noted in the eCRF as required. If the death is attributable to an event that occurred after the safety information collection period and the event is considered to be due to delayed toxicity to the investigational drug, it must also be reported as an SAE.

AstraZeneca retains the right to request additional information for subjects with ongoing AE(s)/SAE(s) at the end of the trial if deemed necessary.

8.16. Reportable Adverse Events

The Investigator or Co-Investigator will ensure that all adverse events that occurred from the time consent is obtained until 28 days after the end of study drug administration are consistently described in the case report form and that adverse events disappear or that the study period is observed until 4 weeks after the end of the study period (after discontinuation). Adverse events that are judged to have a causal relationship with the study drug will continue to be observed as much as possible until the end of the study period. However, this does not apply if the investigator determines that the safety of the subject is sufficiently ensured and further follow-up is not necessary.

8.17. Reporting of serious adverse events

All serious adverse events during the clinical trial (regardless of whether there is a causal relationship with the investigational drug or not) and serious adverse events suspected to be related to the investigational drug after the completion (discontinuation) of the clinical trial shall be reported according to the following procedures. The details of the reporting procedures are separately stipulated in the “Standard Operating Procedures for Handling Safety Information". An outline of the reporting procedure is provided below.

1） Reports from the investigator to the head of the site and to investigators at other sites

If any serious safety information is recognized during the clinical trial period, the investigator shall immediately report the details to the head of his/her institution, the investigational drug supplier, and the investigators at other sites using the “Report on Serious Adverse Events and Malfunctions” (Uniform Form 12, or the same detailed description form, if necessary). In reporting, the investigator shall specify whether or not the serious safety information is an event that cannot be predicted from the investigator’s brochures, etc.

2） Discussion between the investigator and the Clinical Trial Coordinating Committee

The investigator shall consult with the Trial Coordinating Committee and report his/her opinion as the investigator (including the necessity of reporting to the Minister of Health, Labour and Welfare) to the Trial Coordinating Committee. The investigator shall report his/her opinion (including the necessity of reporting to the Minister of Health, Labour and Welfare) to the Trial

Coordinating Committee. If the Independent Data Monitoring Committee is consulted regarding the investigator's judgment, the opinion of the Independent Data Monitoring Committee shall be followed.

3） Report to the Minister of Health, Labour and Welfare and the heads of other medical institutions

1 Report to the Minister of Health, Labor and Welfare

If the Clinical Trial Coordinating Committee determines that the case is subject to reporting as stipulated in Article 273 of the Enforcement Regulations of the Act on Quality, Efficacy and Safety Assurance of Pharmaceuticals, Medical Devices and Other Products (Pharmaceuticals and Medical Devices Law), it shall report to the Minister of Health, Labor and Welfare (the Pharmaceuticals and Medical Devices Agency, an independent administrative agency (PMDA)). In addition, when the Independent Data Monitoring Committee is consulted, it shall be notified of the contents of the report made to this authority.

2 Reporting to the heads of other implementing medical institutions

When a report is made to the Minister of Health, Labour and Welfare, the investigator of the other site shall report the contents of the “Failure/Infectious Case Report” obtained from the Trial Coordinating Committee to the head of his/her site as soon as possible.

4） Actions to be taken when additional information is obtained

When additional information regarding the adverse event is obtained, the investigator of the site where the adverse event occurred shall make an additional report to the head of the site as soon as possible, as well as to the Clinical Trial Coordinating Committee and the investigational drug supplier. The handling of such additional information shall be in accordance with the procedures described in 1) to 3) above, and reports shall be made to the NIH as necessary.

5） Annual report to the Minister of Health, Labor and Welfare

If the investigational drug has not been approved in Japan, or if the sponsor is not conducting a clinical trial, the person conducting the clinical trial shall submit an annual report to PMDA in accordance with Article 273 of the Ordinance for Enforcement of the Pharmaceutical Affairs Law.

The investigator will report all SAEs to AstraZeneca. The investigator will send a copy of the SAE report submitted to PMDA to the investigational drug provider in accordance with a separate Standard Operating Procedure (SOP). If any changes are made to the follow-up report, the following information will also be promptly sent to the investigational drug provider.

8.17.1. Response to subjects

When a serious adverse event is observed, the investigator or subinvestigator should immediately take appropriate measures, discontinue administration of the investigational drug to ensure the safety of the subject, and inform the subject if treatment for the adverse event becomes necessary. If an adverse event that was considered to be causally related to the investigational drug at the time of discontinuation has not yet recovered, observation will be continued as much as possible until the adverse event recovers or becomes mild, in principle. However, this does not apply when the investigator determines that the subject's safety has been sufficiently ensured and further follow-up is not necessary.

8.18. OTHER EVENTS REQUIRING REPORTING

8.18.1. Overdose

Use of durvalumab or tremelimumab in excess of the prescribed dose of the drug constitutes an overdose. Currently, there is no established treatment for durvalumab or tremelimumab overdose and no established symptoms of a possible overdose. Overdoses with associated AEs are recorded as an AE diagnosis or symptom in the relevant AE module of the eCRF. Overdoses without symptoms are only recorded and reported to the site investigator.

Overdoses of durvalumab or tremelimumab, with or without associated AEs/SAEs, must be recorded and reported to the site investigator. The investigator must report these to the investigational drug provider as described in the SOP.

8.18.2. Hepatic function abnormality

Abnormal liver function that meets Hy's Law criteria should be reported as an SAE regardless of the presence or absence of clinical symptoms. Hy's Law criteria are met if AST or ALT is at least 3 times the upper limit of normal (ULN) or TBL is at least 2 times the upper limit of normal at any time after initiation of treatment with the investigational drug, regardless of elevated ALP.

If it cannot be determined that there is no causal relationship to the investigational drug (e.g., due to cholelithiasis or bile duct obstruction), the investigator should report these events to the study provider in accordance with SOPs.

If the cause of the hepatic dysfunction is established and there is no causal relationship to the investigational drug, the investigator will decide whether to continue the subject's treatment based on the investigator's clinical judgment.

- If the cause of the liver function abnormality is not established, the subject's administration should be discontinued immediately. Follow-up investigations and inquiries will be initiated by the investigational site without delay.

The investigator will follow up on each reported event of liver function abnormality.

8.18.3. Pregnancy

All pregnancies and pregnancy outcomes should be reported to AstraZeneca and the site investigator, except in the following cases

- Pregnancy discovered before the subject received the investigational drug.
- Pregnancy of the male subject's female partner. (If the male subject is not restricted from having children.)

8.18.4. Exposure to pregnant woman

If a subject becomes pregnant during a clinical trial, the investigational drug should be discontinued immediately. Pregnancy itself shall not be considered an AE unless there is a suspicion that the investigational drug interfered with the effectiveness of the contraceptive. Birth defects or birth defects and spontaneous abortions should be reported and treated as SAEs. Uncomplicated elective abortions shall not be treated as AEs. The outcome of all pregnancies (spontaneous abortion, elective abortion, ectopic pregnancy, normal delivery, and congenital anomalies) shall be followed up and documented even if the clinical trial is discontinued. If pregnancy occurs during the clinical trial, the investigator shall notify AstraZeneca in accordance with the SOP.

8.18.5. Exposure to partner

Male subjects should refrain from sexual intercourse or sperm donation with their partners during the study and for 180 days after the last dose of durvalumab plus tremelimumab combination therapy or 90 days after the last dose of durvalumab monotherapy, whichever is longer. Pregnancy in the subject's partner is not considered an AE. However, the outcome of all pregnancies (spontaneous abortion, elective abortion, ectopic pregnancy, normal delivery, or

congenital anomaly) occurring between the date of first dose and 180 days after the last dose of durvalumab + tremelimumab combination therapy or 90 days after the last dose of durvalumab monotherapy, whichever is longer, will be followed up and documented if possible. Follow-up and documentation, if possible, is desirable. Upon receipt of a report of pregnancy, the investigator must obtain the consent of the subject's partner prior to obtaining any information regarding the pregnancy. Therefore, the study team should adopt the generic ICF template according to the procedure and submit it to the Institutional Review Board (IRB) prior to use.

8.19. Medication error

For this study, a medication error is an unintentional error in the course of treatment with an investigational drug that may cause harm to the subject. A medication error is not a lack of efficacy of the investigational drug, but an artificial or process-related failure while the investigational drug is under the control of the site staff or subject. Medication errors include the circumstances under which the error occurred.

- A medication error occurred.
- A medication error occurred, but was identified before the subject received the medication.
- No medication errors occurred, but circumstances were observed that could have resulted in errors.

Examples of events that should be reported in a clinical trial as medication errors

- Confusion of drug names
- Dispensing errors (e.g., some medications were incorrectly dispensed even though they were not actually administered to the subject.
- Drugs that were not administered as directed, such as by incorrect route or site of administration
- Drugs not taken as directed, such as tablets dissolved in water when they should be taken as solid tablets
- Drugs not stored as directed (e.g., in a refrigerator when they should be at room temperature.)
- Subjects who received medication by mistake
  Drugs administered to the wrong subject

Examples of events that need not to be reported as medication errors in clinical research

- Including those that lead to any of the above events or that result in a dose error.
- If the subject fails to take the medication (e.g., forgets to take the medication）
- Overdose Accidents
- Subject did not return unused or empty packaged medications
- Errors associated with background or rescue drugs, even AstraZeneca products, or standard of care drugs in open label studies

Medication errors are not considered AEs, but AEs may occur because of medication errors. If a medication error occurs during a clinical trial, the investigator or other site personnel should contact AstraZeneca within one day.

8.20. Predicted Side Effects

The following is a tabulation of adverse events that occurred in clinical trials of durvalumab (Frequency of occurrence: 5% or more). For other adverse events, please refer to the Investigators brochure.

（1）Information on adverse events in patients treated with only durvalumab (all grades, incidence >5%)

Side effect

Fatigue
anorexia
cough
Nausea
Breathe bitterly
constipation
diarrhea
fever
anaemia
Back pain
vomiting
Itching
lethargy
Joint pain
Hypothyroidism
rash
headache
Peripheral edema
sleeplessness
bellyache
Weight loss
Musculoskeletal pain
dizziness
pneumonia
Urinary tract infections
Muscle pain
Upper respiratory tract infections
AST elevation
Pain in the extremities
Hyponatremia
ALT elevation
Wet cough
Nasopharyngitis

（2）Information on adverse events in patients treated with durvalumab and tremelimumab in combination (all grades, incidence >5%)

Side effect
fatigue
diarrhea
anorexia
nousea
pruritic
constipation
dyspnea
anaemia

fever
vomiting
coughing
backache
rash
lethargy
abdominal pain
peripheral edema
weight loss
arthralgia
hypothyroidism
insomnia
AST elevation
hyponatremia
ALT elevation
headache
Lipase rise elevation
dizziness
amylase elevation
hypokalemia
pneumonia
maculopapular eruption
Urinary tract infections
ALP elevation
dehydration
Hyperthyroidism
hyperglycemia
γ-GTP elevation
Hypoalbuminemia
Musculoskeletal pain
Muscle pain
Dry skin
Wet cough
Xerostomia
musculoskeletal chest pain
upper respiratory tract infection

（3） Refer to Section 2.3.2.3 for more information on the risks of Carbon ion radio therapy.

9. ENDPOINT

9.1. Primary endpoint

Percentage of dose-limiting toxicities (DLT) and adverse events/serious adverse events

Rationale for Setting the Primary Endpoint<br><br>To evaluate the tolerability and safety of durvalumab tremelimumab in combination with carbon ion radio therapy in subjects with advanced hepatocellular carcinoma with vascular invasion.

9.2. Secondary endpoint

Overall survival (OS), 6-month survival, objective response rate (ORR) calculated by RECIST ver 1.1 and mRECIST, 6-month progression-free survival (PFS), Time to progression (TTP)

Rationale for Setting Secondary Endpoints<br><br>To evaluate the efficacy of durvalumab tremelimumab in combination with carbon ion radio therapy in subjects with advanced hepatocellular carcinoma with vascular invasion.

9.3. Exploratory endpoints

In addition to consent for this study, consent for "Exploratory Study of Tumor Cells and Tumor Microenvironment in Hepatocellular Carcinoma Using Tumor and Non-tumor Biopsy Specimens, Blood Specimens, and Stool Specimens" and "Exploratory Study of Tumor Cells and Tumor Microenvironment by Secondary Use of Biological Specimens in Hepatobiliary Pancreatic Cancer" and a liver biopsy or liver tumor biopsy specimen must be In patients with sufficient liver biopsy/tumor biopsy samples, biomarkers associated with clinical outcome of combination therapy with durvalumab/tremelimumab and carbon ion radio therapy will be explored using blood and liver biopsy/tumor biopsy samples. The analysis will be performed in the laboratory of the Department of Gastroenterology, Graduate School of Medicine, Chiba University, and may be contracted to an outside vendor or outside research organization.

10. STATISTICAL METHODS AND SAMPLE SIZE DETERMINATION

10.1. Description of Analysis set

10.1.1. DLT analysis set

All subjects enrolled in the study, who have completed at least one investigational drug regimen (IR), and for whom a DLT evaluation has been performed, are considered the DLT analysis set.

10.1.2. Safety analysis set: SAF

All subjects who have had at least one dose of the investigational drug or investigational drug regimen (IR) are considered in the Safety analysis set.

10.1.3. Full Analysis set: FAS

All subjects with eligible disease who are properly enrolled and have received at least one dose of the investigational drug regimen are considered Full Analysis set (FAS).

10.1.4 Efficacy Evaluable set: EES

The efficacy evaluation analysis(EES) population is defined as a subset of the FAS. That is, it consists of all subjects who are eligible for the trial, receive the study drug/IR, and have had at least one post-dose efficacy endpoint assessed. In addition to subjects with at least one baseline and at least one post-dose efficacy evaluation, the population will include cases of early death or early progression before the evaluation.

10.1.5 Per protocol set：PPS

Subjects from the FAS who do not have any of the following serious violations of the study protocol, including the study protocol, such as the method of study or concomitant therapy, will be considered to be in compliance with the study protocol (PPS).

- Violation of inclusive criteria
- Violation of exclusion criteria
- Violation of concomitant use of prohibited drugs
- Violation of concomitant use of prohibited therapies

10.2. Target number of cases and rationale for setting

Target cases: 3 to 15

A modified “3+3 design” will be used in this trial. Cohort A will be used to evaluate DLT for carbon ion radio therapy + durumab, and instead of dose escalation, the group with toremelimumab will be used as Cohort B to evaluate DLT.

10.3. Case Handling

The coordinating investigators and the statistical analyst will discuss and decide how to handle the registered cases. The coordinating investigator and the statistical analyst will also discuss and decide how to handle cases in case new problems arise.

10.4. Data Handling

In principle, the handling of data during data compilation and analysis shall be as follows. In case of doubt, the statistician and the coordinating Investigators shall discuss and decide the handling of the data. Details are described in the statistical analysis plan.

1) Missing values: Supplementation of missing values is not performed.
2) Reference values: Data with unreliable measured values due to hemolysis, etc. are not used in the aggregate analysis.
3) Time lag: Data not conducted during the specified observation period will not be used in the aggregate analysis for that period. For other data requiring consideration, the coordinating investigators will consult with medical experts to determine how to handle the data for analysis.

10.5. Statistical analysis items and analysis plan

All patients will be analyzed after completion of treatment with the investigational drug and after the data have been fixed, using the DLT evaluation population for DLT evaluation, the ESS analysis population for efficacy evaluation, and the safety analysis population for safety evaluation. For the efficacy evaluation using RECIST without survival analysis, FAS and PPS analysis and sensitivity analysis will be performed as necessary.

10.5.1. Analysis of subject background

The distribution of subject background data and summary statistics are calculated for each cohort. For nominal variables, frequencies and proportions of categories are shown. For continuous variables, summary statistics (number of cases, mean, standard deviation, median, range, and interquartile range) are calculated.

10.5.2. Analysis of primary endpoints

10.5.2.1. DLT Evaluation

1) DLT incidence rate

The number of DLT cases and incidence rate will be calculated for each cohort.

10.5.2.2. Safety Evaluation

1) Adverse event rate
2) Serious adverse event rate
3) Adverse events coded by MedDRA
4) List of SOC, PT, severity, relevance, etc.

10.5.3. Analysis of secondary endpoints

10.5.3.1 Efficacy Analysis

Overall survival (OS), 6-month survival rate, objective response rate (ORR), 6-month progression-free survival (PFS), and time to progression (TTP) will be determined as secondary parameters to evaluate efficacy in each cohort. For survival analysis, the Kaplan-Meier method will be used to determine the incidence rate at 6 months and the median and mean survival times using the ESS analysis group.

Overall Response Rate（ORR）

ORR (based on evaluation by investigators using RECIST 1.1) is defined as the percentage of patients with a CR or PR at one or more visits. Patients who had a response after discontinuation of treatment without PD and initiation of post-treatment are not included in the ORR response cases.

Progression-Free Survival（PFS）

PFS (based on evaluation using RECIST 1.1 by investigators and others) is defined as the time from allocation to objective disease progression or death (or cause of death in the absence of disease progression), regardless of whether the patient discontinued treatment or received other anticancer therapy before disease progression. Patients who have not progressed or died at the time of analysis will be censored at the date of the last RECIST 1.1-based evaluation. However, if a patient has progressed or died after two or more consecutive missed visits, the patient will be censored as of the date of the last RECIST 1.1-based evaluation. In the absence of evaluable visit data or baseline data, censor at Day 1 unless the patient has died between baseline and the second visit, in which case treat as an event with the date of death as the date of the event.

The PFS is always calculated based on the date the imaging study/evaluation was performed, not the date of the visit.

It is possible that an evaluation/imaging study based on RECIST 1.1 scheduled for a specific visit may be performed over several different days. In such cases, the following principles apply.

- For evaluation by the investigator, the earliest RECIST 1.1 evaluation/imaging test date when an element indicating progression is identified is the progression date.PFS
- When an evaluation is terminated, it shall be terminated on the last imaging inspection date of the inspection related to the specific overall effectiveness evaluation.

Time to Progression (TTP)

TTP (based on evaluation using RECIST 1.1 by the investigator and others) is defined as the period from the date of random assignment to the date of objective tumor progression. Death is not included in the definition of TTP. Death without progression is not included in the definition of TTP.

Patients who die without progression will be terminated at the time of death.

Image Evaluation

Imaging evaluation For all imaging evaluations, in addition to evaluation by the investigator or subinvestigators, a central judgment will be made by multiple radiologists to be separately determined.

10.5.4. Interim Analysis

No interim analysis will be performed in this clinical trial.

10.6. Final Analysis

After the follow-up period, analysis will be conducted after the data are obtained and the cases are fixed. The person in charge of statistical analysis compiles the "Analysis Report" and submits it to the coordinating investigator and the principal investigator. The coordinating investigator summarizes the contents of the analysis report and prepares a "summary report" summarizing the overall conclusions of the trial, problems, interpretation and discussion of the results, and future policies mainly from a clinical perspective, and obtains approval from the principal investigator.

10.7. Data Monitoring Committee

An Independent Data Monitoring Committee will be established for this clinical trial. The Independent Data Monitoring Committee will be established as an independent body from the investigators and will consist of three or more expert members who are independent of the study. The Independent Data Monitoring Committee will be established for the purpose of ensuring the safety of subjects. It will provide appropriate advice and recommendations in accordance with a separate protocol If the study is terminated before the end of the DLT evaluation period for reasons other than DLT criteria, the investigator will ask the Independent Data Monitoring Committee for its opinion on the addition of a case If a second DLT occurs, the investigator will ask the Independent Data Monitoring Committee to determine whether the study regimen is If a second DLT occurs, the investigator will ask the Independent Data Monitoring Committee for an opinion on whether the study regimen is intolerable. If necessary, the investigator will confirm whether tolerability was not an issue in the other cases.

If an SAE is reported during the trial, the investigator will check with the Independent Data Monitoring Committee whether to continue the trial and whether any changes should be made to the protocol. The Independent Data Monitoring Committee will provide the investigator with the results of the discussion in writing.

If the investigator determines that precautionary emergency measures are warranted, depending on the importance and scope of the report, actions may include suspension of enrollment and emergency communication to all participating sites.

11. COMPLIANCE AND DEVIATION FROM THE PROTOCOL

1) The investigator or subinvestigator shall conduct the clinical trial in compliance with this protocol.
2) The investigator or subinvestigator shall record the details and reasons for all deviations from the study protocol.
3) In the event of deviation from the protocol for the purpose of avoiding immediate danger to subjects or for other unavoidable medical reasons, the investigator shall immediately submit a document describing the details of the deviation and the reasons for it to the head of the implementing medical institution, and shall also promptly report the contents of said document to the Trial Review Committee via the head of the implementing medical institution. The contents of said documents shall be promptly reported to the Clinical Trial Review Committee via the head of the investigational institution.

12. CHANGES TO THE CLINICAL TRIAL PROTOCOL, CASE REPORT FORM, OR ANALYSIS PLAN

12.1. Revision of Clinical Trial Protocol and Case Report Form

The following procedures shall be followed when revising the clinical trial protocol and case report forms.

1) When the investigator becomes aware of matters related to the quality, efficacy and safety of the investigational drug or other information important for the proper conduct of the clinical trial, the investigator shall revise the relevant protocol as necessary. When a revision is made, a history of the revision shall be prepared and stored.
2) The coordinating investigator shall revise the case report form as necessary in conjunction with the revision of the protocol or for other reasons. Whenever necessary, the coordinating investigator shall revise the case report form in conjunction with the revision of the protocol or for other reasons.
3) The investigator shall promptly submit the revised protocol and revised case report form to the head of the site and obtain approval from the investigational review committee via the head of the site.
4) The same procedure shall be followed when the investigator amends the protocol and case report form within the scope of the investigator's acceptable instructions from the head of the site based on the opinion of the investigational review committee.

12.2. Changes in statistical analysis plan

If the statistical analysis manager changes the contents of the statistical analysis plan, all changes shall be documented in the statistical analysis report for this clinical trial. In addition, the circumstances of any changes to the statistical analysis plan shall be recorded.

13. DISCONTINUATION, SUSPENSION, OR TERMINATION OF THE CLINICAL TRIAL

13.1. Criteria for discontinuation or suspension of the clinical trial as a whole

When the following information is obtained and it is considered difficult to continue the entire clinical trial, the coordinating investigator will consult with the principal investigator and make a decision to discontinue or suspend the entire clinical trial.

- When it becomes difficult to ensure the safety of this clinical trial due to new safety information or serious adverse event information concerning the investigational drug, etc.
- When the site has committed a serious violation of the drug GCP ordinance or a serious deviation from the clinical trial protocol and no improvement has been made.
- Other new information obtained during the conduct of the clinical trial that may necessitate discontinuation or suspension of the clinical trial.

13.2. Procedures for discontinuation or suspension of a clinical trial as a whole

If the coordinating investigator, after consultation with other investigators, decides to discontinue or suspend the entire clinical trial, he/she shall promptly notify the head of the investigational institution and the regulatory authorities in writing to that effect and the reasons in detail. In addition, the investigator shall promptly inform the subjects undergoing the clinical trial and take appropriate measures such as changing to appropriate treatment.

13.3. Procedures for discontinuation or suspension of this clinical trial at an individual clinical site

When the investigator discontinues or suspends a clinical trial, the investigator shall promptly notify the head of the site in writing to that effect and explain the details of the discontinuation or suspension in writing in detail. When notified of the discontinuation or suspension of a clinical trial, the investigator shall promptly notify in writing all investigators and regulatory authorities involved in the said clinical trial to that effect and explain the discontinuation or suspension in detail.

13.4. Termination of this clinical trial

After the completion of the clinical trial, the investigator shall notify the head of the investigational institution in writing that the clinical trial has been completed and report a summary of the results of the clinical trial in writing.

14. DATA MANAGEMENT

14.1. Data management procedure

Detailed procedures for data management shall be described in the data management plan.

14.2. Data Collection

The investigator or sub-investigator shall prepare a case report using Electronic Data Capture (EDC) that meets the requirements of 21 CFR Part 11, the Pharmaceutical GCP Ordinance, and the ER/ES guidelines. The investigator or subinvestigator shall prepare a case report using EDC. The investigator or subinvestigator shall make any changes, corrections or additions to the contents of the case report form on the EDC that generated the case report form, and record all of the changes, corrections or additions as electronic information. When a subinvestigator prepares a case report form or when a collaborator transcribes a case report form from source documents (source data), the investigator shall check the contents of the case report form before submitting it to the EDC and confirm that there are no problems. The investigator provides the final electronic case report form to the institution on an electronic medium (CD-R, etc.). The investigator shall ensure the readability and archivability of the electronic case report form.

When using the EDC system, the site should receive training on EDC and refer to the manual for details on how to input data.

14.3. Identification of documents that are directly described in the case report and should be interpreted as source documents (source data)

In this clinical trial, the following documents and others shall be considered source documents (source data).

1) Medical records, nursing records, clinical laboratory data, imaging films, and other records that form the basis for preparing case reports. Data stored in electronic medical records are also considered source documents.
2) Records of investigational drug administration
3) Documents or records related to the clinical trial that are required under the GCP ordinance for pharmaceutical products related to the clinical trial

Of the data described in the case report form, the following items shall be regarded as source documents (source data) when they are described in the case report form. However, if the data are recorded in the medical record, the medical record shall be regarded as the source documents (original data).

1) Purpose of concomitant medications/adjunctive therapy
2) Determination of the extent of adverse events, outcomes (including results at follow-up), severity, and causal relationship to the study treatment and the basis for the determination
3) Reasons for discontinuation of clinical trials by subjects
4) Comments by the investigator or subinvestigator

15. RETENTION OF SOURCE DOCUMENTS AND OTHER RECORDS

15.1. Retention of records by the clinical site

Documents or records pertaining to a clinical trial to be retained at the investigational site as stipulated in the Pharmaceutical GCP ordinance shall be retained by the hospital director until the later of the following dates.

1) The date on which five years have elapsed since the date of marketing approval for the relevant indication of the test product (if development has been discontinued, the date on which three years have elapsed since the date on which the decision to discontinue

development was made). However, for drugs that are subject to post-approval reexamination in accordance with the provisions of the “Law Concerning Quality, Efficacy and Safety Assurance of Pharmaceuticals and Medical Devices” and for which the period until the reexamination is completed exceeds five years, the date on which the reexamination is completed.

2) The date on which 3 years have elapsed since the discontinuation or termination of the clinical trial.

The investigator shall notify the clinicalsite when the records to be retained by the investigational site or the investigational review committee are no longer required to be retained.

15.2. Retention of records by principal investigators

Documents or records pertaining to clinical trials to be retained by principal investigators as stipulated in the Pharmaceutical GCP Ordinance shall be retained at a storage location deemed appropriate until the later of the following dates.

1) Three years have elapsed since the date of marketing approval for the relevant indication of the test product (if development has been discontinued, three years have elapsed since the date on which the decision to discontinue development was made).
2) The date on which 3 years have elapsed since the discontinuation or termination of the clinical trial.

16. RETENTION OF SAMPLES AND USE OF SAMPLES PROVIDED BY OTHER INSTITUTIONS

16.1. Retention of sample

Samples will be stored in the Laboratory of Gastroenterology, Graduate School of Medicine, Chiba University for a period not exceeding 20 years after completion of the clinical trial. The method of preservation shall be cryopreservation using liquid nitrogen.

16.2. Disposal of samples

If a subject withdraws consent, if a specimen is mistaken or contaminated or is strongly suspected of being mistaken or contaminated, or if the need for disposal is otherwise recognized, the anonymizing numbers, etc., will be deleted and the specimen will be disposed of.

16.3. Reuse of samples

Secondary use of samples and sample-related information (genomic or epigenomic analysis at Chiba University) may be conducted. In such cases, the ethical review and method of obtaining consent shall be in accordance with the corresponding ethical guidelines, etc.

17. SOURCE DOCUMENT VERIFICATION

The head of the investigational site and the investigator ensure that the personnel in charge of monitoring, audits and Institutinal Review Board or regulatory authorities have access to all records, including source documents. In anddtion , The head of the investigational site and the investigator confirm that the clinical trial is conducted appropriately and that the data are sufficiently reliable. The method and timing of source document verification shall be specified separately in the monitoring procedures.

18. Quality Assurance

In order to ensure that clinical trials are conducted and that data preparation, recording and reporting are appropriately conducted in compliance with the protocol and the GCP ordinances for pharmaceutical products, independent auditors from the departments related to the clinical trials, including the department in charge of monitoring, will conduct audits at the investigational

site and other sites where the clinical trials are conducted to confirm that quality control is appropriately conducted.The audit shall be conducted by an auditor independent from the departments related to the clinical trial, including the department in charge of monitoring, to confirm that quality control is appropriately implemented. Audits shall be conducted in accordance with the “Standard Operating Procedures for Audits” and “Audit Plan” separately stipulated.

19. QUALITY CONTROL FOR THIS STUDY

19.1 Training of study site personnel

The Principal Investigator will ensure that appropriate training relevant to the study is given to all of these staff, and that any new information relevant to the performance of this study is forwarded to the staff involved.

19.2. Quality control

In conducting clinical trial monitoring, the investigator shall consider priorities and develop a systematic risk-based approach. In the event of any deviation from this protocol, the investigator or subinvestigator shall follow the provisions of this protocol. The investigator or subinvestigator shall prepare the case report form in accordance with this protocol. The investigator will ensure that all data and other records in the case report form are accurate and complete. If any of the data in the case report form is inconsistent in any way with the original data, the investigator shall prepare and maintain a record explaining the reason for the inconsistency. The investigator shall designate a person who is not engaged in the relevant clinical trial at the site subject to the monitoring as a monitor and have him/her conduct the monitoring in accordance with the monitoring protocol that has been reviewed by the investigational review committee. The monitors shall confirm the following items in accordance with the monitoring protocol separately prepared.

- The human rights, safety and welfare of subjects are protected.
- The clinical trial is conducted in compliance with the Pharmaceutical GCP ordinance, the latest clinical trial protocol, and the procedure manual for the relevant clinical trial.
- To confirm that the data, etc. reported by the investigator or subinvestigator are accurate and complete and check them against the source documents and other clinical trial-related records.

The person in charge of data management shall formulate the data management plan in accordance with the separately established standard operating procedures and shall ensure the quality of the data through quality control at each stage of data handling.

20. ETHICAL CONDUCT AND GOOD CLINICAL PRACTICE(GCP)

This clinical trial will be conducted in accordance with the “Declaration of Helsinki”,the “Law Concerning Quality, Efficacy and Safety Assurance of Pharmaceuticals and Medical Devices” and the “Pharmaceutical GCP Ministerial Ordinance”. In addition, this clinical trial shall be conducted in compliance with the protocol and procedures for this clinical trial.

In selecting subjects, the investigator or subinvestigator shall carefully consider the appropriateness of requesting subjects to participate in this clinical trial based on the selection criteria and exclusion criteria from the perspective of protecting human rights, taking into consideration the subjects' health condition, symptoms, age, gender, ability to consent, degree of dependence on the investigator, and participation status in clinical trials including other clinical trials.

21. INSTITUTIONAL REVIEW BOARD(IRB)

Prior to the implementation of this clinical trial, the Institutional Review Board of the site will review the ethical, scientific and medical appropriateness of this clinical trial. This clinical trial shall be conducted after obtaining the approval of the Clinical Trial Review Committee. If the result of the deliberation by the Institutional Review Board is “Approval with modification,” the protocol or case report form, consent document, etc. shall be modified and approved based on the result of the deliberation, and then this clinical trial shall be conducted. The Institutional Review Board shall also continuously review whether or not this clinical trial is being conducted appropriately at least once a year.

22. HEALTH DAMAGE COVERAGE AND INSURANCE

If a subject suffers health problems as a result of participation in this clinical trial, the investigator will provide treatment and appropriate medical care for the subject's recovery.

As a response to liability for compensation and indemnification arising from health damage caused by this clinical trial, the investigators, subinvestigators, medical institution, Clinical Trial Coordinating Committee and other parties involved in this clinical trial will be covered by the Physician-initiated Clinical Trial Insurance.

23. COST BURDEN FOR THIS TRIAL

The investigational drug to be used in this study will be provided by AstraZeneca. Payment of the burden reduction fee and other expenses to subjects will be in accordance with the rules separately established by each investigational site.

24. TRIAL FUNDS AND CONFLICT OF INTEREST

This clinical trial will be conducted with funds provided to Chiba University Hospital by AstraZeneca. AstraZeneca personnel will not be involved in the conduct or analysis of this clinical trial when the investigators conduct the clinical trial.

In addition, prior to the deliberation of the Clinical Trial Review Committee at each site, the Conflict of Interest Management Committee will deliberate whether conflicts of interest are being properly managed, and it will be confirmed that the investigators and collaborators are not in a state of conflict of interest.

25. PROVISION OF THE INVESTIGATIONAL PRODUCTS AND INTELLECTUAL PROPERTY RIGHTS

In this study, the investigational products are provided by AstraZeneca Pharmaceuticals, Inc. (England) The ownership of the rights to any inventions, discoveries, or improvements of any nature (the “Inventions”) derived from this study will be decided in accordance with the contracts with AstraZeneca.

26.PUBLICATION

The results of the clinical trial will be submitted as a report by the Principal Investigator to the head of the clinical trial site upon completion of this clinical. Results that do not meet expected outcomes despite the proper conduct of the clinical trial must also be disclosed in publication. In case of disclosure of the results to the public, the subject’s personal information must be kept confidential.

27. REGISTRATION FOR CLINICAL TRIAL

This clinical trial will be registered in Japan Registry of Clinical Trial(jRCT) (https://jrct.niph.go.jp/) prior to obtaining consent from the first subject.

28. STUDY IMPLEMANTATION GRPUP

See ANNEX TO PROTOCOL 1

29. LIST OF INVESTIGATIONAL PRODUCT(S) FOR THIS STUDY

Investigational product	Dosage form and strength	Manufacturer
Durvalumab	*50 mg/mL solution for infusion after dilution*	MedImmune/AstraZeneca
Tremelimumab	*20 mg/mL solution for infusion after dilution*	MedImmune/AstraZeneca

30. LIST OF REFERENCES

1) Ministry of Health, Labor and Welfare 2014 Patient Survey
2) Ministry of Health, Labor and Welfare 2017 Vital Statistics
3) El Serag HB, et al., Hepatocellular Carcinoma N Engl J Med 2011
4) The Japan Society of Hepatology Clinical Practice Guidelines for Hepatocellular Carcinoma
5) Llovet JM, Ricci S, Mazzaferro V, et al. Sorafenib in advanced hepatocellular carcinoma. N Engl J Med 2008; 359: 378-90.
6) Cheng AL, Kang YK, Chen Z, et al. Efficacy and safety of sorafenib in patients in the Asia-Pacific region with advanced hepatocellular carcinoma: a phase III randmised, double- blind, placebo-controlled trial. Lancet Oncol 2009; 10:25-34.
7) Bruix J, Qin S, Merle P, et al. Regorafenib for patients with hepatocellular carcinoma who progressed on sorafenib treatment (RESORCE): a randmised, double-blind, placebo-controlled, phase 3 trial. Lancet 2017; 389: 56-66.
8) Kudo M, Finn RS, Qin S, et al. Lenvatinib versus sorafenib in first-line treatment of patients with unresectable hepatocellular carcinoma: a randomised phase 3 non-inferiority trial. Lancet 2018 Mar 24, 391 (10126): 1163-1173
9) Zhu AX, Kan YK, Yen CJ, et al. Ramucirumab after sorafenib in patients with advanced hepatocellular carcinoma and increased α-fetoprotein concentrations (REACH-2): a randomised, double-blind, placebo-controlled, phase 3 trial. Lancet Oncol.2019 Feb;20(2):282-296.
10) Bonze D, Meirson T, Azoulay D. Atezolizumab and Bevacizumab in Hepatocellular Carcinoma. NEJM. 2020 Aug 13;383(7):693-694.
11) Costentin CE, Ferroone CR, Arellano RS, et al. Hepatocellular Carcinoma with Macrovascular Invasion: Defining the Optimal Treatment Strategy Liver Cancer 2017; Nov;6(4):360-374.
12) Dunn GP, Old LJ, Schreiber RD. The three Es of cancer immunoediting. Annu Rev Immunol 2004;22:329-60.
13) Keir ME, Butte MJ, Freeman GJ, Sharpe AH. PD-1 and its ligands in tolerance and immunity. Annu Rev Immunol. 2008;26:677-704.
14) Okazaki T, Honjo T. PD-1 and PD-1 ligands: from discovery to clinical application. Int Immunol 2007;19(7):813-824.
15) Qin A, Coffey DG, Warren EH, Ramnath N. Mechanisms of immune evasion and current status of checkpoint inhibitors in non-small cell lung cancer. Cancer Med 2016;9:2567–2578.
16) Pardoll DM. The blockade of immune checkpoints in cancer immunotherapy. Nat Rev Cancer 2012;12:252-64.
17) Brahmer JR, Tykodi SS, Chow LQM, Hwu WJ, Topalian SL, Hwu P, et al. Safety and activity of

anti-PD-L1 antibody in patients with advanced cancer. N Engl J Med. 2012 Jun;366 (26):2455-65.

18) Hirano F, Kaneko K, Tamura H, Dong H, Wang S, Ichikawa M, et al. Blockade of B7-H1 and PD-1 by monoclonal antibodies potentiates cancer therapeutic immunity. Cancer Res. 2005;65(3):1089-96.

19) Iwai Y, Ishida M, Tanaka Y, Okazaki T, Honjo T, Minato N. Involvement of PD-L1 on tumor cells in the escape from host immune system and tumor immunotherapy by PD-L1 blockade. Proc Natl Acad Sci USA. 2002 Sep 17;99:12293-7.

20) Okudaira K, Hokari R, Tsuzuki Y, Okada Y, Komoto S, Watanabe C, et al. Blockade of B7-H1 or B7-DC induces an anti-tumor effect in a mouse pancreatic cancer model. Int J Oncol. 2009 Sep;35(4):741-9.

21) Topalian SL, Hodi FS, Brahmer JR, Gettinger SN, Smith DC, McDermott DF, et al. Safety, activity, and immune correlates of anti-PD-1 antibody in cancer. N Engl J Med. 2012;366:2443-54.

22) Zhang C, Wu S, Xue X, Li M, Qin X, Li W, et al. Anti-tumor immunotherapy by blockade of the PD-1/PD-L1 pathway with recombinant human PD-1-IgV. Cytotherapy. 2008;10(7):711-9.

23) Powles T, Eder JP, Fine GD, Braiteh FS, Loriot Y, Cruz C, et al. MPDL3280A (anti-PD-L1) treatment leads to clinical activity in metastatic bladder cancer. Nature. 2014 Nov 27;515(7528):558-62.

24) Rizvi N, Brahmer J, Ou S-H, Segal NH, Khleif SN, Hwu WJ. Safety and clinical activity of MEDI4736, an anti-programmed cell death-ligand-1 (PD-L1) antibody, in patients with nonsmall cell lung cancer (NSCLC). J Clin Oncol 2015;33:Abstract 8032.

25) Segal NH, Ou S-HI, Balmanoukian AS, Fury MG, Massarelli E, Brahmer JR, et al. Safety and efficacy of MEDI4736, an anti-PD-L1 antibody, in patients from a squamous cell carcinoma of the head and neck (SCCHN) expansion cohort. J Clin Oncol 2015;33:Abstract 3011.

26) Alexandrov LB, Nik-Zainal S, Wedge DC, Aparicio SAJR, Behjati S, Blankin AV, et al. Signatures of mutational processes in human cancer. Nature. 2013 Aug 22;500:415-21.

27) Fife BT, Bluestone JA. Control of peripheral T-cell tolerance and autoimmunity via the CTLA-4 and PD-1 pathways. Immunol Rev. 2008;224:166-82.

28) El-Khoueiry AB, Sangro B, Yau T, et al. Nivolumab in patients with advanced hepatocellular carcinoma (CheckMate 040): an open-label, non-comparative, phase 1/2 dose escalation and expansion trial Lancet 2017; Jun 24;389(10088):2492-2502.

29) Zhu AX, Finn RS, Edeline J, et al. Pembrolizumab in patients with advanced hepatocellular carcinoma previously treated with sorafenib (KEYNOTE-224): a non-randomised, open-label phase 2 trial. Lancet Oncol. 2018; Jul;19(7):940-952.

30) Kudo M. Targeted and immune therapies for hepatocellular carcinoma: Predictions for 2019 and beyond. World J Gastroenterol 2019 Feb 21;25(7):789-807.

31) Kelly RK, Abou-Alfa GK, Bendell JC, et al. Phase I/II study of durvalumab and tremelimumab in patients with unresectable hepatocellular carcinoma (HCC): Phase I safety and efficacy analyses. J Clin Oncol.2017; Abstract 4073.

32) Pedroni E, Bacher R, Blattmann H, Böhringer T, Coray A, Lomax A, Lin S, Munkel G, Scheib S, Schneider U, et al. The 200-MeV proton therapy project at the Paul Scherrer Institute: conceptual design and practical realization. Med Phys. 1995 Jan;22(1):37-53.

33) Mohamad O., Makishima H., Kamada T. Evolution of Carbon Ion Radiotherapy at the National Institute of Radiological Sciences in Japan. Cancers (Basel) 2018;10(3): pii: E66

34) Kasuya G, Kato H, Yasuda S, Tsuji H, Yamada S, Haruyama Y, Kobashi G, Ebner DK, Okada NN, Makishima H, Miyazaki M, Kamada T, Tsujii H; Liver Cancer Working Group. Progressive

hypofractionated carbon-ion radiotherapy for hepatocellular carcinoma: Combined analyses of 2 prospective trials. Cancer. 2017;123(20):3955-3965

35) Fukuda K, Okumura T, Abei M, Fukumitsu N, Ishige K, Mizumoto M et al. Long-term outcomes of proton beam therapy in patients with previously untreated hepatocellular carcinoma. Cancer Sci. 2017;108(3):497-503

36) Sorin Y, Ikeda K, Kawamura Y, Fujiyama S, Kobayashi M, Hosaka T. Effectiveness of Particle Radiotherapy in Various Stages of Hepatocellular Carcinoma: A Pilot Study. Liver Cancer. 2018 Oct;7(4):323-334

37) Igaki H, Mizumoto M, Okumura T, Hasegawa K, Kokudo N, Sakurai H. A systematic review of publications on charged particle therapy for hepatocellular carcinoma. Int J Clin Oncol. 2018;23(3):423-433

38) Komatsu S, Fukumoto T, Demizu Y, Miyawaki D, Terashima K, Niwa Y, Mima M, Fujii O, Sasaki R, Yamada I, Hori Y, Hishikawa Y, Abe M, Ku Y, Murakami M. The effectiveness of particle radiotherapy for hepatocellular carcinoma associated with inferior vena cava tumor thrombus. J Gastroenterol. 2011 Jul;46(7):913-20.

39) Lee SU, Park JW, Kim TH, Kim YJ, Woo SM, Koh YH, Lee WJ, Park SJ, Kim DY, Kim CM. Effectiveness and safety of proton beam therapy for advanced hepatocellular carcinoma with portal vein tumor thrombosis. Strahlenther Onkol. 2014 Sep;190(9):806-14.

40) Sugahara S, Nakayama H, Fukuda K, Mizumoto M, Tokita M, Abei M, Shoda J, Matsuzaki Y, Thono E, Tsuboi K, Tokuuye K. Proton-beam therapy for hepatocellular carcinoma associated with portal vein tumor thrombosis. Strahlenther Onkol 2009;185:782–788.

41) Antonia SJ, Daniel VD, Vicente D, et al. Durvalumab after Chemoradiotherapy in Stage III Non-Small-Cell Lung Cancer. N Engl J Med. 377;1919-29. 2017

42) Rizvi NA, Cho BC, Reinmuth N, et al. Durvalumab with or without tremelimumab vs platinum-based chemotherapy as first-line treatment for metastatic non-small cell lung cancer: MYSTIC Annals of Oncology.2018 Dec 1;29;supp10

43) Helm A, Ebner DK, Tinganelli W, Simoniello P, Bisio A, Marchesano V, Durante M, Yamada S, Shimokawa T. Combining Heavy-Ion Therapy with Immunotherapy: An Update on Recent Developments. Int J Part Ther. 2018 Summer;5(1):84-93.

44) Takahashi Y, Yasui T, Minami K, Tamari K, Hayashi K, Otani K, Seo Y, Isohashi F, Koizumi M, Ogawa K. Carbon ion irradiation enhances the antitumor efficacy of dual immune checkpoint blockade therapy both for local and distant sites in murine osteosarcoma. Oncotarget. 2019 Jan 18;10(6):633-646.

45) Iijima M, Okonogi N,Izumi Nakajima N, Morokoshi Y, Kanda H, Yamada T, Kobayashi Y, Banno K, Wakatsuki M, Yamada S, Kamada T, Aoki D, Hasegawa S. Significance of PD-L1 expression in carbon-ion radiotherapy for uterine cervical adeno/adenosquamous carcinoma. J Gynecol Oncol. 2020;31:e19.

46) Golden EB, Pellicciotta I, Demaria S, et al. The convergence of radiation and immunogenic cell death signaling pathways. Front Oncol, 2012 Aug 7;2:88.

47) Onishi M, Okonogi N, Oike T, Yoshimoto Y, Sato H, Suzuki Y, Kamada T, Nakano T. High linear energy transfer carbon-ion irradiation increases the release of the immune mediator high mobility group box 1 from human cancer cells. J Radiat Res. 2018 Sep 1;59(5):541-546.

48) Matsunaga A, Ueda Y, Yamada S, Harada Y, Shimada H, Hasegawa M, Tsujii H, Ochiai T, Yonemitsu Y. Carbon-ion beam treatment induces systemic antitumor immunity against murine squamous cell carcinoma. Cancer. 2010 Aug 1;116(15):3740-8.

49) Ando K, Fujita H, Hosoi A, Ma L, Wakatsuki M, Seino KI, Kakimi K, Imai T, Shimokawa T,

Nakano T. Intravenous dendritic cell administration enhances suppression of lung metastasis induced by carbon-ion irradiation. J Radiat Res. 2017 Jul 1;58(4):446-455.

50) Sato H, Niimi A, Yasuhara T, Permata TBM, Hagiwara Y, Isono M, Nuryadi E, Sekine R, Oike T, Kakoti S, Yoshimoto Y, Held KD, Suzuki Y, Kono K, Miyagawa K, Nakano T, Shibata A. DNA double-strand break repair pathway regulates PD-L1 expression in cancer cells. Nat Commun. 2017 Nov 24;8(1):1751. doi: 10.1038/s41467-017-01883-9.

51) Oike T, Niimi A, Okonogi N, Murata K, Matsumura A, Noda SE, Kobayashi D, Iwanaga M, Tsuchida K, Kanai T, Ohno T, Shibata A, Nakano T. Visualization of complex DNA double-strand breaks in a tumor treated with carbon ion radiotherapy. Sci Rep. 2016 Mar 1;6:22275. doi: 10.1038/srep22275.

52) Stewart R, Morrow M, Hammond SA, Mulgrew K, Marcus D, Poon E, et al. Identification and characterization of MEDI4736, an antagonistic anti-PD-L1 monoclonal antibody. Cancer Immunol Res 2015;3(9):1052-62.

53) Tarhini AA, Kirkwood JM. Tremelimumab (CP-675,206): a fully human anticytotoxic T lymphocyte-associated antigen 4 monoclonal antibody for treatment of patients with advanced cancers. Expert Opin Biol Ther 2008;8:1583-93.

54) Pardee AD, Butterfield LH. Immunotherapy of hepatocellular carcinoma: Unique challenges and clinical opportunities. Oncoimmunology 2012;1(1):48-55.

55) Gao Q, Wang XY, Qiu SJ, Yamato I, Sho M, Nakajima Y, et al. Overexpression of PD-L1 significantly associates with tumor aggressiveness and postoperative recurrence in human hepatocellular carcinoma. Clin Cancer Res 2009;15(3):971-9.

56) Hato T, Goyal L, Greten TF, Duda DG, Zhu AX. Immune checkpoint blockade in hepatocellular carcinoma: current progress and future directions. Hepatology 2014;60(5):1776-82.

57) Miroux C, Vausselin T, Delhem N. Regulatory T cells in HBV and HCV liver diseases: implication of regulatory T lymphocytes in the control of immune response. Expert Opin Biol Ther 2010;10(11):1563-72.

58) Golden-Mason L, Palmer B, Klarquist J, Mengshol JA, Castelblanco N, Rosen HR. Upregulation of PD-1 expression on circulating and intrahepatic hepatitis C virus-specific CD8+ T cells associated with reversible immune dysfunction. J Virol 2007;81(17):9249-58.

59) Peng G, Li S, Wu W, Tan X, Chen Y, Chen Z. PD-1 upregulation is associated with HBV-specific T cell dysfunction in chronic hepatitis B patients. Mol Immunol 2008;45(4):963-70.

60) Klein JP, Logan B, Harhoff M, Andersen PK. Analyzing survival curves at a fixed point in time. Stat Med 2007;26(24):4505-19.

61) Sangro B, Gomez MC, Mata M, et al. A clinical trial of CTLA-4 blockade with tremelimumab in patients with hepatocellular carcinoma and chronic hepatitis Cq. J Hepatol. 2013 Jul;59(1):81-8.

62) Duffy AG, Ulahannan SV, Makorova-Rusher O, Rahmer O, Wedemeyer H, Pratt D, et al. Tremelimumab in combination with ablation in patients with advanced hepatocellular carcinoma. J Hepatol 2017;66(3):545-51.

63) Melero I, Sangro B, Cheung Yae T, Hsu C, Kudo M, Crocenzi TS, et al. Nivolumab dose escalation and expansion in patients with advanced hepatocellular carcinoma (HCC): The CheckMate 040 study. J Clin Oncol 2017;35 (suppl 4S:abstract 226).

64) Kelly RK, Sangro B, Harris W, et al. Efficacy, tolerability, and biologic activity of a novel regimen of tremelimumab in combination with durvalumab for patients with advanced hepatocellular carcinoma, J Clin Oncol. 2020; Abstract 4508.

65) Takahashi Y, Fujikawa K, Sagawa T, et al. A phase 1 study to assess the safety and tolerability of tremelimumab alone and in combination with MEDI4736 in Japanese patients withadvanced solid

malignancies. Eur J Cancer. 2015(suppl 3; abst512)

66) Wang E, Kang D, Bae KS, Marshall MA, Pavlov D, Parivar K. Population pharmacokinetic and pharmacodynamics analysis of tremelimumab in patients with metastatic melanoma. J Clin Pharmacol 2014;54(10):1108-16.

67) Narwal R, Roskos LK, Robbie GJ. Population pharmacokinetics of sifalimumab, an investigational anti-interferonalpha monoclonal antibody, in systemic lupus erythematosus. Clin Pharmacokinet 2013;52:1021–27.

68) Ng CM, Lum BL, Gimenez V, Kelsey S, Allison D. Rationale for fixed dosing of pertuzumab in cancer patients based on population pharmacokinetic analysis. Pharm Res 2006;23(6):1275–84.

69) Wang DD, Zhang S, Zhao H, Men AY, Parivar K. Fixed dosing versus body size based dosing of monoclonal antibodies in adult clinical trials. J Clin Pharmacol 2009;49(9):1012–24.

70) Wolchok JD, Kluger H, Callahan MK, Postow MA, Rizvi NA, Lesokhin AM, et al. Nivolumab plus ipilimumab in advanced melanoma. N Engl J Med 2013;369:122-33.

71) Reed GB Jr, Cox AJ Jr. The human liver after radiation injury. A form of veno-occlusive disease. Am J Pathol. 1966 Apr;48(4):597-611.

72) Kanai T, Endo M, Minohara S, Miyahara N, Koyama-ito H, Tomura H, Matsufuji N, Futami Y, Fukumura A, Hiraoka T, Furusawa Y, Ando K, Suzuki M, Soga F, Kawachi K. Biophysical characteristics of HIMAC clinical irradiation system for heavy-ion radiation therapy. Int J Radiat Oncol Biol Phys. 1999 Apr 1;44(1):201-10.

73) Shibuya K, Ohno T, Terashima K, Toyama S, Yasuda S, Tsuji H, et al. Short-course carbon-ion radiotherapy for hepatocellular carcinoma: a multi-institutional retrospective study. Liver Int. 2018;38(12):2239-47.

74) Yasuda S, Kato H, Imada H, et al. Long-Term Results of High-Dose 2-Fraction Carbon Ion Radiation Therapy for Hepatocellular Carcinoma. Adv Radiat Oncol. 2019 Sep 27;5(2):196-203.

75) Shiba S, Shibuya K, Katoh H, et al. A comparison of carbon ion radiotherapy and transarterial chemoembolization treatment outcomes for single hepatocellular carcinoma: a propensity score matching study Radiat Oncol. 2019 Aug 2;14(1):137.

76) Shiba S, Shibuya K, Okamoto M, et al. Clinical impact of Hypofractionated carbon ion radiotherapy on locally advanced hepatocellular carcinoma. Radiat Oncol 2020 Aug 14;15(1):195.

77) Makishima S, Yasuda S, Isozaki Y, et al. Single fraction carbon ion radiotherapy for colorectal cancer liver metastasis: A dose escalation study. Cancer Sci. 2019 Jan;110(1):303-309.

78) Ebner DK, Tinganelli W, Helm A, Bisio A, Yamada S, Kamada T, Shimokawa T, Durante M. The immunoregulatory potential of particle radiation in cancer therapy. Front Immunol. 2017;8:1–8.

Clinical Study Protocol

A phase Ib study of durvalumab (MEDI4736) ± tremelimumab combined with Carbon ion radiotherapy in advanced hepatocellular carcinoma patients with macrovascular invasion

Study Number: CCRC2002
Investigational drugs : Durvalumab, Tremelimumab（Concominant therapy : Carbon ion radiotherapy）
Version Number　2.1
Date　23 May 2023

Revision history

Date	Version Number
24 Feb 2021	1.0
26 Mar 2021	1.1
20 Apl 2021	1.2
24 May 2022	2.0
23 May 2023	2.1

This protocol includes information and data that contain privileged or confidential information and, is provided only to the investigators, clinical team staff, associates, IRBs, or the Data Monitoring Committee. Therefore, this information must not be made public without written permission from the Chief Investigator, and AstraZeneca except when explaining to subjects. These restrictions on disclosure will apply equally to all or part of the data obtained in the clinical trial for publishing or presentation.

＜Abbreviation and terms＞

Definitions of abbreviations, acronyms, and terms in this study protocol are as follows

Abbreviation /terms	Definition
AE	adverse event
AESI	adverse event of special interest
AFP	α-fetoprotein
AFP-L3	α-fetoprotein - L3
ALP	alkaline phosphatase
ALT	alanine aminotransferase
AMED	Japan Agency for Medical Research and Development
APTT	activated partial thromboplastin time
AST	aspartate aminotransferase
AUC	area under the blood concentration time curve
BICR	blinded independent central review
BP	blood pressure
BSA	body surface area
CD	cluster of differentiation
CI	confidence interval
CIRT	carbon-ion radiotherapy
Cmax	maximum observed concentration
Cmin	minimum concentration during a dosing interval
COI	conflicts of interest
CRP	C-reactive protein
CT	computed tomography
Ctrough	drug concentration at the end of the dosing interval
CTCAE	common terminology criteria for adverse events
CTLA-4	cytotoxic T lymphocyte antigen 4
CTV	clinical target volume
DAMP	damage-associated molecular pattern
DC	dendritic cell
DLT	dose limiting toxicity
DMC	data monitoring committee
DNA	deoxyribonucleic acid
ECG	electrocardiogram
ECOG	Eastern Cooperative Oncology Group
eCRF	electronic case report form
EDC	electronic data capture
EGFR	epidermal growth factor receptor
ER	electronic record
ES	electronic signature
FAS	full analysis set
FTIH	first-time-in-human
FU	follow-up
G-CSF	granulocyte-colony stimulating factor
GCP	Good Clinical Practice
GI	gastrointestinal
HAIC	hepatic arterial infusion chemotherapy

Abbreviation /terms	Definition
HBc	hepatitis B core
HBs	hepatitis B surface
HBV	hepatitis B virus
HCC	hepatocellular carcinoma
HCV	hepatitis C virus
HIMAC	Heavy Ion Medical Accelerator in Chiba
HIV	human immunodeficiency virus
HMGB1	high mobility group box protein1
HR	hazard ratio
HR	heart rate
HRQoL	health-related quality of life
ICH	International Council for Harmonisation of Technical Requirements for Pharmaceuticals for Human Use
ICI	immune checkpoint inhibitor
IFN-γ	interferon-γ
Ig	immunoglobulin
IL	interleukin
ILD	interstitial lung disease
imAE	immune mediated adverse event
IR	investigational regimen
IRB	institutional review board
KL-6	Krebs von den Lungen-6
LDH	lactate dehydrogenase
LET	linear energy transfer
LFT	liver function test
mAb	monoclonal antibody
MedDRA	Medical Dictionary for Regulatory Activities
MOA	mechanism of action
mRECIST	modified RECIST
MRI	magnetic resonance imaging
MST	mean survival time
MTD	maximum tolerated dose
MVI	macroscopic vascular invasion
NIRS	National Institute of Radiological Sciences
NSCLC	non-small cell lung cancer
NTL	non-target lesion
NYHA	New York Heart Association
OAR	off-axis ratio
ORR	objective response rate
OS	overall survival
PD	progressive disease
PD-1	programmed cell death 1
PD-L1	programmed cell death ligand 1
PD-L2	programmed cell death ligand 2
PIVKA-II	protein induced by vitamin K absence-II
PK	Pharmacokinetics

Abbreviation /terms	Definition
PMDA	Pharmaceuticals and Medical Devices Agency
PPS	per protocol set
PR	partial response
PRO	patient reported outcome
PS	performance status
PT	preferred term
PT-INR	prothrombin time-international normalized ratio
PTV	planning target volume
QxD	quaque x day
QxW	quaque x week
QST	National Institutes for Quantum and Radiological Science and Technology
QTcF	corrected QT interval using Fridericia's formula
Q2W	quaque 2 weeks
Q3W	quaque 3 weeks
Q4W	quaque 4 weeks
RBE	relative biological effectiveness
RECIST	Response Evaluation Criteria In Solid Tumors
RESORCE	Regorafenib after Sorafenib in Patients with Hepatocellular Carcinoma
RFA	radiofrequency ablation
RILD	radiation induced liver damage
RNA	ribonucleic acid
SAE	serious adverse event
SD	stable disease
SHARP	Sorafenib Hepatocellular Carcinoma Assessment Randomized Protocol
SOP	standard operating procedure
SP-D	surfactant protein-D
SpO2	saturation of peripheral oxygen
TACE	transcatheter arterial chemoembolization
TAI	transcatheter arterial infusion
TBL	total bilirubin
TKI	tyrosine kinase inhibitor
TL	target lesion
TLR4	Toll-like receptor 4
TMGs	Toxicity Management Guidelines
TSH	thyroid stimulating hormone
TTP	time to progression
ULN	upper limit of normal
VEGF	vascular endothelial growth factor

TABLE OF CONTENTS

0. CLINICAL TRIAL OVERVIEW....9
1. INTRODUCTION....16
1.1. Introduction....16
1.2. Standard Treatment for Advanced Hepatocellular Carcinoma....16
1.3. HCC with vascular invasion and its treatment....17
1.4. Immunotherapy....17
1.5. Carbon ion radiotherapy....18
1.6. Induction of Immunogenicity by Radiotherapy....18
1.7. Combination of immune checkpoint inhibitors and carbon ion radiotherapy....19
1.8. Investigational drugs....19
1.8.1. Durvalumab....19
1.8.2. Tremelimumab....20
1.8.3. Durvalumab + tremelimumab combination therapy....20
1.8.4. Rationale for Durvalumab and Tremelimumab as Treatment Options for HCC 20
1.9. hypothesis....21
2. STUDTY OBJECTIVE....23
2.1. Objective....23
2.2. Study results regarding the appropriateness of conducting this clinical trial, efficacy, and safety for the subject disease....23
2.2.1. Durvalumab + tremelimumab combination therapy dose rationale....23
2.2.2. Dose rationale for combination regimen of durvalumab 1500 mg Q4W plus tremelimumab 300 mg × 1 dose....23
2.2.2.1. Rationale for utilizing a fixed-dose regimen for durvalumab and tremelimumab....24
2.2.3 Rationale for carbon-ion radiotherapy....24
2.2.4 Rationale for combined treatment of carbon-ion radiotherapy and immunotherapy....25
2.3. Benefit-risk and ethical assessment....25
2.3.1. Potential benefits....25
2.3.2. Overall risks....25
2.3.3. Overall benefit-risk....28
3. ELIGIBILITY....29
3.1. Diagnostic Criteria and Stage, Type, and Condition Classification....29
3.2. Inclusion criteria....29
3.3. Exclusion criteria....30
4. INFORMED CONSENT....33
4.1. Preparation and revision of informed consent form....33
4.2. Method of Obtaining Informed Consent....33
4.3. Information to be provided to subjects....34
5. STUDY DESIGN....35
5.1. Overview of study design....35
5.2. Target number of subjects and study duration....38
5.3. Monitoring for safety assessment....38
5.4. Institutional and case registration methods....39
5.4.1. Facility registration....39
5.4.2. Subject resistration....39
5.4.3. Handling of subjects who are found to be ineligible....40
5.5. Dosing schedule and dosage/administration method....40
5.5.1. Criterion for reduction....40
5.5.2. Criterion for drug withdrawal....41
5.6. Discontinuation of Investigational Drug....41
5.7. Discontinuation of individual cases from participation in clinical trials....41

5.7.1. In case of untraceable cases ... 42
5.7.2. Withdrawal of consent ... 42
5.7.3. Clinical investigator's decision ... 42
5.7.4. Subject weight loss ... 42
5.7.5. Other cases ... 42
5.8. Subject replacement ... 42
5.9. Concomitant Restricted Drugs and Concomitant Restricted Therapy ... 43
5.10. Follow-up treatment ... 44
5.11. After discontinuation of this clinical trial ... 44
6. CLINICAL TRIAL TREATMENT ... 45
6.1. Durvalumab and tremelimumab ... 45
6.1.1. Durvalumab ... 45
6.2. Control Drugs ... 46
6.3. Monitoring during administration ... 46
6.4. Management of investigational drugs ... 46
6.5. Disposal of unused investigational drugs ... 47
6.6. Packaging and labeling of investigational drugs ... 47
6.7. Carbon ion radiotherapy ... 47
6.8. subject inclusion ... 48
6.9. Definition of Dose-Limiting Toxicity (DLT) ... 49
6.10. Toxicity Management ... 51
6.11. Restrictions during the clinical trial ... 51
6.11.1. Restrictions during the clinical trial ... 51
6.12. Clinical Trial Procedures ... 52
7. OBSERVATION, EXAMINATION, AND ASSESSMENT, METHODS, AND TIMING OF IMPLEMENTATION ... 53
7.1. Implementation Schedule and Procedures ... 53
7.2. Observation, tests and assessment ... 56
7.2.1. Screening period ... 56
7.2.2. DLT assessment period ... 57
7.2.3. Durvalumab q4W dosing period ... 59
7.2.4. At the time of discontinuation of investigational drug administration ... 60
7.2.5. Follow up period ... 60
7.3. Biological sampling procedures ... 60
7.3.1. Guideline for blood sampling volume ... 60
7.3.2. Blood samples for archiving ... 61
7.3.3. Hepatic tumor biopsy sample ... 61
7.4. Asessment of efficiency ... 61
7.5. Assessment of safety ... 62
7.5.1. Clinical laboratory tests ... 62
7.5.2. Physical examinations ... 64
7.5.3. Electrocardiogram (ECG) ... 64
7.5.4. Vital signs ... 64
7.5.5. ECOG performance status ... 65
7.5.6. Other safety assesements ... 65
8. HANDLING OF ADVERSE EVENT ... 65
8.1. Definition ... 65
8.1.1. Adverse event ... 65
8.1.2. Severe adverse event ... 66
8.1.3. Adverse Events of Special Interest (AESI) ... 66
8.1.4. Confirmation of interstitial lung disease (ILD) ... 67
8.2. Assessment of severity ... 68
8.3. Record of adverse events and serious adverse events ... 68
8.4. Duration of recording and follow-up of adverse events and serious adverse events ... 69

8.5. Causal relationship with investigational therapy 69
8.6. Outcome definition 70
8.7. Treatment of investigational drug in the event of an adverse event 70
8.8. Treatment of heavy particle therapy equipment in the event of an adverse event 70
8.9. Relationship to Protocol Procedures 70
8.10. Adverse events based on signs and symptoms 71
8.11. Adverse events based on tests and examinations 71
8.12. Hy's Law 71
8.13. Disease progression 71
8.14. New cancer 71
8.15. Deaths 71
8.16. Reportable Adverse Events 72
8.17. Reporting of serious adverse events 72
8.17.1. Response to subjects 73
8.18. OTHER EVENTS REQUIRING REPORTING 73
8.18.1. Overdose 74
8.18.2. Hepatic function abnormality 74
8.18.3. Pregnancy 74
8.18.4. Exposure to pregnant woman 74
8.18.5. Exposure to partner 74
8.19. Medication error 75
8.20. Predicted Side Effects 75
9. ENDPOINT 78
9.1. Primary endpoint 78
9.2. Secondary endpoint 78
9.3. Exploratory endpoints 78
10. STATISTICAL METHODS AND SAMPLE SIZE DETERMINATION 78
10.1. Description of Analysis set 78
10.1.1. DLT analysis set 78
10.1.2. Safety analysis set: SAF 78
10.1.3. Full Analysis set: FAS 78
10.1.4 Efficacy Evaluable set: EES 78
10.1.5 Per protocol set ： PPS 79
10.2. Target number of cases and rationale for setting 79
10.3. Case Handling 79
10.5.1. Analysis of subject background 79
10.5.2. Analysis of primary endpoints 79
10.5.2.1. DLT Evaluation 79
10.5.2.2. Safety Evaluation 80
11. COMPLIANCE AND DEVIATION FROM THE PROTOCOL 81
12. CHANGES TO THE CLINICAL TRIAL PROTOCOL, CASE REPORT FORM, OR ANALYSIS PLAN 81
13. DISCONTINUATION, SUSPENSION, OR TERMINATION OF THE CLINICAL TRIAL 82
14. DATA MANAGEMENT 83
15. RETENTION OF SOURCE DOCUMENTS AND OTHER RECORDS 83
16. RETENTION OF SAMPLES AND USE OF SAMPLES PROVIDED BY OTHER INSTITUTIONS 84
17. SOURCE DOCUMENT VERIFICATION 84
18. QUALITY ASSURANCE 84
19. QUALITY CONTROL FOR THIS STUDY 85
20. ETHICAL CONDUCT AND GOOD CLINICAL PRACTICE(GCP) 85
21. INSTITUTIONAL REVIEW BOARD(IRB) 85
22. HEALTH DAMAGE COVERAGE AND INSURANCE 86

23. COST BURDEN FOR THIS TRIAL 86
24. TRIAL FUNDS AND CONFLICT OF INTEREST 86
25. PROVISION OF THE INVESTIGATIONAL PRODUCTS AND INTELLECTUAL PROPERTY RIGHTS 86
26.PUBLICATION 86
27. REGISTRATION FOR CLINICAL TRIAL 86
28. STUDY IMPLEMANTATION GRPUP 87
29. LIST OF INVESTIGATIONAL PRODUCT(S) FOR THIS STUDY 87
30. LIST OF REFERENCES 87

0. Clinical Trial Overview

<table>
<tr><td>Title</td><td>A phase Ib study of durvalumab (MEDI4736) ± tremelimumab combined with particle therapy in advanced hepatocellular carcinoma patients with macrovascular invasion</td></tr>
<tr><td>Objective</td><td>

Objectives:

Primary Objective:

To assess the safety and tolerability of combination therapy of durvalumab ± tremelimumab with particle therapy in advanced hepatocellular carcinoma patients with macrovascular invasion

Secondary Objectives:

To assess the efficacy of combination therapy of durvalumab ± tremelimumab with particle therapy in advanced hepatocellular carcinoma patients with macrovascular invasion

</td></tr>
<tr><td>Design</td><td>

Ib Phase, interventional, open-label, single arm

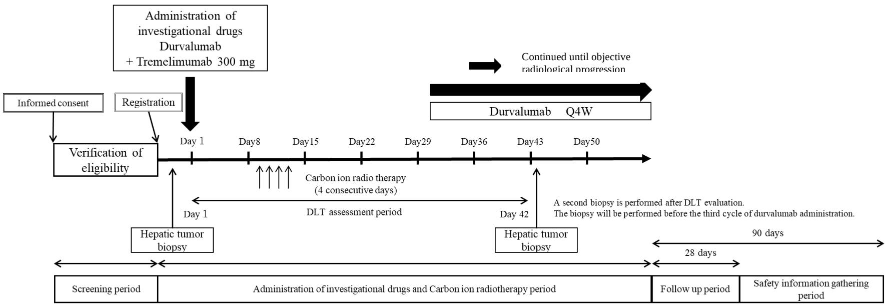

</td></tr>
<tr><td>Phase</td><td>Ib Phase</td></tr>
<tr><td>Investigational Products and Combination Therapy</td><td>

Investigational Products and Combination Therapy:

Investigational Products: Durvalumab and Tremelimumab

Durvalumab (MEDI4736) solution for infusion after dilution will be supplied in glass vials containing 500 mg durvalumab at a concentration of 50 mg/mL.

Tremelimumab solution for infusion after dilution will be supplied in glass vials containing 400 mg or 25 mg tremelimumab at a concentration of 20 mg/mL.

Combination Therapy

Carbon-Ion Radiation Therapy (CIRT) as particle therapy by Toshiba, Carbon ion radio therapy CI-1000S (PMDA approval no. 22800BZX00096000).

</td></tr>
<tr><td>Inclusion Criteria</td><td>

1) Capable of giving signed informed consent which includes compliance with the requirements and restrictions listed in the informed consent form (ICF) and in this protocol. Written informed consent and any locally required authorization obtained from the patient/legal representative prior to performing any protocol-related procedures, including screening

</td></tr>
</table>

evaluations. For patients aged <20 years and enrolling, a written informed consent should be obtained from the patient and his or her legally acceptable representative.

2) Age ≥20 years at time of study entry
3) Eastern Cooperative Oncology Group (ECOG) performance status of 0 or 1
4) Body weight >30 kg
5) Adequate normal organ and marrow function as defined below:
   - Haemoglobin ≧9.0 g/dL
   - Absolute neutrophil count (ANC) ≧1,500/mm^3
   - Platelet count ≧75×10^9/L （≧75,000/mm^3）
   - Serum bilirubin ≦ULN×3.0
   - AST ≦ULN×5.0
   - ALT ≦ULN×5.0
   - Measured creatinine clearance (CL) >40 mL/min or Calculated creatinine clearance CL>40 mL/min by the Cockcroft-Gault formula (Cockcroft and Gault 1976) or by 24-hour urine collection for determination of creatinine clearance
6) Evidence of post-menopausal status or negative urinary or serum pregnancy test for female pre-menopausal patients. Women will be considered post-menopausal if they have been amenorrheic for 12 months without an alternative medical cause. The following age-specific requirements apply:
   - Women <50 years of age would be considered post-menopausal if they have been amenorrheic for 12 months or more following cessation of exogenous hormonal treatments and if they have luteinizing hormone and follicle-stimulating hormone levels in the post-menopausal range for the institution or underwent surgical sterilization (bilateral oophorectomy or hysterectomy).

   - Women ≥50 years of age would be considered post-menopausal if they have been amenorrheic for 12 months or more following cessation of all exogenous hormonal treatments, had radiation-induced menopause with last menses >1 year ago, had chemotherapy-induced menopause with last menses >1 year ago, or underwent surgical sterilization (bilateral oophorectomy, bilateral salpingectomy or hysterectomy).

7) Patient is willing and able to comply with the protocol for the duration of the study including undergoing treatment and scheduled visits and examinations including follow up.
8) Advanced HCC confirmed histologically or by the typical findings of a hypervascular tumor on computed tomography or angiography
9) (Cohort A and Cohort B only) Patients who have received at least one prior systemic chemotherapy regimen including atezolizumab bevacizumab combination, sorafenib, or lenvatinib and who are determined to be refractory or intolerant to standard therapy.
10) Must not be eligible for locoregional therapy for unresectable HCC. For patients who progressed after locoregional therapy for HCC, locoregional therapy must have been completed ≥28 days prior to the baseline scan for the current study. Acceptable locoregional therapy for HCC are Ethanol Infusion Therapy, Radio Wave ablation Therapy, Transcatheter Arterial chemoembolization (TACE), Transcatheter arterial infusion (TAI). Hepatic Arterial Infusion Chemotherapy (HAIC) is not treated as a locoregional therapy.

	11) Patients who have been diagnosed with HCC showing MVI. MVI is defined as a tumor thrombus in the major hepatic and/or portal vein branches (Vp2, Vp3, Vp4, Vv2, and Vv3) identified by imaging studies.<br>12) Child-Pugh A<br>13) At least one measurable lesion other than the MVI and feeding nodule based on mRECIST.
Exclusion Criteria	1. Involvement in the planning and/or conduct of the study (applies to both sponser and/or staff at the study site)<br>2. Patients who have participated in another clinical trial using the investigational drug within 28 days prior to obtaining consent or who have received another investigational drug within 28 days prior to the first dose of the investigational drug in this study. The exception is if the patient is in the follow-up period of an interventional trial or is participating in an observational (non-interventional) clinical trial.<br>3. Any unresolved toxicity NCI CTCAE Grade ≥2 from previous anticancer therapy with the exception of alopecia, vitiligo, and the laboratory values defined in the inclusion criteria<br>− Patients with Grade ≥2 neuropathy will be evaluated on a case-by-case basis after consultation with the Study Physician.<br>− Patients with irreversible toxicity not reasonably expected to be exacerbated by treatment with durvalumab or tremelimumab may be included only after consultation with the Study Physician.<br>4. Radiotherapy treatment to more than 30% of the bone marrow or with a wide field of radiation within 4 weeks of the first dose of study drug<br>5. Major surgical procedure (as defined by the Investigator) within 28 days prior to the first dose of IP. Note: Local surgery of isolated lesions for palliative intent is acceptable.<br>6. History of allogenic organ transplantation.<br>7. Active or prior documented autoimmune or inflammatory disorders (including inflammatory bowel disease [e.g., colitis or Crohn's disease], diverticulitis [with the exception of diverticulosis], systemic lupus erythematosus, Sarcoidosis syndrome, or Wegener syndrome [granulomatosis with polyangiitis, Graves' disease, rheumatoid arthritis, hypophysitis, uveitis, etc.]). The following are exceptions to this criterion:<br>– Patients with vitiligo or alopecia<br>– Patients with hypothyroidism (e.g., following Hashimoto syndrome) stable on hormone replacement<br>– Any chronic skin condition that does not require systemic therapy<br>– Patients without active disease in the last 5 years may be included but only after consultation with the study physician<br>– Patients with celiac disease controlled by diet alone<br>8. Uncontrolled intercurrent illness, including but not limited to, ongoing or active infection, symptomatic congestive heart failure, uncontrolled hypertension, unstable angina pectoris, cardiac arrhythmia, interstitial lung disease, serious chronic gastrointestinal conditions associated with diarrhea, or psychiatric illness/social situations that would limit compliance with study requirement, substantially increase risk of incurring AEs or compromise the ability of the patient to give written informed consent<br>9. History of another primary malignancy except for<br>– Malignancy treated with curative intent and with no known active disease ≥5 years before the first dose of IP and of low potential risk for recurrence

	– Adequately treated non-melanoma skin cancer or lentigo maligna without evidence of disease<br>– Adequately treated carcinoma in situ without evidence of disease<br>However, the following cases are eligible for enrollment<br>Early stage cancer (epithelial cancer of the cervix, basal cell carcinoma, superficial bladder cancer (Tis and T1), early stage gastric cancer, and early stage colorectal cancer) that has been treated for curative purposes, has not been confirmed active for at least 3 years prior to inclusion in the study, and has a low risk of recurrence.<br>10. History of leptomeningeal carcinomatosis<br>11. History of, or current, brain metastases or spinal cord compression. Patients with suspected brain metastases at screening should have an MRI (preferred) or CT, each preferably with IV contrast of the brain prior to study entry.<br>12. Mean QT interval corrected for heart rate using Fridericia's formula (QTcF) ≥470 ms calculated from 3 ECGs (within 15 minutes at 5 minutes apart) Regardless of whether this criteria stays or not, all patients should have a baseline ECG<br>13. History of active primary immunodeficiency<br>14. Patients co-infected with HBV and HCV. HBV positive (presence of HBsAg and/or anti-HBcAb with detectable HBV DNA); HCV positive (presence of anti-HCV antibodies), and active infection including tuberculosis (clinical evaluation that includes clinical history, physical examination and radiographic findings, and TB testing in line with local practice).<br>15. Current or prior use of immunosuppressive medication within 14 days before the first dose of durvalumab or tremelimumab. The following are exceptions to this criterion:<br>– Intranasal, inhaled, topical steroids, or local steroid injections (e.g., intra articular injection)<br>– Systemic corticosteroids at physiologic doses not to exceed 10 mg/day of prednisone or its equivalent<br>– Steroids as premedication for hypersensitivity reactions (e.g., CT scan premedication)<br>16. Receipt of live attenuated vaccine within 30 days prior to the first dose of IP. Note: Patients, if enrolled, should not receive live vaccine whilst receiving IP and up to 30 days after the last dose of IP.<br>17. Female patients who are pregnant or breastfeeding or male or female patients of reproductive potential who are not willing to employ effective birth control from screening to 90 days after the last dose of durvalumab monotherapy or180 days after the last dose of durvalumab + tremelimumab combination therapy.<br>18. Known allergy or hypersensitivity to any of the study drugs or any of the study drug excipients.<br>19. Prior randomisation or treatment in a previous durvalumab and/or tremelimumab clinical study regardless of treatment arm assignment.<br>20. Judgment by the investigator that the patient is unsuitable to participate in the study and the patient is unlikely to comply with study procedures, restrictions and requirements.<br>21. Patients who have been treated with anti-PD-1, anti-PD-L1 inhibitors, or other drugs that act on other stimulatory or co-suppressive T-cell receptors and their combinations (including atezolizumab plus bevacizumab) and

	have failed to tolerate the same treatment.<br>22. Prior radiotherapy involving the liver.<br>23. Renal failure requiring hemodialysis or peritoneal dialysis<br>24. Any of the following cardiac diseases:<br>– NYHA Class III or IV chronic heart failure<br>– Current coronary artery disease or history of ischemic heart disease such as myocardial infarction within 6 months before the study<br>– Serious arrhythmia (grade 3 or higher according to the CTCAE ver. 5.0: arrhythmia that cannot be controlled by oral medications or requires mechanical control).<br>25. Poorly controlled hypertension<br>26. Serious and active infection, excluding hepatitis viral infection<br>27. Persistent proteinuria of NCI-CTCAE version 5.0 grade 3 or higher.<br>28. Arterial or venous thrombotic or embolic events such as cerebrovascular accident, deep vein thrombosis, or pulmonary embolism within 6 months before the start of study medication.<br>29. Refractory pleural effusion or ascites<br>30. History of hepatic encephalopathy within past 12 months<br>31. Oral intake impossible<br>32. HIV-positive<br>33. Pulmonary fibrosis or interstitial pneumonitis<br>34. Other serious complications as follows: serious mental disease or history of gastrointestinal bleeding or active hemoptysis<br>35. Unsatisfactory general condition for participation in the study as judged by the primary physician
Endoints	**Primary Endpoints:**<br>AEs/SAEs including DLTs<br><br>**Secondary Endpoints:**<br>Overall Survival (OS), Survival Rate at 6 months, Objective Response Rate (ORR), Time to Progression (TTP), Progression Free Survival (PFS) at 6 months (in accordance with mRECIST)_
Dosage and treatment	Each cycle of this study will last for 28 days, and the DLT evaluation period will be 42 days from the start of study drug administration.<br>Durvalumab 1,500 mg alone or in combination with 300 mg tremelimumab IV on Cycle 1, Day 1.<br>Carbon ion radio theray (60 Gy (RBE) / 4 times / 4 days) is administered between days 8-15 of the first cycle.<br>After the DLT evaluation period, durvalumab is continued every 4 weeks until tumor progression is confirmed.
Criteria for discontinuation of individual cases	1) In case it is judged difficult to continue the clinical trial for some reason on the subject's side, such as non-attendance or transfer to a different hospital.<br>2) In case the subject requests to discontinue participation in the clinical trial<br>3) In case the investigator or subinvestigator determines that the subject is unable to continue the clinical trial.<br>4) In case the subject's body weight becomes less than 30 kg.<br>5) In case the investigator/participating investigator determines that a decision to discontinue.

Number of Patients Planned:	**Number of Patients Planned:**<br>15 subjects.<br><br>The number of patients enrolled for DLT assessment in either Cohort A or Cohort B may vary from 3 to 6 depending on the frequency of DLT. If both of Cohort A and B regimens were confirmed tolerable after DLT assessment, additional patients will be enrolled in Cohort B up to a total of 15 subjects. If only Cohort A regimen was determined to be tolerable, additional patients may be enrolled in Cohort A up to a total of 15 subjects.<br><br>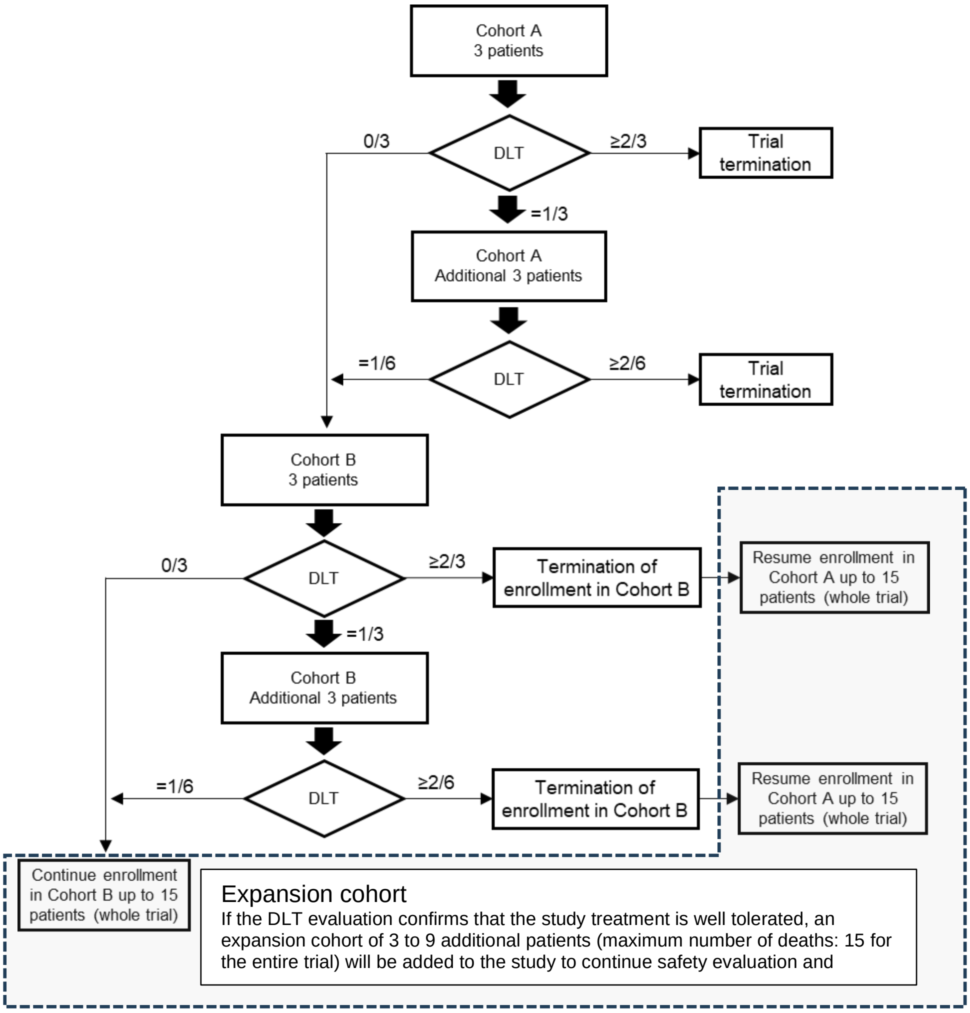
Clinical trial period	Estimated study start date 30 Apr 2021<br>Estimated study completion date 30 Sep 2023
Subject registration period	Estimated date of first patient enrolled 30 Apr 2021<br>Estimated date of last patient enrolled 31 Mar 2023
Clnical trial sites	2Sites<br>Enrollment of patients in the trial and administration of IPs will be conducted at Chiba University Hospital, and CIRT will be administered at QST Hospital.

ETHICAL AND REGULATORY REQUIREMENTS	The study will be performed in accordance with ethical principles that have their origin in the Declaration of Helsinki and are consistent with Good Clinical Practice, and applicable regulatory requirements Patient data protection.
Institutional Review Board (IRB)	Prior to the start of the study, the Institutional Review Board (IRB) will evaluate the ethical, scientific and medical relevance of this study. The study will be conducted after obtaining approval from the IRB. If the evaluation results of the IRB are “approved with modification”, the study will be conducted after the protocol, case report, or consent forms are modified based on the review results. At the frequency of more than once a year, the IRB will continuously review whether the study is being performed appropriately.

1. INTRODUCTION

1.1. Introduction

Primary liver cancer is broadly classified into hepatocellular carcinoma (HCC) and intrahepatic cholangiocarcinoma, of which HCC accounts for more than 90% in Japan. According to a recent survey report, the number of patients with hepatocellular carcinoma in Japan is approximately 47,000 [1], and the number of deaths is approximately 27,000 per year [2].

The development of HCC is strongly associated with chronic hepatitis and cirrhosis caused mainly by hepatitis B virus (HBV) or hepatitis C virus (HCV) infection, and carriers of these viruses are considered to be at high risk of developing HCC ([3] El Serag HB, et al. NEJM. 2011). Risk factors other than viral hepatitis include cirrhosis, male age, older age, alcohol consumption, smoking, obesity, fatty liver, and diabetes mellitus [4]. Many patients are diagnosed after the disease has reached an advanced stage. The choice of treatment depends on the stage of the disease and the patient's condition. Since most patients with HCC also have chronic hepatitis or cirrhosis, individual assessment of liver function is essential to patient selection. Although several agents have been shown to be effective in the treatment of unresectable advanced HCC, there are still limited options and further therapeutic development is needed.

1.2. Standard Treatment for Advanced Hepatocellular Carcinoma

Sorafenib demonstrated a survival advantage over placebo in unresectable advanced HCC in the Sorafenib Hepatocellular Carcinoma Assessment Randomized Protocol (SHARP) trial in 2008, followed by the Asia-Pacific trial in 2009 ([5] Llovet JM, et al. Asia-Pacific trial also demonstrated a survival benefit ([5] Llovet JM, et al. NEJM. 2008, [6] Cheng AL, et al Lancet Oncol. 2009). In the SHARP trial, median survival (OS) was 10.7 months in the sorafenib group versus 7.9 months in the placebo group, with a hazard ratio (HR) of 0.69 (95% confidence interval (CI): 0.55, 0.87), P < 0.001. months vs. 4.2 months, HR 0.68 (95% CI: 0.50, 0.93), P = 0.014. The median progression-free survival in the SHARP trial was 5.5 months in the sorafenib group versus 2.8 months in the placebo group, HR 0.58 (95% CI: 0.5, 0.7), and in the Asia-Pacific trial the median progression-free survival was 2.8 months versus 1.4 months, HR 0.6 (95% CI: 0.4, 0.8).

Regorafenib after Sorafenib in Child-Pugh classification A patients with image progression after sorafenib treatment and who tolerated sorafenib (400 mg or more for at least 20 days in the 28 days before the end of treatment) compared regorafenib to placebo Patients with Hepatocellular Carcinoma (RESORCE) trial, regorafenib showed for the first time a survival advantage as second-line therapy after sorafenib treatment ([7] Bruix J, et al. Lancet. 2017). In that study, the median OS was 10.6 months (95% CI: 9.1, 12.1) in the regorafenib group and 7.8 months (95% CI: 6.3, 8.8) in the placebo group, HR 0-63 (95% CI: 0.50, 0.79) P < 0-0001. 2017, lenvatinib was non-inferiority to sorafenib for the primary endpoint of survival, making it the first-line treatment of choice for advanced hepatocellular carcinoma ([8] Kudo M, et al. Lancet. 2018). In that study, the median OS was 13.6 months (95%CI: 12.1, 14.9) in the lenvatinib arm and 12.3 months (95%CI: 10.4, 13.9) in the sorafenib arm with a HR of 0.92 (95%CI : 0.79, 1.06). In addition, ramucirumab became available in June 2019 for unresectable HCC with serum AFP levels of 400 ng/mL or higher, exacerbated after cancer chemotherapy ([9]Zhu AX, et al. Lancet Oncol. 2019).

Combination therapy with atezolizumab, an anti-PD-L1 humanized monoclonal antibody, and bevacizumab, an anti-VEGF monoclonal antibody, was shown to significantly prolong OS versus sorafenib in patients with Child-Pugh classification A who had not received systemic therapy (IMbrave 150 study) ([7 Bomze D et al. NEJM. 2020). In that study, 12-month survival rates were reported as 67.2% (95% CI: 61.3, 73.1) for atezolizumab plus bevacizumab and 54.6% (95% CI: 45.2, 64.0) for sorafenib, with an HR for OS of 0.58 (95% CI: 0.42, 0.79), P < 0 .001. Based on these results, the combination of atezolizumab and bevacizumab became available in Japan in September 2020. Based on these results, the combination of atezolizumab and

bevacizumab is now the first-line treatment for advanced hepatocellular carcinoma. Other first-line treatment options include sorafenib or lenvatinib, and second-line treatment options include regorafenib, ramucirumab, and cabozantinib. However, the survival gains obtained have been limited and do not fully meet the medical needs of patients.

1.3. HCC with vascular invasion and its treatment

HCC with vascular invasion (MVI) extending into the portal and/or hepatic veins has a poorer prognosis compared to cases without MVI ([11]Costentin CE, et al. Liver Cancer 2017). Although systemic chemotherapy is the standard of care, several studies have suggested the efficacy of alternative treatment approaches (e.g., resection, hepatic arterial chemoembolization, intravenous chemotherapy, and radiation therapy) after determining the benefit for each patient, and are recommended in Japanese guidelines. MVI Although it is empirically known that controlling MVI dramatically improves the prognosis of advanced HCC with MVI, sufficient evidence does not exist.

1.4. Immunotherapy

In recent years, research on cancer immunity has advanced dramatically, and the mechanisms by which the immune system controls or eliminates tumors are becoming clearer ([12] Dunn, et al. Annu Rev Immunol. 2004). PD-L1 is part of a complex system of receptors and ligands involved in the regulation of T cell activation. The PD-1 receptor (CD279) is expressed on the surface of activated T cells ([13] Keir ME, et al. Rev Immunol. 2008). It has two known ligands: PD-L1 (B7 H1; CD274) and PD-L2 (B7 DC; CD273) ([14] Okazaki and Honjo 2007). PD-1 and PD-L1 / PD-L2 act as co-inhibitors that can arrest or limit T cell responses When PD-L1 binds to PD-1, inhibitory signals are transmitted to T cells, cytokine production is reduced, and T cell proliferation is inhibited. Tumor cells have been shown to utilize this immune checkpoint pathway as a mechanism to evade detection and inhibit immune responses.

PD-L1 has been found to be expressed in a wide range of carcinomas, and anti-PD-L1 antibodies can be used therapeutically to augment anti-tumor immune responses in cancer patients.Results from preclinical and clinical studies of monoclonal antibodies (mAbs) targeting the PD-L1 / PD-1 pathway provide evidence of clinical activity and manageable Anti-PD-L1 antibodies can be used to augment anti-tumor immune responses in cancer patients for therapeutic purposes, showing evidence of clinical activity and manageable safety profiles ([17] Brahmer JR, et al. N Engl J Med. 2012, [18] Hirano F, et al. Cancer Res. 2005, [19] Iwai Y, et al. Proc Natl Acad Sci USA. 2002,[20] Okudaira K, et al. Int J Oncol. 2009,[21] Topalian SL, et al. N Engl J Med. 2012,[22] Zhang C,et al. Cytotherapy. 2008), The hypothesis that its efficacy is higher in patients with tumors expressing PD-L1 ([23] Powles T, et al. Nature. 2014; [24] Rizvi N, et al. J Clin Oncol. 2015; [25] Segal NH, et al. J Clin Oncol. 2015) is now supported The hypothesis of a “bladder cancer” (e.g., [24] Rizvi N et al. The high frequency and number of mutations in bladder cancer ([26] Alexandrov et al. Nature. 2013), for example, may contribute to the responses seen with immunotherapy.

In contrast, cytotoxic T lymphocyte-associated antigen 4 (CTLA-4) is structurally expressed on regulatory T cells and is enhanced on the surface of activated T cells; CTLA-4 sends negative regulatory signals to T cells when it binds to CD80 (B7.1) or CD86 (B7.2) ligands on antigen-presenting cells ([27] (Fife BT, Bluestone JA. Immnol Rev. 2008). In animal models, blockade of CTLA-4 binding to CD80 / 86 by anti-CTLA-4 antibodies has also been shown to markedly enhance T cell activation and antitumor activity, as exemplified by the killing of established solid tumors in mice and induction of protective antitumor immunity. Therefore, treatment with anti-CTLA-4 antibodies is expected to enhance activation of the human immune system and anti-tumor activity in patients with solid tumors.

Preclinical data have been added along with abundant clinical data, indicating that inhibition of negative regulatory signals to T cells, such as cytotoxic T lymphocyte antigen 4 (CTLA-4) and PD-L1, is a promising approach with promising clinical results. Ipilimumab has received U.S. Food and Drug Administration (FDA) approval for the treatment of metastatic melanoma,

and nivolumab and pembrolizumab (two anti-PD-1 agents) and atezolizumab (an anti-PD- L1 agent) are currently approved by agencies such as the U.S. Food and Drug Administration and the European Medicines Agency for the treatment of metastatic melanoma, squamous cell and non-squamous cell non-small cell lung cancer, urothelial carcinoma, and other malignancies. In addition, data from agents in the anti-PD-1 / PD-L1 class have shown clinical activity against a wide range of carcinomas.

In HCC as in other cancer types, two clinical trials (nivolumab and pembrolizumab) using ICI showed that 17-20% of patients achieved an objective response and most of the responders had a durable response ([28]El-Khoueiry AB, et al . Lancet 2017, [29]Zhu AX, et al. Lancet Oncol. 2018). The combination of ICI with tyrosine kinase inhibitors or anti-VEGF monoclonal antibodies has shown promise with the potential to achieve extremely high sustained response rates in a variety of cancer types, including HCC ([30]Kudo M. World J Gastroenterol 2019). Recently, the IMbrave 150 trial showed that atezolizumab plus bevacizumab therapy extended both OS and PFS compared with sorafenib, positioning atezolizumab plus bevacizumab as the first-line treatment for advanced hepatocellular carcinoma. Immunotherapy is also playing an increasingly important role in advanced HCC, and a phase I study of durvalumab, an anti-PD-L1 antibody, and tremelimumab, an anti-cytotoxic T lymphocyte-associated antigen 4 (CTLA-4) monoclonal antibody, in patients with HCC demonstrated a response rate of 25% ([31 was demonstrated ([31] Kelly RK et al. ASCO2017). An expanded portion of a Phase II trial is underway. A Phase III trial evaluating the efficacy and safety of the combination of durvalumab and tremelimumab, as well as monotherapy with sorafenib and durvalumab (HIMALAYA trial) are also ongoing and awaiting results.

1.5. Carbon ion radiotherapy

Particle therapy, especially carbon ion radio therapy, has advantages in dose concentration and biological effects over photon therapy, such as x-rays and gamma rays, which are usually used in radiation therapy.

X-rays and gamma rays are most intense near the body's surface and penetrate the body with decreasing intensity as they travel deeper. For this reason, the most powerful way to treat a specific area with X-rays or gamma rays is to focus the radiation from many directions, concentrating the high-dose area on the lesion. However, for tumors that are close to vital organs and spread malformedly, it is difficult to avoid surrounding normal organs. In contrast, heavy particle beams, which become intense at a certain depth depending on their energy, but are weak before and after that point, can be easily focused by aligning the peak with the tumor ([32] Pedroni E, et al. Med Phys. 1995).

The principle of action of radiotherapy is the double-strand break of DNA by ionizing radiation. Carbon ion radio therapy is known to cause dense ionization. Therefore, DNA double-strand breaks can be caused more efficiently and more densely than with X-rays. This is the reason for the favorable biological effects of heavy particle beams. This, combined with the good dose distribution resulting from the physical characteristics of heavy-ion beams, enables them to efficiently exert their anti-tumor effects. In Japan, the National Institute of Radiological Sciences (now renamed the National Institute of Quantum Science and Technology) started heavy particle therapy in 1994 and has treated more than 10,000 patients with good results ([33] Mohamad O., et al. Cancer (Basel), 2018).

In HCC, high local control rates have also been shown with particle therapy ([34] Kasuya G, et al. Cancer. 2017, [35] Fukuda, K, et al. Cancer Sci. 2017, [36] Sorin Y, et al. Liver Cancer 2018, [37] (Igaki H, et al. Int J Clin Oncol. 2018). Furthermore, good outcomes have been shown for hepatocellular carcinoma with vascular invasion. ([38] Komatsu S, et al. J Gastroenterol. 2011, [39] Lee SU, et al. Strahlenther Onkol. 2014, [40] Sugahara S, et al. Strahlenther Onkol. 2009).

1.6. Induction of Immunogenicity by Radiotherapy

Radiation therapy is known to stimulate immunogenicity through multiple mechanisms. The major immunological effects of irradiation include increased antigen presentation through

elevated expression of major histocompatibility gene complex class I, induction of apoptosis through elevated membrane expression of Fas ligands, calreticulin expression and high mobility group box-1 (HMGB1) and other (HMGB1), and induction of phagocytosis and immunity through the release of damage-associated molecular patterns (DAMPs).

Clinically, the PACIFIC trial showed that the combination of durvalumab and chemoradiotherapy can provide high therapeutic efficacy with intolerable toxicity ([41]Antonia Sj, et al. NEJM. 2017). Despite the fact that this study did not include only PD-L1-high expressing patients, it showed significant improvements in both PFS and OS compared to existing therapy. On the other hand, in the combination of durvalumab and tremelimumab (MYSTIC trial), there was no improvement from existing therapy, even when only PD-L1 high-expressing patients were targeted ([42 Rizvi NA, et al. Annals of Oncol. 2018). The difference between these two trials was the presence or absence of radiotherapy, and a subset analysis of the PACIFIC trial showed better results in patients with a shorter time between completion of radiotherapy and durvalumab administration, suggesting that radiotherapy plays an important role in tumor immunity Radiotherapy plays an important role in tumor immunity.

1.7. Combination of immune checkpoint inhibitors and carbon ion radiotherapy

Heavy ion therapy is known to enhance both local immunostimulation and immunosuppression more strongly than conventional photon beam therapy in both animal models and human clinical specimens ([43] Helm A, et al. Int J Part Ther. 2018, [44] Takahashi Y, et al. Oncotarget. 2019, [45] Iijima M, et al. J Gynecol Oncol. 2020).

Irradiated tumor cells present HMGB1, a key factor among damage-related molecules, alongside numerous others ([46] Golden EB, et al. Front Oncol, 2012). HMGB1 functions as an immune-activating cytokine and is a key factor for toll-like receptor 4 (TLR4) and activates dendritic cells.

It has been suggested that HMGB1 is strongly induced by heavy particle therapy ([47] Onishi M, et al. J Radiat Res. 2018). A study in mice showed stronger immune activation when heavy ion therapy was combined with dendritic cell infusion. The combination of heavy particle therapy and immunotherapy has also been shown to enhance anti-tumor immunity and reduce metastases compared to x-ray therapy, immunotherapy alone, or a combination of both ([48] Matsunaga A, et al. Cancer. 2010, [49] Ando K, et al. J Radiat Res. 2017).

On the other hand, immunosuppressive molecules such as PD-L1 have also been found to be induced more strongly than with X-ray therapy ([45] Iijima et al. J Gynecol Oncol. 2020). Enhanced PD-L1 expression by irradiation has been reported via the AMT/AT/Chk1 pathway induced by DNA double-strand breaks ([50] Sato N, et al. Nat Commun. 2017). Heavy ion therapy is known to efficiently generate complex DNA double-strand breaks ([51] Oike T, et al. Sci Rep. 2016), supporting this phenomenon.

Clinical trials investigating the combination of immune checkpoint inhibitors with radiation in a number of carcinomas, including HCC, are ongoing. However, most of them are limited to combinations with conventional radiation therapy using X-rays, and only a few with particle therapy. Currently, there are no clinical trials testing the combination of heavy ion beams and immunotherapy, and this clinical trial is a pilot case. From basic research and clinical studies using X-rays, the combination of heavy ion therapy and immune checkpoint inhibitors is expected to have a greater synergistic effect.

1.8. Investigational drugs

1.8.1. Durvalumab

Durvalumab is a human immunoglobulin (Ig) subclass Grade 1κ (IgGrade 1κ) mAb that inhibits the interaction of PD-L1 with PD-1 on T cells and CD80 on immune cells but not with PD-L2. Durvalumab is being developed by AstraZeneca/MedImmune for the treatment of cancer.

The mechanism of action of durvalumab is said to be inhibition of the interaction of PD-L1 with PD-1 and CD80, which disrupts the suppression of the immune response and immune

response to tumor elimination in in vitro studies, durvalumab was found to antagonize PD-L1-mediated suppression on primary human T cells, restore T cell proliferation, and release interferon gamma (IFN-γ) ([52] Stewart R, et al. Cancer Immunol Res. 2015) In vivo studies showed that durvalumab inhibited tumor growth in a xenograft model through a T cell-dependent mechanism ([52] Stewart R, et al. Cancer Immunol Res. 2015). Based on these data, durvalumab is expected to stimulate anti-tumor immune responses in patients by binding to PD-L1 and shifting the balance toward anti-tumor immune responses. Durvalumab is designed to reduce antibody-dependent and complement-dependent cellular injury.

To date, more than 8,000 patients have received durvalumab as a single agent or in combination with other anticancer agents in studies sponsored by AstraZeneca throughout the clinical development program. Please refer to the most recent Durvalumab investigational brochure for nonclinical and clinical information, including safety, efficacy, and pharmacokinetics.

1.8.2. Tremelimumab

Tremelimumab is a human immunoglobulin (Ig) Grade 2 mAb that targets CTLA-4 (CTLA-4; cluster of differentiation [CD]152), a cell surface receptor that is primarily expressed on activated T cells and blocks their activation. Tremelimumab completely inhibits the interaction of human CTLA-4 with CD80 and CD86 and increases the release of cytokines (interleukin [IL]-2 and interferon [IFN]-γ) from human T cells, peripheral blood mononuclear cells and whole blood ([53] Tarhini and Kirkwood. Expert Opin Biol Ther. 2008). Tremelimumab is being developed by AstraZeneca for the treatment of cancer.

To date, it has been administered to more than 1,500 patients as a single agent or in combination with other anticancer agents. For a summary of nonclinical and clinical information, including safety, efficacy, and pharmacokinetics, please refer to the Tremelimumab investigational brochure.

1.8.3. Durvalumab + tremelimumab combination therapy

Since there is no overlap in the mechanisms of action of CTLA-4 and PD-1, targeting both PD-1 and CTLA-4 pathways may provide additive or synergistic effects ([16] Pardoll DM, et al. Nat Rev Cancer. 2012). Therefore, in addition to investigating both drugs as monotherapy for various cancer indications, AstraZeneca is also investigating the combination of durvalumab plus tremelimumab for the treatment of cancer.

Study D4190C00006 is a late phase I dose escalation study to establish the safety, pharmacokinetics/pharmacodynamics and preliminary antitumor activity of the combination of durvalumab plus tremelimumab in patients with advanced non-small cell lung cancer (NSCLC). The dosing schedule used in this study is durvalumab administered Q2W or Q4W until Month 12, followed by 7 doses of tremelimumab at Q4W until Week 24, then 2 additional doses every 12 weeks for up to 12 months. The study is ongoing and enrollment is ongoing. Other trials investigating combination therapy for NSCLC and other tumor indications have also been initiated.

To date, 3,000 patients have received the combination in various doses and dosing regimens throughout the clinical development program. For a complete summary of non-clinical and clinical trial information, including safety, pharmacokinetics, and efficacy, please refer to the most recent Durvalumab and Tremelimumab investigational brochure.

1.8.4. Rationale for Durvalumab and Tremelimumab as Treatment Options for HCC

The liver has multiple regulatory mechanisms to maintain an immunosuppressive environment. The normal liver is inherently prone to induce immune tolerance to prevent aberrant immunity to exposed pathogens ([54]Pardee AD and Butterfield LH.Oncoimmunology. 2012). Clinical and nonclinical data indicate that HCC increases the expression of immunosuppressive cell

populations such as regulatory T cells (Treg) and myeloid-derived suppressor cells, as well as suppressive signaling molecules including CTLA-4 and PD-1 ([54] ,[55] Gao O, et al. Clin Cancer Res. 2009, [56] Hato T, et al. Hepatology. 2014); HBV and HCV infection also increase Treg and PD-L1/PD-1 expression, suggesting that this pathway is involved in HBV and HCV-mediated hepatocellular carcinogenesis ([54],[57] Miroux C, et al. Expert Opin Biol Ther. 2010,[58] Golden-Mason L, et al. J Virol. 2007,[59]Peng G, et al. Mol Immunol. 2008).

It has also been shown that overexpression of PD-L1 results in higher malignancy, disease progression, and mortality in HCC ([54], [60] Klein Jp, et al. Stat Med. 2007). Therefore, suppression of PD-L51 and CTLA-4 function could reverse the immunosuppressive nature of HCC and promote host immunity against HCC and improve clinical outcome.

At this time, early promising clinical data suggest that anti-CTLA-4 and anti-PD-L1/PD-1 antibody agents are active against HCC and may help improve response rates and survival.

In 20 patients with HCV-associated HCC (43% Child-Pugh classification B) treated with 15 mg/kg of intravenous tremelimumab every 90 days ([61] Sangro B, et al. J Hepatol. 2013), tremelimumab was generally well tolerated and no patients received systemic steroids No patients received systemic steroids and there were no deaths related to the study drug. The majority of patients had transient increases in transaminases after the first dose, 45% of which were Grade 3 or higher, but not accompanied by a concurrent decline in liver function. 17 responses could be evaluated, with 3 (17.6%) patients achieving a definite partial response (PR).

In another phase I/II study, 32 patients with unresectable advanced HCC (Child-Pugh classification A/B7) received two dose levels of tremelimumab (3.5 and 10 mg/kg IV Q4W) with partial ablation (RFA/TACE) during a 6-week treatment period ([62] Duffy AG,. et al. J Hepatol. 2017). Safety assessment results showed no clear trend in the occurrence of adverse events throughout the entire dose-specific cohort of tremelimumab, and no dose-limiting toxicities (DLTs) were observed in the study. The major Grade 3 or 4 adverse events were increased AST (21%), increased ALT (9%), and hyperbilirubinemia (9%); no Grade 3 or 4 diarrhea, colitis, or pneumonitis occurred; of the 19 patients with evaluable response outside the area directly treated with TACE/RFA, 5 (26.3%) had confirmed PR. The safety and efficacy of durvalumab (anti-PD-L1 antibody) monotherapy were investigated in a phase I/II study (CDON-MEDI4736-1108) in 40 patients with HCC, with an objective response rate (ORR) of 10.3% and a median OS of 13.2 months. Detailed safety and efficacy data from the study are described in Section 1.2.2.1. Similar results were obtained with another anti-PD-1 antibody, with an ORR of 18.6% and a median OS of 13.2 months in patients with advanced HCC ([63] Melero I, et al. J Clin Oncol. 2017).

Although the data from these trials are exploratory, they suggest that monotherapy with both durvalumab and tremelimumab has an antitumor effect against HCC. The combination of durvalumab plus tremelimumab in patients with unresectable HCC is being investigated in an ongoing phase I/II study (D4190C00022). In that study, patients with unresectable HCC who had not been previously treated with immunotherapy received the combination of durvalumab plus tremelimumab four times in Q4W followed by durvalumab alone in Q4W. Interim data from 40 patients followed for more than 16 weeks in the study showed an ORR of 18%. The combination of durvalumab plus tremelimumab was generally well tolerated in the population of patients with unresectable HCC. phase II results presented at ASCO 2020 reported promising results for durvalumab 1,500 mg Q4W plus tremelimumab 300 mg once. ORR was 24% and median OS was 18.73 months (95% CI: 10.78-27.27). It was reported to have the most favorable risk-benefit profile compared to the other dose arms ([64]Kelly RK, et al. J Clin Oncol. 2020).

The combined clinical evidence suggests that both durvalumab and tremelimumab have clinical activity as monotherapy in patients with HCC, and that combination therapy with both agents may provide an even greater antitumor effect in this patient population. A phase III, randomized, global study is underway to evaluate the safety and efficacy of these two agents in patients with HCC.

1.9. hypothesis

The purpose of this study is to confirm the synergistic effect of durvalumab-tremelimumab in

combination with heavy ion therapy in patients with advanced HCC. The study was designed based on the following two hypotheses

- Activation of tumor immunity by heavy ion therapy will be enhanced by immune checkpoint inhibitors.
- The control of primary lesions with MVI in patients with advanced HCC will have clinically significant results.

2. STUDTY OBJECTIVE

2.1. Objective

A phase Ib, open-label, uncontrolled study to evaluate the safety and efficacy of durvalumab-tremelimumab in combination with heavy ion therapy in patients with advanced hepatocellular carcinoma with MVI to assess safety and tolerability as measured by frequency of adverse events including DLT. Efficacy will be evaluated based on overall survival, 6-month survival, objective response rate, 6-month progression-free survival, and progression-free interval.

2.2. Study results regarding the appropriateness of conducting this clinical trial, efficacy, and safety for the subject disease

2.2.1. Durvalumab + tremelimumab combination therapy dose rationale

The durvalumab + tremelimumab doses and regimen selected for this study are based on the goal of selecting an optimal combination dose of durvalumab and tremelimumab that would yield sustained target suppression (sPD-L1), demonstrate promising efficacy, and have an acceptable safety profile.

2.2.2. Dose rationale for combination regimen of durvalumab 1500 mg Q4W plus tremelimumab 300 mg × 1 dose

A summary of the existing PK and pharmacodynamic data has been utilized to guide theregimen selection for the combination of durvalumab 1500 mg plus single dose of tremelimumab 300 mg.

Pharmacokinetics/Pharmacodynamics data

The supporting data for this regimen are based on PK and pharmacodyamic data from regimens that used tremelimumab doses of greater than 1 mg/kg from Study D4190C00006. An approximate dose-proportional increases in PK exposure (maximum serum concentration and area under the serum drug concentration-time curve from time 0 to Day 28 post-dose) was observed with increasing doses of tremelimumab (1, 3, and 10 mg/kg). An exploratory pharmacodynamic analysis bioanalytically evaluated the effects of tremelimumab on proliferating T-cells from NSCLC patients who received tremelimumab (1, 3, or 10 mg/kg) and durvalumab (15 or 20 mg/kg) combination treatment. Monotonic increases in pharmacodynamic activity with the combination (increased activation/ proliferation markers on CD4 and CD8 T-cells in periphery) were observed with increasing doses of tremelimumab (1, 3, 10 mg/kg). The peak increase (%) from baseline of CD4+Ki67+ T-cells was observed 8 days post administration, and the peak level was significantly increased ($p \leq 0.05$) as increasing dose of tremelimumab in the range of 1 to 10 mg/kg. Study data also suggested that higher peak exposure (maximum serum concentration [Cmax]) of tremelimumab is related to a higher maximum pharmacodynamic effect in the NSCLC patient population. Overall, the PK/pharmacodynamic data suggest that tremelimumab of dose greater than 1 mg/kg with a higher peak exposure may be associated with a higher pharmacodynamic effect.

Additionally, based on simulation data, the Cmax (78 μg/mL) post single dose administration of tremelimumab 4 mg/kg is approximately 4-fold higher than the predicted Cmax (19 μg/mL) post the first dose of tremelimumab 1 mg/kg, and is 3-fold higher than the predicted Cmax (25 μg/mL) post the fourth dose of tremelimumab 1 mg/kg in a Q4W×4 doses setting.

Clinical data

The safety and preliminary efficacy of combination of durvalumab 1500 mg plus single dose of tremelimumab 300 mg in unresectable HCC population is being evaluated in the ongoing Phase I/II study (Study D4190C00022).

In summary, a single dose of tremelimumab 4 mg/kg, while maintaining a similar overall

exposure, has a 3- to 4-fold higher Cmax compared to the 4 doses of tremelimumab 1 mg/kg. Therefore, this single administration of the higher dose of tremelimumab may have the potential for better anti-tumor activity while potentially avoiding any cumulative toxicity associated with repeated dosing of the 1 mg/kg tremelimumab. Therefore, the regimen of durvalumab 1500 mg plus tremelimumab 300 mg×1 dose is being evaluated in the current study.

Rationale for fixed dosing

A fixed-dose regimen of 1500 mg (equivalent to 20 mg/kg) of durvalumab plus 300 mg (equivalent to 4 mg/kg) of tremelimumab will be used in this study.

2.2.2.1. Rationale for utilizing a fixed-dose regimen for durvalumab and tremelimumab

A population PK model was developed for durvalumab using monotherapy data from a Phase I study (Study CD-ON-MEDI4736-1108; N=292; doses=0.1 to 10 mg/kg Q2W or 15 mg/kg Q3W; solid tumors). Similarly, a population PK model was developed for tremelimumab using data from Phase I through Phase III (N=654; doses=0.01 to 15 mg/kg Q4W or Q90D; metastatic melanoma) ([66]Wang et al 2014).

Population PK analysis indicated only minor impact of body weight on the PK of durvalumab and also tremelimumab (coefficient of ≤0.5). The weight-based versus fixed-dose (based on median weight of approximately 75 kg) regimens of both durvalumab and tremelimumab were compared using predicted PK concentrations (5th, 50th, and 95th percentiles) using a population PK model. A total of 1000 patients were simulated using weight distribution of 40 kg to 120 kg. Simulation results demonstrate that weight-based versus fixed dosing regimens of both durvalumab and tremelimumab yield similar median steady state PK concentrations with slightly less overall between-subject variability.

Similar findings have been reported by others ([22], [67] Narwal R, et al. Clin Pharmacokinet. 2013、[68] Ng CM, et al. Pharm Res. 2006, [69] Wang DD, et al. J Clin Pharmacol 2009, [70] Wolchok JD, et.al. N Engl J Med. 2013). Wang and colleagues investigated 12 monoclonal antibodies and found that fixed and body size-based dosing perform similarly, with fixed dosing being better for 7 of 12 antibodies ([21], [70] Wolchok JD, et al. N Engl J Med. 2013). In addition, they investigated 18 therapeutic proteins and peptides and showed that fixed dosing performed better for 12 of 18 in terms of reducing the between-subject variability in PK/pharmacodynamics parameters ([70] Wolchok JD, et al. N Engl J Med. 2013).

A fixed-dose approach is preferred by the prescribing community due to ease of use and reduced dosing errors. Given expectation of similar PK exposure and variability, we considered it feasible to switch to fixed-dose regimens. Based on the average body weight of 75 kg, a fixed dose of 1500 mg durvalumab (equivalent to 20 mg/kg) and a fixed dose of 300 mg tremelimumab (equivalent to 4 mg/kg) are selected for the current study. Therefore, the selected regimen of the durvalumab (+tremelimumab) cohort combined with particle therapy are:

【Cohort A】
Durvalumab monotherapy of 1500 mg Q4W

【Cohort B】
Durvalumab 1500 mg plus tremelimumab 300 mg for 1 dose, followed by durvalumab monotherapy 1500 mg Q4W

2.2.3 Rationale for carbon-ion radiotherapy

Historically, the role of radiation therapy in the treatment of liver tumors has been limited because of radiation-induced hepatic insufficiency caused by whole-liver irradiation ([71] Reed GB, et al. Am J Pathol, 1966). Particle therapy, including proton and carbon, overcame this problem with its physical dose distribution, enabling to treat large tumors while sparing normal liver tissue ([32], [72] Kanai et al. Int J Radiat Oncol Biol Phys, 1999). Results of CIRT have

been reported in multiple single institutional prospective studies and multi-institutional retrospective studies with high efficacy and mild toxicities ([34], [73] Shibuya K, et al. Liver Int. 2018, [74] Yasuda S, et al. Adv Radiat Oncol. 2019). Cases with MVI are also well treated with CIRT [35] Komatsu S, et al. J Gastroenterol. 2011). A single-arm clinical trial of CIRT in patients without large vessel or bile duct invasion is currently underway as an advanced medical treatment B, with the aim of developing a curative treatment for first-episode HCC that is not suitable for surgery (liver transplantation is not suitable) or radiofrequency ablation (jRCT1032200036).

Dose escalation studies have been conducted for 4 fraction CIRT ([34], [75]Shiba S, et al. Radiat Oncol. 2019). 60Gy (RBE) in 4 fractions appear to be well tolerated and multi-institutional retrospective study confirms its efficacy ([73] Shibuya K, et al. Liver Int. 2018). Constraints for each risk organ were determined based on previously published reports ([34], [73], [76] Shiba S, et al. Radiat Oncol 2020, [77] Makishima et al. Cancer Sci. 2018).

2.2.4 Rationale for combined treatment of carbon-ion radiotherapy and immunotherapy

In the PACIFIC trial, the combination of durvalumab and chemoradiotherapy (X-rays) has been shown to provide high therapeutic efficacy with intolerable toxicity, as discussed in 1.7 above ([41]Antonia SJ et al. NEJM. 2017). There are only ongoing trials of the combination of immune checkpoint inhibitors, including durvalumab, with particle therapy, and no reports exist as of November 2020. Trials of combinations with heavy particle therapy are similarly unregistered. As noted in 1.7 above, the results of basic research and clinical trials of combination therapy with X-rays suggest a higher synergistic effect.

2.3. Benefit-risk and ethical assessment

2.3.1. Potential benefits

2.3.1.1. Durvalumab monotherapy

Information on the potential benefit of durvalumab 1500 mg monotherapy or equivalent in patients with HCC are based on Study CD-ON-MEDI4736-1108 and are presented in Section 1.8.1. For other tumor types, see the most current durvalumab IB.

2.3.1.2. Durvalumab plus tremelimumab combination therapy

The potential benefits of adding tremelimumab to durvalumab is presented in Section 1.8.3. Information on the data supporting the selected combination regimen of durvalumab plus tremelimumab in patients with HCC are presented in Section 1.8.4. For other tumor types, see the most current durvalumab and tremelimumab IBs.

2.3.1.3. Durvalumab (+tremelimumab) combined with particle therapy

As mentioned in section 1.7, CIRT, as with traditional photon irradiation, is known to modify cancer immune reactions, but at a stronger level (Helm A, et al. Int J Part Ther. 2018, Ebner et al. Front Oncol. 2017). By combining CIRT with innunotherapy [durvalumab (+ tremelimumab)], there may be a larger improvement in efficacy compared to monotherapy of immunotherapy drugs.

2.3.2. Overall risks

Monoclonal antibodies directed against immune checkpoint proteins, such as programmed cell death ligand 1 (PD-L1) as well as those directed against programmed cell death-1 (PD-1) or cytotoxic T-lymphocyte antigen-4 (CTLA-4), aim to boost endogenous immune responses directed against tumor cells. By stimulating the immune system however, there is the potential for adverse effects on other tissues.

Most adverse drug reactions seen with the immune checkpoint inhibitor class of agents are thought to be due to the effects of inflammatory cells on specific tissues. These risks are generally

events with a potential inflammatory or immune mediated mechanism and which may require more frequent monitoring and/or unique interventions such as immunosuppressants and/or endocrine therapy. These immune mediated effects, can occur in nearly any organ system, and are most commonly seen as gastrointestinal AEs such as colitis and diarrhea, pneumonitis/interstitial lung disease (ILD), hepatic AEs such as hepatitis and liver enzyme elevations, skin events such as rash and dermatitis and endocrinopathies including hypo- and hyper-thyroidism.

2.3.2.1. Durvalumab

Risks with durvalumab include, but are not limited to, diarrhea/colitis, pneumonitis/ILD, endocrinopathies (hypo- and hyper-thyroidism, type I diabetes mellitus, including diabetic ketoacidosis, hypophysitis and adrenal insufficiency) hepatitis/increases in transaminases, nephritis/increases in creatinine, diabetes insipidus, pancreatitis/increases in amylase and lipase, rash/pruritus/dermatitis, encephalitis, myocarditis, immune thrombocytopenia, myositis/polymyositis, other rare or less frequent inflammatory events including neurotoxicities, infusion-related reactions, injection site reactions,

hypersensitivity reactions and infections/serious infections.

For information on all identified and potential risks with durvalumab please always refer to the current version of the durvalumab IB.

In monotherapy clinical studies AEs (all grades) reported very commonly (≥ 15% of patients) are fatigue, nausea, decreased appetite, dyspnea, cough, constipation, diarrhea, vomiting, back pain, pyrexia, asthenia, anemia, arthralgia, peripheral edema, headache, rash, and pruritus. Approximately 9.4% of patients experienced an AE that resulted in permanent discontinuation of durvalumab and approximately 6.5% of patients experienced an SAE that was related to durvalumab by the study investigator.

Most treatment-related AEs were manageable with dose delays, symptomatic treatment, and in the case of events suspected to have an immune basis, the use of established treatment guidelines for immune-mediated toxicity.

A detailed summary of durvalumab monotherapy AE data can be found in the current version of the durvalumab IB.

2.3.2.2. Tremelimumab

Risks with tremelimumab monotherapy include, but are not limited to, GI effects (colitis, diarrhoea, enterocolitis and intestinal perforation), endocrine disorders (hypo and hyperthyroidism, hypophysitis and adrenal insufficiency), skin effects (rash, and pruritus), elevations in lipase and amylase and clinical manifestations of pancreatitis, other gastrointestinal events e.g. ulcerative colitis, dehydration, nausea and vomiting; hepatic events including hepatitis, and liver enzyme elevations; pneumonitis and ILD; nervous system events including encephalitis, peripheral motor and sensory neuropathies, Guillain-Barre and proximal muscle weakness; cytopenias including thrombocytopenia, anemia and neutropenia; infusion-related reactions, anaphylaxis, and allergic reactions; renal events including renal failure, acute kidney injury, nephritis, nephrotic syndrome, autoimmune nephritis and electrolyte abnormalities such as hypokalemia; autoimmune diseases including autoimmune arthritis, Sjogren's syndrome and giant cell temporal arteritis; hyperglycemia and diabetes mellitus; and pyrexia.

For information on all identified and potential risks with tremelimumab please always refer to the current version of the tremelimumab IB.

Using pooled data from monotherapy clinical studies AEs (all grades) reported very commonly (≥ 10% of patients) were diarrhea, nausea, fatigue, pruritus, decreased appetite, rash, vomiting, dyspnoea, constipation, cough, pyrexia, abdominal pain, decreased weight, headache, asthenia, and anaemia. Approximately 16% of patients experienced an AE that resulted in permanent discontinuation of tremelimumab and approximately 45% of patients experienced an SAE.

A detailed summary of tremelimumab monotherapy AE data can be found in the current version of the tremelimumab IB.

2.3.2.3. Durvalumab + tremelimumab

The safety of durvalumab + tremelimumab combination therapy was initially evaluated in the ongoing dose escalation and dose expansion Study 006, in patients with NSCLC, and is being studied in a number of other ongoing clinical trials, in a number of different indications, and has to date shown a manageable safety and tolerability profile.

The types of risks with the combination of durvalumab + tremelimumab (based on an equivalent durvalumab dose of 20m/kg and a tremelimumab dose of 1mg/kg) are similar to those for durvalumab and tremelimumab monotherapy. Emerging data from study 006, other studies evaluating the combination, and from combinations of other agents in the same class indicate an increased frequency and/or severity of some of these immune-mediated toxicities.

For information on all identified and potential risks with the durvalumab+tremelimumab combination please always refer to the current version of the durvalumab IB

In durvalumab+tremelimumab combination studies at the dose of durvalumab 20mg/kg and tremelimumab 1mg/kg AEs (all grades) reported very commonly (≥ 10% of patients) are fatigue, diarrhoea,nausea, dyspnea, decreased appetite, pruritus, vomiting, anaemia, constipation, cough, abdominal pain, pyrexia, back pain, arthralgia, hypothyroidism, asthenia, oedema peripheral, decreased weight, decreased hyponatraemia and rash.

Approximately 15% of patients experienced an AE that resulted in permanent discontinuation of study drug and approximately 16% of patients experienced an SAE that was considered to be related to durvalumab and tremelimumab by the study investigator.

A detailed summary of durvalumab + tremelimumab combination AE data can be found in the current version of the durvalumab IB.

2.3.2.4. Carbon-ion radiotherapy

Safety of CIRT for HCCs as a monotherapy are confirmed through multiple single institutional prospective studies and multi-institutional retrospective studies ([34], [73], [74]). Grade 3 or severe acute toxicities were seen in skin (1%) and ALT elevation (0.6%), with both being G3. G3 or severe late toxicities were seen in skin (G3 2%, G4 0.6%), hepatic coma (G3 1%), myositis and rib fracture (both G3 0.6%). RILD was seen in 2%. No treatment related deaths were observed.

Since CIRT is a form of radiotherapy, potential risks will be confined within the radiation field. The following may be observed.

- Bone marrow suppression
- Radiation pneumonitis, pleuritis, pleural effusion
- Nausea, vomiting, anorexia, diarrhoea, GI bleeding, ulceration, perforation, stricture
- Loss of hepatic function, hepatic failure, RILD, bile duct stricture, occlusion, cholangitis, aneurism
- Radiation dermatitis, ulceration
- Pericarditis, pericardial effusion, congestive heart failure, myocarditis, arrythmia
- Myelopathy, Peripheral neuropathy
- Rib fracture, subcutaneous induration, subsequent primary cancer, renal failure

2.3.2.5. durvalumab (+tremelimumab) combined with carbon-ion radiotherapy

Currently, there are no clinical trial results open yet for durvalumab + tremelimumab + radiotherapy for liver tumors, thus risks are unknown. Combination of immuno-oncology drugs and radiotherapy appear to have minimal excessive toxicity compared to immune-oncology drugs alone across multiple trials, including thoracic irradiation ([41] Antonia SJ, et al. NEJM 2018).

Potential risks would be toxicities closely related to immune reaction and microangiopathy, such as RILD, radiation pneumonitis and GI tract ulceration. In CIRT as a monotherapy, these toxicities are less common compared to photon radiotherapy, owing to the better dose distribution of CIRT as explained above ([34], [73], [74]). While the impact of difference in immune-

modulation against toxicity between photon and carbon-ion is unknown, there is no definite evidence that excessive toxicity will be intolerable.

2.3.3. Overall benefit-risk

Durvalumab and tremelimumab have shown encouraging anti-tumor activity as single agents in advanced HCC population. The summary of this efficacy data is presented in Section 1.8.4. The combination regimen of these two agents shows a higher response rate in HCC population compared to either of the monotherapies. Thus, durvalumab plus tremelimumab combination therapy may potentially offer benefit to this patient population. Both durvalumab monotherapy and durvalumab plus tremelimumab combination therapy was tolerable in advanced HCC. The current study design aims to minimize potential risks by providing for early and intensive safety monitoring for any unexpected safety signals and for managing those risks deemed to be most likely based on prior experience with durvalumab, tremelimumab, and carbon-ion therapy. Two combination dose regimens of durvaluamb plus tremelimumab combination therapy were selected for this study with the aim to select the regimen with the most benefit for patients with advanced HCC.

HCC patients with MVI have very limited systemic therapeutic options and a poor life expectancy and health-related quality of life (HRQoL) based on the currently available treatments. While the main prognosticator in these patients is the intravascular growth of MVI, thus focal treatment by particle therapy has shown prolonged MST up to 2 years if lesions are confined in number ([40] Sugahara S, et al. Strahlether Onkol. 2009)). But those with extensive disease (ie multiple hepatic lesions and extrahepatic lesions) are currently not treated with particle therapy in fear of the out-of-treatment-field lesions. HCC patients with MVI, therefore, represents a significant unmet medical need and underlines the need for novel therapies for this patient population. CIRT combined with durvalumab or durvalumab plus tremelimumab proposed in this study may demonstrate a meaningful clinical benefit and a manageable safety profile. The overall benefit-risk profile of durvalumab (+tremelimumab) combined with CIRT is expected to be favorable, therefore supporting the current study design.

3. ELIGIBILITY

Each patient must meet all of the inclusion criteria (Section 3.2) and none of the exclusion criteria (Section 3.3) for this study. Under no circumstances will there be exceptions to this rule.

3.1. Diagnostic Criteria and Stage, Type, and Condition Classification

【CohortA & CohortB】

Eligible patients will be advanced HCC with MVI and Child-Pugh classification A over 20 years of age who are refractory or intolerant to standard systemic chemotherapy.

【Expansion cohort】

Eligible patients are advanced HCC with MVI, aged 20 years or older, with Child-Pugh classification A, with or without prior drug therapy.

3.2. Inclusion criteria

For inclusion in the study, patients should fulfill the following criteria:

1） Capable of giving signed informed consent which includes compliance with the requirements and restrictions listed in the informed consent form (ICF) and in this protocol. Written informed consent and any locally required authorization obtained from the patient/legal representative prior to performing any protocol-related procedures, including screening evaluations. For patients aged <20 years and enrolling, a written informed consent should be obtained from the patient and his or her legally acceptable representative.

2） Age >20 years at time of study entry

3） Eastern Cooperative Oncology Group (ECOG) performance status of 0 or 1

4） Body weight >30 kg

5） Adequate normal organ and marrow function as defined below:
- Haemoglobin ≧9.0 g/dL
- Absolute neutrophil count (ANC) ≧1,500/mm3
- Platelet count ≧75×109/L（≧75,000/mm3）
- Serum bilirubin ≦ULN×3.0
- AST ≦ULN×5.0
- ALT ≦ULN×5.0
- Measured creatinine clearance (CL) >40 mL/min or Calculated creatinine clearance CL>40 mL/min by the Cockcroft-Gault formula (Cockcroft and Gault 1976) or by 24-hour urine collection for determination of creatinine clearance

6） Evidence of post-menopausal status or negative urinary or serum pregnancy test for female pre-menopausal patients. Women will be considered post-menopausal if they have been amenorrheic for 12 months without an alternative medical cause. The following age-specific requirements apply:
- Women <50 years of age would be considered post-menopausal if they have been amenorrheic for 12 months or more following cessation of exogenous hormonal treatments and if they have luteinizing hormone and follicle-stimulating hormone levels in the post-menopausal range for the institution or underwent surgical sterilization (bilateral oophorectomy or hysterectomy).
- Women ≥50 years of age would be considered post-menopausal if they have been amenorrheic for 12 months or more following cessation of all exogenous hormonal treatments, had radiation-induced menopause with last menses >1 year ago, had chemotherapy-induced menopause with last menses >1 year ago, or underwent surgical sterilization (bilateral oophorectomy, bilateral salpingectomy or hysterectomy).

7） Patient is willing and able to comply with the protocol for the duration of the study including undergoing treatment and scheduled visits and examinations including follow up.

8） Advanced HCC confirmed histologically or by the typical findings of a hypervascular tumor

on computed tomography or angiography

9) (Cohort A and Cohort B only) Patients who have received at least one prior systemic chemotherapy regimen including atezolizumab bevacizumab combination, sorafenib, or lenvatinib and who are determined to be refractory or intolerant to standard therapy.

10) Must not be eligible for locoregional therapy for unresectable HCC. For patients who progressed after locoregional therapy for HCC, locoregional therapy must have been completed ≥28 days prior to the baseline scan for the current study. Acceptable locoregional therapy for HCC are Ethanol Infusion Therapy, Radio Wave ablation Therapy, Transcatheter Arterial chemoembolization (TACE), Transcatheter arterial infusion (TAI). Hepatic Arterial Infusion Chemotherapy (HAIC) is not treated as a locoregional therapy.

11) Patients who have been diagnosed with HCC showing MVI. MVI is defined as a tumor thrombus in the major hepatic and/or portal vein branches (Vp2, Vp3, Vp4, Vv2, and Vv3) identified by imaging studies.

12) Child-Pugh A

13) At least one measurable lesion other than the MVI and feeding nodule based on mRECIST.

3.3. Exclusion criteria

Patients should not enter the study if any of the following exclusion criteria are fulfilled:

1) Involvement in the planning and/or conduct of the study (applies to both sponser and/or staff at the study site)

2) Patients who have participated in another clinical trial using the investigational drug within 28 days prior to obtaining consent or who have received another investigational drug within 28 days prior to the first dose of the investigational drug in this study. The exception is if the patient is in the follow-up period of an interventional trial or is participating in an observational (non-interventional) clinical trial.

3) Any unresolved toxicity NCI CTCAE Grade ≥2 from previous anticancer therapy with the exception of alopecia, vitiligo, and the laboratory values defined in the inclusion criteria
   - Patients with Grade ≥2 neuropathy will be evaluated on a case-by-case basis after consultation with the Study Physician.
   - Patients with irreversible toxicity not reasonably expected to be exacerbated by treatment with durvalumab or tremelimumab may be included only after consultation with the Study Physician.

4) Radiotherapy treatment to more than 30% of the bone marrow or with a wide field of radiation within 4 weeks of the first dose of study drug

5) Major surgical procedure (as defined by the Investigator) within 28 days prior to the first dose of IP. Note: Local surgery of isolated lesions for palliative intent is acceptable.

6) History of allogenic organ transplantation.

7) Active or prior documented autoimmune or inflammatory disorders (including inflammatory bowel disease [e.g., colitis or Crohn's disease], diverticulitis [with the exception of diverticulosis], systemic lupus erythematosus, Sarcoidosis syndrome, or Wegener syndrome [granulomatosis with polyangiitis, Graves' disease, rheumatoid arthritis, hypophysitis, uveitis, etc.]). The following are exceptions to this criterion:
   - Patients with vitiligo or alopecia
   - Patients with hypothyroidism (e.g., following Hashimoto syndrome) stable on hormone replacement
   - Any chronic skin condition that does not require systemic therapy
   - Patients without active disease in the last 5 years may be included but only after consultation with the study physician
   - Patients with celiac disease controlled by diet alone

8) Uncontrolled intercurrent illness, including but not limited to, ongoing or active infection, symptomatic congestive heart failure, uncontrolled hypertension, unstable angina pectoris,

cardiac arrhythmia, interstitial lung disease, serious chronic gastrointestinal conditions associated with diarrhea, or psychiatric illness/social situations that would limit compliance with study requirement, substantially increase risk of incurring AEs or compromise the ability of the patient to give written informed consent

9) History of another primary malignancy except for
   - Malignancy treated with curative intent and with no known active disease ≥5 years before the first dose of IP and of low potential risk for recurrence
   - Adequately treated non-melanoma skin cancer or lentigo maligna without evidence of disease
   - Adequately treated carcinoma in situ without evidence of disease
   - However, the following cases are eligible for enrollment
   - Early stage cancer (epithelial cancer of the cervix, basal cell carcinoma, superficial bladder cancer (Tis and T1), early stage gastric cancer, and early stage colorectal cancer) that has been treated for curative purposes, has not been confirmed active for at least 3 years prior to inclusion in the study, and has a low risk of recurrence.

10) History of leptomeningeal carcinomatosis

11) History of, or current, brain metastases or spinal cord compression. Patients with suspected brain metastases at screening should have an MRI (preferred) or CT, each preferably with IV contrast of the brain prior to study entry.

12) Mean QT interval corrected for heart rate using Fridericia's formula (QTcF) ≥470 ms calculated from 3 ECGs (within 15 minutes at 5 minutes apart) Regardless of whether this criteria stays or not, all patients should have a baseline ECG

13) History of active primary immunodeficiency

14) Patients co-infected with HBV and HCV. HBV positive (presence of HBsAg and/or anti-HBcAb with detectable HBV DNA); HCV positive (presence of anti-HCV antibodies), and active infection including tuberculosis (clinical evaluation that includes clinical history, physical examination and radiographic findings, and TB testing in line with local practice).

15) Current or prior use of immunosuppressive medication within 14 days before the first dose of durvalumab or tremelimumab. The following are exceptions to this criterion:
   - Intranasal, inhaled, topical steroids, or local steroid injections (e.g., intra articular injection)
   - Systemic corticosteroids at physiologic doses not to exceed 10 mg/day of prednisone or its equivalent
   - Steroids as premedication for hypersensitivity reactions (e.g., CT scan premedication)

16) Receipt of live attenuated vaccine within 30 days prior to the first dose of IP. Note: Patients, if enrolled, should not receive live vaccine whilst receiving IP and up to 30 days after the last dose of IP.

17) Female patients who are pregnant or breastfeeding or male or female patients of reproductive potential who are not willing to employ effective birth control from screening to 90 days after the last dose of durvalumab monotherapy or180 days after the last dose of durvalumab + tremelimumab combination therapy.

18) Known allergy or hypersensitivity to any of the study drugs or any of the study drug excipients.

19) Prior randomisation or treatment in a previous durvalumab and/or tremelimumab clinical study regardless of treatment arm assignment.

20) Judgment by the investigator that the patient is unsuitable to participate in the study and the patient is unlikely to comply with study procedures, restrictions and requirements.

21) Patients who have been treated with anti-PD-1, anti-PD-L1 inhibitors, or other drugs that act on other stimulatory or co-suppressive T-cell receptors and their combinations (including atezolizumab plus bevacizumab) and have failed to tolerate the same treatment.

22) Prior radiotherapy involving the liver.

23) Renal failure requiring hemodialysis or peritoneal dialysis

24) Any of the following cardiac diseases:

- NYHA Class III or IV chronic heart failure
- Current coronary artery disease or history of ischemic heart disease such as myocardial infarction within 6 months before the study
- Serious arrhythmia (grade 3 or higher according to the CTCAE ver. 5.0: arrhythmia that cannot be controlled by oral medications or requires mechanical control).

25） Poorly controlled hypertension

26） Serious and active infection, excluding hepatitis viral infection

27） Persistent proteinuria of NCI-CTCAE version 5.0 grade 3 or higher.

28） Arterial or venous thrombotic or embolic events such as cerebrovascular accident, deep vein thrombosis, or pulmonary embolism within 6 months before the start of study medication.

29） Refractory pleural effusion or ascites

30） History of hepatic encephalopathy within past 12 months

31） Oral intake impossible

32） HIV-positive

33） Pulmonary fibrosis or interstitial pneumonitis

34） Other serious complications as follows: serious mental disease or history of gastrointestinal bleeding or active hemoptysis

35） Unsatisfactory general condition for participation in the study as judged by the primary physician

4. Informed consent

4.1. Preparation and revision of informed consent form

The investigator prepares the consent form and other information documents used to obtain consent for participation in the clinical trial from the subject in plain language as much as possible. If it is considered necessary to revise the consent document and other explanatory documents, the investigator revises these documents.

The investigator submits the prepared or revised consent documents and other explanatory documents to the IRB for approval.

Amendments to the study protocol and informed consent forms will follow the below procedures:

1. When amendments are considered to be necessary, the Principal Investigator will provide to the Investigator(s) the study protocol amendment draft, informed consent form amendment drafts, and the latest version of the investigator's brochure and other necessary material/information.
2. The Principal Investigator will provide the Investigator(s) with necessary time to adequately consider the aforementioned study protocol amendment draft and material/information and discuss the details with the Principal Investigator.
3. After discussion with the Principal Investigator, the Investigator(s) will promptly submit the amended version of the study protocol or informed consent form to the head of the trial site, and receive approval of the IRB via the head of the trial site.
4. Within acceptable limits of the Principal Investigator, the same procedures will apply to amendments to be made to the study protocol and informed consent form according to instructions given by the head of the trial site based the opinions of the IRB.

4.2. Method of Obtaining Informed Consent

1) Informed consent

The investigator or subinvestigator should hand the consent document and other explanatory documents to the subject and provide sufficient explanation of the contents as indicated in "4.3". If necessary, the clinical trial coordinator also provides supplementary explanations to the subject. After confirming that the patient has a good understanding of the contents of the clinical trial, the subject's signed and dated informed consent should be obtained before the pre-study (screening) test is conducted.

2) When explaining to subjects

The investigator or sub-investigator shall give the subject the opportunity to ask questions and sufficient time to decide whether or not to participate in the trial before obtaining informed consent and shall answer the subject's questions to the subject's satisfaction.

3) Signing and delivery of consent form

The investigator or subinvestigator who provided the explanation should sign the consent form with the date of the explanation. The subject signs the consent form with the date of consent. If a collaborator provides supplementary explanation, the collaborator should also sign and enter the date of the explanation. After obtaining informed consent, a copy of the information document and the consent form shall be given to the subject.

4) Amendments to informed consent forms

When the investigator or subinvestigator revises the informed consent form or other explanatory documents due to the acquisition of new information that may be relevant to the subject's consent, the investigator or subinvestigator shall explain to the subject again using the revised informed consent form and other explanatory documents, and obtain consent in writing for the subject's continued participation in the clinical trial. If new important information is obtained that may affect the subject's consent, the information shall be immediately provided to the subject, recorded in writing, and the subject's continued participation in the clinical trial shall be confirmed.

4.3. Information to be provided to subjects

The informed consent form to be prepared by the investigator shall include the following information.

1. What is a clinical trial?
2. The purpose of the clinical trial
3. Name, title and contact information of the investigator
4. Method of the clinical trial
5. Anticipated clinical benefits and risks or inconveniences
6. Availability of other treatment options for the subject and the expected important benefits and risks associated with such treatment options
7. The expected duration of the subject's participation in the clinical trial
8. That participation in the clinical trial is of the subject's own free will and that the subject may refuse or withdraw from participation in the clinical trial at any time. Furthermore, the subject shall not be treated unfavorably due to refusal or withdrawal, and shall not lose any benefits that he/she would have received if he/she had not participated in the clinical trial.
9. Monitors, auditors, clinical trial review committees, and regulatory authorities must be able to view source documents related to medical care. In such cases, the confidentiality of the subject shall be maintained. In addition, the subject's signature on the consent document shall be considered as authorization for access.
10. Subjects' confidentiality shall be maintained even if the results of the clinical trial are made public.
11. Compensation and treatment to which subjects are entitled in the event of adverse health effects related to the clinical trial.
12. Information that may influence the subject's decision to continue participation in the clinical trial will be promptly communicated to the subject.
13. Conditions or reasons for discontinuation of participation in the clinical trial
14. Expenses to be borne by the subject in relation to the clinical trial
15. Details of any financial or other payments to be made to the subject in connection with the clinical trial (e.g., arrangements for calculating the amount to be paid)
16. The medical institution's contact person to whom subjects should refer or contact if they require further information regarding the clinical trial and their rights or if they experience any health problems related to the clinical trial.
17. Items to be observed by the subject
18. Types of investigational review committees that will investigate and deliberate on the appropriateness of the clinical trial, matters to be investigated and deliberated by each investigational review committee, and other matters related to the investigational review committee for the clinical trial in question
19. Intellectual property
20. Conflicts of interest

5. STUDY DESIGN

5.1. Overview of study design

This is a Phase Ib study to assess the safety of durvalumab combined with particle therapy (Cohort A) and durvalumab plus tremelimumab combined with particle therapy (Cohort B) in advanced hepatocellular carcinoma patients with macrovascular invasion. This study consists of four periods: the screening period, DLT assessment period, durvalumab q4W dosing period, and follow up period. After the signed informed consent is obtained and the screening is conducted, the patient will be registered for enrollment in the trial. Patients will be administered with the first IP followed by administration of CIRT.

DLT assessment period is for 42days starting from the first dose of durvalumab on Day1 of Cycle1. The first administration of continuous durvalumab q4W on Day 1 of Cycle 2 starts only after the safety of Cycle 1 was confirmed (durvalumab q4W: 28 day cycle).

DLT analysis will be made when more than one DLT was observed in each cohort.

Patients will to continue to receive durvalumab every 4 week after completion of the DLT assessment period until clinical progression/withdrawal from the trial if there may be potential clinical benefit at the investigator's discretion.

Follow up visit will be made 28 days after study termination due to PD or withdrawal from the study. Safety information will be collected until 90 days after the last dose of study treatment or until initiation of alternative anticancer therapy. In this study, three patients are initially enrolled into cohort A. If there is no DLT observed in any of these subjects, the trial proceeds to enroll additional subjects into the cohort B, whose regimen does not contain higher dose of durvalumab but contains an additional drug of tremelimumab instead. If one subject develops a DLT at cohort A or cohort B, an additional three subjects are enrolled into that same cohort. Development of DLTs in more than 1 of 6 subjects in either cohort suggests that the regimen is not tolerable. If cohort A turns out to be intolerable, then cohort B regimen will not be pursued The evaluation of DLTs shall be performed by the investigator of Chiba University Hospital in consultation with the investigator(s). The DLTs determined shall be discussed with the Independent Data Monitoring Committee and their opinion shall be sought in accordance with the standards separately stipulated (10.7. Independent Data Monitoring Committee).

Duration of DLT assessment is defined for 42 days starting from the first administration of IP on Day1 of Cycle 1. Dose of durvalumab is fixed on 1500 mg. CIRT will be performed between Day 8 to Day 14 of Cycle 1 after the first duvalumab administration on Day1 (CIRT within 14 days after 1stcycle of durvalumab).

In both cohorts, if the investigators determined that there may be potential clinical benefit, patients will to continue to receive durvalumab every 4 week until clinical progression (Durvalumab q4W dosing period).

- **Cohort A**: Durvalumab 1500mg will be administered every 4 weeks in principle. Particle therapy, in form of CIRT, will be performed after Day8 of Cycle1 following the 1st dose of Durvalumab on Day1. 2nd cycle of durvalumab will be administrated only after the safety during DLT assessment was confirmed.

- **Cohort B**: Durvalumab 1500mg will be administered every 4 weeks in principle, and Tremelimumab 300mg will be administered only on Day1 of Cycle1. Particle therapy, in form of CIRT, will be performed after Day8 of Cycle1 following 1st cycle of Durvalumab + Tremelimumab. 2nd cycle of durvalumab will be administrated only after the safety during DLT assessment was confirmed.

CIRT will be given to both arms. Dose prescription and fractionations will be 60Gy (RBE) / 4Fr /

1week. Target lesion of the particle therapy will be focused on intrahepatic nodule with MVI. A 1cm margin will be taken as a clinical target volume margin for the feeding nodule, and 2cm margin alongside the vessel for the MVI lesion. Internal motion will be compensated according to 4D-CT movement assessment. Inter-fractional margin will be set to 3mm and combined with internal motion compensation forming a field specific planning treatment volume. Dose constraints for OARs will be prioritized over target volume coverage.

If both of Cohort A and B regimens were confirmed tolerable after DLT assessment, additional patients will be enrolled in Cohort B up to a total of 15 subjects. If only Cohort A regimen was determined to be tolerable, additional patients may be enrolled in Cohort A up to a total of 15 subjects.

Schedule for administration of investigational drugs and carbon ion radio therapy（Figure1）

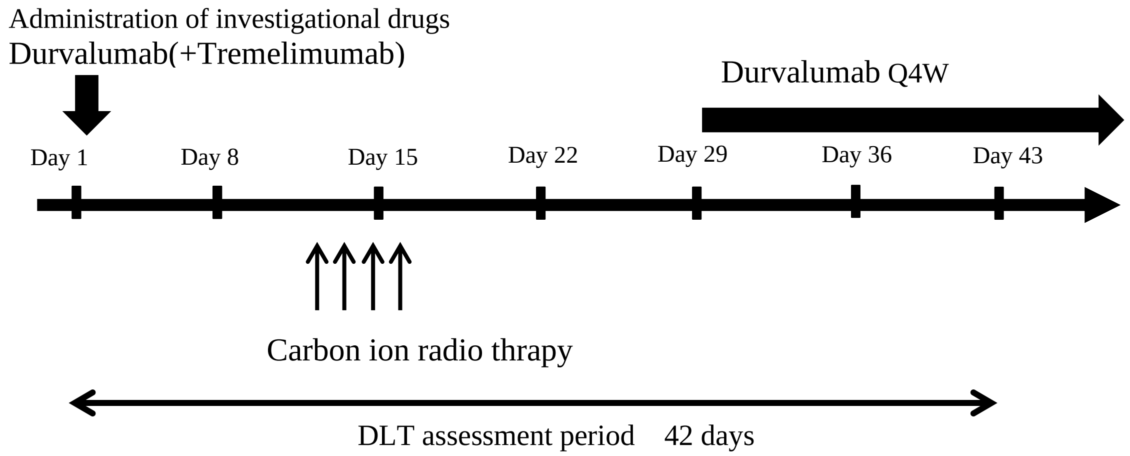

Tumor assessments, based on RECIST v.1.1 and mRECIST, will be performed every 6 weeks (Q6W) (±1 week) for the first 12 weeks from the date of randomization and then Q8W (±1 week) thereafter until RECIST 1.1-defined radiological progression followed by a subsequent scan if clinically feasible, evaluated by Confirmation of Radiological Progression criteria (Appendix B). Patients who permanently discontinue study drug(s) for reasons other than PD should continue to have radiographic scans performed per their original schedule until confirmed PD

Subjects with rapid tumor progression or tumor-associated syndromes requiring urgent medical intervention (e.g., central nervous system metastases, respiratory failure due to tumor compression, spinal cord compression) will be deemed ineligible for continued durvalumab.

The overview of this clinical trial is as follows.

Cohort A

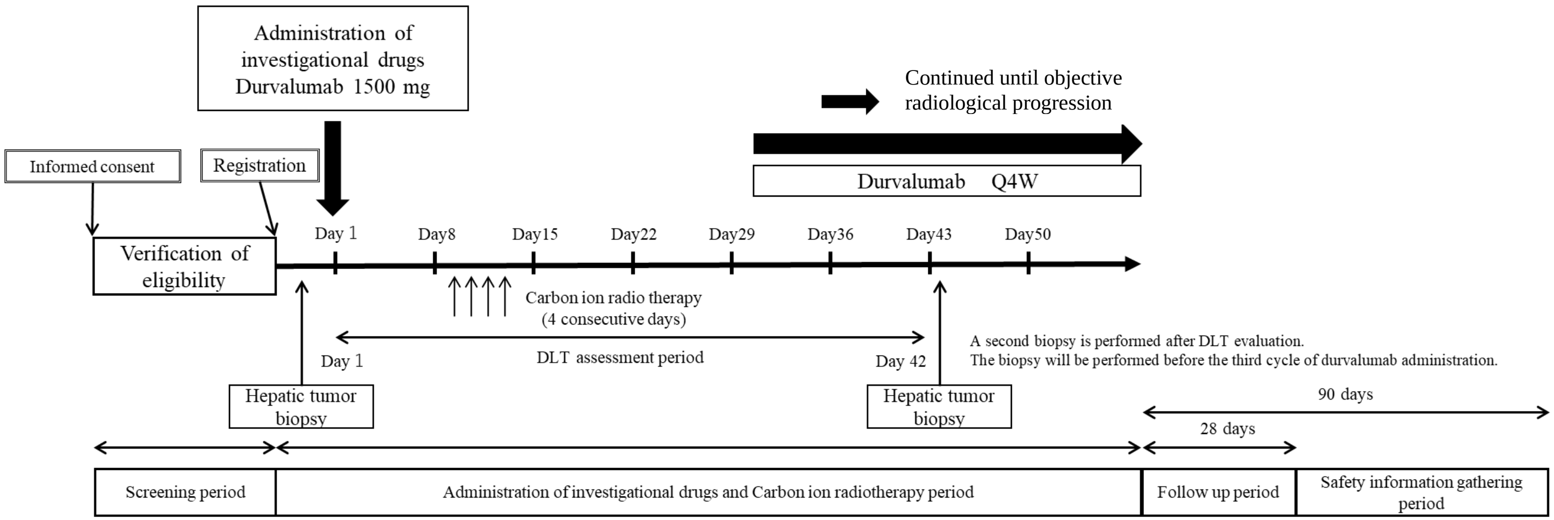

Cohort B

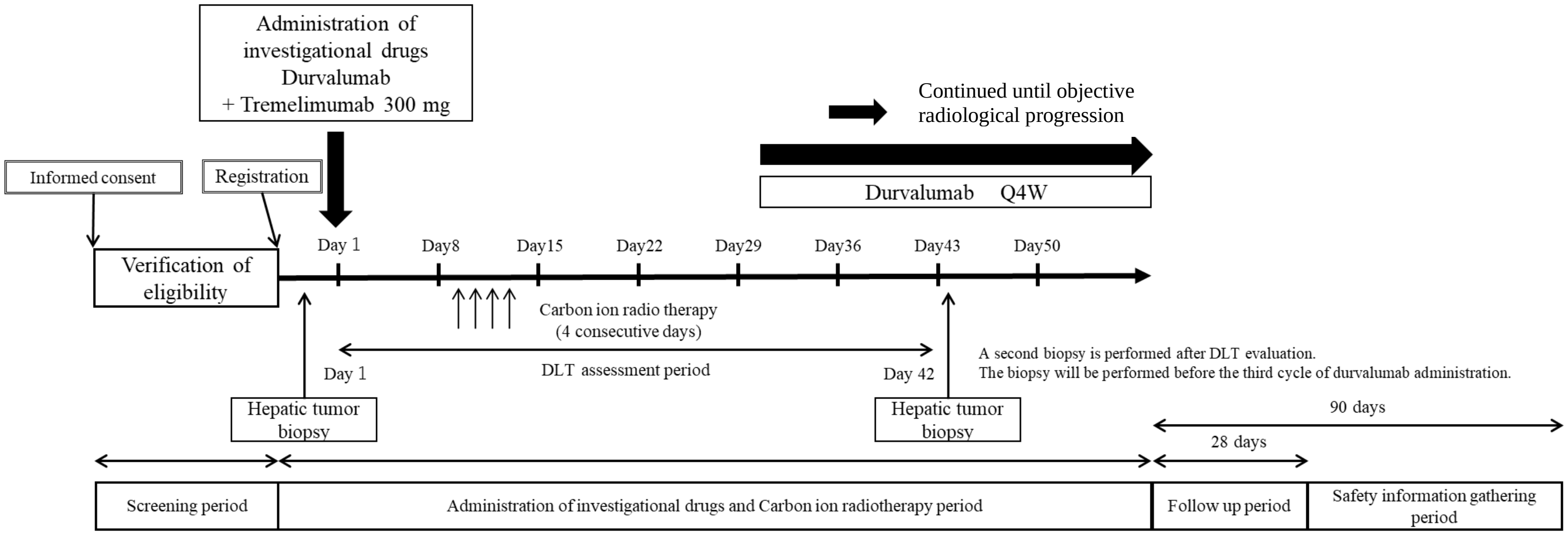

5.2. Target number of subjects and study duration

Target number of subjects：15

Study Period：
Estimated study start date 30 Apr 2021
Estimated study completion date 30 Sep 2023
Subject registration period:
Estimated date of first patient enrolled 30 Apr 2021
Estimated date of last patient enrolled 31 Mar 2023

Study schema

This study consists of four periods: the screening period, DLT assessment period, durvalumab q4W dosing period, and follow up period.

Signed informed consent will be obtained and patients will be screened prior to enrollment. The investigational treatment will include carbon ion radiotherapy on or after day 8 of cycle 1 after the first dose of study drug; the DLT evaluation period is 42 days from the first dose of durvalumab on day 1 of cycle 1. After confirmation of the safety of Cycle 1, durvalumab will be administered sequentially in Q4W beginning on Day 1 of Cycle 2. If multiple DLTs are observed in each cohort, an independent data monitoring committee will be convened to provide input on the determination of intolerability.

If no DLT is observed after the DLT evaluation period, durvalumab will be continued every 4 weeks until objective confirmation of disease progression (7.4) or until the criteria for 5.6 are met. Post-study follow-up for individual cases will occur 28 days after discontinuation of investigational drug (date of decision to discontinue). Adverse events and serious adverse event outcomes in subjects will be collected 90 days after discontinuation of study treatment (date of decision to discontinue) or until initiation of alternative anticancer therapy.

Figure2.

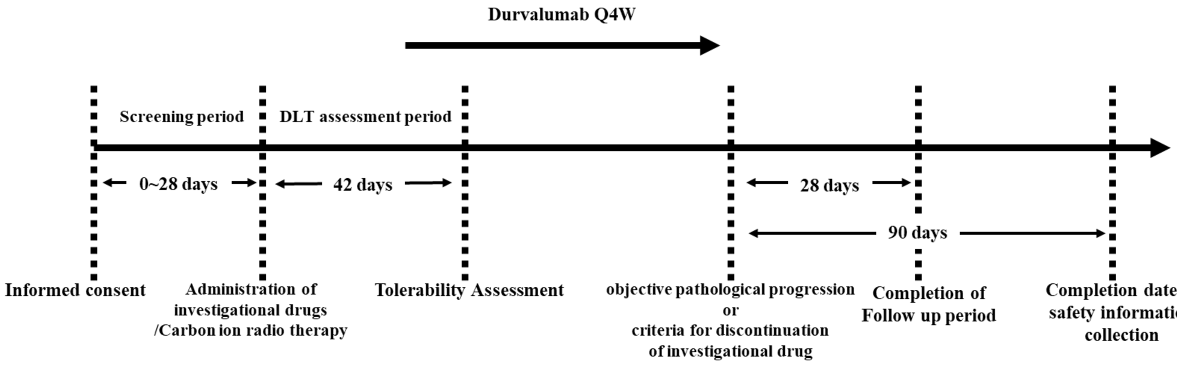

5.3. Monitoring for safety assessment

In situations where the below information is obtained and study patients are placed at under risk, or the continuation of the clinical trial is determined to be difficult, the Principal Investigator may decide on the termination or suspension of the entire clinical trial upon discussion with the Investigators. In addition, the study may be stopped based on the opinions by the DMC.

1. Occurrence of an unpredictable serious adverse reaction
2. Any information that indicates that the number, frequency, and condition of predictable serious adverse reactions cannot be predicted from the investigator’s brochure

3. SAEs that have been determined to have no causal relations with the IP, but are later determined that there is a reasonable possibility, due to the number, frequency, and condition of occurrences
4. Research reports indicating the tendency of the number, frequency, and condition of occurrences of adverse reactions having changed drastically
5. Research reports indicating the possibility that cancer, other serious diseases, disabilities, or death may occur
6. Information indicating that efficacy of the study drug cannot be expected in this clinical trial
7. Information indicating that the IP does not have any efficacy or effect on the target disease of the clinical trial
8. Information on any of the following related to marketed drugs that include the same ingredients as the IP:
    - Termination of manufacturing, import, or retail
    - Collection or disposal
    - Any other measures taken against health and hygiene related risks

Regardless of the reason for discontinuation, all data available to the subject at the time of discontinuation must be documented in the eCRF. All reasons for discontinuation should be documented. In terminating a trial, the investigator ensures that the protection of the subject's interests is given due consideration.

5.4. Institutional and case registration methods

Site registration and case registration will be conducted under the central registration system at the Data Management Office, Department of Clinical Trials, Chiba University Hospital. Once the site registration is completed, case registration will be available from the site. The following procedures will be used for site registration by fax and case registration via the Web.

5.4.1. Facility registration

1) The investigator at each participating site shall send a copy of the approval letter from the investigational review committee and a request form for site registration to the site registration center by fax after approval is obtained from the investigational review committee at the site.
2) The site registration center registers the sites and sends a notification of completion of site registration to the investigator.

Registration Center (Department of Data Management, Department of Clinical Trials, Chiba University Hospital)<br>fax number: 043-222-1207<br>Tel：043-222-1206<br>※Open hours are Monday through Friday, 9:00 a.m. to 5:00 p.m. (except Saturdays, Sundays, national holidays, and year-end and New Year holidays)<br>If a fax is received outside of the receptionist's office hours, it will be accepted on the next working day. (except at the beginning of the New Year)

5.4.2. Subject resistration

1) The investigator or subinvestigator obtains written consent and registers the subject in the case registration system. The case registration will be done via the website. After registration, a screening test is conducted to confirm that subjects meet the selection criteria and do not violate the exclusion criteria.

2) The investigator, subinvestigator or collaborator accesses the designated URL and enters the information necessary for case registration on the website. The investigator or sub investigator confirms the eligibility determination on the screen, and if the subject is determined to be eligible, protocol treatment is initiated. Once a subject is enrolled, enrollment will not be cancelled.

* The investigator or sub-investigator shall not administer the investigational drug until the subject is enrolled and “eligible” by screening test.

Registration Web site（DATATRAK Enterprise Cloud）<br>URL：https://secure.datatrak.net<br>TEL：043-222-1206<br>※Open 365 days a year, 24 hours a day, including Saturdays, Sundays, and holidays<br>(except for system downtime due to maintenance checks, etc.)

5.4.3. Handling of subjects who are found to be ineligible

Subjects who do not meet "eligibility" for any reason, such as ineligibility, are not eligible for enrollment and administration of the investigational product. The investigator or sub-investigator will explain to the subject that he/she is not eligible for enrollment in the study. The investigator or sub-investigator will also record the reason for the subject's ineligibility in the source documents.

If a subject who does not meet all eligibility criteria is inadvertently enrolled or started on the investigational product, the investigator will discuss whether or not to continue the investigational product. The investigator will ensure that all decisions resulting from this discussion are properly documented. If consensus cannot be reached, administration of the investigational product to the subject will be discontinued.

5.5. Dosing schedule and dosage/administration method

- Durvalumab 1,500 mg single agent arm (Cohort A)

  Cycle 1 Durvalumab 1,500 mg intravenous infusion will start on Day 1; after the first dose of durvalumab, heavy ion therapy will be administered on or after Day 8 of Cycle 1. Cycle 2 durvalumab will be administered after the safety of Cycle 1 is confirmed. Thereafter, dosing will be continued every 4 weeks until objective disease progression is confirmed (7.4.), “5.6. Discontinuation of Investigational Drug” is met, and the study is terminated.

- Durvalumab 1,500 mg + tremelimumab 300 mg once combination therapy (Cohort B)

  Cycle 1 One dose of combination therapy with durvalumab 1,500 mg and tremelimumab (both intravenous infusions) will be administered starting on Day 1. Durvalumab infusion will be started approximately 1 hour (maximum 2 hours) after completion of tremelimumab administration; after the first dose of durvalumab plus tremelimumab, heavy ion therapy will be administered on or after Day 8 of Cycle 1. Begin durvalumab 1,500 mg monotherapy Q4W after Cycle 2. Cycle 2 durvalumab will be administered after the safety of Cycle 1 is confirmed. Thereafter, durvalumab will be administered every 4 weeks until the objective disease progression is confirmed (7.4.), “5.6. Discontinuation of Investigational New Drug” is met, and the study is terminated.

5.5.1. Criterion for reduction

No dose reductions will be made for either durvalumab or tremelimumab in this study.

5.5.2. Criterion for drug withdrawal

With reference to Appendix 2 “Guidelines for Toxicity Management”, investigators and subinvestigators may suspend the investigational drug.

5.6. Discontinuation of Investigational Drug

If any of the following criteria are met, the investigator or sub-investigator will discontinue the investigational product and perform an Investigational Product Discontinuation Study. A follow-up visit will be scheduled 28 days after discontinuation of investigational drug (date of decision to discontinue).

1) When a subject requests to withdraw from the clinical trial treatment.
2) When it is difficult to continue administration of the investigational drug due to exacerbation of complications
3) When it is difficult to continue administration of the investigational drug due to adverse events.
4) Pregnancy is detected.
5) Other cases in which the investigator or subinvestigator judges it necessary to discontinue the administration of the investigational drug.

Subjects may discontinue investigational treatment at any time, at their own discretion, without prejudice to subsequent treatment. Subjects who decide to discontinue an investigational drug should always be questioned about the reason for discontinuation and the presence or absence of AEs. Subjects who completely discontinue subsequent doses of study medication, regardless of the reason, must continue to attend the clinic for evaluation according to the study protocol. If the subject does not agree to continue the visit, the follow-up procedure may be modified to ensure collection of endpoints and safety information. This follow-up may include telephone contact with the subject, contact with relatives or the treating physician, or information from medical records. Any change in the method of follow-up should be documented in the medical record. Subjects who agree to the change in follow-up are not considered to have withdrawn their consent or to have withdrawn from the trial.

Subjects who discontinue continued treatment with the investigational drug for any reason will be identified as treatment discontinuation. Subjects who discontinue treatment will be transferred to the follow-up period (refer to 7.1.).

Subjects who discontinue treatment for reasons other than objective tumor progression as assessed by imaging will continue to receive imaging every 6 weeks (±1 week) until 12 weeks after study drug initiation, then radiologic PD with imaging every 8 weeks (±1 week), plus additional imaging as defined in the study plan, or death (whichever occurs first), RECIST assessment will continue.

If a subject is determined to have PD as defined by RECIST 1.1, additional imaging studies should be performed within 4 weeks of the determination. (refer to 7.4.).

All subjects will be followed for survival until the end of the trial. Subjects who are unable to return for evaluation will be contacted by telephone as indicated in the trial schedule as an alternative.

5.7. Discontinuation of individual cases from participation in clinical trials

If any of the following criteria are met, the investigator or subinvestigator will discontinue administration of the investigational drug and the subject's participation in the clinical trial.

1) When it is judged difficult to continue the clinical trial for some reason on the subject's side, such as non-attendance or transfer to another hospital.
2) 2When the subject requests to discontinue participation in the clinical trial.

3) When the investigator or subinvestigator determines that the subject is unable to continue the clinical trial.
4) If the subject weighs less than 30 kg
5) When the investigator/divisional investigator determines that a decision to discontinue the study is necessary due to a serious violation of the study protocol, etc.

5.7.1. In case of untraceable cases

A subject is considered lost only if the subject cannot be contacted until the end of the trial and there is insufficient information to determine the subject's status at that time. Subjects who refuse to continue participation in the trial (including telephone contact) will be recorded as "withdrawing consent" rather than "untraceable". Investigators will document the means used to re-establish contact with subjects lost to follow-up throughout the duration of the trial. If the subject who was lost to follow-up is re-contacted, the subject will not be marked as "lost to follow-up" and the evaluation will resume according to the study protocol.

At the time of the OS analysis, the survival status of all subjects in the overall analysis population (FAS) and the safety analysis population will be reconfirmed. Subjects who withdrew consent and subjects classified as "possibly untraceable" will also be subject to this reconfirmation.

5.7.2. Withdrawal of consent

The subject is free to withdraw consent for this clinical trial at any time without prejudice to subsequent treatment.

Subjects who withdraw their consent will not receive any further doses of the investigational drug or follow-up as specified in the protocol. However, consent for survival follow-up will be confirmed separately. Additional tests may be performed after discontinuation to ensure subject safety.

If a subject withdraws consent, the investigator must confirm the reason and the presence or absence of adverse events.

The subject withdrawing consent shall be specifically asked about the following items regarding the details of the withdrawal of consent.

- Withdraw consent for all further participation in the clinical trial, including subsequent follow-up (e.g., telephone calls to investigate survival status).
- Withdraw consent for use of clinical trial data.
- Withdraw consent for the use of any sample.

5.7.3. Clinical investigator's decision

If the risks of participation in a clinical trial outweigh the benefits of the subject's participation in the trial, such as if a life-threatening infusion reaction or systemic infection occurs, the investigator will determine that the subject's participation in the trial cannot continue.

5.7.4. Subject weight loss

If the subject weighs less than 30 kg after enrollment, he/she will be removed from the study.

5.7.5. Other cases

If the investigator determines that the subject is unable to continue participation in the study for other reasons, such as a serious deviation from the protocol, the subject will be removed from the study.

5.8. Subject replacement

If a subject is found to be ineligible for the clinical trial prior to enrollment, the subject will not be enrolled in this trial. In such cases, the investigator will explain to the subject that enrollment in this trial is not possible.

For subjects who do not complete the study treatment for reasons other than DLT criteria during the DLT evaluation period, the investigator will seek input from the Data Monitoring Committee on whether to recruit a new subject to replace that subject.

5.9. Concomitant Restricted Drugs and Concomitant Restricted Therapy

The coordinating investigator must be informed of all medications taken from the time of screening until the end of the clinical phase (last visit) as soon as possible. All concomitant medications, including herbal medications taken during the trial are recorded in the CRF.

Restricted, prohibited, and permitted concomitant medications are listed in Tables 1 and 2.

Table1. 5.9. Concomitant Restricted Drugs and Concomitant Restricted Therapy

**Prohibited drug**	**Rules of use**
Anticancer drugs as investigational drugs other than the investigational drugs in this study	Concurrent use is prohibited during administration of investigational drugs.
mAb against CTLA-4, PD-1 or PD-L1 other than the investigational drug mAb for PD-L1 in this study	Concurrent use is prohibited during administration of investigational drugs.
Any concomitant chemotherapy, radiation therapy, immunotherapy, biologic therapy, or hormonal therapy for the treatment of cancer other than the investigational drug in this study	Any concomitant chemotherapy, radiation therapy, immunotherapy, biologic therapy, or hormonal therapy for the treatment of cancer other than the investigational drug in this study
Immunosuppressive agents such as systemic corticosteroids, methotrexate, azathioprine, or tumor necrosis factor alpha inhibitors at doses of prednisone or its equivalent greater than 10 mg/day<br>Immunosuppressive agents such as systemic corticosteroids, methotrexate, azathioprine, or tumor necrosis factor alpha inhibitors in doses greater than 10 mg/day. Immunosuppressive agents such as, but not limited to, the following	Concomitant administration or premedication is prohibited. The following exceptions are permitted<br>・ Use of immunosuppressive agents for the management of adverse events related to the investigational drug<br>・ Use in subjects allergic to contrast media<br>・ Use of inhaled, topical, and intranasal corticosteroids<br>・ Non-immunotherapy that is clinically necessary and has occurred in the subject<br>・ Temporary use of steroids is acceptable if deemed essential for the management of related events (e.g., chronic obstructive pulmonary disease, radiation therapy, nausea)
Epidermal Growth Factor Receptor Tyrosine Kinase Inhibitors（Epidermal Growth Factor Receptor Tyrosine Kinase Inhibitors (EGFR TKIs)	Prohibit concomitant use.<br>Use with caution for 90 days after the last dose of durvalumab. An increased incidence of pulmonary inflammation (in combination with a third-generation EGFR TKI) and an increased incidence of transaminases (in combination with a first-generation EGFR TKI) have been reported when durvalumab is used concomitantly.
Attenuated live vaccine	Concomitant use of the investigational drug is prohibited until 30 days after the last dose of the investigational drug.
Drugs with laxative action and herbs or natural remedies for constipation	Use with caution during participation in clinical trials.

Prohibited drug	Rules of use
Blood transfusion (red blood cell concentrate, platelets)	Concomitant use is prohibited during the DLT evaluation period.
Granulocyte colony-forming stimulating factor (G-CSF) preparation	Concomitant use is prohibited during the DLT evaluation period.

Table2. Concomitant tolerated drugs

Tolerated Drugs	Rules of use
Concomitant medications or treatments (e.g., acetaminophen or diphenhydramine) as deemed necessary for appropriate prophylactic or symptomatic treatment. However, drugs included in the "Prohibited Drugs" section above are excluded.	Administer according to the prescription of the investigator, etc.
Best supportive care (including antimicrobials, nutritional support, correction of metabolic disturbances, optimal symptom control and pain management [e.g., palliative radiation therapy for non-target lesions])	For all subjects, use when necessary.
Inactivated viruses such as influenza vaccines	Can be inoculated (e.g. with vaccine)

5.10. Follow-up treatment

Post-treatment after completion or discontinuation of the clinical trial is not specified.

5.11. After discontinuation of this clinical trial

Subjects who discontinue the clinical trial will be subjected to necessary examination and observation, and appropriate measures will be taken as necessary, until it can be medically determined that the subject can be discharged or transferred to a hospital.

6. Clinical trial treatment

6.1. Durvalumab and tremelimumab

Refer to the investigator's brochure for details and handling of the investigational drug. The following are the investigational drugs to be used in this clinical trial.

6.1.1. Durvalumab

Durvalumab (MEDI4736) will be supplied by AstraZeneca as a 500-mg vial solution for infusion after dilution. The solution contains 50 mg/mL durvalumab, 26 mM histidine/histidine hydrochloride, 275 mM trehalose dihydrate, and 0.02% weight/volume (w/v) polysorbate 80; it has a pH of 6.0 and density of 1.054 g/mL. The nominal fill volume is 10.0 mL.

Durvalumab is a sterile, clear to opalescent, colorless to slightly yellow solution, free from visible particles.

Investigational product vials are stored at 2°C to 8°C (36°F to 46°F) and must not be frozen. Investigational products should be kept in original packaging until use to prevent prolonged light exposure.

6.1.1.1. Preparation of Durvalumab Dose by Infusion Bag

Each dose of durvalumab must be prepared by aseptic manipulation by the investigator or other investigational drug administrator designated by the site. The time between the puncture of the vial and the start of administration of Durvalumab must not exceed the following

- 2°C to 8°C for 24 hours
- 4 hours at room temperature
- Dosing solutions must be brought to room temperature before administration.

Dose 1,500 mg of durvalumab is administered with an infusion bag containing 0.9% saline or 5% dextrose to achieve a final concentration of 1 to 20 mg/mL of durvalumab and an intravenous administration set with a 0.2 μm or 0.22 μm filter. Add 1,500 mg of durvalumab (i.e., 30.0 mL of durvalumab) to the infusion bag. The infusion bag should be selected to achieve a final concentration within 1 to 20 mg/mL. Gently invert and mix the infusion bag until the administered fluid in the bag is uniform. The standard infusion time is 1 hour (±5 minutes). If the infusion is interrupted, the total infusion time should not exceed 8 hours at room temperature. No other drugs should be administered simultaneously in the same IV line.

After the contents of the infusion bag have been completely administered, flush the IV line with an IV diluent equal to the priming volume of the IV set used, or complete the infusion according to the provider's policy to ensure that the full volume is administered. If the line is not flushed, document this in the record. If the preparation time or infusion time limit is exceeded, a new vial must be used to prepare a new dose solution. Since durvalumab does not contain preservatives, unused preparation solutions must be discarded.

The preparation should be made in accordance with the “Procedures for the Administration of Investigational Drugs” for this clinical trial.

6.1.2. Tremelimumab

Tremelimumab will be supplied by AstraZeneca either as a 400-mg or a 25-mg vial solution for infusion after dilution. The solution contains 20 mg/mL tremelimumab, 20 mM histidine/histidine hydrochloride, 222 mM trehalose dihydrate, 0.27 mM disodium edetate dihydrate, and 0.02% weight/volume (w/v) polysorbate 80; it has a pH of 5.5 and density of 1.034 g/mL. The nominal fill volume is 20.0 mL for the 400-mg vial and 1.25 mL for the 25-mg vial.

Tremelimumab is a sterile, clear to opalescent, colorless to slightly yellow solution, free from or practically free from visible particles.

Investigational product vials are stored at 2°C to 8°C (36°F to 46°F) and must not be frozen.

Investigational products should be kept in original container packaging until use to prevent prolonged light exposure.

6.1.2.1. Preparation of Tremelimumab Dose by Infusion Bag

Each dose of tremelimumab must be prepared by aseptic manipulation by the investigator or the investigation drug manager designated by the site. The time from vial puncture to administration must not exceed

- 2°2°C to 8°C for 24 hours
- 4 hours at room temperature
- Doing solutions must be brought to room temperature before administration.

Tremelimumab is administered in an infusion bag containing 0.9% saline or 5% dextrose to achieve a final concentration of 0.10-10 mg/mL of tremelimumab, using an intravenous administration set with a 0.2 μm or 0.22 μm filter.

Add 300 mg of tremelimumab (i.e., 15.0 mL) to the infusion bag. Gently invert and mix the infusion bag until the dosing solution in the bag is homogeneous. The standard infusion time is 1 hour (±5 minutes); any infusion time less than 55 minutes will be considered a deviation from the study protocol. If the infusion is interrupted, the total infusion time should not exceed 8 hours at room temperature. No other drugs should be administered simultaneously in the same IV line. After the contents of the infusion bag have been completely administered, flush the IV line with an IV diluent equal to the priming volume of the IV set used, or complete the infusion according to the site's policy to ensure that the full volume is administered. If the line is not flushed, document this. If the preparation time or infusion time limit is exceeded, a new vial must be used to prepare a new dose solution. Since tremelimumab does not contain preservatives, unused preparation solutions must be discarded. The preparation should be made in accordance with the "Protocol for the Administration of Investigational Medicinal Products" for this clinical trial.

6.2. Control Drugs

There is no control drug in this study.

6.3. Monitoring during administration

During and after the infusion, the subject's condition should be monitored by assessment of vital signs at the times specified in the study protocol.

If an infusion-related reaction of grade 2 or less is observed, the infusion rate of the study drug may be reduced by 50% or discontinued until the event is resolved, and the infusion may be restarted at 50% of the initial infusion rate until the infusion is completed. Subjects who experience an infusion-related reaction of grade 2 or less may receive subsequent infusions at 50% of the initial rate. Acetaminophen and/or antihistamines (e.g., diphenhydramine) or equivalent drugs according to institutional standards may be administered at the investigator's discretion. If infusion-related reactions are grade 3 or greater, the investigational drug should be discontinued. The standard infusion duration is 1 hour, but if interrupted, the infusion should not exceed 8 hours at room temperature. Refer to the Toxicology and Management Guidelines in the protocol appendix for management of subjects who experience an infusion-related reaction.

As with other antibodies, allergic reactions to dose administration may occur. Appropriate drugs and medical devices to treat acute anaphylactic reactions must be readily available, and investigators must be trained to recognize and treat anaphylaxis. The site must have immediate access to an emergency resuscitation team and medical equipment, and the ability to admit subjects to the intensive care unit if necessary.

6.4. Management of investigational drugs

1) The investigator coordinator will deliver the investigational drug to the investigator of Chiba University Hospital in accordance with the agreement with the investigational drug

provider.

2) The investigator coordinator will properly manage the investigational drug in accordance with the protocol provided by the investigator through the site director.
3) The investigator shall prepare a document explaining the storage conditions, expiration date, and other handling methods of the investigational product and deliver it to the site manager, investigators collaborators, and investigational product manager.

6.5. Disposal of unused investigational drugs

1) After the investigational monitor checks the inventory and obtains the investigator's approval, the investigational drug manager discards used, unused, expired, or damaged investigational drugs and empty containers.
2) The investigational drug manager shall dispose of the investigational drug in accordance with the guidelines for disposal of pharmaceuticals.

6.6. Packaging and labeling of investigational drugs

The label should indicate that the product is for investigational use, the name, title and address of the coordinating investigator (representative), chemical name, volume, serial number, storage method and expiration date of the investigational drug. The label should appear on the package insert.

Labels for investigational new drugs shall be prepared in accordance with “Good Manufacturing Practice (GMP)” and GCP ordinances. The investigational drug label should be written in Japanese.

Investigational drug: Durvalumab (genetical recombination) (MEDI4736) + Tremelimumab (genetical recombination)

The drug name on the durvalumab label shall be “MEDI4736” or “Durvalumab (MEDI4736)”.

6.7. Carbon ion radiotherapy

Information on Carbon ion radotherapy

- Name of medical device: Cardon ion radiotherapy Device
- Indications: Treatment of solid tumors
- Model Number: CI-1000S
- PMDA approval number: 22800BZX00096000
- Manufacturing facility: Toshiba Energy Systems&Solutions Corporation

Carbon Ion Therapy will be performed at the Quantum Science and Technology Agency QST Hospital, and irradiation will be performed for four consecutive days between Day 8 and Day 14 of Cycle 1 as per the schedule in Section 7.1. (excluding holidays)

Carbon ion radiotherapy device will be treated as an device used for this clinical trial.

Dose prescription and fractionations will be 60Gy (RBE) / 4Fr / 1week for CIRT. RBE calculation is done by modified microdosimetric kinetic model (Inaniwa et al. Phys Med Biol. 2010). Pencil beam scanning technique will be used. The pencil beam covers the PTV voxel by voxel in successive layers. An optimization function drives the dose distribution in each treatment spot to reach the desired target coverage and organs at risk sparing. If a coil or other device implanted in the previous treatment is considered to be substitutable as a marker for Carbon ion radiotherapy, the marker may be omitted after consultation with the investigators.

All cases will undergo fiducial marker insertion prior to treatment preparation. Fiducial markers may be implanted under ultrasonography/fluoroscopy surveillance percutaneously or transarterialy.

For cases treated in an orthogonal fixed beam room, immobilization will be achieved with a relatively thick shell (3-mm thickness) made of a low-temperature thermoplastic and hydraulic

urethane resin or vacuum-formed cushion to allow a range of beam angles by rotating the treatment table. However, a thinner shell may be used if a rotating gantry is available. In either case, the shell device is fixed by tapping to the table bottom, with tightening or loosening adjustment as required.

A 4D-CT simulation is required to allow for assessment of tumor motion. Fasting for 3 to 8 hours prior to simulation is required to control stomach/duodenum volume. Simulation CTs should be done with a CT slice thickness no greater than 3 mm. The 4D dataset is separated into 10 separate breathing phase bins. The simulation scan used for planning should NOT be performed with intravenous contrast; simulation CT after this planning CT may include contrast (for anatomic information).

Target lesion of the particle therapy will be focused on intrahepatic nodule with MVI. A 5mm margin will be taken as a clinical target volume margin for the feeding nodule, and 1cm margin alongside the vessel for the MVI lesion. Internal motion will be compensated according to 4D-CT movement assessment. Inter-fractional margin will be set to 3mm and combined with internal motion compensation forming a field specific planning treatment volume.

Dose constraints for risk organs are set as follows

- GI tract: D2cm3 ≤ 30Gy (RBE)
- Spinal cord: Dmax ≤ 25Gy (RBE)
- Remnant liver volume (liver volume receiving 30Gy (RBE) or less): 500cm3

In addition, the following liver volume information is collected in conjunction with the remaining liver volume

- Liver V5 Gy (RBE)
- Liver V20 Gy（RBE）

Confirmation of patient positioning is confirmed by orthogonal X-ray images. A maximum displacement of 3 mm in all directions is allowed between the reference and treatment images and is achieved by movement of the couch. Respiratory gating during treatment is mandatory. External respiratory surrogate systems or fluoroscopic tracking may be used for respiratory motion detection.

6.8. subject inclusion

In this “modified 3 + 3 design”, the first three subjects will be enrolled in cohort A. If no DLT is observed in any of these subjects, the trial will enroll additional subjects in Cohort B, who will also receive tremelimumab as follows.

If one subject develops DLT in any cohort, three additional subjects will be enrolled in that same cohort. The development of two or more DLTs in Cohort A will mean that the entire trial will be terminated, and two or more DLTs in Cohort B will indicate that the MTD has been exceeded and the regime in Cohort B will be discontinued. In that case, up to a total of 15 additional subjects will be enrolled in Cohort A.

Schematic of subject incorporation

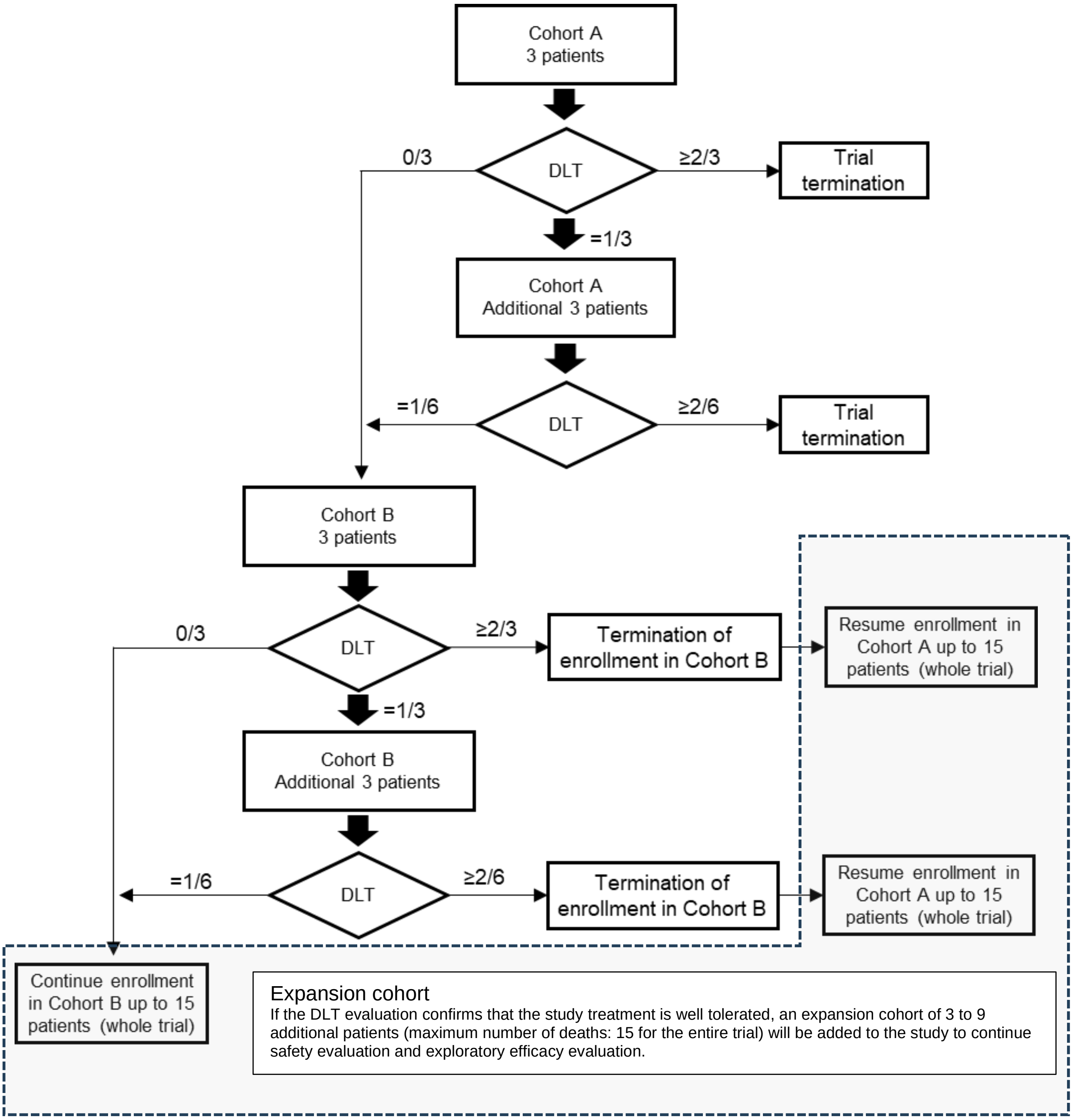

6.9. Definition of Dose-Limiting Toxicity (DLT)

During the 42-day period from the start of study drug administration (Cycle 1/DLT evaluation period), the following toxicities associated with the study treatment are considered DLTs: Grade determination follows CTCAE ver. 5.0.

Dose-limiting toxicity (DLT) will be assessed during the DLT evaluation period of this study, which will be 42 days from the time of the first dose on Cycle 1, Day 1. The severity of DLT will follow the guidelines described in the CTCAE ver. 5.0.

DLT is defined as the occurrence of an adverse event (AE) that is at least potentially related to the investigational drug or regimen (IR), with two exceptions: a Grade of vitiligo or alopecia is not a DLT. An AE that is at least potentially related to a regimen containing durvalumab and/or tremelimumab will be evaluated as a DLT if it meets one of the following criteria.

If a patient initiates heavy ion therapy but does not complete heavy ion therapy within the allowed time due to an adverse event that cannot be excluded as being causally related to durvalumab, tremelimumab, or heavy ion therapy, such adverse event will be considered a

DLT.

- **Hematologic toxicity:**
  - Grade ≥3 neutropenia complicated by fever >38.3°C
  - Grade 4 neutropenia (lasting more than 7 days)
  - Grade ≥3 thrombocytopenia with significant bleeding
  - Grade 4 thrombocytopenia (regardless of duration)
  - Grade 4 anemia (regardless of duration)
  -
- **Non-hematologic toxicity:**
  - Any Grade 4 non-immune-mediated AE
  - Any Grade 4 immune-mediated AE, excluding endocrinopathies
  - Any Grade 3 non-immune mediated AE that does not resolve to ≤Grade 1 or baseline within 30 days with optimal medical management
  - Any Grade 3 immune-mediated AE – excluding diarrhea/colitis, pneumonitis, hepatitis, rash, neurotoxicity, myocarditis, myositis/polymyositis, endocrinopathies and nephritis – that does not resolve to ≤Grade 1 or baseline within 30 days after onset of the event despite optimal medical management including systemic corticosteroids
  - Grade 3 diarrhea or colitis that does not resolve to ≤Grade 1 within 14 days [both immune- and non-immune-mediated indicated here; the same is the case if not specified in remaining bullet points below]
  - Grade 3 noninfectious pneumonitis
  - Grade 2 noninfectious pneumonitis that does not resolve to ≤Grade 1 within 3 days of the initiation of maximal supportive care
  - Aspartate aminotransferase (AST) or alanine aminotransferase (ALT) ≥3×ULN with concurrent increase in total bilirubin (TBL) ≥2×ULN without evidence of cholestasis or alternative explanations (e.g., viral hepatitis, disease progression in the liver; i.e., “Hy’s Law”)
  - ALT or AST >8×ULN or TBL>5×ULN
  - Grade 3 immune-mediated rash that does not resolve to ≤Grade 1 or baseline within 30 days
  - Grade 2 rash covering > 30% BSA that does not resolve to ≤Grade 1 or baseline within 30 days
  - Any grade of immune-mediated rash with bullous formation
  - Grade 3 immune-mediated neurotoxicity (excluding Guillain-Barre and myasthenia gravis) that does not resolve to ≤Grade 1 within 30 days
  - Grade 2 or 3 immune-mediated peripheral neuromotor syndrome (such as Guillain-Barre and myasthenia gravis) that does not resolve to ≤Grade 1 within 30 days or that exhibits signs of respiratory insufficiency or autonomic instability
  - Grade 3 immune-mediated myocarditis
  - Any symptomatic immune-mediated myocarditis that does not become asymptomatic within 3 days of initiating optimal medical management including systemic corticosteroids
  - Grade 2 or 3 immune-mediated myositis/polymyositis that does not resolve to Grade ≤1 within 30 days of initiating optimal medical management including systemic corticosteroids or that exhibits signs of respiratory insufficiency regardless of optimal medical management
  - Immune-mediated increase in creatinine >3×ULN, or >3×baseline for patients with a baseline creatinine elevated above ULN
  - Transfusion (Red cell concentrate, Platelet), or the use of G-CSF during DLT period

The DLT evaluation period will be from the time of the first dose of study drug/IR until 42 days post-dose. The first dose of durvalumab in Cycle 2 will be administered after the safety of Cycle 1

is confirmed.

The first treatment-related toxicity that occurs during the DLT period must be followed up to determine if the event qualifies as a DLT as defined in the DLT criteria above.

An immune-related adverse event is defined as an immune (inflammatory) adverse event without a definite other etiology. If an immune-related adverse event is suspected but there are no significant laboratory findings, a repeat laboratory test should be performed before a determination of DLT is made. Subjects who do not complete the DLT evaluation period for reasons other than DLT will not be considered DLT evaluable cases, and DLT will be evaluated after supplementation. The investigator will ask the Independent Data Monitoring Committee for an opinion on the supplementation decision.

Subjects will be administered the first dose of the study drug after hospitalization at Chiba University Hospital. After administration of the investigational drug, the subject will continue to be closely monitored in the hospital and will be transferred to the QST Hospital only for heavy ion therapy, taking into consideration the subject's condition and the means of transportation (taxi, private ambulance, etc.). After completion of heavy particle therapy, the patient should remain in the hospital for at least 7 days. During the transition to outpatient treatment, the investigator will perform the tests specified in 7.2.2 and confirm the safety of the treatment before allowing the patient to continue treatment as an outpatient.This inspection may be combined with the immediately preceding regulatory inspection.In addition, the investigator should be able to contact the site and the medical facility near the home in the event of an adverse event and establish a system to promptly contact the subject in an emergency.

6.10. Toxicity Management

Guidelines for the management of immune-mediated reactions, infusion reactions, and non-immune-mediated reactions to durvalumab are provided in the Durvalumab/Tremelimumab Toxicity Management Guidelines (TMGs).

Appropriate efforts should be made to thoroughly evaluate the subject and rule out neoplastic, infectious, metabolic, toxic, or other etiologies of imAE. Serologic, immunologic, and histologic (biopsy) data should be used to support the diagnosis of imAE, if appropriate. In the absence of a clear alternative etiology, the possibility of an immune-related etiology should be considered. In addition, there are situations in which durvalumab and tremelimumab should be discontinued (see Toxicity Management Guidelines). Dose reductions are not permitted. In case of doubt, consult the investigator. All toxicities will be evaluated according to CTCAE ver. 5.0.

6.11. Restrictions during the clinical trial

6.11.1. Restrictions during the clinical trial

The following restrictions apply during and before and after a given period of time while undergoing investigational treatment.

About Women of Childbearing Potential

Female subjects of childbearing potential who are not abstinent and who plan to have sex with an unprotected male partner must use at least one effective method of contraception from the time of screening until the drug treatment and drug discontinuation period (180 days after the last dose of durvalumab + tremelimumab combination therapy). Unprotected male partners of female subjects of childbearing potential must use a male condom and spermicide during this period. Discussion of stopping contraception after this time should be discussed with the family physician. Temporary abstinence, the rhythm method, and external ejaculation are not acceptable methods of contraception. Females should also not breast-feed during this period.

About men with female partners of childbearing potential

For non-contraceptive male subjects who are unprotected and plan to have sex with a female

partner of childbearing potential, the use of a male condom plus spermicide is mandatory from screening through the entire drug treatment and drug washout period (180 days after the last dose of durvalumab + tremelimumab combination therapy). However, periodic abstinence, rhythm and withdrawal methods are not acceptable methods of contraception. Male subjects will refrain from donating sperm during this period.

Female partners of male subjects (of childbearing potential) should also use highly effective contraceptive methods during this period.

Note: Pregnant women are defined as women who have not undergone sterilization (i.e., bilateral oophorectomy, bilateral oophorectomy, or total hysterectomy) or as premenopausal.

A woman is considered postmenopausal if she has been amenorrheic for 12 months without another medical cause. The following age-specific requirements apply

- Women under age 50 are considered postmenopausal if they have been amenorrheic for at least 12 months after discontinuation of exogenous hormone therapy and have luteinizing hormone and follicle stimulating hormone levels in the postmenopausal range of the institution.
- Women over age 50 are considered postmenopausal if they have been amenorrheic for at least 12 months after discontinuation of all exogenous hormone therapy, if they had radiation-induced menopause more than 1 year before their last menstrual period, and if they had chemotherapy-induced menopause more than 1 year before their last menstrual period.

A highly effective contraceptive method is defined as having a low failure rate (i.e., less than 1% per year) when used consistently and correctly.

6.12. Clinical Trial Procedures

The RECIST evaluation date must be performed as scheduled, regardless of any dosing delays. All other scheduled assessments must be performed at the start of the dosing cycle, and all laboratory and other tests required for dosing must be performed at least 3 days prior to dosing.

Subjects may be allowed to delay dosing under certain circumstances, as described below.

- With the judgment of the principal investigator and subinvestigator,dosing may be delayed for either immune- or non-immune-related AEs in accordance with toxicity management guidelines.
- If dosing must be delayed for reasons other than treatment-related toxicities, dosing should be resumed as soon as possible.
- The dosing interval may be shortened as clinically appropriate to gradually match the treatment cycle to the tumor response plan (RECIST). Based on the half-life of durvalumab and tremelimumab, the interval between two consecutive doses should not be less than 22 days (for durvalumab and tremelimumab, see the current investigator's brochure).

7. OBSERVATION, EXAMINATION, AND ASSESSMENT, METHODS, AND TIMING OF IMPLEMENTATION

7.1. Implementation Schedule and Procedures

Cycle	Screening period	First tumor biopsy ※only in consented patients		DLT evaluation period							Second tumor biopsy ※only in consented patients	Durvalumab q4W dosing period					ST	Follow up period（28 days after the last administration date）	Safety information collection（90 days after the last administration date）
				Cycle 1				Cycle 2 *15				Cycle 3	Cycle43	Cycle 5	Cycle 6	Cycle 6< until PD			
Cycle Day	D -28 ～D -1	D -28～D -1		1	8 to 14	15	22	1	8	14	DLT評価終了後	1*1	1	1	1	1	—	—	—
Allowable period（Day）				—	±3	±3	±3	±3	±3	±3		—	±3	±3	±3	±3	+14	+14	+14
Informed Consent ／ Subject backgroud information／ Review of eligibility	●*2																		
Enrollment			●*2																
Durvalumab administration (cohort A and B)				●				●				●	●	●	●	●			
Tremelimumab administration (cohort B)*3				●															
Tumor biopsy		●									●								
CIRT					4days between Day8 and Day14*4														
Fiducial marker insertion*5*6		●																	
Fixation, simulation CT (for CIRT)*5	●																		
Weight*7	●			●	●	●	●	●	●	●		●	●	●	●	●	●	●	
Height	●																		
Physical exam	●			●	●	●	●	●	●	●		●	●	●	●	●	●	●	
Physical exam（Specific site based on case）	●			●	●	●	●	●	●	●		●	●	●	●	●	●	●	
Vital signs	●			●	●	●	●	●	●	●		●	●	●	●	●	●	●	
ECOG PS	●			●	●	●	●	●	●	●		●	●	●	●	●	●	●	
Clinical Chemistry／Hematology*8	●			●	●	●	●	●	●	●		●	●	●	●	●	●	●	
Coagulation	●			●	●	●	●	●	●	●		●	●	●	●	●	●	●	
Urinalysis	●			●								●	●	●	●	●	●	●	
ECG*9	●			(●)								(●)	(●)	(●)	(●)	(●)	(●)	(●)	
Hepatitis serology *10	●			(●)				(●)				(●)	(●)	(●)	(●)	(●)	(●)		
HIVtests	●																		
TSH、fT3、fT4 *11	●			●	●	●	●	●	●	●		●	●	●	●	●	●	●	
Pregnancy test *12	●			(●)				(●)				(●)	(●)	(●)	(●)	(●)			
Chest X ray	●																●	●	
Assesment of Child-Pugh score	●			●				●				●	●	●	●	●	●	●	
CT/MRI*13	●			← Every 6 weeks (±1 week) for the first 12 weeks from Cycle1 day1, and every 8 weeks (±1 week) thereafter until PD. →														●	
Tumor marker（AFP、PIVKA-II）	●							●				●	●	●	●	●			
Confirmation of survival																		●	
Cocmitant medication	←																	→	
Assessment of AE/SAE*14	←																		→

*1. The baseline for the durvalumab Q4W dosing period will be the first day of the third cycle.
*2. To be performed after completion of all screening tests and prior to administration of Cycle1Day1.Whenever possible, minimize the time between enrollment and Cycle1Day1.
*3. In combination therapy, tremelimumab should be administered first, and durvalumab infusion should be started approximately 1 hour (maximum 2 hours) after tremelimumab administration is completed.
*4. Excluding holidays.
*5. To be administered to subjects who are eligible by other screening tests.
*6. If a coil or other device implanted in the previous treatment is considered to be substitutable as a marker for Carbon ion radiotherapy, the marker may be omitted after consultation with the investigators.
*7. Weight will be measured along with vital signs at the visit.
*8. Serum or plasma biochemical tests (including LFT monitoring) and blood tests may be performed more frequently if clinically indicated.
If screening biochemical and hematological evaluations were performed no more than 3 days prior to Day 1 (the first infusion day), they need not be performed again on Day 1.
Results of LFTs, electrolytes, complete blood count, and creatinine must be obtained prior to the start of infusion (within 3 days) and confirmed by the attending physician or investigator prior to administration.
*9. At a screening period,if clinically significant abnormalities are detected, two additional ECG measurements should be performed.
*10. If HBs-Ag, HBs-Ab, or HBc-Ab is positive, HBV-DNA should be measured (before and every 4 weeks after administration).
If HCV antibodies are positive, measure HCV-RNA (pre-test).
*11. Free T3 or free T4 should be measured only if TSH is abnormal or endocrine system-related AEs are clinically suspected. If TSH is measured up to 14 days prior to the first dose, it need not be measured again on Day 1.
*12. For women of childbearing potential only. For women of childbearing potential, a pregnancy test should be performed every 4 weeks starting 7 days prior to the first dose. Pregnancy tests can be performed on Day 1, but results must be confirmed by the treating physician or investigator before administration is initiated.
*13. RECIST evaluation is performed with CT (preferred) or MRI imaging of the chest, abdomen (including liver and adrenal glands), and pelvis IV contrast preferred. Imaging of the pelvis is recommended only if there is a possibility of primary or metastatic disease in the pelvic region. Additional anatomic imaging should be performed based on the individual subject's signs and symptoms at baseline and at follow-up. Baseline assessments should be performed no later than 28 days prior to the start of study drug administration for each cohort and should be performed as close as possible to the start of study drug or prior to the start of study drug if possible. Confirmatory testing should be performed within 4 weeks of the prior PD evaluation, preferably at the next scheduled imaging visit (provided there is no clinically significant deterioration). If an unscheduled evaluation is performed and the subject has not progressed, every effort should be made to perform a subsequent evaluation at the next scheduled visit.
*14. AEs and SAEs should be collected from the time of the first dose of study drug until the follow-up date 28 days after discoutinuation of study drug. However, AE and SAE outcomes should be collected until the end of the safety information collection period (90 days after the discontinuation of study drug) or until the start of alternative anticancer therapy. In addition, events occurring after the 28-day follow-up period after the discoutinuation of the study drug and considered to be attributable to delayed toxicity to the study drug will be collected as AEs or SAEs until the end of the safety information collection period or until the initiation of alternative anticancer therapy.
*15. *n the Expansion cohort, patients will be moved to Cycle 2 after the end of Cycle 1.
*16. After completion of heavy particle therapy, the patient should remain in the hospital for at least 7 days. During the transition to outpatient treatment, the investigator will perform the tests specified in 7.2.2 and confirm the safety of the treatment before allowing the patient to continue treatment as an outpatient. This inspection may be combined with the immediately preceding regulatory inspection.

Note: All assessments on the treatment day shall be performed prior to infusion unless otherwise indicated.
ECG Electrocardiogram; LFT Liver function tests; T3 Triiodothyronine; T4 Thyroxine; TSH Thyroid stimulating hormone.

7.2. Observation, tests and assessment

7.2.1. Screening period

The investigator or sub-investigator will perform the following screening tests and enroll subjects who meet the selection criteria and do not violate the exclusion criteria. The tests will be performed after consent is obtained and between 28 days before the start of the investigational drug and the day before the start of the investigational drug. Test items will be as described below. However, test results obtained prior to consent as part of routine medical care using procedures similar to those used in this study may be used as screening tests if they fall within the 28-day screening period.

- Informed Consent
- Subject background information*
- Review of eligibility criteria
- Complete physical exam
- ECOG Performance Status
- Vital signs**, weight and height
- Chest X ray
- 12-lead ECG (in triplicate [2-5 minutes apart])
- Assessment of Child-Pugh score
- Imaging by CT/MRI, if applicable to study
- Clinical laboratory tests for:
    - Clinical Chemistry (see Table 5)
    - Hematology (see Table 4)
    - TSH, fT3, fT4
    - Coagulation (PT, APTT, PT-INR)
    - Creatinine Clearance
    - Pregnancy test (for women of childbearing potential only)
    - Hepatitis serology（HBs antigen, HBs antibody, HBc antibody, and HCV antibody）
    - HIV test
    - Urinalysis
    - Tumor marker (AFP, PIVKA-II)
- Fiducial marker insertion
- Fixation, simulation CT (for CIRT)
- Concomitant medication

* **Subject background information and medical history**

Subject identification code number, race, sex, age at obtaining IC, medical history, concurrent diseases, alcohol consumption, smoking history, and information for HCC with initial diagnosis, pathological diagnosis and past treatment.

** **Vital signs**

Vital signs will be measured at every visit and will include assessments of systolic and diastolic BP, temperature, and HR. Systolic and diastolic BPs will be documented in mmHg. Temperature will be obtained in degrees Celsius. HR will be documented in beats per minute. Generally, each patient will have blood pressure tested in the same arm. Measurement device and measurement time will not be indicated.

7.2.2. DLT assessment period

Day1

- Durvalumab administration (cohort A and B)
- Tremelimumab administration (cohort B）
- Complete physical exam
- ECOG Performance Status
- Vitals signs and weight
- 12-lead ECG (if necessary)
- Assessment of Child-Pugh score
- Clinical laboratory tests for:
  - Clinical chemistry
  - Hematology
  - TSH、fT3、fT4
  - Coagulation（PT、APTT、PT-INR）
  - Creatinine Clearence
  - Pregnancy test (women of childbearing potential only)
  - Urinalysis
- Assessment of AE/SAE
- Confirmation of concomitant therapy

Day 8-14

- CIRT (60 Gy (RBE) / 4 Fr) (performed at QST hospital)
- Complete physical exam
- ECOG Performance Status
- Vitals signs and weight
- Assessment of Child-Pugh score
- Clinical laboratory tests for:
  - Clinical chemistry
  - Hematology
  - TSH、fT3、fT4
  - Coagulation（PT、APTT、PT-INR）
  - Creatinine Clearence
- Assessment of AE/SAE
- Confirmation of concomitant therapy

Day 15

- Complete physical exam
- ECOG Performance Status
- Vitals signs and weight
- Assessment of Child-Pugh score
- Clinical laboratory tests for:
  - Clinical chemistry
  - Hematology
  - TSH、fT3、fT4
  - Coagulation（PT、APTT、PT-INR）
  - Creatinine Clearence

- Assessment of AE/SAE
- Confirmation of concomitant therapy

Day 22

- Complete physical exam
- ECOG Performance Status
- Vitals signs and weight
- Assessment of Child-Pugh score
- Clinical laboratory tests for:
  - Clinical chemistry
  - Hematology
  - TSH、fT3、fT4
  - Coagulation（PT、APTT、PT-INR）
  - Creatinine Clearence
- Assessment of AE/SAE
- Confirmation of concomitant therapy

Day 29（Cycle 2 Day1）

- Durvalumab administration (cohort A and B)
- Tremelimumab administration (cohort B）
- Complete physical exam
- ECOG Performance Status
- Vitals signs and weight
- 12-lead ECG (if necessary)
- Assessment of Child-Pugh score
- Clinical laboratory tests for:
  - Clinical chemistry
  - Hematology
  - TSH、fT3、fT4
  - Coagulation（PT、APTT、PT-INR）
  - Creatinine Clearence
  - Pregnancy test (women of childbearing potential only)
  - Tumor maker (AFP, PIVKA-II)
- Assessment of AE/SAE
- Confirmation of concomitant therapy

Day 36（Cycle2 Day8）

- Complete physical exam
- ECOG Performance Status
- Vitals signs and weight
- Assessment of Child-Pugh score
- Clinical laboratory tests for:
  - Clinical chemistry
  - Hematology
  - TSH、fT3、fT4
  - Coagulation（PT、APTT、PT-INR）

- Creatinine Clearence
- Assessment of AE/SAE
- Confirmation of concomitant therapy

Day 43（Cycle 2 Day14）

- Complete physical exam
- ECOG Performance Status
- Vitals signs and weight
- Assessment of Child-Pugh score
- Clinical laboratory tests for:
  - Clinical chemistry
  - Hematology
  - TSH、fT3、fT4
  - Coagulation（PT、APTT、PT-INR）
  - Creatinine Clearence
- Assessment of AE/SAE
- Confirmation of concomitant therapy

At discharge during the DLT evaluation period

- Complete physical exam
- ECOG Performance Status
- Vitals signs and weight
- Assessment of Child-Pugh score
- Clinical laboratory tests for:
  - Clinical chemistry
  - Hematology
  - TSH、fT3、fT4
  - Coagulation（PT、APTT、PT-INR）
  - Creatinine Clearence
- Assessment of AE/SAE
- Confirmation of concomitant therapy

7.2.3. Durvalumab q4W dosing period

At Each cycle Day1

- Durvalumab administration (cohort A and B)
- Complete physical exam
- ECOG Performance Status
- Vitals signs and weight
- 12-lead ECG (if necessary)
- Assessment of Child-Pugh score
- Clinical laboratory tests for:
  - Clinical chemistry
  - Hematology
  - TSH、fT3、fT4
  - Coagulation（PT、APTT、PT-INR）
  - Creatinine Clearence
  - Pregnancy test (women of childbearing potential only)

- Tumor maker (AFP, PIVKA-II)

- Assessment of AE/SAE
- Confirmation of concomitant therapy

7.2.4. At the time of discontinuation of investigational drug administration

- Complete physical exam
- ECOG Performance Status
- Vitals signs and weight
- 12-lead ECG (if necessary)
- Assessment of Child-Pugh score
- Clinical laboratory tests for:
  - Clinical chemistry
  - Hematology
  - TSH、fT3、fT4
  - Coagulation（PT、APTT、PT-INR）
  - Creatinine Clearence
  - Tumor maker (AFP, PIVKA-II)
- Assessment of AE/SAE
- Confirmation of concomitant therapy

7.2.5. Follow up period

- Complete physical exam
- ECOG Performance Status
- Vitals signs and weight
- 12-lead ECG (if necessary)
- Assessment of Child-Pugh score
- Clinical laboratory tests for:
  - Clinical chemistry
  - Hematology
  - TSH、fT3、fT4
  - Coagulation（PT、APTT、PT-INR）
  - Creatinine Clearence
  - Urinalysis
  - Tumor maker (AFP, PIVKA-II)
- confirmation of survival
- Assessment of AE/SAE
- Confirmation of concomitant therapy

7.3. Biological sampling procedures

7.3.1. Guideline for blood sampling volume

The total volume of blood to be drawn from each subject in this study is as follows

Amount of blood to be collected from each subject

Assesment		Sample volume (mL) / visit
**Safety**	**Clinical Chemistry Tests**	10

Amount of blood to be collected from each subject

Assesment	Sample volume (mL) / visit
**Hematology Tests**	10
**Total**	20

7.3.2. Blood samples for archiving

When consent is obtained from the subject for storage of blood specimens, residual blood specimens designated for biochemistry and blood tests will be stored at the Department of Gastroenterology, Chiba University Hospital. Blood specimens will be handled in such a way as to prevent leakage, confusion, theft, or loss of personal information by anonymization with an identification code. Blood specimens will be stored for a period not exceeding 20 years after the completion of the clinical trial, after which all specimens will be properly disposed of. If a subject withdraws consent for specimen storage, the specimens will be destroyed and this will be documented. In addition, if specimens are to be used in future research, a new research protocol must document the use of this specimen and be submitted to the IRB for approval.

7.3.3. Hepatic tumor biopsy sample

In this study, a percutaneous liver biopsy/liver tumor biopsy will be performed before the first dose and between 43 and 56 days after the first dose, if the subject is deemed safe for such a biopsy/liver tumor biopsy by the investigator or sub-investigator and if the subject consents. The tissue samples (tumor and non-tumor) obtained will be used for exploratory studies (refer to 9.3.). At that time, the tissue samples will be handled to prevent disclosure, mix-up, theft, or loss of personal information by anonymization with an identification code. The investigator or sub-investigator may discontinue the percutaneous liver biopsy or liver tumor biopsy for the safety of the subject. Failure to perform a percutaneous liver biopsy/liver tumor biopsy at the discretion of the investigator or sub-investigator, or failure to perform a percutaneous liver biopsy/liver tumor biopsy without the consent of the subject, will not preclude enrollment or dosing in this study. Tissue specimens will be retained for a maximum of 20 years after completion of the study, after which all specimens will be properly disposed of. If a subject withdraws consent for specimen storage, the specimen will be discarded and this will be documented. In addition, if specimens are to be used in future research, the use of that specimen must be documented in a new research protocol and submitted to the IRB for approval.

7.4. Asessment of efficiency

(Tumor evaluation by mRECIST in addition to RECIST ver. 1.1.)
The following are guidelines for confirming image evaluations to assess efficacy.

- Imaging evaluations are performed for subject management and treatment decisions.
- Image evaluation is performed using RECIST ver. 1.1.
- In the absence of clinically evident evidence of disease progression, the patient should be re-evaluated after disease progression (PD) is determined by RECSIST ver. 1.1 in order to distinguish between immune checkpoint inhibitor-induced pseudo progression and true disease progression (this is to reduce the risk of study termination due to incorrect evaluation by the investigator/participating physician). (This is to reduce the risk of termination of the study due to incorrect assessment by the investigator/associated investigator.)

The definition of objective disease progression (definite PD) is as follows

1) The presence of clinically evident evidence of disease progression and disease progression (PD) according to RECIST ver. 1.1 is confirmed as objective disease progression.
2) In the absence of clinically evident findings of disease progression (PD) by RECIST ver. 1.1,

a second imaging study to evaluate PD according to the specific criteria below should be performed after disease progression (PD). The partially modified RECIST ver. 1.1 used for objective confirmation of progression is used only to confirm objective disease progression (confirmed PD). Imaging evaluations to determine objective disease progression should be performed within 4 weeks of the first imaging evaluation that determined PD using RECIST ver. 1.1.

The following is RECIST ver. 1.1 modified to establish objective disease progression.

- On two consecutive image evaluations, the sum of the diameters of the target lesions (TL) increases by more than 20% compared to the sum of the smallest diameters and the sum of the diameters increases by more than 5 mm compared to the sum of the smallest diameters.
- Non-target lesions (NTL) and/or pre-existing new lesions showed significant progression (worsening) at the time of the confirmatory examination compared to the most recent imaging evaluation (note: new lesions at the time of imaging evaluation that are determined for the first time to be PD by RECIST ver. 1.1 are evaluated as NTL at the second imaging evaluation).
- The appearance of an obvious new lesion that was not present at the first imaging evaluation that determined PD according to RECIST ver. 1.1 but was present at the second imaging evaluation.

Two consecutive assessments meeting the definition of PD (first PD by RECIST ver. 1.1 and a second PD using the progression confirmation criteria (above)) are required to establish objective disease progression (determination of definite PD). If PD by RECIST ver. 1.1 does not confirm objective disease progression, evaluation will continue until the next PD by RECIST ver. 1.1. In the absence of significant clinical progression, treatment with investigational agents may continue between the first assessment of progression and imaging studies to confirm progression. If PD is confirmed on confirmatory imaging, the date of progression will be the date PD was confirmed on the previous visit. If objective disease progression is not confirmed, the subject will continue to receive study drug and on-therapy evaluation until the next PD, if there is no clinically significant worsening, at which time another confirmatory scan will be required, even if objective disease progression is confirmed. If the initial PD is not immediately confirmed at the next scan, the investigator should not change the PD assessment from the initial scan.

If subjects discontinue treatment (and/or receive subsequent anticancer therapy) prior to radiographic progression, subjects should be followed until objective disease progression is confirmed. Once progression is confirmed, subjects should continue to be followed for survival every 2 months (8 weeks) according to the evaluation follow-up schedule.

7.5. Assessment of safety

7.5.1. Clinical laboratory tests

Blood and urine samples for determination of clinical chemistry, hematology, and urinalysis will be taken at the times indicated in the assessment schedules and as clinically indicated（refer to 7.1.）

Clinical laboratory safety testing, including serum pregnancy testing, is performed in a licensed clinical laboratory according to local standard procedures. Specimen tubes and specimen sizes may vary depending on the laboratory method used and routine practices at the site. Pregnancy testing may be performed at the site using an approved test (urine or serum pregnancy test). Abnormal clinically significant laboratory results should be repeated as soon as possible (preferably within 24 to 48 hours).

Additional safety samples may be collected if clinically indicated at the discretion of the Investigator. The date, time of collection, and results (values, units, and reference ranges) will be recorded on the appropriate eCRF.

The laboratory variables to be measured are presented in Table 4 (Hematology/ Coagulation), Table 5 (Clinical chemistry), and Table 6 (urinalysis).

Other safety tests to be performed at screening include assessment for HBs antigen, HBs antibody, HBc antibody, and HCV antibody, and HIV antibodies.

The following laboratory variables will be measured:

Table4. Hematology/Coagulation Laboratory Tests

Basophils	Monocytes
Eosinophils	Neutrophils
Hematocrit	Platelet count
Hemoglobin	Red blood cell count
Lymphocytes	Total white cell count [a]
PT-INR	APTT

Table5. Clinical Chemistry (Serum or Plasma) Laboratory Tests

Albumin	Lactate dehydrogenase
Alkaline phosphatase	Lipase
Alanine aminotransferase	Magnesium
Amylase	Potassium
Aspartate aminotransferase	Sodium
Calcium	Total bilirubin[a]
Chloride	Total protein
Creatinine	Urea or blood urea nitrogen, depending on local practice
Gamma glutamyltransferase[b]	Uric acid
Glucose	

a. Tests for ALT, AST, alkaline phosphatase, and total bilirubin must be conducted and assessed concurrently. If total bilirubin is ≥2 × upper limit of normal (and no evidence of Gilbert's syndrome) then fractionate into direct and indirect bilirubin.

b. It is preferable that both amylase and lipase parameters are assessed. For sites where only 1 of these parameters is routinely measured then either lipase or amylase is acceptable.

c. Bicarbonate (where available), chloride, creatinine clearance, gamma glutamyltransferase, and magnesium testing are to be performed at baseline, on Day 1 (unless all screening laboratory clinical chemistry assessments are performed within 3 days prior to Day 1), and if clinically indicated.

d. Creatinine Clearance will be calculated by data management using Cockcroft-Gault (using actual body weight).

e. If TSH is measured within 14 days prior to Day 1 (first infusion day), it does not need to be repeated at day Free T3 or free T4 will only be measured if TSH is abnormal or if there is a clinical suspicion of an AE related to the endocrine system

Table 1. Urinalysis Tests[a]

Bilirubin	pH
Blood	Protein
Glucose	Specific gravity
Ketones	Colour and appearance

a. Microscopy should be used as appropriate to investigate white blood cells and use the high-power field for red blood cells

If a patient shows an AST or ALT ≥3xULN together with total bilirubin ≥2xULN, refer to 8.18.3 for further instructions on cases of increases in liver biochemistry and evaluation of Hy's Law. These cases should be reported as SAEs if, after evaluation, they meet the criteria for a Hy's law case or if any of the individual liver test parameters fulfill any of the SAE criteria.

All patients should have further chemistry profiles performed at 30 days (±3 days), 2 months (±1 week) and 3 months (±1 week) after permanent discontinuation of IP

Any clinically significant abnormal laboratory values should be repeated as clinically indicated and recorded on the eCRF. Situations in which laboratory safety results should be reported as AEs are described in Section8.1.

All patients with Grade 3 or 4 laboratory values at the time of completion or discontinuation from IP must have further tests performed until the laboratory values have returned to Grade 1 or 2, unless these values are likely to improve because of the underlying disease.

7.5.2. Physical examinations

Physical examinations will be performed according to the assessment schedules. Full physical examinations will include assessments of the head, eyes, ears, nose, and throat and the respiratory, cardiovascular, GI, urogenital, musculoskeletal, neurological, dermatological, hematologic/lymphatic, and endocrine systems. Height will be measured at screening only. Targeted physical examinations are to be utilized by the Investigator on the basis of clinical observations and symptomatology. Situations in which physical examination results should be reported as AEs are described in Section 8.1.

7.5.3. Electrocardiogram (ECG)

Resting 12-lead ECGs will be recorded at screening and as clinically indicated throughout the study. ECGs should be obtained after the patient has been in a supine position for 5 minutes and recorded while the patient remains in that position.

At a screening period, in case of clinically significant ECG abnormalities, including a QTcF value >470 ms, 2 additional 12-lead ECGs should be obtained over a brief period (e.g., 30 minutes) to confirm the finding.

Situations in which ECG results should be reported as AEs are described in Section 8.1.

7.5.4. Vital signs

Vital signs (blood pressure [BP], pulse, temperature, and respiration rate) will be evaluated according to the schedule of this clinical trial. Body weight is also recorded at each visit along with vital signs.

First infusion

On the first infusion day, patients will be monitored, and vital signs collected/recorded in eCRF prior to, during and after infusion of IP as presented in the bulleted list below.

BP and pulse will be collected from a subject before, during, and after each infusion at the following times (based on a 60-minute infusion):

- Prior to the beginning of the infusion (measured once from approximately 30 minutes before up to 0 minutes [i.e., the beginning of the infusion])
- Approximately 30 minutes during the infusion (**halfway** through infusion)
- At the end of the infusion ±5 minutes

If the infusion takes longer than 60 minutes, then BP and pulse measurements should follow the principles as described above or be taken more frequently if clinically indicated. A 1-hour observation period is recommended after the first infusion of durvalumab.

Subsequent infusions

BP, pulse and other vital signs should be measured, collected/recorded in eCRF prior to the start of the infusion. Patients should be carefully monitored and BP and other vital signs should be measured during and post infusion as per institution standard and as clinically indicated.

7.5.5. ECOG performance status

ECOG performance status will be assessed at the times specified in the assessment schedules based on the following:

0. Fully active; able to carry out all usual activities without restrictions
1. Restricted in strenuous activity, but ambulatory and able to carry out light work or work of a sedentary nature (e.g., light housework or office work)
2. Ambulatory and capable of self-care, but unable to carry out any work activities; up and about more than 50% of waking hours.
3. Capable of only limited self-care; confined to bed or chair more than 50% of waking hours
4. Completely disabled; unable to carry out any self-care and totally confined to bed or chair
5. Dead

Any significant change from baseline or screening must be reported as an AE.

7.5.6. Other safety assesements

If new pulmonary symptoms (e.g., dyspnea) or radiological abnormalities suggestive of pneumonia/interstitial lung damage (ILD) are observed, toxicity management as detailed in the Toxicity Management Guidelines (see Appendix of Protocol 2) will be applied. Complete diagnostic results (including high-resolution computed tomography (HRCT), blood and sputum cultures, hematological parameters, etc.) should be recorded in the source documents. An accurate diagnosis including consultation with a specialist is strongly recommended to rule out alternative causes such as lymphangitic carcinomatosis, infection, allergy, cardiogenic edema, or pulmonary hemorrhage. In the presence of a confirmatory HRCT scan that excludes other causes of respiratory symptoms, the diagnosis of interstitial lung disease (ILD) should be considered and toxicity management guidelines followed. The investigator is responsible for ensuring that all staff involved in the study are familiar with the contents of this section.

8. HANDLING OF ADVERSE EVENT

8.1. Definition

8.1.1. Adverse event

Adverse events are all unwanted or unintended signs (including abnormal changes in laboratory values), symptoms, or illnesses that occur after the first administration of an

investigational drug, regardless of causal relationship to the study treatment.

8.1.2. Severe adverse event

A serious adverse event is defined as any of the following
(1) results in death
(2) is life-threaening
(3) requires inpatient hospitalisation or prolongation of existing hospitalisation
(4) results in persistent or significant disability/incapacity
(5) is a congenital anomaly/birth defect
(6) Other serious cases according to the above

The term "life-threatening" for the purposes of this definition is defined as an event that the investigator/participating physician determines poses an imminent risk of death to the subject as a result of its manifestation. It does not mean hypothetically that death might have resulted had the manifestation of the event been more severe.

"Hospitalization" in (3) below is not considered a serious adverse event if any of the following apply: (However, a new occurrence during the hospitalization is treated as an adverse event. (However, any new occurrence during that hospitalization will be treated as an adverse event.)

- Hospitalization as defined in this study protocol
- Hospitalization or prolongation of hospital stay that was planned prior to the start of the clinical trial.
- Hospitalization or prolonged hospitalization for social reasons (reasons of convenience or other non-medical necessity)
- Hospitalization or extended hospital stay for examination, education
- Hospitalization for follow-up or prolongation of hospitalization for those who are cured or have a mild illness.
- Hospitalization or prolonged hospitalization for new treatment of the underlying disease after completion of the investigational drug

(6) Other serious, in accordance with the above, are "significant medical events" that may not be immediately life-threatening or result in death or hospitalization, but which may endanger the subject or require treatment or therapy to avoid the consequences as listed in these definitions.

Adverse events (AEs) for malignancy reported during a clinical trial will generally be evaluated as serious AEs. If no other severity criteria are met, the AE will be judged to be a "Significant Medical Event" as described above. However, in certain circumstances, medical judgment based on individual events should be applied to clarify that malignancy events should be evaluated and reported as non-serious AEs. For example, if medical history includes malignancy and the malignancy progresses during the clinical trial, but the progression does not change the treatment or prognosis of the malignancy, the malignancy progression should be reported as an AE but may not meet the attributes to be evaluated as serious.
The causal relationship of the SAE (relationship to all investigational treatments/procedures) should be evaluated by the investigator and reported to AstraZeneca.

8.1.3. Adverse Events of Special Interest (AESI)

AESIs of durvalumab and tremelimumab for heavy particle irradiation include events due to potential inflammatory or immune-mediated mechanisms, which require more frequent monitoring and treatment with steroids, immunosuppressive agents, and/or hormone replacement

therapy.Careful monitoring of these AESIs will be implemented in clinical trials of durvalumab monotherapy and durvalumab plus tremelimumab combination therapy. Immune-mediated adverse events (imAEs) are AESIs, defined as events that occur with the administration of (exposure to) an investigation agent, are consistent with an immune-mediated mechanism of action, and have no apparent other cause. imAEs should be diagnosed using serologic, immunologic, and histologic (biopsy) data, as appropriate, for support. The diagnosis of imAE should be supported by serologic, immunologic, and histologic (biopsy) data as appropriate, and efforts should be made to rule out tumor, infection, metabolism, toxins, and other causes of imAE.

If there is any doubt as to whether an adverse event is an imAE, the investigator should immediately contact the sponsor's medical experts. AESIs for durvalumab and/or tremelimumab and heavy particle irradiation include the following.

- Dysentery/colitis, intestinal perforation
- Pneumonitis/ILD
- Hepatitis/transaminases increase
- Endocrine disorders (i.e., hypophysitis, hypopituitarism, adrenal insufficiency, hyperthyroidism, hypothyroidism and type I diabetes mellitus)
- Rash/dermatitis
- Nephritis/increased blood creatinine
- Pancreatitis/increased serum lipase and amylase
- myocarditis
- pericarditis
- Myositis/polymyositis
- Neuropathy/neuromuscular toxicity (Guillain-Barré syndrome, myasthenia gravis, etc.)
- Other inflammatory reactions of rare / infrequent immune-mediated (but not limited to pericarditis, sarcoidosis, uveitis, and ocular, skin, blood system and rheumatology-related events)

In addition, reactions associated with infusion and hypersensitivity/anaphylactic reactions due to various pharmacologic causes are also considered AESIs.

Details of these risks and presenting symptoms are described in the most recent versions of the durvalumab and tremelimumab investigational new drug summaries. Specific guidelines for the evaluation and treatment of these AESIs are provided in the Dose Modification and Toxicity Management Guidelines. These guidelines were developed by the investigational drug provider to assist investigators and others in making clinical decisions when treating this type of toxicity. This guideline applies to adverse events that the reporting investigator determines are causally related to the investigational drug/regimen.

If new or worsening pulmonary symptoms (e.g., dyspnea) or radiological abnormalities suggestive of pneumonia/interstitial lung disease are observed, toxicity management as detailed in the “Toxicity Management Guidelines” (see Appendix F) will be applied. Results of complete diagnostic tests (including high-resolution computed tomography (HRCT), blood and sputum cultures, hematological parameters, etc.) should be recorded in the source documents.

An accurate diagnosis, including consultation with a specialist, is strongly recommended to rule out alternative causes such as lymphangitic carcinomatosis, infection, allergy, cardiogenic edema, or pulmonary hemorrhage. On confirmatory HRCT scans where other causes of respiratory symptoms have been ruled out, the diagnosis of interstitial lung disease (ILD) should be considered and toxicity management guidelines followed.

8.1.4. Confirmation of interstitial lung disease (ILD)

To ensure thorough investigation and diagnosis of possible cases of pneumonia, the following evaluations, and additional evaluations as needed, will be performed. Collect the results of the evaluation.

- physical examination
- Evaluate signs and symptoms (cough, shortness of breath, fever, etc.), including auscultation of the lung field.
- Peripheral oxygen saturation (SpO2)

Other

If pneumonia (ILD) is suspected during clinical trial treatment, the following markers should be measured.

if possible

(i) ILD markers (KL-6, SP-D) and β-D-glucan

(ii) Tumor markers. Specific tumor markers associated with disease progression.

Other biochemistry: CRP, LDH

8.2. Assessment of severity

AEs and SAEs; severity will be determined in accordance with CTCAE ver. 5.0 The severity of all other events not listed in the CTCAE will be determined by the severity category from Grade 1 to 5, as determined by the investigator based on medical judgment, will be as follows.

- Grade 1 (mild)
  Events that are usually transient and require only minimal Treatment or therapeutic intervention. The event generally does not interfere with normal activities of daily living.

- Grade 2(Moderate)
  An event that is usually alleviated by additional specific therapeutic intervention. The event interferes with normal daily activities and causes discomfort, but does not pose a risk of serious or permanent harm to the subject.

- Grade 3(Severe)
  An event requiring intensive therapeutic intervention. An event that interferes with normal daily activities or significantly affects the subject's clinical condition.

- Grade 4(life-threatening）
  Events and/or immediate sequelae related to the following Imminent risk of death or a physical or mental impairment that affects or limits the ability to perform activities of daily living (eating, walking, toileting, etc.).

- Grade 5(deadly)
  Death as a result of an event. it is important to distinguish between serious criteria and severity of AEs.

It is important to distinguish between severity and severity of an AE. Severity is a measure of intensity, and severity is defined by the criteria in section 10.3.1 A Grade 3 AE need not necessarily be considered an SAE. For example, a Grade 3 headache lasting several hours would not meet the regulatory definition of an SAE and would be considered a non-serious event, whereas a Grade 2 attack leading to hospitalization would be considered an SAE.

8.3. Record of adverse events and serious adverse events

AEs and SAEs will be collected from the time of the first dose of study drug until the follow-up date 28 days after the discontinuation of study drug. However, AE and SAE outcomes will be collected until the end of the safety information collection period (90 days after the discontinuation of study drug) or until the start of alternative anticancer therapy. Of events occurring after the 28-day follow-up period after the discontinuation of the investigational drug,

if the event is considered to be due to delayed toxicity to the investigational drug, it will be collected as an AE or SAE until the end of the safety information collection period or until the start of alternative anticancer therapy. All AEs and SAEs will be actively followed up for each subject for the duration of the trial as long as the event is ongoing. Every effort will be made to resolve all events, even if the event continues after the subject discontinues the investigational drug or after the study is terminated.

AEs unresolved at the subject's last clinical trial visit will be followed up by the investigator as long as medically necessary but will not be further documented in the eCRF. AstraZeneca reserves the right to request additional information from subjects with ongoing AEs/SAEs at the end of the trial if deemed necessary.

For each AE, the following information should be collected

- Adverse event name
- Date of adverse event and date of disappearance
- Maximum CTCAE Grade
- Severity
- Assessment of causal relationship with study treatment
- Treatments related to investigational drugs
- Treatment of adverse events: Administration of AE medication
- outcome

In addition to the above, the following are also confirmed for SAE.

- Date of serious adverse event
- Date when the investigator becomes aware of the onset of the serious adverse event
- Definitions applicable to the determination of serious adverse events
- Date of admission
- Date of discharge
- Presumed reason for death
- Date of death
- Autopsy
- Evaluation of causal relationship with clinical trial procedures
- Evaluation of causal relationship with other drugs
- Details of serious adverse events

The grading scale described in CTCAE ver. 5.0 is used for all events that have been assigned to a CTCAE Grade; for events that have not been assigned a CTCAE Grade, the CTCAE criteria for converting mild, moderate, and severe events to a CTCAE Grade A copy of CTCAE ver. 5.0 can be downloaded from the Cancer Treatment Evaluation Program website at http://www.jcog.jp/doctor/tool/ctcaev5.html.

8.4. Duration of recording and follow-up of adverse events and serious adverse events

If the subject discontinues treatment for reasons other than objective confirmation of disease progression and therefore continues tumor evaluation, the drug- or treatment-related SAEs will be tracked until the subject confirms PD and no further tumor evaluation is performed.

The investigator is responsible for tracking all SAEs until the subject returns to baseline status or until the condition stabilizes with the expectation that the chronic condition will be maintained, even if it continues beyond study participation, until all SAEs are resolved.

8.5. Causal relationship with investigational therapy

The following examples will be used to determine the causal relationship between the clinical trial treatment and the patient.

Causative: if a reasonable possibility can be explained that the investigational treatment caused the adverse event in question (examples are given below).

1) If the event is time-related to the onset of the event, and the event attenuates with the passage of time after the study treatment but recurs or worsens with subsequent re-administration of the study treatment.
2) The presence of confounding risk factors is negative, such as the subject's general condition, complications, concomitant medications, or concomitant therapies.

No causal relationship: other than above

8.6. Outcome definition

Outcomes after adverse events are determined from the following

1) Recovery: when the patient recovers to the state before the adverse event occurred.
2) Recovered but with sequelae: When the adverse event has recovered but the effects of the adverse event remain as sequelae
3) Death: when the adverse event that occurred was the direct cause of death
4) Lightening of symptoms: adverse events continue, but symptoms are improving
5) Unrecovered: adverse events continue (symptoms are not improving)
6) Unknown: When the subject is no longer traceable

8.7. Treatment of investigational drug in the event of an adverse event

1) No change: When an adverse event occurs but there is no change in the conditions under which the investigational drug is administered.
2) Discontinuation: Discontinuation of study drug administration due to the occurrence of an adverse event
3) Withdrawal: Temporary suspension of study drug administration due to the occurrence of an adverse event
4) Not applicable: Adverse events occur before the start of study drug administration or after the end of the study period

8.8. Treatment of heavy particle therapy equipment in the event of an adverse event

1) Suspension: Temporary suspension of heavy-ion radiation therapy due to the occurrence of adverse events
2) Discontinuation: Discontinuation of heavy-ion radiation therapy due to the occurrence of an adverse event
3) Not applicable: Adverse events occur before or after the start or completion of carbon ion radio therapy

8.9. Relationship to Protocol Procedures

The investigator must also provide an assessment of the relationship between SAEs and protocol procedures on the SAE Report Form. This includes both non-therapeutic emergencies (SAEs that occur prior to the administration of the investigational drug) and therapeutic emergency SAEs. Protocol-related SAEs may occur because of a required procedure or intervention (e.g., blood draw) during the clinical trial. The investigator should use the following guidelines to assess the relationship between SAEs and protocols

- Protocol-related: The event occurred because of a procedure or intervention described in the protocol for which no alternative etiology exists in the subject's medical record.
- Not protocol-related: The event is related to an etiology other than the procedure or intervention

described in the protocol. The alternative etiology must be documented in the study subject's medical record.

In the case of AEs, AEs on treatment (or AEs appearing on treatment) are defined as AEs that began after or before administration and worsened after exposure to treatment between the start dose date and 90 days after discontinuation of study treatment.

8.10. Adverse events based on signs and symptoms

All AEs reported spontaneously by the subject or in response to questions from the investigator, subinvestigator, or collaborator (example of a visit: “Have you had any health problems since your last visit / have you been asked any questions since your last visit?”) “)

When collecting AEs, recording the diagnosis is preferred over recording a list of signs and symptoms, if possible. However, if the diagnosis is known and there are other signs and symptoms that are not generally part of the diagnosis, record the diagnosis and each sign or symptom separately.

8.11. Adverse events based on tests and examinations

Protocol-mandated laboratory values and vital signs measurements will be summarized in the CSR. Therefore, protocol-mandated worsening of laboratory values and vital signs relative to baseline should only be reported as an AE if it meets one of the SAE criteria or is a reason for discontinuation of treatment with the investigational drug.

If the worsening of laboratory values or vital signs is associated with clinical signs or symptoms, the signs or symptoms should be reported as an AE and the associated laboratory results or vital signs should be reported as additional information, as appropriate. Whenever possible, the reporter uses clinical terms rather than laboratory terms (e.g., “anemia” rather than “low hemoglobin level”). In the absence of clinical signs or symptoms, the worsening of such laboratory values should be reported as an AE.

Laboratory deterioration attributable to apparent disease progression shall not be considered an adverse event or serious adverse event.

Report as an adverse event any new or worsening clinically significant abnormal findings at the time of presentation compared to the baseline evaluation.

8.12. Hy's Law

Biochemical elevations suggestive of abnormal liver function may require further evaluation, and the occurrence of AST or ALT ≥ 3 x ULN and total bilirubin ≥ 2 x ULN may require reporting as an SAE. For cases of elevated liver biochemistry and Hy's Law evaluation, see Ref.

8.13. Disease progression

Disease progression is considered a worsening of the subject's condition due to the disease for which the investigational drug is being studied and is an increase in the severity of the disease under study and/or an increase in the symptoms of the disease. The development of new metastases or progression of existing metastases to the primary cancer being studied is considered disease progression and is not considered an AE. Events that are clearly attributable to disease progression should not be reported as AEs during a clinical trial.

8.14. New cancer

The development of new cancer shall be considered a serious adverse event. New cancers are those that occur after a patient is enrolled in the study, rather than those that were the primary reason for enrollment in the study. New metastatic lesions are considered progression of the cancer under study and are not reported as a second cancer.

8.15. Deaths

All deaths occurring during the investigational treatment period or within the protocol-defined follow-up period after the discontinuation of the investigational drug shall be reported as follows.

- Events that are clearly attributable to disease progression will not be treated as investigational AEs, but if the outcome of the event is found to be severe, all SAEs, not limited to death, will be reported, as described in Section 10.14.
- The investigator shall immediately report to the site director and the investigational drug provider, regardless of the causal relationship between the SAE and the investigational drug/IR, regardless of whether the death is due to progression of the disease under study. In addition, a note will be made in the eCRF.
- The causes are identified and described in the eCRF.
- Deaths of unknown cause should always be reported as SAEs.
- If an autopsy is performed, the autopsy results should be reported to AstraZeneca.

Deaths that occur after the discontinuation of the investigational drug and after the protocol-defined safety information collection period should also be noted in the eCRF as required. If the death is attributable to an event that occurred after the safety information collection period and the event is considered to be due to delayed toxicity to the investigational drug, it must also be reported as an SAE.

AstraZeneca retains the right to request additional information for subjects with ongoing AE(s)/SAE(s) at the end of the trial if deemed necessary.

8.16. Reportable Adverse Events

The Investigator or Co-Investigator will ensure that all adverse events that occurred from the time consent is obtained until 28 days after the end of study drug administration are consistently described in the case report form and that adverse events disappear or that the study period is observed until 4 weeks after the end of the study period (after discontinuation). Adverse events that are judged to have a causal relationship with the study drug will continue to be observed as much as possible until the end of the study period. However, this does not apply if the investigator determines that the safety of the subject is sufficiently ensured and further follow-up is not necessary.

8.17. Reporting of serious adverse events

All serious adverse events during the clinical trial (regardless of whether there is a causal relationship with the investigational drug or not) and serious adverse events suspected to be related to the investigational drug after the completion (discontinuation) of the clinical trial shall be reported according to the following procedures. The details of the reporting procedures are separately stipulated in the “Standard Operating Procedures for Handling Safety Information". An outline of the reporting procedure is provided below.

1） Reports from the investigator to the head of the site and to investigators at other sites

If any serious safety information is recognized during the clinical trial period, the investigator shall immediately report the details to the head of his/her institution, the investigational drug supplier, and the investigators at other sites using the “Report on Serious Adverse Events and Malfunctions” (Uniform Form 12, or the same detailed description form, if necessary). In reporting, the investigator shall specify whether or not the serious safety information is an event that cannot be predicted from the investigator’s brochures, etc.

2） Discussion between the investigator and the Clinical Trial Coordinating Committee

The investigator shall consult with the Trial Coordinating Committee and report his/her opinion as the investigator (including the necessity of reporting to the Minister of Health, Labour and Welfare) to the Trial Coordinating Committee. The investigator shall report his/her opinion (including the necessity of reporting to the Minister of Health, Labour and Welfare) to the Trial

Coordinating Committee. If the Independent Data Monitoring Committee is consulted regarding the investigator's judgment, the opinion of the Independent Data Monitoring Committee shall be followed.

3) Report to the Minister of Health, Labour and Welfare and the heads of other medical institutions

1 Report to the Minister of Health, Labor and Welfare

If the Clinical Trial Coordinating Committee determines that the case is subject to reporting as stipulated in Article 273 of the Enforcement Regulations of the Act on Quality, Efficacy and Safety Assurance of Pharmaceuticals, Medical Devices and Other Products (Pharmaceuticals and Medical Devices Law), it shall report to the Minister of Health, Labor and Welfare (the Pharmaceuticals and Medical Devices Agency, an independent administrative agency (PMDA)). In addition, when the Independent Data Monitoring Committee is consulted, it shall be notified of the contents of the report made to this authority.

2 Reporting to the heads of other implementing medical institutions

When a report is made to the Minister of Health, Labour and Welfare, the investigator of the other site shall report the contents of the "Failure/Infectious Case Report" obtained from the Trial Coordinating Committee to the head of his/her site as soon as possible.

4) Actions to be taken when additional information is obtained

When additional information regarding the adverse event is obtained, the investigator of the site where the adverse event occurred shall make an additional report to the head of the site as soon as possible, as well as to the Clinical Trial Coordinating Committee and the investigational drug supplier. The handling of such additional information shall be in accordance with the procedures described in 1) to 3) above, and reports shall be made to the NIH as necessary.

5) Annual report to the Minister of Health, Labor and Welfare

If the investigational drug has not been approved in Japan, or if the sponsor is not conducting a clinical trial, the person conducting the clinical trial shall submit an annual report to PMDA in accordance with Article 273 of the Ordinance for Enforcement of the Pharmaceutical Affairs Law.

The investigator will report all SAEs to AstraZeneca. The investigator will send a copy of the SAE report submitted to PMDA to the investigational drug provider in accordance with a separate Standard Operating Procedure (SOP). If any changes are made to the follow-up report, the following information will also be promptly sent to the investigational drug provider.

8.17.1. Response to subjects

When a serious adverse event is observed, the investigator or subinvestigator should immediately take appropriate measures, discontinue administration of the investigational drug to ensure the safety of the subject, and inform the subject if treatment for the adverse event becomes necessary. If an adverse event that was considered to be causally related to the investigational drug at the time of discontinuation has not yet recovered, observation will be continued as much as possible until the adverse event recovers or becomes mild, in principle. However, this does not apply when the investigator determines that the subject's safety has been sufficiently ensured and further follow-up is not necessary.

8.18. OTHER EVENTS REQUIRING REPORTING

8.18.1. Overdose

Use of durvalumab or tremelimumab in excess of the prescribed dose of the drug constitutes an overdose. Currently, there is no established treatment for durvalumab or tremelimumab overdose and no established symptoms of a possible overdose. Overdoses with associated AEs are recorded as an AE diagnosis or symptom in the relevant AE module of the eCRF. Overdoses without symptoms are only recorded and reported to the site investigator.

Overdoses of durvalumab or tremelimumab, with or without associated AEs/SAEs, must be recorded and reported to the site investigator. The investigator must report these to the investigational drug provider as described in the SOP.

8.18.2. Hepatic function abnormality

Abnormal liver function that meets Hy's Law criteria should be reported as an SAE regardless of the presence or absence of clinical symptoms. Hy's Law criteria are met if AST or ALT is at least 3 times the upper limit of normal (ULN) or TBL is at least 2 times the upper limit of normal at any time after initiation of treatment with the investigational drug, regardless of elevated ALP.

If it cannot be determined that there is no causal relationship to the investigational drug (e.g., due to cholelithiasis or bile duct obstruction), the investigator should report these events to the study provider in accordance with SOPs.

If the cause of the hepatic dysfunction is established and there is no causal relationship to the investigational drug, the investigator will decide whether to continue the subject's treatment based on the investigator's clinical judgment.

- If the cause of the liver function abnormality is not established, the subject's administration should be discontinued immediately. Follow-up investigations and inquiries will be initiated by the investigational site without delay.

The investigator will follow up on each reported event of liver function abnormality.

8.18.3. Pregnancy

All pregnancies and pregnancy outcomes should be reported to AstraZeneca and the site investigator, except in the following cases

- Pregnancy discovered before the subject received the investigational drug.
- Pregnancy of the male subject's female partner. (If the male subject is not restricted from having children.)

8.18.4. Exposure to pregnant woman

If a subject becomes pregnant during a clinical trial, the investigational drug should be discontinued immediately. Pregnancy itself shall not be considered an AE unless there is a suspicion that the investigational drug interfered with the effectiveness of the contraceptive. Birth defects or birth defects and spontaneous abortions should be reported and treated as SAEs. Uncomplicated elective abortions shall not be treated as AEs. The outcome of all pregnancies (spontaneous abortion, elective abortion, ectopic pregnancy, normal delivery, and congenital anomalies) shall be followed up and documented even if the clinical trial is discontinued. If pregnancy occurs during the clinical trial, the investigator shall notify AstraZeneca in accordance with the SOP.

8.18.5. Exposure to partner

Male subjects should refrain from sexual intercourse or sperm donation with their partners during the study and for 180 days after the last dose of durvalumab plus tremelimumab combination therapy or 90 days after the last dose of durvalumab monotherapy, whichever is longer. Pregnancy in the subject's partner is not considered an AE. However, the outcome of all pregnancies (spontaneous abortion, elective abortion, ectopic pregnancy, normal delivery, or

congenital anomaly) occurring between the date of first dose and 180 days after the last dose of durvalumab + tremelimumab combination therapy or 90 days after the last dose of durvalumab monotherapy, whichever is longer, will be followed up and documented if possible. Follow-up and documentation, if possible, is desirable. Upon receipt of a report of pregnancy, the investigator must obtain the consent of the subject's partner prior to obtaining any information regarding the pregnancy. Therefore, the study team should adopt the generic ICF template according to the procedure and submit it to the Institutional Review Board (IRB) prior to use.

8.19. Medication error

For this study, a medication error is an unintentional error in the course of treatment with an investigational drug that may cause harm to the subject. A medication error is not a lack of efficacy of the investigational drug, but an artificial or process-related failure while the investigational drug is under the control of the site staff or subject. Medication errors include the circumstances under which the error occurred.

- A medication error occurred.
- A medication error occurred, but was identified before the subject received the medication.
- No medication errors occurred, but circumstances were observed that could have resulted in errors.

Examples of events that should be reported in a clinical trial as medication errors

- Confusion of drug names
- Dispensing errors (e.g., some medications were incorrectly dispensed even though they were not actually administered to the subject.
- Drugs that were not administered as directed, such as by incorrect route or site of administration
- Drugs not taken as directed, such as tablets dissolved in water when they should be taken as solid tablets
- Drugs not stored as directed (e.g., in a refrigerator when they should be at room temperature.)
- Subjects who received medication by mistake
  Drugs administered to the wrong subject

Examples of events that need not to be reported as medication errors in clinical research

- Including those that lead to any of the above events or that result in a dose error.
- If the subject fails to take the medication (e.g., forgets to take the medication）
- Overdose Accidents
- Subject did not return unused or empty packaged medications
- Errors associated with background or rescue drugs, even AstraZeneca products, or standard of care drugs in open label studies

Medication errors are not considered AEs, but AEs may occur because of medication errors. If a medication error occurs during a clinical trial, the investigator or other site personnel should contact AstraZeneca within one day.

8.20. Predicted Side Effects

The following is a tabulation of adverse events that occurred in clinical trials of durvalumab (Frequency of occurrence: 5% or more). For other adverse events, please refer to the Investigators brochure.

（1）Information on adverse events in patients treated with only durvalumab (all grades, incidence >5%)

Side effect

Fatigue
anorexia
cough
Nausea
Breathe bitterly
constipation
diarrhea
fever
anaemia
Back pain
vomiting
Itching
lethargy
Joint pain
Hypothyroidism
rash
headache
Peripheral edema
sleeplessness
bellyache
Weight loss
Musculoskeletal pain
dizziness
pneumonia
Urinary tract infections
Muscle pain
Upper respiratory tract infections
AST elevation
Pain in the extremities
Hyponatremia
ALT elevation
Wet cough
Nasopharyngitis

（2）Information on adverse events in patients treated with durvalumab and tremelimumab in combination (all grades, incidence >5%)

Side effect
fatigue
diarrhea
anorexia
nousea
pruritic
constipation
dyspnea
anaemia

fever
vomiting
coughing
backache
rash
lethargy
abdominal pain
peripheral edema
weight loss
arthralgia
hypothyroidism
insomnia
AST elevation
hyponatremia
ALT elevation
headache
Lipase rise elevation
dizziness
amylase elevation
hypokalemia
pneumonia
maculopapular eruption
Urinary tract infections
ALP elevation
dehydration
Hyperthyroidism
hyperglycemia
γ-GTP elevation
Hypoalbuminemia
Musculoskeletal pain
Muscle pain
Dry skin
Wet cough
Xerostomia
musculoskeletal chest pain
upper respiratory tract infection

（3）Refer to Section 2.3.2.3 for more information on the risks of Carbon ion radio therapy.

9. ENDPOINT

9.1. Primary endpoint

Percentage of dose-limiting toxicities (DLT) and adverse events/serious adverse events

Rationale for Setting the Primary Endpoint<br><br>To evaluate the tolerability and safety of durvalumab tremelimumab in combination with carbon ion radio therapy in subjects with advanced hepatocellular carcinoma with vascular invasion.

9.2. Secondary endpoint

Overall survival (OS), 6-month survival, objective response rate (ORR) calculated by RECIST ver 1.1 and mRECIST, 6-month progression-free survival (PFS), Time to progression (TTP)

Rationale for Setting Secondary Endpoints<br><br>To evaluate the efficacy of durvalumab tremelimumab in combination with carbon ion radio therapy in subjects with advanced hepatocellular carcinoma with vascular invasion.

9.3. Exploratory endpoints

In addition to consent for this study, consent for “Exploratory Study of Tumor Cells and Tumor Microenvironment in Hepatocellular Carcinoma Using Tumor and Non-tumor Biopsy Specimens, Blood Specimens, and Stool Specimens” and “Exploratory Study of Tumor Cells and Tumor Microenvironment by Secondary Use of Biological Specimens in Hepatobiliary Pancreatic Cancer” and a liver biopsy or liver tumor biopsy specimen must be In patients with sufficient liver biopsy/tumor biopsy samples, biomarkers associated with clinical outcome of combination therapy with durvalumab/tremelimumab and carbon ion radio therapy will be explored using blood and liver biopsy/tumor biopsy samples. The analysis will be performed in the laboratory of the Department of Gastroenterology, Graduate School of Medicine, Chiba University, and may be contracted to an outside vendor or outside research organization.

10. STATISTICAL METHODS AND SAMPLE SIZE DETERMINATION

10.1. Description of Analysis set

10.1.1. DLT analysis set

All subjects enrolled in the study, who have completed at least one investigational drug regimen (IR), and for whom a DLT evaluation has been performed, are considered the DLT analysis set.

10.1.2. Safety analysis set: SAF

All subjects who have had at least one dose of the investigational drug or investigational drug regimen (IR) are considered in the Safety analysis set.

10.1.3. Full Analysis set: FAS

All subjects with eligible disease who are properly enrolled and have received at least one dose of the investigational drug regimen are considered Full Analysis set (FAS).

10.1.4 Efficacy Evaluable set: EES

The efficacy evaluation analysis(EES) population is defined as a subset of the FAS. That is, it consists of all subjects who are eligible for the trial, receive the study drug/IR, and have had at least one post-dose efficacy endpoint assessed. In addition to subjects with at least one baseline and at least one post-dose efficacy evaluation, the population will include cases of early death or early progression before the evaluation.

10.1.5 Per protocol set：PPS

Subjects from the FAS who do not have any of the following serious violations of the study protocol, including the study protocol, such as the method of study or concomitant therapy, will be considered to be in compliance with the study protocol (PPS).

- Violation of inclusive criteria
- Violation of exclusion criteria
- Violation of concomitant use of prohibited drugs
- Violation of concomitant use of prohibited therapies

10.2. Target number of cases and rationale for setting

Target cases: 3 to 15

A modified “3+3 design” will be used in this trial. Cohort A will be used to evaluate DLT for carbon ion radio therapy + durumab, and instead of dose escalation, the group with toremelimumab will be used as Cohort B to evaluate DLT.

10.3. Case Handling

The coordinating investigators and the statistical analyst will discuss and decide how to handle the registered cases. The coordinating investigator and the statistical analyst will also discuss and decide how to handle cases in case new problems arise.

10.4. Data Handling

In principle, the handling of data during data compilation and analysis shall be as follows. In case of doubt, the statistician and the coordinating Investigators shall discuss and decide the handling of the data. Details are described in the statistical analysis plan.

1) Missing values: Supplementation of missing values is not performed.
2) Reference values: Data with unreliable measured values due to hemolysis, etc. are not used in the aggregate analysis.
3) Time lag: Data not conducted during the specified observation period will not be used in the aggregate analysis for that period. For other data requiring consideration, the coordinating investigators will consult with medical experts to determine how to handle the data for analysis.

10.5. Statistical analysis items and analysis plan

All patients will be analyzed after completion of treatment with the investigational drug and after the data have been fixed, using the DLT evaluation population for DLT evaluation, the ESS analysis population for efficacy evaluation, and the safety analysis population for safety evaluation. For the efficacy evaluation using RECIST without survival analysis, FAS and PPS analysis and sensitivity analysis will be performed as necessary.

10.5.1. Analysis of subject background

The distribution of subject background data and summary statistics are calculated for each cohort. For nominal variables, frequencies and proportions of categories are shown. For continuous variables, summary statistics (number of cases, mean, standard deviation, median, range, and interquartile range) are calculated.

10.5.2. Analysis of primary endpoints

10.5.2.1. DLT Evaluation

1) DLT incidence rate

The number of DLT cases and incidence rate will be calculated for each cohort.

10.5.2.2. Safety Evaluation

1) Adverse event rate
2) Serious adverse event rate
3) Adverse events coded by MedDRA
4) List of SOC, PT, severity, relevance, etc.

10.5.3. Analysis of secondary endpoints

10.5.3.1 Efficacy Analysis

Overall survival (OS), 6-month survival rate, objective response rate (ORR), 6-month progression-free survival (PFS), and time to progression (TTP) will be determined as secondary parameters to evaluate efficacy in each cohort. For survival analysis, the Kaplan-Meier method will be used to determine the incidence rate at 6 months and the median and mean survival times using the ESS analysis group.

Overall Response Rate（ORR）

ORR (based on evaluation by investigators using RECIST 1.1) is defined as the percentage of patients with a CR or PR at one or more visits. Patients who had a response after discontinuation of treatment without PD and initiation of post-treatment are not included in the ORR response cases.

Progression-Free Survival（PFS）

PFS (based on evaluation using RECIST 1.1 by investigators and others) is defined as the time from allocation to objective disease progression or death (or cause of death in the absence of disease progression), regardless of whether the patient discontinued treatment or received other anticancer therapy before disease progression. Patients who have not progressed or died at the time of analysis will be censored at the date of the last RECIST 1.1-based evaluation. However, if a patient has progressed or died after two or more consecutive missed visits, the patient will be censored as of the date of the last RECIST 1.1-based evaluation. In the absence of evaluable visit data or baseline data, censor at Day 1 unless the patient has died between baseline and the second visit, in which case treat as an event with the date of death as the date of the event.

The PFS is always calculated based on the date the imaging study/evaluation was performed, not the date of the visit.

It is possible that an evaluation/imaging study based on RECIST 1.1 scheduled for a specific visit may be performed over several different days. In such cases, the following principles apply.

- For evaluation by the investigator, the earliest RECIST 1.1 evaluation/imaging test date when an element indicating progression is identified is the progression date.PFS
- When an evaluation is terminated, it shall be terminated on the last imaging inspection date of the inspection related to the specific overall effectiveness evaluation.

Time to Progression (TTP)

TTP (based on evaluation using RECIST 1.1 by the investigator and others) is defined as the period from the date of random assignment to the date of objective tumor progression. Death is not included in the definition of TTP. Death without progression is not included in the definition of TTP.

Patients who die without progression will be terminated at the time of death.

Image Evaluation

Imaging evaluation For all imaging evaluations, in addition to evaluation by the investigator or subinvestigators, a central judgment will be made by multiple radiologists to be separately determined.

10.5.4. Interim Analysis

No interim analysis will be performed in this clinical trial.

10.6. Final Analysis

After the follow-up period, analysis will be conducted after the data are obtained and the cases are fixed. The person in charge of statistical analysis compiles the “Analysis Report” and submits it to the coordinating investigator and the principal investigator. The coordinating investigator summarizes the contents of the analysis report and prepares a “summary report” summarizing the overall conclusions of the trial, problems, interpretation and discussion of the results, and future policies mainly from a clinical perspective, and obtains approval from the principal investigator.

10.7. Data Monitoring Committee

An Independent Data Monitoring Committee will be established for this clinical trial. The Independent Data Monitoring Committee will be established as an independent body from the investigators and will consist of three or more expert members who are independent of the study. The Independent Data Monitoring Committee will be established for the purpose of ensuring the safety of subjects. It will provide appropriate advice and recommendations in accordance with a separate protocol If the study is terminated before the end of the DLT evaluation period for reasons other than DLT criteria, the investigator will ask the Independent Data Monitoring Committee for its opinion on the addition of a case If a second DLT occurs, the investigator will ask the Independent Data Monitoring Committee to determine whether the study regimen is If a second DLT occurs, the investigator will ask the Independent Data Monitoring Committee for an opinion on whether the study regimen is intolerable. If necessary, the investigator will confirm whether tolerability was not an issue in the other cases.

If an SAE is reported during the trial, the investigator will check with the Independent Data Monitoring Committee whether to continue the trial and whether any changes should be made to the protocol. The Independent Data Monitoring Committee will provide the investigator with the results of the discussion in writing.

If the investigator determines that precautionary emergency measures are warranted, depending on the importance and scope of the report, actions may include suspension of enrollment and emergency communication to all participating sites.

11. COMPLIANCE AND DEVIATION FROM THE PROTOCOL

1) The investigator or subinvestigator shall conduct the clinical trial in compliance with this protocol.
2) The investigator or subinvestigator shall record the details and reasons for all deviations from the study protocol.
3) In the event of deviation from the protocol for the purpose of avoiding immediate danger to subjects or for other unavoidable medical reasons, the investigator shall immediately submit a document describing the details of the deviation and the reasons for it to the head of the implementing medical institution, and shall also promptly report the contents of said document to the Trial Review Committee via the head of the implementing medical institution. The contents of said documents shall be promptly reported to the Clinical Trial Review Committee via the head of the investigational institution.

12. CHANGES TO THE CLINICAL TRIAL PROTOCOL, CASE REPORT FORM, OR ANALYSIS PLAN

12.1. Revision of Clinical Trial Protocol and Case Report Form

The following procedures shall be followed when revising the clinical trial protocol and case report forms.

1) When the investigator becomes aware of matters related to the quality, efficacy and safety of the investigational drug or other information important for the proper conduct of the clinical trial, the investigator shall revise the relevant protocol as necessary. When a revision is made, a history of the revision shall be prepared and stored.
2) The coordinating investigator shall revise the case report form as necessary in conjunction with the revision of the protocol or for other reasons. Whenever necessary, the coordinating investigator shall revise the case report form in conjunction with the revision of the protocol or for other reasons.
3) The investigator shall promptly submit the revised protocol and revised case report form to the head of the site and obtain approval from the investigational review committee via the head of the site.
4) The same procedure shall be followed when the investigator amends the protocol and case report form within the scope of the investigator's acceptable instructions from the head of the site based on the opinion of the investigational review committee.

12.2. Changes in statistical analysis plan

If the statistical analysis manager changes the contents of the statistical analysis plan, all changes shall be documented in the statistical analysis report for this clinical trial. In addition, the circumstances of any changes to the statistical analysis plan shall be recorded.

13. DISCONTINUATION, SUSPENSION, OR TERMINATION OF THE CLINICAL TRIAL

13.1. Criteria for discontinuation or suspension of the clinical trial as a whole

When the following information is obtained and it is considered difficult to continue the entire clinical trial, the coordinating investigator will consult with the principal investigator and make a decision to discontinue or suspend the entire clinical trial.

- When it becomes difficult to ensure the safety of this clinical trial due to new safety information or serious adverse event information concerning the investigational drug, etc.
- When the site has committed a serious violation of the drug GCP ordinance or a serious deviation from the clinical trial protocol and no improvement has been made.
- Other new information obtained during the conduct of the clinical trial that may necessitate discontinuation or suspension of the clinical trial.

13.2. Procedures for discontinuation or suspension of a clinical trial as a whole

If the coordinating investigator, after consultation with other investigators, decides to discontinue or suspend the entire clinical trial, he/she shall promptly notify the head of the investigational institution and the regulatory authorities in writing to that effect and the reasons in detail. In addition, the investigator shall promptly inform the subjects undergoing the clinical trial and take appropriate measures such as changing to appropriate treatment.

13.3. Procedures for discontinuation or suspension of this clinical trial at an individual clinical site

When the investigator discontinues or suspends a clinical trial, the investigator shall promptly notify the head of the site in writing to that effect and explain the details of the discontinuation or suspension in writing in detail. When notified of the discontinuation or suspension of a clinical trial, the investigator shall promptly notify in writing all investigators and regulatory authorities involved in the said clinical trial to that effect and explain the discontinuation or suspension in detail.

13.4. Termination of this clinical trial

After the completion of the clinical trial, the investigator shall notify the head of the investigational institution in writing that the clinical trial has been completed and report a summary of the results of the clinical trial in writing.

14. DATA MANAGEMENT

14.1. Data management procedure

Detailed procedures for data management shall be described in the data management plan.

14.2. Data Collection

The investigator or sub-investigator shall prepare a case report using Electronic Data Capture (EDC) that meets the requirements of 21 CFR Part 11, the Pharmaceutical GCP Ordinance, and the ER/ES guidelines. The investigator or subinvestigator shall prepare a case report using EDC. The investigator or subinvestigator shall make any changes, corrections or additions to the contents of the case report form on the EDC that generated the case report form, and record all of the changes, corrections or additions as electronic information. When a subinvestigator prepares a case report form or when a collaborator transcribes a case report form from source documents (source data), the investigator shall check the contents of the case report form before submitting it to the EDC and confirm that there are no problems. The investigator provides the final electronic case report form to the institution on an electronic medium (CD-R, etc.). The investigator shall ensure the readability and archivability of the electronic case report form.

When using the EDC system, the site should receive training on EDC and refer to the manual for details on how to input data.

14.3. Identification of documents that are directly described in the case report and should be interpreted as source documents (source data)

In this clinical trial, the following documents and others shall be considered source documents (source data).

1） Medical records, nursing records, clinical laboratory data, imaging films, and other records that form the basis for preparing case reports. Data stored in electronic medical records are also considered source documents.
2） Records of investigational drug administration
3） Documents or records related to the clinical trial that are required under the GCP ordinance for pharmaceutical products related to the clinical trial

Of the data described in the case report form, the following items shall be regarded as source documents (source data) when they are described in the case report form. However, if the data are recorded in the medical record, the medical record shall be regarded as the source documents (original data).

1） Purpose of concomitant medications/adjunctive therapy
2） Determination of the extent of adverse events, outcomes (including results at follow-up), severity, and causal relationship to the study treatment and the basis for the determination
3） Reasons for discontinuation of clinical trials by subjects
4） Comments by the investigator or subinvestigator

15. RETENTION OF SOURCE DOCUMENTS AND OTHER RECORDS

15.1. Retention of records by the clinical site

Documents or records pertaining to a clinical trial to be retained at the investigational site as stipulated in the Pharmaceutical GCP ordinance shall be retained by the hospital director until the later of the following dates.

1) The date on which five years have elapsed since the date of marketing approval for the relevant indication of the test product (if development has been discontinued, the date on which three years have elapsed since the date on which the decision to discontinue

development was made). However, for drugs that are subject to post-approval reexamination in accordance with the provisions of the "Law Concerning Quality, Efficacy and Safety Assurance of Pharmaceuticals and Medical Devices" and for which the period until the reexamination is completed exceeds five years, the date on which the reexamination is completed.

2) The date on which 3 years have elapsed since the discontinuation or termination of the clinical trial.

The investigator shall notify the clinicalsite when the records to be retained by the investigational site or the investigational review committee are no longer required to be retained.

15.2. Retention of records by principal investigators

Documents or records pertaining to clinical trials to be retained by principal investigators as stipulated in the Pharmaceutical GCP Ordinance shall be retained at a storage location deemed appropriate until the later of the following dates.

1) Three years have elapsed since the date of marketing approval for the relevant indication of the test product (if development has been discontinued, three years have elapsed since the date on which the decision to discontinue development was made).
2) The date on which 3 years have elapsed since the discontinuation or termination of the clinical trial.

16. RETENTION OF SAMPLES AND USE OF SAMPLES PROVIDED BY OTHER INSTITUTIONS

16.1. Retention of sample

Samples will be stored in the Laboratory of Gastroenterology, Graduate School of Medicine, Chiba University for a period not exceeding 20 years after completion of the clinical trial. The method of preservation shall be cryopreservation using liquid nitrogen.

16.2. Disposal of samples

If a subject withdraws consent, if a specimen is mistaken or contaminated or is strongly suspected of being mistaken or contaminated, or if the need for disposal is otherwise recognized, the anonymizing numbers, etc., will be deleted and the specimen will be disposed of.

16.3. Reuse of samples

Secondary use of samples and sample-related information (genomic or epigenomic analysis at Chiba University) may be conducted. In such cases, the ethical review and method of obtaining consent shall be in accordance with the corresponding ethical guidelines, etc.

17. SOURCE DOCUMENT VERIFICATION

The head of the investigational site and the investigator ensure that the personnel in charge of monitoring, audits and Institutinal Review Board or regulatory authorities have access to all records, including source documents. In anddtion , The head of the investigational site and the investigator confirm that the clinical trial is conducted appropriately and that the data are sufficiently reliable. The method and timing of source document verification shall be specified separately in the monitoring procedures.

18. Quality Assurance

In order to ensure that clinical trials are conducted and that data preparation, recording and reporting are appropriately conducted in compliance with the protocol and the GCP ordinances for pharmaceutical products, independent auditors from the departments related to the clinical trials, including the department in charge of monitoring, will conduct audits at the investigational

site and other sites where the clinical trials are conducted to confirm that quality control is appropriately conducted.The audit shall be conducted by an auditor independent from the departments related to the clinical trial, including the department in charge of monitoring, to confirm that quality control is appropriately implemented. Audits shall be conducted in accordance with the “Standard Operating Procedures for Audits” and “Audit Plan” separately stipulated.

19. QUALITY CONTROL FOR THIS STUDY

19.1 Training of study site personnel

The Principal Investigator will ensure that appropriate training relevant to the study is given to all of these staff, and that any new information relevant to the performance of this study is forwarded to the staff involved.

19.2. Quality control

In conducting clinical trial monitoring, the investigator shall consider priorities and develop a systematic risk-based approach. In the event of any deviation from this protocol, the investigator or subinvestigator shall follow the provisions of this protocol. The investigator or subinvestigator shall prepare the case report form in accordance with this protocol. The investigator will ensure that all data and other records in the case report form are accurate and complete. If any of the data in the case report form is inconsistent in any way with the original data, the investigator shall prepare and maintain a record explaining the reason for the inconsistency. The investigator shall designate a person who is not engaged in the relevant clinical trial at the site subject to the monitoring as a monitor and have him/her conduct the monitoring in accordance with the monitoring protocol that has been reviewed by the investigational review committee. The monitors shall confirm the following items in accordance with the monitoring protocol separately prepared.

- The human rights, safety and welfare of subjects are protected.
- The clinical trial is conducted in compliance with the Pharmaceutical GCP ordinance, the latest clinical trial protocol, and the procedure manual for the relevant clinical trial.
- To confirm that the data, etc. reported by the investigator or subinvestigator are accurate and complete and check them against the source documents and other clinical trial-related records.

The person in charge of data management shall formulate the data management plan in accordance with the separately established standard operating procedures and shall ensure the quality of the data through quality control at each stage of data handling.

20. ETHICAL CONDUCT AND GOOD CLINICAL PRACTICE(GCP)

This clinical trial will be conducted in accordance with the “Declaration of Helsinki”,the “Law Concerning Quality, Efficacy and Safety Assurance of Pharmaceuticals and Medical Devices” and the “Pharmaceutical GCP Ministerial Ordinance”. In addition, this clinical trial shall be conducted in compliance with the protocol and procedures for this clinical trial.

In selecting subjects, the investigator or subinvestigator shall carefully consider the appropriateness of requesting subjects to participate in this clinical trial based on the selection criteria and exclusion criteria from the perspective of protecting human rights, taking into consideration the subjects' health condition, symptoms, age, gender, ability to consent, degree of dependence on the investigator, and participation status in clinical trials including other clinical trials.

21. INSTITUTIONAL REVIEW BOARD(IRB)

Prior to the implementation of this clinical trial, the Institutional Review Board of the site will review the ethical, scientific and medical appropriateness of this clinical trial. This clinical trial shall be conducted after obtaining the approval of the Clinical Trial Review Committee. If the result of the deliberation by the Institutional Review Board is “Approval with modification,” the protocol or case report form, consent document, etc. shall be modified and approved based on the result of the deliberation, and then this clinical trial shall be conducted. The Institutional Review Board shall also continuously review whether or not this clinical trial is being conducted appropriately at least once a year.

22. HEALTH DAMAGE COVERAGE AND INSURANCE

If a subject suffers health problems as a result of participation in this clinical trial, the investigator will provide treatment and appropriate medical care for the subject's recovery.

As a response to liability for compensation and indemnification arising from health damage caused by this clinical trial, the investigators, subinvestigators, medical institution, Clinical Trial Coordinating Committee and other parties involved in this clinical trial will be covered by the Physician-initiated Clinical Trial Insurance.

23. COST BURDEN FOR THIS TRIAL

The investigational drug to be used in this study will be provided by AstraZeneca. Payment of the burden reduction fee and other expenses to subjects will be in accordance with the rules separately established by each investigational site.

24. TRIAL FUNDS AND CONFLICT OF INTEREST

This clinical trial will be conducted with funds provided to Chiba University Hospital by AstraZeneca. AstraZeneca personnel will not be involved in the conduct or analysis of this clinical trial when the investigators conduct the clinical trial.

In addition, prior to the deliberation of the Clinical Trial Review Committee at each site, the Conflict of Interest Management Committee will deliberate whether conflicts of interest are being properly managed, and it will be confirmed that the investigators and collaborators are not in a state of conflict of interest.

25. PROVISION OF THE INVESTIGATIONAL PRODUCTS AND INTELLECTUAL PROPERTY RIGHTS

In this study, the investigational products are provided by AstraZeneca Pharmaceuticals, Inc. (England) The ownership of the rights to any inventions, discoveries, or improvements of any nature (the “Inventions”) derived from this study will be decided in accordance with the contracts with AstraZeneca.

26.PUBLICATION

The results of the clinical trial will be submitted as a report by the Principal Investigator to the head of the clinical trial site upon completion of this clinical. Results that do not meet expected outcomes despite the proper conduct of the clinical trial must also be disclosed in publication. In case of disclosure of the results to the public, the subject’s personal information must be kept confidential.

27. REGISTRATION FOR CLINICAL TRIAL

This clinical trial will be registered in Japan Registry of Clinical Trial(jRCT) (https://jrct.niph.go.jp/) prior to obtaining consent from the first subject.

28. STUDY IMPLEMANTATION GRPUP

See ANNEX TO PROTOCOL 1

29. LIST OF INVESTIGATIONAL PRODUCT(S) FOR THIS STUDY

Investigational product	Dosage form and strength	Manufacturer
Durvalumab	*50 mg/mL solution for infusion after dilution*	MedImmune/AstraZeneca
Tremelimumab	*20 mg/mL solution for infusion after dilution*	MedImmune/AstraZeneca

30. LIST OF REFERENCES

1) Ministry of Health, Labor and Welfare 2014 Patient Survey
2) Ministry of Health, Labor and Welfare 2017 Vital Statistics
3) El Serag HB, et al., Hepatocellular Carcinoma N Engl J Med 2011
4) The Japan Society of Hepatology Clinical Practice Guidelines for Hepatocellular Carcinoma
5) Llovet JM, Ricci S, Mazzaferro V, et al. Sorafenib in advanced hepatocellular carcinoma. N Engl J Med 2008; 359: 378-90.
6) Cheng AL, Kang YK, Chen Z, et al. Efficacy and safety of sorafenib in patients in the Asia-Pacific region with advanced hepatocellular carcinoma: a phase III randmised, double- blind, placebo-controlled trial. Lancet Oncol 2009; 10:25-34.
7) Bruix J, Qin S, Merle P, et al. Regorafenib for patients with hepatocellular carcinoma who progressed on sorafenib treatment (RESORCE): a randmised, double-blind, placebo-controlled, phase 3 trial. Lancet 2017; 389: 56-66.
8) Kudo M, Finn RS, Qin S, et al. Lenvatinib versus sorafenib in first-line treatment of patients with unresectable hepatocellular carcinoma: a randomised phase 3 non-inferiority trial. Lancet 2018 Mar 24, 391 (10126): 1163-1173
9) Zhu AX, Kan YK, Yen CJ, et al. Ramucirumab after sorafenib in patients with advanced hepatocellular carcinoma and increased α-fetoprotein concentrations (REACH-2): a randomised, double-blind, placebo-controlled, phase 3 trial. Lancet Oncol.2019 Feb;20(2):282-296.
10) Bonze D, Meirson T, Azoulay D. Atezolizumab and Bevacizumab in Hepatocellular Carcinoma. NEJM. 2020 Aug 13;383(7):693-694.
11) Costentin CE, Ferroone CR, Arellano RS, et al. Hepatocellular Carcinoma with Macrovascular Invasion: Defining the Optimal Treatment Strategy Liver Cancer 2017; Nov;6(4):360-374.
12) Dunn GP, Old LJ, Schreiber RD. The three Es of cancer immunoediting. Annu Rev Immunol 2004;22:329-60.
13) Keir ME, Butte MJ, Freeman GJ, Sharpe AH. PD-1 and its ligands in tolerance and immunity. Annu Rev Immunol. 2008;26:677-704.
14) Okazaki T, Honjo T. PD-1 and PD-1 ligands: from discovery to clinical application. Int Immunol 2007;19(7):813-824.
15) Qin A, Coffey DG, Warren EH, Ramnath N. Mechanisms of immune evasion and current status of checkpoint inhibitors in non-small cell lung cancer. Cancer Med 2016;9:2567–2578.
16) Pardoll DM. The blockade of immune checkpoints in cancer immunotherapy. Nat Rev Cancer 2012;12:252-64.
17) Brahmer JR, Tykodi SS, Chow LQM, Hwu WJ, Topalian SL, Hwu P, et al. Safety and activity of

anti-PD-L1 antibody in patients with advanced cancer. N Engl J Med. 2012 Jun;366 (26):2455-65.

18) Hirano F, Kaneko K, Tamura H, Dong H, Wang S, Ichikawa M, et al. Blockade of B7-H1 and PD-1 by monoclonal antibodies potentiates cancer therapeutic immunity. Cancer Res. 2005;65(3):1089-96.

19) Iwai Y, Ishida M, Tanaka Y, Okazaki T, Honjo T, Minato N. Involvement of PD-L1 on tumor cells in the escape from host immune system and tumor immunotherapy by PD-L1 blockade. Proc Natl Acad Sci USA. 2002 Sep 17;99:12293-7.

20) Okudaira K, Hokari R, Tsuzuki Y, Okada Y, Komoto S, Watanabe C, et al. Blockade of B7-H1 or B7-DC induces an anti-tumor effect in a mouse pancreatic cancer model. Int J Oncol. 2009 Sep;35(4):741-9.

21) Topalian SL, Hodi FS, Brahmer JR, Gettinger SN, Smith DC, McDermott DF, et al. Safety, activity, and immune correlates of anti-PD-1 antibody in cancer. N Engl J Med. 2012;366:2443-54.

22) Zhang C, Wu S, Xue X, Li M, Qin X, Li W, et al. Anti-tumor immunotherapy by blockade of the PD-1/PD-L1 pathway with recombinant human PD-1-IgV. Cytotherapy. 2008;10(7):711-9.

23) Powles T, Eder JP, Fine GD, Braiteh FS, Loriot Y, Cruz C, et al. MPDL3280A (anti-PD-L1) treatment leads to clinical activity in metastatic bladder cancer. Nature. 2014 Nov 27;515(7528):558-62.

24) Rizvi N, Brahmer J, Ou S-H, Segal NH, Khleif SN, Hwu WJ. Safety and clinical activity of MEDI4736, an anti-programmed cell death-ligand-1 (PD-L1) antibody, in patients with nonsmall cell lung cancer (NSCLC). J Clin Oncol 2015;33:Abstract 8032.

25) Segal NH, Ou S-HI, Balmanoukian AS, Fury MG, Massarelli E, Brahmer JR, et al. Safety and efficacy of MEDI4736, an anti-PD-L1 antibody, in patients from a squamous cell carcinoma of the head and neck (SCCHN) expansion cohort. J Clin Oncol 2015;33:Abstract 3011.

26) Alexandrov LB, Nik-Zainal S, Wedge DC, Aparicio SAJR, Behjati S, Blankin AV, et al. Signatures of mutational processes in human cancer. Nature. 2013 Aug 22;500:415-21.

27) Fife BT, Bluestone JA. Control of peripheral T-cell tolerance and autoimmunity via the CTLA-4 and PD-1 pathways. Immunol Rev. 2008;224:166-82.

28) El-Khoueiry AB, Sangro B, Yau T, et al. Nivolumab in patients with advanced hepatocellular carcinoma (CheckMate 040): an open-label, non-comparative, phase 1/2 dose escalation and expansion trial Lancet 2017; Jun 24;389(10088):2492-2502.

29) Zhu AX, Finn RS, Edeline J, et al. Pembrolizumab in patients with advanced hepatocellular carcinoma previously treated with sorafenib (KEYNOTE-224): a non-randomised, open-label phase 2 trial. Lancet Oncol. 2018; Jul;19(7):940-952.

30) Kudo M. Targeted and immune therapies for hepatocellular carcinoma: Predictions for 2019 and beyond. World J Gastroenterol 2019 Feb 21;25(7):789-807.

31) Kelly RK, Abou-Alfa GK, Bendell JC, et al. Phase I/II study of durvalumab and tremelimumab in patients with unresectable hepatocellular carcinoma (HCC): Phase I safety and efficacy analyses. J Clin Oncol.2017; Abstract 4073.

32) Pedroni E, Bacher R, Blattmann H, Böhringer T, Coray A, Lomax A, Lin S, Munkel G, Scheib S, Schneider U, et al. The 200-MeV proton therapy project at the Paul Scherrer Institute: conceptual design and practical realization. Med Phys. 1995 Jan;22(1):37-53.

33) Mohamad O., Makishima H., Kamada T. Evolution of Carbon Ion Radiotherapy at the National Institute of Radiological Sciences in Japan. Cancers (Basel) 2018;10(3): pii: E66

34) Kasuya G, Kato H, Yasuda S, Tsuji H, Yamada S, Haruyama Y, Kobashi G, Ebner DK, Okada NN, Makishima H, Miyazaki M, Kamada T, Tsujii H; Liver Cancer Working Group. Progressive

hypofractionated carbon-ion radiotherapy for hepatocellular carcinoma: Combined analyses of 2 prospective trials. Cancer. 2017;123(20):3955-3965

35) Fukuda K, Okumura T, Abei M, Fukumitsu N, Ishige K, Mizumoto M et al. Long-term outcomes of proton beam therapy in patients with previously untreated hepatocellular carcinoma. Cancer Sci. 2017;108(3):497-503

36) Sorin Y, Ikeda K, Kawamura Y, Fujiyama S, Kobayashi M, Hosaka T. Effectiveness of Particle Radiotherapy in Various Stages of Hepatocellular Carcinoma: A Pilot Study. Liver Cancer. 2018 Oct;7(4):323-334

37) Igaki H, Mizumoto M, Okumura T, Hasegawa K, Kokudo N, Sakurai H. A systematic review of publications on charged particle therapy for hepatocellular carcinoma. Int J Clin Oncol. 2018;23(3):423-433

38) Komatsu S, Fukumoto T, Demizu Y, Miyawaki D, Terashima K, Niwa Y, Mima M, Fujii O, Sasaki R, Yamada I, Hori Y, Hishikawa Y, Abe M, Ku Y, Murakami M. The effectiveness of particle radiotherapy for hepatocellular carcinoma associated with inferior vena cava tumor thrombus. J Gastroenterol. 2011 Jul;46(7):913-20.

39) Lee SU, Park JW, Kim TH, Kim YJ, Woo SM, Koh YH, Lee WJ, Park SJ, Kim DY, Kim CM. Effectiveness and safety of proton beam therapy for advanced hepatocellular carcinoma with portal vein tumor thrombosis. Strahlenther Onkol. 2014 Sep;190(9):806-14.

40) Sugahara S, Nakayama H, Fukuda K, Mizumoto M, Tokita M, Abei M, Shoda J, Matsuzaki Y, Thono E, Tsuboi K, Tokuuye K. Proton-beam therapy for hepatocellular carcinoma associated with portal vein tumor thrombosis. Strahlenther Onkol 2009;185:782–788.

41) Antonia SJ, Daniel VD, Vicente D, et al. Durvalumab after Chemoradiotherapy in Stage III Non-Small-Cell Lung Cancer. N Engl J Med. 377;1919-29. 2017

42) Rizvi NA, Cho BC, Reinmuth N, et al. Durvalumab with or without tremelimumab vs platinum-based chemotherapy as first-line treatment for metastatic non-small cell lung cancer: MYSTIC Annals of Oncology.2018 Dec 1;29;supp10

43) Helm A, Ebner DK, Tinganelli W, Simoniello P, Bisio A, Marchesano V, Durante M, Yamada S, Shimokawa T. Combining Heavy-Ion Therapy with Immunotherapy: An Update on Recent Developments. Int J Part Ther. 2018 Summer;5(1):84-93.

44) Takahashi Y, Yasui T, Minami K, Tamari K, Hayashi K, Otani K, Seo Y, Isohashi F, Koizumi M, Ogawa K. Carbon ion irradiation enhances the antitumor efficacy of dual immune checkpoint blockade therapy both for local and distant sites in murine osteosarcoma. Oncotarget. 2019 Jan 18;10(6):633-646.

45) Iijima M, Okonogi N,Izumi Nakajima N, Morokoshi Y, Kanda H, Yamada T, Kobayashi Y, Banno K, Wakatsuki M, Yamada S, Kamada T, Aoki D, Hasegawa S. Significance of PD-L1 expression in carbon-ion radiotherapy for uterine cervical adeno/adenosquamous carcinoma. J Gynecol Oncol. 2020;31:e19.

46) Golden EB, Pellicciotta I, Demaria S, et al. The convergence of radiation and immunogenic cell death signaling pathways. Front Oncol, 2012 Aug 7;2:88.

47) Onishi M, Okonogi N, Oike T, Yoshimoto Y, Sato H, Suzuki Y, Kamada T, Nakano T. High linear energy transfer carbon-ion irradiation increases the release of the immune mediator high mobility group box 1 from human cancer cells. J Radiat Res. 2018 Sep 1;59(5):541-546.

48) Matsunaga A, Ueda Y, Yamada S, Harada Y, Shimada H, Hasegawa M, Tsujii H, Ochiai T, Yonemitsu Y. Carbon-ion beam treatment induces systemic antitumor immunity against murine squamous cell carcinoma. Cancer. 2010 Aug 1;116(15):3740-8.

49) Ando K, Fujita H, Hosoi A, Ma L, Wakatsuki M, Seino KI, Kakimi K, Imai T, Shimokawa T,

Nakano T. Intravenous dendritic cell administration enhances suppression of lung metastasis induced by carbon-ion irradiation. J Radiat Res. 2017 Jul 1;58(4):446-455.

50) Sato H, Niimi A, Yasuhara T, Permata TBM, Hagiwara Y, Isono M, Nuryadi E, Sekine R, Oike T, Kakoti S, Yoshimoto Y, Held KD, Suzuki Y, Kono K, Miyagawa K, Nakano T, Shibata A. DNA double-strand break repair pathway regulates PD-L1 expression in cancer cells. Nat Commun. 2017 Nov 24;8(1):1751. doi: 10.1038/s41467-017-01883-9.

51) Oike T, Niimi A, Okonogi N, Murata K, Matsumura A, Noda SE, Kobayashi D, Iwanaga M, Tsuchida K, Kanai T, Ohno T, Shibata A, Nakano T. Visualization of complex DNA double-strand breaks in a tumor treated with carbon ion radiotherapy. Sci Rep. 2016 Mar 1;6:22275. doi: 10.1038/srep22275.

52) Stewart R, Morrow M, Hammond SA, Mulgrew K, Marcus D, Poon E, et al. Identification and characterization of MEDI4736, an antagonistic anti-PD-L1 monoclonal antibody. Cancer Immunol Res 2015;3(9):1052-62.

53) Tarhini AA, Kirkwood JM. Tremelimumab (CP-675,206): a fully human anticytotoxic T lymphocyte-associated antigen 4 monoclonal antibody for treatment of patients with advanced cancers. Expert Opin Biol Ther 2008;8:1583-93.

54) Pardee AD, Butterfield LH. Immunotherapy of hepatocellular carcinoma: Unique challenges and clinical opportunities. Oncoimmunology 2012;1(1):48-55.

55) Gao Q, Wang XY, Qiu SJ, Yamato I, Sho M, Nakajima Y, et al. Overexpression of PD-L1 significantly associates with tumor aggressiveness and postoperative recurrence in human hepatocellular carcinoma. Clin Cancer Res 2009;15(3):971-9.

56) Hato T, Goyal L, Greten TF, Duda DG, Zhu AX. Immune checkpoint blockade in hepatocellular carcinoma: current progress and future directions. Hepatology 2014;60(5):1776-82.

57) Miroux C, Vausselin T, Delhem N. Regulatory T cells in HBV and HCV liver diseases: implication of regulatory T lymphocytes in the control of immune response. Expert Opin Biol Ther 2010;10(11):1563-72.

58) Golden-Mason L, Palmer B, Klarquist J, Mengshol JA, Castelblanco N, Rosen HR. Upregulation of PD-1 expression on circulating and intrahepatic hepatitis C virus-specific CD8+ T cells associated with reversible immune dysfunction. J Virol 2007;81(17):9249-58.

59) Peng G, Li S, Wu W, Tan X, Chen Y, Chen Z. PD-1 upregulation is associated with HBV-specific T cell dysfunction in chronic hepatitis B patients. Mol Immunol 2008;45(4):963-70.

60) Klein JP, Logan B, Harhoff M, Andersen PK. Analyzing survival curves at a fixed point in time. Stat Med 2007;26(24):4505-19.

61) Sangro B, Gomez MC, Mata M, et al. A clinical trial of CTLA-4 blockade with tremelimumab in patients with hepatocellular carcinoma and chronic hepatitis Cq. J Hepatol. 2013 Jul;59(1):81-8.

62) Duffy AG, Ulahannan SV, Makorova-Rusher O, Rahmer O, Wedemeyer H, Pratt D, et al. Tremelimumab in combination with ablation in patients with advanced hepatocellular carcinoma. J Hepatol 2017;66(3):545-51.

63) Melero I, Sangro B, Cheung Yae T, Hsu C, Kudo M, Crocenzi TS, et al. Nivolumab dose escalation and expansion in patients with advanced hepatocellular carcinoma (HCC): The CheckMate 040 study. J Clin Oncol 2017;35 (suppl 4S:abstract 226).

64) Kelly RK, Sangro B, Harris W, et al. Efficacy, tolerability, and biologic activity of a novel regimen of tremelimumab in combination with durvalumab for patients with advanced hepatocellular carcinoma, J Clin Oncol. 2020; Abstract 4508.

65) Takahashi Y, Fujikawa K, Sagawa T, et al. A phase 1 study to assess the safety and tolerability of tremelimumab alone and in combination with MEDI4736 in Japanese patients withadvanced solid

malignancies. Eur J Cancer. 2015(suppl 3; abst512)

66) Wang E, Kang D, Bae KS, Marshall MA, Pavlov D, Parivar K. Population pharmacokinetic and pharmacodynamics analysis of tremelimumab in patients with metastatic melanoma. J Clin Pharmacol 2014;54(10):1108-16.

67) Narwal R, Roskos LK, Robbie GJ. Population pharmacokinetics of sifalimumab, an investigational anti-interferonalpha monoclonal antibody, in systemic lupus erythematosus. Clin Pharmacokinet 2013;52:1021–27.

68) Ng CM, Lum BL, Gimenez V, Kelsey S, Allison D. Rationale for fixed dosing of pertuzumab in cancer patients based on population pharmacokinetic analysis. Pharm Res 2006;23(6):1275–84.

69) Wang DD, Zhang S, Zhao H, Men AY, Parivar K. Fixed dosing versus body size based dosing of monoclonal antibodies in adult clinical trials. J Clin Pharmacol 2009;49(9):1012–24.

70) Wolchok JD, Kluger H, Callahan MK, Postow MA, Rizvi NA, Lesokhin AM, et al. Nivolumab plus ipilimumab in advanced melanoma. N Engl J Med 2013;369:122-33.

71) Reed GB Jr, Cox AJ Jr. The human liver after radiation injury. A form of veno-occlusive disease. Am J Pathol. 1966 Apr;48(4):597-611.

72) Kanai T, Endo M, Minohara S, Miyahara N, Koyama-ito H, Tomura H, Matsufuji N, Futami Y, Fukumura A, Hiraoka T, Furusawa Y, Ando K, Suzuki M, Soga F, Kawachi K. Biophysical characteristics of HIMAC clinical irradiation system for heavy-ion radiation therapy. Int J Radiat Oncol Biol Phys. 1999 Apr 1;44(1):201-10.

73) Shibuya K, Ohno T, Terashima K, Toyama S, Yasuda S, Tsuji H, et al. Short-course carbon-ion radiotherapy for hepatocellular carcinoma: a multi-institutional retrospective study. Liver Int. 2018;38(12):2239-47.

74) Yasuda S, Kato H, Imada H, et al. Long-Term Results of High-Dose 2-Fraction Carbon Ion Radiation Therapy for Hepatocellular Carcinoma. Adv Radiat Oncol. 2019 Sep 27;5(2):196-203.

75) Shiba S, Shibuya K, Katoh H, et al. A comparison of carbon ion radiotherapy and transarterial chemoembolization treatment outcomes for single hepatocellular carcinoma: a propensity score matching study Radiat Oncol. 2019 Aug 2;14(1):137.

76) Shiba S, Shibuya K, Okamoto M, et al. Clinical impact of Hypofractionated carbon ion radiotherapy on locally advanced hepatocellular carcinoma. Radiat Oncol 2020 Aug 14;15(1):195.

77) Makishima S, Yasuda S, Isozaki Y, et al. Single fraction carbon ion radiotherapy for colorectal cancer liver metastasis: A dose escalation study. Cancer Sci. 2019 Jan;110(1):303-309.

78) Ebner DK, Tinganelli W, Helm A, Bisio A, Yamada S, Kamada T, Shimokawa T, Durante M. The immunoregulatory potential of particle radiation in cancer therapy. Front Immunol. 2017;8:1–8.
