# Supplementary material for: MVI-targeted carbon-ion radiotherapy combined with immunotherapy for advanced hepatocellular carcinoma: Phase Ib DEPARTURE trial
Source: JHEP Rep. 2026 Feb 5;8(5):101765. doi: 10.1016/j.jhepr.2026.101765 (PMC13054414; doi:10.1016/j.jhepr.2026.101765)
Supplement: Multimedia component 4 [file mmc4.zip › ClinicalTrial/SAP_ver.1.0.pdf]

A phase Ib study of durvalumab (MEDI4736) ± tremelimumab combined with Carbon ion radiotherapy in advanced hepatocellular carcinoma patients with macrovascular invasion

[DEPARTURE trial]

Phase Ib study

statistical analysis plan

Principal Investigator (Representative):

Sadahisa Ogasawara

Department of Gastroenterology, Chiba University Hospital

Approval Date： Apl.20,2021

Responsible Person for Statistical Analysis:

Yohei Kawasaki

Biostatistics Office, Chiba University Hospital Clinical Research Center

Revision History

Apr.19,2021, ver.1.0

Table of Contents

1. Purpose of the Study 3
 1.1. Primary Endpoint 3
 1.2. Secondary Endpoints 3
2. General Matters in Statistical Analysis 3
 2.1. Interim Analysis 3
 2.2. Data Handling 3
 2.3. Significance Level and Confidence Level 4
 2.4. Adjustment for Multiplicity 4
 2.5. Subgroup Analysis 4
 2.6. Data Monitoring 4
 2.7. Common Rules for Data Processing 4
 2.8. Statistical Analysis Software 5
 2.9. General Formatting of Figures and Tables 5
3. Analysis Populations 5
 3.1. Definition of Analysis Populations 5
  3.1.1. DLT Analysis Set 5
  3.1.2. Safety Analysis Set (SAF) 6
  3.1.3. Full Analysis Set (FAS) 6
  3.1.4. Efficacy Evaluable Set (EES) 6
  3.1.5. Per Protocol Set (PPS) 6
 3.2. Correspondence with Statistical Analysis Items 6
4. Analysis Plan for Subject Breakdown 6
 4.1. Subject Breakdown 6
 4.2. Aggregation of Discontinued or Interrupted Cases 7
5. Analysis Plan for Subject Background 7
6. Analysis Plan for Primary Endpoint 7
7. Safety Evaluation 7
8. Analysis Plan for Secondary Endpoints 7
9. Analysis Plan for Other Items 8
10. Revision History 9
 10.1. Changes from the Clinical Trial Protocol 9
 10.2. Revision History of the Statistical Analysis Plan 9
11. Statistical Analysis Implementation System and Environment 9
 11.1. Responsible Person for Statistical Analysis 9
 11.2. Persons in Charge of Statistical Analysis 9

1. Purpose of the Study

Primary Objective
To evaluate the tolerability and safety of the combination therapy of Durvalumab, Tremelimumab, and carbon ion radiotherapy in patients with advanced hepatocellular carcinoma with vascular invasion.

Secondary Objective
To evaluate the efficacy of the combination therapy of Durvalumab, Tremelimumab, and carbon ion radiotherapy in patients with advanced hepatocellular carcinoma with vascular invasion.

1.1. Primary Endpoint

Adverse events/serious adverse events including dose-limiting toxicity (DLT).

1.2. Secondary Endpoints

1. Overall survival
2. 6-month survival rate
3. RECIST ver 1.1
4. Objective response rate (ORR) calculated by mRECIST
5. 6-month progression-free survival rate (PFS)
6. Progression-free survival period

Safety

1. Incidence of adverse events
2. Incidence of serious adverse events
3. Adverse events coded by MedDRA
4. List of SOC, PT severity, and relevance

2. General Matters in Statistical Analysis

2.1. Interim Analysis

No interim analysis will be conducted in this study.

2.2. Data Handling

Data handling during data aggregation and analysis will be as follows:

1. Missing values: Missing values will not be imputed.
2. Reference values: Data with low reliability due to hemolysis, etc., will not be used in aggregation analysis.
3. Timing discrepancies: Data not collected at the specified observation time will not be used in aggregation analysis.
4. Data conversion: Data conversion will not be performed.

2.3. Significance Level and Confidence Level

The significance level used in the test will be 5% (two-sided) or 2.5% (one-sided), and the confidence interval will be 95% (two-sided).

2.4. Adjustment for Multiplicity

No adjustment for multiplicity will be performed.

2.5. Subgroup Analysis

Subgroup analysis will not be performed.

2.6. Data Monitoring

Data monitoring will be established to ensure the safety of subjects as specified in the clinical trial protocol.

2.7. Common Rules for Data Processing

Rules regarding days and periods:

- The number of days is calculated by subtracting the start date from the end date and adding 1.
    - Example:
        - Medication days: If the medication is started and ended on the same day, the medication period is 1 day.
        - Period until the occurrence of an adverse event: If an adverse event occurs the day after the start of medication, the period until the first occurrence of the adverse event is 2 days.
        - Survival period: If the subject dies the day after the start of medication, the survival period is 2 days.
    - When converting days to years, months, or weeks, unless otherwise specified, 1 year is 365.25 days, 1 month is 30.4375 days, and 1 week is 7 days (age is not applied as an example).
    - When displaying the period from the start of medication (days) or the time of occurrence (days), the start date of medication is 1, the day before the start date is -1, and so on, counting up or down by 1 day. There is no 0 day in the period from the start of medication.

Rules for Decimal Places and Rounding

- The mean, standard deviation, and median are displayed to one decimal place below the raw data (rounding to two decimal places), but the final number of decimal places is adjusted considering significant figures.
- Percentages are displayed to one decimal place (xx.x%), rounding to two decimal places. However, for percentage displays of 100% and 0%, mixed integer displays are also considered.
- When calculating statistical quantities such as mean, standard deviation, and median, rounding is not performed during the calculation process, and the final calculation result is rounded. However, when handling ranks, rounding is performed just before handling rank data to avoid rank errors due to calculation errors.

- Example:
    - 1 ÷ 3 × 3 = 1, but very small errors such as 0.9999999999999999 may occur due to calculators or calculation logic. Ranks occur between separately entered values of 1 (exact 1.00000000). In this case, rounding (1/3*3) to an appropriate number of decimal places, such as eight decimal places, ensures the same rank.

Confidence Interval for Incidence Rates

- The confidence interval for incidence rates (or incidence rates) and response rates (or response rates) based on the binomial distribution is calculated using the exact method based on the F distribution, Clopper-Pearson (1934) [a)].
- The confidence interval for incidence rates in survival time analysis is calculated using Greenwood's formula, Kalbfleisch and Prentice (1980) [b)]. The confidence interval for the median survival time in survival time analysis is calculated using the method of Brookmeyer and Crowley (1982) [c)]. The estimated value and confidence interval for the mean survival time up to 6 months in survival time analysis are calculated using the method of Lee (1992) [d)] based on the restricted mean survival time (RMST).

a) Clopper, C. and Pearson,E. S. (1934). The use of confidence or fiducial limits illustrated in the case of the binomial. Biometrika 26 (4): 404-413.
b) Kalbfleisch, J. D., and Prentice, R. L. (1980). The Statistical Analysis of Failure Time Data. New York: John Wiley & Sons.
c) Brookmeyer, R., and Crowley, J. (1982). "A Confidence Interval for the Median Survival Time." Biometrics 38:29-41
d) Lee, E.T. (1992), Statistical Methods for Survival Data Analysis, Second Edition, New York: John Wiley & Sons.

2.8. Statistical Analysis Software

The analysis will primarily use SAS 9.4. Outputs will be generated using Microsoft Office.

2.9. General Formatting of Figures and Tables

The default paper size is A4. Figures and summary tables will be in portrait orientation, while lists and data tables will be in landscape orientation, depending on the characteristics of individual figures and tables. Outputs will be selected as appropriate in Excel, Word, PDF, etc.

3. Analysis Populations

3.1. Definition of Analysis Populations

3.1.1. DLT Analysis Set

All subjects enrolled in this study who received at least one regimen of the investigational drug (IR) and were evaluated for DLT will be included in the DLT analysis set.

3.1.2. Safety Analysis Set (SAF)

All subjects who received at least one regimen of the investigational drug or investigational drug regimen (IR) will be included in the Safety Analysis Set.

3.1.3. Full Analysis Set (FAS)

All subjects who have the target disease of this study, are correctly enrolled, and have received at least one regimen of the investigational drug will be included in the Full Analysis Set (FAS).

3.1.4. Efficacy Evaluable Set (EES)

The Efficacy Evaluable Set (EES) is defined as a subset of the FAS. It consists of all subjects who are eligible for the study, have received the investigational drug/IR, and have had at least one efficacy endpoint evaluated after administration. This group includes cases where efficacy evaluation was performed at least once after baseline and administration, as well as cases of early death or early progression before evaluation.

3.1.5. Per Protocol Set (PPS)

Subjects in the FAS who do not have major protocol violations such as deviations from study methods or prohibited concomitant therapies will be included in the Per Protocol Set (PPS). Major violations include:

- Violation of inclusion criteria
- Violation of exclusion criteria
- Violation of prohibited concomitant medications
- Violation of prohibited concomitant therapies

3.2. Correspondence with Statistical Analysis Items

The analysis of the primary endpoint, DLT evaluation, will be conducted using the DLT analysis set. For efficacy evaluation, the primary analysis will be conducted using the Full Analysis Set (FAS), but the results will be presented as the Efficacy Evaluable Set (EES), which excludes missing values from the FAS. Additionally, analyses will be conducted using the Per Protocol Set (PPS) as needed. Safety analyses other than DLT evaluation will be conducted using the Safety Analysis Set.

4. Analysis Plan for Subject Breakdown

4.1. Subject Breakdown

The breakdown of subjects will be aggregated and the frequency and percentage will be shown for

the following items. The results will be presented in figures and tables:

- DLT Analysis Set
- Safety Analysis Set (SAF)
- Full Analysis Set (FAS)
- Efficacy Evaluable Set (EES)
- Per Protocol Set (PPS)

4.2. Aggregation of Discontinued or Interrupted Cases

For all cases where the study was discontinued or interrupted, a list or aggregation of the frequency and percentage of the discontinuation/interruption criteria will be provided.

5. Analysis Plan for Subject Background

The distribution and summary statistics of subject background data will be calculated for each cohort. For nominal variables, the frequency and percentage of categories will be shown. For continuous variables, summary statistics (number of cases, mean, standard deviation, median, range, interquartile range) will be calculated.

6. Analysis Plan for Primary Endpoint

The incidence of DLT will be calculated for each cohort.

7. Safety Evaluation

1. Incidence of adverse events
2. Incidence of serious adverse events
3. Incidence of related adverse events
4. A list of adverse events coded by MedDRA (including SOC, PT, severity, and relevance)

8. Analysis Plan for Secondary Endpoints

The analysis of secondary endpoints will be conducted to supplement the main analysis results of this study. No adjustment for multiplicity will be performed in the analysis of secondary endpoints.

- The incidence rate at 6 months and the mean survival time and median survival time up to 6 months for the following items will be calculated:

1. Overall survival (OS)

2. 6-month survival rate
3. Objective response rate (ORR)
    - ORR, based on the evaluation using RECIST 1.1 by the investigator, is defined as the percentage of patients who achieved CR or PR at least once during the visit. The evaluation of ORR will use data collected up to PD or, if there is no PD, up to the final evaluable assessment. Patients who discontinued treatment without PD and started subsequent treatment will not be included in the ORR response cases.
4. 6-month progression-free survival rate (PFS)
    - PFS, based on the evaluation using RECIST 1.1 by the investigator, is defined as the period from randomization to objective disease progression or death (regardless of the cause of death if there is no disease progression). For patients who have not shown disease progression or have not died at the time of analysis, the date of the last RECIST 1.1 evaluation will be used as the cutoff. If the patient did not visit for more than two consecutive times and then showed disease progression or died, the date of the last RECIST 1.1 evaluation will be used as the cutoff. If there are no evaluable visit data or baseline data, the cutoff will be Day 1 unless the patient died before the second visit from baseline, in which case the date of death will be considered the event date. PFS is calculated based on the date of imaging/evaluation, not the visit date. If the planned RECIST 1.1 evaluation/imaging is conducted over multiple days, the earliest date showing progression will be considered the progression date. For PFS evaluation cutoff, the final imaging date related to the specific overall effect evaluation will be used.
5. Time to progression (TTP)
    - TTP, based on the evaluation using RECIST 1.1 by the investigator, is defined as the period from randomization to the date of objective tumor progression. Death is not included in the definition of TTP. Patients who died without progression will be censored at the time of death.

9. Analysis Plan for Other Items

Other items such as concomitant medications, concomitant therapies, vital signs, and blood tests will be listed and aggregated as necessary.

10. Revision History

10.1. Changes from the Clinical Trial Protocol

Date	Name	Revisions
NA	Yoshihito Ozawa	NA

10.2. Revision History of the Statistical Analysis Plan

日付	Name	Revisions
Apr. 19, 2021	Yoshihito Ozawa	**Ver. 1.0:** Added a note regarding the calculation of confidence intervals in addition to the estimated values for the analysis plan of secondary endpoints. Also, added a description limiting the Mean Survival Time to 6 months RMST at this point. Supplemented the handling of the Efficacy Evaluable Set (EES) as a subset of the Full Analysis Set (FAS).

11. Statistical Analysis Implementation System and Environment

11.1. Responsible Person for Statistical Analysis

Yohei Kawasaki, Head of the Biostatistics Office, Clinical Trial Department, Chiba University Hospital
(Researcher Number: 90711573)

11.2. Persons in Charge of Statistical Analysis

Yoshito Ozawa, Biostatistics Office, Chiba University Hospital Clinical Research Center
(Researcher Number: 20859441)
Yuki Shiko, Biostatistics Office, Chiba University Hospital Clinical Research Center
(Researcher Number: 00859663)
