# Supplementary material for: MVI-targeted carbon-ion radiotherapy combined with immunotherapy for advanced hepatocellular carcinoma: Phase Ib DEPARTURE trial
Source: JHEP Rep. 2026 Feb 5;8(5):101765. doi: 10.1016/j.jhepr.2026.101765 (PMC13054414; doi:10.1016/j.jhepr.2026.101765)
Supplement: Multimedia component 5 [file mmc5.pdf]

MVI-targeted carbon-ion radiotherapy combined with immunotherapy for advanced hepatocellular carcinoma: Phase Ib DEPARTURE trial

Authors

Sadahisa Ogasawara, Keisuke Koroki, Hirokazu Makishima, …, Hideki Hanaoka, Shigeru Yamada, Hitoshi Ishikawa

Correspondence

ogasawaras@chiba-u.jp (S. Ogasawara).

Graphical abstract

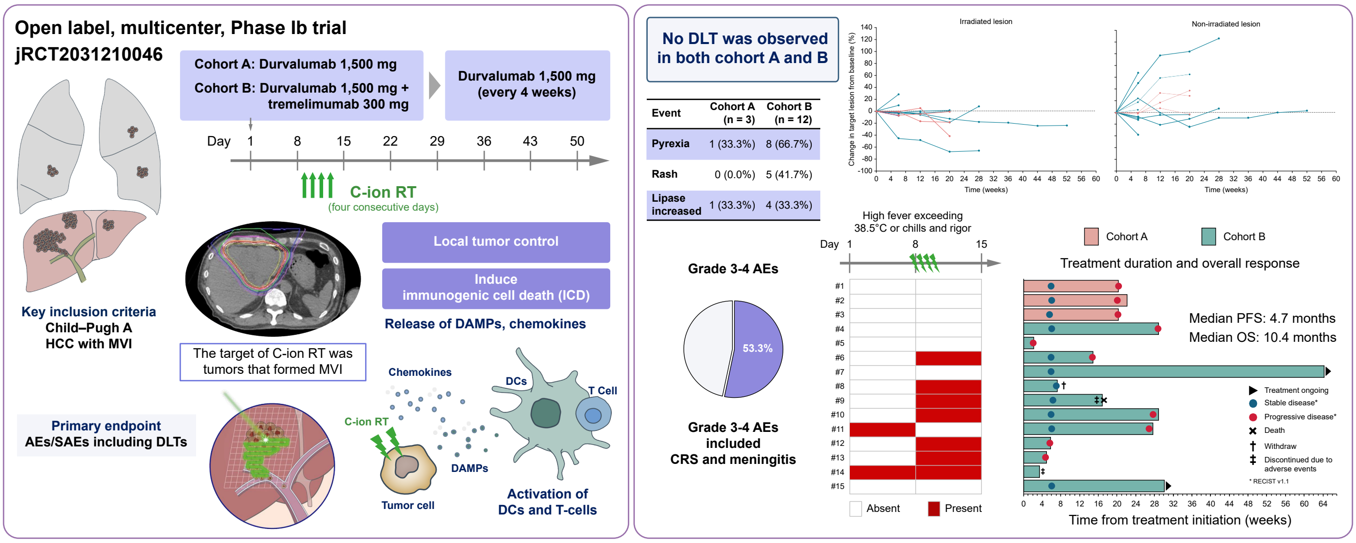

Highlights:

- The trial evaluated the C-ion RT plus ICIs for HCC with macrovascular invasion.
- No DLTs were observed, and the combination showed a manageable safety profile.
- Many patients experienced transient high fevers after C-ion RT.
- The approach achieved safety and effective local control.
- The results highlight the need for further optimization of this combination strategy.

Impact and implications:

Advanced hepatocellular carcinoma with macrovascular invasion (MVI) has a poor prognosis, highlighting the need for new therapeutic strategies. Our phase Ib study suggests that carbon-ion radiotherapy targeting MVI combined with immune checkpoint inhibitors is feasible and achieves sustained local tumor control. RNA-sequencing revealed that immune activation pathways were enriched in responders, while resistance was associated with mesenchymal and angiogenesis signatures. These results reinforce the potential of MVI-targeted irradiation combined with immune checkpoint inhibitors as a promising treatment strategy for these high-risk patients, warranting further investigation to improve systemic tumor control.

https://doi.org/10.1016/j.jhepr.2026.101765
© 2026 The Author(s). Published by Elsevier B.V. on behalf of European Association for the Study of the Liver (EASL). This is an open access article under the CC BY license (http://creativecommons.org/licenses/by/4.0/).

MVI-targeted carbon-ion radiotherapy combined with immunotherapy for advanced hepatocellular carcinoma: Phase Ib DEPARTURE trial

Sadahisa Ogasawara[1,*,†], **Keisuke Koroki**[1,†], **Hirokazu Makishima**[2,†], **Masaru Wakatsuki**[2,†], Asahi Takahashi[3], Makoto Fujiya[1], Sae Yumita[1], Miyuki Nakagawa[1], Hiroaki Kanzaki[1,4], Kazufumi Kobayashi[1], Masanori Inoue[1], Masato Nakamura[1], Naoya Kanogawa[1], Takayuki Kondo[1], Shingo Nakamoto[1], Tomoya Kurokawa[3], Yoshihito Ozawa[3], Yosuke Inaba[3], Soumith Paritala[4], Jingxuan Chen[5], Jeon Lee[5], Yujin Hoshida[4], Hideki Hanaoka[3], Shigeru Yamada[2], Hitoshi Ishikawa[2]

JHEP Reports **2026**. vol. 8 | 1–14

Check for updates

Background & Aims: Advanced hepatocellular carcinoma (HCC) with macrovascular invasion (MVI) carries an extremely poor prognosis, necessitating novel therapeutic strategies. This phase Ib trial evaluated the safety and preliminary efficacy of combining carbon-ion radiotherapy (C-ion RT) with immune checkpoint inhibitors (ICIs) in patients with advanced HCC with MVI.

Methods: Fifteen patients with MVI-positive advanced HCC were enrolled (Cohort A: durvalumab monotherapy, n = 3; Cohort B: durvalumab plus tremelimumab, n = 12). C-ion RT (60 Gy, four fractions) was delivered to the MVI-containing primary tumor, while systemic therapy with durvalumab (+tremelimumab) was administered concurrently. The primary endpoints included dose-limiting toxicities and adverse events. Secondary endpoints included progression-free survival and overall survival.

Results: No dose-limiting toxicities were observed, and the combination exhibited a manageable safety profile. The most common adverse events were pyrexia, rash, and elevated lipase levels. Grade 3–4 adverse events occurred in 53.3%, including cytokine release syndrome and meningitis. Median progression-free survival and overall survival were 4.7 and 10.4 months, respectively. Although C-ion RT achieved effective local control of irradiated lesions, non-irradiated lesions showed limited systemic immune responses.

Conclusions: The combination of MVI-targeted C-ion RT and immune checkpoint inhibitors demonstrated safe and effective local tumor control in advanced HCC. This novel approach of selective irradiation to MVI-containing tumors, combined with systemic immunotherapy, warrants further investigation to optimize the synergistic effects and enhance systemic efficacy in this poor-prognosis group.

Clinical Trials Registration: jRCT2031210046.

© 2026 The Author(s). Published by Elsevier B.V. on behalf of European Association for the Study of the Liver (EASL). This is an open access article under the CC BY license (http://creativecommons.org/licenses/by/4.0/).

Introduction

Hepatocellular carcinoma (HCC) is a leading cause of cancer-related mortality worldwide, with a 5-year survival rate of only 18% owing to late diagnosis and limited treatment options.[1,2] Even when detected early, its high recurrence rate often leads to advanced disease. A key feature of HCC progression is macrovascular invasion (MVI), characterized by tumor infiltration into hepatic vessels such as the portal and hepatic veins, distinguishing it from other malignancies.[3,4] MVI disrupts vascular flow, often leading to liver failure, making it a critical prognostic factor requiring targeted therapies.[5] Local MVI control through surgical resection, transarterial chemoembolization (TACE), and radiotherapy has been associated with improved outcomes.[6–8] Recent findings from our group further highlight that effective MVI control with systemic therapy significantly enhances overall survival (OS), underscoring its pivotal role in advanced HCC management.[9]

The treatment paradigm for advanced HCC has evolved from single-agent tyrosine kinase inhibitors (TKIs) to combination immunotherapy strategies.[10–12] Atezolizumab plus bevacizumab is widely adopted as first-line treatment, targeting both programmed cell death ligand 1 (PD-L1) and vascular endothelial growth factor (VEGF). Bevacizumab inhibits VEGF, reducing angiogenesis and enhancing T-cell infiltration, whereas atezolizumab reactivates exhausted T cells by blocking PD-L1, simultaneously addressing immune evasion and angiogenesis.[13] In contrast, durvalumab (anti-PD-L1) plus tremelimumab (anti-CTLA-4) focuses on dual checkpoint blockade,

* Corresponding author. Address: Department of Gastroenterology, Graduate School of Medicine, Chiba University, 1-8-1 Inohana, Chuo-ku, Chiba 260-8670, Japan. Tel: +81-43-226-2083 (ext 72013).
E-mail address: ogasawaras@chiba-u.jp (S. Ogasawara).
† These authors contributed equally to this work.
https://doi.org/10.1016/j.jhepr.2026.101765

with PD-L1 inhibition reactivating T cells in the tumor and CTLA-4 blockade promoting T-cell activation in lymphoid organs, enhancing systemic immunity.[14] The HIMALAYA trial (NCT03298451) established this combination as a standard first-line therapy, showing significant survival benefits over sorafenib.[12] Early evidence for combining local therapy with immunotherapy came from a phase I study of tremelimumab plus tumor ablation, demonstrating safety and increased intratumoral CD8+ T-cell infiltration in responders.[15] These findings provided proof of concept for integrating local therapy with immune checkpoint inhibition. However, therapeutic efficacy in patients with MVI remains suboptimal, underscoring the need for novel strategies.

Carbon-ion radiotherapy (C-ion RT) has emerged as a promising local treatment modality, offering superior dose-distribution characteristics that enable precise targeting of hepatic tumors while preserving surrounding healthy tissue.[16,17] The physical properties of carbon ions allow for enhanced biological effectiveness and reduced oxygen dependence compared with conventional radiotherapy, potentially offering advantages in treating radioresistant tumors.[18] Recent data have demonstrated exceptional local control rates with C-ion RT in HCC, leading to its regulatory approval in Japan for cases unsuitable for radical hepatic resection.[19] Unlike photon-based approaches such as stereotactic body radiotherapy (SBRT), which are generally recommended for small lesions (<3–5 cm) owing to liver tolerance and carry increased risks of radiation-induced liver injury in large or perihilar tumors, C-ion RT enables safe, high-dose delivery even for large or vascular-invasive lesions by virtue of its Bragg-peak distribution and superior biological effectiveness.[20–22] This makes C-ion RT particularly suitable for controlling MVI-positive tumors, where SBRT is often not feasible, and provides a rationale for its use in this trial. Importantly, radiation therapy has been shown to enhance immunogenic cell death (ICD), increase neoantigen presentation, and favorably modulate the tumor microenvironment, suggesting potential synergistic effects with immunotherapy.[23,24] This precise targeting capability makes C-ion RT particularly suitable for selective irradiation of MVI-positive lesions while sparing other hepatic regions.

Given this scientific rationale, we designed this phase Ib trial to evaluate a novel therapeutic approach that combines selective C-ion RT targeting only the MVI-containing tumors with durvalumab plus tremelimumab immunotherapy. Our strategic focus on MVI-positive lesions, rather than treating all tumor sites, was chosen to address the most critical prognostic factor while relying on systemic immunotherapy to control potential disseminated disease. This study represents the first clinical investigation of this selective targeting approach, aiming to leverage the precise local tumor control capabilities of C-ion RT specifically for MVI-positive lesions, while utilizing dual checkpoint inhibition for systemic disease control.

Patients and methods

Detailed information on the study rationale, design, and treatment plan has been previously published in the protocol paper.[25] We conducted a phase Ib trial to evaluate the safety and tolerability of a novel therapeutic approach combining selective C-ion RT directed at MVI-containing tumors with durvalumab plus tremelimumab in patients with advanced HCC (jRCT2031210046). Given the extensive study methodology, including eligibility criteria, treatment regimens, and key aspects such as sample size determination, statistical analysis, dose-limiting toxicity (DLT) definitions, prospective observation after trial completion, exploratory biomarker analysis with tumor biopsy samples, data management, monitoring, and ethics, these details are comprehensively described in the Supplementary Methods.

Patients

Key eligibility criteria are summarized below, with full details in Table S1. Patients aged ≥20 years with histologically confirmed advanced HCC or typical hypervascular findings on imaging were eligible. Key inclusion criteria included macrovascular invasion, ECOG performance status 0–1, body weight >30 kg, and Child–Pugh class A. The patients except for expansion cohort required prior failure of systemic therapy. Both viral and non-viral HCC patients were eligible. Adequate normal organ and marrow function as defined below: hemoglobin ≥9.0 g/dl, absolute neutrophil count ≥1,500/mm^3, platelet count ≥75,000/mm^3, serum bilirubin ≤ upper limit of normal (ULN) × 3.0, aspartate aminotransferase (AST) ≤ ULN × 5.0, alanine aminotransferase (ALT) ≤ ULN × 5.0, and measured creatinine clearance >40 ml/min or calculated creatinine clearance >40 ml/min by the Cockcroft–Gault formula or by 24-h urine collection for determination of creatinine clearance. Patients with disease up to Vp4 and Vv3 could also be included (patients with bile duct invasion were excluded). Patients with extrahepatic spread were also eligible for inclusion in the trial.

Exclusion criteria included unresolved grade ≥2 toxicities, extensive prior radiotherapy, major recent surgery, organ transplantation, primary immunodeficiency, autoimmune disorders, active brain metastases, hepatitis B/C or B/D coinfection, recent immunosuppressive use, or hypersensitivity to study drugs. Eligibility was assessed by investigators. Patients who are not eligible for irradiation in terms of surrounding risk organs cannot be incorporated during C-ion RT, as described below.

Study design and treatment

A detailed trial methodology is available in the Supplementary material. This phase Ib, multicenter, open-label trial (jRCT2031210046) evaluates the safety and tolerability of combining selective C-ion RT targeting MVI-containing tumors with immunotherapy in advanced HCC. Patients were enrolled at two centers in Japan (Chiba University Hospital and QST Hospital) into two cohorts: durvalumab monotherapy combined with C-ion RT (Cohort A) or durvalumab plus a single tremelimumab dose combined with C-ion RT (Cohort B). Durvalumab was administered every 4 weeks until disease progression in either cohort (Fig. S1).

In this trial, a minimum 7-day hospitalization period was pre-set following completion of C-ion RT to manage adverse events (AEs). During this period, patients received rigorous systemic management, including repeated vital sign monitoring, and blood tests. After the 7-day period, patients were discharged only if their general condition was stable and their blood test results showed no severe AEs.

The primary objective is to assess safety, with DLTs evaluated over 42 days using CTCAE v5.0 (Table S2). Primary

endpoints include DLT, AEs, and severe AEs. Secondary endpoints include OS, objective response rate (ORR), progression-free survival (PFS) and time to progression (TTP). Tumor response is assessed via imaging every 6 weeks, with confirmation required for complete and partial responses. The primary criterion used in this study was Response Evaluation Criteria in Solid Tumors (RECIST) version 1.1.

Definition and assessment period of DLT

Three patients were initially enrolled in Cohort A (durvalumab monotherapy). If no DLTs occurred during the 42-day evaluation period, enrollment proceeded to Cohort B (durvalumab plus tremelimumab). If a DLT occurred, three additional patients were enrolled in the same cohort. A regimen was considered intolerable if more than one DLT occurred among six patients; if Cohort A was intolerable, Cohort B was not initiated. DLTs were assessed during a 42-day period starting from the initiation of investigational treatment (Day 1 of Cycle 1). This period was defined to capture acute radiotherapy-related toxicities and was determined following discussions with the Japanese regulatory authorities. Adverse events were graded using CTCAE v5.0, and DLTs were defined as treatment-related events meeting predefined criteria (Table S2).

C-ion RT

C-ion RT (60 Gy in four fractions) was initiated on Day 8 of Cycle 1. The gross tumor volume (GTV) included intrahepatic nodules forming MVI and the MVI itself. The clinical target volume and field-specific planning target volume were defined based on respiratory motion analysis using 4D-CT.[26] Dose calculation was performed using the microdosimetric kinetic model, and all doses are expressed as relative biological equivalent (RBE).[27] Details of target delineation, motion management, and dose constraints are provided in the Supplementary Methods.[16,28–31]

Results

Patient characteristics

Informed consent was obtained from 18 patients, of whom 15 were enrolled. All 15 enrolled patients received the investigational treatment. In Cohort A, tolerability was assessed in three individuals, while in Cohort B, 12 patients were enrolled (four for tolerability evaluation, eight for the expansion cohort; Fig. S2).

The median age of the patients was 69 years (range, 25–81 years), and 13 patients (86.7%) were male. Non-viral etiologies were the most common underlying cause of liver disease (HBV: n = 3 [20.0%], HCV: n = 1 [13.3%], non-viral: n = 10 [66.7%]). All patients were classified as Barcelona Clinic Liver Cancer (BCLC) stage C and presented with MVI. Among the 15 patients included in this study, major portal vein invasion (Vp3–4) was absent in eight individuals and present in seven. Among the 15 patients enrolled, four (26.7%) were systemic therapy-naive (Table 1). Representativeness of the study participants is described in Table S3. The median follow-up duration for the overall study population during the trial period was 5.7 months. With the inclusion of the post-trial prospective observational period (Supplementary Methods), the median follow-up duration extended to 10.4 months.

Table 1. Patient characteristics.

	Whole population (N = 15)	Cohort A (n = 3)	Cohort B (n = 12)
Age, median (range)	69.0 (25.0–81.0)	61.0 (58.0–74.0)	70.0 (25.0–81.0)
Sex, male, n (%)	13 (86.7)	3 (100.0)	10 (83.3)
HBV positive, n (%)	3 (20.0)	0 (0.0)	3 (25.0)
HCV positive, n (%)	2 (13.3)	1 (33.3)	1 (8.3)
Non-viral, n (%)	10 (66.7)	2 (66.7)	8 (66.7)
Child–Pugh score, n (%)			
5	10 (66.7)	3 (100.0)	7 (58.3)
6	5 (33.3)	0 (0.0)	5 (41.7)
ECOG-PS, n (%)			
0	13 (86.7)	3 (100.0)	10 (83.3)
1	2 (13.3)	0 (0.0)	2 (16.7)
Median maximum tumor size, mm (range)	91.1 (32–221.8)	92.4 (54–103)	89.6 (32–221.8)
Number of intrahepatic tumors ≥8, n (%)	10 (66.7)	3 (100.0)	7 (58.3)
Portal vein invasion, n (%)			
Vp2	7 (46.7)	2 (66.7)	5 (41.7)
Vp3	3 (20.0)	1 (33.3)	2 (16.7)
Vp4	4 (26.7)	0 (0.0)	4 (33.3)
Hepatic vein invasion, n (%)			
Vv2	1 (6.7)	0 (0.0)	1 (8.3)
Vv3	1 (6.7)	0 (0.0)	1 (8.3)
Extrahepatic spread, n (%)	7 (46.7)	1 (33.3)	6 (50.0)
AFP (ng/ml), median (range)	213.7 (3.5–270,208.0)	10,444.3 (56.7–29,082.1)	165.3 (3.5–270,208.0)
Systemic therapy-naive, n (%)	4 (26.7)	–	4 (33.3)
Presence of prior systemic therapy	11	3	8
Treatment regimen of prior systemic therapy			
Atezolizumab + bevacizumab	8	2	6
Lenvatinib	1	0	1
Sorafenib	1	0	1
Ramucirumab	1	1	0

AFP, alpha-fetoprotein; ECOG-PS, Eastern Cooperative Oncology Group performance status; EHM, extrahepatic metastasis; MVI, macrovascular invasion.

Primary outcomes: safety and adverse events

Of the six patients included in the DLT evaluation (three in Cohort A and three in Cohort B). DLT observation rate were 0% (0/3) in Cohort A, 0% (0/3) in Cohort B, respectively. AEs of any grade were reported in all 15 patients (100%). Serious AEs (SAEs) occurred in five patients (33.3%) for a total of 11 events. Two of these SAEs led to treatment discontinuation. No SAEs were reported in Cohort A. In Cohort B, SAEs were reported in four patients, including mucosal inflammation, adrenal insufficiency, interstitial lung disease, enterocolitis, cytokine release syndrome, and meningitis. Seven patients (46.7%) experienced grade 3 AEs, two patients (13.3%) experienced grade 4 AEs, and one patient (6.7%) experienced grade 5 AE (heart failure). Treatment-emergent AEs (TEAEs) occurring in ≥10% of patients are summarized in Table 2. The most common TEAEs were pyrexia, rash, elevated lipase levels, and radiation-induced skin lesions. In Table 2, we also report the frequency of treatment-emergent AEs (TEAEs) stratified by the presence or absence of major portal vein invasion (Vp3–4). Detailed data on Grade 2–4 treatment-related AEs (TRAEs) are provided in Table S4.

Secondary outcomes: efficacy analysis

The median PFS time for all patients, based on RECIST version 1.1, was 4.7 months (95% CI, 1.4–6.4). In Cohort A, the median PFS time was 4.7 months (95% CI, 4.6–4.7), whereas in Cohort B, it was 3.8 months (95% CI, 1.1–6.6). The 6-month PFS rates were 0% in Cohort A and 47.6% in Cohort B. Consistent with the PFS findings, the median TTP for all patients was 4.7 months (95% CI, 1.3–6.4). The median TTP was 4.7 months (95% CI, 4.6–4.7) in Cohort A and 6.2 months (95% CI, 1.1–6.6) in Cohort B. Based on RECIST version 1.1, the disease control rate was 73.3% in whole population. At the end of the study period, only one death had occurred and OS data were immature. Therefore, OS data are presented based on subsequent follow-up. The median OS was 28.2 months (95% CI, 5.5–50.9) in Cohort A and 10.2 months (95% CI, 4.3–16.1) in Cohort B. The 6-month OS rates were 100% for Cohort A and 63.6% for Cohort B. For the entire cohort of 15 patients, the median OS was 10.4 months (95% CI, 5.6–15.2), with 6-month and 12-month OS rates of 66.7% and 46.7%, respectively. An additional *post-hoc* efficacy analysis was conducted according to the presence of major portal vein invasion (Vp3–4). Median PFS was comparable between patients without and with Vp3–4 (4.6 months, 95% CI, 1.1–6.5 *vs.* 4.7 months, 95% CI, 0.5–6.6; p = 0.960). Median OS was 14.5 months (95% CI, 4.9–NE) in the Vp3–4–absent group and 9.5 months (95% CI, 3.2–28.2) in the Vp3–4–present group (p = 0.222) (Figs. S3 and S4 and Table S5).

Fig. 1 provides an overview of treatment outcomes, including a swimmer plot for treatment duration and response timing (Fig. 1A), and a waterfall plot indicating depth of tumor response (Fig. 1B). The median duration of disease control of the overall study population was 3.4 months (95% CI, 1.4–5.3). These data illustrate that, excluding three patients in Cohort B, the majority of patients exhibited tumor shrinkage, supporting the potential efficacy of the treatment. The waterfall plot shows tumor shrinkage in all patients except for three in Cohort B,

Table 2. TEAEs occurring in ≥10% of whole study population, stratified by treatment cohort and major portal vein invasion.

Event, n (%)	Whole population (N = 15)	Cohort A: Durvalumab + C-ion RT (n = 3)	Cohort B: Durvalumab + tremelimumab + C-ion RT (n = 12)	Major portal vein invasion (Vp3, 4)	
				Absent (n = 8)	Present (n = 7)
Any event	15 (100.0)	3 (100.0)	12 (100.0)	8 (100.0)	7 (100.0)
Pyrexia	9 (60.0)	1 (33.3)	8 (66.7)	5 (62.5)	4 (57.1)
Rash	5 (33.3)	0 (0.0)	5 (41.7)	2 (25.0)	3 (42.9)
Lipase increased	5 (33.3)	1 (33.3)	4 (33.3)	3 (37.5)	2 (28.6)
Radiation skin injury	5 (33.3)	2 (66.7)	3 (25.0)	3 (37.5)	2 (28.6)
Decreased appetite	4 (26.7)	1 (33.3)	3 (25.0)	2 (25.0)	2 (28.6)
Constipation	3 (20.0)	0 (0.0)	3 (25.0)	0 (0.0)	3 (42.9)
Diarrhea	3 (20.0)	0 (0.0)	3 (25.0)	1 (12.5)	2 (28.6)
Malaise	3 (20.0)	0 (0.0)	3 (25.0)	1 (12.5)	2 (28.6)
ALT increased	3 (20.0)	1 (33.3)	2 (16.7)	2 (25.0)	1 (14.3)
Amylase increased, n (%)	3 (20.0)	1 (33.3)	2 (16.7)	2 (25.0)	1 (14.3)
AST increased	3 (20.0)	1 (33.3)	2 (16.7)	2 (25.0)	1 (14.3)
COVID-19	2 (13.3)	0 (0.0)	2 (16.7)	2 (25.0)	0 (0.0)
Interstitial lung disease	2 (13.3)	0 (0.0)	2 (16.7)	1 (12.5)	1 (14.3)
Pleural effusion	2 (13.3)	0 (0.0)	2 (16.7)	1 (12.5)	1 (14.3)
Abdominal pain	2 (13.3)	1 (33.3)	1 (8.3)	1 (12.5)	1 (14.3)
Platelet count decreased	2 (13.3)	1 (33.3)	1 (8.3)	2 (25.0)	0 (0.0)

ALT, alanine aminotransferase; AST, aspartate aminotransferase; TEAEs, treatment-emergent adverse events.

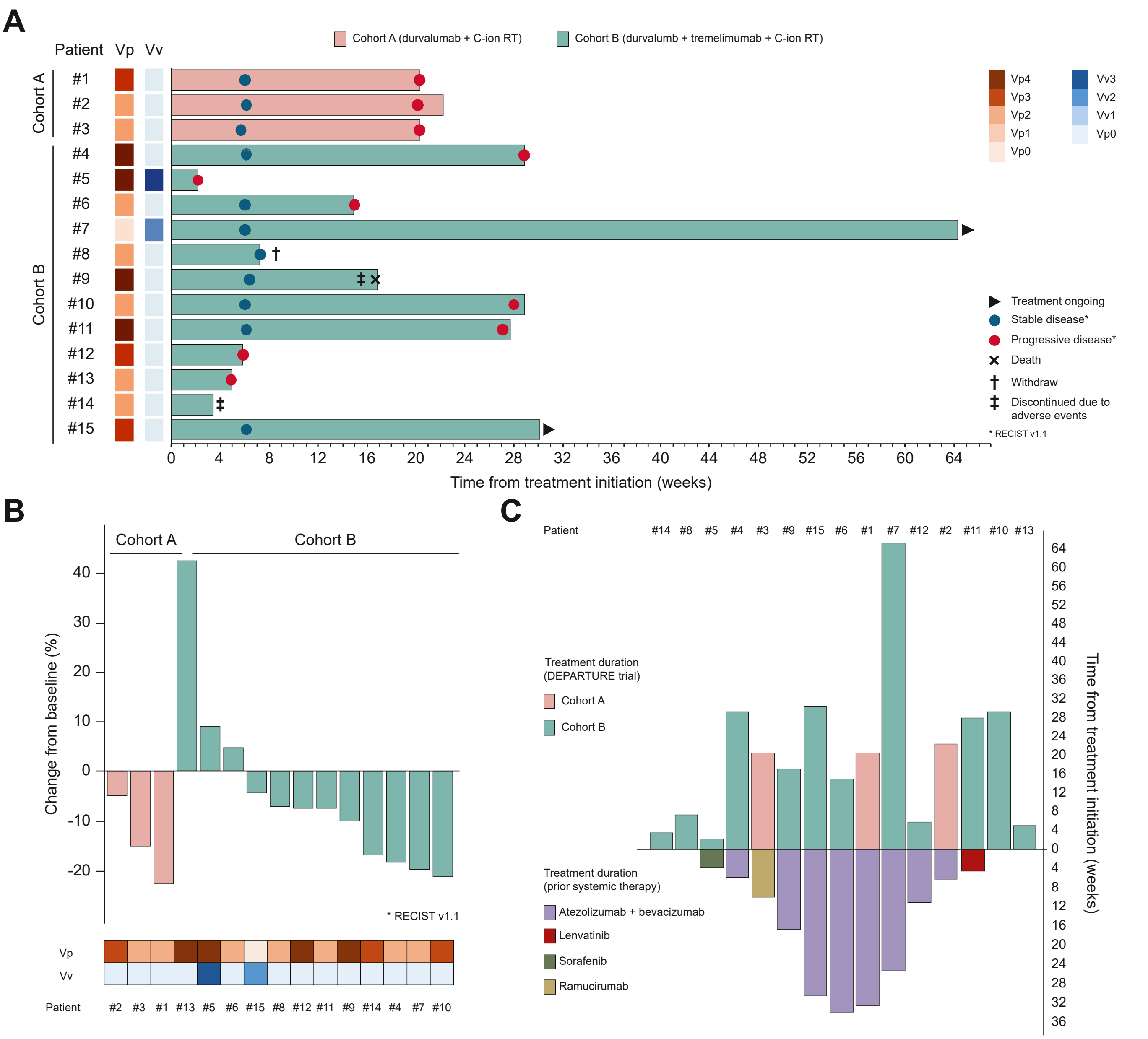

Fig. 1. Efficacy of investigational treatment. (A) Swimmer's plot showing treatment duration and best response. (B) Waterfall plot showing the change in the sum of target lesion diameters. (C) Iceberg plot of treatment duration before and during the investigational therapy. Prior systemic therapy duration is shown below the baseline, and treatment duration with the investigational regimen is shown above.

indicating tumor size reduction in the majority of cases. Among the 15 patients included in this study, 11 had received prior systemic therapy. The prior systemic treatments included atezolizumab plus bevacizumab in eight patients, lenvatinib in one patient, sorafenib in one patient, and ramucirumab in one patient. An iceberg plot illustrating the duration of the immediately preceding systemic therapy and the investigational treatment is shown in Fig. 1C. In this plot, the duration of prior systemic therapy is displayed below the baseline, and the treatment duration of the investigational therapy is displayed above the baseline. The plot includes patients with relatively short durations of prior systemic therapy who subsequently had longer durations of treatment with the investigational therapy.

Tumor response analysis inside and outside the irradiation field

This exploratory analysis investigated tumor behavior inside and outside the C-ion RT field, highlighting significant differences in response. Fig. 2 presents a spider plot summarizing the overall changes in target lesion sizes during the study. Tumor imaging dynamics were compared between lesions located inside the irradiation field and those outside. Of the 11 patients who experienced progressive disease during follow-up, it was attributed to new lesions in eight individuals and target lesion growth in four.

No cases of rapid tumor enlargement were observed in lesions treated with C-ion RT. Tumor control was achieved in the

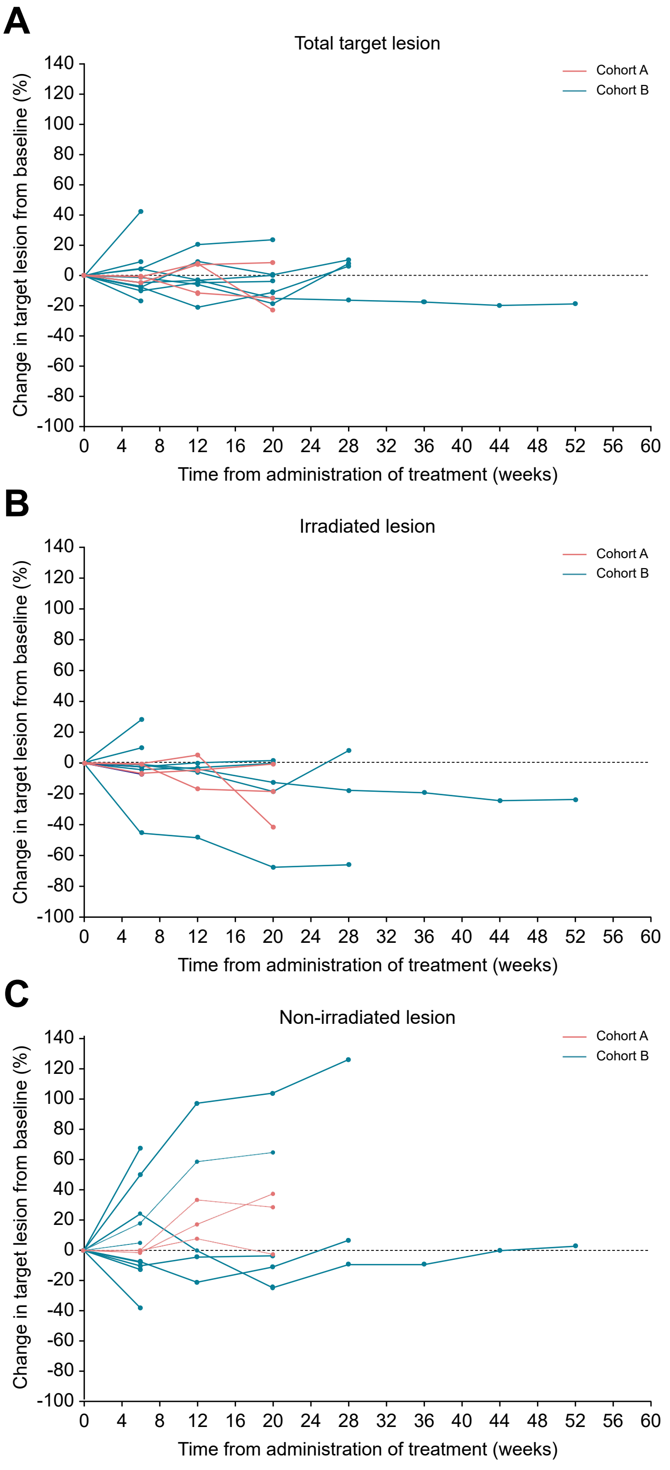

Fig. 2. Spider plot showing changes in tumor measurements over time. (A) Sum of all target lesions. (B) MVI-positive lesion treated with C-ion RT. (C) Sum of lesions outside C-ion RT field. C-ion RT, carbon-ion radiotherapy; MVI, macrovascular invasion.

majority of patients, with significant shrinkage shown in one individual. Based on our previously reported criteria for evaluation of MVI progression,[9] the progression of MVI during the treatment period was evaluated. Regarding MVI progression itself, only one individual experienced progression during the treatment period. In contrast, non-irradiated lesions displayed more heterogeneous behavior. Significant tumor enlargement (≥+50%) was observed in several cases, while some lesions remained stable without noticeable growth over an extended period over 20 weeks. Importantly, no instances of substantial tumor shrinkage were observed among non-irradiated lesions.

Prolonged pyrexia and clinical course of cytokine release syndrome

The most common AE of the present study was pyrexia, which occurred primarily during the administration of the investigational treatment and during C-ion RT. The characteristics of onset of pyrexia is summarized in Fig. 3. Of note, no cases of pyrexia were observed in Cohort A, whereas six cases of pyrexia with chills and high fever occurred immediately after C-ion RT in Cohort B. As a *post-hoc* exploratory analysis, we compared PFS between patients who experienced pyrexia (n = 6) and those who did not (n = 9). Median PFS was 2.8 months (95% CI, 1.1–6.4) in the pyrexia group and 4.7 months (95% CI, 0.5–6.6) in the non-pyrexia group, with no significant difference observed (p = 0.204) (Fig. S6).

One individual demonstrated a notable clinical course (Fig. 4). A man in his 50s with portal vein invasion (Vp2) was enrolled in the expansion cohort and developed cytokine release syndrome (CRS). After receiving durvalumab and tremelimumab on Day 1, he experienced chills and fever following his first fraction of C-ion RT. On Day 13, after the third fraction, he developed tachycardia and hypotension. Despite initial concerns about sepsis, negative cultures led to a CRS diagnosis, and he responded well to appropriate supportive therapy. Additionally, on Day 22, the patient had headache and rigidity, so a lumbar puncture was performed, which revealed inflammatory findings in the cerebrospinal fluid indicating meningeal involvement. Tests for bacterial and viral infection were negative, so the patient was considered to have central nervous system symptoms associated with CRS. Exploratory analysis using the ELISA method revealed increases in IL-6, IFN-γ, and damage-associated molecular patterns (DAMPs) (S100A8/S100A9 and HMGB1) during febrile episodes after radiotherapy, correlating with inflammatory dynamics. These findings support a potential relationship between C-ion RT and inflammatory cytokine activation when under the influence of immune checkpoint inhibitors (ICIs).

Long-term biliary effects of C-ion RT

In the post-trial observational study, intrahepatic bile duct strictures attributed to C-ion RT were identified as notable AEs. Of the four cases observed, all had tumors that extended beyond the hepatic hilum and were included in the radiation field. Over the long term, these patients developed intrahepatic bile duct dilatation. Three of these four cases required biliary drainage tube placement to manage the condition. Stent placement, performed under endoscopic retrograde cholangiopancreatography (ERCP), was effective in preserving liver function in these patients. Fig. 5 illustrates the imaging of the one case. Table S6 shows the size of nodules with MVI that received carbon-ion irradiation, and the proportion of tumors that were adjacent to the hepatic hilum or contained major portal vein invasion (Vp3, 4) in the four cases with bile duct dilatation and the 11 cases without. To further characterize the

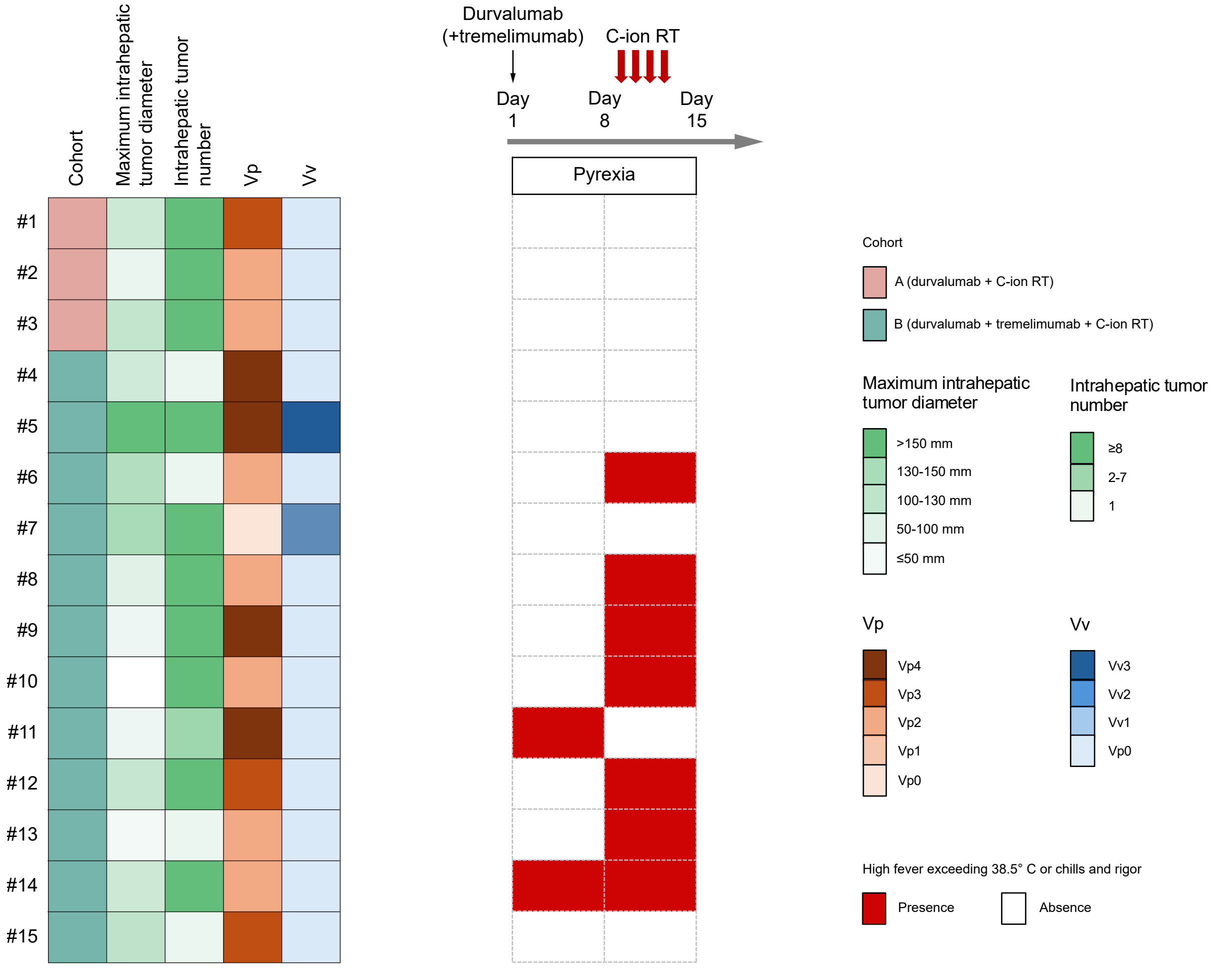

Fig. 3. The characteristics of onset of pyrexia. No cases of pyrexia were observed in cohort A. In Cohort B, two patients developed a high fever of 38.5 °C or higher or chills within 7 days of starting durvalumab plus tremelimumab; six patients had a new onset of high fever after starting C-ion RT. C-ion RT, carbon ion radiotherapy.

relationship between biliary events and the irradiation field, we assessed whether the hepatic hilum was included in the C-ion RT treatment field. C-ion RT involved the hepatic hilum in 12 of 15 patients (80%), among whom biliary strictures with distal bile duct dilatation developed in four patients (33% of those with hilar involvement). In contrast, no biliary strictures were observed in patients whose irradiation fields did not include the hepatic hilum. Radiotherapy treatment plans for all patients are shown in Fig. S5. The determination of whether tumors were adjacent to the hepatic hilum was based on a previously reported evaluation method.[32]

RNA-sequencing analysis: insights into tumor microenvironment and treatment response

RNA-sequencing was performed on biopsy specimens collected immediately before treatment initiation from nine patients enrolled in the trial, with follow-up analysis conducted on non-irradiated lesions from three patients at least 42 days after treatment initiation. To assess the impact of combining C-ion RT with immunotherapy while minimizing the influence of direct radiation effects, the analysis focused on non-irradiated lesions. Patients were stratified into reduction (relative change ≤-5%) and progression groups (relative change >-5%) based on lesion dynamics (Fig. 6A). Four patients were classified into the reduction group, four into the progression group, and one was unclassifiable due to the absence of measurable non-irradiated lesions. The analysis examined the relationship between tumor microenvironmental factors, PFS, and treatment outcomes. Immune cell proportions were evaluated using CIBERSORTx, with regulatory T cells (Tregs) assessed via FoxP3 expression. In post-immunotherapy biopsy specimens, one case in the progression group demonstrated an increase in Treg proportions and elevated FoxP3 expression. No consistent changes were observed in other immune cell populations (Fig. 6B). Gene Set Enrichment Analysis (GSEA) was conducted to identify pathways associated with treatment response. The reduction group showed enrichment of pathways related to inflammation, complement activation, which are often associated with immune activity. In contrast, the progression group exhibited enrichment of pathways linked to mesenchymal characteristics and angiogenesis, processes that may correlate with reduced therapy efficacy (Fig. 6C). After excluding cases treated with durvalumab monotherapy, the reduction group demonstrated enrichment of pathways involved in antigen presentation, suggesting enhanced immune recognition in responding patients (Fig. 6D). A heatmap (Fig. 6E) visualizes the gene expression profiles linked to these

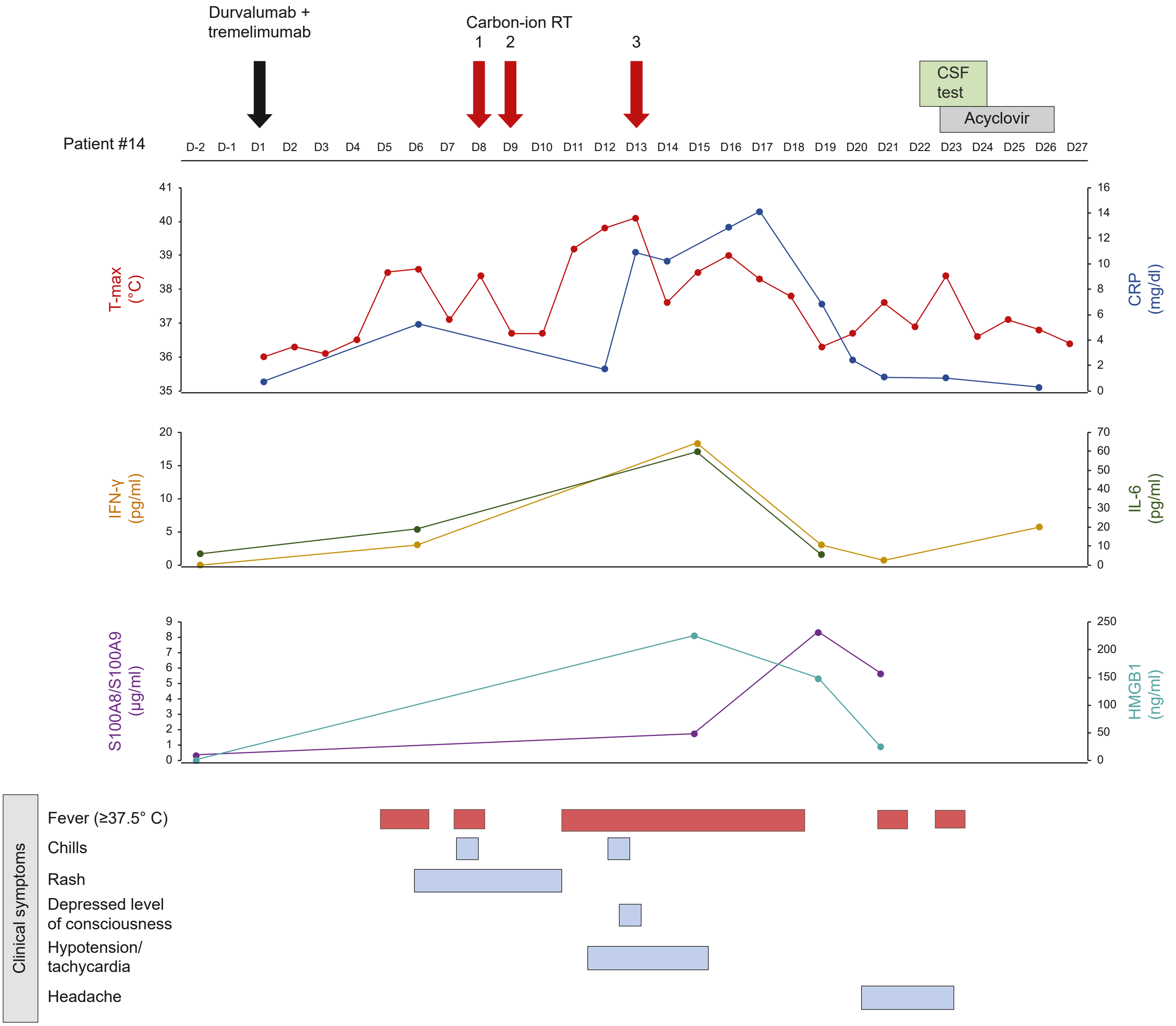

Fig. 4. Case presentation of cytokine release syndrome (CRS). The patient experienced chills and fever following his first fraction of C-ion RT. On Day 13, after the third fraction, he developed tachycardia and hypotension. On Day 22, the patient had headache and rigidity. Inflammatory findings were found in the cerebrospinal fluid indicating meningeal involvement. Tests for infection were negative, so the patient was considered to have CNS symptoms associated with CRS. Exploratory analysis using the ELISA method revealed increases in IL-6, IFN-γ, and DAMPs (S100A8/S100A9 and HMGB1) during febrile episodes after C-ion RT, correlating with inflammatory dynamics. C-ion RT, carbon-ion radiotherapy; CNS, central nervous system; CRS, cytokine release syndrome; DAMPs: damage-associated molecular patterns.

pathways, aligning immune cell dynamics with molecular alterations. This analysis highlights differences in tumor microenvironmental pathways between reduction and progression groups. Enrichment of inflammatory and antigen presentation pathways in non-irradiated lesions was associated with reduction, whereas mesenchymal and angiogenesis-related pathways were linked to progression.

Discussion

The present study demonstrated that our novel therapeutic approach combining selective C-ion RT targeting MVI-containing tumors with durvalumab plus tremelimumab was well tolerated, with no DLTs observed and a manageable safety profile. A total of 15 patients were enrolled (Cohort A: durvalumab monotherapy, n = 3, Cohort B: durvalumab plus tremelimumab, n = 12) with a median PFS of 4.7 months and OS of 10.4 months. Considering that all patients in our cohort had MVI, which typically indicates a poor prognosis, these survival outcomes were encouraging. Although the median OS appeared longer in Cohort A (durvalumab monotherapy, 28.2 months) compared with Cohort B (durvalumab plus tremelimumab, 10.2 months), this observation should be interpreted with caution. The analysis is based on very small numbers, with imbalances in prior systemic therapy exposure and MVI status that may have influenced outcomes. Given the exploratory,

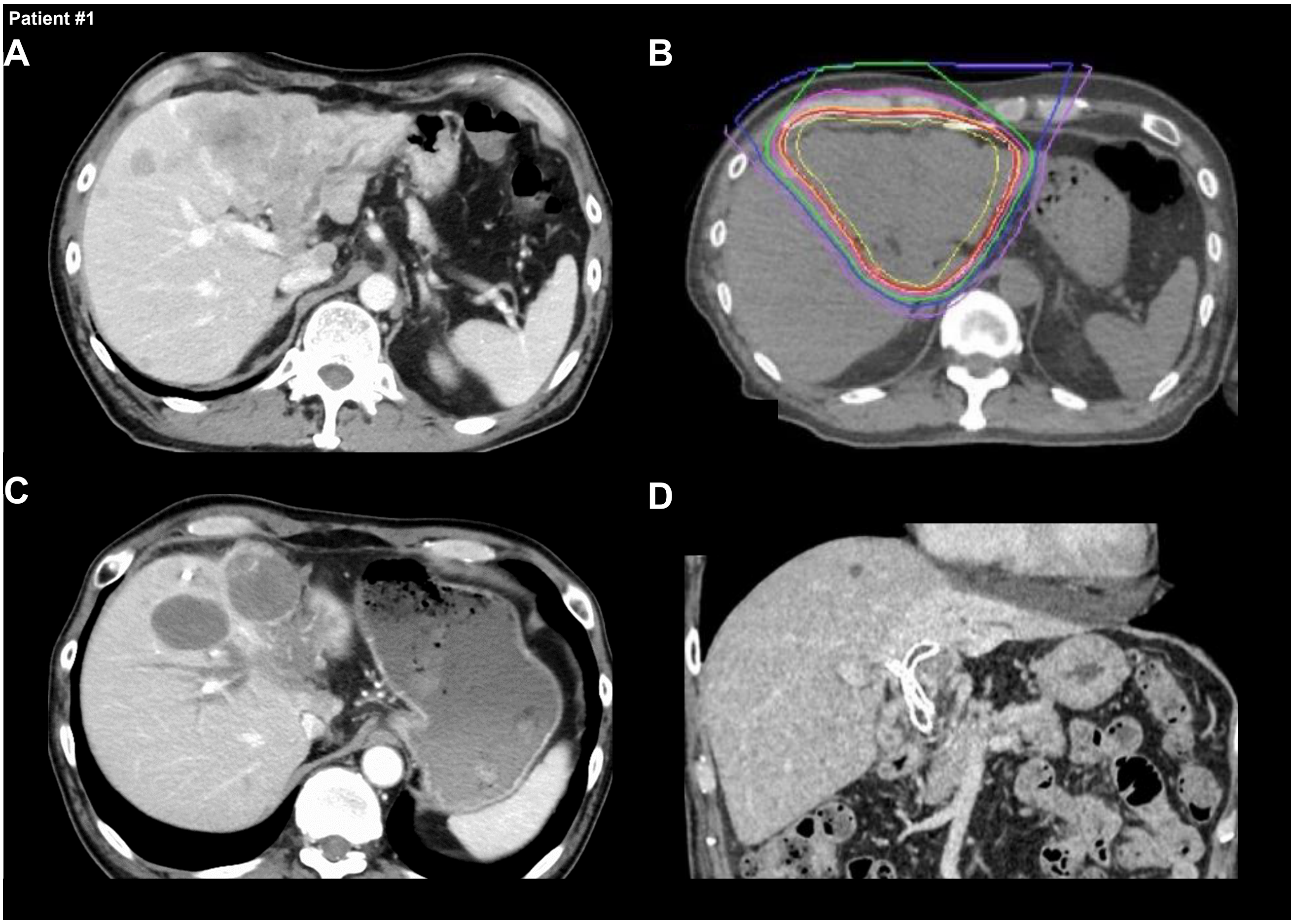

Fig. 5. CT imaging of the case which developed intrahepatic bile duct dilatation during long-term observation. (A) CT scan imaging of baseline. (B) Dose distribution of C-ion RT superimposed on contrast-enhanced computed tomography (B). (C) Bile duct dilatation development. (D) After stenting (metallic stent) in the bile duct. C-ion RT, carbon-ion radiotherapy; CT, computed tomography.

non-comparative design of this study, the apparent OS difference should be considered hypothesis-generating rather than evidence of regimen-specific effects. In the HIMALAYA trial,[12] which evaluated durvalumab plus tremelimumab and durvalumab monotherapy in a broader HCC group, the reported PFS durations were comparable to our findings. C-ion RT effectively controlled irradiated lesions, showing no rapid tumor enlargement and in some cases significant tumor shrinkage, while non-irradiated lesions showed variable responses, including progression in some patients. RNA-sequencing analysis revealed that enrichment of inflammatory and antigen presentation pathways correlated with favorable treatment outcomes, whereas mesenchymal and angiogenesis pathways were associated with resistance in unirradiated lesions.

From a safety perspective, a key finding of this study was the frequent occurrence of fever after C-ion RT administration following durvalumab, with or without tremelimumab. The significance of this observation is underscored by the fact that fever has rarely been reported as an AE in prospective studies of particle therapy, including C-ion RT. Additionally, a systematic review of toxicities associated with combined ICI and radiation therapy[33] evaluated the toxicity profile of this combination and reported no notable adverse events in combined ICI and RT therapy compared to ICI monotherapy. Previous studies have suggested that high-linear energy transfer (LET) radiation, such as carbon ions, induces stronger immune responses compared to low-LET radiation such as X-rays or α-rays.[34] Specifically, C-ion RT has been shown to cause greater upregulation of HMGB1, which promotes dendritic cell activation, and stronger induction of MHC-I compared with X-rays.[34,35] These findings support our observation that the high frequency of fever in our study represents a distinctive response, and the fever pattern suggests a potential immune response induced by C-ion RT when administered after ICI therapy. Emerging evidence indicates that combining C-ion RT with ICIs is feasible. A phase Ib trial of durvalumab plus C-ion RT with cisplatin in cervical cancer showed good tolerability, with only one serious hypothyroidism case,[36] and a retrospective analysis in melanoma reported grade ≥3 AEs in 21%, similar to rates with either therapy alone.[37] These data support our finding that C-ion RT with ICIs can be delivered safely with a manageable profile.

Among long-term adverse events, bile duct strictures were observed in patients with tumors extending beyond the hepatic hilum within the radiation field. Three out of 15 cases required biliary drainage, with stent placement under ERCP preserving liver function. The causes of this stricture could include tumor infiltration, scarring of tumor tissue, normal tissue remodeling, or any combination of these factors, with ICIs augmenting the

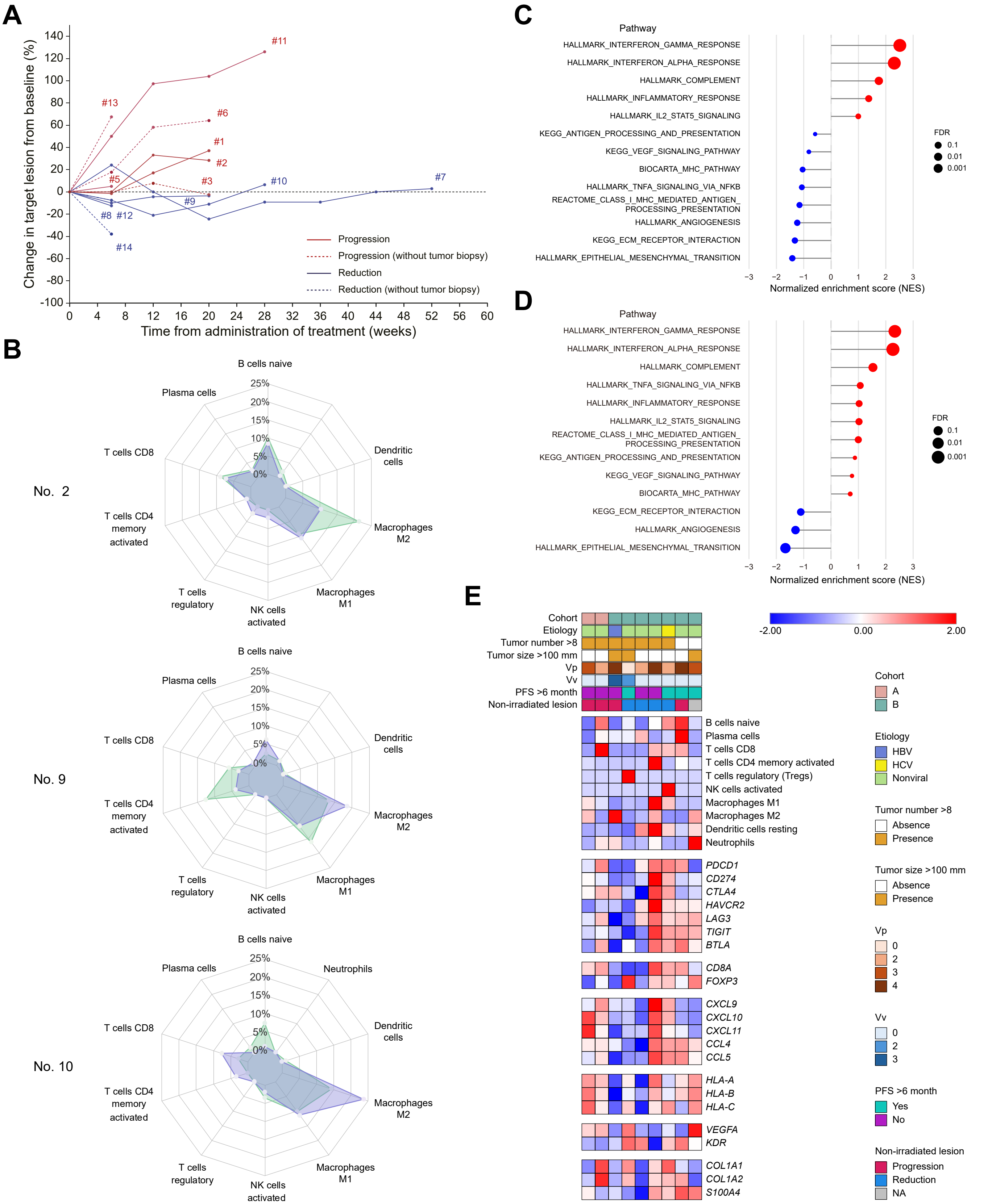

Fig. 6. RNA-seq analysis of non-irradiated lesions and tumor microenvironment in patients receiving combined C-ion RT and immunotherapy. (A) Spider plot showing the dynamics of non-irradiated lesions. Patients were stratified into reduction (relative change ≤-5%) and progression groups (relative change >-5%) based on lesion size changes. (B) Radar charts comparing immune cell proportions between pre- and post-treatment biopsy specimens, as calculated by CIBERSORTx. Patient #2 in the progression group showed an increase in Treg proportions after treatment. (C) Lollipop plot displaying Gene Set Enrichment Analysis (GSEA) results

effects. According to past reports, intrahepatic bile duct stricture are rare.[22,38] However, recent reports indicate that bile duct strictures may emerge as a late complication of C-ion RT. The frequency and severity of these strictures increase in perihilar-type tumors near the portal vein trunk, necessitating long-term rigorous follow-up.[32] Our results did not confirm that the presence of Vp3 or Vp4 itself led to worsening bile duct dilatation. However, the occurrence of bile duct dilatation may have been influenced by the fact that many cases involved very large irradiated tumor diameters and tumor progression to the hepatic hilum. Further research is needed on dose planning for cases involving such tumor conditions. Whether caused by tumor infiltration or normal tissue remodeling remains unclear, but these findings highlight the importance of risk assessment, monitoring, and careful treatment planning for tumors near the hepatic hilum. In this context, and in contrast to previous reports describing vascular toxicities after short-course C-ion RT for pancreatic cancer,[39,40] no irradiation field-related vascular AEs were observed in the present study, including portal vein stenosis or other clinically significant vascular complications. Given that vascular injury represents a critical determinant of liver function, particularly in treatments involving major vascular structures, the absence of such events provides important safety insights for this treatment strategy in patients with HCC.

Of particular interest was a case presenting with CRS complicated by meningitis following treatment. The CRS was accompanied by elevated levels of DAMPs, including S100A8/S100A9 and HMGB1, suggesting ICD as a potential trigger. In hematologic malignancies, CRS is a well-known complication following CAR-T cell therapy,[41] which can be accompanied by immune effector cell-associated neurotoxicity syndrome (ICANS). The diagnosis of ICANS typically requires brain MRI and cerebrospinal fluid examination,[42] with the underlying pathophysiology involving direct damage to the central nervous system by activated effector T cells and cytokines.[43] The presentation of meningitis in our case, a manifestation rarely seen with ICI therapy alone, coupled with the pattern of immune activation, suggests that this may represent a case of ICANS. The combination of C-ion RT with ICIs appears to have induced distinct patterns of immune activation similar to those observed in CAR-T cell therapy. CRS we experienced was most likely related to the combination of C-ion RT and ICIs. Importantly, with appropriate monitoring and management, the patient's symptoms resolved, enabling hospital discharge. This case highlights the critical importance of rigorous systemic monitoring and proper management of immune-related adverse events when administering this combination therapy. As demonstrated in our trial, strict management of adverse events related to immune enhancement following C-ion RT for at least 7 days under continued hospitalization likely contributed to improved AE management.

The therapeutic efficacy analysis revealed a distinct pattern of responses. In this study, a dissociation was observed between local tumor control within the irradiation field and systemic disease control. Local tumor control was evaluated as an exploratory measure of local effectiveness rather than as a surrogate for systemic efficacy, although sustained control of vascular-invasive lesions may have clinical relevance in HCC with major vascular invasion. Although C-ion RT achieved strong tumor control within irradiated fields, limited antitumor effects were observed at non-irradiated sites, suggesting a potential lack of systemic responses such as abscopal effects, which have been reported in some radiotherapy-immunotherapy combinations.[44] The clinical experience with radiotherapy-immunotherapy combinations has shown varying degrees of success across different cancer types in phase III trials. The PACIFIC trial (NCT02125461) demonstrated significant improvements in both PFS and OS when durvalumab was administered following concurrent chemoradiotherapy (CRT) in stage III non–small-cell lung cancer.[45] Similarly, in esophageal and gastroesophageal junction cancers, nivolumab showed significant survival benefits when administered to patients who did not achieve pathological complete response after CRT,[46] While large-scale trials predominantly support post-radiation ICI administration, as exemplified by the successful PACIFIC trial and esophageal cancer studies, emerging data specifically in HCC suggests potential benefits from alternative approaches. Recent smaller-scale studies have shown promising results with concurrent administration or radiation delivery after ICI initiation, suggesting that optimal sequencing strategies may be disease-specific.[47–49] In our study, while we did not observe clear synergistic effects, the combination of ICI and C-ion RT demonstrated promising additive effects with acceptable safety. Importantly, C-ion RT achieved effective control of MVI-containing tumors, consistent with several previous reports demonstrating the efficacy of particle beam therapy in treating MVI-positive HCC lesions.[32,50–52] The achievement of long-term survival in some patients with highly advanced HCC through this selective targeting approach warrants attention and further investigation. Given the extremely steep dose gradients of C-ion RT, accurate target definition was essential. In this study, irradiation was confined to MVI-containing tumors based on high-quality imaging and careful contouring, which likely contributed to the favorable local control observed in irradiated fields, in contrast to the limitations of photon-based SBRT in large or perihilar lesions. Future studies should focus on enhancing systemic efficacy through optimized treatment scheduling and potentially incorporating additional immunomodulatory agents, with particular attention to HCC-specific sequencing strategies that may differ from established paradigms in other cancer types.

This early-phase study primarily focused on safety and evaluated two treatment regimens: durvalumab plus C-ion RT and durvalumab plus tremelimumab plus C-ion RT. No DLTs were observed with either regimen, and TRAEs were generally manageable. An independent monitoring committee confirmed that no major safety concerns were identified. Based primarily on these safety findings, with preliminary efficacy signals

comparing reduction and progression groups, highlighting differentially enriched pathways. (D) Lollipop plot showing GSEA results after excluding durvalumab monotherapy cases, comparing pathway enrichment between reduction and progression groups. (E) Heatmap of gene expression profiles from pretreatment biopsy specimens, illustrating the relationship between patient characteristics, treatment outcomes, and gene expression patterns. C-ion RT, carbon-ion radiotherapy; Treg, regulatory T cell.

regarded as supportive, the combination of durvalumab, tremelimumab, and C-ion RT is considered a tolerable regimen and a recommended phase II dose candidate for further clinical development.

In this phase Ib study, no clear abscopal effect was observed in non-irradiated lesions, consistent with the rarity of clinically meaningful abscopal responses.[53] Several factors may account for this. Irradiation was confined to MVI-containing tumors, potentially limiting systemic antigen release and T-cell priming,[54] and all patients had advanced disease with extensive MVI, where the immunosuppressive tumor microenvironment and large tumor burden likely constrained systemic immunity.[55–57] Although the high-LET properties of C-ion RT favor local control, the optimal fractionation and timing of ICI administration to elicit systemic responses remain undefined.[58] Future strategies to enhance systemic activity in HCC may involve optimizing sequencing approaches, for example administering PD-1/PD-L1 blockade early after RT or initiating ICI followed by selective C-ion RT. Another promising avenue is broadening antigen release with multisite hypofractionated RT or using low-dose RT to inflame non-irradiated lesions and enhance ICI sensitivity.[59,60] In addition, anti-VEGF–mediated vascular normalization may improve immune cell trafficking and drug delivery, thereby supporting RT–ICI synergy.[61]

This study examined tumor microenvironmental pathways using pretreatment and post-treatment samples, focusing on unirradiated lesions, to evaluate the systemic effects of combining ICIs with C-ion RT. Although the limited sample size precludes definitive conclusions, the results showed clear differences between the reduction and progression groups. Reduction was characterized by enrichment of inflammatory and antigen presentation pathways, findings consistent with previous reports linking these processes to ICI efficacy.[62] Conversely, progression was associated with mesenchymal and angiogenic pathways, which have also been widely implicated as drivers of immune resistance and tumor progression.[63]

This study has several limitations. First, it was a small, phase Ib trial primarily designed to assess tolerability and safety. Accordingly, the analyses of PFS and OS are exploratory and should be interpreted with caution. The apparent OS difference between Cohorts A and B is also explained by the very small sample size, baseline imbalances, and the non-comparative design, and therefore should be considered hypothesis-generating rather than indicative of regimen-specific differences. In addition, enrollment was restricted to patients with advanced HCC and macrovascular invasion, a population with inherently poor prognosis, which further complicates cross-trial comparisons and interpretation of efficacy outcomes. Second, the RNA-sequencing results were derived from a limited subset of pretreatment biopsies and should be regarded as hypothesis-generating. They do not allow firm conclusions about combination-specific mechanisms or an abscopal effect, and no evidence was obtained that the treatment altered unfavorable pathways such as mesenchymal or angiogenic programs. To clarify potential synergistic effects, future studies will require larger cohorts with paired pre- and post-treatment samples, comparisons across radiation dose levels, and rational combinations such as the addition of VEGF inhibitors.

In conclusion, this study demonstrated the feasibility and safety of a novel therapeutic approach combining selective C-ion RT targeting MVI-containing tumors with ICIs in advanced HCC patients. Although C-ion RT achieved effective local control of MVI-positive lesions, a critical adverse prognostic factor, systemic immune responses in non-irradiated lesions remained limited, emphasizing the need for further optimization of combination strategies. Despite the small cohort, this proof of concept study provides a foundation for further investigation of this selective targeting approach, potentially offering a new treatment paradigm for advanced HCC patients with MVI.

Affiliations

[1]*Department of Gastroenterology, Graduate School of Medicine, Chiba University, Chiba, Japan;* [2]*National Institutes for Quantum Science and Technology, QST Hospital, Chiba, Japan;* [3]*Clinical Research Center, Chiba University Hospital, Chiba, Japan;* [4]*Division of Digestive and Liver Diseases, Department of Internal Medicine, University of Texas Southwestern Medical Center, Dallas, TX, USA;* [5]*Lyda Hill Department of Bioinformatics, The University of Texas Southwestern Medical Center, Dallas, TX, USA*

Abbreviations

AEs, adverse events; AFP, α-fetoprotein; BCLC, Barcelona Clinic Liver Cancer; C-ion RT, carbon-ion radiotherapy; CRS, cytokine release syndrome; CRT, chemoradiotherapy; DAMPs, damage-associated molecular patterns; DLT, dose-limiting toxicity; ECOG-PS, Eastern Cooperative Oncology Group performance status; EHM, extrahepatic metastasis; ERCP, endoscopic retrograde cholangiopancreatography; GSEA, Gene Set Enrichment Analysis; GTV, gross tumor volume; HCC, hepatocellular carcinoma; ICANS, immune effector cell-associated neurotoxicity syndrome; ICD, immunogenic cell death; ICIs, immune checkpoint inhibitors; LET, linear energy transfer; MVI, macrovascular invasion; ORR, objective response rate; OS, overall survival; PD-L1, programmed cell death ligand 1; PFS, progression-free survival; RBE, relative biological equivalent; RECIST, Response Evaluation Criteria in Solid Tumors; SAEs, serious adverse events; SBRT, stereotactic body radiotherapy; TACE, transarterial chemoembolization; TEAEs, treatment-emergent adverse events; TKIs, tyrosine kinase inhibitors; TRAEs, treatment-related adverse events; Tregs, regulatory T cells; TTP, time to progression; VEGF, vascular endothelial growth factor.

Financial support

This phase Ib trial was supported by AstraZeneca K.K. (Osaka, Japan; grant number ESR-19-20168), which provided funding and study drugs (durvalumab and tremelimumab). The trial operations were partially supported by management expense grants from the National Institutes for Quantum Science and Technology, Chiba, Japan. Analysis of tumor microenvironment using clinical specimens was supported by the Project Mirai Cancer Research Grants (operated by Relay for Life) and JSPS KAKENHI (grant numbers JP22K08049 and JP23K27577) (to SO). This analysis was additionally supported by the UT Southwestern Simmons Comprehensive Cancer Center (SCCC) grant P30CA142543 (to JC and JL), U.S. National Institute of Health (R01CA233794, R01CA255621, R01CA282178, U01CA288375, U01CA283935, P30CA142543), European Commission (ERC-AdG-2020-101021417), and Cancer Prevention and Research Institute of Texas (RR180016, RP200554) (YH).

Conflicts of interest

SO received honoraria from Bayer, Leverkusen, Germany; Eisai, Tokyo, Japan; Eli Lilly, Indianapolis, IN, USA; Chugai Pharma, Tokyo, Japan; AstraZeneca, Cambridge, UK; and Merck & Co., Inc., Kenilworth, NJ, USA; consulting or advisory fees from Bayer, Eisai, Merck & Co., Inc., Chugai Pharma, Eli Lilly, and AstraZeneca; and research grants from Bayer, AstraZeneca, and Eisai. KK received honoraria from Chugai Pharma, Tokyo, Japan, Eisai, Tokyo, Japan and AbbVie Inc. YH serves as an advisory for Helio Genomics, Espervita Therapeutics, Roche Diagnostics, and Elevar Therapeutics, and a shareholder for Alentis

Therapeutics and Espervita Therapeutics. The other authors have no conflicts of interest to declare.
Please refer to the accompanying ICMJE disclosure forms for further details.

Authors contributions

Drafted the manuscript: SO, KK. Designed the protocol: SO, KK, HM, MW. Primarily involved in and contributed to the procedures for conduct of the clinical trial: SO, KK, HM, MW, AT, HH. Performed statistical analyses for the clinical part of the study: YO, YI. Performed analyses for the exploratory part of the study: KK, MF, HK, SP, JC, JL, YH. Aided in the assessment and revisions of the protocol and manuscript: SY, MN, HK, KKobayashi, MI, MNakamura, NK, TK, SN, TK, SYamada, HI. Recruited and/or treated patients: SO, KK, HM, MW, SY, MN, HK, KKobayashi, MI, MNakamura, NKanogawa, TKondo. Created the automated RNA-seq analysis pipeline onto Astrocyte platform, hosted by the BioHPC supercomputing facility: SP, JC.

Declaration of generative AI and AI-assisted technologies in the writing process

During the preparation of this work the authors used ChatGPT in order to assist with English proofreading. After using this tool/service, the authors reviewed and edited the content as needed and take full responsibility for the content of the publication.

Data availability

This article contains all of the data collected or analyzed during the course of this study. Raw data are not made publicly available because it would jeopardize patient privacy or consent, but deidentified raw data are available upon reasonable request. The RNA-sequencing dataset is publicly available at NCBI GEO (accession numbers: GSE287319). Further inquiries should be directed to the corresponding author.

Acknowledgements

The authors are grateful to the following people for their contributions: Tokuhiko Omatsu and Riwa Kishimoto contributed to the radiological evaluation during the study treatment. The bioinformatics analyses were done by utilizing computational resources provided by the BioHPC supercomputing facility located in Lyda Hill Department of Bioinformatics, UT Southwestern Medical Center.

Supplementary data

Supplementary data to this article can be found online at https://doi.org/10.1016/j.jhepr.2026.101765.

References

Author names in bold designate shared co-first authorship

[1] Rumgay H, Arnold M, Ferlay J, et al. Global burden of primary liver cancer in 2020 and predictions to 2040. J Hepatol 2022;77:1598–1606.
[2] Singal AG, Kanwal F, Llovet JM. Global trends in hepatocellular carcinoma epidemiology: implications for screening, prevention and therapy. Nat Rev Clin Oncol 2023;20:864–884.
[3] Craig AJ, von Felden J, Garcia-Lezana T, et al. Tumour evolution in hepatocellular carcinoma. Nat Rev Gastroenterol Hepatol 2020;17:139–152.
[4] Vogel A, Meyer T, Sapisochin G, et al. Hepatocellular carcinoma. Lancet 2022;400:1345–1362.
[5] Villanueva A. Hepatocellular carcinoma. N Engl J Med 2019;380:1450–1462.
[6] Kokudo T, Hasegawa K, Matsuyama Y, et al. Survival benefit of liver resection for hepatocellular carcinoma associated with portal vein invasion. J Hepatol 2016;65:938–943.
[7] Yoon SM, Ryoo BY, Lee SJ, et al. Efficacy and safety of transarterial chemoembolization plus external beam radiotherapy vs sorafenib in hepatocellular carcinoma with macroscopic vascular invasion: a randomized clinical trial. JAMA Oncol 2018;4:661–669.
[8] Kosaka Y, Kimura T, Kawaoka T, et al. Hepatic arterial infusion chemotherapy combined with radiation therapy for advanced hepatocellular carcinoma with tumor thrombosis of the main trunk or bilobar of the portal vein. Liver Cancer 2021;10:151–160.
[9] Inoue M, Ogasawara S, Kobayashi K, et al. Assessment of macrovascular invasion in advanced hepatocellular carcinoma: clinical implications and treatment outcomes with systemic therapy. Liver Cancer 2024;14:8–18.
[10] Finn RS, Qin S, Ikeda M, et al. Atezolizumab plus bevacizumab in unresectable hepatocellular carcinoma. N Engl J Med 2020;382:1894–1905.
[11] Cheng AL, Qin S, Ikeda M, et al. Updated efficacy and safety data from IMbrave150: atezolizumab plus bevacizumab vs. sorafenib for unresectable hepatocellular carcinoma. J Hepatol 2022;76:862–873.
[12] Abou-Alfa GK, Lau G, Kudo M, et al. Tremelimumab plus durvalumab in unresectable hepatocellular carcinoma. NEJM Evid 2022;1. EVIDoa2100070.
[13] Zhu AX, Abbas AR, de Galarreta MR, et al. Molecular correlates of clinical response and resistance to atezolizumab in combination with bevacizumab in advanced hepatocellular carcinoma. Nat Med 2022;28:1599–1611.
[14] Kelley RK, Sangro B, Harris W, et al. Safety, efficacy, and pharmacodynamics of tremelimumab plus durvalumab for patients with unresectable hepatocellular carcinoma: randomized expansion of a phase I/II study. J Clin Oncol 2021;39:2991–3001.
[15] Duffy AG, Ulahannan SV, Makorova-Rusher O, et al. Tremelimumab in combination with ablation in patients with advanced hepatocellular carcinoma. J Hepatol 2017;66:545–551.
[16] Shibuya K, Ohno T, Terashima K, et al. Short-course carbon-ion radiotherapy for hepatocellular carcinoma: a multi-institutional retrospective study. Liver Int 2018;38:2239–2247.
[17] Shibuya K, Katoh H, Koyama Y, et al. Efficacy and safety of 4 fractions of carbon-ion radiation therapy for hepatocellular carcinoma: a prospective study. Liver Cancer 2021;11:61–74.
[18] Mohamad O, Makishima H, Kamada T. Evolution of carbon ion radiotherapy at the national Institute of radiological sciences in Japan. Cancers (Basel) 2018;10:66.
[19] Igaki H, Mizumoto M, Okumura T, et al. A systematic review of publications on charged particle therapy for hepatocellular carcinoma. Int J Clin Oncol 2018;23:423–433.
[20] Matsuo Y, Yoshida K, Nishimura H, et al. Efficacy of stereotactic body radiotherapy for hepatocellular carcinoma with portal vein tumor thrombosis/inferior vena cava tumor thrombosis: evaluation by comparison with conventional three-dimensional conformal radiotherapy. J Radiat Res 2016;57:512–523.
[21] Rim CH, Kim CY, Yang DS, et al. Comparison of radiation therapy modalities for hepatocellular carcinoma with portal vein thrombosis: a meta-analysis and systematic review. Radiother Oncol 2018;129:112–122.
[22] Kaneko T, Makishima H, Wakatsuki M, et al. Carbon-ion radiotherapy for hepatocellular carcinoma with major vascular invasion: a retrospective cohort study. BMC Cancer 2024;24:383.
[23] Zhu M, Yang M, Zhang J, et al. Immunogenic cell death induction by ionizing radiation. Front Immunol 2021;12:705361.
[24] Procureur A, Simonaggio A, Bibault JE, et al. Enhance the immune checkpoint inhibitors efficacy with radiotherapy induced immunogenic cell death: a comprehensive review and latest developments. Cancers (Basel) 2021;13:678.
[25] **Ogasawara S**, **Koroki K**, **Makishima H**, et al. Durvalumab with or without tremelimumab combined with particle therapy for advanced hepatocellular carcinoma with macrovascular invasion: protocol for the DEPARTURE phase Ib trial. BMJ Open 2022;12:e059779.
[26] Inaniwa T, Furukawa T, Kase Y, et al. Treatment planning for a scanned carbon beam with a modified microdosimetric kinetic model. Phys Med Biol 2010;55:6721–6737.
[27] Kase Y, Kanai T, Matsufuji N, et al. Biophysical calculation of cell survival probabilities using amorphous track structure models for heavy-ion irradiation. Phys Med Biol 2008;53:37–59.
[28] Kasuya G, Kato H, Yasuda S, et al. Progressive hypofractionated carbon-ion radiotherapy for hepatocellular carcinoma: combined analyses of 2 prospective trials. Cancer 2017;123:3955–3965.
[29] Shiba S, Shibuya K, Katoh H, et al. A comparison of carbon ion radiotherapy and transarterial chemoembolization treatment outcomes for single hepatocellular carcinoma: a propensity score matching study. Radiat Oncol 2019;14:137.
[30] Shiba S, Shibuya K, Okamoto M, et al. Clinical impact of hypofractionated carbon ion radiotherapy on locally advanced hepatocellular carcinoma. Radiat Oncol 2020;15:195.
[31] Ebner DK, Tsuji H, Yasuda S, et al. Respiration-gated fast-rescanning carbon-ion radiotherapy. Jpn J Clin Oncol 2017;47:80–83.
[32] Maki K, Haga H, Katsumi T, et al. Adverse events after carbon-ion radiotherapy (CIRT) for hepatocellular carcinoma and risk factors for biliary stricture after CIRT: a retrospective study. Cancers (Basel) 2025;17:2542.

[33] Sha CM, Lehrer EJ, Hwang C, et al. Toxicity in combination immune checkpoint inhibitor and radiation therapy: a systematic review and meta-analysis. Radiother Oncol 2020;151:141–148.
[34] Helm A, Ebner DK, Tinganelli W, et al. Combining heavy-ion therapy with immunotherapy: an update on recent developments. Int J Part Ther 2018;5:84–93.
[35] Takahashi Y, Yasui T, Minami K, et al. Carbon ion irradiation enhances the antitumor efficacy of dual immune checkpoint blockade therapy both for local and distant sites in murine osteosarcoma. Oncotarget 2019;18(10):633–646.
[36] Okonogi N, Murata K, Yamada S, et al. A phase Ib study of durvalumab (MEDI4736) in combination with carbon-ion radiotherapy and weekly cisplatin for patients with locally advanced cervical cancer (DECISION study): the early safety and efficacy results. Int J Mol Sci 2023;24:10565.
[37] Cavalieri S, Ronchi S, Barcellini A, et al. Toxicity of carbon ion radiotherapy and immune checkpoint inhibitors in advanced melanoma. Radiother Oncol 2021;164:1–5.
[38] **Fujita N**, **Kanogawa N**, **Makishima H**, et al. Carbon-ion radiotherapy versus radiofrequency ablation as initial treatment for early-stage hepatocellular carcinoma. Hepatol Res 2022;52:1060–1071.
[39] Barcellini A, Molinelli S, Vanoli A, et al. Preoperative chemo-CIRT in Re/BRe pancreatic cancer: insights from a multicenter prospective phase II clinical study (NCT03822936). Tumori 2024;110:470–474.
[40] Shinoto M, Yamada S, Yasuda S, et al. Phase 1 trial of preoperative, short-course carbon-ion radiotherapy for patients with resectable pancreatic cancer. Cancer 2013;119:45–51.
[41] Fajgenbaum DC, June CH. Cytokine storm. N Engl J Med 2020;383:2255–2273. 3.
[42] Lee DW, Santomasso BD, Locke FL, et al. ASTCT consensus grading for cytokine release syndrome and neurologic toxicity associated with immune effector cells. Biol Blood Marrow Transpl 2019;25:625–638.
[43] Morris EC, Neelapu SS, Giavridis T, et al. Cytokine release syndrome and associated neurotoxicity in cancer immunotherapy. Nat Rev Immunol 2022;22:85–96.
[44] Koller KM, Mackley HB, Liu J, et al. Improved survival and complete response rates in patients with advanced melanoma treated with concurrent ipilimumab and radiotherapy versus ipilimumab alone. Cancer Biol Ther 2017;18:36–42.
[45] Antonia SJ, Villegas A, Daniel D, et al. Overall survival with durvalumab after chemoradiotherapy in stage III NSCLC. N Engl J Med 2018;379:2342–2350.
[46] Kelly RJ, Ajani JA, Kuzdzal J, et al. Adjuvant nivolumab in resected esophageal or gastroesophageal junction cancer. N Engl J Med 2021;384:1191–1203. 1.
[47] Kim BH, Park HC, Kim TH, et al. Concurrent nivolumab and external beam radiation therapy for hepatocellular carcinoma with macrovascular invasion: a phase II study. JHEP Rep 2023;6:100991.
[48] Juloori A, Katipally RR, Lemons JM, et al. Phase 1 randomized trial of stereotactic body radiation therapy followed by nivolumab plus ipilimumab or nivolumab alone in advanced/unresectable hepatocellular carcinoma. Int J Radiat Oncol Biol Phys 2023;115:202–213.
[49] Chiang CL, Chiu KWH, Chan KSK, et al. Sequential transarterial chemoembolisation and stereotactic body radiotherapy followed by immunotherapy as conversion therapy for patients with locally advanced, unresectable hepatocellular carcinoma (START-FIT): a single-arm, phase 2 trial. Lancet Gastroenterol Hepatol 2023;8:169–178.
[50] Sugahara S, Nakayama H, Fukuda K, et al. Proton-beam therapy for hepatocellular carcinoma associated with portal vein tumor thrombosis. Strahlenther Onkol 2009;185:782–788.
[51] Lee SU, Park JW, Kim TH, et al. Effectiveness and safety of proton beam therapy for advanced hepatocellular carcinoma with portal vein tumor thrombosis. Strahlenther Onkol 2014;190:806–814.
[52] **Yoshida R**, **Koroki K**, **Makishima H**, et al. Controlling major portal vein invasion progression during lenvatinib treatment by carbon-ion radiotherapy in patients with advanced hepatocellular carcinoma. Case Rep Oncol 2021;14:1103–1110. 15.
[53] Daguenet E, Louati S, Wozny AS, et al. Radiation-induced bystander and abscopal effects: important lessons from preclinical models. Br J Cancer 2020;123:339–348.
[54] Ngwa W, Irabor OC, Schoenfeld JD, et al. Using immunotherapy to boost the abscopal effect. Nat Rev Cancer 2018;18:313–322.
[55] Sia D, Jiao Y, Martinez-Quetglas I, et al. Identification of an Immune-specific class of hepatocellular carcinoma, based on molecular features. Gastroenterology 2017;153:812–826.
[56] Sangro B, Sarobe P, Hervás-Stubbs S, et al. Advances in immunotherapy for hepatocellular carcinoma. Nat Rev Gastroenterol Hepatol 2021;18:525–543.
[57] Topalian SL, Hodi FS, Brahmer JR, et al. Safety, activity, and immune correlates of anti-PD-1 antibody in cancer. N Engl J Med 2012 28;366:2443–2454.
[58] Twyman-Saint Victor C, Rech AJ, Maity A, et al. Radiation and dual checkpoint blockade activate non-redundant immune mechanisms in cancer. Nature 2015;520:373–377.
[59] Herrera FG, Ronet C, Ochoa de Olza M, et al. Low-dose radiotherapy reverses tumor immune desertification and resistance to immunotherapy. Cancer Discov 2022;12:108–133.
[60] Li S, Li K, Wang K, et al. Low-dose radiotherapy combined with dual PD-L1 and VEGFA blockade elicits antitumor response in hepatocellular carcinoma mediated by activated intratumoral CD8+ exhausted-like T cells. Nat Commun 2023;14:7709.
[61] Shigeta K, Datta M, Hato T, et al. Dual programmed death receptor-1 and vascular endothelial growth factor receptor-2 blockade promotes vascular normalization and enhances antitumor immune responses in hepatocellular carcinoma. Hepatology 2020;71:1247–1261.
[62] Mellman I, Chen DS, Powles T, et al. The cancer-immunity cycle: indication, genotype, and immunotype. Immunity 2023;56:2188–2205.
[63] Perez-Gutierrez L, Ferrara N. Biology and therapeutic targeting of vascular endothelial growth factor A. Nat Rev Mol Cell Biol 2023;24:816–834.

Keywords: Carbon-ion radiotherapy; Durvalumab; Tremelimumab; Hepatocellular carcinoma.
Received 13 June 2025; received in revised form 21 January 2026; accepted 27 January 2026; Available online 5 February 2026

Journal of Hepatology, Volume *8*

Supplemental information

MVI-targeted carbon-ion radiotherapy combined with immunotherapy for advanced hepatocellular carcinoma: Phase Ib DEPARTURE trial

Sadahisa Ogasawara, Keisuke Koroki, Hirokazu Makishima, Masaru Wakatsuki, Asahi Takahashi, Makoto Fujiya, Sae Yumita, Miyuki Nakagawa, Hiroaki Kanzaki, Kazufumi Kobayashi, Masanori Inoue, Masato Nakamura, Naoya Kanogawa, Takayuki Kondo, Shingo Nakamoto, Tomoya Kurokawa, Yoshihito Ozawa, Yosuke Inaba, Soumith Paritala, Jingxuan Chen, Jeon Lee, Yujin Hoshida, Hideki Hanaoka, Shigeru Yamada, and Hitoshi Ishikawa

MVI-targeted carbon-ion radiotherapy combined with Immunotherapy for advanced hepatocellular carcinoma: Phase Ib DEPARTURE trial

Sadahisa Ogasawara, Keisuke Koroki, Hirokazu Makishima, Masaru Wakatsuki, Asahi Takahashi, Makoto Fujiya, Sae Yumita, Miyuki Nakagawa, Hiroaki Kanzaki, Kazufumi Kobayashi, Masanori Inoue, Masato Nakamura, Naoya Kanogawa, Takayuki Kondo, Shingo Nakamoto, Tomoya Kurokawa, Yoshihito Ozawa, Yosuke Inaba, Soumith Paritala, Jingxuan Chen, Jeon Lee, Yujin Hoshida, Hideki Hanaoka, Shigeru Yamada, Hitoshi Ishikawa

Table of contents

Supplementary methods……………………………………………………………2

Supplementary tables…………………………………………………….……...7

Supplementary figures………………………………………………………….16

Supplementary methods

This phase Ib, multicenter, open-label trial evaluates the safety and tolerability of a novel approach combining selective C-ion RT directed at MVI-containing tumors with immunotherapy in advanced HCC patients. Treatment arms consisted of durvalumab monotherapy (Cohort A) and durvalumab plus tremelimumab (Cohort B). The trial is conducted at two participating centers in Japan, Chiba University Hospital and QST Hospital.

Patients

Patients aged ≥20 years with histologically confirmed advanced HCC or a diagnosis based on typical hypervascular findings on CT or angiography were eligible. Additional requirements included macrovascular invasion, ECOG performance status 0–1, body weight >30 kg, Child-Pugh class A liver function, and adequate organ and bone marrow function. For the initial cohort, patients must have been refractory or intolerant to at least one prior systemic therapy, including atezolizumab plus bevacizumab, sorafenib, or lenvatinib. A life expectancy of at least 12 weeks and ineligibility for locoregional therapy were also required.

Patients were excluded if they had unresolved grade ≥2 toxicities (except alopecia and vitiligo), prior radiotherapy to >30% of bone marrow or involving the liver, major surgery within 28 days, history of organ transplantation, active primary immunodeficiency, or autoimmune disorders. Other exclusions included prior or current brain metastases, coinfection with hepatitis B and C or B and D viruses, immunosuppressive medication use within 14 days, and hypersensitivity to study drugs or excipients. Eligibility was determined by the principal or associate investigators based on Suppl. Table 1.

Treatment (including the procedure of C-ion RT)

C-ion RT (60 Gy in four fractions) began on day eight of cycle one. The gross tumour volume (GTV) for the radiotherapy were the intrahepatic nodules that formed MVI and the MVI itself.

Even when there were multiple intrahepatic nodules, only the tumors that formed MVI were irradiated. Clinical target volume was formed with a margin of 5mm from the GTV for the intrahepatic nodule and 10 mm along the portal / hepatic vein for the MVI. A fiducial marker was inserted adjacent to the target lesion for patient positioning. 4D-CT was used to determine respiratory motion. The data sets were then reconstructed and subdivided into 10 phases (T00: peak inhalation; T50: around exhalation). Combined with a interfractional margin of 3mm, T50 and phases below 3mm deviation from it were used to form a field-specific planning target volume (FTV) [22, 31]. Irradiation was done within these phases. C-ion RT was performed at a dose of 60 Gy / 4 fractions / week. The microdosimetric kinetic model [26] was used for calculations and all doses described in this report are relative biological equivalent weighted dose. Dose constraints for risk organs are as follows: gastrointestinal tract, $D2cm^3 \leq 30$Gy; spinal cord: $D_{(max)} \leq 25$ Gy; Residual liver volume (volume of the liver for which the dose is less than 30 Gy described as liver volume - V_{30}): 500 cm^3. The following liver volume information is also collected to accompany the residual liver volume: Liver V5 Gy and liver V20 Gy. Where dose constraints to the organ at risks and dose requirements for the FTV were in conflict, the dose to the risk organs was prioritized. These restrictions were determined based on previously published reports [16, 28–31]. Furthermore, cases where a metallic stent is present in the irradiation field are also excluded from C-ion RT treatment.

Tumor responses were evaluated via imaging (computed tomography or magnetic resonance imaging) at baseline and every six weeks. All complete and partial responses require confirmation by a subsequent scan at least four weeks after the initial response documentation.

Sample Size Determination, Statistical Analysis, and Definition of Dose-Limiting Toxicity

The study employs a modified 3+3 design to evaluate safety and tolerability, with a planned sample size of 15 patients, including the expansion cohort. We decided on the Rule-Based Dose-Escalation Design, 3+3 design, for this study because it fits the feasibility within the study period

and the need for sufficient safety data.

Grading of AEs follows the Common Terminology Criteria for Adverse Events (CTCAE) version 5.0. DLTs are defined as adverse events (AEs) at least possibly related to the treatment regimen, meeting the criteria outlined in Suppl. Table 2. Any treatment-related toxicity occurring during the DLT assessment period must be followed to resolution to determine whether it qualifies as a DLT, as specified in Suppl. Table 2.

Secondary efficacy endpoints include overall survival (OS), 6-month survival rate, objective response rate (ORR), progression-free survival (PFS) and time to progression (TTP). Survival analyses were conducted using the Kaplan-Meier method, with median survival times and their confidence intervals estimated using the Brookmeyer and Crowley method. All statistical analyses were performed using SAS for Windows, release 9.4 (SAS Institute, Cary, NC, USA).

Prospective Observation After Trial Completion

To assess long-term outcomes, we conducted a prospective observational study after trial completion with a data cutoff date of March 31, 2024. Overall survival was evaluated through regular follow-up visits, phone interviews, and medical records. Extended safety assessments included monitoring for AEs graded per CTCAE v5.0. Data on post-trial therapies, including systemic treatments, locoregional interventions, and supportive care, were analyzed to understand their impact on long-term survival.

Exploratory Biomarker Analysis with Tumor Biopsy Samples

For patients who provided additional informed consent, paired tumor biopsy samples were collected from non-irradiated liver lesions at baseline and 42 days after starting immunotherapy. RNA sequencing (RNA-seq) was conducted to investigate changes in the tumor

microenvironment, aiming to identify molecular signatures predictive of response or resistance to the combination therapy.

Total RNA was isolated from frozen tissue samples using the AllPrep DNA/RNA Mini Kit (Qiagen, CA, USA) according to the manufacturer's instructions. RNA-seq libraries were prepared using the SMART-Seq Stranded Kit (Takara Bio, Shiga, Japan) and sequenced on the NovaSeq 6000 system (Illumina, San Diego, CA, USA) using 150-bp paired-end reads. Raw reads in FASTQ format were trimmed using cutadapt (v. 2.5) with parameters "-q 20 --max-n 3 --cores=0 --minimum-length 75 -a "A{75}" -a "G{75}" -a "C{75}" -a "T{75}" -a Ill_Univ_Adapt". The trimmed reads were aligned to the human reference genome assembly GRCh38 (UCSC version hg38) using STAR (v. 2.7.3a) with parameters "--chimSegmentMin 15 --chimJunctionOverhangMin 15". Reads aligned to each gene annotated in GENCODE (v45) were quantified using featureCounts (v. 1.6.3). Normalized expression levels were calculated using the relative log expression (RLE) method implemented in the R package DESeq2 (v. 1.42.0). To ensure robust downstream analysis, filtering criteria were applied in R (v. 4.3.1) to remove lowly expressed and invariant genes. Specifically, genes with a coefficient of variation (CV) less than 0.01 across samples and those expressed in less than 10% of the samples were excluded from subsequent analyses. Gene Set Enrichment Analysis (GSEA) was performed using GenePattern (https://cloud.genepattern.org) against the Molecular Signatures Database (MSigDB v2024.1.Hs). The relative proportions of infiltrating immune cells were estimated using CIBERSORTx (https://cibersortx.stanford.edu) with the LM22 signature matrix (Supplementary CTAT table).

Data Management, Monitoring, and Ethics

Data management and monitoring are conducted in compliance with J-GCP guidelines. Case report forms are used to accurately record trial data, and monitors ensure adherence to the study protocol and regulatory standards. Independent audits are performed at the investigational sites to confirm quality control measures. A data monitoring committee, composed of clinical trial experts

and biostatisticians not involved in the study, evaluates trial data and assesses the safety and progress of the treatment cohorts.

All participants provided written informed consent before enrollment in the DEPARTURE trial. Ethics approval was obtained from the ethics committees of Chiba University Hospital and the National Institutes for Quantum and Radiological Science and Technology (approval numbers: 2020040 and C20-001). The study complies with the International Committee of Medical Journal Editors (ICMJE) guidelines for authorship. Results will be published in a peer-reviewed journal and registered on the Japan registry of clinical trials (jRCT). Amendments to the protocol are subject to approval by the institutional review boards. A follow-up study for long-term survival was approved by the Chiba University Hospital Ethics Committee (clinical trial No. HK202311-03).

Supplementary tables

Table S1. Eligibility criteria

Inclusion criteria

1. Capable of giving signed informed consent which includes compliance with the requirements and restrictions listed in the informed consent form (ICF) and in this protocol. Written informed consent and any locally required authorization obtained from the patient/legal representative prior to performing any protocol-related procedures, including screening evaluations. For patients aged <20 years and enrolling, a written informed consent should be obtained from the patient and his or her legally acceptable representative.
2. Age ≥20 years at time of study entry
3. Eastern Cooperative Oncology Group (ECOG) performance status of 0 or 1
4. Body weight >30 kg
5. Adequate normal organ and marrow function as defined below:
6. Haemoglobin ≥9.0 g/dL
7. Absolute neutrophil count (ANC) > 1500 per mm3
8. Platelet count ≥75 x 109/L (>75,000 per mm3)
9. Serum bilirubin ≤3.0 x institutional upper limit of normal (ULN)
10. AST (SGOT)/ALT (SGPT) ≤2.5 x institutional upper limit of normal unless liver metastases are present, in which case it must be ≤5x ULN
11. Measured creatinine clearance (CL) >40 mL/min or Calculated creatinine clearance CL>40 mL/min by the Cockcroft-Gault formula (Cockcroft and Gault 1976) or by 24-hour urine collection for determination of creatinine clearance
12. Evidence of post-menopausal status or negative urinary or serum pregnancy test for female pre-menopausal patients. Women will be considered post-menopausal if they have been amenorrheic for 12 months without an alternative medical cause. The following age-specific requirements apply:

    Women <50 years of age would be considered post-menopausal if they have been amenorrheic for 12 months or more following cessation of exogenous hormonal treatments and if they have luteinizing hormone and follicle-stimulating hormone levels in the post-menopausal range for the institution or underwent surgical sterilization (bilateral oophorectomy or hysterectomy).

    Women ≥50 years of age would be considered post-menopausal if they have been amenorrheic for 12 months or more following cessation of all exogenous hormonal treatments, had radiation-induced menopause with last menses >1 year ago, had chemotherapy-induced menopause with last menses >1 year ago, or underwent surgical sterilization (bilateral oophorectomy, bilateral salpingectomy or hysterectomy).

13. Patient is willing and able to comply with the protocol for the duration of the study including undergoing treatment and scheduled visits and examinations including follow up.
14. Advanced HCC confirmed histologically or by the typical findings of a hypervascular tumor on computed tomography or angiography
15. (Cohort A and Cohort B) Patients who have received at least one prior systemic chemotherapy regimen including atezolizumab bevacizumab combination, sorafenib, or lenvatinib and who are judged to be refractory or intolerant to standard therapy (not included in selection criteria in the expansion cohort).
16. Must not be eligible for locoregional therapy for unresectable HCC. For patients who progressed after locoregional therapy for HCC, locoregional therapy must have been completed ≥28 days prior to the baseline scan for the current study. Acceptable locoregional therapy for HCC are Ethanol Infusion Therapy, Radio Wave ablation Therapy, Transcatheter Arterial chemoembolization (TACE), Transcatheter arterial infusion (TAI). Hepatic Arterial Infusion Chemotherapy (HAIC) is not allowed.
17. Patients who have been diagnosed with HCC showing MVI. MVI is defined as a tumor thrombus in the major hepatic and/or portal vein branches (Vp2, Vp3, Vp4, Vv2, and Vv3) identified by imaging studies.
18. Child-Pugh A
19. Must have a life expectancy of at least 12 weeks

Exclusion criteria

1. Persons involved in the planning and conduct of this clinical trial (employees or staff of the sponsor and the site).
2. Patients who have participated in another clinical trial using the investigational drug within 28 days prior to obtaining consent, or who have received another investigational drug within 28 days prior to the first dose of the investigational drug in this study. The exception to this rule is if the patient is in an observational (non-interventional) clinical trial or during the follow-up period of an interventional trial.
3. Any unresolved NCI CTCAE grade ≥2 toxicity from previous anticancer therapy, with the exception of alopecia, vitiligo, and the laboratory values defined in the inclusion criteria.
4. Radiotherapy treatment to more than 30% of the bone marrow or with a wide field of radiation within four weeks of the first dose of the study drug
5. Major surgical procedure, as defined by the investigator, within 28 days prior to the first dose of IP
6. History of allogenic organ transplantation
7. Active or prior documented autoimmune or inflammatory disorders (including inflammatory bowel disease [e.g., colitis or Crohn's disease], diverticulitis [with the exception of diverticulosis], systemic lupus erythematosus, Sarcoidosis syndrome, or Wegener syndrome [granulomatosis with polyangiitis, Graves' disease, rheumatoid arthritis, hypophysitis, uveitis, etc.]). The following are exceptions to this criterion:
   - Patients with vitiligo or alopecia

- Patients with hypothyroidism (e.g., following Hashimoto syndrome) stable on hormone replacement
- Any chronic skin condition that does not require systemic therapy
- Patients without active disease in the last 5 years may be included but only after consultation with the study physician
- Patients with celiac disease controlled by diet alone

8. Uncontrolled intercurrent illness, including but not limited to, ongoing or active infection, symptomatic congestive heart failure, uncontrolled hypertension, unstable angina pectoris, cardiac arrhythmia, interstitial lung disease, serious chronic gastrointestinal conditions associated with diarrhea, or psychiatric illness/social situations that would limit compliance with study requirement, substantially increase risk of incurring AEs or compromise the ability of the patient to give written informed consent.
9. History of another primary malignancy except for
   - Malignancy treated with curative intent and with no known active disease ≥5 years before the first dose of IP and of low potential risk for recurrence
   - Adequately treated non-melanoma skin cancer or lentigo maligna without evidence of disease
   - Adequately treated carcinoma in situ without evidence of disease
10. History of leptomeningeal carcinomatosis
11. Prior or current brain metastases or spinal cord compression
12. Mean QT interval corrected for heart rate using Fridericia's formula (QTcF) ≥470 ms calculated from 3 ECGs (within 15 minutes at 5 minutes apart) Regardless of whether this criteria stays or not, all patients should have a baseline ECG
13. History of active primary immunodeficiency
14. Patients coinfected with hepatitis B and C viruses or with hepatitis B and D viruses
15. Current or prior use of immunosuppressive medication within 14 days before the first dose of durvalumab or tremelimumab
16. Patients who have received attenuated live attenuated vaccine within 30 days prior to the first dose of the investigational drug. Note: After incorporation, no live vaccination is allowed during the administration of the investigational drug and until 30 days after the last dose of the investigational drug.
17. Pregnant or lactating female patients, or male or female patients of reproductive potential who are unwilling to use an effective contraceptive method from screening until 90 days after the last dose of durvalumab monotherapy or 180 days after the last dose of durvalumab plus tremelimumab combination therapy.
18. Known allergy or hypersensitivity to any of the study drugs or any of the study drug excipients
19. Patients randomized or treated in a previous clinical trial using durvalumab and/or tremelimumab (regardless of the dose group to which they were assigned).
20. Patients who are determined by the investigator to be ineligible for participation, e.g., unlikely to comply with study procedures, restrictions, or requirements.
21. Patients who have been treated with anti-PD-1 antibody drugs, anti-PD-L1 antibody drugs, or other drugs acting on other stimulatory or co-inhibitory T-cell receptors and their

combinations (including atezolizumab and bevacizumab combination therapy) and have not tolerated the same therapy.<br>22. Prior radiotherapy involving the liver<br>23. Renal failure requiring hemodialysis or peritoneal dialysis<br>24. Presence of any severe cardiac disease<br>- NYHA Class III or IV chronic heart failure<br>- Current coronary artery disease or history of ischemic heart disease such as myocardial infarction within 6 months before the study<br>- Serious arrhythmia (grade 3 or higher according to the CTCAE ver. 4.0: arrhythmia that cannot be controlled by oral medications or requires mechanical control).<br>25. Poorly controlled hypertension<br>26. Serious and active infection, excluding hepatitis virus infection<br>27. Persistent proteinuria of NCI-CTCAE version 5.0 grade ≥3; urine dipstick result of 3+ is allowed if protein excretion is <3.5 g/ 24 hours<br>28. Arterial or venous thrombotic or embolic events, such as cerebrovascular accident, deep vein thrombosis, or pulmonary embolism within six months before the start of the study medication<br>29. Refractory pleural effusion or ascites<br>30. History of hepatic encephalopathy within the past 12 months<br>31. Patients who are unable to take oral intake.<br>32. Patients who test positive for HIV antibodies.<br>33. Patients with pulmonary fibrosis or interstitial pneumonia.<br>34. Patients with serious complications such as severe psychiatric disorders, history of gastrointestinal bleeding, or active hemoptysis.<br>35. Other patients deemed ineligible by the attending physician.

Abbreviations: HCC, hepatocellular carcinoma; NCI CTCAE, National Cancer Institute Common Terminology Criteria for Adverse Events

Table S2. Criteria for DLT

A DLT will be defined as the occurrence of an adverse event (AE) that is at least possibly related with the investigational product (IP) or investigational regimen (IR), with the two following exceptions: any grade of vitiligo or alopecia. AEs that are at least possibly related with durvalumab- and/ or tremelimumab-containing regimens will be defined as DLTs if the following criteria are met:<br>If a patient initiated on C-ion RT is unable to complete the C-ion RT within the allowable time period because of AEs that cannot be ruled out as causally related with durvalumab, tremelimumab, or C-ion RT, the AEs will be considered as DLT.<br><br>**Hematologic toxicity:**<br>• Grade ≥3 neutropenia complicated by fever of >38.3 °C<br>• Grade 4 neutropenia lasting more than seven days<br>• Grade ≥3 thrombocytopenia with significant bleeding<br>• Grade 4 thrombocytopenia, regardless of duration<br>• Grade 4 anemia, regardless of duration<br><br>**Nonhematologic toxicity:**<br>• Any grade 4 nonimmune-mediated AE<br>• Any grade 4 immune-mediated AE, excluding endocrinopathies<br>• Any grade 3 nonimmune-mediated AE that does not resolve to grade ≤1 or baseline within 30 days of optimal medical management<br>• Any grade 3 immune-mediated AE, excluding diarrhea/ colitis, pneumonitis, hepatitis, rash, neurotoxicity, myocarditis, myositis/ polymyositis, endocrinopathies and nephritis, which does not resolve to grade ≤1 or baseline within 30 days after onset of the event despite optimal medical management, including systemic corticosteroids<br>• Grade 3 diarrhea or colitis that does not resolve to grade ≤1 within 14 days (both immune- and nonimmune-mediated; the same applies if not specified in the remaining bullet points below]<br>• Grade 3 noninfectious pneumonitis<br>• Grade 2 noninfectious pneumonitis that does not resolve to grade ≤1 within three days of initiation of maximal supportive care<br>• Aspartate aminotransferase (AST) or alanine aminotransferase (ALT) ≥5 × ULN or 5× the baseline, if the baseline is abnormal, with concurrent increase in total bilirubin (TBL) ≥3 × ULN or 3× the baseline, if the baseline is abnormal without evidence of cholestasis or alternative explanations, such as viral hepatitis, disease progression in the liver (i.e., Hy's Law)<br>• ALT or AST >8 × ULN or 8× the baseline, if the baseline is abnormal, or TBL >5 × ULN or 5× the baseline, if the baseline is abnormal<br>• Grade 3 immune-mediated rash that does not resolve to grade ≤1 or baseline within 30 days<br>• Grade 2 rash covering >30% BSA that does not resolve to grade ≤1 or baseline within 30 days<br>• Any grade of immune-mediated rash with bullous formation

- Grade 3 immune-mediated neurotoxicity, excluding Guillain–Barre and myasthenia gravis, that does not resolve to grade ≤1 within 30 days
- Grade 2 or 3 immune-mediated peripheral neuromotor syndrome, such as Guillain–Barre and myasthenia gravis, that does not resolve to grade ≤1 within 30 days or that exhibits signs of respiratory insufficiency or autonomic instability
- Grade 3 immune-mediated myocarditis
- Any symptomatic immune-mediated myocarditis that does not become asymptomatic within three days of initiating optimal medical management, including systemic corticosteroids
- Grade 2 or 3 immune-mediated myositis/ polymyositis that does not resolve to grade ≤1 within 30 days of initiating optimal medical management, including systemic corticosteroids, or that exhibits signs of respiratory insufficiency, regardless of optimal medical management
- Immune-mediated increase in creatinine >3 × ULN or >3 × the baseline for patients with baseline creatinine that is above the ULN
- Transfusion of red cell concentrate or platelet or use of G-CSF during the DLT period

Table S3. Representativeness of study participants

Cancer type	Hepatocellular Carcinoma (HCC)
Considerations related to:	
Sex	HCC is more common in men, accounting for 70–85% of all patients.
Race/Ethnicity	The age at diagnosis of HCC ranges from 50–70 years, although it varies by race, region, and background liver disease. Currently, the age of onset of HCC in Japanese is reported to be in the early 70s.
Geography	Worldwide, 830,000 people are diagnosed as liver cancer annually, with an age adjusted incidence rate of 10.1 per 100,000 population. Most of them are HCC. In Japan, about 37,000 were diagnosed with HCC and about 25,000 died due to HCC. The age adjusted incidence rate is 12 per 100,000 population in Japan.
Overall representativeness of this study	The subjects in the present study were considered comparable in age (median 69 years) compared to recent articles on clinical trials in Japanese patient populations.

Table S4. Grade ≥2 Treatment related AEs of whole study population, cohort A and B.

	Cohort<br>A: Durvalumab + C-ion RT<br>B: Durvalumab + Tremelimumab + C-ion RT								
	**Whole population (n = 15)**			**Cohort A (n = 3)**			**Cohort B (n = 12)**		
**Event, n (%)**	Grade			Grade			Grade		
	G2	G3	G4	G2	G3	G4	G2	G3	G4
Any event	10 (66.7)	4 (26.7)	1 (6.7)	1 (33.3)	0 (0)	0 (0)	9 (75.0)	4 (33.3)	1 (8.3)
Lipase increased	2 (13.3)	2 (13.3)	0 (0)	0 (0)	0 (0)	0 (0)	2 (16.7)	2 (16.7)	0 (0)
AST increased	2 (13.3)	0 (0)	0 (0)	1 (33.3)	0 (0)	0 (0)	1 (8.3)	0 (0)	0 (0)
ALT increased	2 (13.3)	0 (0)	0 (0)	1 (33.3)	0 (0)	0 (0)	1 (8.3)	0 (0)	0 (0)
Platelet count decreased	1 (6.7)	1 (6.7)	0 (0)	1 (33.3)	0 (0)	0 (0)	0 (0)	1 (8.3)	0 (0)
Abdominal pain	1 (6.7)	0 (0)	0 (0)	1 (33.3)	0 (0)	0 (0)	0 (0)	0 (0)	0 (0)
Amylase increased	2 (13.3)	0 (0)	0 (0)	0 (0)	0 (0)	0 (0)	2 (16.7)	0 (0)	0 (0)
Cytokine release syndrome	0 (0)	1 (6.7)	0 (0)	0 (0)	0 (0)	0 (0)	0 (0)	1 (8.3)	0 (0)
Diarrhea	1 (6.7)	1 (6.7)	0 (0)	0 (0)	0 (0)	0 (0)	1 (8.3)	1 (8.3)	0 (0)
Nausea	1 (6.7)	0 (0)	0 (0)	0 (0)	0 (0)	0 (0)	1 (8.3)	0 (0)	0 (0)
Stasis dermatitis	1 (6.7)	0 (0)	0 (0)	0 (0)	0 (0)	0 (0)	1 (8.3)	0 (0)	0 (0)
Dyshidrotic eczema	1 (6.7)	0 (0)	0 (0)	0 (0)	0 (0)	0 (0)	1 (8.3)	0 (0)	0 (0)
Interstitial lung disease	1 (6.7)	0 (0)	0 (0)	0 (0)	0 (0)	0 (0)	1 (8.3)	0 (0)	0 (0)
Decreased appetite	1 (6.7)	0 (0)	0 (0)	0 (0)	0 (0)	0 (0)	1 (8.3)	0 (0)	0 (0)
Meningitis	0 (0)	1 (6.7)	0 (0)	0 (0)	0 (0)	0 (0)	0 (0)	1 (8.3)	0 (0)
Enterocolitis	0 (0)	1 (6.7)	0 (0)	0 (0)	0 (0)	0 (0)	0 (0)	1 (8.3)	0 (0)
Hypoalbuminemia	1 (6.7)	0 (0)	0 (0)	0 (0)	0 (0)	0 (0)	1 (8.3)	0 (0)	0 (0)
Hypoxia	1 (6.7)	0 (0)	0 (0)	0 (0)	0 (0)	0 (0)	1 (8.3)	0 (0)	0 (0)
Mucosal inflammation	0 (0)	0 (0)	1 (6.7)	0 (0)	0 (0)	0 (0)	0 (0)	0 (0)	1 (8.3)
Pyrexia	1 (6.7)	0 (0)	0 (0)	0 (0)	0 (0)	0 (0)	1 (8.3)	0 (0)	0 (0)
Rash	2 (13.3)	0 (0)	0 (0)	0 (0)	0 (0)	0 (0)	2 (16.7)	0 (0)	0 (0)
Adrenal insufficiency	0 (0)	1 (6.7)	0 (0)	0 (0)	0 (0)	0 (0)	0 (0)	1 (8.3)	0 (0)
Dysgeusia	1 (6.7)	0 (0)	0 (0)	0 (0)	0 (0)	0 (0)	1 (8.3)	0 (0)	0 (0)

Table S5. Progression-free survival (RECIST v1.1), Time to progression (RECIST v1.1) and Overall survival based on subsequent follow-up data (data cutoff date of OS analysis was March 31, 2024).

		**Cohort**<br>**A: Dur + C-ion RT**<br>**B: Dur + Treme + C-ion RT**		**Major portal vein invasion (Vp3, 4)**	
	**Whole population (n = 15)**	**Cohort A (n = 3)**	**Cohort B (n = 12)**	**Absent (n = 8)**	**Present (n = 7)**
PFS, months (95% CI)	4.7 (1.4–6.4)	4.7 (4.6–4.7)	3.8 (1.1–6.6)	4.6 (1.1–6.4)	4.7 (0.5–6.6)
TTP, months (95% CI)	4.7 (1.3–6.4)	4.7 (4.6–4.7)	6.2 (1.1–6.6)	4.6 (0.1–0.8)	4.7 (0.1–0.8)
OS, months (95% CI), based on subsequent follow-up data	10.4 (5.6–15.2)	28.2 (5.5–50.9)	10.2 (4.3–16.1)	14.5 (4.9–NE)	9.5 (3.2–28.2)

Abbreviations: PFS, progression-free survival; OS, Overall survival; Dur, durvalumab; Treme, tremelimumab

Supplementary figures

Fig. S1. Dosing schedule of the study.

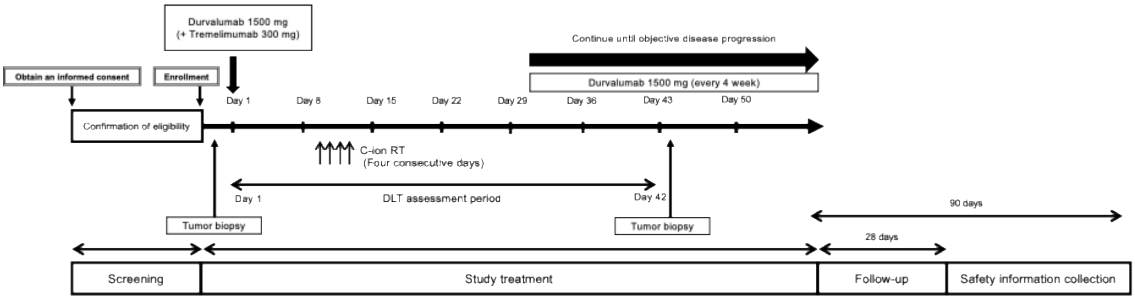

Patients were enrolled at two centers in Japan (Chiba University Hospital and QST Hospital) into two cohorts: durvalumab monotherapy (Cohort A) or durvalumab plus a single tremelimumab dose (Cohort B). C-ion RT (60 Gy in four fractions) begins on day 8 of Cycle 1, targeting MVI-containing intrahepatic lesions. DLTs are assessed during the 42-day evaluation period starting from the administration of durvalumab on Day 1 of Cycle 1. Study treatments continue until disease progression, according to RECIST ver. 1.1.

Abbreviations: C-ion RT, Carbon-ion radiotherapy; DLT, Dose-limiting toxicity

Fig. S2. Patient flow of the DEPARTURE trial

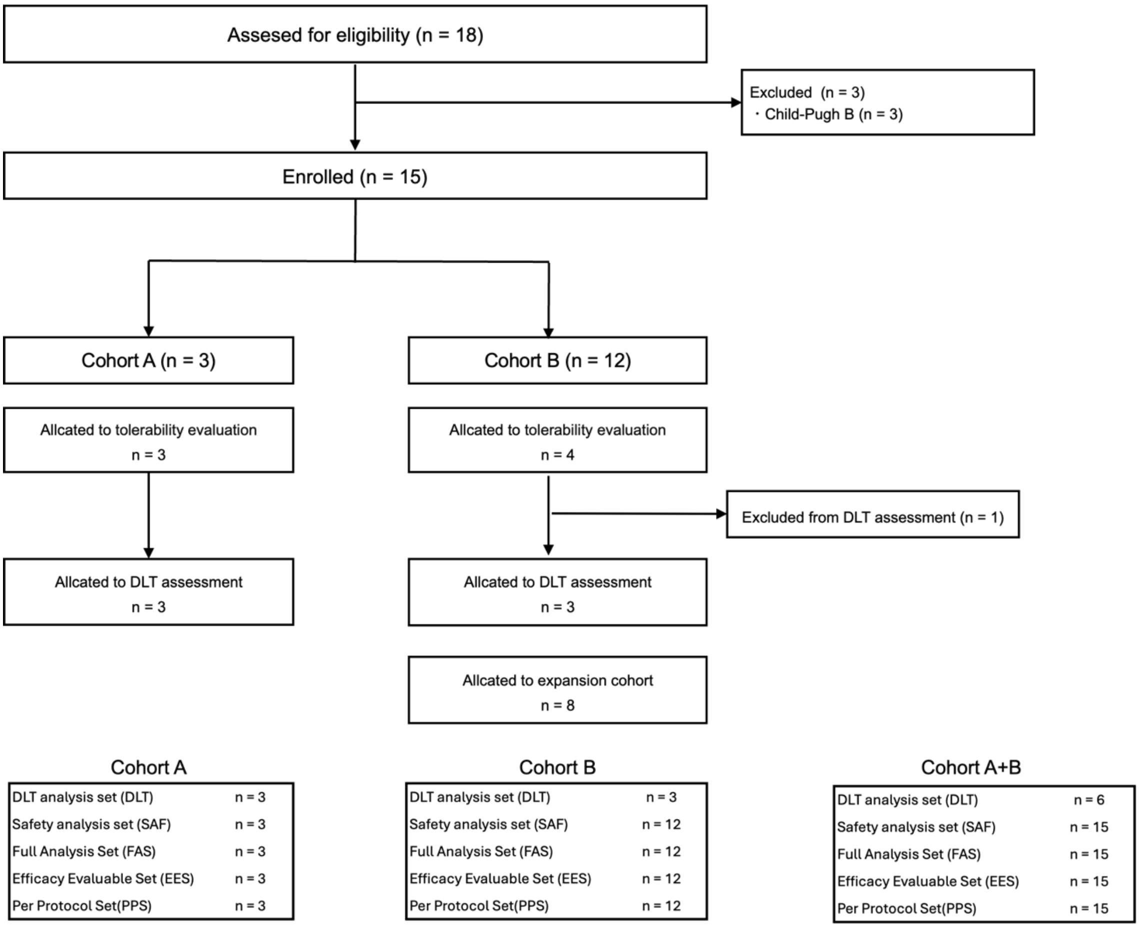

Informed consent was obtained from 18 patients, of whom 15 were enrolled. All 15 enrolled patients received the investigational treatment. In Cohort A, tolerability was assessed in 3 subjects, while in Cohort B, 12 patients were enrolled (4 for tolerability evaluation, 8 for the expansion cohort.

Abbreviation: DLT, Dose-limiting toxicity

Fig. S3. PFS (RECIST ver. 1.1) data of (A) whole population (n = 15), (B) stratified by cohort A and B, (C) stratified by major portal vein invasion (Vp3, 4).

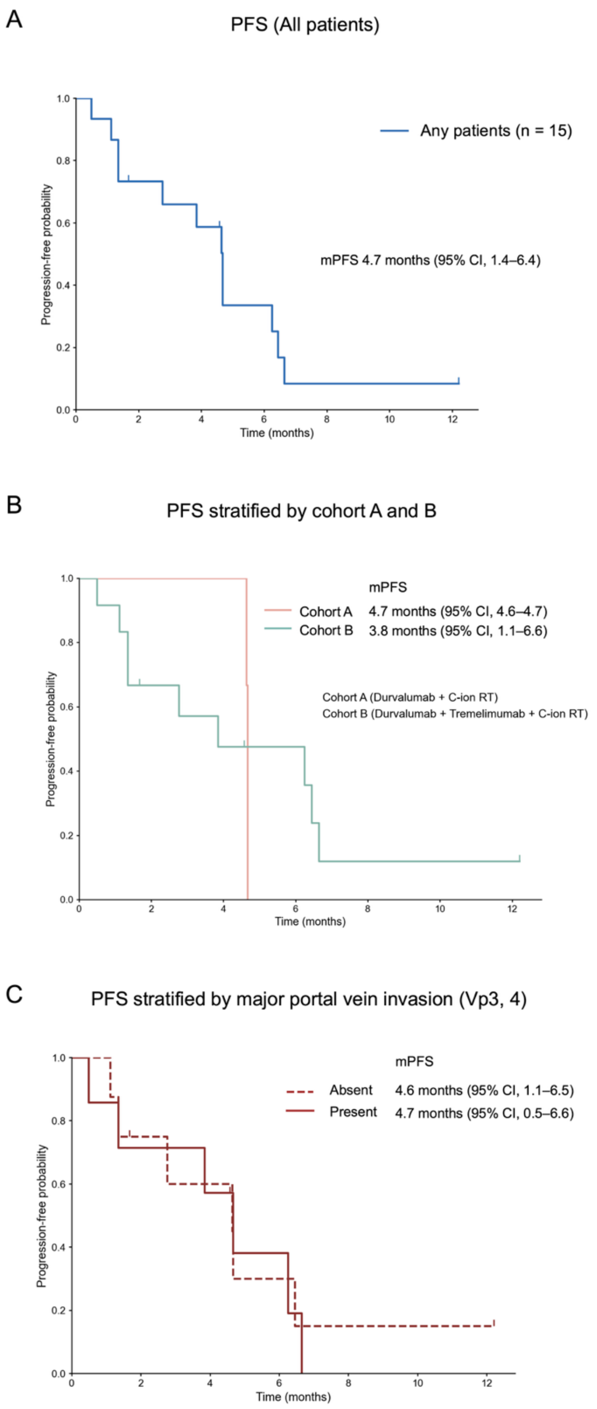

Abbreviations: PFS, Progression-free survival.

Fig. S4. Overall survival based on subsequent follow-up data (data cutoff date of OS analysis was March 31, 2024). (A) All patients (n = 15), (B) stratified by treatment cohort, (C) stratified by major portal vein invasion.

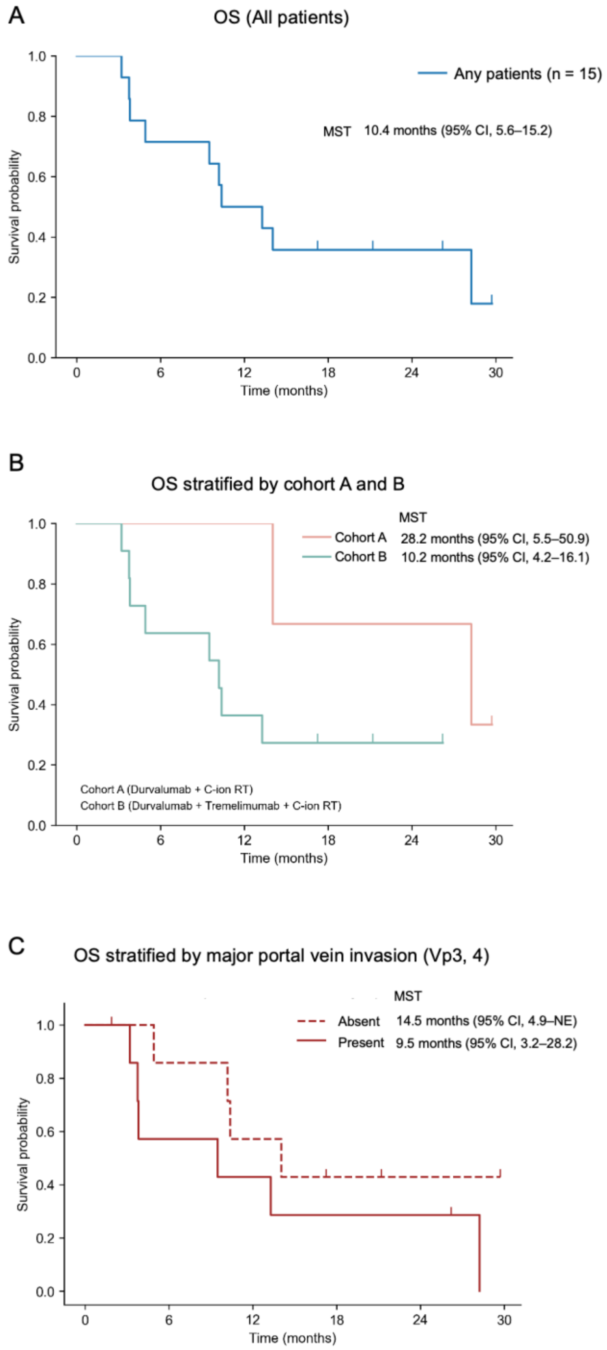

Abbreviations: OS, Overall survival; MST, median survival time

Supplementary Table 6. Tumor characteristics of lesions treated with C-ion RT. (A) Patient who experienced bile duct dilatation. (B) Patient without complications of bile duct.

A

No.	Cohort	Diameter of irradiated lesion		Perihilar type / Distal type		Major portal vein invasion (Vp3, 4)	
1	A	92.4 mm		Perihilar type		Present	
2	A	54.0 mm		Perihilar type		Absent	
7	B	135.0 mm		Perihilar type		Absent	
15	B	109.2 mm		Perihilar type		Present	
		Median diameter	100.8 mm	Rate of perihilar type	100.0 %	Rate of Vp3, 4	50.0 %

B

No.	Cohort	Diameter of irradiated lesion		Perihilar type / Distal type		Major portal vein invasion (Vp3, 4)	
3	A	102.8 mm		Distal type		Absent	
4	B	88.0 mm		Perihilar type		Present	
5	B	227.3 mm		Perihilar type		Present	
6	B	123.0 mm		Perihilar type		Absent	
8	B	77.5 mm		Perihilar type		Absent	
9	B	Diffuse		Perihilar type		Present	
10	B	Diffuse		Distal type		Absent	
11	B	86.7 mm		Perihilar type		Present	
12	B	99.4 mm		Perihilar type		Present	
13	B	44.4 mm		Distal type		Absent	
14	B	42.0 mm		Perihilar type		Absent	
		Median diameter	88.0 mm	Rate of perihilar type	72.7 %	Rate of Vp3, 4	45.5 %

Abbreviations. C-ion RT, Carbon-ion radiotherapy.

Fig. S5. Carbon-ion irradiation planning image. (A) Patient who experienced bile duct during follow-up period. (B) Patient without complications of bileduct during follow up period.

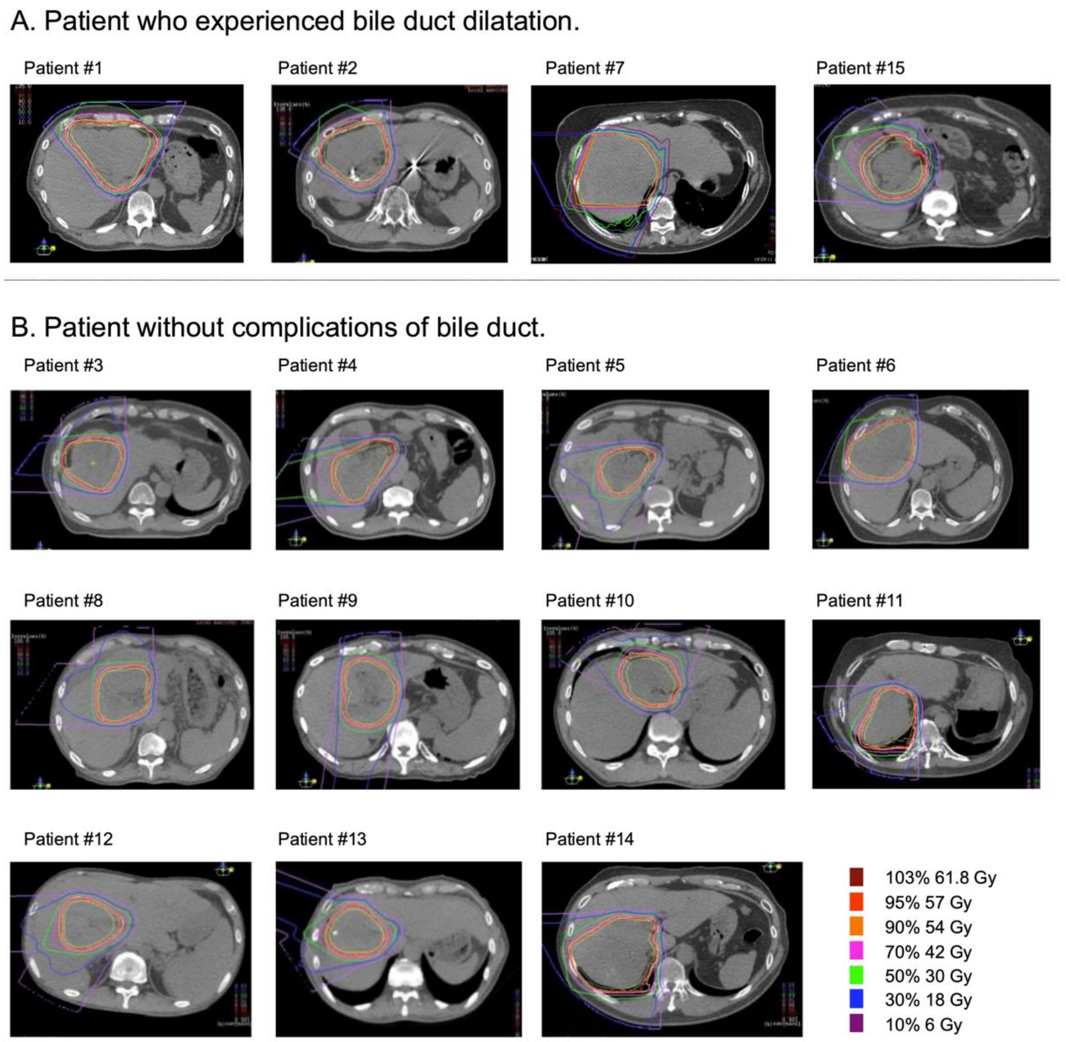

Fig. S6. PFS stratified by present or absent of High fever exceeding 38.5 ℃ or chills and rigor.

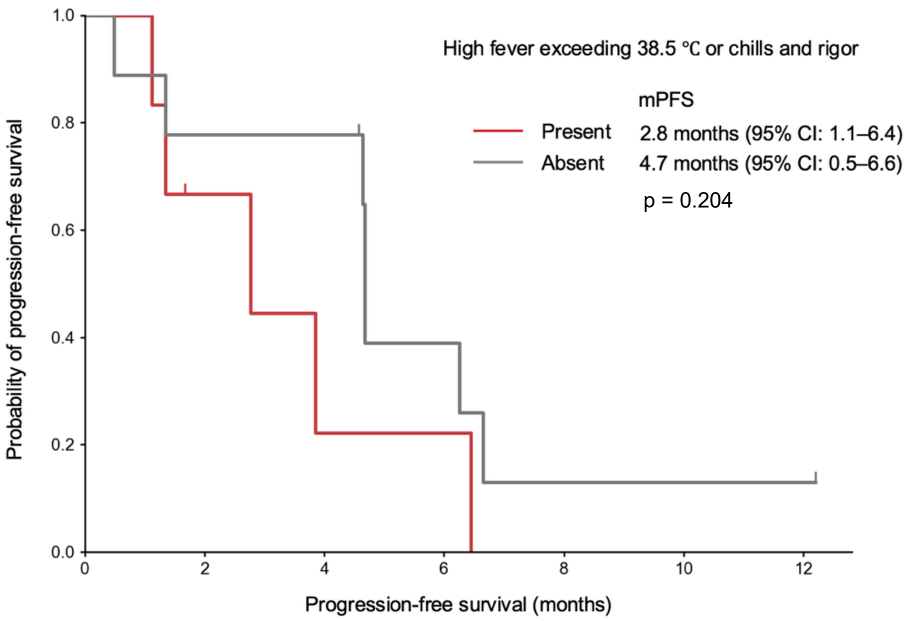

Abbreviations: PFS, Progression-free survival.
